# Tandem Repeats in *Bacillus*: Unique Features and Taxonomic Distribution

**DOI:** 10.3390/ijms22105373

**Published:** 2021-05-20

**Authors:** Juan A. Subirana, Xavier Messeguer

**Affiliations:** Department of Computer Science, Polytechnic University of Catalonia, 08034 Barcelona, Spain; peypoch@lsi.upc.edu

**Keywords:** tandem repeats, satellites, *Bacillus*, *Bacillus coagulans*, bacteria, non-coding DNA, bacterial nucleoid, small RNA

## Abstract

Little is known about DNA tandem repeats across prokaryotes. We have recently described an enigmatic group of tandem repeats in bacterial genomes with a constant repeat size but variable sequence. These findings strongly suggest that tandem repeat size in some bacteria is under strong selective constraints. Here, we extend these studies and describe tandem repeats in a large set of *Bacillus*. Some species have very few repeats, while other species have a large number. Most tandem repeats have repeats with a constant size (either 52 or 20–21 nt), but a variable sequence. We characterize in detail these intriguing tandem repeats. Individual species have several families of tandem repeats with the same repeat length and different sequence. This result is in strong contrast with eukaryotes, where tandem repeats of many sizes are found in any species. We discuss the possibility that they are transcribed as small RNA molecules. They may also be involved in the stabilization of the nucleoid through interaction with proteins. We also show that the distribution of tandem repeats in different species has a taxonomic significance. The data we present for all tandem repeats and their families in these bacterial species will be useful for further genomic studies.

## 1. Introduction

Bacterial genomes contain repetitive sequences, some of which have been studied in great detail [1]. Many of these repeats are identical or inverted sequences of different sizes interspersed throughout the genome. Some of these repeats do not have any adaptive value, while others influence gene expression. Another large group of repetitive sequences are tandem repeats or satellites. Many of them are polymorphic and present a variable number of tandem repeats in bacterial populations. These differences allow the characterization of different strains of a given bacterial species. A database covering tandem repeats suitable for this application was developed by Denoeud and Vergnaud [2].

In the previous work, we have studied the distribution of tandem repeats in 1241 bacterial genomes which had been fully sequenced and contain more than 19 tandem repeats per genome [3]. Among them we found species with an enigmatic group of tandem repeats, with a constant repeat size but variable sequence. Surprisingly, only two repeat sizes were found, either 20–21 or 52 nt. The constant size of repeats in many distantly related species strongly suggests that these sizes are important for their function. Tandem 20–23 nt repeats are found in individual species in many bacterial groups, whereas those with 52 nt repeats are only found in *Firmicutes*, mainly in *Bacillus*. For this reason, we decided to study in greater detail the tandem repeats in *Bacillus*.

In this paper, we provide a catalogue of all the DNA tandem repeats present in the 176 genomes of *Bacillus* recently studied by Khurana et al. [4]. The parameters of our search have been adjusted to obtain all tandem repeats with a minimum of four repeats and repeat length 10–270 nt. We have determined the families of tandem repeats found in individual and related species. We include all species, whereas in our previous work [3] we only studied species with more than 20 tandem repeats, which excluded a large proportion of bacterial genomes. We have also analyzed in greater detail the sequences of tandem repeats and found that in many species short characteristic nucleotide sequences are present throughout all the tandem repeats of the same species.

A detailed analysis of the tandem repeats present in different species of *Bacillus* will allow determining if they have any phylogenetic significance. The taxonomy of *Bacillus* is controversial [5] and different approaches yield conflicting results [4,5,6,7]. Our detailed analysis of tandem repeats demonstrates that different taxonomic groups have a unique distribution of tandem repeats in their genomes. Finally, we explore the possibility that transcribed tandem repeats play a role as small RNAs. We also compare the results obtained with *Bacillus* and those found in eukaryotes.

## 2. Materials and Methods

### 2.1. Detection of Tandem Repeats

We have chosen to analyze the set of 176 *Bacillus* genomes studied by Khurana et al. [4], downloaded from the NCBI website [8]. All these genomes have been fully sequenced, but many of them have not been aligned. A complete list is given in Supplementary Table S1, including genome size, number of tandem repeats, and CG% for each species. For comparison, we have also used some of the fully assembled genomes we studied in our previous paper [3]. Tandem repeats have been searched with the SATFIND program, which is available in our website [9] and is described in great detail elsewhere [10,11]. The program determines the localization of clusters of any short sequence of a prefixed size without internal repetitions and repeated a minimum number of times in regions with a fixed size. Repeats of 1–5 nt are automatically eliminated. The minimum length of a repeat is taken as 10 nt in our search. As a result, short repeats, for example 7 nt, will appear as repeats with a double size, 14 nt. Once a tandem repeat is located, the program continues its search along the genome until no further neighboring repeats are detected, with no upper limit for the number of repeats. This program allows a precise definition of tandem repeats (repeat size, number of repeats, and internal regularity). We have adjusted the parameters in order to capture short tandem repeats with at least four repeats in a genome region of 800 nt. In order to eliminate the most irregular tandem repeats, we have only accepted those which have at least 60% of their repeats with an identical length (±1 nt). In this way, most irregular tandem repeats are eliminated, although with these parameters some tandem repeats with only four repeats may still be irregular. Occasionally, we have also changed the parameters of the program to detect additional tandem repeats with a decreased regularity by requiring that only 10% of them had an identical length.

Each tandem repeat has also been characterized by a similarity score obtained upon alignment of all its repeats which have an identical size, thus excluding all repeats with indels. Each tandem repeat may also be characterized by a homogeneity parameter which gives the proportion of repeats with the same length in each tandem repeat. This parameter varies between 0.6 (60%) and 1.0 (100%), since tandem repeats with low homogeneity have not been accepted, as mentioned above. The regularity of each tandem repeat is thus characterized by two parameters. *Ni* gives the number of repeats in the tandem repeat which have an identical length and an alignment score is calculated for these *Ni* repeats.

A limitation of our study is due to the difficulty to determine accurately the complete sequence of tandem repeats due to common sequencing errors [12,13]. However, when sequences of the same genome reported by different authors are compared [12], all of the found tandem repeats are in the same positions, although their length may differ due to limitations of the sequencing methodologies. Often when different strains of the same species are compared, fewer tandem repeats are found in the genomes that have not been fully assembled, an indication that some tandem repeats may be lost. For this reason, we have studied in more detail tandem repeats of *Bacillus coagulans*, for which we have complete sequences of several strains.

### 2.2. Identification of Tandem Repeat Families

In order to detect related tandem repeats, we have used MALIG, a progressive multiple sequence alignment algorithm, which we have developed to align tandem repeats and identify families with a related sequence, available on our website [9]. It has been described in detail elsewhere [10]. The program considers reverse sequences as well, normalizes the alignment score to the maximum possible value, and selects the cyclic permutation with the highest score. Then, the progressive multi-alignment is applied to the matrix of pairwise alignment scores. The process finishes when the score is smaller than a similarity threshold (input parameter) which we set to 0.6.

We have searched for tandem repeat families in the whole set of genomes studied. Each family is characterized by three values: Fam_a_b_c. The order in the list of families is given by a, starting with those families with the largest number of members. The second value b gives the size of the consensus repeat, and c gives the number of members in the family. The consensus sequence of the repeat in each family is calculated taking into account the circularly permuted sequence of all repeats. Individual families may contain tandem repeats with slightly different repeat lengths (±1 nt). The b parameter should be interpreted with caution. When the sequences of the tandem repeats which are compared to build a family have indels, their average repeat length may not coincide with b. For example, a tandem repeat family with b = 53 may have most of its tandem repeats with a shorter length of 51–52 nt.

The use of all the tandem repeats from all the *Bacillus* genomes together gives families which either include tandem repeats of several species or families which are only present in a single species. In the list of families, tandem repeats which have unique repeats, appear as families with a single member, c = 1.

### 2.3. Genome Alignments

We have determined the correspondence of genomes of different strains of *Bacillus coagulans*. We have used the multiple genome comparison and alignment tool (M-GCAT), a multiple genome alignment tool based on the search of maximal unique matches (MUMs) between genomes on both strands [14]. First, a set of anchor MUMs is found where those MUMs shorter than a specific parameter (minimum anchor length) or randomly found (shorter than log base 4 on the length of the genome) are discarded. These sets of anchor MUMs divide the genomes in several short parts in which a recursive search of MUMs is made. This recursive search is made until the length of the part is shorter that a given parameter (100 nucleotides in our case). Finally, close consecutive MUMs, separated by less than a given parameter (in our case 2000 nucleotides), are grouped in clusters. The program provides a numerical and a graphical representation of the alignment.

## 3. Results

### 3.1. General Features of Tandem Repeats

**Figure 1:**
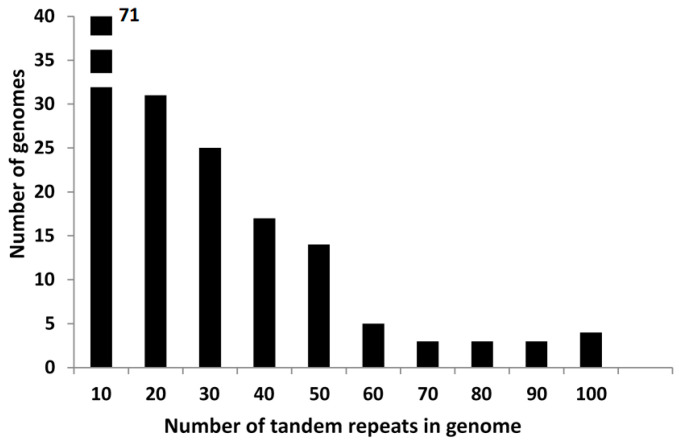
Number of genomes as a function of the number of tandem repeats in bins of 10 tandem repeats. Genomes with few tandem repeats (0–10) represent 40% of the total genomes analyzed.

**Figure 2:**
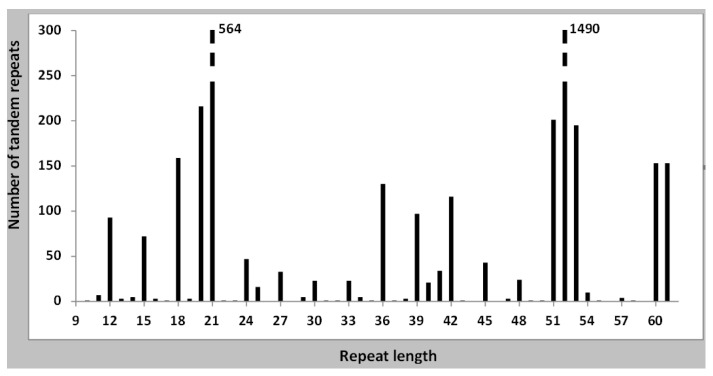
The number of tandem repeats with each repeat length is shown. The entirety of all tandem repeats of all Bacillus genomes has been used. The bar at 61 nt includes all lengths over 60 nt. The distribution is clearly non-random, three types of repeat lengths predominate: 20–21, 51–53, and multiples of three nucleotides. A few repeats of size 40–41 nt are also present, which are related to the 20–21 nt tandem repeats.

We have searched all the tandem repeats found in the 176 genomes which we have downloaded from GenBank, and detected 4029 tandem repeats in them, an average of 22.8 tandem repeats per genome. A complete list is given in Supplementary Table S2. For the study of the different types of tandem repeats we have prepared a list in which the size of all tandem repeats for each individual genome is given (Supplementary Table S1). A large number of species has very few tandem repeats, as shown in Figure 1. *B. subtilis* and the related species are found in this group. The overall distribution of tandem repeats, as a function of repeat size is presented in Figure 2. They fall into two main classes, with either a 20–21 repeat (781 cases) or a 51–53 repeat (1886 cases). Together they represent 66.2% of all tandem repeats. A third class is formed by those tandem repeats which have a repeat which is a multiple of three. We have next determined the tandem repeat families. A complete list is given in Supplementary Table S3, including all tandem repeats in each family. A summary of all families is also given in Supplementary Table S4. In the following, we will analyze separately each class of tandem repeats.

### 3.2. Tandem Repeats with a 52 nt Repeat

These tandem repeats offer a particular interest, since they have a very constant repeat length, while their composition is very variable. This is the most abundant class of tandem repeats in *Bacillus* (Figure 2), but they are only present in about 30% of the species we have studied. The repeat length is not a multiple of three, therefore these tandem repeats will not be able to code for amino acid repeats. A complete list of the species which have these tandem repeats is given in Supplementary Table S1. Each of them has a different group of tandem repeat families and unique tandem repeats, all with a constant repeat size but a different sequence. Occasionally, the repeat size may vary by one nucleotide (51 or 53 nt). The constant repeat length indicates that it is required for their function. The sequences of the repeats found in the species with a larger number of tandem repeats are given in Table 1. They are clearly species specific, with a few exceptions of closely related species. An additional feature of this group of species is the surprisingly small proportion of tandem repeats with different repeat lengths, as it is also apparent in Table 1. In most cases, when a species acquires 52 nt tandem repeats, other tandem repeats are absent, even those which might code for amino acid repeats in proteins. Most of these tandem repeats are rather short, with 4–10 repeats, but in all species a few longer tandem repeats are found. Histograms for a few species are presented in Figure S1. A particular case is *B. cellulosilyticus*, which has two long tandem repeats, with 25 and 37 repeats, the last one with over 2 Kb in length.

Each genome contains several families of these tandem repeats, but a close inspection demonstrates that in many cases all 52 nt repeats in a genome present conserved short sequences. A few examples are given in Table 2, note that each species has different sequences. Often these characteristic short sequences and their reverse are present in the same repeat. A list of all tandem repeats in the species shown in Table 2 is given in Supplementary Table S5. This nucleotide structure is reminiscent of the CRISPR repeats, which also present constant and variable sequence regions. However, the length of the sequences is completely different and, most important, the 52 nt tandem repeats are very abundant. CRISPR sequences are only present once or a few times in any bacterial genome [15]. Furthermore, there is no evidence for a protein similar to Cas which might interact specifically with the 52 nt repeats.

**Figure 3:**
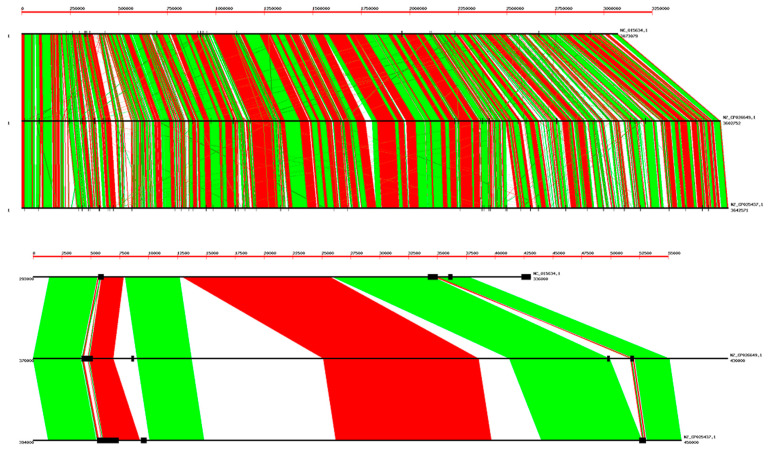
Alignment of three *B. coagulans* genomes; NCBI codes: NC_015634.1, NZ_CP026649.1, and NZ_CP025437.1. Tandem repeats are plotted as vertical black lines with a thickness proportional to the tandem repeat length. The whole genomes are presented in the upper frame; there is an extensive overall alignment, but many small gaps are apparent. The lower frame shows a small amplified region (50 Kb). Further examples are given in Figure S2. The correspondence of tandem repeats in different genomes is only approximate.

The next question is to find out if there is any turnover or specific position of the 52 nt tandem repeats in the genome. For this purpose, we have carried out a comparison of these tandem repeats in the genome of three strains of *B. coagulans*, as shown in Figure 3. As it is apparent, there is only a partial conservation of tandem repeats in different strains, in particular the length of tandem repeats is seldom conserved. More examples are presented in Figure S2.

In summary, several tandem repeat families with the 52 nt repeat are found in many *Bacillus* species. Their sequence is species specific. In different strains of the same species they are only partially conserved, each strain contains tandem repeats not found in other strains. All the 52 nt repeats in a particular species present a limited number (2–5) of characteristic short nucleotide sequences, which may be important for their function. There is no obvious explanation for the conservation of a constant 52 nt repeat in these groups of tandem repeats. It appears that the 52 nt length is required for whatever function these tandem repeats might have.

### 3.3. Tandem Repeats with a 20–21 nt Repeat

Another group of *Bacillus* species presents several families of tandem repeats with a repeat of 20–21 nt which are very abundant (Figure 1). In general, they do not code for amino acid repeats, a list is given in Supplementary Table S1. This type of tandem repeats is not exclusive of *Bacillus*, it is found in many other bacterial groups, including *Cyanobacteria, Actinobacteria*, etc. [3]. Some of the *Bacillus* species also present 52 nt tandem repeats. Each genome in this group has species-specific families of related tandem repeats, with the exception of *B. simplex* and *B. muralis.* These two species share several families of related tandem repeats, in agreement with their close taxonomic relationship. When all tandem repeats of a large family belong to the same species, it is clear that they are not part of protein coding genes. Practically all families with more than four tandem repeats are in this class. For example, all tandem repeats in the 13_21_15, 93_21_6, and 135_21_5 families, found in *B. weihaiensis,* are also not part of protein coding genes. On the other hand, when all members of a tandem repeat family belong to different species, it is likely that the tandem repeats correspond to part of a related gene with the same protein repeat: The 62_21_8 tandem repeat family is the largest example, with a repeat of seven amino acids found in the “choice-of-anchor A family protein” of *B. cereus* and several related species.

Tandem repeats in this group also present a characteristic short nucleotide sequence conserved in all repeats of most tandem repeats. Its sequence is given in Table 2 for *B. weihaiensis* and its position in all tandem repeats is found in the complete list of tandem repeats of this species, presented in Supplementary Table S5. Thus, this group of tandem repeats has features similar to the 52 nt tandem repeats: They are also species specific. However, it is not clear if their role in the genome is related in some way.

### 3.4. Tandem Repeats with Repeat Length Multiple of Three

Most *Bacillus* genomes contain a few of these tandem repeats. They usually correspond to regions coding for amino acid repeats in proteins. In some cases, they form families which include related genomes. Many of these families correspond to *B. cereus* and related species, the largest ones are 21_36_13 and 27_39_11. A few other species also have abundant tandem repeats of this type. For example, all tandem repeats in the 54_114_8 family are found in *B. indicus*, which contains several proteins with identical 38 amino acid repeats in its genome. A striking example is the 1_60_41 family, which is the largest family of *Bacillus* tandem repeats that we have detected. Tandem repeats in this family are part of genes coding for a general stress protein, which has four or five twenty amino acid repeats. This protein is found in several *Bacillus* species and in many other bacteria, including *Escherichia coli*. A few examples are given in Table 3. A striking case is *B. subtilis*, whose single tandem repeat belongs to this family. On the other hand, no species of the *B. cereus* group presents tandem repeats in this family.

These tandem repeats are found as part of the coding region for a general stress protein. This gene is found in many bacterial species, but the 60 nt repeat is only present in some cases. In *B. coagulans,* a tandem repeat is only found in one strain. In the other strain shown in the Table, the corresponding gene is heavily mutated and does not appear as a tandem repeat.

### 3.5. Taxonomic Distribution of Tandem Repeats

The distribution of tandem repeats in different species allows their classification in different groups, as shown in Table 4. Species which contain tandem repeats with either 20–21 or 52 nt repeats do not appear to correspond to a particular taxonomic group, they are found in several unrelated species. In order to ascertain whether tandem repeats have any taxonomic significance, we have compared our results with the taxonomic classifications proposed by different authors [4,5,6,7], as shown in detail in Supplementary Table S6. Such comparison allows us to include tandem repeats as a taxonomic feature of *Bacillus*, as summarized in Table 5. We have tried to include the genomes we have analyzed in the groups suggested by Secaira-Morocho et al. [7], which in most cases agree with other suggestions [5,6]. Nevertheless, some *Bacillus* species do not fit in any of these groups. The clusters suggested by Khurana et al. [4] only show a partial agreement with the classifications suggested by the other authors [5,6,7]. Only genomes which are covered by at least two groups have been analyzed, which limits our analysis to only 66 of the 176 genomes we have studied. When the taxonomic placement of a species suggested by different authors does not coincide, we have placed the species in a MISCELLANEOUS group.

The CEREUS group is very homogeneous, with similar genome sizes and CG%. A moderate amount of tandem repeats (25.7 average) is found. Practically all these tandem repeats have repeats with a size multiple of three, which correspond to amino acid repeats in proteins, other tandem repeats are practically absent. Tandem repeat families contain tandem repeats from different species: They correspond to conserved genes. In fact, the boundaries between members of this group are difficult to define [5], so the genomes in this group might be considered as different strains of a single species. We have not detected any species outside this group with such an abundance of genes containing tandem repeats coding for amino acid strings.

A characteristic feature of several groups is the presence of very few tandem repeats: SUBTILIS, PUMILUS, and MEGATERIUM. The HALODURANS group does not appear to be homogeneous and probably should be subdivided: Some species have many tandem repeats (group A), while other species have few tandem repeats (group B). Genome size and base composition are also different. The same situation is found in the METHANOLICUS group, which may also be divided in several groups, as suggested by Gupta et al. [16,17].

The SIMPLEX group has a considerable amount of tandem repeats, most of them with a 21 nt repeat, a characteristic feature of this group. We have detected other genomes with this feature (Supplementary Table S1), but we have been unable to place them in any of the groups shown in Table 5. We should also note that some authors combine MEGATERIUM and SIMPLEX in a single group [5]. Tandem repeats with a 52 nt repeat are a characteristic feature of the COAGULANS and HALODURANS-A groups, also found in some genomes in the METHANOLICUS group.

In summary, the distribution of tandem repeats among *Bacillus* species is not random. Each of the phylogenetic groups shown in Table 5 has a characteristic distribution of tandem repeats, which may help complement other phylogenetic studies. For example, the HALODURANS and METHANOLICUS groups may be clearly divided, depending on the presence or absence of 52 nt tandem repeats. In any case, it is clear that further work is required for a complete taxonomic classification, many species do not fit in any group: The genus *Bacillus* is in need of taxonomic revision [18]. In fact, Gupta et al. have recently suggested a division of *Bacillus* in 25 different groups [16,17].

## 4. Discussion

### 4.1. Unique Features of Tandem Repeats

An unexpected result of our study is the strict length conservation of tandem repeats. We have only detected families of tandem repeats of two repeat lengths (20–21 and 52 nt), with no sequence conservation. These tandem repeats have appeared in different groups of *Bacillus* and also in other *Firmicutes*. They are particularly abundant in the genus *Paenibacillus* [3], which is a clearly separate group from *Bacillus* [19]. The constant size of the repeats suggests a biological role for these tandem repeats, but there is no obvious function for them. In addition to our bioinformatic analysis, further experimental studies will be useful to answer this question. A possible explanation is that there is a protein or group of proteins which recognize some feature of the sequence and require a constant repeat size to polymerize on the DNA. Such protein-DNA complex might be involved in the stabilization of the bacterial nucleoid, as we have discussed in detail in our previous publication [3]. The presence of characteristic short sequences in all repeats (Table 2) gives support to this interpretation. An alternative is that these tandem repeats are transcribed as short RNAs, a hypothesis that we will discuss below.

**Figure 4:**
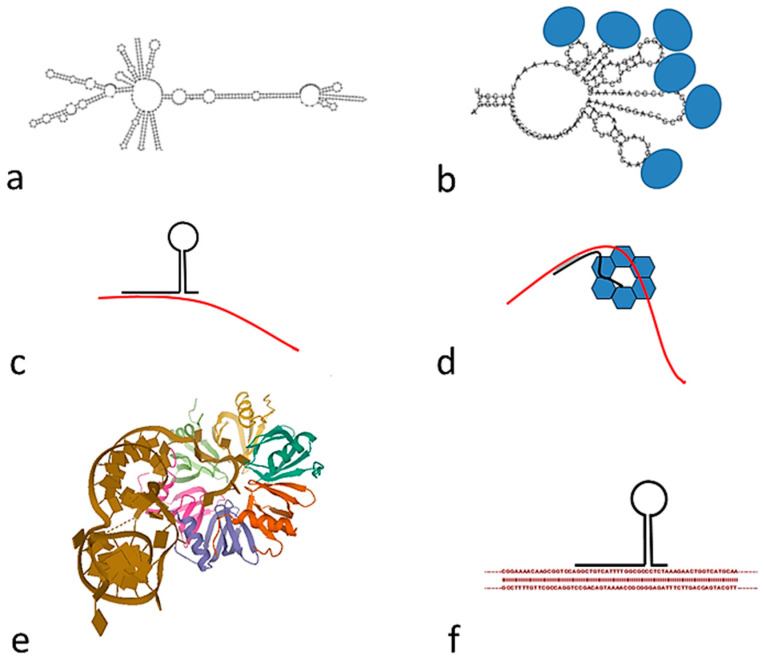
Models of transcribed RNA tandem repeats: (**a**) Model of an RNA with nine repeats of 52 nt, prepared with RNA; (**b**) RNA sponge: Fragment of folded RNA (five repeats) interacting with several proteins; (**c**) interaction of tandem repeat RNA (black) with messenger RNA (red); (**d**) interaction of tandem repeat RNA (black) with messenger RNA (red), facilitated by interaction with the Hfq protein hexamer; (**e**) model of the complex of a partially doubly stranded RNA and the Hfq protein, PDB code 4V2S [29]; (**f**) direct interaction of an RNA tandem repeat with DNA.

Bacteria contain hundreds of short RNAs with different lengths. They display many distinct mechanisms of action, usually through effects on target mRNA translation [20]. We wonder if tandem repeats may be transcribed and included in the pool of short RNAs. An analysis of the RNA-seq studies of *B. coagulans* [21] shows that the 52 nt tandem repeats are indeed transcribed. There are many RNA-seq studies of other *Bacillus* species, but we have not found any study with other species which contain 52 nt tandem repeats. However, it is generally accepted that most of the DNA in the genome of bacteria may be transcribed, including regions not coding for proteins. The 52 nt tandem repeats might be either completely or partially transcribed. Their structure may be either preserved or degraded as small RNAs [22].After transcription they may fold in different ways, as determined with RNAfold [23]; a substantial number of double-helical regions are found, as shown in Figure 4. 

Tandem repeat RNAs may interact directly with mRNA (Figure 4c). Many small non-coding RNAs exert their regulatory function by directly base pairing with mRNA targets to alter their stability and/or affect their translation [24]. This RNA-RNA interaction is often facilitated by the hexameric Hfq protein [25], as it is schematically shown in Figure 4d. Hfq is an abundant bacterial RNA binding protein, present in most bacterial species. It has many important physiological roles that are usually mediated by interacting with Hfq binding small RNAs. Hfq is now recognized as an RNA chaperone that interacts with many RNAs and plays crucial roles in riboregulation [26]. A crystallographic model of the interaction of an RNA fragment with Hfq [27] is shown in Figure 4e. This is not the only type of interaction, RNA may interact with the Hfq hexamer in multiple regions, involving both faces and the sides of the hexamer [26]. Thus, tandem repeat RNA might bind in multiple places, but there is no evidence for a requirement of a 52 nt repeat. Furthermore, Hfq may also bind directly to specific DNA sequences [28], we cannot exclude that it directly recognizes tandem repeat DNA.

Other mechanisms of action are possible for small RNAs, which will influence gene transcription [20]. They may have a role as an RNA sponge (Figure 4b), sequestering either Hfq or other proteins usually associated with mRNA [25]. They may also interact directly with double stranded DNA, as shown in Figure 4f.

### 4.2. Origin of Tandem Repeats and Comparison with Eukaryotes

The most general feature in bacteria is the very low number of tandem repeats in most species [3]. *Bacillus* genomes are exceptional, since many species present a significant number of tandem repeats (Figure 1). The number of repeats per Mb in these species is similar to that found in many eukaryotes. We will compare the different types of tandem repeats we have found in *Bacillus* with similar features in eukaryotic tandem repeats.

Tandem repeats coding for amino acid repeats are found in a small proportion in all species, but the prominent number found in the *B. cereus* group is unique. These species probably benefit from this feature to easily change the details of protein structure, this may help them invade different hosts and ecological niches. Other cases, similar to the tandem repeats coding for the 20 amino acid repeats we have described (Table 3), are also found in eukaryotes. The abundant tandem repeats with a repeat of 84 nucleotides found in mammals [10] are a clear example: They code for 28 amino acid repeats in zinc-finger proteins [30].

Tandem 20–21 nt repeats are present in a small proportion in many bacteria [3], including *Bacillus* (Supplementary Table S1). Unexpectedly, *B. simplex* and a few other species present a large proportion of these repeats. We have found no similar case in eukaryotes. The constant length of this repeat in different species indicates that they play some specific role. We can only note that this length corresponds to two turns of the DNA helix, which might indicate some function in either DNA replication or transcription.

The most unusual case we have detected are the constant length 52 nt repeats found in some *Bacillus* and in a few other *Firmicutes*. In spite of their constant size, they do not show any sequence conservation. They may play an important role associated to their constant size and may appear by chance in species in which their presence improves some of the functions we have discussed. The horizontal DNA transfer between closely related species [31] may also help spread these tandem repeats. Their sequence may then change by adapting to the new invaded genome. The horizontal gene transfer between closely related *Bacillus* species has been demonstrated [7,32]. A search of the *B. coagulans* genome indeed shows that it contains the gene for the ComEA protein required for the horizontal gene transfer [33]. In eukaryotes, we also find abundant tandem repeats of specific sizes, for example, in *Caenorhabditis elegans,* we find a predominance tandem repeat with a 35 nt repeat, not found in the related species [10].

We may also ask if the role played by tandem repeats is similar in eukaryotes and in *Bacillus*. Although the general role played by tandem repeats is not clear for any species, some similarities are worth mentioning. In the case of human tandem repeats [34], their role is only clear in some cases: The abundant alpha tandem repeats required in mitosis [35] are a clear example. In *C. elegans,* we also find the tandem repeats required for mitosis [10] and for meiotic chromosome pairing [36]. In a similar way, some bacterial tandem repeats may play a role in nucleoid structure and function [3]. Another related feature is the transcription of tandem repeats to RNA we have suggested in *Bacillus* (Figure 4), which has been clearly demonstrated in Drosophila [37]. It is likely that the transcription of tandem repeats may be found in other species, a question which deserves further investigation.

## 5. Conclusions

We have determined the tandem repeats and their families in 176 genomes of *Bacillus*. These genomes only present three types of tandem repeats: Repeats of either 20–21 or 52 nt and coding tandem repeats which are part of genes.

We describe in detail a unique group of tandem repeats with a repeat of 52 nt, with a strict conservation of length and variable sequence. We analyze their possible role as transcribed small RNA molecules.

The distribution of tandem repeats in different species of *Bacillus* is variable and differs substantially from eukaryotes: Some groups have very few tandem repeats, while other groups have a large proportion of tandem repeats.

Cereus group genomes are unique: They have a large proportion of tandem repeats which correspond to parts of the genes coding for repeats of amino acid stretches.

## Figures and Tables

**Table 1:** Main *Bacillus* 52 nt tandem repeat families. Sequence of repeats.

*Bacillus* Species	Number of Tandem Repeats	% 52 nt Repeat	Family Code	Consensus Repeat
*cellulosilyticus*	64	73.4	37_52_10	**gTGTaTCATACgaaggCAATGACACgtGAgAAAGtaGaaGaaacgnAATAAa**
			61_52_6	**CAcTCAACGAAGGTcATCATAAGcAAGCAATGCTaCCCCAAAACCAAAcCcn**
*coagulans*	53av	90.8	1_52_139	**GTgAAGgAAGgcCnTCnTTTTTcCncGCTTcCTTAACGTAGACGcgCTCTAT**
			2_52_35	**TTTTGTCCTTTTGaCaGcTTCAAAAnGACATTTCGgGCCCgGATgCAgCntG**
			8_52_18	**TGTCCTTCATaagggtGATGAAaGACAAAACaCnGGcCgggaAAcGgCgAAt**
*horikoshii*	64	71.9	22_52_12	**ATGAAGACaTcAGTGAcGAGaAAtcAGGAGgAGAGAaGTCcTCATcGncGTt**
*kochii*	59	88.1	52_51_7	**TTCnTTCTGAGTGACtTGCtAatCCcTTTTGCGAAGCgCTCAnCtCtgGtt**
			92_54_4	**CTTTTaTTAGTcGCGaTTcTcacTTTTgCcCTACTtATCttnTcacTnatTCTT**
*litoralis*	80	73.7	42_52_9	**TGTCGTTCATaAGggtgATGAACGACAAAAGtGnTnaGAAAAGagngnGtag**
			77_52_5	**AATCGGGACAGAAAAaaGAgcgaGCAGtgaAAnTgaGTCTCGATAgTgggng**
*oceanisediminis*	38	84.2	79_52_5	**nCgCcaAcTTCGGACTCatTCtCtCggTTTTccgcttCTtCTGTCCGAAgtn**
			96_54_4	**cGATTAACTACCATTTTnCctTncaCCnnccTcaTTTTCGtCGTtAatcCaTcc**
*thermoamylovorans*	70	78.6	4_52_21	**AAAAtGacGACGAGAAnnGGTCTCGTCGCCAAAAAatGGAGTTTTcCGgctc**
			5_52_21	**TTGgCGACGAGaCcnatTCTCGTCaCCaTTTTgaGGtGAAAAAnGCtCnaTT**
			30_53_11	**TGTCCAATAGAACGGcTCTCgTGGACAAAATnGAGgnnTCAATCAGgAAAAAc**
New species				
*pseudalcaliphilus*	103	92.2	3_50_31	**gAATCnCGGGGTTGCGaGCnGAAAAAGaGGAGAAAgCCCGAGCAAAcGcn**
			5_52_25	**GTTAATGTGaAGATACgGAgGCcAAACcttGgAGTAtCTGCACAAAGAGggG**
			26_53_11	**nGtTGGTCGACATGATCAtGgGnAAAAAAGGncaAGAACcTGTCGATGAaGGn**
*alkalitelluris*	91	74.7	4_52_27	**AAgGGAATCAaACAAcgCtTTTCaTTCCCTTTgttggGCTTTtGGCATgAGA**
			71_52_7	**TGTCTGAAGTaGCCTnGnGTTCGGACAGcTTTgaTtgtTTnnaaGcnAAAGC**
			72_52_7	**CATAGgCctTCTATGATtcAGTTGCcgaaGCgAAAACAAGGAGnAAGTgAAT**
*indicus*	90av	96.6	7_52_18	**ATCGTACCCTcgnAAACcGaaAAaCGATnTgggAGGGTAAGCAAanGcnnGA**
*alcalophilus*	103	86.4	9_52_16	**CCTtTGATTCCCTTTncGGCTtTtATTcaatgGCTTTtGgcaTCATTGcnGn**
			11_51_15	**TTTTCATTACcTATtcCnntTTaTTCGCACCcTAATtcnncaCAgCnCgGc**
			41_51_9	**TTTTTCATTACcTATcCncnnTTTTTCGCACcnTAATTTggcCtgCtcggC**
sp.m3-13	72	73.6	20_52_13	**ATGAAGACnTcAGTGACgAggAAaaAGGAGgAGAGAaGTCCTCATcGccGTt**
sp.SG-1	77	81.8	42_53_9	**CanaCCAACAtCcCTcncAtAATcCatTCTCaTTGGnctGaTTActCCcTTTT**
*selenatarsenatis*	82	59.8	Many	
*mesonae*	95	88.4	Many	

In this table, the main families with the 52 nt repeat in each species are shown. We also include the results obtained in our previous study [3]. New species are those added in the present work. Species which contain many small tandem repeat families are indicated by “many”. Tandem repeats in B. *selenatarsenatis* are shared with two closely related species: *B. boroniphilus* and *B. subterraneus.*

**Table 2:** Characteristic signals in tandem repeats.

*Bacillus* Species	NCBI Code	Repeat (nt)	Characteristic Signals
*oagulans*	NZ_CP026649.1	52	TCTAYG AARGACA
*cellulosilyticus*	NC_014829.1	52	GGTCATCAT CAATGCT ACGAAGG ATGACAC
*alkalitelluris*	NZ_KV917374.1	52	AAAgGGAAT AAAGCTGTC AATCATAG
*mesonae*	NZ_KV440949.1	52	TTTTC TTCAT
*weihaiensis*	NZ_CP016020.1	21	TCGCGG

**Table 3:** Coding features of *Bacillus* tandem repeats with a repeat length of 60 nt.

NCBI Code	*Bacillus* Species	Tandem Repeat		Protein Gene		
		Start	Length	Start	Length	NCBI Code
NC_015634.1	*coagulans*	2718394	301	2718351	447	WP_013860576
NZ_CP026649.1	*coagulans*	Heavily mutated		3215973	387	WP_035183339
NZ_LT603683.1	*glycinifermentans*	580499	301	580438	438	WP_065894177
NC_006582.1	*clausii*	3644328	241	3644228	402	WP_011248345
NC_017190.1	*amyloliquefaciens*	438961	301	438923	369	WP_014471456
NC_014551.1	*amyloliquefaciens*	456884	241	456846	309	WP_013351072
NC_006322.1	*licheniformis*	540638	301	540531	441	WP_011197566
NZ_CP007640.1	*atrophaeus*	4062283	301	4062244	375	WP_010789649
NC_000964	*subtilis*	494545	301	494506	372	WP_003246542

**Table 4:** Distribution of tandem repeats in different groups of *Bacillus*.

NCBI Code	*Bacillus* Species	Genome Size (Mb)	GC%	Number of Tandem Repeats with a Given Repeat Size					
				Total	10–19	20–21	22–50	51–53	>53
	**Rich in 52 nt repeat**								
NZ_KV917374.1	*alkalitelluris*	5.43	36.4	91	7	7	3	**69**	5
NC_014829.1	*cellulosilyticus*	4.68	36.5	64	7	0	2	**47**	8
NC_015634.1	*coagulans*	3.07	47.3	26	0	0	1	**21**	4
NC_016023.1	*coagulans*	3.55	46.5	63	0	1	1	**57**	4
NZ_CP023704.1	*thermoamylovorans*	4.02	37.5	70	2	4	7	**55**	2
	**Intermediate**								
NC_022524.1	*infantis*	4.88	46	50	2	**12**	8	**27**	1
NZ_BASE01000145	*selenatarsenitis*	4.76	42.1	82	1	**30**	1	**49**	1
	**Rich in 21 nt repeat**								
NZ_CP016020.1	*weihaiensis*	4.36	36.5	32	2	**29**	0	0	1
NZ_CP011008.1	*simplex*	5.52	39.8	40	3	**27**	9	0	1
NZ_CP017080.1	*muralis*	5.01	42.3	38	1	**22**	13	0	2
	***Cereus* group**								
NC_004722.1	*cereus*	5.51	35.3	25	9	1	**15**	0	0
NZ_CP018931.1	*cereus*	5.24	35.4	31	5	3	**21**	0	2
NZ_CP007512.1	*bombysepticus*	5.88	35.0	31	9	4	**15**	0	3
NC_003997.3	*anthracis*	5.23	35.4	19	3	5	**11**	0	0
NZ_CP009692.1	*mycoides*	5.64	35.4	26	7	2	**15**	0	2
	**Tandem repeat poor**								
NC_014103.1	*megaterium*	5.1	38.1	**8**	7	1	0	0	0
NC_017138.1	*megaterium*	5.08	38.1	**5**	2	2	0	0	1
NZ_CP011007.1	*pumilus*	3.88	41.5	**4**	0	0	4	0	0
NC_014551.1	*amyloliquefaciens*	3.98	46.1	**3**	1	0	0	0	2
NC_000964.3	*subtilis*	4.22	43.5	**1**	0	0	0	0	1
NZ_CP012024.1	*smithii*	3.38	40.8	**0**					
NZ_CP012502.1	*beveridgei*	3.58	46.1	**0**					
NZ_CP017786.1	*xiamenensis*	3.64	41.5	**0**					

Only a few examples of each group are shown. The characteristic feature of each group is enhanced in bold.

**Table 5:** Tandem repeats in different phylogenetic groups of *Bacillus*.

Group	Genome (Mb)	CG%	Number of Tandem Repeats	52 nt Tandem Repeats
CEREUS	5.5	35.4	25.7	NO
SUBTILIS	4.2	44.9	3.8	NO
PUMILUS	3.8	41.3	3.2	NO
METHANOLICUS	3.3–6.4	36–42	12–54	Variable
MEGATERIUM	3.9–5.5	35–38	2–7	NO
SIMPLEX	4.6–5.5	39–42	12–40	NO
HALODURANS-A	4.6	38.7	80.3	YES
HALODURANS-B	4.1	42.3	4	NO
COAGULANS	3.6	37–46	56	YES
MISCELLANEOUS	3.2–5.3	33–45	0–49	Variable

Average values are given when the group is homogeneous. Details are given in Supplementary Table S6. The SIMPLEX group is the only group characterized by the presence of abundant tandem repeats with a 21 nt repeat.

## Data Availability

The sequence of all tandem repeats and a list of all tandem repeat families and their members are available in the Supplementary materials.

## Supplementary Materials

The following are available online at https://www.mdpi.com/article/10.3390/ijms22105373/s1. Figure S1: Histograms of tandem repeat lengths; Figure S2: Comparison of genome regions; Supplementary Table S1: Genome properties. Distribution of repeat sizes; Supplementary Table S2: Sequence of all tandem repeats; Supplementary Table S3: Tandem repeat families, including all tandem repeats; Supplementary Table S4: List of *Bacillus* tandem repeat families; Supplementary Table S5: Examples of complete tandem repeat sequences; Supplementary Table S6: *Bacillus* Phylogeny.
