# Supplementary material for: Tandem Repeats in Bacillus: Unique Features and Taxonomic Distribution
Source: Int J Mol Sci. 2021 May 20;22(10):5373. doi: 10.3390/ijms22105373 (PMC8161180; doi:10.3390/ijms22105373)

Figure S1. Histograms showing the number of 52 nt repeats in individual tandem repeats (satellites) of selected species. The 21 class includes all tandem repeats with more than 20 repeats.

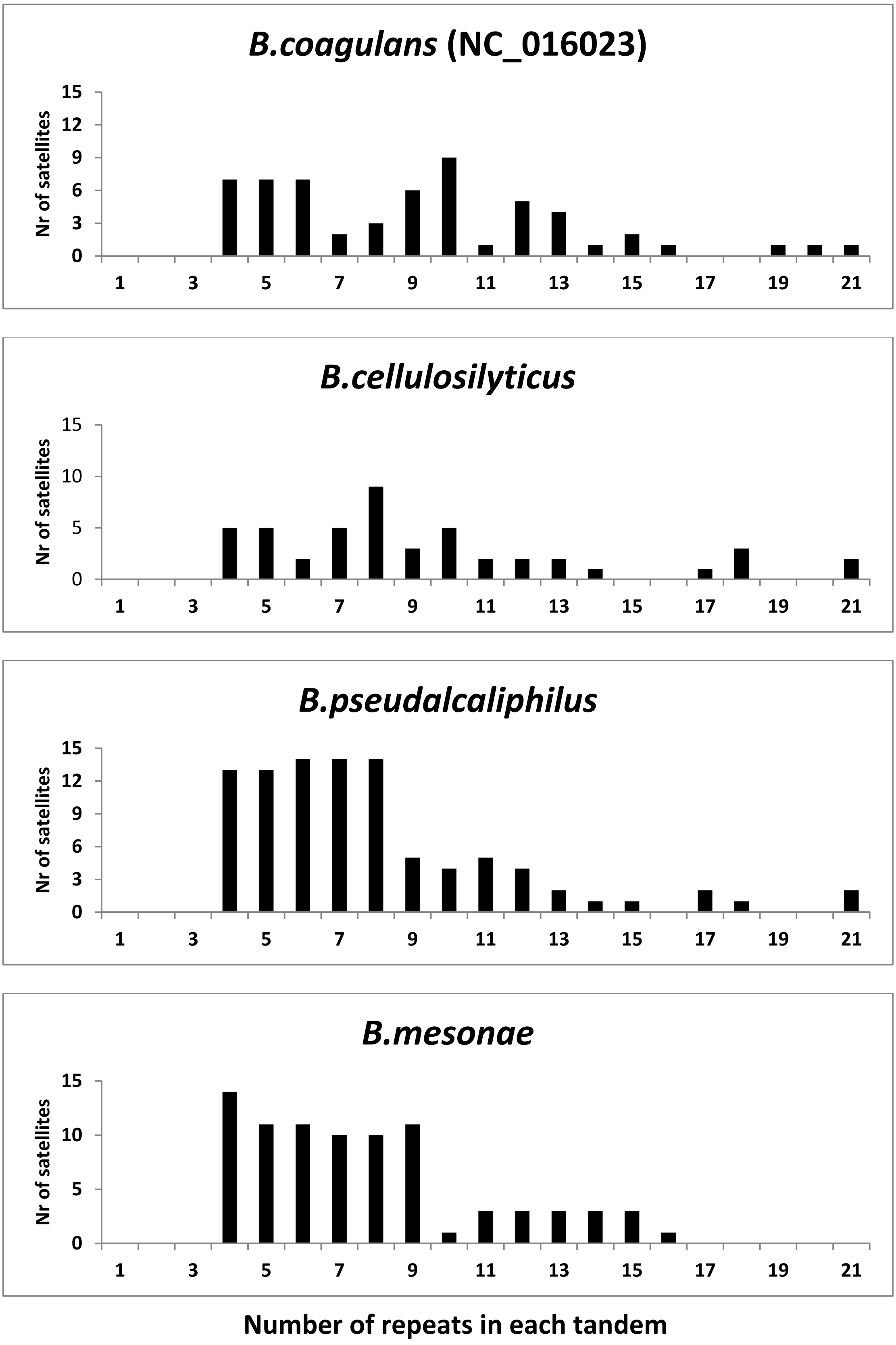

Supplement: Supplementary file 1 [file ijms-22-05373-s001.zip › Figure S1.Histograms of tandem repeat lengths.pdf]
