# Supplementary material for: Tandem Repeats in Bacillus: Unique Features and Taxonomic Distribution
Source: Int J Mol Sci. 2021 May 20;22(10):5373. doi: 10.3390/ijms22105373 (PMC8161180; doi:10.3390/ijms22105373)
Supplement: Supplementary file 1 [file ijms-22-05373-s001.zip › Figure S2.Comparison of genome regions.pdf]

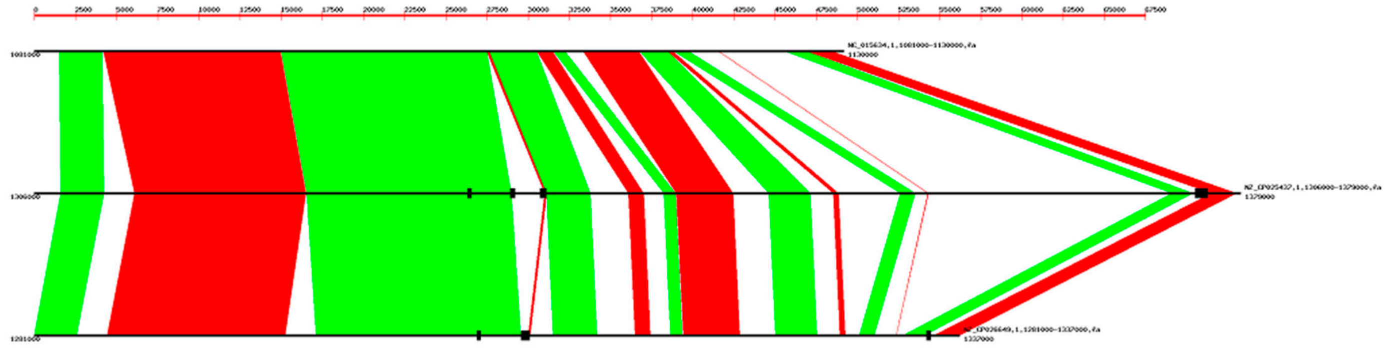

A

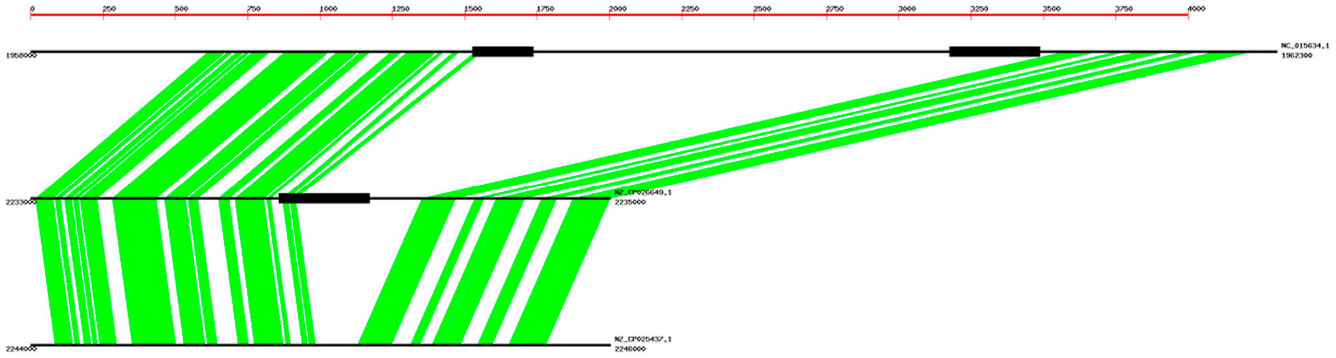

B

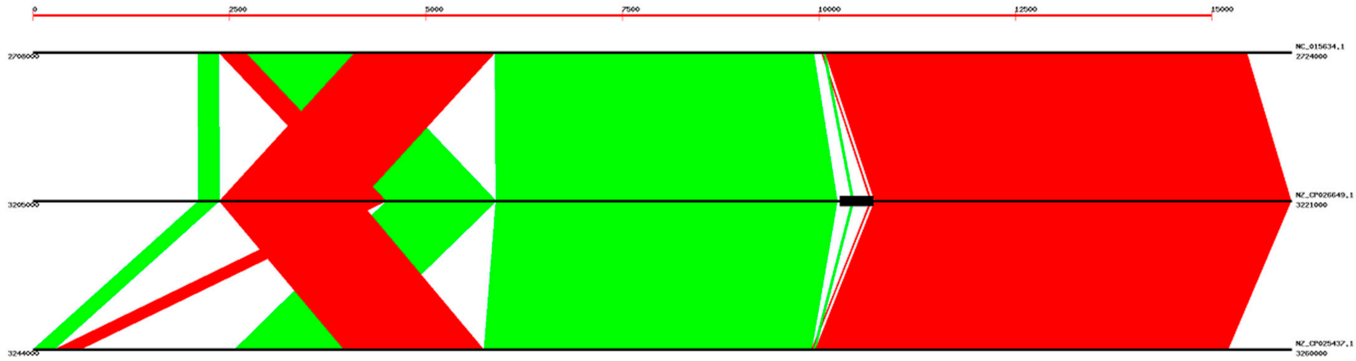

C

Figure S2. Comparison of selected genome regions of 3 strains of *B. coagulans*. (A), A small tandem repeat is found at the center of the upper NC_015634 genome, not visible in the drawing. Corresponding tandem repeats are found in the other two genomes, but they differ in length. A comparison of sequences is shown in the next page. (B), The tandem repeat in NC_015634 is broken in two by a non-repetitive region. A corresponding tandem repeat is only found in one of the other two genomes. (C), A tandem repeat is only found in one genome. A few bases at right a gene is found for a stress protein, which appears as a 60 nt repeat tandem repeat at the corresponding region of NC_015634.

Here the sequences of the central tandem repeat in the A frame are shown. The characteristic signals in the sequence are underlined. In **NC_025437** two tandem repeats

are present, separated by an unrelated sequence. Only a small portion of the sequence (shown in red) is identical with the corresponding tandem repeat in **NZ_CP026649.**

NC_015634 (Tandem repeat not shown in the figure)

> ACCGGAGCAA Con:1 reps=6 [1108127,1108450] l-int=324 l-seq = 52
ACCGGAGCAAATTGTTCTTTTAGAGCATTCAAAGAGACAAAAGGAGCCATGA
ACCGGAGCAAATTGTTCTTTTGGAGCATTCAAAAAGACAAAAGGAGCCCGTG
GACCGGAGCAATCGTCCTTTTGGAGCATTCAA**AAGGACA**AAAGAACTCGTGA
ACCGGAGCAAATTGTTCTTTTGGAGCATTCAA**AAGGACA**AAAGGAGCCATGA
ACCGGAGCAAAATGTCTTTTTGGAGTATTCAA**AAGGACA**AAAGAACTCGTGG
ACCGGAGCAAAT**TGTCCTT**TTAGAGCATTCAAAAAGACAAAAGGAGCCCGTA

NZ_CP026649

> CAAAAGGACA Con:1 reps=10 [1310475,1311005] l-int=531 l-seq = 52
CAA**AAGGACA**AAACACGCTGCATCCGGGCCCGAAATGTCTTTTTGAAGCTGC
CAA**AAGGACA**AAACACGTTGCATCTGGTCTCGGAA**TGTCCTT**TTGAAGCTGC
CAA**AAGGACA**AAACGCGTTGCATCTGGGATCGGAA**TGTCCTT**TTGAAGCAGT
CAA**AAGGACA**AAAGGAGCCGTGAACTGAAACAAAA**TGTCCTT**TTGAAGCTGT
CAA**AAGGACA**AAAGGAGCCGTGGACCGGAGCAAAAAGTCCTTTTGGAGTATT
CAA**AAGGACA**AAAGGAGCCGTGGACCGGAGCAAAAAGTCCTTTTGGAGTATT
CAA**AAGGACA**AAAGGAGCCGTGAACCAGAGCAAAAAGTCCTTTTGGAGTATT
CAA**AAGGACA**AAAGGAGCCGTGAACTGAAACAAAA**TGTCCTT**TTGAAGCTGT
CAA**AAGGACA**AAAGGAGCCGTGGACCGGAGCAAATTGTTCTTTTGGAGCATC
CAA**AAGGACA**AAAGGAGCCATTGACCGGAACAAAATGTCTTTTTGGAGTGTT

NZ_CPO25437

> AAAAGGACAA Con:1 reps=6 [1334805,1335122] l-int=318 l-seq = 52
AA**AAGGACA**AATGAAATCGCGGGACCATAGCAAATTTTTTTTGGAGTATTC
--AAGACAAAAAGGAGCCGCACCTAGGCCTGAAATGTTTTTTTCAAGCTGTC
AA**AAGGACA**AAACACCCTGCATCCGGGCTCGGAATGTCTTTTTGAAGCTGCC
AA**AAGGACA**AAACACGTTGCATCTGGGCTCGGAA**TGTCCTT**TTGAAAGTC
AA**AAGGACA**AAACAAGCTGTATCTGGGCCCGAAATGTCTTTTTGAAGCTGTC
AA**AAGGACA**AAACAAGCTGTATCTGGTCTCGGAA**TGTCCTT**TTGAAGCTGCC
> CAAAAGGACA Con:1 reps=7 [1336628,1337002] l-int=375 l-seq = 52
CAA**AAGGACA**AAACAAGCTGTATCTGGGCTCGGAA**TGTCCTT**TTGAAGCTGC
CAA**AAGGACA**AAACACGTTGCATCCGGTCCCGAAA**TGTCCTT**TTGAAGCAGT
CAA**AAGGACA**AAACGCGTTGCATCGGGGTCCAAAATGTCTTTTTGAAGCTGT
CAA**AAGGACA**AAACAAGCTGTATCTGGGCTCGGAA**TGTCCTT**TTGAAGCTGC
CAA**AAGGACA**AAAGGAGCCGTGAACTGAAACAAAA**TGTCCTT**TTGAAGCTGT
CAA**AAGGACA**AAAGGAGCCGTGGACCGGAGCAAATTGTTCTTTTGGAGCATC
CAA**AAGGACA**AAAGGAGCCATTGACCGGAACAAAATGTCTTTTTGGAGTGTT
