# Supplementary material for: Tandem Repeats in Bacillus: Unique Features and Taxonomic Distribution
Source: Int J Mol Sci. 2021 May 20;22(10):5373. doi: 10.3390/ijms22105373 (PMC8161180; doi:10.3390/ijms22105373)
Supplement: Supplementary file 1 [file ijms-22-05373-s001.zip › Table S3.Tandem repeat families, including all tandem repeats.pdf]

```
The program is running under the following parameters:
   Output:
      Prefix of the family name Bacillus
      Circular permutations are allowed
   Reverse sequences are considered.
   Sequences are aligned while similarity: 0.600000
   Alignment score: Normalized respect to the length and number of
sequences.
   Alignment parameters:
      opening gap: -3.000000
      extensio gap: -1.000000
      extreme gaph: -0.500000


 ************************************************************
Number of sequences=8058
There are 1967 alignments:

>Bacillus_Fam_1_60_41  Nr. of seq. 41 Alignment length(with gaps) = 64
Alignment score = 0.721735
Bacillus_murimartini_Bacillusgenomic.fna_7:140667-140967 Satlength=301 Nr
of Repeats=5 RepeatLength=60 seed=TCCGCCTTTT  Num.seqs=5
Similarity=0.815556
0    TCCGCC-TTTTTNG-CCGATTTCTTGATAGAATTCTTTGTCATGGTT-TTT-GCTAGTGGCTTC
Rev.of_GCF_000245335.1_ASM24533v1_genomic.fna_8:299494-299734
Satlength=241 Nr of Repeats=4 RepeatLength=60 seed=AAAGGCGGAG  Num.seqs=4
Similarity=0.790984                                               9
TCCGCC-TTTTTCG-CCGATTTCTTGATAGAATTCTTTGTCATGGTT-TTT-GCTAGTGGCTTC
Rev.of_GCF_001517115.1_ASM151711v1_genomic.fna_21:315882-316122
Satlength=241 Nr of Repeats=4 RepeatLength=60 seed=AAAGGCGGAG  Num.seqs=4
Similarity=0.816485                                              9
TCCGCC-TTTTTCG-CCGATTTCTTGATAGAATTCTTTGTCATGGTT-TTT-GCTAGTGGCTTC
Rev.of_GCF_000009045.1_ASM904v1_genomic.fna_1:494545-494845 Satlength=301
Nr of Repeats=5 RepeatLength=60 seed=AAAAGGCGGA  Num.seqs=5
Similarity=0.800546                                                10
TCCGCC-TTTTTCA-CCGATTTCTTGATAGAATTCTTTGTCATGGTT-TTT-GCTAGTGGCTTC
Rev.of_GCF_001719185.1_ASM171918v1_genomic.fna_1:3366512-3366812
Satlength=301 Nr of Repeats=5 RepeatLength=60 seed=AAAAGGCGGA  Num.seqs=5
Similarity=0.800546                                           10
TCCGCC-TTTTTCA-CCGATTTCTTGATAGAATTCTTTGTCATGGTT-TTT-GCTAGTGGCTTC
GCF_001517135.1_ASM151713v1_genomic.fna_12:117121-117361 Satlength=241 Nr
of Repeats=4 RepeatLength=60 seed=TTCTCCGCCT  Num.seqs=4
Similarity=0.837037                                                   57
TCCGCC-TTTTTCG-CCGATTTCTTGATAGAATTCTTTATCATGGTT-TTT-GCTAGTGGCTTC
GCF_000245315.1_ASM24531v1_genomic.fna_74:144784-145024 Satlength=241 Nr
of Repeats=4 RepeatLength=60 seed=TCTCCGCCTT  Num.seqs=4
Similarity=0.833333
58    TCCGCC-TTTTTCG-CCGATTTCTTGATAGAATTCTTTGTCATGGTT-TTC-GCTAGTGGCTTC
GCF_000507145.1_KCTC_13622_01_genomic.fna_31:58182-58422 Satlength=241 Nr
of Repeats=4 RepeatLength=60 seed=TTCTCCGCCT  Num.seqs=4
Similarity=0.803704                                                  117
TCCGCC-TTTTTCG-CCAATTTCTTGATAGAATTCTTTGTCATGGTT-TTC-GCTAGTGGCTTC
Rev.of_GCF_000769555.1_ASM76955v1_genomic.fna_1:447703-447943
Satlength=241 Nr of Repeats=4 RepeatLength=60 seed=AAAAAGGCGG  Num.seqs=4
```

Similarity=0.840741 11
TCCGCC-TTTTTGG-CCGATTTCTTGATAGAATTCTTTGTCATGGTT-TTT-GCTTGTGGCTTC
Rev.of_GCF_000742675.1_ASM74267v1_genomic.fna_1:4062283-4062583
Satlength=301 Nr of Repeats=5 RepeatLength=60 seed=AAAAGGCGGA Num.seqs=5
Similarity=0.886667 10
TCCGCC-TTTTTCG-CCAATCTCTTGATAGAATTCTTTGTCGTGGTT-TTT-GCTAGTGGCTTC
Rev.of_GCF_000196735.1_ASM19673v1_genomic.fna_1:456884-457124
Satlength=241 Nr of Repeats=4 RepeatLength=60 seed=AAAAAGGCGG Num.seqs=4
Similarity=0.881481 11
TCCGCC-TTTTTGA-CCGATTTCCTGATAGAATTCTTTGTCGTGATT-TTT-GCTTGTGGCTTC
Rev.of_GCF_000204275.1_ASM20427v1_genomic.fna_1:438961-439261
Satlength=301 Nr of Repeats=5 RepeatLength=60 seed=AAAAAGGCGG Num.seqs=5
Similarity=0.893333 11
TCCGCC-TTTTTCA-CCGATTTCCTGATAGAATTCTTTGTCATGATT-TTT-GCTTGTGGCTTC
GCF_000262045.1_KCTC_13613_01_genomic.fna_37:124118-124418 Satlength=301
Nr of Repeats=5 RepeatLength=60 seed=TCCGCCTTTT Num.seqs=5
Similarity=0.866667 60
TCCGCC-TTTTTGA-CCGATTTCTTGATAGAATTCTTTGTCATGATT-TTT-GCTTGTGGCTTC
Rev.of_GCF_000008425.1_ASM842v1_genomic.fna_1:540638-540938 Satlength=301
Nr of Repeats=5 RepeatLength=60 seed=AAAAAGGCGG Num.seqs=5
Similarity=0.844444 11
TCCGCC-TTTTTCG-CCGATTTCCTGGTAGAATTCTTTGTCATGGTT-GCG-GCTTGTGGCTTC
GCF_000186125.1_Bacillus_BT1B_CT2_V1_genomic.fna_3:99314-99614
Satlength=301 Nr of Repeats=5 RepeatLength=60 seed=CCGCCTTTTT Num.seqs=5
Similarity=0.840000 61
TCCGCC-TTTTTCG-CCGATTTCCTGGTAGAATTCTTTGTCATGGTT-TTG-GCTTGTGGCTTC
Rev.of_GCF_000408885.1_ASM40888v1_genomic.fna_1:560702-560942
Satlength=241 Nr of Repeats=4 RepeatLength=60 seed=AAAGAATTCT Num.seqs=4
Similarity=0.881481 96
TCCGCC-TTTTTCG-CCGATTTCCTGGTAGAATTCTTTGTCATGATT-TTG-GCTTGTGGCTTC
Rev.of_GCF_000009825.1_ASM982v1_genomic.fna_1:3644328-3644568
Satlength=241 Nr of Repeats=4 RepeatLength=60 seed=AAAGAATTCT Num.seqs=4
Similarity=0.859259 36
TCCGCC-TTTTTCG-CCAATTTCTTGGTAGAATTCTTTGTCGTGGTT-GTG-GCTAGTAGCTTC
Rev.of_GCF_900142675.1_IMG-
taxon_2654588196_annotated_assembly_genomic.fna_7:422068-422308
Satlength=241 Nr of Repeats=4 RepeatLength=60 seed=AAAGAATTCT Num.seqs=4
Similarity=0.862963 36 TCCGCC-TTTTTCG-
CCAATTTCTTGGTAGAATTCTTTGTCGTGGTT-GTG-GCTAGTAGCTTC
Rev.of_GCF_900093775.1_EVONIK_BGLY_genomic.fna_1:580499-580799
Satlength=301 Nr of Repeats=5 RepeatLength=60 seed=ACAAGCAAAA Num.seqs=5
Similarity=0.807650 54
TCCGCC-TTTTTCA-CCGATCTCCTGGTAGAATTCTTTATCGTGGTT-TTT-GCTTGTTGCTTC
Rev.of_GCF_000217835.1_ASM21783v1_genomic.fna_1:2718394-2718694
Satlength=301 Nr of Repeats=5 RepeatLength=60 seed=AAAGGCGGAG Num.seqs=5
Similarity=0.811111 9
TCCGCC-TTTTTTG-CCGATTTCTTGATAGAATTCTTTATCATGNTT-TTCNGC-AGTAGNTTC
Rev.of_GCF_000832905.1_ASM83290v1_genomic.fna_1:1791266-1791506
Satlength=241 Nr of Repeats=4 RepeatLength=60 seed=GAATTCTATC Num.seqs=4
Similarity=0.788889 33
TCCGCC-TTTTTTG-CCGATTTCCTGATAGAATTCTTTGTCATGGTT-TTCAGC-AGTGGCTTC
GCF_001700275.1_ASM170027v1_genomic.fna_26:46937-47426 Satlength=490 Nr
of Repeats=4 RepeatLength=60 seed=GCTTCGCCGC Num.seqs=3

Similarity=0.940741
55 GCCGCC-TTTTTTG-CCGATTTCTTGATAGAAGTCTTTGTCGTGGTT-TTCAGC-AGTGGCTTC
Rev.of_GCF_000812025.2_ASM81202v2_genomic.fna_65:14068-14964
Satlength=897 Nr of Repeats=6 RepeatLength=60 seed=GGAGAAGCTA Num.seqs=4
Similarity=0.707650 63
TCCGCC-TTTNTGG-CCGATTTCTTGGTAGAATTCTTTATCGTGGTT-TTCGGC-GGTAGCTTC
Rev.of_GCF_000374345.1_ASM37434v1_genomic.fna_32:2694-2934 Satlength=241
Nr of Repeats=4 RepeatLength=60 seed=AAAGGCGGAG Num.seqs=4
Similarity=0.740741 9
TCCGCC-TTTTTGG-CCGATTTCCTGATAGAATTCTTTGTCATGTTT-GTCAGC-TGTCGTTTC
GCF_000430765.1_ASM43076v1_genomic.fna_12:86711-87701 Satlength=991 Nr of
Repeats=11 RepeatLength=60 seed=GCTTCTCCGC Num.seqs=9
Similarity=0.760494
55 TCCGCC-TTTTTTG-CCAATTTCCTGGTAGAATTCTTTGTCATGATT-CTCNGA-AGTAGCTTC
GCF_001591545.1_ASM159154v1_genomic.fna_1:1026037-1026277 Satlength=241
Nr of Repeats=4 RepeatLength=60 seed=GTAGCTTCTC Num.seqs=4
Similarity=0.829630 112
TCCACC-TTTTTGG-CCGATTTCCTCATAAAAGTCTTTGTCATGATT-TTCGGA-AGTAGCTTC
Rev.of_GCF_001587375.1_ASM158737v1_genomic.fna_44:3736-4036 Satlength=301
Nr of Repeats=5 RepeatLength=60 seed=GGAGAAGCTA Num.seqs=5
Similarity=0.793333 64
TCCACC-TT-TTTGACCAATTTCTTGATAGAATTCTTTGTCATGGTT-TTTNGC-GGTAGCTTC
Rev.of_GCF_001645705.1_ASM164570v1_genomic.fna_56:31150-31450
Satlength=301 Nr of Repeats=5 RepeatLength=60 seed=CATGATAAAG Num.seqs=5
Similarity=0.911111 103
TCCACC-TT-TTTGTCCGATTTCTTGATAGAATTCTTTATCATGATT-CTTGGC-AGTAGCTTC
GCF_000181495.1_ASM18149v1_genomic.fna_185:150083-150323 Satlength=241 Nr
of Repeats=4 RepeatLength=60 seed=TGATAGAATT Num.seqs=4
Similarity=0.844444 22
TCCGCC-TTTTTCA-CCAATCTCCTGATAGAATTCTTTATCATGGTT-ATTAGA-CGTTGCTTC
GCF_000473245.1_ASM47324v1_genomic.fna_1:4494162-4494402 Satlength=241 Nr
of Repeats=4 RepeatLength=60 seed=GCTTCTCCGC Num.seqs=4
Similarity=0.763441 55
TCCGCC-TTTTTGA-CCGATCTCCTGATAGAATTCCTTGTCATGGTT-TTCAGA-AGTAGCTTC
Rev.of_GCF_000153365.1_ASM15336v1_genomic.fna_17:33525-33765
Satlength=241 Nr of Repeats=4 RepeatLength=60 seed=GGCGGAGAAG Num.seqs=4
Similarity=0.749559 66
TCCGCCNTTTTTGG-CCGATTTCCTGATAGAATTCTTTGTCATGGTT-TTCAGA-AGTTGCTTC
Rev.of_GCF_001636425.1_ASM163642v1_genomic.fna_1:2982966-2983206
Satlength=241 Nr of Repeats=4 RepeatLength=60 seed=AAAGGCGGAG Num.seqs=4
Similarity=0.751852 69
TCCGCC-TTTTTGT-CCAATTTCCTGATAGAATTCTTTGTCATGGCT-TTTAGA-AGTTGCTTC
Rev.of_GCF_002019635.1_ASM201963v1_genomic.fna_3:166192-166432
Satlength=241 Nr of Repeats=4 RepeatLength=60 seed=TTCTATCAAG Num.seqs=4
Similarity=0.900000 90
TCCGCC-TTTTTGT-CCTATTTCTTGATAGAATTCCTTATCATGATT-TTCAGA-AGTCGCTTC
GCF_001243895.1_Bacillus_testis_genomic.fna_9:994577-995012 Satlength=436
Nr of Repeats=7 RepeatLength=60 seed=GCTTCTCCGC Num.seqs=6
Similarity=0.824408 55
TCCGCC-TTTTTCG-CCGATTTCTTGATAGAATTCTTTGCCATGGCT-ATCAGA-AGTAGCTTC
Rev.of_GCF_001375535.1_Bacillus_niameyensis_genomic.fna_6:3621472-3621832
Satlength=361 Nr of Repeats=6 RepeatLength=60 seed=GGTGGAGAAG Num.seqs=6
Similarity=0.833333 66 TCCACC-
CTTTTGA-CCGATCTCTTGATAGAATTCCTTATCATGATT-CTCAGC-CGTTGCTTC

GCF_001578185.1_ASM157818v1_genomic.fna_1:4250078-4250593 Satlength=516
Nr of Repeats=5 RepeatLength=60 seed=CTCCGCCTTT Num.seqs=4
Similarity=0.918519 119
TCCGCC-TTTTTGG-CCAATCTCTTGATAAAATTCCTTATCATGATT-TTCGGA-TGTTGCTTC
Rev.of_GCF_001636315.1_ASM163631v1_genomic.fna_1:3918880-3919180
Satlength=301 Nr of Repeats=5 RepeatLength=60 seed=CATGATAAAG Num.seqs=5
Similarity=0.915556 162
TCCGCC-TTTTCGT-CCAATCTCTTGATAAAAATCTTTATCATGATT-CTCGGC-TGTTGCTTC
Rev.of_GCF_900098925.1_PRJEB15625_genomic.fna_19:237981-238620
Satlength=640 Nr of Repeats=6 RepeatLength=60 seed=GGCGGCGAAG Num.seqs=4
Similarity=0.739982 66
GCCGCC-TTTTTGT-CCGATTTCCTGGTAAAATTCTTTATCATGATT-TTTGGA-AGTTGCTTC
GCF_900111815.1_PRJEB17078_genomic.fna_1:2949352-2949652 Satlength=301 Nr
of Repeats=5 RepeatLength=60 seed=GCTTCGCCGC Num.seqs=5
Similarity=0.815301 115
GCCGCC-TTTTTTA-CCGATCTCCTGGTAGAATTCTTTGTCATGATT-TTCNGC-TGTTGCTTC
GCF_001654695.1_ASM165469v1_genomic.fna_22:26665-26893 Satlength=229 Nr
of Repeats=4 RepeatLength=60 seed=TTTTTCTCCA Num.seqs=3
Similarity=0.866667
54   TCCACC-TTTTCGA-CCAATCTCTTGGTAAAATTCTTTATCATGTTC-CTCAGC-TGTTTTTTC
Rev.of_GCF_001889165.1_ASM188916v1_genomic.fna_1:1762840-1763080
Satlength=241 Nr of Repeats=4 RepeatLength=60 seed=ACAGCAAGAG Num.seqs=4
Similarity=0.840741 54
TCCGCC-CTTTTGG-CCAATCTCTTGATAGAATTCCTTATCATGCTCTCT-TGC-TGTTTTTTC

** ** *   ** ** ** * ** ** ** ** * **   *   **   ***
Consensus:

TCCGCCTTTTTngCCgATtTCtTGaTAGAATTCTTTgTCATGgTTtTnnGcaGTnGCTTC

>Bacillus_Fam_2_52_36 Nr. of seq. 36 Alignment length(with gaps) = 57
Alignment score = 0.678177
GCF_000169195.2_ASM16919v2_genomic.fna_1:114628-114888 Satlength=261 Nr
of Repeats=4 RepeatLength=52 seed=CTTAACGTAG Num.seqs=3
Similarity=0.760684
0   CTTAACGTAGACGGGCTCTAGGTGAAGGAAG-GACTTTGTTTTTN-CN---CGCTTC
Rev.of_GCF_000169195.2_ASM16919v2_genomic.fna_1:485819-486339
Satlength=521 Nr of Repeats=10 RepeatLength=52 seed=ACATAGAGCC
Num.seqs=10 Similarity=0.791475
23   CTTAACGTAGACGGGCTCTATGTAAAGGAAG-GTCTTCGTTTTTC-CG---CGCTTT
GCF_000169195.2_ASM16919v2_genomic.fna_1:1467180-1467492 Satlength=313 Nr
of Repeats=6 RepeatLength=52 seed=CTTAACGTAG Num.seqs=6
Similarity=0.707692 52
CTTAACGTAGACGGGCTCTATGTGAAGAAAG-GCCTTCGTTTTTC-CT---CGCTTC
Rev.of_GCF_000217835.1_ASM21783v1_genomic.fna_1:328870-329234
Satlength=365 Nr of Repeats=6 RepeatLength=52 seed=TTAAGGAAGC Num.seqs=5
Similarity=0.800000 57
CTTAACGTAGACGGGTTCTATGTGAAGAAAG-GCCGTCTTTTTTC-CG---CGCTTC
Rev.of_GCF_000169195.2_ASM16919v2_genomic.fna_1:2663853-2664582
Satlength=730 Nr of Repeats=14 RepeatLength=52 seed=GTCTACGTTA
Num.seqs=13 Similarity=0.703596
64   CTTAACGTAGACGCGCTCTATGTNAAGAAAG-GCCNTCGTTTTTC-CG---TGCTTC
Rev.of_GCF_000169195.2_ASM16919v2_genomic.fna_1:2467497-2467705
Satlength=209 Nr of Repeats=4 RepeatLength=52 seed=GTTAAGGAAG Num.seqs=4

Similarity=0.829060 58
CTTAACGTAGACGGGGTCTATGTTAAGGAAG-GCCTTCGTTTTTC-CA---TGCTTC
GCF_000217835.1_ASM21783v1_genomic.fna_1:1961170-1961482 Satlength=313 Nr of Repeats=6 RepeatLength=52 seed=CTCTATGTTA Num.seqs=6
Similarity=0.823932 67
CTTAACTTAGACGGGCTCTATGTTAAGAAAG-GCCTTCGTTTTTC-CA---TGCTTC
Rev.of_GCF_000169195.2_ASM16919v2_genomic.fna_1:261360-261828
Satlength=469 Nr of Repeats=9 RepeatLength=52 seed=CTTCACATAG Num.seqs=9
Similarity=0.817664 27
CTTAACGTAGACGNGCTCTATGTGAAGGAAG-GCCNTCATTTTTG-CN---CGCTTT
GCF_000169195.2_ASM16919v2_genomic.fna_1:2075586-2075846 Satlength=261 Nr of Repeats=5 RepeatLength=52 seed=GCTTCCTTAA Num.seqs=5
Similarity=0.841026 47
CTTAACGTAGACGGGCTCTATGTGAAGGAAG-GCCNTCATTTTTG-CA---CGCTTC
Rev.of_GCF_000169195.2_ASM16919v2_genomic.fna_1:1126423-1126942
Satlength=520 Nr of Repeats=10 RepeatLength=52 seed=CTACGTTAAG
Num.seqs=9 Similarity=0.768519
62 CTTAACGTAGCCGGGCTCTATGTGAAGGAAG-GCCGTCATTTTTN-CA---TGCTTT
GCF_000217835.1_ASM21783v1_genomic.fna_1:231429-232210 Satlength=782 Nr of Repeats=15 RepeatLength=52 seed=GTGAAGGAAG Num.seqs=14
Similarity=0.859115 73
CTTAACGTAGCCGGGCTCTATGTGAAGGAAG-GCCGTCAATTTTG-CA---AGCTTT
Rev.of_GCF_000169195.2_ASM16919v2_genomic.fna_1:1080639-1081263
Satlength=625 Nr of Repeats=12 RepeatLength=52 seed=CGTCTACGTT
Num.seqs=12 Similarity=0.785548
65 CTTAACGTAGACGGGCTCTATGTGAAGAAAG-GCCNTCNTTTTTT-CG---TGCTTT
GCF_000217835.1_ASM21783v1_genomic.fna_1:935120-935692 Satlength=573 Nr of Repeats=10 RepeatLength=52 seed=CTTAACGTAG Num.seqs=9
Similarity=0.777428
104 CTTAACGTAGACGNGCTCTATGTGAAGNAAG-GCCNTCATTTTTT-CG---TGCTTT
GCF_000217835.1_ASM21783v1_genomic.fna_1:261415-261675 Satlength=261 Nr of Repeats=5 RepeatLength=52 seed=CTTAACGTAG Num.seqs=5
Similarity=0.803774
104 CTTAACGTAGACGGGCTCTATGTGAAGAAAG-ACCNTCAATTTTT-CG---TGCTTC
Rev.of_GCF_000169195.2_ASM16919v2_genomic.fna_1:305183-306170
Satlength=988 Nr of Repeats=13 RepeatLength=52 seed=CCTTCCTTAA
Num.seqs=9 Similarity=0.810541
32 CTTAACATAGACGAGCTCTAAGTTAAGGAAG-GCCTTCATTTTTT-CG---TGCTTT
GCF_000169195.2_ASM16919v2_genomic.fna_1:1990467-1990883 Satlength=417 Nr of Repeats=8 RepeatLength=52 seed=GCTTTCTTAA Num.seqs=8
Similarity=0.800366 47
CTTAACATAGACGCGCTCTATGTGAAGGAAG-GCCGTCAATTTTC-CG---TGCTTT
GCF_000169195.2_ASM16919v2_genomic.fna_1:2001242-2001502 Satlength=261 Nr of Repeats=5 RepeatLength=52 seed=CTATGTGAAG Num.seqs=5
Similarity=0.742138 69
CTTAACGTAGNCGTGCNCTATGTGAAGAAAG-GCCTTCANTTTTC-CG---TGCTTC
GCF_000169195.2_ASM16919v2_genomic.fna_1:266768-267444 Satlength=677 Nr of Repeats=13 RepeatLength=52 seed=CTTCCTTAAC Num.seqs=13
Similarity=0.710351 48
CTTAACATAGACGCCCTCTACGTGAAGAAAG-ACCNTCNTTTTTC-CN---TGCTTC
GCF_000217835.1_ASM21783v1_genomic.fna_1:1959525-1959733 Satlength=209 Nr of Repeats=4 RepeatLength=52 seed=AAGAAAGACC Num.seqs=4
Similarity=0.794872 76
CTTAACGTAGACGGCCTCTACGTGAAGAAAG-ACCTTCGTTTTTC-CG---TGCTTC

GCF_000169195.2_ASM16919v2_genomic.fna_1:988693-989005 Satlength=313 Nr of Repeats=6 RepeatLength=52 seed=GCTTCCTTAA Num.seqs=6 Similarity=0.777778
99 CTTAACATAGACGCACTCTAGGTGAAGGAAG-GCCGTCTTTTTTC-CA---CGCTTC
GCF_000169195.2_ASM16919v2_genomic.fna_1:1031511-1032032 Satlength=522 Nr of Repeats=9 RepeatLength=52 seed=AAGACCTCCT Num.seqs=7 Similarity=0.848596 132
CTTAACNTAGACGGGCTCTATGTTAAGGAAG-ACCTCCTTTTTTC-CG---CGCTTC
Rev.of_GCF_000169195.2_ASM16919v2_genomic.fna_1:2791625-2792093 Satlength=469 Nr of Repeats=9 RepeatLength=52 seed=GTTAAGGAAG Num.seqs=9 Similarity=0.647498 162
CTTAACATAGACGCGCTCTATGTNAAGGAAG-ACNGACTTTTTTC-CN---CGCTTC
GCF_000169195.2_ASM16919v2_genomic.fna_1:1374642-1375162 Satlength=521 Nr of Repeats=10 RepeatLength=52 seed=GCTTCCTTAA Num.seqs=10 Similarity=0.751166 151
CTTAACGTAGACGCGCTCTATGTNAAGGAAG-ACCTTCGTTTTTG-CTT---GCTTC
Rev.of_GCF_000169195.2_ASM16919v2_genomic.fna_1:1690005-1690680 Satlength=676 Nr of Repeats=12 RepeatLength=52 seed=TTAAGGAAGC Num.seqs=10 Similarity=0.748987
162 CTTAACNTAGACGCNCTCTATGTGAAGGAAG-ACCTTCATTTTTC-CG---CGCTTC
Rev.of_GCF_000169195.2_ASM16919v2_genomic.fna_1:2018548-2019171 Satlength=624 Nr of Repeats=12 RepeatLength=52 seed=GTTAAGGAAG Num.seqs=11 Similarity=0.690572
163 CTTAACGTAGACGCGCTCTATGTGAAGGAAG-GCCTTCATTTTTC-CN---TGCTTC
Rev.of_GCF_000169195.2_ASM16919v2_genomic.fna_1:2631291-2631603 Satlength=313 Nr of Repeats=6 RepeatLength=52 seed=TCTACGTTAA Num.seqs=6 Similarity=0.791195 168
CTTAACGTAGACGCTCTCTATGTGAAGGAAGNGCCTTCGTTTTTC-CG---CGCTTC
GCF_000169195.2_ASM16919v2_genomic.fna_1:1043778-1044356 Satlength=579 Nr of Repeats=10 RepeatLength=52 seed=CCTTAACGTA Num.seqs=8 Similarity=0.815043 51
CTTAACGTAGACGCCCTCTAGGTTAAGGAAG-AGCCTCGTTTTTT-CA---CGCTTC
Rev.of_GCF_000169195.2_ASM16919v2_genomic.fna_1:2627959-2628323 Satlength=365 Nr of Repeats=7 RepeatLength=52 seed=TTAAGGAAGC Num.seqs=7 Similarity=0.775336 57
CTTAACGTAGACGCGCCCTATGTTAAGGAAC-ANCTTCTTTTTTT-CA---CGCTTC
GCF_000217835.1_ASM21783v1_genomic.fna_1:2106488-2106748 Satlength=261 Nr of Repeats=5 RepeatLength=52 seed=CGCTTCCTTA Num.seqs=5 Similarity=0.789937 98
CTTAACGTAGACGCGCCCTATGTTAAGGAAC-ATCTTCTTTTTTT-CA---CGCTTC
Rev.of_GCF_000169195.2_ASM16919v2_genomic.fna_1:867678-868249 Satlength=572 Nr of Repeats=11 RepeatLength=52 seed=GTTAAGAAAG Num.seqs=10 Similarity=0.750997
6 CTTAACGTAGGCGTGCTCTATGTAAAGGAAG-GNCGCCGAATTTT-CT---TGCTTT
GCF_000217835.1_ASM21783v1_genomic.fna_1:429182-429858 Satlength=677 Nr of Repeats=13 RepeatLength=52 seed=CTTTCTTAAC Num.seqs=9 Similarity=0.751747
48 CTTAACGTAGACGTGCTCTATGTAAAGGAAG-GACGCCGATTTTT-CT---NNCTTT
Rev.of_GCF_000169195.2_ASM16919v2_genomic.fna_1:1607690-1608264 Satlength=575 Nr of Repeats=9 RepeatLength=52 seed=TCTACGTTAA Num.seqs=6 Similarity=0.834188 11
CTTAACGTAGACGCGCTCTATGTTAAGAAAC-AAGCTC-ATTTTC-CT--TCGCTTC
Rev.of_GCF_000169195.2_ASM16919v2_genomic.fna_1:1815223-1815483 Satlength=261 Nr of Repeats=5 RepeatLength=52 seed=AAGGAAGCGA Num.seqs=5

Similarity=0.746154 55
CTTAACGTAGACGCCTTCTATGTTAAGATAG-ANCCGCAATTTTT-C---TCGCTTC
GCF_000169195.2_ASM16919v2_genomic.fna_1:3359605-3359865 Satlength=261 Nr of Repeats=4 RepeatLength=52 seed=TACGTTAAGA Num.seqs=3
Similarity=0.692308 18
CTTAACNTAGACGCACTCTACGTTAAGAAAC-A---TCGATTTTTNCN-CNCGCTTC
GCF_000169195.2_ASM16919v2_genomic.fna_1:1835633-1836413 Satlength=781 Nr of Repeats=15 RepeatLength=52 seed=CTTAACGTAG Num.seqs=15
Similarity=0.802198 0
CTTAACGTAGACANCCTCTATGTGAAGGAAC-AAGGCCAATTTTN-CA---TGCTTT
Rev.of_GCF_000217835.1_ASM21783v1_genomic.fna_1:2666582-2667102
Satlength=521 Nr of Repeats=10 RepeatLength=52 seed=TCTACGTTAA
Num.seqs=8 Similarity=0.784799
11 CTTAACGTAGACAGCCTCTATGTAAAGGAAC-AAGGCCATTTTTG-CA---TGCTTC

****** *** * *** ** *** * *** * ***
Consensus:

CTTAACgTAGACGngCTCTATGTgAAGgAAGgcCnTCntTTTTnCnnGCTTc

>Bacillus_Fam_3_50_31 Nr. of seq. 31 Alignment length(with gaps) = 64
Alignment score = 0.605623
BACILLUS_PSEUDALCALIPHILUS_genomic.fna_1:120942-121911 Satlength=970 Nr of Repeats=18 RepeatLength=51 seed=GAATCGCGGG Num.seqs=16
Similarity=0.881866 0
GAATCGCGGGGTTGCGAGT--CGA-A--AAA-GGAG--G--A--GAAACCCCGAGCAAAGGCG-
BACILLUS_PSEUDALCALIPHILUS_genomic.fna_2:41923-42331 Satlength=409 Nr of Repeats=8 RepeatLength=51 seed=CCCGAGCAAA Num.seqs=8 Similarity=0.830065
37 GAATCGCGGGGTTACGAGC--GGA-A--AAA-GGAG--G--A--GAAACCCCGAGCAAAAGCG-
BACILLUS_PSEUDALCALIPHILUS_genomic.fna_5:467020-467377 Satlength=358 Nr of Repeats=7 RepeatLength=51 seed=GGAATCGCGG Num.seqs=7 Similarity=0.855587
50 GAATCGCGGGGTTGCGAGC--GGA-A--AAA-GCTG--G--A--GAAAGCCCGAGCAAACGTG-
BACILLUS_PSEUDALCALIPHILUS_genomic.fna_10:87072-87479 Satlength=408 Nr of Repeats=8 RepeatLength=51 seed=GAATCGCGGG Num.seqs=7 Similarity=0.809524
51 GAATCGCGGGGTTGCGAGC--GGA-A--AAA-GCGG--G--A--GAAACCCCGAGTAAACGNG-
BACILLUS_PSEUDALCALIPHILUS_genomic.fna_10:200782-200986 Satlength=205 Nr of Repeats=4 RepeatLength=51 seed=CCCGAGCAAA Num.seqs=4
Similarity=0.869281 88
GAATCGCGGGGTTGCGAGC--TGG-A--AAA-GCGG--G--A--GTAACCCCGAGCAAACGCG-
BACILLUS_PSEUDALCALIPHILUS_genomic.fna_5:163285-163591 Satlength=307 Nr of Repeats=6 RepeatLength=51 seed=CCCGAGCAAA Num.seqs=6 Similarity=0.874510
37 GAATCTCGGGGTTGCGAGC--GAA-A--AAA-GTAG--G--A--GAAAGCCCGAGCAAAAGCG-
Rev.of_BACILLUS_PSEUDALCALIPHILUS_genomic.fna_6:546985-547648
Satlength=664 Nr of Repeats=13 RepeatLength=51 seed=TTTGCTCGGG
Num.seqs=13 Similarity=0.861440
47 GAATCGCGGGGTTGCGGGC--TGA-A--AAA-GTAG--G--A--GAAAGCCCGAGCAAAAGCG-
Rev.of_BACILLUS_PSEUDALCALIPHILUS_genomic.fna_6:198906-199161
Satlength=256 Nr of Repeats=5 RepeatLength=51 seed=CGGGCTTTCT Num.seqs=5
Similarity=0.761635 41
GAATCACGGGGTTGCGNGC--GGA-A--AAA-GNAG--G--A--GAAAGCCCGAGCAAAAGCC-
Rev.of_BACILLUS_PSEUDALCALIPHILUS_genomic.fna_6:236716-237058
Satlength=343 Nr of Repeats=7 RepeatLength=51 seed=CAACCCCGAG Num.seqs=6
Similarity=0.833333 65
GAATCTCGGGGTTGTGGGC--TGA-AN-AAA-GNAG--G--A--GAAAGCCCGAGCAAAAGCG-

Rev.of_BACILLUS_PSEUDALCALIPHILUS_genomic.fna_6:531203-531509
Satlength=307 Nr of Repeats=6 RepeatLength=51 seed=GCAACCCCGC Num.seqs=6
Similarity=0.824145 15
GAATCGCGGGGTTGCGATG--GGA-A-AAAA-AGAG--G--A--GAAAGCCCGAGCAAACGCG-
BACILLUS_PSEUDALCALIPHILUS_genomic.fna_1:440937-442057 Satlength=1121 Nr
of Repeats=22 RepeatLength=51 seed=AAGCCCGAGC Num.seqs=20
Similarity=0.839835 34
GAATCACGGGGTTGCGAGC--TGA-A--AAA-G-GA--G--A-AGAAAGCCCGAGCAAAAGCT-
BACILLUS_PSEUDALCALIPHILUS_genomic.fna_1:490720-491158 Satlength=439 Nr of
Repeats=8 RepeatLength=51 seed=GAAGAAAGCC Num.seqs=6 Similarity=0.879739
80 GAATCGCGGGGTTGCGAGC--TGA-A--AAA-A-CG--G--A-AGAAAGCCCGAGCAAAAGCT-
BACILLUS_PSEUDALCALIPHILUS_genomic.fna_5:415567-415873 Satlength=307 Nr of
Repeats=6 RepeatLength=51 seed=GAATCACGGG Num.seqs=6 Similarity=0.853595
51 GAATCACGGGGTTGCGGGC--TGA-A--AAA-G-CG--G--A-AGAAAGCCCGAGCAAACGCT-
BACILLUS_PSEUDALCALIPHILUS_genomic.fna_3:325147-325506 Satlength=360 Nr of
Repeats=7 RepeatLength=51 seed=AAAGCCCGAG Num.seqs=6 Similarity=0.835470
84 GAATCACGGGGTTACGAGC--AGA-A--AAA-G-AA--G--A-AGAAAGCCCGAGCAAACGCT-
BACILLUS_PSEUDALCALIPHILUS_genomic.fna_5:683344-683599 Satlength=256 Nr of
Repeats=5 RepeatLength=51 seed=AACGCCGAAT Num.seqs=5 Similarity=0.963399
45 GAATCGCGGGGTTGCGAGC--TGA-A--AAA-G-AG--G--T-AGAAAGCCCGAGCAAACGCC-
BACILLUS_PSEUDALCALIPHILUS_genomic.fna_1:519811-520121 Satlength=311 Nr of
Repeats=6 RepeatLength=51 seed=GAATCACGGG Num.seqs=4 Similarity=0.947712
50 GAATCACGGGGTTGCGAGC--TGA-A--AAAGG-AA--G--A-GGA-AGCCCGAGCAAACGCC-
BACILLUS_PSEUDALCALIPHILUS_genomic.fna_12:22376-22706 Satlength=331 Nr of
Repeats=7 RepeatLength=51 seed=GAGGAGAAAG Num.seqs=6 Similarity=0.855338
77 GAATCACGGGGTTACGAGC--TGA-A--AAAG-AG--G--A-GAA-AGCCCGAGCAAACGCG-
Rev.of_BACILLUS_PSEUDALCALIPHILUS_genomic.fna_6:68984-69392 Satlength=409
Nr of Repeats=8 RepeatLength=51 seed=AACCCCGAGA Num.seqs=8
Similarity=0.815018 64
GAATCTCGGGGTTGCGAGC--NGA-A--AAA-G-AGA-G--A-AGTAAGCCCGAGCAAAAGCC-
BACILLUS_PSEUDALCALIPHILUS_genomic.fna_3:181573-181930 Satlength=358 Nr of
Repeats=7 RepeatLength=51 seed=GAATCACGGG Num.seqs=7 Similarity=0.905384
50 GAATCACGGGGTTGCGAGC--AGA-A--AAA-G-CG-AA--A-GGA-AGCCCGAGCAAACGTG-
Rev.of_BACILLUS_PSEUDALCALIPHILUS_genomic.fna_6:167904-168436
Satlength=533 Nr of Repeats=9 RepeatLength=51 seed=TCTCTTTTTC Num.seqs=6
Similarity=0.823965 80
GAATCGCGGGGTTGCGAGC--AGA-A--AAA-G-AG-AG--A-GGA-AGCCCGAGCAAACGCG-
BACILLUS_PSEUDALCALIPHILUS_genomic.fna_1:180186-180593 Satlength=408 Nr of
Repeats=7 RepeatLength=51 seed=CGGAATCACG Num.seqs=6 Similarity=0.786325
100 GAATCACGGGGTTGCGNGCT-TGA-A--AAA-G-AG-AG--A-GGA-AGCCCGAGCAAAAGCG-
BACILLUS_PSEUDALCALIPHILUS_genomic.fna_5:932806-933113 Satlength=308 Nr of
Repeats=6 RepeatLength=51 seed=GAGCAAACGC Num.seqs=5 Similarity=0.734591
40 GAATCACGGGGTTGC-AGC--TGG-A--AAA-G-NN--G--AGAGAAAGCCCGAGCAAACGCT-
BACILLUS_PSEUDALCALIPHILUS_genomic.fna_2:160131-160593 Satlength=463 Nr of
Repeats=8 RepeatLength=51 seed=CGGGGTTGCG Num.seqs=6 Similarity=0.805983
5 -AATCACGGGGTTGCGAGC--GG--A--AAA-A-NG-AGT-A--GAAACCCCGAGCAAACGTCG
BACILLUS_PSEUDALCALIPHILUS_genomic.fna_5:550534-551044 Satlength=511 Nr of
Repeats=10 RepeatLength=51 seed=AATCTCGGGG Num.seqs=10
Similarity=0.822934 51
-AATCTCGGGGTTGCGGGC--TG--A--AAA-G-TG-AGG-A--GAAACCCCGAGCAAACGTCG
Rev.of_BACILLUS_PSEUDALCALIPHILUS_genomic.fna_2:186956-187518
Satlength=563 Nr of Repeats=11 RepeatLength=51 seed=AACCCCGAGA
Num.seqs=10 Similarity=0.780627
12 -AATCTCGGGGTTGCGAGC--GGA-A--AAA-G-GG-CG--A--GGAACCCCGAGCAAACGCCG

Rev.of_BACILLUS_PSEUDALCALIPHILUS_genomic.fna_2:188762-189169
Satlength=408 Nr of Repeats=8 RepeatLength=51 seed=AACCCCGAGA Num.seqs=7
Similarity=0.795788   12   -
AATCTCGGGGTTGCGAGC--GGA-A--AAA-G-NG-NG--A--GGAACCCCGTGCAAACGCNG
BACILLUS_PSEUDALCALIPHILUS_genomic.fna_5:312159-312465 Satlength=307 Nr of
Repeats=6 RepeatLength=51 seed=GAGAAAGCCC Num.seqs=6 Similarity=0.919826
29   -AATCTCGGGATTGCGGGC--GGA-A--AAA-G-CG-AG--A--GAAAGCCCGAGCAAACGGTG
BACILLUS_PSEUDALCALIPHILUS_genomic.fna_16:40561-41234 Satlength=674 Nr of
Repeats=12 RepeatLength=51 seed=GAGAAAGCCC Num.seqs=10
Similarity=0.811184   29
-AATCTCGGGGTTGCGAGC--GGA-A--AAA-G-AG-AG--A--GAAAGCCCGAGTAAACGNGG
BACILLUS_PSEUDALCALIPHILUS_genomic.fna_16:49541-49796 Satlength=256 Nr of
Repeats=5 RepeatLength=51 seed=CCCGAGCAAA Num.seqs=5 Similarity=0.789744
36   -AATCTCGGGGTTGCGAGC--GGA-A--AAA-G-CG-AG--A--GAAAGCCCGAGCAAACGCCG
BACILLUS_PSEUDALCALIPHILUS_genomic.fna_10:308612-309476 Satlength=865 Nr
of Repeats=17 RepeatLength=51 seed=CCCGAGCAAA Num.seqs=13
Similarity=0.762619   36
-AATCACGGGGTTGCGGGC---GAGA--AAA-G-AG--G-NA--GAAAGCCCGAGCAAAAGTCG
BACILLUS_PSEUDALCALIPHILUS_genomic.fna_2:175088-175495 Satlength=408 Nr of
Repeats=8 RepeatLength=51 seed=AAGCCCGAGC Num.seqs=7 Similarity=0.757113
34   GAATCGCGGGGTTACATGC-CGGA-A--AAT-G-AG-----A-AGAAAGCCCGAGCAAAAGCG-

**** **** **   * **   * **** * *** *
Consensus:

gAATCnCGGGGTTGCGaGCnGAAAAAGaGGAGAAAgCCCGAGCAAAcGcn

>Bacillus_Fam_4_52_27 Nr. of seq. 27 Alignment length(with gaps) = 53
Alignment score = 0.745847
GCF_002019645.1_ASM201964v1_genomic.fna_1:953299-954474 Satlength=1176 Nr
of Repeats=23 RepeatLength=51 seed=AAGGGAATCA Num.seqs=21
Similarity=0.949953   0
AAGGGAATCAGACA-GCGTCTTTCGTTCCCTTTCTTCGACTTTTGGCATCAA-
Rev.of_GCF_002019645.1_ASM201964v1_genomic.fna_1:542786-543150
Satlength=365 Nr of Repeats=7 RepeatLength=52 seed=AGGGAACGAA Num.seqs=7
Similarity=0.881563   30
AAAGGAATCTGACA-GCACCTTTCGTTCCCTTTCTTCGACTTTTAGCACCAGT
Rev.of_GCF_002019645.1_ASM201964v1_genomic.fna_1:991762-991970
Satlength=209 Nr of Repeats=4 RepeatLength=52 seed=AGGGAATCAA Num.seqs=4
Similarity=0.897436   30
AAAGGAATCAGACACAC-TCTTTGATTCCCTTTCTCAGGCTTTTGGCATCAGA
Rev.of_GCF_002019645.1_ASM201964v1_genomic.fna_1:2696032-2696338
Satlength=307 Nr of Repeats=6 RepeatLength=52 seed=CCAAGAAAGG Num.seqs=4
Similarity=0.944444   37
AAGGGAATCAGACA-ACGCCTTTGATTCCCTTTCTTGGGCTTTTGACAACAGT
Rev.of_GCF_002019645.1_ASM201964v1_genomic.fna_1:3491116-3491372
Satlength=257 Nr of Repeats=5 RepeatLength=52 seed=TGTCTGATTC Num.seqs=3
Similarity=0.752137   66
AAGGGAATCAGACA-ACCNCTTTGATTCCCTTTGTTGGGCTTTTGGCATCAGA
GCF_002019645.1_ASM201964v1_genomic.fna_1:2379685-2379997 Satlength=313
Nr of Repeats=6 RepeatLength=52 seed=TCAGACAACC Num.seqs=6
Similarity=0.938462   7
AAGGGAATCAGACA-ACCACTTCCGTTCCCTTTCTCGCGCTCTTGGCACCAGA

Rev.of_GCF_002019645.1_ASM201964v1_genomic.fna_1:341445-341964
Satlength=520 Nr of Repeats=10 RepeatLength=52 seed=TGTTTGATTC
Num.seqs=9 Similarity=0.982906
14 AAGGGAATCAAACA-ATGCCTTTCATTCCCTTTGTTGGGCTTTTGGCATGAGA
Rev.of_GCF_002019645.1_ASM201964v1_genomic.fna_1:4262761-4263071
Satlength=311 Nr of Repeats=6 RepeatLength=52 seed=AAAGGGAATG Num.seqs=5
Similarity=0.912821 32
AAGGGAATCAAACA-ATGCTTTTCATTCCCTTTGTTGGGCTTTTGGCATGAGA
Rev.of_GCF_002019645.1_ASM201964v1_genomic.fna_1:1336769-1336977
Satlength=209 Nr of Repeats=4 RepeatLength=52 seed=TGTTTGATTC Num.seqs=4
Similarity=0.957265 14
AAGGGAATCAAACA-GTGCCTTTCATTCCCTTTGTTGGGCTTTGGGCATGAGA
GCF_002019645.1_ASM201964v1_genomic.fna_1:1412262-1412728 Satlength=467
Nr of Repeats=9 RepeatLength=52 seed=TTTGGCATGA Num.seqs=7
Similarity=0.890110 40
AAGGGAATCAGACA-ACTCCTTTCATTCCCTTTGTTGGGCTTTTGGCATGAGA
Rev.of_GCF_002019645.1_ASM201964v1_genomic.fna_1:2792762-2793074
Satlength=313 Nr of Repeats=6 RepeatLength=52 seed=AAAGGGAATG Num.seqs=6
Similarity=0.897436 84
AAGGGAATCAAACA-ACTCTTTTCATTCCCTTTGTTGGGCTTTGGGCATGAGA
GCF_002019645.1_ASM201964v1_genomic.fna_1:4144195-4144455 Satlength=261
Nr of Repeats=5 RepeatLength=52 seed=TTCATTCCCT Num.seqs=5
Similarity=0.882051 124
AAGGGAATCAAACA-ACTCTTTTCATTCCCTTTGTTGGGCTTTGGGCATGAGA
GCF_002019645.1_ASM201964v1_genomic.fna_1:4292959-4293477 Satlength=519
Nr of Repeats=9 RepeatLength=52 seed=AAACAACTCT Num.seqs=7
Similarity=0.899878 113
AAGGGAATCAAACA-ACTCTTTTCATTCCCTTTGTTGGGCTTTTGGCATGAGA
GCF_002019645.1_ASM201964v1_genomic.fna_1:3630624-3630935 Satlength=312
Nr of Repeats=6 RepeatLength=52 seed=ATCAAACAAC Num.seqs=5
Similarity=0.948718 110
AAGGGAATCAAACA-ACTCTTTTCATTCCCTTTGTTGGGCTTATGGCATGAGA
GCF_002019645.1_ASM201964v1_genomic.fna_1:5084517-5084881 Satlength=365
Nr of Repeats=7 RepeatLength=52 seed=GGAATCAAAC Num.seqs=7
Similarity=0.941392 107
AAAGGAATCAAACA-ACTCTTTTCATTCCCTTTGTTGGGCTTTAGGCATGAGA
GCF_002019645.1_ASM201964v1_genomic.fna_1:3924084-3924292 Satlength=209
Nr of Repeats=4 RepeatLength=52 seed=TTGGCATGAG Num.seqs=4
Similarity=0.901709 41
AAGGGAATGAAACA-ACGCTTTTCATTCCCTTTGTTGAGCTTTTGGCATGAGA
Rev.of_GCF_002019645.1_ASM201964v1_genomic.fna_1:3983763-3984127
Satlength=365 Nr of Repeats=7 RepeatLength=52 seed=GGAATGAAAA Num.seqs=7
Similarity=0.899878 80
AAGGGAATGAAACA-ACGCTTTTCATTCCCTTTGTTGGGCTTTGGGCATGAGA
Rev.of_GCF_002019645.1_ASM201964v1_genomic.fna_1:1400861-1401069
Satlength=209 Nr of Repeats=4 RepeatLength=52 seed=CAAAGGGAAT Num.seqs=4
Similarity=0.872642 33
AAAGGAATGAAACA-ATGCTTTTCATTCCCTTTGGATAGCTTTTGGCATGAGA
Rev.of_GCF_002019645.1_ASM201964v1_genomic.fna_1:4004918-4005178
Satlength=261 Nr of Repeats=5 RepeatLength=52 seed=TTTCTCATGC Num.seqs=5
Similarity=0.892308 54
AAAGGAATGAAACA-ATGCTTTTCATTCCCTTTGGATAGCTTTTGGCATGAGA
GCF_002019645.1_ASM201964v1_genomic.fna_1:2268253-2268461 Satlength=209
Nr of Repeats=4 RepeatLength=52 seed=GCATGAGAAA Num.seqs=4

Similarity=0.841880 44
AAAGGAATGAAACA-ACGCTTTTCATTCCCTTTGGAGAGCTTTGGGCATGAGA
Rev.of_GCF_002019645.1_ASM201964v1_genomic.fna_1:2767735-2768306
Satlength=572 Nr of Repeats=11 RepeatLength=52 seed=CAAAGGGAAT
Num.seqs=10 Similarity=0.903424
33 AAGGGAATCAAACA-ACGCTTTTCATTCCCTTTGGATGGCTTTTGGCCTGAGA
GCF_002019645.1_ASM201964v1_genomic.fna_1:3095308-3095622 Satlength=315
Nr of Repeats=6 RepeatLength=52 seed=TTTCATTCCC Num.seqs=5
Similarity=0.893082 71
AAGGGAATCAAACA-ATGCTTTTCATTCCCTTTGGATGGCTTTTGGCATGAGA
GCF_002019645.1_ASM201964v1_genomic.fna_1:4272434-4272694 Satlength=261
Nr of Repeats=5 RepeatLength=52 seed=TTTCATTCCC Num.seqs=5
Similarity=0.897436 71
AAGGGAATCAAACA-ACGCTTTTCATTCCCTTTGTAGGGCTTTTGGCATGAGA
GCF_002019645.1_ASM201964v1_genomic.fna_1:908246-908556 Satlength=311 Nr
of Repeats=6 RepeatLength=52 seed=GAAAGGGAAT Num.seqs=4
Similarity=0.804527
50 AAGGGAATCAGACA-ACACCTTTCAATCCCTTTCTTGGACTTTTTGCGTGAGA
GCF_002019645.1_ASM201964v1_genomic.fna_1:1747684-1748098 Satlength=415
Nr of Repeats=8 RepeatLength=52 seed=GAGAAAGGGA Num.seqs=6
Similarity=0.873504 48
AAGGGAATCAAACA-GCGCCTTTCGTTCCCTTGCACCGGCTTTTGGCGAGAGA
GCF_002019645.1_ASM201964v1_genomic.fna_1:1602707-1604007 Satlength=1301
Nr of Repeats=25 RepeatLength=52 seed=TTTCGTTCCC Num.seqs=25
Similarity=0.865470 19
AAGGGAATCAGAGA-ACATCTTTCGTTCCCTTTCATGGGCTATTGGTGTGGGA
GCF_002019645.1_ASM201964v1_genomic.fna_1:4783299-4783558 Satlength=260
Nr of Repeats=5 RepeatLength=52 seed=CTTTCGTTCC Num.seqs=4
Similarity=0.833333 70
AAGGGAACCTGAGA-ACACCTTTCGTTCCCTTTCTCGAGCTTTTGGTGTAAGA

** **** * * ** ****** **
Consensus:

AAgGGAATCAaACAAcgCtTTTCaTTCCCTTTgttggGCTTTtGGCATgAGA

>Bacillus_Fam_5_52_25 Nr. of seq. 25 Alignment length(with gaps) = 56
Alignment score = 0.706369
BACILLUS_PSEUDALCALIPHILUS_genomic.fna_1:47214-47630 Satlength=417 Nr of
Repeats=8 RepeatLength=52 seed=GTTAATGTGA Num.seqs=8 Similarity=0.884615
0 GTTAATGTGAAGATAACGAGACCAAACCTTGGAGT---ATCTGCACAAAGAGAG-G
BACILLUS_PSEUDALCALIPHILUS_genomic.fna_5:348032-348606 Satlength=575 Nr of
Repeats=11 RepeatLength=52 seed=GGTTAATGTG Num.seqs=10
Similarity=0.814186 51
GTTAATGTGCAGATAAGGAGNCCAAACCATGGAGT---ATCTGCACAAAGAGGT-G
BACILLUS_PSEUDALCALIPHILUS_genomic.fna_1:223083-223654 Satlength=572 Nr of
Repeats=11 RepeatLength=52 seed=CTGCACAAAG Num.seqs=10
Similarity=0.874074 37
GTTAATGTGAAGATACAGAGTCCAAACCTTGGAGT---AACTGCACAAAGAGGC-G
BACILLUS_PSEUDALCALIPHILUS_genomic.fna_2:413864-414427 Satlength=564 Nr of
Repeats=11 RepeatLength=52 seed=GGTTAATGTG Num.seqs=10
Similarity=0.883476 51
GTTAATGTGAAGATACAGAGGCCAAACCTTGGAGT---AACTGCACAAAGAGAG-G

Rev.of_BACILLUS_PSEUDALCALIPHILUS_genomic.fna_9:0-207 Satlength=208 Nr of Repeats=4 RepeatLength=52 seed=CAGTATCTGC Num.seqs=4 Similarity=0.901709
70 GTTAATGTGCAGATACTGAGGCCAAACCTTGGAGT---AACTGCACAAAGAGGG-G
BACILLUS_PSEUDALCALIPHILUS_genomic.fna_10:340482-340690 Satlength=209 Nr of Repeats=4 RepeatLength=52 seed=CTGCACAAAG Num.seqs=4 Similarity=0.871795 37
GTTAATGTGCAGATACAGAGGCCAAACCTTGAAGT---ATCTGCACAAAGAGGC-G
BACILLUS_PSEUDALCALIPHILUS_genomic.fna_5:905229-906112 Satlength=884 Nr of Repeats=17 RepeatLength=52 seed=CGGAGTAACT Num.seqs=15 Similarity=0.876923 81
GTTAATGTGAAGATACGGAGGCCAAACTTCGGAGT---AACTGCACAAAGAGGN-G
BACILLUS_PSEUDALCALIPHILUS_genomic.fna_1:525442-526534 Satlength=1093 Nr of Repeats=21 RepeatLength=52 seed=GGTTAATGTG Num.seqs=21 Similarity=0.801385 51
GTTAATGTGAAGATACGGAAGCCAAACCTTGGAGT---ATCTGCACAAAGAAGG-G
BACILLUS_PSEUDALCALIPHILUS_genomic.fna_5:1292643-1292851 Satlength=209 Nr of Repeats=4 RepeatLength=52 seed=GTATCTGCAC Num.seqs=4 Similarity=0.876068 85
GTTAATGTGTAGATACTGAGGCCAAACCTTGGAGT---ATCTGCACAAAGAAGG-G
BACILLUS_PSEUDALCALIPHILUS_genomic.fna_5:791934-792403 Satlength=470 Nr of Repeats=9 RepeatLength=52 seed=TGTGAAGATA Num.seqs=8 Similarity=0.939560
57 GTTAATGTGAAGATACGGACGCCAAACCTTAGAGT---ATCTGCACAAAGAGCG-G
BACILLUS_PSEUDALCALIPHILUS_genomic.fna_2:112951-113306 Satlength=356 Nr of Repeats=7 RepeatLength=52 seed=TGTGCAGATA Num.seqs=6 Similarity=0.875052
5 GTTAATGTGCAGATACGGAGGCAAAACCACGGAGT---ATCTGCACAAAGAGAG-G
BACILLUS_PSEUDALCALIPHILUS_genomic.fna_5:269767-270183 Satlength=417 Nr of Repeats=8 RepeatLength=52 seed=AATGTGAAGA Num.seqs=8 Similarity=0.903846
55 GTTAATGTGAAGATACGGAGGCCAAACCTCGGAGT---ATCTGCACAAAGAGAG-G
BACILLUS_PSEUDALCALIPHILUS_genomic.fna_10:253447-254227 Satlength=781 Nr of Repeats=15 RepeatLength=52 seed=TGTGAAGATA Num.seqs=13 Similarity=0.965812 57
GTTAATGTGAAGATACGGAGGCAAAACCTCGGAGT---ATCTGAACAAAGAGAG-G
BACILLUS_PSEUDALCALIPHILUS_genomic.fna_3:374971-375234 Satlength=264 Nr of Repeats=5 RepeatLength=52 seed=AAGATACGGA Num.seqs=4 Similarity=0.893162
9 GTTAATGTAAAGATACGGAGGCAAAACCTTGGAGT---AACTGCACAAAGAGAG-G
BACILLUS_PSEUDALCALIPHILUS_genomic.fna_5:285662-285974 Satlength=313 Nr of Repeats=6 RepeatLength=52 seed=TGAAGATACT Num.seqs=6 Similarity=0.852991
7 GTTAATGTGAAGATACTAAGGCAAAACCATGGAGT---ATCTGCACAAAGAGCG-G
BACILLUS_PSEUDALCALIPHILUS_genomic.fna_1:210419-210887 Satlength=469 Nr of Repeats=8 RepeatLength=52 seed=GGAGTAACTG Num.seqs=7 Similarity=0.887668
30 GTTAATGTTCAGATAAGGAGGCCAAACCTTGGAGT---AACTGCACAAAAAGGC-G
BACILLUS_PSEUDALCALIPHILUS_genomic.fna_2:443820-444288 Satlength=469 Nr of Repeats=9 RepeatLength=52 seed=AGTATCTGCA Num.seqs=9 Similarity=0.915954
31 -TTAATGTGAAGATACTGAGGTCAAACGATGAAGT---ATCTGCACAAAGAGGGTG
BACILLUS_PSEUDALCALIPHILUS_genomic.fna_3:62621-62933 Satlength=313 Nr of Repeats=6 RepeatLength=52 seed=CAAACCAAGT Num.seqs=6 Similarity=0.960684
73 -TTAATGTGCAGATACGGAGGTCAAACCAAGTAGT---ATCTGCACAAAGAGGGTG
Rev.of_BACILLUS_PSEUDALCALIPHILUS_genomic.fna_6:515438-515906 Satlength=469 Nr of Repeats=9 RepeatLength=52 seed=ATCTGCACAT Num.seqs=9 Similarity=0.891738 65 -
TTAATGTGCAGATACGGAAGCTAAACCANGTAGT---AACTGCACAAAGAGGGGG
BACILLUS_PSEUDALCALIPHILUS_genomic.fna_1:201723-201931 Satlength=209 Nr of Repeats=4 RepeatLength=52 seed=GTTTATGTGA Num.seqs=4 Similarity=0.927350
0 GTTTATGTGAAGATGCTGGGTCCAAACCTTGGAGT---ATCTGCACAAAAAGAC-A

BACILLUS_PSEUDALCALIPHILUS_genomic.fna_5:501746-502107 Satlength=362 Nr of Repeats=7 RepeatLength=52 seed=AGTTTATGTG Num.seqs=6 Similarity=0.892308
51 GTTTATGTGAAGATACGGAGGCCAAACCTTGGAGT---ATCTGCACAAAAAGAC-A
BACILLUS_PSEUDALCALIPHILUS_genomic.fna_6:486162-486682 Satlength=521 Nr of Repeats=10 RepeatLength=52 seed=AGTATCTGCA Num.seqs=10 Similarity=0.835150 32
TTTTATGTGCAGATACTGATGNCAAACCTTGNAGT---ATCTGCACAAAGAGNC-G
BACILLUS_PSEUDALCALIPHILUS_genomic.fna_1:503624-503988 Satlength=365 Nr of Repeats=7 RepeatLength=52 seed=AATGTGAAGA Num.seqs=7 Similarity=0.714002
3 GTTAATGTGAAGATACGAGAG-CAAAAG--CGAGTGGTATCTGCACAAAAAGAG-A
BACILLUS_PSEUDALCALIPHILUS_genomic.fna_1:511772-512978 Satlength=1207 Nr of Repeats=13 RepeatLength=52 seed=GTTAATGTGA Num.seqs=12 Similarity=0.891997 52
GTTAATGTGAAGATAAGAAAGCCAAAAGGACGATT---ATCTGCACAAAGAGGC-A
BACILLUS_PSEUDALCALIPHILUS_genomic.fna_6:363633-363841 Satlength=209 Nr of Repeats=4 RepeatLength=52 seed=ATGTGAAGAT Num.seqs=4 Similarity=0.935897
56 GTTTATGTGAAGATACGGGAGCCGAGAGAGCGACT---ATCTTCACAAAGAGGC-G

** **** **** * * * * ** ***** *
Consensus:

GTTAATGTGaAGATACgGAgGCcAAACcttGgAGTAtCTGCACAAAGAGggG

>Bacillus_Fam_6_20_21 Nr. of seq. 21 Alignment length(with gaps) = 22 Alignment score = 0.672366
GCF_000153365.1_ASM15336v1_genomic.fna_13:128542-128642 Satlength=101 Nr of Repeats=5 RepeatLength=20 seed=TTGCTAATAA Num.seqs=5 Similarity=0.693651
0 TTGCTAATAAAC--NGAAAAGN
Rev.of_GCF_000153365.1_ASM15336v1_genomic.fna_16:86330-86410 Satlength=81 Nr of Repeats=4 RepeatLength=20 seed=TTTATTAGCA Num.seqs=4 Similarity=0.593915 11
TTGCTAATAAAA--TGAAAAAC
Rev.of_GCF_000153365.1_ASM15336v1_genomic.fna_18:319407-319507 Satlength=101 Nr of Repeats=4 RepeatLength=20 seed=TTATTAGCAA Num.seqs=3 Similarity=0.730159 30
TTGCTAATAAAA--TGAAAAAC
GCF_000473245.1_ASM47324v1_genomic.fna_1:632989-633089 Satlength=101 Nr of Repeats=4 RepeatLength=20 seed=TTGCTAATAA Num.seqs=3 Similarity=0.735450
20 TTGCTAATAAAA--TGAAAAAN
Rev.of_GCF_000473245.1_ASM47324v1_genomic.fna_1:3206997-3207097 Satlength=101 Nr of Repeats=5 RepeatLength=20 seed=TTTATTAGCA Num.seqs=5 Similarity=0.707937 11
TTGCTAATAAAC--TGAAAAAN
GCF_000153365.1_ASM15336v1_genomic.fna_16:171173-171253 Satlength=81 Nr of Repeats=4 RepeatLength=20 seed=AAAAGTTGCT Num.seqs=4 Similarity=0.731481
14 -TGCTAATAAAA-CAAAAAAGT
Rev.of_GCF_000473245.1_ASM47324v1_genomic.fna_1:3892181-3892261 Satlength=81 Nr of Repeats=4 RepeatLength=20 seed=TTTATTAGCA Num.seqs=4 Similarity=0.661376 30 -
TGCTAATAAAATCACAAAAGT

GCF_000153365.1_ASM15336v1_genomic.fna_17:76317-76417 Satlength=101 Nr of Repeats=5 RepeatLength=20 seed=AGTTGCTAAT Num.seqs=5 Similarity=0.646970
17 -TGCTAATAAAN-CGGAAAAGT
GCF_000473245.1_ASM47324v1_genomic.fna_1:662499-662693 Satlength=195 Nr of Repeats=5 RepeatLength=20 seed=TGCTAATAAA Num.seqs=4 Similarity=0.722222
20 -TGCTAATAAAA-CGGAAAAGT
Rev.of_GCF_000153365.1_ASM15336v1_genomic.fna_18:289784-289884 Satlength=101 Nr of Repeats=5 RepeatLength=20 seed=TTATTAGCAA Num.seqs=5 Similarity=0.740000 29 -TGCTAATAAAA-CNGAAAAGT
Rev.of_GCF_000473245.1_ASM47324v1_genomic.fna_1:4451397-4451497 Satlength=101 Nr of Repeats=5 RepeatLength=20 seed=TATTAGCAAC Num.seqs=5 Similarity=0.700000 28 -TGCTAATAAAN-NGGAAAAGT
Rev.of_GCF_000153365.1_ASM15336v1_genomic.fna_18:63090-63190 Satlength=101 Nr of Repeats=5 RepeatLength=20 seed=TTTATTAGCA Num.seqs=5 Similarity=0.666667 30 -TGCTAATAAAA-NGNAAAAGT
GCF_000473245.1_ASM47324v1_genomic.fna_1:889265-889365 Satlength=101 Nr of Repeats=5 RepeatLength=20 seed=AGTTGCTAAT Num.seqs=3 Similarity=0.688889
37 -TGCTAATAAAA-NGNAAAAGT
GCF_000153365.1_ASM15336v1_genomic.fna_18:304350-304945 Satlength=596 Nr of Repeats=11 RepeatLength=20 seed=AGTTGCTAAT Num.seqs=9 Similarity=0.716667 17 -TGCTAATATAA-CGNAAAAGT
GCF_000153365.1_ASM15336v1_genomic.fna_18:620027-620127 Satlength=101 Nr of Repeats=5 RepeatLength=20 seed=AGTTGCTAAT Num.seqs=5 Similarity=0.642857
17 -TGCTAATAAAN-TNGAAAAGT
GCF_000153365.1_ASM15336v1_genomic.fna_17:566797-566897 Satlength=101 Nr of Repeats=5 RepeatLength=20 seed=AGTTGCTAAT Num.seqs=5 Similarity=0.713333
17 -TGCTAATAAAN-NNNAAAAGT
Rev.of_GCF_000473245.1_ASM47324v1_genomic.fna_1:3960943-3961043 Satlength=101 Nr of Repeats=5 RepeatLength=20 seed=TATTAGCAAC Num.seqs=5 Similarity=0.713333 28 -TGCTAATAAAN-NNNAAAAGT
Rev.of_GCF_000473245.1_ASM47324v1_genomic.fna_1:2638864-2638944 Satlength=81 Nr of Repeats=4 RepeatLength=20 seed=TAGCAACTTT Num.seqs=4 Similarity=0.711640 25 -TGCTAGTAAAA-GTGAAAAGT
GCF_000153365.1_ASM15336v1_genomic.fna_17:471750-471850 Satlength=101 Nr of Repeats=5 RepeatLength=20 seed=AGTTGCTAAT Num.seqs=5 Similarity=0.733333
17 -TGCTAATATAT-GGGGAAAGT
Rev.of_GCF_000473245.1_ASM47324v1_genomic.fna_1:4055915-4056015 Satlength=101 Nr of Repeats=5 RepeatLength=20 seed=TATTAGCAAC Num.seqs=5 Similarity=0.733333 28 -TGCTAATATAT-GGGGAAAGT
Rev.of_GCF_000473245.1_ASM47324v1_genomic.fna_1:647550-648145 Satlength=596 Nr of Repeats=11 RepeatLength=20 seed=TATTAGCAAC

Num.seqs=9 Similarity=0.652357
28   -TGCTAATATAA-ANGAAAAGT

***** ** *   ***
Consensus:

TGCTAATAAAanngAAAAGt

>Bacillus_Fam_7_52_18 Nr. of seq. 18 Alignment length(with gaps) = 60
Alignment score = 0.620516
GCF_000708755.2_ASM70875v2_genomic.fna_1:271081-271444 Satlength=364 Nr of Repeats=7 RepeatLength=52 seed=AATCGTACCC Num.seqs=6 Similarity=0.800000
0   AATCGTACCCTATAAAACTGAGAAACGAATTTAT--AGGGTAAGCAAA--CG--CC-GG-
GCF_000709935.2_ASM70993v2_genomic.fna_1:283764-284023 Satlength=260 Nr of Repeats=5 RepeatLength=52 seed=AATCGTACCC Num.seqs=4 Similarity=0.760684
0   AATCGTACCCTATAAAACCGAAAAACAAATTTAT--AGGGTAAGCAAA--AG--CC-GG-
GCF_000708755.2_ASM70875v2_genomic.fna_1:448824-449084 Satlength=261 Nr of Repeats=5 RepeatLength=52 seed=CGTACCCTCG Num.seqs=5 Similarity=0.953846
2   -AACGTACCCTCGAAAACCGTAAAACG-ATTTGC-GAGGGTAAGCAAAC-AG---G-AGA
Rev.of_GCF_000708755.2_ASM70875v2_genomic.fna_2:874968-875332 Satlength=365 Nr of Repeats=7 RepeatLength=52 seed=TTGCTTACCC Num.seqs=7 Similarity=0.824199   44   -ATCGTACCCTCGCAAACCGAAAAACG-ATGTGG-GAGGGTAAGCAAG--GC--CG-CGA
Rev.of_GCF_000708755.2_ASM70875v2_genomic.fna_2:926049-926309 Satlength=261 Nr of Repeats=5 RepeatLength=52 seed=TTGCTTACCC Num.seqs=5 Similarity=0.825926   44   -ATCGTACCCTCGGAAACCGAGAAACG-ATTTGG-GAGGGTAAGCAAG--NG--CG-CGA
Rev.of_GCF_000709935.2_ASM70993v2_genomic.fna_4:181059-181318 Satlength=260 Nr of Repeats=5 RepeatLength=52 seed=TTGCTTACCC Num.seqs=4 Similarity=0.856394   44   -ATCGTACCCTCGGAAACCGAGAAACG-ATTTGG-GAGGGTAAGCAAG--CG--CG-CGA
GCF_000709935.2_ASM70993v2_genomic.fna_24:175334-175594 Satlength=261 Nr of Repeats=5 RepeatLength=52 seed=TACCCTCGGA Num.seqs=5 Similarity=0.877987
56   -ATCGTACCCTCGGAAACCGAGAAACG-ATCTGG-GAGGGTAAGCAAA--AG--CG-TGA
GCF_000709935.2_ASM70993v2_genomic.fna_13:142596-142908 Satlength=313 Nr of Repeats=6 RepeatLength=52 seed=GGAATCGTAC Num.seqs=6 Similarity=0.841026
49   -ATCGTACCCTGCCAAACCGAAAAACG-ATATGG-GAGGGTAAGCAAG--CG--CC-GGA
Rev.of_GCF_000708755.2_ASM70875v2_genomic.fna_2:1045503-1045815 Satlength=313 Nr of Repeats=6 RepeatLength=52 seed=TTGCTTACCC Num.seqs=6 Similarity=0.761934   44   -ATCGTACCCTCGTAAACTGTAAAACG-ATCTGG-GAGGGTAAGCAA---AG-NCA-TGA
Rev.of_GCF_000709935.2_ASM70993v2_genomic.fna_4:300377-300585 Satlength=209 Nr of Repeats=4 RepeatLength=52 seed=TTGCTTACCC Num.seqs=4 Similarity=0.755241   44   -ATCGTACCCTCGTAAACTGTAAAACG-ATCTGG-GAGGGTAAGCAA---AG-TCA-TGA
Rev.of_GCF_000708755.2_ASM70875v2_genomic.fna_2:1167641-1168164 Satlength=524 Nr of Repeats=8 RepeatLength=52 seed=TTTGCTTACC Num.seqs=5 Similarity=0.969231   45   -ATCGTACCCTCGGAAACTGAGAAACG-ATGTGG-GAGGGTAAGCAA---AG-TGG-TGA

Rev.of_GCF_000709935.2_ASM70993v2_genomic.fna_14:92603-92864
Satlength=262 Nr of Repeats=5 RepeatLength=52 seed=TTGCTTACCC Num.seqs=4
Similarity=0.709119 44
-ATCGTACCCTGGGAAACCGTGAAACG-ATTTGG-GAGGGTAAGCAA---AGTTCA--GA
GCF_000709935.2_ASM70993v2_genomic.fna_1:437645-437907 Satlength=263 Nr
of Repeats=5 RepeatLength=52 seed=GAATCGTACC Num.seqs=3
Similarity=0.716981
50 -ATCGTACCCTCGGAAACCGTAAAACG-NTATTG-GAGGGTAAGCAAA--NG--CC-NGA
Rev.of_GCF_000709935.2_ASM70993v2_genomic.fna_11:58473-58740
Satlength=268 Nr of Repeats=5 RepeatLength=53 seed=TGGGTAGGAT Num.seqs=3
Similarity=0.715151 62
-ATCCTACCCACCAAAACCGTAAAACG-ATATCG-GAGGGTAAGCAAA--AG--CC-TGA
GCF_000708755.2_ASM70875v2_genomic.fna_1:653531-653947 Satlength=417 Nr
of Repeats=8 RepeatLength=52 seed=GAGGGTAAGC Num.seqs=8
Similarity=0.938645
32 -ATCGTACCCTCTCAAACTGAAAATCG-ATATCG-GAGGGTAAGCAAA--TG---ATGGA
GCF_000709935.2_ASM70993v2_genomic.fna_1:670892-671152 Satlength=261 Nr
of Repeats=5 RepeatLength=52 seed=GGAGGGTAAG Num.seqs=5
Similarity=0.912821
83 -ATCGTACCCTCTCAAACTGAAAATCG-ATATCG-GAGGGTAAGCAAA--TG---ATTGA
Rev.of_GCF_000708755.2_ASM70875v2_genomic.fna_2:988076-988440
Satlength=365 Nr of Repeats=7 RepeatLength=52 seed=CTTTGCTTAC Num.seqs=7
Similarity=0.803534 45 -
ATCGTACCCTGCAAAACCGTAAATCG-ATNTGNG-AGGGTAAGCAAA-GTG---A-GGA
Rev.of_GCF_000709935.2_ASM70993v2_genomic.fna_4:243132-243340
Satlength=209 Nr of Repeats=4 RepeatLength=52 seed=AGGGTACGAT Num.seqs=4
Similarity=0.837607 61 -
ATCGTACCCTGCAAAACTGTAAATCG-ATCTGGG-AGGGTAAGCAAA-GTG---A-GGA

* * *****   **** * ** *   * *   ***********   *
Consensus:

ATCGTACCCTcgnAAACcGaaAAaCGATnTgggAGGGTAAGCAAanGcnnGA

>Bacillus_Fam_8_52_17 Nr. of seq. 17 Alignment length(with gaps) = 56
Alignment score = 0.680278
GCF_000751775.1_A1A_genomic.fna_10:40891-41880 Satlength=990 Nr of
Repeats=19 RepeatLength=52 seed=GACGACGAGA Num.seqs=18
Similarity=0.730730
25 AAAAGT-GAGTTTCCGGCC--AAAAAAN-GACGACGAGAACGGGTCTCGTCGCCAA
GCF_000751775.1_A1A_genomic.fna_20:26659-27491 Satlength=833 Nr of
Repeats=16 RepeatLength=52 seed=TCGTCGCCAA Num.seqs=16
Similarity=0.651343
41 AAAAGG-GGGTTTCCCGTG--CAAAAATG-ACGACGAGAAAGGGTCTCGTCGCCAA
GCF_000751775.1_A1A_genomic.fna_30:19681-20513 Satlength=833 Nr of
Repeats=16 RepeatLength=52 seed=GTCTCGTCGC Num.seqs=14
Similarity=0.737114
90 AAAANG-GAGTTTCCCGCC--CAAAAACG-ACGACGAGAAAGGGTCTCGTCGCCAA
Rev.of_GCF_000751775.1_A1A_genomic.fna_86:16-431 Satlength=416 Nr of
Repeats=8 RepeatLength=52 seed=TTTGGCGACG Num.seqs=7 Similarity=0.832723
52 AAAATG-GAGTTTCCCGCN--CAAAAGTG-ACGACGAGAAGNGGTCTCGTCGCCAA
Rev.of_GCF_000751775.1_A1A_genomic.fna_88:33038-34338 Satlength=1301 Nr
of Repeats=26 RepeatLength=52 seed=TTTTTGGCGA Num.seqs=24

Similarity=0.695501 54
AAAGTG-GAGTTTTCCGCC--CAAAAATG-ACGACGAGAANGAGTNTCGTCGCCAA
Rev.of_GCF_000751775.1_A1A_genomic.fna_85:17849-18108 Satlength=260 Nr of Repeats=5 RepeatLength=52 seed=TTTTGGCGAC Num.seqs=4 Similarity=0.727778
106 AAAGTG-GAGTTTCCCGNCT-CAAAAATG-ACGACGAGAAGTGGTCTCGTCGCCAA
GCF_000751775.1_A1A_genomic.fna_10:73198-73666 Satlength=469 Nr of Repeats=9 RepeatLength=52 seed=ACGACGAGAA Num.seqs=9 Similarity=0.774217
26 AAAATG-AGGTTTTNCGGC-NCAAAAC--GACGACGAGAAGTGGTCTCGTCGCCAA
Rev.of_GCF_000751775.1_A1A_genomic.fna_22:78517-79089 Satlength=573 Nr of Repeats=11 RepeatLength=52 seed=TTTTTTGGCG Num.seqs=11 Similarity=0.773223
56 AAAAGG-GAGTTTCTCGGC-TCAAAAT--GACGACGAGAANAGGTCTCGTCGCCAA
Rev.of_GCF_000751775.1_A1A_genomic.fna_35:49719-50032 Satlength=314 Nr of Repeats=5 RepeatLength=52 seed=CCATTTCTCG Num.seqs=3 Similarity=0.859539
92 AAAAAG-GAGTTTCTCGGC-TCAAAAT--GACGACGAGAAATGGCCTCGTCGCCAA
Rev.of_GCF_000751775.1_A1A_genomic.fna_101:14447-15227 Satlength=781 Nr of Repeats=15 RepeatLength=52 seed=TTTTTGGCGA Num.seqs=15 Similarity=0.743563 107
AAAANG-GAGTTTTTCGCC-TCAAAAT--GNCGACGAGAANGGGTCTCGTCGCCAA
Rev.of_GCF_000751775.1_A1A_genomic.fna_26:642-1369 Satlength=728 Nr of Repeats=14 RepeatLength=52 seed=TCTCGTCACC Num.seqs=13 Similarity=0.674451
87 AAAATN-GAGTTTCTCGCC-TCAAAAT--GGTGACGAGATCAGGNCTCGTCGCCAA
Rev.of_GCF_000751775.1_A1A_genomic.fna_65:14846-15626 Satlength=781 Nr of Repeats=15 RepeatLength=52 seed=TTCTCGTCAC Num.seqs=15 Similarity=0.727883
88 AAAAGA-GNGTTTTNCGGC-TCAAAAT--GGTGACGAGAANGGGTCTCGTCGTCAA
GCF_000751775.1_A1A_genomic.fna_77:13214-13941 Satlength=728 Nr of Repeats=13 RepeatLength=52 seed=AAAATGGTGA Num.seqs=12 Similarity=0.764371
124 AAAATG-GGGTTTTCCGGC-TCAAAAT--GGTGACGAGAATAGGTTTCGTCGTCAA
Rev.of_GCF_000751775.1_A1A_genomic.fna_83:74740-75571 Satlength=832 Nr of Repeats=16 RepeatLength=52 seed=CATTCTCGTC Num.seqs=15 Similarity=0.748847
142 AAAATG-GGGTTTTCCGGC-TCAAAAT--GGTGACGAGAATGGGTTTCGTCGCCAA
GCF_000751775.1_A1A_genomic.fna_65:50685-51777 Satlength=1093 Nr of Repeats=21 RepeatLength=52 seed=GTCGCCAAAA Num.seqs=21 Similarity=0.668548
96 AAAANG-GNGTTTTTCGAC-TCAAAAC--GGTGACGAGAATTGGTCTCGTCGCCAA
Rev.of_GCF_000751775.1_A1A_genomic.fna_47:1465-1879 Satlength=415 Nr of Repeats=8 RepeatLength=52 seed=TTTTGGCGAC Num.seqs=6 Similarity=0.677183
54 AAAAGC-AAGTTTTTCGGC-TTAAAAT--GGCGACGAGAACGGGGTTCGTCGCCAA
Rev.of_GCF_000751775.1_A1A_genomic.fna_27:14100-14724 Satlength=625 Nr of Repeats=12 RepeatLength=52 seed=TTTTGGCGAC Num.seqs=12 Similarity=0.702525
53 AATATGAGANTTTT-CGAC--CAAAAACG-GTGACGAGAACAGGTTTCGTCGCCAA

******* * ****** ***** *** * ****
Consensus:

gacGACGAGAAnnGGTcTCGTCGCCAAAAAAngGaGTTTtnCGnCtCAAAAn

>Bacillus_Fam_9_52_16 Nr. of seq. 16 Alignment length(with gaps) = 60 Alignment score = 0.601759

GCF_000292245.2_ASM29224v2_genomic.fna_7:40548-40756 Satlength=209 Nr of Repeats=4 RepeatLength=52 seed=CTTTGATTCC Num.seqs=4 Similarity=0.764403
0   ---CTTTGATTCCCTTT-ACGGCTCTTATTCAATCGCTTTTGAAATCATT--GAC-AGG-
Rev.of_GCF_000292245.2_ASM29224v2_genomic.fna_82:9184-9652 Satlength=469 Nr of Repeats=9 RepeatLength=52 seed=AATCAAAGGC Num.seqs=9 Similarity=0.915954   8
---CTTTGATTCCCTTT-CTGGCTCTGATTCGATGGCTTCTGGCTTCATT--GAC-AGC-
GCF_000292245.2_ASM29224v2_genomic.fna_91:13346-13554 Satlength=209 Nr of Repeats=4 RepeatLength=52 seed=TGATTCCCTT Num.seqs=4 Similarity=0.751048
3   ---CTTTGATTCCCTTT-CCGACTTTTATTCAATGGCTTTCGGACTCATT--GAC--GCT
Rev.of_GCF_000292245.2_ASM29224v2_genomic.fna_38:13764-14076 Satlength=313 Nr of Repeats=6 RepeatLength=52 seed=AAAGGGAATC Num.seqs=6 Similarity=0.713939   14
C---TTTGATTCCCTTT-NCGGCTTTGATTTGATGGCTTTTGGTTTCATTG-G-C-TGG-
Rev.of_GCF_000292245.2_ASM29224v2_genomic.fna_38:24849-25109 Satlength=261 Nr of Repeats=5 RepeatLength=52 seed=CAATGAATCC Num.seqs=5 Similarity=0.753086   47
CT-CTTTGATTCCCTTT-GAGGCTTTTATTTGATGGCTTTCGGATTCATT--G---GGG-
Rev.of_GCF_000292245.2_ASM29224v2_genomic.fna_38:34016-34224 Satlength=209 Nr of Repeats=4 RepeatLength=52 seed=CAAAGGGAAT Num.seqs=4 Similarity=0.901709   67
C--CTTTGATTCCCTTT-GCGGCTTTTATTTGCTGGCTTTTGGCTTCATT--G-C-GGG-
Rev.of_GCF_000292245.2_ASM29224v2_genomic.fna_38:38220-38584 Satlength=365 Nr of Repeats=8 RepeatLength=52 seed=GAATCAAAGG Num.seqs=6 Similarity=0.846541   113
C--CTTTGATTCCCTTT-GCGGCTTTTATTTGATGGCTTTCGGCTTCATT--G-C-TGG-
GCF_000292245.2_ASM29224v2_genomic.fna_41:5487-5747 Satlength=261 Nr of Repeats=5 RepeatLength=52 seed=CTTTGATTCC Num.seqs=5 Similarity=0.666667
104   C--CTTTGATTCCCTTT-CCGGCTTTTATTCGATGGCTTCGGGCTTCATT--G-C-GGG-
GCF_000292245.2_ASM29224v2_genomic.fna_84:3892-4206 Satlength=315 Nr of Repeats=6 RepeatLength=52 seed=TTTGATTCCC Num.seqs=4 Similarity=0.746399
53   C--CTTTGATTCCCTTT-CCGGCTTTGATTCAACGGCTTCCGGATTCATT--G-C-GGG-
GCF_000292245.2_ASM29224v2_genomic.fna_10:69101-69413 Satlength=313 Nr of Repeats=6 RepeatLength=52 seed=TATGATTCCC Num.seqs=6 Similarity=0.775354
1   C---TATGATTCCCTTT-GCAGCTCTGATTCATATGCTTTTGACATCATT-CG-AGCT--
GCF_000292245.2_ASM29224v2_genomic.fna_126:27-495 Satlength=469 Nr of Repeats=9 RepeatLength=52 seed=CTCTGATTCA Num.seqs=9 Similarity=0.782492
18   C-CCTATGATTCCCTTTGGCA-CTCTGATTCATATGCTTTTGGCATCATT--G-A-CT--
Rev.of_GCF_000292245.2_ASM29224v2_genomic.fna_31:24471-25040 Satlength=570 Nr of Repeats=11 RepeatLength=52 seed=AAGGGAATCA Num.seqs=9 Similarity=0.793447
13   C-CCTTTGATTCCCTTT-GCGGCTTTTATTCATATGCTTTAGGAATCATT--G-G-CG--
GCF_000292245.2_ASM29224v2_genomic.fna_77:841-1153 Satlength=313 Nr of Repeats=7 RepeatLength=52 seed=TGATTCCCTT Num.seqs=5 Similarity=0.841026
55   C-CCTATGATTCCCTTT-GCGGCTCTTATTCATATGCTTTTGGCATCATT--G-A-CG--
GCF_000292245.2_ASM29224v2_genomic.fna_92:15221-15533 Satlength=313 Nr of Repeats=6 RepeatLength=52 seed=CTCTATGATT Num.seqs=6 Similarity=0.863248
50   C-TCTATGATTCCCTTT-CTGGCTTTGATTCACCGGCTTTTGACATCATT--G-C-GG--
Rev.of_GCF_000292245.2_ASM29224v2_genomic.fna_171:47-724 Satlength=678 Nr of Repeats=13 RepeatLength=52 seed=AAAGGGAATC Num.seqs=12 Similarity=0.761673   66
C-TCTATGATTCCCTTT-CTGGCTTTGATTCACCGGCTTTTGACATCATT--G-C-GG--
Rev.of_GCF_000292245.2_ASM29224v2_genomic.fna_106:11691-12195 Satlength=505 Nr of Repeats=8 RepeatLength=52 seed=GAAAGGGAAT Num.seqs=7

Similarity=0.843965 67
C-TCTATGATTCCCTTT-CCGGCTTTAATTCACTGGCTTTTGACATCATT--G-C-NG--

* ***********   ** * ***   **** *   ***** *
Consensus:

CCTtTGATTCCCTTTncGGCTtTtATTcaatgGCTTTtGgcaTCATTGcnGn

>Bacillus_Fam_10_20_16 Nr. of seq. 16 Alignment length(with gaps) = 22
Alignment score = 0.662626
GCF_000934845.1_ASM93484v1_genomic.fna_170:34797-34878 Satlength=82 Nr of Repeats=4 RepeatLength=20 seed=TCGCCAATAA Num.seqs=3 Similarity=0.528889
0 TCGCCAATAAAA-NAAATT-T-
Rev.of_GCF_001315165.1_ASM131516v1_genomic.fna_41:14151-14232
Satlength=82 Nr of Repeats=4 RepeatLength=20 seed=TTTATTGGCG Num.seqs=3
Similarity=0.514493 11
TCGCCAATAAAA-TCATTT-T-
Rev.of_GCF_000813125.1_ASM81312v1_genomic.fna_75:42581-42683
Satlength=103 Nr of Repeats=5 RepeatLength=20 seed=TTATTGGCGA Num.seqs=3
Similarity=0.733333 10
TCGCCAATAAAATTATATT-T-
GCF_001315165.1_ASM131516v1_genomic.fna_6:81809-81911 Satlength=103 Nr of Repeats=5 RepeatLength=20 seed=CGCCAATAAA Num.seqs=3 Similarity=0.688889
21 TCGCCAATAAAATTAAATT-T-
Rev.of_GCF_000934845.1_ASM93484v1_genomic.fna_207:5245-5347 Satlength=103
Nr of Repeats=5 RepeatLength=20 seed=TTTATTGGCG Num.seqs=3
Similarity=0.688889 11
TCGCCAATAAAATTTTATT-T-
GCF_001315165.1_ASM131516v1_genomic.fna_3:16512-16593 Satlength=82 Nr of Repeats=4 RepeatLength=20 seed=TCGCCAATAA Num.seqs=3 Similarity=0.711111
20 TCGCCAATAAAATTTNATT-T-
Rev.of_GCF_000813125.1_ASM81312v1_genomic.fna_100:28725-28826
Satlength=102 Nr of Repeats=5 RepeatLength=20 seed=TTATTGGCGA Num.seqs=4
Similarity=0.788889 10
TCGCCAATAAAATTGGATT-T-
Rev.of_GCF_000934845.1_ASM93484v1_genomic.fna_140:1784-1886 Satlength=103
Nr of Repeats=5 RepeatLength=20 seed=TTATTGGCGA Num.seqs=3
Similarity=0.714286 10
TCGCCAATAAAAATAAATT-T-
Rev.of_GCF_000813125.1_ASM81312v1_genomic.fna_129:61647-61728
Satlength=82 Nr of Repeats=4 RepeatLength=20 seed=TTTATTGGCG Num.seqs=3
Similarity=0.711111 11
TCGCCAATAAAANNANATT-T-
Rev.of_GCF_000934845.1_ASM93484v1_genomic.fna_68:88019-88120
Satlength=102 Nr of Repeats=4 RepeatLength=20 seed=TATTGGCGAA Num.seqs=3
Similarity=0.714286 29
TCGCCAATANAATTTTNTT-T-
Rev.of_GCF_000813125.1_ASM81312v1_genomic.fna_100:19594-19675
Satlength=82 Nr of Repeats=4 RepeatLength=20 seed=TTTATTGGCG Num.seqs=3
Similarity=0.688889 10
-CGCCAATAAAATCATAATAT-
GCF_001315165.1_ASM131516v1_genomic.fna_22:40668-40770 Satlength=103 Nr of Repeats=5 RepeatLength=20 seed=CGCCAATAAA Num.seqs=3

Similarity=0.711111
20   -CGCCAATAAAAANATATTAT-
GCF_001315165.1_ASM131516v1_genomic.fna_3:41031-41132 Satlength=102 Nr of Repeats=5 RepeatLength=20 seed=CGCCAATAAA Num.seqs=4 Similarity=0.722222
20   -CGCCAATAAAATGAAATTAT-
Rev.of_GCF_001315165.1_ASM131516v1_genomic.fna_16:12683-12764 Satlength=82 Nr of Repeats=4 RepeatLength=20 seed=TTTATTGGCG Num.seqs=3 Similarity=0.656566   30
-CGCCAATAAANTNAAANTAT-
Rev.of_GCF_000813125.1_ASM81312v1_genomic.fna_98:38828-38909 Satlength=82 Nr of Repeats=4 RepeatLength=20 seed=TTTATTGGCG Num.seqs=3 Similarity=0.777778   11
TCGCCAATAAAAATCGA-T-TA
GCF_000813125.1_ASM81312v1_genomic.fna_51:14452-14553 Satlength=102 Nr of Repeats=5 RepeatLength=20 seed=TCGCCAATAA Num.seqs=4 Similarity=0.487778
21   TCGCCAATAAAAATCGATT-TN

******** *   * *
Consensus:

tCGCCAATAAAAttanATTT

>Bacillus_Fam_11_51_15 Nr. of seq. 15 Alignment length(with gaps) = 56 Alignment score = 0.625454
GCF_000292245.2_ASM29224v2_genomic.fna_2:54137-54392 Satlength=256 Nr of Repeats=5 RepeatLength=51 seed=TTTTCATTAC Num.seqs=5 Similarity=0.691667
0   TTTTCATTACCTATT-ANC--ATTTATTCGCACCCTAAT-TCCTCACACCNCGNN-
GCF_000292245.2_ASM29224v2_genomic.fna_8:10287-10542 Satlength=256 Nr of Repeats=5 RepeatLength=51 seed=TTTTCATTAC Num.seqs=5 Similarity=0.717284
0   TTTTCATTACCTATT-AAC--NGTGATTCGCACCGTAAT-CNCTCACAGCACGGG-
GCF_000292245.2_ASM29224v2_genomic.fna_2:95942-96554 Satlength=613 Nr of Repeats=12 RepeatLength=51 seed=TTTCATTACC Num.seqs=12 Similarity=0.821747
1   TTTTCATTACCTATT-ACC--GTTTATTCGCACCGTAAT-TCCGA-CAACNCGGCT
GCF_000292245.2_ASM29224v2_genomic.fna_84:12497-12803 Satlength=307 Nr of Repeats=6 RepeatLength=51 seed=CTTTTTCATT Num.seqs=6 Similarity=0.706080
49   TTTTCATTACCTATT-ACC--AATTTTTCGCACCCTAAT-CCGGC-CAACTCNGCT
GCF_000292245.2_ASM29224v2_genomic.fna_105:6378-7343 Satlength=966 Nr of Repeats=18 RepeatLength=51 seed=TTTCATTACC Num.seqs=14 Similarity=0.681019
1   TTTTCATTACCTATTCANC---ATTATTCGCACCTTAATCTCCG--CAACTCGGCT
GCF_000292245.2_ASM29224v2_genomic.fna_3:26666-27023 Satlength=358 Nr of Repeats=7 RepeatLength=51 seed=TCATTACCTA Num.seqs=7 Similarity=0.625037
3   TTTTCATTACCTATT--CC-NGTTTTTTCGCACCCAAATC-CGNCACAGCTCGGC-
GCF_000292245.2_ASM29224v2_genomic.fna_133:14-269 Satlength=256 Nr of Repeats=5 RepeatLength=51 seed=TTTTCATTAC Num.seqs=5 Similarity=0.639766
51   TTTTCATTACCTATT--CC-TGTTTATTCGCACCCTAATCTCTT-ACAGCACGGC-
GCF_000292245.2_ASM29224v2_genomic.fna_43:36326-36680 Satlength=355 Nr of Repeats=7 RepeatLength=51 seed=TTTTCATTAC Num.seqs=6 Similarity=0.790598
51   TTTTCATTACCTATT--CC-TGNTTATTCGCACCCTAATNTCTTCACAGCACGGC-
Rev.of_GCF_000292245.2_ASM29224v2_genomic.fna_59:24274-24682 Satlength=409 Nr of Repeats=8 RepeatLength=51 seed=GGTTCGAAAA Num.seqs=8 Similarity=0.909430   31
TTTTCATTACCTATT--CA-AGTTTTTTCGAACCCTAAT-TCTTAACACCTCGGC-

GCF_000292245.2_ASM29224v2_genomic.fna_5:34399-34863 Satlength=465 Nr of Repeats=9 RepeatLength=51 seed=TTCGCACCCT Num.seqs=7 Similarity=0.833639
23 TTTTCATTACTTATC--CC-GCTTTATTCGCACCCTAAT-TTGACTCAGCTCGGC-
GCF_000292245.2_ASM29224v2_genomic.fna_20:40449-40806 Satlength=358 Nr of Repeats=7 RepeatLength=51 seed=ATTCGCACCC Num.seqs=7 Similarity=0.788260
73 TTTTCATTACCTATC--CC-GCTCAATTCGCACCCTAAT-TTGACTCAGCACCGC-
Rev.of_GCF_000292245.2_ASM29224v2_genomic.fna_26:8-518 Satlength=511 Nr of Repeats=10 RepeatLength=51 seed=GTAATGAAAA Num.seqs=10 Similarity=0.770940 61
TTTTCATTACTTATT--CC-AGTTTATTCGCACCCTAAT-TTGGCACTGCTCGGC-
Rev.of_GCF_000292245.2_ASM29224v2_genomic.fna_139:4619-4975 Satlength=357 Nr of Repeats=7 RepeatLength=51 seed=GTAATGAAAA Num.seqs=6 Similarity=0.788889 61
TTTTCATTACTTATC--CC-AGTTTATTCGCACTCTAAT-TTGGCACTGCACAGC-
GCF_000292245.2_ASM29224v2_genomic.fna_42:17059-17365 Satlength=307 Nr of Repeats=6 RepeatLength=51 seed=TTTTTCGCAC Num.seqs=6 Similarity=0.814675
70 TTTTCATTACTTATT--CCAGCTTTT-TCGCACCGTAAT-CTGACTCAGCACGGT-
GCF_000292245.2_ASM29224v2_genomic.fna_8:67209-67464 Satlength=256 Nr of Repeats=5 RepeatLength=51 seed=TTTTCATTAC Num.seqs=5 Similarity=0.824837
51 TTTTCATTACCAATT--CC-CTTTTATTCGCACCNTAAT-TTCTCGCAGCACCGC-

```
**********  **              *** **   ***        *  * *
```

Consensus:

TTTTCATTACcTATtcCnntTTaTTCGCACCcTAATtcnncaCAgCnCgGc

>Bacillus_Fam_12_52_15 Nr. of seq. 15 Alignment length(with gaps) = 54 Alignment score = 0.703939
GCF_000751775.1_A1A_genomic.fna_10:11861-12903 Satlength=1043 Nr of Repeats=20 RepeatLength=52 seed=TTTTGACGAC Num.seqs=18 Similarity=0.678447
0 TTTTGACGACGAAA-CGCATTCTCGTCACCATTTTGAGGTGAAAAACGC-TCAA
GCF_000751775.1_A1A_genomic.fna_21:16757-17329 Satlength=573 Nr of Repeats=11 RepeatLength=52 seed=TCTCGTCACC Num.seqs=11 Similarity=0.696398
19 TTTTGACGACGAGA-TCTAATCTCGTCACCATTTTGAGGTGGGAAATGC-TCAA
GCF_000751775.1_A1A_genomic.fna_10:2556-2815 Satlength=260 Nr of Repeats=5 RepeatLength=52 seed=TCTCGTCACC Num.seqs=4 Similarity=0.633230
20 TTTTGACGACGAAATCCTATTCTCGTCACCAATTTGAGGCGAGAAACAC-CCAA
GCF_000751775.1_A1A_genomic.fna_35:77020-77852 Satlength=833 Nr of Repeats=16 RepeatLength=52 seed=TTTTGGCGAC Num.seqs=14 Similarity=0.698921
0 TTTTGGCGACGAGC-CCCGTTCTCGTCGTCATTTTGAGGTGAGAAATGC-TCAA
Rev.of_GCF_000751775.1_A1A_genomic.fna_42:1584-2449 Satlength=866 Nr of Repeats=17 RepeatLength=52 seed=GTGACGAGAA Num.seqs=13 Similarity=0.799803
28 TTTTGGCGACGAGA-CCCGTTCTCGTCACCATTTTGAGGTGAAAAATNC-TCGT
Rev.of_GCF_000751775.1_A1A_genomic.fna_42:28877-29449 Satlength=573 Nr of Repeats=10 RepeatLength=52 seed=AAAATGGTGA Num.seqs=9 Similarity=0.760631
34 TTTTGGCGACGAGA-CCCNTTCTCGTCACCATTTTGAGGCGNGAAATGC-TCTT
GCF_000751775.1_A1A_genomic.fna_70:5639-6159 Satlength=521 Nr of Repeats=9 RepeatLength=52 seed=CTCGTCACCA Num.seqs=8 Similarity=0.803792
72 TTTTGGCGACGAGA-CCCTTTCTCGTCACCATTTTGGGGCGAAAATTGC-TCGA

Rev.of_GCF_000751775.1_A1A_genomic.fna_102:57207-57779 Satlength=573 Nr of Repeats=9 RepeatLength=52 seed=GGTGACGAGA Num.seqs=7 Similarity=0.688230
29 TTTTGGCGACGAGA-CCCATTCTCGTCACCGTTTTGAAGTGAAAAATGC-NCNA
Rev.of_GCF_000751775.1_A1A_genomic.fna_81:37277-38265 Satlength=989 Nr of Repeats=17 RepeatLength=52 seed=GGTGACGAGA Num.seqs=15 Similarity=0.724298
29 TTTTGGCGACGAGC-CTCAATCTCGTCACCATTTTGAAGTGAAAAANGC-TCTN
Rev.of_GCF_000751775.1_A1A_genomic.fna_87:17389-18013 Satlength=625 Nr of Repeats=12 RepeatLength=52 seed=TTTTCACTTC Num.seqs=12 Similarity=0.770716
44 TTTTGGCGACGAGA-CTCGATCTCGTCACCATTTTGAAGTGAAAACCGC-TCAA
GCF_000751775.1_A1A_genomic.fna_19:0-467 Satlength=468 Nr of Repeats=9 RepeatLength=52 seed=TTTTTGGCGA Num.seqs=9 Similarity=0.756344
51 TTTTGGCGACGAGC-CTCGTTTTCGTCACCATTTTTGGGTGAGAAACGCTTG-T
Rev.of_GCF_000751775.1_A1A_genomic.fna_101:46-670 Satlength=625 Nr of Repeats=11 RepeatLength=52 seed=GGTGACGAGA Num.seqs=10 Similarity=0.652271
81 TTTTGGCGACGAGN-GCTGTTCTCGTCACCATTTTTAGTTGAGAAACGC-TCAA
GCF_000751775.1_A1A_genomic.fna_12:28203-29296 Satlength=1094 Nr of Repeats=20 RepeatLength=52 seed=TTTTGACGAC Num.seqs=18 Similarity=0.652961
105 TTTTGACGACGAGA-CCCGTTCTCGTCACCGTTTTTGGTTGAGAAANGCNTCAA
Rev.of_GCF_000751775.1_A1A_genomic.fna_53:18-223 Satlength=206 Nr of Repeats=4 RepeatLength=52 seed=TCGTCAAAAA Num.seqs=3 Similarity=0.811966
9 TTTTGACGACGAGA-CTGAATCTCGTCACCATTTTTCGTTGAGGAATGC-ACAT
Rev.of_GCF_000751775.1_A1A_genomic.fna_72:3229-3749 Satlength=521 Nr of Repeats=9 RepeatLength=52 seed=GACGAGATTC Num.seqs=8 Similarity=0.817766
26 TTTTGACGACGAGC-CTGAATCTCGTCACCATTTTNAGGTGAAAAATGC-ACTT

***** ****** * ***** * *** * * *
Consensus:

TTTTGgCGACGAGaCccntTCTCGTCACCATTTTgaggtGAgAAAtGCtCaa

>Bacillus_Fam_13_21_15 Nr. of seq. 15 Alignment length(with gaps) = 24 Alignment score = 0.642196
GCF_900111815.1_PRJEB17078_genomic.fna_1:83842-84237 Satlength=396 Nr of Repeats=7 RepeatLength=21 seed=TTACCGTAGA Num.seqs=6 Similarity=0.679798
0 TTACCGTAGATA-AAAGGCNG--T
GCF_900111815.1_PRJEB17078_genomic.fna_9:3907-4741 Satlength=835 Nr of Repeats=11 RepeatLength=21 seed=TTACCGTAGA Num.seqs=9 Similarity=0.742504
0 TTACCGTAGATA-AATGGTGG--T
Rev.of_GCF_900111815.1_PRJEB17078_genomic.fna_1:368287-368371 Satlength=85 Nr of Repeats=4 RepeatLength=21 seed=TATCTACGGT Num.seqs=4 Similarity=0.642677 12
TTACCGTAGATA-AATNGACG--T
GCF_900111815.1_PRJEB17078_genomic.fna_1:2689763-2690346 Satlength=584 Nr of Repeats=5 RepeatLength=21 seed=TTTACCGTAG Num.seqs=3 Similarity=0.873016 20
TTACCGTAGATA-AATTGCCG--T
Rev.of_GCF_900111815.1_PRJEB17078_genomic.fna_1:2705984-2706732 Satlength=749 Nr of Repeats=12 RepeatLength=21 seed=TATCTACGGT

Num.seqs=10 Similarity=0.714654
33   TTACCGTAGATA-AATTGCCG--T
GCF_900111815.1_PRJEB17078_genomic.fna_1:541624-541708 Satlength=85 Nr of Repeats=4 RepeatLength=21 seed=TTACCGTAGA Num.seqs=4 Similarity=0.642361
21   TTACCGTAGATA-AATTGCAG--N
GCF_900111815.1_PRJEB17078_genomic.fna_1:397204-397592 Satlength=389 Nr of Repeats=7 RepeatLength=21 seed=TTACCGTAGA Num.seqs=6 Similarity=0.716162
0   TTACCGTAGATA-AANTGNCGA-T
Rev.of_GCF_900111815.1_PRJEB17078_genomic.fna_1:2954420-2954813 Satlength=394 Nr of Repeats=7 RepeatLength=21 seed=TATCTACGGT Num.seqs=6 Similarity=0.708081   11   -TACCGTAGATA-AATGGAGN-AT
Rev.of_GCF_900111815.1_PRJEB17078_genomic.fna_1:136654-137062 Satlength=409 Nr of Repeats=8 RepeatLength=21 seed=TATCAACGGT Num.seqs=7 Similarity=0.645503   12   TTACCGTTGATA-AATCGCNG--T
Rev.of_GCF_900111815.1_PRJEB17078_genomic.fna_1:2902508-2902991 Satlength=484 Nr of Repeats=7 RepeatLength=21 seed=TATCAACGGT Num.seqs=6 Similarity=0.631313   12   TTACCGTTGATA-AATGGCNA--T
Rev.of_GCF_900111815.1_PRJEB17078_genomic.fna_1:2906204-2907642 Satlength=1439 Nr of Repeats=21 RepeatLength=21 seed=TATCTACGGT Num.seqs=13 Similarity=0.791616
12   TTACCGTAGATA-AATCGCNA--T
GCF_900111815.1_PRJEB17078_genomic.fna_1:2936585-2937431 Satlength=847 Nr of Repeats=11 RepeatLength=21 seed=TTACCGTTGA Num.seqs=8 Similarity=0.767587   21   TTACCGTAGATA-AATCGTGA--T
GCF_900111815.1_PRJEB17078_genomic.fna_1:80313-80667 Satlength=355 Nr of Repeats=5 RepeatLength=21 seed=TTACCGTTGA Num.seqs=4 Similarity=0.664251
22   TTACCGTTGATAAAATGGCTG--A
GCF_900111815.1_PRJEB17078_genomic.fna_1:2407942-2408247 Satlength=306 Nr of Repeats=4 RepeatLength=21 seed=TTACCGTTGA Num.seqs=3 Similarity=0.666667   21   TTACCGTTGATA-AATGNNCA--N
GCF_900111815.1_PRJEB17078_genomic.fna_11:38238-39212 Satlength=975 Nr of Repeats=13 RepeatLength=21 seed=TTACCGTTGA Num.seqs=10 Similarity=0.709428
21   TTACCGTTGATA-AATGNNGA--N

****** **** **
Consensus:

TTACCGTaGATAAATnGcngt

>Bacillus_Fam_14_21_15 Nr. of seq. 15 Alignment length(with gaps) = 23 Alignment score = 0.651484
GCF_001889165.1_ASM188916v1_genomic.fna_1:21005-21172 Satlength=168 Nr of Repeats=8 RepeatLength=21 seed=AATCGCGGAT Num.seqs=7 Similarity=0.782828
0   AATCGCGGAT-A-GAGCCCGGAA
GCF_001889165.1_ASM188916v1_genomic.fna_1:25511-25700 Satlength=190 Nr of Repeats=9 RepeatLength=21 seed=CGGATAGAGC Num.seqs=9 Similarity=0.873016
5   AATCGCGGAT-A-GAGCACTGAA

Rev.of_GCF_001889165.1_ASM188916v1_genomic.fna_1:3155990-3156777 Satlength=788 Nr of Repeats=15 RepeatLength=21 seed=TCCGCGATTT Num.seqs=10 Similarity=0.732323
9   AATCGCGGAT-A-GAGCGCCGAA
GCF_001889165.1_ASM188916v1_genomic.fna_1:497918-498023 Satlength=106 Nr of Repeats=5 RepeatLength=21 seed=AATCGCGGAT Num.seqs=5 Similarity=0.875758
0   AATCGCGGAT-A-GAGGGCTGAA
Rev.of_GCF_001889165.1_ASM188916v1_genomic.fna_1:2579124-2579418 Satlength=295 Nr of Repeats=14 RepeatLength=21 seed=TATCCGCGAT Num.seqs=14 Similarity=0.806035
11   AATCGCGGAT-A-GAGCGCTCAG
Rev.of_GCF_001889165.1_ASM188916v1_genomic.fna_1:3277057-3277141 Satlength=85 Nr of Repeats=4 RepeatLength=21 seed=TATCCGCGAT Num.seqs=4 Similarity=0.666667   11
AATCGCGGATAA-GAGCGGTGAT
Rev.of_GCF_001889165.1_ASM188916v1_genomic.fna_1:790108-790234 Satlength=127 Nr of Repeats=6 RepeatLength=21 seed=TATCCGCGAT Num.seqs=6 Similarity=0.817989   11
AATCGCGGAT-A-CAGCACCTAT
Rev.of_GCF_001889165.1_ASM188916v1_genomic.fna_1:2862732-2862858 Satlength=127 Nr of Repeats=6 RepeatLength=21 seed=CTATCCGCGA Num.seqs=6 Similarity=0.713636   11
AATCGCGGAT-AGCAGCATGTAT
GCF_001889165.1_ASM188916v1_genomic.fna_1:29979-30084 Satlength=106 Nr of Repeats=5 RepeatLength=21 seed=AATCGCGGAT Num.seqs=5 Similarity=0.701515
0   AATCGCGGAT-A-GACGNACCAG
GCF_001889165.1_ASM188916v1_genomic.fna_1:757325-757823 Satlength=499 Nr of Repeats=14 RepeatLength=21 seed=AATCGCGGAT Num.seqs=13 Similarity=0.655147   0
AATCGCGGAT-A-GAAGCCCCAA
GCF_001889165.1_ASM188916v1_genomic.fna_1:3362340-3362445 Satlength=106 Nr of Repeats=5 RepeatLength=21 seed=ATCGCGGATA Num.seqs=5 Similarity=0.847619   1
AATCGCGGAT-A-GAACCCCCAT
GCF_001889165.1_ASM188916v1_genomic.fna_1:4295617-4296096 Satlength=480 Nr of Repeats=10 RepeatLength=21 seed=AATCGCGGAT Num.seqs=8 Similarity=0.577877   0
AATCGCGGAT-A-GAGGNCCNAT
GCF_001889165.1_ASM188916v1_genomic.fna_1:802803-802887 Satlength=85 Nr of Repeats=4 RepeatLength=21 seed=AATCGCGGAT Num.seqs=4 Similarity=0.767196
0   AATCGCGGAT-A-GAGCATCCAA
GCF_001889165.1_ASM188916v1_genomic.fna_1:3702467-3702782 Satlength=316 Nr of Repeats=15 RepeatLength=21 seed=ATCGCGGATA Num.seqs=15 Similarity=0.860922   1
AATCGCGGAT-A-GAGCACCCAC
GCF_001889165.1_ASM188916v1_genomic.fna_1:2986923-2987175 Satlength=253 Nr of Repeats=12 RepeatLength=21 seed=ATCGCGGATA Num.seqs=12 Similarity=0.785474   1
AATCGCGGAT-A-GAGCNCCCTT

********** * *
Consensus:

AATCGCGGATAGAgcnccnAn

>Bacillus_Fam_15_52_14 Nr. of seq. 14 Alignment length(with gaps) = 54
Alignment score = 0.680301
GCF_000374345.1_ASM37434v1_genomic.fna_44:21064-21532 Satlength=469 Nr of Repeats=9 RepeatLength=52 seed=ATGAACGCCC Num.seqs=9 Similarity=0.580460
0   ATGAACGCCCGCGAGCACGGATTTT-NTNCCGGCACGGTCTTTCATACC-CNT-
GCF_000374345.1_ASM37434v1_genomic.fna_5:37937-38145 Satlength=209 Nr of Repeats=4 RepeatLength=52 seed=GGCTTTCATT Num.seqs=4 Similarity=0.803419
36   ATGAACGACCACGGGCACGGGTTTT-CCACGGGAACGGGCTTTCATTCCCCTT-
GCF_000374345.1_ASM37434v1_genomic.fna_22:25197-25509 Satlength=313 Nr of Repeats=6 RepeatLength=52 seed=ATGAACGACC Num.seqs=6 Similarity=0.724109
52   ATGAACGACCATGGGCGCGGCTTTT-TCTTGGGAACGGGCATTCATTCGCCGT-
Rev.of_GCF_000374345.1_ASM37434v1_genomic.fna_9:32790-34036 Satlength=1247 Nr of Repeats=24 RepeatLength=52 seed=GGTCGTTCAT Num.seqs=20 Similarity=0.767414
62   ATGAACGACCGNGCGCGCGGGTTTTTCT-CCGGCACGGGCTTTCATTCCTCCT-
Rev.of_GCF_000374345.1_ASM37434v1_genomic.fna_42:4571-4935 Satlength=365 Nr of Repeats=7 RepeatLength=52 seed=GAATGAAAGC Num.seqs=7 Similarity=0.738240   99
ATGAACGACCGGAAGCGCGGGTTTTACT-CCGGCNTGGGCTTTCATTCCACTT-
Rev.of_GCF_000374345.1_ASM37434v1_genomic.fna_13:64454-65078 Satlength=625 Nr of Repeats=12 RepeatLength=52 seed=CACGGTCGTT Num.seqs=12 Similarity=0.837995
65   ATGAACGACCGTGGGCGCGGATTTT-CTTCCGGCACGGGCTTTCATTTCCCTT-
GCF_000374345.1_ASM37434v1_genomic.fna_29:21895-22466 Satlength=572 Nr of Repeats=11 RepeatLength=52 seed=GGGCTTTCAT Num.seqs=10 Similarity=0.851852
87   ATGAACGACCGTGGGCGGGGATTTT-CTCCCGGCGCGGGCTTTCATTCCCCTT-
Rev.of_GCF_000374345.1_ASM37434v1_genomic.fna_57:9268-10100 Satlength=833 Nr of Repeats=16 RepeatLength=52 seed=AATGAAAGCC Num.seqs=16 Similarity=0.831838   98
ATGAACGACCGCGGGCACGAATTTT-CTTCCGACGCGGGCTTTCATTTCTCCT-
Rev.of_GCF_000374345.1_ASM37434v1_genomic.fna_43:29468-30092 Satlength=625 Nr of Repeats=12 RepeatLength=52 seed=GTCGTTCATA Num.seqs=12 Similarity=0.758353
113   ATGAACGACCGCGATCATGGTTTTT-CTTCCAGCGCGGGCTTTCATTCCCCTT-
Rev.of_GCF_000374345.1_ASM37434v1_genomic.fna_53:47-463 Satlength=417 Nr of Repeats=8 RepeatLength=52 seed=GGTCGTTCAT Num.seqs=8 Similarity=0.782051
114   ATGAACGACCGCGCACGTGGGTTTT-CTTCCGCCGCGGGCTTTCATTCCCCTT-
GCF_000374345.1_ASM37434v1_genomic.fna_99:27-339 Satlength=313 Nr of Repeats=6 RepeatLength=52 seed=CTTATGAACG Num.seqs=6 Similarity=0.769231
153   ATGAACGACCGCGCACACGGGTTTT-CTCCCGCCGCGGGCTTTCATTCCCCTT-
Rev.of_GCF_000374345.1_ASM37434v1_genomic.fna_47:7566-7826 Satlength=261 Nr of Repeats=5 RepeatLength=52 seed=GGTCGTTCAT Num.seqs=5 Similarity=0.691358   62
ATGAACGACCNCGAACNCAGATTTT-CCTCCGTCACGGGCTTTCATTCCACTT-
Rev.of_GCF_000374345.1_ASM37434v1_genomic.fna_8:71582-72050 Satlength=469 Nr of Repeats=9 RepeatLength=52 seed=ATGAAAGCCC Num.seqs=9 Similarity=0.760117   45
ATGAACGACCGCGGGTGCTGTTTTT-CTTCCGGTGCGGGCTTTCAT-CGCCTTG

GCF_000374345.1_ASM37434v1_genomic.fna_84:32-604 Satlength=573 Nr of Repeats=10 RepeatLength=52 seed=ATGAACGCCC Num.seqs=9 Similarity=0.650594
52 ATGAACGCCCGTGGANGCGGAAATT-CTTCCGGNGCGGTCTTTCATTCGCCTT-

******* ** ** ** * ***** * *
Consensus:

ATGAACGACCgcGggCgcGGnTTTTCttCCGgcgCGGGCTTTCATTCccCtT

>Bacillus_Fam_16_52_14 Nr. of seq. 14 Alignment length(with gaps) = 54 Alignment score = 0.664021
GCF_900156875.1_PRJEB18969_genomic.fna_11:432073-432853 Satlength=781 Nr of Repeats=11 RepeatLength=52 seed=ATGAGAACCG Num.seqs=7 Similarity=0.762482 0
ATGAGAACCGAAACAAG-TTCAG-CGAGAGCAGTAAACGGTCATCATGAAGGCG
GCF_900156875.1_PRJEB18969_genomic.fna_11:453409-453827 Satlength=419 Nr of Repeats=8 RepeatLength=52 seed=TCATGAAGGA Num.seqs=6 Similarity=0.885470
41 ATGAGAACCGAAA-AAGCTCCAG-CAAAAGTGGTCAACGGTCTTCATGAAGGAG
GCF_900156875.1_PRJEB18969_genomic.fna_11:462089-462557 Satlength=469 Nr of Repeats=9 RepeatLength=52 seed=GGAGATGAGA Num.seqs=9 Similarity=0.888889
48 ATGAGAACCGAAA-GAGCTCCAG-CAAAAGTGGTCAACGGTCTTCATAAAGGAG
GCF_900156875.1_PRJEB18969_genomic.fna_11:474004-474472 Satlength=469 Nr of Repeats=9 RepeatLength=52 seed=AGAACCGAAA Num.seqs=9 Similarity=0.812678
3 ATGAGAACCGAAA-GAGGNCCAG-CGGAANCAGGNAATGGTCTTCATAAGAGGG
GCF_900156875.1_PRJEB18969_genomic.fna_11:497598-498797 Satlength=1200 Nr of Repeats=24 RepeatLength=52 seed=ATGAGAACCG Num.seqs=18 Similarity=0.715978 52
ATGAGAACCGAAA-GAGGTCCAG-CGCAATCAGGAAACGGTCTTCATAAGAGGN
GCF_900156875.1_PRJEB18969_genomic.fna_11:530839-531463 Satlength=625 Nr of Repeats=12 RepeatLength=52 seed=ATGAGAACCG Num.seqs=12 Similarity=0.810463 52
ATGAGAACCGAAA-GAGGTCNAG-CGCAATCAGGNAACGGTCTTCATAAGAGGA
GCF_900156875.1_PRJEB18969_genomic.fna_11:542001-542937 Satlength=937 Nr of Repeats=18 RepeatLength=52 seed=GAGAACCGAA Num.seqs=18 Similarity=0.709819 54
ATGAGAACCGAAA-GAGATTCAG-CGCAAGCAGCNAACGGTCTTCATAAGAAGA
GCF_900156875.1_PRJEB18969_genomic.fna_11:824930-825451 Satlength=522 Nr of Repeats=10 RepeatLength=52 seed=AAATGGTCTT Num.seqs=9 Similarity=0.879630 32
ATGAGAACCGAAA-GAGGATTAG-CGGAANCAGTAAATGGTCTTCATAGAAGGA
GCF_900156875.1_PRJEB18969_genomic.fna_11:488438-488698 Satlength=261 Nr of Repeats=5 RepeatLength=52 seed=GTCTTCATAA Num.seqs=5 Similarity=0.871795
37 ATGAGAACCGAAA-GAGGTTGAG-TGCAAGCGGCCGACGGTCTTCATAAAGGGA
GCF_900156875.1_PRJEB18969_genomic.fna_11:526444-527550 Satlength=1107 Nr of Repeats=20 RepeatLength=53 seed=GATGAGAACC Num.seqs=14 Similarity=0.777855 51
ATGAGAACCGAAA-GAAGTCCAGCCGCAAGCGGGTAACGGTCATCATAAAGGGG
GCF_900156875.1_PRJEB18969_genomic.fna_11:480683-481359 Satlength=677 Nr of Repeats=13 RepeatLength=52 seed=GGTCTTCATA Num.seqs=13

Similarity=0.514423 36
ATGAGAACCGNAA-NAGGACCAG-NGNTAGGAGGAANCGGTCTTCATAAAGGAG
GCF_900156875.1_PRJEB18969_genomic.fna_11:540121-540433 Satlength=313 Nr of Repeats=6 RepeatLength=52 seed=CGGTCTTCAT Num.seqs=6 Similarity=0.841026
87 ATGAGAACCGAAA-CAGGATCAG-CGGTAGAAGGAAACGGTCTTCATAAAGGAA
GCF_900156875.1_PRJEB18969_genomic.fna_11:512429-513105 Satlength=677 Nr of Repeats=13 RepeatLength=52 seed=CGGTCTTCAT Num.seqs=11 Similarity=0.790744 87
ATGAGAACCGATC-CAGGATCAG-TGGAAGCAAGAACCGGTCTTCATAAAGGAG
GCF_900156875.1_PRJEB18969_genomic.fna_11:443658-443970 Satlength=313 Nr of Repeats=6 RepeatLength=52 seed=GACCGAAAGA Num.seqs=6 Similarity=0.928205
5 ATGAGGACCGAAA-GATGATCAG-GGGAAGCAGAAACCGGTCATCATCAAGGGA

***** **** * ** * **** ****
Consensus:

ATGAGAACCGAAAgAGgtccAGcGnaAgcaGgaAaCGGTCtTCATaAagGgg

>Bacillus_Fam_17_12_14 Nr. of seq. 14 Alignment length(with gaps) = 14 Alignment score = 0.607535
GCF_000292245.2_ASM29224v2_genomic.fna_17:22115-22158 Satlength=44 Nr of Repeats=4 RepeatLength=11 seed=TTTTTACTTT Num.seqs=3 Similarity=1.000000
0 -TTTTTACTTTC--
Rev.of_GCF_000290995.1_Baci_cere_AND1407_G13175_V1_genomic.fna_1:1238234-1238324 Satlength=91 Nr of Repeats=7 RepeatLength=12 seed=AAAGGAAAAG Num.seqs=6 Similarity=1.000000 4 CTTT-T-CCTTTTC
Rev.of_GCF_000305495.1_BacPsy2.0_genomic.fna_263:11580-11634 Satlength=55 Nr of Repeats=4 RepeatLength=12 seed=GAAAAAGAAA Num.seqs=3 Similarity=0.925926 12
CTTT-T-CTTTTTC
GCF_000299035.1_ASM29903v1_genomic.fna_3:203147-203195 Satlength=49 Nr of Repeats=4 RepeatLength=12 seed=TTTCTTTTTC Num.seqs=4 Similarity=0.888889
15 CTTTTT-CTTTTT-
GCF_000712615.1_ASM71261v1_genomic.fna_1:55537-55585 Satlength=49 Nr of Repeats=4 RepeatLength=12 seed=TTCTTTTTCT Num.seqs=4 Similarity=0.944444
16 CTTTTT-CTTTTT-
GCF_000775975.1_ASM77597v1_genomic.fna_1:5340410-5341236 Satlength=827 Nr of Repeats=6 RepeatLength=12 seed=CTTTTTCTTT Num.seqs=4 Similarity=0.833333 18
CTTTTT-CTTTTT-
Rev.of_GCF_001439965.1_ASM143996v1_genomic.fna_122:196979-197027 Satlength=49 Nr of Repeats=4 RepeatLength=12 seed=AAGAAAAAGA Num.seqs=4 Similarity=0.925926 15
CTTTTT-CTTTTT-
GCF_000380245.2_ASM38024v2_genomic.fna_8:153502-153556 Satlength=55 Nr of Repeats=4 RepeatLength=12 seed=TTCTTTTTCT Num.seqs=3 Similarity=0.925926
16 CTTTTT-CTTCTT-
Rev.of_GCF_001654695.1_ASM165469v1_genomic.fna_2:100746-101358 Satlength=613 Nr of Repeats=4 RepeatLength=12 seed=TGAAAAAGAA Num.seqs=3 Similarity=1.000000 20
CTTTTT-CATATT-

GCF_900109925.1_IMG-taxon_2651870116_annotated_assembly_genomic.fna_21:131911-131965 Satlength=55 Nr of Repeats=4 RepeatLength=12 seed=TTTTCTTTTT Num.seqs=3 Similarity=0.925926 20 CTTTTT-CATTTT-
GCF_000430765.1_ASM43076v1_genomic.fna_20:41962-42022 Satlength=61 Nr of Repeats=5 RepeatLength=12 seed=ATCTTTATCT Num.seqs=5 Similarity=1.000000 4 CTTTAT-CTTTAT-
GCF_000621445.1_ASM62144v1_genomic.fna_4:61862-61916 Satlength=55 Nr of Repeats=4 RepeatLength=12 seed=TCTTTGTCTT Num.seqs=3 Similarity=0.925926 5 CTTTGT-CTTTGT-
Rev.of_GCF_001315065.1_ASM131506v1_genomic.fna_4:107347-107395 Satlength=49 Nr of Repeats=4 RepeatLength=12 seed=AAGGATAAAG Num.seqs=4 Similarity=0.925926 4 CCTTGT-CTTTAT-
GCF_001591585.1_ASM159158v1_genomic.fna_33:29459-29615 Satlength=157 Nr of Repeats=13 RepeatLength=12 seed=TTCTTTATCT Num.seqs=13 Similarity=1.000000 4 CTATTT-CTTTAT-

* * * *
Consensus:

CTTTtTCtTTtT

>Bacillus_Fam_18_52_13 Nr. of seq. 13 Alignment length(with gaps) = 57 Alignment score = 0.612086
GCF_001712755.1_ASM171275v1_genomic.fna_1:340959-341167 Satlength=209 Nr of Repeats=4 RepeatLength=52 seed=GTTCATGAGT Num.seqs=4 Similarity=0.827044
0 -GTTCATGAGTAAGTAAAAAGGAGAA-AAAT-TG--ATTTATAGTAACTCATAGAGA
Rev.of_GCF_001712755.1_ASM171275v1_genomic.fna_34:211464-211828 Satlength=365 Nr of Repeats=6 RepeatLength=52 seed=TTTTACTTAC Num.seqs=5 Similarity=0.800000 18 -GTTCATGAGTAAGTAAAAAAGTGAA-AATG-AG--AAATTTAGTAACTCATAGAGG
Rev.of_GCF_001712755.1_ASM171275v1_genomic.fna_34:282789-283049 Satlength=261 Nr of Repeats=5 RepeatLength=52 seed=CTTACTCATG Num.seqs=5 Similarity=0.953846 65 -GTTCATGAGTAAGTAAAAAAG-GAA-AAAGAAG--AAATATAGTAACTCATAAAGG
GCF_001712755.1_ASM171275v1_genomic.fna_2:181356-181770 Satlength=415 Nr of Repeats=8 RepeatLength=52 seed=GGTTCATGAG Num.seqs=7 Similarity=0.877900
0 GGTTCATGAGTAAGTAAAAATG-NAAGAAAT-CG--CGATTTAGTGACTCATA-ATA
GCF_001712755.1_ASM171275v1_genomic.fna_2:298439-299011 Satlength=573 Nr of Repeats=11 RepeatLength=52 seed=TACTCATAAG Num.seqs=11 Similarity=0.876457 40
-GTTCATGAGTAAGTAAAATAG-CAA-AAANATG-AAAA-ATGGTTACTCATAAGGA
GCF_001712755.1_ASM171275v1_genomic.fna_33:281199-281458 Satlength=260 Nr of Repeats=5 RepeatLength=52 seed=TTGGTTACTC Num.seqs=4 Similarity=0.829060 87
-CTTCATGAGTATGTACAAAAG-CAA-AAACATG-ATAA-TTGGTTACTCATAAGGG
Rev.of_GCF_001712755.1_ASM171275v1_genomic.fna_9:99562-99924 Satlength=363 Nr of Repeats=7 RepeatLength=52 seed=GTAAGTTATT Num.seqs=5 Similarity=0.958974 43
-GTTCATGAGTAAGTAAAAAAG-CAA-AAAGATG-AAAA-TAACTTACTCATAGGAG

Rev.of_GCF_001712755.1_ASM171275v1_genomic.fna_9:90464-91454
Satlength=991 Nr of Repeats=5 RepeatLength=52 seed=TTTGCTGTTT Num.seqs=3
Similarity=0.888889 77
-GTTCATGAGTAAGTAAAACAG-CAA-AAATAAG-AAAA-AAGGTNCCGCATGAAGG
Rev.of_GCF_001712755.1_ASM171275v1_genomic.fna_33:181107-181419
Satlength=313 Nr of Repeats=6 RepeatLength=52 seed=ACTTACTCAT Num.seqs=6
Similarity=0.941880 118 -
GTTCATGAGTAAGTAAAACAG-CAA-AAAGAAG-CAAA-TAGGTTACTCATGAGTG
GCF_001712755.1_ASM171275v1_genomic.fna_5:85395-85810 Satlength=416 Nr of
Repeats=8 RepeatLength=52 seed=TAACTCATAA Num.seqs=7 Similarity=0.934066
91 -GTTCATGAGTAAGTAAAACAG-CGA-GAATCTG--AAACTTAGTAACTCATAAGAG
GCF_001712755.1_ASM171275v1_genomic.fna_11:53806-54274 Satlength=469 Nr
of Repeats=9 RepeatLength=52 seed=ATGAGTAAGT Num.seqs=9
Similarity=0.906695
108 -GTTCATGAGTAAGTAAAAGAG-CAN-GAATCCG--AAATATAGTTCCTCATAAAGG
GCF_001712755.1_ASM171275v1_genomic.fna_13:154313-154676 Satlength=364 Nr
of Repeats=7 RepeatLength=52 seed=TACTTACTCA Num.seqs=6
Similarity=0.928205 140
-GCTCATGAGTAAGTAAAACAG-CAA-AAATCAGAGAAA--TACTTACTCATAACAG
Rev.of_GCF_001712755.1_ASM171275v1_genomic.fna_35:106552-106864
Satlength=313 Nr of Repeats=6 RepeatLength=52 seed=ACTTACTCAT Num.seqs=6
Similarity=0.886792 170 -
GTTCATGAGTAAGTAAAACAG-TAA-AAAGAAG-GAAA-GTACTTACTCATAAAGG

********* *** ** *   *   *   * * ***
Consensus:

GTTCATGAGTAAGTAAAAnAGcAAAAAnanGnAAAntagTtACTCATAaagg

>Bacillus_Fam_19_53_13 Nr. of seq. 13 Alignment length(with gaps) = 57
Alignment score = 0.601589
GCF_000708755.2_ASM70875v2_genomic.fna_1:435766-436081 Satlength=316 Nr
of Repeats=6 RepeatLength=53 seed=TGCTGCCACT Num.seqs=5
Similarity=0.904402
0 TGCTGCCACTTTCATCGGAAG--AGGAACCTCATTTGTGGCGTTATTC--GAGGTAG
GCF_000709935.2_ASM70993v2_genomic.fna_1:450592-450857 Satlength=266 Nr
of Repeats=5 RepeatLength=53 seed=TTTGTGGCGT Num.seqs=5
Similarity=0.924528
31 TGCTGCCACTTTCATCGGAAG--AGGAACCTCATTTGTGGCGTTATTC--GAGGTAG
Rev.of_GCF_000708755.2_ASM70875v2_genomic.fna_2:1471847-1472112
Satlength=266 Nr of Repeats=5 RepeatLength=53 seed=ACCACTCTGG Num.seqs=5
Similarity=0.869182 4
TGGTACCACTTTTATCGGAAG--TTGATCCACATTAGTGGCGTCATTC--GCCAGAG
Rev.of_GCF_000709935.2_ASM70993v2_genomic.fna_5:422395-422607
Satlength=213 Nr of Repeats=4 RepeatLength=53 seed=GAATGACGCC Num.seqs=4
Similarity=0.882600 46
TGGTGCCACTTTAATCGGAAG--TTGATCCACATTTGTGGCGTCATTC--TCCAGAG
GCF_000709935.2_ASM70993v2_genomic.fna_15:156751-157015 Satlength=265 Nr
of Repeats=5 RepeatLength=53 seed=CTCATTTGTG Num.seqs=4
Similarity=0.899371
27 TGCTGCCACTTTTATCTGAGG--TGAATCCTCATTTGTGGCGCCATTC--GCCCGAG
Rev.of_GCF_000708755.2_ASM70875v2_genomic.fna_6:281885-282097
Satlength=213 Nr of Repeats=4 RepeatLength=53 seed=CGCCACAAAC Num.seqs=4

Similarity=0.890985 40
TGCTGCCACTTTT-CATGT-GAAAGCATCCATGTTTGTGGCGTCATTC-CCC-GTAG
Rev.of_GCF_000709935.2_ASM70993v2_genomic.fna_15:188408-188619
Satlength=212 Nr of Repeats=4 RepeatLength=53 seed=CGCCACAAAC Num.seqs=3
Similarity=0.899371 40
TGCCGCCACTTTT-CATGT-GAAAGCATCCATGTTTGTGGCGTCATTC-CCC-GTAG
GCF_000708755.2_ASM70875v2_genomic.fna_22:121820-122085 Satlength=266 Nr of Repeats=5 RepeatLength=53 seed=TGGCGTCATT Num.seqs=5
Similarity=0.899371
88 TGCTGCCACTTTTGCCGGA-G-AAGGGTTCTTGTTTGTGGCGTCATTC-CGC-GAAG
GCF_000709935.2_ASM70993v2_genomic.fna_24:232406-232777 Satlength=372 Nr of Repeats=7 RepeatLength=53 seed=TGGCGTCATT Num.seqs=7
Similarity=0.901767
88 TGCTGCCACTTTTGCCAGA-G-AAGGGTTCTNGTTTGTGGCGTCATTC-CGC-GTAG
GCF_000709935.2_ASM70993v2_genomic.fna_1:491832-492044 Satlength=213 Nr of Repeats=4 RepeatLength=53 seed=ATCCTAGTTT Num.seqs=4
Similarity=0.809671
77 TGGTGCCACTTTTATCGTA-G-TTGGATCCTAGTTTGTGGCGTCATTC--GCTGTAG
Rev.of_GCF_000709935.2_ASM70993v2_genomic.fna_5:571905-572276
Satlength=372 Nr of Repeats=7 RepeatLength=53 seed=ACGAATGACG Num.seqs=7
Similarity=0.841869 101
TGCCGCCACTTTTACCAGA-G-ATGGATGATGGTTAGTGGCGTCATTC--GTTGTAG
GCF_000709935.2_ASM70993v2_genomic.fna_4:195544-195756 Satlength=213 Nr of Repeats=4 RepeatLength=53 seed=CCACTTTTTC Num.seqs=4
Similarity=0.895178
111 TGACGCCACTTTTTCCAGT-G-ATGGATCCTCATTTGTGGCGTCATTCA-GC-TTAG
Rev.of_GCF_000709935.2_ASM70993v2_genomic.fna_15:32457-32775
Satlength=319 Nr of Repeats=5 RepeatLength=53 seed=CACTACCGCG Num.seqs=4
Similarity=0.756813 161
TGGCGCCACTTTTGCCTTT-G-ATGAATGCTCATTTGTGGCGTCATTC--GCGGTAG

```
**   *******        *            ** ******  ****       **
```

Consensus:

TGctGCCACTTTtacCnGaGatGgATcCtcaTTTGTGGCGTCATTCgcngtAG

>Bacillus_Fam_20_52_13 Nr. of seq. 13 Alignment length(with gaps) = 53
Alignment score = 0.740364
GCF_000175075.1_ASM17507v1_genomic.fna_7:33338-33546 Satlength=209 Nr of Repeats=4 RepeatLength=52 seed=ATGAAGACCT Num.seqs=4 Similarity=0.867521
0 ATGAAGACCTGAGTGACCAGGAAAAAGGAGAAGAGATGTCCTCATC-GCCGTG
GCF_000175075.1_ASM17507v1_genomic.fna_34:118251-118459 Satlength=209 Nr of Repeats=4 RepeatLength=52 seed=AGTCCTCATA Num.seqs=4
Similarity=0.910256
36 ATGAAGACCTGAGTGACGAGGAAAAAGGAGGAGAGAAGTCCTCATA-GCGGTG
GCF_000175075.1_ASM17507v1_genomic.fna_36:211307-211515 Satlength=209 Nr of Repeats=4 RepeatLength=52 seed=TCAGTGACGA Num.seqs=4
Similarity=0.854701
9 ATGAAGACCTCAGTGACGAGGAAAAAGGACGAGAGACGTCCTCATC-GCCGTG
GCF_000175075.1_ASM17507v1_genomic.fna_36:173392-173704 Satlength=313 Nr of Repeats=6 RepeatLength=52 seed=GGTATGAAGA Num.seqs=6
Similarity=0.865199
48 -TGAAGACCTGAGTGACAAGGAAAACGGAGAAGAGAAGTCCTCATCGGCGGTA

Rev.of_GCF_000175075.1_ASM17507v1_genomic.fna_29:100527-100736 Satlength=210 Nr of Repeats=4 RepeatLength=52 seed=GGTCTTCATA Num.seqs=3 Similarity=0.880342 9
ATGAAGACCTCAGTGGCAAAGAAAAAGGAGAAGAGAGGTTCTCATC-GCCGTT
GCF_000175075.1_ASM17507v1_genomic.fna_39:32383-32643 Satlength=261 Nr of Repeats=5 RepeatLength=52 seed=GTTATGAAGA Num.seqs=5 Similarity=0.902564
49 ATGAAGACNCCAGTGACAAGGAAAAAGGAGGAGAGAAGTCTTCATC-GCTGTT
GCF_000175075.1_ASM17507v1_genomic.fna_42:230894-231206 Satlength=313 Nr of Repeats=5 RepeatLength=52 seed=GTGATGAAGA Num.seqs=4 Similarity=0.860587
49 ATGAAGACATGAGTGACGAGGAAAAAGGAATAGAGGAGTCCTCATG-GTTGTG
Rev.of_GCF_000175075.1_ASM17507v1_genomic.fna_31:61128-61440 Satlength=313 Nr of Repeats=6 RepeatLength=52 seed=TCACTGATGT Num.seqs=6 Similarity=0.900855 16
ATGAAGACATCAGTGACGGGAAATTAGGAGGAGAGAGGTCCTCATC-GGTGTT
GCF_000175075.1_ASM17507v1_genomic.fna_32:122785-122993 Satlength=209 Nr of Repeats=4 RepeatLength=52 seed=CGTTATGAAG Num.seqs=4 Similarity=0.841880
48 ATGAAGACATCAGTGACGAGAAATTAGGAGGAGAGAAGTCCTCATT-AGCGTT
GCF_000175075.1_ASM17507v1_genomic.fna_32:237136-237500 Satlength=365 Nr of Repeats=7 RepeatLength=52 seed=CCGTTATGAA Num.seqs=7 Similarity=0.931624
99 ATGAAGACATCAGTGACGAGAAATCAGGAGGAGAGATGTCCTCATC-ACCGTT
GCF_000175075.1_ASM17507v1_genomic.fna_34:65970-66178 Satlength=209 Nr of Repeats=4 RepeatLength=52 seed=GTTATGAAGA Num.seqs=4 Similarity=0.876068
49 ATGAAGACTTCAGTGACGAAGAATTAGGAGGAGAAAGGTCCTCATC-GCCGTT
GCF_000175075.1_ASM17507v1_genomic.fna_39:143077-143336 Satlength=260 Nr of Repeats=5 RepeatLength=52 seed=GTCCTCATCG Num.seqs=4 Similarity=0.910256
37 ATGAAGACCTCAGTGACGAAAAATCAGGAGGAGAGAAGTCCTCATC-GCCCGT
GCF_000175075.1_ASM17507v1_genomic.fna_42:276011-276219 Satlength=209 Nr of Repeats=4 RepeatLength=52 seed=GAGAGAAGTC Num.seqs=4 Similarity=0.846154
82 ATGAAGACATCAGTGACGAGAAATCAGGAGGAGAGAAGTCCTCATC-GGCCGT

*******   **** *   **   *** ***   ** ****
Consensus:

ATGAAGACnTcAGTGACgAggAAaaAGGAGgAGAGAaGTCCTCATcGccGTt

>Bacillus_Fam_21_36_13 Nr. of seq. 13 Alignment length(with gaps) = 36 Alignment score = 0.755935
GCF_000007825.1_ASM782v1_genomic.fna_1:4360738-4360990 Satlength=253 Nr of Repeats=7 RepeatLength=36 seed=TGTTATCCAT Num.seqs=7 Similarity=0.827160
0 TGTTATCCATAATCGGCATTATATTCGGCGGTTGGT
Rev.of_GCF_000299035.1_ASM29903v1_genomic.fna_1:106604-107612 Satlength=1009 Nr of Repeats=28 RepeatLength=36 seed=ATGATGGATA Num.seqs=28 Similarity=0.959436
13 TATTATCCATCATTGGCATCATATTCGGCATTTGGT
GCF_000712615.1_ASM71261v1_genomic.fna_1:665208-666336 Satlength=1129 Nr of Repeats=27 RepeatLength=36 seed=TTATCCATCA Num.seqs=26 Similarity=0.882051 38
TATTATCCATCATTGGCATTATGTTTGGCATTTGGT

GCF_000496285.1_ASM49628v1_genomic.fna_1:1627516-1628017 Satlength=502 Nr of Repeats=11 RepeatLength=36 seed=GGCATCATAT Num.seqs=10 Similarity=0.912757 14 TGTTATCCATAATCGGCATCATATTCGGCATTTGAT
GCF_000831065.1_ASM83106v1_genomic.fna_1:4140717-4141077 Satlength=361 Nr of Repeats=10 RepeatLength=36 seed=TTGTTATCCA Num.seqs=10 Similarity=0.897942 35 TGTTATCCATAATTGGCATCATATTCGGCATTTGAT
Rev.of_GCF_000775975.1_ASM77597v1_genomic.fna_1:459960-460176 Satlength=217 Nr of Repeats=6 RepeatLength=36 seed=ATAATGGATA Num.seqs=6 Similarity=0.935802 49 TGTTATCCATTATTGGCATCATGTTTGGCATTTGGT
Rev.of_GCF_000832605.1_ASM83260v1_genomic.fna_1:1527851-1528355 Satlength=505 Nr of Repeats=14 RepeatLength=36 seed=ATAATGGATA Num.seqs=14 Similarity=0.853073
49 TGTTATCCATTATCGGCATCATGTTTGGCATTTGAT
Rev.of_GCF_001645555.1_ASM164555v1_genomic.fna_1:182266-182692 Satlength=427 Nr of Repeats=8 RepeatLength=36 seed=AATATGATGC Num.seqs=6 Similarity=0.859259 61 TGTTATCCATTATCGGCATCATATTTGGCATTTGAT
GCF_000007845.1_ASM784v1_genomic.fna_1:4233572-4233860 Satlength=289 Nr of Repeats=5 RepeatLength=36 seed=GGTGGTTGAT Num.seqs=4 Similarity=0.981481
26 TGTTATCCATGATTGGCATAATGTTTGGTGGTTGAT
GCF_000008505.1_ASM850v1_genomic.fna_1:4223082-4223442 Satlength=361 Nr of Repeats=6 RepeatLength=36 seed=GGTGGTTGAT Num.seqs=4 Similarity=0.888889
26 TATTATCCATGATCGGCATAATGTTTGGTGGTTGAT
Rev.of_GCF_000290995.1_Baci_cere_AND1407_G13175_V1_genomic.fna_2:421558-421882 Satlength=325 Nr of Repeats=9 RepeatLength=36 seed=TGGATAACAA Num.seqs=9 Similarity=0.842593 45 TGTTATCCATAATTGGCATGATGTTTGGTGGTTGAT
GCF_000017425.1_ASM1742v1_genomic.fna_1:3190001-3190505 Satlength=505 Nr of Repeats=14 RepeatLength=36 seed=GATTATTTTC Num.seqs=14 Similarity=0.928775 33 TATTTTCCGTCATCGGCATAATGTTTGGCATTGGAT
Rev.of_GCF_000712595.1_ASM71259v1_genomic.fna_7:58-238 Satlength=181 Nr of Repeats=5 RepeatLength=36 seed=CCGATGCCAA Num.seqs=5 Similarity=0.933333
34 TATTCCCCATCATTGGCATTACATTTGGCATCGGGT

```
* **  ** * ** ***** *  ** **     * *
```
Consensus:

TgTTATCCATnATtGGCATnATgTTtGGcatTTGaT

>Bacillus_Fam_22_53_12 Nr. of seq. 12 Alignment length(with gaps) = 56 Alignment score = 0.655032
GCF_002019665.1_ASM201966v1_genomic.fna_1:26343-26811 Satlength=469 Nr of Repeats=9 RepeatLength=52 seed=GTTCCCTCTA Num.seqs=9 Similarity=0.864672
0 GTTCCCTCTATTT-TCGTTTTTC-AC-GATTTCAGGGCACGAT-TACCCCTTGATC
Rev.of_GCF_002019665.1_ASM201966v1_genomic.fna_1:250907-251218 Satlength=312 Nr of Repeats=6 RepeatLength=52 seed=GATCAAGGCG Num.seqs=5

Similarity=0.911950 0
GTTCCCTCTATTC-TCTTTTTTCGAA-G-TTTCAGGGCACGAT-GACGCCTTGATC
GCF_002019665.1_ASM201966v1_genomic.fna_1:2689409-2689721 Satlength=313
Nr of Repeats=6 RepeatLength=52 seed=TTGATCGTTA Num.seqs=6
Similarity=0.943590 46
GTTACCTCTATTT-TCACTTTCC-CC-AATTTGAGGGCACGAT-TAGACCTTGATC
Rev.of_GCF_002019665.1_ASM201966v1_genomic.fna_1:1816049-1816621
Satlength=573 Nr of Repeats=11 RepeatLength=52 seed=AAAAGAGAGG
Num.seqs=11 Similarity=0.875991
14 GTTCCCTCTCTTT-TNGTTTTTG-GC-AATTTCAGGGCACGATCGGCGCC-TCATC
Rev.of_GCF_002019665.1_ASM201966v1_genomic.fna_1:422515-422775
Satlength=261 Nr of Repeats=5 RepeatLength=52 seed=ATCGTGCCCT Num.seqs=5
Similarity=0.969231 40
GTTCCCTCTTTTTCTCGTTTTTC-GG--TTTTGAGGGCACGAT-TACGCTCTGATC
Rev.of_GCF_002019665.1_ASM201966v1_genomic.fna_1:584690-585472
Satlength=783 Nr of Repeats=15 RepeatLength=52 seed=CCCTCAAAAA
Num.seqs=11 Similarity=0.894639
86 GTTCCCTCTTTTTCTCTTTTTTC-GT--TTTTGAGGGCACGAT-CACGCGCTGATC
Rev.of_GCF_002019665.1_ASM201966v1_genomic.fna_1:1391349-1391817
Satlength=469 Nr of Repeats=9 RepeatLength=52 seed=GGGAACGATC Num.seqs=9
Similarity=0.933048 58
GTTCCCCCTTTTTCTCGTTTTTC-GT--TTTTGAGGGAACGAT-TATGCTCTGATC
Rev.of_GCF_002019665.1_ASM201966v1_genomic.fna_1:1774595-1775572
Satlength=978 Nr of Repeats=11 RepeatLength=52 seed=AAAAGAGGGA
Num.seqs=9 Similarity=0.883191
64 GTTCCCTCTTTTTCTCATTTTTC-AT--TTTTGAGGGCACGAT-CACGCTCCGATC
Rev.of_GCF_002019665.1_ASM201966v1_genomic.fna_1:1834884-1835195
Satlength=312 Nr of Repeats=6 RepeatLength=52 seed=GAACGATTAG Num.seqs=5
Similarity=0.894872 56
GTTCCACCTATTTCTCTTTTTTC-GT--TTTTGAGGGCACGAT-CACGCGCTAATC
GCF_002019665.1_ASM201966v1_genomic.fna_1:3150058-3150578 Satlength=521
Nr of Repeats=10 RepeatLength=52 seed=GTTTTGAGGG Num.seqs=10
Similarity=0.849573 76
GTTCCCTCTATTTCGTGGTTTTC-GG--TTTTGAGGGCACGAT-CACGCTNCGATC
Rev.of_GCF_002019665.1_ASM201966v1_genomic.fna_1:1307617-1308294
Satlength=678 Nr of Repeats=13 RepeatLength=52 seed=AAGAAAATAG
Num.seqs=12 Similarity=0.815371
69 GTTCCCTCTATTT-TC-TTTTTG-GGNGTTTTGAGGGCACGAT-TGCGCTCTGATC
GCF_002019665.1_ASM201966v1_genomic.fna_1:3411347-3411970 Satlength=624
Nr of Repeats=11 RepeatLength=52 seed=TTTGCTTTTT Num.seqs=9
Similarity=0.841880 114
GTTCCCTCTATTT-GC-TTTTTC-GGCTTTTTGAGGGNACGAT-TACGCCCTGATC

*** * ** ** *** *** **** ***** * ***
Consensus:

GTTCCCTCTaTTTcTCnTTTTTCgnntTTTgAGGGCACGATtACGCncTGATC

>Bacillus_Fam_23_52_12 Nr. of seq. 12 Alignment length(with gaps) = 55
Alignment score = 0.736639
GCF_000169195.2_ASM16919v2_genomic.fna_1:334744-335940 Satlength=1197 Nr
of Repeats=21 RepeatLength=52 seed=TTTTGTCCTT Num.seqs=19
Similarity=0.483296 0
T---TTTGTCCTTTTGACNNNNTNAAAAAGACATTTCGAGCCCNGGNGCATC-CG

GCF_000169195.2_ASM16919v2_genomic.fna_1:137480-138203 Satlength=724 Nr of Repeats=13 RepeatLength=52 seed=TTTGTCCTTT Num.seqs=11 Similarity=0.651623 1
T---TTTGTCCTTTTGACAGCTTCAAAAGGACATTTCGNGCCCAGATACAGCTTG
Rev.of_GCF_000169195.2_ASM16919v2_genomic.fna_1:2466680-2467304 Satlength=625 Nr of Repeats=12 RepeatLength=52 seed=CAAAAGGACA Num.seqs=12 Similarity=0.709783
13   T---TTTGTCCTTTTGACAGCTTCAAAAGGACATTTCGGGCCCGGATGCAGCTCC
GCF_000217835.1_ASM21783v1_genomic.fna_1:2234859-2235431 Satlength=573 Nr of Repeats=11 RepeatLength=52 seed=TTTGTCCTTT Num.seqs=11 Similarity=0.715600 53
T---TTTGTCCTTTTGACAGCTTCAAAAGGACATTTCGAGCCCGGATGCAGCTCN
Rev.of_GCF_000169195.2_ASM16919v2_genomic.fna_1:979431-979847 Satlength=417 Nr of Repeats=8 RepeatLength=52 seed=TGTCCTTTTG Num.seqs=8 Similarity=0.771062 30
T---TTTGTCCTTTTGACAGGTTCAAAAGGACATTTCGTTCCCGGATGCNGCTCG
GCF_000217835.1_ASM21783v1_genomic.fna_1:335197-336033 Satlength=837 Nr of Repeats=14 RepeatLength=52 seed=TTGTCCTTTT Num.seqs=9 Similarity=0.777778
54   T---TTTGTCCTTTTGACAGGATCAAAAGGACATTTCGTTCCCGGATGCNGCACG
GCF_000832905.1_ASM83290v1_genomic.fna_1:2697159-2697888 Satlength=730 Nr of Repeats=14 RepeatLength=52 seed=TCAAAAGGAC Num.seqs=13 Similarity=0.784309 71
T---TTTGTCCTTTTGACAGGATCAAAAGGACATTTCGTTCCCGGATGCNGCACG
Rev.of_GCF_000169195.2_ASM16919v2_genomic.fna_1:320698-321219 Satlength=522 Nr of Repeats=10 RepeatLength=52 seed=CAAAAGGACA Num.seqs=9 Similarity=0.800570
13   T---TTTGTCCTTTTGACGGNTTCAAAAAGACATTTCGGGCCCGGATGCANCGTG
Rev.of_GCF_000169195.2_ASM16919v2_genomic.fna_1:1299605-1300125 Satlength=521 Nr of Repeats=9 RepeatLength=52 seed=ATGTCTTTTT Num.seqs=8 Similarity=0.791217 31   T---TTTGTCTTTTTGACGGCTTCAAAAGGACATTTCGGGCCCGGATGCAACGTG
GCF_000169195.2_ASM16919v2_genomic.fna_1:2593824-2594862 Satlength=1039 Nr of Repeats=20 RepeatLength=52 seed=AAAAAGACAT Num.seqs=18 Similarity=0.816323 21
T---TTTGTCTTTTTGGCAGGTTCAAAAAGACATTTCGGGCCCGGATGCAGCGTG
GCF_000217835.1_ASM21783v1_genomic.fna_1:921872-922184 Satlength=313 Nr of Repeats=6 RepeatLength=52 seed=TGTTTTGTCC Num.seqs=6 Similarity=0.866667
50   T---TTTGTCCCTTTGTCGGCTTCAAAAAGACATTTCGAGCCCGGATGCAACATG
Rev.of_GCF_000217835.1_ASM21783v1_genomic.fna_1:908745-908953 Satlength=209 Nr of Repeats=4 RepeatLength=52 seed=TCAAAAGGAC Num.seqs=4 Similarity=0.655136 14
TCCTTTTGTCCTTTTGACGGCTTCAAAAAGACATTTCGGGCCC--AAGCGNCA-G

*   ****** **** *   * **** ********* ***   * *
Consensus:

TTTTGTCCTTTTGACaGctTCAAAAgGACATTTCGngCCCgGATGCagCncG

>Bacillus_Fam_24_21_12 Nr. of seq. 12 Alignment length(with gaps) = 23 Alignment score = 0.627141

GCF_000307875.1_BABA1.0_genomic.fna_58:10690-10774 Satlength=85 Nr of Repeats=4 RepeatLength=21 seed=CGCTCATTGA Num.seqs=4 Similarity=0.638889
0   CGCTCATTG-ACAGC-TTAAATT
Rev.of_GCF_000307875.1_BABA1.0_genomic.fna_137:4596-4680 Satlength=85 Nr of Repeats=4 RepeatLength=21 seed=ATGAGCGAAT Num.seqs=4 Similarity=0.703704
7   CGCTCATTGAACA-T-CGAAATT
GCF_000508325.1_BAVI_1_genomic.fna_122:15786-15870 Satlength=85 Nr of Repeats=4 RepeatLength=21 seed=AATTCGCCCA Num.seqs=4 Similarity=0.642677
17   CGCCCATAGAACA-C-TGAAATT
Rev.of_GCF_001591665.1_ASM159166v1_genomic.fna_72:12480-12564 Satlength=85 Nr of Repeats=4 RepeatLength=21 seed=ATGGGCGAAT Num.seqs=4 Similarity=0.613636   28
CGCCCATTGAACT-C-TGAAATT
GCF_001591445.1_ASM159144v1_genomic.fna_17:116034-116118 Satlength=85 Nr of Repeats=4 RepeatLength=21 seed=ATTCGCTGAT Num.seqs=4 Similarity=0.820106
18   CGCTGATAGAACC-T-GGAAATT
GCF_000508325.1_BAVI_1_genomic.fna_85:26959-27124 Satlength=166 Nr of Repeats=4 RepeatLength=21 seed=GAAAATTCGC Num.seqs=3 Similarity=0.830688
14   CGCCCATTGAACC-C-GAAAATT
GCF_000508325.1_BAVI_1_genomic.fna_92:110555-110639 Satlength=85 Nr of Repeats=4 RepeatLength=21 seed=ATTCGCCCAT Num.seqs=4 Similarity=0.512731
18   CGCCCATTGAANG-C-TAAAATT
Rev.of_GCF_001591665.1_ASM159166v1_genomic.fna_58:4850-4934 Satlength=85 Nr of Repeats=4 RepeatLength=21 seed=AATGGGCGAA Num.seqs=4 Similarity=0.625604   29
CGCCCATTGAACG-T-CAAAATT
GCF_000508325.1_BAVI_1_genomic.fna_87:23723-23807 Satlength=85 Nr of Repeats=4 RepeatLength=21 seed=AATTCGCCCA Num.seqs=4 Similarity=0.717172
39   CGCCCATTGAACC-TGCAAAATT
Rev.of_GCF_000508325.1_BAVI_1_genomic.fna_105:66812-66896 Satlength=85 Nr of Repeats=4 RepeatLength=21 seed=ATGGGCGAAT Num.seqs=4 Similarity=0.589372   27
CGCCCATTGAAAC-CC-GAAATT
Rev.of_GCF_000508325.1_BAVI_1_genomic.fna_180:2145-2229 Satlength=85 Nr of Repeats=4 RepeatLength=21 seed=ATGGGCGAAT Num.seqs=4 Similarity=0.671958
27   CGCCCATTGAACA-CC-NAAATT
Rev.of_GCF_001591665.1_ASM159166v1_genomic.fna_60:47968-48052 Satlength=85 Nr of Repeats=4 RepeatLength=21 seed=ATGGGCGAAT Num.seqs=4 Similarity=0.664141   27
CGCCCATTGAATA-CC-AAAATT

*** ** * *   *****
Consensus:

CGCcCATTGAAcncnnAAATT

>Bacillus_Fam_25_20_12 Nr. of seq. 12 Alignment length(with gaps) = 21 Alignment score = 0.723545
GCF_000813125.1_ASM81312v1_genomic.fna_20:18031-18110 Satlength=80 Nr of Repeats=4 RepeatLength=20 seed=TTTTTATAGC Num.seqs=3 Similarity=0.656566
0   TTTTTAT-AGCGANAATTTCT

Rev.of_GCF_001315165.1_ASM131516v1_genomic.fna_16:40325-40425
Satlength=101 Nr of Repeats=5 RepeatLength=20 seed=CGCTATAAAA Num.seqs=5
Similarity=0.563636 11
NTTTTAT-AGCGAAAATTTNA
GCF_001315165.1_ASM131516v1_genomic.fna_27:5637-5717 Satlength=81 Nr of
Repeats=4 RepeatLength=20 seed=TTTTATAGCG Num.seqs=4 Similarity=0.666667
21 TTTTTAT-AGCGAAAATTTCA
Rev.of_GCF_001315165.1_ASM131516v1_genomic.fna_79:1018-1119 Satlength=102
Nr of Repeats=5 RepeatLength=20 seed=GCTATAAAAA Num.seqs=4
Similarity=0.711640 30
TTTTTAT-AGCGAAAAGTTTA
GCF_000813125.1_ASM81312v1_genomic.fna_39:13920-14020 Satlength=101 Nr of
Repeats=4 RepeatLength=20 seed=TTTTTATAGC Num.seqs=3 Similarity=0.611111
0 TTTTTAT-AGCGAAAATTCAN
Rev.of_GCF_001315165.1_ASM131516v1_genomic.fna_53:15227-15327
Satlength=101 Nr of Repeats=5 RepeatLength=20 seed=TCGCTATAAA Num.seqs=5
Similarity=0.621739 12
TTTTTAT-AGCGAAATTTTAN
Rev.of_GCF_000813125.1_ASM81312v1_genomic.fna_73:60683-60763 Satlength=81
Nr of Repeats=4 RepeatLength=20 seed=TTCGCTATAA Num.seqs=4
Similarity=0.578283 13
TTTTTAT-AGCGAAAATCTAG
Rev.of_GCF_001315165.1_ASM131516v1_genomic.fna_10:70726-70826
Satlength=101 Nr of Repeats=5 RepeatLength=20 seed=TCGCTATAAA Num.seqs=5
Similarity=0.593333 32
TTTTTAT-AGCGAAAATCTNG
Rev.of_GCF_001315165.1_ASM131516v1_genomic.fna_15:43524-43604
Satlength=81 Nr of Repeats=4 RepeatLength=20 seed=TTTCGCTATA Num.seqs=4
Similarity=0.667989 14
TTTTTAT-AGCGAAAATCTAA
Rev.of_GCF_001591465.1_ASM159146v1_genomic.fna_6:87823-87925
Satlength=103 Nr of Repeats=5 RepeatLength=20 seed=TCGCTCATAA Num.seqs=3
Similarity=0.606061 12
-TTTTATGAGCGAAAATTTAN
GCF_000813125.1_ASM81312v1_genomic.fna_65:30212-30291 Satlength=80 Nr of
Repeats=4 RepeatLength=20 seed=TTATAGCGAA Num.seqs=3 Similarity=0.644444
3 ATATTAT-AGCGAAAATNTAN
Rev.of_GCF_001315165.1_ASM131516v1_genomic.fna_16:47524-47624
Satlength=101 Nr of Repeats=5 RepeatLength=20 seed=TTTTCGCTAT Num.seqs=5
Similarity=0.544000 15
TTTNTAT-AGCGAAAANAAAN

* *** ***** *
Consensus:

tTTTTATAGCGAAAATtTan

>Bacillus_Fam_26_53_11 Nr. of seq. 11 Alignment length(with gaps) = 58
Alignment score = 0.666458
BACILLUS_PSEUDALCALIPHILUS_genomic.fna_1:484476-484813 Satlength=338 Nr of
Repeats=7 RepeatLength=53 seed=GGTTAGTCGA Num.seqs=5 Similarity=0.869182
0 GGTTAGTCGACATGATCATGGGCAAAAAAGGTCGAGAACCTGTCGATGAGG---AC--

BACILLUS_PSEUDALCALIPHILUS_genomic.fna_2:427050-427315 Satlength=266 Nr of Repeats=5 RepeatLength=53 seed=CATGTCGATG Num.seqs=5 Similarity=0.854088
38 GGTTGGTCGACATGATCTTGGGCCAAAAAGGACGAGAACATGTCGATGAAG---GC--
Rev.of_BACILLUS_PSEUDALCALIPHILUS_genomic.fna_6:417351-418025 Satlength=675 Nr of Repeats=12 RepeatLength=53 seed=CTCGACCTTT Num.seqs=10 Similarity=0.911111
89 AGTTGGTCGACATGATCATGGGTAAAAAAGGTCGAGATCATGTCGATGAAG---GC--
BACILLUS_PSEUDALCALIPHILUS_genomic.fna_5:150308-150520 Satlength=213 Nr of Repeats=4 RepeatLength=53 seed=CGATGAAGGA Num.seqs=4 Similarity=0.861635
42 -GTTGGTCGACATGATCAAGAGCAAAAAAGGTCAAGAACCTGTCGATGAAG---GAG-
BACILLUS_PSEUDALCALIPHILUS_genomic.fna_5:327893-328213 Satlength=321 Nr of Repeats=6 RepeatLength=53 seed=TGTCGATGAA Num.seqs=4 Similarity=0.928721
92 -GCTGGTCGACATGAACATGAGTAAAAAAGGCCAAGAACCTGTCGATGAAG---GAA-
BACILLUS_PSEUDALCALIPHILUS_genomic.fna_5:528815-529185 Satlength=371 Nr of Repeats=7 RepeatLength=53 seed=CTGTCGATGA Num.seqs=6 Similarity=0.921174
144 -GCTGGTCGACATGATCATGAGTAAAAAAGGTCAAGAACCTGTCGATGAAG---GAA-
BACILLUS_PSEUDALCALIPHILUS_genomic.fna_16:65979-66245 Satlength=267 Nr of Repeats=5 RepeatLength=53 seed=GACATGATCA Num.seqs=4 Similarity=0.928721
60 -GCTGGTCGACATGATCATGGGCAAAAAAGCTGAAGAACCTGTCGATGAAG---GAA-
BACILLUS_PSEUDALCALIPHILUS_genomic.fna_1:496997-497526 Satlength=530 Nr of Repeats=10 RepeatLength=53 seed=GACATGATCA Num.seqs=9 Similarity=0.889588 8
GGTTAGTCGACATGATCACGAGAAAAAAAGGGTAAGAACCTGTCGATAAAGA--G---
BACILLUS_PSEUDALCALIPHILUS_genomic.fna_10:92314-92960 Satlength=647 Nr of Repeats=12 RepeatLength=53 seed=GTGGTCGACA Num.seqs=11 Similarity=0.828931 1
AG-TGGTCGACATGATCAGGANGAAAAAAGGCNGAGAACCTGTCGATGAAG--GGC--
BACILLUS_PSEUDALCALIPHILUS_genomic.fna_5:157629-157946 Satlength=318 Nr of Repeats=6 RepeatLength=53 seed=AGAACATGTC Num.seqs=5 Similarity=0.904402
32 --ATGGACGACATGATCATGGGCAAAAAAGTCGAAGAACATGTCGATGA-G--TGAGT
BACILLUS_PSEUDALCALIPHILUS_genomic.fna_4:6835-7366 Satlength=532 Nr of Repeats=10 RepeatLength=53 seed=TGGTCGACAT Num.seqs=9 Similarity=0.831962
3 CGTTGGTCGACATGATGTAGGTTGAAAAAGGGCCGGATCATGTCGATGA-G-AAG---

* * ******** * ****** ** * ******* * *
Consensus:

nGtTGGTCGACATGATCAtGgGnAAAAAAGGncaAGAACcTGTCGATGAaGGn

>Bacillus_Fam_27_39_11 Nr. of seq. 11 Alignment length(with gaps) = 40 Alignment score = 0.799394
GCF_000007825.1_ASM782v1_genomic.fna_1:4361056-4361329 Satlength=274 Nr of Repeats=7 RepeatLength=39 seed=CTACATTTTC Num.seqs=7 Similarity=0.810298
0 CTACATTTTCTTTCTTCACTTGCGGCGATACTATATT-TC
GCF_000007845.1_ASM784v1_genomic.fna_1:4233977-4234409 Satlength=433 Nr of Repeats=7 RepeatLength=39 seed=TTTTCTTTCT Num.seqs=6 Similarity=0.920228
5 CTACATTTTCTTTCTTCACTTGCGGTGATACTATATT-TC
GCF_000008505.1_ASM850v1_genomic.fna_1:4223515-4223749 Satlength=235 Nr of Repeats=6 RepeatLength=39 seed=CTACATTTTC Num.seqs=6 Similarity=0.872365
0 CTACATTTTCTTTCTTTACTTGCGGTGATACTATATT-TC

GCF_000496285.1_ASM49628v1_genomic.fna_1:1628212-1628485 Satlength=274 Nr of Repeats=7 RepeatLength=39 seed=ACATTTTCTT Num.seqs=7 Similarity=0.863248 2
CTACATTTTCTTTCTTTACTTGCGGCGATACTATGTT-TC
Rev.of_GCF_000290995.1_Baci_cere_AND1407_G13175_V1_genomic.fna_2:421236-421470 Satlength=235 Nr of Repeats=6 RepeatLength=39 seed=ATAGTGTCAC Num.seqs=6 Similarity=0.872365 34
CTACATTTTCTTTCTTTACTTGTGGTGACACTATGTT-TC
Rev.of_GCF_001645555.1_ASM164555v1_genomic.fna_1:181927-182083 Satlength=157 Nr of Repeats=4 RepeatLength=39 seed=ATAGTATCAC Num.seqs=4 Similarity=0.914530 34
CTACATTTTCTTTCTTTACTTGTGGTGATACTATGTT-TC
Rev.of_GCF_000775975.1_ASM77597v1_genomic.fna_1:459197-459950 Satlength=754 Nr of Repeats=13 RepeatLength=39 seed=GCAAGTGAAG Num.seqs=12 Similarity=1.000000
23 CTACATTCTCTTTCTTCACTTGCGGCGACACTATATT-CC
Rev.of_GCF_000832605.1_ASM83260v1_genomic.fna_1:1527503-1527698 Satlength=196 Nr of Repeats=5 RepeatLength=39 seed=CCGCAAGTGA Num.seqs=5 Similarity=0.945299 25
CTACATTNTCTTTCTTCACTTGCGGCGACACTATATT-CC
GCF_000831065.1_ASM83106v1_genomic.fna_1:4141206-4141440 Satlength=235 Nr of Repeats=6 RepeatLength=39 seed=GACACTATAT Num.seqs=6 Similarity=0.906553 26
CTACATTTTCTTTTTTCACTTGCGGCGACACTATATT-TC
GCF_000161455.1_ASM16145v1_genomic.fna_1:3733849-3734302 Satlength=454 Nr of Repeats=5 RepeatLength=39 seed=TTTTCTTTTT Num.seqs=3 Similarity=0.749288 5
TTACATTTTCTTTTTTCATTTGCGGCGCAACTATATT-AC
GCF_000017425.1_ASM1742v1_genomic.fna_1:3190635-3191025 Satlength=391 Nr of Repeats=10 RepeatLength=39 seed=ATTTTCTTTT Num.seqs=10 Similarity=0.900475 3
-TATATTTTCTTTTTTCACTTGTGGTGCTGCGATATTCTC

** *** ***** ** * *** ** * * ** ** *
Consensus:

CTACATTTTCTTTcTTcACTTGcGGcGAtACTATaTTtC

>Bacillus_Fam_28_21_11 Nr. of seq. 11 Alignment length(with gaps) = 25 Alignment score = 0.604364
GCF_001645685.2_ASM164568v2_genomic.fna_1:3317387-3317534 Satlength=148 Nr of Repeats=7 RepeatLength=21 seed=TTTACTCGCC Num.seqs=7 Similarity=0.848828 0
TTTACTCGCCAATT--CGCG-GGC-
GCF_001645685.2_ASM164568v2_genomic.fna_1:3318620-3318745 Satlength=126 Nr of Repeats=6 RepeatLength=21 seed=TTTACTCGCC Num.seqs=5 Similarity=0.815873 0
TTTACTCGCCAATT--CGCA-NGC-
GCF_001645685.2_ASM164568v2_genomic.fna_1:3443422-3443693 Satlength=272 Nr of Repeats=12 RepeatLength=21 seed=CTTTACTCGC Num.seqs=11 Similarity=0.826446 20
TTTACTCGCCAATT--CGAG-TGC-
GCF_001645685.2_ASM164568v2_genomic.fna_1:3920495-3920579 Satlength=85 Nr of Repeats=4 RepeatLength=21 seed=CTCGCCAATT Num.seqs=4

Similarity=0.851852 25
TTTACTCGCCAATT--CGAG-CAC-
GCF_001645685.2_ASM164568v2_genomic.fna_1:3972827-3973253 Satlength=427 Nr of Repeats=9 RepeatLength=21 seed=TTTACTCGCC Num.seqs=6
Similarity=0.729101 0
TTTACTCGCCAATT--CG-G-GGNT
GCF_001645685.2_ASM164568v2_genomic.fna_1:3656001-3656127 Satlength=127 Nr of Repeats=6 RepeatLength=21 seed=CTCGCCAATT Num.seqs=6
Similarity=0.856085 4
TTTACTCGCCAATT-CCG-G-TCG-
GCF_001645685.2_ASM164568v2_genomic.fna_1:4110462-4110546 Satlength=85 Nr of Repeats=4 RepeatLength=21 seed=TTTACTCGCC Num.seqs=4
Similarity=0.683081 21
TTTACTCGCCAATT-TCG-G-CGT-
Rev.of_GCF_001645685.2_ASM164568v2_genomic.fna_1:180575-180782 Satlength=208 Nr of Repeats=6 RepeatLength=21 seed=TGGCGAGTAA Num.seqs=5
Similarity=0.660606 32
TTTACTCGCCAATTNTCG-G-TGN-
GCF_001645685.2_ASM164568v2_genomic.fna_1:4286180-4286302 Satlength=123 Nr of Repeats=4 RepeatLength=21 seed=TTTACTCGCC Num.seqs=3
Similarity=0.647343 0
TTTACTCGCCAATT--TGNA-CGA-
GCF_001645685.2_ASM164568v2_genomic.fna_1:4375763-4376192 Satlength=430 Nr of Repeats=11 RepeatLength=21 seed=TTTACTCGCC Num.seqs=7
Similarity=0.788600 0
TTTACTCGCCAATT--TGAG-CNG-
GCF_001645685.2_ASM164568v2_genomic.fna_1:4377725-4378156 Satlength=432 Nr of Repeats=11 RepeatLength=21 seed=TTTACTCGCC Num.seqs=7
Similarity=0.753968 0
TTTACTCGCCAATT--TNAGNCT--

****
Consensus:

TTTACTCGCCAATTcGnGngn

>Bacillus_Fam_29_20_11 Nr. of seq. 11 Alignment length(with gaps) = 22
Alignment score = 0.720386
GCF_000813125.1_ASM81312v1_genomic.fna_10:26480-26581 Satlength=102 Nr of Repeats=4 RepeatLength=20 seed=TTTATTGGCG Num.seqs=3 Similarity=0.676768
10 ATNTT--CAAATTTTATTGGCG
GCF_000813125.1_ASM81312v1_genomic.fna_92:129270-129372 Satlength=103 Nr of Repeats=5 RepeatLength=20 seed=TTTATTGGCG Num.seqs=3
Similarity=0.622222
10 ATTTT--NAAATTTTATTGGCG
GCF_000813125.1_ASM81312v1_genomic.fna_26:19181-19302 Satlength=122 Nr of Repeats=6 RepeatLength=20 seed=TTTATTGGCG Num.seqs=5 Similarity=0.771429
10 ATATT--CTAATTTTATTGGCG
GCF_000813125.1_ASM81312v1_genomic.fna_128:14716-14818 Satlength=103 Nr of Repeats=5 RepeatLength=20 seed=TTTATTGGCG Num.seqs=3
Similarity=0.671958
10 ATATT--TTAATTTTATTGGCG

GCF_000813125.1_ASM81312v1_genomic.fna_40:39070-39170 Satlength=101 Nr of Repeats=4 RepeatLength=20 seed=TTATTGGCGA Num.seqs=3 Similarity=0.688889
11 ATTTT--CTAATTTTATTGGCG
Rev.of_GCF_001315165.1_ASM131516v1_genomic.fna_54:20708-20789 Satlength=82 Nr of Repeats=4 RepeatLength=20 seed=TCGCCAATAA Num.seqs=3 Similarity=0.622222 21
ATTTT--CNAATTTTATTGGCG
GCF_000813125.1_ASM81312v1_genomic.fna_69:8482-8584 Satlength=103 Nr of Repeats=5 RepeatLength=20 seed=TTATTGGCGA Num.seqs=3 Similarity=0.560606
11 ANTTT--CANATTTTATTGGCG
GCF_000813125.1_ASM81312v1_genomic.fna_144:11821-11921 Satlength=101 Nr of Repeats=5 RepeatLength=20 seed=TTTTATTGGC Num.seqs=5 Similarity=0.723810
29 ATTTT--CAGTTTTTATTGGCG
Rev.of_GCF_000934845.1_ASM93484v1_genomic.fna_189:8715-8817 Satlength=103 Nr of Repeats=5 RepeatLength=20 seed=CGCCAATAAA Num.seqs=3 Similarity=0.755556 20
ATAAT--CANATTTTATTGGCG
GCF_000813125.1_ASM81312v1_genomic.fna_40:72461-72583 Satlength=123 Nr of Repeats=6 RepeatLength=20 seed=TTATTGGCGA Num.seqs=4 Similarity=0.633102
32 ATAATC-CAAATTTTATTGGCG
Rev.of_GCF_000934845.1_ASM93484v1_genomic.fna_68:108728-108809 Satlength=82 Nr of Repeats=4 RepeatLength=20 seed=CGCCAATAAA Num.seqs=3 Similarity=0.666667 19
ATTTT-TTNCAT-TTATTGGCG

********** * *
Consensus:

TTTATTGGCGATtTTcaaAT

>Bacillus_Fam_30_18_11 Nr. of seq. 11 Alignment length(with gaps) = 19 Alignment score = 0.651675
GCF_000007825.1_ASM782v1_genomic.fna_1:2602019-2602271 Satlength=253 Nr of Repeats=13 RepeatLength=18 seed=CCTGTTACTC Num.seqs=12 Similarity=0.975309 0
CCTGTTACTCCAGTTGGA-
Rev.of_GCF_000712595.1_ASM71259v1_genomic.fna_10:146400-146544 Satlength=145 Nr of Repeats=8 RepeatLength=18 seed=GGTCCAACTG Num.seqs=8 Similarity=0.928571 2
CCTGTTGCTCCAGTTGGA-
GCF_000775975.1_ASM77597v1_genomic.fna_1:2396734-2396859 Satlength=126 Nr of Repeats=7 RepeatLength=18 seed=GGACCCGTTA Num.seqs=6 Similarity=0.906173 15
CCCGTTACTCCCGTTGGA-
GCF_000832605.1_ASM83260v1_genomic.fna_1:3447183-3447381 Satlength=199 Nr of Repeats=11 RepeatLength=18 seed=GTTGGACCCG Num.seqs=11 Similarity=0.935354 30
CCCGTTACTCCTGTTGGA-
Rev.of_GCF_001307105.1_ASM130710v1_genomic.fna_16:4592-4880 Satlength=289 Nr of Repeats=14 RepeatLength=18 seed=CCAACGGGAG Num.seqs=10 Similarity=0.840329 17
CCGGTTACTCCCGTTGGG-

Rev.of_GCF_001584325.1_ASM158432v1_genomic.fna_5:37432-37900 Satlength=469 Nr of Repeats=19 RepeatLength=18 seed=ACCGGAGTAA Num.seqs=12 Similarity=0.787347
14 CCTGTTACTCCGGTT-GAC
Rev.of_GCF_000305495.1_BacPsy2.0_genomic.fna_162:38-785 Satlength=748 Nr of Repeats=21 RepeatLength=18 seed=CAGGAGCAAC Num.seqs=13 Similarity=0.711651 4
CCTGTTACTCCTGTTGCT-
GCF_900096965.1_IMG-taxon_2671180223_annotated_assembly_genomic.fna_5:141837-142206 Satlength=370 Nr of Repeats=20 RepeatLength=18 seed=CCTGTCACTC Num.seqs=19 Similarity=0.843621 18
CCTGTCACTCCNGTTGCT-
Rev.of_GCF_000831065.1_ASM83106v1_genomic.fna_1:2131967-2132240 Satlength=274 Nr of Repeats=13 RepeatLength=18 seed=GGAGCAACAG Num.seqs=10 Similarity=0.856790
20 CCTGTTAAACCTGTTGCT-
Rev.of_GCF_000380245.2_ASM38024v2_genomic.fna_12:1413968-1414058 Satlength=91 Nr of Repeats=5 RepeatLength=18 seed=ACAGGAGTAA Num.seqs=5 Similarity=0.770000 14
CCAGTTACTCCTGTCTCA-
GCF_001307105.1_ASM130710v1_genomic.fna_2:66844-66916 Satlength=73 Nr of Repeats=4 RepeatLength=18 seed=TCCTGTGGCA Num.seqs=4 Similarity=0.802469
26 CCCGTAACTCCTGTGGCA-

** ** ** **
Consensus:

CCtGTTACTCCnGTTGga

>Bacillus_Fam_31_51_10 Nr. of seq. 10 Alignment length(with gaps) = 56 Alignment score = 0.632407
GCF_000752035.1_Bacillus_rubiinfantis_genomic.fna_11:206605-207962 Satlength=1358 Nr of Repeats=18 RepeatLength=51 seed=GTTGTCCATT Num.seqs=17 Similarity=0.781297 0
GTTGTCCATTAGAA-GCGNGCTATTGGACANGAATCTGG---CGAATAGTGAAAA-
Rev.of_GCF_000752035.1_Bacillus_rubiinfantis_genomic.fna_11:595095-595401 Satlength=307 Nr of Repeats=6 RepeatLength=51 seed=GACAACTTTT Num.seqs=6 Similarity=0.752621 6
GTTGTCCAATAGAA-GCGGTCTAATGGACAAGAAACTGG---CGGAAAGCGAAAA-
Rev.of_GCF_000752035.1_Bacillus_rubiinfantis_genomic.fna_12:6507-6864 Satlength=358 Nr of Repeats=7 RepeatLength=51 seed=TGTCCATTAG Num.seqs=7 Similarity=0.750649 29
GTTGTCCAATAGAA-GCGGTCTAATGGACAAGAATCCGG---TGAAAAGGGAAAA-
Rev.of_GCF_000752035.1_Bacillus_rubiinfantis_genomic.fna_16:36137-37771 Satlength=1635 Nr of Repeats=32 RepeatLength=51 seed=GACAACTTTT Num.seqs=30 Similarity=0.860404 6
GTTGTCCAATAGAA-GCGGTCTAATGGACAAGAAT-TCGA--GAAAANGCGAAAA-
Rev.of_GCF_000752035.1_Bacillus_rubiinfantis_genomic.fna_30:179952-181023 Satlength=1072 Nr of Repeats=21 RepeatLength=51 seed=TTGTCCATTA Num.seqs=21 Similarity=0.886905 30
GATGTCCAATAGAA-GCAGTCTAATGGACAAGAATCTCG---TAAAAAGCGAAAA-
Rev.of_GCF_000752035.1_Bacillus_rubiinfantis_genomic.fna_33:32726-35276 Satlength=2551 Nr of Repeats=50 RepeatLength=51 seed=TTGGACAGCT

Num.seqs=50 Similarity=0.848494 60
GCTGTCCAATAGAA-GCGGTCTAATGGACAAGAATNTGG---TAAAAAGCGAAAA-
Rev.of_GCF_000752035.1_Bacillus_rubiinfantis_genomic.fna_11:535628-535985 Satlength=358 Nr of Repeats=6 RepeatLength=51 seed=CCAATAGACC Num.seqs=5 Similarity=0.921569 25 -
TTGTCCAATAGAA-GAGGTCTATTGGACAAAAATTCGG---TAAAATCCAGAAAG
Rev.of_GCF_000752035.1_Bacillus_rubiinfantis_genomic.fna_12:28322-28577 Satlength=256 Nr of Repeats=5 RepeatLength=51 seed=CACCTTCTAT Num.seqs=5 Similarity=0.932026 68 -
CTGTCCAATAGAA-GGTGTCTATTGGACAAAAACAGAG---AGAAACGCGAAAAG
Rev.of_GCF_000752035.1_Bacillus_rubiinfantis_genomic.fna_11:525341-525954 Satlength=614 Nr of Repeats=12 RepeatLength=51 seed=TGTCCATTAG Num.seqs=11 Similarity=0.773310 29
GTTGTCCAATAGGAGGCG-TCTAATGGACAAGAATCTAG-TTTTTCAA--GAAAA-
Rev.of_GCF_000752035.1_Bacillus_rubiinfantis_genomic.fna_11:608592-608898 Satlength=307 Nr of Repeats=6 RepeatLength=51 seed=CAACTTTTCT Num.seqs=6 Similarity=0.925054 55
GTTGTCCGATAGGA-GCGTTCTAATGGACAAGAAACTGG-ATTTTTAA--GAAAA-

***** *** * * *** ****** ** * ***
Consensus:

gtTGTCCAATAGaAGcggTCTAaTGGACAAgAAtctgGtnaaAagcGAAAA

>Bacillus_Fam_32_52_10 Nr. of seq. 10 Alignment length(with gaps) = 56 Alignment score = 0.659061
GCF_001439965.1_ASM143996v1_genomic.fna_95:88322-88842 Satlength=521 Nr of Repeats=10 RepeatLength=52 seed=TTTCGTTACC Num.seqs=10 Similarity=0.717710 0
TTTCGTTACCCTGGACACNCGTTTTCCTCCTTCTCGGTAACCAA--ACGCTCCC--
GCF_001887185.1_ASM188718v1_genomic.fna_43:14953-15212 Satlength=260 Nr of Repeats=5 RepeatLength=52 seed=TTTCGTTACC Num.seqs=4 Similarity=0.759259
0 TTTCGTTACCCTGCACAACCATTTTCCTCCTTCTCGGTAACCAATCACGCTTCC--
Rev.of_GCF_001439965.1_ASM143996v1_genomic.fna_97:186923-187395 Satlength=473 Nr of Repeats=8 RepeatLength=52 seed=AAAAAGCGTG Num.seqs=6 Similarity=0.810256 25
TTTAGTTACCCTGCACACGCTTTTTCTCCTTCTCGGTAATCATT--CGCTCAC--
GCF_001887185.1_ASM188718v1_genomic.fna_124:9302-9509 Satlength=208 Nr of Repeats=4 RepeatLength=52 seed=TTTTCCTCCT Num.seqs=3 Similarity=0.871795
21 TTTGTTACCNGACACACATATTTTCCTCCTTCTCGGTAACCAAT--CACTCTC--
Rev.of_GCF_001887185.1_ASM188718v1_genomic.fna_447:7-267 Satlength=261 Nr of Repeats=5 RepeatLength=52 seed=ACCGAGAAGG Num.seqs=5 Similarity=0.887179 38
TTTTGTTACCCGACACACATATTTTCCTCCTTCTCGGTAACCAAT--CACTCTC--
GCF_002009555.1_ASM200955v1_genomic.fna_401:30074-30282 Satlength=209 Nr of Repeats=4 RepeatLength=52 seed=CTCCTTCTCG Num.seqs=4 Similarity=0.897436
26 TTTTGTTACCAGACACACATATTTTCCTCCTTCTCGGTAACCAAT--CGCTCTC--
GCF_002009555.1_ASM200955v1_genomic.fna_259:2631-2838 Satlength=208 Nr of Repeats=4 RepeatLength=52 seed=CTCCTTCTCG Num.seqs=3 Similarity=0.709402
26 TTTCGTTACCCGACACACACATTTTCCTCCTTCTCGGTCACCAAT--CGCTCTC--
Rev.of_GCF_001439965.1_ASM143996v1_genomic.fna_122:142659-143596 Satlength=938 Nr of Repeats=18 RepeatLength=52 seed=AGAGTTTGAT

Num.seqs=17 Similarity=0.797323
49   TTTCGTTACCGGGCTCGCGCTATTTCCTCCTTCANGGTAATCAAA--CTCT-TC-C
Rev.of_GCF_002009555.1_ASM200955v1_genomic.fna_552:2070-2278
Satlength=209 Nr of Repeats=4 RepeatLength=52 seed=GGTAACGAAA Num.seqs=4
Similarity=0.820513   63
TTTCGTTACCGTGCTCGCGCAATTTTCTCCTTCAAGGTAATCAAT--CGC--TCTC
Rev.of_GCF_002009555.1_ASM200955v1_genomic.fna_138:2615-3236
Satlength=622 Nr of Repeats=10 RepeatLength=52 seed=GAGAGTTTGA
Num.seqs=8 Similarity=0.873626
49   TTTGGTTACCGTGCACGCTCATTTTCCTCCTTCAAAGGAATCAAA--CTCTCTC--

*** ******   *   *** *******   * * **   * *   *
Consensus:

TTTcGTTACCcggCaCaCncatTTTcCTCCTTCtcGGTAAcCAAtCgCTctC

>Bacillus_Fam_33_52_10 Nr. of seq. 10 Alignment length(with gaps) = 55
Alignment score = 0.634747
GCF_000177235.2_ASM17723v2_genomic.fna_1:18432-18848 Satlength=417 Nr of
Repeats=8 RepeatLength=52 seed=GTGTATCATA Num.seqs=8 Similarity=0.638889
0   GTGTATCATACGAGG-CCAATGACACGCGAGAAAGTA-G-TAGGAACGAAATAAA
GCF_000177235.2_ASM17723v2_genomic.fna_1:43977-44445 Satlength=469 Nr of
Repeats=9 RepeatLength=52 seed=AATAAAGTGT Num.seqs=7 Similarity=0.689594
46   GTGTATCATACGAAG-GCAATGACACGNGAGAAAGTA-G-TGGGAACGNAATAAA
GCF_000177235.2_ASM17723v2_genomic.fna_1:112827-113243 Satlength=417 Nr
of Repeats=8 RepeatLength=52 seed=AATAAAGTGT Num.seqs=6
Similarity=0.628283
47   GTGTATCATACGAAG-GCAATGACACATAAGAAAGTAGG-GAGGAACGGAATAAA
Rev.of_GCF_000177235.2_ASM17723v2_genomic.fna_1:2556156-2556519
Satlength=364 Nr of Repeats=7 RepeatLength=52 seed=ACACTTTATT Num.seqs=6
Similarity=0.648148   4
GTGTGTCATAGAAGT-GCAATGACACGTGAGAAAGTANG-TGGAAACG-AATAAA
Rev.of_GCF_000177235.2_ASM17723v2_genomic.fna_1:2731003-2731368
Satlength=366 Nr of Repeats=7 RepeatLength=52 seed=ACACTTTATT Num.seqs=5
Similarity=0.677987   4
GTGTATCATACGAGN-GCAATGACACGTGAGAAAGTANG-TGGAAACG-AATAAA
Rev.of_GCF_000177235.2_ASM17723v2_genomic.fna_1:3973488-3975773
Satlength=2286 Nr of Repeats=37 RepeatLength=52 seed=TTTATTTGCT
Num.seqs=27 Similarity=0.882460
0   GTGTTTCATACGNAG-TCAATGACACATGAGAATGAA-GAAAGAAGCA-AATAAA
Rev.of_GCF_000177235.2_ASM17723v2_genomic.fna_1:4069392-4069808
Satlength=417 Nr of Repeats=8 RepeatLength=52 seed=CTTCGTATGA Num.seqs=6
Similarity=0.780247   15
GTGTATCATACGAAG-TCAATGACACGTGAAAAAGCC-G-AAGAGCCAGAATAAT
Rev.of_GCF_000177235.2_ASM17723v2_genomic.fna_1:2719945-2720257
Satlength=313 Nr of Repeats=6 RepeatLength=52 seed=GCGTGTCATT Num.seqs=6
Similarity=0.817094   27
ATGTGTCATACGGGG-TCAATGACACGCGAGAAAGCT-G-AAGAATTGAAATAAG
Rev.of_GCF_000177235.2_ASM17723v2_genomic.fna_1:2784025-2784389
Satlength=365 Nr of Repeats=7 RepeatLength=52 seed=TCGTGTGTCA Num.seqs=7
Similarity=0.880204   29
ATGTATCATACAAAG-GCAATGACACACGAGAAAGAA-G-AAGAATGAAAATAAG
GCF_000177235.2_ASM17723v2_genomic.fna_1:275907-276426 Satlength=520 Nr
of Repeats=9 RepeatLength=52 seed=ATAAGATGTG Num.seqs=8

Similarity=0.823302
47   ATGTGTCATACGAGGAGCAATGCCACGCGAAAAAGTC-G-AGTCGTCAAAATAAG

*** *****   ***** ***   * ** *   *   *****
Consensus:

gTGTaTCATACgaaggCAATGACACgtGAgAAAGtaGaaGaaacgnAATAAa

>Bacillus_Fam_34_52_10 Nr. of seq. 10 Alignment length(with gaps) = 54
Alignment score = 0.660905
GCF_000186145.1_Bacillus_sp_2_A_57_CT2_V1_genomic.fna_2:429780-430040
Satlength=261 Nr of Repeats=5 RepeatLength=52 seed=TGTCCGAAGT Num.seqs=5
Similarity=0.928205   0
TGTCCGAAGTGGCGCTAAGTTCGGACTCACTCTCCCTGTTTTCTG-CT-TCTTC
GCF_000294775.2_ASM29477v2_genomic.fna_1:5091581-5091934 Satlength=354 Nr
of Repeats=5 RepeatLength=52 seed=TCCCTGTTTT Num.seqs=3
Similarity=0.931624   32
TGTCCGAAGTAGCGCTAAGTTCGGACTCACTCTCCCTGTTTTCCG-CT-TCTTC
GCF_000186145.1_Bacillus_sp_2_A_57_CT2_V1_genomic.fna_2:563975-564288
Satlength=314 Nr of Repeats=6 RepeatLength=52 seed=GATTTCTGCT Num.seqs=5
Similarity=0.864103   37
TGTCCGAAGTGGCTTCAGGTTCGGACTCTTTCTCCCCGATTTCTG-CT-TCTCC
Rev.of_GCF_000186145.1_Bacillus_sp_2_A_57_CT2_V1_genomic.fna_5:65621-
65881 Satlength=261 Nr of Repeats=5 RepeatLength=52 seed=AAGCGAAAAA
Num.seqs=5 Similarity=0.906289   48
TGTCCGAACTCGCACCAACTTCGGACTCCTTCTCTCCCTTTTTCG-CTTT-TCC
GCF_000294775.2_ASM29477v2_genomic.fna_1:2845024-2845284 Satlength=261 Nr
of Repeats=5 RepeatLength=52 seed=GCACCAACTT Num.seqs=5
Similarity=0.907692   64
TGTCCGAAGTCGCACCAACTTCGGACTCTTTCTCTCCCTTTTTCG-CT-TCTTC
GCF_000294775.2_ASM29477v2_genomic.fna_1:5362631-5363411 Satlength=781 Nr
of Repeats=15 RepeatLength=52 seed=CTGTCCGAAG Num.seqs=15
Similarity=0.870574   51
TGTCCGAAGTTACACCAAGTTCGGACTCTATCTCTCGGTTTTCCGNCT--CTCC
GCF_000186145.1_Bacillus_sp_2_A_57_CT2_V1_genomic.fna_2:825884-826248
Satlength=365 Nr of Repeats=7 RepeatLength=52 seed=CTGTCCGAAT Num.seqs=7
Similarity=0.768010   51
TGTCCGAATCNGCGCCAACTTCGGACTCATTCNCTCGGATTTCTC-CT-CCTCC
GCF_000294775.2_ASM29477v2_genomic.fna_1:5435232-5435596 Satlength=365 Nr
of Repeats=7 RepeatLength=52 seed=CTGTCCGAAT Num.seqs=7
Similarity=0.768010   51
TGTCCGAATCNGCGCCAACTTCGGACTCATTCNCTCGGATTTCTC-CT-CCTCC
GCF_000294775.2_ASM29477v2_genomic.fna_1:3419345-3419761 Satlength=417 Nr
of Repeats=8 RepeatLength=52 seed=TTCGGACTCA Num.seqs=6
Similarity=0.837607   71
TGTCCGAAGGTGCGCCGACTTCGGACTCATTCCCTCGGATTCTTG-CT-TCTTC
GCF_000294775.2_ASM29477v2_genomic.fna_1:5335150-5335462 Satlength=313 Nr
of Repeats=6 RepeatLength=52 seed=ACTTCGGACT Num.seqs=6
Similarity=0.593487   69
TGTCCGAATAACTGCCAACTTCGGACTCAACCTCTCCGATTTCTA-CT-CCTAC

********   *********   * * *   **   **   * *
Consensus:

TGTCCGAAgtngCgCcAAcTTCGGACTCatTCtCtCngaTTTctgCTtCTcC

>Bacillus_Fam_35_52_10 Nr. of seq. 10 Alignment length(with gaps) = 53
Alignment score = 0.701887
GCF_000708755.2_ASM70875v2_genomic.fna_1:11198-11612 Satlength=415 Nr of Repeats=8 RepeatLength=52 seed=TTGATGGTTC Num.seqs=7 Similarity=0.831502
0 TTGATGGTTCCGATCACGGAGATAAGTGCCATCAAAACAGAAAATCCTCNGT-
GCF_000708755.2_ASM70875v2_genomic.fna_6:420022-420334 Satlength=313 Nr of Repeats=6 RepeatLength=52 seed=TTGATGGTTC Num.seqs=6 Similarity=0.788034
0 TTGATGGTTCCTATCCCGGAGATAAGTGCCACCAAAANGGAAAATCCACCGC-
GCF_000709935.2_ASM70993v2_genomic.fna_5:627734-628098 Satlength=365 Nr of Repeats=7 RepeatLength=52 seed=TTGATGGTTC Num.seqs=7 Similarity=0.785104
0 TTGATGGTTCCTATCTCCGAGATAAGTGCCACCAAGATGGAAAATCCACCGC-
GCF_000708755.2_ASM70875v2_genomic.fna_6:153329-153693 Satlength=365 Nr of Repeats=7 RepeatLength=52 seed=GATAAGTGCC Num.seqs=7 Similarity=0.846154
20 TTGATGGTTCCGAACTCAGAGATAAGTGCCACCAAAAAGGATATTCCTCCGT-
GCF_000709935.2_ASM70993v2_genomic.fna_15:70593-70957 Satlength=365 Nr of Repeats=7 RepeatLength=52 seed=TTGATGGTTC Num.seqs=7 Similarity=0.829060
52 TTGATGGTTCCGAACTCAGAGATAAGTGCCACCAAAAATGATATTCCTCCGT-
GCF_000708755.2_ASM70875v2_genomic.fna_22:240402-240610 Satlength=209 Nr of Repeats=4 RepeatLength=52 seed=GCTTGATGGT Num.seqs=4 Similarity=0.773504
50 TTGATGGTACCGAACTCGGAGATAAGTGCCATCAAACCGGAAAA-CCGCTGGC
GCF_000709935.2_ASM70993v2_genomic.fna_24:351132-351644 Satlength=513 Nr of Repeats=10 RepeatLength=52 seed=GAGATAAGTG Num.seqs=8 Similarity=0.879605 70
TTGATGGTTCCGAACTCGGAGATAAGTGCCATCAAACGGGAGAA-ACGCTGGC
Rev.of_GCF_000708755.2_ASM70875v2_genomic.fna_1:387582-387842 Satlength=261 Nr of Repeats=5 RepeatLength=52 seed=GGAACCATCA Num.seqs=5 Similarity=0.838462 11
TTGATGGTTCCTATCTCACCGATAAGTGCCATCAAATCAGATTTCCCGCCGT-
Rev.of_GCF_000709935.2_ASM70993v2_genomic.fna_1:408489-408848 Satlength=360 Nr of Repeats=7 RepeatLength=52 seed=GGAACCATCA Num.seqs=6 Similarity=0.882051 11
TTGATGGTTCCGATCTCACCGATAAGTGCCATCAAATCAGATTCTCCGCCGC-
GCF_000709935.2_ASM70993v2_genomic.fna_1:824423-824735 Satlength=313 Nr of Repeats=6 RepeatLength=52 seed=ATAAGTGCCA Num.seqs=6 Similarity=0.840042
21 TTGATGGTACCGAACGCACCGATAAGTGCCATCAAAACAGAATTCCAACCGC-

******** ** * * * *********** *** ** * *
Consensus:

TTGATGGTtCCgAaCtCagaGATAAGTGCCAtCAAAacgGAaaatCCnCcGn

>Bacillus_Fam_36_20_10 Nr. of seq. 10 Alignment length(with gaps) = 26
Alignment score = 0.611966
GCF_001591805.1_ASM159180v1_genomic.fna_23:9242-9347 Satlength=106 Nr of Repeats=5 RepeatLength=21 seed=GTTTTGGCTA Num.seqs=5 Similarity=0.668056
7 TTCAA---G-G-GTTTTGGCTATTT-

Rev.of_GCF_001591805.1_ASM159180v1_genomic.fna_27:63705-63986 Satlength=282 Nr of Repeats=8 RepeatLength=21 seed=TAGCCAAAAC Num.seqs=5 Similarity=0.568056 17 TTCNA---G-G-GTTTTGGCTATTT-
Rev.of_GCF_000508325.1_BAVI_1_genomic.fna_15:39118-39285 Satlength=168 Nr of Repeats=8 RepeatLength=21 seed=TAGCCAAAAC Num.seqs=7 Similarity=0.753247 17 TTCAA---GAG-GTTTTGGCTATTT-
Rev.of_GCF_001591805.1_ASM159180v1_genomic.fna_27:93496-93601 Satlength=106 Nr of Repeats=5 RepeatLength=21 seed=TAGCCAAAAC Num.seqs=5 Similarity=0.651515 17 TTCGAN--G-G-GTTTTGGCTATTT-
GCF_001591805.1_ASM159180v1_genomic.fna_40:28416-28500 Satlength=85 Nr of Repeats=4 RepeatLength=21 seed=TTTTGGCTAT Num.seqs=4 Similarity=0.765700 8 TCCAA---G-G-GTTTTGGCTATTTC
GCF_000508325.1_BAVI_1_genomic.fna_48:11356-11461 Satlength=106 Nr of Repeats=5 RepeatLength=21 seed=GTTTTGGCTA Num.seqs=5 Similarity=0.647826 7 TTCAA---G-GAGTTTTGGCTACTT-
GCF_001591805.1_ASM159180v1_genomic.fna_5:147425-147508 Satlength=84 Nr of Repeats=4 RepeatLength=21 seed=GTTTTGGCTA Num.seqs=3 Similarity=0.661616
7 TTCGA---N-GAGTTTTGGCTANTT-
GCF_001591805.1_ASM159180v1_genomic.fna_7:49374-49506 Satlength=133 Nr of Repeats=5 RepeatLength=21 seed=TTTGTCTATT Num.seqs=4 Similarity=0.788360 9 TCCAA--AG-G-GTTTTGTCTATTT-
GCF_000508325.1_BAVI_1_genomic.fna_43:5059-5143 Satlength=85 Nr of Repeats=4 RepeatLength=21 seed=TTGGCTATTT Num.seqs=4 Similarity=0.714286 10 TTCNA-CCT---GTTTTGGCTATTT-
GCF_000508325.1_BAVI_1_genomic.fna_87:25255-25339 Satlength=85 Nr of Repeats=4 RepeatLength=21 seed=GTTTTGGCTA Num.seqs=4 Similarity=0.724868 28 TCTGA-CCG---GTTTTGGCTATTT-

****** *** ** * *
Consensus:

GTTTTGGCTAtTTTtCaAgg

>Bacillus_Fam_37_21_10 Nr. of seq. 10 Alignment length(with gaps) = 24 Alignment score = 0.601389
GCF_001636345.1_ASM163634v1_genomic.fna_1:1542440-1542856 Satlength=417 Nr of Repeats=20 RepeatLength=21 seed=TTTGATAGGA Num.seqs=18 Similarity=0.543615 0 TTTGATAGGAAACCCAGG-AA---
GCF_001636345.1_ASM163634v1_genomic.fna_1:847129-847687 Satlength=559 Nr of Repeats=16 RepeatLength=21 seed=TTTGATAGGA Num.seqs=14 Similarity=0.627456 0 TTTGATAGGAAACNCAGA-AAT--
GCF_001636345.1_ASM163634v1_genomic.fna_1:967178-968025 Satlength=848 Nr of Repeats=23 RepeatLength=21 seed=TTGATAGGAA Num.seqs=20 Similarity=0.549379 1 TTTGATAGGAAACANNGA-AAT--
Rev.of_GCF_001636345.1_ASM163634v1_genomic.fna_1:3423391-3423622 Satlength=232 Nr of Repeats=11 RepeatLength=21 seed=TTTCCTATCA

Num.seqs=11 Similarity=0.678237
12   TTTGATAGGAAACTGAAA-AAT--
Rev.of_GCF_001636345.1_ASM163634v1_genomic.fna_1:3846604-3846688
Satlength=85 Nr of Repeats=4 RepeatLength=21 seed=TTCCTATCAA Num.seqs=4
Similarity=0.661376   32
TTTGATAGGAAAGTNAAA-AAN--
Rev.of_GCF_001636345.1_ASM163634v1_genomic.fna_1:3810945-3811113
Satlength=169 Nr of Repeats=8 RepeatLength=21 seed=TCCTATCAAA Num.seqs=8
Similarity=0.608383   10
TTTGATAGGAAAGNCAGANAAG--
GCF_001636345.1_ASM163634v1_genomic.fna_1:481918-482023 Satlength=106 Nr of Repeats=5 RepeatLength=21 seed=TTTGATAGGA Num.seqs=5
Similarity=0.630303
0   TTTGATAGGAAAANCGAA-AAN--
GCF_001636345.1_ASM163634v1_genomic.fna_1:1838104-1838250 Satlength=147 Nr of Repeats=7 RepeatLength=21 seed=TTTGATAGGA Num.seqs=6
Similarity=0.705556   0
TTTGATAGGAAAA-CGAA-AATTN
Rev.of_GCF_001636345.1_ASM163634v1_genomic.fna_1:3259418-3259523
Satlength=106 Nr of Repeats=5 RepeatLength=21 seed=TCCTATCAAA Num.seqs=5
Similarity=0.533333   10
TTTGATAGGAAAAGCNNT-AAN--
Rev.of_GCF_001636345.1_ASM163634v1_genomic.fna_1:4039943-4040090
Satlength=148 Nr of Repeats=7 RepeatLength=21 seed=TTCCTATCAA Num.seqs=7
Similarity=0.639061   11
TTTGATAGGAAAANCGAT-TAA--

************   *
Consensus:

TTTGATAGGAAAnncaaaAAn

>Bacillus_Fam_38_15_10 Nr. of seq. 10 Alignment length(with gaps) = 15
Alignment score = 0.786667
GCF_000007825.1_ASM782v1_genomic.fna_1:428801-429311 Satlength=511 Nr of Repeats=27 RepeatLength=15 seed=CCAGAACCGT Num.seqs=22
Similarity=0.802982
0   CCAGAACCGTTACCA
GCF_000496285.1_ASM49628v1_genomic.fna_1:3052985-3053600 Satlength=616 Nr of Repeats=34 RepeatLength=15 seed=CACCAGAACC Num.seqs=27
Similarity=0.816651   13
CCAGAACCGTTGCCA
GCF_000007845.1_ASM784v1_genomic.fna_1:416172-416412 Satlength=241 Nr of Repeats=11 RepeatLength=15 seed=CCAGAACCGT Num.seqs=7
Similarity=0.881481
0   CCAGAACCGTTGCCG
Rev.of_GCF_000290995.1_Baci_cere_AND1407_G13175_V1_genomic.fna_5:83347-83542 Satlength=196 Nr of Repeats=8 RepeatLength=15 seed=TGGTTCTGGT
Num.seqs=5 Similarity=0.893333   9
CCAGAACCATTGCCA
GCF_000775975.1_ASM77597v1_genomic.fna_1:4964492-4964898 Satlength=407 Nr of Repeats=25 RepeatLength=15 seed=TGCCACCAGA Num.seqs=23
Similarity=0.846113   10
CCAGAACCATTGCCA

Rev.of_GCF_000832605.1_ASM83260v1_genomic.fna_1:71948-72608 Satlength=661 Nr of Repeats=38 RepeatLength=15 seed=TCTGGTGGCA Num.seqs=32 Similarity=0.872043 20 CCAGAACCATTGCCA
GCF_000831065.1_ASM83106v1_genomic.fna_1:282158-282248 Satlength=91 Nr of Repeats=6 RepeatLength=15 seed=TGCCACCAGA Num.seqs=6 Similarity=0.733333 10 CCAGAGCCGTTGCCA
Rev.of_GCF_001645555.1_ASM164555v1_genomic.fna_5:1617-1782 Satlength=166 Nr of Repeats=8 RepeatLength=15 seed=TCTGGTGGCA Num.seqs=6 Similarity=0.845926 5 CCAGAACCGCTGCCA
GCF_000831065.1_ASM83106v1_genomic.fna_1:282271-282451 Satlength=181 Nr of Repeats=8 RepeatLength=15 seed=GAACCGTTAC Num.seqs=5 Similarity=1.000000
3 CCGGAACCGTTACCG
GCF_001439635.1_ASM143963v1_genomic.fna_175:41594-41820 Satlength=227 Nr of Repeats=15 RepeatLength=15 seed=ATTGCCAGCT Num.seqs=14 Similarity=0.948230 8 GCTGACCCATTGCCA

* ** ** * **
Consensus:

CCaGAaCCgTTgCCa

>Bacillus_Fam_39_81_9 Nr. of seq. 9 Alignment length(with gaps) = 83 Alignment score = 0.715640
GCF_000196735.1_ASM19673v1_genomic.fna_1:1355402-1356375 Satlength=974 Nr of Repeats=13 RepeatLength=81 seed=AGCAAATTCA Num.seqs=10 Similarity=0.779313 0 AGCAAA-TTCAGCCCGGAAACGATATTAACAGAACTGGATAAGTTCACAACCTCATTCATGGGGAAAGTGGACGAAATCG-CA
GCF_000769555.1_ASM76955v1_genomic.fna_1:1225964-1227098 Satlength=1135 Nr of Repeats=13 RepeatLength=81 seed=GCAAATTCAG Num.seqs=12 Similarity=0.748152 1 AGCAAA-TTCAGCCCGGAAACGATATTAGCAGAACTGGATAAGTTCACAACNTCATTCATGGAAAAGTAGACGAAATCG-CN
GCF_000204275.1_ASM20427v1_genomic.fna_1:1272554-1273283 Satlength=730 Nr of Repeats=9 RepeatLength=81 seed=AAATTCAGTC Num.seqs=9 Similarity=0.677599 3 AGCAAA-TTCAGTCCNGAAACGATATTAACAGAGCTGGATAAATTCACGACCTCATTCATGGGGAAAGTNGACGANATCG-CN
Rev.of_GCF_000262045.1_KCTC_13613_01_genomic.fna_13:664127-664856 Satlength=730 Nr of Repeats=9 RepeatLength=81 seed=GGACTGAATT Num.seqs=7 Similarity=0.675630 14 AGCAAA-TTCAGTCCGGAAACNATTTTAACCGAGCTGGATAAGTTCACGACNTCATTTATGGGAAAAGTGGACGANATCG-CN
GCF_001584325.1_ASM158432v1_genomic.fna_36:27-513 Satlength=487 Nr of Repeats=5 RepeatLength=81 seed=GCAAATTCAG Num.seqs=4 Similarity=0.690041 0 -GCAAA-

TTCAGCCCGGAATCAATATTAAAAGAACTGGACAAATTTACGGCTTCATTTATGAAAAAAATAGACGACATCG
TCA
GCF_000245315.1_ASM24531v1_genomic.fna_63:23745-24231 Satlength=487 Nr of Repeats=6 RepeatLength=81 seed=CTGGATAAGT Num.seqs=6 Similarity=0.698217
33 A-
CAAAGTTCAGTCCGGAAACNATTCTNAAACAGCTGGATAAGTTCACAACATCATTTATGAGAAAAGTGGATGC
AATCG-CA
GCF_000245335.1_ASM24533v1_genomic.fna_10:342257-342905 Satlength=649 Nr of Repeats=6 RepeatLength=81 seed=CTGGATAAGT Num.seqs=5
Similarity=0.838683
33 A-
CAAAGTTCAGTCCAGANACGATTTTGACAGAGCTGGATAAGTTTACAACATCGTTTATGAGTAAGGTGGATGC
AATTG-CG
GCF_001719185.1_ASM171918v1_genomic.fna_1:102140-102707 Satlength=568 Nr of Repeats=7 RepeatLength=81 seed=AGCTGGATAA Num.seqs=7
Similarity=0.844797
112 A-
CGAAGTTCAGTCCAGAAACGATTTTGTCGCAGCTGGATAAATTTACAACATCGTTCATGAGTAAAGTGGACGC
AATTG-CN
GCF_001719185.1_ASM171918v1_genomic.fna_1:102277-103168 Satlength=892 Nr of Repeats=9 RepeatLength=81 seed=TTCAGTCCAG Num.seqs=6
Similarity=0.769241
169
AGCAAANTTCAGTCCAGAAACGATTTTGNCGCAGCTGGATAAGTTTACAACATCGTTCATGAGTAAAGTGGAC
GCAATCG-CA

* ** ***** ** ** * ** * * ***** ** ** ** * ** ** *** ** * ** *
** * *
Consensus:

AgCAAATTCAGtCCgGAAACgATtTTaacagAgCTGGATAAgTTcACaACnTCaTTcATGaGnAAAGTgGAcG
aaATcGCn

>Bacillus_Fam_40_52_9 Nr. of seq. 9 Alignment length(with gaps) = 58
Alignment score = 0.648467
GCF_000169195.2_ASM16919v2_genomic.fna_1:960957-961477 Satlength=521 Nr of Repeats=10 RepeatLength=52 seed=TCTTTTGTCT Num.seqs=10
Similarity=0.839316 0
TCTTTTGTCTTTTTGAGCCCTCC-A-AAAGGACAATTCGCCC-CT-GAT-C-CGGCTC
Rev.of_GCF_000217835.1_ASM21783v1_genomic.fna_1:352300-352924
Satlength=625 Nr of Repeats=12 RepeatLength=52 seed=TCAAAAAGAC
Num.seqs=12 Similarity=0.858974
16 TCTTTTGTCTTTTTGAGCTCTCC-A-AAAGGACAATTTGCTC-CT-GGT-C-CGGCTT
Rev.of_GCF_000832905.1_ASM83290v1_genomic.fna_1:2714385-2715373
Satlength=989 Nr of Repeats=19 RepeatLength=52 seed=AAAAGACAAA
Num.seqs=19 Similarity=0.858450
65 TCTTTTGTCTTTTTGAGCCCTCC-A-AAAAGACAATTTGCTC-CT-GGT-C-CGGCTC
Rev.of_GCF_000217835.1_ASM21783v1_genomic.fna_1:327324-327948
Satlength=625 Nr of Repeats=12 RepeatLength=52 seed=AAAGACAAAA
Num.seqs=12 Similarity=0.619500
12 TCTTTTGTCTTTTTGANACCCCC-A-AAAGGACAATTTG-CC-CG-GGTNC-CAGCTT
GCF_000169195.2_ASM16919v2_genomic.fna_1:989979-990499 Satlength=521 Nr of Repeats=10 RepeatLength=52 seed=CTTTTGTCTT Num.seqs=10

Similarity=0.599686 54
TCTTTTGTCTTTTTGAAACCTCC-A-AAAGGACAATTTGCCC-CG-GATNC-CAGCTC
Rev.of_GCF_000832905.1_ASM83290v1_genomic.fna_1:88046-88410 Satlength=365
Nr of Repeats=7 RepeatLength=52 seed=AAAAGGACAA Num.seqs=5
Similarity=0.769811 13
-CTTTTGTCCTTTTGAATACTCC-A-AAAGGACANTTTGCTC-CG-GTT-CATGGCTC
Rev.of_GCF_000832905.1_ASM83290v1_genomic.fna_1:2689393-2690174
Satlength=782 Nr of Repeats=15 RepeatLength=52 seed=AAAGACAAAA
Num.seqs=14 Similarity=0.488918
12 TCTTTTGTCTTTTTGATACCTCCGATGAAGGACANTTTGCNN-CGNGGT-C-CGNCTC
Rev.of_GCF_000217835.1_ASM21783v1_genomic.fna_1:298642-299112
Satlength=471 Nr of Repeats=9 RepeatLength=52 seed=AAATGTCCTT Num.seqs=7
Similarity=0.657848 35
GCTTTTGTCTTTTTGAANCCTCC-A-AAAGGACATTTCGANCACT--AC-C-CGGGCC
Rev.of_GCF_000832905.1_ASM83290v1_genomic.fna_1:2653134-2654217
Satlength=1084 Nr of Repeats=20 RepeatLength=52 seed=AAATGTCCTT
Num.seqs=18 Similarity=0.450699
35 CCTTTTGTCTTTTTGAACCCTCC-A-AAAGGACATTTCGATCACT--GN-C-CGGGTC

******** ****** * ** * ** **** ** * * *
Consensus:

tCTTTTGTCTTTTTGAnncCTCCAAAAGGACAaTTtGcnCCtggtCCgGcTc

>Bacillus_Fam_41_51_9 Nr. of seq. 9 Alignment length(with gaps) = 57
Alignment score = 0.622157
GCF_000292245.2_ASM29224v2_genomic.fna_34:46830-47085 Satlength=256 Nr of
Repeats=5 RepeatLength=51 seed=TTTTTTATTA Num.seqs=5 Similarity=0.853595
0 TTTTTTATTACTTATTACT-GGTTTTTCGCACCGTAATTTT---NACT-GGCT-GGC
Rev.of_GCF_000292245.2_ASM29224v2_genomic.fna_38:11355-11712
Satlength=358 Nr of Repeats=7 RepeatLength=51 seed=GTAATGAAAA Num.seqs=7
Similarity=0.776580 11
TTTTTCATTACTTATTACG-GGTTTTTCGCNCCTTAATTTG---GACT-GGCT-GGC
GCF_000292245.2_ASM29224v2_genomic.fna_52:29439-29796 Satlength=358 Nr of
Repeats=7 RepeatLength=51 seed=TTTTTCATTA Num.seqs=7 Similarity=0.895425
0 TTTTTCATTACCTA-TCCTCACTTTTTCGCACCGTAATTTT---GCCT--GCTCGGC
Rev.of_GCF_000292245.2_ASM29224v2_genomic.fna_57:27020-27280
Satlength=261 Nr of Repeats=5 RepeatLength=51 seed=GTAATGAAAA Num.seqs=4
Similarity=0.610063 11
TTTTTCATTACCTATTCCTCA-TTTTTCGCACCGTAATTTT---GCCTCAGCATGGC
GCF_000292245.2_ASM29224v2_genomic.fna_61:1751-1955 Satlength=205 Nr of
Repeats=4 RepeatLength=51 seed=TTTTCATTAC Num.seqs=4 Similarity=0.656061
1 TTTTTCATTACCTA-TCCGCCCTTTTTCGCACCCTAATTTG---GCCC--ACTCTCC
Rev.of_GCF_000292245.2_ASM29224v2_genomic.fna_66:18-323 Satlength=306 Nr
of Repeats=6 RepeatLength=51 seed=GTAATGAAAA Num.seqs=5
Similarity=0.724359
11 TTTTTCATTACCTATTACACTNTTTTTCGCACCCTAATTTG---GCCN--GCTCNCC
GCF_000292245.2_ASM29224v2_genomic.fna_80:17413-17718 Satlength=306 Nr of
Repeats=6 RepeatLength=51 seed=TTTCATTACC Num.seqs=5 Similarity=0.869281
2 TTTTTCATTACCTA-TTCACCCTTTTTCGCACCTTAATTTG---GCAT--TCACCGC
GCF_000292245.2_ASM29224v2_genomic.fna_53:28960-29215 Satlength=256 Nr of
Repeats=4 RepeatLength=51 seed=CGGCTTTTTC Num.seqs=3 Similarity=0.912854
47 TTTTTCATTACTTA-TCTCAAGTTTTTCGCACTCTAATTTG---ACCT--GCTCGGC

GCF_000292245.2_ASM29224v2_genomic.fna_5:9-315 Satlength=307 Nr of Repeats=6 RepeatLength=51 seed=TTTTCATTAC Num.seqs=6 Similarity=0.673939
52 TTTTTCATTACCTA-TCCCAAGTTTTTCGCACTCTAATTTGCNCACCT--GCTCGAC

***** ***** ** * ******** * ****** * *
Consensus:

TTTTTCATTACcTATcCncnnTTTTTCGCACcnTAATTTggcCtgCtcggC

>Bacillus_Fam_42_53_9 Nr. of seq. 9 Alignment length(with gaps) = 56
Alignment score = 0.615245
GCF_000181495.1_ASM18149v1_genomic.fna_69:3432-3803 Satlength=372 Nr of Repeats=6 RepeatLength=53 seed=CGACCCAACA Num.seqs=5 Similarity=0.749383
0 CGACCCAACAACCATTCAATA-ATC-CATTCTCCTTGGCCCGATTACNCCCT-TTT
GCF_000181495.1_ASM18149v1_genomic.fna_112:1323-1747 Satlength=425 Nr of Repeats=8 RepeatLength=53 seed=CGCCCTATTC Num.seqs=6 Similarity=0.775262
44 CGACCCAACATCCCCTCTATA-AGC-CAATCTCCTTGGATTGATTACGCCCT-ATT
Rev.of_GCF_000181495.1_ASM18149v1_genomic.fna_151:10383-10754 Satlength=372 Nr of Repeats=7 RepeatLength=53 seed=ACCAGTGAGA Num.seqs=7 Similarity=0.857442 37
CANACCAACATCCCTTCCATA-ATC-CGTTCTCACTGGTCTGTTTACTCTCT-TTT
Rev.of_GCF_000181495.1_ASM18149v1_genomic.fna_170:36099-36417 Satlength=319 Nr of Repeats=6 RepeatLength=53 seed=GATGTTGGTC Num.seqs=6 Similarity=0.847379 65
CAGACCAACATCCCTTCCATC-ATC-CGTTCTCATTGGTTTATTTACTNCCT-TTT
Rev.of_GCF_000181495.1_ASM18149v1_genomic.fna_186:370855-371491 Satlength=637 Nr of Repeats=9 RepeatLength=53 seed=TGAAAAGGGA Num.seqs=6 Similarity=0.812159 55
CAAACCAACATCCCTCACATA-ATC-CATTCTCATTGGTCTGATTACTCCCT-TTT
GCF_000181495.1_ASM18149v1_genomic.fna_143:4307-4677 Satlength=371 Nr of Repeats=7 RepeatLength=53 seed=CGTTTACTCC Num.seqs=6 Similarity=0.931237
38 CGCACCAACACCCCTC-CAAA-ATTTCATTCTCATTGGGCCGTTTACTCCCT-TTT
Rev.of_GCF_000181495.1_ASM18149v1_genomic.fna_185:152078-153191 Satlength=1114 Nr of Repeats=21 RepeatLength=53 seed=GAGTAAACGA Num.seqs=21 Similarity=0.835626
47 CGACCCAACATCTCTC-CAAA-ATTTCAATCTCATTGGGTCGTTTACTCCCT-TTT
Rev.of_GCF_000181495.1_ASM18149v1_genomic.fna_186:315613-316090 Satlength=478 Nr of Repeats=9 RepeatLength=53 seed=CAATGAGATT Num.seqs=7 Similarity=0.858613 88
CACACCAACATC-CTCACAAAGATT-CAATCTCATTGGGCTGATTAGTCCGT-TTT
GCF_000181495.1_ASM18149v1_genomic.fna_185:82017-82493 Satlength=477 Nr of Repeats=9 RepeatLength=53 seed=TTTCACACCA Num.seqs=8 Similarity=0.806878
104 CACACCAACTTC-CTCACAAA-ATTCCATTCTCATCGGGGTGATTAGTCCGTCTTT

* ***** * * * * **** ** *** * **
Consensus:

CanaCCAACAtCcCTcncAtAATcCatTCTCaTTGGnctGaTTActCCcTTTT

>Bacillus_Fam_43_53_9 Nr. of seq. 9 Alignment length(with gaps) = 56
Alignment score = 0.632275
GCF_000813125.1_ASM81312v1_genomic.fna_101:16844-17056 Satlength=213 Nr of Repeats=4 RepeatLength=53 seed=GGGCTGCATA Num.seqs=4

Similarity=0.903564
0 GGGCTGCATAAGGGTATCATTTGCCCCT-AAAGGTACCCTTATG-AGG-AGTGAAT
Rev.of_GCF_000934845.1_ASM93484v1_genomic.fna_85:6860-7521 Satlength=662 Nr of Repeats=4 RepeatLength=53 seed=CATAAGGGCA Num.seqs=3
Similarity=0.746914 43
CGCCTGCATAAGGGTATC-TTTCATCCGGAATGGTGCCCTTATG-CGGAAG-GATT
GCF_001315165.1_ASM131516v1_genomic.fna_11:72146-72410 Satlength=265 Nr of Repeats=4 RepeatLength=53 seed=AACGGTACCC Num.seqs=3
Similarity=0.849057
81 GGCTTGCATAAGGGTATC-TTTCTGCCTGAACGGTACCCTTATG-AGGAAG-GGAT
GCF_000934845.1_ASM93484v1_genomic.fna_170:22317-22583 Satlength=267 Nr of Repeats=5 RepeatLength=53 seed=TACCCTTATA Num.seqs=4
Similarity=0.807128
86 GGCCTGCATAAGGGCATC-TTTCCTCCTGAATAGTACCCTTATAGCGG-AG-CAAA
Rev.of_GCF_000934845.1_ASM93484v1_genomic.fna_6:12222-12434 Satlength=213 Nr of Repeats=4 RepeatLength=53 seed=GCGGAATGAT Num.seqs=4
Similarity=0.849057 25
CGCCCGCATAAGGGTATC-ATTCCGCAAGAAAGATGCCCTTATCGAGG-AG-GAAA
Rev.of_GCF_000934845.1_ASM93484v1_genomic.fna_8:72848-73060 Satlength=213 Nr of Repeats=4 RepeatLength=53 seed=GAAAGATACC Num.seqs=4
Similarity=0.865828 75
TGCTCGCATAAGGGTATC-TTTCTGCTTGAGAGATGCCCTTATAGAGG-TG-AAAA
Rev.of_GCF_000934845.1_ASM93484v1_genomic.fna_10:8591-8803 Satlength=213 Nr of Repeats=4 RepeatLength=53 seed=GAAAGATACC Num.seqs=4
Similarity=0.867284 75
TGCTTGCATAAGGGTATC-TTTCTGCTTGAGAGATGCCCTTATGGAGG-TG-GAAA
Rev.of_GCF_000934845.1_ASM93484v1_genomic.fna_31:34768-34980 Satlength=213 Nr of Repeats=4 RepeatLength=53 seed=GATACCCTTA Num.seqs=4
Similarity=0.899371 124
TGCTTGCATAAGGGTATC-TTTCCGCATGAGAGATGCTCTTATAGAGG-TG-AAAT
GCF_000934845.1_ASM93484v1_genomic.fna_145:149-362 Satlength=214 Nr of Repeats=4 RepeatLength=53 seed=ATACCCTTAT Num.seqs=3 Similarity=0.932914
85 TGCTCGCAAAAGGGTATC-ATTCCGCTCGAAAGATACCCTTATAGAGG-TG-GAAG

*   *** ***** *** **   *   *   * * *****   ** *
Consensus:

nGCttGCATAAGGGTATCtTTCcgCntGAaaGaTgCCCTTATngaGGaGgAAn

>Bacillus_Fam_44_51_9 Nr. of seq. 9 Alignment length(with gaps) = 54
Alignment score = 0.728567
GCF_000331575.1_ASM33157v1_genomic.fna_1:158394-159108 Satlength=715 Nr of Repeats=14 RepeatLength=51 seed=GTTTTGCTGA Num.seqs=14
Similarity=0.979315 0
GTTTTGCTGATAGTAAA-CCTTATTTATCGTCAGCAAATTGAGAA-T-ACGGCC
GCF_000331575.1_ASM33157v1_genomic.fna_1:158415-160735 Satlength=2321 Nr of Repeats=32 RepeatLength=51 seed=ATTTATCGTC Num.seqs=31
Similarity=0.819137 21
GTTTTGCTGATAGTAAA-CCTTATTTATCGTCAGCAAATTGAGAA-T-ACGGCC
GCF_000331575.1_ASM33157v1_genomic.fna_1:1646807-1647470 Satlength=664 Nr of Repeats=11 RepeatLength=51 seed=CTGATAGTAA Num.seqs=9
Similarity=0.885258 6
GGTTTGCTGATAGTAAA-CTTTATTTATCGTCAGCAAATCGNGAA-T-TCGGCT

GCF_000331575.1_ASM33157v1_genomic.fna_1:244438-244999 Satlength=562 Nr of Repeats=11 RepeatLength=51 seed=TTTTGCTGAT Num.seqs=11 Similarity=0.932501 1
GTTTTGCTGATAGTAAATCCTT-TTTATCGTCAGCAAATTGAGGA-TCGCGAC-
Rev.of_GCF_000331575.1_ASM33157v1_genomic.fna_2:526077-526485 Satlength=409 Nr of Repeats=8 RepeatLength=51 seed=ATCAGCAAAA Num.seqs=8 Similarity=0.831158 11
GTTTTGCTGATAGTAAATCCGT-TTTATCGTCAGCAAATTGAGGA-TCGCTCC-
GCF_000331575.1_ASM33157v1_genomic.fna_1:651421-651726 Satlength=306 Nr of Repeats=6 RepeatLength=51 seed=GTTTTGCTGA Num.seqs=5 Similarity=0.853595
51   GTTTTGCTGATCGTAAATCCTT-TTTATCGTCAGCAAATCCAGTC-TCACGCC-
Rev.of_GCF_000331575.1_ASM33157v1_genomic.fna_2:25217-25727 Satlength=511 Nr of Repeats=10 RepeatLength=51 seed=AAAAGGATTT Num.seqs=10 Similarity=0.957008 75
GTTTTGCTGATAGTAAATCCTT-TTTATCGTCAGCAAATCCAGTC-TCACGCC-
Rev.of_GCF_000331575.1_ASM33157v1_genomic.fna_2:599172-599835 Satlength=664 Nr of Repeats=13 RepeatLength=51 seed=TATCAGCAAA Num.seqs=13 Similarity=0.884699
12   ATTTTGCTGATAATAAATCCAT-TTTATCGTCAGCAAATCGAGCA-TCACACC-
Rev.of_GCF_000331575.1_ASM33157v1_genomic.fna_2:632825-633284 Satlength=460 Nr of Repeats=9 RepeatLength=51 seed=ATTTGGTGAC Num.seqs=9 Similarity=0.936093 38
GTTTTGCTGATAGTAAATCCGT-TTTATCGTCACCAAATCGAAAATTCA-ATC-

```
*********  **** *  * ********** *****       *     *
```

Consensus:

GTTTTGCTGATAGTAAAtCCtTTTATCGTCAGCAAATcgAGnaTcaCgnC

>Bacillus_Fam_45_52_9 Nr. of seq. 9 Alignment length(with gaps) = 53 Alignment score = 0.615129
GCF_000169195.2_ASM16919v2_genomic.fna_1:320228-320592 Satlength=365 Nr of Repeats=7 RepeatLength=52 seed=GATGAAGGAC Num.seqs=5 Similarity=0.739623
0   GATGAAGGACAATTNGGCTTTNCCAGNCCNGTGTTTTGTCCTTCATCACCTC-
Rev.of_GCF_000217835.1_ASM21783v1_genomic.fna_1:922243-922973 Satlength=731 Nr of Repeats=14 RepeatLength=52 seed=TGTCCTTCAT Num.seqs=10 Similarity=0.774144
11   GATGAAGGACAATTTGGCTTTGCCAGGCCTGTGTTTTGTCCTTCATCACC-CN
Rev.of_GCF_000169195.2_ASM16919v2_genomic.fna_1:2537506-2537922 Satlength=417 Nr of Repeats=8 RepeatLength=52 seed=GTCCTTCATA Num.seqs=8 Similarity=0.715939 10
TATGAAGGACAGTTCGTCGTTTCCTGGCCGGTGTTTTGTCTTTCATCGGC-CC
GCF_000169195.2_ASM16919v2_genomic.fna_1:2655058-2655318 Satlength=261 Nr of Repeats=5 RepeatLength=52 seed=ATGAAGGACA Num.seqs=5 Similarity=0.637107 53
TATGAAGGACATTTCGCCGTTTGCCGGCACNTGTTTTGTCCTTCATCATN-CT
GCF_001591485.1_ASM159148v1_genomic.fna_63:10648-10908 Satlength=261 Nr of Repeats=5 RepeatLength=52 seed=TTTCCTTTTT Num.seqs=5 Similarity=0.820513
12   AATGAAGGACAATTTCCTTTTTTCAANCCCCGGATTTGTCCTTCATCGCC-CC
GCF_000169195.2_ASM16919v2_genomic.fna_1:990416-990781 Satlength=366 Nr of Repeats=6 RepeatLength=52 seed=ATGAAAGACA Num.seqs=4

Similarity=0.790598
1   GATGAAAGACATTTCGCTGCCCGGACACCTCTCTTTTGTCCTTCATCACC-CC
Rev.of_GCF_000217835.1_ASM21783v1_genomic.fna_1:327095-327408
Satlength=314 Nr of Repeats=5 RepeatLength=52 seed=TGTCTTTCAT Num.seqs=3
Similarity=0.760684   11
GATGAAAGACATTTCGCTGCNTGGGCACCTCNCTTTTGTCCTTCATCACC-CC
Rev.of_GCF_000832905.1_ASM83290v1_genomic.fna_1:2689111-2689632
Satlength=522 Nr of Repeats=10 RepeatLength=52 seed=AATGTCCTTC
Num.seqs=9 Similarity=0.772792
13   GATGAAGGACATTTCGCCCCNCGGACGCCCCTCTTTTGTCCTTCATCACC-CC
GCF_000169195.2_ASM16919v2_genomic.fna_1:2031833-2032093 Satlength=261 Nr of Repeats=5 RepeatLength=52 seed=TGTCCTTCAT Num.seqs=5
Similarity=0.874359   36
GATGAAGGACATTTCGTTGCAAGGGAGCCGGTGATTTGTCCTTCATAACC-GT

***** **** **   *   ****** *****
Consensus:

gATGAAgGACAtTTcGccgttngcangCCnntgtTTTGTCCTTCATCacCCc

>Bacillus_Fam_46_36_9 Nr. of seq. 9 Alignment length(with gaps) = 36
Alignment score = 0.777263
GCF_000715205.1_SOAPdenovo_v1.05_genomic.fna_90:66-390 Satlength=325 Nr of Repeats=8 RepeatLength=36 seed=ATCTCCGGTA Num.seqs=6
Similarity=0.822222
0   ATCTCCGGTAACACCTGTAGCGCCAGTAGAACCAGT
GCF_001038845.1_ASM103884v1_genomic.fna_31:5-185 Satlength=181 Nr of Repeats=4 RepeatLength=36 seed=CCAGTTACAC Num.seqs=3 Similarity=0.834835
4   ATCACCAGTTACACCAGTGGCNCCGGTAGGACCGGT
GCF_001457015.1_ASM145701v1_genomic.fna_2:17-395 Satlength=379 Nr of Repeats=10 RepeatLength=36 seed=GTATCTCCAG Num.seqs=8
Similarity=0.850529
25   TACACCAGTGGCACCAGTAGGNCCAGTATCTCCAGT
GCF_001896335.1_ASM189633v1_genomic.fna_5:59-1301 Satlength=1243 Nr of Repeats=31 RepeatLength=36 seed=GTATCTCCAG Num.seqs=19
Similarity=0.797488
25   TACACCAGTGGCACCGGTAGGTCCNGTATCTCCAGT
GCF_001896335.1_ASM189633v1_genomic.fna_49:56-272 Satlength=217 Nr of Repeats=6 RepeatLength=36 seed=GTATCTCCAG Num.seqs=6 Similarity=0.834568
25   TACACCAGTGGCACCGGTAGGTCCAGTATCTCCAGT
GCF_001896335.1_ASM189633v1_genomic.fna_54:44-188 Satlength=145 Nr of Repeats=4 RepeatLength=36 seed=GTATCTCCAG Num.seqs=4 Similarity=0.839506
25   TACACCAGTGGCACCGGTAGGTCCAGTATCTCCAGT
Rev.of_GCF_001896335.1_ASM189633v1_genomic.fna_52:35-170 Satlength=136 Nr of Repeats=4 RepeatLength=36 seed=ACTGGAGATA Num.seqs=3
Similarity=0.876543   36
TACACCAGTGGCACCGGTAGGTCCGGTATCTCCAGT
GCF_001457015.1_ASM145701v1_genomic.fna_5:226551-227512 Satlength=962 Nr of Repeats=13 RepeatLength=36 seed=GTATCTCCAG Num.seqs=8
Similarity=0.828042   25
TACACCAGTNGCACCGGTAGGTCCAGTATCACCAGT
Rev.of_GCF_001896335.1_ASM189633v1_genomic.fna_8:50-437 Satlength=388 Nr of Repeats=10 RepeatLength=36 seed=CCTACCGGTG Num.seqs=6

Similarity=0.832099 57
AACACCAGTNGCACCGGTAGGTCCAGTATCTCCAGT

* ** ** **** ** * ** ***   ** **
Consensus:

taCACCAGTggCACCgGTAGgtCCaGTAtctCCAGT

>Bacillus_Fam_47_21_9 Nr. of seq. 9 Alignment length(with gaps) = 24
Alignment score = 0.626157
GCF_001578185.1_ASM157818v1_genomic.fna_1:1878234-1879214 Satlength=981
Nr of Repeats=38 RepeatLength=21 seed=CACGAGTAAA Num.seqs=27
Similarity=0.615271 0
-CACGAGTAAA--TAGC-CAAAAT
Rev.of_GCF_001273755.1_ASM127375v1_genomic.fna_17:4099551-4099980
Satlength=430 Nr of Repeats=21 RepeatLength=21 seed=TTTACTCGTG
Num.seqs=13 Similarity=0.817405
10   -CACGAGTAAA-GCAGC-CAAACT
Rev.of_GCF_001578185.1_ASM157818v1_genomic.fna_1:3721377-3721608
Satlength=232 Nr of Repeats=11 RepeatLength=21 seed=TTACTCGTGA
Num.seqs=11 Similarity=0.805785
30   -CACGAGTAAA-GCGCC-CAAACT
GCF_001273755.1_ASM127375v1_genomic.fna_17:4304916-4305337 Satlength=422
Nr of Repeats=20 RepeatLength=21 seed=TCACGAGTAA Num.seqs=15
Similarity=0.793507 0
TCACGAGTAAA--TTGCTCAAAC-
GCF_001578185.1_ASM157818v1_genomic.fna_1:4390176-4390628 Satlength=453
Nr of Repeats=15 RepeatLength=21 seed=TCACGAGTAA Num.seqs=12
Similarity=0.732553 0
TCACGAGTAAA--CTGCTNAAAC-
GCF_001578185.1_ASM157818v1_genomic.fna_1:4868615-4868909 Satlength=295
Nr of Repeats=14 RepeatLength=21 seed=CGAGTAAACA Num.seqs=14
Similarity=0.891156 3
TCACGAGTAAA--CAGCTCAAAC-
GCF_001578185.1_ASM157818v1_genomic.fna_1:1732763-1732929 Satlength=167
Nr of Repeats=7 RepeatLength=21 seed=CACGAGTAAA Num.seqs=5
Similarity=0.746032 1
TCACGAGTAAA--GTGCNCAAAT-
Rev.of_GCF_001578185.1_ASM157818v1_genomic.fna_1:4121151-4122285
Satlength=1135 Nr of Repeats=50 RepeatLength=21 seed=TTTACTCGTG
Num.seqs=41 Similarity=0.780101
11   TCACGAGTAAA--AGGCTCAAAT-
Rev.of_GCF_001578185.1_ASM157818v1_genomic.fna_1:4151346-4151430
Satlength=85 Nr of Repeats=4 RepeatLength=21 seed=TTTACTCGTG Num.seqs=4
Similarity=0.681159 11
TCACGAGTAAACTAGGCTGAAAT-

**********   * ***
Consensus:

tCACGAGTAAAnnGCtcAAAc

>Bacillus_Fam_48_21_9 Nr. of seq. 9 Alignment length(with gaps) = 23
Alignment score = 0.644525

GCF_000508325.1_BAVI_1_genomic.fna_68:3560-3643 Satlength=84 Nr of Repeats=4 RepeatLength=21 seed=ATTCGCTAAT Num.seqs=3 Similarity=0.537778
0 ATTCGCTAAT-AAACCCTCN-A-
Rev.of_GCF_000508325.1_BAVI_1_genomic.fna_103:18741-18824 Satlength=84 Nr of Repeats=4 RepeatLength=21 seed=TTATTAGCGA Num.seqs=3 Similarity=0.640212 12
ATTCGCTAAT-AAACNNTCN-AG
Rev.of_GCF_001591805.1_ASM159180v1_genomic.fna_12:67671-67793 Satlength=123 Nr of Repeats=5 RepeatLength=21 seed=TATTAGCGAA Num.seqs=3 Similarity=0.597884 32
ATTCGCTAAT-AAACCNNCN-AG
GCF_000508325.1_BAVI_1_genomic.fna_94:20272-20356 Satlength=85 Nr of Repeats=4 RepeatLength=21 seed=ATTCGCTGAT Num.seqs=4 Similarity=0.557971
0 ATTCGCTGATAAAACCCTCG-A-
GCF_000508325.1_BAVI_1_genomic.fna_98:31365-31448 Satlength=84 Nr of Repeats=4 RepeatLength=21 seed=ATTCGCTAAT Num.seqs=3 Similarity=0.671498
0 ATTCGCTAAT-AAACTNGCA-A-
Rev.of_GCF_000508325.1_BAVI_1_genomic.fna_119:10301-10384 Satlength=84 Nr of Repeats=4 RepeatLength=21 seed=TTTATTAGCG Num.seqs=3 Similarity=0.661616 13
ATTCGCTAAT-AAACATGGA-AG
Rev.of_GCF_001591805.1_ASM159180v1_genomic.fna_1:35221-35304 Satlength=84 Nr of Repeats=4 RepeatLength=21 seed=TTTATTAGCG Num.seqs=3 Similarity=0.661616 13
ATTCGCTAAT-AAACTTGGA-AG
Rev.of_GCF_001591805.1_ASM159180v1_genomic.fna_12:66529-66612 Satlength=84 Nr of Repeats=4 RepeatLength=21 seed=TTTATTAGCG Num.seqs=3 Similarity=0.626263 13
ATTCGCTAAT-AAATNTGGA-AG
GCF_001591665.1_ASM159166v1_genomic.fna_53:46428-46511 Satlength=84 Nr of Repeats=4 RepeatLength=21 seed=ATTCGCTAAT Num.seqs=3 Similarity=0.545455
0 ATTCGCTAAT-AAACTNNNAGA-

******* ** *** *
Consensus:

ATTCGCTAATAAACnnncaAg

>Bacillus_Fam_49_21_9 Nr. of seq. 9 Alignment length(with gaps) = 22 Alignment score = 0.671296
GCF_001273755.1_ASM127375v1_genomic.fna_17:3993724-3993858 Satlength=135 Nr of Repeats=5 RepeatLength=21 seed=TTACTCGTGA Num.seqs=4 Similarity=0.746032 12
AATTC-CGAGCATTTACTCGTG
Rev.of_GCF_001578185.1_ASM157818v1_genomic.fna_1:724068-724640 Satlength=573 Nr of Repeats=21 RepeatLength=21 seed=AATTCACGAG Num.seqs=13 Similarity=0.733683
25 AATTT-NGAGCAAATACTCGTG
Rev.of_GCF_001578185.1_ASM157818v1_genomic.fna_1:4101854-4102160 Satlength=307 Nr of Repeats=15 RepeatLength=21 seed=TCACGAGTAT Num.seqs=10 Similarity=0.964727
43 AGTTT-GGAGCAAATACTCGTG
GCF_001578185.1_ASM157818v1_genomic.fna_1:2283060-2283368 Satlength=309 Nr of Repeats=9 RepeatLength=21 seed=TACTCGTGAA Num.seqs=7

Similarity=0.696248 13
ANATT-NGTGCATTTACTCGTG
GCF_001578185.1_ASM157818v1_genomic.fna_1:4201354-4201438 Satlength=85 Nr of Repeats=4 RepeatLength=21 seed=ATTTACTCGT Num.seqs=4
Similarity=0.737374 32
AATTTNAGTGCATTTACTCGTG
GCF_001578185.1_ASM157818v1_genomic.fna_1:4254210-4254294 Satlength=85 Nr of Repeats=4 RepeatLength=21 seed=TTTACTCGTG Num.seqs=4
Similarity=0.737374 33
AATTTTGAAGCATTTACTCGTG
Rev.of_GCF_001578185.1_ASM157818v1_genomic.fna_1:4716658-4716888 Satlength=231 Nr of Repeats=11 RepeatLength=21 seed=CTCACGAGTA Num.seqs=8 Similarity=0.770975
23 AGTTT-GGTTCGTTTACTCGTG
GCF_001578185.1_ASM157818v1_genomic.fna_1:5100829-5100974 Satlength=146 Nr of Repeats=7 RepeatLength=21 seed=TTTACTCGTG Num.seqs=6
Similarity=0.655556 32
AATTT-NGTGCGTTTACTCGTG
GCF_001273755.1_ASM127375v1_genomic.fna_17:4575317-4575422 Satlength=106 Nr of Repeats=5 RepeatLength=21 seed=TTTACTCGTG Num.seqs=5
Similarity=0.726984 32
AATTA-GATGGATTTACTCGTG

********* *
Consensus:

tTACTCGTGAaTTtngtGCat

>Bacillus_Fam_50_18_9 Nr. of seq. 9 Alignment length(with gaps) = 21
Alignment score = 0.615520
GCF_000007825.1_ASM782v1_genomic.fna_1:1257786-1258002 Satlength=217 Nr of Repeats=11 RepeatLength=18 seed=CAAAAGAAAG Num.seqs=10
Similarity=0.878189 0
CAAAAGAAAG--CAGAAGAA-
GCF_000496285.1_ASM49628v1_genomic.fna_1:3845536-3845770 Satlength=235 Nr of Repeats=13 RepeatLength=18 seed=AGAAGAACAG Num.seqs=13
Similarity=0.914530 11
CAGAAGAAAG--TAGAAGAA-
GCF_000831065.1_ASM83106v1_genomic.fna_1:1083186-1083312 Satlength=127 Nr of Repeats=7 RepeatLength=18 seed=GAAGAACAGA Num.seqs=7
Similarity=0.915344 12
CAGAAGAAAG--TAGAAGAA-
Rev.of_GCF_001645555.1_ASM164555v1_genomic.fna_49:95444-95588 Satlength=145 Nr of Repeats=8 RepeatLength=18 seed=TCTTCTACTT Num.seqs=6
Similarity=0.950617 17
CAGAAGAAAG--TAGAAGAA-
GCF_000007825.1_ASM782v1_genomic.fna_1:2224259-2224349 Satlength=91 Nr of Repeats=4 RepeatLength=18 seed=CAAGAAGAAC Num.seqs=3 Similarity=0.728395
9 CTNAAGAAAC--AAGAAGAA-
Rev.of_GCF_000775975.1_ASM77597v1_genomic.fna_1:2528886-2528958 Satlength=73 Nr of Repeats=4 RepeatLength=18 seed=TCTTGTTTCT Num.seqs=4
Similarity=0.814815 14
CAAAAGAAAC--AAGAAGAG-

Rev.of_GCF_001645555.1_ASM164555v1_genomic.fna_12:477954-478062
Satlength=109 Nr of Repeats=5 RepeatLength=18 seed=TTGTTTCTTT Num.seqs=4
Similarity=0.864198 30
GAAAAGAAAC--AAGAAGAG-
GCF_000008505.1_ASM850v1_genomic.fna_1:1260925-1260997 Satlength=73 Nr of
Repeats=4 RepeatLength=18 seed=CAAGCGGAAG Num.seqs=4 Similarity=0.901235
6 CAGAAACAAG--CGGAAGAA-
Rev.of_GCF_001591665.1_ASM159166v1_genomic.fna_10:16518-16623
Satlength=106 Nr of Repeats=5 RepeatLength=21 seed=TCTTTCTTAT Num.seqs=3
Similarity=0.746032 11
NATAAGAAAGACAAGAAGAAN

** ** *****
Consensus:

cAnAAGAAAgnAGAAGAa

>Bacillus_Fam_51_21_9 Nr. of seq. 9 Alignment length(with gaps) = 21
Alignment score = 0.772046
GCF_001273755.1_ASM127375v1_genomic.fna_17:3615490-3615885 Satlength=396
Nr of Repeats=19 RepeatLength=21 seed=ACTCGTGAGT Num.seqs=15
Similarity=0.766667 27
ACNNTTACTCGTGAGTTTCAG
GCF_001273755.1_ASM127375v1_genomic.fna_17:3619849-3620172 Satlength=324
Nr of Repeats=15 RepeatLength=21 seed=TTTACTCGTG Num.seqs=11
Similarity=0.831457 45
CCTTTTACTCGTGAGTTTCAN
GCF_001273755.1_ASM127375v1_genomic.fna_17:4533871-4534039 Satlength=169
Nr of Repeats=8 RepeatLength=21 seed=TTTACTCGTG Num.seqs=8
Similarity=0.759637 45
TCATTTACTCGTGAGTTTCAC
GCF_001578185.1_ASM157818v1_genomic.fna_1:4693833-4694085 Satlength=253
Nr of Repeats=12 RepeatLength=21 seed=TTTACTCGTG Num.seqs=12
Similarity=0.904762 45
CCATTTACTCGTGAGTTTCGG
Rev.of_GCF_001273755.1_ASM127375v1_genomic.fna_17:4054385-4054658
Satlength=274 Nr of Repeats=13 RepeatLength=21 seed=TCACGAGTAA
Num.seqs=13 Similarity=0.759870
35 ACTTTTACTCGTGAGTTTGGC
GCF_001578185.1_ASM157818v1_genomic.fna_1:4048935-4049535 Satlength=601
Nr of Repeats=25 RepeatLength=21 seed=TTTACTCGTG Num.seqs=20
Similarity=0.749482 45
ACGTTTACTCGTGAGTTTCGC
GCF_001578185.1_ASM157818v1_genomic.fna_1:4644456-4644561 Satlength=106
Nr of Repeats=5 RepeatLength=21 seed=TTTACTCGTG Num.seqs=5
Similarity=0.873016 45
GCTTTTACTCGTGAGTTTTGC
GCF_001578185.1_ASM157818v1_genomic.fna_1:5224710-5224918 Satlength=209
Nr of Repeats=9 RepeatLength=21 seed=TTTACTCGTG Num.seqs=8
Similarity=0.709627 45
GCTTTTACTCGTGAATTTCGC
GCF_001578185.1_ASM157818v1_genomic.fna_1:3591601-3592013 Satlength=413
Nr of Repeats=12 RepeatLength=21 seed=TTTACTCGTG Num.seqs=10

Similarity=0.673302 45
ACNTTTACTCGTGAATTTTGC

******** *** * **
Consensus:

ACTCGTGAgTTTcgcnCnTTT

>Bacillus_Fam_52_18_9 Nr. of seq. 9 Alignment length(with gaps) = 18
Alignment score = 0.911008
GCF_000007825.1_ASM782v1_genomic.fna_1:797166-797535 Satlength=370 Nr of Repeats=18 RepeatLength=18 seed=GAGCTTTAGC Num.seqs=16
Similarity=0.733951
0 GAGCTTTAGCTGCTTCTT
GCF_000007845.1_ASM784v1_genomic.fna_1:811902-812082 Satlength=181 Nr of Repeats=10 RepeatLength=18 seed=GAGCTTTAGC Num.seqs=10
Similarity=0.759671
0 GAGCTTTAGCTGCTTCTT
GCF_000008505.1_ASM850v1_genomic.fna_1:810171-810495 Satlength=325 Nr of Repeats=18 RepeatLength=18 seed=GAGCTTTAGC Num.seqs=16
Similarity=0.764198
0 GAGCTTTAGCTGCTTCTT
GCF_000290995.1_Baci_cere_AND1407_G13175_V1_genomic.fna_9:66816-67098
Satlength=283 Nr of Repeats=15 RepeatLength=18 seed=GAGCTTTAGC
Num.seqs=14 Similarity=0.766382 0
GAGCTTTAGCTGCTTCTT
Rev.of_GCF_000775975.1_ASM77597v1_genomic.fna_1:3984486-3984747
Satlength=262 Nr of Repeats=14 RepeatLength=18 seed=GCTAAAGCTC
Num.seqs=13 Similarity=0.771130
10 GAGCTTTAGCTGCTTCTT
GCF_001645555.1_ASM164555v1_genomic.fna_48:62690-62960 Satlength=271 Nr of Repeats=13 RepeatLength=18 seed=GAGCTTTAGC Num.seqs=8
Similarity=0.788360
0 GAGCTTTAGCTGCTTCTT
GCF_000496285.1_ASM49628v1_genomic.fna_1:3419837-3420107 Satlength=271 Nr of Repeats=13 RepeatLength=18 seed=GAGCTTTAGC Num.seqs=11
Similarity=0.714478 0
GAGCTTTAGCTGCTTCNT
Rev.of_GCF_000832605.1_ASM83260v1_genomic.fna_1:4931692-4932007
Satlength=316 Nr of Repeats=17 RepeatLength=18 seed=GCTAAAGCTC
Num.seqs=16 Similarity=0.683626
10 GAGCTTTAGCTGCTTCNC
GCF_000299035.1_ASM29903v1_genomic.fna_2:1062701-1063439 Satlength=739 Nr of Repeats=41 RepeatLength=18 seed=CTTCTTGCGC Num.seqs=41
Similarity=0.916474 12
GCGCTTTCGCTACTTCTT

* ***** *** ****
Consensus:

GAGCTTTAGCTGCTTCtT

>Bacillus_Fam_53_18_9 Nr. of seq. 9 Alignment length(with gaps) = 18
Alignment score = 0.913580

GCF_000007825.1_ASM782v1_genomic.fna_1:4490484-4491069 Satlength=586 Nr of Repeats=9 RepeatLength=18 seed=CCTTGGTTGT Num.seqs=6 Similarity=0.930864
0 CCTTGGTTGTTGTTTCCA
Rev.of_GCF_000290995.1_Baci_cere_AND1407_G13175_V1_genomic.fna_2:317567-318278 Satlength=712 Nr of Repeats=13 RepeatLength=18 seed=ACAACCAAGG Num.seqs=11 Similarity=0.849158 10
CCTTGGTTGTCGTTTCCA
Rev.of_GCF_001645555.1_ASM164555v1_genomic.fna_1:82827-83484 Satlength=658 Nr of Repeats=14 RepeatLength=18 seed=ACAACCAAGG Num.seqs=13 Similarity=0.827160
10 CCTTGGTTGTCGTTTCCA
GCF_000712615.1_ASM71261v1_genomic.fna_1:773424-773550 Satlength=127 Nr of Repeats=7 RepeatLength=18 seed=TTGTCTCCAC Num.seqs=7 Similarity=0.844797
9 CCTTGGTTGTTGTCTCCA
GCF_000008505.1_ASM850v1_genomic.fna_1:4343825-4344464 Satlength=640 Nr of Repeats=9 RepeatLength=18 seed=CCTTGGTTGT Num.seqs=7 Similarity=0.823633
0 CCTTGGTTGTCGTTTCCG
GCF_000496285.1_ASM49628v1_genomic.fna_1:1747374-1748121 Satlength=748 Nr of Repeats=18 RepeatLength=18 seed=TTTCCGCCTT Num.seqs=17 Similarity=0.909586 12
CCTTGGTTGTCGTTTCCG
Rev.of_GCF_000775975.1_ASM77597v1_genomic.fna_1:319755-320394 Satlength=640 Nr of Repeats=13 RepeatLength=18 seed=ACAACCAAGG Num.seqs=12 Similarity=0.849607
10 CCTTGGTTGTCGTTTCCG
GCF_000831065.1_ASM83106v1_genomic.fna_1:4247332-4247404 Satlength=73 Nr of Repeats=4 RepeatLength=18 seed=CCTTGGTTGT Num.seqs=4 Similarity=0.962963
0 CCTTGGTTGTCGTTTCCG
Rev.of_GCF_000832605.1_ASM83260v1_genomic.fna_1:1427286-1427889 Satlength=604 Nr of Repeats=11 RepeatLength=18 seed=ACAACCAAGG Num.seqs=10 Similarity=0.889712
10 CCTTGGTTGTCGTTTCCG

********** ** ***
Consensus:

CCTTGGTTGTcGTTTCCg

>Bacillus_Fam_54_114_8 Nr. of seq. 8 Alignment length(with gaps) = 115 Alignment score = 0.790890
GCF_000242895.2_ASM24289v3_genomic.fna_1:2821534-2822245 Satlength=712 Nr of Repeats=5 RepeatLength=114 seed=CCGCTTACAT Num.seqs=4 Similarity=0.752437 0
CCGCTTACATCGCATTGGCCATACTTATTATAACCCACAGCCGCCACCGTGCCGTCAGATTTAAGGCCGACGG
TATGANTGC-AACCCGCCGCTACCGCCACAATACCACGCCAG
GCF_001278705.1_ASM127870v1_genomic.fna_1:2862067-2862637 Satlength=571 Nr of Repeats=4 RepeatLength=114 seed=CGCGCCAGTC Num.seqs=3 Similarity=0.641762 106
TCGCTTACATCGCATTGACCATACTCATTNTCACCCACAGCAACCACCGTNCCATCAGATTTAAGGCCGATGG
TATGANNAC-AACCCGCCGCAACCGCCACAATACCGCGCCAG

Rev.of_GCF_000759675.1_ASM75967v1_genomic.fna_152:2346-2916 Satlength=571 Nr of Repeats=4 RepeatLength=114 seed=CTTAAATCGG Num.seqs=3 Similarity=0.610136 65
TCGTTTACATTGCATTGGCCATNCTTATTNCNACCCACAGCTGCCACCGTACCGTCCGATTTAAGCCCAATGG
TATGACANT-AACCCGCCGCAACCGCCACAATATCGCGCCAG
Rev.of_GCF_001591625.1_ASM159162v1_genomic.fna_135:4361-5072 Satlength=712 Nr of Repeats=6 RepeatLength=114 seed=GGGCTTAAAT Num.seqs=5 Similarity=0.608832
68
TCGCTTACATTGCATTGGCCATACTTATTNCNACCCACAGCCACCACCGTACCGTCCGATTTAAGNCCAATNG
TATGNAGNC-AACCCGCCGCNACCGCCACAATATCGTGCCAG
Rev.of_GCF_001591665.1_ASM159166v1_genomic.fna_6:40523-41234 Satlength=712 Nr of Repeats=6 RepeatLength=114 seed=CTTAAATCGG Num.seqs=5 Similarity=0.636182
179
TCGCTTACATTGCATTGGCCATNCTTATTCCANCCCACAGCCATCACCGTTCCGTCCGATTTAAGNCCGATGG
TATGANNNC-AACCCGCCGCNACCGCCACAATATCGCGCCAG
Rev.of_GCF_001591805.1_ASM159180v1_genomic.fna_74:3413-4124 Satlength=712 Nr of Repeats=6 RepeatLength=114 seed=GGGCTTAAAT Num.seqs=5 Similarity=0.632749 68
TCGCTTACATTGCATTGGCCATNCTTATTNNNACCCACAGCNGCCACNGTACCGTCCGATTTAAGNCCGATNG
TATGNNNAC-AACCCGCCGCAACCGCCACAATATCGNNCCAG
GCF_000307875.1_BABA1.0_genomic.fna_35:121244-121841 Satlength=598 Nr of Repeats=4 RepeatLength=114 seed=CGCCACAATA Num.seqs=3 Similarity=0.648309
96
TCACTTACATTGCATTGGCCATACTNATTCNAACCTACAGCCATCACNGTACCGTCCGATTTAAGNCCGATNG
TATGANNANTAGCCCGCCGCTATCGCCACAATATCGCGCCAG
GCF_000504145.1_SOAPdenovo_for_version2.04_of_Bacillus_sp._FJAT-14515_genome_genomic.fna_1:606481-607078 Satlength=598 Nr of Repeats=4 RepeatLength=114 seed=CGCCACAATA Num.seqs=3 Similarity=0.686957 96
TCGCTTACATTGCATTGGCGATACTNATTCNAACCCACAGCCGACACTGTACCGTCCGATTTAAGACCGATGG
TATGANNANTCNCCAGCCGCGATCGCCACAATATCGCGCCAG

```
*  ****** ****** * ** ** ***    ** *****   *** ** ** ** ******** ** *
*****        ** ***** * ********** *   ****
```

Consensus:

TCGCTTACATtGCATTGGCCATaCTtATTnnaACCCACAGCcacCACcGTaCCGTCcGATTTAAGnCCgATgG
TATGannacAaCCCGCCGCnAcCGCCACAATAtCGcGCCAG

>Bacillus_Fam_55_52_8 Nr. of seq. 8 Alignment length(with gaps) = 59 Alignment score = 0.632062
GCF_000311725.1_ASM31172v1_genomic.fna_4:214050-214934 Satlength=885 Nr of Repeats=17 RepeatLength=52 seed=TCATGAGGAC Num.seqs=13 Similarity=0.774714 0
TCATGAGGACA-AAGTC-GGGC-GAAA-ATCAGGAAAGTTTGTCCTCAT--AGAGG-GT
Rev.of_GCF_000311725.1_ASM31172v1_genomic.fna_4:573981-574346 Satlength=366 Nr of Repeats=7 RepeatLength=52 seed=ACTTTGTCCT Num.seqs=6 Similarity=0.777778 15
TCATGAGGACA-AAGTC-GGAC-GAAA-ACCAGGAAAGTTTGTCCTCAT--AGAGG-GT
Rev.of_GCF_000311725.1_ASM31172v1_genomic.fna_4:290049-290519 Satlength=471 Nr of Repeats=9 RepeatLength=52 seed=TATGAGGACA Num.seqs=7

Similarity=0.867026 46
TCATGAGGACA-AAGTC-GAAC-GAAA-AGGAAGAAAGTTTGTCCTCAT--AGAGN-GT
GCF_000311725.1_ASM31172v1_genomic.fna_11:254-826 Satlength=573 Nr of Repeats=11 RepeatLength=52 seed=GTTCATGAGG Num.seqs=11 Similarity=0.792453
50 TCATGAGGACA-AAGTC-GAAC-GAAA-TCCAAGAAAGANTGTCCTCAT--AGAGG-GT
Rev.of_GCF_000311725.1_ASM31172v1_genomic.fna_12:53034-53294 Satlength=261 Nr of Repeats=5 RepeatLength=52 seed=GACTTTGTCC Num.seqs=5 Similarity=0.763030 16
TCATGAGGACA-AAGTC-AGAC-GAAA-ATCAAGAAAGTTTGTCCTCAT--A-AGGNGT
Rev.of_GCF_000311725.1_ASM31172v1_genomic.fna_4:326412-326730 Satlength=319 Nr of Repeats=6 RepeatLength=53 seed=TGTGGACAAA Num.seqs=6 Similarity=0.906080 44 -
CATGAGGACA-AACTCTGGAA-GAAA-AGCGGTGAAGTTTGTCCACATA-AGAGG-TT
GCF_001591665.1_ASM159166v1_genomic.fna_17:30304-30511 Satlength=208 Nr of Repeats=4 RepeatLength=52 seed=GGGCTCATGA Num.seqs=3 Similarity=0.897436
48 TCATGAGGACAGAAAT--GGCGAGAAA-ACCAAGAAA-ATTGTCCTCAT-GA-AGG-GC
GCF_001636315.1_ASM163631v1_genomic.fna_1:3657440-3657908 Satlength=469 Nr of Repeats=9 RepeatLength=52 seed=ATGAGGACAA Num.seqs=9 Similarity=0.884615 54
TCATGAGGACA-AAATC-GGAG-GAAACCCCCAGGAA-TCTGTCCTCAT-GA-AAG-GC

********** ** * **** ** ***** *** * *
Consensus:

TCATGAGGACAAAgTCGgacGAAAacCaaGaAAgttTGTCCTCATAgAGGGt

>Bacillus_Fam_56_52_8 Nr. of seq. 8 Alignment length(with gaps) = 58 Alignment score = 0.632389
GCF_000621445.1_ASM62144v1_genomic.fna_1:29213-29577 Satlength=365 Nr of Repeats=7 RepeatLength=52 seed=GAGTTTTGTC Num.seqs=7 Similarity=0.892183
117 T-CTC--TTTTC-CCTCGAGTTT-TGTCCTTCATCTCTCTTATGAAGGACACTTC-TC
GCF_000621445.1_ASM62144v1_genomic.fna_4:243825-244085 Satlength=261 Nr of Repeats=5 RepeatLength=52 seed=GGACACTTCT Num.seqs=5 Similarity=0.837736
145 T-CNC--TTTTT-CTTC-TCTTTCTGTCCTTCATCGCTCTTATGAAGGACACTTC-TC
Rev.of_GCF_000621445.1_ASM62144v1_genomic.fna_17:35462-35826 Satlength=365 Nr of Repeats=7 RepeatLength=52 seed=GATGAAGGAC Num.seqs=7 Similarity=0.907204 134
A-CTC--TTTCC-TCTCCTCTTT-TGTCCTTCATCAAGCTTATGAAGGACACTTC-TC
Rev.of_GCF_000621445.1_ASM62144v1_genomic.fna_2:192691-193107 Satlength=417 Nr of Repeats=8 RepeatLength=52 seed=TGTCCTTCAT Num.seqs=8 Similarity=0.905678 150
TGCT---TTTTC-AC-CTAGTTT-TGTCCTTCATCACCCTTATGAAGGACACTTCTTT
GCF_000621445.1_ASM62144v1_genomic.fna_3:29111-29423 Satlength=313 Nr of Repeats=7 RepeatLength=52 seed=GTTTTGTCCT Num.seqs=5 Similarity=0.941026
171 T-CTA--TTTTC-AC-CTCGTTT-TGTCCTTCATCAACCTTATGAAGGACACTTCCTC
GCF_000621445.1_ASM62144v1_genomic.fna_22:34839-35099 Satlength=261 Nr of Repeats=5 RepeatLength=52 seed=TGTCCTTCAT Num.seqs=5 Similarity=0.815385
122 --CTC-ATTTTCCACTC-CATTT-TGTCCTTCATCNGCCTTATGAAGGACACTTC-TC
GCF_001591805.1_ASM159180v1_genomic.fna_52:18377-18636 Satlength=260 Nr of Repeats=5 RepeatLength=52 seed=TGTCCTTCAT Num.seqs=4

Similarity=0.841880
122   --CTCGATTTCCAACTG-AGTTT-TGTCCTTCATCAGCTTTATGAAGGACACTTC--C
GCF_000621445.1_ASM62144v1_genomic.fna_12:17218-17686 Satlength=469 Nr of Repeats=9 RepeatLength=52 seed=TGTCCTTCAT Num.seqs=9 Similarity=0.821803
122   A-CCG-TTTTTC-GCTC-ACTTT-TGTCCTTCATCACGCTTATGAAGGACACTTT-AC

*** ***********   ***************   *   ***
Consensus:

nagTTTTGTCCTTCATCaccCTTATGAAGGACACTTCtCtCtcTTTtCaCtC

>Bacillus_Fam_57_52_8 Nr. of seq. 8 Alignment length(with gaps) = 57
Alignment score = 0.671261
GCF_000751775.1_A1A_genomic.fna_13:16-692 Satlength=677 Nr of Repeats=13 RepeatLength=52 seed=GGACAAAATG Num.seqs=13 Similarity=0.818930
0   GGACAAAA--TGGAGNC-TCAATCAGGAAA--AACTGTCCAATAGAACGGTTCTCAT
Rev.of_GCF_000751775.1_A1A_genomic.fna_98:24731-25094 Satlength=364 Nr of Repeats=7 RepeatLength=52 seed=CTATTGGACA Num.seqs=6 Similarity=0.760684
40   GGACAAAA--TGGGGGG-TCAATCAGGAAA--AACTGTCCAATAGAACGGCTCTCGT
Rev.of_GCF_000751775.1_A1A_genomic.fna_98:28702-29324 Satlength=623 Nr of Repeats=12 RepeatLength=52 seed=TCTATTGGAC Num.seqs=8
Similarity=0.787062
41   GGACAAAA--TGAGGAG-TCAATCAGGAAA--AACTGTCCAATAGAACGGTTCTCGT
GCF_000751775.1_A1A_genomic.fna_94:14879-15191 Satlength=313 Nr of Repeats=6 RepeatLength=52 seed=TCCAATAGAA Num.seqs=6 Similarity=0.834188
32   GGACAAAA--TGGAGCC-TCAACCAGGTGA--AACTGTCCAATAGAACGACTCTCGT
GCF_000751775.1_A1A_genomic.fna_93:1659-2387 Satlength=729 Nr of Repeats=14 RepeatLength=52 seed=TGTCCAATAG Num.seqs=14
Similarity=0.827631
30   GGACAAAA--TGAGGAG-GCAATCTGCAAA--AAATGTCCAATAGAACGGCTCTCAT
Rev.of_GCF_000751775.1_A1A_genomic.fna_100:11954-12735 Satlength=782 Nr of Repeats=15 RepeatLength=52 seed=TTGTCCATGA Num.seqs=14
Similarity=0.800055   58
GGACAAAA---AAGGNGTCCAATCAGNAAA--AATTGTCCAATAGAACGGCTTTCAT
Rev.of_GCF_000751775.1_A1A_genomic.fna_81:7055-7471 Satlength=417 Nr of Repeats=8 RepeatLength=52 seed=TTTTGTCCAT Num.seqs=8 Similarity=0.750433
8   GGACAAAACTTGAAG---TCAACCAGCAAA--AACTGTCCAATAGACCGGCTCTAAT
GCF_000751775.1_A1A_genomic.fna_12:34323-34582 Satlength=260 Nr of Repeats=5 RepeatLength=52 seed=AACTGTCCAA Num.seqs=4 Similarity=0.528283
28   GGACAAAA--TTGAG-N-TAAAAAAAGAAAGGAACTGTCCAAGAGAACGGTTCTCAT

********   *   **   * ** ******* *** **   * * *
Consensus:

GGACAAAATgaaGngtCAAtCAGgAAAAAcTGTCCAATAGAACGGcTCTCaT

>Bacillus_Fam_58_52_8 Nr. of seq. 8 Alignment length(with gaps) = 57
Alignment score = 0.619779
GCF_000813125.1_ASM81312v1_genomic.fna_49:14856-15064 Satlength=209 Nr of Repeats=4 RepeatLength=52 seed=TTTCTTGACA Num.seqs=4 Similarity=0.884615
2   G-TTTTCTTGACAAC-TTTGCGCTCT-GA-AC-CCTGAAACCTGTCACAATAACGGC
GCF_000813125.1_ASM81312v1_genomic.fna_50:13-221 Satlength=209 Nr of Repeats=4 RepeatLength=52 seed=AAACCTGTCA Num.seqs=4 Similarity=0.777778
32   T-TTTTCTTGACAGC-TTTGGGCTCC-AC-TA-CCTGAAACCTGTCACAATAACGGC

Rev.of_GCF_000813125.1_ASM81312v1_genomic.fna_136:11-271 Satlength=261 Nr of Repeats=5 RepeatLength=52 seed=CTGTCAAGAA Num.seqs=5
Similarity=0.761538 65
T-TTTTCTTGACAGC-TTTGGGCTCC-GC-AG-CCTGAAACCTGTCACAATAACTGC
Rev.of_GCF_001315165.1_ASM131516v1_genomic.fna_42:3-211 Satlength=209 Nr of Repeats=4 RepeatLength=52 seed=GTTATTGTGA Num.seqs=4
Similarity=0.863248
49   T-TTTTCTTGACAGC-TTTGGGCTAT-GT-AC-CTTGAAAGCTGTCACAATAACTAC
Rev.of_GCF_000813125.1_ASM81312v1_genomic.fna_100:44990-45353
Satlength=364 Nr of Repeats=7 RepeatLength=52 seed=GTTATTGTGA Num.seqs=6
Similarity=0.841026 49
T-TTTTCTTGACAGC-TTTTCCGTGT-TC-GC-CCCCAAACCTGTCACAATAACGGC
Rev.of_GCF_000813125.1_ASM81312v1_genomic.fna_60:30-342 Satlength=313 Nr of Repeats=5 RepeatLength=52 seed=AAAGCTGTCA Num.seqs=4
Similarity=0.764403
69   T-CTTTCTTGACAGC-TTTTCGCTCTATC-NCACCCCAAACCTGTCACAATAACGAC
Rev.of_GCF_001315165.1_ASM131516v1_genomic.fna_89:4898-5417 Satlength=520 Nr of Repeats=9 RepeatLength=52 seed=GAAAAGCTGT Num.seqs=6
Similarity=0.771279 71
T-TTTTCTTGACAGC-TTTTC-CTCA-TCAGC-ATCCAAACCTGTCACGATTACCTC
Rev.of_GCF_000934845.1_ASM93484v1_genomic.fna_1:28106-28314 Satlength=209 Nr of Repeats=4 RepeatLength=52 seed=TTATTTTGAC Num.seqs=4
Similarity=0.881027 47
TCT-TTCTTGACAACTTTTGCCCTTT-TC-AC--CTGAAAGTTGTCAAAATAACCTC

********* * ***   *   *** ***** ** ** *
Consensus:

TTTCTTGACAgCTTTgcgCTcttcaccctgAAAcCTGTCACAATAACggCTT

>Bacillus_Fam_59_55_8 Nr. of seq. 8 Alignment length(with gaps) = 57
Alignment score = 0.625731
GCF_000708755.2_ASM70875v2_genomic.fna_6:281885-282097 Satlength=213 Nr of Repeats=4 RepeatLength=53 seed=CGCCACAAAC Num.seqs=4
Similarity=0.890985
13   CTAC-GG-GGAATGACGCCACAAAC-ATGGATGCTTTCACATG-AAAAGTGGCAGCA
GCF_000709935.2_ASM70993v2_genomic.fna_15:188408-188619 Satlength=212 Nr of Repeats=4 RepeatLength=53 seed=CGCCACAAAC Num.seqs=3
Similarity=0.899371
13   CTAC-GG-GGAATGACGCCACAAAC-ATGGATGCTTTCACATG-AAAAGTGGCGGCA
Rev.of_GCF_000708755.2_ASM70875v2_genomic.fna_22:121820-122085
Satlength=266 Nr of Repeats=5 RepeatLength=53 seed=TGGCGTCATT Num.seqs=5
Similarity=0.899371 18
CTTC-GC-GGAATGACGCCACAAAC-A-AGAACCCTTCTCCGGCAAAAGTGGCAGCA
Rev.of_GCF_000709935.2_ASM70993v2_genomic.fna_24:232406-232777
Satlength=372 Nr of Repeats=7 RepeatLength=53 seed=TGGCGTCATT Num.seqs=7
Similarity=0.901767 18
CTAC-GC-GGAATGACGCCACAAAC-N-AGAACCCTTCTCTGGCAAAAGTGGCAGCA
Rev.of_GCF_000709935.2_ASM70993v2_genomic.fna_1:491832-492044
Satlength=213 Nr of Repeats=4 RepeatLength=53 seed=ATCCTAGTTT Num.seqs=4
Similarity=0.809671 29
CTACAGC--GAATGACGCCACAAACTA-GGATCC-AACTACGATAAAAGTGGCACCA
GCF_000709935.2_ASM70993v2_genomic.fna_5:571905-572276 Satlength=372 Nr of Repeats=7 RepeatLength=53 seed=ACGAATGACG Num.seqs=7

Similarity=0.841869
58   CTACAAC--GAATGACGCCACTAACCA-TCATCC-ATCTCTGGTAAAAGTGGCGGCA
Rev.of_GCF_000709935.2_ASM70993v2_genomic.fna_4:195544-195756
Satlength=213 Nr of Repeats=4 RepeatLength=53 seed=CCACTTTTTC Num.seqs=4
Similarity=0.895178   48
CTA-AGCT-GAATGACGCCACAAATGA-GGATCC-ATCACTGGAAAAAGTGGCGTCA
GCF_000709935.2_ASM70993v2_genomic.fna_15:32457-32775 Satlength=319 Nr of
Repeats=5 RepeatLength=53 seed=CACTACCGCG Num.seqs=4 Similarity=0.756813
51   CTACCGC--GAATGACGCCACAAATGA-GCATTC-ATCAAAGGCAAAAGTGGCGCCA

****** **   * *   *   ********* ****   ******
Consensus:

CGCCACAAAcnAggAtcCnaTCacngGnAAAAGTGGCagCACTACnGcgGAATGA

>Bacillus_Fam_60_52_8 Nr. of seq. 8 Alignment length(with gaps) = 56
Alignment score = 0.673044
GCF_000708755.2_ASM70875v2_genomic.fna_1:172527-172891 Satlength=365 Nr
of Repeats=7 RepeatLength=52 seed=ATGGCACTTA Num.seqs=7
Similarity=0.787546
0   ATGGCACTTATCCGGNAGAATAGAACCCTCAAGCAGAGAAAT-CTC---AGAGTTG
GCF_000709935.2_ASM70993v2_genomic.fna_1:173354-173666 Satlength=313 Nr
of Repeats=6 RepeatLength=52 seed=GCACTTATCC Num.seqs=6
Similarity=0.810256
3   ATGGCACTTATCCGGGAGAATAGAACCCTCAAGCAGAGNAAT-CAC---AGAGTTG
Rev.of_GCF_000708755.2_ASM70875v2_genomic.fna_2:379262-379469
Satlength=208 Nr of Repeats=4 RepeatLength=52 seed=GATAAGTGCC Num.seqs=3
Similarity=0.783951   12
AGGGCACTTATCAGGAAGTCTAGAACCATCAAACTGAAGGATTCTC---AG-GTTG
GCF_000708755.2_ASM70875v2_genomic.fna_5:265879-266243 Satlength=365 Nr
of Repeats=7 RepeatLength=52 seed=ACCATCAAAA Num.seqs=7
Similarity=0.811966
24   ATGGCACTTATCGGGGAGAACAGTACCATCAAAAGGAAGAATGTTT---TG-GTTG
GCF_000709935.2_ASM70993v2_genomic.fna_13:199669-200033 Satlength=365 Nr
of Repeats=7 RepeatLength=52 seed=ACCATCAAAA Num.seqs=7
Similarity=0.781441
24   ATGGCACTTATCNGGGAGAACAGAACCATCAAAAGGAAGAATGTTC---TG-GTTG
Rev.of_GCF_000708755.2_ASM70875v2_genomic.fna_7:77605-78021 Satlength=417
Nr of Repeats=8 RepeatLength=52 seed=TTTTGATGGT Num.seqs=8
Similarity=0.850517   34
ATGGCACTTATCCGGAAGAATAGAACCATCAAAAAGAAGAATCCAC---AG-GTTG
Rev.of_GCF_000709935.2_ASM70993v2_genomic.fna_19:84981-85241
Satlength=261 Nr of Repeats=5 RepeatLength=52 seed=TTTTGATGGT Num.seqs=5
Similarity=0.838462   34
ATGGCACTTATCCGGAAGAATAGAACCATCAAAAAGAAGAATNTAC---AG-GTTG
Rev.of_GCF_000709935.2_ASM70993v2_genomic.fna_19:30893-31257
Satlength=365 Nr of Repeats=7 RepeatLength=52 seed=CCTCTGTTGA Num.seqs=5
Similarity=0.815385   38
GTGGTACTTATCAGGGAGAATAGAACCATCAA-CAGAGG-AT-CTCGTTTG-GTTG

** ******* ** **   ** *** ****   **   **   * ****
Consensus:

ATGGCACTTATCcGGgAGAAtAGAACCaTCAAaaaGAagaATnctCaGGTTG

>Bacillus_Fam_61_52_8 Nr. of seq. 8 Alignment length(with gaps) = 56
Alignment score = 0.638924
GCF_001587375.1_ASM158737v1_genomic.fna_89:58073-58489 Satlength=417 Nr of Repeats=7 RepeatLength=52 seed=CCATCTTGGT Num.seqs=6 Similarity=0.759539
0   -CCATCTTGGTCGTCAAAGCG-AGCGAATGAAGCCCAAGATCCAAAACTA-AAAC-
GCF_001587375.1_ASM158737v1_genomic.fna_126:13547-13807 Satlength=261 Nr of Repeats=5 RepeatLength=52 seed=AAGATCCAAA Num.seqs=5 Similarity=0.821384
34   -CCATCTTGGTCATCAAAGCG-AGCGAATGGAGCCCAAGATCCAAAACTT-AAAT-
GCF_001587375.1_ASM158737v1_genomic.fna_137:20846-21262 Satlength=417 Nr of Repeats=8 RepeatLength=52 seed=TCCATCTTGG Num.seqs=8 Similarity=0.821204
0   TCCATCTTGGTCGACAAAGCG-AGCGAATGAAGCCCAAGAACCAAAACTT-AAAN-
GCF_001587375.1_ASM158737v1_genomic.fna_113:48010-48219 Satlength=210 Nr of Repeats=4 RepeatLength=52 seed=AACTTGGTCG Num.seqs=3 Similarity=0.846154
2   -GGAACTTGGTCGTCAAAGCG-AGTGAATGAATCCCAAGATTGGAATCCA-AAAG-
GCF_001587375.1_ASM158737v1_genomic.fna_167:68730-69198 Satlength=469 Nr of Repeats=9 RepeatLength=52 seed=AGCCCAAGAT Num.seqs=9 Similarity=0.800570
29   -GGAACTTGGTCGTCAAAGTG-AGAGAATGCAGCCCAAGATTGGAATTCC-AAAG-
Rev.of_GCF_001587375.1_ASM158737v1_genomic.fna_171:128706-129382 Satlength=677 Nr of Repeats=13 RepeatLength=52 seed=TGACGACCAA Num.seqs=13 Similarity=0.782843
67   -GGAACTTGGTCGTCATAGCG-AGAGAATGCAGCCCAAGATTGGAATTCA-AAAG-
Rev.of_GCF_001587375.1_ASM158737v1_genomic.fna_101:16594-16854 Satlength=261 Nr of Repeats=5 RepeatLength=52 seed=CGACCAAGAT Num.seqs=5 Similarity=0.853846   12   -AAATCTTGGTCGCCAAAGAGAAG-GAATGAAGCCCAAGAT--GCATGAAGAAAGT
Rev.of_GCF_001587375.1_ASM158737v1_genomic.fna_154:39116-39324 Satlength=209 Nr of Repeats=4 RepeatLength=52 seed=CGACCAAGAT Num.seqs=4 Similarity=0.858974   12   -GAATCTTGGTCGTCAAAGCGGAG-GAATGAAGCCCAAGAT--GCATGAAGAAAGT

* ******* ** ** * ** ***** * *******   *   ***
Consensus:

gnAtCTTGGTCGtCAAAGcGAGnGAATGaAGCCCAAGATnngaAtcnaAAAg

>Bacillus_Fam_62_52_8 Nr. of seq. 8 Alignment length(with gaps) = 55
Alignment score = 0.629437
GCF_000153365.1_ASM15336v1_genomic.fna_13:187910-188326 Satlength=417 Nr of Repeats=8 RepeatLength=52 seed=GGTCTTAGTG Num.seqs=8 Similarity=0.846154
0   GGTCTTAGTGAAGCGGACTAGCACCCTCT-ATGCG-GAAAAAAGG-GAAACAGAG
Rev.of_GCF_000473245.1_ASM47324v1_genomic.fna_1:3147313-3147729 Satlength=417 Nr of Repeats=8 RepeatLength=52 seed=TTCACTAAGA Num.seqs=8 Similarity=0.832547   12   GGTCTTAGTGAAGCGGACTAGTACCCTCT-ATGCG-GAAAAAAGG-GAAACAGAG
GCF_000153365.1_ASM15336v1_genomic.fna_16:263922-264130 Satlength=209 Nr of Repeats=4 RepeatLength=52 seed=GAAACAGAGG Num.seqs=4

Similarity=0.790598
43   GGTACTAGTCATACGGACTAAAACCCTCTGAACAGAGAAAAAA---GAAACAGAG
Rev.of_GCF_000473245.1_ASM47324v1_genomic.fna_1:2545989-2546197
Satlength=209 Nr of Repeats=4 RepeatLength=52 seed=CCCTCTGTTT Num.seqs=4
Similarity=0.790598 54
GGTACTAGTCATGCGGACTAAGACCCTCTGAACGGTGAAAAAA---GAAACAGAG
GCF_000153365.1_ASM15336v1_genomic.fna_72:65371-65750 Satlength=380 Nr of Repeats=5 RepeatLength=52 seed=AAAAAAGAAA Num.seqs=3 Similarity=0.829060
88   GGTCTTAGTCAAGGCCACTAAGACCCTCTGACCAG-GAAAAAA-G-AAAACAGAT
GCF_000473245.1_ASM47324v1_genomic.fna_1:1667185-1667564 Satlength=380 Nr of Repeats=5 RepeatLength=52 seed=AAAAAAGAAA Num.seqs=3
Similarity=0.829060 88
GGTCTTAGTCAAGGCCACTAAGACCCTCTGACCAG-GAAAAAA-G-AAAACAGAT
GCF_000153365.1_ASM15336v1_genomic.fna_16:461270-461740 Satlength=471 Nr of Repeats=9 RepeatLength=52 seed=GGTCTTAGTC Num.seqs=7
Similarity=0.854701
0   GGTCTTAGTCAGTGCGACTAACACCATCT-AGCCA-CAGAAAA-GCGAATCAGAC
Rev.of_GCF_000473245.1_ASM47324v1_genomic.fna_1:2350437-2350907
Satlength=471 Nr of Repeats=9 RepeatLength=52 seed=CTGACTAAGA Num.seqs=7
Similarity=0.854701 12
GGTCTTAGTCAGTGCGACTAACACCATCT-AGCCA-CAGAAAA-GCGAATCAGAC

*** **** *   **** *** *** *   * ****   ** ****
Consensus:

GGTctTAGTcAagccgACTAanACCcTCTgAnccggAaAAAAggAAaCAGAg

>Bacillus_Fam_63_52_8 Nr. of seq. 8 Alignment length(with gaps) = 54
Alignment score = 0.661155
GCF_001278705.1_ASM127870v1_genomic.fna_1:569178-569542 Satlength=365 Nr of Repeats=7 RepeatLength=52 seed=GATGAAGGAC Num.seqs=7
Similarity=0.862027
0   GATGAAGGACATTTCTTTGGCTATC-TTGCGTCATTTTGTCCTTCAGCACCCG-
Rev.of_GCF_001278705.1_ASM127870v1_genomic.fna_1:2433779-2434247
Satlength=469 Nr of Repeats=9 RepeatLength=52 seed=ATGTCCTTCA Num.seqs=9
Similarity=0.754274 12
AATGAAGGACATTTCTCTGNCTCAC-TTGCGTCATTTTGTCCTTCATAACCCG-
Rev.of_GCF_001278705.1_ASM127870v1_genomic.fna_1:577894-578363
Satlength=470 Nr of Repeats=9 RepeatLength=52 seed=GAAGGACAAA Num.seqs=8
Similarity=0.786388 44
TATGAAGGACTTTTCTCGAGCTCTC-TTACTCTTTTTTGTCCTTCACTACCCG-
Rev.of_GCF_001278705.1_ASM127870v1_genomic.fna_1:3589135-3589394
Satlength=260 Nr of Repeats=5 RepeatLength=52 seed=TGAAGGACAA Num.seqs=4
Similarity=0.777778 45
GATGAAGGACTTTTCTCTGGCTCTT-TTACGCCTTTTTGTCCTTCATGACCCG-
Rev.of_GCF_001278705.1_ASM127870v1_genomic.fna_1:3837620-3837827
Satlength=208 Nr of Repeats=4 RepeatLength=52 seed=GTTCTGAAGG Num.seqs=3
Similarity=0.965812 49
TATGAAGGACTTTTCCTTGGATTTC-CTGCTCCTTTTTGTCCTTCAGAACCCT-
GCF_001636425.1_ASM163642v1_genomic.fna_1:3113621-3114088 Satlength=468 Nr of Repeats=9 RepeatLength=52 seed=TTGTCCTTCA Num.seqs=8
Similarity=0.810984 87
TATGAAGGACGTTTCCCTGGATTTCTCT-CACGGTTTTGTCCTTCATCACCCT-

GCF_001278705.1_ASM127870v1_genomic.fna_1:1906710-1907023 Satlength=314 Nr of Repeats=5 RepeatLength=52 seed=TGAAGGACTT Num.seqs=3 Similarity=0.982906 1
G-TGAAGGACTTTTCTCTGGATCTA-TTCCCTCTTTTCGTCCTTCAGCTCCCCT
GCF_001278705.1_ASM127870v1_genomic.fna_1:2231666-2231875 Satlength=210 Nr of Repeats=4 RepeatLength=52 seed=TTTTCTCTGG Num.seqs=3 Similarity=0.897436 9
GATGATGGACTTTTCTCTGGATCTT-TTCCCTCTTTTTGTCCTTCAGAACCCC-

```
*** **** ****       *      * *     *** ********    ***
```

Consensus:

gATGAAGGACtTTTCtcTGGaTcTctTnCncctTTTTGTCCTTCAgnACCCg

>Bacillus_Fam_64_45_8 Nr. of seq. 8 Alignment length(with gaps) = 45
Alignment score = 0.881481
GCF_000007825.1_ASM782v1_genomic.fna_1:2082917-2083097 Satlength=181 Nr of Repeats=4 RepeatLength=45 seed=TTTTTGCTCT Num.seqs=4 Similarity=0.901235
0 TTTTTGCTCTTCTTGTTGCTTACGAGCTTGCTCATCAGCTAGACG
GCF_000007845.1_ASM784v1_genomic.fna_1:2006658-2006928 Satlength=271 Nr of Repeats=6 RepeatLength=45 seed=TCTTGTTGTT Num.seqs=4 Similarity=0.812500
7 TTTTTGTTCTTCTTGTTGTTTACGAGCTTGTTCATCAGCCTGGCG
GCF_000290995.1_Baci_cere_AND1407_G13175_V1_genomic.fna_1:1160788-1161253 Satlength=466 Nr of Repeats=8 RepeatLength=45 seed=TCTTGTTGTT Num.seqs=5 Similarity=0.806944 7
TTTTTGTTCTTCTTGTTGTTTACGAGCTTGTTCATCAGCCTGGCG
GCF_000831065.1_ASM83106v1_genomic.fna_1:1900224-1900491 Satlength=268 Nr of Repeats=6 RepeatLength=45 seed=TTACGAGCTT Num.seqs=5 Similarity=0.988148 19
TTTTTGCTCTTCTTGTTGTTTACGAGCTTGTTCATCAGCCTGACG
Rev.of_GCF_000832605.1_ASM83260v1_genomic.fna_1:3728877-3729057 Satlength=181 Nr of Repeats=4 RepeatLength=45 seed=GCTGATGAAC Num.seqs=4 Similarity=0.930864 39
TTTTTGCTCTTCTTGTTGTTTACGAGCCTGTTCATCAGCTTGGCG
Rev.of_GCF_001645555.1_ASM164555v1_genomic.fna_12:642752-643016 Satlength=265 Nr of Repeats=5 RepeatLength=45 seed=GATGAACAAG Num.seqs=4 Similarity=0.916049 81
TTTTTGCTCTTCTTGTTGTTTACGAGCTTGTTCATCAGCTTGGCG
GCF_000161455.1_ASM16145v1_genomic.fna_1:1905065-1905245 Satlength=181 Nr of Repeats=4 RepeatLength=45 seed=GCTTGACGTT Num.seqs=4 Similarity=0.911111 37
TTTTTGCTCTTCTTGTTGTTTTTGAGCTTGTTCCTCAGCTTGACG
Rev.of_GCF_000299035.1_ASM29903v1_genomic.fna_2:20736-20973 Satlength=238 Nr of Repeats=5 RepeatLength=45 seed=GAACAAGCTC Num.seqs=4 Similarity=0.837037 78
TTGTTGCTCTTCCTGTTGTTTACGAGCTTGTTCTTCAGCTTGACG

```
** *** ***** ***** **  **** ** ** *****  * **
```

Consensus:

TTTTTGcTCTTCTTGTTGTTTACGAGCTTGTTCaTCAGCtTGaCG

>Bacillus_Fam_65_26_8 Nr. of seq. 8 Alignment length(with gaps) = 26 Alignment score = 0.630495
GCF_000181495.1_ASM18149v1_genomic.fna_175:18883-19008 Satlength=126 Nr of Repeats=5 RepeatLength=25 seed=AACTGAGCCA Num.seqs=5 Similarity=0.807692
0   AACTGAGCCAGGCTCAAAAGGTGTG-
GCF_000181495.1_ASM18149v1_genomic.fna_181:9837-9962 Satlength=126 Nr of Repeats=5 RepeatLength=25 seed=TCTGAGCCAG Num.seqs=5 Similarity=0.740741
1   ATCTGAGCCAGGCTCAAAAGG-GTGG
Rev.of_GCF_001877785.1_PlanoSAMM_genomic.fna_96:412299-412474 Satlength=176 Nr of Repeats=7 RepeatLength=25 seed=TTTGTGCCTG Num.seqs=7 Similarity=0.910867   18
ATTTGAGCCAGGCACAAAAGG-GTTG
GCF_001877785.1_PlanoSAMM_genomic.fna_106:16546-16696 Satlength=151 Nr of Repeats=6 RepeatLength=25 seed=TTTGAGCCAG Num.seqs=6 Similarity=0.655967
1   GTTTGAGCCAGGCTCAAAAGGTGTA-
Rev.of_GCF_001877785.1_PlanoSAMM_genomic.fna_96:184758-184883 Satlength=126 Nr of Repeats=4 RepeatLength=25 seed=GTGCCTGGCT Num.seqs=3 Similarity=0.964444   15
ATTTGAGCCAGGCACAAACGCCCTG-
GCF_001877785.1_PlanoSAMM_genomic.fna_128:45834-46035 Satlength=202 Nr of Repeats=8 RepeatLength=25 seed=GCCAGGCACA Num.seqs=7 Similarity=0.838217
31   ATTTGAGCCAGGCACAAACGTGATG-
Rev.of_GCF_001877785.1_PlanoSAMM_genomic.fna_139:90007-90157 Satlength=151 Nr of Repeats=6 RepeatLength=25 seed=TTTGAGCCTG Num.seqs=6 Similarity=0.621811   17
ATC-GAGCCAGGCTCAAAACGGCCAC
Rev.of_GCF_001877785.1_PlanoSAMM_genomic.fna_117:70844-70944 Satlength=101 Nr of Repeats=4 RepeatLength=25 seed=TTTGAGCCTG Num.seqs=4 Similarity=0.850427   17
ATCTGAGCCAGGCTCAAACCAGCCGA

********* ****
Consensus:

ATcTGAGCCAGGCtCAAAaggngtgn

>Bacillus_Fam_66_21_8 Nr. of seq. 8 Alignment length(with gaps) = 24 Alignment score = 0.630208
GCF_000508325.1_BAVI_1_genomic.fna_132:10204-10328 Satlength=125 Nr of Repeats=4 RepeatLength=21 seed=TTCGCAAATA Num.seqs=3 Similarity=0.583333
0   -TTCGCAAATAANA--CAGGNGAA
GCF_001591805.1_ASM159180v1_genomic.fna_27:26552-26636 Satlength=85 Nr of Repeats=4 RepeatLength=21 seed=ATTCGCAAAT Num.seqs=4 Similarity=0.676768
0   ATTCGCAAATAAAA--CNGGAGAT
GCF_000612665.1_FF4_genomic.fna_70:25409-25493 Satlength=85 Nr of Repeats=4 RepeatLength=21 seed=ATTCGCAAAT Num.seqs=4 Similarity=0.563131
20   -TTCGCAAATAAAA-TC-GACAAA
GCF_001591665.1_ASM159166v1_genomic.fna_6:121036-121119 Satlength=84 Nr of Repeats=4 RepeatLength=21 seed=TTCGCAAATA Num.seqs=3 Similarity=0.545455
20   -TTCGCAAATAAAACNC-GTCAA-
Rev.of_GCF_001591665.1_ASM159166v1_genomic.fna_57:31859-31943 Satlength=85 Nr of Repeats=4 RepeatLength=21 seed=TTTATTTGCG Num.seqs=4

Similarity=0.635266 33
-TTCGCAAATAAAN--CGGATAAA
Rev.of_GCF_001591665.1_ASM159166v1_genomic.fna_59:63831-63915
Satlength=85 Nr of Repeats=4 RepeatLength=21 seed=TTTATTTGCG Num.seqs=4
Similarity=0.582071 33
-TTCGCAAATAAAA--CGGATTAA
GCF_001591805.1_ASM159180v1_genomic.fna_21:4565-4670 Satlength=106 Nr of Repeats=5 RepeatLength=21 seed=TCGCAAATAA Num.seqs=5 Similarity=0.709091
43 -TTCGCAAATAAAA--CNGGTANA
GCF_001591805.1_ASM159180v1_genomic.fna_27:83451-83535 Satlength=85 Nr of Repeats=4 RepeatLength=21 seed=TTCGCAAATA Num.seqs=4 Similarity=0.622475
63 -TTCGCAAATAAAA--CAGGNAAA

*********** * *
Consensus:

TTCGCAAATAAAACnGgnaAa

>Bacillus_Fam_67_21_8 Nr. of seq. 8 Alignment length(with gaps) = 21
Alignment score = 0.806689
GCF_000007825.1_ASM782v1_genomic.fna_1:871016-871268 Satlength=253 Nr of Repeats=11 RepeatLength=21 seed=TGAAGGAACA Num.seqs=10
Similarity=0.865961
0 TGAAGGAACAGGAAAACCAGG
GCF_000007845.1_ASM784v1_genomic.fna_1:880306-880558 Satlength=253 Nr of Repeats=11 RepeatLength=21 seed=AAGAAACAGA Num.seqs=10
Similarity=0.957672
2 TGAAGAAACAGAAAAGCCAGG
GCF_000290995.1_Baci_cere_AND1407_G13175_V1_genomic.fna_1:43675-44110
Satlength=436 Nr of Repeats=15 RepeatLength=21 seed=AAGAAACAGA
Num.seqs=13 Similarity=0.894180 2
TGAAGAAACAGAAAAGCCAGG
GCF_000831065.1_ASM83106v1_genomic.fna_1:719782-719908 Satlength=127 Nr of Repeats=6 RepeatLength=21 seed=TGAAGGAACA Num.seqs=6
Similarity=0.894180
21 TGAAGGAACAGAAAAGCCAGG
GCF_001645555.1_ASM164555v1_genomic.fna_48:126076-126286 Satlength=211 Nr of Repeats=10 RepeatLength=21 seed=GAAGAAACAG Num.seqs=8
Similarity=0.861678 22
TGAAGAAACAGAAAAAACCAGG
GCF_001889165.1_ASM188916v1_genomic.fna_1:134977-135082 Satlength=106 Nr of Repeats=4 RepeatLength=21 seed=GGAACAGATA Num.seqs=3
Similarity=0.851852
4 NGAAGGAACAGATAACCCAGG
GCF_000496285.1_ASM49628v1_genomic.fna_1:3488675-3488864 Satlength=190 Nr of Repeats=9 RepeatLength=21 seed=ACACCAGGTG Num.seqs=9
Similarity=0.925926 13
TGGAGGAACAGAAACACCAGG
Rev.of_GCF_000832605.1_ASM83260v1_genomic.fna_1:4861442-4861757
Satlength=316 Nr of Repeats=15 RepeatLength=21 seed=CTCCACCTGG
Num.seqs=15 Similarity=0.958881
26 TGGAGAGACAGAAACACCAGG

* ** **** * *****

Consensus:

TGaAGaAACAGAAAaaCCAGG

>Bacillus_Fam_68_52_7 Nr. of seq. 7 Alignment length(with gaps) = 54
Alignment score = 0.639624
GCF_000759675.1_ASM75967v1_genomic.fna_13:145209-145417 Satlength=209 Nr of Repeats=4 RepeatLength=52 seed=CGGTCACTTA Num.seqs=4 Similarity=0.730084
0 CGGTCACTTATAGCCCTTATTAGTGACCACTTTTTCGAATTTCCTT-TGATTA-
Rev.of_GCF_000759675.1_ASM75967v1_genomic.fna_155:58436-59008 Satlength=573 Nr of Repeats=11 RepeatLength=52 seed=TGGTAACTAA Num.seqs=11 Similarity=0.890452
29 CGGTCACTTATAGCCTCTATTAGTTACCACTTTTTCG-ATTTTTTC-TCATTTT
GCF_000759675.1_ASM75967v1_genomic.fna_57:134608-134868 Satlength=261 Nr of Repeats=5 RepeatLength=52 seed=ATTTCGGTCA Num.seqs=5 Similarity=0.794969
48 CGGTCACTTATAGCCTTCATTAGTGACCACTCTTTCT-ATTTTAACAAGATTT-
Rev.of_GCF_000759675.1_ASM75967v1_genomic.fna_134:29911-30379 Satlength=469 Nr of Repeats=9 RepeatLength=52 seed=TATGAGTGAC Num.seqs=9 Similarity=0.912393 64
CGGTCACTCATAGCCTTTATGAGTGACCACTTTTTAA-ATTTAAACAAGATTA-
GCF_000759675.1_ASM75967v1_genomic.fna_85:197429-198209 Satlength=781 Nr of Repeats=15 RepeatLength=52 seed=AGCCTTCATG Num.seqs=15 Similarity=0.914530 63
CGGTCACTCATAGCCTTCATGAGTGCCCGGTTTTCCA-ATTCTAACAAGATTC-
GCF_000759675.1_ASM75967v1_genomic.fna_73:261668-261928 Satlength=261 Nr of Repeats=5 RepeatLength=52 seed=ATTTCGGTCA Num.seqs=5 Similarity=0.583929
48 CGGTCACCNATAGCACTCATTGATGACCACTTTCTCT-ATTTCAACCANATTT-
GCF_000759675.1_ASM75967v1_genomic.fna_153:33935-34143 Satlength=209 Nr of Repeats=4 RepeatLength=52 seed=TTGGTTACCA Num.seqs=4 Similarity=0.816239
71 CGGTCACCTATAGTCCTCATTGGTTACCACTTTCTCT-ATTCCAACCAGATTT-

```
*******  ****     **   *  **  * *     ***        ***
```
Consensus:

CGGTCACttATAGCCtTcATtaGTgACCACTTTtTCnATTtnaaCnagATTt

>Bacillus_Fam_69_52_7 Nr. of seq. 7 Alignment length(with gaps) = 54
Alignment score = 0.627572
GCF_000813125.1_ASM81312v1_genomic.fna_25:23691-23951 Satlength=261 Nr of Repeats=5 RepeatLength=52 seed=GGACAACTTT Num.seqs=5 Similarity=0.928205
0 GGACAACTTTTC-GTTCTTTTCCT-CTAAACCTGTCTGAAGTTGCACCTCCTTC
GCF_000934845.1_ASM93484v1_genomic.fna_4:21973-22597 Satlength=625 Nr of Repeats=12 RepeatLength=52 seed=GTTCGGACTA Num.seqs=10 Similarity=0.788889
48 GGACTACTTTTC-G-CCTTTTCCTGCAAAACCTGTCCGAAGTTGGCTCTTGTTC
Rev.of_GCF_000934845.1_ASM93484v1_genomic.fna_57:39159-39367 Satlength=209 Nr of Repeats=4 RepeatLength=52 seed=CAACTTCGGA Num.seqs=4 Similarity=0.770576 94
GGACTGCTTTTC-G-CTTTTTCCGACAAAACCAGTCCGAAGTTGGCTCTAGTTC

Rev.of_GCF_000813125.1_ASM81312v1_genomic.fna_64:18546-18857 Satlength=312 Nr of Repeats=5 RepeatLength=52 seed=ACAGGTTTGG Num.seqs=4 Similarity=0.676440 33 TGACAGCTTTTCTG-GCTTTCCCT-CCAAACCTGTCTGAAGTTGGGCGATGTTC

GCF_000813125.1_ASM81312v1_genomic.fna_75:60242-60554 Satlength=313 Nr of Repeats=6 RepeatLength=52 seed=GACAGCTTTT Num.seqs=6 Similarity=0.788034 1   TGACAGCTTTTC-C-ACTTCTTCCTCCAAACCTGTCCGAACTTGGGGCTACTTC

Rev.of_GCF_000813125.1_ASM81312v1_genomic.fna_92:118219-118635 Satlength=417 Nr of Repeats=8 RepeatLength=52 seed=GGAAAAGCTG Num.seqs=8 Similarity=0.619260 13 TGACAGCTTTTC-C-TCTTNCCCGACCAAACCTGTCCGAACTTGGNGCTTCTTT

GCF_000813125.1_ASM81312v1_genomic.fna_133:9463-9775 Satlength=313 Nr of Repeats=5 RepeatLength=52 seed=CTGACAGCTT Num.seqs=4 Similarity=0.690329 51   TGACAGCTTTTC-C-TCNGTTCCCATCAAACCTGTCCGAACTTGCCTCTACTTC

*** ******   *   ***** *** *** ***   **

Consensus:

tGACagCTTTTCgnCTTttCCnnCcAAACCTGTCcGAAgTTGgnnCTncTTC

>Bacillus_Fam_70_52_7 Nr. of seq. 7 Alignment length(with gaps) = 54 Alignment score = 0.638448

GCF_001636345.1_ASM163634v1_genomic.fna_1:931350-932077 Satlength=728 Nr of Repeats=14 RepeatLength=52 seed=AATCTGGAAA Num.seqs=13 Similarity=0.791566 0 AATCTGGAAAATGGTGTCGTGA-AAAGGGAAGAAACACCCCTCTATAATCCAN-

GCF_001636345.1_ASM163634v1_genomic.fna_1:1522369-1522681 Satlength=313 Nr of Repeats=6 RepeatLength=52 seed=CAGTATCTGG Num.seqs=6 Similarity=0.885470 49 TATCTGGAAAATGGTGTCGTGA-AAAGGAGAGAAACACCCCTCTAAAGTCCAG-

Rev.of_GCF_001636345.1_ASM163634v1_genomic.fna_1:3881060-3881632 Satlength=573 Nr of Repeats=11 RepeatLength=52 seed=GGTGTTTCTC Num.seqs=11 Similarity=0.874126 38   AAAAAGGTAAACAGTGTCGCGA-AAAGCAGAGAAACACCCCTCTATAATCCAG-

Rev.of_GCF_001636345.1_ASM163634v1_genomic.fna_1:4336082-4336445 Satlength=364 Nr of Repeats=7 RepeatLength=52 seed=GGGGTGTTTC Num.seqs=6 Similarity=0.835897 40 AATCTAGAAAATAGAGTCGCGG-AAAGGGGAGAAACACCCCCGTGGAATCCAG-

Rev.of_GCF_001636345.1_ASM163634v1_genomic.fna_1:3624890-3625255 Satlength=366 Nr of Repeats=7 RepeatLength=52 seed=AGGGGTGTTT Num.seqs=6 Similarity=0.870085 40 AAAAGGGAAAAT-GTGTCTTGATTAAGG-GAGAAACACCCCTCTGAAATCCAGA

Rev.of_GCF_001636345.1_ASM163634v1_genomic.fna_1:3412758-3413277 Satlength=520 Nr of Repeats=10 RepeatLength=52 seed=CTGTTTTCGA Num.seqs=9 Similarity=0.849715 27   -AAATGGTAAATGGTGTCTCGA-AAACAGNGGAAACACCCCACTAAAAACCAGT

GCF_001636345.1_ASM163634v1_genomic.fna_1:1414565-1414877 Satlength=313 Nr of Repeats=6 RepeatLength=52 seed=TCCAGAAAAA Num.seqs=6 Similarity=0.822222 47 AAAAAGGAAAATGGTGTCGCGG-AAACCNGATAAACACACCCGTAAAGTCCAG-

*   * *** * *** *   **   ****** ** * * ***

Consensus:

aAaatGGaAAATgGTGTCgcGaAAAggngAGAAACACCCCtcTaaAaTCCAG

>Bacillus_Fam_71_52_7 Nr. of seq. 7 Alignment length(with gaps) = 54
Alignment score = 0.641387
GCF_002019645.1_ASM201964v1_genomic.fna_1:579578-581402 Satlength=1825 Nr of Repeats=35 RepeatLength=52 seed=TGTCTGAAGT Num.seqs=34
Similarity=0.899173 0
TGTCTGAAGTAGC-CTCGNGTTCGGACAGCTTTGA-TTGTTTAGAAGTAAAAGC
Rev.of_GCF_002019645.1_ASM201964v1_genomic.fna_1:2223040-2223404
Satlength=365 Nr of Repeats=7 RepeatLength=52 seed=GCTACTTCAG Num.seqs=7
Similarity=0.912088 13
TGTCTGAAGTAGC-CTCGGGTTCGGACAGCTTTGA-TTGTTTTAAAGCAAAAGC
Rev.of_GCF_002019645.1_ASM201964v1_genomic.fna_1:4753408-4753928
Satlength=521 Nr of Repeats=10 RepeatLength=52 seed=GCTACTTCAG
Num.seqs=10 Similarity=0.850142
13 TGTCTGAAGTAGC-GTTGGGTTCGGACAGGTTTGG-TTGGTTTTAGGCTAAAGC
Rev.of_GCF_002019645.1_ASM201964v1_genomic.fna_1:1988468-1989404
Satlength=937 Nr of Repeats=18 RepeatLength=52 seed=AAGCTGTCCG
Num.seqs=18 Similarity=0.815904
31 TGTCTGAAGTTGC-CTTGGGTTCGGACAGC-TTGACTTCATTTCTTGCTAAAGC
Rev.of_GCF_002019645.1_ASM201964v1_genomic.fna_1:2086406-2086926
Satlength=521 Nr of Repeats=10 RepeatLength=52 seed=TTCAGACAGC
Num.seqs=10 Similarity=0.776068
8 TGTCTGAAGTTCC-CTCCACTTCGGACAGCTTTGA-TTCGTTCTGGGCCAAAGC
GCF_002019645.1_ASM201964v1_genomic.fna_1:1098526-1099150 Satlength=625
Nr of Repeats=12 RepeatLength=52 seed=TGTCCGAAGT Num.seqs=12
Similarity=0.860917 0
TGTCCGAAGTTGCTCT-GAGTTCAGACAGGTTTAG-GGGTTTGGAAGCAAAAGC
Rev.of_GCF_002019645.1_ASM201964v1_genomic.fna_1:4437435-4438782
Satlength=1348 Nr of Repeats=26 RepeatLength=52 seed=TACTTCAGAC
Num.seqs=24 Similarity=0.790994
11 TGTCTGAAGTAGCTCT-GTGTTCGGACAGCTTTCG-TGCTTTACAAGGCAAAGT

**** ***** * * *** ***** ** ** * ****
Consensus:

TGTCTGAAGTaGCCTnGnGTTCGGACAGcTTTgaTtgtTTnnaaGcnAAAGC

>Bacillus_Fam_72_52_7 Nr. of seq. 7 Alignment length(with gaps) = 52
Alignment score = 0.731380
GCF_002019645.1_ASM201964v1_genomic.fna_1:2495463-2495723 Satlength=261
Nr of Repeats=4 RepeatLength=52 seed=CATAGGCCTT Num.seqs=3
Similarity=0.811966 0
CATAGGCCTTCTATGATTTAGTTGTCTGAGCGAAAACAAGGAGAAAGTAAAT
Rev.of_GCF_002019645.1_ASM201964v1_genomic.fna_1:4542766-4542973
Satlength=208 Nr of Repeats=4 RepeatLength=52 seed=CAGGCAACTT Num.seqs=3
Similarity=0.761006 28
CATAGGCCTTCTATGATCAAGTTGCCTGGACGAAAACGAGGAGNAAGTAAAC
GCF_002019645.1_ASM201964v1_genomic.fna_1:2741337-2741701 Satlength=365
Nr of Repeats=7 RepeatLength=52 seed=AAGGAGGAAG Num.seqs=7
Similarity=0.854701 37
CATACNCCCTCTATGATCCAGTTGCCGAGGCGAAACCAAGGAGGAAGTGAAT

Rev.of_GCF_002019645.1_ASM201964v1_genomic.fna_1:3938951-3939315
Satlength=365 Nr of Repeats=7 RepeatLength=52 seed=GCCTATGATT Num.seqs=7
Similarity=0.866427 59
CATAGGCCTTCTATGAGTCAGTTGCCGAAGCGAAAACAAGGAGGAAGTGAAT
Rev.of_GCF_002019645.1_ASM201964v1_genomic.fna_1:3790499-3790810
Satlength=312 Nr of Repeats=6 RepeatLength=52 seed=TTTCTCCTTG Num.seqs=5
Similarity=0.838462 46
CATAGGCTCTCTATGATTCAGTTGCTGAGGCAAAAACAAGGAGAAAGTGAAT
GCF_002019645.1_ASM201964v1_genomic.fna_1:5108997-5109209 Satlength=213
Nr of Repeats=4 RepeatLength=52 seed=AAGTGAATCA Num.seqs=3
Similarity=0.897436 96
CATTGGCTCTCTATGATTCAGTTGCTAAAGCGGAAACAAGGAGAAAGTGAAT
GCF_002019645.1_ASM201964v1_genomic.fna_1:4292665-4292873 Satlength=209
Nr of Repeats=4 RepeatLength=52 seed=AGGAACTCAA Num.seqs=4
Similarity=0.910256 41
CATAGACCTGCTTTGATCCAGTTGCAGAAGCAAAAACAAAGAGGAACTCAAT

```
***   *   ** ***   *****      *  ** * * *** ** * **
```

Consensus:

CATAGgCctTCTATGATtcAGTTGCcgaaGCgAAAACAAGGAGnAAGTgAAT

>Bacillus_Fam_73_20_7 Nr. of seq. 7 Alignment length(with gaps) = 22
Alignment score = 0.655123
GCF_001315165.1_ASM131516v1_genomic.fna_64:18994-19094 Satlength=101 Nr
of Repeats=5 RepeatLength=20 seed=TTTTATAGCG Num.seqs=5
Similarity=0.515278
0   TTTTAT-AGCGA--NTTTCNAT
Rev.of_GCF_001591625.1_ASM159162v1_genomic.fna_70:0-79 Satlength=80 Nr of
Repeats=4 RepeatLength=20 seed=TCGCTGATAA Num.seqs=4 Similarity=1.000000
12   TTTTATCAGCGA--CTTTCCCT
Rev.of_GCF_001591625.1_ASM159162v1_genomic.fna_110:11874-11954
Satlength=81 Nr of Repeats=4 RepeatLength=20 seed=GGGAAAGTCG Num.seqs=4
Similarity=1.000000 19
TTTTATCAGCGA--CTTTCCCT
Rev.of_GCF_001315165.1_ASM131516v1_genomic.fna_3:8732-8832 Satlength=101
Nr of Repeats=5 RepeatLength=20 seed=TCGCTATAAA Num.seqs=5
Similarity=0.631746 11
TTTTAT-AGCGA-NTTTNTCNT
Rev.of_GCF_001315165.1_ASM131516v1_genomic.fna_53:86-186 Satlength=101 Nr
of Repeats=5 RepeatLength=20 seed=TCGCTATAAA Num.seqs=5
Similarity=0.631746 11
TTTTAT-AGCGA-NTTTTTCNT
GCF_001315165.1_ASM131516v1_genomic.fna_77:6171-6271 Satlength=101 Nr of
Repeats=5 RepeatLength=20 seed=TTTATAGCGA Num.seqs=5 Similarity=0.661905
21   TTTTAT-AGCGA-GTTTTACTT
Rev.of_GCF_001315165.1_ASM131516v1_genomic.fna_213:71-191 Satlength=121
Nr of Repeats=6 RepeatLength=20 seed=CGCTATAAAA Num.seqs=6
Similarity=0.631313 31
TTTTAT-AGCGNAATTTTNCTT

```
****** ****    **    *
```

Consensus:

TTTTATAGCGAntTTTnCnT

>Bacillus_Fam_74_21_7 Nr. of seq. 7 Alignment length(with gaps) = 22
Alignment score = 0.698413
GCF_001591425.1_ASM159142v1_genomic.fna_1:10873-11167 Satlength=295 Nr of Repeats=14 RepeatLength=21 seed=TTTATTTATC Num.seqs=14
Similarity=0.725275
0 TTTATTTATCACCGCACTCCG-
Rev.of_GCF_001591425.1_ASM159142v1_genomic.fna_40:10038-10143
Satlength=106 Nr of Repeats=5 RepeatLength=21 seed=TGATAAATAA Num.seqs=5
Similarity=0.733333 11
TTTATTTATCACCGACCNCNG-
Rev.of_GCF_001591425.1_ASM159142v1_genomic.fna_40:16835-16961
Satlength=127 Nr of Repeats=6 RepeatLength=21 seed=TGATAAATAA Num.seqs=6
Similarity=0.762963 11
TTTATTTATCACCGACCCCCG-
Rev.of_GCF_001591425.1_ASM159142v1_genomic.fna_40:19702-19849
Satlength=148 Nr of Repeats=7 RepeatLength=21 seed=TGATAAATAA Num.seqs=7
Similarity=0.794407 11
TTTATTTATCACCGAACGCNT-
Rev.of_GCF_001591425.1_ASM159142v1_genomic.fna_40:23367-23615
Satlength=249 Nr of Repeats=12 RepeatLength=21 seed=TGATAAATAA
Num.seqs=11 Similarity=0.710245
11 TTTATTTATCANCGANCTCNN-
Rev.of_GCF_001591425.1_ASM159142v1_genomic.fna_62:9174-9363 Satlength=190
Nr of Repeats=9 RepeatLength=21 seed=TGATAAATAA Num.seqs=9
Similarity=0.514245 11
TTTATTTATCAACGA-CTCCNA
GCF_001591425.1_ASM159142v1_genomic.fna_54:21619-21808 Satlength=190 Nr of Repeats=9 RepeatLength=21 seed=TTATTTATCA Num.seqs=9
Similarity=0.576892
1 TTTATTTATCACC-AACNCNAG

*********** * * *
Consensus:

TTTATTTATCAcCGAnCnCnn

>Bacillus_Fam_75_22_7 Nr. of seq. 7 Alignment length(with gaps) = 22
Alignment score = 0.622655
GCF_001645685.2_ASM164568v2_genomic.fna_1:3341360-3341445 Satlength=86 Nr of Repeats=4 RepeatLength=21 seed=TTTACTCGCC Num.seqs=3
Similarity=0.686869 0
TTTACTCGCCAATTTAGCGAC-
Rev.of_GCF_001645685.2_ASM164568v2_genomic.fna_1:4482743-4483219
Satlength=477 Nr of Repeats=13 RepeatLength=21 seed=TGGCGAGTAA
Num.seqs=11 Similarity=0.662534
11 TTTACTCGCCAAATTAGCGNG-
Rev.of_GCF_001645685.2_ASM164568v2_genomic.fna_1:4454332-4454416
Satlength=85 Nr of Repeats=4 RepeatLength=21 seed=TTGGCGAGTA Num.seqs=4
Similarity=0.682540 12
TTTACTCGCCAAATTTG-GATG

GCF_001645685.2_ASM164568v2_genomic.fna_1:4248891-4249324 Satlength=434 Nr of Repeats=5 RepeatLength=21 seed=ACTCGCCAAT Num.seqs=3 Similarity=0.957672 3
TTTACTCGCCAA-TTCAGCACT
GCF_001645685.2_ASM164568v2_genomic.fna_1:4241205-4241656 Satlength=452 Nr of Repeats=10 RepeatLength=21 seed=TTACTCGCCA Num.seqs=6 Similarity=0.818687 23
TTTACTCGCCAATTTGNGCACT
Rev.of_GCF_001645685.2_ASM164568v2_genomic.fna_1:4466835-4466919 Satlength=85 Nr of Repeats=4 RepeatLength=21 seed=TCGCGAGTAA Num.seqs=4 Similarity=0.596618 11
TTTACTCGCGAATTTGTCNACT
GCF_001591665.1_ASM159166v1_genomic.fna_66:6860-6944 Satlength=85 Nr of Repeats=4 RepeatLength=21 seed=TTTACTCGCG Num.seqs=4 Similarity=0.600242
0   TTTACTCGCGAATTCTGNCATN

********* ** *
Consensus:

TTTACTCGCcAAtTTngnnAcn

>Bacillus_Fam_76_21_7 Nr. of seq. 7 Alignment length(with gaps) = 21 Alignment score = 0.702948
GCF_001591425.1_ASM159142v1_genomic.fna_25:49292-49439 Satlength=148 Nr of Repeats=7 RepeatLength=21 seed=TTACTTAACA Num.seqs=7 Similarity=0.550725
0   TTACTTAACAAGTNTTGAAA-
Rev.of_GCF_001591425.1_ASM159142v1_genomic.fna_38:25074-25221 Satlength=148 Nr of Repeats=7 RepeatLength=21 seed=TGTTAAGTAA Num.seqs=7 Similarity=0.761149 10
TTACTTAACANGTANTGAAAA
Rev.of_GCF_001591425.1_ASM159142v1_genomic.fna_88:2823-2970 Satlength=148 Nr of Repeats=7 RepeatLength=21 seed=TGTTAAGTAA Num.seqs=7 Similarity=0.667423 10
TTACTTAACANGTATTNAAAN
Rev.of_GCF_001591425.1_ASM159142v1_genomic.fna_5:44236-44383 Satlength=148 Nr of Repeats=7 RepeatLength=21 seed=CTGTTAAGTA Num.seqs=5 Similarity=0.659420 11
ATACTTAACAGCTTTTCAAAA
GCF_001591425.1_ASM159142v1_genomic.fna_161:2289-2478 Satlength=190 Nr of Repeats=9 RepeatLength=21 seed=TTACTTAACA Num.seqs=9 Similarity=0.738977
21   TTACTTAACAGCNTTTCAAAA
GCF_001591425.1_ASM159142v1_genomic.fna_16:280-445 Satlength=166 Nr of Repeats=6 RepeatLength=21 seed=ACTTAACAGA Num.seqs=4 Similarity=0.622475
23   TTACTTAACAAATTGACAAAA
GCF_001591425.1_ASM159142v1_genomic.fna_34:0-104 Satlength=105 Nr of Repeats=5 RepeatLength=21 seed=TTACTTAACA Num.seqs=5 Similarity=0.746032
42   TTACTTAACAACTTTCCAAAT

*********   ***
Consensus:

TTACTTAACAnnTtttcAAAa

>Bacillus_Fam_77_15_7 Nr. of seq. 7 Alignment length(with gaps) = 16
Alignment score = 0.610119
GCF_000009825.1_ASM982v1_genomic.fna_1:3550903-3551497 Satlength=595 Nr of Repeats=11 RepeatLength=15 seed=CCTGGTTCTT Num.seqs=7 Similarity=0.741799
0 CCTGGTTCTTCAGTN-
Rev.of_GCF_000305495.1_BacPsy2.0_genomic.fna_17:24629-24719 Satlength=91 Nr of Repeats=6 RepeatLength=15 seed=GGTACTGAAA Num.seqs=6 Similarity=0.946667 2
CCTGGTTTTTCAGTA-
GCF_002019645.1_ASM201964v1_genomic.fna_1:2043017-2043122 Satlength=106 Nr of Repeats=7 RepeatLength=15 seed=CCAGGCTCTT Num.seqs=7 Similarity=0.915344 0
CCAGGCTCTTCAGTA-
GCF_900142675.1_IMG-taxon_2654588196_annotated_assembly_genomic.fna_7:329335-329959 Satlength=625 Nr of Repeats=10 RepeatLength=15 seed=TCGGTGCCTG Num.seqs=8 Similarity=0.888889 9
CCTGGCTCTTCGGTG-
Rev.of_GCF_001591585.1_ASM159158v1_genomic.fna_18:75061-75166 Satlength=106 Nr of Repeats=7 RepeatLength=15 seed=GGAGCAGAAG Num.seqs=7 Similarity=0.881481 1 -
CCGGTTCTTCTGCTC
GCF_002019645.1_ASM201964v1_genomic.fna_1:5231068-5231188 Satlength=121 Nr of Repeats=7 RepeatLength=15 seed=TCTTCTGTTC Num.seqs=6 Similarity=0.851852 5
-CCGGTTCTTCTGTTC
Rev.of_GCF_002019645.1_ASM201964v1_genomic.fna_1:422359-422539 Satlength=181 Nr of Repeats=11 RepeatLength=15 seed=GATCCAGGAA Num.seqs=10 Similarity=0.796543
7 -CTGGATCTTCTGTTC

* ** * *** *
Consensus:

cCtGGtTCTTCnGTn

>Bacillus_Fam_78_12_7 Nr. of seq. 7 Alignment length(with gaps) = 12
Alignment score = 0.830688
GCF_000025805.1_ASM2580v1_genomic.fna_1:1178251-1178455 Satlength=205 Nr of Repeats=12 RepeatLength=12 seed=CGGCGAGTAG Num.seqs=8 Similarity=0.908730 0
CGGCGAGTAGAA
GCF_000025825.1_ASM2582v1_genomic.fna_1:1189082-1189262 Satlength=181 Nr of Repeats=12 RepeatLength=12 seed=CGAGTAGAAC Num.seqs=11 Similarity=0.919192 3
CGGCGAGTAGAA
Rev.of_GCF_000225265.1_ASM22526v1_genomic.fna_1:3779323-3779479 Satlength=157 Nr of Repeats=13 RepeatLength=12 seed=CTACTCGCCG Num.seqs=13 Similarity=0.854701
10 CGGCGAGTAGAA
GCF_000025805.1_ASM2580v1_genomic.fna_1:1377397-1377736 Satlength=340 Nr of Repeats=26 RepeatLength=12 seed=AGAACGGCGA Num.seqs=24

Similarity=0.884461 8
CGGCGAGAAGAA
GCF_000025805.1_ASM2580v1_genomic.fna_1:1377397-1377820 Satlength=424 Nr of Repeats=29 RepeatLength=12 seed=AGAACGGCGA Num.seqs=24
Similarity=0.884461 8
CGGCGAGAAGAA
Rev.of_GCF_000242895.2_ASM24289v3_genomic.fna_1:2771381-2771945
Satlength=565 Nr of Repeats=41 RepeatLength=12 seed=CTACTCGTCG
Num.seqs=35 Similarity=0.758242
10 CGACGAGTAGAG
Rev.of_GCF_000956595.1_ASM95659v1_genomic.fna_17:23745-23997
Satlength=253 Nr of Repeats=21 RepeatLength=12 seed=CTACTCGCCG
Num.seqs=21 Similarity=0.863492
10 CGGCGAGTAGTG

** **** **
Consensus:

CGGCGAGtAGAa

>Bacillus_Fam_79_12_7 Nr. of seq. 7 Alignment length(with gaps) = 12
Alignment score = 0.640212
GCF_000331575.1_ASM33157v1_genomic.fna_1:153900-154010 Satlength=111 Nr of Repeats=7 RepeatLength=12 seed=ATTTTGATTT Num.seqs=5
Similarity=0.777778
0 ATTTTGATTTTG
GCF_000331575.1_ASM33157v1_genomic.fna_4:18792-18873 Satlength=82 Nr of Repeats=5 RepeatLength=12 seed=GTTGATGTTG Num.seqs=4 Similarity=1.000000
2 ATGTTGATGTTG
Rev.of_GCF_001591625.1_ASM159162v1_genomic.fna_36:9709-9757 Satlength=49 Nr of Repeats=4 RepeatLength=12 seed=TCAAAGTCAA Num.seqs=4
Similarity=0.702991 1
ACTTTGACTTTG
Rev.of_GCF_000496285.1_ASM49628v1_genomic.fna_1:1498693-1498783
Satlength=91 Nr of Repeats=5 RepeatLength=12 seed=CAATATCAAC Num.seqs=3
Similarity=0.851852 0
CTGTTGATATTG
Rev.of_GCF_001315085.1_ASM131508v1_genomic.fna_9:7125-7221 Satlength=97 Nr of Repeats=7 RepeatLength=12 seed=CAACAACAGC Num.seqs=5
Similarity=0.800000 9
CTGTTGTTGTTG
Rev.of_GCF_001578185.1_ASM157818v1_genomic.fna_1:771792-772062
Satlength=271 Nr of Repeats=8 RepeatLength=12 seed=TCAACAGCAA Num.seqs=5
Similarity=0.911111 7
CTGTTGACCTTG
Rev.of_GCF_001420595.1_ASM142059v1_genomic.fna_11:2152936-2153020
Satlength=85 Nr of Repeats=7 RepeatLength=12 seed=TCAACAACAA Num.seqs=7
Similarity=0.851852 7
TTGTTGAGTTTG

*** ***
Consensus:

nTgTTGAtnTTG

>Bacillus_Fam_80_52_6 Nr. of seq. 6 Alignment length(with gaps) = 58
Alignment score = 0.624904
GCF_000759675.1_ASM75967v1_genomic.fna_125:36622-37090 Satlength=469 Nr of Repeats=9 RepeatLength=52 seed=CGGTCACTCA Num.seqs=9 Similarity=0.875356
0 -CGGTCACTCATAG--GGTAGATGAGAGCCCGAGAATAGTTAAAAGAAGGAAG-TG--
GCF_000759675.1_ASM75967v1_genomic.fna_130:33497-34016 Satlength=520 Nr of Repeats=10 RepeatLength=52 seed=AGATGAGAGC Num.seqs=9 Similarity=0.860399
16 -CGGTCACTCATAG--GGTAGATGAGAGCCCGTGAATAGCTAAAAGAAGGAAG-AG--
Rev.of_GCF_000759675.1_ASM75967v1_genomic.fna_134:133322-134102 Satlength=781 Nr of Repeats=15 RepeatLength=52 seed=AGTGACCGCT Num.seqs=15 Similarity=0.888889
8 -CGGTCACTCATAA--AGTAGATGAGAGCCCGNGAATAGCAAAAAGAAGAAAG-AG--
Rev.of_GCF_000759675.1_ASM75967v1_genomic.fna_134:112507-112871 Satlength=365 Nr of Repeats=7 RepeatLength=52 seed=ATGAGTGACC Num.seqs=7 Similarity=0.958486 11 -TGGTCACTCATAGC-GGT-TATGAGAGCCCGGAAACAGCAAAAAGGAGAAAG-AG--
Rev.of_GCF_000759675.1_ASM75967v1_genomic.fna_151:23767-24183 Satlength=417 Nr of Repeats=8 RepeatLength=52 seed=TCATCACCTT Num.seqs=8 Similarity=0.877289 22 -CGGTCACTCATAA-AGGT-GATGAGAGCCCGAAGACTGCAAAAAGAAG--AGCTGA-
Rev.of_GCF_000759675.1_ASM75967v1_genomic.fna_153:338216-338424 Satlength=209 Nr of Repeats=4 RepeatLength=52 seed=TAAGATTACG Num.seqs=4 Similarity=0.965812 38
GCGGTCACTCATAA-GGGTA-ATGAGAGCCCGT-AAT-CTTATAATAAG--AGCAGAA

*********** ** *********** * * ** ** ** *
Consensus:

CGGTCACTCATAaGGTagATGAGAGCCCGngAAtaGcaAAAAGAAGnaAGaG

>Bacillus_Fam_81_52_6 Nr. of seq. 6 Alignment length(with gaps) = 57
Alignment score = 0.684016
GCF_000621445.1_ASM62144v1_genomic.fna_2:115234-115494 Satlength=261 Nr of Repeats=5 RepeatLength=52 seed=ATGAAGGACA Num.seqs=5 Similarity=0.823899
75 GAGAAGTGTCCTTCATAAAGG-GAATGAAGGACAAAACTAGT--GAGAAAC-AG-AG
GCF_000621445.1_ASM62144v1_genomic.fna_2:130298-130558 Satlength=261 Nr of Repeats=5 RepeatLength=52 seed=TGAAGGACAA Num.seqs=5 Similarity=0.920513
75 CAAAAGTGTCCTTCATAAAGGNGA-TGAAGGACAAAACAAG---GAGAAAC-CGAAG
Rev.of_GCF_000621445.1_ASM62144v1_genomic.fna_3:128510-128771 Satlength=262 Nr of Repeats=5 RepeatLength=52 seed=ATGAAGGACA Num.seqs=4 Similarity=0.901709 119
CAAAAGTGTCCTTCATAAAGGTGA-TGAAGGACAAAACAAG---GAGAAAG-CGAAG
Rev.of_GCF_000621445.1_ASM62144v1_genomic.fna_3:303079-303287 Satlength=209 Nr of Repeats=4 RepeatLength=52 seed=TATGAAGGAC Num.seqs=4 Similarity=0.888889 120
CGAAAGTGTCCTTCATAAAGGCGA-TGAAGGACAAAAGAAG---GAGAAAA-CGAAG
GCF_000621445.1_ASM62144v1_genomic.fna_20:46586-46794 Satlength=209 Nr of Repeats=4 RepeatLength=52 seed=AGAAAGTGTC Num.seqs=4 Similarity=0.828189
103 --AAAGTGTCCTTCATAAAGGCGA-TGAAGGACAAAACGAG-AGGAAAAACTTG-AG

Rev.of_GCF_900104555.1_IMG-taxon_2651870172_annotated_assembly_genomic.fna_19:114955-115475 Satlength=521 Nr of Repeats=9 RepeatLength=52 seed=ATGAAGGACT Num.seqs=7 Similarity=0.797766 83 --AGTGAATC-AGATGGAAAA-AGTCCTTCATAGAGCTGA-TGAAGGACAAAACCAG

* * ** * ** ** * *
Consensus:

GGAGAAAccGaAGcaAAAGTGTCCTTCATAAAGGnGATGAAGGACAAAAcaA

>Bacillus_Fam_82_52_6 Nr. of seq. 6 Alignment length(with gaps) = 57 Alignment score = 0.644444
GCF_000153365.1_ASM15336v1_genomic.fna_10:208343-208606 Satlength=264 Nr of Repeats=5 RepeatLength=53 seed=AAAAGGAGCA Num.seqs=3 Similarity=0.855967
0 AAAAG-GAGCAGTCATGTATCCCGGGAGGAGTGCGGGATGCATGAG-TG-G-GTCGA
Rev.of_GCF_000473245.1_ASM47324v1_genomic.fna_1:2810130-2811347 Satlength=1218 Nr of Repeats=23 RepeatLength=53 seed=GACTGCTCCT Num.seqs=21 Similarity=0.867984
13 AAAAG-GAGCAGTCATGTATCCCGGGAGGAGTGCGGGATACATGAG-TG-G-GTCGA
GCF_000153365.1_ASM15336v1_genomic.fna_12:72983-73301 Satlength=319 Nr of Repeats=6 RepeatLength=53 seed=TCATGTATTC Num.seqs=6 Similarity=0.948008
9 -GAAG-G-GCAGTCATGTATTCGGGGAGCGTTCCGGGATACATGAGCTGTG-CTCGG
GCF_000473245.1_ASM47324v1_genomic.fna_1:1054274-1054592 Satlength=319 Nr of Repeats=6 RepeatLength=53 seed=TCATGTATTC Num.seqs=6 Similarity=0.948008 9 -GAAG-G-GCAGTCATGTATTCGGGGAGCGTTCCGGGATACATGAGCTGTG-CTCGG
Rev.of_GCF_000153365.1_ASM15336v1_genomic.fna_18:460556-461987 Satlength=1432 Nr of Repeats=27 RepeatLength=53 seed=ACTCATGTAC Num.seqs=25 Similarity=0.833795
46 AAAAGAG-GAAGTCATGTATGCCGGGATCGTTTTGGAGTACATGAG-TG-GTGT-GA
GCF_000473245.1_ASM47324v1_genomic.fna_1:491395-492826 Satlength=1432 Nr of Repeats=27 RepeatLength=53 seed=GTACATGAGT Num.seqs=27 Similarity=0.833934 89 AAAAGAG-GAAGTCATGTATGCCGGGATCGTTTTGGAGTACATGAG-TG-GTGT-GA

*** * * ********** * **** * ** * ****** ** * * *
Consensus:

aaAAGGGcAGTCATGTATnCcGGGAgcgtTncGGgaTACATGAGTGGgTcGa

>Bacillus_Fam_83_52_6 Nr. of seq. 6 Alignment length(with gaps) = 56 Alignment score = 0.775397
GCF_000752395.1_Bacillus_andreraoultii_genomic.fna_23:1814-2438 Satlength=625 Nr of Repeats=12 RepeatLength=52 seed=AACCCATTTT Num.seqs=12 Similarity=0.899276
0 AACC--CA-TTTTCGCGAAATCCTCTTCATTTTCGGCTCATAG-ACTCACTCTATG
GCF_000752395.1_Bacillus_andreraoultii_genomic.fna_23:79057-79681 Satlength=625 Nr of Repeats=12 RepeatLength=52 seed=TTCTATGAAC Num.seqs=12 Similarity=0.757766
45 AACC-AC--TTTTCGCGAAATCCTCATCATTTTNGGCTCATAGAACTC-TTCTATG
GCF_000752395.1_Bacillus_andreraoultii_genomic.fna_24:6181-8365 Satlength=2185 Nr of Repeats=42 RepeatLength=52 seed=TTCTATGAAC

Num.seqs=42 Similarity=0.829254
45   AACC-AC--TTTTCGCGAAATCCACATCATTTTCGGCTCATAGAACTC-TTCTATG
GCF_000752395.1_Bacillus_andreraoultii_genomic.fna_24:948-3548
Satlength=2601 Nr of Repeats=50 RepeatLength=52 seed=TTCTATGAAC
Num.seqs=50 Similarity=0.770286
45   AACC-AC--TTTTCGTGAAATCCNCATCATTTTNGGCTCATAGAACTC-TTCTATG
Rev.of_GCF_000752395.1_Bacillus_andreraoultii_genomic.fna_41:405982-406190 Satlength=209 Nr of Repeats=4 RepeatLength=52 seed=GGTTCATAGA
Num.seqs=4 Similarity=0.769392 56
AACCNAC--TTTTCGCGGAATCCCCATCATTTTCGGCTCATAGCACTC-TTCTATG
Rev.of_GCF_000752395.1_Bacillus_andreraoultii_genomic.fna_41:77609-78025
Satlength=417 Nr of Repeats=8 RepeatLength=52 seed=GGTTCATAGA Num.seqs=8
Similarity=0.812390 4 AACC--C-TTTTTTGCGGAATCCCAATCATTTTTGGCTCATAGANCAC-TTCTATG

**** * **** * * ***** ******* ********* * * ******
Consensus:

AACCaCTTTTCGCGaAATCCnCATCATTTTcGGCTCATAGaACTCTTCTATG

>Bacillus_Fam_84_53_6 Nr. of seq. 6 Alignment length(with gaps) = 56
Alignment score = 0.659127
GCF_000708755.2_ASM70875v2_genomic.fna_2:1053748-1054173 Satlength=426 Nr of Repeats=8 RepeatLength=53 seed=CTTTGCTTAC Num.seqs=7
Similarity=0.857442 0
CTTTGCTTACCCTGCTNACCCGTTTTT-TCCGCT-TTGCTGGGTAAGATTC-CTCC
GCF_000709935.2_ASM70993v2_genomic.fna_4:308519-308731 Satlength=213 Nr of Repeats=4 RepeatLength=53 seed=CTTTGCTTAC Num.seqs=4
Similarity=0.836478
0   CTTTGCTTACCCTGCTCACCCGTTTTT-TCCGCT-TTGCGGGGTAGGATTC-CTCC
GCF_000708755.2_ASM70875v2_genomic.fna_6:66915-67179 Satlength=265 Nr of Repeats=5 RepeatLength=53 seed=GGGTACGATT Num.seqs=4 Similarity=0.878407
38   CTTTGCTTACCCTGCCCATGCCGTTTT-T-CACTCTTGCTGGGTACGATTC-ATCC
Rev.of_GCF_000709935.2_ASM70993v2_genomic.fna_1:463602-463814
Satlength=213 Nr of Repeats=4 RepeatLength=53 seed=AGGGTAAGCA Num.seqs=4
Similarity=0.878407 66
TTTTGCTTACCCTCCCAATTCGGTTTT-T-CGCTCTTGCTGGGTACGATTC-CGGC
Rev.of_GCF_000708755.2_ASM70875v2_genomic.fna_22:9836-10048 Satlength=213
Nr of Repeats=4 RepeatLength=53 seed=AAAAACGGTT Num.seqs=4
Similarity=0.823899 26
-TTTGCTTACCCACCCCAACCGTTTTT-T-CGGTCTAGCTGGGTACGATTCACTCC
GCF_000709935.2_ASM70993v2_genomic.fna_5:333641-333853 Satlength=213 Nr of Repeats=4 RepeatLength=53 seed=TTTGCTTACC Num.seqs=4
Similarity=0.844864
54   -TTTGCTTACCCTCCACCACC-TGGTTTT-CGGCTTTGCTGGGTACGATTCACTCC

*********** * * ** * * * ** ***** ***** *
Consensus:

cTTTGCTTACCCTcCccAncCgtTTTTTCGcTcTTGCTGGGTAcGATTCCTCC

>Bacillus_Fam_85_52_6 Nr. of seq. 6 Alignment length(with gaps) = 55
Alignment score = 0.734949

GCF_000177235.2_ASM17723v2_genomic.fna_1:18889-19512 Satlength=624 Nr of Repeats=10 RepeatLength=52 seed=CACTCAACGA Num.seqs=7 Similarity=0.815403
0   CACTCAACGAAGGTAATCAT--AGTAAGCCAATGCTACCCCAAAA-CCAAACCCG
GCF_000177235.2_ASM17723v2_genomic.fna_1:2263203-2263619 Satlength=417 Nr of Repeats=8 RepeatLength=52 seed=AAGGTAATCA Num.seqs=8 Similarity=0.794974 9
CACTCAACAAAGGTAATCATA-AGCAAG-CAATGCTACCCCAAAA-CCAAACCGG
GCF_000177235.2_ASM17723v2_genomic.fna_1:44519-44779 Satlength=261 Nr of Repeats=5 RepeatLength=52 seed=CAATGCTACC Num.seqs=5 Similarity=0.752830
27   CACTCAACGAAGGTCATCATA-NGCAAG-CAATGCTACCCCAAAA-GCTAATCCT
GCF_000177235.2_ASM17723v2_genomic.fna_1:113351-113768 Satlength=418 Nr of Repeats=8 RepeatLength=52 seed=AAGGTCATCA Num.seqs=7 Similarity=0.714286
61   CACTCAACGAAGGTCATCATA-AGCAAG-CAATGCTNCCCCAAAA-CCAAANCCN
Rev.of_GCF_000177235.2_ASM17723v2_genomic.fna_1:2555929-2556137 Satlength=209 Nr of Repeats=4 RepeatLength=52 seed=AGCATTGCTT Num.seqs=4 Similarity=0.691468 34
CATTCAACGAAGGTCATCATAGAG-AAG-CAATGCTGCCCCAAAAGCCAAACCT-
Rev.of_GCF_000177235.2_ASM17723v2_genomic.fna_1:2730527-2730944 Satlength=418 Nr of Repeats=8 RepeatLength=52 seed=CTATGATGAC Num.seqs=7 Similarity=0.678424 74
CAATCCACGAAGGTCATCATAGAG-AAG-CAATGCTGCCCGAAAATCCAAAANN-

** ** ** ***** *****   * *** ******* *** **** * **
Consensus:

CAcTCAACGAAGGTcATCATAAGcAAGCAATGCTaCCCCAAAACCAAAcCcn

>Bacillus_Fam_86_53_6 Nr. of seq. 6 Alignment length(with gaps) = 55 Alignment score = 0.654545
GCF_001636335.1_ASM163633v1_genomic.fna_1:186192-186660 Satlength=469 Nr of Repeats=9 RepeatLength=52 seed=CTAGATTTTC Num.seqs=9 Similarity=0.938746
0   CTAGA-TTTTCTGTTCGTTCGGGGTTCAATAGACCT-TATAGAAGCCCCTTTCG-
GCF_001636335.1_ASM163633v1_genomic.fna_1:3084736-3085256 Satlength=521 Nr of Repeats=10 RepeatLength=52 seed=AAGACTCTTT Num.seqs=10 Similarity=0.920798 40
CTGGATTTTTCCG-TCGTTCGGGGTTCTATACACCT-TATTGAAGACTCTTTCG-
Rev.of_GCF_001636335.1_ASM163633v1_genomic.fna_1:1182339-1183064 Satlength=726 Nr of Repeats=14 RepeatLength=52 seed=TCAATAAGGT Num.seqs=12 Similarity=0.878399
40   C--GATTTTTCTGTTCATTCAGGGCTCTATACACCT-TATTGAAGACTCTTTCAA
GCF_001636335.1_ASM163633v1_genomic.fna_1:3149857-3150741 Satlength=885 Nr of Repeats=17 RepeatLength=52 seed=ATTGAGGACT Num.seqs=17 Similarity=0.854638 87
CT-GA-TTTTCTGTTCGTTCGGGCCTCTATACGGCT-GATTGAGGACTCTTTCAT
Rev.of_GCF_001636335.1_ASM163633v1_genomic.fna_2:190727-191455 Satlength=729 Nr of Repeats=14 RepeatLength=52 seed=AACGAAAAAA Num.seqs=14 Similarity=0.822203
13   CT-ATTTTTTC-GTTCGTTCGGGGTTCTAT-CTGCNCTATTGAGGACTCTTTCGC
Rev.of_GCF_001636335.1_ASM163633v1_genomic.fna_2:192943-193202 Satlength=260 Nr of Repeats=5 RepeatLength=52 seed=AAGAGTCCTC Num.seqs=4

Similarity=0.914530 48
CT-GTTTTTC-GTTCGTTCGGGGTTCTAT-CTGCTCTATTGAGGACTCTTCCGC

*   ***** * ** *** ** ** **   *   ** ** * * *** *
Consensus:

CTGatTTTTCtGTTCGTTCGGGGtTCTATaCacCTTATTGAaGACTCTTTCgn

>Bacillus_Fam_87_52_6 Nr. of seq. 6 Alignment length(with gaps) = 53
Alignment score = 0.628512
GCF_001636315.1_ASM163631v1_genomic.fna_1:2367649-2367858 Satlength=210
Nr of Repeats=4 RepeatLength=52 seed=TCTACGTTAC Num.seqs=3
Similarity=0.717172 0
TCTACGTTACCCAA-GCC-CTATTTTTCCTCCGNTTCGGGCACCTAGAACCCC
GCF_001636315.1_ASM163631v1_genomic.fna_1:247094-247354 Satlength=261 Nr
of Repeats=5 RepeatLength=52 seed=AACCTCTCTA Num.seqs=5
Similarity=0.910256
46   TCTACGTGACCACACGAC-CACATTTTCATCCGGTTCGGTCACGTAGAACCTC
GCF_001636315.1_ASM163631v1_genomic.fna_1:263113-263529 Satlength=417 Nr
of Repeats=8 RepeatLength=52 seed=ACCTTTTTCT Num.seqs=8
Similarity=0.888278
70   TCTACGTGACCCAACATC-ACCTTTTTCTTCNGCTACGGTCACGTAGAACCTC
GCF_001636315.1_ASM163631v1_genomic.fna_1:4729327-4729587 Satlength=261
Nr of Repeats=5 RepeatLength=52 seed=TCTACGTGAC Num.seqs=5
Similarity=0.871795 104
TCTACGTGACCGAACAAC-CCCTTTTTCTTCGGCTACGGTCACGTAGAACCTC
GCF_000612665.1_FF4_genomic.fna_50:1971-2335 Satlength=365 Nr of
Repeats=6 RepeatLength=52 seed=TGTTTTCGGT Num.seqs=5 Similarity=0.897436
29   TCTACGTGACCCATTTTCATTCTTTCTTCT-GTTTTCGGTCGCGTAGAACCCC
GCF_000612665.1_FF4_genomic.fna_72:27-391 Satlength=365 Nr of Repeats=7
RepeatLength=52 seed=TCTACGTGAC Num.seqs=7 Similarity=0.823300
53   TCTACGTGACCC-GTGTCACTGGTTTTTATCCATTTCGGTCGCGTAGAACCTC

******* ***   *   ** * *   * *** * * ******* *
Consensus:

TCTACGTGACCcaacgtCctctTTTTcnTCcgnTtCGGTCaCGTAGAACCtC

>Bacillus_Fam_88_52_6 Nr. of seq. 6 Alignment length(with gaps) = 53
Alignment score = 0.782809
GCF_001375535.1_Bacillus_niameyensis_genomic.fna_6:97548-97860
Satlength=313 Nr of Repeats=6 RepeatLength=52 seed=GTCAATCGTT Num.seqs=6
Similarity=0.803145 0
GTCAATCGTTACCGTTTCACAGTAGATCAACTCATTACGGGCACGATTC-ACC
GCF_001375535.1_Bacillus_niameyensis_genomic.fna_6:2964303-2964563
Satlength=261 Nr of Repeats=5 RepeatLength=52 seed=CAATCGTTAC Num.seqs=5
Similarity=0.928205 2
GTCAATCGTTACCGTTTCACAGAAGATCAGCTCATTACGGGCACGATTC-AGT
Rev.of_GCF_001375535.1_Bacillus_niameyensis_genomic.fna_6:3681028-3687820
Satlength=6793 Nr of Repeats=123 RepeatLength=52 seed=AATGAGTTGA
Num.seqs=122 Similarity=0.935809 36
GTCAATCGTTACCGTTTCACAGAAGTTCAACTCATTATGGGCACGATTC-AGT
Rev.of_GCF_001375535.1_Bacillus_niameyensis_genomic.fna_6:3691066-3691326
Satlength=261 Nr of Repeats=5 RepeatLength=52 seed=GAATCGTGCC Num.seqs=5

Similarity=0.900000 49
GTCAATCGTTACCGTTTCACAGAAGTTCAACTCATTATGGGCACGATTC-AGT
GCF_001375535.1_Bacillus_niameyensis_genomic.fna_6:1865881-1866297
Satlength=417 Nr of Repeats=8 RepeatLength=52 seed=AGAAGATCAG Num.seqs=8
Similarity=0.939560 20
GTCAATCGTTACCATTTCACAGAAGATCAGTGCATTACGGGCACGATTC-AGC
Rev.of_GCF_001375535.1_Bacillus_niameyensis_genomic.fna_6:3933103-3935130
Satlength=2028 Nr of Repeats=39 RepeatLength=52 seed=GAATCGTTCC
Num.seqs=38 Similarity=0.998650 48 CT-
TATCGTTACCGTTTTTCCGGAGTTCATCACATAACGGGAACGATTCAAGA

* ********* *** * * ** ***   *** * *** ******* *
Consensus:

GTCAATCGTTACCGTTTCACAGaAGaTCAaCtCATTAcGGGCACGATTCAGt

>Bacillus_Fam_89_53_6 Nr. of seq. 6 Alignment length(with gaps) = 53
Alignment score = 0.780503
GCF_001439965.1_ASM143996v1_genomic.fna_104:39216-39670 Satlength=455 Nr of Repeats=9 RepeatLength=52 seed=AGCAAAAATG Num.seqs=7
Similarity=0.803235
0 AG-CAAAAATGTGTGTCCAAGGCGGAAGTGGAGACACATTACGAAGGAAAAAC
GCF_001887185.1_ASM188718v1_genomic.fna_175:122798-123214 Satlength=417 Nr of Repeats=8 RepeatLength=52 seed=AGTGGAGACA Num.seqs=8
Similarity=0.709821 25
AG-CAACAATGTGTGTCCAAGGCGNAAGTGGAGACACATGACGAAGGAAAAAC
GCF_002009555.1_ASM200955v1_genomic.fna_90:39540-39956 Satlength=417 Nr of Repeats=8 RepeatLength=52 seed=AGTGGAGACA Num.seqs=8
Similarity=0.709821
25 AG-CAACAATGTGTGTCCAAGGCGNAAGTGGAGACACATGACGAAGGAAAAAC
GCF_001439965.1_ASM143996v1_genomic.fna_125:141897-142307 Satlength=411 Nr of Repeats=8 RepeatLength=52 seed=AAAATGTGTG Num.seqs=7
Similarity=0.755189 5
GGTCAAAAATGTGTGTCCAAGGNGAGAGTGGAGACAGATAATGAGGAAAAAA-
Rev.of_GCF_002009555.1_ASM200955v1_genomic.fna_334:40008-40529
Satlength=522 Nr of Repeats=10 RepeatLength=52 seed=TGTCTCCACT
Num.seqs=9 Similarity=0.772792
36 GGTCAAAAATGTGTGTCAGAGGNGAGAGTGGAGACAGATAATGAGGAAAAAA-
GCF_001887185.1_ASM188718v1_genomic.fna_106:36381-37057 Satlength=677 Nr of Repeats=13 RepeatLength=52 seed=AGTGGAGACA Num.seqs=13
Similarity=0.812623 26
GGNCAAAAATGTGTGTCCAAGGCAAGAGTGGAGACAGATAATGAGGGAAAAA-

* *** ********** ***   ********** ** * ** * *****
Consensus:

aGnCAAaAATGTGTGTCCAAGGcGaaAGTGGAGACAcATaAcGAaGgAAAAAc

>Bacillus_Fam_90_42_6 Nr. of seq. 6 Alignment length(with gaps) = 42
Alignment score = 0.812169
GCF_000007825.1_ASM782v1_genomic.fna_1:4624384-4624759 Satlength=376 Nr of Repeats=9 RepeatLength=42 seed=TCTTCCACTT Num.seqs=8
Similarity=0.875283
0 TCTTCCACTTGTGGTTCTACTACCACCTGCTGCACTGGGCGT

GCF_000831065.1_ASM83106v1_genomic.fna_1:4382555-4382849 Satlength=295 Nr of Repeats=7 RepeatLength=42 seed=TCTTCCACTT Num.seqs=7 Similarity=0.860922 0
TCTTCCACTTGTGGTTCTGCTACTACTTGCTGCACTGGGTGC
GCF_000007845.1_ASM784v1_genomic.fna_1:4485994-4486366 Satlength=373 Nr of Repeats=9 RepeatLength=42 seed=TCTTCCACTT Num.seqs=7 Similarity=0.900227
0 TCTTCCACTTGTGGTTCCACTACTACTTGTTGCATTGGTTTT
Rev.of_GCF_000290995.1_Baci_cere_AND1407_G13175_V1_genomic.fna_2:177294-177504 Satlength=211 Nr of Repeats=5 RepeatLength=42 seed=GAACCACAAG Num.seqs=5 Similarity=0.892063 17
TCTTCCACTTGTGGTTCCACTACTACTTGCTGCACTGGTTTT
Rev.of_GCF_000832605.1_ASM83260v1_genomic.fna_1:1288569-1288821 Satlength=253 Nr of Repeats=6 RepeatLength=42 seed=TGCAGCAAGT Num.seqs=6 Similarity=0.833850 34
TCTTCCACTTGNGGTTCTACTACCACTTGCTGCATCGGTTTT
Rev.of_GCF_000775975.1_ASM77597v1_genomic.fna_1:181687-181855 Satlength=169 Nr of Repeats=4 RepeatLength=42 seed=TGCAGCAAGT Num.seqs=4 Similarity=0.825397 34
TCTTCTGCTTGTGGTTCTACTACCACTTGCTGCATTGGCTTT

***** **** ***** **** ** ** **** **
Consensus:

TCTTCCACTTGTGGTTCtACTACcACTTGCTGCAcTGGtTtT

>Bacillus_Fam_91_20_6 Nr. of seq. 6 Alignment length(with gaps) = 22 Alignment score = 0.625758
GCF_001712755.1_ASM171275v1_genomic.fna_4:95498-95778 Satlength=281 Nr of Repeats=14 RepeatLength=20 seed=AATTTAATTG Num.seqs=14 Similarity=0.761120
8 ATACCCGGAA-TTTAATTGC-G
GCF_001712755.1_ASM171275v1_genomic.fna_4:102562-102861 Satlength=300 Nr of Repeats=14 RepeatLength=20 seed=GAAATTAATT Num.seqs=13 Similarity=0.916239 27
ATTCACGGAA-ATTAATTGC-G
Rev.of_GCF_001712755.1_ASM171275v1_genomic.fna_32:127615-127715 Satlength=101 Nr of Repeats=5 RepeatLength=20 seed=GCAATTAAAT Num.seqs=3 Similarity=0.866667 19
ATTCTCTTAA-TTTAATTGC-G
GCF_001712755.1_ASM171275v1_genomic.fna_2:242324-242407 Satlength=84 Nr of Repeats=4 RepeatLength=21 seed=TTTAATTGCA Num.seqs=3 Similarity=0.626263
31 ATTCNCTAGA-TTTAATTGCAG
Rev.of_GCF_001712755.1_ASM171275v1_genomic.fna_13:327923-328123 Satlength=201 Nr of Repeats=10 RepeatLength=20 seed=CGCAATTAAA Num.seqs=8 Similarity=0.668831
41 ATTCTCTAGATTTTAATTGC-G
Rev.of_GCF_001712755.1_ASM171275v1_genomic.fna_32:95561-95681 Satlength=121 Nr of Repeats=6 RepeatLength=20 seed=GCAATTAAAA Num.seqs=4 Similarity=0.753968 19
GTTTTCGG-ATTTTAATTGC-G

* ******** * * *

Consensus:

aATTTAATTGCGATTCtCgg

>Bacillus_Fam_92_21_6 Nr. of seq. 6 Alignment length(with gaps) = 22
Alignment score = 0.633333
GCF_000175075.1_ASM17507v1_genomic.fna_2:18020-18125 Satlength=106 Nr of Repeats=5 RepeatLength=21 seed=AAGTCGGAAA Num.seqs=5 Similarity=0.828571
0 AAGTCGGAAA-TCGGCGGAATA
GCF_000307875.1_BABA1.0_genomic.fna_123:41009-41093 Satlength=85 Nr of Repeats=4 RepeatLength=21 seed=TTGGCGGAAT Num.seqs=4 Similarity=0.506667
10 AACTAGAAAG-TTGGCGGAATA
Rev.of_GCF_001591445.1_ASM159144v1_genomic.fna_38:10093-10301 Satlength=209 Nr of Repeats=7 RepeatLength=21 seed=ATTCCGCCAA Num.seqs=5 Similarity=0.556944 20
AACTAGTAAN-TTGGCGGAATA
GCF_001591665.1_ASM159166v1_genomic.fna_21:145523-145647 Satlength=125 Nr of Repeats=4 RepeatLength=21 seed=TTGGCGGAAT Num.seqs=3 Similarity=0.516908 10
AACT-CAAANNTTGGCGGAATN
GCF_001591665.1_ASM159166v1_genomic.fna_16:71486-71593 Satlength=108 Nr of Repeats=5 RepeatLength=21 seed=TTGGCGGAAT Num.seqs=4 Similarity=0.585648
10 AAAGTCAAAN-TTGGCGGAATA
GCF_001591665.1_ASM159166v1_genomic.fna_21:87303-87605 Satlength=303 Nr of Repeats=11 RepeatLength=21 seed=TTGGCGGAAT Num.seqs=10 Similarity=0.478905 10
AANGNANAAG-TTGGCGGAATA

**   ** * ********
Consensus:

AActngaAAnTTGGCGGAATA

>Bacillus_Fam_93_21_6 Nr. of seq. 6 Alignment length(with gaps) = 22
Alignment score = 0.603030
GCF_001889165.1_ASM188916v1_genomic.fna_1:473933-474227 Satlength=295 Nr of Repeats=14 RepeatLength=21 seed=ATCGCGGAAA Num.seqs=14 Similarity=0.849294 0
ATCGCG-GAAAGGAGTGCCGGA
GCF_001889165.1_ASM188916v1_genomic.fna_1:757324-757654 Satlength=331 Nr of Repeats=6 RepeatLength=21 seed=GAATCGCGGA Num.seqs=5 Similarity=0.873016
19 ATCGCG-GAAAGAGGTGCCGGA
Rev.of_GCF_001889165.1_ASM188916v1_genomic.fna_1:3509098-3509350 Satlength=253 Nr of Repeats=12 RepeatLength=21 seed=TCCGCGATTA Num.seqs=12 Similarity=0.755599
29 ATCGCG-GAAAGAGGTGCCGTA
GCF_001889165.1_ASM188916v1_genomic.fna_1:684231-684378 Satlength=148 Nr of Repeats=7 RepeatLength=21 seed=GAATCGCGGA Num.seqs=7 Similarity=0.927438
19 ATCGCG-GAAAGAGAGGTCGGA
Rev.of_GCF_001889165.1_ASM188916v1_genomic.fna_1:3632787-3633039 Satlength=253 Nr of Repeats=12 RepeatLength=21 seed=TCCGCGATTC

Num.seqs=12 Similarity=0.772957
8   ATCGCG-GATAGGAGNGTCGGA
Rev.of_GCF_001889165.1_ASM188916v1_genomic.fna_1:3348404-3348982
Satlength=579 Nr of Repeats=18 RepeatLength=21 seed=CTTTCCGCGA
Num.seqs=16 Similarity=0.700116
3   AAGGGGTGATGGAATCG-CGGA

* * * ** *   * ** *
Consensus:

ATCGCGGAaAGaagtGcCGGA

>Bacillus_Fam_94_21_6 Nr. of seq. 6 Alignment length(with gaps) = 21
Alignment score = 0.647619
GCF_000508325.1_BAVI_1_genomic.fna_103:54980-55146 Satlength=167 Nr of
Repeats=7 RepeatLength=21 seed=ATTTCGCCAA Num.seqs=6 Similarity=0.517460
0   ATTTCGCCAAACTNACGNTTA
GCF_001591805.1_ASM159180v1_genomic.fna_7:208460-208544 Satlength=85 Nr
of Repeats=4 RepeatLength=21 seed=TTATTTCGCC Num.seqs=4
Similarity=0.640212
19   ATTTCGCCAAACTCAGGATTT
GCF_001591805.1_ASM159180v1_genomic.fna_5:205275-205400 Satlength=126 Nr
of Repeats=6 RepeatLength=21 seed=ATTTCGCCAA Num.seqs=5
Similarity=0.822222
0   ATTTCGCCAAACCNAGCAATA
GCF_001591445.1_ASM159144v1_genomic.fna_22:80175-80280 Satlength=106 Nr
of Repeats=5 RepeatLength=21 seed=ATTCCGCCAA Num.seqs=5
Similarity=0.473611
0   ATTCCGCCAAATNNCNGATTT
Rev.of_GCF_001591665.1_ASM159166v1_genomic.fna_21:177804-177908
Satlength=105 Nr of Repeats=5 RepeatLength=21 seed=TTGGCGGAAT Num.seqs=3
Similarity=0.727273   10
ATTCCGCCAAACTTCGGATTA
Rev.of_GCF_001591445.1_ASM159144v1_genomic.fna_25:3465-3548 Satlength=84
Nr of Repeats=4 RepeatLength=21 seed=TTGGCGGAAT Num.seqs=3
Similarity=0.430556   10
ATTCCGCCAANCTNGCNATTT

*** ******   *
Consensus:

ATTcCGCCAAACtnaggATTa

>Bacillus_Fam_95_20_6 Nr. of seq. 6 Alignment length(with gaps) = 20
Alignment score = 0.731111
GCF_000374565.1_ASM37456v1_genomic.fna_3:124095-124776 Satlength=682 Nr
of Repeats=32 RepeatLength=20 seed=ATACTCTTCA Num.seqs=22
Similarity=0.695355   0
ATACTCTTCACATTTGCGAA
GCF_000374565.1_ASM37456v1_genomic.fna_18:12540-13648 Satlength=1109 Nr
of Repeats=51 RepeatLength=20 seed=ATACTCTTCA Num.seqs=33
Similarity=0.575442   0
ATACTCTTCANAATTCAGAA

Rev.of_GCF_000374565.1_ASM37456v1_genomic.fna_4:170298-171100 Satlength=803 Nr of Repeats=32 RepeatLength=20 seed=AAGAGTATTT Num.seqs=23 Similarity=0.639118
8   ATACTCTTCACTTTCAAGNA
Rev.of_GCF_000374565.1_ASM37456v1_genomic.fna_30:38245-38545 Satlength=301 Nr of Repeats=14 RepeatLength=20 seed=AAGTGAAGAG Num.seqs=13 Similarity=0.779487
13   ATACTCTTCACTTTCGGGAA
GCF_000374565.1_ASM37456v1_genomic.fna_34:28543-28664 Satlength=122 Nr of Repeats=5 RepeatLength=20 seed=TACTCTTCAC Num.seqs=3 Similarity=0.777778
21   TTACTCTTCACTTTCGAGAA
Rev.of_GCF_000374565.1_ASM37456v1_genomic.fna_38:0-139 Satlength=140 Nr of Repeats=7 RepeatLength=20 seed=AAGTGAAGAG Num.seqs=7 Similarity=0.888889
13   ATACTCTTCACTTTCCGCAA

*********   *   *
Consensus:

ATACTCTTCACtTTcgaGAA

>Bacillus_Fam_96_15_6 Nr. of seq. 6 Alignment length(with gaps) = 15 Alignment score = 0.751111
GCF_000007825.1_ASM782v1_genomic.fna_1:953796-953898 Satlength=103 Nr of Repeats=4 RepeatLength=15 seed=TAAACAAGAA Num.seqs=3 Similarity=0.644444
0   TAAACAAGAAAATAC
GCF_000831065.1_ASM83106v1_genomic.fna_1:794707-794809 Satlength=103 Nr of Repeats=4 RepeatLength=15 seed=TAAACAAGAA Num.seqs=3 Similarity=0.644444
0   TAAACAAGAAAATAC
Rev.of_GCF_000831065.1_ASM83106v1_genomic.fna_2:182684-182786 Satlength=103 Nr of Repeats=4 RepeatLength=15 seed=TTCTTGTTTA Num.seqs=3 Similarity=0.644444   10
TAAACAAGAAAATAC
GCF_000225265.1_ASM22526v1_genomic.fna_1:1128295-1128356 Satlength=62 Nr of Repeats=4 RepeatLength=15 seed=AAAAGAAAAA Num.seqs=3 Similarity=0.847222
3   TGAAAAAGAAAAAGC
GCF_000474275.1_Bmar1.0_genomic.fna_8:157466-157541 Satlength=76 Nr of Repeats=5 RepeatLength=15 seed=AAATAGAAAA Num.seqs=5 Similarity=1.000000
17   TAAAATAGAAAAAGC
Rev.of_GCF_000712615.1_ASM71261v1_genomic.fna_2:274011-274086 Satlength=76 Nr of Repeats=5 RepeatLength=15 seed=TCTTCTTTTT Num.seqs=5 Similarity=0.822222   12
TAAAAAAGAAGAAGC

* ** **** * *
Consensus:

TAAAaAAGAAAAaaC

>Bacillus_Fam_97_12_6 Nr. of seq. 6 Alignment length(with gaps) = 12 Alignment score = 0.807407

GCF_000311725.1_ASM31172v1_genomic.fna_9:43921-43981 Satlength=61 Nr of Repeats=5 RepeatLength=12 seed=GGTTCTGGCT Num.seqs=5 Similarity=1.0000000   GGTTCTGGCTTT
Rev.of_GCF_001700275.1_ASM170027v1_genomic.fna_45:377163-377241 Satlength=79 Nr of Repeats=4 RepeatLength=12 seed=CCAAAGCCAG Num.seqs=3 Similarity=1.000000   2
GGTTCTGGCTTT
Rev.of_GCF_001439635.1_ASM143963v1_genomic.fna_175:44104-44236 Satlength=133 Nr of Repeats=9 RepeatLength=12 seed=GAACCAGAAC Num.seqs=6 Similarity=0.814815   5
GGTTCTGGTTTT
GCF_000972245.2_ASM97224v2_genomic.fna_1:3422882-3423134 Satlength=253 Nr of Repeats=21 RepeatLength=12 seed=GGATTTGGTT Num.seqs=21 Similarity=0.971429   6
GGTTCTGGATTT
Rev.of_GCF_001742425.1_ASM174242v1_genomic.fna_25:16787-17924 Satlength=1138 Nr of Repeats=16 RepeatLength=12 seed=CCAGAACCAA Num.seqs=10 Similarity=0.775309
8   GGTTCTGGTTTT
Rev.of_GCF_000612805.1_FF3_genomic.fna_1:431588-431636 Satlength=49 Nr of Repeats=4 RepeatLength=12 seed=CCAGAGCCAA Num.seqs=4 Similarity=0.888889
2   GGCTTTGGCTCT

** * *** * *
Consensus:

GGTTCTGGcTTT

>Bacillus_Fam_98_52_5 Nr. of seq. 5 Alignment length(with gaps) = 61 Alignment score = 0.610929
GCF_000186145.1_Bacillus_sp_2_A_57_CT2_V1_genomic.fna_2:72771-73392 Satlength=622 Nr of Repeats=12 RepeatLength=52 seed=TCATGGCGAC Num.seqs=10 Similarity=0.852422   78
TNTCA-ATTTT-CGGCTT-CATG-AA-CCC-TTCATGGCGACCA--TTTTCT-TCTCTGGT
Rev.of_GCF_000186145.1_Bacillus_sp_2_A_57_CT2_V1_genomic.fna_9:73399-73710 Satlength=312 Nr of Repeats=6 RepeatLength=52 seed=GAAAAATGGT Num.seqs=5 Similarity=0.848718   95   TC-CTGATTTT-CGGCTT-CATG-AAGCGC-T-CATGGCGACCAT-TTTTCT--CTTTGCT
Rev.of_GCF_000294775.2_ASM29477v2_genomic.fna_1:1168663-1168919 Satlength=257 Nr of Repeats=5 RepeatLength=52 seed=GAAAAATGGT Num.seqs=3 Similarity=0.811966   95   TC-CTGATTTT-CGGCTT-CATG-AAGTGC-T-CATGGCGACCAT-TTTTCT--CTTTGCT
Rev.of_GCF_000294775.2_ASM29477v2_genomic.fna_1:900085-900448 Satlength=364 Nr of Repeats=7 RepeatLength=52 seed=TGAAGCCGAA Num.seqs=6 Similarity=0.810256   121
TC-CT--TTTT-CGGCTT-CATG--AGAGCTT-CATGGCGACCAT-TTTTCTGGCTCTGGT
Rev.of_GCF_000186145.1_Bacillus_sp_2_A_57_CT2_V1_genomic.fna_4:197835-198199 Satlength=365 Nr of Repeats=7 RepeatLength=52 seed=ATGAAGCCGA Num.seqs=7 Similarity=0.710758   83
TTTCT---TATCGCTNC-ACG-ACTTTTCGGCTTCATG-ACTGC-CT-CATGGCGACCG-T

* ** *   * *   *   *   *
Consensus:

tttctnctctGnTTcCtatTtTcggcttCatgaagngctcatgGcgaccatT

>Bacillus_Fam_99_61_5 Nr. of seq. 5 Alignment length(with gaps) = 61
Alignment score = 0.663934
GCF_000161455.1_ASM16145v1_genomic.fna_1:4024244-4024484 Satlength=241 Nr of Repeats=4 RepeatLength=60 seed=CCTGTTTGCA Num.seqs=4 Similarity=0.752205 0
CCTGTTTGCATTGCTCCGCTTCCGTTGAAGTAGTACCATGTACCG-TTAATTGCTTGCCAG
Rev.of_GCF_000496285.1_ASM49628v1_genomic.fna_1:4380225-4380585 Satlength=361 Nr of Repeats=6 RepeatLength=60 seed=GCGATGCAAA Num.seqs=6 Similarity=0.896296 14
CCTGTTTGCATCGCTCCGCTTCCGTTGAAGTAATACCATGCTCCGTTTACCTGC-TGCCAA
GCF_000712595.1_ASM71259v1_genomic.fna_14:75165-75465 Satlength=301 Nr of Repeats=5 RepeatLength=60 seed=TTTGCATTGC Num.seqs=5 Similarity=0.806557
4 CCTTTTTGCATTGCACCGTTTCCATTAAAGTAGTACCATGCTCCATTTA-TTGCTTGCCAA
Rev.of_GCF_000712595.1_ASM71259v1_genomic.fna_10:96270-96750 Satlength=481 Nr of Repeats=8 RepeatLength=60 seed=ACATGGTACT Num.seqs=8 Similarity=0.801587 41
CCTGTTTGCATTGTACCGTTTTCGTTTAAGTAGTACCATGTTCCACCAATTTG-TTTCCAT
Rev.of_GCF_000496285.1_ASM49628v1_genomic.fna_1:3570725-3571145 Satlength=421 Nr of Repeats=7 RepeatLength=60 seed=TGGCAATCAA Num.seqs=7 Similarity=0.880423 59
CCNGTTAACATTGCACCTTCACCATTTAAGTAGTACCATTTACCACCTA-TTGATTGCCAA

** ** *** * **   * ** ***** ******   **   * ** * ***
Consensus:

CCtgTTtgCATtGcaCCgtttcCgTTnAAGTAgTACCATgttCCanttAntTGctTgCCAa

>Bacillus_Fam_100_52_5 Nr. of seq. 5 Alignment length(with gaps) = 59
Alignment score = 0.628531
GCF_000759675.1_ASM75967v1_genomic.fna_48:7-215 Satlength=209 Nr of Repeats=4 RepeatLength=52 seed=AGAGTGTGCC Num.seqs=4 Similarity=0.888889
0 AG-AGTGTGCCTAGTGACC-AAAAG---TAAGAT-AGGAATCCATAGAAGTGGTCACA-
GCF_000759675.1_ASM75967v1_genomic.fna_150:19-279 Satlength=261 Nr of Repeats=5 RepeatLength=52 seed=GTGACCAAAA Num.seqs=5 Similarity=0.866667
12 AG-AGTGCGCCTAGTGACC-AAAAG---TAAGCT-CGGAATCCATAGAAGTGGTCACA-
Rev.of_GCF_000759675.1_ASM75967v1_genomic.fna_164:20-228 Satlength=209 Nr of Repeats=4 RepeatLength=52 seed=ACCACTTCTA Num.seqs=4 Similarity=0.850427 48
AG-AGTGTGTCTTGTGACC-AAAAG---TAAGCT-CGGAATCCATAGAAGTGGTCACA-
Rev.of_GCF_000759675.1_ASM75967v1_genomic.fna_151:544090-544350 Satlength=261 Nr of Repeats=5 RepeatLength=52 seed=CTTGTGACCA Num.seqs=5 Similarity=0.879487 2
AGAAGTGA-CCTTGTGACC-AAACGCTGTAA--T--TGAAATCATAAAAGTGGTCACA-
Rev.of_GCF_000759675.1_ASM75967v1_genomic.fna_154:26046-26254 Satlength=209 Nr of Repeats=4 RepeatLength=52 seed=ATGTGACCAC Num.seqs=4 Similarity=0.829060 52
AG-AGCGT-CCTTGTGACCAAAAAGC---AA--TGGTGATTTCATCAAAGTGGTCACAT

** ** *   ** ****** *** *   ** *   **   *** ***********
Consensus:

AGAGtGtgcCTtGTGACCAAAaGtAAgnTngGAatcCATagAAGTGGTCACA

>Bacillus_Fam_101_51_5 Nr. of seq. 5 Alignment length(with gaps) = 58 Alignment score = 0.637356
GCF_000813125.1_ASM81312v1_genomic.fna_86:7113-7633 Satlength=521 Nr of Repeats=10 RepeatLength=52 seed=GGAATCGTGA Num.seqs=10 Similarity=0.880342
5 ACCTCGGAATCGTGACAGGTTTGGGTGT----G-AAA-GCCGAAAAGCTGTCAAGAAA
Rev.of_GCF_000813125.1_ASM81312v1_genomic.fna_129:103315-103679 Satlength=365 Nr of Repeats=7 RepeatLength=52 seed=TTTTCTTGAC Num.seqs=7 Similarity=0.802198 53
AGCCCGGAATCGTGACAGGTTNGGCAGT----G-AAA-GCCGAAAAGCTGTCAAGAAA
GCF_001315165.1_ASM131516v1_genomic.fna_31:17940-18200 Satlength=261 Nr of Repeats=5 RepeatLength=52 seed=AAAGCTGTCA Num.seqs=5 Similarity=0.887179
37 AGCACGGAATTATGACAGGTTT-GAAGTG---G-AAA-GCGGAAAAGCTGTCAAGAAA
Rev.of_GCF_000813125.1_ASM81312v1_genomic.fna_134:126961-127481 Satlength=521 Nr of Repeats=9 RepeatLength=52 seed=CCAAAGCTGT Num.seqs=8 Similarity=0.808625 24
ACGAAGGAATTATGACAGCTTT-GGAGT----GTAAATGAAGAAAAGCTGTCAAGAAA
Rev.of_GCF_000813125.1_ASM81312v1_genomic.fna_122:32607-32815 Satlength=209 Nr of Repeats=4 RepeatLength=52 seed=TTTCTTGACA Num.seqs=4 Similarity=0.858974 52
ACAAAGGAATTGTGACAGGTAT-GAGGT-TCTG---A-GACCAAAAGCTGTCAAGAAA

***** ****** * * ** * * * ****************
Consensus:

GGAATtgTGACAGgTttGnaGTGaaAGccgAAAAGCTGTCAAGAAAAccac

>Bacillus_Fam_102_52_5 Nr. of seq. 5 Alignment length(with gaps) = 58 Alignment score = 0.601149
GCF_001636315.1_ASM163631v1_genomic.fna_1:3520892-3521308 Satlength=417 Nr of Repeats=8 RepeatLength=52 seed=AGCCTATTGG Num.seqs=8 Similarity=0.868486 0
-AGCCTATTGGCGACATTTCTCTTAG-C-AAAAA--CAACAAAAGTGTTTCCAATAA-
Rev.of_GCF_001636315.1_ASM163631v1_genomic.fna_1:3785428-3785688 Satlength=261 Nr of Repeats=5 RepeatLength=52 seed=TTGGAAACAC Num.seqs=5 Similarity=0.820513 49 -
GGCCTATTGGCGACATTTCTCTTAG-C-AAAAG--CAACAAAAGTGTTTCCAATGG-
Rev.of_GCF_001636315.1_ASM163631v1_genomic.fna_1:3568862-3569174 Satlength=313 Nr of Repeats=6 RepeatLength=52 seed=ATAGAAATGT Num.seqs=6 Similarity=0.849573 21 -AG-
CTATTGGCGACATTTCTATT---CAAAAAGT-CAACATAAGTGTTTCCAATGGC
Rev.of_GCF_001636315.1_ASM163631v1_genomic.fna_1:3535170-3535534 Satlength=365 Nr of Repeats=7 RepeatLength=52 seed=GTTGTAATTT Num.seqs=7 Similarity=0.894994 35 CGG-
CTATTGCCGACAATACT-CTAG---AAAATTACAACCAAAGTGTTTCCAATGG-
Rev.of_GCF_001636315.1_ASM163631v1_genomic.fna_1:3851718-3851925 Satlength=208 Nr of Repeats=4 RepeatLength=52 seed=CTATTGGAAA Num.seqs=3 Similarity=0.897436 52 CCG-
GTATTGGCGACAATACT-TGAGTT-AAAATTGGA--CAAAGTGTTTCCAATAG-

* ***** ***** * **   ****   *   *************
Consensus:

nGcTATTGgCGACAtTtCTnttagcAAAAntcAacaaAAGTGTTTCCAATgg

>Bacillus_Fam_103_53_5 Nr. of seq. 5 Alignment length(with gaps) = 57
Alignment score = 0.626901
GCF_000708755.2_ASM70875v2_genomic.fna_1:37544-37809 Satlength=266 Nr of Repeats=4 RepeatLength=53 seed=CACTCGGTGT Num.seqs=3 Similarity=0.730453
0   CACTCGGTGT-AGTGGCGCCACAAACAAGG-NTCCAAGT-NCGAG-AAAAGTGGTAT
GCF_000709935.2_ASM70993v2_genomic.fna_1:37723-37935 Satlength=213 Nr of Repeats=4 RepeatLength=53 seed=CACTCGGTGT Num.seqs=4 Similarity=0.715278
0   CACTCGGTGT-AGTGGCGCCACAAACAAGG-ATCCAAGT-GCAGG-AAAAGTGGCAT
Rev.of_GCF_000708755.2_ASM70875v2_genomic.fna_2:860672-860884
Satlength=213 Nr of Repeats=4 RepeatLength=53 seed=TCATTTGTGG Num.seqs=4
Similarity=0.790356   27
CACTCTGGGT-AGTGGTGCCACAAATGAGGTAAAC---TCACGAGAAAAAGTGGCAG
Rev.of_GCF_000709935.2_ASM70993v2_genomic.fna_4:114946-115370
Satlength=425 Nr of Repeats=8 RepeatLength=53 seed=CTGCCACTTT Num.seqs=6
Similarity=0.817191   53
CACTCGGGGT-AATGGTGCCACAAATGAGGTAAAC---TCACGAGAAAAAGTGGCAG
Rev.of_GCF_000709935.2_ASM70993v2_genomic.fna_5:581943-582154
Satlength=212 Nr of Repeats=4 RepeatLength=53 seed=CTGCCACTTT Num.seqs=3
Similarity=0.714885   53
CACTC-GAGTGAGTGGCGCCAGAAAAGAGG-ATCC-A-TCACCTGAAAAAGTGGCAG

***** * ** * *** **** *** ***   *   * * * ******** *
Consensus:

CACTCgGnGTAgTGGcGCCAcAAAngAGGatcCaTcaCgaGaAAAAGTGGcAg

>Bacillus_Fam_104_52_5 Nr. of seq. 5 Alignment length(with gaps) = 56
Alignment score = 0.618750
GCF_000813125.1_ASM81312v1_genomic.fna_40:52980-53241 Satlength=262 Nr of Repeats=5 RepeatLength=52 seed=ATGACGGACA Num.seqs=4 Similarity=0.852201
0   ATGACGGACACTTTTCCTC--TGT-TTCCTTCGATTTCTGTCCGTCATG-GCCTTG
GCF_000813125.1_ASM81312v1_genomic.fna_88:27406-27823 Satlength=418 Nr of Repeats=8 RepeatLength=52 seed=GATGACGGAC Num.seqs=7 Similarity=0.837607
51   ATGACGGACAGTTTT-NTCGAT-T-TTCCATCGATTTGTGTCCGTCATG-GCCTTG
GCF_000813125.1_ASM81312v1_genomic.fna_75:30268-30683 Satlength=416 Nr of Repeats=8 RepeatLength=52 seed=CTGTCCGTCA Num.seqs=7 Similarity=0.828092
34   ATGACGGACACTTTT-C-CGGNAC-TTCTCTAATTTTCTGTCCGTCATG-ACCTTG
GCF_000813125.1_ASM81312v1_genomic.fna_122:22295-22555 Satlength=261 Nr of Repeats=5 RepeatLength=52 seed=GATGACGGAC Num.seqs=5
Similarity=0.798148
51   ATGACGGACACTTTT--TC-ATGAGTTCACGNGATTTCTGTCCGTCATA-GCCTNG
Rev.of_GCF_001315165.1_ASM131516v1_genomic.fna_3:27965-28173
Satlength=209 Nr of Repeats=4 RepeatLength=52 seed=ATGACGGACA Num.seqs=4
Similarity=0.839099   96
ATGACGGACACTTTT--GC-TTCTGTTTCGGTGATTTCTGTCCGTCATACCCCTT-

********* ****   *   **   *** *********   ***
Consensus:

ATGACGGACAcTTTTntCntntTTccntngaTTTcTGTCCGTCATggCCTtg

>Bacillus_Fam_105_53_5 Nr. of seq. 5 Alignment length(with gaps) = 56
Alignment score = 0.664286
GCF_000153365.1_ASM15336v1_genomic.fna_15:302007-302379 Satlength=373 Nr of Repeats=7 RepeatLength=53 seed=AGTCATGTAT Num.seqs=6 Similarity=0.859119
0   AGTCATGTATCCCAACAAGCA-TCC-GGGATACATAACAAGGCTGAGAAAAGAG-A
GCF_000473245.1_ASM47324v1_genomic.fna_1:1507348-1507827 Satlength=480 Nr of Repeats=9 RepeatLength=53 seed=AGTCATGTAT Num.seqs=7 Similarity=0.868224 0
AGTCATGTATCCCACCGGGCA-TCC-GGGATACATAACCAGGCTGAGAAAAGAG-A
GCF_000153365.1_ASM15336v1_genomic.fna_16:360419-360896 Satlength=478 Nr of Repeats=9 RepeatLength=53 seed=GGGATACATG Num.seqs=7 Similarity=0.940102
24   AGTCATGTACTTCAACAGGC--TCCTGGGATACATGACCCGGCCGTGCAAAGGG-A
Rev.of_GCF_000473245.1_ASM47324v1_genomic.fna_1:2449207-2449684 Satlength=478 Nr of Repeats=9 RepeatLength=53 seed=CCCTTTGCAC Num.seqs=9 Similarity=0.938505 52
AGTCATGTACTTCAACAGGC--TCCTGGGATACATGACCCGGCCGTGCAAAGGG-A
Rev.of_GCF_000473245.1_ASM47324v1_genomic.fna_1:4524426-4525114 Satlength=689 Nr of Repeats=11 RepeatLength=53 seed=CTGCTTTTGG Num.seqs=9 Similarity=0.870021
21   ACTCATGTATCCCAA-AAGCAGTCT-GAGATACATGACCCGGC-GAAAAAAGCGCA

* *******   **   ** ** * ******* ** *** *   **** * *
Consensus:

AgTCATGTAtccCAacagGCaTCcGgGATACATgACccGGCnGagaAAAGnGA

>Bacillus_Fam_106_53_5 Nr. of seq. 5 Alignment length(with gaps) = 56
Alignment score = 0.671429
GCF_900156865.1_PRJEB18960_genomic.fna_3:359545-359916 Satlength=372 Nr of Repeats=7 RepeatLength=53 seed=ATGGATAAAA Num.seqs=7 Similarity=0.881402
0   ATGGATAAAAATAGGAGAAAGCGAACCAGNAAAGGC-G-T-TAATCATCCACGCAA
Rev.of_GCF_900156865.1_PRJEB18960_genomic.fna_3:1976860-1977284 Satlength=425 Nr of Repeats=8 RepeatLength=53 seed=TCTCCTAATT Num.seqs=8 Similarity=0.739443 18
TTGGATGAAATTAGGAGAAAGCGAACCAGTAAAGTN-G-T-GAATCGTCCTCGCAC
Rev.of_GCF_900156865.1_PRJEB18960_genomic.fna_3:2163225-2163596 Satlength=372 Nr of Repeats=7 RepeatLength=53 seed=TCTCCTAATT Num.seqs=7 Similarity=0.766397 18
ATGGATGAAATTAGGAGAAAGCGAACCAGTAAAGT--G-TCGAATCATCCTCGCAA
GCF_900156865.1_PRJEB18960_genomic.fna_3:759210-759634 Satlength=425 Nr of Repeats=8 RepeatLength=53 seed=TTGGATAAAA Num.seqs=8 Similarity=0.710097
0   TTGGATAAAATTAGGNGAAAGCGAACCCGCAAACTCNG-T--AATCATCCTTGCAA
Rev.of_GCF_900156865.1_PRJEB18960_genomic.fna_3:3854012-3854277 Satlength=266 Nr of Repeats=4 RepeatLength=53 seed=ATGCAAGGAC Num.seqs=3 Similarity=0.874214 0
TTGGGTAAATTTAGGAGAATGCGGACCTGTAAAGGCNGCT---TTCGTCCTTGCAT

*** * ** **** *** *** *** * ***   * *   ** ***   ***
Consensus:

tTGGaTaAAatTAGGaGAAaGCGaACCaGtAAAgtcGTnaaTCaTCCtcGCAa

>Bacillus_Fam_107_52_5 Nr. of seq. 5 Alignment length(with gaps) = 55
Alignment score = 0.752727
BACILLUS_PSEUDALCALIPHILUS_genomic.fna_1:422934-423246 Satlength=313 Nr of Repeats=6 RepeatLength=52 seed=GAGTAAAGTA Num.seqs=6 Similarity=0.905660
0   GAGTAAAGTA-ACGAAAAGAGCCGAACGAGACAGTTACTCGGG-GGAAGTGAGA-
BACILLUS_PSEUDALCALIPHILUS_genomic.fna_3:348761-349177 Satlength=417 Nr of Repeats=8 RepeatLength=52 seed=GAGTAAAGTA Num.seqs=8 Similarity=0.867925
0   GAGTAAAGTA-ACGAAAAGAGCCGAACGAGACAGTTACTCGAG-GGAAGTGAGT-
Rev.of_BACILLUS_PSEUDALCALIPHILUS_genomic.fna_6:81732-82096 Satlength=365 Nr of Repeats=7 RepeatLength=52 seed=AGTAACTGAC Num.seqs=7
Similarity=0.762204   38
GAGTAAAGTA-ACGAAAAGGGCNGAACGTGTCAGTTACTCGAG-GGAAGTGAGT-
BACILLUS_PSEUDALCALIPHILUS_genomic.fna_2:122808-123431 Satlength=624 Nr of Repeats=12 RepeatLength=52 seed=GTAAAGTAAC Num.seqs=11
Similarity=0.794282   2
GAGTAAAGTA-ACGAAAAGAGGCGAACGAGNCAGTTACTCGAG-GGAAGT-AGNT
BACILLUS_PSEUDALCALIPHILUS_genomic.fna_1:538341-538653 Satlength=313 Nr of Repeats=6 RepeatLength=52 seed=TGTCAGTTAC Num.seqs=6 Similarity=0.738272
27   AAGT-AAGTACACGAAAAGAGCTGAACATGTCAGTTACT-GAGTGGTAGGGAGG-

*** ***** ******** * **** * ******** * * ** ** **
Consensus:

gAGTaAAGTAACGAAAAGaGccGAACgaGnCAGTTACTcGaGGGaAGtgAGn

>Bacillus_Fam_108_52_5 Nr. of seq. 5 Alignment length(with gaps) = 55
Alignment score = 0.681818
BACILLUS_PSEUDALCALIPHILUS_genomic.fna_2:93343-93855 Satlength=513 Nr of Repeats=9 RepeatLength=52 seed=TAATGTGCAG Num.seqs=8 Similarity=0.848160
0   TAATGTGCAGATACTGAG-GAGAAGAG-GGTTAGTATCTGCACAAAGA-GGAGGC
BACILLUS_PSEUDALCALIPHILUS_genomic.fna_5:78874-79289 Satlength=416 Nr of Repeats=8 RepeatLength=52 seed=TTAATGTGCA Num.seqs=7 Similarity=0.858341
51   TAATGTGCAGATACGGAA-GAGAGAAG-GGAGAGTAACTGCACAAAGA-GGCGTT
BACILLUS_PSEUDALCALIPHILUS_genomic.fna_5:376488-377061 Satlength=574 Nr of Repeats=11 RepeatLength=52 seed=TAATGTGCAG Num.seqs=10
Similarity=0.793621   0
TAATGTGCAGATANGGAA-GAGAAAAG-AGCTAGTATCTGAACAAAAAGGGCGAT
BACILLUS_PSEUDALCALIPHILUS_genomic.fna_5:354718-354978 Satlength=261 Nr of Repeats=5 RepeatLength=52 seed=GTATCTGCAC Num.seqs=5 Similarity=0.846154
31   TTATGTGCAGATAAGGAA-GAGT-AAGTAGCCGGTATCTGCACAAAGA-GTCGGT
BACILLUS_PSEUDALCALIPHILUS_genomic.fna_16:108274-109024 Satlength=751 Nr of Repeats=14 RepeatLength=52 seed=AGGCGGTTTA Num.seqs=13
Similarity=0.902435   45
TTATGTGCAGATACGGAAGGAGA-AAG-GGCTGGTATCTGTACAAAGA-GGCGGT

* ********** ** ***   ** *   *** *** ***** * * *
Consensus:

```
TaATGTGCAGATAcgGAaGAGanaAGgGctaGTAtCTGcACAAAgAGgcGgt

>Bacillus_Fam_109_52_5  Nr. of seq. 5 Alignment length(with gaps) = 55
Alignment score = 0.613333
GCF_000986785.1_ASM98678v1_genomic.fna_3:105763-105970 Satlength=208 Nr
of Repeats=4 RepeatLength=52 seed=AGGCTGTCAG  Num.seqs=3
Similarity=0.846154
0    -AGGCTGTCAGTGCCTTTTTTTCCAGCTTCCCCCG--ATTTTTGGGCACTGAGAG
Rev.of_GCF_000986785.1_ASM98678v1_genomic.fna_31:17-328 Satlength=312 Nr
of Repeats=6 RepeatLength=52 seed=GCTCTCAGTG  Num.seqs=5
Similarity=0.856410
1    -CTCCTGTCAGTTACCTTTTTTCCAGCTTGACCGG--TTTTTTGGGCACTGAGAG
Rev.of_GCF_000986785.1_ASM98678v1_genomic.fna_47:42-250 Satlength=209 Nr
of Repeats=4 RepeatLength=52 seed=CTCAGTACCC  Num.seqs=4
Similarity=0.719136
51    ACTACTGTCAGTGCCTTTTTTTCGGGTCTCTCTGG--ATTTTTGGGTACTGAGA-
GCF_000986785.1_ASM98678v1_genomic.fna_8:10201-10823 Satlength=623 Nr of
Repeats=12 RepeatLength=52 seed=GTCAGTTACC  Num.seqs=10
Similarity=0.761985
5    -AACCCGTCAGTTACCTTTTTTC--GCCTGNCCNGCAATTTTCGGGCACTGAGAG
GCF_000986785.1_ASM98678v1_genomic.fna_33:41960-42426 Satlength=467 Nr of
Repeats=9 RepeatLength=52 seed=GGGCACTGAG  Num.seqs=7 Similarity=0.746331
40    -AGCCTGTCAGTTNCCTTTTTTC--GGCTCCCCCTTGTATTTCGGGCACTGAGAG

* ******  * *******  *  *  *       *** *** *******
Consensus:

ancCtGTCAGTtnCcTTTTTTCnnGccTcnCcngatTTTtGGGcACTGAGAg

>Bacillus_Fam_110_52_5  Nr. of seq. 5 Alignment length(with gaps) = 54
Alignment score = 0.714815
GCF_000186145.1_Bacillus_sp_2_A_57_CT2_V1_genomic.fna_4:290005-290262
Satlength=258 Nr of Repeats=5 RepeatLength=51 seed=TGGTAGTTAA  Num.seqs=3
Similarity=0.873932                                        0
TGGTAGTTAATC-AGAGCGAATAACGACGAAAAA-CGGAATGGTC-AAGGAAAC
Rev.of_GCF_000294775.2_ASM29477v2_genomic.fna_1:3342184-3342700
Satlength=517 Nr of Repeats=10 RepeatLength=52 seed=TTTTTCGTCG
Num.seqs=6 Similarity=0.911111
33    TGGTAGTTAATC-AGAGCGAATAACGACGAAAAAGCGGAATTCTC-AAGGAAAC
GCF_000186145.1_Bacillus_sp_2_A_57_CT2_V1_genomic.fna_4:183188-183605
Satlength=418 Nr of Repeats=8 RepeatLength=52 seed=AATAACGACG  Num.seqs=7
Similarity=0.890110                                       18
TGGTAGTTAATCGAGA-AGAATAACGACGAAAAAGACGAA-GGTCGAAGGAAAA
Rev.of_GCF_000294775.2_ASM29477v2_genomic.fna_1:3448271-3448740
Satlength=470 Nr of Repeats=9 RepeatLength=52 seed=CCATTTTCCT  Num.seqs=8
Similarity=0.834249                                             55
TGGTAGTTAATCGAGA-AGAATAACGACGAAAAAGACGAA-GGTCGAAGGAAAA
Rev.of_GCF_000294775.2_ASM29477v2_genomic.fna_1:796655-797434
Satlength=780 Nr of Repeats=15 RepeatLength=52 seed=CGATTAACTA
Num.seqs=14 Similarity=0.779529
65    TGGTAGTTAATCGGGA-TGTTTAACGACGAAAATGACATG-GGTCNAGGGAAAA

************  **  *  ************          ** * *****
```

Consensus:

TGGTAGTTAATCgaGAnGaaTAACGACGAAAAagacgaaggTCnAaGGAAAa

>Bacillus_Fam_111_52_5 Nr. of seq. 5 Alignment length(with gaps) = 54
Alignment score = 0.714815
GCF_000708755.2_ASM70875v2_genomic.fna_2:1062403-1062663 Satlength=261 Nr of Repeats=4 RepeatLength=52 seed=TTTGCTACCC Num.seqs=3 Similarity=0.777778 0
TTTGCTACCCCATATTTCACCCG-CTCCGTTTTATAAGGATGCATTTTCTT-CT
GCF_000709935.2_ASM70993v2_genomic.fna_5:8490-8905 Satlength=416 Nr of Repeats=7 RepeatLength=52 seed=TTTGCTACCC Num.seqs=5 Similarity=0.771069
0 TTTGCTACCCCATATTTCACCCG-CTCCGTTTTATAAGGATGCATTTTCTT-CT
GCF_000708755.2_ASM70875v2_genomic.fna_6:445956-446216 Satlength=261 Nr of Repeats=4 RepeatLength=52 seed=TTTCTTCCTT Num.seqs=3 Similarity=0.897436
43 TTTGCTACCCTATATTCCGCTCA-CTCCGATTTATAAGGCAGCA-TTTCTTCCT
GCF_000709935.2_ASM70993v2_genomic.fna_5:651406-651666 Satlength=261 Nr of Repeats=4 RepeatLength=52 seed=TTTCTTCCTT Num.seqs=3 Similarity=0.897436
43 TTTGCTACCCTATATTCCGCTCA-CTCCGATTTATAAGGCAGCA-TTTCTTCCT
GCF_000709935.2_ASM70993v2_genomic.fna_15:49878-50085 Satlength=208 Nr of Repeats=4 RepeatLength=52 seed=CCCATATTTC Num.seqs=3 Similarity=0.725367
8 TTTGATACCCCATATTTC-CGCATCCCATTTTTATAGGGATGCATTTTCNC-NT

**** ***** ***** * * * * * ****** ** *** **** *
Consensus:

TTTGcTACCCcATATTtCnCnCaCtCcgtTTTATAaGGatGCAtTTTCttcT

>Bacillus_Fam_112_52_5 Nr. of seq. 5 Alignment length(with gaps) = 54
Alignment score = 0.676543
GCF_000708755.2_ASM70875v2_genomic.fna_2:1371598-1372014 Satlength=417 Nr of Repeats=8 RepeatLength=52 seed=ACTTATCTCG Num.seqs=8 Similarity=0.817766 0
ACTTATCTCGTTTTTC-AGTACCACCAATTCGGTCTTCCCCTT-CCTTGATGGC
GCF_000708755.2_ASM70875v2_genomic.fna_2:1674648-1674960 Satlength=313 Nr of Repeats=6 RepeatLength=52 seed=ACTTATCTCG Num.seqs=6 Similarity=0.808386 0
ACTTATCTCGGAGTTC-AGAACCACCAACACGCAGATTTCCTT-TTTTGATGGC
GCF_000709935.2_ASM70993v2_genomic.fna_3:97017-97277 Satlength=261 Nr of Repeats=5 RepeatLength=52 seed=GATGGCACTT Num.seqs=5 Similarity=0.882051
46 ACTTATCTCGGAGTTC-AGAACCACCAACACGCAGATTCCCTT-TTTTGATGGC
GCF_000708755.2_ASM70875v2_genomic.fna_6:335157-335781 Satlength=625 Nr of Repeats=12 RepeatLength=52 seed=TTGATGGCAC Num.seqs=10 Similarity=0.807977 44
ACTTATCNCG-CTTACAAGTACCACCAA-ACTCAGAAATCCTTCTCTTGATGGC
GCF_000709935.2_ASM70993v2_genomic.fna_5:541475-541891 Satlength=417 Nr of Repeats=8 RepeatLength=52 seed=TTGATGGCAC Num.seqs=8 Similarity=0.799451
44 ACTTATCTCG-CTTACAAGTACCACCAA-ACTCAGAAATCCTTCTCTTGATGGC

******* ** * * ** ******** * **** ********
Consensus:

ACTTATCtCGnntTtCAGtACCACCAAnaCgcagatntCCTTtcTTGATGGC

>Bacillus_Fam_113_52_5 Nr. of seq. 5 Alignment length(with gaps) = 54
Alignment score = 0.609877
GCF_001636315.1_ASM163631v1_genomic.fna_1:1832094-1832406 Satlength=313
Nr of Repeats=5 RepeatLength=52 seed=GACAGTTTTG Num.seqs=4
Similarity=0.846154 0
GACAGTTTTGCTAGTTTTTGAAC-TG-GTTTTGTCTTCAAGAACACTTATTGAA
GCF_001636315.1_ASM163631v1_genomic.fna_1:2472381-2472589 Satlength=209
Nr of Repeats=4 RepeatLength=52 seed=CTTATTGAAG Num.seqs=4
Similarity=0.897436 43
GACAGTTTTAGTAGTTTTCGCAG-CG-GTTTTGTCTCCAAGAGGCCTTATTGAA
Rev.of_GCF_001636315.1_ASM163631v1_genomic.fna_1:2851663-2851975
Satlength=313 Nr of Repeats=6 RepeatLength=52 seed=GCATAAAAAA Num.seqs=6
Similarity=0.938462 24
GACAGTTTCGATGATTTTTTATG-CT-GTTTTGTCTTCAAGACGGCTTATTGAA
Rev.of_GCF_001636315.1_ASM163631v1_genomic.fna_1:2942377-2942588
Satlength=212 Nr of Repeats=4 RepeatLength=52 seed=AAACTGTCTT Num.seqs=3
Similarity=0.702306 8
GACAGTTTCTC-AATTTTTGTAG-GGCNGTTTGTCTCCAAGAANCCTTATTGAA
Rev.of_GCF_001636315.1_ASM163631v1_genomic.fna_1:3763475-3763890
Satlength=416 Nr of Repeats=8 RepeatLength=52 seed=TGTCTTTAAT Num.seqs=5
Similarity=0.848718 4
GACA-TTTTCATAAGTTCCTAGGCCG-GGTTTGTCTTCTAGAAGCCTTATTAAA

**** *** ** ******* * *** ****** **
Consensus:

GACAgTTTtnntaatTTttgaagcggtTTTGTCTtCaAGAagcCTTATTgAA

>Bacillus_Fam_114_52_5 Nr. of seq. 5 Alignment length(with gaps) = 54
Alignment score = 0.701235
GCF_900156875.1_PRJEB18969_genomic.fna_11:799820-800080 Satlength=261 Nr of Repeats=5 RepeatLength=52 seed=GATGAGAACC Num.seqs=5
Similarity=0.915385
51 ATGAGAACCGAAACAAACGAAACAAGAGAA-GGAAA-CGGTCTTCATCAAGGGG
GCF_900156875.1_PRJEB18969_genomic.fna_11:864748-865216 Satlength=469 Nr of Repeats=9 RepeatLength=52 seed=TGAGAACCGA Num.seqs=9
Similarity=0.790598
53 ATGAGAACCGAAACAACCGCAACAAGAGAA-GGAAC-CGGTCTTCATCAAGAGG
Rev.of_GCF_900156875.1_PRJEB18969_genomic.fna_11:1169427-1169687
Satlength=261 Nr of Repeats=5 RepeatLength=52 seed=ATGAAGACCG Num.seqs=5
Similarity=0.884615 97
ATGAGAACCGAAACAATCGCAGCACGAGAA-GGGAA-CGGTCTTCATTAAGGGA
Rev.of_GCF_900156875.1_PRJEB18969_genomic.fna_11:892286-897590
Satlength=5305 Nr of Repeats=101 RepeatLength=52 seed=GATGAAGACC
Num.seqs=100 Similarity=0.834986
98 ATGAGAACCGTAACTATCACTGCATGAGAA-GGAAA-CGGTCTTCATCATGGAG
Rev.of_GCF_900156875.1_PRJEB18969_genomic.fna_11:2645550-2646025
Satlength=476 Nr of Repeats=9 RepeatLength=53 seed=ACCGCTTTCC Num.seqs=7
Similarity=0.833921 92
ATGAAGACCGAAACCA-GGTAGGAAGAGAAGGGAAAGCGGTCTTCATNAAGAGA

**** **** *** *   * ***** ** * ********** * *
Consensus:

gATGAgaACCGaAACaAncgcagcAaGAGAAGGaAaCGGTCTTCATcAaGgg

>Bacillus_Fam_115_53_5 Nr. of seq. 5 Alignment length(with gaps) = 53 Alignment score = 0.694969
GCF_001590785.1_ASM159078v1_genomic.fna_38:115242-115452 Satlength=211 Nr of Repeats=4 RepeatLength=52 seed=GGTCAAAAGG Num.seqs=3 Similarity=0.445355 0
GGTCAAAAGGAGNAGCNCTATTCACCCGAAAAGCGAG-CGNACCAAGAA--AC
GCF_001590785.1_ASM159078v1_genomic.fna_5:48221-48618 Satlength=398 Nr of Repeats=8 RepeatLength=52 seed=AACGGTCAAA Num.seqs=6 Similarity=0.712103
49   GGTCAAAAGGAGAAGCGCTATTCACCCGAAAAGAGAGCCGCATCA-GAAGAAC
Rev.of_GCF_001590785.1_ASM159078v1_genomic.fna_23:40421-40734 Satlength=314 Nr of Repeats=6 RepeatLength=52 seed=CCTTTTGACC Num.seqs=5 Similarity=0.883019 61
GGTCAAAAGGAGAAGCGCTATTCACCCGAAAAGAGAGCCGCACCACGAAA-AC
GCF_001590785.1_ASM159078v1_genomic.fna_25:115-322 Satlength=208 Nr of Repeats=4 RepeatLength=52 seed=GGTCAAAAGG Num.seqs=3 Similarity=0.675052
0   GGTCAAAAGGCGAAGCTCTATTCACCCGAAAAAGGAGCTG-ANCNAGTAGAAC
Rev.of_GCF_001590785.1_ASM159078v1_genomic.fna_3:106756-107171 Satlength=416 Nr of Repeats=7 RepeatLength=52 seed=CCTTTTGACC Num.seqs=6 Similarity=0.797531 10
GGTCAAAAGGAAGAGCTCTATTCACCTGAAAAAGNTGGTGAGCCAAGAA-AAG

**********   *** ********* *****   * *   * * * *
Consensus:

GGTCAAAAGGagaAGCnCTATTCACCcGAAAAgngaGccGnacCaaGaAnaAc

>Bacillus_Fam_116_51_5 Nr. of seq. 5 Alignment length(with gaps) = 53 Alignment score = 0.735220
BACILLUS_PSEUDALCALIPHILUS_genomic.fna_2:381197-381605 Satlength=409 Nr of Repeats=8 RepeatLength=51 seed=GAGTAACCCC Num.seqs=8 Similarity=0.881525
29   AATCGCGGGGTTGCTGG-TGAAAAA-AGNGGGAGTAACCCCGAGCAAACGGAG
BACILLUS_PSEUDALCALIPHILUS_genomic.fna_10:100326-100580 Satlength=255 Nr of Repeats=5 RepeatLength=51 seed=AACCCCGAGC Num.seqs=4 Similarity=0.839623 33
AATCGCGGGGTTGCGGG-CGTAAAA-AGAAGGAGTAACCCCGAGCAAACGGTG
Rev.of_BACILLUS_PSEUDALCALIPHILUS_genomic.fna_6:367038-367242 Satlength=205 Nr of Repeats=4 RepeatLength=51 seed=CCGTTTGCTC Num.seqs=4 Similarity=0.865385 49
AATCTCGGGGTTACGGGCTGGAAAA-AGCAGGAGGAACCCCGAGCAAACGGAG
Rev.of_BACILLUS_PSEUDALCALIPHILUS_genomic.fna_2:392539-392794 Satlength=256 Nr of Repeats=5 RepeatLength=51 seed=TTGCTCGGGT Num.seqs=5 Similarity=0.869281 45
AATCTCGGGGTTGCAGT-CGGAAAA-AGCAGGAGTAAACCCGAGCAAACGTGG
BACILLUS_PSEUDALCALIPHILUS_genomic.fna_5:184414-184771 Satlength=358 Nr of Repeats=7 RepeatLength=51 seed=CCCGAGCAAA Num.seqs=7 Similarity=0.809219
7   GAGTAACCCCGAGCAAATGGA-AATACGCGGGGTTGCGAG-CAGAAAAAGNGG

*   *   * **   * **   ** *** * *

Consensus:

aAtcncggggttgCnggnGnaAAaaGcaGGagtaaccccgAGcAAAcGgnG

>Bacillus_Fam_117_52_5 Nr. of seq. 5 Alignment length(with gaps) = 53
Alignment score = 0.725786
BACILLUS_PSEUDALCALIPHILUS_genomic.fna_5:475827-476186 Satlength=360 Nr of Repeats=7 RepeatLength=51 seed=AGTAACTGAC Num.seqs=5 Similarity=0.926923
0 AGTAACTGACACGTTCAGGCGTTTTCGTGTAGTTACTCACGCC-ACCCCTCC-
Rev.of_BACILLUS_PSEUDALCALIPHILUS_genomic.fna_4:117009-117217
Satlength=209 Nr of Repeats=4 RepeatLength=52 seed=GAGTAACTAC Num.seqs=4 Similarity=0.948718 38
AGTAACTGTCATGTTCAGCCTTTTTCGTGTAGTTACTCACCCT-CTTCCTTCG
Rev.of_BACILLUS_PSEUDALCALIPHILUS_genomic.fna_5:101702-102015
Satlength=314 Nr of Repeats=6 RepeatLength=52 seed=GTGACAGTTA Num.seqs=5 Similarity=0.816352 64
AGTAACTGTCACCTTCAGCCTNTTTCGTGTCGTTACTCACCTT-CTTCCTCCG
BACILLUS_PSEUDALCALIPHILUS_genomic.fna_6:91832-92092 Satlength=261 Nr of Repeats=5 RepeatLength=52 seed=AGTTACTCAC Num.seqs=5 Similarity=0.883019
82 AGTAACTGACATGTTTGGCCTTTTTCGTGCAGTTACTCACGCT-TTTCCTTTG
BACILLUS_PSEUDALCALIPHILUS_genomic.fna_2:344904-345112 Satlength=209 Nr of Repeats=4 RepeatLength=52 seed=AGTAACTGAC Num.seqs=4 Similarity=0.756813
105 AGTAACTGACATGATTGGCCTTTTTCGTGCAGTTACTCACCCTATTCCCTTCG

******** ** * * * ******* ********* ***
Consensus:

AGTAACTGaCAtgtTcaGcCttTTTCGTGtaGTTACTCACcctnttCCTtcg

>Bacillus_Fam_118_52_5 Nr. of seq. 5 Alignment length(with gaps) = 53
Alignment score = 0.699371
GCF_001439965.1_ASM143996v1_genomic.fna_125:65382-65694 Satlength=313 Nr of Repeats=5 RepeatLength=52 seed=AGGTTACCGA Num.seqs=4
Similarity=0.752137
0 AGGTTACCGAGCCCCCGCCATTTACTCCATCCCGGTAACCAAACTCTCTCTT-
Rev.of_GCF_001887185.1_ASM188718v1_genomic.fna_197:48906-49947
Satlength=1042 Nr of Repeats=17 RepeatLength=52 seed=GTTCGGTAAC
Num.seqs=12 Similarity=0.836981
12 TGGTTACCGAACGCGCGCAATATCGTCCATCCGGGTAACCAATCCCTCTCAA-
Rev.of_GCF_001887185.1_ASM188718v1_genomic.fna_62:29140-29922
Satlength=783 Nr of Repeats=14 RepeatLength=52 seed=CGGGAACTAA
Num.seqs=12 Similarity=0.786382
8 -AGTTCCCGTGTCCGCGTAAAATCCTCCATCAAGGTAACCAAACGCACTCATT
GCF_002009555.1_ASM200955v1_genomic.fna_166:5014-5326 Satlength=313 Nr of Repeats=5 RepeatLength=52 seed=ATTAGTTCCC Num.seqs=4 Similarity=0.884615
49 -AGTTCCCGAGCCCGCGAAAAATCCTCCATCAAGGTAACCAAACGCACTCATT
GCF_002009555.1_ASM200955v1_genomic.fna_217:22-438 Satlength=417 Nr of Repeats=8 RepeatLength=52 seed=CTCCATCAAG Num.seqs=8 Similarity=0.788745
23 -AGTTCCCGTGCCCGCGGAAAATCCTCCATCAAGGTAACCAATCGCACTCATT

*** *** * ** * * ****** ********* * * ***
Consensus:

aGTTcCCGagccCgCGnaAaaTccTCCATCaaGGTAACCAAaCgCaCTCatt

>Bacillus_Fam_119_52_5 Nr. of seq. 5 Alignment length(with gaps) = 53
Alignment score = 0.620755
GCF_900104555.1_IMG-
taxon_2651870172_annotated_assembly_genomic.fna_7:4560-4976 Satlength=417
Nr of Repeats=8 RepeatLength=52 seed=GTTTTGTCTA Num.seqs=8
Similarity=0.776675   0
GTTTTGTCTAATAGAGCTCTTCTATTGAACAAAACCATTCCCCGAGA-CATGT
Rev.of_GCF_900104555.1_IMG-
taxon_2651870172_annotated_assembly_genomic.fna_31:30740-31103
Satlength=364 Nr of Repeats=7 RepeatLength=52 seed=TTTGTCCGAT Num.seqs=6
Similarity=0.818449   33
GTTTTGTCTATTAGAGGGCTTCTATCGGACAAAACCATTGCTC-AGACCATGA
Rev.of_GCF_900104555.1_IMG-
taxon_2651870172_annotated_assembly_genomic.fna_29:79068-79380
Satlength=313 Nr of Repeats=6 RepeatLength=52 seed=TCTATTAGAC Num.seqs=6
Similarity=0.824948   15
GTTTTGTCTAATAGAGNCCGTCTATTGGACAAATTCCTTGCTC-ACAACAGGG
Rev.of_GCF_900104555.1_IMG-
taxon_2651870172_annotated_assembly_genomic.fna_25:114221-114585
Satlength=365 Nr of Repeats=7 RepeatLength=52 seed=GCTCTATTGG Num.seqs=7
Similarity=0.892552   17
GTTTTGTCCAATAGAGCTGTCCTATCGAACATAATCTTTGCTC-GGACCTGGT
GCF_900104555.1_IMG-
taxon_2651870172_annotated_assembly_genomic.fna_19:27781-28613
Satlength=833 Nr of Repeats=16 RepeatLength=52 seed=CTATTGGACA
Num.seqs=16 Similarity=0.823113   21
GGTTTGTCTAATAAAGACCCCCTATTGGACAGAATCATCACTCNGGA-CATGT

* ****** * ** **   **** * *** * * * * *   * * *
Consensus:

GtTTTGTCtAaTAgAGnncttCTATtGgACAaAatCaTtgCtCagAnCatGt

>Bacillus_Fam_120_52_5 Nr. of seq. 5 Alignment length(with gaps) = 52
Alignment score = 0.853846
GCF_001439965.1_ASM143996v1_genomic.fna_95:54401-54661 Satlength=261 Nr
of Repeats=5 RepeatLength=52 seed=ACACATTTTT Num.seqs=5
Similarity=0.800000
0   ACACATTTTTCGCTTCTCCCCATGATAATCTGTCTCAGGCATGGCTTCTGAC
GCF_001887185.1_ASM188718v1_genomic.fna_5:448-656 Satlength=209 Nr of
Repeats=4 RepeatLength=52 seed=TGATAATCTG Num.seqs=4 Similarity=0.987179
22   ACACATTTTTCGCTTCTCCCCATGATAATCTGTCTCAGGCATGGCTTCTGAC
Rev.of_GCF_001887185.1_ASM188718v1_genomic.fna_11:16345-16813
Satlength=469 Nr of Repeats=9 RepeatLength=52 seed=TGAGACAGAT Num.seqs=9
Similarity=0.792181   37
ACACATTTTTCGCTTNTCCCCATGATAATCTGTCTCAGGCATGGCTTCTGAC
GCF_001439965.1_ASM143996v1_genomic.fna_95:56169-56429 Satlength=261 Nr
of Repeats=5 RepeatLength=52 seed=AATCTGTCTC Num.seqs=5
Similarity=0.810256
26   ACACATTTTTGACCTTTACCTTTGATAATCTGTCTCANGCATGGCCTCTGAC
GCF_002009555.1_ASM200955v1_genomic.fna_56:33-448 Satlength=416 Nr of
Repeats=8 RepeatLength=52 seed=AATCTGTCTC Num.seqs=7 Similarity=0.846154
26   ACACATTTTTGGCCTTTACCTTTGATAATCTGTCTCAAGTATGGCCTCTGAC

********** * * * ** ************** * ***** ******
Consensus:

ACACATTTTTcgCtTnTcCCcaTGATAATCTGTCTCAgGcATGGCtTCTGAC

>Bacillus_Fam_121_52_5 Nr. of seq. 5 Alignment length(with gaps) = 52
Alignment score = 0.823077
GCF_001636335.1_ASM163633v1_genomic.fna_1:206310-206778 Satlength=469 Nr of Repeats=9 RepeatLength=52 seed=TTATTGAACC Num.seqs=9 Similarity=0.753258
0 TTATTGAACCCCGAGCAGGCGAAAAAAAGCATCAAAGAGTCTTCAATAGGTG
Rev.of_GCF_001636335.1_ASM163633v1_genomic.fna_1:210863-211279 Satlength=417 Nr of Repeats=8 RepeatLength=52 seed=CTATTGAAGA Num.seqs=8 Similarity=0.879155 49
GGATTGAACCCCGAACTGGCGAAAAAAAGCATCAAAGAGTCTTCAATAGAGG
Rev.of_GCF_001636335.1_ASM163633v1_genomic.fna_1:3030262-3031197 Satlength=936 Nr of Repeats=17 RepeatLength=52 seed=ATTGAAGACT Num.seqs=14 Similarity=0.826042
99 CTATTGAACCCCGAACAAGCGAAAAAAAGCANCAAAGAGTCTTCAATAGAGG
Rev.of_GCF_001636335.1_ASM163633v1_genomic.fna_1:2795926-2796445 Satlength=520 Nr of Repeats=10 RepeatLength=52 seed=TATTGAGGAC Num.seqs=9 Similarity=0.870720
48 TCATTGAACCCCGAACAAGCGAAAAAAACCGTCAAAGCGTCCTCAATAGGGG
Rev.of_GCF_001636335.1_ASM163633v1_genomic.fna_1:2827224-2827795 Satlength=572 Nr of Repeats=11 RepeatLength=52 seed=AGGACTCTTT Num.seqs=10 Similarity=0.822222
95 TCCTTGAACCCCGAACAGGCGAAAAAAATCGTCAAAGAGTCCTCAATAGGGG

*********** * ********** * ***** *** ******* *
Consensus:

tnaTTGAACCCCGAaCagGCGAAAAAAAgCatCAAAGaGTCtTCAATAGggG

>Bacillus_Fam_122_45_5 Nr. of seq. 5 Alignment length(with gaps) = 48
Alignment score = 0.736806
GCF_000007845.1_ASM784v1_genomic.fna_1:3515122-3515563 Satlength=442 Nr of Repeats=9 RepeatLength=45 seed=ACTGGACCTC Num.seqs=7 Similarity=0.874427
0 ACTGGACCTCAAGGTGTTCAAGG--A-CCAGCAGGTGCTACCGGTGCT
GCF_000008505.1_ASM850v1_genomic.fna_1:3540669-3543189 Satlength=2521 Nr of Repeats=56 RepeatLength=45 seed=ACTGGACCTC Num.seqs=45 Similarity=0.827814 0
ACTGGACCTCAAGGTGTTCAAGG--A-CCAGCAGGTGCTACTGGTGCC
Rev.of_GCF_000290995.1_Baci_cere_AND1407_G13175_V1_genomic.fna_3:327060-327582 Satlength=523 Nr of Repeats=11 RepeatLength=45 seed=CCTTGAGGTC Num.seqs=9 Similarity=0.787842 14
ACTGGACCTCAAGGTGTTCAAGG--A-CCGGCGGGTGCTACTGGTGCT
GCF_000831065.1_ASM83106v1_genomic.fna_1:3427920-3429909 Satlength=1990 Nr of Repeats=47 RepeatLength=45 seed=ACTGGACCTC Num.seqs=29 Similarity=0.774327 0
ACTGGACCTCAAGGTGTTCAAGGT-A-ACA-CNGGTGCTACTGGTGCC
GCF_000017425.1_ASM1742v1_genomic.fna_1:2475569-2477171 Satlength=1603 Nr of Repeats=43 RepeatLength=45 seed=GGACCTCAAG Num.seqs=30

Similarity=0.881073 3
ACTGGACCTCAAGGGCCTCAAGG-CATCCA--AGGACCTACAGGGGCT

************** ****** * * ** **** ** **
Consensus:

ACTGGACCTCAAGGtgtTCAAGGAcCagcaGGtgCTACtGGtGCt

>Bacillus_Fam_123_39_5 Nr. of seq. 5 Alignment length(with gaps) = 42
Alignment score = 0.642857
GCF_000017425.1_ASM1742v1_genomic.fna_1:3517703-3517985 Satlength=283 Nr of Repeats=8 RepeatLength=36 seed=TTCTTCTATA Num.seqs=6 Similarity=0.987654
0 TTCTTCT-AT-ACGCTGC-TCTTCTTGCTTGCGTTTTTC---
Rev.of_GCF_001719185.1_ASM171918v1_genomic.fna_1:3645533-3645689 Satlength=157 Nr of Repeats=4 RepeatLength=39 seed=AAGCAAGAGG Num.seqs=4 Similarity=0.897436 30
TTCTGCTAATCGCGCTTCTTCCTCTTGCTTGCGTTTTTC---
GCF_000008505.1_ASM850v1_genomic.fna_1:3065080-3065314 Satlength=235 Nr of Repeats=7 RepeatLength=39 seed=TGCTCTTCTT Num.seqs=5 Similarity=0.938462
13 TTCTACT-CT-TTGCTGC-TCTTCTTGCTTACGTTTTTCTTC
Rev.of_GCF_000832605.1_ASM83260v1_genomic.fna_1:1202055-1202214 Satlength=160 Nr of Repeats=4 RepeatLength=39 seed=AAACGTAAGC Num.seqs=3 Similarity=0.977208 33
CTCTACT-CT-TTGTTGC-TCTTCCTGCTTACGTTTTTCTTC
GCF_000290995.1_Baci_cere_AND1407_G13175_V1_genomic.fna_1:2060357-2060513 Satlength=157 Nr of Repeats=4 RepeatLength=39 seed=TTTGTTGCTC Num.seqs=4 Similarity=0.874644 47 TTCTGCT-CT-TTGTTGC-TCTTCTTGCTTACGCTTCTCTTC

*** ** * * * * ** ** ***** ** ** **
Consensus:

tTCTnCTcTttGcTgCTCtTCtTGCTTaCGtTTtTCttc

>Bacillus_Fam_124_40_5 Nr. of seq. 5 Alignment length(with gaps) = 42
Alignment score = 0.684127
GCF_000285535.1_ASM28553v1_genomic.fna_2:812840-813040 Satlength=201 Nr of Repeats=5 RepeatLength=40 seed=ATTTGCGTCT Num.seqs=5 Similarity=0.753333
0 ATTTGCGTC-TCCTCTANGATTTTTGCGTCGGTTTTCTCAC-
GCF_000285535.1_ASM28553v1_genomic.fna_31:3-163 Satlength=161 Nr of Repeats=4 RepeatLength=40 seed=TTTTCTCACA Num.seqs=4 Similarity=0.905556
31 ATTTGCGTC-TCCCCAGCGATTTTTGCGTCGGTTTTCTCAC-
Rev.of_GCF_000285535.1_ASM28553v1_genomic.fna_51:18-258 Satlength=241 Nr of Repeats=7 RepeatLength=40 seed=AGACGCAAAT Num.seqs=5 Similarity=0.743089
50 ATTTGCGTC-TCCCCAGCGATTTTTGCGTCGATTTTCTCAN-
GCF_000285535.1_ASM28553v1_genomic.fna_2:954754-954914 Satlength=161 Nr of Repeats=5 RepeatLength=40 seed=TTTTTGCGTC Num.seqs=3 Similarity=0.796748
19 ATTTGCGTC-TTCTCCCTGATTTTTGCGTCGATATCCGCGG-

Rev.of_GCF_001439915.1_ASM143991v1_genomic.fna_105:62151-62311 Satlength=161 Nr of Repeats=5 RepeatLength=40 seed=CGACGCAAAT Num.seqs=3 Similarity=0.777778 10
ATTTGCGTCGTGCTC-GAGATTTATGCGTCGGTTTGC-CAAT

********* * * * ***** ******* * * * *
Consensus:

ATTTGCGTCTcCtCngnGATTTtTGCGTCGgTtTtCtCan

>Bacillus_Fam_125_42_5 Nr. of seq. 5 Alignment length(with gaps) = 42 Alignment score = 0.888889
GCF_000496285.1_ASM49628v1_genomic.fna_1:3159834-3160296 Satlength=463 Nr of Repeats=11 RepeatLength=42 seed=AAAGAAGAAG Num.seqs=11 Similarity=0.894949 0
AAAGAAGAAGTAAAAGAGCCGGTAAAAGAAGTTGAAGAAACG
Rev.of_GCF_000775975.1_ASM77597v1_genomic.fna_1:4810089-4810866 Satlength=778 Nr of Repeats=5 RepeatLength=42 seed=TCTTCTTTTG Num.seqs=4 Similarity=0.841270 8
AAAGAAGAAGTAAAAGAGCCGGTAAAAGAAGTTGAAGAAACA
GCF_000831065.1_ASM83106v1_genomic.fna_1:383609-384197 Satlength=589 Nr of Repeats=14 RepeatLength=42 seed=AAAGAAGAAG Num.seqs=14 Similarity=0.913134 0
AAAGAAGAAGTGAAAGAACCGGTAAAAGAAGTTGAAGGAACA
Rev.of_GCF_000832605.1_ASM83260v1_genomic.fna_1:5196965-5197301 Satlength=337 Nr of Repeats=8 RepeatLength=42 seed=CGGCTCTTTT Num.seqs=8 Similarity=0.907029 21
AAAGAAGAAGTAAAAGAGCCGGTAAAAGAAGTTGAAGAAACA
GCF_001645555.1_ASM164555v1_genomic.fna_2:16394-16667 Satlength=274 Nr of Repeats=8 RepeatLength=42 seed=GAAGTAAAAG Num.seqs=5 Similarity=0.828571 6 AAAGAAGAAGTAAAAGAGCCAACAAAAGAAGTTGAAGGANCG

*********** ***** ** ************** * *
Consensus:

AAAGAAGAAGTaAAAGAgCCggtAAAAGAAGTTGAAGaAaCa

>Bacillus_Fam_126_39_5 Nr. of seq. 5 Alignment length(with gaps) = 39 Alignment score = 0.788034
GCF_000007825.1_ASM782v1_genomic.fna_1:4664228-4664735 Satlength=508 Nr of Repeats=13 RepeatLength=39 seed=TAAGCAACAA Num.seqs=13 Similarity=0.780769 0
TAAGCAACAAAGTNCTAAACAAGANGATTCCTCTCAGGA
GCF_000831065.1_ASM83106v1_genomic.fna_1:4422505-4422934 Satlength=430 Nr of Repeats=11 RepeatLength=39 seed=GCTAAACAAG Num.seqs=11 Similarity=0.854545 13
TAAGCAACAAAGTGCTAAACAAGATGATTCNTCTCAAGA
GCF_000496285.1_ASM49628v1_genomic.fna_1:1925714-1926104 Satlength=391 Nr of Repeats=10 RepeatLength=39 seed=TCTCATGAGA Num.seqs=10 Similarity=0.889079 31
GAAACAACAAAATTCTAAACAAGACGATTCCTCTCATGA
Rev.of_GCF_000775975.1_ASM77597v1_genomic.fna_1:143645-144191 Satlength=547 Nr of Repeats=13 RepeatLength=39 seed=TTAGAATTTT

Num.seqs=12 Similarity=0.852370
18   CAAGCAGCAAAATTCTAAGCAAGATGATTCTTCTCAAAA
Rev.of_GCF_000832605.1_ASM83260v1_genomic.fna_1:1250277-1250745
Satlength=469 Nr of Repeats=15 RepeatLength=39 seed=CTTGTTTAGA
Num.seqs=9 Similarity=0.840456
23   CAAACAGCAAAGTTCTAAACAAGAGGATTCTTCTCAAGA

** ** **** * **** ***** ***** ***** *
Consensus:

nAAgCAaCAAAgTtCTAAaCAAGAnGATTCnTCTCAagA

>Bacillus_Fam_127_36_5 Nr. of seq. 5 Alignment length(with gaps) = 36
Alignment score = 0.792593
GCF_000007825.1_ASM782v1_genomic.fna_1:4624379-4625096 Satlength=718 Nr of Repeats=5 RepeatLength=36 seed=GTTGCTCTTC Num.seqs=4
Similarity=0.925926
0   GTTGCTCTTCTACGACTGGCGCTTCTTCTACAACTC
GCF_000496285.1_ASM49628v1_genomic.fna_1:1885490-1886414 Satlength=925 Nr of Repeats=18 RepeatLength=36 seed=TGGTTGTTCT Num.seqs=12
Similarity=0.956229 34
GTTGTTCTTCAGCGACTGGCCCTTCTTCTGCAACTG
GCF_000007845.1_ASM784v1_genomic.fna_1:4486380-4486794 Satlength=415 Nr of Repeats=8 RepeatLength=36 seed=TTGTTCTTCT Num.seqs=5
Similarity=0.837037
1   GTTGTTCTTCTACGACCGGTGCTTCCTCTGCAACTG
GCF_000008505.1_ASM850v1_genomic.fna_1:4481591-4482005 Satlength=415 Nr of Repeats=11 RepeatLength=36 seed=TTGTTCTTCT Num.seqs=10
Similarity=0.853498 1
GTTGTTCTTCTACGACCGGTGTTTCCTCTGCAACTG
Rev.of_GCF_000290995.1_Baci_cere_AND1407_G13175_V1_genomic.fna_2:176867-177083 Satlength=217 Nr of Repeats=7 RepeatLength=36 seed=AGAAGAACAA
Num.seqs=5 Similarity=0.829630 11
ATTGTTCTTCTGCGACCGGTGTTTCTTCTACAACTG

*** ***** **** **   *** *** *****
Consensus:

gTTGtTCTTCtaCGACcGGtgcTTCtTCTgCAACTg

>Bacillus_Fam_128_36_5 Nr. of seq. 5 Alignment length(with gaps) = 36
Alignment score = 0.874074
GCF_000007825.1_ASM782v1_genomic.fna_1:4625342-4625852 Satlength=511 Nr of Repeats=15 RepeatLength=36 seed=CTTCCACTTC Num.seqs=10
Similarity=0.893827 0
CTTCCACTTCTTCTGACTCTTTCGCTTCCGCAATTA
GCF_000831065.1_ASM83106v1_genomic.fna_1:4383407-4384004 Satlength=598 Nr of Repeats=16 RepeatLength=36 seed=GCAATTACTT Num.seqs=14
Similarity=0.859178 29
CTTCNACTTCTTCTGACTCTTCTGCTTCCGCAATTA
GCF_000007845.1_ASM784v1_genomic.fna_1:4486994-4487255 Satlength=262 Nr of Repeats=7 RepeatLength=36 seed=CTTCTGTTTC Num.seqs=5
Similarity=0.896296
18   CTTCTACTTCTTCTGACTCTTCTGTTTCTGCAATTA

GCF_000008505.1_ASM850v1_genomic.fna_1:4482205-4482457 Satlength=253 Nr of Repeats=7 RepeatLength=36 seed=CTTCTGTTTC Num.seqs=7 Similarity=0.890653
18 CTTCCACTTCTTCTAACTCTTCTGTTTCTGCAATTA
Rev.of_GCF_001645555.1_ASM164555v1_genomic.fna_4:74869-75013 Satlength=145 Nr of Repeats=4 RepeatLength=36 seed=GAAACAGAAG Num.seqs=4 Similarity=0.827160 28
CTTCCACTTCTTCTGGCTCTTCTGTTTCCGCAATTA

**** ********* ***** * *** *******
Consensus:

CTTCcACTTCTTCTgaCTCTTctGtTTCcGCAATTA

>Bacillus_Fam_129_29_5 Nr. of seq. 5 Alignment length(with gaps) = 30 Alignment score = 0.824444
GCF_900107275.1_IMG-taxon_2675903024_annotated_assembly_genomic.fna_16:61242-61589 Satlength=348 Nr of Repeats=5 RepeatLength=29 seed=GAGCAGCGGA Num.seqs=3 Similarity=1.000000 0
GAGCAGCGGAGCGTACATCACA-CGTACGT
GCF_900107275.1_IMG-taxon_2675903024_annotated_assembly_genomic.fna_17:131859-132214 Satlength=356 Nr of Repeats=5 RepeatLength=29 seed=GAGCAGCGGA Num.seqs=3 Similarity=0.908046 0 GAGCAGCGGAGCGTACAAGGAA-CGTACGA
Rev.of_GCF_900107275.1_IMG-taxon_2675903024_annotated_assembly_genomic.fna_32:48473-49200 Satlength=728 Nr of Repeats=5 RepeatLength=29 seed=CTCCGCTGCT Num.seqs=3 Similarity=1.000000 11 GAGCAGCGGAGCGTACAAGGGA-CGTACGT
GCF_900107275.1_IMG-taxon_2675903024_annotated_assembly_genomic.fna_51:15077-15428 Satlength=352 Nr of Repeats=5 RepeatLength=29 seed=TGAGCAGCGC Num.seqs=3 Similarity=0.969349 28
GAGCAGCGCAGCGTACAAGGGA-CGTACGT
Rev.of_GCF_900107275.1_IMG-taxon_2675903024_annotated_assembly_genomic.fna_43:86875-87225 Satlength=351 Nr of Repeats=5 RepeatLength=30 seed=CTCCGCTGCT Num.seqs=3 Similarity=0.970370 11
GAGCAGCGGAGCGTACAAGAGATCGTACGT

******** ********   * ******
Consensus:

GAGCAGCGgAGCGTACAagggACGTACGt

>Bacillus_Fam_130_21_5 Nr. of seq. 5 Alignment length(with gaps) = 24 Alignment score = 0.626389
GCF_000813125.1_ASM81312v1_genomic.fna_65:36039-36144 Satlength=106 Nr of Repeats=5 RepeatLength=21 seed=TCGACCACAT Num.seqs=5 Similarity=0.733333
0 TCGACCACATTCC--AAG-ATAAT
Rev.of_GCF_000813125.1_ASM81312v1_genomic.fna_109:33798-33903 Satlength=106 Nr of Repeats=5 RepeatLength=21 seed=TGGTCGAATT Num.seqs=5

Similarity=0.707937 7
TCGACCACATNTC-NAAT--TAAT
Rev.of_GCF_000813125.1_ASM81312v1_genomic.fna_40:56922-57048
Satlength=127 Nr of Repeats=6 RepeatLength=21 seed=ATGTGGTTGA Num.seqs=6
Similarity=0.778283 10
TCGACCACATTTT-GAANT-TAAT
GCF_001315165.1_ASM131516v1_genomic.fna_25:29879-29963 Satlength=85 Nr of Repeats=4 RepeatLength=21 seed=AATAATTCGA Num.seqs=4 Similarity=0.828283
16 TCGACCACATTTTCGAAA--TAAT
Rev.of_GCF_000813125.1_ASM81312v1_genomic.fna_144:1991-2075 Satlength=85 Nr of Repeats=4 RepeatLength=21 seed=TGGTCGAATT Num.seqs=4
Similarity=0.756614 7
TCGACCAGATTTA-GGAT--TAAT

******* ** * ****
Consensus:

TCGACCAcATttngaAnTAAT

>Bacillus_Fam_131_24_5 Nr. of seq. 5 Alignment length(with gaps) = 24
Alignment score = 0.822222
GCF_000007845.1_ASM784v1_genomic.fna_1:1480940-1481189 Satlength=250 Nr of Repeats=8 RepeatLength=24 seed=AGCTTCTTCT Num.seqs=5
Similarity=0.822667
0 AGCTTCTTCTTCAGCTTTTTTTGT
GCF_000008505.1_ASM850v1_genomic.fna_1:1505384-1505633 Satlength=250 Nr of Repeats=8 RepeatLength=24 seed=AGCTTCTTCT Num.seqs=5
Similarity=0.822667
0 AGCTTCTTCTTCAGCTTTTTTTGT
GCF_000496285.1_ASM49628v1_genomic.fna_1:4094822-4095035 Satlength=214 Nr of Repeats=5 RepeatLength=24 seed=AGCTTCTTCT Num.seqs=3
Similarity=0.795556 0
AGCTTCTTCTTCAGCTTTTTTCTT
Rev.of_GCF_000832605.1_ASM83260v1_genomic.fna_1:4215062-4215365
Satlength=304 Nr of Repeats=6 RepeatLength=24 seed=GCTGACGAAG Num.seqs=4
Similarity=0.851852 15
AGCTTCTTCGTCAGCTTTTTTCTT
GCF_000175075.1_ASM17507v1_genomic.fna_2:117151-117274 Satlength=124 Nr of Repeats=5 RepeatLength=24 seed=GCTTCTTTCT Num.seqs=3
Similarity=0.925926
13 AGCTTGCTCTTCGGCTTCTTTCTT

***** ** ** **** *** *
Consensus:

AGCTTctTCtTCaGCTTtTTTctT

>Bacillus_Fam_132_21_5 Nr. of seq. 5 Alignment length(with gaps) = 23
Alignment score = 0.630435
GCF_000242895.2_ASM24289v3_genomic.fna_1:1703717-1703819 Satlength=103 Nr of Repeats=5 RepeatLength=20 seed=AGTCGAGCAT Num.seqs=3
Similarity=0.866667 0
AGTCGAGCATA-AAACCT-GAG-

GCF_000242895.2_ASM24289v3_genomic.fna_1:1503263-1503366 Satlength=104 Nr of Repeats=5 RepeatLength=21 seed=AGTCGAGCAT Num.seqs=3 Similarity=0.808081 0
AGTCGAGCATA-AACCCT-CAAA
GCF_000242895.2_ASM24289v3_genomic.fna_1:1982125-1982248 Satlength=124 Nr of Repeats=5 RepeatLength=21 seed=GTCGAGCATA Num.seqs=3 Similarity=0.690821 1
AGTCGAGCATANNACCCC-GAA-
Rev.of_GCF_000242895.2_ASM24289v3_genomic.fna_1:4665131-4665236 Satlength=106 Nr of Repeats=5 RepeatLength=21 seed=TATGCTCGAC Num.seqs=5 Similarity=0.824242 11
AGTCGAGCATA-AAT-CTCGAAA
Rev.of_GCF_000242895.2_ASM24289v3_genomic.fna_1:1951392-1951496 Satlength=105 Nr of Repeats=5 RepeatLength=21 seed=ATATATGCTC Num.seqs=4 Similarity=0.818182 14
AGTTGAGCATA-TATCCTCGAAA

*** ******* * * *
Consensus:

AGTcGAGCATAaAncCtgAaa

>Bacillus_Fam_133_21_5 Nr. of seq. 5 Alignment length(with gaps) = 23 Alignment score = 0.650725
GCF_000294775.2_ASM29477v2_genomic.fna_1:5051016-5051119 Satlength=104 Nr of Repeats=5 RepeatLength=21 seed=TGCAAATAAA Num.seqs=3 Similarity=0.440000 0
TGCAAATAAACNG-GTNGAA-CC
GCF_001712755.1_ASM171275v1_genomic.fna_2:340961-341149 Satlength=189 Nr of Repeats=9 RepeatLength=21 seed=TGCAAATAAA Num.seqs=8 Similarity=0.655329
0 TGCAAATAAAAAAGGTGAAA-C-
GCF_001712755.1_ASM171275v1_genomic.fna_37:106859-106987 Satlength=129 Nr of Repeats=6 RepeatLength=21 seed=TGCAAATAAA Num.seqs=4 Similarity=0.513889 0
TGCAAATAAAAAA-GTGAAATC-
GCF_001712755.1_ASM171275v1_genomic.fna_3:168469-168573 Satlength=105 Nr of Repeats=4 RepeatLength=21 seed=TGCAAATAAA Num.seqs=3 Similarity=0.621212
0 TGCAAATAAAAATCCTGAAA-C-
GCF_001712755.1_ASM171275v1_genomic.fna_5:11567-11647 Satlength=81 Nr of Repeats=4 RepeatLength=21 seed=TGCAAATAAA Num.seqs=3 Similarity=0.661616
0 TGCAAATAAAAATAGTAAAA-C-

********** * ** *
Consensus:

TGCAAATAAAaanngTgaAAC

>Bacillus_Fam_134_21_5 Nr. of seq. 5 Alignment length(with gaps) = 23 Alignment score = 0.628986
GCF_000508325.1_BAVI_1_genomic.fna_121:21154-21237 Satlength=84 Nr of Repeats=4 RepeatLength=21 seed=TTTATCAGCG Num.seqs=3 Similarity=0.525253
0 TTTATCAGCGGATNTN-GNNCT-

GCF_001591665.1_ASM159166v1_genomic.fna_16:4578-4661 Satlength=84 Nr of Repeats=4 RepeatLength=21 seed=TTTATCAGCG Num.seqs=3 Similarity=0.727273
0   TTTATCAGCGAATTTC-ACGCT-
GCF_001591805.1_ASM159180v1_genomic.fna_23:27706-27789 Satlength=84 Nr of Repeats=4 RepeatLength=21 seed=TTTATCAGCG Num.seqs=3 Similarity=0.727273
0   TTTATCAGCGAATTTT-ATACT-
GCF_000508325.1_BAVI_1_genomic.fna_64:16791-16874 Satlength=84 Nr of Repeats=4 RepeatLength=21 seed=ATCAGCGAAT Num.seqs=3 Similarity=0.681818
3   TTTATCAGCGAATTTTGATAGT-
GCF_001591805.1_ASM159180v1_genomic.fna_9:281564-281647 Satlength=84 Nr of Repeats=4 RepeatLength=21 seed=TTTATCAGCG Num.seqs=3 Similarity=0.500000
0   TTTATCAGCGGAATTC-A-NCTG

********** * *   *
Consensus:

TTTATCAGCGaAttTnanncT

>Bacillus_Fam_135_21_5 Nr. of seq. 5 Alignment length(with gaps) = 23 Alignment score = 0.630435
GCF_001889165.1_ASM188916v1_genomic.fna_1:3505433-3505516 Satlength=84 Nr of Repeats=4 RepeatLength=21 seed=TTTATTTTCG Num.seqs=3 Similarity=0.626263   0
TTTATTTTCGG-NTTTGAGTCA-
GCF_001889165.1_ASM188916v1_genomic.fna_1:3842895-3843000 Satlength=106 Nr of Repeats=4 RepeatLength=21 seed=TTTATTTTCG Num.seqs=3 Similarity=0.562222   0
TTTATTTTCGA-ATTTNA-TTNG
GCF_001889165.1_ASM188916v1_genomic.fna_1:4154491-4154573 Satlength=83 Nr of Repeats=4 RepeatLength=21 seed=TTTATTTTCG Num.seqs=3 Similarity=0.671498   0
TTTATTTTCGA-ATTTGA-TCAT
GCF_001889165.1_ASM188916v1_genomic.fna_1:3860025-3860109 Satlength=85 Nr of Repeats=4 RepeatLength=21 seed=TTTATTTTCG Num.seqs=4 Similarity=0.646465   0
TTTATTTTCGANATTTCA-TCGN
Rev.of_GCF_002019605.1_ASM201960v1_genomic.fna_1:4136408-4136513 Satlength=106 Nr of Repeats=4 RepeatLength=21 seed=AAGTTGAAAA Num.seqs=3 Similarity=1.000000   14
TTTATTTTCAA-CTTTGC-TCAA

*********   ***   *
Consensus:

TTTATTTTCgaaTTTgaTcan

>Bacillus_Fam_136_20_5 Nr. of seq. 5 Alignment length(with gaps) = 22 Alignment score = 0.613636
GCF_000007845.1_ASM784v1_genomic.fna_1:4468427-4468527 Satlength=101 Nr of Repeats=5 RepeatLength=20 seed=GATATATTTT Num.seqs=5 Similarity=0.733333
0   GATAT-ATTTGATAAT-CGCT

GCF_000831065.1_ASM83106v1_genomic.fna_1:4854120-4854260 Satlength=141 Nr of Repeats=6 RepeatLength=20 seed=GATATATTTT Num.seqs=5 Similarity=0.713333 0 GATAT-ATTTTAANAAT-CGCT
Rev.of_GCF_000008505.1_ASM850v1_genomic.fna_1:4898752-4898852 Satlength=101 Nr of Repeats=5 RepeatLength=20 seed=TATATCAGCG Num.seqs=5 Similarity=0.636508 6 GATAT-ATTTNGGNAAT-CGCT
Rev.of_GCF_000008505.1_ASM850v1_genomic.fna_1:2760175-2760255 Satlength=81 Nr of Repeats=4 RepeatLength=20 seed=TATATCAGCG Num.seqs=4 Similarity=0.555556 6 GATATATATTAG-AAATNCGCT
GCF_000290995.1_Baci_cere_AND1407_G13175_V1_genomic.fna_4:613849-613969 Satlength=121 Nr of Repeats=5 RepeatLength=20 seed=GATATAATTT Num.seqs=4 Similarity=0.687831 22 GATAT-AATTAGAAAATGCGCC

***** ** *** ***
Consensus:

GATATatTTnganAATCGCt

>Bacillus_Fam_137_20_5 Nr. of seq. 5 Alignment length(with gaps) = 22 Alignment score = 0.633333
GCF_000242895.2_ASM24289v3_genomic.fna_1:4197965-4198045 Satlength=81 Nr of Repeats=4 RepeatLength=20 seed=CCGATATAAT Num.seqs=4 Similarity=0.711640
0 CCGATATA-ATCGAGATTTTA-
Rev.of_GCF_000311725.1_ASM31172v1_genomic.fna_2:106659-106759 Satlength=101 Nr of Repeats=5 RepeatLength=20 seed=TATATCGGCG Num.seqs=5 Similarity=0.552174 8 CCGATATA-ATT-AAATTTTCG
GCF_001578185.1_ASM157818v1_genomic.fna_1:4205454-4205534 Satlength=81 Nr of Repeats=4 RepeatLength=20 seed=AGATATAATT Num.seqs=4 Similarity=0.822222 21 CAGATATA-ATT-CGATTTTCG
Rev.of_GCF_000311725.1_ASM31172v1_genomic.fna_2:362483-362583 Satlength=101 Nr of Repeats=5 RepeatLength=20 seed=TATATCGGCG Num.seqs=3 Similarity=0.525253 8 CCGATATA-NTC-AGAATATCG
GCF_000311725.1_ASM31172v1_genomic.fna_4:83330-83410 Satlength=81 Nr of Repeats=4 RepeatLength=20 seed=CGCCGATATA Num.seqs=4 Similarity=0.603535
19 CCGATATATCTC-AGATTTTCG

* ****** * * * *
Consensus:

CcGATATAaTcagAtTtTcg

>Bacillus_Fam_138_20_5 Nr. of seq. 5 Alignment length(with gaps) = 22 Alignment score = 0.601515
GCF_000401235.1_BacNeaAAU1_genomic.fna_37:7-109 Satlength=103 Nr of Repeats=5 RepeatLength=20 seed=TATCAGCCAA Num.seqs=3 Similarity=1.000000
0 TATC-AGCCAAACTTTCC-ATT

GCF_001591585.1_ASM159158v1_genomic.fna_21:18-404 Satlength=387 Nr of Repeats=19 RepeatLength=20 seed=TCAGCCAAAC Num.seqs=13 Similarity=0.965812
2   TTTC-AGCCAAACTTCAG-ATT
GCF_001591585.1_ASM159158v1_genomic.fna_43:11976-12101 Satlength=126 Nr of Repeats=6 RepeatLength=21 seed=TATCAGCCAA Num.seqs=5 Similarity=0.784127
21   TATC-AGCCAAACTTCAGTACT
GCF_000508325.1_BAVI_1_genomic.fna_156:10891-11119 Satlength=229 Nr of Repeats=7 RepeatLength=21 seed=ATCTCGCCAA Num.seqs=5 Similarity=0.556522
1   NATCTCGCCAAACTTNGA-ATT
Rev.of_GCF_000508325.1_BAVI_1_genomic.fna_199:5141-5284 Satlength=144 Nr of Repeats=5 RepeatLength=21 seed=TTGGCGAGAT Num.seqs=3 Similarity=0.505050
11   TATCTCGCCAAACTTTNA-GTT

** *********   *
Consensus:

taTCaGCCAAACTTnnnatT

>Bacillus_Fam_139_21_5 Nr. of seq. 5 Alignment length(with gaps) = 22 Alignment score = 0.645455
GCF_001375535.1_Bacillus_niameyensis_genomic.fna_6:1986285-1988125 Satlength=1841 Nr of Repeats=92 RepeatLength=20 seed=TTCGGATTTT Num.seqs=92 Similarity=1.000000   0
TTCG-GATTTTATGAGCGAGA-
Rev.of_GCF_001375535.1_Bacillus_niameyensis_genomic.fna_6:3077746-3079526 Satlength=1781 Nr of Repeats=89 RepeatLength=20 seed=CTCGCTCATA Num.seqs=89 Similarity=1.000000   19
TTCG-GATTTTATGAGCGAGT-
Rev.of_GCF_001375535.1_Bacillus_niameyensis_genomic.fna_6:2460775-2461355 Satlength=581 Nr of Repeats=28 RepeatLength=20 seed=AGATCTCGCT Num.seqs=25 Similarity=0.779524   2   CT-
GTGA-TATATGAGCGAGAT
GCF_001375535.1_Bacillus_niameyensis_genomic.fna_6:4065733-4066073 Satlength=341 Nr of Repeats=17 RepeatLength=20 seed=GATATATGAG Num.seqs=17 Similarity=0.847059   5
-TCGTGA-TATATGAGCAAGAA
GCF_001375535.1_Bacillus_niameyensis_genomic.fna_6:4271828-4272328 Satlength=501 Nr of Repeats=25 RepeatLength=20 seed=ATATGAGCGA Num.seqs=25 Similarity=0.836000   8
-TCGNGA-TATATGAGCGAGAT

* * ** * ******* **
Consensus:

nTcGnGATaTATGAGCgAGan

>Bacillus_Fam_140_22_5 Nr. of seq. 5 Alignment length(with gaps) = 22 Alignment score = 0.630303
GCF_001315045.1_ASM131504v1_genomic.fna_21:8-820 Satlength=813 Nr of Repeats=5 RepeatLength=21 seed=TTTATTGTCC Num.seqs=4 Similarity=0.530193
0   TTTATTGTCC-GATGACCCACA

Rev.of_GCF_001315045.1_ASM131504v1_genomic.fna_25:32585-32669
Satlength=85 Nr of Repeats=4 RepeatLength=21 seed=GGACAATAAA Num.seqs=4
Similarity=0.622475 10
TTTATTGTCC-GATTCGCGCCA
Rev.of_GCF_001315045.1_ASM131504v1_genomic.fna_35:25565-25649
Satlength=85 Nr of Repeats=4 RepeatLength=21 seed=GGACAATAAA Num.seqs=4
Similarity=0.696970 10
TTTATTGTCCAGATGAGCTCCA
Rev.of_GCF_001315045.1_ASM131504v1_genomic.fna_25:36402-36485
Satlength=84 Nr of Repeats=4 RepeatLength=21 seed=GGACAATAAA Num.seqs=3
Similarity=0.462963 10
TTTATTGTCCNGTT-CGTNACA
GCF_001315045.1_ASM131504v1_genomic.fna_150:1706-1790 Satlength=85 Nr of Repeats=4 RepeatLength=21 seed=TTTATTGTCC Num.seqs=4 Similarity=0.445473
21 TTTATTGTCCAGAT-CACAGCA

********** * * **
Consensus:

TTTATTGTCCnGaTncgcnnCA

>Bacillus_Fam_141_20_5 Nr. of seq. 5 Alignment length(with gaps) = 21
Alignment score = 0.666667
GCF_000311725.1_ASM31172v1_genomic.fna_7:70953-71160 Satlength=208 Nr of Repeats=4 RepeatLength=20 seed=ATCGGTCTAA Num.seqs=3 Similarity=0.644444
0 ATCGGTCTAAT-TTCAAATAT
GCF_900156875.1_PRJEB18969_genomic.fna_10:321051-321132 Satlength=82 Nr of Repeats=4 RepeatLength=20 seed=TATATCGGTC Num.seqs=3
Similarity=0.671958
17 ATCGGTCAGAT-TTNNAATAT
GCF_002019645.1_ASM201964v1_genomic.fna_1:898029-898267 Satlength=239 Nr of Repeats=12 RepeatLength=20 seed=TATCGGTCTA Num.seqs=8
Similarity=0.771429 19
ATCGGTCTAAC-TAGAAAAAT
GCF_002019645.1_ASM201964v1_genomic.fna_1:1325761-1326158 Satlength=398 Nr of Repeats=20 RepeatLength=20 seed=TATCGGTCTA Num.seqs=17
Similarity=0.704482 19
ATCGGTCTAAC-TCAAGATAT
GCF_002019645.1_ASM201964v1_genomic.fna_1:1553541-1553821 Satlength=281 Nr of Repeats=13 RepeatLength=20 seed=TATCGGTCTA Num.seqs=12
Similarity=0.763348 19
ATCGGTCTAACTTTAAGATAT

******* * * * **
Consensus:

ATCGGTCtaAcTtnaaAtAT

>Bacillus_Fam_142_20_5 Nr. of seq. 5 Alignment length(with gaps) = 20
Alignment score = 0.746667
GCF_001375535.1_Bacillus_niameyensis_genomic.fna_6:224994-228602
Satlength=3609 Nr of Repeats=180 RepeatLength=20 seed=TTTCTCTATC
Num.seqs=179 Similarity=0.998519 0
TTTCTCTATCCAAGAGTATT

GCF_001375535.1_Bacillus_niameyensis_genomic.fna_6:1933173-1933473 Satlength=301 Nr of Repeats=15 RepeatLength=20 seed=ATACGAGAGT Num.seqs=15 Similarity=0.818292 7
CCTCGCTATACGAGAGTATC
GCF_001375535.1_Bacillus_niameyensis_genomic.fna_6:2574155-2574275 Satlength=121 Nr of Repeats=6 RepeatLength=20 seed=GAGAGTATTT Num.seqs=6 Similarity=0.853333 11
TCACGCTATACGAGAGTATT
GCF_001375535.1_Bacillus_niameyensis_genomic.fna_6:3800918-3801078 Satlength=161 Nr of Repeats=7 RepeatLength=20 seed=GAGAGTATTT Num.seqs=6 Similarity=0.675556 11
TCGCGCTATACGAGAGTATT
GCF_001375535.1_Bacillus_niameyensis_genomic.fna_6:2578467-2578567 Satlength=101 Nr of Repeats=5 RepeatLength=20 seed=GAGAGTATTT Num.seqs=5 Similarity=0.726667 11
TCTCGCTATGTGAGAGTATT

* ****   *******
Consensus:

tctCgCTATacgAGAGTATt

>Bacillus_Fam_143_20_5 Nr. of seq. 5 Alignment length(with gaps) = 20 Alignment score = 0.686667
GCF_001591645.1_ASM159164v1_genomic.fna_5:153867-154047 Satlength=181 Nr of Repeats=9 RepeatLength=20 seed=CGCAAATAAA Num.seqs=7 Similarity=0.746032
0 CGCAAATAAATTCTCAGGAT
GCF_001712755.1_ASM171275v1_genomic.fna_5:39192-39789 Satlength=598 Nr of Repeats=27 RepeatLength=20 seed=GCAAATAAAA Num.seqs=22 Similarity=0.724675
1 GGCAAATAAAATCNTGGAAT
Rev.of_GCF_001712755.1_ASM171275v1_genomic.fna_35:198476-198815 Satlength=340 Nr of Repeats=17 RepeatLength=20 seed=TTTTATTTGC Num.seqs=16 Similarity=0.675694
11 CGCAAATAAAAATTGGGAAT
GCF_001712755.1_ASM171275v1_genomic.fna_37:245563-245843 Satlength=281 Nr of Repeats=11 RepeatLength=20 seed=ATCGCAAATA Num.seqs=9 Similarity=0.706349 18
CGCAAATAAAAACTGGGAAT
GCF_001712755.1_ASM171275v1_genomic.fna_10:18587-18827 Satlength=241 Nr of Repeats=11 RepeatLength=20 seed=GAATCGCAAA Num.seqs=10 Similarity=0.716755 16
CGCAAATAAAANGGCCGAAT

*********   * **
Consensus:

cGCAAATAAAanctngGaAT

>Bacillus_Fam_144_18_5 Nr. of seq. 5 Alignment length(with gaps) = 18 Alignment score = 0.718518
GCF_000290995.1_Baci_cere_AND1407_G13175_V1_genomic.fna_1:1402612-1402892 Satlength=281 Nr of Repeats=11 RepeatLength=18 seed=GGAGCAACAG

Num.seqs=7 Similarity=0.816579 0
GGAGCAACAGGCCCAACA
GCF_900156865.1_PRJEB18960_genomic.fna_3:459675-459846 Satlength=172 Nr of Repeats=10 RepeatLength=18 seed=GGCCCAACAG Num.seqs=8 Similarity=0.896825
9 GGTCCAACAGGCCCAACA
GCF_000408885.1_ASM40888v1_genomic.fna_1:903316-904711 Satlength=1396 Nr of Repeats=62 RepeatLength=18 seed=GGAGCAACCG Num.seqs=39 Similarity=0.802069 0
GGAGCAACCGGNCCGACA
GCF_000712615.1_ASM71261v1_genomic.fna_9:136496-136568 Satlength=73 Nr of Repeats=4 RepeatLength=18 seed=GGAGAAACCG Num.seqs=4 Similarity=0.913580
0 GGAGAAACCGGCCCAACG
Rev.of_GCF_900096965.1_IMG-taxon_2671180223_annotated_assembly_genomic.fna_5:86873-87080 Satlength=208 Nr of Repeats=9 RepeatLength=18 seed=CACCCGTTGG Num.seqs=6 Similarity=0.935802 4 GGTGTAACGGGCCCAACG

** *** ** ** **
Consensus:

GGagcAACnGGcCCaACa

>Bacillus_Fam_145_12_5 Nr. of seq. 5 Alignment length(with gaps) = 12 Alignment score = 0.811111
GCF_000153365.1_ASM15336v1_genomic.fna_16:276957-277038 Satlength=82 Nr of Repeats=6 RepeatLength=12 seed=GAAGACGAAG Num.seqs=4 Similarity=0.851852
0 GAAGACGAAGAT
GCF_000153365.1_ASM15336v1_genomic.fna_16:276984-277131 Satlength=148 Nr of Repeats=12 RepeatLength=12 seed=GAAGATGAAG Num.seqs=9 Similarity=0.833333 0
GAAGATGAAGAT
Rev.of_GCF_000473245.1_ASM47324v1_genomic.fna_1:2532988-2533135 Satlength=148 Nr of Repeats=11 RepeatLength=12 seed=TCTTCATCTT Num.seqs=7 Similarity=0.830688
5 GAAGATGAAGAT
GCF_000299035.1_ASM29903v1_genomic.fna_1:1278771-1279065 Satlength=295 Nr of Repeats=21 RepeatLength=12 seed=GAAGATGAAG Num.seqs=16 Similarity=0.892593 0
GAAGAAGAAGAA
Rev.of_GCF_000956595.1_ASM95659v1_genomic.fna_52:22021-22282 Satlength=262 Nr of Repeats=21 RepeatLength=12 seed=TCTTCCTCTT Num.seqs=13 Similarity=0.931624
2 GAAGAAGANGAA

***** ** **
Consensus:

GAAGAnGAaGAt

>Bacillus_Fam_146_52_4 Nr. of seq. 4 Alignment length(with gaps) = 58 Alignment score = 0.652778

GCF_000186145.1_Bacillus_sp_2_A_57_CT2_V1_genomic.fna_6:238667-238927
Satlength=261 Nr of Repeats=5 RepeatLength=52 seed=GTTCGGACTG Num.seqs=5
Similarity=0.796226 18
TGTCCGAAGTTGCATC-AG--GTTCGGACT-GCT-CTGA-ACGGGTTTCCTCTTCTCC
Rev.of_GCF_000294775.2_ASM29477v2_genomic.fna_1:859052-859364
Satlength=313 Nr of Repeats=6 RepeatLength=52 seed=ACTTCGGACA Num.seqs=6
Similarity=0.783857 62
TGTCCGAAGTTGCATCTAG--GTTCGGACT-GCT-CTGA-ACGGGTTTCTTCTTCTCC
GCF_000186145.1_Bacillus_sp_2_A_57_CT2_V1_genomic.fna_14:48997-49307
Satlength=311 Nr of Repeats=6 RepeatLength=52 seed=GTTCGGACTC Num.seqs=5
Similarity=0.762963 18
TGTCCGAAGTTGCACC-GG--GTTCGGACTCTCTCCTG---CGGATTTCTTCTTCTCC
Rev.of_GCF_000294775.2_ASM29477v2_genomic.fna_1:477876-478136
Satlength=261 Nr of Repeats=5 RepeatLength=52 seed=GTGAGTCCGA Num.seqs=5
Similarity=0.866667 29
TGTCCGAATAAGCA-C-TGTG-TTCGGACT--CA-CCAACTCGGTTTTCTTCTTCTCC

******** * * *** **** *************** *** * *
Consensus:

gTTCGGACTgCtCtgaaCGGgTTTCtTCTTCTCCTGTCCGAAgttGCAtCaG

>Bacillus_Fam_147_54_4 Nr. of seq. 4 Alignment length(with gaps) = 57
Alignment score = 0.632554
GCF_000401235.1_BacNeaAAU1_genomic.fna_155:14912-15172 Satlength=261 Nr of Repeats=5 RepeatLength=52 seed=TTCATCCACT Num.seqs=5
Similarity=0.791195
0 TTCATCCACTCGATGAACACCACTTTTCTCCT--G-CTTCCTCTACTT--TTTGGTG
GCF_001591585.1_ASM159158v1_genomic.fna_2:273831-274142 Satlength=312 Nr of Repeats=6 RepeatLength=52 seed=CACTTTTCCT Num.seqs=5
Similarity=0.886792
20 TTCATCCCTTTGATGAAAACCACTTTT--CCT--G-CTTCTTCTGGTTATTTTGGTG
Rev.of_GCF_001591585.1_ASM159158v1_genomic.fna_18:26242-26552
Satlength=311 Nr of Repeats=6 RepeatLength=52 seed=TGGTGTTCAT Num.seqs=5
Similarity=0.784615 22
TTCATCCGCTTGATGAACACCACTTTT-TCCTCNGCCTTC--CTGGAT--TGTGGTT
Rev.of_GCF_001591585.1_ASM159158v1_genomic.fna_18:28113-28373
Satlength=261 Nr of Repeats=5 RepeatLength=52 seed=GTTCATCAAG Num.seqs=5
Similarity=0.817949 70
TTCATTTGCTTGATGAACACCATTTTT-TCCTCGGTCTTC--CTGGAT--TGTGGTG

***** * ****** **** **** *** * **** ** * * ****
Consensus:

TTCATccgcTtGATGAAcACCAcTTTTtCCTcnGnCTTCntCTggaTTgTGGTg

>Bacillus_Fam_148_52_4 Nr. of seq. 4 Alignment length(with gaps) = 57
Alignment score = 0.626218
GCF_000759675.1_ASM75967v1_genomic.fna_13:55319-55837 Satlength=519 Nr of Repeats=10 RepeatLength=52 seed=GCTCTTGGTG Num.seqs=8
Similarity=0.807952
0 GCTCTTGGTGACCGATA-TCCAAGGGAA-ATCG-AA-ATTGTGGTCACCAAGG-AGT
Rev.of_GCF_000759675.1_ASM75967v1_genomic.fna_151:356127-356582
Satlength=456 Nr of Repeats=4 RepeatLength=52 seed=GATTTTCCTT Num.seqs=3

Similarity=0.914530 30
GCTCTTGGTGACCGAAA-TCAAAGGAAA-ATCG-AA-AAAGTGGTAACCAAGA-AGG
Rev.of_GCF_000759675.1_ASM75967v1_genomic.fna_164:44908-45116
Satlength=209 Nr of Repeats=4 RepeatLength=52 seed=GTCATCAAGA Num.seqs=4
Similarity=0.854701 12
GCTCTTGATGACCGAAA-TCC--GGAAA-AAAGTAAGAAAGTGGTTACCAAGA-AGG
Rev.of_GCF_000759675.1_ASM75967v1_genomic.fna_57:123377-123693
Satlength=317 Nr of Repeats=6 RepeatLength=53 seed=ATTGGTTACC Num.seqs=4
Similarity=0.890985 48 -
CTATTGGTGACCGAAACAGCAAGGGAAGATAG--A-AAAGTGGTAACCAATAGAGG

** *** ******* * ** ** * * * * ***** ***** **
Consensus:

gCTcTTGgTGACCGAaAtccaaGGaAAAtaGaAAaaGTGGTaACCAAgaAGg

>Bacillus_Fam_149_53_4 Nr. of seq. 4 Alignment length(with gaps) = 57
Alignment score = 0.627680
GCF_002019645.1_ASM201964v1_genomic.fna_1:1958292-1959384 Satlength=1093
Nr of Repeats=21 RepeatLength=52 seed=GACAGCTTTG Num.seqs=21
Similarity=0.736162 48
GCTTTGGCT-ATATCAACA-A-GAAAGCTGTCCGAACCT-AGG-TAGACTTCGGACA
GCF_002019645.1_ASM201964v1_genomic.fna_1:1960889-1961149 Satlength=261
Nr of Repeats=5 RepeatLength=52 seed=AAGCTGTCCG Num.seqs=5
Similarity=0.846541 73
GCTTTGGCT-ANATCAACA-A-GAAAGCTGTCCGAACCT-AGG-TAGACTTCGGACA
Rev.of_GCF_002019645.1_ASM201964v1_genomic.fna_1:5249431-5249898
Satlength=468 Nr of Repeats=9 RepeatLength=52 seed=CTGTTCAGAC Num.seqs=8
Similarity=0.826923 88
GCTTTAGCT-AGA--AAAAGAGGGAAGCTGTCTGAACAG-AGCGCA-ACTTCGGACA
GCF_002019645.1_ASM201964v1_genomic.fna_1:5267087-5267710 Satlength=624
Nr of Repeats=11 RepeatLength=52 seed=ACTTCGGACA Num.seqs=10
Similarity=0.699802 94
GCTTCAGCNCTCA--AACAGA-GAAAGCTGTCCGAACACAAGC-CA-ACTTCGGACA

******** ** * ** * * * ******** **** ** * ******
Consensus:

GACAGCTTtaGCtanAtcAAcAgAGaAAGCTGTCcGAACatAGccAgACTTCG

>Bacillus_Fam_150_53_4 Nr. of seq. 4 Alignment length(with gaps) = 56
Alignment score = 0.619048
GCF_000294775.2_ASM29477v2_genomic.fna_1:4687252-4687712 Satlength=461 Nr of Repeats=9 RepeatLength=51 seed=GATTAACTAC Num.seqs=8
Similarity=0.729654 39
TTTT--TCTT--TGCACCCCTT-ATTTTCGGCGTTAATCCCTCCGATTAACTACCA
GCF_000186145.1_Bacillus_sp_2_A_57_CT2_V1_genomic.fna_3:854190-854829
Satlength=640 Nr of Repeats=12 RepeatLength=52 seed=ATTAACTCCC
Num.seqs=9 Similarity=0.824074 40
TTTTC-TCTT-GAGCA--CCTTCATTTTGGGCGTTATTCCCACTGATTAACTCCCA
Rev.of_GCF_000186145.1_Bacillus_sp_2_A_57_CT2_V1_genomic.fna_5:200856-201375 Satlength=520 Nr of Repeats=10 RepeatLength=52 seed=GACGAAATTG
Num.seqs=9 Similarity=0.878205 79
TTTTCGCCTTACTCCA---CTC-AATTTCGTCGTTATTCCATGTGATTAACTACCA

GCF_000294775.2_ASM29477v2_genomic.fna_1:2711828-2712087 Satlength=260 Nr of Repeats=5 RepeatLength=52 seed=ATTAACTACC Num.seqs=4 Similarity=0.772013 92
TTTTCGCCTTACTCCA---CTC-AATTTCGTCGTAAATCCATGTGATTAACTACCA

******** ******* *** ** ** * *** * *** * ***
Consensus:

GATTAACTaCCATTTTcgcCTTactcCAcCTcAaTTTcGgCGTtAaTCCatct

>Bacillus_Fam_151_52_4 Nr. of seq. 4 Alignment length(with gaps) = 56 Alignment score = 0.669643
GCF_000169195.2_ASM16919v2_genomic.fna_1:485549-485757 Satlength=209 Nr of Repeats=4 RepeatLength=52 seed=TTATGAAGGA Num.seqs=4 Similarity=0.794872
0 TTATGAAGGACATTTCAGACT--GCGG--TTTGCACCCTTTTGTCCTTCATCCCGC
GCF_000169195.2_ASM16919v2_genomic.fna_1:2293568-2294088 Satlength=521 Nr of Repeats=10 RepeatLength=52 seed=TTTGTCCTTC Num.seqs=10 Similarity=0.789937 35
TTATGAAGGACATTTCAGCTT--GCGG--TTCGTCCCCTTTTGTCCTTCATCCCGT
Rev.of_GCF_000832905.1_ASM83290v1_genomic.fna_1:2365732-2366096 Satlength=365 Nr of Repeats=7 RepeatLength=52 seed=CGGGATGAAG Num.seqs=7 Similarity=0.912088 51
TTATGAAGGACATTTCAACCT--GCAG--CTCGCNACCTTTTGTCCTTCATCCCGC
Rev.of_GCF_001591485.1_ASM159148v1_genomic.fna_169:2077-2544 Satlength=468 Nr of Repeats=9 RepeatLength=52 seed=ATGAAGGACA Num.seqs=6 Similarity=0.583236 47
TTATGAAGGACAATTC---TTGNGCGGCNTTCATAA-CATTTGTCCTTCATCCGGC

************ *** * ** * * * ************** *
Consensus:

TTATGAAGGACAtTTCagccTGCgGtTcgcaacCtTTTGTCCTTCATCCcGc

>Bacillus_Fam_152_53_4 Nr. of seq. 4 Alignment length(with gaps) = 56 Alignment score = 0.633929
GCF_000177235.2_ASM17723v2_genomic.fna_1:299755-299963 Satlength=209 Nr of Repeats=4 RepeatLength=52 seed=CTGAAGGTCA Num.seqs=4 Similarity=0.867521
0 CTGAAGGTCATCATA-GAAGCG-CAATGCTTACCGAGAAAACAAGA-AGAAG-CAC
GCF_000177235.2_ASM17723v2_genomic.fna_1:1015273-1015845 Satlength=573 Nr of Repeats=11 RepeatLength=52 seed=AAAGCAGAAA Num.seqs=11 Similarity=0.933800 34
-TGAAGGTCATCATACG-AG-GTCAATGCTACCCGAAAAAGCAGAAAAAATG-TTC
Rev.of_GCF_000177235.2_ASM17723v2_genomic.fna_1:4124120-4124692 Satlength=573 Nr of Repeats=10 RepeatLength=52 seed=CTTTCTCGGG Num.seqs=9 Similarity=0.791495
38 -TGAAGGTCATCATACGAAG-G-CAATGCTACCCGAGAAAGTAAAACTGAAG-CGN
Rev.of_GCF_000177235.2_ASM17723v2_genomic.fna_1:4072932-4073556 Satlength=625 Nr of Repeats=12 RepeatLength=52 seed=GTATGATGAC Num.seqs=12 Similarity=0.743874
67 --GACGGTCATCATACGAAG-GNCAATGCTGCCCGAGAAATCAAAACTGAAGTCGC

** ********* * ** * ******* **** *** * * * *

Consensus:

tGAaGGTCATCATAcGaAGGnCAATGCTacCCGAgAAAgcAaaAcagAaGcgc

>Bacillus_Fam_153_52_4 Nr. of seq. 4 Alignment length(with gaps) = 56
Alignment score = 0.611607
GCF_000759675.1_ASM75967v1_genomic.fna_126:182958-183375 Satlength=418 Nr of Repeats=8 RepeatLength=52 seed=CTTTTCATTT Num.seqs=6
Similarity=0.836897 0
CTTTTCATTTTCGATTTCGGTCACTCCAGACCTCCTGGAGTTACC--C-AATC-AT
GCF_000759675.1_ASM75967v1_genomic.fna_151:609047-609411 Satlength=365 Nr of Repeats=7 RepeatLength=52 seed=TCTTCGGTCA Num.seqs=7
Similarity=0.926740 12
-TTTTCATTTCTGTCTTCGGTCACTCCAGACCACCTGCATTGACC--CAAATC-CT
GCF_000759675.1_ASM75967v1_genomic.fna_151:66007-66371 Satlength=365 Nr of Repeats=6 RepeatLength=52 seed=TTTTCGGTCA Num.seqs=5
Similarity=0.872956
13 CTCTTCTTTTCACTTTTCGGTCACTCCAGACCCCTTGGAGTGACC--C-AA-CTTT
GCF_000759675.1_ASM75967v1_genomic.fna_151:108736-108996 Satlength=261 Nr of Repeats=5 RepeatLength=52 seed=TTCGGTCACT Num.seqs=5
Similarity=0.948718 15
CTCTTCTTTTTATGTTTCGGTCACTCCAGCACCCTTGGAGTGACCGTG-AA-C--T

* *** *** ************** * * ** * * *** ** * *
Consensus:

cTcTTCaTTTcagttTTCGGTCACTCCAGacCcCcTGgAgTgACCcAAtCnT

>Bacillus_Fam_154_53_4 Nr. of seq. 4 Alignment length(with gaps) = 56
Alignment score = 0.640377
GCF_001636315.1_ASM163631v1_genomic.fna_1:122623-123141 Satlength=519 Nr of Repeats=9 RepeatLength=52 seed=TCTACGTTAC Num.seqs=6
Similarity=0.791195
0 TCTACGTTACCCTACAGCCTTTCTT-TTC-ACTCT-TTCGGTCACGTAGAC-CCCT
GCF_001636315.1_ASM163631v1_genomic.fna_1:2481435-2482161 Satlength=727 Nr of Repeats=14 RepeatLength=52 seed=TCTACGTTAC Num.seqs=12
Similarity=0.829140 0
TCTACGTTACCCTTGACCCTTATTT-TTT-AC-CTGATCGGGCACGTAGACACCC-
GCF_001636315.1_ASM163631v1_genomic.fna_1:2317829-2318037 Satlength=209 Nr of Repeats=4 RepeatLength=52 seed=CACGTAGAAC Num.seqs=4
Similarity=0.810273 39
TCTACGTTACCCATCTGCTTGATTT-TTCGAC-CT-TTCGGGCACGTAGA-ACCCT
Rev.of_GCF_001636315.1_ASM163631v1_genomic.fna_1:3718417-3718832 Satlength=416 Nr of Repeats=8 RepeatLength=52 seed=GGTAACGTAG Num.seqs=7
Similarity=0.846062 63
TCTACGTTACCC-TCCTCTTCTTTTCTTCGAC-CG-TTCGGGCACGTAGACACCC-

************ * * ** ** ** * **** ******** ***
Consensus:

TCTACGTTACCCttcagCcTtatTTTTcgACCttTCGGgCACGTAGAcaCCCt

>Bacillus_Fam_155_54_4 Nr. of seq. 4 Alignment length(with gaps) = 56
Alignment score = 0.630952

GCF_001636325.1_ASM163632v1_genomic.fna_1:1582614-1583134 Satlength=521 Nr of Repeats=10 RepeatLength=52 seed=CCTGAAACAC Num.seqs=10 Similarity=0.912251 0
CCTGAAACACTCTGCGATCAC-TC-T-CTTCAGAGGAATAAAT-CATCTTTATTCA
Rev.of_GCF_001636325.1_ASM163632v1_genomic.fna_2:51162-51682 Satlength=521 Nr of Repeats=10 RepeatLength=52 seed=GAATAAAGAT Num.seqs=10 Similarity=0.944729
51 CCTGAAACACTCTGCAATCAC-TC-T-CTTCAGAGGAATAAAT-CATCTTTATTCA
GCF_001636325.1_ASM163632v1_genomic.fna_1:2294800-2295110 Satlength=311 Nr of Repeats=6 RepeatLength=52 seed=TTCACCTGAA Num.seqs=4 Similarity=0.858974 48
CCTGAAGCGC--TCCGAT-TCTTCTTCCTTCAGGTGAATAAACGCA-CTTTATTCA
Rev.of_GCF_001636325.1_ASM163632v1_genomic.fna_3:1067228-1067852 Satlength=625 Nr of Repeats=12 RepeatLength=52 seed=GAAGCAGAGA Num.seqs=12 Similarity=0.882673
80 CCTGAAGCGCTTTCTTAT-TC-TC-TGCTTCAGAGGAATAAATGCG-CGTTATCCA

****** * * * ** * ** * ****** ******* * * **** **
Consensus:

CCTGAAaCaCtcTccgATcaCTCTnCTTCAGagGAATAAAtgCatCtTTATtCA

>Bacillus_Fam_156_51_4 Nr. of seq. 4 Alignment length(with gaps) = 55 Alignment score = 0.623232
GCF_000292245.2_ASM29224v2_genomic.fna_2:106784-107395 Satlength=612 Nr of Repeats=12 RepeatLength=51 seed=ATTGGTAATG Num.seqs=11 Similarity=0.743396 0
-ATTGGTAATGNAATT-TTCGCTTTC--CTCCGTTTTCGGTGCGTATTACTCTGA
GCF_000292245.2_ASM29224v2_genomic.fna_60:13365-13773 Satlength=409 Nr of Repeats=8 RepeatLength=51 seed=CGGTGCGTAT Num.seqs=8 Similarity=0.820728
33 -ATTGGTAATGTAATT-TTCACTTTC-TCT-CTTTTACGGTGCGTATTATCCNCA
Rev.of_GCF_000292245.2_ASM29224v2_genomic.fna_57:11835-12090 Satlength=256 Nr of Repeats=5 RepeatLength=51 seed=CATTACCAAT Num.seqs=5 Similarity=0.562346 10
-ATTGGTAATGNANAT-TTCGNGTTCNTCT--TTTTACGGTGCGTATTAACTGGA
GCF_000292245.2_ASM29224v2_genomic.fna_35:28784-28988 Satlength=205 Nr of Repeats=4 RepeatLength=51 seed=TAATGAAAAT Num.seqs=4 Similarity=0.925926
6 TATTCGTAATGAAAATCCTGAC-TTC-TCT-CTTTTACGGTGCGTATTATCCTT-

*** ****** * * * *** ** *** ************
Consensus:

ATTgGTAATGnAaaTtTcactTTCtCTctTTTaCGGTGCGTATTAtcctga

>Bacillus_Fam_157_53_4 Nr. of seq. 4 Alignment length(with gaps) = 55 Alignment score = 0.639899
GCF_000292245.2_ASM29224v2_genomic.fna_33:5368-5572 Satlength=205 Nr of Repeats=4 RepeatLength=51 seed=CTTTTTTCGC Num.seqs=4 Similarity=0.864924
47 TTTCGCTCCCTAATTT--CTGCGATCCTCGATTCTCATTACCTATCAC-GCT-TT
GCF_000292245.2_ASM29224v2_genomic.fna_61:11632-11836 Satlength=205 Nr of Repeats=4 RepeatLength=51 seed=TTTTCATTAC Num.seqs=4 Similarity=0.895425
80 TTTCGCACCCTTATTT--CTGCAAGCCGCGATTTTCATTACCTATCAC-A-TGCT

GCF_000292245.2_ASM29224v2_genomic.fna_58:26892-27198 Satlength=307 Nr of Repeats=6 RepeatLength=51 seed=CAATTTTCAT Num.seqs=6 Similarity=0.919017
77   ATTCGCACCCTAATTTGGCTTC-A-CCTCAATTTTCATTACCAATCAC-GCT-TT
GCF_000292245.2_ASM29224v2_genomic.fna_94:1889-2195 Satlength=307 Nr of Repeats=6 RepeatLength=51 seed=ATTTTCATTA Num.seqs=6 Similarity=0.748428
79   ATTCGCACCCTAATTTGACTAC-ATACACCATTTTCATTACCTATCACAGC--TN

***** **** **** ** * * * * *** ******** *****
Consensus:

ctttaTTCGCaCCCTaATTTgnCTgCnAtcCtCgATTtTCATTACCtATCACg

>Bacillus_Fam_158_51_4 Nr. of seq. 4 Alignment length(with gaps) = 55
Alignment score = 0.638384
GCF_000186145.1_Bacillus_sp_2_A_57_CT2_V1_genomic.fna_5:259376-259584
Satlength=209 Nr of Repeats=4 RepeatLength=52 seed=AGTCCGAACC Num.seqs=4
Similarity=0.789308   0
AGTCCGAACCTGCCACGACTTCGGACAGA-AA-AAGGAGAAAAT-CCCTTGCCAG
Rev.of_GCF_000294775.2_ASM29477v2_genomic.fna_1:2652246-2652610
Satlength=365 Nr of Repeats=7 RepeatLength=52 seed=CTGTCCGAAG Num.seqs=5
Similarity=0.871795   28
AGTCCGAACCTGCCACGACTTCGGACAGT-AA-AAGGA-AAAATCCCCGTGCCAG
Rev.of_GCF_000294775.2_ASM29477v2_genomic.fna_1:2731628-2731940
Satlength=313 Nr of Repeats=6 RepeatLength=52 seed=TTCTCTGTCC Num.seqs=6
Similarity=0.847863   32
AGTCCGAACCTGGGCCAACTTCGGACAGAGAA-ATCGA-AAAAC-CCGGTGTGAG
Rev.of_GCF_001591465.1_ASM159146v1_genomic.fna_7:42047-42307
Satlength=261 Nr of Repeats=5 RepeatLength=52 seed=TTCGGACTCT Num.seqs=5
Similarity=0.720606   61
AGTCCGAACCNGGNTCAACTTCTGACACA-ACCAGCGA-AAAAA-CCGGAGTCAG

********** *   * ***** ****   * * ** **** **   * **
Consensus:

AGTCCGAACCtGccaCaACTTCgGACAgaAaAacGAAAAAtCCcgtGccAG

>Bacillus_Fam_159_52_4 Nr. of seq. 4 Alignment length(with gaps) = 55
Alignment score = 0.621212
GCF_000331575.1_ASM33157v1_genomic.fna_1:1399302-1400446 Satlength=1145
Nr of Repeats=22 RepeatLength=52 seed=GTTCGGACAC Num.seqs=22
Similarity=0.894416   0
GTTCGGACACAATTT--TGC-TACTACGGCCGTTTTCTGTCCGAACTACCACCAA
GCF_000331575.1_ASM33157v1_genomic.fna_1:1688744-1689472 Satlength=729 Nr of Repeats=14 RepeatLength=52 seed=TTTCTGTCCG Num.seqs=14
Similarity=0.876021   30
GTTCGGACTCGATTT--CGC-TTCTNCGGCCTTTTTCTGTCCGAATTCGCACCTA
Rev.of_GCF_000331575.1_ASM33157v1_genomic.fna_3:122725-123817
Satlength=1093 Nr of Repeats=21 RepeatLength=52 seed=GTCCGAACTA
Num.seqs=21 Similarity=0.898291
59   GTTCGGACTCGATTT--CGCTTTCTTCTACCTT-TTCTGTCCGAACTCACCCCTA
GCF_000331575.1_ASM33157v1_genomic.fna_1:1781562-1782030 Satlength=469 Nr of Repeats=9 RepeatLength=52 seed=TCCTGTCCGA Num.seqs=9
Similarity=0.876068   31
GTTCGGACACCATTTGGTGC-TTATGCTAAAAT--CCTGTCCGAACTACCACCAA

******** * ****   ** * * *   *   ********* * * ** *
Consensus:

GTTCGGACaCgATTTcGCTtcTnCgacctTtttCTGTCCGAAcTacCaCCaA

>Bacillus_Fam_160_52_4 Nr. of seq. 4 Alignment length(with gaps) = 55
Alignment score = 0.711616
GCF_000708755.2_ASM70875v2_genomic.fna_2:551545-552118 Satlength=574 Nr of Repeats=11 RepeatLength=52 seed=GATAAGTGCC Num.seqs=10 Similarity=0.772080 0
GATAAGTGCCACCAAGAACAGAGATTNCAGTT-TTGATGGTTCCTTTC-TG-CTG
GCF_000708755.2_ASM70875v2_genomic.fna_2:805597-805857 Satlength=261 Nr of Repeats=5 RepeatLength=52 seed=AGAGATTCCT Num.seqs=5 Similarity=0.874359
18 -ATAAGTGCCATCAACAACAGAGATTCCTGGT-TTGGTGGTTCCTTTC-TGCCTG
GCF_000709935.2_ASM70993v2_genomic.fna_4:61155-61415 Satlength=261 Nr of Repeats=5 RepeatLength=52 seed=AGAGATTCCT Num.seqs=5 Similarity=0.874359
18 -ATAAGTGCCATCAACAACAGAGATTCCTGGT-TTGGTGGTTCCTTTC-TGCCTG
GCF_000708755.2_ASM70875v2_genomic.fna_6:56917-57385 Satlength=469 Nr of Repeats=9 RepeatLength=52 seed=TGGTTCTTAT Num.seqs=9 Similarity=0.820130
35 -TTAAGTGCCATCAAGCAC-NAGATTTCTNCTGTTGGTGGTTCTTATCACG-CTG

********* *** ** ***** *   * *** ****** * ** * ***
Consensus:

aTAAGTGCCAtCAAcaACagAGATTcCtggTTTGgTGGTTCcTtTCtGcCTG

>Bacillus_Fam_161_52_4 Nr. of seq. 4 Alignment length(with gaps) = 55
Alignment score = 0.627778
GCF_000752395.1_Bacillus_andreraoultii_genomic.fna_17:112081-112497
Satlength=417 Nr of Repeats=8 RepeatLength=52 seed=CTCAAGTTTT Num.seqs=8
Similarity=0.883700 0 CTCAAG-TTTTCGCAAAATCNGGCTCATAGAACACTT-CTATGAACCCAAAATC-
GCF_000752395.1_Bacillus_andreraoultii_genomic.fna_24:70990-71406
Satlength=417 Nr of Repeats=8 RepeatLength=52 seed=GTTTACGCGA Num.seqs=8
Similarity=0.815934 5
CTGAAG-TTTACGCGAAAACGGGCGCATACAAC-CTTCCTATAAGCCCAAAATT-
GCF_000752395.1_Bacillus_andreraoultii_genomic.fna_24:160764-161336
Satlength=573 Nr of Repeats=10 RepeatLength=52 seed=TCTATGAACC
Num.seqs=9 Similarity=0.789832 35
CTNAGG-TTTACGCGAAAANGGGNGCATAGAAC-CTTTCTATGAACCTAAAATC-
Rev.of_GCF_000752395.1_Bacillus_andreraoultii_genomic.fna_45:75443-75911
Satlength=469 Nr of Repeats=9 RepeatLength=52 seed=TTTTATCGAA Num.seqs=9
Similarity=0.833333 17 --CACGATTTTCGATAAAACGGGATTATAGAAC-CTTCCTATGAACCCATTATTC

* * *** ** ***   **   *** *** *** **** * ** * **
Consensus:

ctcAaGTTTaCGcgAAAacgGGcgcATAgAACCTTcCTATgAaCCcAaaATc

>Bacillus_Fam_162_53_4 Nr. of seq. 4 Alignment length(with gaps) = 55
Alignment score = 0.636364

GCF_001420715.1_ASM142071v1_genomic.fna_22:1500071-1500279 Satlength=209 Nr of Repeats=4 RepeatLength=52 seed=GGAGAGAATC Num.seqs=4 Similarity=0.880342 11
GGTAACAATAAGGAGAGAATCGTGCCCG-AAATCAAAG-AAAGGC-AAGGAAAAC
Rev.of_GCF_001420715.1_ASM142071v1_genomic.fna_24:534006-534318 Satlength=313 Nr of Repeats=6 RepeatLength=52 seed=CTATTGTTAC Num.seqs=6 Similarity=0.822222 11
GGTAACAATAGAGAGAGAATCGTGCCCG-AAATCGTGG-TNAGGCGAAGG-AAAC
Rev.of_GCF_001420715.1_ASM142071v1_genomic.fna_24:618440-618855 Satlength=416 Nr of Repeats=8 RepeatLength=52 seed=GTATTGTTAC Num.seqs=7 Similarity=0.823300 11
GGTAACAATACGAAGAGAATCGTTCCCGCAAAT--AAG-GCAGGAGAAGAAAAAC
GCF_001420715.1_ASM142071v1_genomic.fna_23:646118-646586 Satlength=469 Nr of Repeats=9 RepeatLength=52 seed=GTTCCCGAAA Num.seqs=9 Similarity=0.800570 21
GGGAACAATNGTGATAGTATTGTTCCCG-AAAT-TAAGAGAAGGC-AAGGAAAAC

```
* ** ** ** **** ****    *   ***  ***  ****** ******
```

Consensus:

ggAgAGaATcGTgCCCGAAATcnaaGgaAGGcgAAGgaAAACGGtAACAATag

>Bacillus_Fam_163_52_4 Nr. of seq. 4 Alignment length(with gaps) = 55 Alignment score = 0.742929
GCF_001636315.1_ASM163631v1_genomic.fna_1:2290722-2291190 Satlength=469 Nr of Repeats=9 RepeatLength=52 seed=CAAAATGGTC Num.seqs=9 Similarity=0.900285 0
CAAAATGGTCTTCATCACCCTTATGAAGACC--ATTTCTCTTC-GGCTTTTCATC
GCF_001636315.1_ASM163631v1_genomic.fna_1:2363645-2364165 Satlength=521 Nr of Repeats=10 RepeatLength=52 seed=AAAATGGTCT Num.seqs=10 Similarity=0.880912 1
CAAAATGGTCTTCATCANCCTTATGAAGGAC--ATTTCTCTTC-GGCTTTTCATC
Rev.of_GCF_001636315.1_ASM163631v1_genomic.fna_1:5707911-5708379 Satlength=469 Nr of Repeats=9 RepeatLength=52 seed=TGTCCTTCAT Num.seqs=9 Similarity=0.877009 32
CAAAATGGTCTTCATCACCNTTATGAAGGAC--ATTTCTCTTN-GGCTTTTCATC
GCF_001636315.1_ASM163631v1_genomic.fna_1:2583930-2584242 Satlength=313 Nr of Repeats=6 RepeatLength=52 seed=TTATGAAGAC Num.seqs=6 Similarity=0.882051 19
-AAAATGGTCTTCATCCTTCTTATGAAGACCAAAATTCTCTTCTGACTCCTC--C

```
***************    ********  *  * *******  * **  **  *
```

Consensus:

cAAAATGGTCTTCATCacccTTATGAAGaaCAtTTCTCTTcGgCTttTCatC

>Bacillus_Fam_164_52_4 Nr. of seq. 4 Alignment length(with gaps) = 55 Alignment score = 0.659596
GCF_001648575.1_ASM164857v1_genomic.fna_5:174970-175340 Satlength=371 Nr of Repeats=7 RepeatLength=52 seed=GCGTTCCATT Num.seqs=6 Similarity=0.873504
0 GCGTTCCATTCCACCT-CTATGACACACGTTTCCCCCTCCCGCTC--CCATTTTC
GCF_001648575.1_ASM164857v1_genomic.fna_62:10871-11688 Satlength=818 Nr of Repeats=16 RepeatLength=52 seed=TCCATACACC Num.seqs=12

Similarity=0.842100 4
GCGTTCCATA-CACCTTCTATGACACGCGAACTC--CTCTCGCTCTCCTCTTTTC
GCF_001648575.1_ASM164857v1_genomic.fna_62:58957-59580 Satlength=624 Nr of Repeats=12 RepeatLength=52 seed=TCTATGACAC Num.seqs=9
Similarity=0.816737
15   GCGTTCCATT-CACCNTCTATGACACGCGTTTTCA-CTCCCGCTCT-CTACTTTC
GCF_001648575.1_ASM164857v1_genomic.fna_67:75226-75644 Satlength=419 Nr of Repeats=8 RepeatLength=52 seed=TGACACGCGT Num.seqs=6
Similarity=0.782922
19   GCGTTTCATT-CACCTCCTATGACACGCGTTTTCA-CTNCCGCTCT-CGAAGTTT

***** *** **** ********* **   * ** ***** *   **
Consensus:

GCGTTcCATtCACCttCTATGACACgCGttttCaCTccCGCTCtCtattTTc

>Bacillus_Fam_165_52_4 Nr. of seq. 4 Alignment length(with gaps) = 55
Alignment score = 0.602525
GCF_002019645.1_ASM201964v1_genomic.fna_1:2030290-2031330 Satlength=1041 Nr of Repeats=20 RepeatLength=52 seed=GTTCAGACAG Num.seqs=20
Similarity=0.811784 3
AAGGTTCAGACAGCTTTTAGCGTCTGG-CTCGANAAGCTGTCCGAAGTNGAGC--
Rev.of_GCF_002019645.1_ASM201964v1_genomic.fna_1:5152559-5153287 Satlength=729 Nr of Repeats=14 RepeatLength=52 seed=AAAAGCTGTC
Num.seqs=14 Similarity=0.904480
17   AAG-TTCAGACAGCTTTTGCCGCATGG-CTCCTAAAGCTGTCCGAAGTAG-GCTC
GCF_002019645.1_ASM201964v1_genomic.fna_1:2116675-2117143 Satlength=469 Nr of Repeats=9 RepeatLength=52 seed=GAAAGCTGTC Num.seqs=9
Similarity=0.798043 31
CGGGTTCAGACAGCTTTTCCGTCCGAG-CACGGAAAGCTGTCCGAAGTTG-GN-C
GCF_002019645.1_ASM201964v1_genomic.fna_1:4452843-4453467 Satlength=625 Nr of Repeats=12 RepeatLength=52 seed=GTTCAGACAG Num.seqs=12
Similarity=0.821290 54
CGGGTTCAGACAGCTTTTGCCTCCTAGCCACCG-AACCTGTCCGAACTTG-AC-T

**************   * * *   ** ********* * *   *
Consensus:

gTTCAGACAGCTTTTgccgcctaGCaCcgaAAgCTGTCCGAAgTtGgccaaG

>Bacillus_Fam_166_52_4 Nr. of seq. 4 Alignment length(with gaps) = 54
Alignment score = 0.626543
GCF_000292245.2_ASM29224v2_genomic.fna_5:78606-78963 Satlength=358 Nr of Repeats=7 RepeatLength=51 seed=TTTCATTACC Num.seqs=7 Similarity=0.737528
0   -TTTCATTACCTATCCCA-GCTTTTTCGCACTCTAATC-CCA-CAACCCACAAG
GCF_000292245.2_ASM29224v2_genomic.fna_2:36979-37183 Satlength=205 Nr of Repeats=4 RepeatLength=51 seed=TTTCATTACC Num.seqs=4 Similarity=0.860566
0   -TTTCATTACCTATCACA-GCTTTTTCGCACTCTAATCTCGA-CATCCCCCAAC
GCF_000292245.2_ASM29224v2_genomic.fna_17:33217-33472 Satlength=256 Nr of Repeats=5 RepeatLength=51 seed=TTTTCATTAC Num.seqs=5 Similarity=0.783007
0   TTTTCATTACTTATCACC-ACTTTTTCGCACCGTAATC-CCAGCACCACGCGG-
Rev.of_GCF_000292245.2_ASM29224v2_genomic.fna_16:189-393 Satlength=205 Nr of Repeats=4 RepeatLength=51 seed=GATAAGTAAT Num.seqs=4

Similarity=0.769231 15
TTTTCATTACTTATCACCTACTTATTCGTACCCTAATT-CCGGCATCTCAAAA-

********* **** * *** **** ** **** * ** * *
Consensus:

tTTTCATTACcTATCaCaaCTTtTTCGcACccTAATcCcagCAtCcCacaan

>Bacillus_Fam_167_52_4 Nr. of seq. 4 Alignment length(with gaps) = 54
Alignment score = 0.618827
GCF_001636325.1_ASM163632v1_genomic.fna_2:1366189-1366546 Satlength=358
Nr of Repeats=7 RepeatLength=51 seed=AAAAATGGTC Num.seqs=7
Similarity=0.891636 0
AAAAATGGTCATAGAGAGCGAAACTGGAACCAAAAATCCACG-AG--AAAACGA
GCF_001636325.1_ASM163632v1_genomic.fna_2:868067-868429 Satlength=363 Nr
of Repeats=7 RepeatLength=52 seed=GAACTGGAAC Num.seqs=5
Similarity=0.871795
20 AAAAATGGTCATAGAGTGAGGAACTGGAACCAAAAAGGCTCG-AGC-AAAGTGC
Rev.of_GCF_001636325.1_ASM163632v1_genomic.fna_1:1668035-1668348
Satlength=314 Nr of Repeats=5 RepeatLength=52 seed=CTTCCCTGCA Num.seqs=3
Similarity=0.897436 50
CAAAATGGTCATAGATTGAGAAACTGGAACCAAAAATGGTTGCAGG-GAAG-GT
GCF_001636325.1_ASM163632v1_genomic.fna_1:898598-898859 Satlength=262 Nr
of Repeats=5 RepeatLength=52 seed=AACTGGAACC Num.seqs=4
Similarity=0.846154
20 -AAAATGGTCATAGATATACAAACTGGAACCAAAATCGAGCG-GGCTGAAGCCA

************** ************** * * **
Consensus:

aAAAATGGTCATAGAgagagaAACTGGAACCAAAAatgctcGaGcaAAgcga

>Bacillus_Fam_168_50_4 Nr. of seq. 4 Alignment length(with gaps) = 54
Alignment score = 0.652778
GCF_900104555.1_IMG-
taxon_2651870172_annotated_assembly_genomic.fna_16:149617-150076
Satlength=460 Nr of Repeats=9 RepeatLength=51 seed=TTTTGTCTTT Num.seqs=9
Similarity=0.819801 24 ACTTTTTCTTGAGTCT-CCAC-C-
GATTTTTGTCTTTCATAGNCTTGATGAAAG
Rev.of_GCF_900104555.1_IMG-
taxon_2651870172_annotated_assembly_genomic.fna_29:94987-95243
Satlength=257 Nr of Repeats=5 RepeatLength=51 seed=ATGAAAGACA Num.seqs=4
Similarity=0.745807 36 ACTTTTTCCAAAAGAT-CCAC-CGGAT-
TTTGTCTTTCATAGCCTCGATGAAAG
GCF_900104555.1_IMG-
taxon_2651870172_annotated_assembly_genomic.fna_19:193092-193875
Satlength=784 Nr of Repeats=7 RepeatLength=51 seed=ACTTTTTCTT Num.seqs=5
Similarity=0.963399 50 ACTTTTTCTTCTAGCT-CAACTC-
AAT-TTTGTCTTTCATAAGCTCGATGAAAG
Rev.of_GCF_900104555.1_IMG-
taxon_2651870172_annotated_assembly_genomic.fna_25:18116-18765
Satlength=650 Nr of Repeats=6 RepeatLength=51 seed=AAAATAGCTG Num.seqs=4
Similarity=0.891068 27 ACTTTTTCACGTGCTTACCAG-C-TAT-
TTTGTCTTTCATAAGCTTGATGAAGG

************* ** ****** *********   * * * * **
Consensus:

TTTGTCTTTCATAagCTcGATGAAaGACTTTTTCttgaagcTCcAcCgAT

>Bacillus_Fam_169_54_4 Nr. of seq. 4 Alignment length(with gaps) = 54
Alignment score = 0.745885
GCF_000186145.1_Bacillus_sp_2_A_57_CT2_V1_genomic.fna_4:107252-107511
Satlength=260 Nr of Repeats=5 RepeatLength=52 seed=GCCGAAAATG Num.seqs=4
Similarity=0.709644   0
GCCGAAAATGTGATAGGGAAG-AG-GAAAAGGGTAGTTAATCAACCGGATTAAC
Rev.of_GCF_000294775.2_ASM29477v2_genomic.fna_1:3523470-3523938
Satlength=469 Nr of Repeats=9 RepeatLength=52 seed=ACATTTTCGG Num.seqs=9
Similarity=0.804682   11
GCCGAAAATGTGGAGGGGAGG-AG-GAAACTGGTAGTTAATCGACTGGATTAAC
GCF_000294775.2_ASM29477v2_genomic.fna_1:910807-911429 Satlength=623 Nr
of Repeats=12 RepeatLength=52 seed=AACGACGAAA Num.seqs=10
Similarity=0.832054   49
GACGAAAACGTGG-AGAGA-GTAGAGAAAATGGTAGCTAATCAACCGGATTAAC
Rev.of_GCF_000186145.1_Bacillus_sp_2_A_57_CT2_V1_genomic.fna_6:186939-
187251 Satlength=313 Nr of Repeats=5 RepeatLength=52 seed=TTTTCGTCGT
Num.seqs=4 Similarity=0.930818   60
GACGAAAACGTGG-AGAGAGGTAGAGAAAATGGTAGCTAATCAACCGGATTAAC

* ****** ***   * ** * ** **** ***** ***** ** ********
Consensus:

GaCGAAAAcGTGgnaGaGAgGtAGaGAAAatGGTAGcTAATCaACcGGATTAAC

>Bacillus_Fam_170_52_4 Nr. of seq. 4 Alignment length(with gaps) = 54
Alignment score = 0.600823
GCF_000430765.1_ASM43076v1_genomic.fna_6:155335-155595 Satlength=261 Nr
of Repeats=5 RepeatLength=52 seed=GGACTACTTT Num.seqs=5
Similarity=0.862893
0   GGACTACTTTCTCCTC--CTGCCGCCTCAACCTGTCCGAACAGAGACCGCCTTC
GCF_000430765.1_ASM43076v1_genomic.fna_14:144417-144731 Satlength=315 Nr
of Repeats=6 RepeatLength=52 seed=TTCGGACTAC Num.seqs=5
Similarity=0.830769
49   GGACTACTTTCTCCGC--CNCCCGCTTCAACCTGTCCGAACCTGGACCGACTTC
GCF_002019645.1_ASM201964v1_genomic.fna_1:287166-287842 Satlength=677 Nr
of Repeats=13 RepeatLength=52 seed=CTTCGGACAG Num.seqs=13
Similarity=0.833333   48
GGACAGCTTTAACCGC--NTGGCTCCTCAACCTGTCTGAACTTACTCTGACTTC
GCF_002019645.1_ASM201964v1_genomic.fna_1:4704557-4705493 Satlength=937
Nr of Repeats=18 RepeatLength=52 seed=TGAACCTGTC Num.seqs=18
Similarity=0.826714   76
GGACAGCTTT--CGGCTTCTGGCGCATGAACCTGTCCGAACTTGCCTTCACTTC

**** **** * *   * * * ******** ****   ****
Consensus:

GGACaaCTTTctCcgCctgcCgCcTcAACCTGTCcGAACttacaccgaCTTC

>Bacillus_Fam_171_53_4 Nr. of seq. 4 Alignment length(with gaps) = 54
Alignment score = 0.641975
GCF_001636315.1_ASM163631v1_genomic.fna_1:3109160-3109628 Satlength=469
Nr of Repeats=9 RepeatLength=52 seed=GTCGCCTATA Num.seqs=9
Similarity=0.729167 0
GTCGCCTATAAGGCGTATACGCGACCG-AACGGNAGCCAAAA-AGAGTCCCTCG
GCF_001636315.1_ASM163631v1_genomic.fna_1:3775783-3776095 Satlength=313
Nr of Repeats=6 RepeatLength=52 seed=GTCGCATAGA Num.seqs=6
Similarity=0.782906 0
GTCGCATAGAAGGGGTATACGCGCCCG-AGCGGCGGAAAAAATGGAGGTGC-CG
GCF_001636315.1_ASM163631v1_genomic.fna_1:5059185-5059445 Satlength=261
Nr of Repeats=5 RepeatLength=52 seed=AGAAAAAGAA Num.seqs=5
Similarity=0.792308 33
GTCGCATAGAAAGAGTATATGCGACTG-AGCGGCAGAAAAAGAAGAGNNGC-CG
GCF_001636315.1_ASM163631v1_genomic.fna_1:4975737-4976309 Satlength=573
Nr of Repeats=11 RepeatLength=52 seed=GTCGCATAGA Num.seqs=9
Similarity=0.720798 0
GTCGCATAGAAGGCGTATACGCGACCGAAGCGGC-GAAAAAATCGTG-AGCTCG

***** ** ** * ***** *** * * * *** * ***   * *   * **
Consensus:

GTCGCaTAgAAgGcGTATAcGCGaCcGAgCGGcaGaaAAAataGaGnngCtCG

>Bacillus_Fam_172_53_4 Nr. of seq. 4 Alignment length(with gaps) = 54
Alignment score = 0.720165
GCF_001636425.1_ASM163642v1_genomic.fna_1:2633742-2634106 Satlength=365
Nr of Repeats=6 RepeatLength=52 seed=ATGAAGGACT Num.seqs=5
Similarity=0.677576 0
ATGAAGGACTTTTCGCGCTCTTCTTGA-CCTGGTTTTGGCCTTCATAAGCCTN-
Rev.of_GCF_001636425.1_ASM163642v1_genomic.fna_1:4369053-4369313
Satlength=261 Nr of Repeats=5 RepeatLength=52 seed=AAAGTCCTTC Num.seqs=5
Similarity=0.776543 11 -
TGAAGGACTTTGCTCGTTCTTCTTGA-CCTGGTTTTGGTCTTCATTAGCTCGA
Rev.of_GCF_001636425.1_ASM163642v1_genomic.fna_1:4370800-4371268
Satlength=469 Nr of Repeats=9 RepeatLength=52 seed=AAAGTCCTTC Num.seqs=9
Similarity=0.820513 11 -
TGAAGGACTTTGCTCGCTCTTCTTGA-CCGGGTTTTGGCCTTCATTAGCTTGA
GCF_001636425.1_ASM163642v1_genomic.fna_1:3011924-3012184 Satlength=261
Nr of Repeats=5 RepeatLength=52 seed=TTTTGTCCTT Num.seqs=5
Similarity=0.730818 32
ATGAAGGACTTTTCTCGTTATTCTCCAGCCT-GTTTTGTCCTTCATTCACTTT-

*********** * ** * **** * ** ****** ******   *
Consensus:

aTGAAGGACTTTgCtCGcTcTTCTtgACCtgGTTTTGgcCTTCATtagCttga

>Bacillus_Fam_173_52_4 Nr. of seq. 4 Alignment length(with gaps) = 54
Alignment score = 0.661523
GCF_002019665.1_ASM201966v1_genomic.fna_1:300861-301737 Satlength=877 Nr
of Repeats=11 RepeatLength=52 seed=ATCGTGCCCT Num.seqs=9
Similarity=0.871795 12
GATCAAGCCGC-GATCGTGCCCTCTAAACGCCGAAAACG-AGAAAACAGGTAAC

GCF_002019665.1_ASM201966v1_genomic.fna_1:548830-549350 Satlength=521 Nr of Repeats=10 RepeatLength=52 seed=TCAAAACAAG Num.seqs=10 Similarity=0.955556 21
GATGAAG-CGTTGATCGTGCCCTCAAAACAAGAAAAACGAAAAATCG-GGTAAC
GCF_002019665.1_ASM201966v1_genomic.fna_1:2070270-2070894 Satlength=625 Nr of Repeats=12 RepeatLength=52 seed=AAAATAGAGG Num.seqs=12 Similarity=0.897436 38
GATGAAG-AGCGGATCGTGCCCTCTAAACGAGAAAAATG-AAAATAGAGGGAAC
Rev.of_GCF_002019665.1_ASM201966v1_genomic.fna_1:4148016-4148588 Satlength=573 Nr of Repeats=11 RepeatLength=52 seed=TTTTCAGGGC Num.seqs=11 Similarity=0.835897
79 GATGGAG-NGCTGATCGTGCCCTGAAAANNTGAAAAACG-AAAATAGAGGAAAC

********** *** **** * * ** ** ****** ** * *
Consensus:

ATCGTGCCCTcaAAAcgagaAAAAcGAaAAtagaGGtAACGATgaAGcGctG

>Bacillus_Fam_174_54_4 Nr. of seq. 4 Alignment length(with gaps) = 54 Alignment score = 0.763375
GCF_000153365.1_ASM15336v1_genomic.fna_16:423251-424048 Satlength=798 Nr of Repeats=15 RepeatLength=53 seed=GTCATGTATC Num.seqs=13 Similarity=0.864861 0
GTCATGTATCCCATCAGGTTCCCGGAATACATGAGTA-GGGAGAAAAAAGGATG
Rev.of_GCF_000473245.1_ASM47324v1_genomic.fna_1:2388131-2388928 Satlength=798 Nr of Repeats=15 RepeatLength=53 seed=GATACATGAC Num.seqs=13 Similarity=0.864861
10 GTCATGTATCCCATCAGGTTCCCGGAATACATGAGTA-GGGAGAAAAAAGGATG
GCF_000153365.1_ASM15336v1_genomic.fna_74:127356-128149 Satlength=794 Nr of Repeats=13 RepeatLength=53 seed=CGGGATACAT Num.seqs=10 Similarity=0.875891 22
GTCATGTATGCCAACAGGTTTCCGGGATACATG-GTATGACTGCAAGAAGGCTA
GCF_000473245.1_ASM47324v1_genomic.fna_1:2184010-2184804 Satlength=795 Nr of Repeats=13 RepeatLength=53 seed=CGGGATACAT Num.seqs=11 Similarity=0.875586 22
GTCATGTATGCCAACAGGTTTCCGGGATACATG-GTATGACTGCAAGAAGGCTA

********* *** ****** **** ******* *** * * ** **** *
Consensus:

GTCATGTATcCCAaCAGGTTcCCGGaATACATGaGTAtGacaGaAAaAAGGaTa

>Bacillus_Fam_175_54_4 Nr. of seq. 4 Alignment length(with gaps) = 54 Alignment score = 0.627572
GCF_000311725.1_ASM31172v1_genomic.fna_1:927879-928355 Satlength=477 Nr of Repeats=9 RepeatLength=53 seed=CATGAAACAT Num.seqs=8 Similarity=0.891285
0 CATGAAACATACTTT-CCCTACAAATCCGCTCTTCTCTTGTCTCATGAACCCCT
Rev.of_GCF_000311725.1_ASM31172v1_genomic.fna_2:132590-132855 Satlength=266 Nr of Repeats=5 RepeatLength=53 seed=ACATAAGTTG Num.seqs=5 Similarity=0.886420 40
CATGAGACATACATT-CTCTAAGAATGTGCTCAACTTATGTTCCATGAGCCCTT
GCF_000311725.1_ASM31172v1_genomic.fna_3:662414-662785 Satlength=372 Nr of Repeats=7 RepeatLength=53 seed=TGTATCATGA Num.seqs=7

Similarity=0.855046
37   CATGTGACATACTTTCCTCTTCCAACCACCT-AACTTATGTATCATGAACCCTT
GCF_000311725.1_ASM31172v1_genomic.fna_4:562666-563196 Satlength=531 Nr of Repeats=10 RepeatLength=53 seed=AGTTTTGTTT Num.seqs=10
Similarity=0.865269   85
CATGTGACATACTTTCCTCTGAAAACACCCT-GAGTTTTGTTTCATGAACCCTT

**** ****** ** * **   **   **   * *** ***** *** *
Consensus:

CATGagACATACtTTcCtCTaaaAAccccCTcaacTtaTGTttCATGAaCCCtT

>Bacillus_Fam_176_53_4 Nr. of seq. 4 Alignment length(with gaps) = 54
Alignment score = 0.659465
GCF_900156865.1_PRJEB18960_genomic.fna_3:147620-148044 Satlength=425 Nr of Repeats=8 RepeatLength=53 seed=GATAAAATTA Num.seqs=8
Similarity=0.733099
0   GATAAAATTAGGAAAAAGCGAACCAGCAAACCCG-AAATGCAGGTACGCATTTG
GCF_900156865.1_PRJEB18960_genomic.fna_3:564737-565055 Satlength=319 Nr of Repeats=6 RepeatLength=53 seed=GGATAAAATT Num.seqs=6
Similarity=0.814815
52   GATAAAATTAGGAAACGGCAAACCCGCAAACGAG-AGATACAGGTTCGCATTTG
GCF_900156865.1_PRJEB18960_genomic.fna_3:163814-164132 Satlength=319 Nr of Repeats=6 RepeatLength=53 seed=CTTAACAGGT Num.seqs=6
Similarity=0.833128
34   GAAGAAATTAGGGAACACCGAACCCGCAAAGGCTCTTA-ACAGGTTCGCATATG
GCF_900156865.1_PRJEB18960_genomic.fna_3:535365-535736 Satlength=372 Nr of Repeats=7 RepeatLength=53 seed=ATACAGGTTC Num.seqs=7
Similarity=0.746914
36   TAAAAAATTAGGGAAANNCGAACCAGTAAAAGCG-ATATACAGGTTCGCAAATG

* ******** **   * **** * ***   * ***** **** **
Consensus:

gAaaAAATTAGGaAAaagCgAACCaGcAAAcgcgatAtaCAGGTtCGCAtaTG

>Bacillus_Fam_177_51_4 Nr. of seq. 4 Alignment length(with gaps) = 53
Alignment score = 0.768344
GCF_000242895.2_ASM24289v3_genomic.fna_1:507909-508266 Satlength=358 Nr of Repeats=7 RepeatLength=51 seed=AAAAATGGGA Num.seqs=7
Similarity=0.820728
0   AAAAATGGGAACGAAAGAGCCGCAATAATGCCCAAAATCCAAGAGTCG-C-AG
Rev.of_GCF_000242895.2_ASM24289v3_genomic.fna_1:4381175-4381532
Satlength=358 Nr of Repeats=7 RepeatLength=51 seed=ATTTTGGGCA Num.seqs=7
Similarity=0.828991   38
AAAAATGGGAACCAAAGAGCCGCAATGATGCCCAAAATCCAAGAGTCG-C-GG
Rev.of_GCF_000242895.2_ASM24289v3_genomic.fna_1:1303702-1303957
Satlength=256 Nr of Repeats=5 RepeatLength=51 seed=GATTTTGGGC Num.seqs=5
Similarity=0.750000   39
AAAAATGGGAATCAAAGAGCCTCANTGATGCCCAAAATCCGAGAGTCG-C-GG
Rev.of_GCF_000242895.2_ASM24289v3_genomic.fna_1:2430720-2430925
Satlength=206 Nr of Repeats=4 RepeatLength=51 seed=ATTTTGGGCA Num.seqs=3
Similarity=0.912854   38
AAAAACGGGAACGAAAGAGCCGTATTGATGCCCAAAATTC--GAGGCGACTGG

***** ***** ******** * * *********** * *** ** * *
Consensus:

AAAAAtGGGAAccAAAGAGCCgcAaTgATGCCCAAAATcCaaGAGtCGCgG

>Bacillus_Fam_178_52_4 Nr. of seq. 4 Alignment length(with gaps) = 53
Alignment score = 0.633124
GCF_001636325.1_ASM163632v1_genomic.fna_1:40630-41088 Satlength=459 Nr of Repeats=9 RepeatLength=51 seed=ATAAGAAAGC Num.seqs=8 Similarity=0.916900
0   ATAAGAAAGCGCCGGATTCGCCTCAAGAAACCCG-CCA-CTTGAGTCAAGTTG
GCF_001636325.1_ASM163632v1_genomic.fna_1:516349-516757 Satlength=409 Nr of Repeats=8 RepeatLength=51 seed=AGTTGATAAG Num.seqs=8 Similarity=0.798319
46   ATAAGAAAGTGCCGGATTCCCATCAAGAAAACGGCCCA-C-CGAGTAAAGTTG
GCF_001636325.1_ASM163632v1_genomic.fna_3:614885-615192 Satlength=308 Nr of Repeats=6 RepeatLength=51 seed=GATAAGAAAG Num.seqs=5 Similarity=0.921569
50   ATAAGAAAGAGCCGTATTTCCATCAAGAACCCCG--AAGCCCGAATAAAGATG
Rev.of_GCF_001636325.1_ASM163632v1_genomic.fna_1:2096119-2096476 Satlength=358 Nr of Repeats=7 RepeatLength=51 seed=TACAGATTTC Num.seqs=7 Similarity=0.927793   43
ATAAGAAAGCACNGGATTCTCATCAAGAAAATCG-AAATC-TGTATAAAGCTG

********* * * *** * *******   *   * * * * *** **
Consensus:

ATAAGAAAGcgCcGgATTccCaTCAAGAAaaccGcaAnCncGaaTaAAGtTG

>Bacillus_Fam_179_53_4 Nr. of seq. 4 Alignment length(with gaps) = 53
Alignment score = 0.742138
GCF_000153365.1_ASM15336v1_genomic.fna_12:126684-126892 Satlength=209 Nr of Repeats=4 RepeatLength=52 seed=CTTAGTCAGC Num.seqs=4 Similarity=0.876068
0   CTTAGTCAGCTGGACTAGAGCCCTCTG-TAAGGCAATTAAGAGTCCAGAGGGC
GCF_000473245.1_ASM47324v1_genomic.fna_1:1107975-1108183 Satlength=209 Nr of Repeats=4 RepeatLength=52 seed=CTTAGTCAGC Num.seqs=4 Similarity=0.876068   0
CTTAGTCAGCTGGACTAGAGCCCTCTG-TAAGGCAATTAAGAGTCCAGAGGGC
GCF_000153365.1_ASM15336v1_genomic.fna_16:507322-507582 Satlength=261 Nr of Repeats=5 RepeatLength=52 seed=GGGTCTTAGT Num.seqs=5 Similarity=0.876923
48   CTTAGTCGGCTGAACTAGTACCCTCTGATTTGAGAATTGCTAG-CCAGAGGGT
Rev.of_GCF_000473245.1_ASM47324v1_genomic.fna_1:2304596-2304856 Satlength=261 Nr of Repeats=5 RepeatLength=52 seed=GACTAAGACC Num.seqs=5 Similarity=0.876923   59
CTTAGTCGGCTGAACTAGTACCCTCTGATTTGAGAATTGCTAG-CCAGAGGGT

******* **** ***** ******* * * ****   ** ********
Consensus:

CTTAGTCaGCTGaACTAGaaCCCTCTGaTaaGacAATTaagAGtCCAGAGGGc

>Bacillus_Fam_180_53_4 Nr. of seq. 4 Alignment length(with gaps) = 53
Alignment score = 0.778826
GCF_000153365.1_ASM15336v1_genomic.fna_16:220396-220760 Satlength=365 Nr of Repeats=7 RepeatLength=52 seed=ACCGCTATTT Num.seqs=7 Similarity=0.796093
0 ACCGCTATTTCNCTGAAAAACTAAAATAATGGTTNCAGC-GNAGAGAGCTAGG
Rev.of_GCF_000473245.1_ASM47324v1_genomic.fna_1:2589359-2589723 Satlength=365 Nr of Repeats=7 RepeatLength=52 seed=GAAATAGCGG Num.seqs=7 Similarity=0.796093 11
ACCGCTATTTCNCTGAAAAACTAAAATAATGGTTNCAGC-GNAGAGAGCTAGG
Rev.of_GCF_000153365.1_ASM15336v1_genomic.fna_63:5871-6183 Satlength=313 Nr of Repeats=6 RepeatLength=52 seed=GGTTCTACCT Num.seqs=6 Similarity=0.789518 3
ACCGCTATTTCTATGAAAATCGAAAATAGTGGTTCCAGCATGAG-GAGGTAGA
GCF_000473245.1_ASM47324v1_genomic.fna_1:974011-974323 Satlength=313 Nr of Repeats=6 RepeatLength=52 seed=AGGTAGAACC Num.seqs=6 Similarity=0.794759
45 ACCGCTATTTCTATGAAAATCGAAAATAGTGGTTCCAGCATGAG-GAGGTAGA

*********** ****** * ****** ***** **** ** *** ***
Consensus:

ACCGCTATTTCtaTGAAAAaCgAAAATAaTGGTTcCAGCaggAGaGAGcTAGa

>Bacillus_Fam_181_53_4 Nr. of seq. 4 Alignment length(with gaps) = 53
Alignment score = 0.796646
GCF_000186145.1_Bacillus_sp_2_A_57_CT2_V1_genomic.fna_1:47246-47506 Satlength=261 Nr of Repeats=5 RepeatLength=52 seed=TTTCTTTTCC Num.seqs=5 Similarity=0.776923 0
TTTCTTTTCCTCTCTTNCATTTTCGTCGTCATGAACCCTCCATGACGACCAT-
Rev.of_GCF_000294775.2_ASM29477v2_genomic.fna_1:2223445-2223705 Satlength=261 Nr of Repeats=5 RepeatLength=52 seed=TCATGACGAC Num.seqs=5 Similarity=0.871795 34
TTTCTTTTCCTCTCTTGCATTTTCGTCGTCATGAACCCTCCATGACGACCAT-
Rev.of_GCF_000186145.1_Bacillus_sp_2_A_57_CT2_V1_genomic.fna_19:25944-26309 Satlength=366 Nr of Repeats=7 RepeatLength=52 seed=GACGAAAAAG Num.seqs=6 Similarity=0.813675 26
TTCCTCGTGC-CTCTTCCTTTTTCGTCTTCATGAACCCTCTATGGCGACCATT
Rev.of_GCF_000294775.2_ASM29477v2_genomic.fna_1:370090-370350 Satlength=261 Nr of Repeats=5 RepeatLength=52 seed=GACGAAAAAG Num.seqs=3 Similarity=0.820513 26
TTCCTCGTTC-CTCTTCCTTTTTCGTCGTCATGAANCCTCTATGACGACCATT

** ** * * ***** * ******** ******* **** *** *******
Consensus:

TTcCTcgTcCtCTCTTcCaTTTTCGTCgTCATGAAcCCTCcATGaCGACCATt

>Bacillus_Fam_182_53_4 Nr. of seq. 4 Alignment length(with gaps) = 53
Alignment score = 0.667715
GCF_000986785.1_ASM98678v1_genomic.fna_18:58013-58221 Satlength=209 Nr of Repeats=4 RepeatLength=52 seed=GTCAGAGCCC Num.seqs=4 Similarity=0.713836
0 GTCAGAGCCCGAAATTGGCGGAGGTC-GGGAAAAAAGGTAACTGAGAGGCGGT

Rev.of_GCF_000986785.1_ASM98678v1_genomic.fna_48:60540-60799 Satlength=260 Nr of Repeats=5 RepeatLength=52 seed=GGGCACTGAC Num.seqs=4 Similarity=0.884615 10 GTCAGTGCCCGAAATCGGCGGCAGCC-GTTCAAAAAGGTACCTGAGAGCCCGT
Rev.of_GCF_000986785.1_ASM98678v1_genomic.fna_26:10-270 Satlength=261 Nr of Repeats=5 RepeatLength=52 seed=TGTCAGTTAC Num.seqs=5 Similarity=0.701818
46 GTCAGTGCCCAAAATCCGC-GAGGCCNGCCGAAAAAGGTAACTGACAGGCGTC
GCF_000986785.1_ASM98678v1_genomic.fna_38:8-216 Satlength=209 Nr of Repeats=4 RepeatLength=52 seed=GTCAGTGCCC Num.seqs=4 Similarity=0.876068
52 GTCAGTGCCCAAAATCCGC-GAGGTCCGCCAAAAAAGGTAACTGACAAGCCCC

***** **** **** ** * * * * ********* **** * *
Consensus:

GTCAGtGCCCaAAATccGCgGagGcCnGccaAAAAAGGTAaCTGAcAggCcgc

>Bacillus_Fam_183_52_4 Nr. of seq. 4 Alignment length(with gaps) = 53 Alignment score = 0.642034
GCF_001315045.1_ASM131504v1_genomic.fna_75:1135-1342 Satlength=208 Nr of Repeats=4 RepeatLength=52 seed=GAGGTGATGA Num.seqs=3 Similarity=0.825996
0 GAGGTGATGAAAGCCGGAACGTGGAGAAAGCCGAGGAGAAAAGTCATTCATG-
Rev.of_GCF_001636425.1_ASM163642v1_genomic.fna_1:2741181-2741389 Satlength=209 Nr of Repeats=4 RepeatLength=52 seed=CGTTTTGTCC Num.seqs=4 Similarity=0.863248 20 G-GTTGATGAAGGACAAAACGTGAGAATGGAGGTTCAGAAAAGTCCTTCATGA
Rev.of_GCF_001636425.1_ASM163642v1_genomic.fna_1:2936242-2936501 Satlength=260 Nr of Repeats=4 RepeatLength=52 seed=GCCTTCATCA Num.seqs=3 Similarity=0.863248 65 G-GGTGATGAAGGCCAAAACTTGGAAAAAGAGAAAGGGAAAAGTCCTTCATAA
Rev.of_GCF_001636425.1_ASM163642v1_genomic.fna_1:3314811-3315123 Satlength=313 Nr of Repeats=6 RepeatLength=52 seed=TTATGAAGGA Num.seqs=6 Similarity=0.858120 104 G-GATGATGAAGGCCAAAACGAGGAAACAGGGAAGCTGAAAAGTCCTTCATAA

* * ******* * * *** * * * ******** *****
Consensus:

GGgTGATGAAgGcCaaAACgtGgaaAaaGagaagcaGAAAAGTCcTTCATaa

>Bacillus_Fam_184_52_4 Nr. of seq. 4 Alignment length(with gaps) = 53 Alignment score = 0.692872
GCF_001591665.1_ASM159166v1_genomic.fna_18:122146-122559 Satlength=414 Nr of Repeats=8 RepeatLength=52 seed=TCCAATAACC Num.seqs=6 Similarity=0.849573 0 TCCAATAACCCCTATTGGATAC-CTTTTCTCCCACTTTTGCCTCCGTACGGGT
Rev.of_GCF_001591665.1_ASM159166v1_genomic.fna_53:24940-25253 Satlength=314 Nr of Repeats=6 RepeatLength=52 seed=GGTATCCAAT Num.seqs=5 Similarity=0.712821 23 TCCAATATGCCCGATTGGATACCCTTTT-TCCNATNTTTCTANCGGAACGGGN
GCF_001591665.1_ASM159166v1_genomic.fna_76:10574-10937 Satlength=364 Nr of Repeats=7 RepeatLength=52 seed=ACCTTTTCTC Num.seqs=6 Similarity=0.870085
73 TCCAATAAGGCCGATTGGAAAC-CTTTTCTCCGATTTTTCCGGTGGAACGGGT

GCF_001591805.1_ASM159180v1_genomic.fna_26:68567-68984 Satlength=418 Nr of Repeats=8 RepeatLength=52 seed=GGCTCCAATC Num.seqs=7 Similarity=0.870574
102 TCCAATCAAGCCTATTGGATAC-CTTTTCTCCAATTTTTCCAGTGGAACGGGC

****** ** ****** ** ***** *** * *** * *****
Consensus:

TCCAATaagcCCgATTGGAtACCTTTTcTCCnAttTTTccagcgGaACGGGt

>Bacillus_Fam_185_52_4 Nr. of seq. 4 Alignment length(with gaps) = 53 Alignment score = 0.707547
GCF_001636315.1_ASM163631v1_genomic.fna_1:1920272-1920895 Satlength=624 Nr of Repeats=14 RepeatLength=52 seed=TTTTGTCCTT Num.seqs=9 Similarity=0.760684 0
TTTTGTCCTTCATCNGCCTTATGAAGACCATTTTGGACCT-GGCTCACGCCTG
Rev.of_GCF_001636315.1_ASM163631v1_genomic.fna_1:2936759-2937122 Satlength=364 Nr of Repeats=7 RepeatLength=52 seed=GATGAAGGAC Num.seqs=6 Similarity=0.921368 14
TTTTGTCCTTCATCAGCCTTATGAAGACCATTTTGAACCT-GGATCCCGCCTG
Rev.of_GCF_001636315.1_ASM163631v1_genomic.fna_1:2922406-2923185 Satlength=780 Nr of Repeats=15 RepeatLength=52 seed=ATGAAGACCA Num.seqs=14 Similarity=0.825021
13 TTTTGGTCTTCATCCNGCTTATGAAGACCATTTCGTTCCTCGGC-CACGGCTG
GCF_001636315.1_ASM163631v1_genomic.fna_1:1954829-1955089 Satlength=261 Nr of Repeats=5 RepeatLength=52 seed=TATGAAGGAC Num.seqs=5 Similarity=0.933333 19
TTTTGGTCTTCATCGGGCTTATGAAGGACATTTTGAACCC-GGGTCCCAGCTA

***** ******* ********* ***** * ** ** * * **
Consensus:

TTTTGgcCTTCATCngcCTTATGAAGacCATTTtGaaCCtGGctCaCgcCTg

>Bacillus_Fam_186_51_4 Nr. of seq. 4 Alignment length(with gaps) = 52 Alignment score = 0.607906
GCF_000292245.2_ASM29224v2_genomic.fna_4:29318-29828 Satlength=511 Nr of Repeats=10 RepeatLength=51 seed=GCACCGTAAT Num.seqs=10 Similarity=0.784242
0 GCACCGTAATTTCNGCTCCCCANCTTTTTCATTACCTATTCCT-TTTTATCC
GCF_000292245.2_ASM29224v2_genomic.fna_4:4185-4491 Satlength=307 Nr of Repeats=6 RepeatLength=51 seed=TTTTTCATTA Num.seqs=6 Similarity=0.780342
24 GCACCNTAATCACGGCAACTCAACTTTTTCATTACCTATTCCTACCCTATTC
GCF_000292245.2_ASM29224v2_genomic.fna_14:17182-17488 Satlength=307 Nr of Repeats=6 RepeatLength=51 seed=TCATTACCTA Num.seqs=6 Similarity=0.750763
28 GCTCCCTAATTTCTAAACCCCACTAGTTTCATTACCTATTCAC-CGTTATTC
Rev.of_GCF_000292245.2_ASM29224v2_genomic.fna_31:19400-19655 Satlength=256 Nr of Repeats=5 RepeatLength=51 seed=ATTGGTAATG Num.seqs=5 Similarity=0.803922 39
GCACCGTAATTTTGGGATTCCTCCTGTTTCATTACCAATTCAN-CGTTATTC

** ** **** * ********** **** *** *
Consensus:

GCaCCgTAATttcggcacccCacctgTTTCATTACCtATTCatcgtTATtC

>Bacillus_Fam_187_51_4 Nr. of seq. 4 Alignment length(with gaps) = 52
Alignment score = 0.695513
GCF_000292245.2_ASM29224v2_genomic.fna_7:14915-15272 Satlength=358 Nr of Repeats=7 RepeatLength=51 seed=TTTCATTACT Num.seqs=7 Similarity=0.601190
0 TTTCATTACTTATCNTCNGTTTTTCGCACCCTAATT-CTGCNAATCTCNAAN
Rev.of_GCF_000292245.2_ASM29224v2_genomic.fna_21:11408-11714 Satlength=307 Nr of Repeats=6 RepeatLength=51 seed=GATAAGTAAT Num.seqs=6 Similarity=0.803050 14
TTTCATTACTTATCCTAGGTTTTTCGCACCCTAATTCCGGCAAATCTC-AAG
Rev.of_GCF_000292245.2_ASM29224v2_genomic.fna_31:11-419 Satlength=409 Nr of Repeats=8 RepeatLength=51 seed=GTAATGAAAA Num.seqs=8 Similarity=0.774043
9 TTTCATTACCTATCCCGGGTTTTTCGCACCGTAATCATTGCTAAATTT-AAT
GCF_000292245.2_ASM29224v2_genomic.fna_46:27-231 Satlength=205 Nr of Repeats=4 RepeatLength=51 seed=ATTTTCATTA Num.seqs=4 Similarity=0.814637
49 TTTCATTACCTATCCCAAGTTTTTCGAACCCTAATCCCTGCAAAACTT-CAT

********* **** ******** *** **** ** ** * *
Consensus:

TTTCATTACcTATCccagGTTTTTCGcACCcTAATccctGCaAAacTcaAt

>Bacillus_Fam_188_52_4 Nr. of seq. 4 Alignment length(with gaps) = 52
Alignment score = 0.668803
GCF_000307875.1_BABA1.0_genomic.fna_182:7089-7293 Satlength=205 Nr of Repeats=4 RepeatLength=51 seed=TGAAGGACAA Num.seqs=4 Similarity=0.904139
0 TGAAGGACAAAACTCTGTAAAAAT-CGGAAGAAATGTCCTTCATGAAGCCAA
Rev.of_GCF_000759675.1_ASM75967v1_genomic.fna_73:164831-165091 Satlength=261 Nr of Repeats=5 RepeatLength=52 seed=AAGGACATTT Num.seqs=5 Similarity=0.876923 41
TGAAGGACAAAACCCCGTAGGAATACAGGAGAAATGTCCTTCATAAAGCCGA
GCF_000759675.1_ASM75967v1_genomic.fna_155:210755-211118 Satlength=364 Nr of Repeats=6 RepeatLength=52 seed=AAAGAAATGT Num.seqs=4 Similarity=0.854701 27
TGAAGGACAAAACGCTGAAGAAAACCAAAAGAAATGTCCTTCATAAGGTCGA
GCF_001420715.1_ASM142071v1_genomic.fna_22:535021-535281 Satlength=261 Nr of Repeats=5 RepeatLength=52 seed=AGGAAAAGTG Num.seqs=5 Similarity=0.979487 26
TGAAGGACAAAACCCGATAGTAAAATAGGAAAAGTGTCCTTCATCTTGTTAA

************* * * ** * ** ********** * *
Consensus:

TGAAGGACAAAACcCtgtAgaAAaacagaAgAAaTGTCCTTCATaaaGccaA

>Bacillus_Fam_189_52_4 Nr. of seq. 4 Alignment length(with gaps) = 52
Alignment score = 0.784188
GCF_000153365.1_ASM15336v1_genomic.fna_18:361319-361684 Satlength=366 Nr of Repeats=7 RepeatLength=52 seed=GGTTCTAGTC Num.seqs=6 Similarity=0.835897
0 GGTTCTAGTCGAGCGAACTAAGACCCTCTATCCTCCAAAATGTGATTCAGAG

Rev.of_GCF_000473245.1_ASM47324v1_genomic.fna_1:590850-591215 Satlength=366 Nr of Repeats=7 RepeatLength=52 seed=GACTAGAACC Num.seqs=6 Similarity=0.835897 10 GGTTCTAGTCGAGCGAACTAAGACCCTCTATTCTCCAAAATGTGATTCAGAG
GCF_000153365.1_ASM15336v1_genomic.fna_59:11476-11844 Satlength=369 Nr of Repeats=7 RepeatLength=52 seed=CACCCTCTAT Num.seqs=5 Similarity=0.687180 21 GGCCTTAGTCGGNCGGACTAACACCCTCTATTCNGCAAAATGACTTTCAGAG
Rev.of_GCF_000473245.1_ASM47324v1_genomic.fna_1:1841148-1841516 Satlength=369 Nr of Repeats=7 RepeatLength=52 seed=ATAGAGGGTG Num.seqs=5 Similarity=0.697436 31 GGCCTTAGTCGGNCGGACTAACACCCTCTATTCNGCAAAATGACTTTCAGAG

**   ****** ** ***** ********* * *******   *******
Consensus:

GGcccTAGTCGagCGaACTAAcACCCTCTATtCtcCAAAATGacaTTCAGAG

>Bacillus_Fam_190_52_4 Nr. of seq. 4 Alignment length(with gaps) = 52 Alignment score = 0.841880
GCF_001439965.1_ASM143996v1_genomic.fna_125:105844-106051 Satlength=208 Nr of Repeats=4 RepeatLength=52 seed=AAAATGTGTC Num.seqs=3 Similarity=0.863248 0 AAAATGTGTCTCACCCGGGTCAATGGAAACACATAAAAAGGGATTTTGCTTA
GCF_001887185.1_ASM188718v1_genomic.fna_106:116-584 Satlength=469 Nr of Repeats=9 RepeatLength=52 seed=CACATAACAA Num.seqs=9 Similarity=0.782051 29 AAAATGTGTCTCACCCCGGTCAATGGAAACACATAACAAGAGATATTGCTCA
Rev.of_GCF_001887185.1_ASM188718v1_genomic.fna_197:90-402 Satlength=313 Nr of Repeats=6 RepeatLength=52 seed=ATGTGTTTCC Num.seqs=6 Similarity=0.838365 34 AAAATGTGTCTCAATCCAGTCGATGGAAACACATAACAAGAGATTATGCTCA
GCF_002009555.1_ASM200955v1_genomic.fna_268:25-597 Satlength=573 Nr of Repeats=11 RepeatLength=52 seed=TCAAAAATGT Num.seqs=11 Similarity=0.854545 49 AAAATGTGTCTCAATCCGGTCGATGGAACCACATAACAAGAGATTATGCTCA

************* * *** ****** ******* *** *** **** *
Consensus:

AAAATGTGTCTCAacCcgGTCaATGGAAaCACATAAcAAGaGATtaTGCTcA

>Bacillus_Fam_191_52_4 Nr. of seq. 4 Alignment length(with gaps) = 52 Alignment score = 0.735043
GCF_001636325.1_ASM163632v1_genomic.fna_1:2372911-2373170 Satlength=260 Nr of Repeats=5 RepeatLength=52 seed=TTTTGGTTCC Num.seqs=4 Similarity=0.756410 0 TTTTGGTTCCAGTTACACTTTCTATGACCATTTTCTGCGCGACCGCACCCCA
GCF_001636325.1_ASM163632v1_genomic.fna_2:107286-107699 Satlength=414 Nr of Repeats=8 RepeatLength=52 seed=AAACTATGAC Num.seqs=5 Similarity=0.812820 18 AATTGGTTCCAGTTAAGCAAACTATGACCATTTTCAGCTTGACTACACCCAA
GCF_001636325.1_ASM163632v1_genomic.fna_2:1098902-1099109 Satlength=208 Nr of Repeats=4 RepeatLength=52 seed=GGTTCCAGTT Num.seqs=3 Similarity=0.846154 56 TTTTGGTTCCAGTTACACAATCTAAGACCATTTTTCGCTTACCTACACCCAA

Rev.of_GCF_001636325.1_ASM163632v1_genomic.fna_2:1233431-1233639
Satlength=209 Nr of Repeats=4 RepeatLength=52 seed=GTGTAGGCAA Num.seqs=4
Similarity=0.901709 100
TTTTGGTTCCAGTTACTCAATCTATGACCATTTTCTGTTTGCCTACACCTAA

************* * *** ********* * * **** *
Consensus:

ttTTGGTTCCAGTTAcaCaatCTAtGACCATTTTctGcttgaCtaCACCcaA

>Bacillus_Fam_192_45_4 Nr. of seq. 4 Alignment length(with gaps) = 45
Alignment score = 0.887654
GCF_000832605.1_ASM83260v1_genomic.fna_1:3713478-3713688 Satlength=211 Nr of Repeats=5 RepeatLength=45 seed=CAAAATCCAT Num.seqs=3
Similarity=0.597222 0
CAAAATCCATATGCAGCANCGC-AACTCAAGAACAACAATATCAG
Rev.of_GCF_000007825.1_ASM782v1_genomic.fna_1:2093339-2093558
Satlength=220 Nr of Repeats=5 RepeatLength=45 seed=CATATGGATT Num.seqs=4
Similarity=0.876543 13
CAAAATCCATATGTAGCACCACAAAACCAAGAACAACAATATCAA
Rev.of_GCF_000831065.1_ASM83106v1_genomic.fna_1:1910267-1910531
Satlength=265 Nr of Repeats=6 RepeatLength=45 seed=CATATGGATT Num.seqs=5
Similarity=0.905185 13
CAAAATCCATATGCAGCACCACAAAACCAAGAACAACAATATCAA
GCF_001645555.1_ASM164555v1_genomic.fna_12:630892-631111 Satlength=220 Nr of Repeats=5 RepeatLength=45 seed=CAAAATCCAT Num.seqs=4
Similarity=0.742754 45
CAAAATCCATATGCAGCACCACAAAATCAAGAACAACAATATCAA

************* **** * * ** *****************
Consensus:

CAAAATCCATATGcAGCAcCaCaAAacCAAGAACAACAATATCAa

>Bacillus_Fam_193_42_4 Nr. of seq. 4 Alignment length(with gaps) = 43
Alignment score = 0.714470
GCF_001591425.1_ASM159142v1_genomic.fna_67:17-228 Satlength=212 Nr of Repeats=5 RepeatLength=42 seed=TGGCCGAAAA Num.seqs=4 Similarity=0.830688
0 TGGCCG-AAAAGCGACTGCAGTTGGCGCGAAAGCAAGTTGGGT
GCF_001591425.1_ASM159142v1_genomic.fna_102:7924-8407 Satlength=484 Nr of Repeats=14 RepeatLength=42 seed=TTGGCGTGAA Num.seqs=9
Similarity=0.848325
20 TGGCCGAAAAAGCGT-CGCAGTTGGCGTGAAAGTGAGCTCAGT
GCF_001591425.1_ASM159142v1_genomic.fna_150:3-528 Satlength=526 Nr of Repeats=11 RepeatLength=42 seed=GGCCGAAAAA Num.seqs=9
Similarity=0.739506
43 TGGCCGAAAAAGCGA-TGCAGTTGGCGTGAAAGTGAGTTCGGT
GCF_001591425.1_ASM159142v1_genomic.fna_97:4685-4958 Satlength=274 Nr of Repeats=7 RepeatLength=42 seed=TGGCCGAAAA Num.seqs=6 Similarity=0.815873
0 TGGCCGAAAAAGTAA-CCCAGTTGGCGCAAAAGTCCGTTCAGT

****** ***** ********* **** * * **
Consensus:

TGGCCGaAAAAGcgacgCAGTTGGCGcgAAAGtgaGtTcaGT

>Bacillus_Fam_194_40_4 Nr. of seq. 4 Alignment length(with gaps) = 40
Alignment score = 0.730556
GCF_000153365.1_ASM15336v1_genomic.fna_18:178288-178478 Satlength=191 Nr of Repeats=5 RepeatLength=38 seed=AAAAGAGCCA Num.seqs=3 Similarity=0.906433
0   AAAAGAGCCAACGGAACCGTCCCCCTGGCCCAGTTTCA--
Rev.of_GCF_000473245.1_ASM47324v1_genomic.fna_1:774018-774208 Satlength=191 Nr of Repeats=5 RepeatLength=38 seed=TTGGCTCTTT Num.seqs=3 Similarity=0.906433   11
AAAAGAGCCAACGGAACCGTCCCCCTGGCCCAGTTTCA--
GCF_000153365.1_ASM15336v1_genomic.fna_18:449580-449794 Satlength=215 Nr of Repeats=5 RepeatLength=40 seed=GGTCTGTCCC Num.seqs=3 Similarity=0.911111
13   AAATGAGCCATGGGGTCTGTCCCCCCGGCCCTGCTCCAAA
Rev.of_GCF_000473245.1_ASM47324v1_genomic.fna_1:503588-503802 Satlength=215 Nr of Repeats=5 RepeatLength=40 seed=GGGACAGACC Num.seqs=3 Similarity=0.911111   23
AAATGAGCCATGGGGTCTGTCCCCCCGGCCCTGCTCCAAA

*** ****** ** * ******* ***** * * **
Consensus:

AAAaGAGCCAacGGaaCcGTCCCCCcGGCCCaGcTcCAaa

>Bacillus_Fam_195_40_4 Nr. of seq. 4 Alignment length(with gaps) = 40
Alignment score = 0.661111
GCF_000612665.1_FF4_genomic.fna_56:12424-12580 Satlength=157 Nr of Repeats=4 RepeatLength=39 seed=GTAAAGCGGT Num.seqs=4 Similarity=0.880342
0   GTAAAGCGGTGATGCGCTGGGGGCCAGTCCCCCGCT-AAG
Rev.of_GCF_000612665.1_FF4_genomic.fna_72:69320-69476 Satlength=157 Nr of Repeats=4 RepeatLength=39 seed=CTTCACCGCT Num.seqs=4 Similarity=0.863248
13   -TAAAGCGGTGAAGCGCTGGGGGACAGGCCCCCGCTGAAG
Rev.of_GCF_000612665.1_FF4_genomic.fna_72:117051-117206 Satlength=156 Nr of Repeats=4 RepeatLength=39 seed=CTGTCCCCCA Num.seqs=3 Similarity=0.977208
27   ATAAAGCGTTACTGTGATGGGGGACAGGCCCCCGCT-GTG
Rev.of_GCF_000612665.1_FF4_genomic.fna_72:111644-111839 Satlength=196 Nr of Repeats=5 RepeatLength=39 seed=GGGGGCCTGT Num.seqs=5 Similarity=0.829060
32   -TAAAGCAGTAACGCACTGAGGGACAGGCCCCCACCCAAG

****** *   *   ** *** *** ***** *   *
Consensus:

nTAAAGCggTaatGcgcTGgGGGaCAGgCCCCCgCtnaaG

>Bacillus_Fam_196_40_4 Nr. of seq. 4 Alignment length(with gaps) = 40
Alignment score = 0.686111
GCF_000285535.1_ASM28553v1_genomic.fna_7:68195-68355 Satlength=161 Nr of Repeats=5 RepeatLength=40 seed=GAGACGCAAA Num.seqs=3 Similarity=0.731707
0   GAGACGCAAAAATCTTAATTTCGACGCAAATCCTCAGAAT

GCF_000285535.1_ASM28553v1_genomic.fna_7:68195-69241 Satlength=1047 Nr of Repeats=16 RepeatLength=40 seed=GAGACGCAAA Num.seqs=12 Similarity=0.745455
0 GAGACGCAAAAATCTGAGTTATGACGCAAATCTTCAGAAC
Rev.of_GCF_000285535.1_ASM28553v1_genomic.fna_56:13-336 Satlength=324 Nr of Repeats=8 RepeatLength=40 seed=TTTTTGCGTC Num.seqs=5 Similarity=0.739683
12 CAGACGCAAAAATAAGGATTTCGACGCAAATCCGTGGANG
GCF_000285535.1_ASM28553v1_genomic.fna_57:33-354 Satlength=322 Nr of Repeats=8 RepeatLength=40 seed=GACGCAAAAA Num.seqs=7 Similarity=0.723810
42 CAGACGCAAAAATTAGGATTTCGACGCAAATCCNTGGGCG

```
************     **  **********    *
```

Consensus:

cAGACGCAAAAATcagaaTTtcGACGCAAATCctcaGaag

>Bacillus_Fam_197_36_4 Nr. of seq. 4 Alignment length(with gaps) = 36 Alignment score = 0.608025
GCF_000007825.1_ASM782v1_genomic.fna_1:2540573-2540735 Satlength=163 Nr of Repeats=5 RepeatLength=36 seed=CCAGTCGGTC Num.seqs=4 Similarity=0.833333
0 CCAGTCGGTCCAGTAATACCAGTCCTTCCAGTAACA
GCF_000017425.1_ASM1742v1_genomic.fna_1:1048468-1048891 Satlength=424 Nr of Repeats=11 RepeatLength=36 seed=CCAGTAATAC Num.seqs=9 Similarity=0.962963 9
CCAGTTGGTCCAGTAATACCAGTTTCTCCTGTAGGT
Rev.of_GCF_001895885.1_ASM189588v1_genomic.fna_1:1987676-1989071 Satlength=1396 Nr of Repeats=31 RepeatLength=36 seed=GGGATCACTG Num.seqs=26 Similarity=0.727521
20 CCAGTTGATCCAGTGATCCCTGTNGCNCCAGTAGGT
GCF_000715205.1_SOAPdenovo_v1.05_genomic.fna_25:483-1086 Satlength=604 Nr of Repeats=10 RepeatLength=36 seed=CCCGTGTTTC Num.seqs=6 Similarity=0.802469 18
CCAGTCGGACCACTTGTACCCGTGTTTCCTGTTGCA

```
***** *  *** *  * ** **    ** **
```

Consensus:

CCAGTcGgtCCAgTaaTaCCaGTntctCCaGTagca

>Bacillus_Fam_198_36_4 Nr. of seq. 4 Alignment length(with gaps) = 36 Alignment score = 0.804012
GCF_000007845.1_ASM784v1_genomic.fna_1:2735864-2736314 Satlength=451 Nr of Repeats=12 RepeatLength=36 seed=TCTTCCTTAG Num.seqs=11 Similarity=0.805387 0
TCTTCCTTAGGTTTNGCTACTTCTTGGACTTTCACT
Rev.of_GCF_001645555.1_ASM164555v1_genomic.fna_51:179190-179442 Satlength=253 Nr of Repeats=7 RepeatLength=36 seed=GAAAGTCCAA Num.seqs=7 Similarity=0.915344 33
TCTTCCTTAGGCTTTACTACTTCTTGGACTTTCGCT
GCF_000008505.1_ASM850v1_genomic.fna_1:2775427-2776093 Satlength=667 Nr of Repeats=18 RepeatLength=36 seed=CTTCTTCCTT Num.seqs=17

Similarity=0.784314 34
TCTTCCTTAGCTTTNGCTATTTCTTGTGCTTTTGCT
GCF_000496285.1_ASM49628v1_genomic.fna_1:232428-232860 Satlength=433 Nr of Repeats=10 RepeatLength=36 seed=GCTATTTCTT Num.seqs=8 Similarity=0.873016
51 TCTTCCTTAGCCTTTGCTATTTCTTGGGCTTTCGCT

********** ** *** ****** **** **
Consensus:

TCTTCCTTAGccTTtgCTAcTTCTTGgaCTTTcgCT

>Bacillus_Fam_199_36_4 Nr. of seq. 4 Alignment length(with gaps) = 36 Alignment score = 0.658951
GCF_000775975.1_ASM77597v1_genomic.fna_1:1148498-1150349 Satlength=1852 Nr of Repeats=44 RepeatLength=36 seed=CCCGTGTTAC Num.seqs=28 Similarity=0.803155 0
CCCGTGTTACCTTGAGCNCCTTGAGGTCCAGTAGCA
Rev.of_GCF_001645555.1_ASM164555v1_genomic.fna_45:36-216 Satlength=181 Nr of Repeats=5 RepeatLength=36 seed=TCAAGGTAAC Num.seqs=3 Similarity=0.851852 15
CCCGTGTTACCTTGAGCACCCTGAGGTCCAGTAGCG
GCF_001645555.1_ASM164555v1_genomic.fna_50:0-323 Satlength=324 Nr of Repeats=9 RepeatLength=36 seed=GTGTTACCTT Num.seqs=9 Similarity=0.797297
3 CCCGTGTTACCTTGAGGTCCAGTAGCACCGGTAGCG
GCF_001439635.1_ASM143963v1_genomic.fna_165:51610-51799 Satlength=190 Nr of Repeats=5 RepeatLength=36 seed=CCCTTATCTC Num.seqs=4 Similarity=0.827160
9 CCTGTGTCACCCTTATCTCCTTGTGGTCCTGTTGCT

** **** *** * *   **   * ** ** **
Consensus:

CCcGTGTtACCtTgAgctCCttgaGgtCCaGTaGCg

>Bacillus_Fam_200_36_4 Nr. of seq. 4 Alignment length(with gaps) = 36 Alignment score = 0.780864
GCF_001439635.1_ASM143963v1_genomic.fna_69:47-182 Satlength=136 Nr of Repeats=4 RepeatLength=36 seed=CCAGTAGGTC Num.seqs=3 Similarity=0.851852
0 CCAGTAGGTCCAGTGTTNCCAGTAAATCCNGTAGCC
GCF_001439635.1_ASM143963v1_genomic.fna_173:47-182 Satlength=136 Nr of Repeats=4 RepeatLength=36 seed=CCAGTAGGTC Num.seqs=3 Similarity=0.925926
0 CCAGTAGGTCCAGTGTTCCCAGTAAATCCGGTAGCC
GCF_001439635.1_ASM143963v1_genomic.fna_175:55401-55545 Satlength=145 Nr of Repeats=4 RepeatLength=36 seed=GTAAATCCGG Num.seqs=4 Similarity=0.889640
21 CCAGTAGGCCCAGTGATTCCGGTAAATCCGGTAGCC
Rev.of_GCF_001645555.1_ASM164555v1_genomic.fna_8:14-248 Satlength=235 Nr of Repeats=5 RepeatLength=36 seed=ACTGGGAACA Num.seqs=4 Similarity=0.787538
5 CCAGTAGGTCCGGTGATGCCGGTAGATCCAGTGTTC

******* ** *** * ** *** **** **   *
Consensus:

CCAGTAGGtCCaGTGaTnCCaGTAaATCCgGTagcC

>Bacillus_Fam_201_33_4 Nr. of seq. 4 Alignment length(with gaps) = 35
Alignment score = 0.651587
GCF_001578185.1_ASM157818v1_genomic.fna_1:3749385-3749913 Satlength=529
Nr of Repeats=16 RepeatLength=33 seed=CCTCCCATCA Num.seqs=16
Similarity=0.768919 0
-CCTCCCATCATTCCTGGTTGACCTCCTGGATGG-
GCF_001578185.1_ASM157818v1_genomic.fna_1:3749385-3750507 Satlength=1123
Nr of Repeats=33 RepeatLength=33 seed=CCTCCCATCA Num.seqs=32
Similarity=0.696781 0
-CCTCCCATCATNCCTGGNTGGCCTCCTTGATGA-
GCF_001578185.1_ASM157818v1_genomic.fna_1:3750017-3750692 Satlength=676
Nr of Repeats=16 RepeatLength=33 seed=CATCATGCCT Num.seqs=12
Similarity=0.815211 5
-CTTCCCATCATGCCTGAATGGCTTCCTTGATGAC
GCF_001645685.2_ASM164568v2_genomic.fna_1:3344937-3345585 Satlength=649
Nr of Repeats=19 RepeatLength=33 seed=CCGCCCATCA Num.seqs=12
Similarity=0.884910 1
ACCGCCCATCATGCCGGGCTGACCTCCATTTTG--

* ******** ** * ** * ***   **
Consensus:

CctCCCATCATgCCtGgnTGaCcTCCttgaTGa

>Bacillus_Fam_202_27_4 Nr. of seq. 4 Alignment length(with gaps) = 28
Alignment score = 0.646825
GCF_000262045.1_KCTC_13613_01_genomic.fna_51:24-285 Satlength=262 Nr of
Repeats=7 RepeatLength=27 seed=CCAGTTACTC Num.seqs=5 Similarity=0.945679
0 -CCAGTTACTCCTGTTGCTCCTGTTGGA
GCF_001712755.1_ASM171275v1_genomic.fna_9:108050-108311 Satlength=262 Nr
of Repeats=11 RepeatLength=27 seed=GCTCCTGTCG Num.seqs=7
Similarity=0.985891 7
TCCAGTTGCTCCTGTCGCTCCAGCTGT-
Rev.of_GCF_000706725.1_ASM70672v1_genomic.fna_1:1393225-1394061
Satlength=837 Nr of Repeats=18 RepeatLength=27 seed=TTAGATGGAG
Num.seqs=13 Similarity=0.953150
8 -CCATCTAATCCTGTCGGTCCCGTTGCT
GCF_002019765.1_ASM201976v1_genomic.fna_1:2612055-2612922 Satlength=868
Nr of Repeats=22 RepeatLength=27 seed=TCCATCTAAT Num.seqs=18
Similarity=0.917050 26
-CCATCTAATCCTGTCGGTCCCGTTGCT

*** * ****** * *** * **
Consensus:

CCAgcTaaTCCTGTcGcTCCcGtTGct

>Bacillus_Fam_203_27_4 Nr. of seq. 4 Alignment length(with gaps) = 27
Alignment score = 0.711934

BACILLUS_PSEUDALCALIPHILUS_genomic.fna_2:129261-129369 Satlength=109 Nr of Repeats=4 RepeatLength=27 seed=GGAGTAACCG Num.seqs=4 Similarity=0.893004
0 GGAGTAACCGGAGCGACAGGAATGCCA
GCF_000007845.1_ASM784v1_genomic.fna_1:2280509-2280779 Satlength=271 Nr of Repeats=9 RepeatLength=27 seed=GGAATAACGG Num.seqs=8 Similarity=0.821869
18 GGAGTGACAGGTCCAACAGGAATAACG
GCF_000008505.1_ASM850v1_genomic.fna_1:2328181-2328613 Satlength=433 Nr of Repeats=14 RepeatLength=27 seed=GGAATAACGG Num.seqs=12 Similarity=0.831650 18
GGAGCAACAGGTCCGACGGGAATAACG
Rev.of_GCF_000017425.1_ASM1742v1_genomic.fna_1:3294429-3294681 Satlength=253 Nr of Repeats=9 RepeatLength=27 seed=GTTGCTCCTG Num.seqs=8 Similarity=0.987654 35
GGAGCAACCGGTCCAACGGGAATAACA

**** ** ** * ** ***** *
Consensus:

GGAGcaACaGGtcCaACaGGAATaaCa

>Bacillus_Fam_204_25_4 Nr. of seq. 4 Alignment length(with gaps) = 25 Alignment score = 0.617778
GCF_000175075.1_ASM17507v1_genomic.fna_44:33119-33407 Satlength=289 Nr of Repeats=11 RepeatLength=24 seed=GCTGCTGCTT Num.seqs=10 Similarity=0.851852
0 GCTGCTGCTTCTGCCTCCGC-TTTG
Rev.of_GCF_001636335.1_ASM163633v1_genomic.fna_1:273946-274090 Satlength=145 Nr of Repeats=6 RepeatLength=24 seed=GCCAAAGCGA Num.seqs=6 Similarity=0.911111 2
GCCTCTGCTTCTGCCTTCGC-TTTG
Rev.of_GCF_000321185.1_ASM32118v1_genomic.fna_111:34583-34703 Satlength=121 Nr of Repeats=5 RepeatLength=24 seed=CAGGCAAAAG Num.seqs=5 Similarity=0.933333 4 -
CCTGCGCCTCTGCTTCAGCTTTTG
Rev.of_GCF_000430785.1_ASM43078v1_genomic.fna_23:12805-12925 Satlength=121 Nr of Repeats=5 RepeatLength=24 seed=AAAGCAGAGG Num.seqs=5 Similarity=0.977778 22
-CTTTCGCATCTGCCTCTGCTTTTG

* ** ***** * ** ****
Consensus:

gCctccGCtTCTGCcTccGCtTTTG

>Bacillus_Fam_205_24_4 Nr. of seq. 4 Alignment length(with gaps) = 24 Alignment score = 0.805556
GCF_000007825.1_ASM782v1_genomic.fna_1:1044010-1044106 Satlength=97 Nr of Repeats=4 RepeatLength=24 seed=CCTGGAACAC Num.seqs=4 Similarity=0.824074
0 CCTGGAACACCAGATCCAGAAAAA
GCF_000831065.1_ASM83106v1_genomic.fna_1:894250-894442 Satlength=193 Nr of Repeats=7 RepeatLength=24 seed=ATCCAGAAAA Num.seqs=6 Similarity=0.881481
13 CCTGGAACACCAGATCCAGAAAAA

Rev.of_GCF_000775975.1_ASM77597v1_genomic.fna_1:3721872-3721992 Satlength=121 Nr of Repeats=5 RepeatLength=24 seed=TCTGGATCTG Num.seqs=5 Similarity=0.833333 20 CCGGAAAAACCAGATCCAGAAAAA
Rev.of_GCF_001645555.1_ASM164555v1_genomic.fna_16:33671-33767 Satlength=97 Nr of Repeats=4 RepeatLength=24 seed=TCTGGATCCG Num.seqs=4 Similarity=0.935185 20 CCAGAAACAACGGATCCAGAGAAA

** * ** * * ******** ***
Consensus:

CCtGaAAcAcCaGATCCAGAaAAA

>Bacillus_Fam_206_20_4 Nr. of seq. 4 Alignment length(with gaps) = 23 Alignment score = 0.718599
GCF_001877785.1_PlanoSAMM_genomic.fna_33:9772-9852 Satlength=81 Nr of Repeats=4 RepeatLength=20 seed=TATGATCCAT Num.seqs=4 Similarity=0.542929 0 TATGATCCATT---CTGAAATTA
Rev.of_GCF_900094975.1_IMG-taxon_2617270721_annotated_assembly_genomic.fna_4:70413-70513 Satlength=101 Nr of Repeats=5 RepeatLength=20 seed=AATGGATCAT Num.seqs=5 Similarity=0.693939 11 TATGATCCATT-T-CNGAAATA-
GCF_001877785.1_PlanoSAMM_genomic.fna_85:133615-133695 Satlength=81 Nr of Repeats=4 RepeatLength=20 seed=TATGATCCAT Num.seqs=4 Similarity=0.671958 21 TATGATCCATT-TCCAGAAATA-
GCF_001877785.1_PlanoSAMM_genomic.fna_106:22377-22457 Satlength=81 Nr of Repeats=4 RepeatLength=20 seed=TATGATCCAT Num.seqs=4 Similarity=0.683862 21 TATGATCCATTCT-CAGAAATT-

*********** * *****
Consensus:

TATGATCCATTtCaGAAATa

>Bacillus_Fam_207_21_4 Nr. of seq. 4 Alignment length(with gaps) = 23 Alignment score = 0.642512
GCF_000612805.1_FF3_genomic.fna_1:291005-291088 Satlength=84 Nr of Repeats=4 RepeatLength=21 seed=AACATGGAAT Num.seqs=3 Similarity=0.809524 0 AACATGGAATAAA-C-TCCGNAA
GCF_000612805.1_FF3_genomic.fna_1:447160-447244 Satlength=85 Nr of Repeats=4 RepeatLength=21 seed=CATGGAATAA Num.seqs=4 Similarity=0.654040 2 TTCATGGAATAAA-C-TCTGAAA
GCF_000612805.1_FF3_genomic.fna_8:67783-67867 Satlength=85 Nr of Repeats=4 RepeatLength=21 seed=TGGAATAAAC Num.seqs=4 Similarity=0.732323 4 AACATGGAATAAA-CNTCTGANA
GCF_000612805.1_FF3_genomic.fna_3:573446-573530 Satlength=85 Nr of Repeats=4 RepeatLength=21 seed=GTAGAATAAA Num.seqs=4 Similarity=0.646465 3 AATGTAGAATAAANC-TCCGAAA

* ******* * ** * *
Consensus:

aacaTgGAATAAACTCcGaaA

>Bacillus_Fam_208_21_4 Nr. of seq. 4 Alignment length(with gaps) = 22
Alignment score = 0.604798
GCF_000294775.2_ASM29477v2_genomic.fna_1:4944734-4944837 Satlength=104 Nr of Repeats=5 RepeatLength=21 seed=TCTATTTGCA Num.seqs=3 Similarity=0.490741 0
TCTATTTGCAG-NTTTNCGGC-
Rev.of_GCF_001591465.1_ASM159146v1_genomic.fna_37:37195-37419 Satlength=225 Nr of Repeats=10 RepeatLength=20 seed=CTGCAAATAG Num.seqs=6 Similarity=0.659788
10   CCTATTTGCAG--TTTCCGGTT
Rev.of_GCF_000986785.1_ASM98678v1_genomic.fna_5:144649-145040 Satlength=392 Nr of Repeats=15 RepeatLength=21 seed=GCAAATAGAA Num.seqs=11 Similarity=0.592496
9   TCTATTTGCCG-NTTTCCNANT
Rev.of_GCF_000986785.1_ASM98678v1_genomic.fna_4:84896-85225 Satlength=330 Nr of Repeats=12 RepeatLength=21 seed=GCAAATAGAA Num.seqs=8 Similarity=0.590278 9
TCTATTTGCCGATTTTCNCATT

******** * ***
Consensus:

tCTATTTGCaGnTTTccgatt

>Bacillus_Fam_209_22_4 Nr. of seq. 4 Alignment length(with gaps) = 22
Alignment score = 0.651515
GCF_000401235.1_BacNeaAAU1_genomic.fna_227:43989-44111 Satlength=123 Nr of Repeats=6 RepeatLength=20 seed=AGTCGCAAGT Num.seqs=4 Similarity=0.837302
0   AGTCGCAAGTAAGAG-GG-GAG
Rev.of_GCF_000401235.1_BacNeaAAU1_genomic.fna_157:4403-4508 Satlength=106 Nr of Repeats=5 RepeatLength=21 seed=TTACTTGCGA Num.seqs=5 Similarity=0.715151 12
AGTCGCAAGTAAAAG-GGTGAA
Rev.of_GCF_000401235.1_BacNeaAAU1_genomic.fna_154:1239-1364 Satlength=126 Nr of Repeats=6 RepeatLength=21 seed=CTTGCGACTC Num.seqs=5 Similarity=0.743939 9
AGTCGCAAGAAAAAGTGTCGG-
Rev.of_GCF_000401235.1_BacNeaAAU1_genomic.fna_164:34058-34338 Satlength=281 Nr of Repeats=13 RepeatLength=21 seed=TTACTTGCGA Num.seqs=12 Similarity=0.826840
12   AGTCGCAAGTAAAGGTGGGGA-

********* ** * * *
Consensus:

AGTCGCAAGtAAaaGtGgnGan

>Bacillus_Fam_210_21_4 Nr. of seq. 4 Alignment length(with gaps) = 22
Alignment score = 0.650253
GCF_000986785.1_ASM98678v1_genomic.fna_3:201618-201723 Satlength=106 Nr of Repeats=5 RepeatLength=21 seed=GTAGAGTAAA Num.seqs=5

Similarity=0.442667
0   GTAGAGTAAACT-AANAAATT-
GCF_001315085.1_ASM131508v1_genomic.fna_38:31481-31582 Satlength=102 Nr of Repeats=4 RepeatLength=20 seed=TTAGTAGATA Num.seqs=3 Similarity=0.631313
17   GTAGA-TAAAAT-CAGAAATTA
GCF_000986785.1_ASM98678v1_genomic.fna_25:55323-55428 Satlength=106 Nr of Repeats=4 RepeatLength=21 seed=GTAGAGTAAA Num.seqs=3 Similarity=0.505050
0   GTAGAGTAAANTNCTGAAANT-
GCF_000986785.1_ASM98678v1_genomic.fna_39:16547-16652 Satlength=106 Nr of Repeats=4 RepeatLength=21 seed=GTAGAGTAAA Num.seqs=3 Similarity=0.505050
0   GTAGAGTAAANTNCTGAAANT-

***** **** *   *** *
Consensus:

GTAGAgTAAAnTncagAAAtT

>Bacillus_Fam_211_21_4 Nr. of seq. 4 Alignment length(with gaps) = 22 Alignment score = 0.656566
GCF_001636335.1_ASM163633v1_genomic.fna_1:2068723-2068964 Satlength=242 Nr of Repeats=12 RepeatLength=20 seed=TTTGCGAATT Num.seqs=9 Similarity=0.766667   0
TTTGCGAATTTCATT-C-TTTA
Rev.of_GCF_000508325.1_BAVI_1_genomic.fna_111:18515-18598 Satlength=84 Nr of Repeats=4 RepeatLength=21 seed=ATTCGCTAAT Num.seqs=3 Similarity=0.626263   9
TTAGCGAATTTCNAA-CATTTA
Rev.of_GCF_000752035.1_Bacillus_rubiinfantis_genomic.fna_30:250624-250707 Satlength=84 Nr of Repeats=4 RepeatLength=21 seed=TCGCTAATAA Num.seqs=3 Similarity=0.746032   29   TTAGCGAA-TTCAAAGCATTTA
Rev.of_GCF_000752035.1_Bacillus_rubiinfantis_genomic.fna_18:190500-190624 Satlength=125 Nr of Repeats=6 RepeatLength=21 seed=TCGCTAATAA Num.seqs=4 Similarity=0.666667   28
TTAGCGATTTTCAAT-CACTTA

** **** ***   * ***
Consensus:

TTaGCGAatTTCaaaCatTTA

>Bacillus_Fam_212_21_4 Nr. of seq. 4 Alignment length(with gaps) = 22 Alignment score = 0.631313
GCF_900104555.1_IMG-taxon_2651870172_annotated_assembly_genomic.fna_16:258288-258369 Satlength=82 Nr of Repeats=4 RepeatLength=20 seed=TATCTACGAA Num.seqs=3 Similarity=0.777778   0   TATCTACG-AAA-ATACAGATA
Rev.of_GCF_900104555.1_IMG-taxon_2651870172_annotated_assembly_genomic.fna_22:509247-509328 Satlength=82 Nr of Repeats=4 RepeatLength=20 seed=TTTCGTAGAT Num.seqs=3 Similarity=0.777778   11   TATCTACG-AAATTTAAA-ATA
Rev.of_GCF_900104555.1_IMG-taxon_2651870172_annotated_assembly_genomic.fna_22:513986-514066

Satlength=81 Nr of Repeats=4 RepeatLength=20 seed=TTCGTAGATA Num.seqs=4 Similarity=0.636243   30   TATCTACG-AAATTTAGA-ATA
Rev.of_GCF_000759675.1_ASM75967v1_genomic.fna_19:516952-517036 Satlength=85 Nr of Repeats=4 RepeatLength=21 seed=TATCTACACA Num.seqs=4 Similarity=0.461353   0
TA-CTACGAAAATGTGTAGATA

** ***** *** * * ***
Consensus:

TAtCTACGAAAttTanAgATA

>Bacillus_Fam_213_21_4 Nr. of seq. 4 Alignment length(with gaps) = 22 Alignment score = 0.611111
GCF_000171615.1_ASM17161v1_genomic.fna_75:39270-39436 Satlength=167 Nr of Repeats=7 RepeatLength=21 seed=TAAAAACAGA Num.seqs=5 Similarity=0.666667
0   TAAAAACAGAAATGCAAGAGG-
GCF_001591425.1_ASM159142v1_genomic.fna_42:3633-3717 Satlength=85 Nr of Repeats=4 RepeatLength=21 seed=ATGAAAGCAG Num.seqs=4 Similarity=0.724868
10   -ACAAACTGAAATGAAAGCAGA
GCF_001591465.1_ASM159146v1_genomic.fna_37:32146-32471 Satlength=326 Nr of Repeats=6 RepeatLength=21 seed=GAAATGAAAA Num.seqs=4 Similarity=0.862434
28   -ACAACAAGAAATGAAAAAAGA
Rev.of_GCF_900109925.1_IMG-taxon_2651870116_annotated_assembly_genomic.fna_21:114898-115087 Satlength=190 Nr of Repeats=8 RepeatLength=21 seed=TCTTGTTTCA Num.seqs=7 Similarity=0.806500   42   -ACTACAAGAAATGAAACAAGA

* *   ****** **   *
Consensus:

AcaAaaaGAAATGaAAgaaGa

>Bacillus_Fam_214_22_4 Nr. of seq. 4 Alignment length(with gaps) = 22 Alignment score = 0.623737
GCF_000331575.1_ASM33157v1_genomic.fna_1:1705037-1705121 Satlength=85 Nr of Repeats=4 RepeatLength=21 seed=ATTTATTGTC Num.seqs=4 Similarity=0.548309
0   ATTTATTGTCCGGAAACAAGG-
GCF_000331575.1_ASM33157v1_genomic.fna_4:111890-112405 Satlength=516 Nr of Repeats=18 RepeatLength=21 seed=TTTATTGTCC Num.seqs=13 Similarity=0.806268   1
TTTTATTGTCCGGAATCAAAT-
Rev.of_GCF_001315045.1_ASM131504v1_genomic.fna_25:44801-44885 Satlength=85 Nr of Repeats=4 RepeatLength=21 seed=GGACAATAAA Num.seqs=4 Similarity=0.453704   11
ATTTATTGTCC-GATTCTAGTG
GCF_001315045.1_ASM131504v1_genomic.fna_42:24951-25035 Satlength=85 Nr of Repeats=4 RepeatLength=21 seed=TTTATTGTCC Num.seqs=4 Similarity=0.420940
22   -TTTATTGTCCAGATACTCATG

********** ** *
Consensus:

aTTTATTGTCCgGAaaCaaatg

>Bacillus_Fam_215_22_4 Nr. of seq. 4 Alignment length(with gaps) = 22 Alignment score = 0.694444
GCF_000401235.1_BacNeaAAU1_genomic.fna_108:5084-5249 Satlength=166 Nr of Repeats=8 RepeatLength=21 seed=ATCAGCCAAA Num.seqs=5 Similarity=0.809524
12   AATGAG-GGGGAAATCAGCCAA
Rev.of_GCF_001591585.1_ASM159158v1_genomic.fna_22:73236-73362 Satlength=127 Nr of Repeats=6 RepeatLength=21 seed=TTTGGCTGAT Num.seqs=6 Similarity=0.771717   21
AGTGAGTGGGGAA-TCAGCCAA
Rev.of_GCF_001591585.1_ASM159158v1_genomic.fna_40:17715-17818 Satlength=104 Nr of Repeats=5 RepeatLength=21 seed=TTTGGCTGAT Num.seqs=3 Similarity=0.809524   22
AGTAAG-ANGGAAATCAGCCAA
Rev.of_GCF_001591585.1_ASM159158v1_genomic.fna_3:180090-180216 Satlength=127 Nr of Repeats=6 RepeatLength=21 seed=CTTTGGCTGA Num.seqs=6 Similarity=0.839153   22
AGCGGGGAGGGAA-TCAGCCAA

*********   *   ****
Consensus:

aTCAGCCAAAgtgaGnagGGAA

>Bacillus_Fam_216_22_4 Nr. of seq. 4 Alignment length(with gaps) = 22 Alignment score = 0.702020
GCF_000948175.1_BFA_2_A_genomic.fna_31:29597-29723 Satlength=127 Nr of Repeats=6 RepeatLength=21 seed=ATCTCTCGGT Num.seqs=6 Similarity=0.911111
0   ATCTCTCGGTTTTGCG-CTTTA
GCF_000948175.1_BFA_2_A_genomic.fna_185:0-83 Satlength=84 Nr of Repeats=4 RepeatLength=21 seed=AATCTCTCGG Num.seqs=4 Similarity=0.968254
20   ATCTCTCGGTTTTGCG-CTTTA
Rev.of_GCF_000948175.1_BFA_2_A_genomic.fna_39:8694-8799 Satlength=106 Nr of Repeats=5 RepeatLength=21 seed=AAACCGAGAG Num.seqs=5 Similarity=0.847619
12   ATCTCTCGGTTT-GCGGGAATA
GCF_000948175.1_BFA_2_A_genomic.fna_14:18049-18299 Satlength=251 Nr of Repeats=12 RepeatLength=21 seed=AATCTCTCGG Num.seqs=10 Similarity=0.679461
21   ATCTCTCGGTTTCGCGTCGNGA

************ ***   *
Consensus:

ATCTCTCGGTTTtGCGnctttA

>Bacillus_Fam_217_22_4 Nr. of seq. 4 Alignment length(with gaps) = 22 Alignment score = 0.601010
GCF_001591485.1_ASM159148v1_genomic.fna_6:6108-6192 Satlength=85 Nr of Repeats=4 RepeatLength=21 seed=TATTTGCGAA Num.seqs=4 Similarity=0.597884
11   T-TAGCACCTTTTATTTGCGAA

Rev.of_GCF_001591665.1_ASM159166v1_genomic.fna_53:38984-39068
Satlength=85 Nr of Repeats=4 RepeatLength=21 seed=ATTCGCAAAT Num.seqs=4
Similarity=0.735450 22
T-TGGGGGCGTTTATTTGCGAA
Rev.of_GCF_001591665.1_ASM159166v1_genomic.fna_78:8523-8607 Satlength=85
Nr of Repeats=4 RepeatLength=21 seed=TTCGCAAATA Num.seqs=4
Similarity=0.671958 20
TCTATGACGTTT-ATTTGCGAA
GCF_001591805.1_ASM159180v1_genomic.fna_15:21542-21647 Satlength=106 Nr
of Repeats=5 RepeatLength=21 seed=TTATTTGCGA Num.seqs=5
Similarity=0.683333
30 TCTAACNCTNTT-ATTTGCGAA

********** * **
Consensus:

tATTTGCGAATcTagcacctTT

>Bacillus_Fam_218_20_4 Nr. of seq. 4 Alignment length(with gaps) = 21
Alignment score = 0.669312
GCF_001439915.1_ASM143991v1_genomic.fna_94:254056-254153 Satlength=98 Nr
of Repeats=5 RepeatLength=19 seed=TTGCGCTTTA Num.seqs=3
Similarity=0.953216
0 TTGCG-CTTT-AGCCACATAT
GCF_000153365.1_ASM15336v1_genomic.fna_11:41215-41295 Satlength=81 Nr of
Repeats=4 RepeatLength=20 seed=TATATTTGCG Num.seqs=4 Similarity=0.648148
15 TTGCGAGTTT-CTCCATATAT
GCF_000473245.1_ASM47324v1_genomic.fna_1:3661356-3661436 Satlength=81 Nr
of Repeats=4 RepeatLength=20 seed=TATATTTGCG Num.seqs=4
Similarity=0.648148
15 TTGCGAGTTT-CTCCATATAT
Rev.of_GCF_001636315.1_ASM163631v1_genomic.fna_1:2663845-2663929
Satlength=85 Nr of Repeats=4 RepeatLength=21 seed=TCGCAGATAT Num.seqs=4
Similarity=0.593434 26
CTGCGAGTTTAGTCAATATAT

**** *** * * ****
Consensus:

tTGCGagTTTctCcAtATAT

>Bacillus_Fam_219_20_4 Nr. of seq. 4 Alignment length(with gaps) = 21
Alignment score = 0.671958
GCF_000401235.1_BacNeaAAU1_genomic.fna_6:1275-1356 Satlength=82 Nr of
Repeats=4 RepeatLength=20 seed=ATGATCCAAA Num.seqs=3 Similarity=0.822222
0 ATGATCCAAAA-ATCAAATAT
GCF_900094975.1_IMG-
taxon_2617270721_annotated_assembly_genomic.fna_9:33588-33669
Satlength=82 Nr of Repeats=4 RepeatLength=20 seed=TATTATCAAA Num.seqs=3
Similarity=0.644444 19 ATTATCAAAAA-
NNCAATTAT
GCF_900094975.1_IMG-
taxon_2617270721_annotated_assembly_genomic.fna_9:41410-41491
Satlength=82 Nr of Repeats=4 RepeatLength=20 seed=TATTATCAAA Num.seqs=3

Similarity=0.666667 19 ATTATCAAAAA-
TNGAATTAT
GCF_001877785.1_PlanoSAMM_genomic.fna_74:382717-382798 Satlength=82 Nr of Repeats=4 RepeatLength=20 seed=TATTATCAAA Num.seqs=3 Similarity=0.603175
19 ATTATCAAAAACAAGNATTAT

** *** ****   * ***
Consensus:

ATtATCaAAAAancaAtTAT

>Bacillus_Fam_220_21_4 Nr. of seq. 4 Alignment length(with gaps) = 21
Alignment score = 0.625661
GCF_000429705.1_ASM42970v1_genomic.fna_2:37535-37615 Satlength=81 Nr of Repeats=4 RepeatLength=20 seed=AATTGGCGGA Num.seqs=4 Similarity=0.805556
0 AATTGGCGGAGTTATTTATG-
Rev.of_GCF_000307875.1_BABA1.0_genomic.fna_7:97-181 Satlength=85 Nr of Repeats=4 RepeatLength=21 seed=ATTCCGCCAA Num.seqs=4 Similarity=0.571256
12 AGTTGGCGGAATTACTTNTGA
GCF_001591665.1_ASM159166v1_genomic.fna_21:113671-113776 Satlength=106 Nr of Repeats=4 RepeatLength=21 seed=TTGGCGGAAT Num.seqs=3
Similarity=0.505050 23
ATTTGGCGGAATTANTGGCGA
Rev.of_GCF_000948175.1_BFA_2_A_genomic.fna_71:44838-44964 Satlength=127 Nr of Repeats=5 RepeatLength=21 seed=AACTCCGTAA Num.seqs=4
Similarity=0.548309 4
AGTTCGCGGTNTTATTTACGG

* ** **** *** *   *
Consensus:

AgTTgGCGGaaTTAtTtacGa

>Bacillus_Fam_221_21_4 Nr. of seq. 4 Alignment length(with gaps) = 21
Alignment score = 0.634921
GCF_000181495.1_ASM18149v1_genomic.fna_184:58106-58409 Satlength=304 Nr of Repeats=5 RepeatLength=21 seed=ACTCGTCGAT Num.seqs=3
Similarity=0.787879
0 ACTCGTCGATAGAACCGCAAN
GCF_001591445.1_ASM159144v1_genomic.fna_34:15168-15252 Satlength=85 Nr of Repeats=4 RepeatLength=21 seed=CGCCAATAGA Num.seqs=4 Similarity=0.724868
3 ATTCGCCAATAGAACCGCAAA
Rev.of_GCF_001274935.1_ASM127493v1_genomic.fna_37:44679-44969
Satlength=291 Nr of Repeats=7 RepeatLength=21 seed=TTCTATCGAT Num.seqs=5
Similarity=0.733333 14
ANTGATCGATAGAAAAGCAAA
GCF_001439635.1_ASM143963v1_genomic.fna_74:26703-26787 Satlength=85 Nr of Repeats=4 RepeatLength=21 seed=TGACGGATAG Num.seqs=4 Similarity=0.733586
23 ACTGACGGATAGAAACTCAAA

* *   ******   ***
Consensus:

AcTcaccgATAGAAacgCAAa

>Bacillus_Fam_222_21_4 Nr. of seq. 4 Alignment length(with gaps) = 21
Alignment score = 0.701058
GCF_000508325.1_BAVI_1_genomic.fna_8:57470-57985 Satlength=516 Nr of Repeats=16 RepeatLength=21 seed=TGTCGTAAGT Num.seqs=10 Similarity=0.553704
0   TGTCGTAAGTGTCGAAAAATN
Rev.of_GCF_001591665.1_ASM159166v1_genomic.fna_65:32-187 Satlength=156 Nr of Repeats=6 RepeatLength=21 seed=ACTTACGACA Num.seqs=5 Similarity=0.809524 10
TGTCGTAAGTGGCTGAAAATA
GCF_001591665.1_ASM159166v1_genomic.fna_77:44-199 Satlength=156 Nr of Repeats=6 RepeatLength=21 seed=TGTCGTAAGT Num.seqs=5 Similarity=0.765079
21   TGTCGTAAGTNGCTGAAAATG
GCF_001591445.1_ASM159144v1_genomic.fna_24:47680-47785 Satlength=106 Nr of Repeats=5 RepeatLength=21 seed=ATGTAATAAG Num.seqs=5 Similarity=0.713636
20   TGTAATAAGNNTCCGAAAATA

*** ****   * *****
Consensus:

TGTcgTAAGtggCtgAAAATa

>Bacillus_Fam_223_21_4 Nr. of seq. 4 Alignment length(with gaps) = 21
Alignment score = 0.666667
GCF_000508325.1_BAVI_1_genomic.fna_47:49007-49090 Satlength=84 Nr of Repeats=4 RepeatLength=21 seed=TATCAGCGAA Num.seqs=3 Similarity=0.545455
0   TATCAGCGAATNTNCACNATA
GCF_000508325.1_BAVI_1_genomic.fna_89:42322-42405 Satlength=84 Nr of Repeats=4 RepeatLength=21 seed=TATCAGCGAA Num.seqs=3 Similarity=0.682540
0   TATCAGCGAATCTAANCAATA
GCF_001591445.1_ASM159144v1_genomic.fna_67:12088-12172 Satlength=85 Nr of Repeats=4 RepeatLength=21 seed=TTCTATCAGC Num.seqs=4 Similarity=0.767196
18   TATCAGCGAATTTAACCATTC
Rev.of_GCF_000508325.1_BAVI_1_genomic.fna_95:8292-8375 Satlength=84 Nr of Repeats=4 RepeatLength=21 seed=CGCTGATAAA Num.seqs=3 Similarity=0.565657
8   TATCAGCGNATTTCCGCANTT

******** ** *   * *
Consensus:

TATCAGCGaATtTaanCaaTa

>Bacillus_Fam_224_21_4 Nr. of seq. 4 Alignment length(with gaps) = 21
Alignment score = 0.724868
GCF_000508325.1_BAVI_1_genomic.fna_48:140-287 Satlength=148 Nr of Repeats=7 RepeatLength=21 seed=TTTACTGTCC Num.seqs=7 Similarity=0.611833
0   TTTACTGTCCACTTCTGNCCA
Rev.of_GCF_001591665.1_ASM159166v1_genomic.fna_53:13657-14008 Satlength=352 Nr of Repeats=10 RepeatLength=21 seed=GGACAGTAAA Num.seqs=7 Similarity=0.608225
10   TTTACTGTCCACTTTTANNCA

Rev.of_GCF_001591805.1_ASM159180v1_genomic.fna_27:47504-47609 Satlength=106 Nr of Repeats=5 RepeatLength=21 seed=GGACAGTAAA Num.seqs=5 Similarity=0.571014 10
TTTACTGTCCGGTTTTANCNA
GCF_001591805.1_ASM159180v1_genomic.fna_35:13044-13128 Satlength=85 Nr of Repeats=4 RepeatLength=21 seed=TCCGGTTTTG Num.seqs=4 Similarity=0.767196 28 TTTACTGTCCGGTTTTGACGA

```
**********  ** *    *
```

Consensus:

TTTACTGTCCacTTtTanccA

>Bacillus_Fam_225_20_4 Nr. of seq. 4 Alignment length(with gaps) = 20 Alignment score = 0.666667
GCF_000181495.1_ASM18149v1_genomic.fna_104:725-825 Satlength=101 Nr of Repeats=5 RepeatLength=20 seed=TTATCGGCGA Num.seqs=5 Similarity=0.682540 0 TTATCGGCGATTTCTGAATT
Rev.of_GCF_001636335.1_ASM163633v1_genomic.fna_1:1148631-1149011 Satlength=381 Nr of Repeats=19 RepeatLength=20 seed=TCGCAAATAA Num.seqs=17 Similarity=0.694742
10 TTATTTGCGATTTCNNATTT
GCF_001712755.1_ASM171275v1_genomic.fna_23:187080-187438 Satlength=359 Nr of Repeats=17 RepeatLength=20 seed=TTTATTTGCG Num.seqs=13 Similarity=0.688441 19
TTATTTGCGATTNCTGATAT
GCF_001645685.2_ASM164568v2_genomic.fna_1:4106820-4106922 Satlength=103 Nr of Repeats=5 RepeatLength=20 seed=TTTTATCTAC Num.seqs=3 Similarity=0.735450 18
TTATCTACGAATTCTGCNTT

```
****   *** * *     *
```

Consensus:

TTATctgCGAtTtCtgattT

>Bacillus_Fam_226_20_4 Nr. of seq. 4 Alignment length(with gaps) = 20 Alignment score = 0.788889
GCF_001648575.1_ASM164857v1_genomic.fna_58:901-1002 Satlength=102 Nr of Repeats=4 RepeatLength=20 seed=TTTCAGGATA Num.seqs=3 Similarity=0.636364 0 TTTCAGGATAAATCCCAAAA
Rev.of_GCF_001648575.1_ASM164857v1_genomic.fna_61:70721-70900 Satlength=180 Nr of Repeats=8 RepeatLength=20 seed=TTATCCTGAA Num.seqs=6 Similarity=0.630688 11
TTTCAGGATAAATCCGAAAT
Rev.of_GCF_001648575.1_ASM164857v1_genomic.fna_67:67316-67396 Satlength=81 Nr of Repeats=4 RepeatLength=20 seed=TTTATCCTGA Num.seqs=4 Similarity=0.805556 12
ATTCAGGATAAATCTCAAAA
Rev.of_GCF_001877785.1_PlanoSAMM_genomic.fna_150:75986-76073 Satlength=88 Nr of Repeats=4 RepeatLength=20 seed=TTTATGCTGA Num.seqs=3 Similarity=0.555556 12
ATTCAGCATAAATCTAAAAA

***** ******* ***
Consensus:

aTTCAGgATAAATCccAAAa

>Bacillus_Fam_227_18_4 Nr. of seq. 4 Alignment length(with gaps) = 19
Alignment score = 0.688596
GCF_000161455.1_ASM16145v1_genomic.fna_1:2935090-2935216 Satlength=127 Nr of Repeats=6 RepeatLength=18 seed=TCGCCTTGAG Num.seqs=5 Similarity=0.837037   0
TCGCCTTGAGGTCCTGGT-
GCF_000299035.1_ASM29903v1_genomic.fna_1:825324-826476 Satlength=1153 Nr of Repeats=60 RepeatLength=18 seed=CCTTGTTCGC Num.seqs=49 Similarity=0.850214   12
TCGCCCTGAGGTCCTTGT-
GCF_000334155.1_L1_genomic.fna_44:19664-22112 Satlength=2449 Nr of Repeats=125 RepeatLength=18 seed=TGAGGCCCTT Num.seqs=117 Similarity=0.883486
6   TCNCCTTGAGGCCCTTGT-
Rev.of_GCF_002019665.1_ASM201966v1_genomic.fna_1:1011858-1012110 Satlength=253 Nr of Repeats=9 RepeatLength=18 seed=GGTCCACAAG Num.seqs=6 Similarity=0.886420   14
TCTCCTTGTGGACCT-GTT

** ** ** ** *** **
Consensus:

TCgCCtTGaGGtCCTtGT

>Bacillus_Fam_228_18_4 Nr. of seq. 4 Alignment length(with gaps) = 18
Alignment score = 0.641975
GCF_000008505.1_ASM850v1_genomic.fna_1:2327569-2327767 Satlength=199 Nr of Repeats=9 RepeatLength=18 seed=GTGATGCCTG Num.seqs=7 Similarity=0.922399
0   GTGATGCCTGTAGGACCA
Rev.of_GCF_001587375.1_ASM158737v1_genomic.fna_6:3784-4054 Satlength=271 Nr of Repeats=15 RepeatLength=18 seed=CCTACTGGCG Num.seqs=15 Similarity=0.882892   14
GTTACGCCAGTAGGTCCA
Rev.of_GCF_001645555.1_ASM164555v1_genomic.fna_12:312575-312683 Satlength=109 Nr of Repeats=6 RepeatLength=18 seed=TTACTGGACC Num.seqs=6 Similarity=0.885965   4
GTAATACCTGTGGGTCCA
Rev.of_GCF_900096965.1_IMG-taxon_2671180223_annotated_assembly_genomic.fna_22:69506-70175 Satlength=670 Nr of Repeats=15 RepeatLength=18 seed=ACTGGTGCTA Num.seqs=14 Similarity=0.797667   2   GTGACCCCGGTAGCACCA

** * ** ** * ***
Consensus:

GTgAcgCCtGTaGgaCCA

>Bacillus_Fam_229_18_4 Nr. of seq. 4 Alignment length(with gaps) = 18
Alignment score = 0.716049
GCF_000177235.2_ASM17723v2_genomic.fna_1:713412-713628 Satlength=217 Nr of Repeats=11 RepeatLength=18 seed=CCAGGAGAAC Num.seqs=10 Similarity=0.899588 0
CCAGGAGAACCAAGTGAC
GCF_900096965.1_IMG-taxon_2671180223_annotated_assembly_genomic.fna_7:2615-2903 Satlength=289 Nr of Repeats=15 RepeatLength=18 seed=CCAGGAGATC Num.seqs=12 Similarity=0.841751 0 CCAGGAGATCCGGGCGAC
Rev.of_GCF_001887185.1_ASM188718v1_genomic.fna_2:899-971 Satlength=73 Nr of Repeats=4 RepeatLength=18 seed=TTGCTTGGAT Num.seqs=4 Similarity=0.802469
17 CCAGGGGATCCAAGCAAA
Rev.of_GCF_001887185.1_ASM188718v1_genomic.fna_105:8418-8580 Satlength=163 Nr of Repeats=9 RepeatLength=18 seed=TTGCTTGGAT Num.seqs=9 Similarity=0.823045 17
CCAGGAGATCCAAGCAAA

***** ** ** * *
Consensus:

CCAGGaGAtCCaaGcaAa

>Bacillus_Fam_230_18_4 Nr. of seq. 4 Alignment length(with gaps) = 18
Alignment score = 0.864198
GCF_000526655.1_ASM52665v1_genomic.fna_3:175348-175420 Satlength=73 Nr of Repeats=4 RepeatLength=18 seed=AGACCATTCG Num.seqs=4 Similarity=0.864198
0 AGACCATTCGGGTTTGGC
GCF_000724485.1_ASM72448v1_genomic.fna_1:630902-631064 Satlength=163 Nr of Repeats=9 RepeatLength=18 seed=GACCATTTGG Num.seqs=9 Similarity=0.925926
1 AGACCATTTGGTTTTGGC
Rev.of_GCF_000724485.1_ASM72448v1_genomic.fna_1:2776674-2776836 Satlength=163 Nr of Repeats=9 RepeatLength=18 seed=GCCAAAACCA Num.seqs=9 Similarity=0.946502 18
AGACCATTTGGTTTTGGC
GCF_001273755.1_ASM127375v1_genomic.fna_17:1520653-1520719 Satlength=67 Nr of Repeats=4 RepeatLength=18 seed=ATTTGGATTT Num.seqs=3 Similarity=0.901235 5
AGACCATTTGGATTTGGG

******** ** *****
Consensus:

AGACCATTtGGtTTTGGc

>Bacillus_Fam_231_15_4 Nr. of seq. 4 Alignment length(with gaps) = 15
Alignment score = 0.700000
GCF_000175075.1_ASM17507v1_genomic.fna_36:203990-204113 Satlength=124 Nr of Repeats=4 RepeatLength=15 seed=ACCAAAACAA Num.seqs=3 Similarity=0.881481
0 ACCAAAACAAGAAGC

Rev.of_GCF_000299035.1_ASM29903v1_genomic.fna_4:202303-202378
Satlength=76 Nr of Repeats=5 RepeatLength=15 seed=TTAGGCTCTT Num.seqs=5
Similarity=0.756250 6
GCCTAANAAAGAAGA
GCF_000724485.1_ASM72448v1_genomic.fna_1:1494972-1495197 Satlength=226 Nr of Repeats=15 RepeatLength=15 seed=CGAAAAAAGC Num.seqs=15
Similarity=0.925503 17
ACCGAAAAAAGCAGA
GCF_000724485.1_ASM72448v1_genomic.fna_1:1494984-1495281 Satlength=298 Nr of Repeats=20 RepeatLength=15 seed=AACCGAAAAA Num.seqs=17
Similarity=0.901961 29
ACCGAAAAAAGCAGA

** ** *** **
Consensus:

aCCgAAaaAAGaAGa

>Bacillus_Fam_232_12_4 Nr. of seq. 4 Alignment length(with gaps) = 12
Alignment score = 0.888889
GCF_000153365.1_ASM15336v1_genomic.fna_71:47849-47909 Satlength=61 Nr of Repeats=5 RepeatLength=12 seed=GGCAACGGCA Num.seqs=5 Similarity=0.866667
0 GGCAACGGCAAT
GCF_000473245.1_ASM47324v1_genomic.fna_1:3082960-3083020 Satlength=61 Nr of Repeats=5 RepeatLength=12 seed=GGCAACGGCA Num.seqs=5
Similarity=0.866667
0 GGCAACGGCAAT
Rev.of_GCF_001712755.1_ASM171275v1_genomic.fna_1:111557-111623
Satlength=67 Nr of Repeats=5 RepeatLength=12 seed=TTGCCATTAC Num.seqs=4
Similarity=1.000000 5
GGCAACGGTAAT
GCF_900156865.1_PRJEB18960_genomic.fna_3:344931-345015 Satlength=85 Nr of Repeats=7 RepeatLength=12 seed=GGCAACGGCA Num.seqs=7 Similarity=0.820106
0 GGCAACGGCAAC

******** **
Consensus:

GGCAACGGcAAt

>Bacillus_Fam_233_234_3 Nr. of seq. 3 Alignment length(with gaps) = 234
Alignment score = 0.864198
GCF_000169195.2_ASM16919v2_genomic.fna_1:908315-909719 Satlength=1405 Nr of Repeats=6 RepeatLength=234 seed=CCAAAACAAC Num.seqs=6
Similarity=0.860589 0
CCAAAACAACGACGAAGTATGTCAATGTGGACAAAGGCTCCCATTTAATATTGCGCTCNAAAGCCTCCACTTC
GTCCAGCATTTTAGCAAGCTTGGCAAGAGGAGAAAAAGTGACGGTTTACTCCATTTCCGGGGATTGGCAAAA
GTAAAAGCCGGGAGCAAAACGGGCTATGTTCATGCATCTTTTTTGGCGAACTCAAATCCCGACAGCAATGCAG
ACACTTCTACTCCGG
Rev.of_GCF_000217835.1_ASM21783v1_genomic.fna_1:416000-417405
Satlength=1406 Nr of Repeats=6 RepeatLength=234 seed=ACATTGACAT
Num.seqs=5 Similarity=0.718207
28
CCAAAACAACGACGAAGTATGTCAATGTGGATAAAGGNTCCCATTTAATATTACGTTCGAAAGCCTCCAAAAC

GTCCAGCATTTTAGCAAGCTTGGCAAGAGGNNAAAAAGTGACGGTTTACTCTATTTCCGGGGANTGGGCAAAA
GTAAAAGCCGGGAGCAAAACGGGCTATGTTCATGCATCTTTTTTGACGAACTCAAATCCCGATAGCAATGCAG
GCACTTCAACCCCAG
Rev.of_GCF_000832905.1_ASM83290v1_genomic.fna_1:2760090-2761260
Satlength=1171 Nr of Repeats=5 RepeatLength=234 seed=TTTTCGCCTC
Num.seqs=5 Similarity=0.877637
109
CCAAAACAACGACGAAGTATGTCAATGTGGATAAGGGTTCACATCTAATCTTACGTTCAAAAGCCTCCGAAAC
GTCCAGCATTTTGGACAGCTTGCCAAGAGGCGAAAAGGTGACGGTGTACTCTATTTCCGGGGACTGGGCGAAA
GTAAAAGCCGGCAGCAAAACGGGCTATGTTCATGCATCTTTTTTGGCGAATTCAAATCCTGATAGCAGTGCAG
ATACTTCTACTCCAG

****************************** ** ** ** *** **** ** ** ** *********
************ * ****** ******* **** ******** ***** *********** *****
************** ******************************** **** ******** ** ****
***** ***** ** ** *
Consensus:

CCAAAACAACGACGAAGTATGTCAATGTGGAtAAaGGnTCcCATtTAATaTTaCGtTCnAAAGCCTCCaaaaC
GTCCAGCATTTTaGcaAGCTTGgCAAGAGGngAAAAaGTGACGGTtTACTCtATTTCCGGGGAnTGGGCaAAA
GTAAAAGCCGGgAGCAAAACGGGCTATGTTCATGCATCTTTTTTGgCGAAcTCAAATCCcGAtAGCAaTGCAG
acACTTCtACtCCaG

>Bacillus_Fam_234_213_3 Nr. of seq. 3 Alignment length(with gaps) = 222
Alignment score = 0.719720
GCF_001420645.1_ASM142064v1_genomic.fna_7:2736-4618 Satlength=1883 Nr of
Repeats=10 RepeatLength=213 seed=TTAAACCTTC Num.seqs=7
Similarity=0.788528
0
TTAAACCTTCGGTCGGGTGCTGGCACGTCTCATCGNGTTTTAACGACCCTTCCTGTTGGACAGAAGCTTGAGC
TTCTTCAAAAACAAGGCAATTGGTATCAAGTTAAAGCTGGTAACCAAACTGGCTGGGTCTCCGCTGATTTCAT
TAAAACAAATGGTAATAATGTCGAAGACAA-------CAAGC--
CTTCTCTTGGCTCTGCGACGACTACAGCTCGC
Rev.of_GCF_002019765.1_ASM201976v1_genomic.fna_1:1075884-1077375
Satlength=1492 Nr of Repeats=8 RepeatLength=213 seed=CGAAGGTTTA
Num.seqs=6 Similarity=0.765152
11
TTAAACCTTCGGTCGGGTGCTGGTACGTCTCATCGNGTTTTAACGACTCTTCCGGTTGGACAGAAGCTTGAGC
TTCTTCAAAAACAAGGCAATTGGTATCAAGTTAAAGCTGGTAACCAAACTGGCTGGGTTTCTGCTGATTTCAT
TAAAACAGATGGTAATAATGTCGAAGACAA-------CAAGC--
CTTCTCTTGGCTCTGCGACGACTACAGCTCGC
GCF_002019705.1_ASM201970v1_genomic.fna_1:1608983-1610081 Satlength=1099
Nr of Repeats=5 RepeatLength=219 seed=TGGTATCAAG Num.seqs=3
Similarity=0.861999 93
TTAAACTTGCGCACTGGGGCTGGCACAAACCACCGTATTATTACAACGCTACCTGTTGGGCAAAAGCTGGAAC
TTCTTAAAAAAGAAGGCAATTGGTATCAAGTTAAAGCCGGGTCTCATACGGGATGGGTTTCTGCTGACTTTAT
TAAATCTGACGGCAAT--GGTGGAAG-
CAATGTCGGGCAATCTTCTTCTATCGGCTCTGCCACTACCACTGCTCGC

****** * ** * ** ***** ** ** ** ** * ** ** ** ** ***** ** ***** **
****** ***** *********************** ** ** ** ** ***** ** ***** **
****** * * ** *** ** **** *** *** * ***** * ******** ** ** **
******
Consensus:

TTAAACcTtCGgtCgGGtGCTGGcACgtctCAtCGngTTtTaACgACnCTtCCtGTTGGaCAgAAGCTtGAgC
TTCTTcAAAAAcAAGGCAATTGGTATCAAGTTAAAGCtGGtaacCAaACtGGcTGGGTtTCtGCTGAtTTcAT
TAAAaCagAtGGtAATaatGTcGAAGaCAACAAgCCTTCTcTtGGCTCTGCgACgACtACaGCTCGC

>Bacillus_Fam_235_166_3 Nr. of seq. 3 Alignment length(with gaps) = 168
Alignment score = 0.659392
GCF_001742425.1_ASM174242v1_genomic.fna_64:52962-53922 Satlength=961 Nr
of Repeats=5 RepeatLength=159 seed=CTTGATTCGT Num.seqs=4
Similarity=0.724668
0   CTTGA-TTC-GTTCCTATATTCCCGTCCCCAAGCTGACCATAATAGTT---
ACTTCCCCACGCATACATCGTTCCATCACTTGCTAATGCTACTGAGTGAAAGATTCCTACAGCTACTTGTTCG
ATCTTGGCTCCTTCGGGCATTGTGATCGCTGTCGGACTAG----
GCF_001742425.1_ASM174242v1_genomic.fna_64:66225-67212 Satlength=988 Nr
of Repeats=6 RepeatLength=165 seed=CCATCACTTG Num.seqs=4
Similarity=0.642857
71   AATAACTCCANATCCACTATCCCCGTCTCCCAACTGACCAAAATCGTT--
NTCNTCCCCACGTATACAACGTCCCATCACTTGCTAACGCTATTGTATGATCTCCCCCTGCTGTTACTTGTTC
GATACTGGCTCCTCCGGGGATCGAAATCGCTATCGGACTAGCTTG
GCF_001742425.1_ASM174242v1_genomic.fna_64:68080-68896 Satlength=817 Nr
of Repeats=5 RepeatLength=165 seed=CATCACTTGC Num.seqs=4
Similarity=0.742275
72   ATCAACTCCCCATCCACTATTACCGTCTCCCAGCTGACC-
ANATCGTTATTTCTCCCCCACGTATACACCGTTCCATCACTTGCTAACGCTACTGTGTGGGCAAATCCTGCCG
ATACTTGTTCGATCTTGGCTCCTCCAGGGATAGGGATCACTGTCGGACTAGATTG

* * *   *** *** ***** ** * ******   ** ***   * ******* ***** ***
************* **** ** **   *** * * *********** ******** * ** ** *
*** ** *********
Consensus:

attaAcTcCnnaTCCacTATtcCCGTCtCCcAgCTGACCaaaATcGTTntCttCCCCACGtATACAnCGTtCC
ATCACTTGCTAAcGCTAcTGtgTGancnantCCTgCnGnTACTTGTTCGATctTGGCTCCTcCgGGgATnGng
ATCgCTgTCGGACTAGnttg

>Bacillus_Fam_236_151_3 Nr. of seq. 3 Alignment length(with gaps) = 159
Alignment score = 0.705101
GCF_001636425.1_ASM163642v1_genomic.fna_1:3237226-3237826 Satlength=601
Nr of Repeats=4 RepeatLength=150 seed=GCCGATGACC Num.seqs=4
Similarity=0.986667   0
GCCGATGACCAAGGTGCTTCCTCTTTTC---
TCTGTCTAGCTCCACCGCCTATCGGCTAGCCGATTTCTCCGTCTCCTCCCTACGATAAGTCAACATCAGCTC-
---GT-CCCTCGCTGTGT-TTCCTTTATCTCAGTCGTAGATTCTGAAATCCGTAC
GCF_001636425.1_ASM163642v1_genomic.fna_1:571154-571780 Satlength=627 Nr
of Repeats=4 RepeatLength=157 seed=CTCGCTGTGT Num.seqs=3
Similarity=1.000000   110
GCCGATGAGCAAGGCGCTTGTGCTTTTCT--
TCTGTCTAGCTCCAGCGCCTATCGGCTAGCGGATTTCTACGTCTTCTCCCTGCGATAAGTCAACATCAGCTCG
TGAGTGACCTCGCTGTGTCGTCCTTTATCTCAGTCGAAGACTCTGAAATCCGTAC
GCF_001636425.1_ASM163642v1_genomic.fna_1:4465616-4467134 Satlength=1519
Nr of Repeats=10 RepeatLength=152 seed=AGCTCCAGCG Num.seqs=8
Similarity=1.000000   37
GCCGATCACGAAGCTCCTCCCGTCTTTCTGAT-

TGTCTAGCTCCAGCGCCTATCGGCTCGCGGGCTTCTCCGTCTCCTCCCTGCGATAAGGCAACATCAGCTC---
-GT-ACCTCGCTGTGT-ATCCTTGATCTCAGGCCCACCCTACGGTACCCGCAC

****** * ***   **   ****   * ************ *********** ** * ****
***** ****** ******* ************   ** *********** ***** ******* * *
* * * *** **
Consensus:

GCCGATgAccAAGgtgCTtccgctTTTCtTcTGTCTAGCTCCAgCGCCTATCGGCTaGCgGatTTCTcCGTCT
cCTCCCTgCGATAAGtCAACATCAGCTCGTaCCTCGCTGTGTnTCCTTtATCTCAGtCgnAgacTctGaaAtC
CGtAC

>Bacillus_Fam_237_144_3 Nr. of seq. 3 Alignment length(with gaps) = 148
Alignment score = 0.644895
GCF_000474275.1_Bmar1.0_genomic.fna_56:91616-92192 Satlength=577 Nr of
Repeats=4 RepeatLength=144 seed=TGGTGGGACG Num.seqs=4
Similarity=0.470569
0   -TGGTGGGACGGCTGAAGGTGGAGCAGGTGGGAAAATCTTGGCGCGCCGCAAGGNGGA-
TTGAAGGCTCNGCC--GAGCTGTCNTCATGGGCACCAAATCGTATCGACTGCTTTGTCAG-
GGAGCGTAATAATCAGATGTGGCATAAA
GCF_000005825.2_ASM582v2_genomic.fna_1:430629-431349 Satlength=721 Nr of
Repeats=5 RepeatLength=144 seed=TGTGGCATAA Num.seqs=5
Similarity=0.515711
133   -
TGGTGGGACGGNCGCAGATGGAGCGANTGGGAAAATCTNGGTTCACCGCAAGGAGGATTTGATGGCTC-
GCCAGGTGCTGTC-TCNTGGGGNCCAAATCGNATCGACTGTTTTGTCAG-
GGGAAGAAATAATCANATGTGGCATAAA
Rev.of_GCF_000175075.1_ASM17507v1_genomic.fna_4:29270-29846 Satlength=577
Nr of Repeats=4 RepeatLength=144 seed=TTGTGCCACA Num.seqs=4
Similarity=0.519371   0
ATGGTGGGATGGACAACGTTGGAGTCAGTGGGAGAATCTTGGTTCCCCACGTGGTGGCTTTGAAGGTNC-
GCCAGCTGCAGTT-TCATGGGGAGCAAATCGNATCGACTGTTTTGTAAGAGGGATG-
NACAAGCGCATGTGGCACAA-

******** **   * *****   ***** ***** ** * ** * ** ** **** ** * ***
** ** ** ****   ******* ******** ***** ** **   * * ** * ******** **
Consensus:

TGGTGGGAcGGncgaaGnTGGAGcnagTGGGAaAATCTtGGttCnCCgCaaGGnGGatTTGAaGGctCGCCag
gtGCtGTcTCaTGGGgacCAAATCGnATCGACTGtTTTGTcAGGGganGnaAtAAtCanATGTGGCAtAAa

>Bacillus_Fam_238_122_3 Nr. of seq. 3 Alignment length(with gaps) = 125
Alignment score = 0.682667
GCF_000299035.1_ASM29903v1_genomic.fna_4:356724-357203 Satlength=480 Nr
of Repeats=4 RepeatLength=122 seed=GGCTCGTTCA Num.seqs=3
Similarity=0.985428
0   GGCTCGTTCAGAATGGGAGGAGGATGGAGCT-TCTGCCGTAGAGGCGCTTTTTGCCTCGTAGGA-
AGACGCGAAGCCACCGACCGTTCTTGCCCCTGGAGCTGGATATC-TTTAAAAGCGGAATT
GCF_000831065.1_ASM83106v1_genomic.fna_1:4750262-4750754 Satlength=493 Nr
of Repeats=4 RepeatLength=123 seed=AAAAGCGAAA Num.seqs=4
Similarity=0.813333   111
GGCTCGCTCAGAATGTGAGGTGGATGGAGCT-TCTGACCTTGAGGCGCTCTTTGCCTCTGGGGA-
AGAAGCGAAGCCGCCGAACATTCTAGCCACTGTAGCTGGATTCCAATTAAAAGCGAAAGT

Rev.of_GCF_000775975.1_ASM77597v1_genomic.fna_1:4477828-4478573
Satlength=746 Nr of Repeats=6 RepeatLength=122 seed=GAGCCGCTTC
Num.seqs=4 Similarity=0.798387
5   GGCTCGCTCAGAATCGCAGGACATTGGAGCTCTC-
GACCTTGAAGCGCTTTTTGCTTCGAGCGAGAGA-
GCGAAATGACCGGAGATTCTAGCCACTGGAGCTGGATATC-ATTAAAAGCGGAAGC

****** *******   ***   ******* ** * * * ** ***** ***** **   ** ***
*****   ***   **** *** *** ******** * ********* **
Consensus:

GGCTCGcTCAGAATgggAGGaggaTGGAGCTTCtGaCcTtGAgGCGCTtTTTGCcTCgnggGAAGAnGCGAAg
ccaCCGaacaTTCTaGCCaCTGgAGCTGGATatCaTTAAAAGCGgAAgt

>Bacillus_Fam_239_104_3 Nr. of seq. 3 Alignment length(with gaps) = 104
Alignment score = 0.978632
GCF_001420645.1_ASM142064v1_genomic.fna_41:140954-141471 Satlength=518 Nr of Repeats=5 RepeatLength=103 seed=ATGCTAGAAA Num.seqs=3
Similarity=0.870550 0
ATGCTAGAAACTGATTCAAAAGCTTGAACAAGAATATCTGTAAACATAAGATCCACCTCCAGTTTGAAAATAA
GGAAAAA-AGAATTAGCCAAGAGTGCCGATA
Rev.of_GCF_000706725.1_ASM70672v1_genomic.fna_1:3741837-3742252
Satlength=416 Nr of Repeats=4 RepeatLength=104 seed=GTTTCTAGCA
Num.seqs=3 Similarity=0.829060
11
ATGCTAGAAACTGATTCAAAAGCTTGAACAAGAATATCTGTAAACATAAGATCCACCTCCAGTTTGAAAATAA
GGAAAAAGAGAATTAGCCAAGAGTGCCGATA
GCF_002019765.1_ASM201976v1_genomic.fna_1:254081-254703 Satlength=623 Nr of Repeats=6 RepeatLength=104 seed=ATGCTAGAAA Num.seqs=4
Similarity=0.952991 104
ATGCTAGAAACTGATTCAAAAGCTTGAACAAGAATATCTGTAAACATAAGATCCACCTCCAGTTTGAAAATAA
GGAAAAAGAGAATTACCCAAGAGTGCCGATA

****
******* ******* ***************
Consensus:

ATGCTAGAAACTGATTCAAAAGCTTGAACAAGAATATCTGTAAACATAAGATCCACCTCCAGTTTGAAAATAA
GGAAAAAgAGAATTAgCCAAGAGTGCCGATA

>Bacillus_Fam_240_84_3 Nr. of seq. 3 Alignment length(with gaps) = 84
Alignment score = 0.825397
GCF_001654695.1_ASM165469v1_genomic.fna_26:0-419 Satlength=420 Nr of Repeats=5 RepeatLength=84 seed=AAGAGTTTTC Num.seqs=5 Similarity=0.834921
0
AAGAGTTTTCAGAGATGCTTGAAGAAAGATCTCCCGAATGTGAAGAGTCTGAATCAATCAAAGAAGAGGATAC
TTCATTAGAAG
Rev.of_GCF_001654695.1_ASM165469v1_genomic.fna_34:68-566 Satlength=499 Nr of Repeats=6 RepeatLength=84 seed=AAAACTCTTC Num.seqs=4
Similarity=0.814815 9
AAGAGTTTTCCGAGATGCTTGAAGAAAGATCTCCTGAATGTGATGATTGTGAATTAATCAAAGAAGAAGATAC
TTCATTAGAAG
Rev.of_GCF_001654695.1_ASM165469v1_genomic.fna_34:67-985 Satlength=919 Nr of Repeats=11 RepeatLength=84 seed=GAAAACTCTT Num.seqs=7

Similarity=0.726237 10
AAGAGTTTTCAGCGATGCTTGAAGAAAGATCTCCCGAATGGGATGAGTCTGAATCAATNAANGAGGAAGTCAC
ATCACTAGAAA

********** * ******************** ***** ** ** * ***** *** ** ** ** * **
*** *****
Consensus:

AAGAGTTTTCaGaGATGCTTGAAGAAAGATCTCCcGAATGtGAtGAgTcTGAATcAATcAAaGAaGAaGatAC
tTCAtTAGAAg

>Bacillus_Fam_241_59_3 Nr. of seq. 3 Alignment length(with gaps) = 66
Alignment score = 0.611111
GCF_000508325.1_BAVI_1_genomic.fna_42:2128-2428 Satlength=301 Nr of
Repeats=4 RepeatLength=60 seed=GGCTGGGTCA Num.seqs=3 Similarity=0.733333
0 GGCTGGGT-CAAAGTGAAAGGAANA-TGGTATTACCTTAA--TNGTAAT--GGAGCTATGCAAACC
GCF_001591805.1_ASM159180v1_genomic.fna_14:1768-2128 Satlength=361 Nr of
Repeats=4 RepeatLength=60 seed=AAATGGTATT Num.seqs=3 Similarity=0.702509
21 GGCTGGCTGCAAACAG-GNGGCAAA-TGGTATTACCTCAA--TC--AATCAGGTGCGATGCAAACT
GCF_000508325.1_BAVI_1_genomic.fna_45:33463-33823 Satlength=361 Nr of
Repeats=6 RepeatLength=60 seed=AAGTGGTATT Num.seqs=4 Similarity=0.483165
22 GGCTGGGTGCAAACTG-GCGGCAAAGTGGTATTACCTTAACATC--NAGC-GGCGCGATGCAAACC

****** * **** * ** * * *********** ** * * ** ** ********
Consensus:

GGCTGGgTgCAAActGgnGGcAaATGGTATTACCTtAATcaAtcGGnGCgATGCAAACc

>Bacillus_Fam_242_66_3 Nr. of seq. 3 Alignment length(with gaps) = 66
Alignment score = 0.752525
GCF_001591805.1_ASM159180v1_genomic.fna_24:108668-109031 Satlength=364 Nr
of Repeats=5 RepeatLength=60 seed=ACTGGCTGGG Num.seqs=4
Similarity=0.479167 0
ACAGGCTGGGCAAAAGTAGGAGGTAAATGGTACTTCCTTGATGC-AA-G-CGGT---GCAATGAAA
Rev.of_GCF_000299035.1_ASM29903v1_genomic.fna_2:1035187-1035517
Satlength=331 Nr of Repeats=5 RepeatLength=66 seed=TAGTACCATT Num.seqs=5
Similarity=0.727363 35
ACAGGNTGGGNACAAGTAGATGGAAAATGGTACTACCTAGATGCAAATGATGGTGGAGCAATGAAA
Rev.of_GCF_000299035.1_ASM29903v1_genomic.fna_2:1035252-1037684
Satlength=2433 Nr of Repeats=17 RepeatLength=66 seed=GTAGTACCAT
Num.seqs=15 Similarity=0.705306
36 ACAGGCTGGGNACAAGTAGANGGNAAATGGTACTACCTAGATGGAAATGATGGTGGAGCAATGAAA

***** **** * ****** ** ********** *** **** ** * *** *********
Consensus:

ACAGGcTGGGnAcAAGTAGanGGnAAATGGTACTaCCTaGATGcaAAtGatGGTggaGCAATGAAA

>Bacillus_Fam_243_66_3 Nr. of seq. 3 Alignment length(with gaps) = 66
Alignment score = 1.000000
GCF_001887185.1_ASM188718v1_genomic.fna_65:9688-9951 Satlength=264 Nr of
Repeats=4 RepeatLength=66 seed=GGGGTCTGAC Num.seqs=3 Similarity=0.865320
0 GGGGTCTGACCCCGTTTGTTATAATCAAGAGCTACGAACCCTTGTCTTTCCTGGAATTAACGCTTG

Rev.of_GCF_001887185.1_ASM188718v1_genomic.fna_362:0-329 Satlength=330 Nr of Repeats=5 RepeatLength=66 seed=GGGGTCAGAC Num.seqs=5 Similarity=0.975758 13 GGGGTCTGACCCCGTTTGTTATAATCAAGAGCTACGAACCCTTGTCTTTCCTGGAATTAACGCTTG
Rev.of_GCF_002009555.1_ASM200955v1_genomic.fna_48:948-1343 Satlength=396 Nr of Repeats=6 RepeatLength=66 seed=GGGGTCAGAC Num.seqs=5 Similarity=0.886869 13 GGGGTCTGACCCCGTTTGTTATAATCAAGAGCTACGAACCCTTGTCTTTCCTGGAATTAACGCTTG

****
Consensus:

GGGGTCTGACCCCGTTTGTTATAATCAAGAGCTACGAACCCTTGTCTTTCCTGGAATTAACGCTTG

>Bacillus_Fam_244_55_3 Nr. of seq. 3 Alignment length(with gaps) = 58 Alignment score = 0.607280
GCF_900109925.1_IMG-taxon_2651870116_annotated_assembly_genomic.fna_16:309953-310163 Satlength=211 Nr of Repeats=4 RepeatLength=53 seed=TATTGGTGAC Num.seqs=3 Similarity=0.865828 0 TATTGGTGACCTTTC-TCCTCTCG-GGCGAC--CCCACTTAGGGCACCAA-GAGACCT
Rev.of_GCF_900109925.1_IMG-taxon_2651870116_annotated_assembly_genomic.fna_26:134607-134815 Satlength=209 Nr of Repeats=4 RepeatLength=53 seed=AGGAAGAAAG Num.seqs=3 Similarity=0.882600 20 TGTTGGTGACCTTTCTTCCTC-AG--AC-TCTTCCCACTTTGGGCACCAATGGG-CCT
GCF_900109925.1_IMG-taxon_2651870116_annotated_assembly_genomic.fna_36:66229-66446 Satlength=218 Nr of Repeats=4 RepeatLength=53 seed=ATTGGTGACC Num.seqs=3 Similarity=0.773663 54 TATTGGTGACCTTTCTTGTTCTAGCAAC-ACTT---ACTTTGGGCACCAATGAG-CCT

* ************* * ** *   * *   **** ********* * * ***
Consensus:

TaTTGGTGACCTTTCtTccTCtaGnaCaCttcccACTTtGGGCACCAAtGaGCCT

>Bacillus_Fam_245_53_3 Nr. of seq. 3 Alignment length(with gaps) = 57 Alignment score = 0.606238
GCF_000181495.1_ASM18149v1_genomic.fna_167:17100-17312 Satlength=213 Nr of Repeats=4 RepeatLength=53 seed=TGGCCCATTT Num.seqs=4 Similarity=0.865828
0 TGGCCCATTTACCACC-TTATTGGCCCAACATC-C-T-CTCATAACTTGATTCTCAT
Rev.of_GCF_000181495.1_ASM18149v1_genomic.fna_186:351607-352031 Satlength=425 Nr of Repeats=8 RepeatLength=53 seed=AGATATAGAA Num.seqs=6 Similarity=0.854088 44 TGGGCCATTTA-CACCTTTAATGGCCCAACATCTCTT-CT-ATATCTT-ATTCTCAT
GCF_000181495.1_ASM18149v1_genomic.fna_186:601490-601914 Satlength=425 Nr of Repeats=6 RepeatLength=53 seed=TGGCCCATTT Num.seqs=4 Similarity=0.616162 0 TGGCCCATTTAGCTCT-TAAATGGGCCAACATC---TACTCAAAAATGGAATCTCAA

*** ******* * * * * *** ********   * ** * * * * *****
Consensus:

TGGcCCATTTAnCaCcTtAaTGGcCCAACATCcTCTcAtAacTtgAtTCTCAt

>Bacillus_Fam_246_53_3 Nr. of seq. 3 Alignment length(with gaps) = 56
Alignment score = 0.684524
GCF_000708755.2_ASM70875v2_genomic.fna_1:523918-524339 Satlength=422 Nr of Repeats=8 RepeatLength=53 seed=AAAAGTGGCG Num.seqs=5 Similarity=0.856604
0   AAAAGTGGCGCCATTCAGNAGAGTGCTGCCACATTT-GAGGTGA--AGACCCCCGN
Rev.of_GCF_000708755.2_ASM70875v2_genomic.fna_6:246542-246807 Satlength=266 Nr of Repeats=5 RepeatLength=53 seed=GCAGAATGGC Num.seqs=5 Similarity=0.859119   17   --AAGTGGCGCCATTCTGCACAGTGGTGCCACTTTTCGA-GTGATGAGATTCTCAA
Rev.of_GCF_000709935.2_ASM70993v2_genomic.fna_15:163135-163347 Satlength=213 Nr of Repeats=4 RepeatLength=53 seed=GAATGGCGCC Num.seqs=4 Similarity=0.878407   67   --AAGTGGCGCCATTCTGCGTAGTGGTGCCACTTTTCGA-GTGAAGAGACTCTCAA

************** *   **** ****** *** ** **** *** * *
Consensus:

AAGTGGCGCCATTCtGcanAGTGgTGCCACtTTTcGAGTGAngAGActCtCaa

>Bacillus_Fam_247_50_3 Nr. of seq. 3 Alignment length(with gaps) = 55
Alignment score = 0.630303
GCF_000292245.2_ASM29224v2_genomic.fna_15:25962-26268 Satlength=307 Nr of Repeats=5 RepeatLength=51 seed=TTTTCATTAC Num.seqs=4 Similarity=0.604663
0   TTTTCATTACCTATTAACCTGTATTCGCACCGTAATTTCGCT-CTGCTTG-GA--
Rev.of_GCF_000292245.2_ASM29224v2_genomic.fna_128:7207-7563 Satlength=357 Nr of Repeats=7 RepeatLength=51 seed=GTAATGAAAA Num.seqs=6 Similarity=0.813508   10
TTTTCATTACCTATTCACCTTGATTCGAACCGTAA-TTCGAC-CAGCTCGTGC--
GCF_000292245.2_ASM29224v2_genomic.fna_83:12733-12989 Satlength=257 Nr of Repeats=5 RepeatLength=51 seed=TTTTCATTAC Num.seqs=4 Similarity=0.702306
2   TTTTCATTACCAATTAACCTTTATTCGCACTCTAA-TTCACCGCANCTCG-CATT

*********** *** **** ***** ** *** ***   * ** *
Consensus:

TTTTCATTACCtATTaACCTttATTCGcACcgTAATTCgcccCagCTcGga

>Bacillus_Fam_248_51_3 Nr. of seq. 3 Alignment length(with gaps) = 55
Alignment score = 0.612121
GCF_000175075.1_ASM17507v1_genomic.fna_2:945-1361 Satlength=417 Nr of Repeats=8 RepeatLength=52 seed=GGTAACAGAG Num.seqs=8 Similarity=0.769558
0   GGTAACAGAGA-GGGCTTCTCAAAGCCCGAATCGAGTTTGAGAATA-GCAAA-AC
Rev.of_GCF_000175075.1_ASM17507v1_genomic.fna_29:154879-155190 Satlength=312 Nr of Repeats=6 RepeatLength=52 seed=TCTCTCTGTT Num.seqs=5 Similarity=0.851572   13
GGTAACAGAGAGA-GCGTCTCAAGGCCCGAAGCAAG-AAGCGAAGCGTCTAA-AC
GCF_000175075.1_ASM17507v1_genomic.fna_35:6050-6310 Satlength=261 Nr of Repeats=5 RepeatLength=52 seed=AACAGAGAAG Num.seqs=5 Similarity=0.943590
56   GGTAACAGAGA-AGGCGTCTCAAGGCCCGAAGCAAG-CAACGAAAC-CCGAAGAC

*********** ** ****** ******* * ** *** * ** **
Consensus:

GGTAACAGAGAagGCgTCTCAAgGCCCGAAgCaAGnagcGAAncnCnAAAC

>Bacillus_Fam_249_53_3 Nr. of seq. 3 Alignment length(with gaps) = 55
Alignment score = 0.616162
GCF_000175075.1_ASM17507v1_genomic.fna_32:472596-472856 Satlength=261 Nr of Repeats=5 RepeatLength=52 seed=AAACGGTAAC Num.seqs=5
Similarity=0.775309
0 AAACGGTAACAGAGAGGCCGTCTCAAAGCCCGAAACTCCTC-NGAACTCCGCC--
Rev.of_GCF_000175075.1_ASM17507v1_genomic.fna_44:72026-72234
Satlength=209 Nr of Repeats=4 RepeatLength=52 seed=TCTCTGTTAC Num.seqs=4
Similarity=0.868973 15
GAACGGTAACAGAGAGGCGGTCTCAGAGCCCGAAGTTGCTC-AG--CTGCGCCAA
GCF_000175075.1_ASM17507v1_genomic.fna_36:83736-83947 Satlength=212 Nr of Repeats=4 RepeatLength=53 seed=AGAGAAGCCG Num.seqs=3 Similarity=0.899371
10 AAACGGTAACAGAGAAGCCGTCTCAACGCCGGAAGCAGCTCGCCAAAAAC-CCA-

************* ** ****** *** *** *** * **
Consensus:

aAACGGTAACAGAGAgGCcGTCTCAaaGCCcGAAgctgCTCngaactnCgCCa

>Bacillus_Fam_250_51_3 Nr. of seq. 3 Alignment length(with gaps) = 55
Alignment score = 0.620202
GCF_000181495.1_ASM18149v1_genomic.fna_100:912-1381 Satlength=470 Nr of Repeats=9 RepeatLength=52 seed=GTTTCTTCTT Num.seqs=8 Similarity=0.828704
0 GTTTCTTCTT-GAATCTTACCATTATAAA-AC-GGTTTCCCTGCTGTAGGGGTAC
Rev.of_GCF_000181495.1_ASM18149v1_genomic.fna_175:57618-58242
Satlength=625 Nr of Repeats=12 RepeatLength=52 seed=GGTACGATTA
Num.seqs=12 Similarity=0.799145
20 GTTTC-TCTTCTAATCGTACCATTATAAACTC-GAATTCCCT-CTGTAGTGGTAC
GCF_000181495.1_ASM18149v1_genomic.fna_149:10584-10950 Satlength=367 Nr of Repeats=7 RepeatLength=52 seed=ATCTTACCAT Num.seqs=5
Similarity=0.864103
12 GTTTA-TCGAGGAATCTTACCATTATAAC-TCTAGTTTCCCT-TTGGAATGGTAC

**** ** **** *********** * ****** ** * *****
Consensus:

GTTTcTCttngAATCtTACCATTATAAatCggtTTCCCTcTGtAgtGGTAC

>Bacillus_Fam_251_52_3 Nr. of seq. 3 Alignment length(with gaps) = 55
Alignment score = 0.634343
GCF_000292245.2_ASM29224v2_genomic.fna_15:39283-39543 Satlength=261 Nr of Repeats=4 RepeatLength=52 seed=TACCACTTTT Num.seqs=3 Similarity=0.914530
0 TACCACTTT--TCACTTCACT-CTTCCTCTTTTTTTGTTTCCATCTCCCTTTGGA
GCF_000292245.2_ASM29224v2_genomic.fna_107:33-293 Satlength=261 Nr of Repeats=5 RepeatLength=52 seed=CCTTTTCTTG Num.seqs=5 Similarity=0.861635
24 TACCTCTTTCCTCCA--CGCTCCTTCC-CTTTTCTTGTCTCCATCCTTGTTTGGT

GCF_000292245.2_ASM29224v2_genomic.fna_136:43-303 Satlength=261 Nr of Repeats=5 RepeatLength=52 seed=ACCTCTTTTC Num.seqs=5 Similarity=0.835897
53 TACCTCTTT--TCCATTCTCTCCTTCC-CATTTATTGTCTCCATCTCCATTTGGT

**** **** ** * ** ***** * *** **** ****** *****
Consensus:

TACCtCTTTTCcattCnCTcCTTCCCtTTTnTTGTcTCCATCtccnTTTGGt

>Bacillus_Fam_252_53_3 Nr. of seq. 3 Alignment length(with gaps) = 55
Alignment score = 0.638384
GCF_000374345.1_ASM37434v1_genomic.fna_3:96044-96563 Satlength=520 Nr of Repeats=10 RepeatLength=52 seed=ATGAAAGCCA Num.seqs=9 Similarity=0.598990
0 ATGAAAGCCANTTCGG--TGAAATTTTTGGNTCGAAATGTTGTTCATAAG-CCGN
GCF_000374345.1_ASM37434v1_genomic.fna_34:20589-20901 Satlength=313 Nr of Repeats=6 RepeatLength=52 seed=GATGAAGGAC Num.seqs=6 Similarity=0.658990
51 ATGAAGGAC-ATTAGGCATGAAATTTTTGG-TGGAAATGACGTTCATAAGCCCG-
GCF_001439915.1_ASM143991v1_genomic.fna_84:73765-74544 Satlength=780 Nr of Repeats=14 RepeatLength=52 seed=GGTCGAAATG Num.seqs=10 Similarity=0.794410 79
ATGAAGGACAAATAGGATTGAAA-TTGAGG-TCGAAATGGTCTTCATAAGCCCG-

***** * * * ** ***** ** ** * ****** ******** ***
Consensus:

ATGAAgGaCaatTaGGnnTGAAAtTTttGGTcGAAATGntgTTCATAAGcCCG

>Bacillus_Fam_253_52_3 Nr. of seq. 3 Alignment length(with gaps) = 55
Alignment score = 0.731313
GCF_000708755.2_ASM70875v2_genomic.fna_7:94412-94828 Satlength=417 Nr of Repeats=8 RepeatLength=52 seed=CCACCAAACT Num.seqs=8 Similarity=0.870879
0 CCACCAAACTTCTTCCTCACGCGC-TTGATGGTTCTTATAACCG-TT-TTCAGTA
GCF_000709935.2_ASM70993v2_genomic.fna_19:101683-101995 Satlength=313 Nr of Repeats=6 RepeatLength=52 seed=CCACCAAACT Num.seqs=6 Similarity=0.844444
0 CCACCAAACTTCTTCCTCACGCGC-TTGATGGTTCTTATAACCG-TT-TTCAGTA
GCF_000708755.2_ASM70875v2_genomic.fna_7:153488-153800 Satlength=313 Nr of Repeats=6 RepeatLength=52 seed=CTTATCCGCT Num.seqs=6 Similarity=0.858281
33 CCACCAAACTTCCAATTC-CGTTCTTTGATGGTTCTTAT--CCGCTTGTTGAGTA

************ ** ** * ************** *** ** ** ****
Consensus:

CCACCAAACTTCttccTCaCGcgCTTGATGGTTCTTATaaCCGTTTTcAGTA

>Bacillus_Fam_254_53_3 Nr. of seq. 3 Alignment length(with gaps) = 55
Alignment score = 0.628283
GCF_000787375.1_ASM78737v1_genomic.fna_7:75363-75623 Satlength=261 Nr of Repeats=5 RepeatLength=52 seed=TTGGCTCCCG Num.seqs=5 Similarity=0.824691
0 TTGGCTCCCGTTCT-TTTGCTTTCTCTCTCAG--TTCGGGAGCCATCACACCTCT
GCF_000787375.1_ASM78737v1_genomic.fna_23:22127-22647 Satlength=521 Nr of Repeats=10 RepeatLength=52 seed=CTCTTTGGCT Num.seqs=8

Similarity=0.848720
48   TTGGCTACCGTTCTCTTCGCGAT-TC-CTCGGT-TTCGGGATCCATCACACCTCT
Rev.of_GCF_000787375.1_ASM78737v1_genomic.fna_12:89119-89327
Satlength=209 Nr of Repeats=4 RepeatLength=52 seed=CGGGGGAGAG Num.seqs=4
Similarity=0.854701   28
TTGGCTACCGTTCT-TACGC--TCTCCCCGCTTTTCGGGCTCCAACCTACCTCT

****** ******* * ** * ** * *   ****** *** * ******
Consensus:

TTGGCTaCCGTTCTTtcGCnnTcTCnCtCggtTTCGGGatCCAtCacACCTCT

>Bacillus_Fam_255_52_3 Nr. of seq. 3 Alignment length(with gaps) = 55
Alignment score = 0.660606
GCF_000813125.1_ASM81312v1_genomic.fna_40:47668-47876 Satlength=209 Nr of
Repeats=4 RepeatLength=52 seed=TTAGACATTT Num.seqs=4 Similarity=0.726496
39   CAGCT-TACCAGCCT-TGAAATGTCCATTAAACACCTCTT-TTTAGACATTTCAT
Rev.of_GCF_000813125.1_ASM81312v1_genomic.fna_138:81898-82158
Satlength=261 Nr of Repeats=5 RepeatLength=52 seed=GTCTAATAAA Num.seqs=5
Similarity=0.828205   44   -
AGCTACAACAGTTTCTGAAATGTCTATTAAACACCTTTTAT-TAGACATTTCA-
Rev.of_GCF_001315165.1_ASM131516v1_genomic.fna_21:7080-7288 Satlength=209
Nr of Repeats=4 RepeatLength=52 seed=AAATGTCTAA Num.seqs=4
Similarity=0.829060   48
-ATCCCCATCAGCTTCTGAAATGTCTATTAAATCCCTTTTAT-TAGACATTTTA-

********* * * *   * *** * ********* ****** *** ** *
Consensus:

TAGACATTTcAAgCtncAnCAGctTcTGAAATGTCtATTAAAcaCCTtTTaT

>Bacillus_Fam_256_52_3 Nr. of seq. 3 Alignment length(with gaps) = 55
Alignment score = 0.668687
GCF_001636315.1_ASM163631v1_genomic.fna_1:379346-379554 Satlength=209 Nr
of Repeats=4 RepeatLength=52 seed=CCTCTATGTG Num.seqs=4
Similarity=0.816239
0   CCTCTATGTGCACTT-AACAAGC-TTTTTCAATCGATTTCGTGTTCATTG-AGAC
GCF_001636315.1_ASM163631v1_genomic.fna_1:2299724-2300141 Satlength=418
Nr of Repeats=8 RepeatLength=52 seed=GGTTTCGTGT Num.seqs=7
Similarity=0.839173   31
-CTCTATGTGCACTT-TCCTCGC-TTTTTTCATTGGTTTCGTGTTCATTGAAGAC
Rev.of_GCF_001636315.1_ASM163631v1_genomic.fna_1:4170771-4171031
Satlength=261 Nr of Repeats=5 RepeatLength=52 seed=AAGAAGTGTC Num.seqs=5
Similarity=0.871795   17   -
CTCTATGGACACTTCTTC-CGCTTTTTTTCA-CGATTTCGTGTTCATTGAGGAC

******* *****   * ** ***** * * ************* ***
Consensus:

CTCTATGtgCACTTtnCncGCTTTTTtcAtcGaTTTCGTGTTCATTGaaGAC

>Bacillus_Fam_257_52_3 Nr. of seq. 3 Alignment length(with gaps) = 55
Alignment score = 0.634343

GCF_001636315.1_ASM163631v1_genomic.fna_1:2912216-2912475 Satlength=260 Nr of Repeats=5 RepeatLength=52 seed=TGCATTGCCC Num.seqs=4 Similarity=0.841880 0
TGCATTGCCCAAAAAACGG-GA--AGAGCCACTAATTTGGTCAAAGGAAGAGGGA
GCF_001636315.1_ASM163631v1_genomic.fna_1:3674698-3674906 Satlength=209 Nr of Repeats=4 RepeatLength=52 seed=AAACGAGGTG Num.seqs=4 Similarity=0.867521 11
TGCATTGCCCAAAACGAGGTGA-TACATCAA--AATTTGGTCAATGGAAGAGGGG
GCF_001636315.1_ASM163631v1_genomic.fna_1:4951452-4952180 Satlength=729 Nr of Repeats=14 RepeatLength=52 seed=ATTGCCCAAA Num.seqs=12 Similarity=0.924631 55
TGCATTGCCCAAAAATCGGTGATTAGATGGA--AA-TTGGTCAATGGACGGGGCT

************** ** ** * * * ** ******** *** * **
Consensus:

TGCATTGCCCAAAAancGGtGAtAgAtcnAAAtTTGGTCAAtGGAaGaGGgn

>Bacillus_Fam_258_51_3 Nr. of seq. 3 Alignment length(with gaps) = 55
Alignment score = 0.612121
GCF_001636325.1_ASM163632v1_genomic.fna_1:443016-443432 Satlength=417 Nr of Repeats=8 RepeatLength=52 seed=GTTTATTCCT Num.seqs=8 Similarity=0.880952
0 GTTTATTCCTCTAAAGCTTCAAAATATG--A-ATGCCTTCAGGTGAATAAATGGG
Rev.of_GCF_001636325.1_ASM163632v1_genomic.fna_1:2689693-2690109 Satlength=417 Nr of Repeats=8 RepeatLength=52 seed=AGAGGAATAA Num.seqs=8 Similarity=0.879409 12
ATTTATTCCTCTGAAGCTGTAGAAGTCGTCA-A-G-CTTCAGGTGAAGAAACGAG
GCF_001636325.1_ASM163632v1_genomic.fna_3:480673-481035 Satlength=363 Nr of Repeats=7 RepeatLength=52 seed=CTAAAGCAGC Num.seqs=6 Similarity=0.924786
62 ATTTCTTCCTCTAAAGCAGCAAAATTCG-CAGA-G-CTTGAGGTGAATAACAGAG

*** ******* **** * ** * * * * *** ******* ** * *
Consensus:

aTTTaTTCCTCTaAAGCtgcAaAAttcGcAAGCTTcAGGTGAAtAAanGaG

>Bacillus_Fam_259_52_3 Nr. of seq. 3 Alignment length(with gaps) = 55
Alignment score = 0.723232
GCF_001887185.1_ASM188718v1_genomic.fna_350:150-462 Satlength=313 Nr of Repeats=6 RepeatLength=52 seed=AATGTGTCTC Num.seqs=6 Similarity=0.854927
0 AATGTGTCTCAAGACTGGATCTTGAGACAGATAAAGAGG---GAGAAAGGAGAAA
Rev.of_GCF_002009555.1_ASM200955v1_genomic.fna_24:43390-43598 Satlength=209 Nr of Repeats=4 RepeatLength=52 seed=TGAGACACAT Num.seqs=4 Similarity=0.864256 11
AATGTGTCTCAAGACTGGATCTTGAGACAGATAAAGAGG---GGGAAAGGAGAAA
GCF_002009555.1_ASM200955v1_genomic.fna_548:51305-51513 Satlength=209 Nr of Repeats=4 RepeatLength=52 seed=AATGTGTCTC Num.seqs=4 Similarity=0.656379
0 AATGTGTCTCAAGACCAGTGCTTGAGACACATAAA-AGGACTGAG-AACTA-AAA

*************** * ********* ***** *** * * ** * ***
Consensus:

AATGTGTCTCAAGACtgGatCTTGAGACAgATAAAgAGGGaGaAAggAgAAA

>Bacillus_Fam_260_52_3 Nr. of seq. 3 Alignment length(with gaps) = 55
Alignment score = 0.717172
GCF_900156875.1_PRJEB18969_genomic.fna_11:1019409-1020084 Satlength=676
Nr of Repeats=13 RepeatLength=52 seed=ATGAAGACCA Num.seqs=12
Similarity=0.841103 0
ATGAAGACCA-ATTAGCTTCTCGGAAACTA-GAAAAACGGTCTTCATCTCCGGC-
GCF_900156875.1_PRJEB18969_genomic.fna_11:1203981-1207193 Satlength=3213
Nr of Repeats=62 RepeatLength=52 seed=AAACGGTCTT Num.seqs=50
Similarity=0.899766 32
ATGAAAACCATTTTCTCTTC-CGG-AACAAGGAGAAACGGTCTTCAT-GACGGCG
GCF_900156875.1_PRJEB18969_genomic.fna_11:1208970-1209385 Satlength=416
Nr of Repeats=8 RepeatLength=52 seed=AAACGGTCTT Num.seqs=7
Similarity=0.818808 32
ATGAAAACCATTTTCTCTTC-CGG-AACAAGGAGAAACGGTCTTCAT-GACGGCG

***** **** ** **** *** *** * ** ************* ****
Consensus:

ATGAAaACCAttTTctCTTCCGGAACaAgGAgAAACGGTCTTCATgaCGGCg

>Bacillus_Fam_261_53_3 Nr. of seq. 3 Alignment length(with gaps) = 55
Alignment score = 0.640404
GCF_001439915.1_ASM143991v1_genomic.fna_83:206289-206524 Satlength=236 Nr of Repeats=5 RepeatLength=53 seed=ACCGCAAGAA Num.seqs=3
Similarity=0.983229 0
ACCGCAAGAAGTGTTTAACGGTACATGCTCAGTCCAAAAAGCAGA--AAGCATGT
GCF_001439915.1_ASM143991v1_genomic.fna_116:178064-178329 Satlength=266
Nr of Repeats=5 RepeatLength=53 seed=TGTACCGCAA Num.seqs=5
Similarity=0.816049 50
ACCGCAAGAGAGGTTTCGTGGTACATGCTCAAT-GAAAAA-AAGCTCAAGCGTGT
Rev.of_GCF_001439915.1_ASM143991v1_genomic.fna_122:86523-87001
Satlength=479 Nr of Repeats=9 RepeatLength=53 seed=TGAGCTTGTA Num.seqs=8
Similarity=0.893082 84
ACCGCAAGAACGGTTTCACGGTACAAGCTCACG-CAAAAA-TAGACCTAGCATGT

********* **** ****** ***** ***** ** *** ***
Consensus:

ACCGCAAGAangGTTTcacGGTACAtGCTCAntcAAAAAnAGancaAGCaTGT

>Bacillus_Fam_262_53_3 Nr. of seq. 3 Alignment length(with gaps) = 55
Alignment score = 0.696970
GCF_002019605.1_ASM201960v1_genomic.fna_1:3456401-3456877 Satlength=477
Nr of Repeats=9 RepeatLength=53 seed=CGGGCACTAT Num.seqs=8
Similarity=0.853549 0
CGGGCACTATGAACCCTTTATAGTTACCGCCACCACTTCTTCT-A-TCCCTCCCA
Rev.of_GCF_002019605.1_ASM201960v1_genomic.fna_1:3950268-3950692
Satlength=425 Nr of Repeats=8 RepeatLength=53 seed=CGGTCACTAT Num.seqs=8
Similarity=0.858940 29
CGGGCACTATGAACTCTCTATAGTGACCG-C-GAACTACTTGTGACTCCCTCCTG

Rev.of_GCF_002019605.1_ASM201960v1_genomic.fna_1:4323665-4324034 Satlength=370 Nr of Repeats=7 RepeatLength=53 seed=CGGTCACTAT Num.seqs=5 Similarity=0.768553 29 CGGGCACTATGAACTCTCTATAGTGACCG-C-TCTCTTCTTTTGACTTCCTCCTG

************** ** ****** **** * ** *** * * * *****
Consensus:

CGGGCACTATGAACtCTcTATAGTgACCGCncaCTtCTTnTgAcTcCCTCCtg

>Bacillus_Fam_263_52_3 Nr. of seq. 3 Alignment length(with gaps) = 54
Alignment score = 0.633745
GCF_000175075.1_ASM17507v1_genomic.fna_32:169519-169779 Satlength=261 Nr of Repeats=5 RepeatLength=52 seed=GTCAAATAGA Num.seqs=5 Similarity=0.864103
0 GTCAAATAGAA-CGGCTCTATACGCCCGAACGC-CCGTTAAAAGAGGAAAACG
GCF_000175075.1_ASM17507v1_genomic.fna_48:8867-9075 Satlength=209 Nr of Repeats=4 RepeatLength=52 seed=GCTTCTATAC Num.seqs=4 Similarity=0.877358
12 GTCAAATAGAA-TGCTTCTATACGCCCGAAGGC-ACGAGGAAAAGCGGTAAACG
GCF_000175075.1_ASM17507v1_genomic.fna_32:398955-399215 Satlength=261 Nr of Repeats=5 RepeatLength=52 seed=CCAAAGGTCA Num.seqs=5 Similarity=0.943590
46 GTCAAATAGAAGCGG-TCTATATGCCCGAA-GCGCCGTGAAAGAAAGTCCAAAG

*********** * ****** ******* ** ** ** * * ** *
Consensus:

GTCAAATAGAAcGgnTCTATAcGCCCGAAnGCcCGtgaAAaAgaGgnaAAcG

>Bacillus_Fam_264_52_3 Nr. of seq. 3 Alignment length(with gaps) = 54
Alignment score = 0.641975
GCF_000181495.1_ASM18149v1_genomic.fna_105:10535-10847 Satlength=313 Nr of Repeats=6 RepeatLength=52 seed=CTCATGACAC Num.seqs=6 Similarity=0.839316
0 CTCATGACACTTTT-CTCTCTCCTTTTTGCCTTTCATGCCCTTGAGACGCTTC-
GCF_000181495.1_ASM18149v1_genomic.fna_178:76540-76957 Satlength=418 Nr of Repeats=8 RepeatLength=52 seed=CTCTCTTTTT Num.seqs=7 Similarity=0.867026
16 CTCACGACACTTTT-CCCTCTCTTTTTCTTNTTTCATGTCCTTGAGAAACTTC-
Rev.of_GCF_000181495.1_ASM18149v1_genomic.fna_146:13461-13667 Satlength=207 Nr of Repeats=4 RepeatLength=52 seed=TCAAGGACAT Num.seqs=3 Similarity=0.636364 44 TTCAAGACACTTTTGCTTTCT-ATTTTGGANTTTGATGTCCTTGAGA-ACTCCC

*** ********* * *** **** *** *** ******** ** *
Consensus:

cTCAnGACACTTTTCtcTCTcnTTTTngnnTTTcATGtCCTTGAGAnaCTtC

>Bacillus_Fam_265_52_3 Nr. of seq. 3 Alignment length(with gaps) = 54
Alignment score = 0.637860
GCF_000186145.1_Bacillus_sp_2_A_57_CT2_V1_genomic.fna_2:775846-776164 Satlength=319 Nr of Repeats=6 RepeatLength=52 seed=ACTTCGGACT Num.seqs=5

Similarity=0.675472 0
ACTTCGGACTCACTTCCTCCA-ATTTCCTCTCTTTCTGTCTGAA-GNTGCACCT
Rev.of_GCF_000186145.1_Bacillus_sp_2_A_57_CT2_V1_genomic.fna_5:390749-391008 Satlength=260 Nr of Repeats=5 RepeatLength=52 seed=GAGTCCGAAG Num.seqs=4 Similarity=0.671908 11
ACTTCGGACTCG-TTCCTCTGGATATCCGCTTTTCCTGTCCGAATGGTG-ACCG
GCF_000294775.2_ASM29477v2_genomic.fna_1:2523541-2523749 Satlength=209 Nr of Repeats=4 RepeatLength=52 seed=CTGTCCGAAT Num.seqs=4 Similarity=0.676101 34
ACTTCGGACTCA-CACCTCCAGATTACCCCTTCTTCTGTCCGAATGATG-ACCA

*********** **** ** ** ** * ***** *** * ** ***
Consensus:

ACTTCGGACTCattCCTCcagATttCCnCTttTtCTGTCcGAAtGnTGACCn

>Bacillus_Fam_266_52_3 Nr. of seq. 3 Alignment length(with gaps) = 54
Alignment score = 0.699588
GCF_000311725.1_ASM31172v1_genomic.fna_3:146549-147069 Satlength=521 Nr of Repeats=10 RepeatLength=52 seed=CGGATTCGGA Num.seqs=10 Similarity=0.860867 0
CGGATTCGGACAA-CATCACCGAT-TCACCCTGCTTCCCTGTCCGAATATCAAG
GCF_000311725.1_ASM31172v1_genomic.fna_3:585727-586091 Satlength=365 Nr of Repeats=7 RepeatLength=52 seed=GATTCGGACA Num.seqs=7 Similarity=0.897436
2 CGGATTCGGACAA-CATTGCCGGT-TCATCCTGCTTCTCTGTCCGAATATCAAG
GCF_000311725.1_ASM31172v1_genomic.fna_6:313304-313604 Satlength=301 Nr of Repeats=6 RepeatLength=52 seed=TGTCCGAATA Num.seqs=5 Similarity=0.836478
37 CGGATTCGGACAATCCCTACTACTCTC-TCCT-CTTTCCTGTCCGAATATGAAG

************* * * * ** *** *** ************ ***
Consensus:

CGGATTCGGACAACattaCcgnTTCatCCTgCTTccCTGTCCGAATATcAAG

>Bacillus_Fam_267_53_3 Nr. of seq. 3 Alignment length(with gaps) = 54
Alignment score = 0.627572
GCF_000430765.1_ASM43076v1_genomic.fna_10:72408-72928 Satlength=521 Nr of Repeats=10 RepeatLength=52 seed=CTAAAGGACA Num.seqs=10 Similarity=0.738721
0 CTAAAGGACAAAACTCGGATGGA-GCATCAAAGATAAGTCCATGAGAAG-GGTC
Rev.of_GCF_000430765.1_ASM43076v1_genomic.fna_14:23161-23577 Satlength=417 Nr of Repeats=8 RepeatLength=52 seed=TTGTCCTTTA Num.seqs=8 Similarity=0.826923 10
-TAAAGGACAAAACTCAGTCGCC-CCAGCAAAGATCTGTCCATGAGAAGCGGTC
GCF_000430765.1_ASM43076v1_genomic.fna_13:190-660 Satlength=471 Nr of Repeats=9 RepeatLength=52 seed=TAAAGGACAA Num.seqs=7 Similarity=0.639250
1 CTAAAGGACAAAAC-AGGATCAAGGCAGCAAAGATNNGTCCATGATAGGCATT-

************* * ** ******* ******** * * *
Consensus:

cTAAAGGACAAAACtcgGatgnagCAgCAAAGATnnGTCCATGAgAaGcggTc

>Bacillus_Fam_268_52_3 Nr. of seq. 3 Alignment length(with gaps) = 54
Alignment score = 0.633745
GCF_000508325.1_BAVI_1_genomic.fna_31:7329-7537 Satlength=209 Nr of Repeats=4 RepeatLength=52 seed=AGTGCCCGTG Num.seqs=4 Similarity=0.880342
0   AGTGCCCGTGCGCATGGAAAAACGCCAA--AGACGGGCACTCTGAGAGGTTCAC
Rev.of_GCF_001591805.1_ASM159180v1_genomic.fna_25:13492-13700 Satlength=209 Nr of Repeats=4 RepeatLength=52 seed=GGGCACTGTG Num.seqs=4 Similarity=0.854701   7
AGTGCCCATGACCATGGGAAAAAGCCAA--GAATGGGCACTCTGAGAGGTCCAC
GCF_001591665.1_ASM159166v1_genomic.fna_53:45335-45543 Satlength=209 Nr of Repeats=4 RepeatLength=52 seed=GGGCACTCTG Num.seqs=4 Similarity=0.820513
32   AGTGCCCAAACGC--GCGAAAAATCGAAGTGCACGGGCACTCTGAGCGGTTGAC

```
*******     *  *  ****  * **     * ************ ***  **
```

Consensus:

AGTGCCCatgcgCatGggAAAAagCcAAgnAcGGGCACTCTGAGaGGTtcAC

>Bacillus_Fam_269_52_3 Nr. of seq. 3 Alignment length(with gaps) = 54
Alignment score = 0.625514
GCF_000708755.2_ASM70875v2_genomic.fna_2:68427-68791 Satlength=365 Nr of Repeats=7 RepeatLength=52 seed=AGTTTTGAGG Num.seqs=7 Similarity=0.893773
0   AGTTTTGAGGGTGCTTATCTGTCTTATAAGTGCCCTCAATTTTTGAT--TTTCA
Rev.of_GCF_000708755.2_ASM70875v2_genomic.fna_22:217896-218468 Satlength=573 Nr of Repeats=10 RepeatLength=52 seed=TTGAGGGCAC Num.seqs=9 Similarity=0.781207
39   ATTTTTGAGGGTACTATTCTGCGGTTTTAGTGCCCTCAA--TTTCCTACTTTCG
Rev.of_GCF_000709935.2_ASM70993v2_genomic.fna_5:41766-41974 Satlength=209 Nr of Repeats=4 RepeatLength=52 seed=AAAAATTGAG Num.seqs=4 Similarity=0.846154   44
GTTTTTGAGGGTACGATTCTGGCTTTTTAGTACCCTCAATTTTTGGT--TTTTG

```
********** *   ****   * * *** *******  ***  *  ***
```

Consensus:

atTTTTGAGGGTaCtatTCTGnctTtTtAGTgCCCTCAAttTTTgnTTTTcg

>Bacillus_Fam_270_52_3 Nr. of seq. 3 Alignment length(with gaps) = 54
Alignment score = 0.703704
GCF_000708755.2_ASM70875v2_genomic.fna_22:181704-182016 Satlength=313 Nr of Repeats=6 RepeatLength=52 seed=GTGGAAATGA Num.seqs=6 Similarity=0.854701
0   GTGGAAATGACCCTTCAG-CAAGATCAAATCTGTGCGAAGGTCACAAAGCCGC-
GCF_000709935.2_ASM70993v2_genomic.fna_24:292765-293078 Satlength=314 Nr of Repeats=6 RepeatLength=52 seed=TGGAAATGAC Num.seqs=5 Similarity=0.846154
1   GTGGAAATGACCCTTCAG-CATGATCAAATCTGTGCGAAGGTCACAAAGGCGC-
GCF_000709935.2_ASM70993v2_genomic.fna_1:11462-11774 Satlength=313 Nr of Repeats=6 RepeatLength=52 seed=TGTCCCTTCA Num.seqs=6 Similarity=0.883761
6   -AGGAAATGTCCCTTCAGCCACGCTTGAA-CAGCGTGAAGGTCACAAAAGCGGA

```
******* ******** ** * *  ** * * * ************  **
```

Consensus:

gtGGAAATGaCCCTTCAGCAnGaTcaAAtCtGtGcGAAGGTCACAAAggCGc

>Bacillus_Fam_271_53_3 Nr. of seq. 3 Alignment length(with gaps) = 54
Alignment score = 0.600823
GCF_000934845.1_ASM93484v1_genomic.fna_162:31645-32113 Satlength=469 Nr of Repeats=9 RepeatLength=52 seed=CGGGCACATG Num.seqs=9
Similarity=0.904558
0 CGGGCACATGTTGGCC-TTTCTTGTGACCGTGCTCTCGCTTTTTCTTCCTTTT-
GCF_001420595.1_ASM142059v1_genomic.fna_11:2811819-2812081 Satlength=263 Nr of Repeats=5 RepeatLength=52 seed=CTCTTGTTAC Num.seqs=3
Similarity=0.863248 17
CGGGCACATGTGCGCC-CCTCTTGTTACCGCTCTCTTGCTTTTTC-TGCTTCCA
GCF_001420595.1_ASM142059v1_genomic.fna_11:4482129-4482596 Satlength=468 Nr of Repeats=9 RepeatLength=52 seed=AGTTTCTGGC Num.seqs=8
Similarity=0.868132 37
CGGGCACATGTT-GCCTTCTCTTGTTACCATGC-CTTCGAGTTTCTGGCTTTCG

*********** *** ****** *** * ** **** ***
Consensus:

CGGGCACATGTtnGCCtcTCTTGTtACCgtgCtCTtgcttTTTCttgCTTtcn

>Bacillus_Fam_272_52_3 Nr. of seq. 3 Alignment length(with gaps) = 54
Alignment score = 0.633745
GCF_001591645.1_ASM159164v1_genomic.fna_2:312008-312320 Satlength=313 Nr of Repeats=6 RepeatLength=52 seed=CTCTCTTACC Num.seqs=6
Similarity=0.744235
0 CTCTCTTACCATCCAACTCTGCTTCCATCACG-AATGGGCACTGA-CAACCACT
Rev.of_GCF_001591645.1_ASM159164v1_genomic.fna_19:50399-50607 Satlength=209 Nr of Repeats=4 RepeatLength=52 seed=TGGATGGTAA Num.seqs=4
Similarity=0.839623 15
CTCTCTTACCATCC-ACCCTCATTTCCTCCCGCAATGGGCACTGA-CAACTTCT
GCF_001591645.1_ASM159164v1_genomic.fna_13:8919-9439 Satlength=521 Nr of Repeats=10 RepeatLength=52 seed=TCTCTCTTAC Num.seqs=10
Similarity=0.780057
51 CTCTCTTACCATCGTACCCCCGTTTACTCTCC-AACGGGCACTGAGCGAC-GCT

************* ** * ** ** * ** ********* * ** **
Consensus:

CTCTCTTACCATCcnACcCtcnTTtccTCnCgAAtGGGCACTGACaACnnCT

>Bacillus_Fam_273_53_3 Nr. of seq. 3 Alignment length(with gaps) = 54
Alignment score = 0.651235
GCF_001591665.1_ASM159166v1_genomic.fna_16:63845-64053 Satlength=209 Nr of Repeats=4 RepeatLength=52 seed=TCGGTAAAAG Num.seqs=4
Similarity=0.864256
0 TCGGTAAAAGGAAGAGTGCTCCATTTACCGAAAAGCAG-AAAAGAGAGCTCTC-
Rev.of_GCF_001591665.1_ASM159166v1_genomic.fna_72:4462-4722 Satlength=261 Nr of Repeats=5 RepeatLength=52 seed=CTTTCATTTA Num.seqs=5
Similarity=0.907692 14
ACGGTAAATGAAAGAGCGGTTCATTTACCGAAAAG-GGAAAAAAAGAGCACTT-

Rev.of_GCF_001591665.1_ASM159166v1_genomic.fna_90:5864-6072 Satlength=209 Nr of Repeats=4 RepeatLength=52 seed=TTCATTTACC Num.seqs=4 Similarity=0.794872 64
ACGGTAAATGAAGGACCGTTTCATTGACCGAAAAGAGGAAAAATA-AGC-CTTC

******* * * ** * * **** ********* * **** * *** **
Consensus:

aCGGTAAAtGaAaGAgcGnTtCATTtACCGAAAAGngGaAAAAnAgAGCnCTt

>Bacillus_Fam_274_52_3 Nr. of seq. 3 Alignment length(with gaps) = 54
Alignment score = 0.720165
GCF_001591665.1_ASM159166v1_genomic.fna_19:47666-47978 Satlength=313 Nr of Repeats=6 RepeatLength=52 seed=CACAGTGCCC Num.seqs=4
Similarity=0.820513
27 TCACCACGACGGGCACTCTGAACTCTTCACAGTGCCCATGCAAGG--CTTTTTT
Rev.of_GCF_001591665.1_ASM159166v1_genomic.fna_22:17-277 Satlength=261 Nr of Repeats=5 RepeatLength=52 seed=CAAAAAAAGC Num.seqs=5
Similarity=0.887179 54
TGGCATCGACGGGCACTCTGGACTCTTCACAGTGCCCGGGCGCGG--CTTTTTT
GCF_001591665.1_ASM159166v1_genomic.fna_39:142722-142982 Satlength=261 Nr of Repeats=5 RepeatLength=52 seed=CACAGTGCCC Num.seqs=5
Similarity=0.800000 27
TGGNCTCGAAGGGCACTCTGAGCTCTTCACAGTGCCC--GCCCGGANCTTTTTT

********** ** ** ******** *** ********** *****
Consensus:

CACAGTGCCCnnGCncGGCTTTTTTTggcctCGAcGGGCACTCTGaaCTCTT

>Bacillus_Fam_275_53_3 Nr. of seq. 3 Alignment length(with gaps) = 54
Alignment score = 0.672840
GCF_002019605.1_ASM201960v1_genomic.fna_1:4870238-4870551 Satlength=314 Nr of Repeats=6 RepeatLength=52 seed=GTAACGAAAG Num.seqs=5
Similarity=0.828931 0
GTAACGAAAGAGGAGGATTAGTGCCCGTAAGAGG-AAAAATCAGGAGA-ATACG
Rev.of_GCF_002019605.1_ASM201960v1_genomic.fna_1:647737-648108 Satlength=372 Nr of Repeats=7 RepeatLength=53 seed=TTCTCCTAAT Num.seqs=5
Similarity=0.844025 49
GGAACGAAAGAGATGCATTCGTTCTCATACGAGGTAAAAATTAGGAGAAAG-CA
GCF_002019605.1_ASM201960v1_genomic.fna_1:1057717-1057929 Satlength=213 Nr of Repeats=4 RepeatLength=53 seed=AAGAGGTGCA Num.seqs=4
Similarity=0.865828 61
GGAACGAAAGAGGTGCATTCGTGCCTGTGAGAGGAAAAAATTAGGAGA-AGCCA

* ********** * *** ** * * **** ****** ****** * *
Consensus:

GgAACGAAAGAGgtGcATTcGTgCccgTaaGAGGnAAAAATtAGGAGAAgnCa

>Bacillus_Fam_276_53_3 Nr. of seq. 3 Alignment length(with gaps) = 54
Alignment score = 0.738683
GCF_001439915.1_ASM143991v1_genomic.fna_94:581745-582063 Satlength=319 Nr of Repeats=6 RepeatLength=53 seed=GTGTACCTCT Num.seqs=6

Similarity=0.824691 0
GTGTACCTCTAGAACCATCTTGCGGTACATGCTCCTTCATATTTCACGCC-AGC
Rev.of_GCF_001439915.1_ASM143991v1_genomic.fna_123:2-265 Satlength=264 Nr of Repeats=5 RepeatLength=53 seed=GGGTGAGCAT Num.seqs=4
Similarity=0.945493 38
ATGTACCGCTAGACTCCTCTTGCGGGACATGCT-CACCCTATTTCACACCAAGC
Rev.of_GCF_001439915.1_ASM143991v1_genomic.fna_126:203282-203547 Satlength=266 Nr of Repeats=5 RepeatLength=53 seed=TGAAATAGGG Num.seqs=5
Similarity=0.876730 45
GTGTACCGCTAGACACTTCATGCGGCACATGCT-CACCCTATTTCACACCAAGC

****** ***** * ** ***** ******* * * ******** ** ***
Consensus:

gTGTACCgCTAGAcnCnTCtTGCGGnACATGCTCacCcTATTTCACaCCaAGC

>Bacillus_Fam_277_51_3 Nr. of seq. 3 Alignment length(with gaps) = 53
Alignment score = 0.639413
GCF_001375535.1_Bacillus_niameyensis_genomic.fna_6:2695072-2695736 Satlength=665 Nr of Repeats=13 RepeatLength=51 seed=GAATAGAAGC Num.seqs=12 Similarity=0.816201 0
GAATAGAAGCGCCCTATTCGACCGTTACA-CG-NGGTTCCACTTCCTTCGGTC
GCF_001375535.1_Bacillus_niameyensis_genomic.fna_6:2711389-2711951 Satlength=563 Nr of Repeats=11 RepeatLength=51 seed=CTATTCGACC Num.seqs=10 Similarity=0.819567 13
GAATAGAAGCTTCCTATTCGACCGTTGCA-CGAGGGTTTCCCTT-CTTCGGGA
GCF_001375535.1_Bacillus_niameyensis_genomic.fna_6:1751787-1752723 Satlength=937 Nr of Repeats=18 RepeatLength=52 seed=CTATTCGACC Num.seqs=18 Similarity=0.885333 13
GAATAGAACCCCTCTATTCGACCGTTCAATCG-ATTTTTCAATTCTTTCGGGC

******** * ************* * ** ** * ** *****
Consensus:

GAATAGAAgCnccCTATTCGACCGTTncACGnggTTtCacTTccTTCGGgc

>Bacillus_Fam_278_51_3 Nr. of seq. 3 Alignment length(with gaps) = 53
Alignment score = 0.635220
GCF_001420595.1_ASM142059v1_genomic.fna_11:4524426-4524886 Satlength=461 Nr of Repeats=9 RepeatLength=51 seed=TGTCTGAACT Num.seqs=8
Similarity=0.829132 0
TGTCTGAACTTGAGCTAACTTCGGACTTCTTC-CGGATTTTTCTCCTG-ATCT
GCF_001420595.1_ASM142059v1_genomic.fna_11:4601981-4602760 Satlength=780 Nr of Repeats=14 RepeatLength=52 seed=ATCTTGTCTG Num.seqs=12
Similarity=0.803807 48
TGTCTGAACTTGAACTAACTTCGGACTCCAGCATGCCTTTTTCTNCGG-ATCT
GCF_000934845.1_ASM93484v1_genomic.fna_37:39135-39695 Satlength=561 Nr of Repeats=11 RepeatLength=52 seed=AAACCTGTCC Num.seqs=9
Similarity=0.792735
47 TGTCCGAACTTTGGCTAACTTCGGACTACTTT-TGCCTGTTTTNCCTGAAACC

**** ****** ************ * * * *** * * * *
Consensus:

TGTCtGAACTTgagCTAACTTCGGACTnCttctGccTtTTTctcCtGAtCt

>Bacillus_Fam_279_51_3 Nr. of seq. 3 Alignment length(with gaps) = 53
Alignment score = 0.681342
GCF_900104555.1_IMG-taxon_2651870172_annotated_assembly_genomic.fna_6:21894-22205
Satlength=312 Nr of Repeats=6 RepeatLength=51 seed=ACTTTATTTT Num.seqs=5
Similarity=0.793590 0 ACTTTATTTTCGATTAT-TGA-TNGGAAATGTCTTTCATAAGGTCGATGAAGG
GCF_900104555.1_IMG-taxon_2651870172_annotated_assembly_genomic.fna_16:59730-59985
Satlength=256 Nr of Repeats=5 RepeatLength=51 seed=TGAAAATGTC Num.seqs=5
Similarity=0.864052 21 ACTTTATGTT-GATTATATAG-TTGAAAATGTCTTTCATGAGCTCGATGAAGG
GCF_900104555.1_IMG-taxon_2651870172_annotated_assembly_genomic.fna_10:55052-55308
Satlength=257 Nr of Repeats=5 RepeatLength=51 seed=GACTTTATTT Num.seqs=4
Similarity=0.777778 50 ACTTTATTTTGGATT-T-TAACTTAAAAATGTCCTTCATAAGGCTGATGAAAG

******* ** **** * *   *   ******* ***** **   ****** *
Consensus:

ACTTTATtTTnGATTaTTaaTtgaAAATGTCtTTCATaAGgtcGATGAAgG

>Bacillus_Fam_280_52_3 Nr. of seq. 3 Alignment length(with gaps) = 53
Alignment score = 0.666667
GCF_000430765.1_ASM43076v1_genomic.fna_5:240069-240277 Satlength=209 Nr of Repeats=4 RepeatLength=52 seed=TTTTGTCCTT Num.seqs=4
Similarity=0.880342
0 TTTTGTCCTTCATCCCCCTGATGAAGGACCGTTCTCCTGCACGTGAC-CCTGG
Rev.of_GCF_001636425.1_ASM163642v1_genomic.fna_1:4233510-4233770
Satlength=261 Nr of Repeats=5 RepeatLength=52 seed=GGTCCTTCAT Num.seqs=5
Similarity=0.793711 30
TTTGGGCCTTCATCCGCCTTATGAAGGACCGTTCT-CTGTATTTTTCTCTTCG
Rev.of_GCF_001636425.1_ASM163642v1_genomic.fna_1:4415449-4415969
Satlength=521 Nr of Repeats=10 RepeatLength=52 seed=ATGAAGGACA
Num.seqs=10 Similarity=0.782099
65 TTTTGTCCTTCATTCCCCCTATGAAGGACGGTTCA-CTGAATTTTGCACCTCG

*** * ******* * ** ********* **** *** * * * * * *
Consensus:

TTTtGtCCTTCATcCcCCttATGAAGGACcGTTCtCTGnAttTtnCnCcTcG

>Bacillus_Fam_281_52_3 Nr. of seq. 3 Alignment length(with gaps) = 53
Alignment score = 0.752621
GCF_000708755.2_ASM70875v2_genomic.fna_2:1643369-1643733 Satlength=365 Nr of Repeats=7 RepeatLength=52 seed=AGTTTCTGCT Num.seqs=7
Similarity=0.855922 0
AGTTTCTGCTGCTAAATCCAGTTAAGAACACTTTCACCCGCAAAC-CAACCAG

GCF_000709935.2_ASM70993v2_genomic.fna_3:65504-65920 Satlength=417 Nr of Repeats=8 RepeatLength=52 seed=GAACACTTTC Num.seqs=8 Similarity=0.821429
25 AGTTTCTGCTGCTAAATCCAGTTAAGAACACTTTCACCCGCAAAC-CAACCAG
GCF_000708755.2_ASM70875v2_genomic.fna_7:166561-166873 Satlength=313 Nr of Repeats=6 RepeatLength=52 seed=TTTACAACAC Num.seqs=6 Similarity=0.847863
20 -GTTTCTGCTGCTAACTCTCGTTTACAACACTTTCAACCGCTCACGCTTCAAA

************** ** *** * ********** **** ** * * *
Consensus:

aGTTTCTGCTGCTAAaTCcaGTTaAgAACACTTTCAcCCGCaaACCaaCcAg

>Bacillus_Fam_282_52_3 Nr. of seq. 3 Alignment length(with gaps) = 53
Alignment score = 0.746331
GCF_000752395.1_Bacillus_andreraoultii_genomic.fna_14:112431-113991 Satlength=1561 Nr of Repeats=30 RepeatLength=52 seed=TACACGAAAA Num.seqs=30 Similarity=0.924727 0
TACACGAAAACGGGCTTATAGAACCT-CTCTATGAACCCAAATTTTATCGAAC
Rev.of_GCF_000752395.1_Bacillus_andreraoultii_genomic.fna_24:24595-25427 Satlength=833 Nr of Repeats=15 RepeatLength=52 seed=GTTCTATGAG Num.seqs=14 Similarity=0.809626 24
CACATGAAAAGTGGCTCATAGAACCT-NTCTATGAACCCGAATTTGACCGAAA
Rev.of_GCF_000752395.1_Bacillus_andreraoultii_genomic.fna_46:151000-151208 Satlength=209 Nr of Repeats=4 RepeatLength=52 seed=GGGTTCATAG Num.seqs=4 Similarity=0.837607 39
CACATGAAAAACGGCTCATAGAA-CTCTTCTATGAACCCAAATTTGATCGAAT

*** ***** **** ****** ** *********** ***** * ****
Consensus:

cACAtGAAAAnnGGCTcATAGAAcCTnTCTATGAACCCaAATTTgAtCGAAn

>Bacillus_Fam_283_52_3 Nr. of seq. 3 Alignment length(with gaps) = 53
Alignment score = 0.658281
GCF_000787375.1_ASM78737v1_genomic.fna_1:181126-181438 Satlength=313 Nr of Repeats=6 RepeatLength=52 seed=TTGACTCCCG Num.seqs=6 Similarity=0.791195
0 TTGACTCCCGTTCTTCCGCCCCTTCGCT-TTTTTCGGGAGTCATCACTCCTCT
GCF_000787375.1_ASM78737v1_genomic.fna_25:25502-25762 Satlength=261 Nr of Repeats=5 RepeatLength=52 seed=CGCTCTTTGG Num.seqs=5 Similarity=0.912821
46 TTGGCTACCGTTCTTTCTCTCTTTCTCC-TTTTTCGGGAGCCATCCCCGCTCT
GCF_000787375.1_ASM78737v1_genomic.fna_4:180648-181012 Satlength=365 Nr of Repeats=5 RepeatLength=52 seed=GTTTTTCGGT Num.seqs=4 Similarity=0.880342
27 TTGACTTCCGTTCTTCC-CACGTTCTCCGTTTTTCGGTAGCCAACACATCCCT

*** ** ******** * * * *** * ******** ** ** * * * **
Consensus:

TTGaCTnCCGTTCTTcCnCnCnTTCtCcTTTTTCGGgAGcCAtCaCnnCtCT

>Bacillus_Fam_284_52_3 Nr. of seq. 3 Alignment length(with gaps) = 53
Alignment score = 0.618449

GCF_000986785.1_ASM98678v1_genomic.fna_2:158190-158798 Satlength=609 Nr of Repeats=12 RepeatLength=52 seed=CCTCCATCAC Num.seqs=11 Similarity=0.821911 0 CCTCCATCACACCCATGGAGGACCTTTTTCTCCNAATTTCACGAAAGNTGGG-
GCF_000986785.1_ASM98678v1_genomic.fna_8:188503-189125 Satlength=623 Nr of Repeats=11 RepeatLength=52 seed=GTCCTCCATC Num.seqs=10 Similarity=0.811966 50 CCTCCATCACCCTCATGGAGGACCGCTTT-TCCATTTCTCACGCTATTTCGGT
GCF_000986785.1_ASM98678v1_genomic.fna_21:12509-12820 Satlength=312 Nr of Repeats=6 RepeatLength=52 seed=CCTCCATCAC Num.seqs=5 Similarity=0.882051
52   CCTCCATCACCCTCATGGAAGACCTTTTT-TCCTCTTCCCTCCCCAAAGCGGT

********** * ****** **** *** ***   * * *   *   **
Consensus:

CCTCCATCACcCtCATGGAgGACCttTTTTCCnntTctCaCgcnAnntcGGt

>Bacillus_Fam_285_52_3 Nr. of seq. 3 Alignment length(with gaps) = 53
Alignment score = 0.907757
GCF_001439965.1_ASM143996v1_genomic.fna_89:30076-30284 Satlength=209 Nr of Repeats=4 RepeatLength=52 seed=AATGTGTGTC Num.seqs=4 Similarity=0.848008
0   AATGTGTGTCCAAGAGA-GAGCTTGAAACACATTATAAGAGAAACCGGAGGAA
GCF_001887185.1_ASM188718v1_genomic.fna_260:30450-30918 Satlength=469 Nr of Repeats=9 RepeatLength=52 seed=TGAAACACAT Num.seqs=9 Similarity=0.791077
22   AATGTGTGTCCAAGAGANGAGN-TGAAACACATTATAAGAGAAACCGGAGGAG
GCF_002009555.1_ASM200955v1_genomic.fna_301:3466-3778 Satlength=313 Nr of Repeats=6 RepeatLength=52 seed=TGAAACACAT Num.seqs=6 Similarity=0.781151
22   AATGTGTGTCCAAGAGAGGAGC-TGAAACACATTATAAGAGAAACCGGAGGAG

***************** *** *****************************
Consensus:

AATGTGTGTCCAAGAGAnGAGcTGAAACACATTATAAGAGAAACCGGAGGAg

>Bacillus_Fam_286_53_3 Nr. of seq. 3 Alignment length(with gaps) = 53
Alignment score = 0.851153
GCF_001439965.1_ASM143996v1_genomic.fna_95:72244-72698 Satlength=455 Nr of Repeats=9 RepeatLength=52 seed=ATTGGTTACC Num.seqs=7 Similarity=0.792430
0   ATTGGTTACCCGGTTGCGC-GATTTCACCCGNACTCGGTCACCTTTCGGCNTG
Rev.of_GCF_001887185.1_ASM188718v1_genomic.fna_11:78-494 Satlength=417 Nr of Repeats=8 RepeatLength=52 seed=GGGTAACCAA Num.seqs=8 Similarity=0.813118 11 ATTGGTTACCCTGTTGCGCGGATTTCACCCGGACTCGGTCACCTTTGGGCNTG
GCF_001887185.1_ASM188718v1_genomic.fna_43:121-747 Satlength=627 Nr of Repeats=12 RepeatLength=52 seed=TTGGTTACCC Num.seqs=9 Similarity=0.765898
1   ATTGGTTACCCGGTTG-GCGGATTTCACGTGGACTCGGTCACCTTTCGGCNTG

*********** **** ** ******** * ************** ******
Consensus:

ATTGGTTACCCgGTTGcGCgGATTTCACccGgACTCGGTCACCTTTcGGCNTG

>Bacillus_Fam_287_52_3 Nr. of seq. 3 Alignment length(with gaps) = 53
Alignment score = 0.633124
GCF_001591665.1_ASM159166v1_genomic.fna_53:52093-52301 Satlength=209 Nr of Repeats=4 RepeatLength=52 seed=TTAAAGACAT Num.seqs=4 Similarity=0.792977
0 TTAAAGACATTTTGAGC-ACATTGATGGGCAGTTATGTCTTTAAGAAAGGCTA
GCF_001591665.1_ASM159166v1_genomic.fna_91:5115-5323 Satlength=209 Nr of Repeats=4 RepeatLength=52 seed=ATTAAAGACA Num.seqs=4 Similarity=0.752058
51 TTAAAGACATTTTGGTC-GGTAAGACAGGGAGTTTTGTCTTTAAGGAGGGCTA
GCF_001591665.1_ASM159166v1_genomic.fna_54:84694-84902 Satlength=209 Nr of Repeats=4 RepeatLength=52 seed=GTATTAAAGA Num.seqs=4 Similarity=0.876068
49 TTAAAGACATTTT-AGCTATTTTTAGAGGTGGTTTTGTCCTTAAGAAGGGGTA

************* * * ** *** **** ***** * ** **
Consensus:

TTAAAGACATTTTgagCanttttgAnaGGnaGTTtTGTCtTTAAGaAgGGcTA

>Bacillus_Fam_288_52_3 Nr. of seq. 3 Alignment length(with gaps) = 53
Alignment score = 0.731656
GCF_001591805.1_ASM159180v1_genomic.fna_5:182944-183204 Satlength=261 Nr of Repeats=5 RepeatLength=52 seed=CTTATAAGCT Num.seqs=5 Similarity=0.851572
0 CTTATAAGCTACCCAACTTGGATGATTTTCTCGGTTTCGGTAGCTTATAC-CT
GCF_001591805.1_ASM159180v1_genomic.fna_68:9875-10082 Satlength=208 Nr of Repeats=4 RepeatLength=52 seed=TTATAAGCTA Num.seqs=3 Similarity=0.880342
1 CTTATAAGCTACCCAACTTGGGCGGCTTTCTCGGTTTCGGTCGCTTATAC-CC
Rev.of_GCF_001591805.1_ASM159180v1_genomic.fna_63:307-671 Satlength=365 Nr of Repeats=7 RepeatLength=52 seed=AAGTTGGGTA Num.seqs=7 Similarity=0.865690 18
-TTATAAGCTACCCAACTTCCGCAAATCCCNCGGCTTCGGTCGCTTATACACC

****************** * * *** ****** ******** *
Consensus:

cTTATAAGCTACCCAACTTgggcganTttCtCGGtTTCGGTcGCTTATACCc

>Bacillus_Fam_289_52_3 Nr. of seq. 3 Alignment length(with gaps) = 53
Alignment score = 0.645702
GCF_001636315.1_ASM163631v1_genomic.fna_1:834005-834369 Satlength=365 Nr of Repeats=7 RepeatLength=52 seed=AAAGTGTTTC Num.seqs=7 Similarity=0.796002
0 AAAGTGTTTCCAATCAAGGCTATTGGCGACATAACTCAGTATAAAAANNTGA-
GCF_001636315.1_ASM163631v1_genomic.fna_1:4300776-4301088 Satlength=313 Nr of Repeats=6 RepeatLength=52 seed=GCGACAAAAC Num.seqs=6 Similarity=0.704527 25
GAAGTGTATCCAATGAAGGCTATTGGCGACAAAACTCTGTTGGAAAAGGGGC-
GCF_001636315.1_ASM163631v1_genomic.fna_1:3938063-3939308 Satlength=1246 Nr of Repeats=11 RepeatLength=52 seed=TGGAAACAAA Num.seqs=9

Similarity=0.925926 22
-AAATGTTTCCAATGAGGGCTATTGGAAACAAAACTCACGAAGAAAGCGGGAG

** *** ****** * ********* *** *****   ***   *
Consensus:

nAAgTGTtTCCAATgAaGGCTATTGGcgACAaAACTCagtangAAAanggGa

>Bacillus_Fam_290_52_3 Nr. of seq. 3 Alignment length(with gaps) = 53
Alignment score = 0.607966
GCF_001636315.1_ASM163631v1_genomic.fna_1:2347811-2348434 Satlength=624
Nr of Repeats=12 RepeatLength=52 seed=TGGAATCACT Num.seqs=11
Similarity=0.844289 0
TGGAATCACTTTTCTC-TNTTTTTCTCCNAGTTTTGTTTCCAATGACCCTGAT
Rev.of_GCF_001636315.1_ASM163631v1_genomic.fna_1:3352830-3353038
Satlength=209 Nr of Repeats=4 RepeatLength=52 seed=GCGACAAAAG Num.seqs=4
Similarity=0.880342 39
TGGATTCACTTTACTC-TCTTTTGCTCCGGCTTTTGTCGCCAATAGCCTTCAT
GCF_001636315.1_ASM163631v1_genomic.fna_1:3814968-3815747 Satlength=780
Nr of Repeats=15 RepeatLength=52 seed=TTGGAAACAC Num.seqs=14
Similarity=0.832884 51
TGGAAACACTTTACTCGTATTTT-CACAGAGATTTGTTTCCAATAGGTGCGAT

**** ****** *** * **** * *   ***** *****   **
Consensus:

TGGAatCACTTTaCTCTnTTTTnCtCcgagtTTTGTttCCAATagccntgAT

>Bacillus_Fam_291_52_3 Nr. of seq. 3 Alignment length(with gaps) = 53
Alignment score = 0.714885
GCF_002019645.1_ASM201964v1_genomic.fna_1:115528-116203 Satlength=676 Nr of Repeats=13 RepeatLength=52 seed=GCTGTCCGAA Num.seqs=12
Similarity=0.753627 0
GCTGTCCGAACTTGGCTGAGGTTCAGACAGCTTGAGGAACAAAAGAGAGAAA-
GCF_002019645.1_ASM201964v1_genomic.fna_1:2536105-2536365 Satlength=261
Nr of Repeats=5 RepeatLength=52 seed=AAGAGGAAAA Num.seqs=5
Similarity=0.929560 42
GCTGTCCGAAGTGGAGTGAGGTTCAGACAGGTTGAGGGTGAGAAGAG-GAAAA
Rev.of_GCF_002019645.1_ASM201964v1_genomic.fna_1:4937598-4938897
Satlength=1300 Nr of Repeats=25 RepeatLength=52 seed=TACTTCGGAC
Num.seqs=24 Similarity=0.864549
65 GCTGTCCGAAGTAGAGTGAGGTTCAGACAGCTTTGGTGTGCGAAGNG-GAAAA

********** * * ************** ** *   *** * ****
Consensus:

GCTGTCCGAAgTnGagTGAGGTTCAGACAGcTTgaGggtgagAAGaGGAAAa

>Bacillus_Fam_292_52_3 Nr. of seq. 3 Alignment length(with gaps) = 53
Alignment score = 0.704403
GCF_002019645.1_ASM201964v1_genomic.fna_1:995262-996146 Satlength=885 Nr of Repeats=17 RepeatLength=52 seed=AAGGTTCGGA Num.seqs=17
Similarity=0.819570 0
AAGGTTCGGACAGCTTTTGCTCGAAG-GCCCTCGAAGTTGTCCGAAGTNGCCC

Rev.of_GCF_002019645.1_ASM201964v1_genomic.fna_1:4106739-4107991 Satlength=1253 Nr of Repeats=24 RepeatLength=52 seed=ACTTCGGACA Num.seqs=20 Similarity=0.924831
47   AAGGTTCGGACAGCTTTAGCTTCCGG-GCATTCGAAGTTGTCCGAAGTCGCTC
Rev.of_GCF_002019645.1_ASM201964v1_genomic.fna_1:4426732-4427356 Satlength=625 Nr of Repeats=12 RepeatLength=52 seed=ACTTCGGACA Num.seqs=12 Similarity=0.829060
47   GGGGTTCGGACAGCTTTTGGTCTCAGAGGA-TCGAAGTTGTCCGAAGTTGTTC

*************** * *   * *   ***************** * *
Consensus:

aaGGTTCGGACAGCTTTtGcTcncaGGcanTCGAAGTTGTCCGAAGTnGctC

>Bacillus_Fam_293_52_3 Nr. of seq. 3 Alignment length(with gaps) = 53
Alignment score = 0.637317
GCF_900104555.1_IMG-taxon_2651870172_annotated_assembly_genomic.fna_20:557960-558325 Satlength=366 Nr of Repeats=7 RepeatLength=52 seed=GTCTGAAGTC Num.seqs=6 Similarity=0.732510   0   GTCTGAAGTC-ACCTTAGGTTCAGACACAGTTGCATGTGAAATCACCAATCCT
Rev.of_GCF_900104555.1_IMG-taxon_2651870172_annotated_assembly_genomic.fna_22:368575-368885 Satlength=311 Nr of Repeats=6 RepeatLength=52 seed=CTTCAGACAG Num.seqs=4 Similarity=0.635288   8   GTCTGAAGTC-AGGNTAGGTTCGGACACGCTTGGATTTGAAATCGCTGATCCT
GCF_900104555.1_IMG-taxon_2651870172_annotated_assembly_genomic.fna_22:384819-385184 Satlength=366 Nr of Repeats=7 RepeatLength=52 seed=TGTCTGAAGT Num.seqs=6 Similarity=0.741090   51   GTCTGAAGTCGACCCTA-GTTCGGACTCTGGTGCACTTGCAATCACTTCTCTT

********** *   ** **** *** *   ** * ** **** *   ** *
Consensus:

GTCTGAAGTCAccnTAgGTTCgGACaCngtTGcAttTGaAATCaCtnaTCcT

>Bacillus_Fam_294_51_3 Nr. of seq. 3 Alignment length(with gaps) = 52
Alignment score = 0.651709
GCF_002019605.1_ASM201960v1_genomic.fna_1:184216-184471 Satlength=256 Nr of Repeats=5 RepeatLength=51 seed=TATAAGGAAG Num.seqs=3 Similarity=0.839744
0   TATAAGGAAGTGATGAGTGCTAAAAGTCAGGATAGCTCATGA-TATTACCAG
Rev.of_GCF_002019605.1_ASM201960v1_genomic.fna_1:1956898-1957102 Satlength=205 Nr of Repeats=4 RepeatLength=51 seed=CCTCATACTG Num.seqs=4 Similarity=0.930283   7
TATGAGGAAGAAATGAGCGCTAAAGCGAAAGATAGAACACTA-TATTACCAG
GCF_002019605.1_ASM201960v1_genomic.fna_1:4935750-4936209 Satlength=460 Nr of Repeats=9 RepeatLength=51 seed=AGTGCTAAAG Num.seqs=9 Similarity=0.852578   15
TATAAGTAAGAAATGAGTGCTAAAGCGGAGGATAGAGC-TTATTGTTTCCAG

*** ** *** ***** ******   * ***** *   * * ** ****
Consensus:

TATaAGgAAGaaATGAGtGCTAAAgcgnAgGATAGanCattATaTTaCCAG

>Bacillus_Fam_295_52_3 Nr. of seq. 3 Alignment length(with gaps) = 52
Alignment score = 0.707265
GCF_000169195.2_ASM16919v2_genomic.fna_1:649385-650061 Satlength=677 Nr of Repeats=13 RepeatLength=52 seed=TTAAGGAAGA Num.seqs=13
Similarity=0.725689 0
TTAAGGAAGATCGAAAAAAATCGTCCCTTTCTTAACGTAGANCCGTTCTACG
Rev.of_GCF_000169195.2_ASM16919v2_genomic.fna_1:669488-670145
Satlength=658 Nr of Repeats=10 RepeatLength=52 seed=CTTCCTTAAC
Num.seqs=7 Similarity=0.743590
9 TTAAGGAAGNCCGNNAAAAAGNCNCGCTATCTTAACGTAGAGGCGTTCTATG
GCF_000169195.2_ASM16919v2_genomic.fna_1:2578666-2579497 Satlength=832 Nr of Repeats=15 RepeatLength=52 seed=GTAAAGGAAG Num.seqs=13
Similarity=0.728270 51
TAAAGGAAGNCCGGCAAAAAGTGTGTCTATCTTAACATAGAGGCGTTCTANG

* ******* ** ***** ** ******* **** ******* *
Consensus:

TtAAGGAAGncCGnnAAAAAgngtcnCTaTCTTAACgTAGAggCGTTCTAnG

>Bacillus_Fam_296_52_3 Nr. of seq. 3 Alignment length(with gaps) = 52
Alignment score = 0.841880
GCF_000171615.1_ASM17161v1_genomic.fna_52:43-615 Satlength=573 Nr of Repeats=10 RepeatLength=52 seed=GGCGCGCTTA Num.seqs=9
Similarity=0.811321
0 GGCGCGCTTACCCTAGCGCGNGGCTCTTCTCTCTTTTTTCTTCTTCTTGGGA
GCF_000171615.1_ASM17161v1_genomic.fna_60:29-599 Satlength=571 Nr of Repeats=8 RepeatLength=52 seed=GCGCTTACCC Num.seqs=5 Similarity=0.892308
3 GGCGCGCTTACCCTAACGCGGGGCTCTTCTCCCTTTTTTCTTCTTCTTGGGA
Rev.of_GCF_000171615.1_ASM17161v1_genomic.fna_130:149-408 Satlength=260 Nr of Repeats=5 RepeatLength=52 seed=AAGAAGAAAA Num.seqs=4
Similarity=0.846154 45
GGGGCGCTGTCCCAAGCGCGTGTCTCTTCTCTCTTTTTTCTTCTTCTTGGGG

** ***** *** * **** * ******** ******************
Consensus:

GGcGCGCTtaCCCtAgCGCGnGgCTCTTCTCtCTTTTTTCTTCTTCTTGGGa

>Bacillus_Fam_297_52_3 Nr. of seq. 3 Alignment length(with gaps) = 52
Alignment score = 0.675214
GCF_000177235.2_ASM17723v2_genomic.fna_1:726998-727934 Satlength=937 Nr of Repeats=18 RepeatLength=52 seed=AAGCGTGTCA Num.seqs=18
Similarity=0.904475 0
AAGCGTGTCATACGAAGGCAATGCCGCATGAAAAATCGGGACGCAGGAAATA
Rev.of_GCF_000177235.2_ASM17723v2_genomic.fna_1:4063448-4064381
Satlength=934 Nr of Repeats=18 RepeatLength=52 seed=CATTGCTCTC
Num.seqs=15 Similarity=0.906227
23 AAGCGTGTCATACGAGAGCAATGCCACATGAAAAAAAGCAAACAAGCAAATA
Rev.of_GCF_000177235.2_ASM17723v2_genomic.fna_1:1193617-1194293
Satlength=677 Nr of Repeats=13 RepeatLength=52 seed=ATGTGTCATT

Num.seqs=9 Similarity=0.752970
29   ACNCGTGTCATACAAGGNCAATGACACATGAAAAATAAGAAGCAATCCAATA

* ********** *   ***** * *********   *   *   ****
Consensus:

AagCGTGTCATACgAgggCAATGcCaCATGAAAAAtaggaAncaAgcaAATA

>Bacillus_Fam_298_52_3 Nr. of seq. 3 Alignment length(with gaps) = 52
Alignment score = 0.688034
GCF_000374565.1_ASM37456v1_genomic.fna_3:72207-73350 Satlength=1144 Nr of Repeats=22 RepeatLength=52 seed=AAACGGTAAC Num.seqs=21
Similarity=0.805750
0   AAACGGTAACTAATCGTGGGGAAATGTTCCCGTTTTGTGCCTGAGTTCGAAC
GCF_000374565.1_ASM37456v1_genomic.fna_16:37328-37794 Satlength=467 Nr of Repeats=9 RepeatLength=52 seed=GAAAAAACGG Num.seqs=7 Similarity=0.672252
48   AAACGGGAACTAATCGTGAGGAAAGGTTACCGGTTGGGGCCCGTGTTNGAAA
GCF_000374565.1_ASM37456v1_genomic.fna_3:246080-246600 Satlength=521 Nr of Repeats=10 RepeatLength=52 seed=CGGTAACTAA Num.seqs=8
Similarity=0.785827
3   AACCGGTAACTAATGGAGAAGAAAGGTGCCCGCTTTGTGTCCGATTTCGAAA

** *** ******* * * **** ** *** ** * * * * ** ***
Consensus:

AAaCGGtAACTAATcGtGagGAAAgGTtcCCGnTTtGtGcCcGagTTcGAAa

>Bacillus_Fam_299_52_3 Nr. of seq. 3 Alignment length(with gaps) = 52
Alignment score = 0.846154
GCF_000708755.2_ASM70875v2_genomic.fna_1:102871-103131 Satlength=261 Nr of Repeats=4 RepeatLength=52 seed=AAAGTGTTCT Num.seqs=3
Similarity=0.752621
0   AAAGTGTTCTAANCCAGAGTAAGCAGCACAAAGTGAGTAGANCACAGGCTCG
GCF_000709935.2_ASM70993v2_genomic.fna_1:103820-104028 Satlength=209 Nr of Repeats=4 RepeatLength=52 seed=CAAAAGTGTT Num.seqs=4
Similarity=0.786325
50   AAAGTGTTCTAAACCAGAGTTAGCAGCACAAACTGAGTAGAGCACAGGCCCA
Rev.of_GCF_000708755.2_ASM70875v2_genomic.fna_6:383976-384236
Satlength=261 Nr of Repeats=5 RepeatLength=52 seed=TGCTAACTCT Num.seqs=5
Similarity=0.853846   25
AAAGTGTTCTAACCCAGAGTTAGCAGCACAAAGTGAGTAGAGCCCGGTCCCG

************ ******* *********** ******** * * * * *
Consensus:

AAAGTGTTCTAAnCCAGAGTtAGCAGCACAAAgTGAGTAGAgCaCaGgCcCg

>Bacillus_Fam_300_52_3 Nr. of seq. 3 Alignment length(with gaps) = 52
Alignment score = 0.811966
GCF_000708755.2_ASM70875v2_genomic.fna_1:366565-366877 Satlength=313 Nr of Repeats=6 RepeatLength=52 seed=GAGTTTGTGC Num.seqs=6
Similarity=0.782906
0   GAGTTTGTGCTGCTAAATCATGTATAGAACACTTTCGGCNGGGCCCCGCACT

Rev.of_GCF_000708755.2_ASM70875v2_genomic.fna_2:47476-47840 Satlength=365 Nr of Repeats=7 RepeatLength=52 seed=ATGAGTTAGC Num.seqs=7 Similarity=0.841858 21
GAGTTTATGCTGCTAACTCATGTTTGGAACACTTTCGCCGCGGACCCGCACC
Rev.of_GCF_000709935.2_ASM70993v2_genomic.fna_24:86775-87035 Satlength=261 Nr of Repeats=5 RepeatLength=52 seed=TAGCAGCACA Num.seqs=5 Similarity=0.856410 67
GAGTTTGTGCTGCTAACTCTTGTTTGGAACACTTTNGCCGTGGACCCGCACC

****** ********* ** *** * ********* * * ** *******
Consensus:

GAGTTTgTGCTGCTAAcTCaTGTtTgGAACACTTTcGcCgnGGaCCCGCACc

>Bacillus_Fam_301_52_3 Nr. of seq. 3 Alignment length(with gaps) = 52
Alignment score = 0.809829
GCF_000708755.2_ASM70875v2_genomic.fna_6:144195-144506 Satlength=312 Nr of Repeats=6 RepeatLength=52 seed=AAGGGTCATA Num.seqs=5 Similarity=0.784615
0 AAGGGTCATAANGACACATGATTTAACCCTTCAGCATGAAGGAAACTCCGTG
GCF_000709935.2_ASM70993v2_genomic.fna_15:61477-61788 Satlength=312 Nr of Repeats=6 RepeatLength=52 seed=TGAAGGGTCA Num.seqs=5 Similarity=0.797436
50 AAGGGTCATAANGACACATGATTTAACCCTTCAGCATGAAGGAAACTCCGTG
Rev.of_GCF_000709935.2_ASM70993v2_genomic.fna_2:33696-33956 Satlength=261 Nr of Repeats=5 RepeatLength=52 seed=CATGCTGAAG Num.seqs=5 Similarity=0.842593 38
AAAGGTCATAAGCCCGCGTGATATAACCCTTCAGCATGAAGGAGAGTGTGTG

** ******** * * **** ******************* * * ***
Consensus:

AAgGGTCATAAngaCaCaTGATtTAACCCTTCAGCATGAAGGAaAcTccGTG

>Bacillus_Fam_302_52_3 Nr. of seq. 3 Alignment length(with gaps) = 52
Alignment score = 0.760684
GCF_000759675.1_ASM75967v1_genomic.fna_13:117550-117862 Satlength=313 Nr of Repeats=6 RepeatLength=52 seed=CTGGAGTGAC Num.seqs=6 Similarity=0.935043
0 CTGGAGTGACCGTAGATCATATAGAAAATGCTTTTGGGTCAATGCAGATAGG
Rev.of_GCF_000759675.1_ASM75967v1_genomic.fna_19:541006-541370 Satlength=365 Nr of Repeats=7 RepeatLength=52 seed=TTCGGTCACT Num.seqs=7 Similarity=0.868132 14
CTGGAGTGACCGAAAAGCATGTAAAAAAGGCATATGGGTCAATGCAGGAAGT
GCF_000759675.1_ASM75967v1_genomic.fna_136:8346-8709 Satlength=364 Nr of Repeats=7 RepeatLength=52 seed=GAGTGACCGA Num.seqs=6 Similarity=0.781197
55 CTGGAGTGACCGAAAAGCATTCAGGAAAAGCATTTGGGTCAATGCAGATAGT

************ * * *** * *** ** * ************* **
Consensus:

CTGGAGTGACCGaAaAgCATntAgaAAAnGCaTtTGGGTCAATGCAGatAGt

>Bacillus_Fam_303_52_3 Nr. of seq. 3 Alignment length(with gaps) = 52
Alignment score = 0.713675

GCF_000813125.1_ASM81312v1_genomic.fna_102:117072-117487 Satlength=416 Nr of Repeats=8 RepeatLength=52 seed=GACAGCTTTG Num.seqs=7 Similarity=0.807082 0
GACAGCTTTGAAGGAAATCAGCGTAAAGCTGTCTGAACTTGGTGCTACTTCT
Rev.of_GCF_000934845.1_ASM93484v1_genomic.fna_83:5-265 Satlength=261 Nr of Repeats=5 RepeatLength=52 seed=GTCCGAAGTA Num.seqs=5 Similarity=0.713333
3 GACTACTTTTACGGAAAAAGGCGGAAACCTGTCCGAACTTGGGCNTACTTCG
Rev.of_GCF_000934845.1_ASM93484v1_genomic.fna_94:227-539 Satlength=313 Nr of Repeats=6 RepeatLength=52 seed=GCAAAAGCAG Num.seqs=6 Similarity=0.862055 12
GACTGCTTTTGCGGAAAAAGGCGGAAAGTGGTCCGAACTTGGGGCTACTTCG

*** **** ***** *** *** *** ******** ******
Consensus:

GACtgCTTTtacGGAAAaagGCGgAAAgctGTCcGAACTTGGggcTACTTCg

>Bacillus_Fam_304_52_3 Nr. of seq. 3 Alignment length(with gaps) = 52 Alignment score = 0.675214
GCF_001375535.1_Bacillus_niameyensis_genomic.fna_6:3162845-3163209 Satlength=365 Nr of Repeats=7 RepeatLength=52 seed=AATCGTTACC Num.seqs=7 Similarity=0.782775 0
AATCGTTACCGTTTTACATGAGAGCACGTCAAGACGGTAACGAATCAGAGCN
Rev.of_GCF_001375535.1_Bacillus_niameyensis_genomic.fna_6:4427320-4431322 Satlength=4003 Nr of Repeats=77 RepeatLength=52 seed=TGAATCGTTC Num.seqs=75 Similarity=0.994622 47
AATCGTTACCTTTTCATATGCGATCACCGCAATACGGGAACGATTCAGAGCC
GCF_001375535.1_Bacillus_niameyensis_genomic.fna_6:4422988-4423300 Satlength=313 Nr of Repeats=6 RepeatLength=52 seed=GGTAACGATT Num.seqs=6 Similarity=0.769231 35
AATCGTGCCCGTTTCGGATGGGANCACGGCAACATGGTAACGATTCGGAGTG

****** ** *** *** ** *** *** * ** ***** ** ***
Consensus:

AATCGTtaCCgTTTcanATGnGAnCACggCAAnAcGGtAACGAtTCaGAGcn

>Bacillus_Fam_305_52_3 Nr. of seq. 3 Alignment length(with gaps) = 52 Alignment score = 0.705128
GCF_001439915.1_ASM143991v1_genomic.fna_83:493100-493309 Satlength=210 Nr of Repeats=4 RepeatLength=52 seed=GGCTTATTCG Num.seqs=3 Similarity=0.914530 0
GGCTTATTCGGACAGAACAAGCAAGAAATGCCAATAGAGAGTCCGAATGCAA
GCF_001439915.1_ASM143991v1_genomic.fna_116:79569-79934 Satlength=366 Nr of Repeats=7 RepeatLength=52 seed=ATTCGGACAG Num.seqs=6 Similarity=0.789744
5 GGCTAATTCGGACAGGACAAGCGGAAAAAGTCGGTAGAGAGTCCGAATGCAA
GCF_001439915.1_ASM143991v1_genomic.fna_123:290092-290300 Satlength=209 Nr of Repeats=4 RepeatLength=52 seed=ATTTGGACAG Num.seqs=4 Similarity=0.586420 5
GGTTCATTTGGACAGACCAATGTAGAACTGCCGNTAGAGAGTCCAAATGCAA

** * *** ****** *** ** * * ********** *******

Consensus:

GGcTnATTcGGACAGaaCAAgcnagAAatGcCgnTAGAGAGTCCgAATGCAA

>Bacillus_Fam_306_52_3 Nr. of seq. 3 Alignment length(with gaps) = 52
Alignment score = 0.726496
GCF_001439965.1_ASM143996v1_genomic.fna_56:1630-1890 Satlength=261 Nr of Repeats=4 RepeatLength=52 seed=TGATTGATTA Num.seqs=3 Similarity=0.794872
0 TGATTGATTACCCTGAAGGNGAAAATTGCATGTGTACGGGAACCATANAGAG
GCF_002009555.1_ASM200955v1_genomic.fna_109:9635-9947 Satlength=313 Nr of Repeats=5 RepeatLength=52 seed=TGATTGATTA Num.seqs=4 Similarity=0.854701
0 TGATTGATTACCCTGAAGATGAAAATTGCATGTGTACGGGAACCATACAGAG
Rev.of_GCF_002009555.1_ASM200955v1_genomic.fna_27:20337-20857
Satlength=521 Nr of Repeats=10 RepeatLength=52 seed=AGGGTAACCA
Num.seqs=10 Similarity=0.871225
14 GGTTTGGTTACCCTGTTGATGGAAATTGCGCCTGTACGGTAACCAATGGGAG

* *** ******** * * ******* ******* ***** ***
Consensus:

tGaTTGaTTACCCTGaaGatGaAAATTGCatgTGTACGGgAACCAtanaGAG

>Bacillus_Fam_307_52_3 Nr. of seq. 3 Alignment length(with gaps) = 52
Alignment score = 0.899573
GCF_001439965.1_ASM143996v1_genomic.fna_108:192897-193886 Satlength=990 Nr of Repeats=20 RepeatLength=52 seed=GGTAACCATT Num.seqs=17 Similarity=0.698483 0
GGTAACCATTGANGGACTTTGGTGACCCTTTTGGNGGAAAACTCCTTTCNTC
GCF_001887185.1_ASM188718v1_genomic.fna_193:64604-65229 Satlength=626 Nr of Repeats=12 RepeatLength=52 seed=AAACTCCTTT Num.seqs=11 Similarity=0.767366 38
GGTAACCATTGAGGGCCTTTGGTGACCCTTTNGGNGGAAAACTCCTTTCTTC
Rev.of_GCF_002009555.1_ASM200955v1_genomic.fna_553:44922-45390
Satlength=469 Nr of Repeats=8 RepeatLength=52 seed=AAAGGAGTTT Num.seqs=6 Similarity=0.735185 48
GGTAACCATTGTGGGCCTTTGGTGACCCTTTGGGNGGAAAACTCCTTTCTTC

*********** ** *************** **************** **
Consensus:

GGTAACCATTGagGGcCTTTGGTGACCCTTTnGGNGGAAAACTCCTTTCtTC

>Bacillus_Fam_308_52_3 Nr. of seq. 3 Alignment length(with gaps) = 52
Alignment score = 0.929487
GCF_001439965.1_ASM143996v1_genomic.fna_121:22311-22571 Satlength=261 Nr of Repeats=5 RepeatLength=52 seed=AGAAAGAGGA Num.seqs=5 Similarity=0.887179
0 AGAAAGAGGAAGAAAGGTAACCAATCGCCCGGAAAGGTTCCCGAAACACGAA
GCF_001887185.1_ASM188718v1_genomic.fna_194:35985-36921 Satlength=937 Nr of Repeats=18 RepeatLength=52 seed=AAAGAAAGAG Num.seqs=18 Similarity=0.771074 50
AGAAAGAGGAAGAAAGGTAACCAATCGCTCGGAAAGGTTACCGAAACTNGAA
GCF_002009555.1_ASM200955v1_genomic.fna_16:8263-9199 Satlength=937 Nr of Repeats=18 RepeatLength=52 seed=AAAGAAAGAG Num.seqs=18

Similarity=0.771074
50   AGAAAGAGGAAGAAAGGTAACCAATCGCTCGGAAAGGTTACCGAAACTNGAA

*************************** ********** *******   ***
Consensus:

AGAAAGAGGAAGAAAGGTAACCAATCGCtCGGAAAGGTTaCCGAAACtnGAA

>Bacillus_Fam_309_52_3 Nr. of seq. 3 Alignment length(with gaps) = 52
Alignment score = 0.739316
GCF_001439965.1_ASM143996v1_genomic.fna_121:87820-88132 Satlength=313 Nr of Repeats=6 RepeatLength=52 seed=TTGAAACACA Num.seqs=6
Similarity=0.820513
0   TTGAAACACATTATAAGAGAATATTAGAGAAAATGTGTCTCAAGTATAGTGT
Rev.of_GCF_001439965.1_ASM143996v1_genomic.fna_124:121491-121767
Satlength=277 Nr of Repeats=5 RepeatLength=52 seed=CAAAATGTGT Num.seqs=3
Similarity=0.773663   15
TGGATACACATTTTGNTAGAATATTGCGGATAATGTGTCTCAAGGATGGCGG
Rev.of_GCF_001887185.1_ASM188718v1_genomic.fna_148:130132-130548
Satlength=417 Nr of Repeats=8 RepeatLength=52 seed=CAAAATGTGT Num.seqs=6
Similarity=0.878632   15
TGGATACACATTTTGCGAGAATATTGCAGATAATCTGTCTCAAGGATGGCGG

* ** ******* *   ********   ** *** ********* ** * *
Consensus:

TgGAtACACATTtTgngAGAATATTgcaGAtAATgTGTCTCAAGgATgGcGg

>Bacillus_Fam_310_52_3 Nr. of seq. 3 Alignment length(with gaps) = 52
Alignment score = 0.982906
GCF_001439965.1_ASM143996v1_genomic.fna_122:46240-46602 Satlength=363 Nr of Repeats=7 RepeatLength=52 seed=CTTAAATAGA Num.seqs=6
Similarity=0.780606
0   CTTAAATAGAGGAAACCAAGTGTGTAGGGTAACTAAAGCGAGCGAAAGGTTA
Rev.of_GCF_001887185.1_ASM188718v1_genomic.fna_62:176111-176735
Satlength=625 Nr of Repeats=12 RepeatLength=52 seed=TTTAAGTAAC
Num.seqs=12 Similarity=0.876845
6   CTTAAATAGAGGAAACCAGGTGTGTAGGGTAACTAAAGCGAGCGAAAGGTTA
Rev.of_GCF_002009555.1_ASM200955v1_genomic.fna_91:21-333 Satlength=313 Nr of Repeats=6 RepeatLength=52 seed=TTTAAGTAAC Num.seqs=6
Similarity=0.873504   6
CTTAAATAGAGGAAACCAGGTGTGTAGGGTAACTAAAGCGAGCGAAAGGTTA

****************** *********************************
Consensus:

CTTAAATAGAGGAAACCAgGTGTGTAGGGTAACTAAAGCGAGCGAAAGGTTA

>Bacillus_Fam_311_52_3 Nr. of seq. 3 Alignment length(with gaps) = 52
Alignment score = 0.730769
GCF_001439965.1_ASM143996v1_genomic.fna_125:170548-171172 Satlength=625 Nr of Repeats=11 RepeatLength=52 seed=TGGTTACCGA Num.seqs=10
Similarity=0.670576   0
TGGTTACCGAGTCCNCGAGATATCCACCTTCAGGGTAACCAAACANGTNTTA

GCF_001887185.1_ASM188718v1_genomic.fna_98:26215-26474 Satlength=260 Nr of Repeats=5 RepeatLength=52 seed=AAGGTTACCG Num.seqs=4 Similarity=0.652263
51 AGGTTACCGAATCCATGAGAAATCCACCTAAAGGGTAACGAAACGAGCGCAA
Rev.of_GCF_002009555.1_ASM200955v1_genomic.fna_334:9897-10157 Satlength=261 Nr of Repeats=5 RepeatLength=52 seed=TCGGTAACCT Num.seqs=5 Similarity=0.744654 62
AGGTTACCGAATCNATGAGAAATCCACCTANAGGGTAACGAACCCAGCGCAA

********* ** **** ******** ******** ** * * *
Consensus:

aGGTTACCGAaTCcatGAGAaATCCACCTanAGGGTAACgAAaCnaGcgcaA

>Bacillus_Fam_312_52_3 Nr. of seq. 3 Alignment length(with gaps) = 52 Alignment score = 0.649573
GCF_001636325.1_ASM163632v1_genomic.fna_1:60206-60777 Satlength=572 Nr of Repeats=11 RepeatLength=52 seed=AAAGGTAGAA Num.seqs=10 Similarity=0.805724
0 AAAGGTAGAAGAGAGTGAGTGTCAACGCCCATCTGAGGTAGAAAAAGAGAAG
GCF_001636325.1_ASM163632v1_genomic.fna_2:158704-159484 Satlength=781 Nr of Repeats=15 RepeatLength=52 seed=GAAGAGCGAG Num.seqs=15 Similarity=0.855106 7
AAAGGGAGAAGAGCGAGCGTCTCATCGCCCATCTGAGCGAGAAGAAGAGTAG
GCF_001636325.1_ASM163632v1_genomic.fna_2:308084-308398 Satlength=315 Nr of Repeats=6 RepeatLength=52 seed=TGAAAGGAAG Num.seqs=5 Similarity=0.738462
50 AAAGGAAGAAGAGAGCACGTCTCAGCGCCCAAGTGATAGAGAATAGCCGATG

***** ******* * ** *** ****** *** **** * * *
Consensus:

AAAGGnAGAAGAGaGngcGTcTCAnCGCCCAtcTGAgngAGAAnAagaGaaG

>Bacillus_Fam_313_52_3 Nr. of seq. 3 Alignment length(with gaps) = 52 Alignment score = 0.675214
GCF_001636335.1_ASM163633v1_genomic.fna_1:2043697-2044009 Satlength=313 Nr of Repeats=6 RepeatLength=52 seed=TATGGGACAA Num.seqs=6 Similarity=0.810256 0
TATGGGACAAAAGATTACTGATTTCTGCTAATTTTGTCCCATTGAGCTCTCC
GCF_001636335.1_ASM163633v1_genomic.fna_1:2271770-2272029 Satlength=260 Nr of Repeats=5 RepeatLength=52 seed=GTCTCATTGA Num.seqs=4 Similarity=0.931624 35
TATGGGACACTTGCTTTCCTTTTTCAGCTTTTTTTGTCTCATTGAGCTCTCC
GCF_001636335.1_ASM163633v1_genomic.fna_1:2474604-2475436 Satlength=833 Nr of Repeats=16 RepeatLength=52 seed=TATGGGACAG Num.seqs=16 Similarity=0.867949 52
TATGGGACAGTTCCTTCGGCTTCTCGGCTTGTTTTGTCTCATTGAGCCCTCC

********* ** * ** *** ******* ******** ****
Consensus:

TATGGGACAnttgcTTncnntTtTCnGCTtnTTTTGTCtCATTGAGCtCTCC

>Bacillus_Fam_314_52_3 Nr. of seq. 3 Alignment length(with gaps) = 52
Alignment score = 0.982906
GCF_001645705.1_ASM164570v1_genomic.fna_19:10035-10867 Satlength=833 Nr of Repeats=16 RepeatLength=52 seed=AAAAGGGAAC Num.seqs=16
Similarity=0.930769 0
AAAAGGGAACATAGAGTCCTTGTATGTACGCGAAACTGGTGGGAAGACTCCG
GCF_001645705.1_ASM164570v1_genomic.fna_57:14-794 Satlength=781 Nr of Repeats=15 RepeatLength=52 seed=TTGTATGTAC Num.seqs=15
Similarity=0.903804
19   AAAAGGGAACATAGAGTCCTTGTATGTACGCGAAACTGGTGGGAAGACTCCG
GCF_001645705.1_ASM164570v1_genomic.fna_35:14312-14884 Satlength=573 Nr of Repeats=11 RepeatLength=52 seed=AAAAGGGAAC Num.seqs=11
Similarity=0.938462 0
AAAAGGGAACATAGAGTCCTTGTATGTACGCGAAACTGGTGAGAAGACTCCG

*** **********
Consensus:

AAAAGGGAACATAGAGTCCTTGTATGTACGCGAAACTGGTGgGAAGACTCCG

>Bacillus_Fam_315_52_3 Nr. of seq. 3 Alignment length(with gaps) = 52
Alignment score = 0.946581
GCF_900156865.1_PRJEB18960_genomic.fna_3:4195877-4197024 Satlength=1148 Nr of Repeats=22 RepeatLength=52 seed=GAGCAAAGAA Num.seqs=20
Similarity=0.876788 0
GAGCAAAGAACATGTCATCATCGANCGGATGAAGACACGAATAGAGGTGTAG
GCF_900156865.1_PRJEB18960_genomic.fna_3:4199517-4200091 Satlength=575 Nr of Repeats=11 RepeatLength=52 seed=TGTCATCATC Num.seqs=10
Similarity=0.891168 12
GAGCAAAGAACATGTCATCATCGANAGGATGAAGACACGAATAGAAGTGTAG
GCF_900156865.1_PRJEB18960_genomic.fna_3:4202611-4202819 Satlength=209 Nr of Repeats=4 RepeatLength=52 seed=CAAAGAACAT Num.seqs=4
Similarity=0.914530 55
GAGCAAAGAACATGTCATCATCGAAAGGATGAAGACACGAATAGAAGTGTAG

************************ ******************* ******
Consensus:

GAGCAAAGAACATGTCATCATCGAnaGGATGAAGACACGAATAGAaGTGTAG

>Bacillus_Fam_316_48_3 Nr. of seq. 3 Alignment length(with gaps) = 48
Alignment score = 0.981481
GCF_000706725.1_ASM70672v1_genomic.fna_1:2952140-2952854 Satlength=715 Nr of Repeats=15 RepeatLength=48 seed=TTCTTCTGAT Num.seqs=10
Similarity=0.925309 0
TTCTTCTGATGACGTTGAGAACGACTCAGATGCTGACAGTGACGATGA
Rev.of_GCF_002019765.1_ASM201976v1_genomic.fna_1:1074786-1075170
Satlength=385 Nr of Repeats=8 RepeatLength=48 seed=TCATCAGAAG Num.seqs=8
Similarity=0.870040 12
TTCTTCTGATGACGTTGAGAACGACTCAGATGCTGACAGTGACGATGA
GCF_001420645.1_ASM142064v1_genomic.fna_7:5330-5618 Satlength=289 Nr of Repeats=6 RepeatLength=48 seed=ATTCTTCTGA Num.seqs=6 Similarity=0.874074
47   TTCTTCTGATGACGTTGAGAACGACTCAGACGCTGACAGTGACGATGA

***************************** ****************
Consensus:

TTCTTCTGATGACGTTGAGAACGACTCAGAtGCTGACAGTGACGATGA

>Bacillus_Fam_317_45_3 Nr. of seq. 3 Alignment length(with gaps) = 46
Alignment score = 0.666667
GCF_000017425.1_ASM1742v1_genomic.fna_1:1678527-1678779 Satlength=253 Nr of Repeats=6 RepeatLength=42 seed=GAACAAAAGC Num.seqs=6 Similarity=0.841270
0   GAACAAAAGCCAGAAGATA-C-AAA-AAAACCGAATGACGAAGAC-
GCF_000715205.1_SOAPdenovo_v1.05_genomic.fna_11:26905-27400 Satlength=496 Nr of Repeats=11 RepeatLength=45 seed=AAGCCAGACG Num.seqs=11 Similarity=0.941818   5
GCAC-AAAGCCAGACGATAGCGAAACAAAACCAGATGATCAAAACG
Rev.of_GCF_001895885.1_ASM189588v1_genomic.fna_1:3714696-3714876 Satlength=181 Nr of Repeats=5 RepeatLength=45 seed=TCTGGTTTTG Num.seqs=3 Similarity=0.637188   13
GTAC-AAAACCAGACGATAACGAAACAAAGCCGGATGACCAAAACG

* ** *** ***** **** * *** *** ** **** ** **
Consensus:

GnACAAAgCCAGAcGATAnCgAAAcAAAaCCggATGAccAAaACg

>Bacillus_Fam_318_40_3 Nr. of seq. 3 Alignment length(with gaps) = 43
Alignment score = 0.666667
GCF_000612805.1_FF3_genomic.fna_1:485384-485548 Satlength=165 Nr of Repeats=4 RepeatLength=41 seed=AATTCGCAGA Num.seqs=4 Similarity=0.902439
0   AATTCGCAGATAAGTTAT-GAGAAACCGCAGATAACAC-GTGA
GCF_000612805.1_FF3_genomic.fna_6:82591-82851 Satlength=261 Nr of Repeats=5 RepeatLength=41 seed=GAAATTCGCA Num.seqs=4 Similarity=0.951219
39   AATTCGCAGATAAATGGT-GA-AAACCGCAGATAACTCAGTGA
Rev.of_GCF_000612805.1_FF3_genomic.fna_2:681994-682158 Satlength=165 Nr of Repeats=4 RepeatLength=41 seed=TATGACTTAT Num.seqs=4 Similarity=0.869919
19   AAAACGCAGATAAGTCATAGA-AATTCGCAGATAACTC-GGGG

** ********* * * ** ** ********** * * *
Consensus:

AAttCGCAGATAAgTnaTGAAAacCGCAGATAACtCGtGa

>Bacillus_Fam_319_39_3 Nr. of seq. 3 Alignment length(with gaps) = 42
Alignment score = 0.722222
GCF_000245315.1_ASM24531v1_genomic.fna_41:263357-263714 Satlength=358 Nr of Repeats=9 RepeatLength=39 seed=ATAAAAAATC Num.seqs=6 Similarity=0.904274
0   ATAAAAAATCGTACTGTTCTCAC---AAGAGATCCCGCAGCC
GCF_001584325.1_ASM158432v1_genomic.fna_15:65449-65833 Satlength=385 Nr of Repeats=10 RepeatLength=39 seed=CTCACAAAAG Num.seqs=8 Similarity=0.910867
18   ATAAAAAATCTTTCTGTTCTCAC---AAAAGATCTCGCAGCC

GCF_000245335.1_ASM24533v1_genomic.fna_3:546265-546469 Satlength=205 Nr of Repeats=5 RepeatLength=42 seed=CGCAGCTACA Num.seqs=3 Similarity=0.894180
35   ACAAAAAGTCTTACTGCTCTCACAAGAAAAGATCACGCAGCT

* ***** ** * *** ******   ** ***** ******
Consensus:

AtAAAAAaTCtTaCTGtTCTCACAAaAGATCnCGCAGCc

>Bacillus_Fam_320_42_3 Nr. of seq. 3 Alignment length(with gaps) = 42 Alignment score = 0.978836
GCF_000161455.1_ASM16145v1_genomic.fna_1:840010-840472 Satlength=463 Nr of Repeats=11 RepeatLength=42 seed=GCAGAAGAGA Num.seqs=11 Similarity=0.825108 0
GCAGAAGAGAAAGTAGCAGAATTAAAACAGACGAAGCAAAAT
GCF_000161455.1_ASM16145v1_genomic.fna_1:840138-840729 Satlength=592 Nr of Repeats=11 RepeatLength=42 seed=AGAAGAGAAA Num.seqs=8 Similarity=0.866213
2   GCAGAAGAGAAAGTAGCAGAATTAAAACAGACGAAGCAAAAT
GCF_000712615.1_ASM71261v1_genomic.fna_24:59125-59698 Satlength=574 Nr of Repeats=13 RepeatLength=42 seed=AGAAGAGAAA Num.seqs=10 Similarity=0.747306
2   GCAGAAGAGAAAGTATCAGAATTAAAACAGACGAAGCAAAAT

*************** **************************
Consensus:

GCAGAAGAGAAAGTAgCAGAATTAAAACAGACGAAGCAAAAT

>Bacillus_Fam_321_40_3 Nr. of seq. 3 Alignment length(with gaps) = 40 Alignment score = 0.766667
GCF_000374565.1_ASM37456v1_genomic.fna_1:446313-446554 Satlength=242 Nr of Repeats=6 RepeatLength=40 seed=TAAGAGTATT Num.seqs=5 Similarity=0.886667
0   TAAGAGTATTTCCTAATTCCGAAGAGTTTCCCGGCAAAGT
Rev.of_GCF_000374565.1_ASM37456v1_genomic.fna_3:243558-244159 Satlength=602 Nr of Repeats=15 RepeatLength=40 seed=GGATACTCTT Num.seqs=14 Similarity=0.856410
31   TAAGAGTATTTCCGTTTTCCGAAGAGTATCCTCGCAAAGT
GCF_000374565.1_ASM37456v1_genomic.fna_15:92410-92570 Satlength=161 Nr of Repeats=4 RepeatLength=40 seed=CAAAGTAAAG Num.seqs=4 Similarity=0.900406
34   AAAGAGTATTTCTGATTTCTGAAGAGTATTCACGCAAAGT

***********   *** ******* * * *******
Consensus:

tAAGAGTATTTCcgatTTCcGAAGAGTaTcCncGCAAAGT

>Bacillus_Fam_322_39_3 Nr. of seq. 3 Alignment length(with gaps) = 39 Alignment score = 0.863248
GCF_000007825.1_ASM782v1_genomic.fna_1:2224382-2224538 Satlength=157 Nr of Repeats=4 RepeatLength=39 seed=ACGACACCTC Num.seqs=4

Similarity=0.982906
0   ACGACACCTCCAGCAAATAATGGAGGAGGTCAAGGAAAT
GCF_000008505.1_ASM850v1_genomic.fna_1:2199269-2199461 Satlength=193 Nr of Repeats=5 RepeatLength=39 seed=GGTCAAGGAA Num.seqs=4
Similarity=0.937322
27   ACAACCCCTCCAGCAAATAACGGAGGAGGTCAAGGAAAT
Rev.of_GCF_001645555.1_ASM164555v1_genomic.fna_12:477757-477910
Satlength=154 Nr of Repeats=4 RepeatLength=39 seed=GCTGGAGGTG Num.seqs=3
Similarity=0.931624   14
ACACCACCTCCAGCGAATAACGGAGGAGGCCAAGGAAAT

** * ******** ***** ******** *********
Consensus:

ACaaCaCCTCCAGCaAATAAcGGAGGAGGtCAAGGAAAT

>Bacillus_Fam_323_39_3 Nr. of seq. 3 Alignment length(with gaps) = 39
Alignment score = 0.749288
GCF_000007845.1_ASM784v1_genomic.fna_1:4522244-4522556 Satlength=313 Nr of Repeats=6 RepeatLength=39 seed=TCAGGGGCAA Num.seqs=4
Similarity=0.792361
0   TCAGGGGCAACAGAAACACTCTAAGCAAGACGACTCCGA
GCF_000008505.1_ASM850v1_genomic.fna_1:4519171-4519561 Satlength=391 Nr of Repeats=10 RepeatLength=39 seed=TCTAAGCAAG Num.seqs=10
Similarity=0.822222   19
TCAAGGGCAACAGAAACACTCTAAGCAAGACGACTCCAA
Rev.of_GCF_001645555.1_ASM164555v1_genomic.fna_4:37147-37492
Satlength=346 Nr of Repeats=9 RepeatLength=39 seed=TGATTCTGAT Num.seqs=7
Similarity=0.873016   9
TCAGAATCAACAACAAAACTCTAAACAAGACAACTCGAA

***   ***** ** ******* ****** **** *
Consensus:

TCAggggCAACAgaAAcACTCTAAgCAAGACgACTCcaA

>Bacillus_Fam_324_36_3 Nr. of seq. 3 Alignment length(with gaps) = 36
Alignment score = 0.811728
GCF_000290995.1_Baci_cere_AND1407_G13175_V1_genomic.fna_1:414507-414678
Satlength=172 Nr of Repeats=5 RepeatLength=33 seed=AAGAAACAAG Num.seqs=3
Similarity=0.919192   0   -
AAGAAACAAGAGGAAGCAAAAAAACTAGAAGAG--
Rev.of_GCF_000775975.1_ASM77597v1_genomic.fna_1:3533346-3533562
Satlength=217 Nr of Repeats=6 RepeatLength=36 seed=CTCTCTTCTA Num.seqs=6
Similarity=0.871605   0
TAAGAAGCAAGAAGAGGCGAAAAAGCTAGAAGAGAG
Rev.of_GCF_000832605.1_ASM83260v1_genomic.fna_1:4477135-4477387
Satlength=253 Nr of Repeats=7 RepeatLength=36 seed=CTCTCTTCTA Num.seqs=7
Similarity=0.890653   0
TAAGAAACAAGAAGAGGCGAAAAAGCTAGAAGAGAG

***** ***** ** ** ***** *********
Consensus:

tAAGAAaCAAGAaGAgGCgAAAAAgCTAGAAGAGag

>Bacillus_Fam_325_36_3 Nr. of seq. 3 Alignment length(with gaps) = 36
Alignment score = 0.969136
GCF_000706725.1_ASM70672v1_genomic.fna_1:1338140-1338448 Satlength=309 Nr of Repeats=5 RepeatLength=36 seed=AATGCAACAA Num.seqs=4
Similarity=0.938272 0
AATGCAACAAGGGATGGGAGGAAACCACCATGCCCC
GCF_001420645.1_ASM142064v1_genomic.fna_39:121221-121545 Satlength=325 Nr of Repeats=9 RepeatLength=36 seed=ATGCAACAAG Num.seqs=9
Similarity=0.903292 1
AATGCAACAAGGGATGGGAGGAAACCACCANGCCCC
Rev.of_GCF_002019765.1_ASM201976v1_genomic.fna_1:2724974-2725514
Satlength=541 Nr of Repeats=15 RepeatLength=36 seed=CCTTGTTGCA
Num.seqs=15 Similarity=0.918871
12 AATGCAACAAGGGATGGGAGGAAACCACCACGCCCC

***************************** *****
Consensus:

AATGCAACAAGGGATGGGAGGAAACCACCAnGCCCC

>Bacillus_Fam_326_34_3 Nr. of seq. 3 Alignment length(with gaps) = 35
Alignment score = 0.615873
GCF_000612805.1_FF3_genomic.fna_5:17272-17470 Satlength=199 Nr of Repeats=6 RepeatLength=33 seed=CGACGAGTAA Num.seqs=6 Similarity=1.000000
0 CGACGAGTAATCGCAGGAGCATA--TGTTTTTTTG
Rev.of_GCF_000429725.1_ASM42972v1_genomic.fna_11:96592-96732
Satlength=141 Nr of Repeats=4 RepeatLength=35 seed=TGCTCCTGCG Num.seqs=4
Similarity=0.974603 21
CGACGAGTAATCGCAGGAGCAAACGGGCTGTGATG
Rev.of_GCF_000429725.1_ASM42972v1_genomic.fna_8:26322-26492 Satlength=171 Nr of Repeats=5 RepeatLength=34 seed=CTGCTCCTGC Num.seqs=5
Similarity=0.898039 22
CGACGAGGAAGCGCAGGAGCAGGC-GGTCTACATG

******* ** ********** * **
Consensus:

CGACGAGtAAtCGCAGGAGCAnacgGttttnaTG

>Bacillus_Fam_327_25_3 Nr. of seq. 3 Alignment length(with gaps) = 26
Alignment score = 0.846154
GCF_001274775.1_ASM127477v1_genomic.fna_5:147304-147704 Satlength=401 Nr of Repeats=16 RepeatLength=25 seed=TTGTGCCTGG Num.seqs=16
Similarity=1.000000 0
TTGTGCCTGGCACCTTT-TCCCCTTT
GCF_001274775.1_ASM127477v1_genomic.fna_6:153795-153895 Satlength=101 Nr of Repeats=4 RepeatLength=25 seed=CCTGGCACCT Num.seqs=4
Similarity=0.911111
5 TTGTACCTGGCACCTTT-TCCCCTTT
Rev.of_GCF_001274775.1_ASM127477v1_genomic.fna_11:335137-335437
Satlength=301 Nr of Repeats=12 RepeatLength=25 seed=AGGTGCCAGG

Num.seqs=12 Similarity=0.862179
14   -TGTGCCTGGCACCTTTCNCCCCTTT

*** ************ *******
Consensus:

tTGTgCCTGGCACCTTTtCCCCTTT

>Bacillus_Fam_328_24_3 Nr. of seq. 3 Alignment length(with gaps) = 24
Alignment score = 0.759259
GCF_000161455.1_ASM16145v1_genomic.fna_1:230973-231201 Satlength=229 Nr of Repeats=4 RepeatLength=24 seed=GAAACCGCCC Num.seqs=3 Similarity=0.925926
0   GAAACCGCCCATGCCCATACCGGG
GCF_000430785.1_ASM43078v1_genomic.fna_57:8024-8408 Satlength=385 Nr of Repeats=16 RepeatLength=24 seed=CCCATTCCAG Num.seqs=16 Similarity=0.931481
13   GAAACCGCCCATGCCCATTCCAGG
GCF_001636315.1_ASM163631v1_genomic.fna_1:5557193-5557289 Satlength=97 Nr of Repeats=4 RepeatLength=24 seed=GGGTATCCAC Num.seqs=4 Similarity=0.888889 22
GTATCCACCCATGCCCATTGCTGG

* * ** *********** * **
Consensus:

GaAaCCgCCCATGCCCATtcCnGG

>Bacillus_Fam_329_21_3 Nr. of seq. 3 Alignment length(with gaps) = 23
Alignment score = 0.661836
GCF_000508325.1_BAVI_1_genomic.fna_8:115910-116014 Satlength=105 Nr of Repeats=5 RepeatLength=21 seed=TTTACTGGCG Num.seqs=4 Similarity=0.589120
0   TTTACTGGCGGA-ATCCCCGAC-
Rev.of_GCF_000508325.1_BAVI_1_genomic.fna_86:37292-37542 Satlength=251 Nr of Repeats=11 RepeatLength=21 seed=CGCCAGTAAA Num.seqs=9 Similarity=0.611111 10
TTTACTGGCGGATANCGCC-AC-
GCF_001591665.1_ASM159166v1_genomic.fna_18:66724-66974 Satlength=251 Nr of Repeats=12 RepeatLength=21 seed=TTTACTGGCG Num.seqs=11 Similarity=0.881097 0
TTTACTGGCGGATA-CNCC-TCT

************ * * ** *
Consensus:

TTTACTGGCGGAtAnCnCCaC

>Bacillus_Fam_330_21_3 Nr. of seq. 3 Alignment length(with gaps) = 23
Alignment score = 0.642512
GCF_000508325.1_BAVI_1_genomic.fna_48:16287-16371 Satlength=85 Nr of Repeats=4 RepeatLength=21 seed=TGGCTATTTT Num.seqs=4 Similarity=0.640212
8   GAGGGTTATGGCTATTTTT--CT
Rev.of_GCF_001591805.1_ASM159180v1_genomic.fna_27:88721-88805 Satlength=85 Nr of Repeats=4 RepeatLength=21 seed=TAGACAAAAC Num.seqs=4

Similarity=0.600242 14
GAGNGTTTTGTCTATTTTT--AT
Rev.of_GCF_001591805.1_ASM159180v1_genomic.fna_15:54241-54325
Satlength=85 Nr of Repeats=4 RepeatLength=21 seed=TAGCCATAAC Num.seqs=4
Similarity=0.612269 10
GTTATGGCTATTTTT-TNATGGA

*   * * *   *
Consensus:

gttnTgtcTaTtTttntGagn

>Bacillus_Fam_331_21_3 Nr. of seq. 3 Alignment length(with gaps) = 23
Alignment score = 0.671498
GCF_001591425.1_ASM159142v1_genomic.fna_97:20-272 Satlength=253 Nr of Repeats=8 RepeatLength=21 seed=GTTGGCACAA Num.seqs=5 Similarity=0.822222
19 TGGCACAAAAGCGA-ATC-CGGT
Rev.of_GCF_001591425.1_ASM159142v1_genomic.fna_138:7019-7720
Satlength=702 Nr of Repeats=7 RepeatLength=21 seed=CTTTTGCGCC Num.seqs=6
Similarity=0.801010 32
TGGCGCAAAAG-GT-ATCTCGGT
GCF_001591425.1_ASM159142v1_genomic.fna_193:37-142 Satlength=106 Nr of Repeats=4 RepeatLength=21 seed=ATCTCGGTTG Num.seqs=3 Similarity=0.808081
35 TGGCGCAAAA--GTAATCTCGGT

****** ***** * *** **
Consensus:

GTTGGCgCAAAAgGtATCtCG

>Bacillus_Fam_332_21_3 Nr. of seq. 3 Alignment length(with gaps) = 23
Alignment score = 0.690821
GCF_001591665.1_ASM159166v1_genomic.fna_27:91341-91425 Satlength=85 Nr of Repeats=4 RepeatLength=21 seed=TTCGCAAATA Num.seqs=4 Similarity=0.661376
12 AAAACC-CTGA-TATTCGCAAAT
Rev.of_GCF_001591805.1_ASM159180v1_genomic.fna_3:0-84 Satlength=85 Nr of Repeats=4 RepeatLength=21 seed=TTGCGAATCT Num.seqs=4 Similarity=0.968254
19 AAAACC-ATCA-GATTCGCAAAT
Rev.of_GCF_001591665.1_ASM159166v1_genomic.fna_46:16384-16468
Satlength=85 Nr of Repeats=4 RepeatLength=21 seed=TTTATTTGCG Num.seqs=4
Similarity=0.607639 24
AAANCCTGTCATTATTCGCAAAT

*********** ** * * *
Consensus:

TTCGCAAATAAAaCCnTcAtA

>Bacillus_Fam_333_21_3 Nr. of seq. 3 Alignment length(with gaps) = 23
Alignment score = 0.637681
GCF_001648575.1_ASM164857v1_genomic.fna_59:127044-127915 Satlength=872 Nr of Repeats=19 RepeatLength=21 seed=TGACGGGATT Num.seqs=12
Similarity=0.566755 0
TGACGGGATTNTCT-TGNGTTG-

Rev.of_GCF_001648575.1_ASM164857v1_genomic.fna_61:125456-126083 Satlength=628 Nr of Repeats=10 RepeatLength=21 seed=AATCCCGTCA Num.seqs=6 Similarity=0.385470
10   TGACGGGATTATCA-CGTGTTAN
GCF_001648575.1_ASM164857v1_genomic.fna_3:22935-23103 Satlength=169 Nr of Repeats=5 RepeatLength=21 seed=TGACGGGATT Num.seqs=4 Similarity=0.391865
0   TGACGGGATTTTCTCCGANTTA-

********** **   * **
Consensus:

TGACGGGATTnTCtcGngTTa

>Bacillus_Fam_334_23_3 Nr. of seq. 3 Alignment length(with gaps) = 23 Alignment score = 0.647343
GCF_001484965.1_ASM148496v1_genomic.fna_5:31017-31101 Satlength=85 Nr of Repeats=4 RepeatLength=21 seed=TCATACCGAC Num.seqs=4 Similarity=0.737374
0   TC-ATACCGACAAGAGCGAGCGT
GCF_001484965.1_ASM148496v1_genomic.fna_5:26815-26983 Satlength=169 Nr of Repeats=8 RepeatLength=21 seed=CGGACAAGAG Num.seqs=8 Similarity=0.714286
6   TCNATACGGACAAGAGCGAGAGC
Rev.of_GCF_001484965.1_ASM148496v1_genomic.fna_11:871-997 Satlength=127 Nr of Repeats=6 RepeatLength=21 seed=TGTCCGATTG Num.seqs=6 Similarity=0.755556   11
TCAAT-CGGACAGGAGGNAGCGC

** ** * **** *** ** *
Consensus:

TCnATaCgGACAaGAGcgAGcGc

>Bacillus_Fam_335_23_3 Nr. of seq. 3 Alignment length(with gaps) = 23 Alignment score = 0.743961
GCF_000612665.1_FF4_genomic.fna_55:18802-18886 Satlength=85 Nr of Repeats=4 RepeatLength=21 seed=TATTTGCGAA Num.seqs=4 Similarity=0.552083
0   TATTTGCGAATTTCCTTGCTNTT
Rev.of_GCF_001591805.1_ASM159180v1_genomic.fna_24:33836-33920 Satlength=85 Nr of Repeats=4 RepeatLength=21 seed=ATTCGCAAAT Num.seqs=4 Similarity=0.570602   11
TATTTGCGAATTACCTGGCNTTT
Rev.of_GCF_001591665.1_ASM159166v1_genomic.fna_15:12666-12750 Satlength=85 Nr of Repeats=4 RepeatLength=21 seed=TTCGCAAATA Num.seqs=4 Similarity=0.469017   10
TATTTGCGAATTACNGNGNNTTT

************ *   *   **
Consensus:

TATTTGCGAATTaCctnGcntTT

>Bacillus_Fam_336_20_3 Nr. of seq. 3 Alignment length(with gaps) = 22 Alignment score = 0.676768
GCF_000008505.1_ASM850v1_genomic.fna_1:4488701-4488841 Satlength=141 Nr of Repeats=7 RepeatLength=20 seed=TATATCGGCG Num.seqs=7

Similarity=0.662887
0   TATATCGGCGA-TTC-TCCATT
GCF_000007845.1_ASM784v1_genomic.fna_1:4493623-4493703 Satlength=81 Nr of Repeats=4 RepeatLength=20 seed=TATATCGGCG Num.seqs=4 Similarity=0.567460
0   TATATCGGCGACATC-TCGATT
GCF_001636315.1_ASM163631v1_genomic.fna_1:11705-11788 Satlength=84 Nr of Repeats=4 RepeatLength=21 seed=TTTATCGGCG Num.seqs=3 Similarity=0.504630
19   T-TATCGGCGA-TTCNTCGANT

* ********* ** ** * *
Consensus:

TaTATCGGCGAtTCTCgAtT

>Bacillus_Fam_337_20_3 Nr. of seq. 3 Alignment length(with gaps) = 22
Alignment score = 0.651515
GCF_000285535.1_ASM28553v1_genomic.fna_2:498937-499757 Satlength=821 Nr of Repeats=30 RepeatLength=20 seed=GACGCAAATA Num.seqs=19
Similarity=0.625341 0
GACGCAAAAAACCTNGT--GGN
Rev.of_GCF_000285535.1_ASM28553v1_genomic.fna_6:20567-21714
Satlength=1148 Nr of Repeats=52 RepeatLength=20 seed=TTTTTGCGTC
Num.seqs=41 Similarity=0.672416
10   GACGCAAAAANCTCGGT--GGA
Rev.of_GCF_000285535.1_ASM28553v1_genomic.fna_2:814688-815514
Satlength=827 Nr of Repeats=13 RepeatLength=20 seed=TTTTTGCGTC
Num.seqs=8 Similarity=0.607143
10   GACGCAAAAANNTTGGTCAGGA

**********   ** **
Consensus:

GACGCAAAAAncttgGTGGa

>Bacillus_Fam_338_21_3 Nr. of seq. 3 Alignment length(with gaps) = 22
Alignment score = 0.732323
GCF_000401235.1_BacNeaAAU1_genomic.fna_89:3-145 Satlength=143 Nr of Repeats=7 RepeatLength=20 seed=ATCAGCCAAA Num.seqs=5 Similarity=0.880000
0   ATCAGCCAAAGCAGCTG-G-AT
GCF_000401235.1_BacNeaAAU1_genomic.fna_88:17091-17236 Satlength=146 Nr of Repeats=7 RepeatLength=21 seed=ATCAGCCAAA Num.seqs=5 Similarity=0.688889
0   ATCAGCCAAAGCAGNTGNG-AT
Rev.of_GCF_001591585.1_ASM159158v1_genomic.fna_21:35910-36015
Satlength=106 Nr of Repeats=5 RepeatLength=21 seed=TTTGGCTGAT Num.seqs=5
Similarity=0.671212 10
ATCAGCCAAAGTAG-TGGGTAT

*********** ** ** * **
Consensus:

ATCAGCCAAAGcAGnTGnGAT

>Bacillus_Fam_339_21_3 Nr. of seq. 3 Alignment length(with gaps) = 22
Alignment score = 0.772727

GCF_001591585.1_ASM159158v1_genomic.fna_12:54775-55019 Satlength=245 Nr of Repeats=12 RepeatLength=20 seed=GTTGGCTGAT Num.seqs=8 Similarity=0.681159
0 -TTGGCTGATTTCTCCTTCCG-
GCF_001591585.1_ASM159158v1_genomic.fna_3:245600-245705 Satlength=106 Nr of Repeats=5 RepeatLength=21 seed=TTTGGCTGAT Num.seqs=5 Similarity=0.629167
0 TTTGGCTGATTTCTCC-TCCGC
GCF_001591585.1_ASM159158v1_genomic.fna_25:28599-28911 Satlength=313 Nr of Repeats=15 RepeatLength=21 seed=TTGGCTGATA Num.seqs=13 Similarity=0.736264 1
TTTGGCTGATTTCCCC-TCCAC

************ ** ***
Consensus:

tTTGGCTGATTTCtCCTCCgc

>Bacillus_Fam_340_21_3 Nr. of seq. 3 Alignment length(with gaps) = 22 Alignment score = 0.616162
GCF_001877785.1_PlanoSAMM_genomic.fna_106:177497-177577 Satlength=81 Nr of Repeats=4 RepeatLength=20 seed=AATGGATCAT Num.seqs=4 Similarity=0.755556
0 AATGGATCATAAA-TCTCCAA-
GCF_001315085.1_ASM131508v1_genomic.fna_1:3481-3565 Satlength=85 Nr of Repeats=4 RepeatLength=21 seed=GGATCATAAA Num.seqs=4 Similarity=0.458333
3 GTTGGATCATAAA-TCCGCAAT
GCF_001315085.1_ASM131508v1_genomic.fna_3:99320-99404 Satlength=85 Nr of Repeats=4 RepeatLength=21 seed=GGATCATAAA Num.seqs=4 Similarity=0.518519
3 TCTGGATCATAAATTATCNAAT

*********** * **
Consensus:

nnTGGATCATAAATctccAAt

>Bacillus_Fam_341_21_3 Nr. of seq. 3 Alignment length(with gaps) = 22 Alignment score = 0.727273
GCF_000025805.1_ASM2580v1_genomic.fna_1:339971-340130 Satlength=160 Nr of Repeats=5 RepeatLength=21 seed=ACTCCTATAA Num.seqs=3 Similarity=0.703704
0 ACTCCTATAATAAAAANNA-TA
Rev.of_GCF_000225265.1_ASM22526v1_genomic.fna_1:4636885-4637044 Satlength=160 Nr of Repeats=6 RepeatLength=21 seed=TTATTATAGG Num.seqs=4 Similarity=0.592172 13
ACTCCTATAATAAAGACGG-TA
Rev.of_GCF_001591565.1_ASM159156v1_genomic.fna_136:5321-5468 Satlength=148 Nr of Repeats=4 RepeatLength=21 seed=TTTATTATAG Num.seqs=3 Similarity=0.585859 13
-CTCCTATAATAAAAATGANTA

************* * **
Consensus:

aCTCCTATAATAAAaAngaTA

>Bacillus_Fam_342_22_3 Nr. of seq. 3 Alignment length(with gaps) = 22
Alignment score = 0.636364
GCF_000307875.1_BABA1.0_genomic.fna_55:48119-48266 Satlength=148 Nr of Repeats=7 RepeatLength=21 seed=TTTGGCGGAA Num.seqs=7 Similarity=0.547619
0   TTTGGCGGAATTACCANTCNA-
Rev.of_GCF_001591665.1_ASM159166v1_genomic.fna_36:99463-99691 Satlength=229 Nr of Repeats=10 RepeatLength=21 seed=ATTCCGCCAA Num.seqs=9 Similarity=0.427298
11   TTTGGCGGAATT-CCAANNAAG
Rev.of_GCF_001591445.1_ASM159144v1_genomic.fna_22:36726-36903 Satlength=178 Nr of Repeats=6 RepeatLength=21 seed=ATTCCGCCAA Num.seqs=5 Similarity=0.479710   11
TTTGGCGGAATNNCTNNTNAAG

*********** *   *
Consensus:

TTTGGCGGAATtnCcantnaAg

>Bacillus_Fam_343_21_3 Nr. of seq. 3 Alignment length(with gaps) = 22
Alignment score = 0.737374
GCF_000752035.1_Bacillus_rubiinfantis_genomic.fna_11:292889-293098 Satlength=210 Nr of Repeats=9 RepeatLength=21 seed=ATACGGGAGT Num.seqs=8 Similarity=0.638975   0
ATACGAGAGTAATC-CACTCCT
GCF_000752035.1_Bacillus_rubiinfantis_genomic.fna_16:9832-10553 Satlength=722 Nr of Repeats=28 RepeatLength=21 seed=TTTACGAGAG Num.seqs=21 Similarity=0.773243
19   -TACGAGAGTAANCGCACTCTT
GCF_000752035.1_Bacillus_rubiinfantis_genomic.fna_33:58692-58944 Satlength=253 Nr of Repeats=12 RepeatLength=21 seed=TTACGGGAGT Num.seqs=12 Similarity=0.844156
20   -TACGGGAGTAACCGCACTCGT

**** ****** * ***** *
Consensus:

TACGaGAGTAAnCgCACTCnT

>Bacillus_Fam_344_21_3 Nr. of seq. 3 Alignment length(with gaps) = 22
Alignment score = 0.747475
GCF_001484965.1_ASM148496v1_genomic.fna_1:76098-76366 Satlength=269 Nr of Repeats=13 RepeatLength=21 seed=CTTGAGCGGT Num.seqs=9 Similarity=0.771886
0   CTTGAGCGGTATCTCCCCTCN-
Rev.of_GCF_001484965.1_ASM148496v1_genomic.fna_32:15969-16073 Satlength=105 Nr of Repeats=5 RepeatLength=21 seed=TACCGCTCAA Num.seqs=4 Similarity=0.773990   11
CTTGAGCGGTAT-TCTCCTCCG
GCF_001484965.1_ASM148496v1_genomic.fna_37:19197-19446 Satlength=250 Nr of Repeats=12 RepeatLength=21 seed=CTTGAGCGGT Num.seqs=9 Similarity=0.700176
21   CTTGAGCGGTAT-TCNGCTCCT

************ ** ***
Consensus:

CTTGAGCGGTATTCncCTCcn

>Bacillus_Fam_345_21_3 Nr. of seq. 3 Alignment length(with gaps) = 22
Alignment score = 0.727273
GCF_001578185.1_ASM157818v1_genomic.fna_1:3660606-3660924 Satlength=319
Nr of Repeats=13 RepeatLength=21 seed=ATGAACTGCT Num.seqs=8
Similarity=0.952381 0
ATGAACTGCTGCT-TTCGTCTG
GCF_002019665.1_ASM201966v1_genomic.fna_1:3758998-3759208 Satlength=211
Nr of Repeats=10 RepeatLength=21 seed=CTATTCGTCG Num.seqs=10
Similarity=0.957672 10
-TTAACCACTGCTATTCGTCGG
GCF_002019665.1_ASM201966v1_genomic.fna_1:3759021-3759394 Satlength=374
Nr of Repeats=18 RepeatLength=21 seed=ATTCGTCGGT Num.seqs=13
Similarity=0.766900 12
-TTAACCACTGCTATTCGTCGG

* *** ***** ****** *
Consensus:

TtAACcaCTGCTaTTCGTCgG

>Bacillus_Fam_346_21_3 Nr. of seq. 3 Alignment length(with gaps) = 22
Alignment score = 0.631313
GCF_900156865.1_PRJEB18960_genomic.fna_3:2989497-2989758 Satlength=262 Nr
of Repeats=8 RepeatLength=21 seed=TGTCCCATAT Num.seqs=5
Similarity=0.533333 0
TGTCCCATATAATNGAAGTTN-
Rev.of_GCF_900156865.1_PRJEB18960_genomic.fna_3:3020484-3021148
Satlength=665 Nr of Repeats=13 RepeatLength=21 seed=TTTATGGGAC
Num.seqs=10 Similarity=0.606280
11   TGTCCCATAAANTCCAAGTAN-
Rev.of_GCF_900156865.1_PRJEB18960_genomic.fna_3:3235322-3235665
Satlength=344 Nr of Repeats=9 RepeatLength=21 seed=TTTATGGGAC Num.seqs=7
Similarity=0.541667 11
TGTCCCATA-AATCGAATTNTN

********* * * ** *
Consensus:

TGTCCCATAnAaTcgAAgTnn

>Bacillus_Fam_347_18_3 Nr. of seq. 3 Alignment length(with gaps) = 21
Alignment score = 0.767196
GCF_000430785.1_ASM43078v1_genomic.fna_5:28845-28989 Satlength=145 Nr of
Repeats=8 RepeatLength=18 seed=CCAGGAGCAG Num.seqs=8 Similarity=0.941799
0   CCAGGAGCAGGCTTTGGC---
GCF_000430785.1_ASM43078v1_genomic.fna_5:28846-29137 Satlength=292 Nr of
Repeats=9 RepeatLength=18 seed=CAGGAGCAGG Num.seqs=8 Similarity=0.941799
1   CCAGGAGCAGGCTTTGGC---

GCF_001636345.1_ASM163634v1_genomic.fna_1:1975200-1975335 Satlength=136 Nr of Repeats=5 RepeatLength=21 seed=CAGGACCAGG Num.seqs=4 Similarity=0.788360 1
CCAGGACCAGGNTTTGGCACT

****** **** ******
Consensus:

CCAGGAgCAGGcTTTGGC

>Bacillus_Fam_348_20_3 Nr. of seq. 3 Alignment length(with gaps) = 21 Alignment score = 0.708995
GCF_001273755.1_ASM127375v1_genomic.fna_17:4571297-4571397 Satlength=101 Nr of Repeats=4 RepeatLength=20 seed=GTAGGACGAA Num.seqs=3 Similarity=0.717172 0
GTAGGACGAATTATTC-GCGT
Rev.of_GCF_001578185.1_ASM157818v1_genomic.fna_1:4465368-4465568 Satlength=201 Nr of Repeats=6 RepeatLength=20 seed=GCAAATATTT Num.seqs=4 Similarity=0.866667 18
GTAGGTCGAAATATTT-GCGT
GCF_001578185.1_ASM157818v1_genomic.fna_1:3658103-3658186 Satlength=84 Nr of Repeats=4 RepeatLength=21 seed=AGGACGAATT Num.seqs=3 Similarity=0.788360 2
GAAGGACGAATTATTTGGTGT

* *** **** **** * **
Consensus:

GtAGGaCGAAtTATTtGcGT

>Bacillus_Fam_349_20_3 Nr. of seq. 3 Alignment length(with gaps) = 21 Alignment score = 0.687831
GCF_001439915.1_ASM143991v1_genomic.fna_84:184831-184911 Satlength=81 Nr of Repeats=4 RepeatLength=20 seed=ACTGTCACTA Num.seqs=4 Similarity=0.646825
0 ACTGTCACT-ATCCGCAAAAT
GCF_001439915.1_ASM143991v1_genomic.fna_124:109637-109717 Satlength=81 Nr of Repeats=4 RepeatLength=20 seed=TACTGTCACT Num.seqs=4 Similarity=0.678571 19
ACTGTCACT-ATTCCNAAAAT
Rev.of_GCF_900156865.1_PRJEB18960_genomic.fna_3:2916495-2916575 Satlength=81 Nr of Repeats=4 RepeatLength=20 seed=AGTGGCAGTA Num.seqs=4 Similarity=0.629630 9
ACTGCCACTAATNTCTAAAAT

**** **** ** *****
Consensus:

ACTGtCACTATnccnAAAAT

>Bacillus_Fam_350_21_3 Nr. of seq. 3 Alignment length(with gaps) = 21 Alignment score = 0.687831

GCF_001591425.1_ASM159142v1_genomic.fna_4:87634-87736 Satlength=103 Nr of Repeats=5 RepeatLength=20 seed=TTTATTTGCG Num.seqs=3 Similarity=0.688889
0 TTTATTTGCGATAA-TACGCC
GCF_001591425.1_ASM159142v1_genomic.fna_176:0-79 Satlength=80 Nr of Repeats=4 RepeatLength=20 seed=TTTATTTGCG Num.seqs=4 Similarity=1.000000
0 TTTATTTGCGATAACTACGT-
Rev.of_GCF_001591425.1_ASM159142v1_genomic.fna_108:14449-14552 Satlength=104 Nr of Repeats=5 RepeatLength=21 seed=CGCAAATAAG Num.seqs=3 Similarity=0.915344 10
CTTATTTGCGATACCTTCATC

************ * *
Consensus:

tTTATTTGCGATAacTaCgtc

>Bacillus_Fam_351_21_3 Nr. of seq. 3 Alignment length(with gaps) = 21 Alignment score = 0.957672
GCF_000008505.1_ASM850v1_genomic.fna_1:3003849-3004059 Satlength=211 Nr of Repeats=10 RepeatLength=21 seed=GCCAACCACC Num.seqs=10 Similarity=0.887125 0
GCCAACCACCGTGTCCGCCTT
GCF_000290995.1_Baci_cere_AND1407_G13175_V1_genomic.fna_1:1982219-1982366 Satlength=148 Nr of Repeats=7 RepeatLength=21 seed=GCCAACCACC Num.seqs=7 Similarity=0.873016 0
GCCAACCACCGTGTCCGCCTT
Rev.of_GCF_001645555.1_ASM164555v1_genomic.fna_51:17837-18077 Satlength=241 Nr of Repeats=11 RepeatLength=21 seed=GGTGGTTGGC Num.seqs=10 Similarity=0.815168
10 GCCAACCACCGTGTCCACCTT

**************** ****
Consensus:

GCCAACCACCGTGTCCgCCTT

>Bacillus_Fam_352_21_3 Nr. of seq. 3 Alignment length(with gaps) = 21 Alignment score = 0.788360
GCF_000025825.1_ASM2582v1_genomic.fna_1:1391429-1391846 Satlength=418 Nr of Repeats=24 RepeatLength=21 seed=AGAACGGCGA Num.seqs=15 Similarity=0.824641 0
AGAACGGCGAGTACCGCGGGT
Rev.of_GCF_000225265.1_ASM22526v1_genomic.fna_1:3576340-3576466 Satlength=127 Nr of Repeats=5 RepeatLength=21 seed=CGTTCTACTC Num.seqs=4 Similarity=0.894180 6
AGAACGACGAGAGCGGCGAGT
GCF_000956595.1_ASM95659v1_genomic.fna_23:54484-54736 Satlength=253 Nr of Repeats=11 RepeatLength=21 seed=GAGAGCGGCG Num.seqs=10 Similarity=0.952028
8 AGAACGGCGAGAGCGGCGAGT

****** **** * *** **
Consensus:

AGAACGgCGAGagCgGCGaGT

>Bacillus_Fam_353_21_3 Nr. of seq. 3 Alignment length(with gaps) = 21
Alignment score = 0.661376
GCF_000813125.1_ASM81312v1_genomic.fna_62:25438-25563 Satlength=126 Nr of Repeats=5 RepeatLength=21 seed=ATTCCGCGAA Num.seqs=4 Similarity=0.513228
0   ATTCCGCGAAAACCGAGCGGA
Rev.of_GCF_000813125.1_ASM81312v1_genomic.fna_86:16754-16838 Satlength=85 Nr of Repeats=4 RepeatLength=21 seed=TTCGCGGAAT Num.seqs=4 Similarity=0.452899   10
ATTCCGCGAAATCTGAGNTTA
Rev.of_GCF_900104555.1_IMG-taxon_2651870172_annotated_assembly_genomic.fna_19:24896-25042 Satlength=147 Nr of Repeats=6 RepeatLength=21 seed=TTTCGCGGGA Num.seqs=5 Similarity=0.576389   11   ATCCCGCGAAANNAGAGTNGA

** ********   ***   *
Consensus:

ATtCCGCGAAAncnGAGnngA

>Bacillus_Fam_354_21_3 Nr. of seq. 3 Alignment length(with gaps) = 21
Alignment score = 0.756614
GCF_000948175.1_BFA_2_A_genomic.fna_17:13637-13721 Satlength=85 Nr of Repeats=4 RepeatLength=21 seed=AACCGAGAGA Num.seqs=4 Similarity=0.591435
0   AACCGAGAGAATAAGGTCNGA
Rev.of_GCF_000948175.1_BFA_2_A_genomic.fna_166:2351-2477 Satlength=127 Nr of Repeats=6 RepeatLength=21 seed=TCTCTCGGTT Num.seqs=6 Similarity=0.708081   10
AACCGAGAGATTAAGTGCTGA
Rev.of_GCF_000948175.1_BFA_2_A_genomic.fna_190:1516-1684 Satlength=169 Nr of Repeats=8 RepeatLength=21 seed=TCTCTCGGTT Num.seqs=8 Similarity=0.793651   10
AACCGAGAGATTAATGGCTGG

********** ***   * *
Consensus:

AACCGAGAGAtTAAgggCtGa

>Bacillus_Fam_355_21_3 Nr. of seq. 3 Alignment length(with gaps) = 21
Alignment score = 0.788360
GCF_001484965.1_ASM148496v1_genomic.fna_6:66041-66125 Satlength=85 Nr of Repeats=4 RepeatLength=21 seed=GATGTGAGAG Num.seqs=4 Similarity=0.765152
0   GATGTGAGAGAGAAGAGCGCG
Rev.of_GCF_001484965.1_ASM148496v1_genomic.fna_16:33130-33410 Satlength=281 Nr of Repeats=10 RepeatLength=21 seed=TCTCTCGCAT Num.seqs=6 Similarity=0.847619
11   GATGCGAGAGAGAAGAGCTCG
GCF_001484965.1_ASM148496v1_genomic.fna_26:28029-28279 Satlength=251 Nr of Repeats=10 RepeatLength=21 seed=GGATGTGAGC Num.seqs=7 Similarity=0.848828
20   GATGTGAGCAAGAAGGGCGCG

**** *** ***** ** **
Consensus:

GATGtGAGagAGAAGaGCgCG

>Bacillus_Fam_356_21_3 Nr. of seq. 3 Alignment length(with gaps) = 21
Alignment score = 0.671958
GCF_001591445.1_ASM159144v1_genomic.fna_22:68731-68836 Satlength=106 Nr of Repeats=5 RepeatLength=21 seed=ATTCCGCCAA Num.seqs=5 Similarity=0.476812
0   ATTCCGCCAANTTCGGNNTGN
GCF_001591445.1_ASM159144v1_genomic.fna_33:29-282 Satlength=254 Nr of Repeats=9 RepeatLength=21 seed=ATTCCGCCAA Num.seqs=8 Similarity=0.493271
0   ATTCCGCCAACTTNGGANNGN
Rev.of_GCF_001591445.1_ASM159144v1_genomic.fna_32:27469-27574 Satlength=106 Nr of Repeats=5 RepeatLength=21 seed=TTGGCGGAAT Num.seqs=5 Similarity=0.454167   10
ATTCCGCCAACTNTGGAAGNN

********** * **   *
Consensus:

ATTCCGCCAAcTtnGGanngN

>Bacillus_Fam_357_20_3 Nr. of seq. 3 Alignment length(with gaps) = 20
Alignment score = 0.711111
GCF_001439915.1_ASM143991v1_genomic.fna_105:322815-322955 Satlength=141 Nr of Repeats=7 RepeatLength=20 seed=GACGCAAATA Num.seqs=7 Similarity=0.525132   0
GACGCAAATATCGTGAAN--
GCF_001439915.1_ASM143991v1_genomic.fna_83:505700-505820 Satlength=121 Nr of Repeats=6 RepeatLength=20 seed=ACGCAAATAT Num.seqs=6 Similarity=0.600529   1
GACGCAAATATTGTGAAACT
GCF_001877785.1_PlanoSAMM_genomic.fna_117:26443-26710 Satlength=268 Nr of Repeats=5 RepeatLength=20 seed=GGCGCAATTA Num.seqs=3 Similarity=0.600000
20   GGCGCAATTATTATGAAACT

* ***** *** ****
Consensus:

GaCGCAAaTATtgTGAAact

>Bacillus_Fam_358_20_3 Nr. of seq. 3 Alignment length(with gaps) = 20
Alignment score = 0.877778
GCF_000374565.1_ASM37456v1_genomic.fna_5:154921-155692 Satlength=772 Nr of Repeats=32 RepeatLength=20 seed=CTCGTATAAA Num.seqs=21 Similarity=0.707937   0
CTCGTATAAAAGCTNCAACT
Rev.of_GCF_000374565.1_ASM37456v1_genomic.fna_7:0-79 Satlength=80 Nr of Repeats=4 RepeatLength=20 seed=TATGTGAGTT Num.seqs=4 Similarity=1.000000
6   CACATATAAAAGCTCCAACT

GCF_000374565.1_ASM37456v1_genomic.fna_33:0-79 Satlength=80 Nr of Repeats=4 RepeatLength=20 seed=TAAAAGCTCC Num.seqs=4 Similarity=1.000000
6 CACATATAAAAGCTCCAACT

* * ********** *****
Consensus:

CaCaTATAAAAGCTcCAACT

>Bacillus_Fam_359_20_3 Nr. of seq. 3 Alignment length(with gaps) = 20
Alignment score = 0.788889
GCF_000612665.1_FF4_genomic.fna_33:10627-10708 Satlength=82 Nr of Repeats=4 RepeatLength=20 seed=TATCGGGAAA Num.seqs=3 Similarity=0.629630
0 TATCGGGAAAANATTNAATA
Rev.of_GCF_000759675.1_ASM75967v1_genomic.fna_151:505712-505793 Satlength=82 Nr of Repeats=4 RepeatLength=20 seed=TTCCTGATAT Num.seqs=3 Similarity=0.644444 9
TATCAGGAAAAATTTCAATA
Rev.of_GCF_000759675.1_ASM75967v1_genomic.fna_153:106295-106375 Satlength=81 Nr of Repeats=4 RepeatLength=20 seed=CTGATATATT Num.seqs=4 Similarity=0.731481 26
TATCAGGAAAAAATNAAATA

**** ****** * ****
Consensus:

TATCaGGAAAAaaTtnAATA

>Bacillus_Fam_360_18_3 Nr. of seq. 3 Alignment length(with gaps) = 18
Alignment score = 0.703704
GCF_000175075.1_ASM17507v1_genomic.fna_6:61019-61091 Satlength=73 Nr of Repeats=4 RepeatLength=18 seed=GGCGGTCCTG Num.seqs=4 Similarity=0.962963
0 GGCGGTCCTGGCTTCGGT
GCF_002019665.1_ASM201966v1_genomic.fna_1:3587353-3587695 Satlength=343 Nr of Repeats=19 RepeatLength=18 seed=GGTGGACCAG Num.seqs=19 Similarity=0.984405 0
GGTGGACCAGGGTTCGGT
GCF_001648575.1_ASM164857v1_genomic.fna_62:119312-119492 Satlength=181 Nr of Repeats=10 RepeatLength=18 seed=TTCGGTGGTG Num.seqs=10 Similarity=0.795885 12
GGTGCACCGGGATTCGGT

** * ** ** ******
Consensus:

GGtGgaCCnGGnTTCGGT

>Bacillus_Fam_361_18_3 Nr. of seq. 3 Alignment length(with gaps) = 18
Alignment score = 0.746914
GCF_001439635.1_ASM143963v1_genomic.fna_162:551140-551212 Satlength=73 Nr of Repeats=4 RepeatLength=18 seed=TCAGCAGTAG Num.seqs=4 Similarity=0.802469 0
TCAGCAGTAGAATCAGAA

Rev.of_GCF_001439635.1_ASM143963v1_genomic.fna_174:69602-72152 Satlength=2551 Nr of Repeats=124 RepeatLength=18 seed=CTACTGCTGA Num.seqs=113 Similarity=0.785902
10 TCAGCAGTAGAGTCNGCG
Rev.of_GCF_001439635.1_ASM143963v1_genomic.fna_174:69602-72528 Satlength=2927 Nr of Repeats=142 RepeatLength=18 seed=CTACTGCTGA Num.seqs=129 Similarity=0.788123
10 TCAGCAGTAGCGTCNGCG

```
**********  ** *
```
Consensus:

TCAGCAGTAGagTCnGcg

>Bacillus_Fam_362_18_3 Nr. of seq. 3 Alignment length(with gaps) = 18 Alignment score = 0.679012
GCF_002019595.1_ASM201959v1_genomic.fna_1:3913094-3913256 Satlength=163 Nr of Repeats=9 RepeatLength=18 seed=TGCTCATGAT Num.seqs=9 Similarity=0.759259 0
TGCTCATGATGGTGGTCA
GCF_002019635.1_ASM201963v1_genomic.fna_2:1628719-1628809 Satlength=91 Nr of Repeats=5 RepeatLength=18 seed=TGCTCATGAT Num.seqs=5 Similarity=0.866667 0
TGCTCATGATGATGTTCA
GCF_002019645.1_ASM201964v1_genomic.fna_1:4893349-4893781 Satlength=433 Nr of Repeats=24 RepeatLength=18 seed=ATGTGAATGA Num.seqs=24 Similarity=0.927805 17
TGTGAATGATCGTGATCA

```
**   *****  ** ***
```
Consensus:

TGctcATGATggTGnTCA

>Bacillus_Fam_363_16_3 Nr. of seq. 3 Alignment length(with gaps) = 16 Alignment score = 0.722222
GCF_000177235.2_ASM17723v2_genomic.fna_1:1028903-1029101 Satlength=199 Nr of Repeats=5 RepeatLength=16 seed=AACCCGGGTT Num.seqs=3 Similarity=0.888889 0
AACCCGGGTTAAAGCG
GCF_000177235.2_ASM17723v2_genomic.fna_1:1630177-1630375 Satlength=199 Nr of Repeats=5 RepeatLength=16 seed=GAACCCGGGT Num.seqs=3 Similarity=0.944444 15
AACCCGGGTTAGTGGG
Rev.of_GCF_000177235.2_ASM17723v2_genomic.fna_1:3834979-3835177 Satlength=199 Nr of Repeats=5 RepeatLength=16 seed=GAACCCGGGT Num.seqs=3 Similarity=0.888889 11
AACCCGGGTTCAAGGT

```
**********   *
```
Consensus:

AACCCGGGTTaaaGgg

>Bacillus_Fam_364_15_3 Nr. of seq. 3 Alignment length(with gaps) = 15
Alignment score = 0.762963
GCF_000307875.1_BABA1.0_genomic.fna_140:27298-27463 Satlength=166 Nr of Repeats=11 RepeatLength=15 seed=GTATTTCTTC Num.seqs=11 Similarity=0.844849
0 GTATTTCTTCTTGAG
GCF_000706725.1_ASM70672v1_genomic.fna_1:3260718-3261542 Satlength=825 Nr of Repeats=4 RepeatLength=15 seed=CTTCTTTTGG Num.seqs=3 Similarity=1.000000 6
GTTTTTCTTCTTTTG
GCF_001591465.1_ASM159146v1_genomic.fna_27:2859-2976 Satlength=118 Nr of Repeats=7 RepeatLength=15 seed=GGTTTTTCTT Num.seqs=6 Similarity=0.774815
14 GTTTTTCTTCCTTAG

** ******* * *
Consensus:

GTtTTTCTTCtTtaG

>Bacillus_Fam_365_15_3 Nr. of seq. 3 Alignment length(with gaps) = 15
Alignment score = 0.837037
GCF_000504145.1_SOAPdenovo_for_version2.04_of_Bacillus_sp._FJAT-14515_genome_genomic.fna_28:32586-32916 Satlength=331 Nr of Repeats=22 RepeatLength=15 seed=TCTGTTTTTT Num.seqs=22 Similarity=0.810651 0
TCTGTTTTTTCTACN
GCF_000831065.1_ASM83106v1_genomic.fna_1:4247809-4248064 Satlength=256 Nr of Repeats=17 RepeatLength=15 seed=TCTGGTTTTT Num.seqs=17 Similarity=0.926797 0
TCTGGTTTTTCTACT
Rev.of_GCF_000832605.1_ASM83260v1_genomic.fna_1:1426778-1426853 Satlength=76 Nr of Repeats=5 RepeatLength=15 seed=CCAGAAGTAG Num.seqs=5 Similarity=0.893333 5
TCTGGTTTCTCTACT

**** *** *****
Consensus:

TCTGgTTTtTCTACt

>Bacillus_Fam_366_15_3 Nr. of seq. 3 Alignment length(with gaps) = 15
Alignment score = 0.755556
GCF_000706725.1_ASM70672v1_genomic.fna_1:1313522-1313585 Satlength=64 Nr of Repeats=4 RepeatLength=15 seed=TCTTCATCAG Num.seqs=3 Similarity=0.881481
0 TCTTCATCAGTTGTG
Rev.of_GCF_001315165.1_ASM131516v1_genomic.fna_16:18981-19722 Satlength=742 Nr of Repeats=28 RepeatLength=15 seed=CTGAAGAAGA Num.seqs=23 Similarity=0.787879
10 TCTTCTTCAGTNACG
Rev.of_GCF_001315165.1_ASM131516v1_genomic.fna_16:18981-20088 Satlength=1108 Nr of Repeats=33 RepeatLength=15 seed=CTGAAGAAGA Num.seqs=23 Similarity=0.787879
10 TCTTCTTCAGTNACG

***** *****   *
Consensus:

TCTTCtTCAGTnacG

>Bacillus_Fam_367_12_3 Nr. of seq. 3 Alignment length(with gaps) = 12
Alignment score = 0.675926
GCF_000832605.1_ASM83260v1_genomic.fna_1:4152867-4152911 Satlength=45 Nr of Repeats=4 RepeatLength=11 seed=TCCTTTGTTA Num.seqs=4 Similarity=1.000000
0   TCCTTTGTTAT-
Rev.of_GCF_000331575.1_ASM33157v1_genomic.fna_1:1646263-1646335 Satlength=73 Nr of Repeats=6 RepeatLength=12 seed=TAAGAAAATA Num.seqs=6 Similarity=0.940741   6
TTCTTAGTTATT
GCF_001274935.1_ASM127493v1_genomic.fna_42:123528-124032 Satlength=505 Nr of Repeats=4 RepeatLength=12 seed=TTTACTTTGT Num.seqs=3 Similarity=1.000000   10
TACTTTGTCATT

* *** ** **
Consensus:

TnCTTtGTtATt

>Bacillus_Fam_368_12_3 Nr. of seq. 3 Alignment length(with gaps) = 12
Alignment score = 0.666667
GCF_000007825.1_ASM782v1_genomic.fna_1:4742290-4742338 Satlength=49 Nr of Repeats=4 RepeatLength=12 seed=ATTGAAAAAG Num.seqs=4 Similarity=0.944444
0   ATTGAAAAAGGC
GCF_000007845.1_ASM784v1_genomic.fna_1:4598687-4598747 Satlength=61 Nr of Repeats=5 RepeatLength=12 seed=AAGGTATAGA Num.seqs=5 Similarity=0.933333
7   ATAGAACAAGGT
Rev.of_GCF_000374345.1_ASM37434v1_genomic.fna_7:48950-49010 Satlength=61 Nr of Repeats=4 RepeatLength=12 seed=CCTTCTTTTA Num.seqs=3 Similarity=0.650794   11
ATAAAAGAAGGT

** ** ****
Consensus:

ATagAAnAAGGt

>Bacillus_Fam_369_12_3 Nr. of seq. 3 Alignment length(with gaps) = 12
Alignment score = 0.777778
GCF_000196735.1_ASM19673v1_genomic.fna_1:2209405-2209513 Satlength=109 Nr of Repeats=9 RepeatLength=12 seed=GCTGCTGCAA Num.seqs=9 Similarity=0.944444   0
GCTGCTGCAACG
GCF_000204275.1_ASM20427v1_genomic.fna_1:2293892-2294000 Satlength=109 Nr of Repeats=9 RepeatLength=12 seed=GCTGCTGCAA Num.seqs=9 Similarity=0.944444   0
GCTGCTGCAACG

GCF_000321185.1_ASM32118v1_genomic.fna_45:7876-8053 Satlength=178 Nr of Repeats=8 RepeatLength=12 seed=CTACTGCTGC Num.seqs=5 Similarity=0.911111
7   GCTGCTACTACT

****** * **
Consensus:

GCTGCTgCaACg

>Bacillus_Fam_370_12_3 Nr. of seq. 3 Alignment length(with gaps) = 12
Alignment score = 0.888889
GCF_000401235.1_BacNeaAAU1_genomic.fna_107:6849-6933 Satlength=85 Nr of Repeats=6 RepeatLength=12 seed=CCAGGAGAAG Num.seqs=5 Similarity=0.955556
0   CCAGGAGAAGAA
Rev.of_GCF_001648575.1_ASM164857v1_genomic.fna_60:146867-146939
Satlength=73 Nr of Repeats=6 RepeatLength=12 seed=GGTTCTTCAC Num.seqs=6
Similarity=0.881481 2
CCAGGTGAAGAA
Rev.of_GCF_002019665.1_ASM201966v1_genomic.fna_1:4130554-4130638
Satlength=85 Nr of Repeats=7 RepeatLength=12 seed=CCTGGTTCTT Num.seqs=7
Similarity=0.873016 5
CCAGGCGAAGAA

***** ******
Consensus:

CCAGGnGAAGAA

>Bacillus_Fam_371_12_3 Nr. of seq. 3 Alignment length(with gaps) = 12
Alignment score = 0.777778
GCF_000615945.1_ASM61594v1_genomic.fna_89:34464-35479 Satlength=1016 Nr of Repeats=5 RepeatLength=12 seed=CACCAACACC Num.seqs=3
Similarity=1.000000
0   CACCAACACCAA
Rev.of_GCF_001307105.1_ASM130710v1_genomic.fna_12:24340-24400
Satlength=61 Nr of Repeats=5 RepeatLength=12 seed=TGTTGGTGCT Num.seqs=5
Similarity=1.000000 2
CAACAGCACCAA
GCF_002019645.1_ASM201964v1_genomic.fna_1:657555-657687 Satlength=133 Nr of Repeats=11 RepeatLength=12 seed=GCACCGACAC Num.seqs=11
Similarity=0.963636 5
CACCAGCACCGA

** ** **** *
Consensus:

CAcCAgCACCaA

>Bacillus_Fam_372_12_3 Nr. of seq. 3 Alignment length(with gaps) = 12
Alignment score = 1.000000
GCF_001273755.1_ASM127375v1_genomic.fna_17:544037-544637 Satlength=601 Nr of Repeats=50 RepeatLength=12 seed=CCGTAACCGC Num.seqs=50
Similarity=1.000000 0
CCGTAACCGCCG

GCF_001273755.1_ASM127375v1_genomic.fna_17:1499377-1499425 Satlength=49 Nr of Repeats=4 RepeatLength=12 seed=CCGTAACCGC Num.seqs=4 Similarity=1.000000 0
CCGTAACCGCCG
GCF_001584325.1_ASM158432v1_genomic.fna_41:69109-69169 Satlength=61 Nr of Repeats=5 RepeatLength=12 seed=CCGTAACCGC Num.seqs=5 Similarity=1.000000
0 CCGTAACCGCCG

****
Consensus:

CCGTAACCGCCG

>Bacillus_Fam_373_12_3 Nr. of seq. 3 Alignment length(with gaps) = 12 Alignment score = 0.777778
GCF_001375535.1_Bacillus_niameyensis_genomic.fna_6:1155105-1155231 Satlength=127 Nr of Repeats=8 RepeatLength=12 seed=TGGCCCTGAC Num.seqs=6 Similarity=0.844444 0
TGGCCCTGACCT
GCF_001591425.1_ASM159142v1_genomic.fna_5:37834-37894 Satlength=61 Nr of Repeats=4 RepeatLength=12 seed=CCTTGGCCTT Num.seqs=3 Similarity=0.925926
3 TGACCTTGGCCT
GCF_001591805.1_ASM159180v1_genomic.fna_7:139777-139831 Satlength=55 Nr of Repeats=4 RepeatLength=12 seed=TTGACCTTGA Num.seqs=3 Similarity=0.777778
5 TGACCTTGACCT

** ** ** ***
Consensus:

TGaCCtTGaCCT

>Bacillus_Fam_374_12_3 Nr. of seq. 3 Alignment length(with gaps) = 12 Alignment score = 0.777778
GCF_001591465.1_ASM159146v1_genomic.fna_69:93-309 Satlength=217 Nr of Repeats=17 RepeatLength=12 seed=GAAGCAGAAG Num.seqs=15 Similarity=0.835979
0 GAAGCAGAAGCA
Rev.of_GCF_001591465.1_ASM159146v1_genomic.fna_75:8-188 Satlength=181 Nr of Repeats=13 RepeatLength=12 seed=TCTGCTTCTG Num.seqs=9 Similarity=0.824074 2
GAAGCAGAAGCA
Rev.of_GCF_001654695.1_ASM165469v1_genomic.fna_3:76041-76089 Satlength=49 Nr of Repeats=4 RepeatLength=12 seed=TTCTGATTCA Num.seqs=4 Similarity=1.000000 3
GAAACTGAATCA

*** * *** **
Consensus:

GAAgCaGAAgCA

>Bacillus_Fam_375_240_2 Nr. of seq. 2 Alignment length(with gaps) = 240 Alignment score = 0.920833

GCF_001645705.1_ASM164570v1_genomic.fna_61:170578-171538 Satlength=961 Nr of Repeats=4 RepeatLength=240 seed=GGTTGGGCGA Num.seqs=4 Similarity=0.727375 0
GGTTGGGCGAAAGTGAATGTAAATGGGAAAACAGGATATGTAAGTAGTCAGTATTTAACAGGCAAGACGAACA
CAGCCCCANCAAAACCATCTACATCCACACCAGCAAAGACGGAAACAAAATATGTATCTGTTAACTCAGGTTC
TATTTTGAATCTACGCAGTAGTGCCAGTACAAGTGCATCAATAATCGGGAACTTATCAAATGGCACAGCGGTA
ACGGTTCAATCAGATTCCAAA
GCF_001645705.1_ASM164570v1_genomic.fna_61:170794-172355 Satlength=1562 Nr of Repeats=7 RepeatLength=240 seed=GTAACGGTTC Num.seqs=6 Similarity=0.815768 216
GGTTGGGCGAAAGTGACTGTAAATGGGAAAACAGGGTATGTAAGTAGTCAATATTTAACAGGCAAGACGAACA
CAGCCCCATCAAAACCGTCAACATCCACACCAGCAAAGACGGAAACAAAATATGTATCTGTTAACTCAGGTTC
TANTTTGAATCTACGCAGTAGTGCNAGTACAAGTGCTTCGATAATCGGGAGCCTAACAAATGGCACAGCAGTA
ACGGTTCAATCAGATTCCAAT

**************** ****************** **************
****************************** ******* **
****
********************* *********** ** ********** * ** *************
****
Consensus:

GGTTGGGCGAAAGTGAaTGTAAATGGGAAAACAGGaTATGTAAGTAGTCAaTATTTAACAGGCAAGACGAACA
CAGCCCCAtCAAAACCaTCaACATCCACACCAGCAAAGACGGAAACAAAATATGTATCTGTTAACTCAGGTTC
TAtTTTGAATCTACGCAGTAGTGCcAGTACAAGTGCaTCaATAATCGGGAaCcTAaCAAATGGCACAGCaGTA
ACGGTTCAATCAGATTCCAAa

>Bacillus_Fam_376_210_2 Nr. of seq. 2 Alignment length(with gaps) = 210 Alignment score = 0.853968
GCF_000612625.1_JCE_genomic.fna_21:59255-61145 Satlength=1891 Nr of Repeats=8 RepeatLength=210 seed=GGCTGGTACA Num.seqs=7 Similarity=0.674093
0
GGCTGGTACAGTGATGAAGCACGAACGAATTTGTACGATTTTAATGAGCCAGTNAAAGCAGCGACAAAGTTGT
ATGCGAAATGGACNCATAATCAATATGAAGTCAATTTTGATACNAATGGCGGCAGTNANATTGCTTCGGAAAA
TGTGAANTACAATGAAAAAGCAANGAAGCCANNNGNACCAACGAAAGAAGGGTACACCTTCGCN
GCF_000612625.1_JCE_genomic.fna_21:59327-62057 Satlength=2731 Nr of Repeats=13 RepeatLength=210 seed=TATGCGAAAT Num.seqs=13 Similarity=0.617769
72
GGCTGGTACAGTGATGAAGCACTAACGAATTTGTACGATTTTGANGNGCCAGTNAAAGCAGCNANGANNTTGT
ATGCGAAATGGACCCATAATCAATATGAAGTAAGTTTTGATACNAATGGCGGNAGTNANGTTGCTTCTGAAAA
TGTGAANTACAATGAAAAAGCNANGAAGCCAGNAGTACCAACGAAAGAAGGCTACACCTTCGCN

********************** ******************* * * *************** * *
***************** ***************** * ****************** ****** *******
************************** ********* * * *************** ************
Consensus:

GGCTGGTACAGTGATGAAGCACgAACGAATTTGTACGATTTTaAtGaGCCAGTNAAAGCAGCgAcaAagTTGT
ATGCGAAATGGACcCATAATCAATATGAAGTaAaTTTTGATACNAATGGCGGcAGTNANaTTGCTTCgGAAAA
TGTGAANTACAATGAAAAAGCaANGAAGCCAgNaGtACCAACGAAAGAAGGcTACACCTTCGCN

>Bacillus_Fam_377_200_2 Nr. of seq. 2 Alignment length(with gaps) = 200
Alignment score = 0.746667
GCF_001591665.1_ASM159166v1_genomic.fna_21:41016-42003 Satlength=988 Nr of Repeats=5 RepeatLength=198 seed=ATTGATAAAA Num.seqs=4 Similarity=0.606986
0
ATTGATAAAACAGCTCCAGTCGTAACTGGTGTTAGAANACAACGGTCTTTACAATAATGATGTAACAGTTTCA
TTTAATGAAGGAACAGCGACATTAAATGGTAAAGCAGTGAAAACTGGAACAGTAGTTAAAACAA-
ATGGAACTTACACAGTAGTGGTAACAGATGCAGCAGGAAACAAAACAACTGTGAAGTTCACG
GCF_001591665.1_ASM159166v1_genomic.fna_21:46294-47278 Satlength=985 Nr of Repeats=5 RepeatLength=198 seed=ATTGATAAAA Num.seqs=3 Similarity=0.493290
0 ATTGATAAAACNGCTCCAGTNGTAAGTGGAGTTA-
AAAACNACGGCCTNTACAATCAAGATGTGACANTTTCNTTTAATGAAGGAACNGCNACATTAAATGGTAANGA
ANTTNCATCTGGTACAGCAGTTAANCCAAGANGGANNCTATACANTAGTGGTAACCGACGCAGCAGGNAATAA
AACCACNGTTAAGTTCACN

*********** ******** **** *** **** ** ** **** ** ****** * ***** *** ****
************* ** ************* * * * * **** **** ****** *** * ***
** *** ********* ** ******** ** ***** ** ** ********
Consensus:

ATTGATAAAACaGCTCCAGTcGTAAcTGGaGTTAgAAaACaACGGcCTtTACAATaAaGATGTaACAgTTTCa
TTTAATGAAGGAACaGCgACATTAAATGGTAAaGaAgTgaaAaCTGGaACAGcAGTTAAaaCAAgAtGGAacc
TAcACAgTAGTGGTAACaGAcGCAGCAGGaAAcAAAACaACtGTgAAGTTCACg

>Bacillus_Fam_378_154_2 Nr. of seq. 2 Alignment length(with gaps) = 154
Alignment score = 0.696970
GCF_000177235.2_ASM17723v2_genomic.fna_1:694972-695711 Satlength=740 Nr of Repeats=5 RepeatLength=145 seed=ACATGTGGCA Num.seqs=3 Similarity=0.993870
0 ACATGTGGCATCATTTCTTTACCGCTTCTAAGAAAAACGAAAAACTCG-TT---T---
TTCGTTTTTCAT-AAAAGATATGAGAGT-
ATAAAATATGGCGGTGGAAATGAAGCCACGCGTAGTAGTGAGAAGAGCACTCACTACTACTTTAAAA
Rev.of_GCF_000177235.2_ASM17723v2_genomic.fna_1:2996165-2996769 Satlength=605 Nr of Repeats=4 RepeatLength=151 seed=CTAAAAACGA Num.seqs=4 Similarity=0.986755
69
ACATGTGGCATCACTTCTTCACCGCTTCTAAGAAAACCTAAAAACGAGCTTCACTAAGCTCGTTTTTAGTCTA
TTCATAAGA-AGTGCTAAAA-A-
GGCGGTGGAAGTGATGCCACGCGTAGTAGAGAGAAGAGCACTCACTACTACTTTAAAG

************* ***** *************** * ****** * ** * ******** * *
*** ** *** ***** * ********** *** *************
****
Consensus:

ACATGTGGCATCAcTTCTTcACCGCTTCTAAGAAAAaCgAAAAACgaGcTTcacTaagcTCGTTTTTaaTcaA
aacATAaGAgAGTgaTAAAAtAtGGCGGTGGAAaTGAaGCCACGCGTAGTAGaGAGAAGAGCACTCACTACTA
CTTTAAAa

>Bacillus_Fam_379_153_2 Nr. of seq. 2 Alignment length(with gaps) = 153
Alignment score = 0.644880

GCF_900111815.1_PRJEB17078_genomic.fna_1:368570-369663 Satlength=1094 Nr of Repeats=8 RepeatLength=150 seed=GATAAGTCAA Num.seqs=7 Similarity=0.990688 0
GATAAGTCAACATCGATTCGCTATCGCTCATCGTGTTTCCTTTATCTCGAT-CGAAACCCTCCAGGCCGTACAGCGCTAAACGGGCGCTTCCGCTTTTCTATTGTCCAGCTTCAGCGCCCA-GCGCTTATCGTCCTTCGGCTTTCTC-CCTAC
Rev.of_GCF_000171615.1_ASM17161v1_genomic.fna_137:4281-4859 Satlength=579 Nr of Repeats=4 RepeatLength=152 seed=AAGAAAAGCG Num.seqs=3 Similarity=0.740977 101
AATAAGTCAACATCGAATCGCTGACGCTCTTCGTGTTTCCTTTATTTGATGCCAAGCCCTCCAATCCATACGCCCCTAAACAGTCGGCTCCGCTTTTCT-TTGTCCAGCTCCGCCTCCTAGGGGCTAATGGATTTNCGGTCTTGTCACCTTC

************** ***** ***** ************** * *** * ** ******* ** *** * ****** * ** *********** ********* * * ** * * *** ** * * *** ** ** *** *
Consensus:

aATAAGTCAACATCGAaTCGCTaaCGCTCaTCGTGTTTCCTTTATcTcGATgCcAAaCCCTCCAagCCaTACacCcCTAAACaGgCGccTCCGCTTTTCTaTTGTCCAGCTcCacCgCCcAgGcGCTaATcGaccTtCGGccTTcTCaCCTaC

>Bacillus_Fam_380_148_2 Nr. of seq. 2 Alignment length(with gaps) = 148 Alignment score = 0.718468
GCF_000832605.1_ASM83260v1_genomic.fna_1:988424-988982 Satlength=559 Nr of Repeats=4 RepeatLength=147 seed=TAGATGAGGC Num.seqs=3 Similarity=0.724087
0
TAGATGAGGCGACAAAAGAAAAAGCGAAGTCCATTTTTGAGCAAGAAAAGTCCGGAACATTAACTCGTGAGCAAGCAAAAGAGAAATTGAAAGAACTAGGCGTGAAATTCCCAGAGAAAGGG-AAGCACGGTGATATGTTCGCGAATT
GCF_001645555.1_ASM164555v1_genomic.fna_23:264674-265262 Satlength=589 Nr of Repeats=4 RepeatLength=147 seed=AAAGAAAAAG Num.seqs=4 Similarity=0.756006 14
TAGACGAAGCAACAAAAGAAAAAGCGAAATCAATCTTAGAACAAGAAAAATCCGGCAAACTAACACGTGAACAAGCAAAAGAAGAATTAACAAAGCTAGGTGTGAAATTCCCGGAGAAAGGNTAAGCATGAAGACATGTTTGCTAACT

**** ** ** **************** ** ** ** ** ******** ***** * * **** ***** *********** **** * * * ***** *********** ******** ***** * ** ***** ** ** *
Consensus:

TAGAcGAaGCaACAAAAGAAAAAGCGAAaTCaATcTTaGAaCAAGAAAAaTCCGGaAaAcTAACaCGTGAaCAAGCAAAAGAaaAATTaAaAaAaCTAGGcGTGAAATTCCCaGAGAAAGGgtAAGCAcGaaGAcATGTTcGCgAAcT

>Bacillus_Fam_381_145_2 Nr. of seq. 2 Alignment length(with gaps) = 145 Alignment score = 0.671264
GCF_001591825.1_ASM159182v1_genomic.fna_6:118986-119562 Satlength=577 Nr of Repeats=4 RepeatLength=144 seed=CCACCATTTA Num.seqs=4 Similarity=0.524024 0
CCACCATTTATGCCACAGATTATCGTTGTTTCCACGGACAAAGGTATCGATCCGATTGGCTCCCCATGATAAGGCAGCCGG-AGAACCCTTCAACCCACGCCGCGGAGCTCCAAGGTCCTCCCACTCACTCCAGCTTGAACCGTC

GCF_900094975.1_IMG-
taxon_2617270721_annotated_assembly_genomic.fna_4:152867-153443
Satlength=577 Nr of Repeats=4 RepeatLength=144 seed=ACCATTTATG
Num.seqs=4 Similarity=0.507358 2
CCACCATTTATGCCACATCTGGTCGTTGTCTCCNCTGACAAATGTATCAATGCGGTTCGNCCCCCATGATAAA
GCTCCCGGTGGATCCCTNGAAACCGCCGCGCGGTGCCCCGAGATCTTCCCAATCGCTCCAGCGCGAACCATC

***************** * ******* *** * ****** ***** ** ** ** * ***********
** **** ** **** ** ** * ***** ** ** ** ** ***** ** ******* ***** **
Consensus:

CCACCATTTATGCCACAgaTgaTCGTTGTcTCCaCgGACAAAgGTATCaATcCGaTTcGccCCCCATGATAAa
GCacCCGGtaGAaCCCTtcAAaCCaCccCGCGGaGCcCCaAGaTCcTCCCAaTCaCTCCAGCgcGAACCaTC

>Bacillus_Fam_382_140_2 Nr. of seq. 2 Alignment length(with gaps) = 140
Alignment score = 0.814286
GCF_900107275.1_IMG-
taxon_2675903024_annotated_assembly_genomic.fna_46:1002-1551
Satlength=550 Nr of Repeats=4 RepeatLength=139 seed=ATCGTCCGAA
Num.seqs=3 Similarity=1.000000 0
ATCGTCCGAAAACTATATAACGCCGCGGGGTGGAGCAACGAGCAATGCATCAGTGAAAAAGCAACGAGTTGTC
TCGACGCATAAGCAGCGGAGTAAAGCAGGAAGAGGAAGAGACAGTCAAAGGACATCGAAGC-AGAGC
GCF_900107275.1_IMG-
taxon_2675903024_annotated_assembly_genomic.fna_19:46687-47426
Satlength=740 Nr of Repeats=6 RepeatLength=140 seed=AAGGACGTCG
Num.seqs=4 Similarity=0.972813 120
ATCGTCCGTAAACTTTAAAACGTCGCGGGGTGGAGCAACGAGCGAAGCATCATTGAAAAAACAGCGAGTTGTC
TCGACGCATTCGCATCGAAGCGAAGCAAGAAGAGGAAGAGACAGTCAAAGGACGTCGAAGCAAAAGC

******** ***** ** **** ******************* * ****** ******* **
***************** *** ** ** ***** ************************ ******* *
***
Consensus:

ATCGTCCGaAAACTaTAaAACGcCGCGGGGTGGAGCAACGAGCaAaGCATCAgTGAAAAAaCAaCGAGTTGTC
TCGACGCATaaGCAgCGaAGcaAAGCAaGAAGAGGAAGAGACAGTCAAAGGACaTCGAAGCaAaAGC

>Bacillus_Fam_383_138_2 Nr. of seq. 2 Alignment length(with gaps) = 138
Alignment score = 0.990338
GCF_001517115.1_ASM151711v1_genomic.fna_1:286089-286917 Satlength=829 Nr
of Repeats=6 RepeatLength=138 seed=GTTGTCTCGG Num.seqs=6
Similarity=0.875040 0
GTTGTCTCGGAAGCGAGGCTTCGTGAAGAGCAGGAAAATGAAGAGGAAGAAGCTGAGCGGTCAGCGCCATTGG
CTTTAAAAGCAGAGGAAAGCGGCGGTATAAAAGCAGAAGAAGCGCCAGCTGAGCCGCCAGAGCTT
GCF_001517135.1_ASM151713v1_genomic.fna_26:157017-157839 Satlength=823 Nr
of Repeats=6 RepeatLength=138 seed=GGCTTCGTGA Num.seqs=5
Similarity=0.884058 16
GTTGTCTCGGAAGCGAGGCTTCGTGAAGAGCAGGAAAATGAAGAGGAAGAAGCTGAGCGGTCAGCGCCATTGG
CTTTAAAAGCAGAGGAAAGCGGCGGTATAAAAGCAGAAGAAGCGCCAGCTGAGCCGCCGGAGCTT

****
** ******
Consensus:

GTTGTCTCGGAAGCGAGGCTTCGTGAAGAGCAGGAAAATGAAGAGGAAGAAGCTGAGCGGTCAGCGCCATTGG
CTTTAAAAGCAGAGGAAAGCGGCGGTATAAAAGCAGAAGAAGCGCCAGCTGAGCCGCCaGAGCTT

>Bacillus_Fam_384_128_2 Nr. of seq. 2 Alignment length(with gaps) = 128
Alignment score = 0.695312
GCF_001877785.1_PlanoSAMM_genomic.fna_85:203375-204000 Satlength=626 Nr of Repeats=5 RepeatLength=125 seed=AGCTCGAGGT Num.seqs=5 Similarity=0.978667
0 AGCTCGAGGTCATAAGCTAAACTTACCAAAAAGGCCAAGAGCGCCTTTCCGGTAAGT--TCATCTTATGCTTGTCGCTCCTGAGCGAGCCGCCTCCGCTTTTCT-GTTGTCCAGCTACGGCGGCTAGG
GCF_001274775.1_ASM127477v1_genomic.fna_3:67013-67766 Satlength=754 Nr of Repeats=6 RepeatLength=126 seed=CTTATGCTTG Num.seqs=4 Similarity=0.741602
62 CCCTCGAGGTCATAAGCCAAAAACCCCAAAAAGGCAAAGAACGCCTTTCCGG-GAGTCTTCGTCTTATGCTTGTCGCCTCAGAGCAACCCNCCTCCGCTTTTCTACATGTCCAGCTCCGGCGCGTTGA

*************** *** ********** **** *********** *** **
*************** * **** * ** ************* ********* ***** * *
Consensus:

acCTCGAGGTCATAAGCcAAAaacaCCAAAAAGGCaAAGAaCGCCTTTCCGGtaAGTctTCaTCTTATGCTTG
TCGCccCaGAGCaAcCCgCCTCCGCTTTTCTacaTGTCCAGCTaCGGCGccTaGa

>Bacillus_Fam_385_127_2 Nr. of seq. 2 Alignment length(with gaps) = 127
Alignment score = 0.616798
GCF_000812025.2_ASM81202v2_genomic.fna_49:98712-99319 Satlength=608 Nr of Repeats=5 RepeatLength=121 seed=TTCGCTTTTC Num.seqs=3 Similarity=0.963269
0 TTCGCTTTTCTCTTGTCCAGCTGCAGCGC-CTTGCTCCTCG-GGTCAAATAACCTTCCTCCATGAAGCCAAAGAGCGG-CTTCTTG-GC-GGAAGAACATTTGCCGGTCGGAGCAGAACAGGCG-CT
Rev.of_GCF_002019605.1_ASM201960v1_genomic.fna_1:2798720-2799338 Satlength=619 Nr of Repeats=5 RepeatLength=124 seed=AAGAAAAGCG Num.seqs=3 Similarity=0.863799
12 TCCGCTTTTCT-TTGTCTAGCTGCAG-GCTCTAGCCCCTCGAGGTTAAATAACCTACCTGAAAAAAGTCAAAAAGCGGACTT-TTGTTCAGTCAGAACATTTACTTGTCGGGGCTGACCAAGAGCCT

* ********* ***** ******** ** ** ** ***** *** ********* *** * *** ****
***** *** *** * * ********* * ***** ** ** ** * * **
Consensus:

TcCGCTTTTCTcTTGTCcAGCTGCAGcGCtCTaGCcCCTCGaGGTcAAATAACCTaCCTcaAaaAAGcCAAAa
AGCGGaCTTcTTGtgCaGgaAGAACATTTaCcgGTCGGaGCaGAaCAaGaGcCT

>Bacillus_Fam_386_127_2 Nr. of seq. 2 Alignment length(with gaps) = 127
Alignment score = 0.619423
GCF_000812025.2_ASM81202v2_genomic.fna_36:56533-57148 Satlength=616 Nr of Repeats=5 RepeatLength=123 seed=AGCTAGACAA Num.seqs=5 Similarity=0.666929
0 -AGCTAGACA-A-C-

GAGAAAAGCGGCGCCGACTGCTCTGGGGCGACAAGCATATGACCGGCTGGCGAAGCGGCGCTGTTTGCCGCAC
AGCCAGACGGACATATGACCTCGAGCCCCAAGGAGGCAA
Rev.of_GCF_000294775.2_ASM29477v2_genomic.fna_1:4325726-4326222
Satlength=497 Nr of Repeats=4 RepeatLength=124 seed=GCTGAACAGT
Num.seqs=4 Similarity=0.994624
41 CAGCTGGACATAGCAAAGAAAAGCGGAGGCGACTGTTCAGCCCCGACAAGCATAAGATGGGCT-
GTGGCGCGGCGCAGTTTGCCGCAGAG-CAGACCAGCTTATGACCTCGAGGGGCTAGGAGCCG-

**** **** * * ********** * ****** ** * *********** ** **** * *
******* ********** ** ***** * ************ * ***** *
Consensus:

cAGCTaGACAtAgCaaAGAAAAGCGGaGcCGACTGcTCaGcccCGACAAGCATAaGAccGGCTgGcGaaGCGG
CGCaGTTTGCCGCAcAGcCAGACcaaCaTATGACCTCGAGcccCaAGGAGcCaa

>Bacillus_Fam_387_127_2 Nr. of seq. 2 Alignment length(with gaps) = 127
Alignment score = 0.674541
GCF_001484965.1_ASM148496v1_genomic.fna_27:9418-9822 Satlength=405 Nr of
Repeats=4 RepeatLength=124 seed=GGGCGAAAAA Num.seqs=3
Similarity=0.992832
0 GGGCGAA-
AAATCTCGACGCTCCTACCCGCCGTAGCTAGACAAAGAAAAGCGAAGGCGGGTTGGTCAGGAGCGAGAATGT-
TGGAG-ACCTGACAACAGAAATCCCGGTTTTGGCTAACGCTTGCCA
Rev.of_GCF_001484965.1_ASM148496v1_genomic.fna_4:188320-189058
Satlength=739 Nr of Repeats=6 RepeatLength=123 seed=CGCCTCCGCT
Num.seqs=6 Similarity=0.712522
58
GGGTGAAGAAATCTCGACGCTCGTGGGCGCCGCAGCTGGATCAAGAAAAGCGGAGGCGGGTTGCACAGGAGCG
AGAATTTCTGGAGCGCCTGACAGATGAAGTCCCGCCCTTGGCTTTCG-TTGTCG

*** *** ************* * ***** **** ** ********** **********
************ * ***** ******* *** ***** ****** ** *** *
Consensus:

GGGcGAAgAAATCTCGACGCTCcTaccCGCCGcAGCTaGAcaAAGAAAAGCGaAGGCGGGTTGcaCAGGAGCG
AGAATgTcTGGAGcaCCTGACAaaaGAAaTCCCGcccTTGGCTaaCGcTTGcCa

>Bacillus_Fam_388_96_2 Nr. of seq. 2 Alignment length(with gaps) = 96
Alignment score = 1.000000
GCF_000009825.1_ASM982v1_genomic.fna_1:2905712-2906096 Satlength=385 Nr
of Repeats=4 RepeatLength=96 seed=GTTCGTTCGC Num.seqs=4
Similarity=0.810185
0
GTTCGTTCGCTTCTTCTTGGTCCATTTCGCCCGGCAACGCCTCGGTTCCACTCATTACCTCTGCCTCTTCAAT
CTCTGGTTCCCCGCGTCCATACC
GCF_900142675.1_IMG-
taxon_2654588196_annotated_assembly_genomic.fna_11:278699-279083
Satlength=385 Nr of Repeats=4 RepeatLength=96 seed=GTTCGTTCGC Num.seqs=4
Similarity=0.810185 0
GTTCGTTCGCTTCTTCTTGGTCCATTTCGCCCGGCAACGCCTCGGTTCCACTCATTACCTCTGCCTCTTCAAT
CTCTGGTTCCCCGCGTCCATACC

****
****

Consensus:

GTTCGTTCGCTTCTTCTTGGTCCATTTCGCCCGGCAACGCCTCGGTTCCACTCATTACCTCTGCCTCTTCAAT
CTCTGGTTCCCCGCGTCCATACC

>Bacillus_Fam_389_84_2 Nr. of seq. 2 Alignment length(with gaps) = 84
Alignment score = 0.603175
GCF_000429725.1_ASM42972v1_genomic.fna_2:377721-378441 Satlength=721 Nr of Repeats=5 RepeatLength=83 seed=TGGCGAGCTT Num.seqs=3
Similarity=0.828648
0 TGGCGAGCTTTCCGCT-
CGGGGCGTCGCGGTTCGTCCCGTACCTCGACGTACCTCTCGCCTCCAGTGGCGAGGCGGTCCCCATG
Rev.of_GCF_000429725.1_ASM42972v1_genomic.fna_25:9598-10275 Satlength=678
Nr of Repeats=5 RepeatLength=84 seed=CTCGCCAAAC Num.seqs=3
Similarity=0.813953 7
TGGCGAGGTTGGGGCNGCGAGTCGACGAGGTTGGGAGCATACCTCGACGTACCTCTNCCTTCCAGTGGCGAGG
TAGCGACCGTT

******* ** ** ** * ** ** **** * * **************** * *************
* ** *
Consensus:

TGGCGAGcTTgccGCtgCGaGgCGaCGaGGTTcGgacCaTACCTCGACGTACCTCTccCcTCCAGTGGCGAGG
caGccaCCaTg

>Bacillus_Fam_390_84_2 Nr. of seq. 2 Alignment length(with gaps) = 84
Alignment score = 0.904762
GCF_000496285.1_ASM49628v1_genomic.fna_1:3671784-3672105 Satlength=322 Nr of Repeats=4 RepeatLength=84 seed=GGAAAAGTAA Num.seqs=3
Similarity=0.904762 0
GGAAAAGTAACAGCAGGTTTAAATACGTTAAATGGCAAAACGGGTGAGATGCAGACTGGTATAGGGAAATTAG
TAGACGGTTCA
Rev.of_GCF_000775975.1_ASM77597v1_genomic.fna_1:3704249-3704585
Satlength=337 Nr of Repeats=4 RepeatLength=84 seed=TTTGCTATTT Num.seqs=4
Similarity=0.846561 39
GGAAAAGTAACAGCTGGTTTAAATACGTTAAATAGCAAAACAGGTGAGATGCAGACTGGTATAGGGAAATTAG
TAGATGGGTCT

************** ***************** *******
*********************************** ** **
Consensus:

GGAAAAGTAACAGCaGGTTTAAATACGTTAAATaGCAAAACaGGTGAGATGCAGACTGGTATAGGGAAATTAG
TAGAcGGgTCa

>Bacillus_Fam_391_78_2 Nr. of seq. 2 Alignment length(with gaps) = 78
Alignment score = 0.914530
GCF_000245335.1_ASM24533v1_genomic.fna_3:543340-543808 Satlength=469 Nr of Repeats=6 RepeatLength=78 seed=GAAGATCTTG Num.seqs=6
Similarity=0.949858
0
GAAGATCTTGGTGACCGATATGATTTGGAAGAACTTTTGGAATCTGATGAACGATCATATGTTGACGATTGAT
AATCT

GCF_001517115.1_ASM151711v1_genomic.fna_24:533324-533636 Satlength=313 Nr of Repeats=4 RepeatLength=78 seed=CTGAAGATCT Num.seqs=4 Similarity=0.900285 76
GAAGATCTTGATGACCGATATGATTTGGACGAACTTTTGGTGTCTGATGAACGATCATATTTTGACGATTGATAATCT

********** ***************** ********** *****************
****
Consensus:

GAAGATCTTGaTGACCGATATGATTTGGAaGAACTTTTGGaaTCTGATGAACGATCATATgTTGACGATTGATAATCT

>Bacillus_Fam_392_75_2 Nr. of seq. 2 Alignment length(with gaps) = 75 Alignment score = 0.951111
GCF_000217835.1_ASM21783v1_genomic.fna_1:1910042-1910492 Satlength=451 Nr of Repeats=6 RepeatLength=75 seed=CTTTCCGCCA Num.seqs=6 Similarity=0.732148 0
CTTTCCGCCAACGATGCCGACTCATTTTTACCAGAAAACTCCGGNTCGGCCTGCCATTCATCCTGCGCCACTCCA
GCF_000832905.1_ASM83290v1_genomic.fna_1:924148-924448 Satlength=301 Nr of Repeats=4 RepeatLength=75 seed=CTTTCCGCCA Num.seqs=4 Similarity=0.694815
0
CTTTCCGCCAACGATGCCGACTCATTTTTACCAGGAAACTCCGGNTCGGCCTGCCATTCATCCTGCACCACTCCA

********************************* ******************************
****
Consensus:

CTTTCCGCCAACGATGCCGACTCATTTTTACCAGaAAACTCCGGNTCGGCCTGCCATTCATCCTGCaCCACTCCA

>Bacillus_Fam_393_74_2 Nr. of seq. 2 Alignment length(with gaps) = 74 Alignment score = 0.689189
GCF_000299035.1_ASM29903v1_genomic.fna_4:316574-316862 Satlength=289 Nr of Repeats=4 RepeatLength=72 seed=TTCTGCTGCT Num.seqs=4 Similarity=0.969136
0
TTCTGCTGCTTGTCTTCTGTTTGGCTGTTTGTTGCCTTTTCATCAGCAGAACCATTTGTTGATTTCTCTTCC--
GCF_000712615.1_ASM71261v1_genomic.fna_2:364691-365123 Satlength=433 Nr of Repeats=6 RepeatLength=72 seed=TCTTTTTGAC Num.seqs=6 Similarity=0.987654
67 TTGACCT--
TTGTCATCAGTTTTACTGTTTGTTGCTTTTTCCTCAGTAGAACCGTTCGTTGATTTCTCTTCTTT

** ** ***** ** **** *********** ***** **** ****** ** **************
Consensus:

TTcacCTgcTTGTCaTCaGTTTgaCTGTTTGTTGCcTTTTCaTCAGcAGAACCaTTcGTTGATTTCTCTTCctt

>Bacillus_Fam_394_67_2 Nr. of seq. 2 Alignment length(with gaps) = 67
Alignment score = 0.611940
GCF_000787375.1_ASM78737v1_genomic.fna_58:8-403 Satlength=396 Nr of Repeats=6 RepeatLength=66 seed=GTCATATCTC Num.seqs=5 Similarity=0.538889
0 GTCATATCTCCATCTAATCCGTTGCTGTGATATGATATNAAT--C-GTNTATTTCTGNATNNGNTAT
Rev.of_BACILLUS_PSEUDALCALIPHILUS_genomic.fna_2:420902-424028 Satlength=3127 Nr of Repeats=37 RepeatLength=66 seed=TCACAGCAAC Num.seqs=34 Similarity=0.260606
30 GTCATATCTCCATCTATTCCGTTGCTGTGATATGAT-NNAATGNCNNTATCNNNCNNTATNCNTNAN

```
*************** ******************  ****  *  * *    *   ***    *
```

Consensus:

GTCATATCTCCATCTAaTCCGTTGCTGTGATATGATatNAATgnCngTaTatttCtgtATNcgttAt

>Bacillus_Fam_395_66_2 Nr. of seq. 2 Alignment length(with gaps) = 66
Alignment score = 0.626263
GCF_000712595.1_ASM71259v1_genomic.fna_18:68568-69048 Satlength=481 Nr of Repeats=8 RepeatLength=60 seed=TGGTACTACT Num.seqs=8 Similarity=0.619243
0 TGGTACTACTT----GAATNA--TAGCGGTGCAATGCAAACAGGTTGGTTGTATGACGATGNAAAC
GCF_000161455.1_ASM16145v1_genomic.fna_1:1154071-1154599 Satlength=529 Nr of Repeats=10 RepeatLength=66 seed=TGAAAACAGG Num.seqs=6 Similarity=0.797678 34
TGGTATTACTTGGATGAAGCAGCTAGCGGAGCAATGAAAACAGGTTGGTTGAAATACGGAGAAAAT

```
***** *****    ***  *  ****** ****** ************* *  ***  * ***
```

Consensus:

TGGTAcTACTTggatGAAgcAgcTAGCGGaGCAATGaAAACAGGTTGGTTGaAagACGaaGaAAAc

>Bacillus_Fam_396_62_2 Nr. of seq. 2 Alignment length(with gaps) = 62
Alignment score = 0.618280
GCF_000712595.1_ASM71259v1_genomic.fna_8:140638-141178 Satlength=541 Nr of Repeats=9 RepeatLength=60 seed=GGTGTAATGC Num.seqs=9 Similarity=0.730747
0 GGTGTAATGCAAACAGGTTGGCACAAAGAT-GG-AGGAAGTTGGTACTACTTTGGCNCAAAC
Rev.of_GCF_000752035.1_Bacillus_rubiinfantis_genomic.fna_11:343119-343839 Satlength=721 Nr of Repeats=8 RepeatLength=60 seed=CCAGCCGGTT Num.seqs=7 Similarity=0.904762 21
GGTGTAATGCAAACCGGCTGGGA-AAAGATCGGCAGCAAG-AAGTATTACTTCGACAAAAAT

```
************** ** *** * ****** ** ** ***   *** ***** * *  ***
```

Consensus:

GGTGTAATGCAAACaGGcTGGcAcAAAGATcGGcAGcAAGtaaGTAcTACTTcGaCaaAAAc

>Bacillus_Fam_397_61_2 Nr. of seq. 2 Alignment length(with gaps) = 61
Alignment score = 0.661202
GCF_001591805.1_ASM159180v1_genomic.fna_17:178840-179140 Satlength=301 Nr of Repeats=4 RepeatLength=60 seed=TGGTGCGATG Num.seqs=3 Similarity=0.589065 0
TGGTGCGATGAAAACNGGCTGGGT-TAAACCTGGCTCCACTTGGTATTACTTAGATAAAAG
GCF_001591805.1_ASM159180v1_genomic.fna_46:18986-19349 Satlength=364 Nr of Repeats=5 RepeatLength=60 seed=TGGTATTACT Num.seqs=3

Similarity=0.608379
40   TGGTGCGATGAAGACNGGTTGGTTNTTAA-CTGGTGNGAAATGGTATTACTTAGAAAAAAA

************ ***** *** * * ** ****   * ************** ****
Consensus:

TGGTGCGATGAAaACNGGcTGGgTnTaAAcCTGGcgccAaaTGGTATTACTTAGAaAAAAa

>Bacillus_Fam_398_60_2 Nr. of seq. 2 Alignment length(with gaps) = 60
Alignment score = 0.911111
GCF_000508325.1_BAVI_1_genomic.fna_45:24041-24284 Satlength=244 Nr of Repeats=4 RepeatLength=60 seed=AAATGGTACT Num.seqs=3 Similarity=0.573477
0   AAATGGTACTATCTTAACGCAAACGGCGACATGGCAACAGGCTGGAAGCTGGTTAGCGGA
GCF_001591805.1_ASM159180v1_genomic.fna_13:17036-17279 Satlength=244 Nr of Repeats=4 RepeatLength=60 seed=AAATGGTACT Num.seqs=3
Similarity=0.585185
0   AAATGGTACTACCTTAACGCAAACGGCGAAATGGCAACAGGCTGGAAACTGGTTAGCGGC

*********** **************** **************** ***********
Consensus:

AAATGGTACTAcCTTAACGCAAACGGCGAaATGGCAACAGGCTGGAAaCTGGTTAGCGGa

>Bacillus_Fam_399_60_2 Nr. of seq. 2 Alignment length(with gaps) = 60
Alignment score = 0.811111
GCF_000508325.1_BAVI_1_genomic.fna_65:18797-19643 Satlength=847 Nr of Repeats=6 RepeatLength=60 seed=AAATGGTACT Num.seqs=4 Similarity=0.637978
0   AAATGGTACTACCTCCAAGCTGACGGCGCNATGAAAACCGGCTGGCTTAGCACAAATAAC
Rev.of_GCF_001591805.1_ASM159180v1_genomic.fna_18:13670-14516
Satlength=847 Nr of Repeats=4 RepeatLength=60 seed=AGTACCATTT Num.seqs=3
Similarity=0.683060   10
AAATGGTACTTCCTNCAGGCTGATGGTTCNATGAAAACCGGCTGGCTGAGCACAAACAAC

********** *** ** ***** ** ****************** ******** ***
Consensus:

AAATGGTACTaCCTcCAaGCTGAcGGcgCNATGAAAACCGGCTGGCTgAGCACAAAcAAC

>Bacillus_Fam_400_60_2 Nr. of seq. 2 Alignment length(with gaps) = 60
Alignment score = 0.716667
GCF_000712595.1_ASM71259v1_genomic.fna_1:423888-424608 Satlength=721 Nr of Repeats=12 RepeatLength=60 seed=TATTCATGTA Num.seqs=12
Similarity=0.898990   0
TATTCATGTAATACCATTTGCCTCCGAATTCTANCCAGCCTGTTTGCATTACTCCGGAAC
GCF_000712595.1_ASM71259v1_genomic.fna_21:53926-54706 Satlength=781 Nr of Repeats=13 RepeatLength=60 seed=TAATACCACT Num.seqs=13
Similarity=0.900570
8   TATTTAAGTAATACCACTTACCCCCAACAGATAACCAGCCTGTTTGCATTTCTCCTGAAC

**** * ********* ** ** ** *   ** *************** **** ****
Consensus:

TATTcAaGTAATACCAcTTaCCcCCaAaagaTAaCCAGCCTGTTTGCATTaCTCCgGAAC

>Bacillus_Fam_401_60_2 Nr. of seq. 2 Alignment length(with gaps) = 60
Alignment score = 0.688889
GCF_002019645.1_ASM201964v1_genomic.fna_1:5253347-5253767 Satlength=421
Nr of Repeats=7 RepeatLength=60 seed=CCTGGTAGTG Num.seqs=7
Similarity=0.912169 0
CCTGGTAGTGGACCTTCGAAATTCGGTGGGTATGGTTGGTTCCACTCTCCACCGTTTGGA
GCF_900104555.1_IMG-
taxon_2651870172_annotated_assembly_genomic.fna_12:81185-81485
Satlength=301 Nr of Repeats=5 RepeatLength=60 seed=CTCCTCCATT Num.seqs=5
Similarity=0.964444 46
CCTGGTAATGGGCCCTCAAAATTCGGTGGATATGGGAAATTCCAACCTCCTCCATTTGGG

******* *** ** ** *********** ***** ***** **** ** *****
Consensus:

CCTGGTAaTGGaCCcTCaAAATTCGGTGGaTATGGgaaaTTCCAacCTCCaCCaTTTGGa

>Bacillus_Fam_402_57_2 Nr. of seq. 2 Alignment length(with gaps) = 57
Alignment score = 0.625731
GCF_000186145.1_Bacillus_sp_2_A_57_CT2_V1_genomic.fna_2:649324-649688
Satlength=365 Nr of Repeats=7 RepeatLength=52 seed=GGCTTCATGA Num.seqs=7
Similarity=0.749326 0 -
GGCTTCATGAAGC-NTTCATGGCGACC-TTTTC--CCGGCTTCCCTTCTGTTTTTG
GCF_000186145.1_Bacillus_sp_2_A_57_CT2_V1_genomic.fna_6:279533-280000
Satlength=468 Nr of Repeats=9 RepeatLength=52 seed=CATGGCGACC Num.seqs=8
Similarity=0.767196 18
CGGCTTCATGAAGCACTNCATGGCGACCGTTTTCAGCC--CTACTCNTCGATTTTT-

************* * ********** ***** ** ** * * ** *****
Consensus:

cGGCTTCATGAAGCacTtCATGGCGACCgTTTTCagCCggCTaCcCtTCgaTTTTTg

>Bacillus_Fam_403_57_2 Nr. of seq. 2 Alignment length(with gaps) = 57
Alignment score = 0.605263
GCF_900156875.1_PRJEB18969_genomic.fna_10:1559594-1559802 Satlength=209
Nr of Repeats=4 RepeatLength=52 seed=AGATGAAGAC Num.seqs=4
Similarity=0.790598 0
AGATGAAGACCG-TTTATGTACCGGA---AGGCTTGAGGAAAGGTTTTCATAGC-AG
Rev.of_GCF_900156875.1_PRJEB18969_genomic.fna_11:1462191-1462503
Satlength=313 Nr of Repeats=6 RepeatLength=52 seed=CGGTCTTCAT Num.seqs=6
Similarity=0.825786 11 -
GATGAAGACCGCTTTA-GCATTGGATCCA-GC-AGAGGAAAGGTTCTCATAGCGAG

*********** **** * * *** * ** *********** ******* **
Consensus:

aGATGAAGACCGcTTTAtGcAccGGAtccAgGCtaGAGGAAAGGTTcTCATAGCgAG

>Bacillus_Fam_404_57_2 Nr. of seq. 2 Alignment length(with gaps) = 57
Alignment score = 0.684211
GCF_900109925.1_IMG-
taxon_2651870116_annotated_assembly_genomic.fna_26:134607-134815
Satlength=209 Nr of Repeats=4 RepeatLength=53 seed=AGGAAGAAAG Num.seqs=3

Similarity=0.882600 68 AGTGGGAAGAGTCTG----
AGGAAGAAAGGTCACCAACAAGGCCCATTGGTGCCCAA
Rev.of_GCF_900109925.1_IMG-
taxon_2651870116_annotated_assembly_genomic.fna_36:66229-66446
Satlength=218 Nr of Repeats=4 RepeatLength=53 seed=ATTGGTGACC Num.seqs=3
Similarity=0.773663 87 AGT---AAGTGT-
TGCTAGAACAAGAAAGGTCACCAATAAGGCTCATTGGTGCCCAA

* *************** ***** **************** *** ** **
Consensus:

AacAAGAAAGGTCACCAAcAAGGCcCATTGGTGCCCAAAGTgggAAGaGTcTGctag

>Bacillus_Fam_405_56_2 Nr. of seq. 2 Alignment length(with gaps) = 56
Alignment score = 0.636905
GCF_000175075.1_ASM17507v1_genomic.fna_2:12013-12273 Satlength=261 Nr of
Repeats=5 RepeatLength=52 seed=ATGAAGACCT Num.seqs=5 Similarity=0.897436
0 ATGAAGACCTGTCCATGGCGGAA----AAGTTGGAAGAGATGTCTTCATAAGGTGG
GCF_900156865.1_PRJEB18960_genomic.fna_3:551238-551654 Satlength=417 Nr
of Repeats=8 RepeatLength=52 seed=ATGAAGACCT Num.seqs=8
Similarity=0.723270
0 ATGAAGACCTG--AANGGAGGAATTTGTAGTAGGAAGAG--GTCATCATAAGGTGG

*********** * ** **** *** ******* *** ***********
Consensus:

ATGAAGACCTGtcaAtGGaGGAAtttgaAGTaGGAAGAGatGTCaTCATAAGGTGG

>Bacillus_Fam_406_56_2 Nr. of seq. 2 Alignment length(with gaps) = 56
Alignment score = 0.622024
GCF_000175075.1_ASM17507v1_genomic.fna_4:76487-76903 Satlength=417 Nr of
Repeats=8 RepeatLength=52 seed=TCTCTGTTAC Num.seqs=8 Similarity=0.834249
0 TCTCTGTTACCGTTTTTCCTGTTT---GAATCGCAGTTCGGGCACTGAGACCGCC-
Rev.of_GCF_000175075.1_ASM17507v1_genomic.fna_32:624889-625097
Satlength=209 Nr of Repeats=4 RepeatLength=52 seed=GTCTCTGTGC Num.seqs=4
Similarity=0.868973 48
TCTCTCTTACCG-TTTTCCTTTTTCCCGAA--GTCTTTCGGGCACAGAGA-CGCCT

***** ****** ******* *** *** * ********* **** ****
Consensus:

TCTCTcTTACCGtTTTTCCTgTTTcccGAAtcGcagTTCGGGCACaGAGAcCGCCt

>Bacillus_Fam_407_56_2 Nr. of seq. 2 Alignment length(with gaps) = 56
Alignment score = 0.601190
GCF_001636345.1_ASM163634v1_genomic.fna_1:2699397-2699657 Satlength=261
Nr of Repeats=5 RepeatLength=52 seed=GTTCCCTTCA Num.seqs=5
Similarity=0.744654 0
GTTCCCTTCAGGCTC--CCTTTTCTCTGCTGACGGGCACTTTC-GC-CACAACTTA
Rev.of_GCF_001636345.1_ASM163634v1_genomic.fna_1:1077933-1078141
Satlength=209 Nr of Repeats=4 RepeatLength=52 seed=AAGTGCCCGT Num.seqs=4
Similarity=0.485867 39
GTTCCCGTCA-GCTCTGAGTTTTCNCT-CTGACGGGCACTTCCTGCTNNCACTTTA

****** *** ****   ***** ** ************ * **   ** ***
Consensus:

GTTCCCgTCAgGCTCtgacTTTTCtCTgCTGACGGGCACTTcCtGCtcaCAacTTA

>Bacillus_Fam_408_56_2 Nr. of seq. 2 Alignment length(with gaps) = 56
Alignment score = 0.669643
GCF_002019645.1_ASM201964v1_genomic.fna_1:3948839-3949826 Satlength=988
Nr of Repeats=19 RepeatLength=52 seed=ACTTCGGACA Num.seqs=18
Similarity=0.865091   86
TTGTTTTAGAGC---AAAGCTGTCTGAACTCTGGCCT-ACTTCGGACAGCTTTAGC
GCF_002019645.1_ASM201964v1_genomic.fna_1:4298062-4299581 Satlength=1520
Nr of Repeats=20 RepeatLength=52 seed=CTCTCTTCGG Num.seqs=18
Similarity=0.844469   136
-TCTTTCAG-GCTTCAAAGCTGTCTGAAC-CTGGACTCTCTTCGGACAGCTTTGGC

************** ** * *** ** **   ************** **** **
Consensus:

aCTTCGGACAGCTTTaGCtTcTTTcAGaGCttcAAAGCTGTCTGAACtCTGGaCTc

>Bacillus_Fam_409_56_2 Nr. of seq. 2 Alignment length(with gaps) = 56
Alignment score = 0.625000
GCF_900104555.1_IMG-
taxon_2651870172_annotated_assembly_genomic.fna_16:18479-18947
Satlength=469 Nr of Repeats=9 RepeatLength=52 seed=TTTGTTCAAT Num.seqs=9
Similarity=0.798567   0   TTTGTTCAATAGACAG-
CTTCTATCGGACAAACTCTAT-GATNC--GCCTATCCGT
Rev.of_GCF_900104555.1_IMG-
taxon_2651870172_annotated_assembly_genomic.fna_25:10104-10364
Satlength=261 Nr of Repeats=5 RepeatLength=52 seed=AATAGAGGTC Num.seqs=5
Similarity=0.825641   24   TTTGTTCAATAGA-
AGACCTCTATTGGACAAA--CTATCGATTCAAG-CTATGTGT

************* ** * ***** ******* **** *** * * **** **
Consensus:

TTTGTTCAATAGAcAGaCcTCTATcGGACAAActCTATcGATtCaaGcCTATccGT

>Bacillus_Fam_410_56_2 Nr. of seq. 2 Alignment length(with gaps) = 56
Alignment score = 0.601190
GCF_000181495.1_ASM18149v1_genomic.fna_164:6846-7219 Satlength=374 Nr of
Repeats=7 RepeatLength=53 seed=TTTAGAGGTT Num.seqs=5 Similarity=0.816364
0   TTTAGAGGTTGCCGATTGAAGGTCGTG-GAGGAATC-GAGGTAGTCAAAGAG-GCC
GCF_000181495.1_ASM18149v1_genomic.fna_186:321552-322293 Satlength=742 Nr
of Repeats=14 RepeatLength=53 seed=GGTTGTCGAT Num.seqs=13
Similarity=0.775087   5
TAT-GGGGTTGTCGATTGGNTGT-TTGAGAGGAATCGGTGGT-GTCAAAGAGAGCC

* * * ***** ******   ** ** ******** * *** ********* ***
Consensus:

TaTaGaGGTTGcCGATTGaagGTcgTGaGAGGAATCgGaGGTaGTCAAAGAGaGCC

>Bacillus_Fam_411_55_2 Nr. of seq. 2 Alignment length(with gaps) = 55
Alignment score = 0.654545
GCF_000181495.1_ASM18149v1_genomic.fna_57:492-700 Satlength=209 Nr of Repeats=4 RepeatLength=52 seed=CTCAAGACAC Num.seqs=4 Similarity=0.743187
0   CTCAAGACACTTTCCTCGGC--ATTTTACCCGGTTTATGTCCTTGAGA-ACCTCC
Rev.of_GCF_000181495.1_ASM18149v1_genomic.fna_186:148691-149003 Satlength=313 Nr of Repeats=6 RepeatLength=52 seed=TCTCAAGGAC Num.seqs=6 Similarity=0.745912   46
NTCAAGACACTTT--TGAGCTAATTTTATCCTGTTCATGTCCTTGAGACTCTTCC

************ * ** ****** ** *** ************ * ***
Consensus:

cTCAAGACACTTTccTcaGCtaATTTTAcCCgGTTcATGTCCTTGAGAcaCcTCC

>Bacillus_Fam_412_55_2 Nr. of seq. 2 Alignment length(with gaps) = 55
Alignment score = 0.624242
GCF_000285535.1_ASM28553v1_genomic.fna_3:66531-66842 Satlength=312 Nr of Repeats=6 RepeatLength=52 seed=CTTCATCAAC Num.seqs=5 Similarity=0.858974
0   CTTCATCAACC-CGATGAAGGACTTTCCAGCGA-CACT-TCCGCTGCATTCGCGA
GCF_000285535.1_ASM28553v1_genomic.fna_3:178754-178962 Satlength=209 Nr of Repeats=4 RepeatLength=52 seed=ATGAAGGACT Num.seqs=4 Similarity=0.497428
14   CTTCATCAGCCTCGATGAAGGACTTTCC-CCTAGTAATCTAC-CTCCATTCGAGA

******** ** *************** * * * * * * ** ****** **
Consensus:

CTTCATCAaCCtCGATGAAGGACTTTCCacCgAgcAaTcTaCgCTcCATTCGaGA

>Bacillus_Fam_413_55_2 Nr. of seq. 2 Alignment length(with gaps) = 55
Alignment score = 0.633333
GCF_000294775.2_ASM29477v2_genomic.fna_1:2273821-2274185 Satlength=365 Nr of Repeats=7 RepeatLength=52 seed=CTCAAAAAAT Num.seqs=7 Similarity=0.943834   0
CTCAAAAAATGGGAGCTAATCGAAATGAATAACGACGAAAAAGAAACCTCTA---
Rev.of_GCF_000294775.2_ASM29477v2_genomic.fna_1:2924329-2925107 Satlength=779 Nr of Repeats=15 RepeatLength=52 seed=TTTTCGTCGT Num.seqs=13 Similarity=0.847469
41   CATAAGAAATGGTAGTTAATCGTAATGAATAACGACGAAAAAG---GCTGTGCNC

* ** ****** ** ****** *******************   ** *
Consensus:

CacAAaAAATGGgAGcTAATCGaAATGAATAACGACGAAAAAGaaacCTcTacnc

>Bacillus_Fam_414_55_2 Nr. of seq. 2 Alignment length(with gaps) = 55
Alignment score = 0.639394
GCF_000307875.1_BABA1.0_genomic.fna_114:62513-62721 Satlength=209 Nr of Repeats=4 RepeatLength=52 seed=TTTTGTCTTT Num.seqs=4 Similarity=0.897436
0   TTTTGTCTTTAATAAGGGGTCTTAAAGAC--ATTTCCAGCGAAAGGAACATGCA-
Rev.of_GCF_001591665.1_ASM159166v1_genomic.fna_52:7926-8342 Satlength=417 Nr of Repeats=8 RepeatLength=52 seed=CCCTTATTAA Num.seqs=8

Similarity=0.879155 18
TTTTGTCTTTAATAAGGGCTCTTAAAGACAAAAATGCAG-GAAA-G-CCATGGGG

***************** ********** * * *** **** * ****
Consensus:

TTTTGTCTTTAATAAGGGcTCTTAAAGACaaAaaTcCAGcGAAAgGaaCATGcag

>Bacillus_Fam_415_55_2 Nr. of seq. 2 Alignment length(with gaps) = 55
Alignment score = 0.642424
GCF_000508325.1_BAVI_1_genomic.fna_45:30780-31040 Satlength=261 Nr of Repeats=5 RepeatLength=52 seed=TTCATAAAGC Num.seqs=5 Similarity=0.851282
0 TTCATAAAGCCCG-CGCCAGAGGAAAAAAG--GTCAAAGCGGTCAATAAGAGAGG
Rev.of_GCF_001591805.1_ASM159180v1_genomic.fna_7:179457-179715 Satlength=259 Nr of Repeats=5 RepeatLength=52 seed=GGGCTTTATG Num.seqs=3 Similarity=0.786325 12
TTCATAAAGCCCGTNGAC-GCNGAAAAAAGCCGNC-CA-CGGTCAATAAGAGCGG

************* * * * ******** * * * ************ **
Consensus:

TTCATAAAGCCCGtcGaCaGagGAAAAAAGccGtCaaAgCGGTCAATAAGAGaGG

>Bacillus_Fam_416_55_2 Nr. of seq. 2 Alignment length(with gaps) = 55
Alignment score = 0.624242
GCF_000621445.1_ASM62144v1_genomic.fna_2:234605-234813 Satlength=209 Nr of Repeats=4 RepeatLength=52 seed=TTTGTCTAAT Num.seqs=4
Similarity=0.847737
0 TTTGTCTAATAGAGCCTTTCTATTGGACACTTTTCTCCGATT-TTGCAC-T-CGT
Rev.of_GCF_000813125.1_ASM81312v1_genomic.fna_73:46396-46657 Satlength=262 Nr of Repeats=5 RepeatLength=52 seed=CGGGAAAGAA Num.seqs=4
Similarity=0.738470 47
TTTGTCCGATAGAGCCTTTCTATCGGACA--TTT-TCCGCTTCTTTCCCGTCCCT

****** ************** ***** *** **** ** ** * * * * * *
Consensus:

TTTGTCcaATAGAGCCTTTCTATcGGACActTTTcTCCGaTTcTTgCaCgTcCcT

>Bacillus_Fam_417_55_2 Nr. of seq. 2 Alignment length(with gaps) = 55
Alignment score = 0.636364
GCF_000709935.2_ASM70993v2_genomic.fna_1:279198-279562 Satlength=365 Nr of Repeats=7 RepeatLength=52 seed=AATTGCTCCA Num.seqs=7
Similarity=0.831387
0 AATTGCTCCAGCTGATTTATAGGGAACCATAATTG---CTTTTTGATACCCCATA
Rev.of_GCF_000708755.2_ASM70875v2_genomic.fna_2:135718-136030 Satlength=313 Nr of Repeats=6 RepeatLength=52 seed=GGGAAGCAAA Num.seqs=4
Similarity=0.736625 50
ATTTGCTCCGTCTGATTTATGAGGTACCAT-TTTGACGCTTTTTGCTTCCCTATA

* ******* ********* ** ***** *** ******* * *** ***
Consensus:

AaTTGCTCCagCTGATTTATaaGGaACCATaaTTGacgCTTTTTGaTaCCCcATA

>Bacillus_Fam_418_55_2 Nr. of seq. 2 Alignment length(with gaps) = 55
Alignment score = 0.624242
GCF_000986785.1_ASM98678v1_genomic.fna_1:69648-69960 Satlength=313 Nr of Repeats=6 RepeatLength=52 seed=CCTCCATTCG Num.seqs=6 Similarity=0.831447
0 CCTCCATTCGGACTC-GAGACACCGCTTTC-CTC-CTCGGCCTGTCCGAATCAGA
Rev.of_GCF_001591645.1_ASM159164v1_genomic.fna_5:52435-52699
Satlength=265 Nr of Repeats=5 RepeatLength=53 seed=TTCGGACAGC Num.seqs=4
Similarity=0.878407 48
CCTTCATTCGGACTCAGA-AGATCACTTGCTCTCGCTTTG-CTGTCCGAATCAGT

*** *********** ** * * * *** * *** ** * *************
Consensus:

CCTcCATTCGGACTCaGAgAcAcCaCTTgCtCTCgCTcgGcCTGTCCGAATCAGa

>Bacillus_Fam_419_55_2 Nr. of seq. 2 Alignment length(with gaps) = 55
Alignment score = 0.669697
GCF_001420715.1_ASM142071v1_genomic.fna_22:613446-613812 Satlength=367 Nr of Repeats=7 RepeatLength=52 seed=TTCAAAAGAC Num.seqs=5
Similarity=0.861538 0
TTCAAAAGACCAAGAGGAAGGAAAAATCAG--GTCGAGATGGTCTTTAT-NGGGA
Rev.of_GCF_001420715.1_ASM142071v1_genomic.fna_22:3743530-3743790
Satlength=261 Nr of Repeats=5 RepeatLength=52 seed=TCCTCTTGGT Num.seqs=5
Similarity=0.866667 17 -
TCAAAAGACCAAGAGGAAGG-AAAACCGGAAG-CGAGATGTCCTTTATAAGGGT

******************* **** * * * ******* ****** ***
Consensus:

tTCAAAAGACCAAGAGGAAGGaAAAAcCaGaaGtCGAGATGgcCTTTATaaGGGa

>Bacillus_Fam_420_55_2 Nr. of seq. 2 Alignment length(with gaps) = 55
Alignment score = 0.627273
GCF_001420715.1_ASM142071v1_genomic.fna_22:733261-733781 Satlength=521 Nr of Repeats=10 RepeatLength=52 seed=GGTAACAATA Num.seqs=10
Similarity=0.930484 0
GGTAACAATAGAAGGAGTATCGTGCCCGAAATCAAGCCCG-AAGAAGG-AATAT-
Rev.of_GCF_001420715.1_ASM142071v1_genomic.fna_22:2607422-2607686
Satlength=265 Nr of Repeats=5 RepeatLength=52 seed=TATTGTTACC Num.seqs=3
Similarity=0.897436 10
GGTAACAATAGTCCAAGTATTGTGCCCGAAATCAAG---GTAAGCAGGCAAAAAC

*********** ***** *************** * *** *** ** *
Consensus:

GGTAACAATAGaacaAGTATcGTGCCCGAAATCAAGcccGtAAGaAGGcAAaAac

>Bacillus_Fam_421_55_2 Nr. of seq. 2 Alignment length(with gaps) = 55
Alignment score = 0.630303
GCF_001439915.1_ASM143991v1_genomic.fna_83:102373-102576 Satlength=204 Nr of Repeats=4 RepeatLength=52 seed=GGTTATGAAG Num.seqs=3
Similarity=0.794872 0
GGTTATGAAGGAC---ATTCCGAAGAGAGAACNNCGACGAACAGTCCTTCATCGA

Rev.of_GCF_001636425.1_ASM163642v1_genomic.fna_1:2411742-2412106 Satlength=365 Nr of Repeats=7 RepeatLength=52 seed=ATGAAGGACT Num.seqs=7 Similarity=0.880342 49
GGGGATGAAGGACGAAAGGACGAGGAGAGAA--GCGA-GAAAAGTCCTTCATCGA

** ********* * *** ******* *** *** *************
Consensus:

GGggATGAAGGACgaaAggaCGAaGAGAGAAcngCGAcGAAaAGTCCTTCATCGA

>Bacillus_Fam_422_55_2 Nr. of seq. 2 Alignment length(with gaps) = 55
Alignment score = 0.624242
GCF_001439915.1_ASM143991v1_genomic.fna_122:173650-173910 Satlength=261 Nr of Repeats=4 RepeatLength=52 seed=TTTCGGACTC Num.seqs=3
Similarity=0.811966 0
TTTCGGACTCATCA--TTTCGAAA-TATGCTCGCGGCTGTCCGAATCAAGCGCTC
Rev.of_GCF_001439915.1_ASM143991v1_genomic.fna_124:121562-121979 Satlength=418 Nr of Repeats=8 RepeatLength=52 seed=CCTTGATTCG Num.seqs=7 Similarity=0.800977 48
TTTCGGACTCAACAGGCTAAAAAAGTATGCACTC---TGTCCGAATCAAGGGCTC

*********** ** * *** ***** * * ************* ****
Consensus:

TTTCGGACTCAaCAggcTaaaAAAgTATGCaCgCggcTGTCCGAATCAAGcGCTC

>Bacillus_Fam_423_55_2 Nr. of seq. 2 Alignment length(with gaps) = 55
Alignment score = 0.651515
GCF_001591945.1_ASM159194v1_genomic.fna_9:103168-103374 Satlength=207 Nr of Repeats=4 RepeatLength=52 seed=TTTGTCCTTT Num.seqs=3
Similarity=0.880342
0 TTTGTCCTTTAGCACGCT-TCTCTCGGACATCTTCCTCCCT-TTTCTTCCCCAC-
GCF_001591945.1_ASM159194v1_genomic.fna_9:110417-110677 Satlength=261 Nr of Repeats=5 RepeatLength=52 seed=TCCTTTAGAC Num.seqs=5
Similarity=0.835897
4 TCTGTCCTTTAG-ACGCTCTCTCTTAGACA-CTT-CTCCCTGATTCTCACCCACT

* ********** ***** ***** **** *** ****** **** *****
Consensus:

TcTGTCCTTTAGcACGCTcTCTCTcaGACAtCTTcCTCCCTgaTTCTcaCCCACt

>Bacillus_Fam_424_55_2 Nr. of seq. 2 Alignment length(with gaps) = 55
Alignment score = 0.612121
GCF_001636315.1_ASM163631v1_genomic.fna_1:1916684-1916892 Satlength=209 Nr of Repeats=4 RepeatLength=52 seed=ACCTTTTTCA Num.seqs=4
Similarity=0.846154 0
ACC---TTTTTCATAGAGTTCCCTCTTTTTCGGGCAATGCAACCCACTCTCATTT
GCF_001636315.1_ASM163631v1_genomic.fna_1:972743-973108 Satlength=366 Nr of Repeats=7 RepeatLength=52 seed=CAATGCAACC Num.seqs=6
Similarity=0.733333
33 ACCGAAAATATCA-ATCTTTTCCTCTTTTTCGGGCAATGCAACCCCCTTCCATTA

*** * *** * ** *********************** ** ****

Consensus:

ACCgaaaaTaTCAtAgagTTcCCTCTTTTTCGGGCAATGCAACCCaCTccCATTa

>Bacillus_Fam_425_55_2 Nr. of seq. 2 Alignment length(with gaps) = 55
Alignment score = 0.603030
GCF_001636315.1_ASM163631v1_genomic.fna_1:2344320-2344632 Satlength=313
Nr of Repeats=6 RepeatLength=52 seed=GATGAAGGAC Num.seqs=6
Similarity=0.933333 0
GATGAAGGACATTTTG-CATGGGAAACCTGCTTCG-TTTGGTCTTCATC-ACGCC
GCF_001636425.1_ASM163642v1_genomic.fna_1:2956434-2957110 Satlength=677
Nr of Repeats=13 RepeatLength=52 seed=AAGGGCTTCA Num.seqs=13
Similarity=0.827447 34
-ATGAAGGACTTTTCGCCAT-GG-AACCAACCTCGATAAGGGCTTCATCGACGCC

********* *** * *** ** **** * *** * ** ******* *****
Consensus:

gATGAAGGACaTTTcGcCATgGGaAACCaaCcTCGaTaaGGgCTTCATCgACGCC

>Bacillus_Fam_426_55_2 Nr. of seq. 2 Alignment length(with gaps) = 55
Alignment score = 0.657576
GCF_001636345.1_ASM163634v1_genomic.fna_1:388884-389144 Satlength=261 Nr
of Repeats=5 RepeatLength=52 seed=GAAACAGGAC Num.seqs=5
Similarity=0.820126
0 GAAACAGGACANTAAACCCCAGTAAGTGGAAAATAGAGAGACG-G-AAA-GAGCG
Rev.of_GCF_001636345.1_ASM163634v1_genomic.fna_1:2655784-2656044
Satlength=261 Nr of Repeats=5 RepeatLength=52 seed=TTTCTCTCAT Num.seqs=5
Similarity=0.841026 3 -
AAACAGGACANGAAACCCCAGTAACTGGTAAAT-G-GAGTCGTGTAAATGAGAG

*********** *********** *** **** * *** ** * *** *** *
Consensus:

gAAACAGGACANgAAACCCCAGTAAcTGGaAAATaGaGAGaCGtGtAAAtGAGaG

>Bacillus_Fam_427_55_2 Nr. of seq. 2 Alignment length(with gaps) = 55
Alignment score = 0.690909
GCF_002019605.1_ASM201960v1_genomic.fna_1:4272331-4272850 Satlength=520
Nr of Repeats=10 RepeatLength=52 seed=AACGAGCAGG Num.seqs=9
Similarity=0.846154 28
GGTAACTATGAAGCGTTCATGATGCCCGAACGAGCAGGAGAAGAAA---GAAAAC
GCF_002019605.1_ASM201960v1_genomic.fna_1:4387305-4388137 Satlength=833
Nr of Repeats=16 RepeatLength=52 seed=CGAGAGAAGA Num.seqs=16
Similarity=0.917949 28
GGTAACTATAGAGCGTTCATAATGCCCG--CGAG-AGAAGAAAAAATGAGAGAAC

**** ** **** *** ** ************ ********* *******
Consensus:

aaCGAGcAGaAGAAaAAAtgaGAaAACGGTAACTATaaAGCGTTCATaATGCCCG

>Bacillus_Fam_428_55_2 Nr. of seq. 2 Alignment length(with gaps) = 55
Alignment score = 0.636364

BACILLUS_PSEUDALCALIPHILUS_genomic.fna_1:51279-51543 Satlength=265 Nr of Repeats=5 RepeatLength=53 seed=AAAAAGAGCA Num.seqs=4 Similarity=0.899371
0   AAAAAGAGCAAGAAGGTGCCGACCAAAG--CGTGAGGTCGACATGATCAGAGTGA
BACILLUS_PSEUDALCALIPHILUS_genomic.fna_5:490647-490965 Satlength=319 Nr of Repeats=6 RepeatLength=53 seed=GCATCGACAT Num.seqs=6 Similarity=0.847119
33   AAAAAGGTCAAGAACCTGTCGACTAAAGCTCGTG-CATCGACATGAACAGGGCGA

****** ****** ** **** **** ****   ********* *** * **
Consensus:

AAAAAGagCAAGAAccTGcCGACcAAAGctCGTGacaTCGACATGAaCAGaGcGA

>Bacillus_Fam_429_55_2 Nr. of seq. 2 Alignment length(with gaps) = 55
Alignment score = 0.660606
GCF_001315165.1_ASM131516v1_genomic.fna_30:29786-29998 Satlength=213 Nr of Repeats=4 RepeatLength=53 seed=CCCTTATGCG Num.seqs=4
Similarity=0.882600
0   CCCTTATGC-GAGGAAAATGTCATCTATAAGGGTACAATTA-AGAGCGAATCATA
GCF_001315165.1_ASM131516v1_genomic.fna_78:17736-17948 Satlength=213 Nr of Repeats=4 RepeatLength=53 seed=AATCGTACCC Num.seqs=4
Similarity=0.878407
46   CCCTTATGCAGGGTAACCGGTC-CCTATAAGGGTACAATTAGGGAG-GAATCGTA

********* * * **   *** ***************** *** ***** **
Consensus:

CCCTTATGCaGaGgAAaagGTCacCTATAAGGGTACAATTAgaGAGcGAATCaTA

>Bacillus_Fam_430_55_2 Nr. of seq. 2 Alignment length(with gaps) = 55
Alignment score = 0.660606
GCF_900109925.1_IMG-taxon_2651870116_annotated_assembly_genomic.fna_22:99833-100097
Satlength=265 Nr of Repeats=5 RepeatLength=53 seed=ATTGGTGACC Num.seqs=4
Similarity=0.920335   1   CATTGGTGACCTTTCTTCAAC-TTCACCCACCTAGATAGGGCACCAAT-AGCCTT
GCF_900109925.1_IMG-taxon_2651870116_annotated_assembly_genomic.fna_22:209996-210208
Satlength=213 Nr of Repeats=4 RepeatLength=53 seed=ATTGGTGACC Num.seqs=4
Similarity=0.756173   1
CATTGGTGACCTTTCTTCCTCTTTCATCTACC-ACTTAGGGCACCAATCAACC-C

***************** * **** * *** * ************ * ** *
Consensus:

ATTGGTGACCTTTCTTCaaCtTTCAcCcACCtAcaTAGGGCACCAATcAaCCtcC

>Bacillus_Fam_431_54_2 Nr. of seq. 2 Alignment length(with gaps) = 54
Alignment score = 0.669753
GCF_000171615.1_ASM17161v1_genomic.fna_74:41439-41699 Satlength=261 Nr of Repeats=5 RepeatLength=52 seed=GCGCGGTGCA Num.seqs=5 Similarity=0.912821
0   GCGCGGTGCAGAAAAGAGGAG-AAGGAAGAGAGAACAGCCCCGCGCGAGCCG-G
Rev.of_GCF_000171615.1_ASM17161v1_genomic.fna_128:4002-4209 Satlength=208 Nr of Repeats=4 RepeatLength=52 seed=GCTCTTCTCC Num.seqs=3

Similarity=0.829060 37
-CGCGGTGCA-CAAAGAGGAGAAAAGAAGGGAGAAGAGCACCGCGCGGGGGAG

********* ********* ** **** ***** *** ******* * * *
Consensus:

gCGCGGTGCAgaAAAGAGGAGaAAaGAAGaGAGAAcAGCaCCGCGCGaGccGaG

>Bacillus_Fam_432_54_2 Nr. of seq. 2 Alignment length(with gaps) = 54
Alignment score = 0.743827
GCF_000175075.1_ASM17507v1_genomic.fna_4:86443-86651 Satlength=209 Nr of Repeats=4 RepeatLength=52 seed=AATGAAGACA Num.seqs=4 Similarity=0.876068
0 AATGAAGACACCTCTC-CTCCTAATTTCACCTCACTCACGTCTTCATACC-TCG
Rev.of_GCF_000175075.1_ASM17507v1_genomic.fna_32:274563-274979
Satlength=417 Nr of Repeats=8 RepeatLength=52 seed=ATGTCTTCAT Num.seqs=8
Similarity=0.792125 10 -
ATGAAGACATCTCTCACTCC-ATTTTCACCTCACGGACGTCTTCATACCGTCT

********* ***** **** * *********** ************* **
Consensus:

aATGAAGACAcCTCTCaCTCCtAaTTTCACCTCACgcACGTCTTCATACCgTCg

>Bacillus_Fam_433_54_2 Nr. of seq. 2 Alignment length(with gaps) = 54
Alignment score = 0.629630
GCF_000175075.1_ASM17507v1_genomic.fna_5:134083-134447 Satlength=365 Nr of Repeats=7 RepeatLength=52 seed=GGTGAACATT Num.seqs=7
Similarity=0.865690
0 GGTGAACATTGTCCTCGTAGGAGTG-GAATGACGCCGTCCACCGGTG-AAAAGC
Rev.of_GCF_000175075.1_ASM17507v1_genomic.fna_31:202656-202864
Satlength=209 Nr of Repeats=4 RepeatLength=52 seed=GACAATGTTT Num.seqs=4
Similarity=0.893162 12
GG-AAACATTGTCCTCGTAGG-GTGTCAATGAAGCCCTGAGCCGATGAAAAAGA

** **************** *** ***** *** * *** ** *****
Consensus:

GGtaAACATTGTCCTCGTAGGaGTGtcAATGAaGCCcTcaaCCGaTGaAAAAGa

>Bacillus_Fam_434_54_2 Nr. of seq. 2 Alignment length(with gaps) = 54
Alignment score = 0.771605
GCF_000177235.2_ASM17723v2_genomic.fna_1:3741040-3741248 Satlength=209 Nr of Repeats=4 RepeatLength=52 seed=TCTTACCTTC Num.seqs=4
Similarity=0.816872 0
TCTTACCTTCACACTCTTTTTTCTTTGCTCAAGGGCAGATGGCAGACT-C-CCA
GCF_000177235.2_ASM17723v2_genomic.fna_1:4577046-4578346 Satlength=1301
Nr of Repeats=25 RepeatLength=52 seed=GCTCAAGGGC Num.seqs=25
Similarity=0.816154 26
TCTTACCTTCACACTATTTTTTATTCGCTCAAGGGCAGATGG--GACNGCGCCA

*************** ****** ** *************** *** * ***
Consensus:

TCTTACCTTCACACTaTTTTTTaTTcGCTCAAGGGCAGATGGcaGACtgCgCCA

>Bacillus_Fam_435_54_2 Nr. of seq. 2 Alignment length(with gaps) = 54
Alignment score = 0.654321
GCF_000177235.2_ASM17723v2_genomic.fna_1:4033931-4034762 Satlength=832 Nr of Repeats=17 RepeatLength=52 seed=TTTCCTTTCT Num.seqs=14
Similarity=0.882784   3
GGCTTTCCTTTCT-ATGCACTTTTCATCCTTAGAAAGGAATACTA-ACTCCTCT
GCF_000177235.2_ASM17723v2_genomic.fna_1:4080458-4081081 Satlength=624 Nr of Repeats=12 RepeatLength=52 seed=AAAGGAATGC Num.seqs=11
Similarity=0.865734   32
CGCTTTCCTTTCTAACGCTCGTTTC-TCTTCAGAAAGGAATGCTAGACTCGT-T

********** * ** * **** ** * ********** *** **** * * **
Consensus:

TTTCCTTTCTaAcGCaCgTTTCaTCcTcAGAAAGGAATaCTAgACTCcTcTcGC

>Bacillus_Fam_436_54_2 Nr. of seq. 2 Alignment length(with gaps) = 54
Alignment score = 0.617284
GCF_000181495.1_ASM18149v1_genomic.fna_177:12081-12289 Satlength=209 Nr of Repeats=4 RepeatLength=52 seed=TTCATAAGAC Num.seqs=4
Similarity=0.846154
0   TTCAT-AAGACCAAAACTAAGGTGGAAATGCAG-TTAGGGTGTCTCATGAACGC
Rev.of_GCF_000181495.1_ASM18149v1_genomic.fna_156:9142-9350 Satlength=209 Nr of Repeats=4 RepeatLength=52 seed=TCTTTTTGGC Num.seqs=4
Similarity=0.849057   18
TTCATGGAG-CCAAAAAGAGAGTGGATTTCAAGATTAGGGTGTCTCATGAAAGG

***** ** ****** * ***** * ** ***************** *
Consensus:

TTCATgaAGaCCAAAAagAaaGTGGAaaTcaAGaTTAGGGTGTCTCATGAAaGc

>Bacillus_Fam_437_54_2 Nr. of seq. 2 Alignment length(with gaps) = 54
Alignment score = 0.635802
GCF_000181495.1_ASM18149v1_genomic.fna_177:51711-52127 Satlength=417 Nr of Repeats=8 RepeatLength=52 seed=GTTAGACCCT Num.seqs=8
Similarity=0.792677
0   GTTAGACCCTGTGGAATTGAAGAAAAAAGA-AATCGGCCGTGTTAAA-AGGTTG
GCF_000181495.1_ASM18149v1_genomic.fna_186:402271-402635 Satlength=365 Nr of Repeats=7 RepeatLength=52 seed=AAGAGAATCG Num.seqs=7
Similarity=0.820305   25
TTTGGACCCGGTGGAATCCNA-AAAGAAGAGAATCGACCGGGTTAAAGAGG-TG

** ***** *******   * *** **** ***** *** ****** *** **
Consensus:

gTTaGACCCgGTGGAATccaAgAAAaAAGAgAATCGaCCGgGTTAAAgAGGtTG

>Bacillus_Fam_438_54_2 Nr. of seq. 2 Alignment length(with gaps) = 54
Alignment score = 0.635802
GCF_000242895.2_ASM24289v3_genomic.fna_1:1675220-1675480 Satlength=261 Nr of Repeats=5 RepeatLength=52 seed=AAAAAGTGTG Num.seqs=5

Similarity=0.902564 0
AAAAAGTGTGGAG-CGGTAACTGAAACAAG-GAAGTAGTGACCAAAGAGGGTGG
GCF_000242895.2_ASM24289v3_genomic.fna_1:1925605-1925915 Satlength=311 Nr of Repeats=6 RepeatLength=52 seed=CGGTCACTAA Num.seqs=5
Similarity=0.757862 14
AAAAAGNACCCAGNCGGTCACT-AAACCAGTGAAGTAGTGACCAAAGAGAG-GA

****** ** **** *** **** ** ***************** * *
Consensus:

AAAAAGtacccAGnCGGTaACTgAAACaAGtGAAGTAGTGACCAAAGAGaGtGa

>Bacillus_Fam_439_54_2 Nr. of seq. 2 Alignment length(with gaps) = 54
Alignment score = 0.611111
GCF_000311725.1_ASM31172v1_genomic.fna_2:554922-555286 Satlength=365 Nr of Repeats=7 RepeatLength=52 seed=TGTCCGAATC Num.seqs=7
Similarity=0.875458
0 TGTCCGAATC-CAGCTATTATTCGGACTCCTTCTTTGTTTTTCGCTCCAC-TC
Rev.of_GCF_001439915.1_ASM143991v1_genomic.fna_116:114146-114724
Satlength=579 Nr of Repeats=10 RepeatLength=52 seed=GGTGTGATTC
Num.seqs=6 Similarity=0.815385
15 TGTCCGAATCACACCTACT-TTCAGACTCNACCTTCGCTTTTCCGC-CAACATC

********** ** *** * *** ***** *** * **** *** * ** **
Consensus:

TGTCCGAATCaCAcCTAcTaTTCaGACTCcacCTTcGcTTTTcCGCtCaACaTC

>Bacillus_Fam_440_54_2 Nr. of seq. 2 Alignment length(with gaps) = 54
Alignment score = 0.660494
GCF_000331575.1_ASM33157v1_genomic.fna_1:1524595-1525167 Satlength=573 Nr of Repeats=11 RepeatLength=52 seed=TTTTCTGTCC Num.seqs=11
Similarity=0.902564 0
TTTTCTGTCCGAACTTGGCCTCAGTTCGGACTCAATTTTAGCTTTTGCTGCG--
Rev.of_GCF_000331575.1_ASM33157v1_genomic.fna_4:27225-27953 Satlength=729 Nr of Repeats=14 RepeatLength=52 seed=GACAGGAAAT Num.seqs=14
Similarity=0.915187 9
TTTCCTGTCCGAACTGGGGCTGAGTTCGGACACGA-TTTAGCAGTT-CTGGGGA

*** *********** ** ** ********* * * ****** ** *** *
Consensus:

TTTcCTGTCCGAACTgGGcCTcAGTTCGGACaCaAtTTTAGCagTTgCTGcGga

>Bacillus_Fam_441_54_2 Nr. of seq. 2 Alignment length(with gaps) = 54
Alignment score = 0.626543
GCF_000374345.1_ASM37434v1_genomic.fna_8:71233-71545 Satlength=313 Nr of Repeats=6 RepeatLength=52 seed=AGAAAATATC Num.seqs=6 Similarity=0.796581
0 AGAAAATATCGGGCCGGAATGGCGTTCATAGGGNAAATGAAAGCTATTTTG-G-
Rev.of_GCF_000374345.1_ASM37434v1_genomic.fna_18:16790-17225
Satlength=436 Nr of Repeats=6 RepeatLength=52 seed=TGGCTTTCAT Num.seqs=4
Similarity=0.591919 44
AG-AAATAGCAGG-GGGAATGGCGTTCATTTGGGTGATGAAAGCCATCTTGAGA

** ***** * ** ************** **   ******** ** *** *
Consensus:

AGaAAATAgCaGGccGGAATGGCGTTCATagGGgaaATGAAAGCcATcTTGaGa

>Bacillus_Fam_442_54_2 Nr. of seq. 2 Alignment length(with gaps) = 54
Alignment score = 0.617284
GCF_000374345.1_ASM37434v1_genomic.fna_16:99-567 Satlength=469 Nr of Repeats=9 RepeatLength=52 seed=GCGTTCATCC Num.seqs=9 Similarity=0.717680
0   GCGTTCATCCCGCTNATGAACGTCATTACAANTCAANTTTTGCA-CC-GGGATG
Rev.of_GCF_000374345.1_ASM37434v1_genomic.fna_74:7594-7802 Satlength=209 Nr of Repeats=4 RepeatLength=52 seed=GGGGATGAAC Num.seqs=4 Similarity=0.790598   12
GCGTTCATCCCCCTTATGAACGCCATCTC-GTTCAA-TTTTCCATCCTGTGATA

*********** ** ******* *** *   **** **** ** ** * ***
Consensus:

GCGTTCATCCCcCTtATGAACGcCATcaCaatTCAAnTTTTcCAtCCtGgGATa

>Bacillus_Fam_443_54_2 Nr. of seq. 2 Alignment length(with gaps) = 54
Alignment score = 0.641975
GCF_000401235.1_BacNeaAAU1_genomic.fna_108:2225-2531 Satlength=307 Nr of Repeats=4 RepeatLength=52 seed=AGGTAACAGA Num.seqs=3 Similarity=0.775681
0   AGGTAACAGAGAGAACTGCTCAGTGACCGT-TGCA-GCAAAGGAAAGGAGGAAA
GCF_000401235.1_BacNeaAAU1_genomic.fna_276:14688-14894 Satlength=207 Nr of Repeats=4 RepeatLength=52 seed=GAAAAACAAA Num.seqs=3 Similarity=0.914530
36   AGGTAACAGAGAGGGCTCCTCAGTGCCCGTAAGCACGAAAAACAAA--AGCAAA

************* ** ******* **** *** * *** *** ** ***
Consensus:

AGGTAACAGAGAGaaCTcCTCAGTGaCCGTaaGCAcGaAAAacAAAggAGcAAA

>Bacillus_Fam_444_54_2 Nr. of seq. 2 Alignment length(with gaps) = 54
Alignment score = 0.654321
GCF_000430765.1_ASM43076v1_genomic.fna_6:269287-269548 Satlength=262 Nr of Repeats=5 RepeatLength=52 seed=CACTTCGGAC Num.seqs=4 Similarity=0.871795
0   CACTTCGGACAGATTGCGGACCCTGGT--CTCAAAAGCTGTCCGAACTCAGATC
GCF_002019645.1_ASM201964v1_genomic.fna_1:4520185-4521327 Satlength=1143 Nr of Repeats=22 RepeatLength=52 seed=GTTCGGACAG Num.seqs=20 Similarity=0.812551   2
AAGTTCGGACAGCTT--GGACCCCAATAGATCAAAAGCTGTCCGAAGTCAGAGC

* ********* ** ******   *   *************** ***** *
Consensus:

aAcTTCGGACAGaTTgcGGACCCcaaTagaTCAAAAGCTGTCCGAAcTCAGAgC

>Bacillus_Fam_445_54_2 Nr. of seq. 2 Alignment length(with gaps) = 54
Alignment score = 0.694444

GCF_000508325.1_BAVI_1_genomic.fna_12:41249-41457 Satlength=209 Nr of Repeats=4 RepeatLength=52 seed=AAAAAAGAAG Num.seqs=4 Similarity=0.910256
0 AAAAAAGAAGGAGACGGGCACTGTGGAG-GGTTCAGAGTGCCCGTGGGAGCTG-
Rev.of_GCF_001591805.1_ASM159180v1_genomic.fna_10:78357-78669 Satlength=313 Nr of Repeats=6 RepeatLength=52 seed=TTCACGGGCA Num.seqs=6 Similarity=0.852991 47
AAAAAAGCGGGAGTCGGTCACT-TAGAGAGGTTCATAGTGCCCGT-GAAGCTGA

******* **** *** **** * *** ****** ********* * *****
Consensus:

AAAAAAGaaGGAGaCGGgCACTgTaGAGaGGTTCAgAGTGCCCGTgGaAGCTGa

>Bacillus_Fam_446_54_2 Nr. of seq. 2 Alignment length(with gaps) = 54
Alignment score = 0.604938
GCF_000708755.2_ASM70875v2_genomic.fna_7:27921-28441 Satlength=521 Nr of Repeats=9 RepeatLength=52 seed=TTGACCCTTC Num.seqs=8 Similarity=0.814103
0 TTGACCCTTCAGCTCTNT--TGGTTTGCTCTGAAGAGTCATATGCNATCCTGTT
GCF_000709935.2_ASM70993v2_genomic.fna_4:144384-144644 Satlength=261 Nr of Repeats=5 RepeatLength=52 seed=GAAGGGTCAT Num.seqs=5 Similarity=0.730818
29 TAGACCCTTCAGAT-TATGCTGGTCT-CCGTGAAGGGTCATATGTGCTCCCGTT

* ********** * * * **** * * ***** ******** *** ***
Consensus:

TaGACCCTTCAGaTcTaTgcTGGTcTgCccTGAAGaGTCATATGcgaTCCcGTT

>Bacillus_Fam_447_54_2 Nr. of seq. 2 Alignment length(with gaps) = 54
Alignment score = 0.629630
GCF_000752395.1_Bacillus_andreraoultii_genomic.fna_41:274960-275582 Satlength=623 Nr of Repeats=12 RepeatLength=52 seed=GTCTATGAGC Num.seqs=11 Similarity=0.849285 0
GTCTATGAGCCCGAAAA-AGCAGAAACCCCGTAAAAAGCGGTTC-ATAGAAATG
GCF_000752395.1_Bacillus_andreraoultii_genomic.fna_43:179773-180164 Satlength=392 Nr of Repeats=8 RepeatLength=52 seed=CGTCTATGAA Num.seqs=7 Similarity=0.941392 51
GTCTATGAACCCAAAAATAAC-GAAATTCCGCAAAAA-CGGTCCTATAGAACTC

******** *** **** * * **** *** ***** **** * ****** *
Consensus:

GTCTATGAaCCCaAAAAtAaCaGAAAccCCGcAAAAAgCGGTcCtATAGAAaTc

>Bacillus_Fam_448_54_2 Nr. of seq. 2 Alignment length(with gaps) = 54
Alignment score = 0.620370
GCF_000759675.1_ASM75967v1_genomic.fna_159:74142-74454 Satlength=313 Nr of Repeats=6 RepeatLength=52 seed=TTTGGTTCCC Num.seqs=6 Similarity=0.748971
0 TTTGGTTCCCTT-TATCGTTGAAAAGGTGAGTTCTTGGGAATCAAAAGAGAGT-
GCF_000759675.1_ASM75967v1_genomic.fna_170:98-669 Satlength=572 Nr of Repeats=11 RepeatLength=52 seed=AGGGAATCAA Num.seqs=10 Similarity=0.895157
34 TTGGGTTCCCTTCAAAGGGAAAAAAGCTGAGTT-GAGGGAATCAAAAG-GAGTC

** ********* * * ***** ****** ************ ****
Consensus:

TTgGGTTCCCTTcaAacGgaaAAAAGcTGAGTTcgaGGGAATCAAAAGaGAGTc

>Bacillus_Fam_449_54_2 Nr. of seq. 2 Alignment length(with gaps) = 54
Alignment score = 0.654321
GCF_000787375.1_ASM78737v1_genomic.fna_2:246377-246585 Satlength=209 Nr of Repeats=4 RepeatLength=52 seed=TACTCTTCTC Num.seqs=4 Similarity=0.884615
0 TACTCTTCTCTCAATCTCT-TTTTCGGGCGACCATCCTTCC-CTTTGACTACCG
GCF_000787375.1_ASM78737v1_genomic.fna_76:11-375 Satlength=365 Nr of Repeats=7 RepeatLength=52 seed=CTACCGTAAT Num.seqs=7 Similarity=0.860806
46 TAATCCTCT-TCAGGCTCTCTTTTCGGG-TTCCAACCTTCCTCTTTGGCTACCG

** ** *** *** **** ******** *** ****** ***** ******
Consensus:

TAaTCcTCTcTCAagCTCTcTTTTCGGGcgaCCAaCCTTCCtCTTTGaCTACCG

>Bacillus_Fam_450_54_2 Nr. of seq. 2 Alignment length(with gaps) = 54
Alignment score = 0.654321
GCF_000813125.1_ASM81312v1_genomic.fna_8:49357-49722 Satlength=366 Nr of Repeats=7 RepeatLength=52 seed=GGGGGACTTC Num.seqs=6 Similarity=0.875214
0 GGGGGACTTCGGACAAATT-CTG-CTCTTCCATCAGCTGTTTGTCCGAACCTG
Rev.of_GCF_000813125.1_ASM81312v1_genomic.fna_57:52500-52812 Satlength=313 Nr of Repeats=6 RepeatLength=52 seed=GTTCGGACAA Num.seqs=4 Similarity=0.668724 49
GGGTGACTTCGGACAAATTGAAGCCTC-TCCGGC-GATTCTTTGTCCGAACCTG

*** ************** * *** *** * * * *************
Consensus:

GGGgGACTTCGGACAAATTgaaGcCTCtTCCagCaGaTgcTTTGTCCGAACCTG

>Bacillus_Fam_451_54_2 Nr. of seq. 2 Alignment length(with gaps) = 54
Alignment score = 0.669753
GCF_000813125.1_ASM81312v1_genomic.fna_12:28-289 Satlength=262 Nr of Repeats=5 RepeatLength=52 seed=TGGAAATGTC Num.seqs=4 Similarity=0.846154
0 TGGAAATGTCTGAAGTAGCCCCAGGTTCGGCCAAAACAAC--CACTGGGATCGC
Rev.of_GCF_000813125.1_ASM81312v1_genomic.fna_141:38715-39390 Satlength=676 Nr of Repeats=11 RepeatLength=52 seed=ACTTCAGACA Num.seqs=8 Similarity=0.728938
15 -GGAAATGTCTGAAGTAGCCNCAGGTTCAGCCAAAATACCGTCA-TAGNCACGA

****************** ******* ******* * * ** * * **
Consensus:

tGGAAATGTCTGAAGTAGCCcCAGGTTCaGCCAAAAcAaCgtCAcTaGgaaCGa

>Bacillus_Fam_452_54_2 Nr. of seq. 2 Alignment length(with gaps) = 54
Alignment score = 0.611111

GCF_000934845.1_ASM93484v1_genomic.fna_40:14255-14619 Satlength=365 Nr of Repeats=7 RepeatLength=52 seed=AAACACCAGT Num.seqs=7 Similarity=0.798443
0   AAACACCAGTAACT-GGGAAAACAGTACACCAATAAAGCCG-ATTGGAGCACTA
Rev.of_GCF_000934845.1_ASM93484v1_genomic.fna_37:21000-21475 Satlength=476 Nr of Repeats=9 RepeatLength=53 seed=GCACCAATAC Num.seqs=7 Similarity=0.824221   49
AAAAACCAGAAATTAGGNAAATNAGGTCACCAAT-GAGCCGTATTGGTGCNCGA

*** ***** ** * ** *** ** ******* ***** ***** ** * *
Consensus:

AAAaACCAGaAAcTaGGgAAAacAGgaCACCAATaaAGCCGtATTGGaGCaCgA

>Bacillus_Fam_453_54_2 Nr. of seq. 2 Alignment length(with gaps) = 54
Alignment score = 0.648148
GCF_000986785.1_ASM98678v1_genomic.fna_2:121115-121636 Satlength=522 Nr of Repeats=10 RepeatLength=52 seed=ATCAGTGACC Num.seqs=9 Similarity=0.800570
0   ATCAGTGACC--TTTTCGGAGCTGGCCTGCTCATTTTGGGCACTGACAGCCTCT
Rev.of_GCF_000986785.1_ASM98678v1_genomic.fna_22:27488-27748 Satlength=261 Nr of Repeats=5 RepeatLength=52 seed=TGTCAGTACC Num.seqs=5 Similarity=0.830818   46
CTCAG-GACCACTTTTTGGAG-GGGNCGGCTCATTTTGGGTACTGACAGCGCAT

**** **** **** **** ** * ************ *********   *
Consensus:

aTCAGtGACCacTTTTcGGAGcgGGcCgGCTCATTTTGGGcACTGACAGCccaT

>Bacillus_Fam_454_54_2 Nr. of seq. 2 Alignment length(with gaps) = 54
Alignment score = 0.663580
GCF_000986785.1_ASM98678v1_genomic.fna_5:231430-232157 Satlength=728 Nr of Repeats=14 RepeatLength=52 seed=TGTCAGTGCC Num.seqs=13 Similarity=0.751820   0
TGTCAGTGCCTGTTTTTTGTNGTGAGGA-NGTTTTTTGGGTACTGAGGNGAGT-
Rev.of_GCF_000986785.1_ASM98678v1_genomic.fna_33:21710-22022 Satlength=313 Nr of Repeats=6 RepeatLength=52 seed=GGCACTGACA Num.seqs=6 Similarity=0.745912   10
TGTCAGTGCCCGTTTTTTCGAG-GGGGAGCGAGTTTTGGGTACTGAGGGG-GTC

********** *******   * * *** * ************** * **
Consensus:

TGTCAGTGCCcGTTTTTTcgaGtGaGGAgcGagTTTTGGGTACTGAGGgGaGTc

>Bacillus_Fam_455_54_2 Nr. of seq. 2 Alignment length(with gaps) = 54
Alignment score = 0.617284
GCF_001315085.1_ASM131508v1_genomic.fna_43:21365-21625 Satlength=261 Nr of Repeats=5 RepeatLength=52 seed=AACCAAAAGA Num.seqs=5 Similarity=0.794872
0   AACCAAAA-GAACGTGTG-GAGCAGAGGAAAAAGGTCATCATAAGGTCGATGAG
GCF_001315085.1_ASM131508v1_genomic.fna_73:11538-11746 Satlength=209 Nr of Repeats=4 RepeatLength=52 seed=AAACCAAGCT Num.seqs=4

Similarity=0.801363
5   GCCCAAAACCAAGCTGAGCGAGCACA--AAAAAGGTCATCATAAAGCCGATGAA

****** ** ** * ***** * *************** * ******
Consensus:

aaCCAAAAccAAccTGaGcGAGCAcAggAAAAAGGTCATCATAAaGcCGATGAa

>Bacillus_Fam_456_54_2 Nr. of seq. 2 Alignment length(with gaps) = 54
Alignment score = 0.604938
GCF_001420595.1_ASM142059v1_genomic.fna_11:518620-519088 Satlength=469 Nr of Repeats=9 RepeatLength=52 seed=GGTAACCAAA Num.seqs=9
Similarity=0.764245   0
GGTAACCAAAGGNAAGCAT-TGGTGCCCGTAAACGAGGAAAATAG-CGTTCCAC
Rev.of_GCF_001591445.1_ASM159144v1_genomic.fna_2:21009-21217
Satlength=209 Nr of Repeats=4 RepeatLength=52 seed=TCACGGGCAT Num.seqs=4
Similarity=0.816239   31
GGTAACCATAGG-AAGCGTATGATGCCCGTGAAGGTTGAAAA-AGTGGATCAAC

******** *** **** * ** ******* ** * ***** ** * ** **
Consensus:

GGTAACCAaAGGnAAGCaTaTGaTGCCCGTaAAcGagGAAAAtAGtcGaTCaAC

>Bacillus_Fam_457_54_2 Nr. of seq. 2 Alignment length(with gaps) = 54
Alignment score = 0.632716
GCF_001420595.1_ASM142059v1_genomic.fna_11:2718654-2718914 Satlength=261 Nr of Repeats=5 RepeatLength=52 seed=TCTTGTTACC Num.seqs=5
Similarity=0.912821   0
TCTTGTTACCGTGAACTCACT-TTTTCTGCTTTCAGGGGTACATGACACA-CAC
GCF_001420595.1_ASM142059v1_genomic.fna_11:4652534-4652852 Satlength=319 Nr of Repeats=6 RepeatLength=53 seed=TCTTGTTACC Num.seqs=6
Similarity=0.902725   0
TCTTGTTACCGCGAACACTCTCTTTTCTACTTTCTTGGGCACATGTGGCATCT-

*********** **** * ** ****** ***** *** *****   ** *
Consensus:

TCTTGTTACCGcGAACaCaCTcTTTTCTaCTTTCagGGGcACATGacaCAtCac

>Bacillus_Fam_458_54_2 Nr. of seq. 2 Alignment length(with gaps) = 54
Alignment score = 0.635802
GCF_001420715.1_ASM142071v1_genomic.fna_22:875387-875647 Satlength=261 Nr of Repeats=5 RepeatLength=52 seed=TGTCCTTCAT Num.seqs=5
Similarity=0.546667   0
TGTCCTTCATAG-TGNTAATGAAGGACAAGTTNGAAGGAAA-AATAAGTACAGA
GCF_001420715.1_ASM142071v1_genomic.fna_22:1038345-1038761 Satlength=417 Nr of Repeats=8 RepeatLength=52 seed=ATGAAGGACA Num.seqs=8
Similarity=0.602293   18
TGTCCTTCATAGACGGTNATGAAGGACAAATTCGNAAAAAAGAATAGGCAANGC

************ * * *********** ** * * *** **** * * *
Consensus:

TGTCCTTCATAGacGgTaATGAAGGACAAaTTcGaAaaAAAgAATAaGcAaaGa

>Bacillus_Fam_459_54_2 Nr. of seq. 2 Alignment length(with gaps) = 54
Alignment score = 0.722222
GCF_001591445.1_ASM159144v1_genomic.fna_1:86829-87037 Satlength=209 Nr of Repeats=4 RepeatLength=52 seed=CTCTATGTTA Num.seqs=4 Similarity=0.846154
0   CTCTATGTTACCGTCAACCGCTTTTTTCTT-GCTCCACGGTCACTTA-AAGCCT
GCF_001591445.1_ASM159144v1_genomic.fna_74:6946-7154 Satlength=209 Nr of Repeats=4 RepeatLength=52 seed=TATGTTACCG Num.seqs=4 Similarity=0.615421
3   CTTTATGTTACCGTACACTTC-TTTTTCTTCNCTGCACGGTCACTTAGAAGCCT

** *********** ** * ******** ** ************ ******
Consensus:

CTcTATGTTACCGTaaACcgCtTTTTTCTTcgCTcCACGGTCACTTAgAAGCCT

>Bacillus_Fam_460_54_2 Nr. of seq. 2 Alignment length(with gaps) = 54
Alignment score = 0.608025
GCF_001591485.1_ASM159148v1_genomic.fna_6:19015-20109 Satlength=1095 Nr of Repeats=20 RepeatLength=52 seed=TGTCCTTCAT Num.seqs=12
Similarity=0.790210 0
TGTCCTTCATTCCACTGATGAAGGACATTTTCTCACGA--CACAACCATGGTA
Rev.of_GCF_001636315.1_ASM163631v1_genomic.fna_1:3151872-3152287
Satlength=416 Nr of Repeats=8 RepeatLength=52 seed=AAATGGTCTT Num.seqs=7
Similarity=0.843712 29 -
GGCCTTCATTCCGCTTATGAA-GACCATTTTCACTCGAGCCACAACCTTTTTT

* ********** ** ***** *** ***** * *** ******* * *
Consensus:

tGgCCTTCATTCCaCTgATGAAgGACaaTTTTCaCaCGAgcCACAACCaTggTa

>Bacillus_Fam_461_54_2 Nr. of seq. 2 Alignment length(with gaps) = 54
Alignment score = 0.617284
GCF_001591585.1_ASM159158v1_genomic.fna_16:64946-65206 Satlength=261 Nr of Repeats=5 RepeatLength=52 seed=TCTCTCACTT Num.seqs=5
Similarity=0.948718
16   CCA-CTTTTTCCTTTCCT-CTCTCACTTTTCGGCTTTCATCAANCTCCTGTACA
GCF_001591585.1_ASM159158v1_genomic.fna_21:8882-9244 Satlength=363 Nr of Repeats=7 RepeatLength=52 seed=GCTCTCCTTT Num.seqs=5 Similarity=0.892308
17   CCATCTTTCT-CTTCCCTGCTCTC-CTTTTTGGTTTTCATCANCGTTATGAACA

* ***** ***** ** ********   * ** ****** **** * *** **
Consensus:

TgCTCTCaCTTTTcGGcTTTCATCAaccTcaTGaACACCAtCTTTcTcCTTcCC

>Bacillus_Fam_462_54_2 Nr. of seq. 2 Alignment length(with gaps) = 54
Alignment score = 0.608025
GCF_001591645.1_ASM159164v1_genomic.fna_1:794104-794312 Satlength=209 Nr of Repeats=4 RepeatLength=52 seed=AACGTTCTTC Num.seqs=4
Similarity=0.830713
0   AACGTTCTTCATCCGGCTTATGAAAGACGTTACGGAGCGATTTACTTGA--GCA

GCF_001591645.1_ASM159164v1_genomic.fna_4:234973-235232 Satlength=260 Nr of Repeats=5 RepeatLength=52 seed=TGATTGAAAC Num.seqs=4 Similarity=0.718254
44   -ACGTTCTTCATCAGGAGGATGAAAGACGTTAGGGTGTGA-ATAAATGATTGAA

************ **   ************* ** * ** ** *** * *
Consensus:

aACGTTCTTCATCaGGaggATGAAAGACGTTAcGGaGcGAtaTAaaTGAttGaA

>Bacillus_Fam_463_54_2 Nr. of seq. 2 Alignment length(with gaps) = 54
Alignment score = 0.709877
GCF_001591665.1_ASM159166v1_genomic.fna_35:21709-21917 Satlength=209 Nr of Repeats=4 RepeatLength=52 seed=ATCGAAGTAG Num.seqs=4
Similarity=0.807692
0   ATCGAAGTAGACAG-ATTCTACTTCAACTTTCTAG-TCTAAAATCCAGGGAAAC
Rev.of_GCF_001591665.1_ASM159166v1_genomic.fna_68:10391-10651
Satlength=261 Nr of Repeats=5 RepeatLength=52 seed=CTTCTACTTC Num.seqs=5
Similarity=0.828205   13
GTCGAAGTAGA-AGTGCTCTACTTCAACATTCANGCTC-AAAATCCAGGGAAAC

********** **   *********** *** * ** ***************
Consensus:

aTCGAAGTAGAcAGtacTCTACTTCAACaTTCaaGcTCtAAAATCCAGGGAAAC

>Bacillus_Fam_464_54_2 Nr. of seq. 2 Alignment length(with gaps) = 54
Alignment score = 0.682099
GCF_001591805.1_ASM159180v1_genomic.fna_10:65143-65350 Satlength=208 Nr of Repeats=4 RepeatLength=52 seed=TTTTTTCCGG Num.seqs=3
Similarity=0.948718
49   TTTCCGGGCGACGGGCACTATGAACCTCTTATAGTGCCCATGAACG-CTC-TTT
Rev.of_GCF_001591805.1_ASM159180v1_genomic.fna_16:206404-206612
Satlength=209 Nr of Repeats=4 RepeatLength=52 seed=AGGTTCATAG Num.seqs=4
Similarity=0.897436   79
TTTCCTGTCCGCGGGCACTATGAACCTTTTATAGTGCCC--GTTCGTGTCTTTT

******** * * **************** *********** * ** **
Consensus:

TTTTTTCCgGgCcaCGGGCACTATGAACCTcTTATAGTGCCCatGaaCGtcTCt

>Bacillus_Fam_465_54_2 Nr. of seq. 2 Alignment length(with gaps) = 54
Alignment score = 0.604938
GCF_001636315.1_ASM163631v1_genomic.fna_1:2650478-2651310 Satlength=833 Nr of Repeats=16 RepeatLength=52 seed=TGCTGTCTGA Num.seqs=14
Similarity=0.845590   30
AGTATAGAGAGCAAGTAGAAAGGCGCTAGGTGCTGTCTGAAAAG--GAAAATTG
GCF_001636315.1_ASM163631v1_genomic.fna_1:3094934-3095142 Satlength=209 Nr of Repeats=4 RepeatLength=52 seed=CAAGTAGAAG Num.seqs=4
Similarity=0.863248   63
AATTTAGAGATCAAGTAGAAGCCCTCTAGCTGCTGTCT--AAAGTCAAAAATCC

******** ****   ***** * * ****** *********   * ****

Consensus:

TGCTGTCTgaAAAGtcaAAAATccAaTaTAGAGAgCAAGTAGAAaccCgCTAGc

>Bacillus_Fam_466_54_2 Nr. of seq. 2 Alignment length(with gaps) = 54
Alignment score = 0.703704
GCF_001636315.1_ASM163631v1_genomic.fna_1:3142771-3143187 Satlength=417
Nr of Repeats=8 RepeatLength=52 seed=CTATAGGCGA Num.seqs=8
Similarity=0.869963 0
CTATAGGCGACCGAGAG-GCGAGAAAAAAAGTGAGGA-GGGTAGCGTATAAGGA
GCF_001636315.1_ASM163631v1_genomic.fna_1:3843104-3843416 Satlength=313
Nr of Repeats=6 RepeatLength=52 seed=TAGGCGACCG Num.seqs=6
Similarity=0.844864 3
TTATAGGCGACCGAGAGAGCG-G-AAAAAAGAGATTACGGGTAGCGTAGAAGGG

**************** *** * ******* ** * ********** ****
Consensus:

cTATAGGCGACCGAGAGaGCGaGaAAAAAAGaGAggAcGGGTAGCGTAgAAGGa

>Bacillus_Fam_467_54_2 Nr. of seq. 2 Alignment length(with gaps) = 54
Alignment score = 0.697531
GCF_001636315.1_ASM163631v1_genomic.fna_1:3535170-3535534 Satlength=365
Nr of Repeats=7 RepeatLength=52 seed=GTTGTAATTT Num.seqs=7
Similarity=0.894994 17
CCATTGGAAACACTTTGGTTGTAATTTT--CTAGAGTATTGTCGGCAATAGCCG
GCF_001636315.1_ASM163631v1_genomic.fna_1:3851718-3851925 Satlength=208
Nr of Repeats=4 RepeatLength=52 seed=CTATTGGAAA Num.seqs=3
Similarity=0.897436 52
CTATTGGAAACACTTTG--TCCAATTTTAACTCAAGTATTGTCGCCAATACCGG

* ****** ** ********** ***** * ** ***************
Consensus:

gtTccAATTTTaaCTaaAGTATTGTCGcCAATAcCcGCcATTGGAAACACTTTG

>Bacillus_Fam_468_54_2 Nr. of seq. 2 Alignment length(with gaps) = 54
Alignment score = 0.611111
GCF_001636325.1_ASM163632v1_genomic.fna_1:372049-372413 Satlength=365 Nr
of Repeats=7 RepeatLength=52 seed=ATCGCCCGTT Num.seqs=7
Similarity=0.791255
0 ATCGCCCGTTCGCTGCGNAAAGNTCCA-CAGAAGGTAGAAGAGAAGCGT-CCTC
Rev.of_GCF_001636325.1_ASM163632v1_genomic.fna_1:2619668-2620033
Satlength=366 Nr of Repeats=7 RepeatLength=52 seed=TTCGACCTTC Num.seqs=6
Similarity=0.852991 39
ATCGCCCGTTCGTCGTCCAAAG-TATAGCAGAAGGTCGAATAGAAG-GTGTCTC

************ * **** * * ******** *** ***** ** ***
Consensus:

ATCGCCCGTTCGccGcccAAAGnTacAgCAGAAGGTaGAAgAGAAGcGTgcCTC

>Bacillus_Fam_469_54_2 Nr. of seq. 2 Alignment length(with gaps) = 54
Alignment score = 0.623457

GCF_001636335.1_ASM163633v1_genomic.fna_1:484026-484390 Satlength=365 Nr of Repeats=7 RepeatLength=52 seed=AAAGTCCATC Num.seqs=7 Similarity=0.904762
0   AAAGTCCATCATAGCGTTGATGATGGACAAAACGCGGTGTGAAAGC--AAATGC
Rev.of_GCF_001636335.1_ASM163633v1_genomic.fna_1:2460494-2460702 Satlength=209 Nr of Repeats=4 RepeatLength=52 seed=CTTTTTCCGT Num.seqs=4 Similarity=0.974359   2
AGAGTCCGTCATCAAGGTGATGATGGACAAAACGTGGTCAGAAAACGGAAAA--

* ***** ****   * **************** *** **** * ***
Consensus:

AaAGTCCaTCATaaaGgTGATGATGGACAAAACGcGGTcaGAAAaCggAAAagc

>Bacillus_Fam_470_54_2 Nr. of seq. 2 Alignment length(with gaps) = 54 Alignment score = 0.679012
GCF_001887185.1_ASM188718v1_genomic.fna_332:91302-91717 Satlength=416 Nr of Repeats=8 RepeatLength=52 seed=GGAGGATATT Num.seqs=7 Similarity=0.843712
0   GGAGG-A-TATTCCGTGTCTAGGGTAACCAAAGAGCGTGATAGATTCCCCTGTC
GCF_002009555.1_ASM200955v1_genomic.fna_568:13298-13506 Satlength=209 Nr of Repeats=4 RepeatLength=52 seed=GTGTGCAGGG Num.seqs=4 Similarity=0.732510
14   GGAGGAACCAATCCGTGTGCAGGGTAACCAAAGAACGTGTTAGATTACCCTAAA

***** * * ******* ************** **** ****** ****
Consensus:

GGAGGaAccAaTCCGTGTccAGGGTAACCAAAGAaCGTGaTAGATTaCCCTaaa

>Bacillus_Fam_471_54_2 Nr. of seq. 2 Alignment length(with gaps) = 54 Alignment score = 0.604938
GCF_900104555.1_IMG-taxon_2651870172_annotated_assembly_genomic.fna_19:74323-74582 Satlength=260 Nr of Repeats=5 RepeatLength=52 seed=GTTTTGTCTA Num.seqs=4 Similarity=0.846154   0   GTTTTGTCTAATAGGAGCGC-T-CTATCGGACAAACTAAGCTCTCTGGCCTCCT
GCF_900104555.1_IMG-taxon_2651870172_annotated_assembly_genomic.fna_20:569803-570062 Satlength=260 Nr of Repeats=5 RepeatLength=52 seed=GTTTTGTCTG Num.seqs=4 Similarity=0.807692   0   GTTTTGTCTGATA-GA-CGCTTCCTATTAGACAAACTCTCCTCCCAGACCACCT

********* *** ** *** * **** ********   *** * * ** ***
Consensus:

GTTTTGTCTaATAgGAgCGCtTcCTATcaGACAAACTaacCTCcCaGaCCaCCT

>Bacillus_Fam_472_54_2 Nr. of seq. 2 Alignment length(with gaps) = 54 Alignment score = 0.666667
GCF_900107275.1_IMG-taxon_2675903024_annotated_assembly_genomic.fna_29:32661-32921 Satlength=261 Nr of Repeats=5 RepeatLength=52 seed=GCCGCTTCCA Num.seqs=5

Similarity=0.923077 0 GCCGCTTC-CAAGGCT-GTTTCGAACACGATTAGCTTCTATTCGTTAACGTTCA
Rev.of_GCF_900107275.1_IMG-taxon_2675903024_annotated_assembly_genomic.fna_42:756073-756385 Satlength=313 Nr of Repeats=5 RepeatLength=52 seed=CGAAAGTAGT Num.seqs=4 Similarity=0.931624 21 GCGGCTTCTCAA--CTACTTTCGATCACGATTAGCTCCTTTTCTTTTACGTACA

** ***** *** ** ****** *********** ** *** ** **** **
Consensus:

GCcGCTTCtCAAggCTacTTTCGAaCACGATTAGCTcCTaTTCgTTaACGTaCA

>Bacillus_Fam_473_54_2 Nr. of seq. 2 Alignment length(with gaps) = 54
Alignment score = 0.654321
GCF_000181495.1_ASM18149v1_genomic.fna_106:8005-8522 Satlength=518 Nr of Repeats=8 RepeatLength=53 seed=TCGAAAAGAA Num.seqs=5 Similarity=0.939623
0 TCGAAAAGAAGCTAAACGATCCAATGAGAAGGGGAAACCGAGTGGATGTAGG-G
GCF_000181495.1_ASM18149v1_genomic.fna_107:1831-2096 Satlength=266 Nr of Repeats=5 RepeatLength=53 seed=TGAAAAAGAG Num.seqs=5 Similarity=0.899371
0 T-GAAAAAGAGGTAACCGCCCCAACGAGAAAGGGAAACCAAGCGGATGTTGGTG

* ***** ** *** ** **** ***** ******** ** ****** ** *
Consensus:

TcGAAAAaaAGcTAAaCGacCCAAcGAGAAaGGGAAACCaAGcGGATGTaGGtG

>Bacillus_Fam_474_54_2 Nr. of seq. 2 Alignment length(with gaps) = 54
Alignment score = 0.635802
GCF_000181495.1_ASM18149v1_genomic.fna_139:7643-8120 Satlength=478 Nr of Repeats=9 RepeatLength=53 seed=TGAAATGATG Num.seqs=9 Similarity=0.769818
0 TGAAATGATGTCCCGCGCCTGGC-GAGCGCATGACATCATTATGAAGTTCGTNA
Rev.of_GCF_000181495.1_ASM18149v1_genomic.fna_186:108256-108627 Satlength=372 Nr of Repeats=7 RepeatLength=53 seed=GCGGTACATC Num.seqs=7 Similarity=0.828691 16
TGCAATGATGTACCGCG-CTGACTGTGCGCATGGCATCATTTTGTGATTCCTGT

** ******** ***** *** * * ******* ******* ** *** *
Consensus:

TGaAATGATGTaCCGCGcCTGaCtGaGCGCATGaCATCATTaTGaaaTTCcTga

>Bacillus_Fam_475_54_2 Nr. of seq. 2 Alignment length(with gaps) = 54
Alignment score = 0.709877
GCF_000181495.1_ASM18149v1_genomic.fna_170:14885-15361 Satlength=477 Nr of Repeats=8 RepeatLength=53 seed=ATGTTGGTCT Num.seqs=5 Similarity=0.738272
0 ATGTTGGTCTGATTAGGATGTTTTCAGNCCAATGAGACTCAATATTC-AAGGAG
GCF_000181495.1_ASM18149v1_genomic.fna_183:28650-29233 Satlength=584 Nr of Repeats=10 RepeatLength=53 seed=GAGCTAATCA Num.seqs=9 Similarity=0.796296
16 ATGTTGGTCTGTTTAGGAGCTAATCAGGCCAGTGAGATTCAATTTTCGAAGG-G

*********** ****** * **** *** ***** ***** *** **** *

Consensus:

ATGTTGGTCTGaTTAGGAgcTaaTCAGgCCAaTGAGAcTCAATaTTCgAAGGaG

>Bacillus_Fam_476_54_2 Nr. of seq. 2 Alignment length(with gaps) = 54
Alignment score = 0.827160
GCF_000708755.2_ASM70875v2_genomic.fna_1:195812-196130 Satlength=319 Nr of Repeats=6 RepeatLength=53 seed=TTATCGGCTT Num.seqs=6
Similarity=0.755556
0 TTATCGGCTTGATTAGAACCATCAA-GGAAAGGAAGAAGCATGCTTGAGGGTAC
GCF_000709935.2_ASM70993v2_genomic.fna_1:196645-196910 Satlength=266 Nr of Repeats=5 RepeatLength=53 seed=TGATTAGAAC Num.seqs=5
Similarity=0.861635
9 TTATCGGCGTGATTAGAACCACCAAGGGACAGG-AGAAGCATGCTTGAGGGTAT

******** *********** *** *** *** ******************
Consensus:

TTATCGGCgTGATTAGAACCAcCAAgGGAaAGGaAGAAGCATGCTTGAGGGTAc

>Bacillus_Fam_477_54_2 Nr. of seq. 2 Alignment length(with gaps) = 54
Alignment score = 0.913580
GCF_000708755.2_ASM70875v2_genomic.fna_6:313159-313635 Satlength=477 Nr of Repeats=9 RepeatLength=53 seed=GACTTGTGGC Num.seqs=8
Similarity=0.765983
0 GACTTGTGGCACCACTAGGCGGAGTGATACCACTTTTCACTCTTCCA-CCACCG
GCF_000709935.2_ASM70993v2_genomic.fna_5:520546-520916 Satlength=371 Nr of Repeats=7 RepeatLength=53 seed=TGTGGCACCA Num.seqs=6
Similarity=0.747325
4 GACTTGTGGCACCACTAGGCGGAGTGACACCACTTTTCACTCTTCCANCCATCG

*************************** ****************** *** **
Consensus:

GACTTGTGGCACCACTAGGCGGAGTGAcACCACTTTTCACTCTTCCAnCCAcCG

>Bacillus_Fam_478_54_2 Nr. of seq. 2 Alignment length(with gaps) = 54
Alignment score = 0.669753
GCF_001420715.1_ASM142071v1_genomic.fna_22:1363621-1363829 Satlength=209 Nr of Repeats=4 RepeatLength=52 seed=ATGAAGGACA Num.seqs=4
Similarity=0.570988 40
TGAAGCAAGAGAAAGA-AGGAAGTGTCCTTCACAAAGGTTGATGAAGGACAAAA
Rev.of_GCF_001591485.1_ASM159148v1_genomic.fna_34:25092-25304 Satlength=213 Nr of Repeats=4 RepeatLength=53 seed=CCCTTCATGA Num.seqs=4
Similarity=0.907757 91
CTAAGGAAGAGAAGGATAAAAATTGTCCTTCATGAAGGGTA-TGAAGGACAAAA

************ *** ******* ** * ** ********* **** *
Consensus:

aTGAAGGACAAAAcgAAGcAAGAGAAaGAtAaaAAgTGTCCTTCAcaAAGGgTa

>Bacillus_Fam_479_54_2 Nr. of seq. 2 Alignment length(with gaps) = 54
Alignment score = 0.657407

GCF_001439915.1_ASM143991v1_genomic.fna_83:403240-403615 Satlength=376 Nr of Repeats=8 RepeatLength=53 seed=CAATTTGGAG Num.seqs=5 Similarity=0.851852 0
CAATTTGGAGGTCCACCATATTCCAATAATTAGGAGAATGGTGCCCCTCGAAN-
GCF_001439915.1_ASM143991v1_genomic.fna_84:151113-151792 Satlength=680 Nr of Repeats=13 RepeatLength=53 seed=GAAGGTCACT Num.seqs=11 Similarity=0.830303 6
NGTTTTGAAGGT-CACTATATACCAGTAATTAGGAGAATAGTGCCACTCCATTT

**** **** *** **** *** ************* ***** *** *
Consensus:

caaTTTGaAGGTcCACcATATaCCAaTAATTAGGAGAATaGTGCCaCTCcAatt

>Bacillus_Fam_480_54_2 Nr. of seq. 2 Alignment length(with gaps) = 54 Alignment score = 0.790123
GCF_002019605.1_ASM201960v1_genomic.fna_1:3068298-3068510 Satlength=213 Nr of Repeats=4 RepeatLength=53 seed=TCATAGTGAC Num.seqs=4 Similarity=0.844864 0
TCATAGTGACCGTCCGAGCAGGAAACCAGGTGAGAGCGGTCACTAT-CAAGAGC
GCF_002019605.1_ASM201960v1_genomic.fna_1:3905986-3906776 Satlength=791 Nr of Repeats=15 RepeatLength=53 seed=GCTCATAGTG Num.seqs=10 Similarity=0.746296 52
TCATAGTGCCCATCCGAGAAGGAAATCAGGTGAGTACGGTCACTATGAAAGAGC

******** ** ****** ****** ******** ********** ******
Consensus:

TCATAGTGaCCaTCCGAGaAGGAAAcCAGGTGAGaaCGGTCACTATgaAAGAGC

>Bacillus_Fam_481_54_2 Nr. of seq. 2 Alignment length(with gaps) = 54 Alignment score = 0.882716
GCF_001887185.1_ASM188718v1_genomic.fna_148:138342-138666 Satlength=325 Nr of Repeats=6 RepeatLength=54 seed=TATAACAAAT Num.seqs=4 Similarity=0.868313 0
TATAACAAATATAGTAGCAGAACGAGGAATCGTATCCCCAGCACCAGTAAATAG
GCF_002009555.1_ASM200955v1_genomic.fna_427:1374-1752 Satlength=379 Nr of Repeats=7 RepeatLength=54 seed=GCACCAGTAA Num.seqs=7 Similarity=0.891828 40   TATAACAAATGTAGTAGCGGAGCGAGGAATCGTGTCNCCAGCACCAGTAAATAG

********** ******* ** *********** ** *****************
Consensus:

TATAACAAATaTAGTAGCaGAaCGAGGAATCGTaTCcCCAGCACCAGTAAATAG

>Bacillus_Fam_482_53_2 Nr. of seq. 2 Alignment length(with gaps) = 53 Alignment score = 0.672956
GCF_000181495.1_ASM18149v1_genomic.fna_116:6074-6381 Satlength=308 Nr of Repeats=6 RepeatLength=51 seed=AGGACGATAA Num.seqs=5 Similarity=0.900654 0   AGGACGATAAAATAAG-AAAATTAAAATTAAAGTACTATAGAGAGGTTT-TAA
GCF_000181495.1_ASM18149v1_genomic.fna_182:70379-70940 Satlength=562 Nr of Repeats=11 RepeatLength=51 seed=GAAATTAAAG Num.seqs=9 Similarity=0.963689
22   AGGAC-ATTAAAAAAGCAAAAGCGAAATTAAAGTCCTATA-AAAGGTTTCTAA

***** ** *** *** ****   ********** ***** * ****** ***
Consensus:

AGGACgATaAAAaAAGcAAAAgcaAAATTAAAGTaCTATAgAaAGGTTTcTAA

>Bacillus_Fam_483_53_2 Nr. of seq. 2 Alignment length(with gaps) = 53
Alignment score = 0.676101
GCF_000181495.1_ASM18149v1_genomic.fna_183:116608-117220 Satlength=613 Nr of Repeats=12 RepeatLength=51 seed=AAACTCCAAA Num.seqs=10
Similarity=0.941903   0
AAACTCCAAATTATCGTCCTTTAAAACGGC-TCTATAAGACATTAAT-TACTC
Rev.of_GCF_000181495.1_ASM18149v1_genomic.fna_186:301034-301853
Satlength=820 Nr of Repeats=16 RepeatLength=51 seed=TTTAAAGGAC
Num.seqs=11 Similarity=0.656718
24   -CACCCCAANTTATCGTCCTTT-AAAGGGCTTCTATAGGACGTTAATCTCCTN

** **** ************ *** *** ****** *** ***** * **
Consensus:

aaACcCCAAaTTATCGTCCTTTaAAAcGGCtTCTATAaGACaTTAATcTaCTc

>Bacillus_Fam_484_53_2 Nr. of seq. 2 Alignment length(with gaps) = 53
Alignment score = 0.625786
GCF_000292245.2_ASM29224v2_genomic.fna_23:12860-13115 Satlength=256 Nr of Repeats=5 RepeatLength=51 seed=TTCATTACCT Num.seqs=5 Similarity=0.755128
0   TTCATTACCTATTCCTGACTATTCGCACCTTAATTTTGCA--TTCGCCGTGGT
GCF_000292245.2_ASM29224v2_genomic.fna_87:12883-13293 Satlength=411 Nr of Repeats=8 RepeatLength=51 seed=CTTTTTTCAT Num.seqs=6 Similarity=0.904139
45   -TCATTACTTATCCTTTGTTATTCGCACCTTAATTTGGCACGTTC-CCTTTTT

******* *** * *   **************** *** *** ** * *
Consensus:

tTCATTACcTATcCcTgacTATTCGCACCTTAATTTgGCAcgTTCgCCgTggT

>Bacillus_Fam_485_53_2 Nr. of seq. 2 Alignment length(with gaps) = 53
Alignment score = 0.685535
GCF_000752035.1_Bacillus_rubiinfantis_genomic.fna_11:92768-93125
Satlength=358 Nr of Repeats=7 RepeatLength=51 seed=TTTTTGTCCA Num.seqs=7
Similarity=0.915344   0   T-
TTTTGTCCAATAGACCGCTGCTATCGGACAACTT-TTTCCGAAAACAGGCT
GCF_900104555.1_IMG-
taxon_2651870172_annotated_assembly_genomic.fna_22:525505-525906
Satlength=402 Nr of Repeats=8 RepeatLength=52 seed=GTTTTGTCCA Num.seqs=6
Similarity=0.831687   1
TGTTTTGTCCAATAGACCGCCCCTATCGAACAAATTCTTTCCCCAGAC-CGCT

* ***************** ****** **** ** ***** * ** ***
Consensus:

TgTTTTGTCCAATAGACCGCccCTATCGaACAAaTTcTTTCCcaAaACacGCT

>Bacillus_Fam_486_53_2 Nr. of seq. 2 Alignment length(with gaps) = 53
Alignment score = 0.635220
GCF_001636335.1_ASM163633v1_genomic.fna_1:554579-554937 Satlength=359 Nr of Repeats=7 RepeatLength=51 seed=AAAAAGGAGT Num.seqs=6
Similarity=0.877996
0 AAAAAGGAGTGACTGAAAAATAGGTAGTTAAG--AGGCGTTTAAAACTCCCGA
Rev.of_GCF_001636335.1_ASM163633v1_genomic.fna_1:1522005-1522515
Satlength=511 Nr of Repeats=10 RepeatLength=51 seed=GGGCGTTATA
Num.seqs=10 Similarity=0.911111
49 AAATCGGAGAATCAGAAAAATTGGTAGTTAAGCCA-GC-TTTATAACGCCCGA

*** **** * ******* ********** * ** **** *** *****
Consensus:

AAAaaGGAGaaaCaGAAAAATaGGTAGTTAAGccAgGCgTTTAaAACgCCCGA

>Bacillus_Fam_487_53_2 Nr. of seq. 2 Alignment length(with gaps) = 53
Alignment score = 0.679245
GCF_000169195.2_ASM16919v2_genomic.fna_1:334661-335181 Satlength=521 Nr of Repeats=9 RepeatLength=52 seed=TGAAGGACAT Num.seqs=8
Similarity=0.706570
0 TGAAGGACATTTTAATG-GTTCGTGGCTCGCGTTTTGTCCTTCATTANCCCGA
GCF_001636335.1_ASM163633v1_genomic.fna_1:3219373-3219737 Satlength=365
Nr of Repeats=7 RepeatLength=52 seed=ACTTTTTGAC Num.seqs=7
Similarity=0.936508 6
TGAAGGACTTTTTGACGTGCCCCTTGCTC-CGCTTTGTCCTTCATTCACCCGA

******** **** * * * * * **** ** ************* *****
Consensus:

TGAAGGACaTTTTaAcGtGcccCcTgGCTCgCGcTTTGTCCTTCATTaaCCCGA

>Bacillus_Fam_488_53_2 Nr. of seq. 2 Alignment length(with gaps) = 53
Alignment score = 0.613208
GCF_000169195.2_ASM16919v2_genomic.fna_1:636213-636421 Satlength=209 Nr of Repeats=4 RepeatLength=52 seed=GTTCCTTAAC Num.seqs=4
Similarity=0.806080
0 GTTCCTTAACGTAGAACCACTCTATGTGAACTTATGACCGGATTTTTCGTCT-
GCF_000169195.2_ASM16919v2_genomic.fna_1:1145839-1146151 Satlength=313 Nr of Repeats=6 RepeatLength=52 seed=TCTATGTTAA Num.seqs=6
Similarity=0.768485 20
CTTCCTTAACGTAGAGCCTCTCTATGTTAAGATA-CACGGCAGATTTCAGCTG

************** ** ******** ** ** ** * * **** **
Consensus:

cTTCCTTAACGTAGAaCCaCTCTATGTgAAcaTAtcACcGcAgaTTTCagCTg

>Bacillus_Fam_489_53_2 Nr. of seq. 2 Alignment length(with gaps) = 53
Alignment score = 0.628931
GCF_000169195.2_ASM16919v2_genomic.fna_1:2292482-2293472 Satlength=991 Nr of Repeats=19 RepeatLength=52 seed=AATGTCTTTT Num.seqs=18
Similarity=0.800551 0
AATGTCTTTTACAANGGCTCAAAAGGACAAAAG-GGTGCACGTTGCCGGTTGA

GCF_000217835.1_ASM21783v1_genomic.fna_1:954299-954610 Satlength=312 Nr of Repeats=6 RepeatLength=52 seed=AAAAGACAAA Num.seqs=5 Similarity=0.805128
21   ACTGTCTTTTACAACGGTTCAAAAAGACAAAAGCGTTCCAGCTTTCC-ACCGA

* ************ ** ****** ******** * * ** ** **   **
Consensus:

AaTGTCTTTTACAAcGGcTCAAAAaGACAAAAGcGgTcCAccTTgCCgaccGA

>Bacillus_Fam_490_53_2 Nr. of seq. 2 Alignment length(with gaps) = 53
Alignment score = 0.622642
GCF_000175075.1_ASM17507v1_genomic.fna_5:15548-15860 Satlength=313 Nr of Repeats=6 RepeatLength=52 seed=GTCAGAGAGG Num.seqs=6 Similarity=0.822428
0   GTCAGAGAGGTCTTGATAGCGGTTATGAGGACATCTG-TTGGTGAGAAATGAG
GCF_000175075.1_ASM17507v1_genomic.fna_33:121241-121605 Satlength=365 Nr of Repeats=7 RepeatLength=52 seed=TTATGAAGAC Num.seqs=7 Similarity=0.838828
21   G-GAGAGAGGTCTTCATCGCACTTATGAAGACGTCAGTTTGGAGAGAAAGGCC

* *********** ** ** ****** *** ** * **** ****** *
Consensus:

GtcAGAGAGGTCTTcATaGCacTTATGAaGACaTCaGtTTGGaGAGAAAgGac

>Bacillus_Fam_491_53_2 Nr. of seq. 2 Alignment length(with gaps) = 53
Alignment score = 0.672956
GCF_000175075.1_ASM17507v1_genomic.fna_29:40755-41014 Satlength=260 Nr of Repeats=5 RepeatLength=52 seed=TCTCTGTTAC Num.seqs=4 Similarity=0.948718
41   G-AACGCCATTGTTTTTCGGGGCTTCGGGCTTTGAGACGCCCTCTCTGTTACC
GCF_000175075.1_ASM17507v1_genomic.fna_29:166890-167254 Satlength=365 Nr of Repeats=7 RepeatLength=52 seed=CTCTCTGTTA Num.seqs=7 Similarity=0.794872
92   GTTTCCCTGCTGTTGCTCGGCG-TTCGGGCTTTGAGAGGCCCTCTCTGTTACC

************   * *   **** **** * ************** ****
Consensus:

TCTCTGTTACCGtaaCcCcacTGTTgcTCGGcGcTTCGGGCTTTGAGAcGCCC

>Bacillus_Fam_492_53_2 Nr. of seq. 2 Alignment length(with gaps) = 53
Alignment score = 0.638365
GCF_000175075.1_ASM17507v1_genomic.fna_32:50201-50513 Satlength=313 Nr of Repeats=6 RepeatLength=52 seed=TTCGGTCAAA Num.seqs=6 Similarity=0.829060
0   TTCGGTCAAATAGAAGGGCTCTATACGCCCGAAGCGGAAGTGAGACTGGGAG-
GCF_000175075.1_ASM17507v1_genomic.fna_34:151816-152128 Satlength=313 Nr of Repeats=6 RepeatLength=52 seed=GAGCTTCGGT Num.seqs=6 Similarity=0.837607
48   TTCGGTCGTATAGAAGGGCTCTATAAGCCTAAAGAGCAACT-ATAGCCGGAGC

******* *************** *** *** * ** * * *   ****
Consensus:

TTCGGTCaaATAGAAGGGCTCTATAaGCCcaAAGaGcAAcTgAgAcccGGAGc

>Bacillus_Fam_493_53_2 Nr. of seq. 2 Alignment length(with gaps) = 53
Alignment score = 0.660377
GCF_000175075.1_ASM17507v1_genomic.fna_32:253955-254216 Satlength=262 Nr of Repeats=5 RepeatLength=52 seed=ATGAAGACAT Num.seqs=4
Similarity=0.901709
0 ATGAAGACATCAGTAGCG-TGAAAACCGGTAGAGAGAGGTCTTGAGAAGAGGC
GCF_000175075.1_ASM17507v1_genomic.fna_36:46227-46487 Satlength=261 Nr of Repeats=5 RepeatLength=52 seed=AGAGAGGTCT Num.seqs=5 Similarity=0.823077
31 ATGAAGACATCTGTTGNNCTAATCACAGCT-GAGAGAGGTCTTGAGAAGTGGA

*********** ** *   * * ** * * ***************** **
Consensus:

ATGAAGACATCaGTaGcgcTaAaaACaGcTaGAGAGAGGTCTTGAGAAGaGGa

>Bacillus_Fam_494_53_2 Nr. of seq. 2 Alignment length(with gaps) = 53
Alignment score = 0.660377
GCF_000175075.1_ASM17507v1_genomic.fna_36:67347-67919 Satlength=573 Nr of Repeats=11 RepeatLength=52 seed=AGCGAATGAA Num.seqs=11
Similarity=0.862471
0 AGCGAATGAAGACATCAGTGAAGCAGGAAAC-GAGNNAGAGAAGTCTTCATGA
GCF_000175075.1_ASM17507v1_genomic.fna_43:58907-59115 Satlength=209 Nr of Repeats=4 RepeatLength=52 seed=GTTATGAAGA Num.seqs=4 Similarity=0.901709
2 AGGTTATGAAGACATGAGTG-ACCGGGAATCTGAGTGAGAGATGTCTTCATGG

**   ********** **** * * **** * *** ***** *********
Consensus:

AGcgaATGAAGACATcAGTGaAcCaGGAAaCtGAGtgAGAGAaGTCTTCATGa

>Bacillus_Fam_495_53_2 Nr. of seq. 2 Alignment length(with gaps) = 53
Alignment score = 0.647799
GCF_000175075.1_ASM17507v1_genomic.fna_47:55295-55555 Satlength=261 Nr of Repeats=5 RepeatLength=52 seed=GGGTCCTCAT Num.seqs=5 Similarity=0.835897
0 GGGTCCTCATAAGGCTGATGAAGACATGAGTTGGTT-GAATTTTGTGCGAGAG
Rev.of_GCF_900156865.1_PRJEB18960_genomic.fna_3:2588481-2588898
Satlength=418 Nr of Repeats=8 RepeatLength=52 seed=TCAGGTCTTC Num.seqs=7
Similarity=0.874237 29
AGGTTCTCATAAGTGTTATGAAGACCTGAG-TGGTTGGGATATTATTCGAAAG

*** ******** * ******** **** ***** * ** ** * *** **
Consensus:

aGGTcCTCATAAGgcTgATGAAGACaTGAGtTGGTTgGaATaTTaTgCGAaAG

>Bacillus_Fam_496_53_2 Nr. of seq. 2 Alignment length(with gaps) = 53
Alignment score = 0.713836
GCF_000177235.2_ASM17723v2_genomic.fna_1:3849630-3849838 Satlength=209 Nr of Repeats=4 RepeatLength=52 seed=GCTATTTTAG Num.seqs=4
Similarity=0.943920 0
GCTATTTTAGTTTGCTGCCCTTTCTTATGCTGTTCCTTACTTTGAATGGGCT-
GCF_000177235.2_ASM17723v2_genomic.fna_1:3977361-3977881 Satlength=521 Nr of Repeats=10 RepeatLength=52 seed=CACCTTACAA Num.seqs=10

Similarity=0.899145 36
GCTAATTTAATTTGCTGCCCTTTCTTATGCTTTTTCCACCTTACAAT-GGCTC

**** **** ******************** ** *   *** *** ****
Consensus:

GCTAaTTTAaTTTGCTGCCCTTTCTTATGCTgTTcCcaaCTTacAATgGGCTc

>Bacillus_Fam_497_53_2 Nr. of seq. 2 Alignment length(with gaps) = 53
Alignment score = 0.698113
GCF_000374345.1_ASM37434v1_genomic.fna_5:73203-73463 Satlength=261 Nr of Repeats=5 RepeatLength=52 seed=TTCATCGGCT Num.seqs=5 Similarity=0.797436
0 TTCATC-GGCTCTATGATCTACATTTCGAGCCATATTTTTGCCTGTGATAGCG
Rev.of_GCF_000374345.1_ASM37434v1_genomic.fna_9:34053-34313 Satlength=261 Nr of Repeats=5 RepeatLength=52 seed=ATGAACGCGA Num.seqs=5 Similarity=0.892308 5
TTCATCGGGCT-TATGATAGTCATTTTGAGCCTAAATTTTGTCTGTGATCGCG

****** **** ******   ***** ***** * ***** ******* ***
Consensus:

TTCATCgGGCTcTATGATagaCATTTcGAGCCaaAaTTTTGcCTGTGATaGCG

>Bacillus_Fam_498_53_2 Nr. of seq. 2 Alignment length(with gaps) = 53
Alignment score = 0.672956
GCF_000401235.1_BacNeaAAU1_genomic.fna_233:4508-4820 Satlength=313 Nr of Repeats=6 RepeatLength=52 seed=CCACTTTTTC Num.seqs=4 Similarity=0.884615
18 TCATCTCCTTTATGAACACCACTTTTTCCTTCTACT-ACTGCTTTTCTGGCTT
Rev.of_GCF_001591585.1_ASM159158v1_genomic.fna_13:67731-68094 Satlength=364 Nr of Repeats=7 RepeatLength=52 seed=AGAGGATGAA Num.seqs=6 Similarity=0.823932 61
TCATCCTCTCCATGAACACCACTTTTCCCTCTTGCTGACGGATTTT-TGGCTT

******** *** * ** ** * **** *********** ** *******
Consensus:

CCACTTTTcCCTccTaCTgACgGaTTTTcTGGCTTTCATCccCTccATGAACA

>Bacillus_Fam_499_53_2 Nr. of seq. 2 Alignment length(with gaps) = 53
Alignment score = 0.622642
GCF_000612625.1_JCE_genomic.fna_12:16124-17008 Satlength=885 Nr of Repeats=16 RepeatLength=52 seed=CGTGAACGAA Num.seqs=15 Similarity=0.856899
0 CGTGAACG-AAGAAAAAGATAAAAAACGGTAACTAGTAGAGGCGGCTAGTGCC
Rev.of_GCF_000612625.1_JCE_genomic.fna_97:29-341 Satlength=313 Nr of Repeats=6 RepeatLength=52 seed=CTCGGGCATT Num.seqs=6 Similarity=0.837607
4 CGAGGAGGCAGGAAAAAG-GAAAGGGCGGTAAGTAGTCGAGGCGACTAATGCC

** * * * * ******* ***   ****** **** ****** *** ****
Consensus:

CGaGaAcGcAaGAAAAAGagAAAaaaCGGTAAcTAGTaGAGGCGaCTAaTGCC

>Bacillus_Fam_500_53_2 Nr. of seq. 2 Alignment length(with gaps) = 53
Alignment score = 0.823899
GCF_000612665.1_FF4_genomic.fna_66:68659-68921 Satlength=263 Nr of Repeats=5 RepeatLength=52 seed=GTCACGTAGA Num.seqs=4 Similarity=0.893606
0   GTCACGTAGAGCCTGTCTACGTTACCCAAATCTAG-TCTTTTCAATGTTTACG
GCF_000612665.1_FF4_genomic.fna_72:16126-16334 Satlength=209 Nr of Repeats=4 RepeatLength=52 seed=TACGTTACCC Num.seqs=4 Similarity=1.000000
17   GTCACGTAGAGGCTGTCTACGTTACCCATATCCAGCTC-TTTCAATGTTTTCG

*********** *************** *** ** ** *********** **
Consensus:

GTCACGTAGAGcCTGTCTACGTTACCCAaATCcAGcTCtTTTCAATGTTTaCG

>Bacillus_Fam_501_53_2 Nr. of seq. 2 Alignment length(with gaps) = 53
Alignment score = 0.757862
GCF_000708755.2_ASM70875v2_genomic.fna_22:103405-103718 Satlength=314 Nr of Repeats=6 RepeatLength=52 seed=AATAGAACCA Num.seqs=5 Similarity=0.704321
0   AATAGAACCATCAAAACCGAAAATGAGCAGCTTGATGGCNCTAANCAGGCNG-
GCF_000709935.2_ASM70993v2_genomic.fna_24:214095-214304 Satlength=210 Nr of Repeats=4 RepeatLength=52 seed=AATAGAACCA Num.seqs=3 Similarity=0.708595
0   AATAGAACCATCAAAANCGTGAATCAAAAGCTTGATGGCNCTAAACAGGC-GT

*************** ** *** * *************** ***** *
Consensus:

AATAGAACCATCAAAAcCGaaAATcAaaAGCTTGATGGCNCTAAaCAGGCnGt

>Bacillus_Fam_502_53_2 Nr. of seq. 2 Alignment length(with gaps) = 53
Alignment score = 0.710692
GCF_000752395.1_Bacillus_andreraoultii_genomic.fna_41:44624-45040 Satlength=417 Nr of Repeats=8 RepeatLength=52 seed=TCTATGAGCC Num.seqs=8 Similarity=0.856151   0
TCTATGAGCCCGAAAATCAGGTG-AAGGATCAAAAATGGGTTCATAGACCGAA
Rev.of_GCF_000752395.1_Bacillus_andreraoultii_genomic.fna_24:138370-138786 Satlength=417 Nr of Repeats=8 RepeatLength=52 seed=GGGATCATAG Num.seqs=8 Similarity=0.711310   11
TCTATGATCCCAAAATTCGAGTGNAAAGAGCAAAAATTGGTTGATAGAACGAA

******* *** *** ** *** ** ** ******* **** ***** ****
Consensus:

TCTATGAgCCCaAAAaTCaaGTGnAAaGAgCAAAAATgGGTTcATAGAaCGAA

>Bacillus_Fam_503_53_2 Nr. of seq. 2 Alignment length(with gaps) = 53
Alignment score = 0.672956
GCF_000813125.1_ASM81312v1_genomic.fna_15:34784-35096 Satlength=313 Nr of Repeats=6 RepeatLength=52 seed=TGACAGCTTT Num.seqs=6 Similarity=0.786325
0   TGACAGCTTTTCTGGTGAATGA-GCCAAAGCTGTCATGATTCCTCCGTTATTC
GCF_000813125.1_ASM81312v1_genomic.fna_130:47-256 Satlength=210 Nr of Repeats=4 RepeatLength=52 seed=TGTCATGATT Num.seqs=3 Similarity=0.914530
30   TGACAGGTTTGATGATG-CTGAGGACAAAGCTGTCATGATTACACAGTTATTT

****** *** ** ** *** * *************** * * ******
Consensus:

TGACAGcTTTgaTGaTGaaTGAgGaCAAAGCTGTCATGATTaCaCaGTTATTc

>Bacillus_Fam_504_53_2 Nr. of seq. 2 Alignment length(with gaps) = 53
Alignment score = 0.679245
GCF_000813125.1_ASM81312v1_genomic.fna_25:31-239 Satlength=209 Nr of Repeats=4 RepeatLength=52 seed=GCTTTTCTTG Num.seqs=4 Similarity=0.805556
0   GC-TTTTCTTGACAGCTTTGGCCCCGTATCCTTGAAACCTGTCACAATAACTA
GCF_001315165.1_ASM131516v1_genomic.fna_8:68848-69212 Satlength=365 Nr of Repeats=7 RepeatLength=52 seed=GAATCTTGAC Num.seqs=7 Similarity=0.847375
3   NCGGAATCTTGACAGCTTTGGCCCCGGATCCAGAAAAGCTGTCACAAAAAC-A

*   ******************* ****   *** ********* *** *
Consensus:

gCggaaTCTTGACAGCTTTGGCCCCGgATCCagaAAAcCTGTCACAAaAACtA

>Bacillus_Fam_505_53_2 Nr. of seq. 2 Alignment length(with gaps) = 53
Alignment score = 0.723270
GCF_000813125.1_ASM81312v1_genomic.fna_119:16360-16620 Satlength=261 Nr of Repeats=5 RepeatLength=52 seed=ATGACGGACA Num.seqs=5 Similarity=0.844025
0   ATGACGGACACTTTTCCTCTCAA-TCTCGCTCTTTCTGTCCGTCATCACGTCT
Rev.of_GCF_001315165.1_ASM131516v1_genomic.fna_3:68869-69077 Satlength=209 Nr of Repeats=4 RepeatLength=52 seed=TGTCCGTCAT Num.seqs=4 Similarity=0.867521   10
ATGACGGACACTTTTCTTCTCAATTATCG-AATTTCTGTCCGTCATCGGGGTT

*************** ****** * ***   ************** * *
Consensus:

ATGACGGACACTTTTCcTCTCAAtTaTCGcaaTTTCTGTCCGTCATCacGgcT

>Bacillus_Fam_506_53_2 Nr. of seq. 2 Alignment length(with gaps) = 53
Alignment score = 0.622642
GCF_000813125.1_ASM81312v1_genomic.fna_122:25239-25553 Satlength=315 Nr of Repeats=5 RepeatLength=52 seed=GACATTTCCT Num.seqs=4 Similarity=0.841880
0   GACATTTCCTTGGGATTCTG-CCCCCTGTTTGGCCGATAGACCCCTTCTATCG
Rev.of_GCF_001315165.1_ASM131516v1_genomic.fna_117:8723-8931 Satlength=209 Nr of Repeats=4 RepeatLength=52 seed=TCTATCGGAC Num.seqs=4 Similarity=0.785115   40
GACAGAT-TTTAGGATATTGTCCTTCTGTTTGTCCGATAGAGCCCTTCTAATG

**** * ** **** ** ** ******* ******** ******** *
Consensus:

GACAgaTccTTaGGATacTGtCCccCTGTTTGgCCGATAGAcCCCTTCTAacG

>Bacillus_Fam_507_53_2 Nr. of seq. 2 Alignment length(with gaps) = 53
Alignment score = 0.610063

GCF_000813125.1_ASM81312v1_genomic.fna_134:91815-92127 Satlength=313 Nr of Repeats=6 RepeatLength=52 seed=AAAAACAGGG Num.seqs=6 Similarity=0.830769
0 AAAAACAGGGCACCAATACAGCCTATTGATGACCTAAAT-CTCGAAAATACGA
Rev.of_GCF_001420595.1_ASM142059v1_genomic.fna_11:1279366-1279626 Satlength=261 Nr of Repeats=5 RepeatLength=52 seed=TAGCGGATTT Num.seqs=5 Similarity=0.753846 2
TAATTC-GGTAACCAATACAGCNTATTGGCGACCTTANTCCTCCAAAATCCGC

** * ** *********** ***** ***** * * *** ***** **
Consensus:

aAAaaCaGGgaACCAATACAGCcTATTGacGACCTaAaTcCTCcAAAATaCGa

>Bacillus_Fam_508_53_2 Nr. of seq. 2 Alignment length(with gaps) = 53 Alignment score = 0.915094
GCF_000986785.1_ASM98678v1_genomic.fna_11:5-369 Satlength=365 Nr of Repeats=7 RepeatLength=52 seed=TCTCAGTGCC Num.seqs=7 Similarity=0.799341
0 TCTCAGTGCCCGAAATGTCTCCTGCCACTACAAANAGGTCACTGACANCTCC-
GCF_000986785.1_ASM98678v1_genomic.fna_16:123816-124024 Satlength=209 Nr of Repeats=4 RepeatLength=52 seed=AGGGCACTGA Num.seqs=4 Similarity=0.788260
35 TCTCAGTGCCCGAAATGTCTCCTGCCACTACAAACAGGGCACTGACACCTCCT

********************************* *** ******** ****
Consensus:

TCTCAGTGCCCGAAATGTCTCCTGCCACTACAAAcAGGgCACTGACAcCTCCt

>Bacillus_Fam_509_53_2 Nr. of seq. 2 Alignment length(with gaps) = 53 Alignment score = 0.628931
GCF_001315045.1_ASM131504v1_genomic.fna_27:2070-2330 Satlength=261 Nr of Repeats=5 RepeatLength=52 seed=TGTCCGAAGA Num.seqs=5 Similarity=0.838462
0 TGTCCGAAGAGAGAGNATCTTCGGACAGGAAAGGCGGAACAA-AGCGAAAAGC
GCF_001315045.1_ASM131504v1_genomic.fna_33:22668-22876 Satlength=209 Nr of Repeats=4 RepeatLength=52 seed=GTTCGGACAG Num.seqs=4 Similarity=0.722222
18 TGTCCGAAGAGAGTGCAAGTTCGGACA-GAAAACCGGGTGAAGAGCTAGAAGG

************* * * ******** **** *** ** *** * ***
Consensus:

TGTCCGAAGAGAGaGcAacTTCGGACAgGAAAacCGGaacAAgAGCgAaAAGc

>Bacillus_Fam_510_53_2 Nr. of seq. 2 Alignment length(with gaps) = 53 Alignment score = 0.622642
GCF_001315045.1_ASM131504v1_genomic.fna_43:12-220 Satlength=209 Nr of Repeats=4 RepeatLength=52 seed=AGTTCGGACA Num.seqs=4 Similarity=0.833333
0 AGTTCGGACAAGA-ATGAGGGGAAGAAGCAGAAAGTTGTCCGAAGAAGGCACA
Rev.of_GCF_001420595.1_ASM142059v1_genomic.fna_11:3566111-3566319 Satlength=209 Nr of Repeats=4 RepeatLength=52 seed=TCTTGTCCGA Num.seqs=4 Similarity=0.811966 13
AGTTCGGACAAGATTTCACTTGAATAAGC-CAAAGCAGTCCGAAGTGGGCTCA

************ * * *** **** **** ******** *** **
Consensus:

AGTTCGGACAAGAtaTcAcggGAAgAAGCacAAAGcaGTCCGAAGaaGGCaCA

>Bacillus_Fam_511_53_2 Nr. of seq. 2 Alignment length(with gaps) = 53
Alignment score = 0.650943
GCF_001315085.1_ASM131508v1_genomic.fna_1:106214-106474 Satlength=261 Nr of Repeats=5 RepeatLength=52 seed=AAAAGGTCAT Num.seqs=5 Similarity=0.828931
0   AAAAGGTCATCATCAAGCTGATGAAGA-CCGAAAGCATCTGGAAAGCTGAGAA
Rev.of_GCF_900156865.1_PRJEB18960_genomic.fna_3:3307735-3308723 Satlength=989 Nr of Repeats=18 RepeatLength=52 seed=CCTCTTTCTT Num.seqs=17 Similarity=0.808069
5   -AGAGGTCNTCATCAAGGTAATGAAGACCCGAAAGCANGTCCACCGCAAAGAA

* ***** ******** * ******* ********* * * ** ****
Consensus:

aAaAGGTCaTCATCAAGcTaATGAAGAcCCGAAAGCAtcTccAaaGCaaAGAA

>Bacillus_Fam_512_53_2 Nr. of seq. 2 Alignment length(with gaps) = 53
Alignment score = 0.628931
GCF_001420595.1_ASM142059v1_genomic.fna_11:4880278-4880695 Satlength=418 Nr of Repeats=8 RepeatLength=52 seed=GACTTCGGAC Num.seqs=7 Similarity=0.776280 0
GACTTCGGAC-TTGGTTCAGCTTTTTCCTCTTGTACCTGTCTGAACTCGGGTN
GCF_900104555.1_IMG-taxon_2651870172_annotated_assembly_genomic.fna_19:196185-196445 Satlength=261 Nr of Repeats=5 RepeatLength=52 seed=TTCAGACAAT Num.seqs=5 Similarity=0.907692 3   GACTTCAGACAATCTTTCA-CCTTTTCCTATCACACCTGTCTGAACTTGGATC

****** *** * **** * ******* *   ************* ** *
Consensus:

GACTTCaGACaaTcgTTCAgCcTTTTCCTaTcacACCTGTCTGAACTcGGaTc

>Bacillus_Fam_513_53_2 Nr. of seq. 2 Alignment length(with gaps) = 53
Alignment score = 0.716981
GCF_001420715.1_ASM142071v1_genomic.fna_24:660131-660443 Satlength=313 Nr of Repeats=6 RepeatLength=52 seed=GTCCTTCATC Num.seqs=6 Similarity=0.837607 0
GTCCTTCATCCCTCCGATGAACGACATCTGGAGCTTTTCCTTTTG-CNTTTTT
Rev.of_GCF_001636425.1_ASM163642v1_genomic.fna_1:3965800-3966164 Satlength=365 Nr of Repeats=7 RepeatLength=52 seed=GTCCTTCATC Num.seqs=7 Similarity=0.849817 25
GTCCTTCATCCCNCCGATGAAGGACATCTCANGC-TTTCCTCTCGCCCGTTTT

************ ******** *******   ** ****** * * * ****
Consensus:

GTCCTTCATCCCtCCGATGAAcGACATCTcaaGCtTTTCCTcTcGcCcgTTTT

>Bacillus_Fam_514_53_2 Nr. of seq. 2 Alignment length(with gaps) = 53
Alignment score = 0.663522
GCF_001587375.1_ASM158737v1_genomic.fna_14:64719-64927 Satlength=209 Nr of Repeats=4 RepeatLength=52 seed=GTTGTTTCAT Num.seqs=4 Similarity=0.935897
0 GTTGTT-TCATCTTCCCGCATCCCTCTGATTCGAGCACTTCGCCACTGCTCTC
Rev.of_GCF_001587375.1_ASM158737v1_genomic.fna_105:15709-16177 Satlength=469 Nr of Repeats=9 RepeatLength=52 seed=AGAGATGCGG Num.seqs=9 Similarity=0.864672 24 -TTCTTCTCATCTTCCCGCATCTCTCGGTTTCGAGCATTTCGCTGGGGATCCC

** ** *************** *** * ******** ***** * ** *
Consensus:

gTTcTTcTCATCTTCCCGCATCcCTCgGaTTCGAGCAcTTCGCcacgGaTCcC

>Bacillus_Fam_515_53_2 Nr. of seq. 2 Alignment length(with gaps) = 53
Alignment score = 0.613208
GCF_001587375.1_ASM158737v1_genomic.fna_44:21063-21635 Satlength=573 Nr of Repeats=11 RepeatLength=52 seed=CTCCTTATCT Num.seqs=11 Similarity=0.748650 0
CTCCTTATCTTCCCGCATCCGATGCTTGCGGGTAGTTGAGAGGGNGATCTCT-
GCF_001587375.1_ASM158737v1_genomic.fna_146:5307-5722 Satlength=416 Nr of Repeats=8 RepeatLength=52 seed=TTGCGGGAAC Num.seqs=7 Similarity=0.824498
25 CTCCTCATCTTCCCGTATCCTATCTTTGCGGGAACTTCATTCGG-GCTTTCTT

***** ********* **** ** ******* * ** * ** * * ***
Consensus:

CTCCTcATCTTCCCGcATCCgATccTTGCGGGaAcTTcAgacGGnGaTcTCTt

>Bacillus_Fam_516_53_2 Nr. of seq. 2 Alignment length(with gaps) = 53
Alignment score = 0.622642
GCF_001587375.1_ASM158737v1_genomic.fna_83:277-589 Satlength=313 Nr of Repeats=6 RepeatLength=52 seed=CGAATGGGAT Num.seqs=6 Similarity=0.810256
0 CGAATGGGATGGATGCGGGAACTTG-GAGCAAGAGACCCGGAGTTGAAGTGCC
GCF_001587375.1_ASM158737v1_genomic.fna_137:57747-58007 Satlength=261 Nr of Repeats=5 RepeatLength=52 seed=GAAGCGAAGT Num.seqs=5 Similarity=0.830769
39 CGAATCGGCTGGATACGGGAACTTGATAGTAGGAAAGGTGAAG-CGAAGTGCC

***** ** ***** ********** ** * ** * * ** ********
Consensus:

CGAATcGGaTGGATaCGGGAACTTGagAGcAaGAaAcccGaAGtcGAAGTGCC

>Bacillus_Fam_517_53_2 Nr. of seq. 2 Alignment length(with gaps) = 53
Alignment score = 0.647799
GCF_001591645.1_ASM159164v1_genomic.fna_3:242834-243042 Satlength=209 Nr of Repeats=4 RepeatLength=52 seed=ATTAGTGGAA Num.seqs=4 Similarity=0.776205
0 ATTAGTGGAATCAGTGAACTAAACGATCATTTAGAACATATG-AATTCCAGAA

GCF_001591645.1_ASM159164v1_genomic.fna_13:94849-95106 Satlength=258 Nr of Repeats=5 RepeatLength=52 seed=TTTAGGACAT Num.seqs=4 Similarity=0.700103
29   ACTAGTGGAATTGGTGAACTAAGTTNGCCTTTAGGACATGNGTATTTCCAGAA

* ********* *********   * ***** **** * * ********
Consensus:

AcTAGTGGAATcaGTGAACTAAacgagCaTTTAGaACATatGtAaTTCCAGAA

>Bacillus_Fam_518_53_2 Nr. of seq. 2 Alignment length(with gaps) = 53 Alignment score = 0.710692
GCF_001591805.1_ASM159180v1_genomic.fna_9:77461-77667 Satlength=207 Nr of Repeats=4 RepeatLength=52 seed=TGTCCTTCAT Num.seqs=3 Similarity=0.914530
0   TGTCCTTCATAAGGGAGATGAAGGACATTTATGTTGG-AAAACAAGAGAGATT
Rev.of_GCF_001420715.1_ASM142071v1_genomic.fna_24:84088-84296 Satlength=209 Nr of Repeats=4 RepeatLength=52 seed=CTTACCAAGA Num.seqs=4 Similarity=0.865828 41
TGTCCTTCATAATGCAGATGAAGGACATTTCTCTTGGTAAGTCAGTCGGGATT

************ * *************** * **** ** **   * ****
Consensus:

TGTCCTTCATAAgGcAGATGAAGGACATTTaTcTTGGtAAaaCAagaGaGATT

>Bacillus_Fam_519_53_2 Nr. of seq. 2 Alignment length(with gaps) = 53 Alignment score = 0.672956
GCF_001636315.1_ASM163631v1_genomic.fna_1:3498555-3498923 Satlength=369 Nr of Repeats=7 RepeatLength=52 seed=GTAACGTAGA Num.seqs=5 Similarity=0.800000 0
GTAACGTAGAGCTCGTCTACGTGCCCGACCGGA-GTATTTTTTCGCTGTAAGG
GCF_001636315.1_ASM163631v1_genomic.fna_1:3505767-3506235 Satlength=469 Nr of Repeats=8 RepeatLength=52 seed=GGTAACGTAG Num.seqs=7 Similarity=0.834681 51
GTAACGTAGAGCCCGTCTACGTGCCCGAAGAGATGGA-AATTTAGCAGTTAGG

************ ***************   ** * *   *** ** ** ***
Consensus:

GTAACGTAGAGCcCGTCTACGTGCCCGAacaGAtGgAtaaTTTaGCaGTaAGG

>Bacillus_Fam_520_53_2 Nr. of seq. 2 Alignment length(with gaps) = 53 Alignment score = 0.874214
GCF_001636345.1_ASM163634v1_genomic.fna_1:3361805-3362988 Satlength=1184 Nr of Repeats=15 RepeatLength=52 seed=TTCCTGAAAT Num.seqs=13 Similarity=0.838952 0
TTCCTGAAATCTGTGGCTTCC-CTNCTTTTCGGGAATGACTCGCCTTGGGGGA
GCF_001636345.1_ASM163634v1_genomic.fna_1:3809215-3809682 Satlength=468 Nr of Repeats=9 RepeatLength=52 seed=GGAATGACTC Num.seqs=8 Similarity=0.843407 31
TTCCTGAAATCTGTGGCTTCCGCT-CTTTTCGGGAATGACTCGCCTTGGGTCA

********************* ** ************************* *
Consensus:

TTCCTGAAATCTGTGGCTTCCgCTnCTTTTCGGGAATGACTCGCCTTGGGgcA

>Bacillus_Fam_521_53_2 Nr. of seq. 2 Alignment length(with gaps) = 53
Alignment score = 0.773585
GCF_001648575.1_ASM164857v1_genomic.fna_58:95837-96675 Satlength=839 Nr of Repeats=16 RepeatLength=52 seed=CTGTATGACA Num.seqs=13
Similarity=0.879684 0
CTGTATGACACGCATAATCTTGT-TCCATTAGGATTTTCGCGTTTCATTCGTG
GCF_001648575.1_ASM164857v1_genomic.fna_67:84110-84424 Satlength=315 Nr of Repeats=6 RepeatLength=52 seed=TCATTCGTGC Num.seqs=4
Similarity=0.841880
43   CTGTATGACACGCAAAATCATGTCTCC-TTTGAATGTTCGCGTCTCATTCGTG

************** **** *** *** ** * ** ******* *********
Consensus:

CTGTATGACACGCAaAATCaTGTcTCCaTTaGaATgTTCGCGTcTCATTCGTG

>Bacillus_Fam_522_53_2 Nr. of seq. 2 Alignment length(with gaps) = 53
Alignment score = 0.672956
GCF_002019605.1_ASM201960v1_genomic.fna_1:4201819-4202236 Satlength=418 Nr of Repeats=8 RepeatLength=52 seed=GGTAACTATG Num.seqs=7
Similarity=0.836777 0
GGTAACTATG-AGGCGTTCATAGTGACCAAAGTGAAGTAGAAAAAGCAAGAAC
GCF_002019605.1_ASM201960v1_genomic.fna_1:4298572-4298988 Satlength=417 Nr of Repeats=8 RepeatLength=52 seed=GTGACCGAAC Num.seqs=8
Similarity=0.847985 21
GGTAACTATGAAAGCG-TCATAGTGACCGAACCCAAAGAGAAAAAGCCAAAGC

********** * *** *********** **   ** ********* * * *
Consensus:

GGTAACTATGaAaGCGtTCATAGTGACCaAAcccAAagAGAAAAAGCaAaAaC

>Bacillus_Fam_523_53_2 Nr. of seq. 2 Alignment length(with gaps) = 53
Alignment score = 0.751572
GCF_900107275.1_IMG-taxon_2675903024_annotated_assembly_genomic.fna_42:562044-562408
Satlength=365 Nr of Repeats=7 RepeatLength=52 seed=TGTCCTCAAG Num.seqs=7
Similarity=0.661654 0
TGTCCTCAAGGAGTGTTTATGAGGACAAACTGGGGTTGGAAGCGTAGTCGAA-
GCF_900107275.1_IMG-taxon_2675903024_annotated_assembly_genomic.fna_42:623373-623581
Satlength=209 Nr of Repeats=4 RepeatLength=52 seed=GGACAAACTC Num.seqs=4
Similarity=0.830189 22
TGTCCTCAAGAAGGGCTTATGAGGACAAACTCGAGTCCGAAGCGTCGGCGAAN

********** ** * *************** * ** ******* * ****
Consensus:

TGTCCTCAAGaAGgGcTTATGAGGACAAACTcGaGTccGAAGCGTaGgCGAAn

>Bacillus_Fam_524_53_2 Nr. of seq. 2 Alignment length(with gaps) = 53
Alignment score = 0.855346
GCF_900156865.1_PRJEB18960_genomic.fna_3:4352895-4353155 Satlength=261 Nr of Repeats=5 RepeatLength=52 seed=AGGTCATCAT Num.seqs=5
Similarity=0.727273 0
AGGTCATCATGGAGGTGATGAAGACTC-GAAGCNGTGTTGNTTGGTAGNGAAG
GCF_900156865.1_PRJEB18960_genomic.fna_3:4347937-4349809 Satlength=1873 Nr of Repeats=36 RepeatLength=52 seed=GAAGCGGTGT Num.seqs=36
Similarity=0.899571 28
AGGTCATCATGGAGGTGATGAAGACTCTGAAGCGGTGTTGCTTGGATGGGAAG

*************************** ***** ****** **** * ****
Consensus:

AGGTCATCATGGAGGTGATGAAGACTCtGAAGCgGTGTTGcTTGGaaGgGAAG

>Bacillus_Fam_525_53_2 Nr. of seq. 2 Alignment length(with gaps) = 53
Alignment score = 1.000000
GCF_000153365.1_ASM15336v1_genomic.fna_14:139111-139848 Satlength=738 Nr of Repeats=14 RepeatLength=53 seed=CTCTCTTGAG Num.seqs=9
Similarity=0.849057 0
CTCTCTTGAGTGACTCATATCGGTTCTCTGAGGCACTCTGGGGCGGGGGGTCT
GCF_000473245.1_ASM47324v1_genomic.fna_1:4678509-4679246 Satlength=738 Nr of Repeats=14 RepeatLength=53 seed=CTCTCTTGAG Num.seqs=9
Similarity=0.849057 0
CTCTCTTGAGTGACTCATATCGGTTCTCTGAGGCACTCTGGGGCGGGGGGTCT

****
Consensus:

CTCTCTTGAGTGACTCATATCGGTTCTCTGAGGCACTCTGGGGCGGGGGGTCT

>Bacillus_Fam_526_53_2 Nr. of seq. 2 Alignment length(with gaps) = 53
Alignment score = 0.974843
GCF_000153365.1_ASM15336v1_genomic.fna_16:253052-253476 Satlength=425 Nr of Repeats=8 RepeatLength=53 seed=GTACAGAGTG Num.seqs=8
Similarity=0.816799
0   GTACAGAGTGCCTCAAACGAAGTCTATGAAGCACTCTAAAGATGGAAAGGAGC
Rev.of_GCF_000473245.1_ASM47324v1_genomic.fna_1:2556644-2557068
Satlength=425 Nr of Repeats=8 RepeatLength=53 seed=CACTCTGTAC Num.seqs=8
Similarity=0.809091 10
GTACAGAGTGCCTCAAACGAAGTCTATGAAGCACTCTAGAGATGGAAAGGAGC

************************************** **************
Consensus:

GTACAGAGTGCCTCAAACGAAGTCTATGAAGCACTCTAaAGATGGAAAGGAGC

>Bacillus_Fam_527_53_2 Nr. of seq. 2 Alignment length(with gaps) = 53
Alignment score = 0.943396
GCF_000153365.1_ASM15336v1_genomic.fna_67:19007-19908 Satlength=902 Nr of Repeats=17 RepeatLength=53 seed=CATGTATCCA Num.seqs=17
Similarity=0.747950
0   CATGTATCCAGTAGCTTTTTCGGGATACATGACACCNCNTTTTTTTNCTCGGT

GCF_000473245.1_ASM47324v1_genomic.fna_1:3847460-3848361 Satlength=902 Nr of Repeats=17 RepeatLength=53 seed=CATGTATCCA Num.seqs=17 Similarity=0.747950 0
CATGTATCCAGTAGCTTTTTCGGGATACATGACACCNCNTTTTTTTNCTCGGT

****
Consensus:

CATGTATCCAGTAGCTTTTTCGGGATACATGACACCNCNTTTTTTTNCTCGGT

>Bacillus_Fam_528_53_2 Nr. of seq. 2 Alignment length(with gaps) = 53
Alignment score = 0.654088
GCF_000181495.1_ASM18149v1_genomic.fna_179:56764-57452 Satlength=689 Nr of Repeats=13 RepeatLength=53 seed=AATGATGCCC Num.seqs=12 Similarity=0.841433 0
AATGATGCCCCGCGCGTCCGAAGCGCATGTCATCATTGTGAGTTTTAGGAGGA
GCF_000181495.1_ASM18149v1_genomic.fna_186:61685-62480 Satlength=796 Nr of Repeats=15 RepeatLength=53 seed=TGAAATGACA Num.seqs=15 Similarity=0.850973 50
AATGACACCCCGCGCGGGCGGAGCGCATGNCCTCATTTTGAACATTGAGATGA

***** ********* ** ******** * ***** ***   ** ** **
Consensus:

AATGAcaCCCCGCGCGgcCGaAGCGCATGtCaTCATTgTGAacaTTaaGAgGA

>Bacillus_Fam_529_53_2 Nr. of seq. 2 Alignment length(with gaps) = 53
Alignment score = 0.622642
GCF_000292245.2_ASM29224v2_genomic.fna_4:69040-69412 Satlength=373 Nr of Repeats=7 RepeatLength=53 seed=CTTATGACTT Num.seqs=6 Similarity=0.887631
0 CTTATGACTTTCTCACCTAAAGTCTGATGCTCGTTTCCCGCTCCCGCTCTTTC
Rev.of_GCF_000292245.2_ASM29224v2_genomic.fna_130:6522-6734 Satlength=213 Nr of Repeats=4 RepeatLength=53 seed=GAAAGTCATA Num.seqs=4 Similarity=0.932914 12
CTTATGACTTTCCGGCTTAATGTCGGAATCTAGTTGGCTTTTCCCGCTCTTTG

************   * *** *** ** ** *** *   ***********
Consensus:

CTTATGACTTTCccaCcTAAaGTCgGAagCTaGTTgcCcgcTCCCGCTCTTTc

>Bacillus_Fam_530_53_2 Nr. of seq. 2 Alignment length(with gaps) = 53
Alignment score = 0.930818
GCF_000708755.2_ASM70875v2_genomic.fna_1:293885-294308 Satlength=424 Nr of Repeats=8 RepeatLength=53 seed=AAATAGCATA Num.seqs=7 Similarity=0.890985
0 AAATAGCATAAGATAAGCACGGAATCGTACGCTATAATCTNAAGCCAGTGTAC
GCF_000709935.2_ASM70993v2_genomic.fna_1:311880-312092 Satlength=213 Nr of Repeats=4 RepeatLength=53 seed=AAATAGCATA Num.seqs=4 Similarity=0.819706
0 AAATAGCATAAGATAAGCAAGGAATCGTACGCTATAATCTCCAGCCAGTGTAC

******************* ******************** ***********
Consensus:

AAATAGCATAAGATAAGCAaGGAATCGTACGCTATAATCTcaAGCCAGTGTAC

>Bacillus_Fam_531_53_2 Nr. of seq. 2 Alignment length(with gaps) = 53
Alignment score = 0.811321
GCF_000708755.2_ASM70875v2_genomic.fna_2:36778-37043 Satlength=266 Nr of Repeats=5 RepeatLength=53 seed=TGGTTCTAAA Num.seqs=5 Similarity=0.861635
0   TGGTTCTAAAGCGCGTTAATAGTGCCATCAAAGGGTTGGGGTTGGNAGNTTGA
GCF_000709935.2_ASM70993v2_genomic.fna_24:75878-76198 Satlength=321 Nr of Repeats=6 RepeatLength=53 seed=TTGATGGTTC Num.seqs=4 Similarity=0.802935
49   TGGTTCTAAACCGAGTTAATAGTGCCATCAAAGGAGGGGGATTGGGAGCTTGA

********** ** *******************   *** **** ** ****
Consensus:

TGGTTCTAAAcCGaGTTAATAGTGCCATCAAAGGaggGGGaTTGGgAGcTTGA

>Bacillus_Fam_532_53_2 Nr. of seq. 2 Alignment length(with gaps) = 53
Alignment score = 0.880503
GCF_000708755.2_ASM70875v2_genomic.fna_2:902427-902638 Satlength=212 Nr of Repeats=4 RepeatLength=53 seed=TGTTGCCACT Num.seqs=3
Similarity=0.849057
0   TGTTGCCACTTTACTTCACGCATGGAGCCTGTTTGTGGCGCAACTCACCGGAT
GCF_000709935.2_ASM70993v2_genomic.fna_4:157618-157829 Satlength=212 Nr of Repeats=4 RepeatLength=53 seed=TGTTTGTGGC Num.seqs=3
Similarity=0.773585
29   TGNTGCCACTTTTCTTCACCCATGAAGCCTGTTTGTGGCGCAACTCACCGGAG

** ********* ****** **** ***************************
Consensus:

TGtTGCCACTTTaCTTCACcCATGaAGCCTGTTTGTGGCGCAACTCACCGGAg

>Bacillus_Fam_533_53_2 Nr. of seq. 2 Alignment length(with gaps) = 53
Alignment score = 0.628931
GCF_001439915.1_ASM143991v1_genomic.fna_122:165197-165557 Satlength=361 Nr of Repeats=6 RepeatLength=53 seed=GCGGTACATG Num.seqs=5
Similarity=0.771069 0
GCGGTACATGCTTGGNAGAAATCCGCTTGAGCGTGTACCGCTAGACCACTCTT
GCF_001439915.1_ASM143991v1_genomic.fna_126:34957-35437 Satlength=481 Nr of Repeats=9 RepeatLength=53 seed=AAGCGTGTAC Num.seqs=8
Similarity=0.901168
28   GCGGTACACGCTCGCAAAAAATTCGCCAAAGCGTGTACCGTAAGACGGTTCTA

******** *** * * **** ***   *********** ****   ***
Consensus:

GCGGTACAcGCTcGcaAaAAATcCGCcaaAGCGTGTACCGcaAGACcacTCTa

>Bacillus_Fam_534_52_2 Nr. of seq. 2 Alignment length(with gaps) = 52
Alignment score = 0.826923
GCF_000169195.2_ASM16919v2_genomic.fna_1:2621330-2621590 Satlength=261 Nr of Repeats=5 RepeatLength=52 seed=CAAAAGGACA Num.seqs=5

Similarity=0.615789 0
CAAAAGGACAATTCAGGNAGAAACAAAGNGCAAATTGTCCTTTANAG-GGTT
Rev.of_GCF_000217835.1_ASM21783v1_genomic.fna_1:2113071-2113331
Satlength=261 Nr of Repeats=5 RepeatLength=52 seed=TGTCCTTTTG Num.seqs=5
Similarity=0.648718 10
CAAAAGGACAATTCGGGNAGAGACAAAGAGCAAACTGTCCTTTAGAGNGGTT

************** ****** ****** ***** ********* ** ****
Consensus:

CAAAAGGACAATTCaGGNAGAaACAAAGaGCAAAcTGTCCTTTAgAGnGGTT

>Bacillus_Fam_535_52_2 Nr. of seq. 2 Alignment length(with gaps) = 52
Alignment score = 0.743590
GCF_000292245.2_ASM29224v2_genomic.fna_25:14121-14376 Satlength=256 Nr of Repeats=5 RepeatLength=51 seed=TTTTCATTAC Num.seqs=5 Similarity=0.746296
0 TTTTCATTACCTATTAT-CTGCTATTCGCACCCTAATCTTGGCTTTGCTGGC
GCF_000292245.2_ASM29224v2_genomic.fna_55:71-275 Satlength=205 Nr of Repeats=4 RepeatLength=51 seed=GATTTTCATT Num.seqs=4 Similarity=0.860566
49 TTTTCATTACCTATTATGAAGGT-TACGCACCCTAACCTTGGCTTTGCTAGA

***************** * * * ********** ************ *
Consensus:

TTTTCATTACCTATTATgaaGcTaTaCGCACCCTAAcCTTGGCTTTGCTaGa

>Bacillus_Fam_536_52_2 Nr. of seq. 2 Alignment length(with gaps) = 52
Alignment score = 0.602564
GCF_000292245.2_ASM29224v2_genomic.fna_35:14658-15015 Satlength=358 Nr of Repeats=7 RepeatLength=51 seed=GTAATGAAAT Num.seqs=7 Similarity=0.845627
0 GTAATGAAATTAGNCGACTCACTTGA-TTTCAGTGCGTATTACTCTTTATTG
GCF_000292245.2_ASM29224v2_genomic.fna_111:7118-7883 Satlength=766 Nr of Repeats=15 RepeatLength=51 seed=GATTGGTAAT Num.seqs=15
Similarity=0.816247
46 GTAATGAAATT-CTCGCTTTTCCTGATTTTCATTACCTATTACTCCNGATTG

*********** ** * * *** ***** * * ******** ****
Consensus:

GTAATGAAATTactCGacTcaCcTGAtTTTCAgTaCcTATTACTCctgATTG

>Bacillus_Fam_537_52_2 Nr. of seq. 2 Alignment length(with gaps) = 52
Alignment score = 0.641026
GCF_000331575.1_ASM33157v1_genomic.fna_1:73323-73782 Satlength=460 Nr of Repeats=9 RepeatLength=51 seed=TCAGCAAATC Num.seqs=9 Similarity=0.947712
0 TCAGCAAA-TCGCTGAAAAATCCTTTTTGCTGATAATAAAACCGATTTACCA
GCF_000331575.1_ASM33157v1_genomic.fna_1:1667305-1667917 Satlength=613 Nr of Repeats=12 RepeatLength=51 seed=TTTACTATCA Num.seqs=12
Similarity=0.857794 44
TCACCAAATTCGCT-CTGCGTCCGTTTTGCTGATGATAAATCGGATTTACTA

*** **** ***** *** ********** ***** * ******* *
Consensus:

TCAcCAAAtTCGCTgaaaaaTCCgTTTTGCTGATaATAAAaCcGATTTACcA

>Bacillus_Fam_538_52_2 Nr. of seq. 2 Alignment length(with gaps) = 52
Alignment score = 0.692308
GCF_001636335.1_ASM163633v1_genomic.fna_1:405681-405987 Satlength=307 Nr of Repeats=6 RepeatLength=51 seed=TTAAGAAGGG Num.seqs=6 Similarity=0.893682
0 TTAAGAAGGGTTCAAAACGCCCG-TATTTGTAAAAGAAGGAAAAATTGGTAG
GCF_001636335.1_ASM163633v1_genomic.fna_2:334616-334973 Satlength=358 Nr of Repeats=7 RepeatLength=51 seed=CGCCCGAAAT Num.seqs=5 Similarity=0.788462
17 CCAAGAAGCGTTCATGACGCCCGAAATTTCAAAAAGAA-GAAAAATCGGTAG

****** ***** ******* **** ******* ******* *****
Consensus:

ccAAGAAGcGTTCAaaACGCCCGaaATTTcaAAAAGAAgGAAAAATcGGTAG

>Bacillus_Fam_539_52_2 Nr. of seq. 2 Alignment length(with gaps) = 52
Alignment score = 0.698718
GCF_900104555.1_IMG-taxon_2651870172_annotated_assembly_genomic.fna_15:150210-151014 Satlength=805 Nr of Repeats=6 RepeatLength=51 seed=ACTTTTTTCT Num.seqs=5 Similarity=0.850980 0
ACTTTTTTCTGGTGATCTACTCGATTTTGTCTTTCAT-AGCNCGGATGAAAG
GCF_900104555.1_IMG-taxon_2651870172_annotated_assembly_genomic.fna_19:93292-93547 Satlength=256 Nr of Repeats=5 RepeatLength=51 seed=ATGAAAGACT Num.seqs=5 Similarity=0.830065 44
ACTTTTTCCTATTGAACTATTCNATTTTGTCCTTCATCAGC-CTCATGAAAG

******* ** *** *** ** ******** ***** *** * *******
Consensus:

ACTTTTTcCTagTGAaCTAcTCgATTTTGTCcTTCATcAGCnCgcATGAAAG

>Bacillus_Fam_540_52_2 Nr. of seq. 2 Alignment length(with gaps) = 52
Alignment score = 1.000000
GCF_000153365.1_ASM15336v1_genomic.fna_9:45356-45772 Satlength=417 Nr of Repeats=8 RepeatLength=52 seed=AGAGGGTGTT Num.seqs=8 Similarity=0.840659
0 AGAGGGTGTTAGTGGTAGCGACTAAAGCCGTCTAAGAGAGATTTTGCTAAAC
GCF_000473245.1_ASM47324v1_genomic.fna_1:212820-213236 Satlength=417 Nr of Repeats=8 RepeatLength=52 seed=AGAGGGTGTT Num.seqs=8 Similarity=0.840659
0 AGAGGGTGTTAGTGGTAGCGACTAAAGCCGTCTAAGAGAGATTTTGCTAAAC

****
Consensus:

AGAGGGTGTTAGTGGTAGCGACTAAAGCCGTCTAAGAGAGATTTTGCTAAAC

>Bacillus_Fam_541_52_2 Nr. of seq. 2 Alignment length(with gaps) = 52
Alignment score = 1.000000

GCF_000153365.1_ASM15336v1_genomic.fna_16:445040-445454 Satlength=415 Nr of Repeats=8 RepeatLength=52 seed=AGCCACTATT Num.seqs=6 Similarity=0.823868
0   AGCCACTATTTTCATTTTTCTTCAGGATAGTGGTCCCAGCTCCTGTGGCTGG
Rev.of_GCF_000473245.1_ASM47324v1_genomic.fna_1:2366668-2367085 Satlength=418 Nr of Repeats=8 RepeatLength=52 seed=AAAATAGTGG Num.seqs=5 Similarity=0.795152   12
AGCCACTATTTTCATTTTTCTTCAGGATAGTGGTCCCAGCTCCTGTGGCTGG

****
Consensus:

AGCCACTATTTTCATTTTTCTTCAGGATAGTGGTCCCAGCTCCTGTGGCTGG

>Bacillus_Fam_542_52_2 Nr. of seq. 2 Alignment length(with gaps) = 52 Alignment score = 1.000000
GCF_000153365.1_ASM15336v1_genomic.fna_60:10538-10746 Satlength=209 Nr of Repeats=4 RepeatLength=52 seed=GAACCGCTAT Num.seqs=4 Similarity=0.811966
0   GAACCGCTATTTTAGCTTTACACAGAAATAGTGGTCCCAGCGAGCGTGGCTA
GCF_000473245.1_ASM47324v1_genomic.fna_1:2054544-2054752 Satlength=209 Nr of Repeats=4 RepeatLength=52 seed=GAACCGCTAT Num.seqs=4 Similarity=0.811966   0
GAACCGCTATTTTAGCTTTACACAGAAATAGTGGTCCCAGCGAGCGTGGCTA

****
Consensus:

GAACCGCTATTTTAGCTTTACACAGAAATAGTGGTCCCAGCGAGCGTGGCTA

>Bacillus_Fam_543_52_2 Nr. of seq. 2 Alignment length(with gaps) = 52 Alignment score = 1.000000
GCF_000153365.1_ASM15336v1_genomic.fna_74:14504-15024 Satlength=521 Nr of Repeats=10 RepeatLength=52 seed=GTCACTCTAA Num.seqs=10 Similarity=0.908832
0   GTCACTCTAACCAAAGAACATAGAATCAGAGTGCCTCAAAAAGAGGATATGA
GCF_000473245.1_ASM47324v1_genomic.fna_1:2071154-2071674 Satlength=521 Nr of Repeats=10 RepeatLength=52 seed=GTCACTCTAA Num.seqs=10 Similarity=0.908832   0
GTCACTCTAACCAAAGAACATAGAATCAGAGTGCCTCAAAAAGAGGATATGA

****
Consensus:

GTCACTCTAACCAAAGAACATAGAATCAGAGTGCCTCAAAAAGAGGATATGA

>Bacillus_Fam_544_52_2 Nr. of seq. 2 Alignment length(with gaps) = 52 Alignment score = 1.000000
GCF_000153365.1_ASM15336v1_genomic.fna_74:34309-34724 Satlength=416 Nr of Repeats=7 RepeatLength=52 seed=AAATCAGAGG Num.seqs=6 Similarity=0.894017
0   AAATCAGAGGGCCTCAAACGAGCGAAATGGAACCCTCTAAACTGAAATAATC
GCF_000473245.1_ASM47324v1_genomic.fna_1:2090960-2091375 Satlength=416 Nr of Repeats=7 RepeatLength=52 seed=AAATCAGAGG Num.seqs=6 Similarity=0.894017   0
AAATCAGAGGGCCTCAAACGAGCGAAATGGAACCCTCTAAACTGAAATAATC

****
Consensus:

AAATCAGAGGGCCTCAAACGAGCGAAATGGAACCCTCTAAACTGAAATAATC

>Bacillus_Fam_545_52_2 Nr. of seq. 2 Alignment length(with gaps) = 52
Alignment score = 0.820513
GCF_000177235.2_ASM17723v2_genomic.fna_1:494305-494982 Satlength=678 Nr of Repeats=13 RepeatLength=52 seed=ACTCTATTTC Num.seqs=12
Similarity=0.894716 0
ACTCTATTTCGGACAGAGTTTCTTGTTTAATATTCAACTCTGTCCGATTGCA
GCF_000177235.2_ASM17723v2_genomic.fna_1:4603616-4604344 Satlength=729 Nr of Repeats=14 RepeatLength=52 seed=ATTGCTTCTC Num.seqs=14
Similarity=0.915187 46
TCTCTTTTTCGGACAGAGTTCCTTGTTCATTATTCAACTCTGTCTGATTGCT

**** ************** ****** * ************** ******
Consensus:

aCTCTaTTTCGGACAGAGTTcCTTGTTcAaTATTCAACTCTGTCcGATTGCa

>Bacillus_Fam_546_52_2 Nr. of seq. 2 Alignment length(with gaps) = 52
Alignment score = 0.801282
GCF_000177235.2_ASM17723v2_genomic.fna_1:2323006-2323553 Satlength=548 Nr of Repeats=10 RepeatLength=52 seed=AAGGTCATCA Num.seqs=9
Similarity=0.859687 0
AAGGTCATCATACGAAGTCTATGCTACCCGAAAAATNGAAACCGAGCAGATG
GCF_000177235.2_ASM17723v2_genomic.fna_1:2835835-2836198 Satlength=364 Nr of Repeats=5 RepeatLength=52 seed=AAGGGCAGCA Num.seqs=3
Similarity=0.880342 0
AAGGGCAGCATACGAAGCCTATGCCTCCCGAAAAAATGAAATCGAGCAGATG

**** ** ********* ****** ********* **** **********
Consensus:

AAGGgCAgCATACGAAGcCTATGCcaCCCGAAAAAatGAAAcCGAGCAGATG

>Bacillus_Fam_547_52_2 Nr. of seq. 2 Alignment length(with gaps) = 52
Alignment score = 0.878205
GCF_000177235.2_ASM17723v2_genomic.fna_1:4408835-4409251 Satlength=417 Nr of Repeats=8 RepeatLength=52 seed=CGCTATGATT Num.seqs=8
Similarity=0.869048 0
CGCTATGATTACCTTCAACTCATTTTGGGCTCGTTCTCGGGAAGCATACAAT
GCF_000177235.2_ASM17723v2_genomic.fna_1:4410749-4411009 Satlength=261 Nr of Repeats=5 RepeatLength=52 seed=CTATGATTAC Num.seqs=5
Similarity=0.817949 2
CGCTATGATTACCGTCAACTCATTTTGGGCTCNCTCTCGGGAAGCATACACG

************* ***************** ****************
Consensus:

CGCTATGATTACCgTCAACTCATTTTGGGCTCgcTCTCGGGAAGCATACAag

>Bacillus_Fam_548_52_2 Nr. of seq. 2 Alignment length(with gaps) = 52
Alignment score = 0.955128
GCF_000186145.1_Bacillus_sp_2_A_57_CT2_V1_genomic.fna_4:260068-260380
Satlength=313 Nr of Repeats=6 RepeatLength=52 seed=GTTCAGACAG Num.seqs=6
Similarity=0.890356   0
GTTCAGACAGGAAGAACGAGAAATAGCCTGTTTGTGTCCGAAGAAGGGGCAG
Rev.of_GCF_000294775.2_ASM29477v2_genomic.fna_1:3372157-3372521
Satlength=365 Nr of Repeats=7 RepeatLength=52 seed=TCTTCGGACA Num.seqs=7
Similarity=0.864469   44
GTTCAGACAGGAAGAACGAGAAATAGNCTGTTTGTGTCCGAAGACGGGGCAG

************************** ***************** *******
Consensus:

GTTCAGACAGGAAGAACGAGAAATAGcCTGTTTGTGTCCGAAGAaGGGGCAG

>Bacillus_Fam_549_52_2 Nr. of seq. 2 Alignment length(with gaps) = 52
Alignment score = 0.910256
GCF_000186145.1_Bacillus_sp_2_A_57_CT2_V1_genomic.fna_5:20059-20318
Satlength=260 Nr of Repeats=5 RepeatLength=52 seed=TGGGAGTTAA Num.seqs=4
Similarity=0.816239   0
TGGGAGTTAACCAAAGGGAATAAAGCCGAAATCCCAGAGCCAGCCAACAAAA
Rev.of_GCF_000294775.2_ASM29477v2_genomic.fna_1:2910855-2911062
Satlength=208 Nr of Repeats=4 RepeatLength=52 seed=CTCCCATTTT Num.seqs=3
Similarity=0.811966   6
TGGGAGTTAATCAAANGGAATAAAGNCGAAATCCCAGAGCAAGCCAACAAAA

********** **** ********* ************** ***********
Consensus:

TGGGAGTTAAcCAAAgGGAATAAAGcCGAAATCCCAGAGCaAGCCAACAAAA

>Bacillus_Fam_550_52_2 Nr. of seq. 2 Alignment length(with gaps) = 52
Alignment score = 0.974359
GCF_000186145.1_Bacillus_sp_2_A_57_CT2_V1_genomic.fna_5:39414-39827
Satlength=414 Nr of Repeats=8 RepeatLength=52 seed=GTCGCCATAA Num.seqs=5
Similarity=0.858974   0
GTCGCCATAAGAGGTTCATGACGACGAAAAATTGGAGGTCCAAGTAAAAACA
Rev.of_GCF_000294775.2_ASM29477v2_genomic.fna_1:2898257-2898722
Satlength=466 Nr of Repeats=9 RepeatLength=52 seed=TTATGGCGAC Num.seqs=6
Similarity=0.825641   10
GTCGCCATAAGAGGTTCATGACGACGAAAAATTGGAGGTCCAAGTAAAAACG

****
Consensus:

GTCGCCATAAGAGGTTCATGACGACGAAAAATTGGAGGTCCAAGTAAAAACa

>Bacillus_Fam_551_52_2 Nr. of seq. 2 Alignment length(with gaps) = 52
Alignment score = 0.923077
GCF_000186145.1_Bacillus_sp_2_A_57_CT2_V1_genomic.fna_5:358104-358520
Satlength=417 Nr of Repeats=7 RepeatLength=52 seed=TTCGGACTCA Num.seqs=6
Similarity=0.822222   0
TTCGGACTCAACGACCATCCGTTCGCGGTTTTGAGTCCGAACACCAGTCAAC

Rev.of_GCF_000294775.2_ASM29477v2_genomic.fna_1:2554186-2554498
Satlength=313 Nr of Repeats=5 RepeatLength=52 seed=TGAGTCCGAA Num.seqs=4
Similarity=0.747379 10
TTCGGACTCAGCGAGCATCCGTTCGCGGTTTTGTGTCCGAACACCAGTCAAC

********** *** ***************** *****************
Consensus:

TTCGGACTCAaCGAcCATCCGTTCGCGGTTTTGaGTCCGAACACCAGTCAAC

>Bacillus_Fam_552_52_2 Nr. of seq. 2 Alignment length(with gaps) = 52
Alignment score = 0.974359
GCF_000186145.1_Bacillus_sp_2_A_57_CT2_V1_genomic.fna_6:198141-198401
Satlength=261 Nr of Repeats=5 RepeatLength=52 seed=CATGAAATAT Num.seqs=5
Similarity=0.816352 0
CATGAAATATGGGAGTTAATTCTAATCATTATCGACCAAACAAGAGATGCCA
Rev.of_GCF_000294775.2_ASM29477v2_genomic.fna_1:899649-899960
Satlength=312 Nr of Repeats=6 RepeatLength=52 seed=TAACTCCCAT Num.seqs=5
Similarity=0.871795 18
CATGAAATATGGGAGTTAATCCTAATCATTATCGACCAAACAAGAGATGCCA

******************* ******************************
Consensus:

CATGAAATATGGGAGTTAATcCTAATCATTATCGACCAAACAAGAGATGCCA

>Bacillus_Fam_553_52_2 Nr. of seq. 2 Alignment length(with gaps) = 52
Alignment score = 0.948718
GCF_000186145.1_Bacillus_sp_2_A_57_CT2_V1_genomic.fna_7:64235-64495
Satlength=261 Nr of Repeats=5 RepeatLength=52 seed=CACCTTCGGA Num.seqs=5
Similarity=0.844025 0
CACCTTCGGACTCGCTCACGTTGATTTCTCCCCTTTCTGTCCGAACCTGCTC
Rev.of_GCF_000294775.2_ASM29477v2_genomic.fna_1:658243-658659
Satlength=417 Nr of Repeats=8 RepeatLength=52 seed=GAGTCCGAAG Num.seqs=8
Similarity=0.829739 13
CACCTTCGGACTCGCTCACGTTGATTTCTCCCTTTTCTGTCCGAACCTCCTC

******************************** ************** ***
Consensus:

CACCTTCGGACTCGCTCACGTTGATTTCTCCCcTTTCTGTCCGAACCTcCTC

>Bacillus_Fam_554_52_2 Nr. of seq. 2 Alignment length(with gaps) = 52
Alignment score = 0.897436
GCF_000285535.1_ASM28553v1_genomic.fna_2:1010386-1010802 Satlength=417 Nr of Repeats=8 RepeatLength=52 seed=TCCTCTTCAT Num.seqs=8
Similarity=0.735175 0
TCCTCTTCATAAGCCCCGTGAAGAGGAAACGTAACCAAAATCTCATTCCTTT
Rev.of_GCF_000285535.1_ASM28553v1_genomic.fna_45:36-244 Satlength=209 Nr of Repeats=4 RepeatLength=52 seed=AAGAGGAAAA Num.seqs=4
Similarity=0.794872
7   TCCTCTTCATAAGCTTCGTGAAGGGGAAACGTAACCAAAATCTCATTCTTTT

************** ******* *********************** ***

Consensus:

TCCTCTTCATAAGCccCGTGAAGaGGAAACGTAACCAAAATCTCATTCcTTT

>Bacillus_Fam_555_52_2 Nr. of seq. 2 Alignment length(with gaps) = 52
Alignment score = 0.820513
GCF_000285535.1_ASM28553v1_genomic.fna_3:290400-290816 Satlength=417 Nr of Repeats=8 RepeatLength=52 seed=TGAAGAGGAT Num.seqs=8 Similarity=0.851648
0   TGAAGAGGATTACAAGCTTATGTTCGTCCCGCTTTCCTCTTCATCTAGGCTT
GCF_000285535.1_ASM28553v1_genomic.fna_41:8-216 Satlength=209 Nr of Repeats=4 RepeatLength=52 seed=TGAAGAGGAT Num.seqs=4 Similarity=0.846154
0   TGAAGAGGATTACAGGCTTCAATTCCTCCCACTTTCCTCTTCATCTAGGCTA

************** ****   *** **** ********************
Consensus:

TGAAGAGGATTACAaGCTTaaaTTCcTCCCaCTTTCCTCTTCATCTAGGCTa

>Bacillus_Fam_556_52_2 Nr. of seq. 2 Alignment length(with gaps) = 52
Alignment score = 0.692308
GCF_000307875.1_BABA1.0_genomic.fna_34:24055-24262 Satlength=208 Nr of Repeats=4 RepeatLength=52 seed=TGTCCTCAAT Num.seqs=3 Similarity=0.811966
0   TGTCCTCAATAAGGGCTATTGAGGACAAAACTCGCCTGGATAGCAGGAAAAA
GCF_001591665.1_ASM159166v1_genomic.fna_21:148942-149150 Satlength=209 Nr of Repeats=4 RepeatLength=52 seed=GCTCTTAAAG Num.seqs=4 Similarity=0.846154 14
TGTCTTTAATAAGGGCTCTTAAAGACAAAACTCCGCGGGAAAGCAATCAAAA

**** * ********** ** * ********** * *** ****   ****
Consensus:

TGTCcTcAATAAGGGCTaTTaAaGACAAAACTCccCgGGAaAGCAagaAAAA

>Bacillus_Fam_557_52_2 Nr. of seq. 2 Alignment length(with gaps) = 52
Alignment score = 0.628205
GCF_000311725.1_ASM31172v1_genomic.fna_4:30876-31291 Satlength=416 Nr of Repeats=8 RepeatLength=52 seed=ATTCGGACTC Num.seqs=7 Similarity=0.783550
0   ATTCGGACTCCCTTNCNCCTTTTCCTGCTCGACCTGTCCGAATTCCCCTTCT
GCF_000311725.1_ASM31172v1_genomic.fna_6:46056-46316 Satlength=261 Nr of Repeats=5 RepeatLength=52 seed=TCTATTCGGA Num.seqs=5 Similarity=0.887179
49   ATTCGGACTTACTTATTTGTTTTACAGCTTGGGCTGTCCGAATAGCCCCTCT

********* ***   **** * *** * ********** *** ***
Consensus:

ATTCGGACTcaCTTactccTTTTaCaGCTcGacCTGTCCGAATacCCCcTCT

>Bacillus_Fam_558_52_2 Nr. of seq. 2 Alignment length(with gaps) = 52
Alignment score = 0.641026
GCF_000374345.1_ASM37434v1_genomic.fna_5:87235-87443 Satlength=209 Nr of Repeats=4 RepeatLength=52 seed=GGCTTTCATC Num.seqs=4 Similarity=0.905983
0   GGCTTTCATCCCCTTTATGAGCGACCATACCGATGTGTTTTACCATGGCACG

GCF_000374345.1_ASM37434v1_genomic.fna_49:17746-17954 Satlength=209 Nr of Repeats=4 RepeatLength=52 seed=CCTATGTGTT Num.seqs=4 Similarity=0.876068
29   GGCGGTCATTCCCCTTATGAACGACCGTGCCTATGTGTTTTTCATTCTCGCG

*** **** *** ****** ***** * ** ********* * * * **
Consensus:

GGCggTCATcCCCcTTATGAaCGACCaTaCCgATGTGTTTTaCaaTcgCaCG

>Bacillus_Fam_559_52_2 Nr. of seq. 2 Alignment length(with gaps) = 52
Alignment score = 0.666667
GCF_000374345.1_ASM37434v1_genomic.fna_8:79176-79540 Satlength=365 Nr of Repeats=7 RepeatLength=52 seed=AATCAAACGG Num.seqs=7 Similarity=0.796047
0   AATCAAACGGTCGCTCATCCCCTTTATGAACGCCCGCGCCTGCTGAGTTTTT
GCF_000374345.1_ASM37434v1_genomic.fna_9:12991-13459 Satlength=469 Nr of Repeats=8 RepeatLength=52 seed=TCACACGGTC Num.seqs=7 Similarity=0.789988
2   AATCACACGGTCGATCATTTCCCTTATGAACGACCACGCCGGCTGAAAATCC

***** ******* **** ** ********* ** **** *****   *
Consensus:

AATCAaACGGTCGaTCATccCCcTTATGAACGaCCaCGCCgGCTGAaaaTcc

>Bacillus_Fam_560_52_2 Nr. of seq. 2 Alignment length(with gaps) = 52
Alignment score = 0.724359
GCF_000374565.1_ASM37456v1_genomic.fna_1:698811-699383 Satlength=573 Nr of Repeats=11 RepeatLength=52 seed=ATTAGTGACC Num.seqs=11 Similarity=0.762851   0
ATTAGTGACCGTTATCCGGGATTTTNCCTAAATACGGGTACCTTTTACTGCT
Rev.of_GCF_000374565.1_ASM37456v1_genomic.fna_3:262427-263155 Satlength=729 Nr of Repeats=14 RepeatLength=52 seed=GGTCACTAAT Num.seqs=14 Similarity=0.765004
10   ATTAGTGACCGAGCTACGGGATTTACCGTAAATACGGGAACCTTTTGCCGCG

***********   * ********* * ********** ******* * **
Consensus:

ATTAGTGACCGagaTaCGGGATTTacCcTAAATACGGGaACCTTTTaCcGCg

>Bacillus_Fam_561_52_2 Nr. of seq. 2 Alignment length(with gaps) = 52
Alignment score = 0.615385
GCF_000374565.1_ASM37456v1_genomic.fna_29:5643-6006 Satlength=364 Nr of Repeats=7 RepeatLength=52 seed=TTAGTTACCG Num.seqs=6 Similarity=0.733333
0   TTAGTTACCGTTTTTCGCACTTAAACCCGTATTCGGGCACCTTTCCCCGCGA
Rev.of_GCF_000374565.1_ASM37456v1_genomic.fna_32:3468-3780 Satlength=313 Nr of Repeats=6 RepeatLength=52 seed=CCCGAAAAAG Num.seqs=6 Similarity=0.814675   37
TTAGTGACCGGTTTTCCCTCTCGGACGCTTTTTCGGGCACTATTACTCTCGA

***** **** ***** * **   ** * * ********* ** * * ***
Consensus:

TTAGTgACCGgTTTTCcCaCTcaaACcCgTaTTCGGGCACcaTTaCcCgCGA

>Bacillus_Fam_562_52_2 Nr. of seq. 2 Alignment length(with gaps) = 52
Alignment score = 0.910256
GCF_000708755.2_ASM70875v2_genomic.fna_1:24282-24750 Satlength=469 Nr of Repeats=8 RepeatLength=52 seed=CAGAAAATCA Num.seqs=7 Similarity=0.840049
0   CAGAAAATCAGATTTGATGGCACTGAACATTGAGAATAGNACCACCAACGAA
GCF_000709935.2_ASM70993v2_genomic.fna_1:24497-24861 Satlength=365 Nr of Repeats=7 RepeatLength=52 seed=GATGGCACTG Num.seqs=7 Similarity=0.796646
15   CAGANAATCAGTTTTGATGGCACTGAACATCGAGAATAGGACCACCAACGAA

**** ****** ****************** ******** ************
Consensus:

CAGAaAATCAGaTTTGATGGCACTGAACATcGAGAATAGgACCACCAACGAA

>Bacillus_Fam_563_52_2 Nr. of seq. 2 Alignment length(with gaps) = 52
Alignment score = 1.000000
GCF_000708755.2_ASM70875v2_genomic.fna_1:149824-150187 Satlength=364 Nr of Repeats=7 RepeatLength=52 seed=TGAAAGTGTT Num.seqs=6 Similarity=0.926496
0   TGAAAGTGTTGCTAACCCAGTTTAAGAACACTTTTCCGCAGAGCAGAAGGGT
GCF_000709935.2_ASM70993v2_genomic.fna_1:150814-151022 Satlength=209 Nr of Repeats=4 RepeatLength=52 seed=TTGAAAGTGT Num.seqs=4 Similarity=0.893162
51   TGAAAGTGTTGCTAACCCAGTTTAAGAACACTTTTCCGCAGAGCAGAAGGGT

****
Consensus:

TGAAAGTGTTGCTAACCCAGTTTAAGAACACTTTTCCGCAGAGCAGAAGGGT

>Bacillus_Fam_564_52_2 Nr. of seq. 2 Alignment length(with gaps) = 52
Alignment score = 0.980769
GCF_000708755.2_ASM70875v2_genomic.fna_1:165249-165509 Satlength=261 Nr of Repeats=5 RepeatLength=52 seed=TGATGTGAAG Num.seqs=5 Similarity=0.771795
0   TGATGTGAAGGGTTATAAGTAAGTGAGAATTGACCCTTNAGTGCAGCCGTAG
GCF_000709935.2_ASM70993v2_genomic.fna_1:166061-166321 Satlength=261 Nr of Repeats=5 RepeatLength=52 seed=TGATGTGAAG Num.seqs=5 Similarity=0.771795
0   TGATGTGAAGGGTTATAAGTAAGTGAGAATTGACCCTTNAGTGCAGCCGTAG

****
Consensus:

TGATGTGAAGGGTTATAAGTAAGTGAGAATTGACCCTTNAGTGCAGCCGTAG

>Bacillus_Fam_565_52_2 Nr. of seq. 2 Alignment length(with gaps) = 52
Alignment score = 0.974359
GCF_000708755.2_ASM70875v2_genomic.fna_1:230141-230557 Satlength=417 Nr of Repeats=8 RepeatLength=52 seed=GTGAATGAAC Num.seqs=8 Similarity=0.861722
0   GTGAATGAACCCTTCGTACGAAGAATCAGGAGCTGAAGGGTCAAAAGGCGTC
GCF_000709935.2_ASM70993v2_genomic.fna_1:229818-230026 Satlength=209 Nr of Repeats=4 RepeatLength=52 seed=GTGAATGAAC Num.seqs=4

Similarity=0.858974
0   GTGAATGAACCCTTCATACGAAGAATCAGGAGCTGAAGGGTCAAAAGGCGTC

*************** ************************************
Consensus:

GTGAATGAACCCTTCaTACGAAGAATCAGGAGCTGAAGGGTCAAAAGGCGTC

>Bacillus_Fam_566_52_2 Nr. of seq. 2 Alignment length(with gaps) = 52
Alignment score = 1.000000
GCF_000708755.2_ASM70875v2_genomic.fna_2:776717-776977 Satlength=261 Nr of Repeats=5 RepeatLength=52 seed=CATGAAGGGT Num.seqs=5 Similarity=0.897436
0   CATGAAGGGTCATATCATGCTCGCTTATGACCACTCAGCTCACCCTGCCCTG
GCF_000709935.2_ASM70993v2_genomic.fna_4:32363-32675 Satlength=313 Nr of Repeats=6 RepeatLength=52 seed=GCATGAAGGG Num.seqs=6 Similarity=0.912821
51   CATGAAGGGTCATATCATGCTCGCTTATGACCACTCAGCTCACCCTGCCCTG

****
Consensus:

CATGAAGGGTCATATCATGCTCGCTTATGACCACTCAGCTCACCCTGCCCTG

>Bacillus_Fam_567_52_2 Nr. of seq. 2 Alignment length(with gaps) = 52
Alignment score = 0.826923
GCF_000708755.2_ASM70875v2_genomic.fna_5:240351-240559 Satlength=209 Nr of Repeats=4 RepeatLength=52 seed=CAGGTTTAAG Num.seqs=4 Similarity=0.901709
0   CAGGTTTAAGAGCACAAACGTGTCTACCCAGCTAAGAGTTTGTGCTGCAAAC
GCF_000709935.2_ASM70993v2_genomic.fna_13:174173-174383 Satlength=211 Nr of Repeats=4 RepeatLength=52 seed=AGTTTGTGCT Num.seqs=3 Similarity=0.786325
36   AAGGTTTAAGAGCACTAACGCGTCTTCCCAGANAAGAGTTTGTGCTGCTAAC

************** **** **** ***** *************** ***
Consensus:

aAGGTTTAAGAGCACaAACGcGTCTaCCCAGatAAGAGTTTGTGCTGCaAAC

>Bacillus_Fam_568_52_2 Nr. of seq. 2 Alignment length(with gaps) = 52
Alignment score = 0.948718
GCF_000708755.2_ASM70875v2_genomic.fna_6:356690-356898 Satlength=209 Nr of Repeats=4 RepeatLength=52 seed=TCTTACCCTC Num.seqs=4 Similarity=0.811966
0   TCTTACCCTCCCAAACCGTTATTCGATCTGACAGGGTAAGCAAACCTACGTT
GCF_000709935.2_ASM70993v2_genomic.fna_5:562744-562952 Satlength=209 Nr of Repeats=4 RepeatLength=52 seed=TCTTACCCTC Num.seqs=4 Similarity=0.803419
0   TCTTACCCTCCCAAACCGTTATTCGATCTGACAGGGCAAGCAAACCTACTTT

************************************ ************ **
Consensus:

TCTTACCCTCCCAAACCGTTATTCGATCTGACAGGGcAAGCAAACCTACgTT

>Bacillus_Fam_569_52_2 Nr. of seq. 2 Alignment length(with gaps) = 52
Alignment score = 0.897436
GCF_000708755.2_ASM70875v2_genomic.fna_7:38513-38773 Satlength=261 Nr of Repeats=5 RepeatLength=52 seed=AAGTGTTCTT Num.seqs=5 Similarity=0.820513
0   AAGTGTTCTTAACCTGGGTTAGCAACACAAACTTTGTTGATTGTTCACTGGA
GCF_000709935.2_ASM70993v2_genomic.fna_19:41278-41486 Satlength=209 Nr of Repeats=4 RepeatLength=52 seed=GGAAAGTGTT Num.seqs=4 Similarity=0.820513
49   AAGTGTTCTTAATCTGAGTTAGCAACACAAACTCTGTTGATCGTTCACTGGA

************ *** *************** ******* **********
Consensus:

AAGTGTTCTTAAcCTGaGTTAGCAACACAAACTcTGTTGATcGTTCACTGGA

>Bacillus_Fam_570_52_2 Nr. of seq. 2 Alignment length(with gaps) = 52
Alignment score = 0.794872
GCF_000708755.2_ASM70875v2_genomic.fna_7:121702-121910 Satlength=209 Nr of Repeats=4 RepeatLength=52 seed=TTGATGGTTC Num.seqs=4 Similarity=0.905983
0   TTGATGGTTCTTATAAGCCCGATAAGTACCACCAAACTCTGATTCCCTTGAA
Rev.of_GCF_000708755.2_ASM70875v2_genomic.fna_22:145264-145525 Satlength=262 Nr of Repeats=5 RepeatLength=52 seed=TAAGAACCAT Num.seqs=4 Similarity=0.747863   13
TTGATGGTTCTTATAAGCCCGATAAGTGCCATCAACCTCTGAGTTTTTTGGA

************************** *** *** ****** *   *** *
Consensus:

TTGATGGTTCTTATAAGCCCGATAAGTaCCAcCAAaCTCTGAgTcccTTGaA

>Bacillus_Fam_571_52_2 Nr. of seq. 2 Alignment length(with gaps) = 52
Alignment score = 0.948718
GCF_000708755.2_ASM70875v2_genomic.fna_22:151396-151604 Satlength=209 Nr of Repeats=4 RepeatLength=52 seed=GAAGGTCACA Num.seqs=4 Similarity=0.846154
0   GAAGGTCACAAAACAGGAAGGAATGTGCCCTTCGTGAAGTGTTCCAAGGTGT
GCF_000709935.2_ASM70993v2_genomic.fna_24:262239-262603 Satlength=365 Nr of Repeats=7 RepeatLength=52 seed=GAAGGTCACA Num.seqs=7 Similarity=0.851038
0   GAAGGTCACAAAACGGGAAGGAATGTGCCCTTCGTGAAGTGTTCCAAGATGT

************** ********************************* ***
Consensus:

GAAGGTCACAAAACaGGAAGGAATGTGCCCTTCGTGAAGTGTTCCAAGaTGT

>Bacillus_Fam_572_52_2 Nr. of seq. 2 Alignment length(with gaps) = 52
Alignment score = 1.000000
GCF_000708755.2_ASM70875v2_genomic.fna_22:158758-159018 Satlength=261 Nr of Repeats=5 RepeatLength=52 seed=GGAAAGTGTT Num.seqs=5 Similarity=0.779487
0   GGAAAGTGTTCTAAAAAGGGTTTAGCAACATTAACCCTGGCTGGAGAAGAGA

GCF_000709935.2_ASM70993v2_genomic.fna_24:269755-270118 Satlength=364 Nr of Repeats=7 RepeatLength=52 seed=GGAAAGTGTT Num.seqs=6 Similarity=0.832479
0   GGAAAGTGTTCTAAAAAGGGTTTAGCAACATTAACCCTGGCTGGAGAAGAGA

****
Consensus:

GGAAAGTGTTCTAAAAAGGGTTTAGCAACATTAACCCTGGCTGGAGAAGAGA

>Bacillus_Fam_573_52_2 Nr. of seq. 2 Alignment length(with gaps) = 52 Alignment score = 1.000000
GCF_000708755.2_ASM70875v2_genomic.fna_22:241123-241383 Satlength=261 Nr of Repeats=5 RepeatLength=52 seed=TGAAAGGTCA Num.seqs=5 Similarity=0.866667
0   TGAAAGGTCAATAAGCGGAAGGATAAGACCCTTTATCAAAGCTGGTCCGCCG
GCF_000709935.2_ASM70993v2_genomic.fna_24:352189-352449 Satlength=261 Nr of Repeats=5 RepeatLength=52 seed=TGAAAGGTCA Num.seqs=5 Similarity=0.866667
0   TGAAAGGTCAATAAGCGGAAGGATAAGACCCTTTATCAAAGCTGGTCCGCCG

****
Consensus:

TGAAAGGTCAATAAGCGGAAGGATAAGACCCTTTATCAAAGCTGGTCCGCCG

>Bacillus_Fam_574_52_2 Nr. of seq. 2 Alignment length(with gaps) = 52 Alignment score = 0.641026
GCF_000787375.1_ASM78737v1_genomic.fna_3:77617-77929 Satlength=313 Nr of Repeats=6 RepeatLength=52 seed=CTTTGACTCC Num.seqs=6 Similarity=0.735867
0   CTTTGACTCCCGTTCTCGGGGCTTCCTCCAACTTTCGGGTGCCATCCCCTTC
GCF_000787375.1_ASM78737v1_genomic.fna_4:23329-23745 Satlength=417 Nr of Repeats=6 RepeatLength=52 seed=TCTTTCTGCT Num.seqs=4 Similarity=0.863248
13   CTTTGACTACCGGTCTTTCTGCTTCCTCTCGTTTTCGGTAGTCAACCCCTTC

******** *** ***   ********   ****** * ** *******
Consensus:

CTTTGACTaCCGgTCTcgcgGCTTCCTCcaacTTTCGGgaGcCAaCCCCTTC

>Bacillus_Fam_575_52_2 Nr. of seq. 2 Alignment length(with gaps) = 52 Alignment score = 0.910256
GCF_000934845.1_ASM93484v1_genomic.fna_81:9-269 Satlength=261 Nr of Repeats=5 RepeatLength=52 seed=AATCTCCACA Num.seqs=5 Similarity=0.815385
0   AATCTCCACATACTGGAAAATCGTAGTCCCAATCAGCCCTATTGGTGCTCCN
Rev.of_GCF_000934845.1_ASM93484v1_genomic.fna_105:32-292 Satlength=261 Nr of Repeats=5 RepeatLength=52 seed=TATGTGGAGA Num.seqs=5 Similarity=0.762963 12
AATCTCCACATACTGGTAAATCNTAGTCCCAATAAGCCCTATTGGTGCTCCG

**************** ***** ********** ****************
Consensus:

AATCTCCACATACTGGaAAATCgTAGTCCCAATaAGCCCTATTGGTGCTCCg

>Bacillus_Fam_576_52_2 Nr. of seq. 2 Alignment length(with gaps) = 52
Alignment score = 0.974359
GCF_000986785.1_ASM98678v1_genomic.fna_3:238264-238524 Satlength=261 Nr of Repeats=5 RepeatLength=52 seed=GGGCCTCCAT Num.seqs=5 Similarity=0.876730
0   GGGCCTCCATCAGGGCGATGGACGACCGTTTATTGCTCAATCTCAGGTTTCA
Rev.of_GCF_000986785.1_ASM98678v1_genomic.fna_6:203684-203891 Satlength=208 Nr of Repeats=4 RepeatLength=52 seed=GATGGAGGCC Num.seqs=3 Similarity=0.880342   11
GGGCCTCCATCGGGGCGATGGACGACCGTTTATTGCTCAATCTCAGGTTTCA

*********** **
Consensus:

GGGCCTCCATCaGGGCGATGGACGACCGTTTATTGCTCAATCTCAGGTTTCA

>Bacillus_Fam_577_52_2 Nr. of seq. 2 Alignment length(with gaps) = 52
Alignment score = 0.730769
GCF_000986785.1_ASM98678v1_genomic.fna_9:47447-47966 Satlength=520 Nr of Repeats=10 RepeatLength=52 seed=TTTTTCTCTC Num.seqs=9 Similarity=0.773235
0   TTTTTCTCTCATTCNGCTGATTTCGGGTACTGAGGCCGCCTGTCAGTACCTC
Rev.of_GCF_000986785.1_ASM98678v1_genomic.fna_31:15669-16293 Satlength=625 Nr of Repeats=12 RepeatLength=52 seed=AGAAAAAGAG Num.seqs=12 Similarity=0.747942
7   TTTTTCTCCCNCTCTGCCGATTTCGGGTACTGACACGACCTGTCAGTGACTC

******** * ** ** *************** * ********* ***
Consensus:

TTTTTCTCcCacTCtGCcGATTTCGGGTACTGAcaCcaCCTGTCAGTaaCTC

>Bacillus_Fam_578_52_2 Nr. of seq. 2 Alignment length(with gaps) = 52
Alignment score = 0.666667
GCF_001315085.1_ASM131508v1_genomic.fna_3:24629-24950 Satlength=322 Nr of Repeats=4 RepeatLength=52 seed=GATGAAGACC Num.seqs=3 Similarity=0.846154
0   GATGAAGACCGGAATGAAGCTTGAGTGAGAGGAAAAAGGTATTCATGAGGTT
Rev.of_GCF_001439915.1_ASM143991v1_genomic.fna_122:112785-113045 Satlength=261 Nr of Repeats=5 RepeatLength=52 seed=GGTCTTCATA Num.seqs=5 Similarity=0.716049   10
TATGAAGACCTGAACGAAGCTTGAGCCTGCCCGAAAAGGTCTTCATCGGGTT

********* *** **********   *   ******* ***** ****
Consensus:

gATGAAGACCgGAAcGAAGCTTGAGccaGaccaAAAAGGTaTTCATcaGGTT

>Bacillus_Fam_579_52_2 Nr. of seq. 2 Alignment length(with gaps) = 52
Alignment score = 0.692308
GCF_001375535.1_Bacillus_niameyensis_genomic.fna_6:1820901-1821109 Satlength=209 Nr of Repeats=4 RepeatLength=52 seed=GCGAGTTCGG Num.seqs=4 Similarity=0.935897   0
GCGAGTTCGGGACGTGAGAGTCGCCATCATGACCCATCAACGCGACTTTTCC

Rev.of_GCF_001375535.1_Bacillus_niameyensis_genomic.fna_6:3265006-3265630 Satlength=625 Nr of Repeats=12 RepeatLength=52 seed=GCAAAAGTCG Num.seqs=12 Similarity=0.846154 0
GCGGATTCGGGACATGAGGGACTCCATCATGAACCATTGGCACGACTTTTGC

*** ******** **** * * ********* ****   * ******** *

Consensus:

GCGaaTTCGGGACaTGAGaGaCgCCATCATGAaCCATcaaCaCGACTTTTcC

>Bacillus_Fam_580_52_2 Nr. of seq. 2 Alignment length(with gaps) = 52 Alignment score = 0.705128
GCF_001375535.1_Bacillus_niameyensis_genomic.fna_6:2171941-2172148 Satlength=208 Nr of Repeats=4 RepeatLength=52 seed=GGCCCGTTGG Num.seqs=3 Similarity=0.888889 0
GGCCCGTTGGTTCTCTTTTTTGACACGAACGGGACATGATAAAGCNTCTCAT
GCF_001375535.1_Bacillus_niameyensis_genomic.fna_6:4192776-4193140 Satlength=365 Nr of Repeats=7 RepeatLength=52 seed=TCTCATGTCC Num.seqs=7 Similarity=0.829060 46
GTCCCGTAGGNTCCTTTTTTTTCCGCGAACGGTCCGTGATAAAGCGTCTCAT

* ***** ** ** ****** * ******* * ********* ******

Consensus:

GgCCCGTaGGtTCccTTTTTTgaCaCGAACGGgaCaTGATAAAGCgTCTCAT

>Bacillus_Fam_581_52_2 Nr. of seq. 2 Alignment length(with gaps) = 52 Alignment score = 0.653846
GCF_001420715.1_ASM142071v1_genomic.fna_22:1909065-1909431 Satlength=367 Nr of Repeats=7 RepeatLength=52 seed=ATGAAGGACA Num.seqs=5 Similarity=0.853846 0
ATGAAGGACAAGTTACACCAAGACAAAAAGCAAAATGTCCTTCATCAAGTGG
Rev.of_GCF_001420715.1_ASM142071v1_genomic.fna_22:3763575-3764043 Satlength=469 Nr of Repeats=9 RepeatLength=52 seed=ATGAAGGACA Num.seqs=9 Similarity=0.763627 45
ATGAAGGCCAAGTGACAGCAAGAAATTACGAANAGTGTCCTTCATNAGGTAA

******* ***** *** ***** * * * * * * ********** * **

Consensus:

ATGAAGGaCAAGTgACAcCAAGAaAaaAaGaAaAaTGTCCTTCATcAaGTaa

>Bacillus_Fam_582_52_2 Nr. of seq. 2 Alignment length(with gaps) = 52 Alignment score = 0.801282
GCF_001420715.1_ASM142071v1_genomic.fna_22:3674885-3675145 Satlength=261 Nr of Repeats=5 RepeatLength=52 seed=TGGTCTTTTG Num.seqs=5 Similarity=0.820513 40
NCCTTATAAAGACCATTCGGAACCGGATTCTTCCACCAATTGGTCTTTTGAT
GCF_001420715.1_ASM142071v1_genomic.fna_24:163648-164012 Satlength=365 Nr of Repeats=7 RepeatLength=52 seed=TAAAGACCAT Num.seqs=7 Similarity=0.832723 58
ACCTTATAAAGACCATTGGGATTCGGATTCTTCCTCACATTGGGCTTTTGAT

*** ******** *************** *** *********** * **

Consensus:

TGGgCTTTTGATaCCTTATAAAGACCATTcGGAacCGGATTCTTCCaCaaAT

>Bacillus_Fam_583_52_2 Nr. of seq. 2 Alignment length(with gaps) = 52
Alignment score = 0.775641
GCF_001439965.1_ASM143996v1_genomic.fna_121:145618-145878 Satlength=261
Nr of Repeats=4 RepeatLength=52 seed=AGTTCCCGAA Num.seqs=3
Similarity=0.794549 0
AGTTCCCGAACCCAGGCAAACTCCCCTTCCAGGGTAATNAATCGTTCACTTA
Rev.of_GCF_002009555.1_ASM200955v1_genomic.fna_550:13876-14084
Satlength=209 Nr of Repeats=4 RepeatLength=52 seed=CGGGAACTAA Num.seqs=4
Similarity=0.722222 8
AGTTCCCGTGCCTAGGCAAAATTCCCCTCCAGGGTAATTAATCGGTCACTTT

******** ** ******* * *** *********** ***** ******
Consensus:

AGTTCCCGaaCCcAGGCAAAaTcCCCcTCCAGGGTAATtAATCGgTCACTTa

>Bacillus_Fam_584_52_2 Nr. of seq. 2 Alignment length(with gaps) = 52
Alignment score = 0.666667
GCF_001591665.1_ASM159166v1_genomic.fna_18:135261-135572 Satlength=312 Nr of Repeats=6 RepeatLength=52 seed=TCAGTGCCCG Num.seqs=5
Similarity=0.784615 0
TCAGTGCCCGTGCAGGGTAAAAAAACAGGCTTATGGGCACTCAGACAGGTTG
GCF_001591665.1_ASM159166v1_genomic.fna_19:38371-38679 Satlength=309 Nr of Repeats=6 RepeatLength=52 seed=GGCACTCAGA Num.seqs=4
Similarity=0.830713
35 TCAGTGCCCATGGAGGGGGAAAATGAATGCCCACGGGCACTCAGAAAGGTTT

********* ** **** **** * ** * *********** *****
Consensus:

TCAGTGCCCaTGcAGGGgaAAAAaaaAgGCccAcGGGCACTCAGAaAGGTTg

>Bacillus_Fam_585_52_2 Nr. of seq. 2 Alignment length(with gaps) = 52
Alignment score = 0.647436
GCF_001591665.1_ASM159166v1_genomic.fna_57:39088-39504 Satlength=417 Nr of Repeats=8 RepeatLength=52 seed=ATGAGGACAG Num.seqs=8
Similarity=0.800366
0 ATGAGGACAGGTTAGGCCCTTGCTTAACGATTTTTGTCCTCATGAACACCTC
GCF_001591805.1_ASM159180v1_genomic.fna_32:17384-17591 Satlength=208 Nr of Repeats=4 RepeatLength=52 seed=GTCCTCATGA Num.seqs=3
Similarity=0.610063
35 CTGAGGACAATTTCAGCTCTTGCTCGNTGAGTTCTGTCCTCATGAACGCCCC

******** ** ** ****** ** ** ************* ** *
Consensus:

aTGAGGACAagTTaaGCcCTTGCTcaacGAgTTcTGTCCTCATGAACaCCcC

>Bacillus_Fam_586_52_2 Nr. of seq. 2 Alignment length(with gaps) = 52
Alignment score = 0.717949

GCF_001591805.1_ASM159180v1_genomic.fna_49:24442-24701 Satlength=260 Nr of Repeats=5 RepeatLength=52 seed=CTATTGGACA Num.seqs=4 Similarity=0.824786
0 CTATTGGACAAAACCCCCTTATAAAGCACCCAGAATGTCCATTAGAGCCCTT
Rev.of_GCF_001591805.1_ASM159180v1_genomic.fna_80:5111-5317 Satlength=207 Nr of Repeats=4 RepeatLength=52 seed=TTGTCCAATA Num.seqs=3 Similarity=0.880342 11
CTATTGGACAAAACTCTTTTATAAACCCCTTAAATTGTCCAATAGACCCCTT

************** * ******* * * * * ****** **** *****
Consensus:

CTATTGGACAAAACcCccTTATAAAcCaCccAaAaTGTCCAaTAGAcCCCTT

>Bacillus_Fam_587_52_2 Nr. of seq. 2 Alignment length(with gaps) = 52 Alignment score = 0.717949
GCF_001636315.1_ASM163631v1_genomic.fna_1:1902145-1902509 Satlength=365 Nr of Repeats=7 RepeatLength=52 seed=TGTGCACATT Num.seqs=7 Similarity=0.765568 0
TGTGCACATTGAAGCGTTCAATGAACACGAGAGAAAGGAATATCCACATAGA
Rev.of_GCF_001636315.1_ASM163631v1_genomic.fna_1:5398707-5398968 Satlength=262 Nr of Repeats=5 RepeatLength=52 seed=CTTCAATGTG Num.seqs=4 Similarity=0.696581 14
AGTGCACATTGAAGGCTTCAATGAACACGGGAGCAAGGGAAATCATCGAAGA

************* ************* *** **** * *** * ***
Consensus:

aGTGCACATTGAAGccTTCAATGAACACGaGAGaAAGGaAaATCaaCaaAGA

>Bacillus_Fam_588_52_2 Nr. of seq. 2 Alignment length(with gaps) = 52 Alignment score = 0.621795
GCF_001636325.1_ASM163632v1_genomic.fna_2:129056-129316 Satlength=261 Nr of Repeats=5 RepeatLength=52 seed=TTATTCCTCT Num.seqs=5 Similarity=0.941026
0 TTATTCCTCTGAAGAAGAAAGAATGAGCGGTCTTCCGGTGAATAACCAGAGT
GCF_001636325.1_ASM163632v1_genomic.fna_3:419541-420114 Satlength=574 Nr of Repeats=11 RepeatLength=52 seed=TTCAGGTGAA Num.seqs=10 Similarity=0.909402 32
TTATTCCCCTGAAGCAGCNCAAAAGCACCTTGTTCAGGTGAATAACCACGGT

******* ****** **   ** * * * *** ************ **
Consensus:

TTATTCCcCTGAAGaAGaaaaAAaGaaCcgTcTTCaGGTGAATAACCAcaGT

>Bacillus_Fam_589_52_2 Nr. of seq. 2 Alignment length(with gaps) = 52 Alignment score = 0.641026
GCF_001636335.1_ASM163633v1_genomic.fna_1:413718-414550 Satlength=833 Nr of Repeats=16 RepeatLength=52 seed=AAGAAAAAGT Num.seqs=16 Similarity=0.861325 28
ATGAAGGACAAAACGCAATGAGCAAAGGAAGAAAAAGTCCATCATAGGGCGA
Rev.of_GCF_002019635.1_ASM201963v1_genomic.fna_2:1603355-1603717 Satlength=363 Nr of Repeats=7 RepeatLength=52 seed=TGAAGGACTT Num.seqs=6

Similarity=0.717695 44
ATGAAAGACAAAAGCCAACCGTCACAGAAGGGAAAAGTCCTTCATAGGGGCA

* * ******** ******** ****** ******* ***   ** **
Consensus:

AaGaAAAAGTCCaTCATAGGGccAATGAAaGACAAAAccCAAccagCAaAGa

>Bacillus_Fam_590_52_2 Nr. of seq. 2 Alignment length(with gaps) = 52
Alignment score = 0.698718
GCF_001636335.1_ASM163633v1_genomic.fna_1:1586996-1587464 Satlength=469
Nr of Repeats=9 RepeatLength=52 seed=ATGATGGACT Num.seqs=9
Similarity=0.908832 0
ATGATGGACTTTTCTGGCTTTNCCTGAATCGGTTTTGTCCTTCATACCCTTT
Rev.of_GCF_001636335.1_ASM163633v1_genomic.fna_2:265356-265928
Satlength=573 Nr of Repeats=11 RepeatLength=52 seed=ATCATCAAGG
Num.seqs=11 Similarity=0.870862
5   ATGATGGACTTTTTCCGCGTTCCCTCACACGCTTTTGTCCTTCAGAACCTTG

*************   ** ** *** * ** ************ * ****
Consensus:

ATGATGGACTTTTcccGCgTTcCCTcAaaCGcTTTTGTCCTTCAgAaCCTTg

>Bacillus_Fam_591_52_2 Nr. of seq. 2 Alignment length(with gaps) = 52
Alignment score = 0.769231
GCF_001636345.1_ASM163634v1_genomic.fna_1:791126-791369 Satlength=244 Nr
of Repeats=4 RepeatLength=52 seed=CTGGATTATA Num.seqs=3
Similarity=0.811966
0   CTGGATTATAGAGGTGTGTTTCGCGCTTTATCGGGACTCCGATTACCAAAAT
Rev.of_GCF_001636345.1_ASM163634v1_genomic.fna_1:2232952-2233471
Satlength=520 Nr of Repeats=10 RepeatLength=52 seed=GAGAAACACA
Num.seqs=8 Similarity=0.748168
24   CTGGATTATAGAGGTGTGTTTCTCGCCGAATTGGGACACTGTTTACCAAATT

********************* ***   ** ***** * * ******** *
Consensus:

CTGGATTATAGAGGTGTGTTTCgCGCcgaATcGGGACaCcGaTTACCAAAaT

>Bacillus_Fam_592_52_2 Nr. of seq. 2 Alignment length(with gaps) = 52
Alignment score = 0.647436
GCF_001636345.1_ASM163634v1_genomic.fna_1:1139536-1139952 Satlength=417
Nr of Repeats=8 RepeatLength=52 seed=GGAGAAACGA Num.seqs=8
Similarity=0.882784 0
GGAGAAACGAGCCCGAAAAGTCCAGTAACTGGAAAAAAGTGTCGTGTAAACA
GCF_001636345.1_ASM163634v1_genomic.fna_1:1221728-1222246 Satlength=519
Nr of Repeats=10 RepeatLength=52 seed=GTATATGGAA Num.seqs=9
Similarity=0.850427 24
GGNTAAACGAGACACAATAGTCCAGTATATGGAAAACAGAGTCGTGATTACG

** ******* * ** ********* ******* ** ******   **
Consensus:

GGagAAACGAGaCacAAaAGTCCAGTAaaTGGAAAAaAGaGTCGTGaaaACa

>Bacillus_Fam_593_52_2 Nr. of seq. 2 Alignment length(with gaps) = 52
Alignment score = 0.608974
GCF_001636425.1_ASM163642v1_genomic.fna_1:697484-697900 Satlength=417 Nr of Repeats=8 RepeatLength=52 seed=TCATGAAGGA Num.seqs=8
Similarity=0.717880
0 TCATGAAGGACGTNTCCCTGGATATAGCAGCTCGAATTGTCCTTCATCACGC
Rev.of_GCF_001636425.1_ASM163642v1_genomic.fna_1:4328531-4328895
Satlength=365 Nr of Repeats=7 RepeatLength=52 seed=AAAGTCCTTC Num.seqs=7
Similarity=0.868132 14
TCATGAAGGACTTTTCTCAAGACATCGGACANCCAAAAGTCCTTCATCNCCC

*********** * ** * ** ** * * * ** ********** * *
Consensus:

TCATGAAGGACgTtTCcCaaGAcATaGcAcatCcAAaaGTCCTTCATCaCcC

>Bacillus_Fam_594_52_2 Nr. of seq. 2 Alignment length(with gaps) = 52
Alignment score = 0.974359
GCF_001887185.1_ASM188718v1_genomic.fna_162:152517-152725 Satlength=209
Nr of Repeats=4 RepeatLength=52 seed=CACATTTTTC Num.seqs=4
Similarity=0.854701 0
CACATTTTTCTGCATTTCGCTCCATTATCTGTTTCAAAACCTCTAGTTGACG
GCF_002009555.1_ASM200955v1_genomic.fna_224:12277-12640 Satlength=364 Nr of Repeats=7 RepeatLength=52 seed=ACATTTTTCT Num.seqs=6
Similarity=0.852991
1 CACATTTTTCTGCATTTCGCTCCATTATCTGTTTCAAAACCTCGAGTTGACG

*** ********
Consensus:

CACATTTTTCTGCATTTCGCTCCATTATCTGTTTCAAAACCTCgAGTTGACG

>Bacillus_Fam_595_52_2 Nr. of seq. 2 Alignment length(with gaps) = 52
Alignment score = 0.929487
GCF_001887185.1_ASM188718v1_genomic.fna_178:71698-72478 Satlength=781 Nr of Repeats=15 RepeatLength=52 seed=GGGCACCTTT Num.seqs=15
Similarity=0.850305 0
GGGCACCTTTCTCCCTCTTTCGTTACCGCGAAACCGNTTTTTCCTCTCTAGC
Rev.of_GCF_002009555.1_ASM200955v1_genomic.fna_534:4610-5026
Satlength=417 Nr of Repeats=8 RepeatLength=52 seed=AAAGGTGCCC Num.seqs=8
Similarity=0.880952 10
GGGCACCTTTCTCCCTCTTTCGTTACCGCGAAACCGCTTTTTCCTCTCGGGC

************************************ *********** **
Consensus:

GGGCACCTTTCTCCCTCTTTCGTTACCGCGAAACCGcTTTTTCCTCTCgaGC

>Bacillus_Fam_596_52_2 Nr. of seq. 2 Alignment length(with gaps) = 52
Alignment score = 0.794872

GCF_900109925.1_IMG-taxon_2651870116_annotated_assembly_genomic.fna_16:433491-433753 Satlength=263 Nr of Repeats=5 RepeatLength=52 seed=CTTCAATGTT Num.seqs=3 Similarity=0.914530 0
CTTCAATGTTACCGTTTCTATGATTTCAATAACTTTTGGTCACATTGAGCT
Rev.of_GCF_900109925.1_IMG-taxon_2651870116_annotated_assembly_genomic.fna_34:7995-8202 Satlength=208 Nr of Repeats=4 RepeatLength=52 seed=CAGAGAAACG Num.seqs=3 Similarity=0.948718 22
CTTCAATGTGACCGTTTCTCTGATTTTACTTGCGTTTTGGTCACATTGAGCC

********* ********* ****** * * * ****************
Consensus:

CTTCAATGTgACCGTTTCTaTGATTTcAaTaaCgTTTTGGTCACATTGAGCc

>Bacillus_Fam_597_52_2 Nr. of seq. 2 Alignment length(with gaps) = 52
Alignment score = 0.955128
GCF_900156875.1_PRJEB18969_genomic.fna_11:364264-364471 Satlength=208 Nr of Repeats=4 RepeatLength=52 seed=ATGAAGACCG Num.seqs=3 Similarity=0.863248
0   ATGAAGACCGTTAGTTTTGGTGAATGAGCAAGAACCGGTTATCATAGTTGAG
GCF_900156875.1_PRJEB18969_genomic.fna_11:367968-368330 Satlength=363 Nr of Repeats=7 RepeatLength=52 seed=AACCGGTTAT Num.seqs=5 Similarity=0.858974
32   ATGAAGACCGTTAGTTTTGGTGAATGCNCAAGAACCGGTTATCATAGTTGAG

************************* ***********************
Consensus:

ATGAAGACCGTTAGTTTTGGTGAATGagCAAGAACCGGTTATCATAGTTGAG

>Bacillus_Fam_598_52_2 Nr. of seq. 2 Alignment length(with gaps) = 52
Alignment score = 1.000000
GCF_900156875.1_PRJEB18969_genomic.fna_11:1808457-1808873 Satlength=417 Nr of Repeats=8 RepeatLength=52 seed=GGCTTTCATT Num.seqs=8 Similarity=0.930403 0
GGCTTTCATTCTTCTCATGATGACCTTTTCTCTAGCTCTCTAGAATGATTTC
GCF_900156875.1_PRJEB18969_genomic.fna_11:1817955-1818163 Satlength=209 Nr of Repeats=4 RepeatLength=52 seed=ATTTCGGCTT Num.seqs=4 Similarity=0.948718 47
GGCTTTCATTCTTCTCATGATGACCTTTTCTCTAGCTCTCTAGAATGATTTC

****
Consensus:

GGCTTTCATTCTTCTCATGATGACCTTTTCTCTAGCTCTCTAGAATGATTTC

>Bacillus_Fam_599_51_2 Nr. of seq. 2 Alignment length(with gaps) = 51
Alignment score = 0.712418
GCF_000292245.2_ASM29224v2_genomic.fna_72:15429-15633 Satlength=205 Nr of Repeats=4 RepeatLength=51 seed=TTTCATTACC Num.seqs=4 Similarity=0.818910
82   TATTCGTACCCTAATTAAGGCAACCCTCCATTTTCATTACCAATCCCCTGT

GCF_000292245.2_ASM29224v2_genomic.fna_80:13385-13741 Satlength=357 Nr of Repeats=7 RepeatLength=51 seed=TTTTTTCGTA Num.seqs=6 Similarity=0.836752
100 TTTTCGTACCCTAATTCAGCCAACACTCGAGTTTCATTACCTATTACCATT

********** ** ** ** ************** ** **** *** *
Consensus:

TTTCATTACCaATcaCCagTTaTTCGTACCCTAATTaAGcCAACaCTCcAg

>Bacillus_Fam_600_51_2 Nr. of seq. 2 Alignment length(with gaps) = 51
Alignment score = 0.895425
GCF_000292245.2_ASM29224v2_genomic.fna_92:17-274 Satlength=258 Nr of Repeats=5 RepeatLength=51 seed=TTCGGCTTAT Num.seqs=3 Similarity=0.895425
0 TTCGGCTTATAATTCTTACTCTCCATTCTTTTTACGCCGAACTCTCTCTTA
Rev.of_GCF_000292245.2_ASM29224v2_genomic.fna_125:10-369 Satlength=360 Nr of Repeats=7 RepeatLength=51 seed=TCGGCGTAAA Num.seqs=5
Similarity=0.832680 40
TTCGGCTTATAATTCTTTTTCTCCATTCTTTTTACGCCGAACTGGCTCTTA

***************** ************************ ******
Consensus:

TTCGGCTTATAATTCTTacTCTCCATTCTTTTTACGCCGAACTcgCTCTTA

>Bacillus_Fam_601_51_2 Nr. of seq. 2 Alignment length(with gaps) = 51
Alignment score = 1.000000
GCF_001420645.1_ASM142064v1_genomic.fna_44:115239-115443 Satlength=205 Nr of Repeats=5 RepeatLength=51 seed=TTCTTGCTCT Num.seqs=3
Similarity=0.912854 0
TTCTTGCTCTTCTCTAGCATCGTCCTCGCTGAGCTCTTCTGTACTGTCTTC
Rev.of_GCF_002019765.1_ASM201976v1_genomic.fna_1:514830-515034
Satlength=205 Nr of Repeats=5 RepeatLength=51 seed=GAGAAGAGCA Num.seqs=3
Similarity=0.912854 14
TTCTTGCTCTTCTCTAGCATCGTCCTCGCTGAGCTCTTCTGTACTGTCTTC

****
Consensus:

TTCTTGCTCTTCTCTAGCATCGTCCTCGCTGAGCTCTTCTGTACTGTCTTC

>Bacillus_Fam_602_51_2 Nr. of seq. 2 Alignment length(with gaps) = 51
Alignment score = 1.000000
GCF_002019665.1_ASM201966v1_genomic.fna_1:857991-858195 Satlength=205 Nr of Repeats=4 RepeatLength=51 seed=AAAAGGAAAG Num.seqs=4
Similarity=0.908497
0 AAAAGGAAAGCGCCAAAAGCCCGTTTGTGAACACGAAAATCGAAAAACAGG
GCF_002019665.1_ASM201966v1_genomic.fna_1:859725-860133 Satlength=409 Nr of Repeats=8 RepeatLength=51 seed=CGTTTGTGAA Num.seqs=8
Similarity=0.884220
21 AAAAGGAAAGCGCCAAAAGCCCGTTTGTGAACACGAAAATCGAAAAACAGG

****
Consensus:

AAAAGGAAAGCGCCAAAAGCCCGTTTGTGAACACGAAAATCGAAAAACAGG

>Bacillus_Fam_603_48_2 Nr. of seq. 2 Alignment length(with gaps) = 48
Alignment score = 1.000000
GCF_000153365.1_ASM15336v1_genomic.fna_17:211270-211939 Satlength=670 Nr of Repeats=14 RepeatLength=48 seed=CAGATAGAGG Num.seqs=13 Similarity=0.835470 0
CAGATAGAGGAGGTTGAAGAACCGGAGCAAACTGAGGAGTCAGAAGAG
Rev.of_GCF_000473245.1_ASM47324v1_genomic.fna_1:4315874-4316543 Satlength=670 Nr of Repeats=14 RepeatLength=48 seed=CCTCTATCTG Num.seqs=13 Similarity=0.839744
10   CAGATAGAGGAGGTTGAAGAACCGGAGCAAACTGAGGAGTCAGAAGAG

****
Consensus:

CAGATAGAGGAGGTTGAAGAACCGGAGCAAACTGAGGAGTCAGAAGAG

>Bacillus_Fam_604_48_2 Nr. of seq. 2 Alignment length(with gaps) = 48
Alignment score = 0.951389
GCF_000712615.1_ASM71261v1_genomic.fna_22:80497-81025 Satlength=529 Nr of Repeats=11 RepeatLength=48 seed=TTGGATTATC Num.seqs=11
Similarity=0.853535
0   TTGGATTATCCACTGCATTACTTTCTTCTTGTGAAACAGCACTATTAC
GCF_000712615.1_ASM71261v1_genomic.fna_22:80497-81774 Satlength=1278 Nr of Repeats=16 RepeatLength=48 seed=TTGGATTATC Num.seqs=15 Similarity=0.798942 0
TTGGATTATCCACTGCATTACTNTCTTCTTGAGAAACAGCACTATTAC

********************** ******** ****************
Consensus:

TTGGATTATCCACTGCATTACTtTCTTCTTGaGAAACAGCACTATTAC

>Bacillus_Fam_605_48_2 Nr. of seq. 2 Alignment length(with gaps) = 48
Alignment score = 0.694444
GCF_000715205.1_SOAPdenovo_v1.05_genomic.fna_12:101104-101903
Satlength=800 Nr of Repeats=6 RepeatLength=48 seed=TCTTTTGGCT Num.seqs=5
Similarity=0.933333 0
TCTTTTGGCTCTTCCATTGACCCATCACCAGGCTTTGTTCCGTCCTGA
Rev.of_GCF_001578205.1_ASM157820v1_genomic.fna_1:1255656-1255848
Satlength=193 Nr of Repeats=4 RepeatLength=48 seed=GATCAGGATG Num.seqs=4
Similarity=0.907596 2
TCTTTCGGTTCTTCTGTTGTGCCATCGTCAGGTTTTGTCCCATCCTGA

***** ** ***** *** ***** **** ***** ** ******
Consensus:

TCTTTcGGcTCTTCcaTTGacCCATCacCAGGcTTTGTcCCaTCCTGA

>Bacillus_Fam_606_47_2 Nr. of seq. 2 Alignment length(with gaps) = 47
Alignment score = 1.000000

GCF_000153365.1_ASM15336v1_genomic.fna_34:533-722 Satlength=190 Nr of Repeats=4 RepeatLength=47 seed=TCCGTGGACA Num.seqs=3 Similarity=0.981087
0 TCCGTGGACAGATGGGGTGAAGTGTCTTTTTGTGGTGCCAGGTACGC
Rev.of_GCF_000473245.1_ASM47324v1_genomic.fna_1:4144621-4144810 Satlength=190 Nr of Repeats=4 RepeatLength=47 seed=AAAAGACACT Num.seqs=3 Similarity=0.943262 30
TCCGTGGACAGATGGGGTGAAGTGTCTTTTTGTGGTGCCAGGTACGC

****
Consensus:

TCCGTGGACAGATGGGGTGAAGTGTCTTTTTGTGGTGCCAGGTACGC

>Bacillus_Fam_607_46_2 Nr. of seq. 2 Alignment length(with gaps) = 46
Alignment score = 0.753623
GCF_001274915.1_ASM127491v1_genomic.fna_25:723071-723296 Satlength=226 Nr of Repeats=5 RepeatLength=45 seed=CCAGGTGAAG Num.seqs=5 Similarity=0.760000 0
CCAGGTGAAG-GAGAATCTGGCCAACCGGAAGAGGAAGTAACGGAG
Rev.of_GCF_000011145.1_ASM1114v1_genomic.fna_1:3424216-3424396 Satlength=181 Nr of Repeats=4 RepeatLength=45 seed=CTTCACCTGG Num.seqs=4 Similarity=0.737923 10
CCAGGTGAAGAGGGAATCTGGTCAGCCAGAGGAAGAAATAACGGAG

********** * ******** ** ** ** ** *** ********
Consensus:

CCAGGTGAAGaGaGAATCTGGcCAaCCaGAaGAaGAAaTAACGGAG

>Bacillus_Fam_608_45_2 Nr. of seq. 2 Alignment length(with gaps) = 45
Alignment score = 0.629630
GCF_000290995.1_Baci_cere_AND1407_G13175_V1_genomic.fna_2:809286-809454 Satlength=169 Nr of Repeats=4 RepeatLength=42 seed=AATCCAACGA Num.seqs=4 Similarity=0.962963 0
AATCCAACGACACCACCAA---CAAGTGAAGGAAATAATGGAGAA
Rev.of_GCF_000712615.1_ASM71261v1_genomic.fna_1:387890-388565 Satlength=676 Nr of Repeats=15 RepeatLength=45 seed=CCACCTGTCG Num.seqs=15 Similarity=0.988713
29 AATCCAACAACACCACCAACGACAGGTGGAGACAACAACGGGGAT

******** *********   ** *** ** ** ** ** **
Consensus:

AATCCAACaACACCACCAAcgaCAaGTGaAGaaAAcAAcGGaGAa

>Bacillus_Fam_609_45_2 Nr. of seq. 2 Alignment length(with gaps) = 45
Alignment score = 0.614815
GCF_000612625.1_JCE_genomic.fna_6:32809-33331 Satlength=523 Nr of Repeats=6 RepeatLength=42 seed=GGCATTTGAC Num.seqs=4 Similarity=0.888889
0 GGCATTTGACCGTATCCCGCTGGCA-TTTCT--CCAGCACCCATT
GCF_001591445.1_ASM159144v1_genomic.fna_9:113119-113479 Satlength=361 Nr of Repeats=7 RepeatLength=45 seed=GGCATTTGTC Num.seqs=6 Similarity=0.741844
0 GGCATTTGTCCATATCCTGCTGGTACTTGCTGACCATAACCCTGT

******** ** ***** ***** * ** ** *** **** *
Consensus:

GGCATTTGaCCaTATCCcGCTGGcAcTTgCTgaCCAgaACCCagT

>Bacillus_Fam_610_45_2 Nr. of seq. 2 Alignment length(with gaps) = 45
Alignment score = 0.874074
GCF_000161455.1_ASM16145v1_genomic.fna_1:4124623-4124845 Satlength=223 Nr of Repeats=5 RepeatLength=45 seed=GAAAAAAGAA Num.seqs=4 Similarity=0.851852 0
GAAAAAAGAAGCAGCAATGCAAGAACAACAAAAACAAGATAAAAT
GCF_000712615.1_ASM71261v1_genomic.fna_21:61395-61572 Satlength=178 Nr of Repeats=4 RepeatLength=45 seed=GAAAAAAGAA Num.seqs=3 Similarity=0.628118
0 GAAAAAAGAAGCAGCAATTCAGNNACAACAAAAACAAGATNAAAT

***************** **   *************** ****
Consensus:

GAAAAAAGAAGCAGCAATgCAagaACAACAAAAACAAGATaAAAT

>Bacillus_Fam_611_45_2 Nr. of seq. 2 Alignment length(with gaps) = 45
Alignment score = 0.822222
GCF_000724485.1_ASM72448v1_genomic.fna_1:628866-629091 Satlength=226 Nr of Repeats=5 RepeatLength=45 seed=GATTCCTCAT Num.seqs=5 Similarity=0.952593
0 GATTCCTCATCAGATTCATATTCATCGCTTGACTCGTTATCTTCA
GCF_000724485.1_ASM72448v1_genomic.fna_1:959830-960010 Satlength=181 Nr of Repeats=4 RepeatLength=45 seed=TCTTCAGATT Num.seqs=4 Similarity=0.925926
39 GATTCCTCGTCGGATTCGTAACCATCGCTAGACTCGTTATCTTCA

******** ** ***** ** ******* ***************
Consensus:

GATTCCTCaTCaGATTCaTAacCATCGCTaGACTCGTTATCTTCA

>Bacillus_Fam_612_43_2 Nr. of seq. 2 Alignment length(with gaps) = 43
Alignment score = 0.837209
GCF_000175075.1_ASM17507v1_genomic.fna_41:65761-65929 Satlength=169 Nr of Repeats=4 RepeatLength=42 seed=GAAGCACCGG Num.seqs=4 Similarity=0.915344
0 GAAGC-ACCGGCTGAAGAACCTGTAACAGAGGAACCAGCAGAG
GCF_001648575.1_ASM164857v1_genomic.fna_67:24594-24762 Satlength=169 Nr of Repeats=4 RepeatLength=42 seed=GCTGAAGAGC Num.seqs=4 Similarity=0.906977
10 GAAGCTCCCNGCTGAAGAGCCTGTAACAGAGGCACCAGCAGAG

***** ** ******** ************* **********
Consensus:

GAAGCtaCCgGCTGAAGAaCCTGTAACAGAGGaACCAGCAGAG

>Bacillus_Fam_613_43_2 Nr. of seq. 2 Alignment length(with gaps) = 43
Alignment score = 0.689922

GCF_000430785.1_ASM43078v1_genomic.fna_16:70154-71534 Satlength=1381 Nr of Repeats=21 RepeatLength=42 seed=CATGCCGAAG Num.seqs=15 Similarity=0.532988 0 CATGCNGAAGGTNNNNNTACAACNGCAAGT-GGANATNCTTCN
GCF_000430785.1_ASM43078v1_genomic.fna_16:70112-70730 Satlength=619 Nr of Repeats=9 RepeatLength=42 seed=CATGCGGAAG Num.seqs=6 Similarity=0.576227 0   CATGCGGAAGGTNAAANCACAACAGCAAGTGGGANATGCTTCN

***** *******   * ***** ****** ****** *****
Consensus:

CATGCgGAAGGTNaaaNcACAACaGCAAGTgGGANATgCTTCN

>Bacillus_Fam_614_43_2 Nr. of seq. 2 Alignment length(with gaps) = 43 Alignment score = 0.732558
GCF_000712615.1_ASM71261v1_genomic.fna_10:62613-63201 Satlength=589 Nr of Repeats=14 RepeatLength=42 seed=CCTTCCGCAT Num.seqs=14 Similarity=0.614123
0   CCTTCCGCATGCGAGNCAANTCCACTGGCTATTGTATTAANT-
GCF_000712615.1_ASM71261v1_genomic.fna_10:65183-65897 Satlength=715 Nr of Repeats=16 RepeatLength=42 seed=CCTTCCGCAT Num.seqs=15 Similarity=0.501622
0   CCTTCCGCATGCGANNNANCTCCACTGGCTATTGTNTNCANNN

************** * * *************** * **
Consensus:

CCTTCCGCATGCGAgNcAacTCCACTGGCTATTGTaTtaANtn

>Bacillus_Fam_615_43_2 Nr. of seq. 2 Alignment length(with gaps) = 43 Alignment score = 0.658915
GCF_000986785.1_ASM98678v1_genomic.fna_101:89286-89496 Satlength=211 Nr of Repeats=4 RepeatLength=42 seed=GGAGGAGCGA Num.seqs=3 Similarity=0.873016
0   GGAGGAGCGATGGACGCCCAA-ATGCCGCACATGCCGCAGGTG
Rev.of_GCF_900109925.1_IMG-taxon_2651870116_annotated_assembly_genomic.fna_16:169506-169773 Satlength=268 Nr of Repeats=6 RepeatLength=42 seed=CTTGCGGCAT Num.seqs=5 Similarity=0.917460 40   GCAGGAGCAATGGATGCGCAATAT-CCACATATGCCGCAAGTA

* ****** ***** ** *** ** ** ** ******** **
Consensus:

GcAGGAGCaATGGAcGCcCAAtATgCCaCAcATGCCGCAaGTa

>Bacillus_Fam_616_42_2 Nr. of seq. 2 Alignment length(with gaps) = 42 Alignment score = 0.793651
GCF_000429725.1_ASM42972v1_genomic.fna_2:332298-332770 Satlength=473 Nr of Repeats=5 RepeatLength=41 seed=CGATTGTGAC Num.seqs=3 Similarity=0.913279
0   CGATTGTGACCAAGCGCGGCG-AAACTCCGGCCAATTGGCGA
Rev.of_GCF_000429725.1_ASM42972v1_genomic.fna_10:90786-91168 Satlength=383 Nr of Repeats=8 RepeatLength=41 seed=AATTGTCCGC Num.seqs=6

Similarity=0.785185 37
AGATTGTGACCAAGCGCGGCGNAAAGTGCGGACAATTGGCGG

******************* *** * *** *********
Consensus:

aGATTGTGACCAAGCGCGGCGnAAAcTcCGGaCAATTGGCGa

>Bacillus_Fam_617_42_2 Nr. of seq. 2 Alignment length(with gaps) = 42
Alignment score = 0.690476
GCF_000504145.1_SOAPdenovo_for_version2.04_of_Bacillus_sp._FJAT-14515_genome_genomic.fna_1:1999822-2000150 Satlength=329 Nr of Repeats=8 RepeatLength=41 seed=ATTTCGGATT Num.seqs=8 Similarity=0.809524 0
ATTTCG-GATTTGCGGGCCGGAATCCATACTTTTCATGCCGG
Rev.of_GCF_000504145.1_SOAPdenovo_for_version2.04_of_Bacillus_sp._FJAT-14515_genome_genomic.fna_23:124770-125136 Satlength=367 Nr of Repeats=9 RepeatLength=41 seed=CGCAAATCAC Num.seqs=7 Similarity=0. 14 A-
TTCGTGATTTGCGTGCCAGAACACACTCTTTTCNTGCCGG

* **** ******** *** *** ** ****** ******
Consensus:

AtTTCGtGATTTGCGgGCCaGAAcaCAcaCTTTTCaTGCCGG

>Bacillus_Fam_618_42_2 Nr. of seq. 2 Alignment length(with gaps) = 42
Alignment score = 0.638889
GCF_002019665.1_ASM201966v1_genomic.fna_1:3639369-3639982 Satlength=614 Nr of Repeats=15 RepeatLength=41 seed=TTCGTCCCTA Num.seqs=13
Similarity=0.892850 0
TTCG-TCCCTATCCGTCGGTAAATCCCTTCATTCGTTGGTAG
Rev.of_GCF_002019665.1_ASM201966v1_genomic.fna_1:4598013-4598833
Satlength=821 Nr of Repeats=20 RepeatLength=41 seed=TTACCAACGA
Num.seqs=20 Similarity=0.827213
0 -CCGATCGCTATGCGTGGGTAAAACTCGTTATTCGTTGGTAA

** ** **** *** ****** * * * ***********
Consensus:

tcCGaTCcCTATcCGTcGGTAAAaCcCgTcATTCGTTGGTAa

>Bacillus_Fam_619_42_2 Nr. of seq. 2 Alignment length(with gaps) = 42
Alignment score = 0.809524
GCF_000007825.1_ASM782v1_genomic.fna_1:3435556-3435724 Satlength=169 Nr of Repeats=4 RepeatLength=42 seed=GGAATAACTG Num.seqs=4
Similarity=0.968254
0 GGAATAACTGGTCCTACTGGGGTTACAGGTTCTACCGGAATC
GCF_000008505.1_ASM850v1_genomic.fna_1:3325303-3325513 Satlength=211 Nr of Repeats=5 RepeatLength=42 seed=GGAATTGGCA Num.seqs=5
Similarity=0.923810
36 GGCATTACTGGTCCTACTGGAGTTACAGGACCTACCGGAATT

** ** ************* ******** **********
Consensus:

GGaATaACTGGTCCTACTGGaGTTACAGGacCTACCGGAATc

>Bacillus_Fam_620_42_2 Nr. of seq. 2 Alignment length(with gaps) = 42
Alignment score = 1.000000
GCF_000009825.1_ASM982v1_genomic.fna_1:3313462-3313630 Satlength=169 Nr of Repeats=4 RepeatLength=42 seed=CTTGCTTTCA Num.seqs=4 Similarity=0.899471
0 CTTGCTTTCAATGATGAAATGCGCCATTTCCAAAAGGAGCAG
GCF_900142675.1_IMG-taxon_2654588196_annotated_assembly_genomic.fna_7:92101-92269 Satlength=169 Nr of Repeats=4 RepeatLength=42 seed=CTTGCTTTCA Num.seqs=4 Similarity=0.920635 0
CTTGCTTTCAATGATGAAATGCGCCATTTCCAAAAGGAGCAG

****
Consensus:

CTTGCTTTCAATGATGAAATGCGCCATTTCCAAAAGGAGCAG

>Bacillus_Fam_621_42_2 Nr. of seq. 2 Alignment length(with gaps) = 42
Alignment score = 0.809524
GCF_000169195.2_ASM16919v2_genomic.fna_1:2452745-2452955 Satlength=211 Nr of Repeats=5 RepeatLength=42 seed=GGATCAGGTT Num.seqs=5 Similarity=0.841270 0
GGATCAGGTTCTGCATCCGGAACGACCGCAACGGGCAACACC
Rev.of_GCF_000832905.1_ASM83290v1_genomic.fna_1:1285270-1285522 Satlength=253 Nr of Repeats=6 RepeatLength=42 seed=GAACCCGATC Num.seqs=6 Similarity=0.832804 11
GGATCGGGTTCCGCGTCCGGGACGACTGCAACGGGCAACACT

***** ***** ** ***** ***** **************
Consensus:

GGATCaGGTTCcGCaTCCGGaACGACcGCAACGGGCAACACc

>Bacillus_Fam_622_42_2 Nr. of seq. 2 Alignment length(with gaps) = 42
Alignment score = 1.000000
GCF_000186145.1_Bacillus_sp_2_A_57_CT2_V1_genomic.fna_2:304102-304312 Satlength=211 Nr of Repeats=6 RepeatLength=42 seed=TTTGATTCGG Num.seqs=4 Similarity=0.915344 0
TTTGATTCGGTTGATCAGAAGTTTGATTCCATTGATCAGAGA
GCF_000294775.2_ASM29477v2_genomic.fna_1:4910289-4910520 Satlength=232 Nr of Repeats=6 RepeatLength=42 seed=GATTCCATTG Num.seqs=5 Similarity=0.905426 24
TTTGATTCGGTTGATCAGAAGTTTGATTCCATTGATCAGAGA

****
Consensus:

TTTGATTCGGTTGATCAGAAGTTTGATTCCATTGATCAGAGA

>Bacillus_Fam_623_42_2 Nr. of seq. 2 Alignment length(with gaps) = 42
Alignment score = 0.650794

GCF_000299035.1_ASM29903v1_genomic.fna_5:177403-178117 Satlength=715 Nr of Repeats=17 RepeatLength=42 seed=AGTTGTAGTG Num.seqs=17 Similarity=0.981793 0 AGTTGTAGTGGAAACTCGAGAAGAAGAGAGACCAGTAGAGCG
Rev.of_GCF_000712615.1_ASM71261v1_genomic.fna_6:43769-44105 Satlength=337 Nr of Repeats=8 RepeatLength=42 seed=ACTACTTCTT Num.seqs=8 Similarity=0.878685 9 AGAAGTAGTGGAGGCTCAAGCCGAAGAGAGACCGGTACATCA

** ******** *** ** *********** *** * *
Consensus:

AGaaGTAGTGGAaaCTCaAGaaGAAGAGAGACCaGTAcAgCa

>Bacillus_Fam_624_42_2 Nr. of seq. 2 Alignment length(with gaps) = 42
Alignment score = 0.690476
GCF_000307875.1_BABA1.0_genomic.fna_120:5376-5682 Satlength=307 Nr of Repeats=7 RepeatLength=42 seed=GGAGAAATGC Num.seqs=5 Similarity=0.885714
0   GGAGAAATGCCACAAGGTTACGGCCAGATGCCGACAGGNTAC
GCF_000508325.1_BAVI_1_genomic.fna_148:13-268 Satlength=256 Nr of Repeats=5 RepeatLength=42 seed=AATGCCGGGA Num.seqs=4 Similarity=0.814815
5   GGAGAAATGCCGGGAGCCTATGGCCAAATGCCAGCAGGATAC

***********   ** ** ***** ***** **** ***
Consensus:

GGAGAAATGCCacaAGccTAcGGCCAaATGCCaaCAGGaTAC

>Bacillus_Fam_625_42_2 Nr. of seq. 2 Alignment length(with gaps) = 42
Alignment score = 0.730159
GCF_000331575.1_ASM33157v1_genomic.fna_2:519806-520142 Satlength=337 Nr of Repeats=8 RepeatLength=42 seed=AGATTCGTGG Num.seqs=8 Similarity=0.833333
0   AGATTCGTGGGTAAAGCACCGGGTTATGTGGGTATTCTACTA
GCF_000331575.1_ASM33157v1_genomic.fna_6:31803-31970 Satlength=168 Nr of Repeats=4 RepeatLength=42 seed=GAGTTATATG Num.seqs=3 Similarity=0.839793
20   AAACTCGTGGGTAAANCNCCGAGTTATATGGGTATTCCACCC

* * *********** * *** ***** ********* **
Consensus:

AaAcTCGTGGGTAAAgCaCCGaGTTATaTGGGTATTCcACca

>Bacillus_Fam_626_42_2 Nr. of seq. 2 Alignment length(with gaps) = 42
Alignment score = 0.968254
GCF_000496285.1_ASM49628v1_genomic.fna_1:4580071-4580491 Satlength=421 Nr of Repeats=10 RepeatLength=42 seed=CCAGGAGGCG Num.seqs=10 Similarity=0.899824 0 CCAGGAGGCGGAACGGGAACAGATAACGGAACCGGAGGAAAC
GCF_000831065.1_ASM83106v1_genomic.fna_1:1836001-1836505 Satlength=505 Nr of Repeats=12 RepeatLength=42 seed=CCAGGAGGCG Num.seqs=12 Similarity=0.956229 0 CCAGGAGGCGGAACGGGAACAGACAACGGAACCGGAGGAAAC

********************** *****************
Consensus:

CCAGGAGGCGGAACGGGAACAGAcAACGGAACCGGAGGAAAC

>Bacillus_Fam_627_42_2 Nr. of seq. 2 Alignment length(with gaps) = 42
Alignment score = 1.000000
GCF_001375535.1_Bacillus_niameyensis_genomic.fna_6:3545829-3546081
Satlength=253 Nr of Repeats=6 RepeatLength=42 seed=GAAGTCGCGA Num.seqs=6
Similarity=0.836176 0
GAAGTCGCGAAATTCCGGTGGGAAGTTGAAAGATTAACGGGA
GCF_001375535.1_Bacillus_niameyensis_genomic.fna_6:3549055-3549349
Satlength=295 Nr of Repeats=7 RepeatLength=42 seed=GAAGTCGCGA Num.seqs=7
Similarity=0.838686 0
GAAGTCGCGAAATTCCGGTGGGAAGTTGAAAGATTAACGGGA

****
Consensus:

GAAGTCGCGAAATTCCGGTGGGAAGTTGAAAGATTAACGGGA

>Bacillus_Fam_628_41_2 Nr. of seq. 2 Alignment length(with gaps) = 41
Alignment score = 0.617886
GCF_000321185.1_ASM32118v1_genomic.fna_73:13581-13737 Satlength=157 Nr of
Repeats=4 RepeatLength=39 seed=TTCTTTTACT Num.seqs=4 Similarity=0.794872
0 TTCTTTTACTGGAGCTGTTTTG--ACAATTGGCTTTGGCGC
Rev.of_GCF_000380245.2_ASM38024v2_genomic.fna_12:375741-376014
Satlength=274 Nr of Repeats=7 RepeatLength=39 seed=AAAGAAGCAC Num.seqs=7
Similarity=0.744404 6
TTCTTTTACT--ATTGGTTTTGTTACATTTGGCTGNGGTGC

********** * ****** *** ****** ** **
Consensus:

TTCTTTTACTggAgcgGTTTTGttACAaTTGGCTgtGGcGC

>Bacillus_Fam_629_41_2 Nr. of seq. 2 Alignment length(with gaps) = 41
Alignment score = 0.626016
GCF_001648575.1_ASM164857v1_genomic.fna_3:413-553 Satlength=141 Nr of
Repeats=4 RepeatLength=40 seed=GCGGATAAAA Num.seqs=3 Similarity=0.911111
0 GCGGATAAAACGGA-AAAGTCGCGGATATATCGGAAAGTTA
Rev.of_GCF_000612805.1_FF3_genomic.fna_4:298364-298568 Satlength=205 Nr
of Repeats=5 RepeatLength=41 seed=AATATATCAG Num.seqs=4
Similarity=0.967480
32 GCTGATAAATCAAAGAAAGTCGCTGATATATTTTAAATTTC

** ****** * * ******** ******* *** **
Consensus:

GCgGATAAAaCaaAgAAAGTCGCgGATATATcggAAAgTTa

>Bacillus_Fam_630_40_2 Nr. of seq. 2 Alignment length(with gaps) = 40
Alignment score = 0.754167

GCF_001742425.1_ASM174242v1_genomic.fna_9:30829-31159 Satlength=331 Nr of Repeats=9 RepeatLength=39 seed=TGTTCTTCTG Num.seqs=7 Similarity=0.796500
0 TGTTCTTCTGTTGGCTCTGTAGCTTCTACTTCTTCAG-CT
GCF_001742425.1_ASM174242v1_genomic.fna_9:45797-45992 Satlength=196 Nr of Repeats=5 RepeatLength=39 seed=TTCTACTTCT Num.seqs=5 Similarity=0.816667
22 -GTTCATCTGTTGGCTCTGTTATTTCTACTTCTTCTGTCT

**** **************   ************ * **
Consensus:

tGTTCaTCTGTTGGCTCTGTaacTTCTACTTCTTCaGtCT

>Bacillus_Fam_631_39_2 Nr. of seq. 2 Alignment length(with gaps) = 39
Alignment score = 0.897436
GCF_000007825.1_ASM782v1_genomic.fna_1:4838007-4838475 Satlength=469 Nr of Repeats=12 RepeatLength=39 seed=GGTTTCACTT Num.seqs=12 Similarity=0.948718 0
GGTTTCACTTCTGGCTCCTTCGGCTCTTTCGGATCTTCC
GCF_000831065.1_ASM83106v1_genomic.fna_1:4592019-4592604 Satlength=586 Nr of Repeats=15 RepeatLength=39 seed=TTCACTTCTG Num.seqs=15 Similarity=0.890924 3
GGCTTCACTTCTGGTTCCTTCGGTTCTTTCGGATCTTCC

** *********** ******** ***************
Consensus:

GGcTTCACTTCTGGcTCCTTCGGcTCTTTCGGATCTTCC

>Bacillus_Fam_632_39_2 Nr. of seq. 2 Alignment length(with gaps) = 39
Alignment score = 0.897436
GCF_000008505.1_ASM850v1_genomic.fna_1:5094439-5094634 Satlength=196 Nr of Repeats=5 RepeatLength=39 seed=AATCCGGAAC Num.seqs=5 Similarity=0.972650
0 AATCCGGAACAAAATCCAGCAACTAAACCTGCTACTGAC
GCF_001645555.1_ASM164555v1_genomic.fna_13:56878-57034 Satlength=157 Nr of Repeats=4 RepeatLength=39 seed=CTAAACCTGT Num.seqs=4 Similarity=0.914530
22 AATCCGGAACAAAACCCAGCAACTAAACCTGTTACTGAT

************** **************** ******
Consensus:

AATCCGGAACAAAAcCCAGCAACTAAACCTGcTACTGAc

>Bacillus_Fam_633_39_2 Nr. of seq. 2 Alignment length(with gaps) = 39
Alignment score = 0.760684
GCF_000175075.1_ASM17507v1_genomic.fna_32:156308-156500 Satlength=193 Nr of Repeats=5 RepeatLength=39 seed=TGATCGTGCT Num.seqs=4 Similarity=0.886040
0 TGATCGTGCTCTTCCTCATGAGCTTCTTCCTCATGCTCA
Rev.of_GCF_001648575.1_ASM164857v1_genomic.fna_58:140804-140999 Satlength=196 Nr of Repeats=5 RepeatLength=39 seed=CACGAGCATG Num.seqs=5 Similarity=0.835897 2
TGGTCATGGTCGTCGCCATGAGCTTCTTCCTCATGCTCG

** ** ** ** ** *********************
Consensus:

TGaTCaTGcTCgTCccCATGAGCTTCTTCCTCATGCTCa

>Bacillus_Fam_634_39_2 Nr. of seq. 2 Alignment length(with gaps) = 39
Alignment score = 0.641026
GCF_000331575.1_ASM33157v1_genomic.fna_6:36584-36779 Satlength=196 Nr of Repeats=5 RepeatLength=39 seed=ATGACCATGA Num.seqs=5 Similarity=0.856410
0   ATGACCATGATGGAAGCCATAACCAGGGTAGTAACCGCC
Rev.of_GCF_001420715.1_ASM142071v1_genomic.fna_22:732730-732964 Satlength=235 Nr of Repeats=6 RepeatLength=39 seed=TACCATCATG Num.seqs=6 Similarity=0.783476   15
ATGTCCATGATGGTAGCCTTGACCTGGGTNATAGGTNCC

*** ********* **** * *** **** **   **
Consensus:

ATGaCCATGATGGaAGCCaTaACCaGGGTaaTAaccgCC

>Bacillus_Fam_635_39_2 Nr. of seq. 2 Alignment length(with gaps) = 39
Alignment score = 0.794872
GCF_000474275.1_Bmar1.0_genomic.fna_30:49658-49853 Satlength=196 Nr of Repeats=6 RepeatLength=39 seed=CATGCTCGTG Num.seqs=4 Similarity=0.863248
0   CATGCTCGTGATCATGATCTTCCTCATGCCCGTGCTCTT
GCF_002019665.1_ASM201966v1_genomic.fna_1:1072418-1072646 Satlength=229 Nr of Repeats=6 RepeatLength=39 seed=TGTGCGTCCT Num.seqs=5 Similarity=0.958974   26
CATGTTCGTGATCATGATCTTCCTCATGTGCGTCCTCAC

**** ********************** *** ***
Consensus:

CATGcTCGTGATCATGATCTTCCTCATGccCGTcCTCac

>Bacillus_Fam_636_39_2 Nr. of seq. 2 Alignment length(with gaps) = 39
Alignment score = 0.974359
GCF_000775975.1_ASM77597v1_genomic.fna_1:181115-181253 Satlength=139 Nr of Repeats=4 RepeatLength=39 seed=AGAAGAAGTT Num.seqs=3 Similarity=0.836111
0   AGAAGAAGTTGTGGAATTAGAGAAATCTGAGGAAGCGAC
GCF_000832605.1_ASM83260v1_genomic.fna_1:1287745-1288135 Satlength=391 Nr of Repeats=11 RepeatLength=39 seed=AGAAGAAGTT Num.seqs=9 Similarity=0.860185   0
AGAAGAAGTTGTGGAATTAGAGAAANCTGAGGAAGCGAC

************************* *************
Consensus:

AGAAGAAGTTGTGGAATTAGAGAAAtCTGAGGAAGCGAC

>Bacillus_Fam_637_39_2 Nr. of seq. 2 Alignment length(with gaps) = 39
Alignment score = 0.666667

GCF_001654695.1_ASM165469v1_genomic.fna_23:241573-241729 Satlength=157 Nr of Repeats=4 RepeatLength=39 seed=AAGAAGGAAG Num.seqs=4 Similarity=0.982906 0
AAGAAGGAAGAACAAGCACGTTTAGCTGAAGAACAGCGT
GCF_900156875.1_PRJEB18969_genomic.fna_11:570675-570870 Satlength=196 Nr of Repeats=5 RepeatLength=39 seed=AAGCAAGAAG Num.seqs=5 Similarity=0.815385
0 AAGCAAGAAGAAGCAGATCGNTTAGCAGAGGAACAGCGA

*** * ****** ** ** ***** ** ********
Consensus:

AAGaAaGAAGAAcaAGaaCGtTTAGCaGAaGAACAGCGa

>Bacillus_Fam_638_39_2 Nr. of seq. 2 Alignment length(with gaps) = 39 Alignment score = 0.940171
GCF_001877785.1_PlanoSAMM_genomic.fna_96:168466-169318 Satlength=853 Nr of Repeats=16 RepeatLength=39 seed=GCTCCCATCA Num.seqs=14 Similarity=0.844839 0
GCTCCCATCACNCCGGACGGCTGACCCATCGGGGATTGC
GCF_900094975.1_IMG-taxon_2617270721_annotated_assembly_genomic.fna_4:1240438-1241134 Satlength=697 Nr of Repeats=13 RepeatLength=39 seed=GGCTGACCCA Num.seqs=12 Similarity=0.864802 18
GCTCCCATCACTCCGGACGGCTGACCCATCGGGGATTGT

*********** **************************
Consensus:

GCTCCCATCACtCCGGACGGCTGACCCATCGGGGATTGc

>Bacillus_Fam_639_37_2 Nr. of seq. 2 Alignment length(with gaps) = 37 Alignment score = 0.662162
GCF_000242895.2_ASM24289v3_genomic.fna_1:2712624-2712861 Satlength=238 Nr of Repeats=5 RepeatLength=36 seed=TGTCCTGGAT Num.seqs=3 Similarity=0.678063 0
TGTCCTGGATAACCTCCCATGCCACCGTACCCGCCG-
GCF_001591665.1_ASM159166v1_genomic.fna_7:16103-16484 Satlength=382 Nr of Repeats=8 RepeatLength=36 seed=ATCCTCCCAT Num.seqs=6 Similarity=0.823423
9 TG-CCTGGGTATCCTCCCATGCCTCCATAACCACCCA

** ***** ** *********** ** ** ** **
Consensus:

TGtCCTGGaTAaCCTCCCATGCCaCCaTAaCCaCCca

>Bacillus_Fam_640_37_2 Nr. of seq. 2 Alignment length(with gaps) = 37 Alignment score = 0.603604
GCF_000712615.1_ASM71261v1_genomic.fna_8:125406-125946 Satlength=541 Nr of Repeats=15 RepeatLength=36 seed=TACGGGACCA Num.seqs=15 Similarity=0.931532 0
TACGGGACCAACGGGAG-AACCGGGATCTAGCGGAGT
Rev.of_GCF_000715205.1_SOAPdenovo_v1.05_genomic.fna_19:59022-60552 Satlength=1531 Nr of Repeats=42 RepeatLength=36 seed=GCTCCGGTTG

Num.seqs=34 Similarity=0.944808 0
CACGGGAGCCACGGGAGCAACC-GGTCCAACCGGAGC

****** * ******* **** ** * * *****
Consensus:

cACGGGAcCaACGGGAGcAACCgGGacCaAcCGGAGc

>Bacillus_Fam_641_37_2 Nr. of seq. 2 Alignment length(with gaps) = 37
Alignment score = 0.747748
GCF_001578185.1_ASM157818v1_genomic.fna_1:1822790-1822970 Satlength=181
Nr of Repeats=5 RepeatLength=36 seed=TGCCACTGTT Num.seqs=5
Similarity=0.722522 0
TGCCACTGTTGCCAA-GTANCGTGAGGATGGTGCTGA
GCF_000025825.1_ASM2582v1_genomic.fna_1:3261851-3262067 Satlength=217 Nr
of Repeats=5 RepeatLength=36 seed=TGCCATTGAT Num.seqs=4
Similarity=0.594595
0 TGCCACTGATGCCAAGGTACNGTATGGATGCTGCTGA

******** ****** *** ** ***** ******
Consensus:

TGCCACTGaTGCCAAgGTAccGTaaGGATGcTGCTGA

>Bacillus_Fam_642_36_2 Nr. of seq. 2 Alignment length(with gaps) = 36
Alignment score = 0.666667
GCF_000007825.1_ASM782v1_genomic.fna_1:2324878-2325328 Satlength=451 Nr
of Repeats=12 RepeatLength=36 seed=ACCGGAGCAA Num.seqs=9
Similarity=0.836420
0 ACCGGAGCAACAGGCCCGACTGGAATAACGGGCCCA
Rev.of_GCF_001038845.1_ASM103884v1_genomic.fna_46:6167-6671 Satlength=505
Nr of Repeats=11 RepeatLength=36 seed=CCCGTGGCTC Num.seqs=8
Similarity=0.851852 14
ACGGGAGCCACGGGTTCAACGGGAGTAACTGGCCCA

** ***** ** ** * ** *** **** ******
Consensus:

ACcGGAGCaACaGGccCaACgGGAaTAACgGGCCCA

>Bacillus_Fam_643_36_2 Nr. of seq. 2 Alignment length(with gaps) = 36
Alignment score = 0.898148
GCF_000008425.1_ASM842v1_genomic.fna_1:1177079-1177205 Satlength=127 Nr
of Repeats=4 RepeatLength=36 seed=AAAAGCAAGA Num.seqs=3
Similarity=0.777778
0 AAAAGCAAGAAACCCGATGACTGCAAAAAGCCTGAC
GCF_000408885.1_ASM40888v1_genomic.fna_1:1277423-1277564 Satlength=142 Nr
of Repeats=5 RepeatLength=36 seed=AAAAGCAAGA Num.seqs=3
Similarity=0.690058 0
AAAAGCAAGAAACCTGATGACTGCAAAAAACCNGAC

************* ************* ** ***
Consensus:

AAAAGCAAGAAACCcGATGACTGCAAAAAaCCtGAC

>Bacillus_Fam_644_36_2 Nr. of seq. 2 Alignment length(with gaps) = 36
Alignment score = 0.925926
GCF_000025805.1_ASM2580v1_genomic.fna_1:706167-706311 Satlength=145 Nr of Repeats=5 RepeatLength=36 seed=GAAATTAAGC Num.seqs=3 Similarity=0.861862
0   GAAATTAAGCAGGAAAAAGATATAGGAATTAAAAAG
GCF_000025825.1_ASM2582v1_genomic.fna_1:700384-700528 Satlength=145 Nr of Repeats=4 RepeatLength=36 seed=CAGGAAAAAG Num.seqs=4 Similarity=0.938272
9   GAAATTAAACAGGAAAAAGATATAGGAATTAAGAAG

******** ********************** ***
Consensus:

GAAATTAAaCAGGAAAAAGATATAGGAATTAAaAAG

>Bacillus_Fam_645_36_2 Nr. of seq. 2 Alignment length(with gaps) = 36
Alignment score = 0.925926
GCF_000025805.1_ASM2580v1_genomic.fna_1:2966155-2966335 Satlength=181 Nr of Repeats=5 RepeatLength=36 seed=TCTTTTTGAG Num.seqs=5
Similarity=0.970370
0   TCTTTTTGAGCTTGTTCAGCTTGAGCCTGCTGCTGC
GCF_000025825.1_ASM2582v1_genomic.fna_1:2943369-2943663 Satlength=295 Nr of Repeats=8 RepeatLength=36 seed=GCTTGAGCTT Num.seqs=7
Similarity=0.978836
18   TCTTTTTGAGCCTGTTCAGCTTGAGCTTGCTGCTGC

*********** ************** *********
Consensus:

TCTTTTTGAGCcTGTTCAGCTTGAGCcTGCTGCTGC

>Bacillus_Fam_646_36_2 Nr. of seq. 2 Alignment length(with gaps) = 36
Alignment score = 1.000000
GCF_000153365.1_ASM15336v1_genomic.fna_18:445614-445902 Satlength=289 Nr of Repeats=7 RepeatLength=36 seed=GGTCCTTGAA Num.seqs=6
Similarity=0.790390
0   GGTCCTTGAACCCCTTGTGTGCCTTGATCTCCTTGC
Rev.of_GCF_000473245.1_ASM47324v1_genomic.fna_1:507480-507768
Satlength=289 Nr of Repeats=7 RepeatLength=36 seed=TTCAAGGACC Num.seqs=6
Similarity=0.790390   10
GGTCCTTGAACCCCTTGTGTGCCTTGATCTCCTTGC

****
Consensus:

GGTCCTTGAACCCCTTGTGTGCCTTGATCTCCTTGC

>Bacillus_Fam_647_36_2 Nr. of seq. 2 Alignment length(with gaps) = 36
Alignment score = 0.944444
GCF_000153365.1_ASM15336v1_genomic.fna_74:62534-62903 Satlength=370 Nr of Repeats=10 RepeatLength=36 seed=CAAGGATCAA Num.seqs=9

Similarity=0.802469
0   CAAGGATCAACTGGNGCNCAGGGATCCCAAGGACCG
GCF_000473245.1_ASM47324v1_genomic.fna_1:2119185-2119554 Satlength=370 Nr of Repeats=10 RepeatLength=36 seed=CAAGGATCAA Num.seqs=9 Similarity=0.802469 0
CAAGGATCAACTGGNGCNCAGGGATCCCAAGGACCG

****
Consensus:

CAAGGATCAACTGGNGCNCAGGGATCCCAAGGACCG

>Bacillus_Fam_648_36_2 Nr. of seq. 2 Alignment length(with gaps) = 36
Alignment score = 0.666667
GCF_000161455.1_ASM16145v1_genomic.fna_1:2079145-2079325 Satlength=181 Nr of Repeats=5 RepeatLength=36 seed=GGGAATACAA Num.seqs=5 Similarity=0.800000 0
GGGAATACAACTCCGCCAGGCAGTGGAAATGGCCAA
Rev.of_GCF_000832605.1_ASM83260v1_genomic.fna_1:3388209-3388497 Satlength=289 Nr of Repeats=8 RepeatLength=36 seed=TGTATTTCCT Num.seqs=8 Similarity=0.866402 9
GGAAATACAACGCCGCCAAACAATGGTAGAGGCGAA

** ******** ****** ** *** * *** **
Consensus:

GGaAATACAACgCCGCCAaaCAaTGGaAaaGGCcAA

>Bacillus_Fam_649_36_2 Nr. of seq. 2 Alignment length(with gaps) = 36
Alignment score = 0.962963
GCF_000217835.1_ASM21783v1_genomic.fna_1:1229797-1230013 Satlength=217 Nr of Repeats=5 RepeatLength=36 seed=GCAACAACCG Num.seqs=4 Similarity=0.919753 0
GCAACAACCGGAATAACGGGAACGGCCAGGCCGCAA
GCF_000832905.1_ASM83290v1_genomic.fna_1:203662-203842 Satlength=181 Nr of Repeats=5 RepeatLength=36 seed=GCAACAACCG Num.seqs=5 Similarity=0.859259
0   GCAACAACCGGAATAACGGAAACGGCCAGGCCGCAA

******************* ****************
Consensus:

GCAACAACCGGAATAACGGaAACGGCCAGGCCGCAA

>Bacillus_Fam_650_36_2 Nr. of seq. 2 Alignment length(with gaps) = 36
Alignment score = 0.703704
GCF_000751775.1_A1A_genomic.fna_79:77245-77425 Satlength=181 Nr of Repeats=5 RepeatLength=36 seed=CATTGATTGC Num.seqs=5 Similarity=0.855556
0   CATTGATTGCCGTGCCACCATTCGGGCCTTGACCTC
GCF_001420715.1_ASM142071v1_genomic.fna_23:27567-27783 Satlength=217 Nr of Repeats=4 RepeatLength=36 seed=CCTCCTTGGG Num.seqs=3 Similarity=0.901235
32   CTTGGGCTGCAGTTCCACCATTCGGACCTTGGCCTC

* * * *** ** *********** ***** ****
Consensus:

CaTgGacTGCaGTgCCACCATTCGGaCCTTGaCCTC

>Bacillus_Fam_651_36_2 Nr. of seq. 2 Alignment length(with gaps) = 36
Alignment score = 0.629630
GCF_000769555.1_ASM76955v1_genomic.fna_1:1273558-1274494 Satlength=937 Nr of Repeats=26 RepeatLength=36 seed=CGCCAGTAGC Num.seqs=26 Similarity=0.918405 0
CGCCAGTAGCGCCGGTAAATCCGGTCGGTCCGGTAA
Rev.of_GCF_001578205.1_ASM157820v1_genomic.fna_1:2833063-2833531 Satlength=469 Nr of Repeats=15 RepeatLength=36 seed=CTACTGGCGC Num.seqs=11 Similarity=0.981145
9   CGCCAGTAGCCCCTGAAGGACCAGTAGGACCGGTAG

********** ** * *   ** ** ** ******
Consensus:

CGCCAGTAGCcCCgGaAaaaCCaGTaGGaCCGGTAa

>Bacillus_Fam_652_36_2 Nr. of seq. 2 Alignment length(with gaps) = 36
Alignment score = 0.666667
GCF_001590785.1_ASM159078v1_genomic.fna_10:48242-48455 Satlength=214 Nr of Repeats=6 RepeatLength=36 seed=GGACCGACAG Num.seqs=4 Similarity=0.864198
0   GGACCGACAGGAGCAACAGGAATAGCAGGAATCACC
GCF_002019765.1_ASM201976v1_genomic.fna_1:2106260-2106764 Satlength=505 Nr of Repeats=10 RepeatLength=36 seed=TTAGACGGTG Num.seqs=6 Similarity=0.883951 21
GGTGCTACAGGAGCAACAGGATTAGACGGTGTCACT

** * *************** *** ** ****
Consensus:

GGacCgACAGGAGCAACAGGAaTAGaaGGaaTCACc

>Bacillus_Fam_653_36_2 Nr. of seq. 2 Alignment length(with gaps) = 36
Alignment score = 0.824074
GCF_001887185.1_ASM188718v1_genomic.fna_215:57616-57874 Satlength=259 Nr of Repeats=7 RepeatLength=36 seed=AGTACAGCAA Num.seqs=5 Similarity=0.718018
0   AGTACAGCAAACGGAAGAAATGCAGCCGAGAGTANC
Rev.of_GCF_002009555.1_ASM200955v1_genomic.fna_223:6085-6232 Satlength=148 Nr of Repeats=4 RepeatLength=36 seed=TTGCTGTACT Num.seqs=3 Similarity=0.728395 10
AGTACAGCAAGTGGAAGAAGAGCAGCCGAGAGTACC

********** ******* ************* *
Consensus:

AGTACAGCAAacGGAAGAAaaGCAGCCGAGAGTAcC

>Bacillus_Fam_654_34_2 Nr. of seq. 2 Alignment length(with gaps) = 34
Alignment score = 1.000000
GCF_000153365.1_ASM15336v1_genomic.fna_18:81509-81679 Satlength=171 Nr of Repeats=5 RepeatLength=34 seed=TCTGTCCCCT Num.seqs=5 Similarity=0.890196
0   TCTGTCCCCTTGGCCCAGTTTTCGGGCCAGGGGG
Rev.of_GCF_000473245.1_ASM47324v1_genomic.fna_1:870777-870947 Satlength=171 Nr of Repeats=5 RepeatLength=34 seed=AAGGGGACAG Num.seqs=5 Similarity=0.890196   11
TCTGTCCCCTTGGCCCAGTTTTCGGGCCAGGGGG

****
Consensus:

TCTGTCCCCTTGGCCCAGTTTTCGGGCCAGGGGG

>Bacillus_Fam_655_33_2 Nr. of seq. 2 Alignment length(with gaps) = 33
Alignment score = 1.000000
GCF_001243895.1_Bacillus_testis_genomic.fna_9:552036-552300 Satlength=265 Nr of Repeats=8 RepeatLength=33 seed=CGGCGGGAAT Num.seqs=8 Similarity=0.874459   0
CGGCGGGAATGCCGGAATGCCAGGTCAAGGAAG
GCF_001243895.1_Bacillus_testis_genomic.fna_9:552088-552451 Satlength=364 Nr of Repeats=11 RepeatLength=33 seed=CCAGGCCAAG Num.seqs=11 Similarity=0.841322   19
CGGCGGGAATGCCGGAATGCCAGGTCAAGGAAG

****
Consensus:

CGGCGGGAATGCCGGAATGCCAGGTCAAGGAAG

>Bacillus_Fam_656_30_2 Nr. of seq. 2 Alignment length(with gaps) = 30
Alignment score = 1.000000
GCF_000759675.1_ASM75967v1_genomic.fna_60:16-166 Satlength=151 Nr of Repeats=5 RepeatLength=30 seed=AAAATGAAAG Num.seqs=5 Similarity=0.902222
0   AAAATGAAAGAAACTCCAAATGTAGTACCG
Rev.of_GCF_000759675.1_ASM75967v1_genomic.fna_80:1917-2127 Satlength=211 Nr of Repeats=7 RepeatLength=30 seed=TTCTTTCATT Num.seqs=7 Similarity=0.809524   12
AAAATGAAAGAAACTCCAAATGTAGTACCG

****
Consensus:

AAAATGAAAGAAACTCCAAATGTAGTACCG

>Bacillus_Fam_657_28_2 Nr. of seq. 2 Alignment length(with gaps) = 28
Alignment score = 0.601190
GCF_000712615.1_ASM71261v1_genomic.fna_14:9252-9900 Satlength=649 Nr of Repeats=24 RepeatLength=27 seed=AGCGAAGCAA Num.seqs=24 Similarity=0.973341
0   AGCGAAGCAAAAGCAACAAGAAGAACA-
Rev.of_GCF_001243895.1_Bacillus_testis_genomic.fna_9:2243301-2243598 Satlength=298 Nr of Repeats=11 RepeatLength=27 seed=CGTTGTTGCT

Num.seqs=11 Similarity=0.910213 2
CGCCAAGCTCAAGC-ACAAGAGCAACAA

** **** **** ****** ****
Consensus:

aGCcAAGCaaAAGCaACAAGAacAACAa

>Bacillus_Fam_658_27_2 Nr. of seq. 2 Alignment length(with gaps) = 27
Alignment score = 0.697531
GCF_001877785.1_PlanoSAMM_genomic.fna_139:52829-52954 Satlength=126 Nr of Repeats=5 RepeatLength=25 seed=TTGTACCTGG Num.seqs=5 Similarity=0.537179
0 TTGTACCTGGTACATNG-GNG-TNGAT
GCF_001877785.1_PlanoSAMM_genomic.fna_139:112839-112939 Satlength=101 Nr of Repeats=4 RepeatLength=25 seed=TGTACCTGGT Num.seqs=4 Similarity=0.594246
0 -TGTACCTGGTACATGGAGCGTTTGAT

************** * * * * ***
Consensus:

tTGTACCTGGTACATgGaGcGtTtGAT

>Bacillus_Fam_659_27_2 Nr. of seq. 2 Alignment length(with gaps) = 27
Alignment score = 0.654321
GCF_000831065.1_ASM83106v1_genomic.fna_1:4229089-4229503 Satlength=415 Nr of Repeats=14 RepeatLength=27 seed=GCTCCCGTTG Num.seqs=9 Similarity=0.888889 12
GTTGAACCTGTCGCTCCCGTTGGACCT
GCF_001712755.1_ASM171275v1_genomic.fna_23:0-188 Satlength=189 Nr of Repeats=7 RepeatLength=27 seed=GCTGTTCCAG Num.seqs=7 Similarity=0.971781
12 CCTGTCGCTCCAGCTGTTCCAGTTGCT

** *** * ** **
Consensus:

cCTccaGCTcccccaGgacCTccTGaa

>Bacillus_Fam_660_24_2 Nr. of seq. 2 Alignment length(with gaps) = 24
Alignment score = 0.631944
GCF_001591665.1_ASM159166v1_genomic.fna_19:22435-22519 Satlength=85 Nr of Repeats=4 RepeatLength=21 seed=GGACAGTAAA Num.seqs=4 Similarity=0.661376
0 GGACA-GTAAAA-CANAAAAAGT-
Rev.of_GCF_000496285.1_ASM49628v1_genomic.fna_1:1628788-1628908 Satlength=121 Nr of Repeats=4 RepeatLength=24 seed=ACTTCTTTTT Num.seqs=3 Similarity=0.814815 2
GTACAGGTAAAACCACAAAAAGAA

* *** ****** ** ******
Consensus:

GgACAgGTAAAAcCAcAAAAAGaa

>Bacillus_Fam_661_24_2 Nr. of seq. 2 Alignment length(with gaps) = 24 Alignment score = 0.833333
GCF_000005825.2_ASM582v2_genomic.fna_1:1602362-1602482 Satlength=121 Nr of Repeats=5 RepeatLength=24 seed=ATGGCCGGAG Num.seqs=5 Similarity=0.911111
0 ATGGCCGGAGCGAACATGGGGCCA
GCF_000474275.1_Bmar1.0_genomic.fna_8:180773-180917 Satlength=145 Nr of Repeats=6 RepeatLength=24 seed=ATGGCAGGAG Num.seqs=6 Similarity=0.907407
0 ATGGCAGGAGCGAACATGGCGCCG

***** ************ ***
Consensus:

ATGGCaGGAGCGAACATGGcGCCa

>Bacillus_Fam_662_24_2 Nr. of seq. 2 Alignment length(with gaps) = 24 Alignment score = 0.944444
GCF_000008505.1_ASM850v1_genomic.fna_1:1072242-1072386 Satlength=145 Nr of Repeats=6 RepeatLength=24 seed=CAGAAAAGAA Num.seqs=4 Similarity=0.632222
0 CAGAAAAGAATCAAAAAACAGAGA
GCF_000831065.1_ASM83106v1_genomic.fna_1:895901-896096 Satlength=196 Nr of Repeats=5 RepeatLength=24 seed=AGAAAAAAAT Num.seqs=3 Similarity=0.740741
1 CAGAAAAGAATCAAAAAGCAGAGA

***************** ******
Consensus:

CAGAAAAGAATCAAAAAaCAGAGA

>Bacillus_Fam_663_23_2 Nr. of seq. 2 Alignment length(with gaps) = 23 Alignment score = 0.666667
GCF_001439915.1_ASM143991v1_genomic.fna_122:1-121 Satlength=121 Nr of Repeats=6 RepeatLength=20 seed=TATTTGCGTC Num.seqs=6 Similarity=0.590821
0 TATTTGCGTCGGTCCCA--C-AT
Rev.of_GCF_001439915.1_ASM143991v1_genomic.fna_83:14858-15120 Satlength=263 Nr of Repeats=11 RepeatLength=20 seed=CGACGCAAAT Num.seqs=8 Similarity=0.573499
11 TATTTGCGTCGNTTCCANACNAT

*********** * *** * **
Consensus:

TATTTGCGTCGgTcCCAnaCnAT

>Bacillus_Fam_664_23_2 Nr. of seq. 2 Alignment length(with gaps) = 23 Alignment score = 0.717391
GCF_900094975.1_IMG-taxon_2617270721_annotated_assembly_genomic.fna_4:1334318-1334418 Satlength=101 Nr of Repeats=5 RepeatLength=20 seed=ATTAGGATCG Num.seqs=5 Similarity=0.725397 0 ATTAGGATCGT--ATTTCTG-GA
Rev.of_GCF_900094975.1_IMG-taxon_2617270721_annotated_assembly_genomic.fna_4:483549-483629

Satlength=81 Nr of Repeats=4 RepeatLength=20 seed=TACGATCCTA Num.seqs=4 Similarity=0.636364 11 -TTAGGATCGTAAATTTCTGAGA

********** ******* **
Consensus:

aTTAGGATCGTaaATTTCTGaGA

>Bacillus_Fam_665_23_2 Nr. of seq. 2 Alignment length(with gaps) = 23 Alignment score = 0.615942
GCF_001636315.1_ASM163631v1_genomic.fna_1:1446486-1446570 Satlength=85 Nr of Repeats=4 RepeatLength=21 seed=CCAACTGGCG Num.seqs=4 Similarity=0.862434 0 CCAACTGGCGGAT-A-CACGCTC
Rev.of_GCF_000612665.1_FF4_genomic.fna_75:18910-19034 Satlength=125 Nr of Repeats=5 RepeatLength=21 seed=ATCCGCTAGT Num.seqs=4 Similarity=0.576389 12 -CAACTAGCGGATAACCNCGATC

***** ****** * * ** **
Consensus:

cCAACTaGCGGATaAcCaCGaTC

>Bacillus_Fam_666_23_2 Nr. of seq. 2 Alignment length(with gaps) = 23 Alignment score = 0.760870
GCF_001648575.1_ASM164857v1_genomic.fna_3:346105-346468 Satlength=364 Nr of Repeats=10 RepeatLength=21 seed=TTTATTTTCC Num.seqs=9 Similarity=0.555135 0 TTTATTTTCCATTNCAGCCG-A-
GCF_001648575.1_ASM164857v1_genomic.fna_3:354189-354315 Satlength=127 Nr of Repeats=6 RepeatLength=21 seed=TTTATTTTCC Num.seqs=6 Similarity=0.461111
0 TTTATTTTCCATTCAAGCCGNAT

************* ***** *
Consensus:

TTTATTTTCCATTcaAGCCGnAt

>Bacillus_Fam_667_23_2 Nr. of seq. 2 Alignment length(with gaps) = 23 Alignment score = 0.666667
GCF_000934845.1_ASM93484v1_genomic.fna_5:25497-25581 Satlength=85 Nr of Repeats=4 RepeatLength=21 seed=ATTCCGCGAA Num.seqs=4 Similarity=0.468750
0 ATTCCGCGAAANAACC-AGAATA
GCF_001315165.1_ASM131516v1_genomic.fna_10:42593-42718 Satlength=126 Nr of Repeats=5 RepeatLength=21 seed=ATTCCGCGAA Num.seqs=4 Similarity=0.471014
0 ATTCCGCGAAA-AACNAACGATA

*********** *** * ***
Consensus:

ATTCCGCGAAAnAACcaAcaATA

>Bacillus_Fam_668_22_2 Nr. of seq. 2 Alignment length(with gaps) = 22
Alignment score = 0.772727
GCF_000007845.1_ASM784v1_genomic.fna_1:4827415-4827495 Satlength=81 Nr of Repeats=4 RepeatLength=20 seed=TATATCAGCG Num.seqs=4 Similarity=0.593915
14 AGCGAAACTCCAAA--TATATC
GCF_000831065.1_ASM83106v1_genomic.fna_1:4391098-4391298 Satlength=201 Nr of Repeats=9 RepeatLength=20 seed=TTATATCAGC Num.seqs=8 Similarity=0.688312 14
AGCGCAACTCCCAATTTATATC

********** ****** **
Consensus:

tTATATCAGCGaAACTCCaAAt

>Bacillus_Fam_669_22_2 Nr. of seq. 2 Alignment length(with gaps) = 22
Alignment score = 0.621212
GCF_000311725.1_ASM31172v1_genomic.fna_3:434751-434853 Satlength=103 Nr of Repeats=5 RepeatLength=20 seed=ATTCCGGAAC Num.seqs=3
Similarity=0.814815
0 -ATTCCGGAACTTTT-GGATTA
GCF_000311725.1_ASM31172v1_genomic.fna_2:435289-435392 Satlength=104 Nr of Repeats=5 RepeatLength=21 seed=AATTCCGGAA Num.seqs=3
Similarity=0.830688
0 AATTCCGTAACTTTTCGTGTT-

****** ******* * **
Consensus:

aATTCCGgAACTTTTcGgaTTa

>Bacillus_Fam_670_22_2 Nr. of seq. 2 Alignment length(with gaps) = 22
Alignment score = 0.643939
GCF_000331575.1_ASM33157v1_genomic.fna_3:82032-82150 Satlength=119 Nr of Repeats=6 RepeatLength=20 seed=CTTACATATA Num.seqs=4 Similarity=1.000000
0 CTTAC-AT-ATATTTTTGGGTC
Rev.of_GCF_900156875.1_PRJEB18969_genomic.fna_9:2524-2673 Satlength=150 Nr of Repeats=7 RepeatLength=21 seed=GTTATAGTTA Num.seqs=6
Similarity=0.932275 10
-TAACTATAACATTTTTGGGTC

* ** ** * ***********
Consensus:

cTaACtATaAcATTTTTGGGTC

>Bacillus_Fam_671_22_2 Nr. of seq. 2 Alignment length(with gaps) = 22
Alignment score = 0.628788
GCF_000245315.1_ASM24531v1_genomic.fna_68:3612-3750 Satlength=139 Nr of Repeats=7 RepeatLength=21 seed=AAAAAGTATT Num.seqs=6 Similarity=0.805291
0 -AAAAAGCATTATGATCATGAC
Rev.of_GCF_001375535.1_Bacillus_niameyensis_genomic.fna_6:2731414-2731540 Satlength=127 Nr of Repeats=4 RepeatLength=21 seed=TTTTCTCTTC Num.seqs=3

Similarity=0.724868 4 AAAAAAGCATTA-GNTGAAGAG

*********** * * * **
Consensus:

aAAAAAGCATTAtGaTcAaGAc

>Bacillus_Fam_672_22_2 Nr. of seq. 2 Alignment length(with gaps) = 22
Alignment score = 0.628788
GCF_000305495.1_BacPsy2.0_genomic.fna_37:40246-40493 Satlength=248 Nr of Repeats=6 RepeatLength=21 seed=TATTCCCGTC Num.seqs=5 Similarity=0.513636
0 TATTCCCGTCTGCGNANGNTG-
Rev.of_GCF_000305495.1_BacPsy2.0_genomic.fna_243:5857-5941 Satlength=85 Nr of Repeats=4 RepeatLength=21 seed=AGACGGGAAT Num.seqs=4 Similarity=0.520202 11
TATTCCCGTCTGAGATGGAAGT

************ * * *
Consensus:

TATTCCCGTCTGaGaagGaaGt

>Bacillus_Fam_673_22_2 Nr. of seq. 2 Alignment length(with gaps) = 22
Alignment score = 0.681818
GCF_000305495.1_BacPsy2.0_genomic.fna_66:16974-17058 Satlength=85 Nr of Repeats=4 RepeatLength=21 seed=TTATTCCCGT Num.seqs=4 Similarity=0.613636
0 TTATTCCCGTCTC-GAATAAGC
GCF_000305495.1_BacPsy2.0_genomic.fna_259:12228-12312 Satlength=85 Nr of Repeats=4 RepeatLength=21 seed=TATTCCCGTC Num.seqs=4 Similarity=0.427083
1 TTATTCCCGTCTCATCATGNGC

************* ** **
Consensus:

TTATTCCCGTCTCagaATaaGC

>Bacillus_Fam_674_22_2 Nr. of seq. 2 Alignment length(with gaps) = 22
Alignment score = 0.696970
GCF_000307875.1_BABA1.0_genomic.fna_7:26272-26417 Satlength=146 Nr of Repeats=7 RepeatLength=21 seed=TGCGACAGTA Num.seqs=6 Similarity=0.658586
0 TGCGACAGTAAAGCGTGGC-TA
GCF_000307875.1_BABA1.0_genomic.fna_58:51271-51373 Satlength=103 Nr of Repeats=5 RepeatLength=21 seed=TCCGACAGTA Num.seqs=4 Similarity=0.584541
0 TCCGACAGTAACG-GTGGCTTA

* ********* * ***** **
Consensus:

TcCGACAGTAAaGcGTGGCtTA

>Bacillus_Fam_675_22_2 Nr. of seq. 2 Alignment length(with gaps) = 22
Alignment score = 0.674242

GCF_000311725.1_ASM31172v1_genomic.fna_4:432525-432628 Satlength=104 Nr of Repeats=5 RepeatLength=21 seed=TAATTCCGGA Num.seqs=3 Similarity=0.724868
0   TAATTCCGGAACTTTNACCCA-
GCF_000311725.1_ASM31172v1_genomic.fna_1:120906-121010 Satlength=105 Nr of Repeats=5 RepeatLength=21 seed=AATTCCGGAA Num.seqs=4 Similarity=0.704545
1   NAATTCCGGAACTTTCGAGCAT

**************   **
Consensus:

tAATTCCGGAACTTTcaacCAt

>Bacillus_Fam_676_22_2 Nr. of seq. 2 Alignment length(with gaps) = 22 Alignment score = 0.643939
GCF_000331575.1_ASM33157v1_genomic.fna_1:755726-755838 Satlength=113 Nr of Repeats=4 RepeatLength=21 seed=CTTTATTGTC Num.seqs=3 Similarity=0.601852
0   CTTTATTGTCCGGAATTGC-CN
Rev.of_GCF_000986785.1_ASM98678v1_genomic.fna_10:73543-73915 Satlength=373 Nr of Repeats=11 RepeatLength=21 seed=GACAATAGAA Num.seqs=7 Similarity=0.621212
9   -TTTATTGTCCNGAAACGCTCT

********** *** ** *
Consensus:

cTTTATTGTCCgGAAacGCtCt

>Bacillus_Fam_677_22_2 Nr. of seq. 2 Alignment length(with gaps) = 22 Alignment score = 0.810606
GCF_000374345.1_ASM37434v1_genomic.fna_22:48552-48902 Satlength=351 Nr of Repeats=13 RepeatLength=21 seed=TTCATTCCAA Num.seqs=9 Similarity=0.640572
0   TTCATTCCAAAA-TGGGNTTTT
GCF_000374345.1_ASM37434v1_genomic.fna_31:31-340 Satlength=310 Nr of Repeats=11 RepeatLength=21 seed=TTCATTCCAA Num.seqs=7 Similarity=0.619048
0   TTCATTCCAAAACTGGGNTTT-

************ ********
Consensus:

TTCATTCCAAAAcTGGGNTTTt

>Bacillus_Fam_678_22_2 Nr. of seq. 2 Alignment length(with gaps) = 22 Alignment score = 0.810606
GCF_000429705.1_ASM42970v1_genomic.fna_1:195033-195200 Satlength=168 Nr of Repeats=7 RepeatLength=21 seed=CTAACTCGAC Num.seqs=5 Similarity=0.694444
0   CTAACTCGACTAAAGG-NGAGG
GCF_000429705.1_ASM42970v1_genomic.fna_1:361409-361556 Satlength=148 Nr of Repeats=7 RepeatLength=21 seed=GCTAACTCGA Num.seqs=7

Similarity=0.863636
20   CTAACTCGACTAAAGGCGGAG-

**************** ***
Consensus:

CTAACTCGACTAAAGGcgGAGg

>Bacillus_Fam_679_22_2 Nr. of seq. 2 Alignment length(with gaps) = 22
Alignment score = 0.666667
GCF_000429705.1_ASM42970v1_genomic.fna_1:2283624-2283729 Satlength=106 Nr of Repeats=4 RepeatLength=21 seed=TTTAAACAGG Num.seqs=3
Similarity=0.525253 0
TTTAAACAGGTTNGCACG-NCG
Rev.of_GCF_000429705.1_ASM42970v1_genomic.fna_6:55774-55879 Satlength=106 Nr of Repeats=4 RepeatLength=21 seed=AACCTGTTTA Num.seqs=3
Similarity=0.830688 12
TTTAAACAGGTTGG-AGGCGCG

************ * * * **
Consensus:

TTTAAACAGGTTgGcAcGcgCG

>Bacillus_Fam_680_22_2 Nr. of seq. 2 Alignment length(with gaps) = 22
Alignment score = 0.674242
GCF_000430785.1_ASM43078v1_genomic.fna_6:52001-52085 Satlength=85 Nr of Repeats=4 RepeatLength=21 seed=TTTACTATCC Num.seqs=4 Similarity=0.664141
0   TTTACTATCCAAATTGAACCA-
GCF_001315085.1_ASM131508v1_genomic.fna_12:91450-91534 Satlength=85 Nr of Repeats=4 RepeatLength=21 seed=TTTATGATCC Num.seqs=4 Similarity=0.809524
0   TTTATGATCCAACTT-AACCAA

**** ****** ** *****
Consensus:

TTTAcgATCCAAaTTgAACCAa

>Bacillus_Fam_681_22_2 Nr. of seq. 2 Alignment length(with gaps) = 22
Alignment score = 0.848485
GCF_000473245.1_ASM47324v1_genomic.fna_1:4442972-4443056 Satlength=85 Nr of Repeats=4 RepeatLength=21 seed=GGAATGATTA Num.seqs=4
Similarity=0.643939
0   GGAATGATTATT-GGCGAATCC
Rev.of_GCF_000153365.1_ASM15336v1_genomic.fna_17:84758-84842 Satlength=85 Nr of Repeats=4 RepeatLength=21 seed=ATAATCATTC Num.seqs=4
Similarity=0.644928 11
GGAATGATTATTCGCCGAATCC

************ * *******
Consensus:

GGAATGATTATTcGcCGAATCC

>Bacillus_Fam_682_22_2 Nr. of seq. 2 Alignment length(with gaps) = 22
Alignment score = 0.628788
GCF_000496285.1_ASM49628v1_genomic.fna_1:367662-367830 Satlength=169 Nr of Repeats=7 RepeatLength=21 seed=ACTTCTCCTT Num.seqs=6 Similarity=0.923810
0 ACTTCTCCTTTTGGATCTGTC-
Rev.of_GCF_000007845.1_ASM784v1_genomic.fna_1:554769-555177 Satlength=409 Nr of Repeats=15 RepeatLength=21 seed=AGAAGTTGAA Num.seqs=10 Similarity=0.668182 6
NCTTCTNTCTTTGGNTCTTTCA

*****   ***** *** **
Consensus:

aCTTCTcccTTTGGaTCTgTCa

>Bacillus_Fam_683_22_2 Nr. of seq. 2 Alignment length(with gaps) = 22
Alignment score = 0.727273
GCF_000612625.1_JCE_genomic.fna_3:36022-36122 Satlength=101 Nr of Repeats=5 RepeatLength=21 seed=ATTCTACAAA Num.seqs=3 Similarity=0.661376
0 ATTCTACAAAANT-AAAGANGA
Rev.of_GCF_000612625.1_JCE_genomic.fna_58:7998-8081 Satlength=84 Nr of Repeats=4 RepeatLength=21 seed=TTTGTAGAAT Num.seqs=3 Similarity=0.525253
10 ATTCTACAAAAATCAAAG-GGA

*********** * **** **
Consensus:

ATTCTACAAAAaTcAAAGagGA

>Bacillus_Fam_684_22_2 Nr. of seq. 2 Alignment length(with gaps) = 22
Alignment score = 0.613636
GCF_000752035.1_Bacillus_rubiinfantis_genomic.fna_11:630800-630905 Satlength=106 Nr of Repeats=4 RepeatLength=21 seed=GTTACTCCCG Num.seqs=3 Similarity=0.767677 0
GTTACTCCCGCATAG-CTGCTC
GCF_000752035.1_Bacillus_rubiinfantis_genomic.fna_15:11202-11572 Satlength=371 Nr of Repeats=16 RepeatLength=21 seed=TTACTCCCGT Num.seqs=10 Similarity=0.802469
1 GTTACTCCCGTATCGACTCCA-

********** ** * ** *
Consensus:

GTTACTCCCGcATaGaCTcCac

>Bacillus_Fam_685_22_2 Nr. of seq. 2 Alignment length(with gaps) = 22
Alignment score = 0.727273
GCF_000986785.1_ASM98678v1_genomic.fna_5:144649-145040 Satlength=392 Nr of Repeats=15 RepeatLength=21 seed=GCAAATAGAA Num.seqs=11 Similarity=0.592496 12
ANTNGG-AAANCGGCAAATAGA
GCF_000986785.1_ASM98678v1_genomic.fna_4:84896-85225 Satlength=330 Nr of Repeats=12 RepeatLength=21 seed=GCAAATAGAA Num.seqs=8

Similarity=0.590278
12   AATGNGAAAATCGGCAAATAGA

********** * * *** **
Consensus:

GCAAATAGAAaTggGaAAAtCG

>Bacillus_Fam_686_22_2 Nr. of seq. 2 Alignment length(with gaps) = 22
Alignment score = 0.674242
GCF_001273755.1_ASM127375v1_genomic.fna_17:4476963-4477083 Satlength=121
Nr of Repeats=5 RepeatLength=21 seed=GATAATTCGA Num.seqs=3
Similarity=0.767677   0
GATAATTCGACCTTCATGAGC-
Rev.of_GCF_001645685.2_ASM164568v2_genomic.fna_1:3706271-3706705
Satlength=435 Nr of Repeats=10 RepeatLength=21 seed=CGGTCGAATT
Num.seqs=6 Similarity=0.731818
13   GATAATTCGACCGTCGCG-GCC

************ ** * **
Consensus:

GATAATTCGACCgTCacGaGCc

>Bacillus_Fam_687_22_2 Nr. of seq. 2 Alignment length(with gaps) = 22
Alignment score = 0.613636
GCF_001484965.1_ASM148496v1_genomic.fna_1:33695-33821 Satlength=127 Nr of
Repeats=6 RepeatLength=21 seed=CTCTCGGCGT Num.seqs=6 Similarity=0.881481
0   CTCTCGGCGTTCC-ACGGCTCC
GCF_001484965.1_ASM148496v1_genomic.fna_4:169657-169825 Satlength=169 Nr
of Repeats=7 RepeatLength=21 seed=TCTCGGCGTT Num.seqs=6
Similarity=0.847619
0   -TCTCGGCGTTCCGAGCCCTTC

************ *   ** *
Consensus:

cTCTCGGCGTTCCgAcccCTcC

>Bacillus_Fam_688_22_2 Nr. of seq. 2 Alignment length(with gaps) = 22
Alignment score = 0.803030
GCF_001591805.1_ASM159180v1_genomic.fna_9:281393-281477 Satlength=85 Nr
of Repeats=4 RepeatLength=21 seed=TCGCAAATAA Num.seqs=4
Similarity=0.557971
13   AAACA-GCTANGATTCGCAAAT
Rev.of_GCF_000508325.1_BAVI_1_genomic.fna_12:52698-52782 Satlength=85 Nr
of Repeats=4 RepeatLength=21 seed=TTTATTTGCG Num.seqs=4
Similarity=0.696970
24   AAACAGGATAAGATTCGCAAAT

************* * ** ***
Consensus:

TCGCAAATAAACAgGaTAaGAT

>Bacillus_Fam_689_22_2 Nr. of seq. 2 Alignment length(with gaps) = 22
Alignment score = 0.643939
GCF_001591805.1_ASM159180v1_genomic.fna_32:4437-4855 Satlength=419 Nr of Repeats=4 RepeatLength=21 seed=TTTTGTCTAT Num.seqs=3 Similarity=0.606061
19   TTGTCTATTTTTNAGNTAG-TT
GCF_001591805.1_ASM159180v1_genomic.fna_34:3058-3142 Satlength=85 Nr of Repeats=4 RepeatLength=21 seed=GTTTTGTCTA Num.seqs=4 Similarity=0.683081
19   TTGTCTA-TTTTAATAGAGGTT

********* **** *   **
Consensus:

gTTTTGTCTAtTTTTaAgagAG

>Bacillus_Fam_690_22_2 Nr. of seq. 2 Alignment length(with gaps) = 22
Alignment score = 0.659091
GCF_001648575.1_ASM164857v1_genomic.fna_62:153306-153390 Satlength=85 Nr of Repeats=4 RepeatLength=21 seed=AGTTCGTGCC Num.seqs=4
Similarity=0.544974
0   AGTTCGTGCCAAAATNAGACA-
Rev.of_GCF_001648575.1_ASM164857v1_genomic.fna_64:121834-121917
Satlength=84 Nr of Repeats=4 RepeatLength=21 seed=GGCACGAACT Num.seqs=3
Similarity=0.540404   10
AGTTCGTGCCAAAA-CNGNCCG

**************   * *
Consensus:

AGTTCGTGCCAAAAtcaGaCag

>Bacillus_Fam_691_22_2 Nr. of seq. 2 Alignment length(with gaps) = 22
Alignment score = 0.750000
GCF_002019665.1_ASM201966v1_genomic.fna_1:314590-315478 Satlength=889 Nr of Repeats=19 RepeatLength=21 seed=AGGTTCTCAC Num.seqs=14
Similarity=0.616883   0
AGGTTCTCACGCTTCTC-GNAT
GCF_002019665.1_ASM201966v1_genomic.fna_1:3647860-3648050 Satlength=191 Nr of Repeats=9 RepeatLength=21 seed=CACGCTTCTC Num.seqs=8
Similarity=0.907029   6
-GGTTCTCACGCTTCTCAGCAA

**************** * *
Consensus:

aGGTTCTCACGCTTCTCaGcAa

>Bacillus_Fam_692_22_2 Nr. of seq. 2 Alignment length(with gaps) = 22
Alignment score = 0.621212
GCF_002019665.1_ASM201966v1_genomic.fna_1:3182387-3182471 Satlength=85 Nr of Repeats=4 RepeatLength=21 seed=AGGTTCTCAC Num.seqs=4
Similarity=0.465278   0
AGGTTCTCACCGTTCTC-CCTG

GCF_002019665.1_ASM201966v1_genomic.fna_1:4369800-4369884 Satlength=85 Nr of Repeats=4 RepeatLength=21 seed=AGGTTCTCAC Num.seqs=4 Similarity=0.646465 0 AGGTTCTCACATAACTCACNTG

********** *** * **
Consensus:

AGGTTCTCACagaaCTCaCcTG

>Bacillus_Fam_693_22_2 Nr. of seq. 2 Alignment length(with gaps) = 22 Alignment score = 0.651515
GCF_900094975.1_IMG-taxon_2617270721_annotated_assembly_genomic.fna_3:36555-36709 Satlength=155 Nr of Repeats=5 RepeatLength=21 seed=TTCGATAATA Num.seqs=3 Similarity=0.574879 0 TTCGATAATAAAACTTNG-ATT
GCF_900094975.1_IMG-taxon_2617270721_annotated_assembly_genomic.fna_4:286502-286606 Satlength=105 Nr of Repeats=5 RepeatLength=21 seed=TTTTGATAAT Num.seqs=4 Similarity=0.629630 20 TTTGATAAT-AAATTTGGAATT

** ****** *** ** * ***
Consensus:

TTcGATAATaAAAcTTgGaATT

>Bacillus_Fam_694_22_2 Nr. of seq. 2 Alignment length(with gaps) = 22 Alignment score = 0.795455
GCF_900103955.1_IMG-taxon_2617270767_annotated_assembly_genomic.fna_6:34473-34708 Satlength=236 Nr of Repeats=5 RepeatLength=21 seed=TATACTTTAC Num.seqs=3 Similarity=0.830688 0 -TATACTTTACTCCGATCTCTT
GCF_900103955.1_IMG-taxon_2617270767_annotated_assembly_genomic.fna_7:19890-20391 Satlength=502 Nr of Repeats=6 RepeatLength=21 seed=CTATGCTTTA Num.seqs=4 Similarity=0.743687 0 CTATGCTTTACTCCGATC-CTT

*** ************* ***
Consensus:

cTATaCTTTACTCCGATCtCTT

>Bacillus_Fam_695_21_2 Nr. of seq. 2 Alignment length(with gaps) = 21 Alignment score = 0.603175
GCF_001517135.1_ASM151713v1_genomic.fna_21:91763-91907 Satlength=145 Nr of Repeats=8 RepeatLength=18 seed=AAGAACCTGA Num.seqs=8 Similarity=0.936508
0 AAGAACCTGATAACACA-G--
GCF_001645705.1_ASM164570v1_genomic.fna_60:514544-514944 Satlength=401 Nr of Repeats=14 RepeatLength=20 seed=AAGAAGCCCA Num.seqs=10

Similarity=0.740388 0
AAGAACCCCATAACTCANGCT

******* ***** ** *
Consensus:

AAGAACCccATAACaCAnGct

>Bacillus_Fam_696_21_2 Nr. of seq. 2 Alignment length(with gaps) = 21
Alignment score = 0.603175
GCF_001636325.1_ASM163632v1_genomic.fna_2:1329299-1329389 Satlength=91 Nr of Repeats=5 RepeatLength=18 seed=CAAGCTCAAA Num.seqs=5
Similarity=0.970370 0
C-AAGCTCAAATGCCAATG--
Rev.of_GCF_000612665.1_FF4_genomic.fna_50:5-89 Satlength=85 Nr of Repeats=4 RepeatLength=21 seed=CCCATTGGCA Num.seqs=4 Similarity=0.773990
0 CTACGGTCAGATGCCAATGGG

* * * *** *********
Consensus:

CtAaGcTCAaATGCCAATGgg

>Bacillus_Fam_697_21_2 Nr. of seq. 2 Alignment length(with gaps) = 21
Alignment score = 0.634921
GCF_001591465.1_ASM159146v1_genomic.fna_71:19109-19211 Satlength=103 Nr of Repeats=5 RepeatLength=20 seed=TGCAAATAGA Num.seqs=3
Similarity=0.521739
0 TGCAAATAGAACGCTTTTT--
Rev.of_GCF_000986785.1_ASM98678v1_genomic.fna_10:28785-29115
Satlength=331 Nr of Repeats=10 RepeatLength=21 seed=TCTATTTGCC
Num.seqs=6 Similarity=0.663636
10 GGCAAATAGAACGAGGTTTCC

************ ***
Consensus:

gGCAAATAGAACGaggTTTcc

>Bacillus_Fam_698_21_2 Nr. of seq. 2 Alignment length(with gaps) = 21
Alignment score = 0.658730
GCF_900104555.1_IMG-taxon_2651870172_annotated_assembly_genomic.fna_24:29769-29949
Satlength=181 Nr of Repeats=10 RepeatLength=18 seed=CTCCACCTTG
Num.seqs=10 Similarity=0.690643 0
CTCCACCTTGTGGATTG-GC-
Rev.of_GCF_001591665.1_ASM159166v1_genomic.fna_59:13286-13410
Satlength=125 Nr of Repeats=6 RepeatLength=21 seed=CGCAAGTTGG Num.seqs=5
Similarity=0.707937 12
CTCCAACTTGCGGATAGAGCN

***** **** **** * **
Consensus:

CTCCAaCTTGcGGATaGaGCn

>Bacillus_Fam_699_21_2 Nr. of seq. 2 Alignment length(with gaps) = 21
Alignment score = 0.785714
GCF_000175075.1_ASM17507v1_genomic.fna_5:45232-45332 Satlength=101 Nr of Repeats=5 RepeatLength=20 seed=ATATATCAAG Num.seqs=5 Similarity=0.625758
0   ATATATCAAGAAATTTAG-GA
GCF_000175075.1_ASM17507v1_genomic.fna_32:297990-298092 Satlength=103 Nr of Repeats=5 RepeatLength=20 seed=ATATCAAGAA Num.seqs=3 Similarity=0.777778
1   -TATATCAAGAAATTTTGTGA

*************** * **
Consensus:

aTATATCAAGAAATTTaGtGA

>Bacillus_Fam_700_21_2 Nr. of seq. 2 Alignment length(with gaps) = 21
Alignment score = 0.626984
GCF_000175075.1_ASM17507v1_genomic.fna_41:62510-62591 Satlength=82 Nr of Repeats=4 RepeatLength=20 seed=TCGCCCGTAA Num.seqs=3 Similarity=0.712121
0   TCGCCCGTAAATCTCAAAAN-
GCF_001877785.1_PlanoSAMM_genomic.fna_96:320102-320182 Satlength=81 Nr of Repeats=4 RepeatLength=20 seed=TTTCGCCCTT Num.seqs=4 Similarity=0.648148
18   TCGCCCTTAAATC-CGANATT

****** ****** * * *
Consensus:

TCGCCCgTAAATCtCaAaAtt

>Bacillus_Fam_701_21_2 Nr. of seq. 2 Alignment length(with gaps) = 21
Alignment score = 0.706349
GCF_000612805.1_FF3_genomic.fna_6:10-112 Satlength=103 Nr of Repeats=5 RepeatLength=20 seed=CGCAGATAAC Num.seqs=3 Similarity=0.911111
0   CGCAGATAACTCAAGAAATC-
GCF_000612805.1_FF3_genomic.fna_1:1030894-1030997 Satlength=104 Nr of Repeats=5 RepeatLength=21 seed=CGCAGATAAC Num.seqs=3 Similarity=0.787879
0   CGCAGATAANTCATGAAAAAG

********* *** ****
Consensus:

CGCAGATAAcTCAaGAAAaag

>Bacillus_Fam_702_21_2 Nr. of seq. 2 Alignment length(with gaps) = 21
Alignment score = 0.603175
GCF_000813125.1_ASM81312v1_genomic.fna_118:60365-60738 Satlength=374 Nr of Repeats=7 RepeatLength=21 seed=TTTACGGTTG Num.seqs=5 Similarity=0.585507
0   TTTACGGTTGNACAACTTT-A
Rev.of_GCF_001591425.1_ASM159142v1_genomic.fna_85:16-117 Satlength=102 Nr of Repeats=5 RepeatLength=20 seed=CGCAAATAAA Num.seqs=4

Similarity=0.656085 6
TTTGCGGTAGCACGATTTTNA

*** **** * ** * *** *
Consensus:

TTTaCGGTaGcACaAcTTTnA

>Bacillus_Fam_703_21_2 Nr. of seq. 2 Alignment length(with gaps) = 21
Alignment score = 0.682540
GCF_000986785.1_ASM98678v1_genomic.fna_6:41878-42012 Satlength=135 Nr of Repeats=5 RepeatLength=21 seed=TTTGTCGTAA Num.seqs=3 Similarity=0.448718
0   TTTGTCGTAAG-GCAGCNAGT
GCF_000986785.1_ASM98678v1_genomic.fna_11:136737-137027 Satlength=291 Nr of Repeats=9 RepeatLength=21 seed=TTTGTCGTAA Num.seqs=7 Similarity=0.665224
0   TTTGTCGTAAGTGCTGTTANT

*********** ** * * *
Consensus:

TTTGTCGTAAGtGCaGctAgT

>Bacillus_Fam_704_21_2 Nr. of seq. 2 Alignment length(with gaps) = 21
Alignment score = 0.650794
GCF_001591445.1_ASM159144v1_genomic.fna_32:26340-26445 Satlength=106 Nr of Repeats=5 RepeatLength=21 seed=TTGGCGGAAT Num.seqs=5 Similarity=0.429333
0   TTGGCGGAATNACA-TCAAGN
Rev.of_GCF_000508325.1_BAVI_1_genomic.fna_81:19982-20417 Satlength=436 Nr of Repeats=17 RepeatLength=21 seed=ATTTCGCCAA Num.seqs=12 Similarity=0.524772 10
TTGGCGANATTACNGTCAAGN

****** ** ** ******
Consensus:

TTGGCGaaATtACagTCAAGN

>Bacillus_Fam_705_21_2 Nr. of seq. 2 Alignment length(with gaps) = 21
Alignment score = 0.666667
GCF_001636345.1_ASM163634v1_genomic.fna_1:492235-492415 Satlength=181 Nr of Repeats=9 RepeatLength=20 seed=ACGGCGAAAA Num.seqs=9 Similarity=0.713404
0   AC-GGCGAAAAAAACNTAATA
GCF_000508325.1_BAVI_1_genomic.fna_89:32-156 Satlength=125 Nr of Repeats=5 RepeatLength=21 seed=ACTGGCGAGA Num.seqs=4 Similarity=0.538889
0   ACTGGCGAGAAAAAGCTAATT

** ***** ***** ****
Consensus:

ACtGGCGAaAAAAAccTAATa

>Bacillus_Fam_706_21_2 Nr. of seq. 2 Alignment length(with gaps) = 21
Alignment score = 0.706349
GCF_001712755.1_ASM171275v1_genomic.fna_37:245546-245745 Satlength=200 Nr of Repeats=10 RepeatLength=20 seed=GCAAATAAAA Num.seqs=9 Similarity=0.735185 0
GCAAATAAAAACTGGNAATA-
Rev.of_GCF_001712755.1_ASM171275v1_genomic.fna_33:175176-175416 Satlength=241 Nr of Repeats=12 RepeatLength=20 seed=TTTTTATTTG Num.seqs=12 Similarity=0.736412
11   GCAAATAAAAAATGTGTATAC

*********** **   ***
Consensus:

GCAAATAAAAAaTGggaATAc

>Bacillus_Fam_707_21_2 Nr. of seq. 2 Alignment length(with gaps) = 21
Alignment score = 0.666667
GCF_001877785.1_PlanoSAMM_genomic.fna_106:238188-238447 Satlength=260 Nr of Repeats=5 RepeatLength=20 seed=TTATCAACAA Num.seqs=3 Similarity=0.733333
0   TTATCAACA-ATTTTTAGGAT
GCF_000759675.1_ASM75967v1_genomic.fna_13:38889-38973 Satlength=85 Nr of Repeats=4 RepeatLength=21 seed=TATCTACACA Num.seqs=4 Similarity=0.498843
1   TTATCTACACACTTTTAGANT

***** *** * ****** *
Consensus:

TTATCaACAcAcTTTTAGaaT

>Bacillus_Fam_708_21_2 Nr. of seq. 2 Alignment length(with gaps) = 21
Alignment score = 0.666667
GCF_002019645.1_ASM201964v1_genomic.fna_1:280014-280475 Satlength=462 Nr of Repeats=18 RepeatLength=21 seed=AAAATACGGC Num.seqs=12 Similarity=0.580369 0
AAAATACGGCGGNTTTGA-CA
GCF_002019645.1_ASM201964v1_genomic.fna_1:5314586-5314815 Satlength=230 Nr of Repeats=5 RepeatLength=21 seed=AATACGGCGG Num.seqs=3 Similarity=0.873016 2
CGAATACGGCGGGTTTGAGCC

********** ***** *
Consensus:

aaAATACGGCGGgTTTGAgCa

>Bacillus_Fam_709_21_2 Nr. of seq. 2 Alignment length(with gaps) = 21
Alignment score = 0.619048
GCF_900111815.1_PRJEB17078_genomic.fna_1:2579479-2579833 Satlength=355 Nr of Repeats=10 RepeatLength=21 seed=TTGGTTGATT Num.seqs=8 Similarity=0.642081 0
TTGGTTGATTT-CTAGAGAAG

GCF_900111815.1_PRJEB17078_genomic.fna_1:2923151-2924173 Satlength=1023 Nr of Repeats=29 RepeatLength=21 seed=TGGTTGATTT Num.seqs=19 Similarity=0.555217 1
TTGGTTGATTTCCTGCTGNAN

*********** ** * *
Consensus:

TTGGTTGATTTcCTacaGaAg

>Bacillus_Fam_710_21_2 Nr. of seq. 2 Alignment length(with gaps) = 21
Alignment score = 0.690476
GCF_900156865.1_PRJEB18960_genomic.fna_3:4206861-4206941 Satlength=81 Nr of Repeats=4 RepeatLength=20 seed=GACAGTATTT Num.seqs=4
Similarity=0.666667
0 GACAGTATTTCCAAAATAGC-
Rev.of_GCF_000712595.1_ASM71259v1_genomic.fna_14:34518-34623
Satlength=106 Nr of Repeats=5 RepeatLength=21 seed=TCGGCTATTT Num.seqs=5
Similarity=0.665152 2
GAAAGTGTATCGAAAATAGCC

** *** * ** ********
Consensus:

GAaAGTaTaTCcAAAATAGCc

>Bacillus_Fam_711_21_2 Nr. of seq. 2 Alignment length(with gaps) = 21
Alignment score = 0.809524
GCF_000169195.2_ASM16919v2_genomic.fna_1:1723063-1723231 Satlength=169 Nr of Repeats=6 RepeatLength=21 seed=GCAACATCCT Num.seqs=4
Similarity=0.714286 0
GCAACATCCTGTTTCAGCACT
Rev.of_GCF_000832905.1_ASM83290v1_genomic.fna_1:1842445-1842529
Satlength=85 Nr of Repeats=4 RepeatLength=21 seed=GATGTTGCAG Num.seqs=4
Similarity=0.873016 8
GCAACATCTTGTTTTAGCCCT

******** ***** *** **
Consensus:

GCAACATCcTGTTTcAGCaCT

>Bacillus_Fam_712_21_2 Nr. of seq. 2 Alignment length(with gaps) = 21
Alignment score = 0.873016
GCF_000175075.1_ASM17507v1_genomic.fna_34:92519-92645 Satlength=127 Nr of Repeats=4 RepeatLength=21 seed=TGTTAGTACA Num.seqs=3 Similarity=0.830688
0 TGTTAGTACATTAAAGAGTGA
Rev.of_GCF_001648575.1_ASM164857v1_genomic.fna_48:38804-38909
Satlength=106 Nr of Repeats=5 RepeatLength=21 seed=ACTAACATCA Num.seqs=5
Similarity=0.826087 7
TGTTAGTGTATTAAAGAGTGA

******* ************
Consensus:

TGTTAGTacATTAAAGAGTGA

>Bacillus_Fam_713_21_2 Nr. of seq. 2 Alignment length(with gaps) = 21
Alignment score = 0.746032
GCF_000175075.1_ASM17507v1_genomic.fna_36:48707-48854 Satlength=148 Nr of Repeats=7 RepeatLength=21 seed=GGAGGCTCTT Num.seqs=7 Similarity=0.830688
0 GGAGGCTCTTCGCCATCACCC
Rev.of_GCF_001889165.1_ASM188916v1_genomic.fna_1:146949-147096 Satlength=148 Nr of Repeats=7 RepeatLength=21 seed=GGAGAAGAAA Num.seqs=7 Similarity=0.927438 14
GGTGTTTCTTCTCCATCACCC

** * ***** *********
Consensus:

GGaGgcTCTTCgCCATCACCC

>Bacillus_Fam_714_21_2 Nr. of seq. 2 Alignment length(with gaps) = 21
Alignment score = 0.746032
GCF_000307875.1_BABA1.0_genomic.fna_138:19368-19452 Satlength=85 Nr of Repeats=4 RepeatLength=21 seed=ATCCGACAGT Num.seqs=4 Similarity=0.723485
20 TCCGACAGTTAGCTGCGGTTA
Rev.of_GCF_001591665.1_ASM159166v1_genomic.fna_22:13463-13608 Satlength=146 Nr of Repeats=7 RepeatLength=21 seed=ACTGTCGGAT Num.seqs=5 Similarity=0.675758 30
TCCGACAGTAAGCCGCTGCTA

********** *** ** * *
Consensus:

ATCCGACAGTaAGCcGCgGcT

>Bacillus_Fam_715_21_2 Nr. of seq. 2 Alignment length(with gaps) = 21
Alignment score = 0.809524
GCF_000508325.1_BAVI_1_genomic.fna_59:240543-240669 Satlength=127 Nr of Repeats=5 RepeatLength=21 seed=TTCAAAATTT Num.seqs=4 Similarity=0.820106
0 TTCAAAATTTCACTGTGATCG
Rev.of_GCF_000724485.1_ASM72448v1_genomic.fna_1:3059785-3059911 Satlength=127 Nr of Repeats=5 RepeatLength=21 seed=TTTGAAAGAA Num.seqs=4 Similarity=0.693122 6
TTCAAAGTTTCACTGTGTTCT

****** ********** **
Consensus:

TTCAAAaTTTCACTGTGaTCg

>Bacillus_Fam_716_21_2 Nr. of seq. 2 Alignment length(with gaps) = 21
Alignment score = 0.650794
GCF_000612625.1_JCE_genomic.fna_78:17147-17414 Satlength=268 Nr of Repeats=5 RepeatLength=21 seed=TTTATCAACC Num.seqs=3 Similarity=0.666667
0 TTTATCAACCACTTTTCCTCN

GCF_002019635.1_ASM201963v1_genomic.fna_1:406736-407553 Satlength=818 Nr of Repeats=28 RepeatLength=21 seed=TTCAACCACT Num.seqs=21 Similarity=0.616011 3
TNTTTCAACCACTTCTCTGCT

* * ********** ** *
Consensus:

TtTaTCAACCACTTcTCcgCt

>Bacillus_Fam_717_21_2 Nr. of seq. 2 Alignment length(with gaps) = 21 Alignment score = 0.873016
GCF_000708755.2_ASM70875v2_genomic.fna_7:112578-112785 Satlength=208 Nr of Repeats=7 RepeatLength=21 seed=TTACTACCAA Num.seqs=5 Similarity=0.714286
0   TTACTACCAACTACCGCAGAT
GCF_000709935.2_ASM70993v2_genomic.fna_19:119838-120024 Satlength=187 Nr of Repeats=6 RepeatLength=21 seed=TTACTACCAA Num.seqs=4 Similarity=0.671958
0   TTACTACCAACAACCACAGAT

*********** *** *****
Consensus:

TTACTACCAACaACCaCAGAT

>Bacillus_Fam_718_21_2 Nr. of seq. 2 Alignment length(with gaps) = 21 Alignment score = 0.682540
GCF_000712595.1_ASM71259v1_genomic.fna_21:13433-13580 Satlength=148 Nr of Repeats=7 RepeatLength=21 seed=ATAGCCGATA Num.seqs=7 Similarity=0.860922
0   ATAGCCGATACACCCGTGAAA
Rev.of_GCF_000752035.1_Bacillus_rubiinfantis_genomic.fna_12:4665-5905 Satlength=1241 Nr of Repeats=58 RepeatLength=21 seed=TTACGGGAGT Num.seqs=45 Similarity=0.766697 19
AGCGCGGATACTCCCGTAAAA

* ** ***** ***** ***
Consensus:

AgaGCcGATACaCCCGTaAAA

>Bacillus_Fam_719_21_2 Nr. of seq. 2 Alignment length(with gaps) = 21 Alignment score = 0.698413
GCF_000813125.1_ASM81312v1_genomic.fna_142:26887-26971 Satlength=85 Nr of Repeats=4 RepeatLength=21 seed=AATCACTACG Num.seqs=4 Similarity=0.556713
0   AATCACTACGGAATTCAAANT
Rev.of_GCF_000813125.1_ASM81312v1_genomic.fna_142:36627-36711 Satlength=85 Nr of Repeats=4 RepeatLength=21 seed=TCGTAGTGAT Num.seqs=4 Similarity=0.612374 11
AATCACTACGAAAATTAGAAT

********** ** * * * *
Consensus:

AATCACTACGaAAaTcAaAaT

>Bacillus_Fam_720_21_2 Nr. of seq. 2 Alignment length(with gaps) = 21
Alignment score = 0.746032
GCF_000948175.1_BFA_2_A_genomic.fna_157:32173-32278 Satlength=106 Nr of Repeats=5 RepeatLength=21 seed=GTATTCGACA Num.seqs=5 Similarity=0.974603
0   GTATTCGACACACTTTGGATT
GCF_000948175.1_BFA_2_A_genomic.fna_161:7622-7844 Satlength=223 Nr of Repeats=4 RepeatLength=21 seed=GGATCGTATT Num.seqs=3 Similarity=1.000000
16   GTATTCGACAGAAGTTGGATC

********** * ******
Consensus:

GTATTCGACAcAagTTGGATc

>Bacillus_Fam_721_21_2 Nr. of seq. 2 Alignment length(with gaps) = 21
Alignment score = 0.888889
GCF_001273755.1_ASM127375v1_genomic.fna_17:235079-235207 Satlength=129 Nr of Repeats=6 RepeatLength=21 seed=TTCACGAGTA Num.seqs=5
Similarity=0.710145 30
TCNTGAAAATTCACGAGTAAA
Rev.of_GCF_001273755.1_ASM127375v1_genomic.fna_17:3961735-3962511
Satlength=777 Nr of Repeats=38 RepeatLength=21 seed=TTACTCGTGA
Num.seqs=27 Similarity=0.823814
41   TCGTGAAAACTCACGAGTAAA

************* ******
Consensus:

cTCACGAGTAAATCgTGAAAA

>Bacillus_Fam_722_21_2 Nr. of seq. 2 Alignment length(with gaps) = 21
Alignment score = 0.682540
GCF_001315065.1_ASM131506v1_genomic.fna_30:7615-7720 Satlength=106 Nr of Repeats=5 RepeatLength=21 seed=GCCTTCATCT Num.seqs=5 Similarity=1.000000
0   GCCTTCATCTCCGTCTCCAGG
GCF_002019635.1_ASM201963v1_genomic.fna_1:1180363-1180615 Satlength=253
Nr of Repeats=11 RepeatLength=21 seed=CTGGATCTTG Num.seqs=10
Similarity=0.880071 17
ATCTTGATCTCCGTCACCTGG

*** ********* ** **
Consensus:

acCTTcATCTCCGTCaCCaGG

>Bacillus_Fam_723_21_2 Nr. of seq. 2 Alignment length(with gaps) = 21
Alignment score = 0.698413
GCF_001315165.1_ASM131516v1_genomic.fna_27:15017-15121 Satlength=105 Nr of Repeats=4 RepeatLength=21 seed=GTGGTCGATA Num.seqs=3
Similarity=0.767196
0   GTGGTCGATATAATCANAAAA

GCF_002019645.1_ASM201964v1_genomic.fna_1:659769-659933 Satlength=165 Nr of Repeats=8 RepeatLength=21 seed=CAATAAAACC Num.seqs=6 Similarity=0.817989
5   TTGGTCAATAAAACCAGAAAA

***** *** ** ** ****
Consensus:

gTGGTCaATAaAAcCAgAAAA

>Bacillus_Fam_724_21_2 Nr. of seq. 2 Alignment length(with gaps) = 21 Alignment score = 0.619048
GCF_001591425.1_ASM159142v1_genomic.fna_40:10164-10290 Satlength=127 Nr of Repeats=6 RepeatLength=21 seed=TGTTAAATAA Num.seqs=6 Similarity=0.654589
0   TGTTAAATAAAATCTCAAAGT
GCF_001591425.1_ASM159142v1_genomic.fna_132:2162-2350 Satlength=189 Nr of Repeats=8 RepeatLength=21 seed=CTGTTAAGTA Num.seqs=6 Similarity=0.712169
20   TGTTAAGAAAAACCACAAACC

****** **** * ****
Consensus:

TGTTAAaaAAAAcCaCAAAcc

>Bacillus_Fam_725_21_2 Nr. of seq. 2 Alignment length(with gaps) = 21 Alignment score = 0.825397
GCF_001591445.1_ASM159144v1_genomic.fna_9:109889-109973 Satlength=85 Nr of Repeats=4 RepeatLength=21 seed=CGCCAATAGA Num.seqs=4 Similarity=0.815657
0   CGCCAATAGAACCTCTGAATC
GCF_001591445.1_ASM159144v1_genomic.fna_9:169675-169822 Satlength=148 Nr of Repeats=7 RepeatLength=21 seed=CGCCAATAGA Num.seqs=7 Similarity=0.779289
0   CGCCAATAGAACCTGNAAATC

**************   ****
Consensus:

CGCCAATAGAACCTctaAATC

>Bacillus_Fam_726_21_2 Nr. of seq. 2 Alignment length(with gaps) = 21 Alignment score = 0.619048
GCF_001591665.1_ASM159166v1_genomic.fna_82:10-115 Satlength=106 Nr of Repeats=5 RepeatLength=21 seed=ATCCGACAGT Num.seqs=5 Similarity=0.898413
0   ATCCGACAGTTGGAACACGCT
Rev.of_GCF_001591805.1_ASM159180v1_genomic.fna_15:119662-119746 Satlength=85 Nr of Repeats=4 RepeatLength=21 seed=GCCAACTGTC Num.seqs=4 Similarity=0.904762   14
TTGCGACAGTTGGCTCTTGCT

* ********** * ***
Consensus:

aTcCGACAGTTGGaaCacGCT

>Bacillus_Fam_727_21_2 Nr. of seq. 2 Alignment length(with gaps) = 21
Alignment score = 0.682540
GCF_001591805.1_ASM159180v1_genomic.fna_15:30953-31058 Satlength=106 Nr of Repeats=5 RepeatLength=21 seed=TTGGCGAAAT Num.seqs=5 Similarity=0.587879
0   TTGGCGAAATNCNNNCACAGT
GCF_001591805.1_ASM159180v1_genomic.fna_15:39100-39247 Satlength=148 Nr of Repeats=7 RepeatLength=21 seed=AGTTTGGCGA Num.seqs=7 Similarity=0.818594
18   TTGGCGAAATCCCNGCGTAGT

********** * * * ***
Consensus:

TTGGCGAAATcCcNgCacAGT

>Bacillus_Fam_728_20_2 Nr. of seq. 2 Alignment length(with gaps) = 20
Alignment score = 0.800000
GCF_000175075.1_ASM17507v1_genomic.fna_29:50387-50489 Satlength=103 Nr of Repeats=5 RepeatLength=20 seed=TATCCATTGT Num.seqs=3 Similarity=0.777778
0   TATCCATTGTAAATTTGATA
GCF_001648575.1_ASM164857v1_genomic.fna_66:142753-142855 Satlength=103 Nr of Repeats=5 RepeatLength=20 seed=TATCCATTGT Num.seqs=3 Similarity=0.777778   0
TATCCATTGTTAATTGAATA

********** **** ***
Consensus:

TATCCATTGTaAATTgaATA

>Bacillus_Fam_729_20_2 Nr. of seq. 2 Alignment length(with gaps) = 20
Alignment score = 0.616667
GCF_000181495.1_ASM18149v1_genomic.fna_133:12224-12305 Satlength=82 Nr of Repeats=4 RepeatLength=20 seed=GACGCAATTA Num.seqs=3 Similarity=0.544974
0   GACGCAATTATTTCATATTA
Rev.of_GCF_000181495.1_ASM18149v1_genomic.fna_147:20393-20494 Satlength=102 Nr of Repeats=5 RepeatLength=20 seed=TAATTGCGTC Num.seqs=4 Similarity=0.498843   10
GACGCAATTAAACTGNATTA

**********   ****
Consensus:

GACGCAATTAaaccatATTA

>Bacillus_Fam_730_20_2 Nr. of seq. 2 Alignment length(with gaps) = 20
Alignment score = 0.700000
GCF_000285535.1_ASM28553v1_genomic.fna_2:785830-786454 Satlength=625 Nr of Repeats=30 RepeatLength=20 seed=TTTGCGTCTC Num.seqs=25

Similarity=0.766222 0
TTTGCGTCTCCTCCCNGATA
GCF_000285535.1_ASM28553v1_genomic.fna_62:31-473 Satlength=443 Nr of Repeats=22 RepeatLength=20 seed=ATTTGCGTCT Num.seqs=21 Similarity=0.616594
19 TTTGCGTCTCCCAGNTGATA

*********** ****
Consensus:

TTTGCGTCTCCcacctGATA

>Bacillus_Fam_731_20_2 Nr. of seq. 2 Alignment length(with gaps) = 20 Alignment score = 0.750000
GCF_000311725.1_ASM31172v1_genomic.fna_2:224797-224897 Satlength=101 Nr of Repeats=5 RepeatLength=20 seed=TATATCGGCG Num.seqs=5 Similarity=0.593651
14 GGCGNTTTTAGCAATATATC
GCF_000311725.1_ASM31172v1_genomic.fna_4:95730-95830 Satlength=101 Nr of Repeats=5 RepeatLength=20 seed=TATATCGGCG Num.seqs=3 Similarity=0.671958
14 GGCGTTTTTTGAGATATATC

********** **** * *
Consensus:

TATATCGGCGtTTTTaGaaA

>Bacillus_Fam_732_20_2 Nr. of seq. 2 Alignment length(with gaps) = 20 Alignment score = 0.883333
GCF_000380245.2_ASM38024v2_genomic.fna_9:119172-119272 Satlength=101 Nr of Repeats=5 RepeatLength=20 seed=CTCATATATT Num.seqs=5 Similarity=0.893333
0 CTCATATATTTTGTTCTCNG
GCF_000380245.2_ASM38024v2_genomic.fna_9:119172-119412 Satlength=241 Nr of Repeats=12 RepeatLength=20 seed=CTCATATATT Num.seqs=12 Similarity=0.859596 0
CTCATATATTTCGTTCTCCG

*********** ****** *
Consensus:

CTCATATATTTcGTTCTCcG

>Bacillus_Fam_733_20_2 Nr. of seq. 2 Alignment length(with gaps) = 20 Alignment score = 0.750000
GCF_001591585.1_ASM159158v1_genomic.fna_8:146075-146177 Satlength=103 Nr of Repeats=5 RepeatLength=20 seed=TTTGGCTGAT Num.seqs=3 Similarity=0.575758
0 TTTGGCTGATATTTTTCTNC
Rev.of_GCF_001591585.1_ASM159158v1_genomic.fna_18:3-309 Satlength=307 Nr of Repeats=15 RepeatLength=20 seed=ATCAGCCAAA Num.seqs=9 Similarity=0.798942 10
TTTGGCTGATATTTTCCCAG

*************** *
Consensus:

TTTGGCTGATATTTTcCcac

>Bacillus_Fam_734_19_2 Nr. of seq. 2 Alignment length(with gaps) = 19
Alignment score = 0.649123
BACILLUS_PSEUDALCALIPHILUS_genomic.fna_3:387353-387569 Satlength=217 Nr of Repeats=10 RepeatLength=18 seed=CCCGGATGAG Num.seqs=9
Similarity=0.872428 0
CCCGGATG-AGGTCCTGGT
Rev.of_GCF_001636325.1_ASM163632v1_genomic.fna_2:690747-691053
Satlength=307 Nr of Repeats=17 RepeatLength=18 seed=CCAGCACCAG
Num.seqs=17 Similarity=0.827887
5 GCTGG-TGAAGGTCCTGGT

* ** ** **********
Consensus:

cCcGGaTGaAGGTCCTGGT

>Bacillus_Fam_735_19_2 Nr. of seq. 2 Alignment length(with gaps) = 19
Alignment score = 0.622807
GCF_000008505.1_ASM850v1_genomic.fna_1:4938976-4939084 Satlength=109 Nr of Repeats=6 RepeatLength=18 seed=CTGGACAAGC Num.seqs=6
Similarity=0.911111
0 CTGGACAAGCAAG-ACAAT
GCF_000430785.1_ASM43078v1_genomic.fna_13:56722-56902 Satlength=181 Nr of Repeats=8 RepeatLength=18 seed=TTAGAGAAGT Num.seqs=6 Similarity=0.782716
17 -TAGAGAAGCAAGAACACT

* ** ******* *** *
Consensus:

cTaGAcAAGCAAGaACAaT

>Bacillus_Fam_736_19_2 Nr. of seq. 2 Alignment length(with gaps) = 19
Alignment score = 0.649123
GCF_000177235.2_ASM17723v2_genomic.fna_1:380562-380634 Satlength=73 Nr of Repeats=4 RepeatLength=18 seed=AAGCCAATGC Num.seqs=4 Similarity=0.962963
0 AAGCCAATGCTGCC-CACA
Rev.of_GCF_000242895.2_ASM24289v3_genomic.fna_1:1111116-1111242
Satlength=127 Nr of Repeats=7 RepeatLength=18 seed=GCGGCTTTGG Num.seqs=7
Similarity=0.978836 12 AA-
CCAAAGCCGCCACACA

** **** ** *** ****
Consensus:

AAgCCAAaGCcGCCaCACA

>Bacillus_Fam_737_18_2 Nr. of seq. 2 Alignment length(with gaps) = 18
Alignment score = 0.666667

GCF_000007825.1_ASM782v1_genomic.fna_1:4701846-4702305 Satlength=460 Nr of Repeats=12 RepeatLength=18 seed=ACGGGGAACA Num.seqs=10 Similarity=0.925926 0
ACGGGGAACACCGGACCC
GCF_000769555.1_ASM76955v1_genomic.fna_1:742609-742825 Satlength=217 Nr of Repeats=8 RepeatLength=18 seed=ACCGGAGCAA Num.seqs=5 Similarity=0.770370
9 ACGGGGNTNACCGGAGCA

****** ****** *
Consensus:

ACGGGGaacACCGGAcCa

>Bacillus_Fam_738_18_2 Nr. of seq. 2 Alignment length(with gaps) = 18 Alignment score = 0.629630
GCF_000008505.1_ASM850v1_genomic.fna_1:1992091-1992343 Satlength=253 Nr of Repeats=14 RepeatLength=18 seed=CCTGATACAG Num.seqs=14 Similarity=0.888482 0
CCTGATACAGGAGAAAAA
Rev.of_GCF_000161455.1_ASM16145v1_genomic.fna_1:1437244-1437370 Satlength=127 Nr of Repeats=7 RepeatLength=18 seed=TATCTGGTTT Num.seqs=5 Similarity=0.955556 7
CCAGATACACCAAATAAA

** ****** * * ***
Consensus:

CCaGATACAccAaAaAAA

>Bacillus_Fam_739_18_2 Nr. of seq. 2 Alignment length(with gaps) = 18 Alignment score = 0.629630
GCF_000161455.1_ASM16145v1_genomic.fna_1:4033827-4033917 Satlength=91 Nr of Repeats=5 RepeatLength=18 seed=TGTTGATGCT Num.seqs=5 Similarity=0.970370
0 TGTTGATGCTGACCATGG
Rev.of_GCF_000832605.1_ASM83260v1_genomic.fna_1:1245276-1245432 Satlength=157 Nr of Repeats=6 RepeatLength=18 seed=CATCACCAAG Num.seqs=5 Similarity=0.874074 5
TGATGGTGTTGGCCTTGG

** ** ** ** ** ***
Consensus:

TGaTGaTGcTGaCCaTGG

>Bacillus_Fam_740_18_2 Nr. of seq. 2 Alignment length(with gaps) = 18 Alignment score = 0.648148
GCF_000175075.1_ASM17507v1_genomic.fna_32:87599-88088 Satlength=490 Nr of Repeats=18 RepeatLength=18 seed=AACAACAGCA Num.seqs=17 Similarity=0.869281
0 AACAACAGCAACAGCGGN
GCF_900104555.1_IMG-taxon_2651870172_annotated_assembly_genomic.fna_17:3091-3181 Satlength=91

Nr of Repeats=5 RepeatLength=18 seed=ACAACAACAC Num.seqs=5
Similarity=0.940741 1 CACAACAACACCAGCAGT

****** ** **** *
Consensus:

aACAACAaCAaCAGCaGt

>Bacillus_Fam_741_18_2 Nr. of seq. 2 Alignment length(with gaps) = 18
Alignment score = 0.925926
GCF_000177235.2_ASM17723v2_genomic.fna_1:532212-532500 Satlength=289 Nr of Repeats=16 RepeatLength=18 seed=CCAGGCGAAG Num.seqs=16
Similarity=0.934568 0
CCAGGCGAAGGTGAAGGC
GCF_001315065.1_ASM131506v1_genomic.fna_40:23388-23478 Satlength=91 Nr of Repeats=5 RepeatLength=18 seed=AGAAGGTGAA Num.seqs=5 Similarity=0.970370
5 CCAGGAGAAGGTGAAGGC

***** ************
Consensus:

CCAGGaGAAGGTGAAGGC

>Bacillus_Fam_742_18_2 Nr. of seq. 2 Alignment length(with gaps) = 18
Alignment score = 0.629630
GCF_000299035.1_ASM29903v1_genomic.fna_3:476185-476329 Satlength=145 Nr of Repeats=8 RepeatLength=18 seed=ATCTTGGTCT Num.seqs=8
Similarity=0.960317
0 ATCTTGGTCTTTTCCTGG
Rev.of_GCF_001315065.1_ASM131506v1_genomic.fna_13:100988-101102
Satlength=115 Nr of Repeats=4 RepeatLength=18 seed=AGATGAAGAG Num.seqs=3
Similarity=1.000000 10
CTCTTCATCTAGTCCTGG

**** *** ******
Consensus:

aTCTTcaTCTagTCCTGG

>Bacillus_Fam_743_18_2 Nr. of seq. 2 Alignment length(with gaps) = 18
Alignment score = 0.629630
GCF_000299035.1_ASM29903v1_genomic.fna_4:44054-44162 Satlength=109 Nr of Repeats=4 RepeatLength=18 seed=CACCTAATCC Num.seqs=3 Similarity=1.000000
0 CACCTAATCCAACTCCGC
Rev.of_GCF_001439915.1_ASM143991v1_genomic.fna_85:15375-16048
Satlength=674 Nr of Repeats=4 RepeatLength=18 seed=GTGGATTTGG Num.seqs=3
Similarity=0.802469 12
CACCCAATCCACCTAATC

**** ****** ** *
Consensus:

CACCcAATCCAaCTaagC

>Bacillus_Fam_744_18_2 Nr. of seq. 2 Alignment length(with gaps) = 18
Alignment score = 0.703704
GCF_000305495.1_BacPsy2.0_genomic.fna_210:5429-5585 Satlength=157 Nr of Repeats=8 RepeatLength=18 seed=GCTTCTTGTT Num.seqs=6 Similarity=0.866667
0 GCCTCTTGTTCAGCTTCT
GCF_000972245.2_ASM97224v2_genomic.fna_1:252282-252630 Satlength=349 Nr of Repeats=18 RepeatLength=18 seed=TGAGCTTGAG Num.seqs=17 Similarity=0.881264 9
GCCTCTTGCTGAGCTTGA

******** * *****
Consensus:

GCCTCTTGcTcAGCTTca

>Bacillus_Fam_745_18_2 Nr. of seq. 2 Alignment length(with gaps) = 18
Alignment score = 0.777778
GCF_000430785.1_ASM43078v1_genomic.fna_5:20934-21024 Satlength=91 Nr of Repeats=5 RepeatLength=18 seed=ATTAGTACCA Num.seqs=5 Similarity=0.881481
0 ATTAGTACCAGTGCCTGG
GCF_000934845.1_ASM93484v1_genomic.fna_135:23551-23617 Satlength=67 Nr of Repeats=4 RepeatLength=18 seed=GGATCAGTAC Num.seqs=3 Similarity=0.901235
16 ATCAGTACCAGTACCAGG

** ********* ** **
Consensus:

ATcAGTACCAGTaCCaGG

>Bacillus_Fam_746_18_2 Nr. of seq. 2 Alignment length(with gaps) = 18
Alignment score = 0.629630
GCF_000430785.1_ASM43078v1_genomic.fna_24:4949-5111 Satlength=163 Nr of Repeats=8 RepeatLength=18 seed=AGAACTTTTA Num.seqs=7 Similarity=0.957672
0 AGAACTTTTAGAAGCCGT
Rev.of_GCF_001591445.1_ASM159144v1_genomic.fna_30:53287-53467 Satlength=181 Nr of Repeats=10 RepeatLength=18 seed=TTCTAGAAGT Num.seqs=10 Similarity=0.917695
13 AATACTTCTAGAAACCGG

* **** ***** ***
Consensus:

AaaACTTcTAGAAaCCGg

>Bacillus_Fam_747_18_2 Nr. of seq. 2 Alignment length(with gaps) = 18
Alignment score = 0.777778
GCF_000504145.1_SOAPdenovo_for_version2.04_of_Bacillus_sp._FJAT-14515_genome_genomic.fna_25:69856-70072 Satlength=217 Nr of Repeats=12 RepeatLength=18 seed=GGCCCGAATC Num.seqs=12 Similarity=0.987654 0
GGCCCGAATCCCATGCCG
Rev.of_GCF_001273755.1_ASM127375v1_genomic.fna_17:794926-795034 Satlength=109 Nr of Repeats=6 RepeatLength=18 seed=GGAATGGGAT Num.seqs=6 Similarity=0.891358 17
GGGCCGAATCCCATTCCA

** *********** **
Consensus:

GGcCCGAATCCCATgCCa

>Bacillus_Fam_748_18_2 Nr. of seq. 2 Alignment length(with gaps) = 18
Alignment score = 0.851852
GCF_000708755.2_ASM70875v2_genomic.fna_2:520937-521289 Satlength=353 Nr of Repeats=17 RepeatLength=18 seed=GGAGCACCTG Num.seqs=14 Similarity=0.822548 0
GGAGCACCTGTTACGCCC
GCF_000709935.2_ASM70993v2_genomic.fna_1:1110697-1110931 Satlength=235 Nr of Repeats=13 RepeatLength=18 seed=GGAGCACCTG Num.seqs=13 Similarity=0.851852 0
GGAGCACCTGTTGCGCCT

************ ****
Consensus:

GGAGCACCTGTTaCGCCc

>Bacillus_Fam_749_18_2 Nr. of seq. 2 Alignment length(with gaps) = 18
Alignment score = 0.629630
GCF_000709935.2_ASM70993v2_genomic.fna_6:62-278 Satlength=217 Nr of Repeats=10 RepeatLength=18 seed=CCGGCAGGTC Num.seqs=8 Similarity=0.777778
0 CCGGCAGGTCCAGTAACT
Rev.of_GCF_000712615.1_ASM71261v1_genomic.fna_82:4158-4356 Satlength=199 Nr of Repeats=8 RepeatLength=18 seed=CTCAAGGAAT Num.seqs=5 Similarity=0.911111 7
CCTTGAGATCCAGTAATT

** ** ******** *
Consensus:

CCggcAGaTCCAGTAAcT

>Bacillus_Fam_750_18_2 Nr. of seq. 2 Alignment length(with gaps) = 18
Alignment score = 0.703704
GCF_000759675.1_ASM75967v1_genomic.fna_158:37496-37595 Satlength=100 Nr of Repeats=6 RepeatLength=18 seed=GGTGGTATTG Num.seqs=4 Similarity=0.802469
0 GGTGGTATTGGCGGTCCT
GCF_001591445.1_ASM159144v1_genomic.fna_87:10237-10345 Satlength=109 Nr of Repeats=6 RepeatLength=18 seed=GGACATGGTG Num.seqs=6 Similarity=0.925926
12 GGTGGAATGGGCGGACAT

***** ** ***** * *
Consensus:

GGTGGaATgGGCGGaCaT

>Bacillus_Fam_751_18_2 Nr. of seq. 2 Alignment length(with gaps) = 18
Alignment score = 0.777778
GCF_000813125.1_ASM81312v1_genomic.fna_74:2375-2537 Satlength=163 Nr of Repeats=9 RepeatLength=18 seed=ATACCGGAGG Num.seqs=9 Similarity=0.962963
0   ATACCGGAGGCGAAGGAG
GCF_001315165.1_ASM131516v1_genomic.fna_159:2411-2519 Satlength=109 Nr of Repeats=6 RepeatLength=18 seed=GGTGGTGAAG Num.seqs=6 Similarity=0.841975
5   ATACCGGTGGTGAAGAAG

******* ** **** **
Consensus:

ATACCGGaGGcGAAGaAG

>Bacillus_Fam_752_18_2 Nr. of seq. 2 Alignment length(with gaps) = 18
Alignment score = 0.629630
GCF_001274935.1_ASM127493v1_genomic.fna_36:82635-84309 Satlength=1675 Nr of Repeats=91 RepeatLength=18 seed=CCTACTGGTG Num.seqs=88 Similarity=0.883761 0
CCTACTGGTGCTGCTGGT
GCF_001636425.1_ASM163642v1_genomic.fna_1:2005510-2005852 Satlength=343 Nr of Repeats=19 RepeatLength=18 seed=GTGCTTGTGG Num.seqs=16 Similarity=0.973457 1
CGTGCTTGTGGTGCTCGT

* * ** *** **** **
Consensus:

CcTaCTgGTGcTGCTcGT

>Bacillus_Fam_753_18_2 Nr. of seq. 2 Alignment length(with gaps) = 18
Alignment score = 0.666667
GCF_001420715.1_ASM142071v1_genomic.fna_22:3447196-3448294 Satlength=1099 Nr of Repeats=61 RepeatLength=18 seed=CCAGCAGGTG Num.seqs=61 Similarity=0.861486 0
CCAGCAGGTGCTACCGGA
Rev.of_GCF_001712755.1_ASM171275v1_genomic.fna_33:51006-51924 Satlength=919 Nr of Repeats=48 RepeatLength=18 seed=CCTGTTGCTC Num.seqs=45 Similarity=0.793042
17   CCAGCNGGAGCAACAGGN

***** ** ** ** **
Consensus:

CCAGCaGGaGCaACaGGa

>Bacillus_Fam_754_18_2 Nr. of seq. 2 Alignment length(with gaps) = 18
Alignment score = 0.703704
GCF_001578185.1_ASM157818v1_genomic.fna_1:2941947-2942073 Satlength=127 Nr of Repeats=7 RepeatLength=18 seed=CTGGCTTTGA Num.seqs=7 Similarity=0.887125 0
CTGGCTTTGATTGTTCTG
Rev.of_GCF_001591445.1_ASM159144v1_genomic.fna_68:19027-19129 Satlength=103 Nr of Repeats=5 RepeatLength=18 seed=CCACAACGAC Num.seqs=4

Similarity=0.913580 1
GTGGCTTTGGTCGTTGTG

******** * *** **
Consensus:

cTGGCTTTGaTcGTTcTG

>Bacillus_Fam_755_18_2 Nr. of seq. 2 Alignment length(with gaps) = 18
Alignment score = 0.648148
GCF_001654695.1_ASM165469v1_genomic.fna_9:27-117 Satlength=91 Nr of Repeats=5 RepeatLength=18 seed=CTTGCTGTTT Num.seqs=5 Similarity=0.822222
0 CTTGCTGTTTTTGTTGTT
Rev.of_GCF_001889165.1_ASM188916v1_genomic.fna_1:1564134-1564404
Satlength=271 Nr of Repeats=15 RepeatLength=18 seed=AGCAAGCAAA
Num.seqs=15 Similarity=0.886420
0 CCTGCTGNTTTGCTTGCT

* ***** *** *** *
Consensus:

CcTGCTGtTTTgcTTGcT

>Bacillus_Fam_756_18_2 Nr. of seq. 2 Alignment length(with gaps) = 18
Alignment score = 0.629630
GCF_002019645.1_ASM201964v1_genomic.fna_1:3879414-3879792 Satlength=379
Nr of Repeats=16 RepeatLength=18 seed=GGAACTGGAT Num.seqs=13
Similarity=0.867047 0
GGAACTGGATTTGCTCCT
Rev.of_GCF_900111815.1_PRJEB17078_genomic.fna_1:557922-558030
Satlength=109 Nr of Repeats=6 RepeatLength=18 seed=CAAATCCGGA Num.seqs=6
Similarity=0.911111 13
GGATCCGGATTTGGTGTT

*** * ******* * *
Consensus:

GGAaCcGGATTTGcTccT

>Bacillus_Fam_757_16_2 Nr. of seq. 2 Alignment length(with gaps) = 16
Alignment score = 0.625000
GCF_000331575.1_ASM33157v1_genomic.fna_3:88190-88246 Satlength=57 Nr of Repeats=4 RepeatLength=14 seed=CTAATCAACC Num.seqs=4 Similarity=0.952381
0 CT-AA-TCAACCAACA
GCF_900111815.1_PRJEB17078_genomic.fna_1:149733-149838 Satlength=106 Nr of Repeats=7 RepeatLength=15 seed=GAACTCAACC Num.seqs=7
Similarity=0.923810
2 CTGAACTCAACCAA-A

** ** ******** *
Consensus:

CTgAAcTCAACCAAcA

>Bacillus_Fam_758_15_2 Nr. of seq. 2 Alignment length(with gaps) = 15
Alignment score = 0.688889
GCF_001591545.1_ASM159154v1_genomic.fna_1:1876062-1876140 Satlength=79 Nr of Repeats=6 RepeatLength=13 seed=TTATTGTTTT Num.seqs=6
Similarity=1.000000 0
TTATT--GTTTTTCC
Rev.of_GCF_001591665.1_ASM159166v1_genomic.fna_25:2727-2817 Satlength=91 Nr of Repeats=6 RepeatLength=15 seed=AAAACATAAT Num.seqs=6
Similarity=0.917037 12
TTATTATGTTTTTCG

***** *******
Consensus:

TTATTatGTTTTTCc

>Bacillus_Fam_759_15_2 Nr. of seq. 2 Alignment length(with gaps) = 15
Alignment score = 0.733333
BACILLUS_PSEUDALCALIPHILUS_genomic.fna_6:254208-254418 Satlength=211 Nr of Repeats=14 RepeatLength=15 seed=GGTGAACCCG Num.seqs=14
Similarity=0.940415 0
GGTGAACCCGGTGAT
GCF_000177235.2_ASM17723v2_genomic.fna_1:793103-793946 Satlength=844 Nr of Repeats=20 RepeatLength=15 seed=GAACCAGGAG Num.seqs=19
Similarity=0.916829 3
GGCGAACCAGGAGAT

** ***** ** ***
Consensus:

GGcGAACCaGGaGAT

>Bacillus_Fam_760_15_2 Nr. of seq. 2 Alignment length(with gaps) = 15
Alignment score = 0.666667
GCF_000299035.1_ASM29903v1_genomic.fna_1:1198325-1198505 Satlength=181 Nr of Repeats=9 RepeatLength=15 seed=CAAAAACCAG Num.seqs=7
Similarity=0.751984 0
CAAAAACCAGAGGAN
GCF_001591445.1_ASM159144v1_genomic.fna_34:41259-41319 Satlength=61 Nr of Repeats=4 RepeatLength=15 seed=GCTCCAAAAC Num.seqs=4 Similarity=0.851852
12   CCAAAACCAGGGGCT

* ******** **
Consensus:

CaAAAACCAGaGGat

>Bacillus_Fam_761_15_2 Nr. of seq. 2 Alignment length(with gaps) = 15
Alignment score = 0.733333
GCF_000474275.1_Bmar1.0_genomic.fna_33:30569-30884 Satlength=316 Nr of Repeats=5 RepeatLength=15 seed=TTCTTGTTCT Num.seqs=3 Similarity=0.940741
1   ATTCTTGTTCTTTGT
GCF_001636325.1_ASM163632v1_genomic.fna_1:2526805-2527021 Satlength=217 Nr of Repeats=7 RepeatLength=15 seed=CTTTTTCTTT Num.seqs=5

Similarity=0.822222 3
GTTCTTTTTCTTTTT

***** ****** *
Consensus:

TTCTTgTTCTTTgTa

>Bacillus_Fam_762_15_2 Nr. of seq. 2 Alignment length(with gaps) = 15
Alignment score = 1.000000
GCF_000508325.1_BAVI_1_genomic.fna_72:39782-39857 Satlength=76 Nr of Repeats=5 RepeatLength=15 seed=GGCATGATGT Num.seqs=5 Similarity=0.911111
0 GGCATGATGTGCGGC
GCF_001636315.1_ASM163631v1_genomic.fna_1:5617727-5617892 Satlength=166 Nr of Repeats=8 RepeatLength=15 seed=GGCATGATGT Num.seqs=5
Similarity=0.946667 0
GGCATGATGTGCGGC

****
Consensus:

GGCATGATGTGCGGC

>Bacillus_Fam_763_15_2 Nr. of seq. 2 Alignment length(with gaps) = 15
Alignment score = 0.644444
GCF_001590785.1_ASM159078v1_genomic.fna_1:386490-386730 Satlength=241 Nr of Repeats=16 RepeatLength=15 seed=CCAGGCAATG Num.seqs=16
Similarity=0.902963 0
CCAGGCAATGGCGAT
Rev.of_GCF_001591665.1_ASM159166v1_genomic.fna_3:104200-104350
Satlength=151 Nr of Repeats=9 RepeatLength=15 seed=CCGCCACCGC Num.seqs=8
Similarity=0.866667 14
CCTGGCGGTGGCGGT

** *** ***** *
Consensus:

CCaGGCaaTGGCGaT

>Bacillus_Fam_764_15_2 Nr. of seq. 2 Alignment length(with gaps) = 15
Alignment score = 0.844444
GCF_001591445.1_ASM159144v1_genomic.fna_9:114139-114289 Satlength=151 Nr of Repeats=10 RepeatLength=15 seed=GGCATTTCTT Num.seqs=10
Similarity=0.915062 0
GGCATTTCTTTGATC
Rev.of_GCF_001591485.1_ASM159148v1_genomic.fna_3:51079-51340
Satlength=262 Nr of Repeats=5 RepeatLength=15 seed=AAAGAAATAC Num.seqs=3
Similarity=0.733333 11
GGTATTTCTTTNATC

** ******** ***
Consensus:

GGcATTTCTTTgATC

>Bacillus_Fam_765_14_2 Nr. of seq. 2 Alignment length(with gaps) = 14
Alignment score = 0.738095
GCF_000948175.1_BFA_2_A_genomic.fna_151:142349-142433 Satlength=85 Nr of Repeats=7 RepeatLength=12 seed=TTCTCTTTTT Num.seqs=5 Similarity=0.844444
9   TCTTTTTTC--TTC
GCF_001645555.1_ASM164555v1_genomic.fna_6:7244-7314 Satlength=71 Nr of Repeats=5 RepeatLength=14 seed=TTCTTTTTTC Num.seqs=5 Similarity=1.000000
11   TCTTTTTTCTTTTT

** *********
Consensus:

TTcTCTTTTTTCtt

>Bacillus_Fam_766_14_2 Nr. of seq. 2 Alignment length(with gaps) = 14
Alignment score = 0.714286
GCF_000948175.1_BFA_2_A_genomic.fna_56:4896-4966 Satlength=71 Nr of Repeats=5 RepeatLength=14 seed=TCATTACATA Num.seqs=5 Similarity=1.000000
0   TCATTACATATCCC
Rev.of_GCF_001578185.1_ASM157818v1_genomic.fna_1:2027719-2027775 Satlength=57 Nr of Repeats=4 RepeatLength=14 seed=TAAGTGATAA Num.seqs=4 Similarity=0.888889   3
TTATTACTTATCAC

* ***** **** *
Consensus:

TcATTACaTATCaC

>Bacillus_Fam_767_12_2 Nr. of seq. 2 Alignment length(with gaps) = 12
Alignment score = 0.666667
GCF_000008505.1_ASM850v1_genomic.fna_1:985966-986182 Satlength=217 Nr of Repeats=17 RepeatLength=12 seed=GGAGGAGTAG Num.seqs=16 Similarity=0.905556
0   GGAGGAGTAGTT
GCF_000331575.1_ASM33157v1_genomic.fna_1:1528380-1528428 Satlength=49 Nr of Repeats=4 RepeatLength=12 seed=GGATGAGTTA Num.seqs=4 Similarity=0.944444
0   GGATGAGTTATT

*** **** **
Consensus:

GGAgGAGTaaTT

>Bacillus_Fam_768_12_2 Nr. of seq. 2 Alignment length(with gaps) = 12
Alignment score = 0.694444
GCF_000153365.1_ASM15336v1_genomic.fna_8:85710-85779 Satlength=70 Nr of Repeats=5 RepeatLength=12 seed=CCTGAGCCTG Num.seqs=4 Similarity=0.759259
0   CCTGAACCTGAG
GCF_000294775.2_ASM29477v2_genomic.fna_2:295544-295664 Satlength=121 Nr of Repeats=5 RepeatLength=12 seed=AGCCTAAGCC Num.seqs=3

Similarity=0.666667
4   CCTNAGCCTAAG

*** * *** **
Consensus:

CCTgAaCCTaAG

>Bacillus_Fam_769_12_2 Nr. of seq. 2 Alignment length(with gaps) = 12
Alignment score = 0.666667
GCF_000161455.1_ASM16145v1_genomic.fna_1:5124264-5124372 Satlength=109 Nr of Repeats=4 RepeatLength=12 seed=TGGTTTTTCT Num.seqs=3
Similarity=1.000000 0
TGGTTTTTCTGT
Rev.of_GCF_000474275.1_Bmar1.0_genomic.fna_3:52284-52332 Satlength=49 Nr of Repeats=4 RepeatLength=12 seed=AAAGAAATGC Num.seqs=4
Similarity=0.925926
0   TGGCATTTCTTT

*** ***** *
Consensus:

TGGcaTTTCTgT

>Bacillus_Fam_770_12_2 Nr. of seq. 2 Alignment length(with gaps) = 12
Alignment score = 0.805556
GCF_000334155.1_L1_genomic.fna_72:536-620 Satlength=85 Nr of Repeats=7 RepeatLength=12 seed=GATGCTGATG Num.seqs=7 Similarity=0.851852
0   GATGCTGATGCN
Rev.of_GCF_002019765.1_ASM201976v1_genomic.fna_1:3481582-3481660
Satlength=79 Nr of Repeats=5 RepeatLength=12 seed=GCATCCGCAT Num.seqs=3
Similarity=0.925926 5
GATGCCGATGCG

***** *****
Consensus:

GATGCcGATGCg

>Bacillus_Fam_771_12_2 Nr. of seq. 2 Alignment length(with gaps) = 12
Alignment score = 0.666667
GCF_000430785.1_ASM43078v1_genomic.fna_20:47240-47348 Satlength=109 Nr of Repeats=9 RepeatLength=12 seed=CCTGGAGGAA Num.seqs=9 Similarity=0.913580
0   CCTGGAGGAACA
Rev.of_GCF_900093775.1_EVONIK_BGLY_genomic.fna_1:4560190-4560334
Satlength=145 Nr of Repeats=12 RepeatLength=12 seed=GTTGTTCCAC
Num.seqs=12 Similarity=0.922559
2   ACAGGTGGAACA

* ** ******
Consensus:

aCaGGaGGAACA

>Bacillus_Fam_772_12_2 Nr. of seq. 2 Alignment length(with gaps) = 12
Alignment score = 0.777778
GCF_000986785.1_ASM98678v1_genomic.fna_40:25576-25696 Satlength=121 Nr of Repeats=8 RepeatLength=12 seed=TCATGGCTGT Num.seqs=6 Similarity=0.933333
0   TCATGGCTGTGA
GCF_002009555.1_ASM200955v1_genomic.fna_559:81-135 Satlength=55 Nr of Repeats=4 RepeatLength=12 seed=TGATCATGGT Num.seqs=3 Similarity=0.925926
9   TCATGGTCGTGA

****** ****
Consensus:

TCATGGccGTGA

>Bacillus_Fam_773_12_2 Nr. of seq. 2 Alignment length(with gaps) = 12
Alignment score = 0.777778
GCF_001273755.1_ASM127375v1_genomic.fna_17:3004884-3004968 Satlength=85 Nr of Repeats=6 RepeatLength=12 seed=TTGAACTGGT Num.seqs=5 Similarity=0.866667 0
TTGAACTGGTTG
Rev.of_GCF_001315045.1_ASM131504v1_genomic.fna_15:59765-60074 Satlength=310 Nr of Repeats=6 RepeatLength=12 seed=ATGCAACAAC Num.seqs=4 Similarity=0.925926 6
TTGCATTGGTTG

*** * ******
Consensus:

TTGaAcTGGTTG

>Bacillus_Fam_774_12_2 Nr. of seq. 2 Alignment length(with gaps) = 12
Alignment score = 0.777778
GCF_002019645.1_ASM201964v1_genomic.fna_1:1577470-1577590 Satlength=121 Nr of Repeats=10 RepeatLength=12 seed=CTGGAACAGA Num.seqs=10 Similarity=0.916049 0
CTGGAACAGACC
Rev.of_GCF_900104555.1_IMG-taxon_2651870172_annotated_assembly_genomic.fna_22:386175-386367 Satlength=193 Nr of Repeats=15 RepeatLength=12 seed=TCTGTTCCTG Num.seqs=14 Similarity=0.925519 10   CAGGAACAGATC

* ******** *
Consensus:

CaGGAACAGAcC

>Bacillus_Fam_775_11_2 Nr. of seq. 2 Alignment length(with gaps) = 11
Alignment score = 0.636364
GCF_000238675.1_Baci_smit_7_3_47FAA_V1_genomic.fna_4:38733-38800 Satlength=68 Nr of Repeats=4 RepeatLength=11 seed=GTTTCTATAT Num.seqs=3 Similarity=0.919192 0
GTTTCTATATG

Rev.of_GCF_000751775.1_A1A_genomic.fna_2:555-599 Satlength=45 Nr of Repeats=4 RepeatLength=11 seed=GAAACAATAC Num.seqs=4 Similarity=0.939394
5   GTTTCGGTATT

***** ***
Consensus:

GTTTCgaTATg

>Bacillus_Fam_776_321_1 Nr. of seq. 1 Alignment length(with gaps) = 321
Alignment score = 0.000000
GCF_000242895.2_ASM24289v3_genomic.fna_1:4312051-4313392 Satlength=1342
Nr of Repeats=5 RepeatLength=321 seed=TGAACGTTAC Num.seqs=4
Similarity=0.939772   0
TGAACGTTACGGTATGTCTTCCATCTCTAAATTTGGTTGTGTCGAGAGTATAATGAAAGCCACCATTCCCATT
GTGGTACTCTGGAAAGACTTTCTGGACATCTGGTCTCGCATCTCCATAGATCGCCTGACCTGCCACTACACCG
TCTACCAGTACTTCAATTTTCGACACTCCGCTTTCATCTAAAAACCAGCCCCGTACGTTTTGGGACCCGATAA
GTGTTTTGCCAGAAACCGGATCATCGATATACCCGAGGACATTTGCAATCGTTATAGTCTTTTCAGGCAATGT
TGTGACATAGCCGTTCATTCCTGTTTCTC

****
****
****
****
****
Consensus:

TGAACGTTACGGTATGTCTTCCATCTCTAAATTTGGTTGTGTCGAGAGTATAATGAAAGCCACCATTCCCATT
GTGGTACTCTGGAAAGACTTTCTGGACATCTGGTCTCGCATCTCCATAGATCGCCTGACCTGCCACTACACCG
TCTACCAGTACTTCAATTTTCGACACTCCGCTTTCATCTAAAAACCAGCCCCGTACGTTTTGGGACCCGATAA
GTGTTTTGCCAGAAACCGGATCATCGATATACCCGAGGACATTTGCAATCGTTATAGTCTTTTCAGGCAATGT
TGTGACATAGCCGTTCATTCCTGTTTCTC

>Bacillus_Fam_777_255_1 Nr. of seq. 1 Alignment length(with gaps) = 255
Alignment score = 0.000000
BACILLUS_PSEUDALCALIPHILUS_genomic.fna_16:50-1069 Satlength=1020 Nr of Repeats=4 RepeatLength=255 seed=AGTAGGACGT Num.seqs=3
Similarity=0.920573
0
AGTAGGACGTTGCCAGGCAGATTAAACACANTCCTTTAGGGATGTGTTTTTTAATATGCATAAAGAGTATACT
CGGCGCAAAAGAGCGCTGGTTCGAAGAAGCAGCCCCTACGAAGCGAAGCCCCGGAAAGCGATCGAAGGAAGAA
AACCAAGTACGTTCGCGAGACGAATCGGTTCTCTTCGTGCGACCTCGTGAGAGGCATCGGGGAAATCGCTACA
TGCCATACTTCCCCGAAGAAGAGAATCCGAAGAGAC

****
****
****
****
Consensus:

AGTAGGACGTTGCCAGGCAGATTAAACACANTCCTTTAGGGATGTGTTTTTTAATATGCATAAAGAGTATACT
CGGCGCAAAAGAGCGCTGGTTCGAAGAAGCAGCCCCTACGAAGCGAAGCCCCGGAAAGCGATCGAAGGAAGAA
AACCAAGTACGTTCGCGAGACGAATCGGTTCTCTTCGTGCGACCTCGTGAGAGGCATCGGGGAAATCGCTACA
TGCCATACTTCCCCGAAGAAGAGAATCCGAAGAGAC

>Bacillus_Fam_778_252_1 Nr. of seq. 1 Alignment length(with gaps) = 252
Alignment score = 0.000000
GCF_000169195.2_ASM16919v2_genomic.fna_1:915504-916512 Satlength=1009 Nr of Repeats=4 RepeatLength=252 seed=TTTTGCTTCG Num.seqs=4
Similarity=0.906526 0
TTTTGCTTCGATTTAATCCTGACTGAGAAGCTACCTTTACTACTTGCTGTCCCTGTCCCCAGCACCGTTTTAC
CACGTTTAATCGTCACTTTGGCGCCGGCTTCTGCTTTACCTGTAATCGTTGTTTGATTATCGCCAAACCGATT
GACGGACGGGGCTGATGGTGCTGTTTTGTCTTCCACTTTGAACGATTTGCCTGCACCCGTATTTCCAGCTTTA
TCTGTCGCATAGGCTGTCAGAACCGTTCCTGCC

****
****
****
****
Consensus:

TTTTGCTTCGATTTAATCCTGACTGAGAAGCTACCTTTACTACTTGCTGTCCCTGTCCCCAGCACCGTTTTAC
CACGTTTAATCGTCACTTTGGCGCCGGCTTCTGCTTTACCTGTAATCGTTGTTTGATTATCGCCAAACCGATT
GACGGACGGGGCTGATGGTGCTGTTTTGTCTTCCACTTTGAACGATTTGCCTGCACCCGTATTTCCAGCTTTA
TCTGTCGCATAGGCTGTCAGAACCGTTCCTGCC

>Bacillus_Fam_779_249_1 Nr. of seq. 1 Alignment length(with gaps) = 249
Alignment score = 0.000000
GCF_000832905.1_ASM83290v1_genomic.fna_1:2746746-2747991 Satlength=1246 Nr of Repeats=5 RepeatLength=249 seed=AAACAAAAAG Num.seqs=5
Similarity=0.824409 0
AAACAAAAAGCAGGAACGAAGCTTGTCGTTTATGCGGAAGATGCGGCAGGGAACAAGAGCGNTGAAACNGTNG
TAACGGTTATTGATAAAACGGCTCCGGCAGCGCCGAAAGTCGGAGAAGTAAGCGATACAAGTACTGTGGTTAC
AGGAACCACAGAAGCGGGCGCGAAAGTAACAGTAAAATCCGGCTCGAACATTTTAGGNACGGCAACGGCTGAT
CATACCGGTGCATTTAAGGTAACCATTGCA

****
****
****
****
Consensus:

AAACAAAAAGCAGGAACGAAGCTTGTCGTTTATGCGGAAGATGCGGCAGGGAACAAGAGCGNTGAAACNGTNG
TAACGGTTATTGATAAAACGGCTCCGGCAGCGCCGAAAGTCGGAGAAGTAAGCGATACAAGTACTGTGGTTAC
AGGAACCACAGAAGCGGGCGCGAAAGTAACAGTAAAATCCGGCTCGAACATTTTAGGNACGGCAACGGCTGAT
CATACCGGTGCATTTAAGGTAACCATTGCA

>Bacillus_Fam_780_246_1 Nr. of seq. 1 Alignment length(with gaps) = 246
Alignment score = 0.000000
GCF_001591445.1_ASM159144v1_genomic.fna_67:6172-7645 Satlength=1474 Nr of Repeats=5 RepeatLength=246 seed=GTTTTATCTA Num.seqs=3
Similarity=0.792231
0
GTTTTATCTACTTTTACTTCTACTGTGTTTTTATTTTCNGTATTACCNGCATTATCTACACTGTAGTAAGTTA
GAGTGTGGATGCCTTCTTTTTCTANGGTNAAGCTTGTGCCTTCGCTGAATTCAGCNCCATCGATAGAATAGTA
GGTTGCTTTCACACCGCTTAGGTCATCAGTTGCTGTCAGCTCAACATTCACTGCTTCTGTGTACCATTGATCC
TCGACATTACTTACAGTAGTAGGAGCA

****
****
****
****
Consensus:

GTTTTATCTACTTTTACTTCTACTGTGTTTTTATTTTCNGTATTACCNGCATTATCTACACTGTAGTAAGTTA
GAGTGTGGATGCCTTCTTTTTCTANGGTNAAGCTTGTGCCTTCGCTGAATTCAGCNCCATCGATAGAATAGTA
GGTTGCTTTCACACCGCTTAGGTCATCAGTTGCTGTCAGCTCAACATTCACTGCTTCTGTGTACCATTGATCC
TCGACATTACTTACAGTAGTAGGAGCA

>Bacillus_Fam_781_237_1 Nr. of seq. 1 Alignment length(with gaps) = 237
Alignment score = 0.000000
GCF_000374565.1_ASM37456v1_genomic.fna_37:21075-21996 Satlength=922 Nr of
Repeats=4 RepeatLength=237 seed=CCATTGGCTT Num.seqs=3
Similarity=0.889358
0
CCATTGGCTTTCACCCTTGCCCAGCCGTTCGATTCGGATATCACTGATACTGCCGTTCCGCTGGACAGCTTGC
TTATCACTTTTCCTTGTGTAGACGCCTTGTCTCGGACATTCAGCGAGGATCCAGAGTCGACTTTCACATATTT
GGTAACCGGNTTGGCCGACGGTTTTGGTGTACCTGTGCTGGCTGACGGCTTGCTTGATGACAAATATTGTGAA
CTTACGTAGCCTGTTTTG

****
****
****
****
Consensus:

CCATTGGCTTTCACCCTTGCCCAGCCGTTCGATTCGGATATCACTGATACTGCCGTTCCGCTGGACAGCTTGC
TTATCACTTTTCCTTGTGTAGACGCCTTGTCTCGGACATTCAGCGAGGATCCAGAGTCGACTTTCACATATTT
GGTAACCGGNTTGGCCGACGGTTTTGGTGTACCTGTGCTGGCTGACGGCTTGCTTGATGACAAATATTGTGAA
CTTACGTAGCCTGTTTTG

>Bacillus_Fam_782_237_1 Nr. of seq. 1 Alignment length(with gaps) = 237
Alignment score = 0.000000
GCF_001315065.1_ASM131506v1_genomic.fna_1:413-1597 Satlength=1185 Nr of
Repeats=5 RepeatLength=237 seed=TATACGTTTG Num.seqs=4
Similarity=0.781528
0
TATACGTTTGTTGGCTGGAACACCGAAGCGGATGGAAGTGGTGAAACCTATAAAGAGGGTGACACGTTTACCC
AAGGAATAGGAAATGTGACGTTCTATGCGCAGTGGTCTGTAAATCACTACGAGCTGCGATATGACGGGAACGG
GCAAGATGGAGGAAGTGCGCCAGAAGCGGAATCTGTTGCGTATGGAAGTGAAGTGGAGGTATCGGATTCTCAA
ACGTTAAGCCGAACCGGT

****
****
****
****
Consensus:

TATACGTTTGTTGGCTGGAACACCGAAGCGGATGGAAGTGGTGAAACCTATAAAGAGGGTGACACGTTTACCC
AAGGAATAGGAAATGTGACGTTCTATGCGCAGTGGTCTGTAAATCACTACGAGCTGCGATATGACGGGAACGG

GCAAGATGGAGGAAGTGCGCCAGAAGCGGAATCTGTTGCGTATGGAAGTGAAGTGGAGGTATCGGATTCTCAA
ACGTTAAGCCGAACCGGT

>Bacillus_Fam_783_234_1 Nr. of seq. 1 Alignment length(with gaps) = 234
Alignment score = 0.000000
GCF_000712615.1_ASM71261v1_genomic.fna_7:32135-33194 Satlength=1060 Nr of
Repeats=5 RepeatLength=234 seed=TTACTTTTTC Num.seqs=4
Similarity=0.888889
0
TTACTTTTTCTCCGTTTTTTAAATTTCCGAGAATTGCACTATTTGTATTTGCTTGACTTCTTACTCGCAGGCT
CGTTGCGTTGATTGTTCCGCTTTCTTGTACTCCATTTGTGATGTCTTCAACTGGCTTTTCTTCTTGTTTCCCA
GCTTCTAGCTTCACAAACTCTAACGATACGTAACCTTCTTTTCCTTTATAATTGATCTTCGCCCAGCCGTTTG
CTTTTCCTAAGATTG

****
****
****
****
Consensus:

TTACTTTTTCTCCGTTTTTTAAATTTCCGAGAATTGCACTATTTGTATTTGCTTGACTTCTTACTCGCAGGCT
CGTTGCGTTGATTGTTCCGCTTTCTTGTACTCCATTTGTGATGTCTTCAACTGGCTTTTCTTCTTGTTTCCCA
GCTTCTAGCTTCACAAACTCTAACGATACGTAACCTTCTTTTCCTTTATAATTGATCTTCGCCCAGCCGTTTG
CTTTTCCTAAGATTG

>Bacillus_Fam_784_232_1 Nr. of seq. 1 Alignment length(with gaps) = 232
Alignment score = 0.000000
GCF_000752035.1_Bacillus_rubiinfantis_genomic.fna_11:577590-580142
Satlength=2553 Nr of Repeats=11 RepeatLength=232 seed=TTCCATTTGA
Num.seqs=11 Similarity=0.994984 0
TTCCATTTGACTCCTCTGTGTTAGACGGAGAAATTCCGTTTATCTCGGGAAATACTACTATTTCCCTTGAATT
AAGGGAAGCTTTTCCGCTTATATGATCCAAATCCTTGGATTTTGCCTTTTTTGAAGGTAGTAAGCGGAATTTC
TACCCTTATATCTCCCCTTTAAGCCGCAAAATTTTCATTAGACGGAAATTCTCCGCTTATGCTTCTCCACCCT
ATCCTTTGGCAAC

****
****
****
****
Consensus:

TTCCATTTGACTCCTCTGTGTTAGACGGAGAAATTCCGTTTATCTCGGGAAATACTACTATTTCCCTTGAATT
AAGGGAAGCTTTTCCGCTTATATGATCCAAATCCTTGGATTTTGCCTTTTTTGAAGGTAGTAAGCGGAATTTC
TACCCTTATATCTCCCCTTTAAGCCGCAAAATTTTCATTAGACGGAAATTCTCCGCTTATGCTTCTCCACCCT
ATCCTTTGGCAAC

>Bacillus_Fam_785_231_1 Nr. of seq. 1 Alignment length(with gaps) = 231
Alignment score = 0.000000
GCF_000334155.1_L1_genomic.fna_143:18667-20053 Satlength=1387 Nr of
Repeats=6 RepeatLength=231 seed=GAAAAACTTG Num.seqs=6
Similarity=0.992304
0
GAAAAACTTGGTTGCCGCTAGATTTTCTGTAAATTGCCCTCTTTACTTGTCATTTGCTGTCTTTCCATTCGAA
GATAGCAGTTCAGCGGCAGCCTTAGCTTTTTCTTACCTCCCTTCAGCCCACGCTCTTTCACCCTTAATAATGG

TTGTTCAAGAAAAACAAGCCTGTTTTTCCACCTCTCTTTATGACAGGGAGGAATCACTTCTCTTTTTGCCTTC
AACCTTTATTTC

****
****
****
****
Consensus:

GAAAAACTTGGTTGCCGCTAGATTTTCTGTAAATTGCCCTCTTTACTTGTCATTTGCTGTCTTTCCATTCGAA
GATAGCAGTTCAGCGGCAGCCTTAGCTTTTTCTTACCTCCCTTCAGCCCACGCTCTTTCACCCTTAATAATGG
TTGTTCAAGAAAAACAAGCCTGTTTTTCCACCTCTCTTTATGACAGGGAGGAATCACTTCTCTTTTTGCCTTC
AACCTTTATTTC

>Bacillus_Fam_786_231_1 Nr. of seq. 1 Alignment length(with gaps) = 231
Alignment score = 0.000000
GCF_001273755.1_ASM127375v1_genomic.fna_17:4450455-4451425 Satlength=971
Nr of Repeats=5 RepeatLength=231 seed=GTTGTCGATA Num.seqs=4
Similarity=0.870130   0
GTTGTCGATAAATATTTAGTACTGACATATCCTTCTTTTCCATTAGCTTTGATCTTCGCCCAACCTTTAGATT
CCGAATACACCGTTACCTGCATGCCTTTCGAAAGCTTCGCAACTATGCTGGCAGTTTCCGTCCCACTTTTTCG
CATATTGAGCGTCCCAGAACTTACATTTACATATTTCGTCGTCGTTTTAACAGGGGTGGATGGTGTGGAACCC
GTTACAGGCTTC

****
****
****
****
Consensus:

GTTGTCGATAAATATTTAGTACTGACATATCCTTCTTTTCCATTAGCTTTGATCTTCGCCCAACCTTTAGATT
CCGAATACACCGTTACCTGCATGCCTTTCGAAAGCTTCGCAACTATGCTGGCAGTTTCCGTCCCACTTTTTCG
CATATTGAGCGTCCCAGAACTTACATTTACATATTTCGTCGTCGTTTTAACAGGGGTGGATGGTGTGGAACCC
GTTACAGGCTTC

>Bacillus_Fam_787_230_1 Nr. of seq. 1 Alignment length(with gaps) = 230
Alignment score = 0.000000
GCF_000334155.1_L1_genomic.fna_48:14925-15845 Satlength=921 Nr of
Repeats=4 RepeatLength=230 seed=GAACAACCAT Num.seqs=4
Similarity=0.993237
0
GAACAACCATTATGAAGGGTGAGAGAGGGAAAAGTTTGGGAAGTAAGAAAAGCTAAGGGCTCTGCCGAACTTC
CGTCTTTGAAGGGAGGAAAGAGGACTTCGAAAAGAATGCACTCTTTAAAAAGAATCTGGCAGAGCCCAAGCTT
TGAAACAGAGGTTTATAAGGTCTGAAAAAGAGCGCCCCTCCCCTATCATAAAGAGGGGAGGAAAAACAGCGGT
CTGTTTTTCTC

****
****
****
****
Consensus:

GAACAACCATTATGAAGGGTGAGAGAGGGAAAAGTTTGGGAAGTAAGAAAAGCTAAGGGCTCTGCCGAACTTC
CGTCTTTGAAGGGAGGAAAGAGGACTTCGAAAAGAATGCACTCTTTAAAAAGAATCTGGCAGAGCCCAAGCTT

TGAAACAGAGGTTTATAAGGTCTGAAAAAGAGCGCCCCTCCCCTATCATAAAGAGGGGAGGAAAAACAGCGGT
CTGTTTTTCTC

>Bacillus_Fam_788_226_1 Nr. of seq. 1 Alignment length(with gaps) = 226
Alignment score = 0.000000
GCF_000504145.1_SOAPdenovo_for_version2.04_of_Bacillus_sp._FJAT-14515_genome_genomic.fna_1:1737292-1738401 Satlength=1110 Nr of Repeats=5 RepeatLength=226 seed=ATTTACGTGC Num.seqs=3 Similarity=0.9724   0
ATTTACGTGCCGATTTGGCTTCTTTTCGGTCCGGAACGGAGCTCTTTCGTGCCGAAATCATCACTTTTCGAGC
GAGATTGGCGATTTTCGCGCCGGAGCTTAAATCTCTACGGTTCAACCACTTAATTACTATTTTTTTATGAACC
CCGTTGCTGTCCCAGTTATGTTAGCTATGGCACATCAGCTAATGCACATGCACAAACCATCATTACGGGCCGG
ATTTCAA

****
****
****
****
Consensus:

ATTTACGTGCCGATTTGGCTTCTTTTCGGTCCGGAACGGAGCTCTTTCGTGCCGAAATCATCACTTTTCGAGC
GAGATTGGCGATTTTCGCGCCGGAGCTTAAATCTCTACGGTTCAACCACTTAATTACTATTTTTTTATGAACC
CCGTTGCTGTCCCAGTTATGTTAGCTATGGCACATCAGCTAATGCACATGCACAAACCATCATTACGGGCCGG
ATTTCAA

>Bacillus_Fam_789_226_1 Nr. of seq. 1 Alignment length(with gaps) = 226
Alignment score = 0.000000
GCF_001274935.1_ASM127493v1_genomic.fna_27:538477-539381 Satlength=905 Nr of Repeats=4 RepeatLength=226 seed=GCTTCGCTTT Num.seqs=4
Similarity=0.853314 0
GCTTCGCTTTCCTCGGGGCGAGCGCTGAGCTTCCTCGTCGTTTCTCTCCTGCGGGATCTCACCTGTCTCGCTC
ATCCCGTAGGAGTCTTCTCATCTTTCACTGCCTTCCTTTGGTCTTTCGACTAGGAACTATTTAGTAGCCTGCT
TTTTGGAATGTGTATTTCAGATAAGCTATGTTCATTCCACATGAAACACCCTTTACTTAGTCGGATTCCAGCT
CCAGATT

****
****
****
****
Consensus:

GCTTCGCTTTCCTCGGGGCGAGCGCTGAGCTTCCTCGTCGTTTCTCTCCTGCGGGATCTCACCTGTCTCGCTC
ATCCCGTAGGAGTCTTCTCATCTTTCACTGCCTTCCTTTGGTCTTTCGACTAGGAACTATTTAGTAGCCTGCT
TTTTGGAATGTGTATTTCAGATAAGCTATGTTCATTCCACATGAAACACCCTTTACTTAGTCGGATTCCAGCT
CCAGATT

>Bacillus_Fam_790_225_1 Nr. of seq. 1 Alignment length(with gaps) = 225
Alignment score = 0.000000
GCF_000011145.1_ASM1114v1_genomic.fna_1:3777785-3778688 Satlength=904 Nr of Repeats=4 RepeatLength=225 seed=AAGCTTGATG Num.seqs=3
Similarity=0.854136 0
AAGCTTGATGAGGTTTTAAACGGGAACTCTAGACCTGCCACTCTTCGCTTTGGTGACCGTCATCCGTACGTCA
TTCAGCTTAAAAAAGATTTAGCTGAAGCAGGATTCCCGGTCTCCGGTAGCCCGACGGAGTATTTTGGATCAGT
CACCGAATCCCAAGTCAAGGCTTTCCAACGAGCTCACGGACTTACTGCTAATGGCGTTGTCGGATCTGCTACT
TACGCA

****
****
****
******
Consensus:

AAGCTTGATGAGGTTTTAAACGGGAACTCTAGACCTGCCACTCTTCGCTTTGGTGACCGTCATCCGTACGTCA
TTCAGCTTAAAAAAGATTTAGCTGAAGCAGGATTCCCGGTCTCCGGTAGCCCGACGGAGTATTTTGGATCAGT
CACCGAATCCCAAGTCAAGGCTTTCCAACGAGCTCACGGACTTACTGCTAATGGCGTTGTCGGATCTGCTACT
TACGCA

>Bacillus_Fam_791_222_1 Nr. of seq. 1 Alignment length(with gaps) = 222
Alignment score = 0.000000
GCF_001645705.1_ASM164570v1_genomic.fna_11:129111-130005 Satlength=895 Nr
of Repeats=4 RepeatLength=222 seed=TTATCCAAAC Num.seqs=3
Similarity=0.657698 0
TTATCCAAACTGATTAACCACGCAAAAACATATGAAAAGGATAAATATACCGAAACTTCATACGGNGCNCTGC
AACAAGCAATTAGCCATGCNGAAGCAGTGGTACAAAATGCGAANACACAAGATGAAGTAACGGAAGCNATTAG
TCNTCTCCAAAAAGCGATTAATGGATTAGAAAAAATAGCAGAACCAGAACCAGAANCTGAAGTAGATACGGGC
GAG

****
****
****
***
Consensus:

TTATCCAAACTGATTAACCACGCAAAAACATATGAAAAGGATAAATATACCGAAACTTCATACGGNGCNCTGC
AACAAGCAATTAGCCATGCNGAAGCAGTGGTACAAAATGCGAANACACAAGATGAAGTAACGGAAGCNATTAG
TCNTCTCCAAAAAGCGATTAATGGATTAGAAAAAATAGCAGAACCAGAACCAGAANCTGAAGTAGATACGGGC
GAG

>Bacillus_Fam_792_219_1 Nr. of seq. 1 Alignment length(with gaps) = 219
Alignment score = 0.000000
GCF_001484965.1_ASM148496v1_genomic.fna_12:79253-81209 Satlength=1957 Nr
of Repeats=8 RepeatLength=219 seed=GATGGGTTTC Num.seqs=5
Similarity=0.860578 0
GATGGGTTTCTGCGGAGTACTTAAATGTCAGTGGGTCGGGCAGCGATGCGAGTGAAGGGTCTTCCTCCATTGG
ATCGGCTACCACNACCGCAAGGCTTAACCTTCGCTCCGGTGCAGGGACTTCGAATCGTATTTTAACGACATTG
AACAACGGGCAGAACGTNGCCTTGCTTCAGAAACAAGGCAACTGGTATCAGGTTAAAGCCGGTTCTCAGACGG

****
****
****
Consensus:

GATGGGTTTCTGCGGAGTACTTAAATGTCAGTGGGTCGGGCAGCGATGCGAGTGAAGGGTCTTCCTCCATTGG
ATCGGCTACCACNACCGCAAGGCTTAACCTTCGCTCCGGTGCAGGGACTTCGAATCGTATTTTAACGACATTG
AACAACGGGCAGAACGTNGCCTTGCTTCAGAAACAAGGCAACTGGTATCAGGTTAAAGCCGGTTCTCAGACGG

>Bacillus_Fam_793_214_1 Nr. of seq. 1 Alignment length(with gaps) = 214
Alignment score = 0.000000

GCF_002019645.1_ASM201964v1_genomic.fna_1:4390066-4391018 Satlength=953 Nr of Repeats=5 RepeatLength=214 seed=TCAGGGTAGA Num.seqs=3 Similarity=1.000000 0
TCAGGGTAGACCTTTATGCGAATAAGTATTGGCACCATGGAAGATGAAAAAGTTTTATACTCAGTGTGGAATG
AGCAGAGAGTCACCTGACTCCTGCGGGATCTAGCGGTCTCGTGAGACCCCGCAGGAGCCAAAAGCGACGAGGA
GGCTCACGGGGCGCCCCGCGGAAAGCAGGTGGATCGCAGCGAATGGAACTCCACTATCTAAGTTATTT

****
****
****
Consensus:

TCAGGGTAGACCTTTATGCGAATAAGTATTGGCACCATGGAAGATGAAAAAGTTTTATACTCAGTGTGGAATG
AGCAGAGAGTCACCTGACTCCTGCGGGATCTAGCGGTCTCGTGAGACCCCGCAGGAGCCAAAAGCGACGAGGA
GGCTCACGGGGCGCCCCGCGGAAAGCAGGTGGATCGCAGCGAATGGAACTCCACTATCTAAGTTATTT

>Bacillus_Fam_794_213_1 Nr. of seq. 1 Alignment length(with gaps) = 213 Alignment score = 0.000000
GCF_000009825.1_ASM982v1_genomic.fna_1:3219845-3221123 Satlength=1279 Nr of Repeats=6 RepeatLength=213 seed=CGCCTTAACT Num.seqs=6 Similarity=0.931142 0
CGCCTTAACTTGCGTTCTGGAGCAGGCACTAACCATAGCATTATCACTACCCTTGCTAAAGGACAAAAAGTCG
AGTTGCTTAAAAAGCAAGGTGGCTGGTACCAAGTTAAAGCGGGCAACCGTACTGGATGGGTTTCTGTTGACTA
CTTAAATGTCAGCGGCTCTGGCAATGTGGACAACGCTCCTTCTAACGGCTCTGCCACAACCACTGCA

****
****
****
Consensus:

CGCCTTAACTTGCGTTCTGGAGCAGGCACTAACCATAGCATTATCACTACCCTTGCTAAAGGACAAAAAGTCG
AGTTGCTTAAAAAGCAAGGTGGCTGGTACCAAGTTAAAGCGGGCAACCGTACTGGATGGGTTTCTGTTGACTA
CTTAAATGTCAGCGGCTCTGGCAATGTGGACAACGCTCCTTCTAACGGCTCTGCCACAACCACTGCA

>Bacillus_Fam_795_213_1 Nr. of seq. 1 Alignment length(with gaps) = 213 Alignment score = 0.000000
GCF_000177235.2_ASM17723v2_genomic.fna_1:414216-415493 Satlength=1278 Nr of Repeats=6 RepeatLength=213 seed=CCACCAGAAA Num.seqs=5 Similarity=0.785426 0
CCACCAGAAAAAGAAGGCCATTCATTCAAAGGATGGTATCAAGATGAGTTGCTCACAGAAGCGTGGGATTTCG
AAACGAACACGGTTAGCGAAGATATGACCCTGTATGCAAAGTGGGAAATCAACGTTTATACTGTTAGTTTTGA
ATCGAATGGNGGCAGCCAAGTATCGGAAGTAGAAGCCGAGTATGGTTCGTCNATCACAGAGCCAGCT

****
****
****
Consensus:

CCACCAGAAAAAGAAGGCCATTCATTCAAAGGATGGTATCAAGATGAGTTGCTCACAGAAGCGTGGGATTTCG
AAACGAACACGGTTAGCGAAGATATGACCCTGTATGCAAAGTGGGAAATCAACGTTTATACTGTTAGTTTTGA
ATCGAATGGNGGCAGCCAAGTATCGGAAGTAGAAGCCGAGTATGGTTCGTCNATCACAGAGCCAGCT

>Bacillus_Fam_796_210_1 Nr. of seq. 1 Alignment length(with gaps) = 210 Alignment score = 0.000000

GCF_900107275.1_IMG-
taxon_2675903024_annotated_assembly_genomic.fna_41:390-1233 Satlength=844
Nr of Repeats=4 RepeatLength=210 seed=GACAATCCGA Num.seqs=3
Similarity=0.763693 0
GACAATCCGAATGCTGNTTACGGTCCAACGACGACAACTCGGGTGAAGGAATTCCAGAAATATTATGGATTAG
TGGTCAACGGCATTGGTGATGAAGTAACACTTGCTAAAATTGAAGAGATTTTAGCATCNCCGCTCCAAAACGG
AGGCAGGCATGAAGACGTGATAGCGTTAAAGGAAAACTTNTCTCGCCTCGGTTTCCATGTATCG

****
****
****
Consensus:

GACAATCCGAATGCTGNTTACGGTCCAACGACGACAACTCGGGTGAAGGAATTCCAGAAATATTATGGATTAG
TGGTCAACGGCATTGGTGATGAAGTAACACTTGCTAAAATTGAAGAGATTTTAGCATCNCCGCTCCAAAACGG
AGGCAGGCATGAAGACGTGATAGCGTTAAAGGAAAACTTNTCTCGCCTCGGTTTCCATGTATCG

>Bacillus_Fam_797_208_1 Nr. of seq. 1 Alignment length(with gaps) = 208
Alignment score = 0.000000
GCF_000334155.1_L1_genomic.fna_10:13608-14440 Satlength=833 Nr of
Repeats=4 RepeatLength=208 seed=AGGGTGAGAG Num.seqs=4
Similarity=0.983974
0
AGGGTGAGAGAGGGAGGAGTTTGGGAGGTAAGAAAAACTAAGGCCTCCGCCGATCTGCCGTCTTTGGTAAAGG
GAATCGGACAGTTTTTAGGAGTGCTCTTCTTAACGGGATACAGGCAAGGTAGTGACGCTGAAAAACATCCCGA
TCCGCCCTGTCATAAAGAGCGGTGGAAAAGCAAGCTTGCTTTTCATGATTAAACTTTATGAC

****
****
****
Consensus:

AGGGTGAGAGAGGGAGGAGTTTGGGAGGTAAGAAAAACTAAGGCCTCCGCCGATCTGCCGTCTTTGGTAAAGG
GAATCGGACAGTTTTTAGGAGTGCTCTTCTTAACGGGATACAGGCAAGGTAGTGACGCTGAAAAACATCCCGA
TCCGCCCTGTCATAAAGAGCGGTGGAAAAGCAAGCTTGCTTTTCATGATTAAACTTTATGAC

>Bacillus_Fam_798_206_1 Nr. of seq. 1 Alignment length(with gaps) = 206
Alignment score = 0.000000
GCF_001274775.1_ASM127477v1_genomic.fna_6:313004-313829 Satlength=826 Nr
of Repeats=4 RepeatLength=206 seed=TCGTGCCTGC Num.seqs=3
Similarity=0.961165 0
TCGTGCCTGCGGGGTCTCGGTCATCCCTCTTTTCCGAAGGAGTCCGGGCCCCTTCGCTTCTTTTCACTCTTGG
TGATACTTTGATTCCCTTTTTTCATTCATTGTTTCCGCCTAGTTCGTTTTTGGTTCTGCTTCATTGGTGTGAT
GGATTGGGCGTTCCATCCCGCTGTGGGACCGGCTTTTCTTGGGAGGGCGCCCGAGCCTCT

****
****
****
Consensus:

TCGTGCCTGCGGGGTCTCGGTCATCCCTCTTTTCCGAAGGAGTCCGGGCCCCTTCGCTTCTTTTCACTCTTGG
TGATACTTTGATTCCCTTTTTTCATTCATTGTTTCCGCCTAGTTCGTTTTTGGTTCTGCTTCATTGGTGTGAT
GGATTGGGCGTTCCATCCCGCTGTGGGACCGGCTTTTCTTGGGAGGGCGCCCGAGCCTCT

>Bacillus_Fam_799_206_1 Nr. of seq. 1 Alignment length(with gaps) = 206
Alignment score = 0.000000
GCF_001375535.1_Bacillus_niameyensis_genomic.fna_6:523574-525781
Satlength=2208 Nr of Repeats=11 RepeatLength=206 seed=AAAGAAAAGC
Num.seqs=10 Similarity=0.998706 0
AAAGAAAAGCGTAAACGCCCGTTTAGCGACGTACAAACTTTAATAATAAGGATGAACGGCTAAAGTCGCGCCG
TCCTGGCGCAACGCCGTTCTGACCAACGTCGTGTTGGCCCGAGATAAAGGAAACACAGCGAGGGACGAGCTGA
TGTTGACTTATCGTAGGAAGAAGCTGAAAGTTTGCTAGTCGTTGGGCGTTGAAGCTGGAC

****
****
****
Consensus:

AAAGAAAAGCGTAAACGCCCGTTTAGCGACGTACAAACTTTAATAATAAGGATGAACGGCTAAAGTCGCGCCG
TCCTGGCGCAACGCCGTTCTGACCAACGTCGTGTTGGCCCGAGATAAAGGAAACACAGCGAGGGACGAGCTGA
TGTTGACTTATCGTAGGAAGAAGCTGAAAGTTTGCTAGTCGTTGGGCGTTGAAGCTGGAC

>Bacillus_Fam_800_205_1 Nr. of seq. 1 Alignment length(with gaps) = 205
Alignment score = 0.000000
GCF_001645685.2_ASM164568v2_genomic.fna_1:959082-960714 Satlength=1633 Nr
of Repeats=8 RepeatLength=204 seed=AAAGTGGAAG Num.seqs=8
Similarity=0.770301 0
AAAGTGGAAGATCTGTTCACGAATGATAAGTTCGACACATTGAAAGGAAGTACCGATCAAAGNGCGATTGATG
AAGCTCAAAAAGCNGTCAATAAACTTCCGGAAGGTCCAGAAAAAAAATCGNCTTGAANGATTTAGTGAACAAAG
CACAAGATCTATTAAATAAAAAAAAACAAGCCGAAAAAGACTTGAACGATGCAAAGAAC

****
****
****
Consensus:

AAAGTGGAAGATCTGTTCACGAATGATAAGTTCGACACATTGAAAGGAAGTACCGATCAAAGNGCGATTGATG
AAGCTCAAAAAGCNGTCAATAAACTTCCGGAAGGTCCAGAAAAAAAATCGNCTTGAANGATTTAGTGAACAAAG
CACAAGATCTATTAAATAAAAAAAAACAAGCCGAAAAAGACTTGAACGATGCAAAGAAC

>Bacillus_Fam_801_196_1 Nr. of seq. 1 Alignment length(with gaps) = 196
Alignment score = 0.000000
GCF_002019605.1_ASM201960v1_genomic.fna_1:4043384-4044164 Satlength=781
Nr of Repeats=4 RepeatLength=195 seed=CGAATTAATA Num.seqs=4
Similarity=0.670875 0
CGAATTAATACGTGGATTGGACCGAAGTGGATCAAACCAAACAATCCACTAATAGGGGAACCAACTCCGATCT
CAACGAGGATNCACGATAACAGAAAATACCTATTTGCATAATCAACCGTGGAATAGTGCTAGAAGAAATGAAT
TTTTACGTCCGCAAACAGTGACAGCATTTGAAGAATGGAATGGATGGTAC

****
****
****
Consensus:

CGAATTAATACGTGGATTGGACCGAAGTGGATCAAACCAAACAATCCACTAATAGGGGAACCAACTCCGATCT
CAACGAGGATNCACGATAACAGAAAATACCTATTTGCATAATCAACCGTGGAATAGTGCTAGAAGAAATGAAT
TTTTACGTCCGCAAACAGTGACAGCATTTGAAGAATGGAATGGATGGTAC

>Bacillus_Fam_802_195_1 Nr. of seq. 1 Alignment length(with gaps) = 195
Alignment score = 0.000000
GCF_001274775.1_ASM127477v1_genomic.fna_6:301298-302231 Satlength=934 Nr of Repeats=5 RepeatLength=195 seed=TCCATCACCA Num.seqs=3
Similarity=0.945299 0
TCCATCACCACAAAGTGCAATGCAGCGGAGACCGGCCGACTCCTTCGGGAATAGAGTGAAGTTCGATACCCCG
GACGCGCAGCTGAGGAGGCTCGACTCACTCCCCGAGGAAAGCGGACGGCCGCAGCGAAATGGAACGGACCTTG
ACTCCACCAAGATCCTCATCTAAAGCAGAAAGACATCATGCCTACCTCC

****
****
****
Consensus:

TCCATCACCACAAAGTGCAATGCAGCGGAGACCGGCCGACTCCTTCGGGAATAGAGTGAAGTTCGATACCCCG
GACGCGCAGCTGAGGAGGCTCGACTCACTCCCCGAGGAAAGCGGACGGCCGCAGCGAAATGGAACGGACCTTG
ACTCCACCAAGATCCTCATCTAAAGCAGAAAGACATCATGCCTACCTCC

>Bacillus_Fam_803_195_1 Nr. of seq. 1 Alignment length(with gaps) = 195
Alignment score = 0.000000
GCF_002019605.1_ASM201960v1_genomic.fna_1:4034033-4035008 Satlength=976 Nr of Repeats=5 RepeatLength=195 seed=ATAATCAACC Num.seqs=5
Similarity=0.628407 0
ATAATCAACCATTTGCNAATACAAGAAGATCAGAAGTNTTACGTCCNCAAACGGTACAGGCAACAGGAGAATG
GAATGGCTGGTACCGAATTAATACATGGGTAGGACCAAAGTGGATTAAACCGTCGAATGCGATTGTAGGAGAA
ATTAAANAAATCTCGCAAANTATAACATTAACGCAAAATACGCATTTGC

****
****
****
Consensus:

ATAATCAACCATTTGCNAATACAAGAAGATCAGAAGTNTTACGTCCNCAAACGGTACAGGCAACAGGAGAATG
GAATGGCTGGTACCGAATTAATACATGGGTAGGACCAAAGTGGATTAAACCGTCGAATGCGATTGTAGGAGAA
ATTAAANAAATCTCGCAAANTATAACATTAACGCAAAATACGCATTTGC

>Bacillus_Fam_804_194_1 Nr. of seq. 1 Alignment length(with gaps) = 194
Alignment score = 0.000000
GCF_000292245.2_ASM29224v2_genomic.fna_61:22691-23863 Satlength=1173 Nr of Repeats=6 RepeatLength=195 seed=ACGTCGAATA Num.seqs=5
Similarity=0.575845
0
ACGTCGAATACACTAAGNCCGTTCCACTCGGATTGAGTTTGCTTATAAACACATCAAANTTCCGTTANAACTT
TGATCATATGCTCCNGCGGTCGTTGGGAAATTCGNTGATGTTGTATANCCNGTCACATACGCATTTCCATCCT
CATCGANNGCAATTCCATNTCCTNNATCANNATCACTTCCNCCTAAAT

****
****
****
Consensus:

ACGTCGAATACACTAAGNCCGTTCCACTCGGATTGAGTTTGCTTATAAACACATCAAANTTCCGTTANAACTT
TGATCATATGCTCCNGCGGTCGTTGGGAAATTCGNTGATGTTGTATANCCNGTCACATACGCATTTCCATCCT
CATCGANNGCAATTCCATNTCCTNNATCANNATCACTTCCNCCTAAAT

>Bacillus_Fam_805_190_1 Nr. of seq. 1 Alignment length(with gaps) = 190 Alignment score = 0.000000
GCF_001636425.1_ASM163642v1_genomic.fna_1:2048397-2049156 Satlength=760 Nr of Repeats=4 RepeatLength=190 seed=TTAAATCAAA Num.seqs=3
Similarity=0.840023 0
TTAAATCAAAATTGGGATTGTACATAGANATANGCAGTGTTCTAATCATCCTGCTGGGTTGATTTCCGTTGCA
GGCGCTCGCTTTCCGAGGCCTGCCGGGAGTCTCATCATAGTAAGCTCCTTCGGGGTTTCCATAAAGCCAGCTG
ATCCCGCAGGAGTCTCGCACCTTCCGCTCCAATCAACCTTTGAA

****
****
****
Consensus:

TTAAATCAAAATTGGGATTGTACATAGANATANGCAGTGTTCTAATCATCCTGCTGGGTTGATTTCCGTTGCA
GGCGCTCGCTTTCCGAGGCCTGCCGGGAGTCTCATCATAGTAAGCTCCTTCGGGGTTTCCATAAAGCCAGCTG
ATCCCGCAGGAGTCTCGCACCTTCCGCTCCAATCAACCTTTGAA

>Bacillus_Fam_806_177_1 Nr. of seq. 1 Alignment length(with gaps) = 177 Alignment score = 0.000000
GCF_001636345.1_ASM163634v1_genomic.fna_1:572602-573664 Satlength=1063 Nr of Repeats=6 RepeatLength=177 seed=CAGAAACAAC Num.seqs=6
Similarity=0.773007 0
CAGAAACAACAAATGCTGAAGTAGTAGCAAAAACAGAAGCCATCCAAAAAGCACTAGCAGGCTTAGAATTTGC
AGGCCAAACAGCCTTAAATGCAGCAAAAGCAAAAGCTGAGGAAAAGCAAGAAGCCGACTACACATCTGAAAGC
TATAGCCCATTAAAAGCAGCATTAGAACTGC

****
****
****
Consensus:

CAGAAACAACAAATGCTGAAGTAGTAGCAAAAACAGAAGCCATCCAAAAAGCACTAGCAGGCTTAGAATTTGC
AGGCCAAACAGCCTTAAATGCAGCAAAAGCAAAAGCTGAGGAAAAGCAAGAAGCCGACTACACATCTGAAAGC
TATAGCCCATTAAAAGCAGCATTAGAACTGC

>Bacillus_Fam_807_172_1 Nr. of seq. 1 Alignment length(with gaps) = 172 Alignment score = 0.000000
GCF_000712595.1_ASM71259v1_genomic.fna_6:82276-83300 Satlength=1025 Nr of Repeats=6 RepeatLength=172 seed=TTACATATGG Num.seqs=5
Similarity=0.886364
0
TTACATATGGCTAGAGTAAAAGCTTTGTCGCTCTTTTACTTCGTCCATCATCGTTTGAATTTTTTGATCGTGT
AAAGCTTCGGTTTCAGAAAGCTTATCTTTTTGAATCGTAATTTTAAAAGAAAAAATTTGAAATATCATAATGT
AATCGCTCCTTTAGGTTGTTTTCGTA

****
****
****
Consensus:

TTACATATGGCTAGAGTAAAAGCTTTGTCGCTCTTTTACTTCGTCCATCATCGTTTGAATTTTTTGATCGTGT

AAAGCTTCGGTTTCAGAAAGCTTATCTTTTTGAATCGTAATTTTAAAAGAAAAAATTTGAAATATCATAATGT
AATCGCTCCTTTAGGTTGTTTTCGTA

>Bacillus_Fam_808_171_1 Nr. of seq. 1 Alignment length(with gaps) = 171
Alignment score = 0.000000
GCF_001274935.1_ASM127493v1_genomic.fna_19:74009-74687 Satlength=679 Nr
of Repeats=4 RepeatLength=171 seed=ATGTTGTTCC Num.seqs=3
Similarity=0.849253
0
ATGTTGTTCCACGATTGAAGTTGCGCTACGGTTACGCCGAATTTCACTGCAATTCCACTAAGTGTATCACCTG
ATTTAACGGTGTACGTGGATACGGTTGAATCGCCACCACCACTCGTAGGAGCTTTAACTTTCAAAACTTGTCC
AATCTTAATCACATCAGCGTTTGAG

****
****
****
Consensus:

ATGTTGTTCCACGATTGAAGTTGCGCTACGGTTACGCCGAATTTCACTGCAATTCCACTAAGTGTATCACCTG
ATTTAACGGTGTACGTGGATACGGTTGAATCGCCACCACCACTCGTAGGAGCTTTAACTTTCAAAACTTGTCC
AATCTTAATCACATCAGCGTTTGAG

>Bacillus_Fam_809_168_1 Nr. of seq. 1 Alignment length(with gaps) = 168
Alignment score = 0.000000
GCF_000504145.1_SOAPdenovo_for_version2.04_of_Bacillus_sp._FJAT-
14515_genome_genomic.fna_1:2021519-2027779 Satlength=6261 Nr of
Repeats=38 RepeatLength=168 seed=TCTACCAATT Num.seqs=36 Similarity=0.77
0
TCTACCAATTTTAATNCCGAAAGTTCTTCCATCANTTCTTGATGTGTTTGTTTTAATTGTTCAAGTTCTTGGT
ACTGACTTTGTTCTTTCAANGTAGCNNCCGATAACTGTTCGAGTACTTCTTCATATGATTGNTTTAATTTTTC
TAGTTCTTCGCTTTGGACTTGT

****
****
****
Consensus:

TCTACCAATTTTAATNCCGAAAGTTCTTCCATCANTTCTTGATGTGTTTGTTTTAATTGTTCAAGTTCTTGGT
ACTGACTTTGTTCTTTCAANGTAGCNNCCGATAACTGTTCGAGTACTTCTTCATATGATTGNTTTAATTTTTC
TAGTTCTTCGCTTTGGACTTGT

>Bacillus_Fam_810_164_1 Nr. of seq. 1 Alignment length(with gaps) = 164
Alignment score = 0.000000
GCF_000177235.2_ASM17723v2_genomic.fna_1:4304399-4305217 Satlength=819 Nr
of Repeats=5 RepeatLength=164 seed=AACAGGGACG Num.seqs=3
Similarity=0.917172 0
AACAGGGACGGTTCTCACTTGCCAGTTTGATAGTTTTTTTATGATTTGGTCATAGTTATGTGCGATTTCTTTC
AAGTTACACGCGATTTTGATCGAGTTACGCGCGATTTCACACGAGTTTGAAGCGATTTGGAAACCAACTGGAA
AAACACTTCAATTAGGCT

****
****
****
Consensus:

AACAGGGACGGTTCTCACTTGCCAGTTTGATAGTTTTTTATGATTTGGTCATAGTTATGTGCGATTTCTTTC
AAGTTACACGCGATTTTGATCGAGTTACGCGCGATTTCACACGAGTTTGAAGCGATTTGGAAACCAACTGGAA
AAACACTTCAATTAGGCT

>Bacillus_Fam_811_164_1 Nr. of seq. 1 Alignment length(with gaps) = 164
Alignment score = 0.000000
GCF_001273755.1_ASM127375v1_genomic.fna_16:30431-31253 Satlength=823 Nr of Repeats=5 RepeatLength=164 seed=GGTCACATTC Num.seqs=4
Similarity=0.967480
0
GGTCACATTCCATGAGGTAAGATTCAGCTTGTTCAGTGATTTGATCACCGCACTCGGGACATTGTTTAGGTGG
TAGGGTTCTAAAGAACTCCAACGGATTTTTAGTTTGCATGATGGTTTCCTCCTTTAAATGGTTTAGTAGTCTT
CGCGTTTAGAAAGGCAGT

****
****
****
Consensus:

GGTCACATTCCATGAGGTAAGATTCAGCTTGTTCAGTGATTTGATCACCGCACTCGGGACATTGTTTAGGTGG
TAGGGTTCTAAAGAACTCCAACGGATTTTTAGTTTGCATGATGGTTTCCTCCTTTAAATGGTTTAGTAGTCTT
CGCGTTTAGAAAGGCAGT

>Bacillus_Fam_812_156_1 Nr. of seq. 1 Alignment length(with gaps) = 156
Alignment score = 0.000000
GCF_000504145.1_SOAPdenovo_for_version2.04_of_Bacillus_sp._FJAT-14515_genome_genomic.fna_4:22903-23527 Satlength=625 Nr of Repeats=4
RepeatLength=156 seed=ATAAAGTGAA Num.seqs=4 Similarity=0.968661 0
ATAAAGTGAATCTTCCATCAGTGGAGGGTTTGCTTCATCCTCCACTGATGGTTAGTTGAACCAATCGGGGTTT
TACAGGCTGTTTTATCACTACACACTTCTCTCTATTTCTCCAACGAAAATGAAAGAGGGATTATTACAGCCTG
TTAAACCGGG

****
****
****
Consensus:

ATAAAGTGAATCTTCCATCAGTGGAGGGTTTGCTTCATCCTCCACTGATGGTTAGTTGAACCAATCGGGGTTT
TACAGGCTGTTTTATCACTACACACTTCTCTCTATTTCTCCAACGAAAATGAAAGAGGGATTATTACAGCCTG
TTAAACCGGG

>Bacillus_Fam_813_156_1 Nr. of seq. 1 Alignment length(with gaps) = 156
Alignment score = 0.000000
GCF_001591585.1_ASM159158v1_genomic.fna_12:122004-122892 Satlength=889 Nr of Repeats=6 RepeatLength=156 seed=TTCTTTCTTA Num.seqs=5
Similarity=0.862420 0
TTCTTTCTTAATAGTATTTTTAAGAATTAACAGAAGAACCAGCTTGCGTGACTACTTCCTTTACATTGAATGC
TTTTAGAGGATTTTGCTTTTAGTTGTACATCTTTTGATACAGCTTCTTTTTGTAAAGATAATACTTTGTTCAT
TTCTCATTCC

****
****
****

Consensus:

TTCTTTCTTAATAGTATTTTTAAGAATTAACAGAAGAACCAGCTTGCGTGACTACTTCCTTTACATTGAATGC
TTTTAGAGGATTTTGCTTTTAGTTGTACATCTTTTGATACAGCTTCTTTTTGTAAAGATAATACTTTGTTCAT
TTCTCATTCC

>Bacillus_Fam_814_156_1 Nr. of seq. 1 Alignment length(with gaps) = 156
Alignment score = 0.000000
GCF_001887185.1_ASM188718v1_genomic.fna_192:71627-72199 Satlength=573 Nr
of Repeats=5 RepeatLength=156 seed=ATTTTGACTG Num.seqs=3
Similarity=0.723270 0
ATTTTGACTGGGGGCGGGAACCTAAGGGTGCGGTTGATTACCGAGAAGGAAGAAATTCCGGGGGTTCGGGAAC
CTAAGGGAGGGTTTGGTTACCGGGATGGAGAAAAATGNCTGTGNTCGGTAACTAAAGAGTGTGGTTGGTTACC
CTGGTGGGGA

****
****
****
Consensus:

ATTTTGACTGGGGGCGGGAACCTAAGGGTGCGGTTGATTACCGAGAAGGAAGAAATTCCGGGGGTTCGGGAAC
CTAAGGGAGGGTTTGGTTACCGGGATGGAGAAAAATGNCTGTGNTCGGTAACTAAAGAGTGTGGTTGGTTACC
CTGGTGGGGA

>Bacillus_Fam_815_156_1 Nr. of seq. 1 Alignment length(with gaps) = 156
Alignment score = 0.000000
GCF_002019595.1_ASM201959v1_genomic.fna_1:3715909-3716689 Satlength=781
Nr of Repeats=6 RepeatLength=156 seed=AAAAAGGACT Num.seqs=4
Similarity=0.764241 0
AAAAAGGACTGCCTGGCACTGGAGCAAACCCGCCTGAACCATTACCTAAAAAAACCGAGACATCATCGGAACC
AAAGTTCACGACCGCAAGATCCATAATCCCGTCTCCATTAAAATCTGCAGACGTGATGGAANTTGGACCCAAA
CCTCCACTAG

****
****
****
Consensus:

AAAAAGGACTGCCTGGCACTGGAGCAAACCCGCCTGAACCATTACCTAAAAAAACCGAGACATCATCGGAACC
AAAGTTCACGACCGCAAGATCCATAATCCCGTCTCCATTAAAATCTGCAGACGTGATGGAANTTGGACCCAAA
CCTCCACTAG

>Bacillus_Fam_816_155_1 Nr. of seq. 1 Alignment length(with gaps) = 155
Alignment score = 0.000000
GCF_000299035.1_ASM29903v1_genomic.fna_2:237491-238103 Satlength=613 Nr
of Repeats=4 RepeatLength=153 seed=CGCTTGCCAT Num.seqs=4
Similarity=0.618896
0
CGCTTGCCATTCCGGACCACCACGTTTCTCCCAAATAGCAGCATAACGATCCTGACCATTACTACCGTAACCA
CTTACNTGCATATCAACCGATAGCCTTGCGCAACCAGTTCATTGAACGTCCGCTGATATTGCTCTGCTGTCAA
ACCATGTCG

****

****
****
Consensus:

CGCTTGCCATTCCGGACCACCACGTTTCTCCCAAATAGCAGCATAACGATCCTGACCATTACTACCGTAACCA
CTTACNTGCATATCAACCGATAGCCTTGCGCAACCAGTTCATTGAACGTCCGCTGATATTGCTCTGCTGTCAA
ACCATGTCG

>Bacillus_Fam_817_155_1 Nr. of seq. 1 Alignment length(with gaps) = 155
Alignment score = 0.000000
GCF_000331575.1_ASM33157v1_genomic.fna_1:755995-757235 Satlength=1241 Nr
of Repeats=8 RepeatLength=155 seed=TGCTCTACCG Num.seqs=8
Similarity=0.986645 0
TGCTCTACCGACTGAGCTATCGAGCCATAATAATATTTAACTTACAACTTTATACTCCACTTGCAAAACGTCC
TCTGTCCCTTCCTCTTCTAGCTTATTCTTTGATGCTTATGCGTCGGGACAACTCGCAGCTGATTCGCTGATGT
GGTGCTCGT

****
****
****
Consensus:

TGCTCTACCGACTGAGCTATCGAGCCATAATAATATTTAACTTACAACTTTATACTCCACTTGCAAAACGTCC
TCTGTCCCTTCCTCTTCTAGCTTATTCTTTGATGCTTATGCGTCGGGACAACTCGCAGCTGATTCGCTGATGT
GGTGCTCGT

>Bacillus_Fam_818_155_1 Nr. of seq. 1 Alignment length(with gaps) = 155
Alignment score = 0.000000
GCF_900156865.1_PRJEB18960_genomic.fna_3:4026577-4027197 Satlength=621 Nr
of Repeats=4 RepeatLength=155 seed=TTTGTTGTAA Num.seqs=4
Similarity=0.853763 0
TTTGTTGTAACAAAAGCAGAAAAAAACCATGCACAAATCCAGAATGCGCATGCATTTCAATTAATAGCTGAAGC
AAAGCCATGTGTAATATCCAGGACATGGAAGAAAAACCCATAAAGTCAGTTATAACAGTAAAAAAGTTCCACA
CCCGCATGA

****
****
****
Consensus:

TTTGTTGTAACAAAAGCAGAAAAAAACCATGCACAAATCCAGAATGCGCATGCATTTCAATTAATAGCTGAAGC
AAAGCCATGTGTAATATCCAGGACATGGAAGAAAAACCCATAAAGTCAGTTATAACAGTAAAAAAGTTCCACA
CCCGCATGA

>Bacillus_Fam_819_154_1 Nr. of seq. 1 Alignment length(with gaps) = 154
Alignment score = 0.000000
GCF_900098925.1_PRJEB15625_genomic.fna_18:275436-276206 Satlength=771 Nr
of Repeats=5 RepeatLength=154 seed=GGTTCTCCTA Num.seqs=5
Similarity=0.977489 0
GGTTCTCCTATATCTCGCTCGGCATCAGTCCAGGTCATACGGCGATGAACAGGACCCTTTCGCTTTTCGTTTT
TGTCCAGCAGCAGGGCCCTATCGCCTAGTGTCCTTCCCTTACCTCGGGTCGATAAGTCGAAAACGAAAGGAGA
GCCCTTTC

****

****
****
Consensus:

GGTTCTCCTATATCTCGCTCGGCATCAGTCCAGGTCATACGGCGATGAACAGGACCCTTTCGCTTTTCGTTTT
TGTCCAGCAGCAGGGCCCTATCGCCTAGTGTCCTTCCCTTACCTCGGGTCGATAAGTCGAAAACGAAAGGAGA
GCCCTTTC

>Bacillus_Fam_820_153_1 Nr. of seq. 1 Alignment length(with gaps) = 153
Alignment score = 0.000000
GCF_900156865.1_PRJEB18960_genomic.fna_3:4446177-4446943 Satlength=767 Nr of Repeats=5 RepeatLength=153 seed=CTAGAAAACT Num.seqs=4
Similarity=0.816198 0
CTAGAAAACTCGTAAAACCCATGCGAATCTCACGGTTTCACATGGTTTTATCAAAAGATCTCAAGAAAAGCAT
GTGGAAGTGGAAAAGTTCTCCAAATATTGACCGTTTTAGAGCTAGAATTTCCAAACTATGGATGTTGCGCATG
CCATTTC

****
****
****
Consensus:

CTAGAAAACTCGTAAAACCCATGCGAATCTCACGGTTTCACATGGTTTTATCAAAAGATCTCAAGAAAAGCAT
GTGGAAGTGGAAAAGTTCTCCAAATATTGACCGTTTTAGAGCTAGAATTTCCAAACTATGGATGTTGCGCATG
CCATTTC

>Bacillus_Fam_821_150_1 Nr. of seq. 1 Alignment length(with gaps) = 150
Alignment score = 0.000000
GCF_000238675.1_Baci_smit_7_3_47FAA_V1_genomic.fna_8:63370-64378
Satlength=1009 Nr of Repeats=5 RepeatLength=150 seed=TTCCGGCAAT
Num.seqs=3 Similarity=0.845926
0
TTCCGGCAATGAATTTTTAATGTGCTTCCAAACTCGATCGTCCACAAACACATCCGGAGTGGACAACGAAGTC
GAAAACAATGAGTTATAAGAATCTCCTCTCATCGGTTCAAAAATTTCTTTTGTCACCGGGTTAGGAAGCACAT
AGTT

****
****
****
Consensus:

TTCCGGCAATGAATTTTTAATGTGCTTCCAAACTCGATCGTCCACAAACACATCCGGAGTGGACAACGAAGTC
GAAAACAATGAGTTATAAGAATCTCCTCTCATCGGTTCAAAAATTTCTTTTGTCACCGGGTTAGGAAGCACAT
AGTT

>Bacillus_Fam_822_150_1 Nr. of seq. 1 Alignment length(with gaps) = 150
Alignment score = 0.000000
GCF_000712615.1_ASM71261v1_genomic.fna_42:22825-23572 Satlength=748 Nr of Repeats=5 RepeatLength=150 seed=TATGGTGGAT Num.seqs=4
Similarity=0.920000
0
TATGGTGGATTGGAGCAAACGGCTCAGTGCAAGGGGCTTATTGGTATGAAGGAATGGCACGACGAGAGAGTTA
TGAATTAGCACCAGCGGGAAGCGCTTCTACCAATGGAGGTATTGCAGCGGTGTCGAGAAGGCCAAACACTTTC
GAAC

****
****
****
Consensus:

TATGGTGGATTGGAGCAAACGGCTCAGTGCAAGGGGCTTATTGGTATGAAGGAATGGCACGACGAGAGAGTTA
TGAATTAGCACCAGCGGGAAGCGCTTCTACCAATGGAGGTATTGCAGCGGTGTCGAGAAGGCCAAACACTTTC
GAAC

>Bacillus_Fam_823_150_1 Nr. of seq. 1 Alignment length(with gaps) = 150
Alignment score = 0.000000
GCF_000712615.1_ASM71261v1_genomic.fna_110:463-1366 Satlength=904 Nr of
Repeats=6 RepeatLength=150 seed=ACTACCCACA Num.seqs=5
Similarity=0.584458
0
ACTACCCACATAACGTTTACACGTCCNTGNTTATCTACGAAAAACAAATCAAGTTGCTCATCGGTTTGACGTG
CAGTTGCTNNNACCGNATCCAATGGAACGTAGTTNGGTGCAGTNAGAGGAATTGGTCCTTGCCAATTTCCGGT
TCCG

****
****
****
Consensus:

ACTACCCACATAACGTTTACACGTCCNTGNTTATCTACGAAAAACAAATCAAGTTGCTCATCGGTTTGACGTG
CAGTTGCTNNNACCGNATCCAATGGAACGTAGTTNGGTGCAGTNAGAGGAATTGGTCCTTGCCAATTTCCGGT
TCCG

>Bacillus_Fam_824_148_1 Nr. of seq. 1 Alignment length(with gaps) = 148
Alignment score = 0.000000
GCF_000161455.1_ASM16145v1_genomic.fna_1:395331-396141 Satlength=811 Nr
of Repeats=4 RepeatLength=148 seed=GAACTTTTAA Num.seqs=3
Similarity=0.892593
0
GAACTTTTAAAAGCAGTAATGTAAGTTCATTATTCCGCAATTATTTATAAAAATTATCATACCTTAATACGAA
AAAAGAGGAGGAAATCATATGGCAATCGCAATGGCAGTTTTAAAATTTGTAGGTGGAGCACTTCCATTAGTAC
AA

****
****
**
Consensus:

GAACTTTTAAAAGCAGTAATGTAAGTTCATTATTCCGCAATTATTTATAAAAATTATCATACCTTAATACGAA
AAAAGAGGAGGAAATCATATGGCAATCGCAATGGCAGTTTTAAAATTTGTAGGTGGAGCACTTCCATTAGTAC
AA

>Bacillus_Fam_825_147_1 Nr. of seq. 1 Alignment length(with gaps) = 147
Alignment score = 0.000000
GCF_000430765.1_ASM43076v1_genomic.fna_6:140808-141395 Satlength=588 Nr
of Repeats=4 RepeatLength=147 seed=TTTTTCTTTG Num.seqs=3
Similarity=0.877628
0

TTTTTCTTTGGTGGCTGCATCAAGATTTGCGAACATGCCGTGGCGTTCACCCTTTTCCGGAAGTGTTACACCA
AGCTTTTTCAGCTGGTCCTGTGCCTGGGTCTGTGTCAGGGTGCCGGCCTTCAGCTTTTCCATAATCGCCCGCG
C

****
****
*
Consensus:

TTTTTCTTTGGTGGCTGCATCAAGATTTGCGAACATGCCGTGGCGTTCACCCTTTTCCGGAAGTGTTACACCA
AGCTTTTTCAGCTGGTCCTGTGCCTGGGTCTGTGTCAGGGTGCCGGCCTTCAGCTTTTCCATAATCGCCCGCG
C

>Bacillus_Fam_826_144_1 Nr. of seq. 1 Alignment length(with gaps) = 144
Alignment score = 0.000000
GCF_000712595.1_ASM71259v1_genomic.fna_14:117958-118822 Satlength=865 Nr
of Repeats=6 RepeatLength=144 seed=ATGAAATTAC Num.seqs=6
Similarity=0.946296 0
ATGAAATTACCGAGCTGACTAATCTCGATCCCTCAATGGAAGATACCAATTTGGATGAAGTGGCGTCTGTAAA
GGAAATCACTAATTCCATCGAGATTATTTCTACAAATGAAGATATCGAGACGATCGAAGGTGTCACTTCAC

****
****
Consensus:

ATGAAATTACCGAGCTGACTAATCTCGATCCCTCAATGGAAGATACCAATTTGGATGAAGTGGCGTCTGTAAA
GGAAATCACTAATTCCATCGAGATTATTTCTACAAATGAAGATATCGAGACGATCGAAGGTGTCACTTCAC

>Bacillus_Fam_827_143_1 Nr. of seq. 1 Alignment length(with gaps) = 143
Alignment score = 0.000000
GCF_000177235.2_ASM17723v2_genomic.fna_1:919080-919888 Satlength=809 Nr
of Repeats=4 RepeatLength=143 seed=ACTTTTTCAG Num.seqs=3
Similarity=0.962704
0
ACTTTTTCAGTGCCCTTTTATTTGTATTGCAATGGCTACGTTCGTTGCGCGCCTGATAGGATAAACCCACTCC
TATCAGGCTTTTAAACGATTGCAACGACTACGCACATTGCTTAGGGGCACTTCAAAAGTGAAAGAACACC

****
****
Consensus:

ACTTTTTCAGTGCCCTTTTATTTGTATTGCAATGGCTACGTTCGTTGCGCGCCTGATAGGATAAACCCACTCC
TATCAGGCTTTTAAACGATTGCAACGACTACGCACATTGCTTAGGGGCACTTCAAAAGTGAAAGAACACC

>Bacillus_Fam_828_139_1 Nr. of seq. 1 Alignment length(with gaps) = 139
Alignment score = 0.000000
GCF_000334155.1_L1_genomic.fna_81:1145-1840 Satlength=696 Nr of Repeats=5
RepeatLength=139 seed=TTTTGTGGAA Num.seqs=5 Similarity=0.984652
0
TTTTGTGGAAAAGGGGACGGAGCTTACAGGATGATAAAGCAGGAAGCCTATCGCTCACACCCCTTCATTGAAA
GGTTACTCAGCGTTACTGGACGTACTCATTTTCATCCCAGTGGGAAGAGCGAGCCACTTATGCTAA

****
****

Consensus:

TTTTGTGGAAAAGGGGACGGAGCTTACAGGATGATAAAGCAGGAAGCCTATCGCTCACACCCCTTCATTGAAA
GGTTACTCAGCGTTACTGGACGTACTCATTTTCATCCCAGTGGGAAGAGCGAGCCACTTATGCTAA

>Bacillus_Fam_829_135_1 Nr. of seq. 1 Alignment length(with gaps) = 135
Alignment score = 0.000000
GCF_000712615.1_ASM71261v1_genomic.fna_82:4706-5375 Satlength=670 Nr of
Repeats=5 RepeatLength=135 seed=GACGGTTCCC Num.seqs=3
Similarity=0.802469
0
GACGGTTCCCGTGCCTACACAGCAAATGTTAACAATGACACGGTTTCCGTGATCGGCACGGCCACGAATACTG
TCTTGACCACCATTTCGCTCGCTCCCGCAGGAAACGGTCCTNNTGCTATTGCCATCACGCCG

****
****
Consensus:

GACGGTTCCCGTGCCTACACAGCAAATGTTAACAATGACACGGTTTCCGTGATCGGCACGGCCACGAATACTG
TCTTGACCACCATTTCGCTCGCTCCCGCAGGAAACGGTCCTNNTGCTATTGCCATCACGCCG

>Bacillus_Fam_830_132_1 Nr. of seq. 1 Alignment length(with gaps) = 132
Alignment score = 0.000000
GCF_000225265.1_ASM22526v1_genomic.fna_2:43307-43811 Satlength=505 Nr of
Repeats=4 RepeatLength=126 seed=CATCAATGAC Num.seqs=4
Similarity=0.431287
0
CATCAATGACAGAAACNGTATCACTATTTTCNTCATTAGCTACATAAATACGATTATTAGAGGGATTAACACC
TACATCAGAAGGCTGANTCTCCGACAGGTACTATTCCAATAACAGCATTTGTNNACCTG

****
****
Consensus:

CATCAATGACAGAAACNGTATCACTATTTTCNTCATTAGCTACATAAATACGATTATTAGAGGGATTAACACC
TACATCAGAAGGCTGANTCTCCGACAGGTACTATTCCAATAACAGCATTTGTNNACCTG

>Bacillus_Fam_831_132_1 Nr. of seq. 1 Alignment length(with gaps) = 132
Alignment score = 0.000000
GCF_001590785.1_ASM159078v1_genomic.fna_20:27981-28774 Satlength=794 Nr
of Repeats=5 RepeatLength=132 seed=TTTTCTTCCC Num.seqs=3
Similarity=0.673300
0
TTTTCTTCCCAGAACAATTGAATTTGCTCCAATGCTCCAGAAATAATCTCACTTATAAATGCCATTGCCTCTG
AAATAATGGTCCCTACAACTTCCATCGTACTAGTCAATATTTCCATTATCCATGTGCCG

****
****
Consensus:

TTTTCTTCCCAGAACAATTGAATTTGCTCCAATGCTCCAGAAATAATCTCACTTATAAATGCCATTGCCTCTG
AAATAATGGTCCCTACAACTTCCATCGTACTAGTCAATATTTCCATTATCCATGTGCCG

>Bacillus_Fam_832_132_1 Nr. of seq. 1 Alignment length(with gaps) = 132
Alignment score = 0.000000
GCF_002019605.1_ASM201960v1_genomic.fna_1:1113610-1114144 Satlength=535
Nr of Repeats=4 RepeatLength=132 seed=TCGCGGGGTG Num.seqs=3
Similarity=0.993266 0
TCGCGGGGTGGAGCATGGAGCAAAGCTTCTTGATTTTACATCGACATGTCGTGACGCATCAAGCTACAAAGCG
TTACGAGAAGAGGAAACGATGACCTCTTAGAAAAAACATCATCTGTAAGCTATATAACG

****
****
Consensus:

TCGCGGGGTGGAGCATGGAGCAAAGCTTCTTGATTTTACATCGACATGTCGTGACGCATCAAGCTACAAAGCG
TTACGAGAAGAGGAAACGATGACCTCTTAGAAAAAACATCATCTGTAAGCTATATAACG

>Bacillus_Fam_833_131_1 Nr. of seq. 1 Alignment length(with gaps) = 131
Alignment score = 0.000000
GCF_001590785.1_ASM159078v1_genomic.fna_4:31738-32230 Satlength=493 Nr of
Repeats=4 RepeatLength=123 seed=CATTAAACCA Num.seqs=4
Similarity=0.312261
0
CATTAAACCATNNACTTAAGANTAGCCCATTGTTCCATNACCTAATCCTTTACAGCATCCCACCCATCTGAAA
GCCAGTCCNANNGTTGTCAAAATNTCAACATCTTCCTATAATGGCTCCCATACAGTTT

****
****
Consensus:

CATTAAACCATNNACTTAAGANTAGCCCATTGTTCCATNACCTAATCCTTTACAGCATCCCACCCATCTGAAA
GCCAGTCCNANNGTTGTCAAAATNTCAACATCTTCCTATAATGGCTCCCATACAGTTT

>Bacillus_Fam_834_129_1 Nr. of seq. 1 Alignment length(with gaps) = 129
Alignment score = 0.000000
GCF_900107275.1_IMG-
taxon_2675903024_annotated_assembly_genomic.fna_35:27208-27724
Satlength=517 Nr of Repeats=4 RepeatLength=129 seed=TTTTTGAACA
Num.seqs=4 Similarity=0.613946 0
TTTTTGAACAATCACGAAACGATGAGCGAACAGCCGCAACGCTTTTAAGCCAGTCATGAACGCATTTGTCATG
ATTGGCGAGCGGGGGAGGAAGCCATCGTCCTACAAATAAAAAAGGGCGCTTTTCCC

****
****
Consensus:

TTTTTGAACAATCACGAAACGATGAGCGAACAGCCGCAACGCTTTTAAGCCAGTCATGAACGCATTTGTCATG
ATTGGCGAGCGGGGGAGGAAGCCATCGTCCTACAAATAAAAAAGGGCGCTTTTCCC

>Bacillus_Fam_835_128_1 Nr. of seq. 1 Alignment length(with gaps) = 128
Alignment score = 0.000000
GCF_000615945.1_ASM61594v1_genomic.fna_82:23663-24293 Satlength=631 Nr of
Repeats=4 RepeatLength=126 seed=TGGTTTACAG Num.seqs=3
Similarity=0.464869
0

TGGTTTACAGAANATACAGGTAACAAAATAGGAAGAATNNCAACGGANGGTGAAATTACAGAATATNATATNC
CTACNNCAGATGCAGGTGCNTATNGATTATAGCNNNAGGTNCTGATGGTGCACTN

****
****
Consensus:

TGGTTTACAGAANATACAGGTAACAAAATAGGAAGAATNNCAACGGANGGTGAAATTACAGAATATNATATNC
CTACNNCAGATGCAGGTGCNTATNGATTATAGCNNNAGGTNCTGATGGTGCACTN

>Bacillus_Fam_836_127_1 Nr. of seq. 1 Alignment length(with gaps) = 127
Alignment score = 0.000000
GCF_000025825.1_ASM2582v1_genomic.fna_7:28124-28628 Satlength=505 Nr of
Repeats=4 RepeatLength=126 seed=TTTCTGTAAT Num.seqs=4
Similarity=0.602865
0
TTTCTGTAATTGATGGGTTAACAAATACTGTAATTGCCACGATTCCAGTAGGAAACTCAACCGACCGGTATAG
AGATCAATCCATACATCAATCGAATTTATATAACAAATAGAGGTAGTAATACAG

****
****
Consensus:

TTTCTGTAATTGATGGGTTAACAAATACTGTAATTGCCACGATTCCAGTAGGAAACTCAACCGACCGGTATAG
AGATCAATCCATACATCAATCGAATTTATATAACAAATAGAGGTAGTAATACAG

>Bacillus_Fam_837_127_1 Nr. of seq. 1 Alignment length(with gaps) = 127
Alignment score = 0.000000
GCF_900107275.1_IMG-
taxon_2675903024_annotated_assembly_genomic.fna_38:105111-105565
Satlength=455 Nr of Repeats=4 RepeatLength=127 seed=CGTCGTTTTT
Num.seqs=3 Similarity=0.979003 0
CGTCGTTTTTCCAAAAGGGAAATGCGCCCTTTTATTTTAGTTTACGATGGCTCGCTTTTGTCGCACGTCGGTT
ATGACAAATGCGCTCATAACCTACTTAAAAAACGCGACAGCTCCCTCATCGTAC

****
****
Consensus:

CGTCGTTTTTCCAAAAGGGAAATGCGCCCTTTTATTTTAGTTTACGATGGCTCGCTTTTGTCGCACGTCGGTT
ATGACAAATGCGCTCATAACCTACTTAAAAAACGCGACAGCTCCCTCATCGTAC

>Bacillus_Fam_838_126_1 Nr. of seq. 1 Alignment length(with gaps) = 126
Alignment score = 0.000000
GCF_001636345.1_ASM163634v1_genomic.fna_1:2067064-2067694 Satlength=631
Nr of Repeats=5 RepeatLength=126 seed=GATGTTGAAG Num.seqs=5
Similarity=0.789418 0
GATGTTGAAGCATTAACAACAGAAAAAGAAGCTGTATCTTCTTCTCATNCTGAAGGAAAAGAAACACACGATT
CAGTAAGCAAGGAAGAGAAAGCATCATCTCTAGTTGATGTAAAAGCGGACTTA

****
****
Consensus:

GATGTTGAAGCATTAACAACAGAAAAAGAAGCTGTATCTTCTTCTCATNCTGAAGGAAAAGAAACACACGATT
CAGTAAGCAAGGAAGAGAAAGCATCATCTCTAGTTGATGTAAAAGCGGACTTA

>Bacillus_Fam_839_125_1 Nr. of seq. 1 Alignment length(with gaps) = 125
Alignment score = 0.000000
GCF_001636425.1_ASM163642v1_genomic.fna_1:4638716-4643591 Satlength=4876
Nr of Repeats=39 RepeatLength=125 seed=AGCGCCCTCC Num.seqs=39
Similarity=0.991795 0
AGCGCCCTCCGCATTTCCTTGTCCAGCTTCGGGCGCTATCGGCTCGAGGGCAAATGCCCAGATGCCCAAGAGG
TGGAAGAGCGATCTTCCGTGTCATCTGGGCTTTGGCTGGTCGCCGATGGGCC

****
****
Consensus:

AGCGCCCTCCGCATTTCCTTGTCCAGCTTCGGGCGCTATCGGCTCGAGGGCAAATGCCCAGATGCCCAAGAGG
TGGAAGAGCGATCTTCCGTGTCATCTGGGCTTTGGCTGGTCGCCGATGGGCC

>Bacillus_Fam_840_124_1 Nr. of seq. 1 Alignment length(with gaps) = 124
Alignment score = 0.000000
GCF_000429725.1_ASM42972v1_genomic.fna_10:14934-15732 Satlength=799 Nr of
Repeats=5 RepeatLength=124 seed=GAACGTCCGA Num.seqs=3
Similarity=0.956989
0
GAACGTCCGAGCCGGCCGGCGGAGTGTCCGCCCAGCGCCGGAGTGACCGCTTTGCTCTTCGATGTGACCGCTC
ACCTCCTCCGAAGCGCCGTCCGCGGCCGCCTTCCGACCGCTTTTCCCGGCC

****
****
Consensus:

GAACGTCCGAGCCGGCCGGCGGAGTGTCCGCCCAGCGCCGGAGTGACCGCTTTGCTCTTCGATGTGACCGCTC
ACCTCCTCCGAAGCGCCGTCCGCGGCCGCCTTCCGACCGCTTTTCCCGGCC

>Bacillus_Fam_841_124_1 Nr. of seq. 1 Alignment length(with gaps) = 124
Alignment score = 0.000000
GCF_000986785.1_ASM98678v1_genomic.fna_48:14425-15293 Satlength=869 Nr of
Repeats=7 RepeatLength=124 seed=GGAAGCGCCT Num.seqs=7
Similarity=0.996928
0
GGAAGCGCCTTGGTCAGACCCGACCAGCATAAGTCGCTCCAGGATAGAAGGCGTCCTTTGCCTTCAATCCTGG
AGTGGCTTATGACTCGAGGGTCTAGGCGCTGGAGCTGGACAATCGAAAAGC

****
****
Consensus:

GGAAGCGCCTTGGTCAGACCCGACCAGCATAAGTCGCTCCAGGATAGAAGGCGTCCTTTGCCTTCAATCCTGG
AGTGGCTTATGACTCGAGGGTCTAGGCGCTGGAGCTGGACAATCGAAAAGC

>Bacillus_Fam_842_123_1 Nr. of seq. 1 Alignment length(with gaps) = 123
Alignment score = 0.000000

GCF_000986785.1_ASM98678v1_genomic.fna_50:5702-6836 Satlength=1135 Nr of Repeats=9 RepeatLength=123 seed=GCTTCCGCTT Num.seqs=8
Similarity=0.997290
0
GCTTCCGCTTTTCTTATTGTCCAGCTGCAGCGGCTAGCCCCTCGAGACACTTCGGTCCATCACCTGAAGGCAA
AGAACGCCTTCAACTGCTGGCCCTCCAGTGCTTTTCGGGGCTGGACAGCC

****
****
Consensus:

GCTTCCGCTTTTCTTATTGTCCAGCTGCAGCGGCTAGCCCCTCGAGACACTTCGGTCCATCACCTGAAGGCAA
AGAACGCCTTCAACTGCTGGCCCTCCAGTGCTTTTCGGGGCTGGACAGCC

>Bacillus_Fam_843_122_1 Nr. of seq. 1 Alignment length(with gaps) = 122
Alignment score = 0.000000
GCF_000708755.2_ASM70875v2_genomic.fna_2:743902-744385 Satlength=484 Nr of Repeats=4 RepeatLength=122 seed=CGAAAAGCGG Num.seqs=3
Similarity=0.729391
0
CGAAAAGCGGAAGCGCTCGTTTAGCTCCGGGAGTCAGATAAGGAAGTGGCGGAAAAGGCGCTTTTTGCCTTTN
CNGACAATTCCGTTCTGACAGAGGAGCTGAGCGCTGGAGCTAGACAATG

****
****
Consensus:

CGAAAAGCGGAAGCGCTCGTTTAGCTCCGGGAGTCAGATAAGGAAGTGGCGGAAAAGGCGCTTTTTGCCTTTN
CNGACAATTCCGTTCTGACAGAGGAGCTGAGCGCTGGAGCTAGACAATG

>Bacillus_Fam_844_122_1 Nr. of seq. 1 Alignment length(with gaps) = 122
Alignment score = 0.000000
GCF_000709935.2_ASM70993v2_genomic.fna_5:41241-41730 Satlength=490 Nr of Repeats=4 RepeatLength=122 seed=CGAAAAGCGG Num.seqs=3
Similarity=0.704000
0
CGAAAAGCGGAGCCGACTGTTCAGTCCTNTCAGTCAGATAAGAAATCACCGGAAAAGTCCGGTTTTGACTTTT
TCGGGGATTTTGTTCTGACAGAAGGACTAGGAGGCGGAGCTGGACAATG

****
****
Consensus:

CGAAAAGCGGAGCCGACTGTTCAGTCCTNTCAGTCAGATAAGAAATCACCGGAAAAGTCCGGTTTTGACTTTT
TCGGGGATTTTGTTCTGACAGAAGGACTAGGAGGCGGAGCTGGACAATG

>Bacillus_Fam_845_122_1 Nr. of seq. 1 Alignment length(with gaps) = 122
Alignment score = 0.000000
GCF_001636425.1_ASM163642v1_genomic.fna_1:3988479-3988970 Satlength=492 Nr of Repeats=4 RepeatLength=122 seed=GCATCATTCT Num.seqs=3
Similarity=0.775986 0
GCATCATTCTCTTGTTTCCATTGGTTGTTGTTCATAAAAGCAACATCTCCTTTGGGTCATGAGAATGGTTTAT
ACTTATTAGNTTACAGGTATTCCATNTTTAGTCGCCGGGACTAAAAACN

****
****
Consensus:

GCATCATTCTCTTGTTTCCATTGGTTGTTGTTCATAAAAGCAACATCTCCTTTGGGTCATGAGAATGGTTTAT
ACTTATTAGNTTACAGGTATTCCATNTTTAGTCGCCGGGACTAAAAACN

>Bacillus_Fam_846_121_1 Nr. of seq. 1 Alignment length(with gaps) = 121
Alignment score = 0.000000
GCF_000181495.1_ASM18149v1_genomic.fna_142:19481-19965 Satlength=485 Nr
of Repeats=4 RepeatLength=121 seed=GAAATTCGCC Num.seqs=4
Similarity=0.970615
0
GAAATTCGCCGCAATCTCTCCTCCCCTGACGAACGAGAAGCAAGAAGTTCAAGGAGATGAGAGAGAGAGGACC
GGAGCGTATCTATAATACGTGAGGATCCGAGCGAGCGAAATCGACGCA

****
****
Consensus:

GAAATTCGCCGCAATCTCTCCTCCCCTGACGAACGAGAAGCAAGAAGTTCAAGGAGATGAGAGAGAGAGGACC
GGAGCGTATCTATAATACGTGAGGATCCGAGCGAGCGAAATCGACGCA

>Bacillus_Fam_847_120_1 Nr. of seq. 1 Alignment length(with gaps) = 120
Alignment score = 0.000000
GCF_000161455.1_ASM16145v1_genomic.fna_1:886062-886479 Satlength=418 Nr
of Repeats=4 RepeatLength=120 seed=TGGTATTACT Num.seqs=3
Similarity=0.861566
0
TGGTATTACTTTGAGAGTAATGGCGTAATGAAAACAGATTGGTATTTTGATGGAACGAATTGGTTTTATATGA
ATAGCAATGGTGGAATGGAGACAGGATGGAAAAAGATTCAAGGAACG

****
****
Consensus:

TGGTATTACTTTGAGAGTAATGGCGTAATGAAAACAGATTGGTATTTTGATGGAACGAATTGGTTTTATATGA
ATAGCAATGGTGGAATGGAGACAGGATGGAAAAAGATTCAAGGAACG

>Bacillus_Fam_848_120_1 Nr. of seq. 1 Alignment length(with gaps) = 120
Alignment score = 0.000000
GCF_000169195.2_ASM16919v2_genomic.fna_1:2868366-2869215 Satlength=850 Nr
of Repeats=4 RepeatLength=120 seed=GAAGAAATCG Num.seqs=3
Similarity=0.595556 0
GAAGAAATCGATGTGATTCCTTTCCANGAAACCGACAATGCCGGGCAACGCGAAAAGCTGCCATCAGATGCGG
AAAATGAAACAGTTGGCGGTGCGANAGGTGAAGACACAGACGAACCG

****
****
Consensus:

GAAGAAATCGATGTGATTCCTTTCCANGAAACCGACAATGCCGGGCAACGCGAAAAGCTGCCATCAGATGCGG
AAAATGAAACAGTTGGCGGTGCGANAGGTGAAGACACAGACGAACCG

>Bacillus_Fam_849_118_1 Nr. of seq. 1 Alignment length(with gaps) = 118
Alignment score = 0.000000
Bacillus_murimartini_genomic.fna_9:918630-919220 Satlength=591 Nr of Repeats=5 RepeatLength=118 seed=GCTTATAAGA Num.seqs=5
Similarity=0.995480 0
GCTTATAAGAAACCTCGCCTTTTTGAACTGCGCCGTGCTGTAGCAGCTGGCGGCCATTAATCTCATATAGCAT
TTGACATCTAGTACTCTATTTTAGGAGGGATGCTCAATGAAAAAA

****
****
Consensus:

GCTTATAAGAAACCTCGCCTTTTTGAACTGCGCCGTGCTGTAGCAGCTGGCGGCCATTAATCTCATATAGCAT
TTGACATCTAGTACTCTATTTTAGGAGGGATGCTCAATGAAAAAA

>Bacillus_Fam_850_116_1 Nr. of seq. 1 Alignment length(with gaps) = 116
Alignment score = 0.000000
GCF_000177235.2_ASM17723v2_genomic.fna_1:1679718-1680414 Satlength=697 Nr of Repeats=6 RepeatLength=116 seed=CAAAAGGTCG Num.seqs=6
Similarity=0.989272 0
CAAAAGGTCGAACTTTACCTTTTGGGGATTTTACGTAACGAGAGCGAAGTCGTCACAAAGAAGAGCACAAATG
GAGTGAACTTCTCCATTAGTGCGAGTGACGAACGGAGCCTCGC

****
****
Consensus:

CAAAAGGTCGAACTTTACCTTTTGGGGATTTTACGTAACGAGAGCGAAGTCGTCACAAAGAAGAGCACAAATG
GAGTGAACTTCTCCATTAGTGCGAGTGACGAACGGAGCCTCGC

>Bacillus_Fam_851_115_1 Nr. of seq. 1 Alignment length(with gaps) = 115
Alignment score = 0.000000
GCF_000751775.1_A1A_genomic.fna_102:38116-38692 Satlength=577 Nr of Repeats=5 RepeatLength=115 seed=CTCATAGAAC Num.seqs=4
Similarity=0.868599
0
CTCATAGAACGGCTATATGAGACAAAATTAGTTGAAAATCAAGCCAAAATGTCCCATAGAATTGCCCGGATCC
CCTGCTATGGGACAAAACCAGCGGAAGCCGAAGCAAAAATGG

****
****
Consensus:

CTCATAGAACGGCTATATGAGACAAAATTAGTTGAAAATCAAGCCAAAATGTCCCATAGAATTGCCCGGATCC
CCTGCTATGGGACAAAACCAGCGGAAGCCGAAGCAAAAATGG

>Bacillus_Fam_852_114_1 Nr. of seq. 1 Alignment length(with gaps) = 114
Alignment score = 0.000000
GCF_000621445.1_ASM62144v1_genomic.fna_8:58648-59360 Satlength=713 Nr of Repeats=4 RepeatLength=114 seed=AAAAGGTATA Num.seqs=3
Similarity=0.992203
0

AAAAGGTATAGGGCGACATTTTGGGTCGGGAGACGCTCGTTGCTCGCCATTCAGAAGTACACTGAATGGCTTT
AAGAGCGGGCAACGGCTTTCCCCCTCTTACGAGAAAGAACC

****
****
Consensus:

AAAAGGTATAGGGCGACATTTTGGGTCGGGAGACGCTCGTTGCTCGCCATTCAGAAGTACACTGAATGGCTTT
AAGAGCGGGCAACGGCTTTCCCCCTCTTACGAGAAAGAACC

>Bacillus_Fam_853_114_1 Nr. of seq. 1 Alignment length(with gaps) = 114
Alignment score = 0.000000
GCF_001274935.1_ASM127493v1_genomic.fna_37:230037-231096 Satlength=1060
Nr of Repeats=5 RepeatLength=114 seed=TTCCTCTGTT Num.seqs=3
Similarity=0.937622 0
TTCCTCTGTTCCTTCACTTTCCGACTCCGCTGTTTTTTCCTTCTGCTTCGCGAGAGCCTGTGCTTTTGCCTTT
GCGGCTGCTGCGGCCTTTTTCTTCGCCAGGGCTAGTGCGTC

****
****
Consensus:

TTCCTCTGTTCCTTCACTTTCCGACTCCGCTGTTTTTTCCTTCTGCTTCGCGAGAGCCTGTGCTTTTGCCTTT
GCGGCTGCTGCGGCCTTTTTCTTCGCCAGGGCTAGTGCGTC

>Bacillus_Fam_854_114_1 Nr. of seq. 1 Alignment length(with gaps) = 114
Alignment score = 0.000000
GCF_900109925.1_IMG-
taxon_2651870116_annotated_assembly_genomic.fna_28:73806-74259
Satlength=454 Nr of Repeats=4 RepeatLength=114 seed=CACTTGATTC
Num.seqs=3 Similarity=0.924638 0
CACTTGATTCAATGGAAGAAGATGACTCCTCGTCTGAGACCTCCGAATCAATGCTACTGGATTCATCCGGTGA
GGGGGCAGAGTCATCGCAACAGGTAACCGTTTCATCCTCCA

****
****
Consensus:

CACTTGATTCAATGGAAGAAGATGACTCCTCGTCTGAGACCTCCGAATCAATGCTACTGGATTCATCCGGTGA
GGGGGCAGAGTCATCGCAACAGGTAACCGTTTCATCCTCCA

>Bacillus_Fam_855_111_1 Nr. of seq. 1 Alignment length(with gaps) = 111
Alignment score = 0.000000
GCF_000334155.1_L1_genomic.fna_160:15-793 Satlength=779 Nr of Repeats=7
RepeatLength=111 seed=TGTGGCGGAA Num.seqs=6 Similarity=1.000000
0
TGTGGCGGAATGGCAGACGCGCTAGACTTAGGATCTAGTGTCTTATGACGTGGGGGTTCAAGTCCCTTCACCC
GCACTTTGAATTTTTAGAGTGATGAAAACTGAAGCGGT

****
****
Consensus:

TGTGGCGGAATGGCAGACGCGCTAGACTTAGGATCTAGTGTCTTATGACGTGGGGGTTCAAGTCCCTTCACCC
GCACTTTGAATTTTTAGAGTGATGAAAACTGAAGCGGT

>Bacillus_Fam_856_111_1 Nr. of seq. 1 Alignment length(with gaps) = 111
Alignment score = 0.000000
GCF_001648575.1_ASM164857v1_genomic.fna_66:59259-59688 Satlength=430 Nr
of Repeats=4 RepeatLength=111 seed=TTCGCTGCTG Num.seqs=3
Similarity=0.927928
0
TTCGCTGCTGCGGCGGCTTTCTTTTTGGCTAGCTCCTTGGCGTCATCTGTTTCGGTTGCTGGAGCTTCGCCTG
CTTCACTCTTTTGTTTTGCCAGTGCGGCAGCCTTTGCT

****
****
Consensus:

TTCGCTGCTGCGGCGGCTTTCTTTTTGGCTAGCTCCTTGGCGTCATCTGTTTCGGTTGCTGGAGCTTCGCCTG
CTTCACTCTTTTGTTTTGCCAGTGCGGCAGCCTTTGCT

>Bacillus_Fam_857_111_1 Nr. of seq. 1 Alignment length(with gaps) = 111
Alignment score = 0.000000
GCF_900111815.1_PRJEB17078_genomic.fna_1:2921195-2921687 Satlength=493 Nr
of Repeats=5 RepeatLength=111 seed=CAGCGGCTTT Num.seqs=4
Similarity=0.949950 0
CAGCGGCTTTTTTCTTCGCTAAGTCGTCTGCTTCCCCAGCTGGCGGCGTTTCTGAGTCTGACTCTTTCGCTTT
CATTTTCGCGAGTGCGGCAGCCTTTGCTTTCGCTGCTG

****
****
Consensus:

CAGCGGCTTTTTTCTTCGCTAAGTCGTCTGCTTCCCCAGCTGGCGGCGTTTCTGAGTCTGACTCTTTCGCTTT
CATTTTCGCGAGTGCGGCAGCCTTTGCTTTCGCTGCTG

>Bacillus_Fam_858_110_1 Nr. of seq. 1 Alignment length(with gaps) = 110
Alignment score = 0.000000
GCF_000161455.1_ASM16145v1_genomic.fna_1:5374739-5375117 Satlength=379 Nr
of Repeats=4 RepeatLength=111 seed=GATATGGCAA Num.seqs=3
Similarity=0.448118 0
GATATGGCAAAAGTGTTTCATGTTTTATATGTGTCAAATATTANNGAAACANCTATAAAACGTTGATATATAA
GGGTNTATAAGATGTTTCNNATATTTATTAGGTCAAG

****
****
Consensus:

GATATGGCAAAAGTGTTTCATGTTTTATATGTGTCAAATATTANNGAAACANCTATAAAACGTTGATATATAA
GGGTNTATAAGATGTTTCNNATATTTATTAGGTCAAG

>Bacillus_Fam_859_110_1 Nr. of seq. 1 Alignment length(with gaps) = 110
Alignment score = 0.000000
GCF_000712615.1_ASM71261v1_genomic.fna_61:8384-8914 Satlength=531 Nr of
Repeats=5 RepeatLength=112 seed=AGGATACCGC Num.seqs=3

Similarity=0.546448
0
AGGATACCGCAAACGTGTTGCAAGTTAAGTTATANGTATATGAATAAGAAAAAAACGNCTATCCTTTGATATA
TAAGGNATTCGAGTNAAATGACAAAAAAGTTAGGGTA

****
****
Consensus:

AGGATACCGCAAACGTGTTGCAAGTTAAGTTATANGTATATGAATAAGAAAAAAACGNCTATCCTTTGATATA
TAAGGNATTCGAGTNAAATGACAAAAAAGTTAGGGTA

>Bacillus_Fam_860_108_1 Nr. of seq. 1 Alignment length(with gaps) = 108
Alignment score = 0.000000
GCF_000374565.1_ASM37456v1_genomic.fna_3:32442-32847 Satlength=406 Nr of
Repeats=4 RepeatLength=108 seed=ACAGGTCCAG Num.seqs=3
Similarity=0.732510
0
ACAGGTCCAGTTGGTCCNCNAGGAGCGACGGGCCCCCCGGNCCTACAGGAGCAGTAGGCCCAGCAGGGCCAA
CAGGAGCCCCAGGCCCGATCGGCCCAATGGGAGCG

****
****
Consensus:

ACAGGTCCAGTTGGTCCNCNAGGAGCGACGGGCCCCCCGGNCCTACAGGAGCAGTAGGCCCAGCAGGGCCAA
CAGGAGCCCCAGGCCCGATCGGCCCAATGGGAGCG

>Bacillus_Fam_861_106_1 Nr. of seq. 1 Alignment length(with gaps) = 106
Alignment score = 0.000000
GCF_900156865.1_PRJEB18960_genomic.fna_3:3352570-3353080 Satlength=511 Nr
of Repeats=5 RepeatLength=106 seed=TTTTTAGGAG Num.seqs=4
Similarity=0.830189 0
TTTTTAGGAGATAGCGAACCTGCAACATGCCTTTGCGGGTTCGCAATTGGATAATATTAGGAAACACCATTCC
TGTATAACTCATTTGCATCCTCGCACTTGGATA

****
****
Consensus:

TTTTTAGGAGATAGCGAACCTGCAACATGCCTTTGCGGGTTCGCAATTGGATAATATTAGGAAACACCATTCC
TGTATAACTCATTTGCATCCTCGCACTTGGATA

>Bacillus_Fam_862_105_1 Nr. of seq. 1 Alignment length(with gaps) = 105
Alignment score = 0.000000
GCF_000017425.1_ASM1742v1_genomic.fna_1:3693939-3694896 Satlength=958 Nr
of Repeats=4 RepeatLength=105 seed=CTGAAACAGC Num.seqs=3
Similarity=0.932275 0
CTGAAACAGCAACTGGAAACGAAAGACGAGCAGATTCAGAAGCTAGAACAAGAAAAGCAAAAGTTAGAAACCC
AAATGAATCAGTTGCAAGGAGGGCCAGAAAAT

****
****
Consensus:

CTGAAACAGCAACTGGAAACGAAAGACGAGCAGATTCAGAAGCTAGAACAAGAAAAGCAAAAGTTAGAAACCC
AAATGAATCAGTTGCAAGGAGGGCCAGAAAAT

>Bacillus_Fam_863_105_1 Nr. of seq. 1 Alignment length(with gaps) = 105
Alignment score = 0.000000
GCF_000177235.2_ASM17723v2_genomic.fna_1:913685-914101 Satlength=417 Nr of Repeats=4 RepeatLength=104 seed=AGCGCCCCAT Num.seqs=4
Similarity=0.926455
0
AGCGCCCCATTCTAAAGGGAAAAATATATGTAGAAAGGCGCACAAATCGAAATAGCGGGCCATTGAGAGAAAG
AAAAAGCAATAGAATGGCCAGCAAAATAAAAT

****
Consensus:

AGCGCCCCATTCTAAAGGGAAAAATATATGTAGAAAGGCGCACAAATCGAAATAGCGGGCCATTGAGAGAAAG
AAAAAGCAATAGAATGGCCAGCAAAATAAAAT

>Bacillus_Fam_864_105_1 Nr. of seq. 1 Alignment length(with gaps) = 105
Alignment score = 0.000000
GCF_000813125.1_ASM81312v1_genomic.fna_80:7351-7877 Satlength=527 Nr of Repeats=5 RepeatLength=105 seed=TATCTATGTC Num.seqs=4
Similarity=0.879365
0
TATCTATGTCAACTGGTATAGCAATGAAGCAATTCGATCAAAGCTTAAAAAAACTAACGGCTCTTCCAACTGA
GAATCTAAACAGTGTAATAAATTCCTCATTGC

****
Consensus:

TATCTATGTCAACTGGTATAGCAATGAAGCAATTCGATCAAAGCTTAAAAAAACTAACGGCTCTTCCAACTGA
GAATCTAAACAGTGTAATAAATTCCTCATTGC

>Bacillus_Fam_865_105_1 Nr. of seq. 1 Alignment length(with gaps) = 105
Alignment score = 0.000000
GCF_001591485.1_ASM159148v1_genomic.fna_104:1301-1875 Satlength=575 Nr of Repeats=5 RepeatLength=105 seed=CTTCAGACAG Num.seqs=3
Similarity=0.690112
0
CTTCAGACAGAGGCNGGCATGCCTTTCTCTTTGTTGTCCGAACCTACGGTAACTTCGGACTGTACTCGAGTTT
TTCTTGAGGTTAAGTGTCCAAACCTTCTNCGA

****
Consensus:

CTTCAGACAGAGGCNGGCATGCCTTTCTCTTTGTTGTCCGAACCTACGGTAACTTCGGACTGTACTCGAGTTT
TTCTTGAGGTTAAGTGTCCAAACCTTCTNCGA

>Bacillus_Fam_866_104_1 Nr. of seq. 1 Alignment length(with gaps) = 104
Alignment score = 0.000000

GCF_000294775.2_ASM29477v2_genomic.fna_1:3556334-3556750 Satlength=417 Nr of Repeats=4 RepeatLength=104 seed=GGACTCTGGT Num.seqs=4 Similarity=0.833074 0
GGACTCTGGTTGAGGATATCACGTATTTTGGGTCCAAACCTGAGCCTACTTCAGACTCTGGTTGAGCTCATCCTGAAATGTGAGTCCGAACCTAGTACAACTTC

****
Consensus:

GGACTCTGGTTGAGGATATCACGTATTTTGGGTCCAAACCTGAGCCTACTTCAGACTCTGGTTGAGCTCATCCTGAAATGTGAGTCCGAACCTAGTACAACTTC

>Bacillus_Fam_867_104_1 Nr. of seq. 1 Alignment length(with gaps) = 104 Alignment score = 0.000000
GCF_000752035.1_Bacillus_rubiinfantis_genomic.fna_11:159625-160145 Satlength=521 Nr of Repeats=5 RepeatLength=104 seed=TGGTTTCTTA Num.seqs=5 Similarity=0.882243 0
TGGTTTCTTACCGTACCAATGATTTCCCTCTTTCCTAGGGCAAAATCAACGCTTGATTTTTACCGCCTGAACCGTTTTTTCTCTTCCTTGGGAAAATTCATTAA

****
Consensus:

TGGTTTCTTACCGTACCAATGATTTCCCTCTTTCCTAGGGCAAAATCAACGCTTGATTTTTACCGCCTGAACCGTTTTTTCTCTTCCTTGGGAAAATTCATTAA

>Bacillus_Fam_868_104_1 Nr. of seq. 1 Alignment length(with gaps) = 104 Alignment score = 0.000000
GCF_001636345.1_ASM163634v1_genomic.fna_1:1851553-1851970 Satlength=418 Nr of Repeats=4 RepeatLength=104 seed=GAGGTGTGTT Num.seqs=3 Similarity=0.923077 0
GAGGTGTGTTTCGGAGCTAAACGCGACTATGAAATCCTATATATGGAAAATAGTGTCGTGGAAATGCTCTAATCACGACAGAATAATCCAATAAATGGATAACA

****
Consensus:

GAGGTGTGTTTCGGAGCTAAACGCGACTATGAAATCCTATATATGGAAAATAGTGTCGTGGAAATGCTCTAATCACGACAGAATAATCCAATAAATGGATAACA

>Bacillus_Fam_869_104_1 Nr. of seq. 1 Alignment length(with gaps) = 104 Alignment score = 0.000000
GCF_001645705.1_ASM164570v1_genomic.fna_56:37601-38537 Satlength=937 Nr of Repeats=9 RepeatLength=104 seed=TTTTCTGAAT Num.seqs=9 Similarity=0.892450
0
TTTTCTGAATTGCAACGCTTTATGGGTTCTATGAAACCTGCTATATTACCACTTTTGAAGATTACAACCCATTACGGGAACTATGAACCGCACTATGCGACCGC

****

Consensus:

TTTTCTGAATTGCAACGCTTTATGGGTTCTATGAAACCTGCTATATTACCACTTTTGAAGATTACAACCCATT
ACGGGAACTATGAACCGCACTATGCGACCGC

>Bacillus_Fam_870_104_1 Nr. of seq. 1 Alignment length(with gaps) = 104
Alignment score = 0.000000
GCF_002019645.1_ASM201964v1_genomic.fna_1:591573-591926 Satlength=354 Nr
of Repeats=4 RepeatLength=104 seed=CGTCAAGTCC Num.seqs=3
Similarity=0.891005 0
CGTCAAGTCCCGGTGTANGATTAAAAATCCGGCGGGTTCCGAATGAAATCGGGCCAGATGGAAGACAAATCGG
GCGGGAATCAAGGTAAATCCGGCAAGCCGGG

****
****
Consensus:

CGTCAAGTCCCGGTGTANGATTAAAAATCCGGCGGGTTCCGAATGAAATCGGGCCAGATGGAAGACAAATCGG
GCGGGAATCAAGGTAAATCCGGCAAGCCGGG

>Bacillus_Fam_871_104_1 Nr. of seq. 1 Alignment length(with gaps) = 104
Alignment score = 0.000000
GCF_002019645.1_ASM201964v1_genomic.fna_1:2399755-2400142 Satlength=388
Nr of Repeats=4 RepeatLength=104 seed=GAAAGCGGCG Num.seqs=3
Similarity=0.910256 0
GAAAGCGGCGACTTTCAGAAAGAATCCGGCGGGAAAGCGAGGAAATCCGGCGAGANACATTCGAGTGACTGGT
TCGGGTGAAAAAAACGGGCGGGATTTAGAAG

****
****
Consensus:

GAAAGCGGCGACTTTCAGAAAGAATCCGGCGGGAAAGCGAGGAAATCCGGCGAGANACATTCGAGTGACTGGT
TCGGGTGAAAAAAACGGGCGGGATTTAGAAG

>Bacillus_Fam_872_104_1 Nr. of seq. 1 Alignment length(with gaps) = 104
Alignment score = 0.000000
GCF_002019645.1_ASM201964v1_genomic.fna_1:4123904-4124424 Satlength=521
Nr of Repeats=5 RepeatLength=104 seed=ACCCGCCCGA Num.seqs=3
Similarity=0.940171 0
ACCCGCCCGATTATTCACTTTTCTCGCCGGATAATTGACCCGGATTTGACCCAGATTCCAGAACTGGCCCGAT
TTTCTTTGGATCCCGCCGCATTCGAGCTCAC

****
****
Consensus:

ACCCGCCCGATTATTCACTTTTCTCGCCGGATAATTGACCCGGATTTGACCCAGATTCCAGAACTGGCCCGAT
TTTCTTTGGATCCCGCCGCATTCGAGCTCAC

>Bacillus_Fam_873_104_1 Nr. of seq. 1 Alignment length(with gaps) = 104
Alignment score = 0.000000
GCF_900156875.1_PRJEB18969_genomic.fna_11:1022316-1022802 Satlength=487
Nr of Repeats=5 RepeatLength=104 seed=ACAAGAAATT Num.seqs=3

Similarity=0.931624 0
ACAAGAAATTGGGTCTGAATAAAATCGTAATTCAGACAAGAGATCTGCTAAGGTGAGAAATCAAGTCCGAATG
GGCGGGGGATTCAGACAGAAAAGAGACTTCA

****
****
Consensus:

ACAAGAAATTGGGTCTGAATAAAATCGTAATTCAGACAAGAGATCTGCTAAGGTGAGAAATCAAGTCCGAATG
GGCGGGGGATTCAGACAGAAAAGAGACTTCA

>Bacillus_Fam_874_103_1 Nr. of seq. 1 Alignment length(with gaps) = 103
Alignment score = 0.000000
GCF_000177235.2_ASM17723v2_genomic.fna_1:3310743-3311151 Satlength=409 Nr
of Repeats=4 RepeatLength=103 seed=TTTATTCTAC Num.seqs=3
Similarity=0.892125 0
TTTATTCTACCTTCACACGATTCTCCNCGTCATGACGGGACTTTAAAACATGGTTCTTACCCCTTCAGACCGG
GGCGTTCACTCTAAAAGGATATTAAATCGT

****
****
Consensus:

TTTATTCTACCTTCACACGATTCTCCNCGTCATGACGGGACTTTAAAACATGGTTCTTACCCCTTCAGACCGG
GGCGTTCACTCTAAAAGGATATTAAATCGT

>Bacillus_Fam_875_103_1 Nr. of seq. 1 Alignment length(with gaps) = 103
Alignment score = 0.000000
GCF_001420715.1_ASM142071v1_genomic.fna_22:278070-278585 Satlength=516 Nr
of Repeats=5 RepeatLength=103 seed=AACTTTTTGG Num.seqs=5
Similarity=0.919741 0
AACTTTTTGGAAAAAGTGCTGAACTAAAGAGTGGAAACGCGGAACCGCCAGAGGAATGCTGAAAAAATGGAGA
TTAACGCTGAAATAGGGGAGGAAAACGCTG

****
****
Consensus:

AACTTTTTGGAAAAAGTGCTGAACTAAAGAGTGGAAACGCGGAACCGCCAGAGGAATGCTGAAAAAATGGAGA
TTAACGCTGAAATAGGGGAGGAAAACGCTG

>Bacillus_Fam_876_102_1 Nr. of seq. 1 Alignment length(with gaps) = 102
Alignment score = 0.000000
GCF_000217835.1_ASM21783v1_genomic.fna_1:395335-395743 Satlength=409 Nr
of Repeats=4 RepeatLength=102 seed=GGTTGGAAGC Num.seqs=4
Similarity=0.906419
0
GGTTGGAAGCACATGGAACTCTGCAATCAAAAATATAGATACACTAAAATCATTCTCTGACCCTTTGAAAGAT
ACACGAAAAGCCCTTGCTTCAATTGCAGA

****
****
Consensus:

GGTTGGAAGCACATGGAACTCTGCAATCAAAAATATAGATACACTAAAATCATTCTCTGACCCTTTGAAAGAT
ACACGAAAAGCCCTTGCTTCAATTGCAGA

>Bacillus_Fam_877_99_1 Nr. of seq. 1 Alignment length(with gaps) = 99
Alignment score = 0.000000
GCF_000299035.1_ASM29903v1_genomic.fna_2:1202161-1203221 Satlength=1061
Nr of Repeats=7 RepeatLength=99 seed=AACAAAAATA Num.seqs=6
Similarity=1.000000 0
AACAAAAATAACAGTAACGATGAAGATACAGAAACAACATCGAATGAAAAAATAAACAATACAAAACAAGATA
ACGATAATCAACAAAATTCTAACAAC

****
****
Consensus:

AACAAAAATAACAGTAACGATGAAGATACAGAAACAACATCGAATGAAAAAATAAACAATACAAAACAAGATA
ACGATAATCAACAAAATTCTAACAAC

>Bacillus_Fam_878_99_1 Nr. of seq. 1 Alignment length(with gaps) = 99
Alignment score = 0.000000
GCF_000831065.1_ASM83106v1_genomic.fna_1:1522811-1523306 Satlength=496 Nr
of Repeats=5 RepeatLength=99 seed=CGAAGAAGAA Num.seqs=3
Similarity=0.637540 0
CGAAGAAGAACTACATGTAGANAAACTACTAAGCATNAATTTGGAAAAAACGCTAGAAACGTATGCGCAAAGT
GGTGAGATAGCAGAACAAATNGAACG

****
****
Consensus:

CGAAGAAGAACTACATGTAGANAAACTACTAAGCATNAATTTGGAAAAAACGCTAGAAACGTATGCGCAAAGT
GGTGAGATAGCAGAACAAATNGAACG

>Bacillus_Fam_879_93_1 Nr. of seq. 1 Alignment length(with gaps) = 93
Alignment score = 0.000000
GCF_000430785.1_ASM43078v1_genomic.fna_27:11652-12024 Satlength=373 Nr of
Repeats=4 RepeatLength=93 seed=ATTACCCAAC Num.seqs=4 Similarity=0.947431
0
ATTACCCAACTAGAAATGAACCTGCACCTCCAGATACACAACACATAATGCAAATGATGACAAGTCTCGAGGA
TCGATTAAATAAACTCACTA

****
****
Consensus:

ATTACCCAACTAGAAATGAACCTGCACCTCCAGATACACAACACATAATGCAAATGATGACAAGTCTCGAGGA
TCGATTAAATAAACTCACTA

>Bacillus_Fam_880_90_1 Nr. of seq. 1 Alignment length(with gaps) = 90
Alignment score = 0.000000
GCF_000008505.1_ASM850v1_genomic.fna_2:8851-9571 Satlength=721 Nr of
Repeats=7 RepeatLength=90 seed=CGTTCTACTT Num.seqs=6 Similarity=0.952593
0

CGTTCTACTTGCTGACGATCACCTTTTGCCTGATCGTTTGGCACTTCATTTCTTCCTGGGTTATTTATTCGAT
TAGCTACTTCCAATTCT

****
****
Consensus:

CGTTCTACTTGCTGACGATCACCTTTTGCCTGATCGTTTGGCACTTCATTTCTTCCTGGGTTATTTATTCGAT
TAGCTACTTCCAATTCT

>Bacillus_Fam_881_84_1 Nr. of seq. 1 Alignment length(with gaps) = 84
Alignment score = 0.000000
GCF_000008505.1_ASM850v1_genomic.fna_2:8167-8587 Satlength=421 Nr of
Repeats=5 RepeatLength=84 seed=ACTTCTCGTT Num.seqs=5 Similarity=0.876863
0
ACTTCTCGTTGAACTGTCGTTCCATTTACGCTCACGTTTCCGCTATTTACTGATCCTTGATTCTTCACATTAT
CCTGTACGTTC

****
****
Consensus:

ACTTCTCGTTGAACTGTCGTTCCATTTACGCTCACGTTTCCGCTATTTACTGATCCTTGATTCTTCACATTAT
CCTGTACGTTC

>Bacillus_Fam_882_84_1 Nr. of seq. 1 Alignment length(with gaps) = 84
Alignment score = 0.000000
GCF_000831065.1_ASM83106v1_genomic.fna_1:918773-919151 Satlength=379 Nr
of Repeats=5 RepeatLength=84 seed=AATTAGCAGA Num.seqs=3
Similarity=0.862434
0
AATTAGCAGATGGCTCAAGTCAAGTAACAGGCGGTTTAGGAACATTATCTGTAGGAGCAAATCAAATGGCAGG
TGGAGTAAATC

****
****
Consensus:

AATTAGCAGATGGCTCAAGTCAAGTAACAGGCGGTTTAGGAACATTATCTGTAGGAGCAAATCAAATGGCAGG
TGGAGTAAATC

>Bacillus_Fam_883_84_1 Nr. of seq. 1 Alignment length(with gaps) = 84
Alignment score = 0.000000
GCF_001712755.1_ASM171275v1_genomic.fna_23:202276-202863 Satlength=588 Nr
of Repeats=7 RepeatLength=84 seed=AACTCTTTCT Num.seqs=6
Similarity=0.671318 0
AACTCTTTCTTTGCGCATTCATCAATTACTTCTTCGCTAATAAATGGTGCCACNCNTGTTAATCCTTTAATAC
CATTCGTTTCA

****
****
Consensus:

AACTCTTTCTTTGCGCATTCATCAATTACTTCTTCGCTAATAAATGGTGCCACNCNTGTTAATCCTTTAATAC
CATTCGTTTCA

>Bacillus_Fam_884_84_1 Nr. of seq. 1 Alignment length(with gaps) = 84
Alignment score = 0.000000
GCF_002019765.1_ASM201976v1_genomic.fna_1:1293407-1293689 Satlength=283
Nr of Repeats=4 RepeatLength=84 seed=AAGCTGTGGA Num.seqs=3
Similarity=0.947090 0
AAGCTGTGGAACAAGAAGACACAGAATCGAAACAAGCTGATGCAGAAATCGCTGTTCAAGAAAAGGAAGAGCT
AAAACAAGTTG

****
****
Consensus:

AAGCTGTGGAACAAGAAGACACAGAATCGAAACAAGCTGATGCAGAAATCGCTGTTCAAGAAAAGGAAGAGCT
AAAACAAGTTG

>Bacillus_Fam_885_81_1 Nr. of seq. 1 Alignment length(with gaps) = 81
Alignment score = 0.000000
GCF_001315085.1_ASM131508v1_genomic.fna_36:14169-14493 Satlength=325 Nr
of Repeats=4 RepeatLength=81 seed=ACTCAGGTTG Num.seqs=4
Similarity=0.895748
0
ACTCAGGTTGGTTGACTTGGTCATTAAGATCTTCAATAACTTCTGTGATTGGAGCAGAGTCAATAGGTATTTC
TGAACCTG

****
****
Consensus:

ACTCAGGTTGGTTGACTTGGTCATTAAGATCTTCAATAACTTCTGTGATTGGAGCAGAGTCAATAGGTATTTC
TGAACCTG

>Bacillus_Fam_886_78_1 Nr. of seq. 1 Alignment length(with gaps) = 78
Alignment score = 0.000000
GCF_001420715.1_ASM142071v1_genomic.fna_22:1482589-1482958 Satlength=370
Nr of Repeats=5 RepeatLength=78 seed=AGGAGCAAGA Num.seqs=3
Similarity=0.886040 0
AGGAGCAAGAATCGGAAGAAGAAGTAGCATTAAATTCTGATGCAAATCAACCAGAAGTAGAAGAACAATCACT
GCAAG

****
*****
Consensus:

AGGAGCAAGAATCGGAAGAAGAAGTAGCATTAAATTCTGATGCAAATCAACCAGAAGTAGAAGAACAATCACT
GCAAG

>Bacillus_Fam_887_78_1 Nr. of seq. 1 Alignment length(with gaps) = 78
Alignment score = 0.000000
GCF_900107275.1_IMG-
taxon_2675903024_annotated_assembly_genomic.fna_42:634601-635147

Satlength=547 Nr of Repeats=7 RepeatLength=78 seed=CAGAGAACAA Num.seqs=7 Similarity=0.931624 0
CAGAGAACAATCAGGAAGGAACAGCGAAAACAGTGTTGGCAGAAAGTAATCAGGCAGGAGCATCAGAACCAGC GTCGT

****
Consensus:

CAGAGAACAATCAGGAAGGAACAGCGAAAACAGTGTTGGCAGAAAGTAATCAGGCAGGAGCATCAGAACCAGC GTCGT

>Bacillus_Fam_888_76_1 Nr. of seq. 1 Alignment length(with gaps) = 76 Alignment score = 0.000000
GCF_001700275.1_ASM170027v1_genomic.fna_23:136895-137354 Satlength=460 Nr of Repeats=6 RepeatLength=76 seed=TGAAGCTTGA Num.seqs=5
Similarity=0.957895 0
TGAAGCTTGACGCGAAAAGATGAAGTAAAACGTTGATATAAGGGATAGAACTGCGCATAGCTTGACGTAAGCA ACG

****
Consensus:

TGAAGCTTGACGCGAAAAGATGAAGTAAAACGTTGATATAAGGGATAGAACTGCGCATAGCTTGACGTAAGCA ACG

>Bacillus_Fam_889_75_1 Nr. of seq. 1 Alignment length(with gaps) = 75 Alignment score = 0.000000
GCF_001273755.1_ASM127375v1_genomic.fna_17:1713467-1714367 Satlength=901 Nr of Repeats=12 RepeatLength=75 seed=TATTATCTGG Num.seqs=12
Similarity=0.997037 0
TATTATCTGGGTCAGTTGCATCTTTAGGGTCTTCAATCGGATCTTCTTTATTAGTGCCCCCATCATTTTCATC GA

****
Consensus:

TATTATCTGGGTCAGTTGCATCTTTAGGGTCTTCAATCGGATCTTCTTTATTAGTGCCCCCATCATTTTCATC GA

>Bacillus_Fam_890_75_1 Nr. of seq. 1 Alignment length(with gaps) = 75 Alignment score = 0.000000
GCF_002019705.1_ASM201970v1_genomic.fna_1:1610549-1611374 Satlength=826 Nr of Repeats=11 RepeatLength=75 seed=GAGCAAGATT Num.seqs=11
Similarity=0.929535 0
GAGCAAGATTCTGATTCCTCTCTAGATGAAGATGTTTCTCTTGATAACGACGCTTCTGAAGACCAGGATGCAG AT

****
Consensus:

GAGCAAGATTCTGATTCCTCTCTAGATGAAGATGTTTCTCTTGATAACGACGCTTCTGAAGACCAGGATGCAG
AT

>Bacillus_Fam_891_74_1 Nr. of seq. 1 Alignment length(with gaps) = 74
Alignment score = 0.000000
GCF_001591485.1_ASM159148v1_genomic.fna_259:34-767 Satlength=734 Nr of
Repeats=10 RepeatLength=73 seed=CTTTCAATCC Num.seqs=6
Similarity=0.458642
0
CTTTCAATCCCCTAAATCGGGTCACATCTTTTGCTACGANNTATGAAGATTCGTTAANAATCTAACAGTTATT
N

****
*
Consensus:

CTTTCAATCCCCTAAATCGGGTCACATCTTTTGCTACGANNTATGAAGATTCGTTAANAATCTAACAGTTATT
N

>Bacillus_Fam_892_73_1 Nr. of seq. 1 Alignment length(with gaps) = 73
Alignment score = 0.000000
GCF_000331575.1_ASM33157v1_genomic.fna_4:228971-229263 Satlength=293 Nr
of Repeats=4 RepeatLength=73 seed=CACGCAAGTA Num.seqs=4
Similarity=0.978691
0
CACGCAAGTATCACTATGGTAATTTGACGGACATAACTCCACAACGAGATCCCAGAAAGAATCTCTAAAATGA

****
Consensus:

CACGCAAGTATCACTATGGTAATTTGACGGACATAACTCCACAACGAGATCCCAGAAAGAATCTCTAAAATGA

>Bacillus_Fam_893_72_1 Nr. of seq. 1 Alignment length(with gaps) = 72
Alignment score = 0.000000
GCF_000712615.1_ASM71261v1_genomic.fna_39:34707-34986 Satlength=280 Nr of
Repeats=4 RepeatLength=72 seed=CAGTAACACC Num.seqs=3 Similarity=0.618656
0
CAGTAACACCAGTTGCGCCAATCGAAAATGAAGTACCAAATTCAGAAGTGGCCGCTGGGCCAATGTCAACAC

****
Consensus:

CAGTAACACCAGTTGCGCCAATCGAAAATGAAGTACCAAATTCAGAAGTGGCCGCTGGGCCAATGTCAACAC

>Bacillus_Fam_894_70_1 Nr. of seq. 1 Alignment length(with gaps) = 70
Alignment score = 0.000000
GCF_900142675.1_IMG-
taxon_2654588196_annotated_assembly_genomic.fna_5:575590-575991
Satlength=402 Nr of Repeats=6 RepeatLength=66 seed=TGAGTGCGTG Num.seqs=4
Similarity=0.434942 0
TGAGTGCGTGGATTGAAATAACTACGNCNTTGANCAATGCAATATCNCAATTAGCTAGTCGCACTTTTTG

****

Consensus:

TGAGTGCGTGGATTGAAATAACTACGNCNTTGANCAATGCAATATCNCAATTAGCTAGTCGCACTTTTTG

>Bacillus_Fam_895_69_1 Nr. of seq. 1 Alignment length(with gaps) = 69
Alignment score = 0.000000
GCF_000331575.1_ASM33157v1_genomic.fna_3:79046-79529 Satlength=484 Nr of Repeats=7 RepeatLength=69 seed=TAATTTCTGA Num.seqs=7 Similarity=0.994479
0
TAATTTCTGACTTTGTACCTTCAATAGATGTTCCGTTTGTTAATGTTCAGGTAAAGAGAATTATATGGT

****
Consensus:

TAATTTCTGACTTTGTACCTTCAATAGATGTTCCGTTTGTTAATGTTCAGGTAAAGAGAATTATATGGT

>Bacillus_Fam_896_69_1 Nr. of seq. 1 Alignment length(with gaps) = 69
Alignment score = 0.000000
GCF_000508325.1_BAVI_1_genomic.fna_9:15902-17486 Satlength=1585 Nr of Repeats=24 RepeatLength=66 seed=GTCGCACCCT Num.seqs=16 Similarity=0.306590
0
GTCGCACCCTACATGGGTGCGTGGATTGAAATNAANGNGNCAATNAANNCGNATNNTTNATNAGNNNCN

****
Consensus:

GTCGCACCCTACATGGGTGCGTGGATTGAAATNAANGNGNCAATNAANNCGNATNNTTNATNAGNNNCN

>Bacillus_Fam_897_68_1 Nr. of seq. 1 Alignment length(with gaps) = 68
Alignment score = 0.000000
GCF_000948175.1_BFA_2_A_genomic.fna_164:33120-33442 Satlength=323 Nr of Repeats=5 RepeatLength=64 seed=GTTTTATCTG Num.seqs=4 Similarity=0.452635
0 GTTTTATCTGAACGTAGTGGGATATAAAGTTTAANTTCGCATAANAANCTNTNACCTTAGCCGANNNT

****
Consensus:

GTTTTATCTGAACGTAGTGGGATATAAAGTTTAANTTCGCATAANAANCTNTNACCTTAGCCGANNNT

>Bacillus_Fam_898_68_1 Nr. of seq. 1 Alignment length(with gaps) = 68
Alignment score = 0.000000
GCF_900107275.1_IMG-taxon_2675903024_annotated_assembly_genomic.fna_13:136989-137520 Satlength=532 Nr of Repeats=8 RepeatLength=66 seed=GTCGCACTCT Num.seqs=5 Similarity=0.485897 0
GTCGCACTCTATGTGAGTGCGTGGATTGAAATANCTNTTTGNNCCNGNTCAGCNTANNTTNCAGTACN

****
Consensus:

GTCGCACTCTATGTGAGTGCGTGGATTGAAATANCTNTTTGNNCCNGNTCAGCNTANNTTNCAGTACN

>Bacillus_Fam_899_68_1 Nr. of seq. 1 Alignment length(with gaps) = 68
Alignment score = 0.000000
GCF_900107275.1_IMG-
taxon_2675903024_annotated_assembly_genomic.fna_13:161926-162191
Satlength=266 Nr of Repeats=4 RepeatLength=66 seed=TCGCACTCTA Num.seqs=3
Similarity=0.526190 0
TCGCACTCTATATGAGTGCGTGGATTGAAATTCTTTCTNCTNTTTCTTTAGCCGTTCNNCGNTTGCAG

****
Consensus:

TCGCACTCTATATGAGTGCGTGGATTGAAATTCTTTCTNCTNTTTCTTTAGCCGTTCNNCGNTTGCAG

>Bacillus_Fam_900_68_1 Nr. of seq. 1 Alignment length(with gaps) = 68
Alignment score = 0.000000
GCF_900142675.1_IMG-
taxon_2654588196_annotated_assembly_genomic.fna_5:556930-557330
Satlength=401 Nr of Repeats=6 RepeatLength=67 seed=GTCGCACTCT Num.seqs=4
Similarity=0.700980 0
GTCGCACTCTTAGTGAGTGCGTGGATTGAAATNTAATGGCAACTACACCCAAAAGAAAAAGGCAAATC

****
Consensus:

GTCGCACTCTTAGTGAGTGCGTGGATTGAAATNTAATGGCAACTACACCCAAAAGAAAAAGGCAAATC

>Bacillus_Fam_901_68_1 Nr. of seq. 1 Alignment length(with gaps) = 68
Alignment score = 0.000000
GCF_900142675.1_IMG-
taxon_2654588196_annotated_assembly_genomic.fna_5:580013-580479
Satlength=467 Nr of Repeats=7 RepeatLength=66 seed=TGGATTGAAA Num.seqs=5
Similarity=0.446053 0
TGGATTGAAATNNGATTANNCAGNNTTNAATGATTNNAATAATCNNTGTCGCACTCCTTGTGAGTGCG

****
Consensus:

TGGATTGAAATNNGATTANNCAGNNTTNAATGATTNNAATAATCNNTGTCGCACTCCTTGTGAGTGCG

>Bacillus_Fam_902_67_1 Nr. of seq. 1 Alignment length(with gaps) = 67
Alignment score = 0.000000
GCF_000787375.1_ASM78737v1_genomic.fna_72:8860-10166 Satlength=1307 Nr of
Repeats=20 RepeatLength=66 seed=GTCATATCTC Num.seqs=13
Similarity=0.412873
0 GTCATATCTCCATCTAATCCGTTGCTGTGATATGATANNNGCNNTCANCANNTNGATGNTGNNAANN

****
Consensus:

GTCATATCTCCATCTAATCCGTTGCTGTGATATGATANNNGCNNTCANCANNTNGATGNTGNNAANN

>Bacillus_Fam_903_67_1 Nr. of seq. 1 Alignment length(with gaps) = 67
Alignment score = 0.000000

GCF_001375535.1_Bacillus_niameyensis_genomic.fna_6:2530174-2531360 Satlength=1187 Nr of Repeats=18 RepeatLength=66 seed=AAAAACTGAA Num.seqs=16 Similarity=0.346635 0
AAAAACTGAAAGAGTGCCAAAACTANGTTNANATNGTNCATNTTNNTCNANANNGTTTTCCTATACC

****
Consensus:

AAAAACTGAAAGAGTGCCAAAACTANGTTNANATNGTNCATNTTNNTCNANANNGTTTTCCTATACC

>Bacillus_Fam_904_67_1 Nr. of seq. 1 Alignment length(with gaps) = 67 Alignment score = 0.000000
GCF_001587375.1_ASM158737v1_genomic.fna_53:71-465 Satlength=395 Nr of Repeats=6 RepeatLength=66 seed=GGTACTCTCT Num.seqs=4 Similarity=0.532724
0 GGTACTCTCTCAGTTTTCGGTATAGAGAAACAAGTCCACGANTGTGNAAAGANTACGATTNNGTTTT

****
Consensus:

GGTACTCTCTCAGTTTTCGGTATAGAGAAACAAGTCCACGANTGTGNAAAGANTACGATTNNGTTTT

>Bacillus_Fam_905_66_1 Nr. of seq. 1 Alignment length(with gaps) = 66 Alignment score = 0.000000
GCF_000017425.1_ASM1742v1_genomic.fna_1:3154400-3155030 Satlength=631 Nr of Repeats=5 RepeatLength=66 seed=ATCTTTTGAT Num.seqs=3 Similarity=0.905724
0 ATCTTTTGATAATTCGTAGCCCGCTGGTGCTTCTATTTCAACGATTTTGTATTCACCTATCGGTAA

****
Consensus:

ATCTTTTGATAATTCGTAGCCCGCTGGTGCTTCTATTTCAACGATTTTGTATTCACCTATCGGTAA

>Bacillus_Fam_906_66_1 Nr. of seq. 1 Alignment length(with gaps) = 66 Alignment score = 0.000000
GCF_000238675.1_Baci_smit_7_3_47FAA_V1_genomic.fna_17:14392-16174 Satlength=1783 Nr of Repeats=27 RepeatLength=66 seed=ATCATATCAC Num.seqs=25 Similarity=0.325146 0
ATCATATCACAGCAATAATCTTAGGGGAACTATGACNGNTNNTNNNGANNNATNTNNCGNTNNNNN

****
Consensus:

ATCATATCACAGCAATAATCTTAGGGGAACTATGACNGNTNNTNNNGANNNATNTNNCGNTNNNNN

>Bacillus_Fam_907_66_1 Nr. of seq. 1 Alignment length(with gaps) = 66 Alignment score = 0.000000
GCF_000307855.1_BAZO1.0_genomic.fna_92:44514-45037 Satlength=524 Nr of Repeats=8 RepeatLength=65 seed=GTCGCATCCC Num.seqs=5 Similarity=0.490909
0 GTCGCATCCCACGCGGATGCGTGGATTGAAATGNCNANCATGNCGAGATANANCCGNNANTCTNGN

****
Consensus:

GTCGCATCCCACGCGGATGCGTGGATTGAAATGNCNANCATGNCGAGATANANCCGNNANTCTNGN

>Bacillus_Fam_908_66_1 Nr. of seq. 1 Alignment length(with gaps) = 66
Alignment score = 0.000000
GCF_000429705.1_ASM42970v1_genomic.fna_2:243049-243709 Satlength=661 Nr of Repeats=10 RepeatLength=66 seed=GACAGCACCA Num.seqs=10
Similarity=0.846016 0
GACAGCACCACCAACGCTACTGATACTTCCACCGCTACAAGCGGCTCCACCAGCGAGTCCGACACG

****
Consensus:

GACAGCACCACCAACGCTACTGATACTTCCACCGCTACAAGCGGCTCCACCAGCGAGTCCGACACG

>Bacillus_Fam_909_66_1 Nr. of seq. 1 Alignment length(with gaps) = 66
Alignment score = 0.000000
GCF_001274935.1_ASM127493v1_genomic.fna_31:6009-6274 Satlength=266 Nr of Repeats=4 RepeatLength=66 seed=ATCATAATAC Num.seqs=3 Similarity=0.558559
0 ATCATAATACAGCAATGGCTTTAATGGAACTATGACNTACAAATGTANGANAAATATTNGATTTNA

****
Consensus:

ATCATAATACAGCAATGGCTTTAATGGAACTATGACNTACAAATGTANGANAAATATTNGATTTNA

>Bacillus_Fam_910_66_1 Nr. of seq. 1 Alignment length(with gaps) = 66
Alignment score = 0.000000
GCF_900096965.1_IMG-taxon_2671180223_annotated_assembly_genomic.fna_7:104929-105260
Satlength=332 Nr of Repeats=5 RepeatLength=66 seed=GGATTGAAAT Num.seqs=3
Similarity=0.470209 0
GGATTGAAATNNCGNTNGTATGCTNANNAGGTTCGTTGGCNGNCGTCGCATCCCATGTGGGTGCGC

****
Consensus:

GGATTGAAATNNCGNTNGTATGCTNANNAGGTTCGTTGGCNGNCGTCGCATCCCATGTGGGTGCGC

>Bacillus_Fam_911_66_1 Nr. of seq. 1 Alignment length(with gaps) = 66
Alignment score = 0.000000
GCF_900107275.1_IMG-taxon_2675903024_annotated_assembly_genomic.fna_49:6168-6961
Satlength=794 Nr of Repeats=12 RepeatLength=66 seed=GTCGCACTCT
Num.seqs=8 Similarity=0.424771 0
GTCGCACTCTTCATGAGTGCGTGGATTGAAATNTTATANTCNTGATGGTCGAGNGCATNNGATCGN

****
Consensus:

GTCGCACTCTTCATGAGTGCGTGGATTGAAATNTTATANTCNTGATGGTCGAGNGCATNNGATCGN

>Bacillus_Fam_912_65_1 Nr. of seq. 1 Alignment length(with gaps) = 65
Alignment score = 0.000000

GCF_000374345.1_ASM37434v1_genomic.fna_66:11358-11621 Satlength=264 Nr of Repeats=4 RepeatLength=66 seed=ATTTCAATCC Num.seqs=3 Similarity=0.460526
0 ATTTCAATCCTCGCACTCACATGGAGTGCGACCANTAAGTCTATTNCTGTTATGCAAGANNGNCT

****
Consensus:

ATTTCAATCCTCGCACTCACATGGAGTGCGACCANTAAGTCTATTNCTGTTATGCAAGANNGNCT

>Bacillus_Fam_913_65_1 Nr. of seq. 1 Alignment length(with gaps) = 65
Alignment score = 0.000000
GCF_001484965.1_ASM148496v1_genomic.fna_8:75646-77564 Satlength=1919 Nr of Repeats=29 RepeatLength=66 seed=TCCACGCACT Num.seqs=19
Similarity=0.282332 0
TCCACGCACTCACATGGAGTGCGATTACGNCNNANNNATNNCGNNGANAACGTCNGANATTTCAA

****
Consensus:

TCCACGCACTCACATGGAGTGCGATTACGNCNNANNNATNNCGNNGANAACGTCNGANATTTCAA

>Bacillus_Fam_914_65_1 Nr. of seq. 1 Alignment length(with gaps) = 65
Alignment score = 0.000000
GCF_900142675.1_IMG-taxon_2654588196_annotated_assembly_genomic.fna_5:564317-564649
Satlength=333 Nr of Repeats=5 RepeatLength=66 seed=GTCGCACTCT Num.seqs=3
Similarity=0.526636 0
GTCGCACTCTTTGTGAGTGCGTGGATTGAAATAANCATNTTTCTGTAANCGGCAAAATAATNCNG

****
Consensus:

GTCGCACTCTTTGTGAGTGCGTGGATTGAAATAANCATNTTTCTGTAANCGGCAAAATAATNCNG

>Bacillus_Fam_915_63_1 Nr. of seq. 1 Alignment length(with gaps) = 63
Alignment score = 0.000000
GCF_000429705.1_ASM42970v1_genomic.fna_3:200483-201166 Satlength=684 Nr of Repeats=8 RepeatLength=63 seed=TTTTTTCTAC Num.seqs=5
Similarity=0.664646
0 TTTTTTCTACCGCAAAGAGAGATATTCCTACCACAAANAGCAATAATCCTACCGAAAATCCGA

****
Consensus:

TTTTTTCTACCGCAAAGAGAGATATTCCTACCACAAANAGCAATAATCCTACCGAAAATCCGA

>Bacillus_Fam_916_63_1 Nr. of seq. 1 Alignment length(with gaps) = 63
Alignment score = 0.000000
GCF_000430785.1_ASM43078v1_genomic.fna_32:20641-20977 Satlength=337 Nr of Repeats=5 RepeatLength=63 seed=CAAGAAATAG Num.seqs=4 Similarity=0.915344
0 CAAGAAATAGAAAATTTAAATGAAACAATCATCAGTTACAAAACAAAAGAAACTGAACATAAA

****
Consensus:

CAAGAAATAGAAAATTTAAATGAAACAATCATCAGTTACAAAACAAAAGAAACTGAACATAAA

>Bacillus_Fam_917_63_1 Nr. of seq. 1 Alignment length(with gaps) = 63
Alignment score = 0.000000
GCF_000508325.1_BAVI_1_genomic.fna_19:13794-14166 Satlength=373 Nr of Repeats=4 RepeatLength=63 seed=AATACCATTT Num.seqs=3 Similarity=0.823633
0   AATACCATTTTNCNCCNGATTGAACCCATGCTTTTGTTACCATAGCTCCATCAGCACCTAAGT

****
Consensus:

AATACCATTTTNCNCCNGATTGAACCCATGCTTTTGTTACCATAGCTCCATCAGCACCTAAGT

>Bacillus_Fam_918_63_1 Nr. of seq. 1 Alignment length(with gaps) = 63
Alignment score = 0.000000
GCF_000751775.1_A1A_genomic.fna_22:47265-47558 Satlength=294 Nr of Repeats=5 RepeatLength=63 seed=TTTGTCCCAT Num.seqs=3 Similarity=0.887125
0   TTTGTCCCATAGACCGTGAATCCGGGAATTCTATGGGACATTTTATTTTACTTTTCAACTAAA

****
Consensus:

TTTGTCCCATAGACCGTGAATCCGGGAATTCTATGGGACATTTTATTTTACTTTTCAACTAAA

>Bacillus_Fam_919_63_1 Nr. of seq. 1 Alignment length(with gaps) = 63
Alignment score = 0.000000
GCF_001274935.1_ASM127493v1_genomic.fna_16:22732-23194 Satlength=463 Nr of Repeats=5 RepeatLength=63 seed=TAGGAAAACT Num.seqs=4
Similarity=0.841270
0   TAGGAAAACTATCTGACGGATCTACTCAGCTAGCAACAGGAAAAGGCGATTTATCAAATGGTT

****
Consensus:

TAGGAAAACTATCTGACGGATCTACTCAGCTAGCAACAGGAAAAGGCGATTTATCAAATGGTT

>Bacillus_Fam_920_63_1 Nr. of seq. 1 Alignment length(with gaps) = 63
Alignment score = 0.000000
GCF_001587375.1_ASM158737v1_genomic.fna_12:70-2906 Satlength=2837 Nr of Repeats=44 RepeatLength=66 seed=AGAGTACCAA Num.seqs=38
Similarity=0.266813
0   AGAGTACCAAAACNTNNNNANANNNNTNNGNTANTANGTNGTTTCTCTATACCGAAAACTGAG

****
Consensus:

AGAGTACCAAAACNTNNNNANANNNNTNNGNTANTANGTNGTTTCTCTATACCGAAAACTGAG

>Bacillus_Fam_921_63_1 Nr. of seq. 1 Alignment length(with gaps) = 63
Alignment score = 0.000000
GCF_002019595.1_ASM201959v1_genomic.fna_1:3679532-3679910 Satlength=379 Nr of Repeats=6 RepeatLength=63 seed=GAAAAGAGC Num.seqs=6

Similarity=0.646875 0
GAAAAAGAGCAGCAAGCCAGNCTTACCATCCAAGAGCTGGAGAACAAAATCCAACACTCTTTG

****
Consensus:

GAAAAAGAGCAGCAAGCCAGNCTTACCATCCAAGAGCTGGAGAACAAAATCCAACACTCTTTG

>Bacillus_Fam_922_62_1 Nr. of seq. 1 Alignment length(with gaps) = 62
Alignment score = 0.000000
GCF_000374345.1_ASM37434v1_genomic.fna_18:23841-24079 Satlength=239 Nr of Repeats=4 RepeatLength=62 seed=ATGAAAGCCA Num.seqs=3 Similarity=0.600694
0 ATGAAAGCCATNATGAAGGAAAAACGCGGGTNAANTNGCTTTCATCTGTAAAAAAGATCCAA

****
Consensus:

ATGAAAGCCATNATGAAGGAAAAACGCGGGTNAANTNGCTTTCATCTGTAAAAAAGATCCAA

>Bacillus_Fam_923_61_1 Nr. of seq. 1 Alignment length(with gaps) = 61
Alignment score = 0.000000
GCF_001645685.2_ASM164568v2_genomic.fna_1:1321762-1322553 Satlength=792 Nr of Repeats=13 RepeatLength=61 seed=AATCGAAACA Num.seqs=11
Similarity=0.861302 0
AATCGAAACAAGGACGAAATACTCCAATGTAGGCCGAATTAATAGCAGTCAAGGCCGGATT

****
Consensus:

AATCGAAACAAGGACGAAATACTCCAATGTAGGCCGAATTAATAGCAGTCAAGGCCGGATT

>Bacillus_Fam_924_60_1 Nr. of seq. 1 Alignment length(with gaps) = 60
Alignment score = 0.000000
GCF_000161455.1_ASM16145v1_genomic.fna_1:1619478-1620237 Satlength=760 Nr of Repeats=7 RepeatLength=60 seed=AATACCAAGT Num.seqs=5
Similarity=0.661376 0
AATACCAAGTAGATCCTTCTTTTAACCAGCCAGTAGCCATAGAACCATTTGCATTTAGAT

****
Consensus:

AATACCAAGTAGATCCTTCTTTTAACCAGCCAGTAGCCATAGAACCATTTGCATTTAGAT

>Bacillus_Fam_925_60_1 Nr. of seq. 1 Alignment length(with gaps) = 60
Alignment score = 0.000000
GCF_000496285.1_ASM49628v1_genomic.fna_1:3811091-3811391 Satlength=301 Nr of Repeats=4 RepeatLength=60 seed=TGGTATTACT Num.seqs=3
Similarity=0.644444 0
TGGTATTACTTGAAAGAAAATGGATCNATGATGACAGGTTGGACGTTAGTAAATGGTNAG

****
Consensus:

TGGTATTACTTGAAAGAAAATGGATCNATGATGACAGGTTGGACGTTAGTAAATGGTNAG

>Bacillus_Fam_926_60_1 Nr. of seq. 1 Alignment length(with gaps) = 60
Alignment score = 0.000000
GCF_000496285.1_ASM49628v1_genomic.fna_1:3813346-3813826 Satlength=481 Nr of Repeats=7 RepeatLength=60 seed=TGGTATTATT Num.seqs=6 Similarity=0.575788 0
TGGTATTATTTAAACGCTAATGGAGCAATGAAAACGGGCTGGCTATTANATCAAGGAGTT

****
Consensus:

TGGTATTATTTAAACGCTAATGGAGCAATGAAAACGGGCTGGCTATTANATCAAGGAGTT

>Bacillus_Fam_927_60_1 Nr. of seq. 1 Alignment length(with gaps) = 60
Alignment score = 0.000000
GCF_000712595.1_ASM71259v1_genomic.fna_18:41788-42208 Satlength=421 Nr of Repeats=7 RepeatLength=60 seed=TAGTACCAAG Num.seqs=7 Similarity=0.572161
0   TAGTACCAAGTATTANCAAGNTGAAGCCATCCNGTATGCATNNCACCGTTATTNTTTAAN

****
Consensus:

TAGTACCAAGTATTANCAAGNTGAAGCCATCCNGTATGCATNNCACCGTTATTNTTTAAN

>Bacillus_Fam_928_60_1 Nr. of seq. 1 Alignment length(with gaps) = 60
Alignment score = 0.000000
GCF_000712615.1_ASM71261v1_genomic.fna_59:11461-11761 Satlength=301 Nr of Repeats=5 RepeatLength=60 seed=ATTTACATTT Num.seqs=5 Similarity=0.769312
0   ATTTACATTTTTCTTACATTCACTCTCTTGTGATACCCTTGCTTTTACCTCATCCTTTGA

****
Consensus:

ATTTACATTTTTCTTACATTCACTCTCTTGTGATACCCTTGCTTTTACCTCATCCTTTGA

>Bacillus_Fam_929_60_1 Nr. of seq. 1 Alignment length(with gaps) = 60
Alignment score = 0.000000
GCF_000832605.1_ASM83260v1_genomic.fna_2:284188-284428 Satlength=241 Nr of Repeats=4 RepeatLength=60 seed=ATCACAACAC Num.seqs=4 Similarity=0.933333
0   ATCACAACACCACAGTCAGGAACAACACCATCACCAGCGAATCCGATGGAAAGTAAGCCA

****
Consensus:

ATCACAACACCACAGTCAGGAACAACACCATCACCAGCGAATCCGATGGAAAGTAAGCCA

>Bacillus_Fam_930_60_1 Nr. of seq. 1 Alignment length(with gaps) = 60
Alignment score = 0.000000
GCF_001439915.1_ASM143991v1_genomic.fna_105:204636-204873 Satlength=238 Nr of Repeats=4 RepeatLength=60 seed=CCCAACCAGT Num.seqs=3 Similarity=0.664903 0
CCCAACCAGTAGCCATCGCACCACTAGGATGCATGAAGTACCAGGTGCTCCCATCNNNTA

****
Consensus:

CCCAACCAGTAGCCATCGCACCACTAGGATGCATGAAGTACCAGGTGCTCCCATCNNNTA

>Bacillus_Fam_931_60_1 Nr. of seq. 1 Alignment length(with gaps) = 60
Alignment score = 0.000000
GCF_001591485.1_ASM159148v1_genomic.fna_100:1375-1615 Satlength=241 Nr of Repeats=4 RepeatLength=60 seed=AGGTAGTACC Num.seqs=4 Similarity=0.685792
0   AGGTAGTACCACTTCCCACCTGTATAAAGCCAACCGGTCTTCATCGAGCCATTACTAGTC

****
Consensus:

AGGTAGTACCACTTCCCACCTGTATAAAGCCAACCGGTCTTCATCGAGCCATTACTAGTC

>Bacillus_Fam_932_60_1 Nr. of seq. 1 Alignment length(with gaps) = 60
Alignment score = 0.000000
GCF_001591485.1_ASM159148v1_genomic.fna_130:5865-6531 Satlength=667 Nr of Repeats=5 RepeatLength=60 seed=TCAGCCAGCC Num.seqs=3 Similarity=0.336650
0   TCAGCCAGCCNTTNTGAATNAGACCGTTTGATNCAAAGTAATANTGCTNNNATCCCTNGG

****
Consensus:

TCAGCCAGCCNTTNTGAATNAGACCGTTTGATNCAAAGTAATANTGCTNNNATCCCTNGG

>Bacillus_Fam_933_60_1 Nr. of seq. 1 Alignment length(with gaps) = 60
Alignment score = 0.000000
GCF_001591805.1_ASM159180v1_genomic.fna_49:31667-31967 Satlength=301 Nr of Repeats=5 RepeatLength=60 seed=AAGAGCGGAG Num.seqs=3
Similarity=0.911111
0   AAGAGCGGAGCCATGCAAACGGGCTGGCTGAAAGACGGATCGAAATGGTACTACCTCGGT

****
Consensus:

AAGAGCGGAGCCATGCAAACGGGCTGGCTGAAAGACGGATCGAAATGGTACTACCTCGGT

>Bacillus_Fam_934_60_1 Nr. of seq. 1 Alignment length(with gaps) = 60
Alignment score = 0.000000
GCF_001712755.1_ASM171275v1_genomic.fna_3:55980-56220 Satlength=241 Nr of Repeats=4 RepeatLength=60 seed=GGAGTAATGA Num.seqs=4 Similarity=0.769841
0   GGAGTAATGAAAACTAGTTGGTTAGTATTAGGTAATAAACATTATTACTTTGATAAATAT

****
Consensus:

GGAGTAATGAAAACTAGTTGGTTAGTATTAGGTAATAAACATTATTACTTTGATAAATAT

>Bacillus_Fam_935_60_1 Nr. of seq. 1 Alignment length(with gaps) = 60
Alignment score = 0.000000

GCF_900109925.1_IMG-
taxon_2651870116_annotated_assembly_genomic.fna_16:475148-475388
Satlength=241 Nr of Repeats=4 RepeatLength=60 seed=TGACGGTAGC Num.seqs=4
Similarity=0.933333 0
TGACGGTAGCCATGAGGAGGCAGGACATGAAGACGAAAATCATGAAGCTGACGCTCATGG

****
Consensus:

TGACGGTAGCCATGAGGAGGCAGGACATGAAGACGAAAATCATGAAGCTGACGCTCATGG

>Bacillus_Fam_936_58_1 Nr. of seq. 1 Alignment length(with gaps) = 58
Alignment score = 0.000000
GCF_001591485.1_ASM159148v1_genomic.fna_14:19820-20120 Satlength=301 Nr of Repeats=5 RepeatLength=60 seed=TGGTATTATT Num.seqs=5
Similarity=0.504040
0 TGGTATTATTTTGATNNNACCGGTGCAATGAAAACAGGNTGGCTGCTATNGTGGCAAN

****
Consensus:

TGGTATTATTTTGATNNNACCGGTGCAATGAAAACAGGNTGGCTGCTATNGTGGCAAN

>Bacillus_Fam_937_58_1 Nr. of seq. 1 Alignment length(with gaps) = 58
Alignment score = 0.000000
GCF_900107275.1_IMG-
taxon_2675903024_annotated_assembly_genomic.fna_42:885338-885570
Satlength=233 Nr of Repeats=4 RepeatLength=58 seed=AGGATGGCGA Num.seqs=4
Similarity=1.000000 0
AGGATGGCGACATTCCGCTGTTACTCACAGGACGTGAGTGGTACTTAAGCGGAATTGG

****
Consensus:

AGGATGGCGACATTCCGCTGTTACTCACAGGACGTGAGTGGTACTTAAGCGGAATTGG

>Bacillus_Fam_938_57_1 Nr. of seq. 1 Alignment length(with gaps) = 57
Alignment score = 0.000000
GCF_000292245.2_ASM29224v2_genomic.fna_16:66119-66404 Satlength=286 Nr of Repeats=5 RepeatLength=57 seed=TTTCCAATGC Num.seqs=5 Similarity=0.887719
0 TTTCCAATGCCAAGCCACCATGGAGGAATGATGCCAATGCCAGAGCAAAGTGGAGGA

****
Consensus:

TTTCCAATGCCAAGCCACCATGGAGGAATGATGCCAATGCCAGAGCAAAGTGGAGGA

>Bacillus_Fam_939_57_1 Nr. of seq. 1 Alignment length(with gaps) = 57
Alignment score = 0.000000
GCF_000321185.1_ASM32118v1_genomic.fna_73:59292-59609 Satlength=318 Nr of Repeats=5 RepeatLength=57 seed=AATGACTCAT Num.seqs=3 Similarity=0.953216
0 AATGACTCATAAATACTCGATTCCTTTTTTATCATGGATTCTTCAGCTGGTCTTATT

****

Consensus:

AATGACTCATAAATACTCGATTCCTTTTTTATCATGGATTCTTCAGCTGGTCTTATT

>Bacillus_Fam_940_57_1 Nr. of seq. 1 Alignment length(with gaps) = 57
Alignment score = 0.000000
GCF_000712615.1_ASM71261v1_genomic.fna_6:87271-88026 Satlength=756 Nr of Repeats=7 RepeatLength=57 seed=CAAATGTTGG Num.seqs=5 Similarity=0.985965
0 CAAATGTTGGAAATGTAGTTGGTGCTCCTACTGGAACTCCGCCTTGCGCCCCTCCTA

****
Consensus:

CAAATGTTGGAAATGTAGTTGGTGCTCCTACTGGAACTCCGCCTTGCGCCCCTCCTA

>Bacillus_Fam_941_57_1 Nr. of seq. 1 Alignment length(with gaps) = 57
Alignment score = 0.000000
GCF_000775975.1_ASM77597v1_genomic.fna_1:2705156-2705429 Satlength=274 Nr of Repeats=6 RepeatLength=57 seed=GGGCCTACTG Num.seqs=4
Similarity=0.910331 0
GGGCCTACTGGACCTACCGGAATAACTGGAGCAACTGGACCTTCTGGTGGACCTCCA

****
Consensus:

GGGCCTACTGGACCTACCGGAATAACTGGAGCAACTGGACCTTCTGGTGGACCTCCA

>Bacillus_Fam_942_56_1 Nr. of seq. 1 Alignment length(with gaps) = 56
Alignment score = 0.000000
GCF_001587375.1_ASM158737v1_genomic.fna_120:58369-58681 Satlength=313 Nr of Repeats=6 RepeatLength=52 seed=TTTGGAGGTA Num.seqs=4
Similarity=0.625000
0 TTTGGAGGTACTTCCTTTTAGCTATTCGCTAGCATGATGTGACNTCATGACACTCT

****
Consensus:

TTTGGAGGTACTTCCTTTTAGCTATTCGCTAGCATGATGTGACNTCATGACACTCT

>Bacillus_Fam_943_55_1 Nr. of seq. 1 Alignment length(with gaps) = 55
Alignment score = 0.000000
GCF_000217835.1_ASM21783v1_genomic.fna_1:697043-697355 Satlength=313 Nr of Repeats=6 RepeatLength=52 seed=GTCTTCCTTA Num.seqs=6
Similarity=0.674206
0 GTCTTCCTTAACNTAGANGCGNTTCTACGTTAAGATACCCGGAAAAATTCCGANG

****
Consensus:

GTCTTCCTTAACNTAGANGCGNTTCTACGTTAAGATACCCGGAAAAATTCCGANG

>Bacillus_Fam_944_55_1 Nr. of seq. 1 Alignment length(with gaps) = 55
Alignment score = 0.000000

GCF_000331575.1_ASM33157v1_genomic.fna_1:1650370-1650647 Satlength=278 Nr of Repeats=5 RepeatLength=55 seed=GGACCATGAT Num.seqs=3
Similarity=0.903030 0
GGACCATGATTAAGCTGCTATTTGTTGGTAGTTTTATACATAGCACTTGATTCGC

****
Consensus:

GGACCATGATTAAGCTGCTATTTGTTGGTAGTTTTATACATAGCACTTGATTCGC

>Bacillus_Fam_945_55_1 Nr. of seq. 1 Alignment length(with gaps) = 55
Alignment score = 0.000000
GCF_001420715.1_ASM142071v1_genomic.fna_22:511669-512190 Satlength=522 Nr of Repeats=11 RepeatLength=52 seed=ATGAAGGACA Num.seqs=8
Similarity=0.624362 0
ATGAAGGACAANTTGAAGGAAAGAATCGAAGGAAAATCAGTCCTTCATCGGAAGT

****
Consensus:

ATGAAGGACAANTTGAAGGAAAGAATCGAAGGAAAATCAGTCCTTCATCGGAAGT

>Bacillus_Fam_946_55_1 Nr. of seq. 1 Alignment length(with gaps) = 55
Alignment score = 0.000000
GCF_001636315.1_ASM163631v1_genomic.fna_1:1907579-1907897 Satlength=319 Nr of Repeats=6 RepeatLength=53 seed=CTTTATTTTG Num.seqs=6
Similarity=0.823434 0
CTTTATTTTGACCGGTGACCTCCTTCTTGGTACCTTTGCGGGAAGAATCNGACGN

****
Consensus:

CTTTATTTTGACCGGTGACCTCCTTCTTGGTACCTTTGCGGGAAGAATCNGACGN

>Bacillus_Fam_947_55_1 Nr. of seq. 1 Alignment length(with gaps) = 55
Alignment score = 0.000000
GCF_001636315.1_ASM163631v1_genomic.fna_1:2333462-2333670 Satlength=209 Nr of Repeats=4 RepeatLength=52 seed=CTCATGAACC Num.seqs=4
Similarity=0.606061 0
CTCATGAACCCTCTATGAGAACAATTGCNAGCACCCTCCTTCANCCCAGTCTGTC

****
Consensus:

CTCATGAACCCTCTATGAGAACAATTGCNAGCACCCTCCTTCANCCCAGTCTGTC

>Bacillus_Fam_948_54_1 Nr. of seq. 1 Alignment length(with gaps) = 54
Alignment score = 0.000000
GCF_000169195.2_ASM16919v2_genomic.fna_1:140555-140771 Satlength=217 Nr of Repeats=4 RepeatLength=54 seed=CCCGTCTTTC Num.seqs=4
Similarity=0.786008
0 CCCGTCTTTCAGCATGATGTTCAGGCAAATCCGGGGGAACACGGCGCTCTATTT

****

Consensus:

CCCGTCTTTCAGCATGATGTTCAGGCAAATCCGGGGGAACACGGCGCTCTATTT

>Bacillus_Fam_949_54_1 Nr. of seq. 1 Alignment length(with gaps) = 54 Alignment score = 0.000000
GCF_000169195.2_ASM16919v2_genomic.fna_1:451571-451779 Satlength=209 Nr of Repeats=4 RepeatLength=52 seed=TGTCTTTCAT Num.seqs=4 Similarity=0.507937
0 TGTCTTTCATCANACCGATGAAGGACAAATAGCTACCAGCCACNCCTTGTAAAA

****
Consensus:

TGTCTTTCATCANACCGATGAAGGACAAATAGCTACCAGCCACNCCTTGTAAAA

>Bacillus_Fam_950_54_1 Nr. of seq. 1 Alignment length(with gaps) = 54 Alignment score = 0.000000
GCF_000708755.2_ASM70875v2_genomic.fna_2:1546077-1546501 Satlength=425 Nr of Repeats=8 RepeatLength=53 seed=TTCAGTGCCA Num.seqs=8 Similarity=0.784832 0
TTCAGTGCCATCAAAACCGATTCCGGACGCTTCTTGAGGGTTCTTATCCTGNTG

****
Consensus:

TTCAGTGCCATCAAAACCGATTCCGGACGCTTCTTGAGGGTTCTTATCCTGNTG

>Bacillus_Fam_951_54_1 Nr. of seq. 1 Alignment length(with gaps) = 54 Alignment score = 0.000000
GCF_000712595.1_ASM71259v1_genomic.fna_14:27849-28929 Satlength=1081 Nr of Repeats=20 RepeatLength=54 seed=ACAAAGACTT Num.seqs=20 Similarity=0.765132 0
ACAAAGACTTCCGGCTCTTCTTGGNTTTCCTCTACAACTTCTGCTTCAGGTTCC

****
Consensus:

ACAAAGACTTCCGGCTCTTCTTGGNTTTCCTCTACAACTTCTGCTTCAGGTTCC

>Bacillus_Fam_952_54_1 Nr. of seq. 1 Alignment length(with gaps) = 54 Alignment score = 0.000000
GCF_000712615.1_ASM71261v1_genomic.fna_77:5423-5639 Satlength=217 Nr of Repeats=4 RepeatLength=54 seed=ATTGTTGGAA Num.seqs=4 Similarity=0.897119
0 ATTGTTGGAAACACAAACATCACGATGATTGTTCAAAACATAAACATCATTGTG

****
Consensus:

ATTGTTGGAAACACAAACATCACGATGATTGTTCAAAACATAAACATCATTGTG

>Bacillus_Fam_953_54_1 Nr. of seq. 1 Alignment length(with gaps) = 54 Alignment score = 0.000000

GCF_000775975.1_ASM77597v1_genomic.fna_1:2007949-2008246 Satlength=298 Nr of Repeats=6 RepeatLength=54 seed=AGAGAAAGAA Num.seqs=5
Similarity=0.690476 0
AGAGAAAGAAAGTGAAGAAACTGAATCAAAAAAAGAAGATAAAGCAATTGAATT

****
Consensus:

AGAGAAAGAAAGTGAAGAAACTGAATCAAAAAAAGAAGATAAAGCAATTGAATT

>Bacillus_Fam_954_54_1 Nr. of seq. 1 Alignment length(with gaps) = 54
Alignment score = 0.000000
GCF_000986785.1_ASM98678v1_genomic.fna_101:238687-238999 Satlength=313 Nr of Repeats=5 RepeatLength=52 seed=CTGACAGCCC Num.seqs=4
Similarity=0.763375 0
CTGACAGCCCTCCTCAGTGCCCGTTTCTTGGGAATAGGAAACCATTTTAGGGCT

****
Consensus:

CTGACAGCCCTCCTCAGTGCCCGTTTCTTGGGAATAGGAAACCATTTTAGGGCT

>Bacillus_Fam_955_54_1 Nr. of seq. 1 Alignment length(with gaps) = 54
Alignment score = 0.000000
GCF_001243895.1_Bacillus_testis_genomic.fna_9:434776-435244 Satlength=469 Nr of Repeats=5 RepeatLength=54 seed=ATGGGGCCAA Num.seqs=4
Similarity=0.864198 0
ATGGGGCCAACAGGAGCCACCGGATTAGCAGGAGCAACCGGGCCAGCAGGAGTA

****
Consensus:

ATGGGGCCAACAGGAGCCACCGGATTAGCAGGAGCAACCGGGCCAGCAGGAGTA

>Bacillus_Fam_956_54_1 Nr. of seq. 1 Alignment length(with gaps) = 54
Alignment score = 0.000000
GCF_001375535.1_Bacillus_niameyensis_genomic.fna_6:201591-201847 Satlength=257 Nr of Repeats=5 RepeatLength=51 seed=TTTGTCTCTG Num.seqs=4
Similarity=0.598765 0
TTTGTCTCTGAGCACGTCTATCAGAGACATTAATTATCCAATTAAAAGCGAAGA

****
Consensus:

TTTGTCTCTGAGCACGTCTATCAGAGACATTAATTATCCAATTAAAAGCGAAGA

>Bacillus_Fam_957_54_1 Nr. of seq. 1 Alignment length(with gaps) = 54
Alignment score = 0.000000
GCF_002019705.1_ASM201970v1_genomic.fna_1:3847198-3848008 Satlength=811 Nr of Repeats=14 RepeatLength=54 seed=TAAGCCAGTA Num.seqs=9
Similarity=1.000000 0
TAAGCCAGTAGCGCCAGTAGGCCCAGGATCGCCGGTGGCCCCAGTAGCCCCAGC

****

Consensus:

TAAGCCAGTAGCGCCAGTAGGCCCAGGATCGCCGGTGGCCCCAGTAGCCCCAGC

>Bacillus_Fam_958_54_1 Nr. of seq. 1 Alignment length(with gaps) = 54
Alignment score = 0.000000
GCF_900107275.1_IMG-
taxon_2675903024_annotated_assembly_genomic.fna_32:122431-122863
Satlength=433 Nr of Repeats=8 RepeatLength=54 seed=CTCAGCCAGC Num.seqs=8
Similarity=0.973545 0
CTCAGCCAGCAGCTCGGTTTCTTCTGCAGCCTCTTCGAGTAATTCTGTCTCATT

****
Consensus:

CTCAGCCAGCAGCTCGGTTTCTTCTGCAGCCTCTTCGAGTAATTCTGTCTCATT

>Bacillus_Fam_959_54_1 Nr. of seq. 1 Alignment length(with gaps) = 54
Alignment score = 0.000000
GCF_900109925.1_IMG-
taxon_2651870116_annotated_assembly_genomic.fna_26:45696-46032
Satlength=337 Nr of Repeats=4 RepeatLength=54 seed=AAAGCAGAAG Num.seqs=3
Similarity=0.983539 0
AAAGCAGAAGAGCAACAGCGTGAAGCATTGAAGAAACAAGAAGAGGCCCGTAAA

****
Consensus:

AAAGCAGAAGAGCAACAGCGTGAAGCATTGAAGAAACAAGAAGAGGCCCGTAAA

>Bacillus_Fam_960_53_1 Nr. of seq. 1 Alignment length(with gaps) = 53
Alignment score = 0.000000
BACILLUS_PSEUDALCALIPHILUS_genomic.fna_3:207312-207737 Satlength=426 Nr of
Repeats=8 RepeatLength=53 seed=GGTCGACATG Num.seqs=7 Similarity=0.892183
0 GGTCGACATGATCGAGGAGAAAATAGAGAAAGAACAGGTCGATGAGTAGCCGA

****
Consensus:

GGTCGACATGATCGAGGAGAAAATAGAGAAAGAACAGGTCGATGAGTAGCCGA

>Bacillus_Fam_961_53_1 Nr. of seq. 1 Alignment length(with gaps) = 53
Alignment score = 0.000000
GCF_000153365.1_ASM15336v1_genomic.fna_16:88973-89184 Satlength=212 Nr of
Repeats=4 RepeatLength=53 seed=ATATGAGTCA Num.seqs=3 Similarity=0.740042
0 ATATGAGTCACTNTGGTGGTGGNGAAAGCCGNACAGAGTGACTCAAGAGGAAG

****
Consensus:

ATATGAGTCACTNTGGTGGTGGNGAAAGCCGNACAGAGTGACTCAAGAGGAAG

>Bacillus_Fam_962_53_1 Nr. of seq. 1 Alignment length(with gaps) = 53
Alignment score = 0.000000

GCF_000169195.2_ASM16919v2_genomic.fna_1:2227978-2228290 Satlength=313 Nr of Repeats=6 RepeatLength=52 seed=GCTTCCTTAA Num.seqs=6 Similarity=0.792243 0
GCTTCCTTAACATAGAAGCGCTCTACATTAAGGAAAGGCGAAAATTCGTGACN

****
Consensus:

GCTTCCTTAACATAGAAGCGCTCTACATTAAGGAAAGGCGAAAATTCGTGACN

>Bacillus_Fam_963_53_1 Nr. of seq. 1 Alignment length(with gaps) = 53 Alignment score = 0.000000
GCF_000181495.1_ASM18149v1_genomic.fna_71:4024-4448 Satlength=425 Nr of Repeats=8 RepeatLength=53 seed=GAATCGGTAG Num.seqs=8 Similarity=0.919137
0   GAATCGGTAGGGTCAAAAGAGCACTTAGACATGGTCGAATCCAGGTAAGAGAA

****
Consensus:

GAATCGGTAGGGTCAAAAGAGCACTTAGACATGGTCGAATCCAGGTAAGAGAA

>Bacillus_Fam_964_53_1 Nr. of seq. 1 Alignment length(with gaps) = 53 Alignment score = 0.000000
GCF_000181495.1_ASM18149v1_genomic.fna_89:2084-2402 Satlength=319 Nr of Repeats=6 RepeatLength=53 seed=GTAGGTCATA Num.seqs=6 Similarity=0.932914
0   GTAGGTCATAAGGGTCTCAACATGAAAATGAAGCCATGCGCTCGTCCCGCGCG

****
Consensus:

GTAGGTCATAAGGGTCTCAACATGAAAATGAAGCCATGCGCTCGTCCCGCGCG

>Bacillus_Fam_965_53_1 Nr. of seq. 1 Alignment length(with gaps) = 53 Alignment score = 0.000000
GCF_000181495.1_ASM18149v1_genomic.fna_99:4910-5176 Satlength=267 Nr of Repeats=4 RepeatLength=53 seed=TCTCAATGGG Num.seqs=3 Similarity=0.829218
0   TCTCAATGGGCTAATTACGTCGTTTACGGGCCAACACCTCATCAAAAAGTCAA

****
Consensus:

TCTCAATGGGCTAATTACGTCGTTTACGGGCCAACACCTCATCAAAAAGTCAA

>Bacillus_Fam_966_53_1 Nr. of seq. 1 Alignment length(with gaps) = 53 Alignment score = 0.000000
GCF_000181495.1_ASM18149v1_genomic.fna_130:505-717 Satlength=213 Nr of Repeats=4 RepeatLength=53 seed=TACATGAGTT Num.seqs=4 Similarity=0.689727
0   TACATGAGTTACTTCAGATGGTCCGACTCATGTATGAAAAACTNGATCNTTGA

****
Consensus:

TACATGAGTTACTTCAGATGGTCCGACTCATGTATGAAAAACTNGATCNTTGA

>Bacillus_Fam_967_53_1 Nr. of seq. 1 Alignment length(with gaps) = 53
Alignment score = 0.000000
GCF_000181495.1_ASM18149v1_genomic.fna_138:4971-5501 Satlength=531 Nr of Repeats=10 RepeatLength=53 seed=GTCCCTTTGA Num.seqs=10
Similarity=0.877009
0   GTCCCTTTGACGACTCCGATTCTCCCTGACTTCCGATTCCACACCCTCATTGA

****
Consensus:

GTCCCTTTGACGACTCCGATTCTCCCTGACTTCCGATTCCACACCCTCATTGA

>Bacillus_Fam_968_53_1 Nr. of seq. 1 Alignment length(with gaps) = 53
Alignment score = 0.000000
GCF_000181495.1_ASM18149v1_genomic.fna_140:18323-18531 Satlength=209 Nr of Repeats=4 RepeatLength=52 seed=CTAAAGACCA Num.seqs=4
Similarity=0.901468
0   CTAAAGACCAAAACGAATGAAATTCCAGGTGTAAAAGTGTCTTTAGAGACGTT

****
Consensus:

CTAAAGACCAAAACGAATGAAATTCCAGGTGTAAAAGTGTCTTTAGAGACGTT

>Bacillus_Fam_969_53_1 Nr. of seq. 1 Alignment length(with gaps) = 53
Alignment score = 0.000000
GCF_000181495.1_ASM18149v1_genomic.fna_141:950-1213 Satlength=264 Nr of Repeats=5 RepeatLength=53 seed=AAAGGGTGTA Num.seqs=4 Similarity=0.916143
0   AAAGGGTGTAAACGGGCCAACGAGAAAGCAGAACCAGAAGGATGCTGGCCCGC

****
Consensus:

AAAGGGTGTAAACGGGCCAACGAGAAAGCAGAACCAGAAGGATGCTGGCCCGC

>Bacillus_Fam_970_53_1 Nr. of seq. 1 Alignment length(with gaps) = 53
Alignment score = 0.000000
GCF_000181495.1_ASM18149v1_genomic.fna_150:24659-25400 Satlength=742 Nr of Repeats=14 RepeatLength=53 seed=CATCAAAAGG Num.seqs=13
Similarity=0.894856 0
CATCAAAAGGTGTGGACGGTGAAAATGATGTATGAAAAGAGGGATGATTCCAG

****
Consensus:

CATCAAAAGGTGTGGACGGTGAAAATGATGTATGAAAAGAGGGATGATTCCAG

>Bacillus_Fam_971_53_1 Nr. of seq. 1 Alignment length(with gaps) = 53
Alignment score = 0.000000
GCF_000181495.1_ASM18149v1_genomic.fna_157:28244-28774 Satlength=531 Nr of Repeats=10 RepeatLength=53 seed=ACGTTTCTTC Num.seqs=10
Similarity=0.838435 0
ACGTTTCTTCTCTAATCGTGCACCTTCACNTCAGATTCCCGCCTTCTAGGGGC

****
Consensus:

ACGTTTCTTCTCTAATCGTGCACCTTCACNTCAGATTCCCGCCTTCTAGGGGC

>Bacillus_Fam_972_53_1 Nr. of seq. 1 Alignment length(with gaps) = 53
Alignment score = 0.000000
GCF_000181495.1_ASM18149v1_genomic.fna_166:38412-38623 Satlength=212 Nr of Repeats=4 RepeatLength=53 seed=GTTGTTGAAG Num.seqs=3
Similarity=0.932914
0   GTTGTTGAAGGGTAAAGTTTAAGAGCTAAACGTACCCCTTGAGTGCAGATTGA

****
Consensus:

GTTGTTGAAGGGTAAAGTTTAAGAGCTAAACGTACCCCTTGAGTGCAGATTGA

>Bacillus_Fam_973_53_1 Nr. of seq. 1 Alignment length(with gaps) = 53
Alignment score = 0.000000
GCF_000181495.1_ASM18149v1_genomic.fna_173:33950-34639 Satlength=690 Nr of Repeats=13 RepeatLength=53 seed=GTACATCATT Num.seqs=13
Similarity=0.832608 0
GTACATCATTCGAGAGTCAACCAGGAAATGACACCCCGCGCGGGAGGAGCGCG

****
Consensus:

GTACATCATTCGAGAGTCAACCAGGAAATGACACCCCGCGCGGGAGGAGCGCG

>Bacillus_Fam_974_53_1 Nr. of seq. 1 Alignment length(with gaps) = 53
Alignment score = 0.000000
GCF_000181495.1_ASM18149v1_genomic.fna_173:43930-44406 Satlength=477 Nr of Repeats=8 RepeatLength=53 seed=CCAACATCTC Num.seqs=6
Similarity=0.823251
0   CCAACATCTCGAGGAATTCTGAATCACAATGGTCCGATAACCCTCTAAACGGA

****
Consensus:

CCAACATCTCGAGGAATTCTGAATCACAATGGTCCGATAACCCTCTAAACGGA

>Bacillus_Fam_975_53_1 Nr. of seq. 1 Alignment length(with gaps) = 53
Alignment score = 0.000000
GCF_000181495.1_ASM18149v1_genomic.fna_174:16203-16625 Satlength=423 Nr of Repeats=8 RepeatLength=53 seed=GTAAACGTAC Num.seqs=7
Similarity=0.832285
47   GTACCACTACAGAAGGNGAATCGGGCTTCTAATGGTACGATTAGCTTGTAAAC

****
Consensus:

GTAAACGTACCACTACAGAAGGNGAATCGGGCTTCTAATGGTACGATTAGCTT

>Bacillus_Fam_976_53_1 Nr. of seq. 1 Alignment length(with gaps) = 53
Alignment score = 0.000000
GCF_000181495.1_ASM18149v1_genomic.fna_175:7654-7968 Satlength=315 Nr of Repeats=6 RepeatLength=52 seed=AAAAGAGAAT Num.seqs=4 Similarity=0.752058
0 AAAAGAGAATCGACCCTGTCGAAGAGGCGTAAAGACCAGGTCGAATGGTTGCA

****
Consensus:

AAAAGAGAATCGACCCTGTCGAAGAGGCGTAAAGACCAGGTCGAATGGTTGCA

>Bacillus_Fam_977_53_1 Nr. of seq. 1 Alignment length(with gaps) = 53
Alignment score = 0.000000
GCF_000181495.1_ASM18149v1_genomic.fna_178:36078-36870 Satlength=793 Nr of Repeats=13 RepeatLength=53 seed=GGCTTCATTT Num.seqs=11
Similarity=0.778072 0
GGCTTCATTTGTGGATTCAGGTGCTTATGACAACACGCGCTCGAGGTGCGCGG

****
Consensus:

GGCTTCATTTGTGGATTCAGGTGCTTATGACAACACGCGCTCGAGGTGCGCGG

>Bacillus_Fam_978_53_1 Nr. of seq. 1 Alignment length(with gaps) = 53
Alignment score = 0.000000
GCF_000181495.1_ASM18149v1_genomic.fna_179:78015-78438 Satlength=424 Nr of Repeats=7 RepeatLength=53 seed=CATCATTCTG Num.seqs=5
Similarity=0.864151
0 CATCATTCTGGGCCGCCGTGAAAAATGATGTAGGAAAACAATCGGAATTAGGG

****
Consensus:

CATCATTCTGGGCCGCCGTGAAAAATGATGTAGGAAAACAATCGGAATTAGGG

>Bacillus_Fam_979_53_1 Nr. of seq. 1 Alignment length(with gaps) = 53
Alignment score = 0.000000
GCF_000181495.1_ASM18149v1_genomic.fna_181:51513-52096 Satlength=584 Nr of Repeats=11 RepeatLength=53 seed=TTGGTACATC Num.seqs=11
Similarity=0.914923 0
TTGGTACATCAAATCAAGGTGGTCCACGCAATGATGTCCTAATAAAGTCAGAA

****
Consensus:

TTGGTACATCAAATCAAGGTGGTCCACGCAATGATGTCCTAATAAAGTCAGAA

>Bacillus_Fam_980_53_1 Nr. of seq. 1 Alignment length(with gaps) = 53
Alignment score = 0.000000
GCF_000181495.1_ASM18149v1_genomic.fna_183:56196-56408 Satlength=213 Nr of Repeats=4 RepeatLength=53 seed=TGGCCCATTT Num.seqs=4
Similarity=0.767677
0 TGGCCCATTTAACCATTTAATGGACCAACACCCTTTAGAGTCGCAACTCTCAT

****
Consensus:

TGGCCCATTTAACCATTTAATGGACCAACACCCTTTAGAGTCGCAACTCTCAT

>Bacillus_Fam_981_53_1 Nr. of seq. 1 Alignment length(with gaps) = 53
Alignment score = 0.000000
GCF_000181495.1_ASM18149v1_genomic.fna_184:47614-48038 Satlength=425 Nr of Repeats=8 RepeatLength=53 seed=GTCATTTTGT Num.seqs=8
Similarity=0.828593
0 GTCATTTTGTGAGAATTGGTCGAATGACACCCNGCGCCCCGTCCGCGCGGTAG

****
Consensus:

GTCATTTTGTGAGAATTGGTCGAATGACACCCNGCGCCCCGTCCGCGCGGTAG

>Bacillus_Fam_982_53_1 Nr. of seq. 1 Alignment length(with gaps) = 53
Alignment score = 0.000000
GCF_000181495.1_ASM18149v1_genomic.fna_186:247447-247818 Satlength=372 Nr of Repeats=7 RepeatLength=53 seed=CACTGGCCCA Num.seqs=7
Similarity=0.744834 0
CACTGGCCCAATAATCCAATAAATGGGCCAACATGATCTGGTTTTAGCAATCT

****
Consensus:

CACTGGCCCAATAATCCAATAAATGGGCCAACATGATCTGGTTTTAGCAATCT

>Bacillus_Fam_983_53_1 Nr. of seq. 1 Alignment length(with gaps) = 53
Alignment score = 0.000000
GCF_000181495.1_ASM18149v1_genomic.fna_186:666606-666977 Satlength=372 Nr of Repeats=7 RepeatLength=53 seed=AAATGATGAC Num.seqs=7
Similarity=0.856244 0
AAATGATGACATGCGCGGCGNGNGCGCGGGGTGTCATTGTGTTGGATTTGCGG

****
Consensus:

AAATGATGACATGCGCGGCGNGNGCGCGGGGTGTCATTGTGTTGGATTTGCGG

>Bacillus_Fam_984_53_1 Nr. of seq. 1 Alignment length(with gaps) = 53
Alignment score = 0.000000
GCF_000181495.1_ASM18149v1_genomic.fna_186:674905-675223 Satlength=319 Nr of Repeats=6 RepeatLength=53 seed=GTAATCGACC Num.seqs=6
Similarity=0.839506 48
CGACCCAACATCTTCTCCAAAACCCAATCTCCTTGGGCTGTTTACCTCGTAAT

****
Consensus:

GTAATCGACCCAACATCTTCTCCAAAACCCAATCTCCTTGGGCTGTTTACCTC

>Bacillus_Fam_985_53_1 Nr. of seq. 1 Alignment length(with gaps) = 53
Alignment score = 0.000000
GCF_000285535.1_ASM28553v1_genomic.fna_3:225928-226269 Satlength=342 Nr of Repeats=8 RepeatLength=53 seed=ATACCATTTA Num.seqs=5 Similarity=0.793711
0   ATACCATTTATCGTTGCTCGGCTTCGTGATATTCCGTTTACGCCGTTTGCGGT

****
Consensus:

ATACCATTTATCGTTGCTCGGCTTCGTGATATTCCGTTTACGCCGTTTGCGGT

>Bacillus_Fam_986_53_1 Nr. of seq. 1 Alignment length(with gaps) = 53
Alignment score = 0.000000
GCF_000285535.1_ASM28553v1_genomic.fna_61:20-436 Satlength=417 Nr of Repeats=8 RepeatLength=52 seed=ATGAAGAGGA Num.seqs=8 Similarity=0.769180
0   ATGAAGAGGATACACCACGAATGAAAGCTACGAAATCCCCTTCATGAGAGCTA

****
Consensus:

ATGAAGAGGATACACCACGAATGAAAGCTACGAAATCCCCTTCATGAGAGCTA

>Bacillus_Fam_987_53_1 Nr. of seq. 1 Alignment length(with gaps) = 53
Alignment score = 0.000000
GCF_000292245.2_ASM29224v2_genomic.fna_27:54281-54494 Satlength=214 Nr of Repeats=4 RepeatLength=53 seed=GAGGAAAGTC Num.seqs=3 Similarity=0.823899
0   GAGGAAAGTCAGAAGGAGAAGCTNNGNACGCGTCGGCAGGTTCAGACTTTAGG

****
Consensus:

GAGGAAAGTCAGAAGGAGAAGCTNNGNACGCGTCGGCAGGTTCAGACTTTAGG

>Bacillus_Fam_988_53_1 Nr. of seq. 1 Alignment length(with gaps) = 53
Alignment score = 0.000000
GCF_000311725.1_ASM31172v1_genomic.fna_2:378341-378913 Satlength=573 Nr of Repeats=11 RepeatLength=52 seed=TTGTCCGAAT Num.seqs=11 Similarity=0.674931 0
TTGTCCGAATAANCCTTCTATTCGGACTANAACCACTTGGTGCTTCCNTCNTC

****
Consensus:

TTGTCCGAATAANCCTTCTATTCGGACTANAACCACTTGGTGCTTCCNTCNTC

>Bacillus_Fam_989_53_1 Nr. of seq. 1 Alignment length(with gaps) = 53
Alignment score = 0.000000
GCF_000311725.1_ASM31172v1_genomic.fna_3:509966-510547 Satlength=582 Nr of Repeats=11 RepeatLength=53 seed=TTTGTCTCAT Num.seqs=9 Similarity=0.858840
0   TTTGTCTCATGGAGCNTTCATGTTACATGAGTTACTCCCTTTCTTGAGGAAAG

****

Consensus:

TTTGTCTCATGGAGCNTTCATGTTACATGAGTTACTCCCTTTCTTGAGGAAAG

>Bacillus_Fam_990_53_1 Nr. of seq. 1 Alignment length(with gaps) = 53
Alignment score = 0.000000
GCF_000374565.1_ASM37456v1_genomic.fna_4:225418-226666 Satlength=1249 Nr of Repeats=24 RepeatLength=52 seed=ATTAGTGACC Num.seqs=24
Similarity=0.592525 0
ATTAGTGACCGTTTTCTNTTGCAACTACAAAAAACGGGCATCTANTACACTCG

****
Consensus:

ATTAGTGACCGTTTTCTNTTGCAACTACAAAAAACGGGCATCTANTACACTCG

>Bacillus_Fam_991_53_1 Nr. of seq. 1 Alignment length(with gaps) = 53
Alignment score = 0.000000
GCF_000708755.2_ASM70875v2_genomic.fna_6:240161-240426 Satlength=266 Nr of Repeats=5 RepeatLength=53 seed=ATTATAACGT Num.seqs=5
Similarity=0.854088
0 ATTATAACGTAAGGAAACACTCGAATCGTACGCTATACGTACACCGGATGAAA

****
Consensus:

ATTATAACGTAAGGAAACACTCGAATCGTACGCTATACGTACACCGGATGAAA

>Bacillus_Fam_992_53_1 Nr. of seq. 1 Alignment length(with gaps) = 53
Alignment score = 0.000000
GCF_000709935.2_ASM70993v2_genomic.fna_1:146658-146976 Satlength=319 Nr of Repeats=6 RepeatLength=53 seed=CCAGAAATGG Num.seqs=6
Similarity=0.773585
0 CCAGAAATGGTGACCGGAAACAGAGAAAAGTGGCGTCACTAAGGCGAGTGGCG

****
Consensus:

CCAGAAATGGTGACCGGAAACAGAGAAAAGTGGCGTCACTAAGGCGAGTGGCG

>Bacillus_Fam_993_53_1 Nr. of seq. 1 Alignment length(with gaps) = 53
Alignment score = 0.000000
GCF_000709935.2_ASM70993v2_genomic.fna_1:748009-748380 Satlength=372 Nr of Repeats=7 RepeatLength=53 seed=AGAGTGCTGC Num.seqs=7
Similarity=0.783169
0 AGAGTGCTGCCACTTTTGCCGTTGAGGGATCATCGGAAGTGGCGTCAATCCAC

****
Consensus:

AGAGTGCTGCCACTTTTGCCGTTGAGGGATCATCGGAAGTGGCGTCAATCCAC

>Bacillus_Fam_994_53_1 Nr. of seq. 1 Alignment length(with gaps) = 53
Alignment score = 0.000000

GCF_000709935.2_ASM70993v2_genomic.fna_17:22189-22454 Satlength=266 Nr of Repeats=5 RepeatLength=53 seed=TGACGCCACA Num.seqs=5 Similarity=0.778616
0 TGACGCCACAAAGCTGTTTGCAATATAAGTGAAAGTGGCGCAACTCNCCGGAG

****
Consensus:

TGACGCCACAAAGCTGTTTGCAATATAAGTGAAAGTGGCGCAACTCNCCGGAG

>Bacillus_Fam_995_53_1 Nr. of seq. 1 Alignment length(with gaps) = 53
Alignment score = 0.000000
GCF_000709935.2_ASM70993v2_genomic.fna_24:63175-63438 Satlength=264 Nr of Repeats=5 RepeatLength=53 seed=TGCCACTTTT Num.seqs=3 Similarity=0.882600
0 TGCCACTTTTTGCGTGATGGGTTTGCCAATTGTGGTGTCATTGGCCGTAGTGG

****
Consensus:

TGCCACTTTTTGCGTGATGGGTTTGCCAATTGTGGTGTCATTGGCCGTAGTGG

>Bacillus_Fam_996_53_1 Nr. of seq. 1 Alignment length(with gaps) = 53
Alignment score = 0.000000
GCF_000752035.1_Bacillus_rubiinfantis_genomic.fna_11:288723-288988 Satlength=266 Nr of Repeats=5 RepeatLength=53 seed=CGGGCTCCTA Num.seqs=5 Similarity=0.909434 0
CGGGCTCCTATTTCAACGATTGGCTACCGTTTCCACTCCTTGAGAGCTACGCT

****
Consensus:

CGGGCTCCTATTTCAACGATTGGCTACCGTTTCCACTCCTTGAGAGCTACGCT

>Bacillus_Fam_997_53_1 Nr. of seq. 1 Alignment length(with gaps) = 53
Alignment score = 0.000000
GCF_000759675.1_ASM75967v1_genomic.fna_151:483245-483566 Satlength=322 Nr of Repeats=5 RepeatLength=53 seed=GTTTTGTCCT Num.seqs=3 Similarity=0.740042 0
GTTTTGTCCTCAAGACCCCCGATTGAGGACGAATCCTCTGGGGACTCTTGNTA

****
Consensus:

GTTTTGTCCTCAAGACCCCCGATTGAGGACGAATCCTCTGGGGACTCTTGNTA

>Bacillus_Fam_998_53_1 Nr. of seq. 1 Alignment length(with gaps) = 53
Alignment score = 0.000000
GCF_000813125.1_ASM81312v1_genomic.fna_10:31192-31510 Satlength=319 Nr of Repeats=6 RepeatLength=53 seed=AAGAAAGATA Num.seqs=6 Similarity=0.788683
0 AAGAAAGATACCCTTATGCAGCCGTTTGTCATCACATAAGGGTACGAAAACCG

****
Consensus:

AAGAAAGATACCCTTATGCAGCCGTTTGTCATCACATAAGGGTACGAAAACCG

>Bacillus_Fam_999_53_1 Nr. of seq. 1 Alignment length(with gaps) = 53
Alignment score = 0.000000
GCF_000813125.1_ASM81312v1_genomic.fna_118:4594-4855 Satlength=262 Nr of Repeats=5 RepeatLength=52 seed=TATTGGTTAC Num.seqs=4 Similarity=0.660377
0 TATTGGTTACCTATAATTCTAGTTTCAGCCCTTTTTAGGGCACCAATAACGCC

****
Consensus:

TATTGGTTACCTATAATTCTAGTTTCAGCCCTTTTTAGGGCACCAATAACGCC

>Bacillus_Fam_1000_53_1 Nr. of seq. 1 Alignment length(with gaps) = 53
Alignment score = 0.000000
GCF_001315165.1_ASM131516v1_genomic.fna_1:59355-59632 Satlength=278 Nr of Repeats=5 RepeatLength=53 seed=GTACCCTTAT Num.seqs=4 Similarity=0.878407
0 GTACCCTTATGGAGAGCTTTCGCTTGGTATAAGGATACGATTAGGAGAAAATC

****
Consensus:

GTACCCTTATGGAGAGCTTTCGCTTGGTATAAGGATACGATTAGGAGAAAATC

>Bacillus_Fam_1001_53_1 Nr. of seq. 1 Alignment length(with gaps) = 53
Alignment score = 0.000000
GCF_001315165.1_ASM131516v1_genomic.fna_18:0-258 Satlength=259 Nr of Repeats=5 RepeatLength=52 seed=CTTCAGACAA Num.seqs=4 Similarity=0.834382
0 CTTCAGACAAAATCTGCACGCCTAAGACGTGGTTTTGGCCGAACATGGCATCA

****
Consensus:

CTTCAGACAAAATCTGCACGCCTAAGACGTGGTTTTGGCCGAACATGGCATCA

>Bacillus_Fam_1002_53_1 Nr. of seq. 1 Alignment length(with gaps) = 53
Alignment score = 0.000000
GCF_001315165.1_ASM131516v1_genomic.fna_110:6-377 Satlength=372 Nr of Repeats=7 RepeatLength=53 seed=TATCCTTATG Num.seqs=7 Similarity=0.834681
0 TATCCTTATGAGGTAAAATCCAGCAACATAAGGGTACCGTTCAAGCCNAATCG

****
Consensus:

TATCCTTATGAGGTAAAATCCAGCAACATAAGGGTACCGTTCAAGCCNAATCG

>Bacillus_Fam_1003_53_1 Nr. of seq. 1 Alignment length(with gaps) = 53
Alignment score = 0.000000
GCF_001439915.1_ASM143991v1_genomic.fna_83:366387-367075 Satlength=689 Nr of Repeats=13 RepeatLength=53 seed=CCTAAAAAGA Num.seqs=12 Similarity=0.861254 0
CCTAAAAAGAGGAAAATGGTGACCGTCAAAACGAAAGCAATGGTCACCAATCT

****
Consensus:

CCTAAAAAGAGGAAAATGGTGACCGTCAAAACGAAAGCAATGGTCACCAATCT

>Bacillus_Fam_1004_53_1 Nr. of seq. 1 Alignment length(with gaps) = 53
Alignment score = 0.000000
GCF_001439915.1_ASM143991v1_genomic.fna_116:264620-264828 Satlength=209
Nr of Repeats=4 RepeatLength=52 seed=GTATTGCGAC Num.seqs=4
Similarity=0.578704 0
GTATTGCGACACAAAGCAAGAATTTTCAGCAAGCCGAGTCCGAATANAGCNTC

****
Consensus:

GTATTGCGACACAAAGCAAGAATTTTCAGCAAGCCGAGTCCGAATANAGCNTC

>Bacillus_Fam_1005_53_1 Nr. of seq. 1 Alignment length(with gaps) = 53
Alignment score = 0.000000
GCF_001439915.1_ASM143991v1_genomic.fna_125:212481-212689 Satlength=209
Nr of Repeats=4 RepeatLength=52 seed=CTTATGAAGA Num.seqs=4
Similarity=0.859539 0
CTTATGAAGACCTTTTCGACGAGCCACGAGTTCTGATTCGAGTCTTCATCACG

****
Consensus:

CTTATGAAGACCTTTTCGACGAGCCACGAGTTCTGATTCGAGTCTTCATCACG

>Bacillus_Fam_1006_53_1 Nr. of seq. 1 Alignment length(with gaps) = 53
Alignment score = 0.000000
GCF_001591485.1_ASM159148v1_genomic.fna_83:11410-11726 Satlength=317 Nr
of Repeats=6 RepeatLength=53 seed=ATGAAAGACA Num.seqs=5
Similarity=0.849057
0   ATGAAAGACAAAACTCAGGAGAATGGGTGAGGGAATTGTCCTTCATCCGGTGT

****
Consensus:

ATGAAAGACAAAACTCAGGAGAATGGGTGAGGGAATTGTCCTTCATCCGGTGT

>Bacillus_Fam_1007_53_1 Nr. of seq. 1 Alignment length(with gaps) = 53
Alignment score = 0.000000
GCF_001591485.1_ASM159148v1_genomic.fna_133:5376-5693 Satlength=318 Nr of
Repeats=5 RepeatLength=53 seed=GATGAAGCCC Num.seqs=3 Similarity=0.614256
0   GATGAAGCCCAAANCCCACTAAGCCCACNCAAGGATATGTNCTTCATAGGCNC

****
Consensus:

GATGAAGCCCAAANCCCACTAAGCCCACNCAAGGATATGTNCTTCATAGGCNC

>Bacillus_Fam_1008_53_1 Nr. of seq. 1 Alignment length(with gaps) = 53
Alignment score = 0.000000

GCF_001591665.1_ASM159166v1_genomic.fna_7:59019-59227 Satlength=209 Nr of Repeats=4 RepeatLength=52 seed=GTCAATGAAA Num.seqs=4 Similarity=0.701010
0 GTCAATGAAAGCCTCTTCCATTTACCATGGAGGCCCNATTATTTCTCTTTTCG

****
Consensus:

GTCAATGAAAGCCTCTTCCATTTACCATGGAGGCCCNATTATTTCTCTTTTCG

>Bacillus_Fam_1009_53_1 Nr. of seq. 1 Alignment length(with gaps) = 53 Alignment score = 0.000000
GCF_001591665.1_ASM159166v1_genomic.fna_89:10754-11013 Satlength=260 Nr of Repeats=5 RepeatLength=52 seed=TATTGGAGCC Num.seqs=4 Similarity=0.840671
0 TATTGGAGCCCATTCTGTTTGAAAACTTGGGCAAAAGGTATCCAATCGATCCG

****
Consensus:

TATTGGAGCCCATTCTGTTTGAAAACTTGGGCAAAAGGTATCCAATCGATCCG

>Bacillus_Fam_1010_53_1 Nr. of seq. 1 Alignment length(with gaps) = 53 Alignment score = 0.000000
GCF_001636315.1_ASM163631v1_genomic.fna_1:69909-70760 Satlength=852 Nr of Repeats=9 RepeatLength=53 seed=TTTTTACCGC Num.seqs=6 Similarity=0.647879
0 TTTTTACCGCCTGACCCCCTTCTCAGCACTCCGCGGGCAATAGAACCATCTTC

****
Consensus:

TTTTTACCGCCTGACCCCCTTCTCAGCACTCCGCGGGCAATAGAACCATCTTC

>Bacillus_Fam_1011_53_1 Nr. of seq. 1 Alignment length(with gaps) = 53 Alignment score = 0.000000
GCF_001636315.1_ASM163631v1_genomic.fna_1:3822748-3823380 Satlength=633 Nr of Repeats=12 RepeatLength=53 seed=TATTGCCCGA Num.seqs=8 Similarity=0.682760 0
TATTGCCCGATTGNGGTGTCCAAGGGGTGCAAACGGTAAAAAGAACAGGCTTC

****
Consensus:

TATTGCCCGATTGNGGTGTCCAAGGGGTGCAAACGGTAAAAAGAACAGGCTTC

>Bacillus_Fam_1012_53_1 Nr. of seq. 1 Alignment length(with gaps) = 53 Alignment score = 0.000000
GCF_001636335.1_ASM163633v1_genomic.fna_1:112399-112815 Satlength=417 Nr of Repeats=8 RepeatLength=52 seed=TGTCCATCAT Num.seqs=8 Similarity=0.780423
0 TGTCCATCATCCGTTAGATGAAGGCCATTTTTAGCAGGCAAATCATGGAGTAA

****
Consensus:

TGTCCATCATCCGTTAGATGAAGGCCATTTTTAGCAGGCAAATCATGGAGTAA

>Bacillus_Fam_1013_53_1 Nr. of seq. 1 Alignment length(with gaps) = 53
Alignment score = 0.000000
GCF_900107275.1_IMG-
taxon_2675903024_annotated_assembly_genomic.fna_42:309000-309527
Satlength=528 Nr of Repeats=10 RepeatLength=53 seed=TTCTGTCCCG
Num.seqs=7 Similarity=0.952081 0
TTCTGTCCCGAATCCGTTTCATACGGGACACTAGGGCATAAAAGTGCAGACGC

****
Consensus:

TTCTGTCCCGAATCCGTTTCATACGGGACACTAGGGCATAAAAGTGCAGACGC

>Bacillus_Fam_1014_53_1 Nr. of seq. 1 Alignment length(with gaps) = 53
Alignment score = 0.000000
GCF_900156865.1_PRJEB18960_genomic.fna_3:2816531-2816795 Satlength=265 Nr of Repeats=5 RepeatLength=53 seed=GTCCTCATCC Num.seqs=4
Similarity=0.879115 0
GTCCTCATCCCTACCATGATAACAATTCCGAACCCTACTTTCTCTCCAAACAT

****
Consensus:

GTCCTCATCCCTACCATGATAACAATTCCGAACCCTACTTTCTCTCCAAACAT

>Bacillus_Fam_1015_53_1 Nr. of seq. 1 Alignment length(with gaps) = 53
Alignment score = 0.000000
GCF_900156865.1_PRJEB18960_genomic.fna_3:2955854-2956755 Satlength=902 Nr of Repeats=17 RepeatLength=53 seed=TTGCGGGTTC Num.seqs=17
Similarity=0.813356 0
TTGCGGGTTCGCACTTGGAACTTTCTAGGAGATACCGAACCCGATTCCACCAC

****
Consensus:

TTGCGGGTTCGCACTTGGAACTTTCTAGGAGATACCGAACCCGATTCCACCAC

>Bacillus_Fam_1016_53_1 Nr. of seq. 1 Alignment length(with gaps) = 53
Alignment score = 0.000000
GCF_900156865.1_PRJEB18960_genomic.fna_3:3971592-3971910 Satlength=319 Nr of Repeats=6 RepeatLength=53 seed=AGAAAATAGG Num.seqs=6
Similarity=0.914465 0
AGAAAATAGGTAACAGCGAACCAGTAAAACCATTATAGTGGTATGCAAATGGA

****
Consensus:

AGAAAATAGGTAACAGCGAACCAGTAAAACCATTATAGTGGTATGCAAATGGA

>Bacillus_Fam_1017_52_1 Nr. of seq. 1 Alignment length(with gaps) = 52
Alignment score = 0.000000

BACILLUS_PSEUDALCALIPHILUS_genomic.fna_2:394969-395177 Satlength=209 Nr of Repeats=4 RepeatLength=52 seed=TAATCGTGTA Num.seqs=4 Similarity=0.797694
0   TAATCGTGTAGATACTGTGACCATTGTGGGGAGTAACTGACATGAAAAGGGC

****
Consensus:

TAATCGTGTAGATACTGTGACCATTGTGGGGAGTAACTGACATGAAAAGGGC

>Bacillus_Fam_1018_52_1 Nr. of seq. 1 Alignment length(with gaps) = 52
Alignment score = 0.000000
BACILLUS_PSEUDALCALIPHILUS_genomic.fna_12:22087-22323 Satlength=237 Nr of Repeats=4 RepeatLength=52 seed=TAGCAACATA Num.seqs=3 Similarity=0.931624
0   TAGCAACATATCGAGTGAAAAGGGCAGAGTTGTTGTAGGAAAAGTCGGGAAG

****
Consensus:

TAGCAACATATCGAGTGAAAAGGGCAGAGTTGTTGTAGGAAAAGTCGGGAAG

>Bacillus_Fam_1019_52_1 Nr. of seq. 1 Alignment length(with gaps) = 52
Alignment score = 0.000000
GCF_000169195.2_ASM16919v2_genomic.fna_1:619606-619865 Satlength=260 Nr of Repeats=5 RepeatLength=52 seed=GCTTTTGTCC Num.seqs=4
Similarity=0.811966
0   GCTTTTGTCCATTTAGAACAGCTAACGGGACAATTCCACCGGAGCTGCCAGC

****
Consensus:

GCTTTTGTCCATTTAGAACAGCTAACGGGACAATTCCACCGGAGCTGCCAGC

>Bacillus_Fam_1020_52_1 Nr. of seq. 1 Alignment length(with gaps) = 52
Alignment score = 0.000000
GCF_000169195.2_ASM16919v2_genomic.fna_1:1522401-1523027 Satlength=627 Nr of Repeats=12 RepeatLength=52 seed=TGAAGGACAT Num.seqs=10
Similarity=0.525016   0
TGAAGGACATTTTAGGGTTGTGGCGNCTTTGTTTTGTCCTTCATNGGGCGGA

****
Consensus:

TGAAGGACATTTTAGGGTTGTGGCGNCTTTGTTTTGTCCTTCATNGGGCGGA

>Bacillus_Fam_1021_52_1 Nr. of seq. 1 Alignment length(with gaps) = 52
Alignment score = 0.000000
GCF_000169195.2_ASM16919v2_genomic.fna_1:1523363-1523674 Satlength=312 Nr of Repeats=6 RepeatLength=52 seed=AAAAGCCAAA Num.seqs=5
Similarity=0.700000   0
AAAAGCCAAAAGGCACCTGGNNANCGTCCAAACTGTCCTTTAGAACGTCTCA

****
Consensus:

AAAAGCCAAAAGGCACCTGGNNANCGTCCAAACTGTCCTTTAGAACGTCTCA

>Bacillus_Fam_1022_52_1 Nr. of seq. 1 Alignment length(with gaps) = 52
Alignment score = 0.000000
GCF_000171615.1_ASM17161v1_genomic.fna_81:65762-66019 Satlength=258 Nr of Repeats=5 RepeatLength=52 seed=GCTCTTCTCT Num.seqs=4 Similarity=0.747379
0   GCTCTTCTCTTCTTCCTGCTGCCTTTGAGACACGCGCTACCCCTAGCGCGGC

****
Consensus:

GCTCTTCTCTTCTTCCTGCTGCCTTTGAGACACGCGCTACCCCTAGCGCGGC

>Bacillus_Fam_1023_52_1 Nr. of seq. 1 Alignment length(with gaps) = 52
Alignment score = 0.000000
GCF_000171615.1_ASM17161v1_genomic.fna_88:78004-78212 Satlength=209 Nr of Repeats=4 RepeatLength=52 seed=GGCGCGGTGC Num.seqs=4 Similarity=0.854701
0   GGCGCGGTGCAGAAAGTAGGTTTGGGCAGGGGTGAGAGCACCGCGCGGGGGT

****
Consensus:

GGCGCGGTGCAGAAAGTAGGTTTGGGCAGGGGTGAGAGCACCGCGCGGGGGT

>Bacillus_Fam_1024_52_1 Nr. of seq. 1 Alignment length(with gaps) = 52
Alignment score = 0.000000
GCF_000171615.1_ASM17161v1_genomic.fna_129:29159-29367 Satlength=209 Nr of Repeats=4 RepeatLength=52 seed=TTTTGGGCAC Num.seqs=4
Similarity=0.813941
0   TTTTGGGCACCGCGCGGACGCGGCGCGGCGCTCTTCTCACGCTGCAACTCAC

****
Consensus:

TTTTGGGCACCGCGCGGACGCGGCGCGGCGCTCTTCTCACGCTGCAACTCAC

>Bacillus_Fam_1025_52_1 Nr. of seq. 1 Alignment length(with gaps) = 52
Alignment score = 0.000000
GCF_000175075.1_ASM17507v1_genomic.fna_3:45105-45313 Satlength=209 Nr of Repeats=4 RepeatLength=52 seed=GAGATGTCTT Num.seqs=4 Similarity=0.897436
0   GAGATGTCTTCATCCATGCGATGAGGACCTGTCTACGCACAAATAGGAACGA

****
Consensus:

GAGATGTCTTCATCCATGCGATGAGGACCTGTCTACGCACAAATAGGAACGA

>Bacillus_Fam_1026_52_1 Nr. of seq. 1 Alignment length(with gaps) = 52
Alignment score = 0.000000
GCF_000175075.1_ASM17507v1_genomic.fna_29:76482-76950 Satlength=469 Nr of Repeats=9 RepeatLength=52 seed=AGGTCTTCAT Num.seqs=9 Similarity=0.832285
0   AGGTCTTCATACACCTTATGAAGACACTTCCAAGCCTGAAATCCTTCCACTA

****
Consensus:

AGGTCTTCATACACCTTATGAAGACACTTCCAAGCCTGAAATCCTTCCACTA

>Bacillus_Fam_1027_52_1 Nr. of seq. 1 Alignment length(with gaps) = 52
Alignment score = 0.000000
GCF_000175075.1_ASM17507v1_genomic.fna_31:72973-73233 Satlength=261 Nr of Repeats=5 RepeatLength=52 seed=GACATCTCTC Num.seqs=5 Similarity=0.851282
0   GACATCTCTCCACCGGAAATCTTCTCTCACATGTCTTCATTCCGCTTATGAG

****
Consensus:

GACATCTCTCCACCGGAAATCTTCTCTCACATGTCTTCATTCCGCTTATGAG

>Bacillus_Fam_1028_52_1 Nr. of seq. 1 Alignment length(with gaps) = 52
Alignment score = 0.000000
GCF_000175075.1_ASM17507v1_genomic.fna_31:101319-101579 Satlength=261 Nr of Repeats=5 RepeatLength=52 seed=GATGAAGACA Num.seqs=5
Similarity=0.907692
0   GATGAAGACATTCCCATGATGATTTCCTTTTCCAAAGTGTCTTCATCATGCG

****
Consensus:

GATGAAGACATTCCCATGATGATTTCCTTTTCCAAAGTGTCTTCATCATGCG

>Bacillus_Fam_1029_52_1 Nr. of seq. 1 Alignment length(with gaps) = 52
Alignment score = 0.000000
GCF_000175075.1_ASM17507v1_genomic.fna_32:508521-508729 Satlength=209 Nr of Repeats=4 RepeatLength=52 seed=CAACATTGTC Num.seqs=4
Similarity=0.747863
0   CAACATTGTCCTCGTAGGTAGCGTACGACTCCCTGAGAAGGTGAAAACCCGC

****
Consensus:

CAACATTGTCCTCGTAGGTAGCGTACGACTCCCTGAGAAGGTGAAAACCCGC

>Bacillus_Fam_1030_52_1 Nr. of seq. 1 Alignment length(with gaps) = 52
Alignment score = 0.000000
GCF_000175075.1_ASM17507v1_genomic.fna_34:87713-88025 Satlength=313 Nr of Repeats=6 RepeatLength=52 seed=AACGAGGACA Num.seqs=6 Similarity=0.912821
0   AACGAGGACAATGTTGACGTGATTTCGGGGTTACCGGGTTTCATAGGAGTTC

****
Consensus:

AACGAGGACAATGTTGACGTGATTTCGGGGTTACCGGGTTTCATAGGAGTTC

>Bacillus_Fam_1031_52_1 Nr. of seq. 1 Alignment length(with gaps) = 52
Alignment score = 0.000000

GCF_000175075.1_ASM17507v1_genomic.fna_36:83466-83673 Satlength=208 Nr of Repeats=4 RepeatLength=52 seed=TGTTTGGTAA Num.seqs=3 Similarity=0.863248
0   TGTTTGGTAAGATAGAGGGGTTCTATACGCCCGAAGCAGCATGGTAAAAGGC

****
Consensus:

TGTTTGGTAAGATAGAGGGGTTCTATACGCCCGAAGCAGCATGGTAAAAGGC

>Bacillus_Fam_1032_52_1 Nr. of seq. 1 Alignment length(with gaps) = 52 Alignment score = 0.000000
GCF_000175075.1_ASM17507v1_genomic.fna_38:28329-28537 Satlength=209 Nr of Repeats=4 RepeatLength=52 seed=GCTTCTATAC Num.seqs=4 Similarity=0.806080
0   GCTTCTATACTACCGAAGACCTCGGGATTCCTACCGTTCGGGCGTATAGAAC

****
Consensus:

GCTTCTATACTACCGAAGACCTCGGGATTCCTACCGTTCGGGCGTATAGAAC

>Bacillus_Fam_1033_52_1 Nr. of seq. 1 Alignment length(with gaps) = 52 Alignment score = 0.000000
GCF_000175075.1_ASM17507v1_genomic.fna_39:50471-50679 Satlength=209 Nr of Repeats=4 RepeatLength=52 seed=AGGTAGTCAT Num.seqs=4 Similarity=0.710391
0   AGGTAGTCATACGCTTTGAACGAGGACAATGTTTTACACTTTTCAGTGGGGA

****
Consensus:

AGGTAGTCATACGCTTTGAACGAGGACAATGTTTTACACTTTTCAGTGGGGA

>Bacillus_Fam_1034_52_1 Nr. of seq. 1 Alignment length(with gaps) = 52 Alignment score = 0.000000
GCF_000175075.1_ASM17507v1_genomic.fna_44:72380-72692 Satlength=313 Nr of Repeats=6 RepeatLength=52 seed=CTATACGACC Num.seqs=6 Similarity=0.864957
0   CTATACGACCGAAGCTACTTATCAAGCTCCCCTTCCGTCTTATAGAACCCCT

****
Consensus:

CTATACGACCGAAGCTACTTATCAAGCTCCCCTTCCGTCTTATAGAACCCCT

>Bacillus_Fam_1035_52_1 Nr. of seq. 1 Alignment length(with gaps) = 52 Alignment score = 0.000000
GCF_000175075.1_ASM17507v1_genomic.fna_46:18322-18634 Satlength=313 Nr of Repeats=6 RepeatLength=52 seed=GACCGAAGCA Num.seqs=6 Similarity=0.909402
0   GACCGAAGCAACCCCTATTCACCTCGAAACGTCGTATAGAACGCTTCTATAC

****
Consensus:

GACCGAAGCAACCCCTATTCACCTCGAAACGTCGTATAGAACGCTTCTATAC

>Bacillus_Fam_1036_52_1 Nr. of seq. 1 Alignment length(with gaps) = 52
Alignment score = 0.000000
GCF_000177235.2_ASM17723v2_genomic.fna_1:339003-339210 Satlength=208 Nr of Repeats=4 RepeatLength=52 seed=CCCGTTAGAG Num.seqs=3
Similarity=0.931624
0   CCCGTTAGAGCAGGATTCAATAACTAAGTGTCGATTAAACAGGGTTTAAAAT

****
Consensus:

CCCGTTAGAGCAGGATTCAATAACTAAGTGTCGATTAAACAGGGTTTAAAAT

>Bacillus_Fam_1037_52_1 Nr. of seq. 1 Alignment length(with gaps) = 52
Alignment score = 0.000000
GCF_000177235.2_ASM17723v2_genomic.fna_1:650939-651407 Satlength=469 Nr of Repeats=8 RepeatLength=52 seed=GTGTCATAGC Num.seqs=7
Similarity=0.668464
0   GTGTCATAGCACTCGTATGACGCATCTTATATTGAATCTCTAGCTATGTNGT

****
Consensus:

GTGTCATAGCACTCGTATGACGCATCTTATATTGAATCTCTAGCTATGTNGT

>Bacillus_Fam_1038_52_1 Nr. of seq. 1 Alignment length(with gaps) = 52
Alignment score = 0.000000
GCF_000177235.2_ASM17723v2_genomic.fna_1:857866-858230 Satlength=365 Nr of Repeats=7 RepeatLength=52 seed=TAGTCTGAGT Num.seqs=7
Similarity=0.904163
0   TAGTCTGAGTGGATATAAAACACGAAATAAAATCTCGTTGTGATGGTGAGTC

****
Consensus:

TAGTCTGAGTGGATATAAAACACGAAATAAAATCTCGTTGTGATGGTGAGTC

>Bacillus_Fam_1039_52_1 Nr. of seq. 1 Alignment length(with gaps) = 52
Alignment score = 0.000000
GCF_000177235.2_ASM17723v2_genomic.fna_1:1710465-1710724 Satlength=260 Nr of Repeats=5 RepeatLength=52 seed=CCCGTTAGAA Num.seqs=4
Similarity=0.893162 0
CCCGTTAGAATGATTATCCCGTGCTGAAGGTAGATAAAACAGCGTTTAAAAG

****
Consensus:

CCCGTTAGAATGATTATCCCGTGCTGAAGGTAGATAAAACAGCGTTTAAAAG

>Bacillus_Fam_1040_52_1 Nr. of seq. 1 Alignment length(with gaps) = 52
Alignment score = 0.000000
GCF_000177235.2_ASM17723v2_genomic.fna_1:3148329-3148588 Satlength=260 Nr of Repeats=5 RepeatLength=52 seed=TGCGAGTGGA Num.seqs=4
Similarity=0.824786 0
TGCGAGTGGACTTTAAAGTGCCTATAAAATCCCATGAGGTAGAGGGAGCATG

****
Consensus:

TGCGAGTGGACTTTAAAGTGCCTATAAAATCCCATGAGGTAGAGGGAGCATG

>Bacillus_Fam_1041_52_1 Nr. of seq. 1 Alignment length(with gaps) = 52
Alignment score = 0.000000
GCF_000177235.2_ASM17723v2_genomic.fna_1:3704894-3705310 Satlength=417 Nr of Repeats=8 RepeatLength=52 seed=TTTTCCGTTC Num.seqs=8
Similarity=0.891941 0
TTTTCCGTTCAGTGGATCCTCTCTTCCTTGAACGGAATTCTTTGTCATTTTC

****
Consensus:

TTTTCCGTTCAGTGGATCCTCTCTTCCTTGAACGGAATTCTTTGTCATTTTC

>Bacillus_Fam_1042_52_1 Nr. of seq. 1 Alignment length(with gaps) = 52
Alignment score = 0.000000
GCF_000177235.2_ASM17723v2_genomic.fna_1:3773627-3773939 Satlength=313 Nr of Repeats=6 RepeatLength=52 seed=TTGCTTTCCT Num.seqs=6
Similarity=0.904274 0
TTGCTTTCCTTTCTAAGGCACTCAGCTTCCTGGAAAGGAATACTAAGCCACT

****
Consensus:

TTGCTTTCCTTTCTAAGGCACTCAGCTTCCTGGAAAGGAATACTAAGCCACT

>Bacillus_Fam_1043_52_1 Nr. of seq. 1 Alignment length(with gaps) = 52
Alignment score = 0.000000
GCF_000177235.2_ASM17723v2_genomic.fna_1:3821441-3821909 Satlength=469 Nr of Repeats=9 RepeatLength=52 seed=AATGGGTTGC Num.seqs=9
Similarity=0.868274 0
AATGGGTTGCTATTTTGTTTTGCGTGCCTTTTGCGCGTTGTTTTTCTGCTCG

****
Consensus:

AATGGGTTGCTATTTTGTTTTGCGTGCCTTTTGCGCGTTGTTTTTCTGCTCG

>Bacillus_Fam_1044_52_1 Nr. of seq. 1 Alignment length(with gaps) = 52
Alignment score = 0.000000
GCF_000177235.2_ASM17723v2_genomic.fna_1:3996570-3997090 Satlength=521 Nr of Repeats=10 RepeatLength=52 seed=AAGGAATGGG Num.seqs=10
Similarity=0.924217 0
AAGGAATGGGGCGCTATTCTTATTTGCTGGCCTTTACCCTCTGATTTTTGTA

****
Consensus:

AAGGAATGGGGCGCTATTCTTATTTGCTGGCCTTTACCCTCTGATTTTTGTA

>Bacillus_Fam_1045_52_1 Nr. of seq. 1 Alignment length(with gaps) = 52
Alignment score = 0.000000
GCF_000177235.2_ASM17723v2_genomic.fna_1:4116764-4117128 Satlength=365 Nr of Repeats=7 RepeatLength=52 seed=CTTTCTCGGA Num.seqs=7
Similarity=0.888889 0
CTTTCTCGGATAGTTTTCCTTTCAGAGACTTTTTCCTTCTTTGAACGGAACG

****
Consensus:

CTTTCTCGGATAGTTTTCCTTTCAGAGACTTTTTCCTTCTTTGAACGGAACG

>Bacillus_Fam_1046_52_1 Nr. of seq. 1 Alignment length(with gaps) = 52
Alignment score = 0.000000
GCF_000177235.2_ASM17723v2_genomic.fna_1:4188801-4189419 Satlength=619 Nr of Repeats=11 RepeatLength=52 seed=TTCCTTTCTA Num.seqs=9
Similarity=0.856125 0
TTCCTTTCTACTAGTAAAATCAGCCATGAAAGGAATACTAAACGCCTTCACT

****
Consensus:

TTCCTTTCTACTAGTAAAATCAGCCATGAAAGGAATACTAAACGCCTTCACT

>Bacillus_Fam_1047_52_1 Nr. of seq. 1 Alignment length(with gaps) = 52
Alignment score = 0.000000
GCF_000177235.2_ASM17723v2_genomic.fna_1:4365838-4366774 Satlength=937 Nr of Repeats=18 RepeatLength=52 seed=TAAAGGAATA Num.seqs=18
Similarity=0.863415 59
CTCGTCCTAAAGGAATACTAAGCACTCTGGCTTTCCTTTCTACGTCGATTCC

****
Consensus:

TAAAGGAATACTAAGCACTCTGGCTTTCCTTTCTACGTCGATTCCCTCGTCC

>Bacillus_Fam_1048_52_1 Nr. of seq. 1 Alignment length(with gaps) = 52
Alignment score = 0.000000
GCF_000181495.1_ASM18149v1_genomic.fna_145:9012-9583 Satlength=572 Nr of Repeats=11 RepeatLength=52 seed=GTTGTCGATT Num.seqs=10
Similarity=0.876353
0   GTTGTCGATTAGGATTATGAAAAAATCGATGGTGCCAAAAGGTGCGAATGGG

****
Consensus:

GTTGTCGATTAGGATTATGAAAAAATCGATGGTGCCAAAAGGTGCGAATGGG

>Bacillus_Fam_1049_52_1 Nr. of seq. 1 Alignment length(with gaps) = 52
Alignment score = 0.000000
GCF_000181495.1_ASM18149v1_genomic.fna_158:13126-13858 Satlength=733 Nr of Repeats=14 RepeatLength=52 seed=CCCCGTCAAA Num.seqs=10
Similarity=0.862678 0
CCCCGTCAAAGAGAGGGTATGAGGGTGTCGAATTGAATTTTGGTTGAATCGG

****
Consensus:

CCCCGTCAAAGAGAGGGTATGAGGGTGTCGAATTGAATTTTGGTTGAATCGG

>Bacillus_Fam_1050_52_1 Nr. of seq. 1 Alignment length(with gaps) = 52
Alignment score = 0.000000
GCF_000181495.1_ASM18149v1_genomic.fna_163:107-991 Satlength=885 Nr of
Repeats=17 RepeatLength=52 seed=GAAACGTACC Num.seqs=15
Similarity=0.871795
0   GAAACGTACCAATATAGAGTCAGAATCCTGCTGTAGTGGTAAGATTCGAGAA

****
Consensus:

GAAACGTACCAATATAGAGTCAGAATCCTGCTGTAGTGGTAAGATTCGAGAA

>Bacillus_Fam_1051_52_1 Nr. of seq. 1 Alignment length(with gaps) = 52
Alignment score = 0.000000
GCF_000181495.1_ASM18149v1_genomic.fna_174:41004-41469 Satlength=466 Nr
of Repeats=9 RepeatLength=52 seed=AGGAATGATG Num.seqs=6
Similarity=0.909402
0   AGGAATGATGTAGGAATGCAGGGGTAATTAGAGCATCAAATCGGAGAAGTGA

****
Consensus:

AGGAATGATGTAGGAATGCAGGGGTAATTAGAGCATCAAATCGGAGAAGTGA

>Bacillus_Fam_1052_52_1 Nr. of seq. 1 Alignment length(with gaps) = 52
Alignment score = 0.000000
GCF_000181495.1_ASM18149v1_genomic.fna_175:27082-27342 Satlength=261 Nr
of Repeats=5 RepeatLength=52 seed=GGGTGTCTCA Num.seqs=5
Similarity=0.830769
0   GGGTGTCTCATGAACGCTTCATGGGGCCATTTTGCCTGCATATTTGAAGTTA

****
Consensus:

GGGTGTCTCATGAACGCTTCATGGGGCCATTTTGCCTGCATATTTGAAGTTA

>Bacillus_Fam_1053_52_1 Nr. of seq. 1 Alignment length(with gaps) = 52
Alignment score = 0.000000
GCF_000181495.1_ASM18149v1_genomic.fna_178:14811-15278 Satlength=468 Nr
of Repeats=9 RepeatLength=52 seed=GGTGGTCGAA Num.seqs=8
Similarity=0.883700
0   GGTGGTCGAATCCCAAAAAAAGAGAATCGGCCCCGTCAAAGTGCTGCTTCGA

****
Consensus:

GGTGGTCGAATCCCAAAAAAAGAGAATCGGCCCCGTCAAAGTGCTGCTTCGA

>Bacillus_Fam_1054_52_1 Nr. of seq. 1 Alignment length(with gaps) = 52
Alignment score = 0.000000
GCF_000181495.1_ASM18149v1_genomic.fna_186:206325-206533 Satlength=209 Nr of Repeats=4 RepeatLength=52 seed=GGTGGAATTT Num.seqs=4
Similarity=0.751048 0
GGTGGAATTTAGTTTTGGAGAAATCGGCATGGTCAAAGCGGGCGAAAGACAG

****
Consensus:

GGTGGAATTTAGTTTTGGAGAAATCGGCATGGTCAAAGCGGGCGAAAGACAG

>Bacillus_Fam_1055_52_1 Nr. of seq. 1 Alignment length(with gaps) = 52
Alignment score = 0.000000
GCF_000186145.1_Bacillus_sp_2_A_57_CT2_V1_genomic.fna_4:74543-74803
Satlength=261 Nr of Repeats=5 RepeatLength=52 seed=CTCAACCAGA Num.seqs=3
Similarity=0.676955 0
CTCAACCAGAGTCCGAAGTTGTACTAGGTTCGGACTCAAATTNCAGGATGAG

****
Consensus:

CTCAACCAGAGTCCGAAGTTGTACTAGGTTCGGACTCAAATTNCAGGATGAG

>Bacillus_Fam_1056_52_1 Nr. of seq. 1 Alignment length(with gaps) = 52
Alignment score = 0.000000
GCF_000186145.1_Bacillus_sp_2_A_57_CT2_V1_genomic.fna_4:197835-198199
Satlength=365 Nr of Repeats=7 RepeatLength=52 seed=ATGAAGCCGA Num.seqs=7
Similarity=0.710758 0
ATGAAGCCGAAAAGTCGTGNAGCGATAAGAAAACGGTCGCCATGAGGCAGTC

****
Consensus:

ATGAAGCCGAAAAGTCGTGNAGCGATAAGAAAACGGTCGCCATGAGGCAGTC

>Bacillus_Fam_1057_52_1 Nr. of seq. 1 Alignment length(with gaps) = 52
Alignment score = 0.000000
GCF_000186145.1_Bacillus_sp_2_A_57_CT2_V1_genomic.fna_5:287610-288102
Satlength=493 Nr of Repeats=9 RepeatLength=52 seed=GACGAAAATT Num.seqs=7
Similarity=0.750916 0
GACGAAAATTCAGTGGCTCACCAANAAATGGTCGACATAGAGCCTTCATGAA

****
Consensus:

GACGAAAATTCAGTGGCTCACCAANAAATGGTCGACATAGAGCCTTCATGAA

>Bacillus_Fam_1058_52_1 Nr. of seq. 1 Alignment length(with gaps) = 52
Alignment score = 0.000000
GCF_000285535.1_ASM28553v1_genomic.fna_1:1333233-1333493 Satlength=261 Nr of Repeats=5 RepeatLength=52 seed=TCACAGACAA Num.seqs=5
Similarity=0.776923 0
TCACAGACAATTCATTCAACTTTTTACTNCGNTTTGTCTTCGAGACTGGCTA

****
Consensus:

TCACAGACAATTCATTCAACTTTTTACTNCGNTTTGTCTTCGAGACTGGCTA

>Bacillus_Fam_1059_52_1 Nr. of seq. 1 Alignment length(with gaps) = 52
Alignment score = 0.000000
GCF_000285535.1_ASM28553v1_genomic.fna_2:421697-421905 Satlength=209 Nr of Repeats=4 RepeatLength=52 seed=TATGAAGGAC Num.seqs=4
Similarity=0.738994
0 TATGAAGGACTTTCTCACCGGCCAGGCTGCTCGTTCGAGGCTTCATCGGCCT

****
Consensus:

TATGAAGGACTTTCTCACCGGCCAGGCTGCTCGTTCGAGGCTTCATCGGCCT

>Bacillus_Fam_1060_52_1 Nr. of seq. 1 Alignment length(with gaps) = 52
Alignment score = 0.000000
GCF_000285535.1_ASM28553v1_genomic.fna_2:945617-946345 Satlength=729 Nr of Repeats=14 RepeatLength=52 seed=TCAGTTTCTC Num.seqs=14
Similarity=0.766234 0
TCAGTTTCTCAGACCNAATCCCCTTCATAGACCACTTGAAGAGGATACACCT

****
Consensus:

TCAGTTTCTCAGACCNAATCCCCTTCATAGACCACTTGAAGAGGATACACCT

>Bacillus_Fam_1061_52_1 Nr. of seq. 1 Alignment length(with gaps) = 52
Alignment score = 0.000000
GCF_000285535.1_ASM28553v1_genomic.fna_3:73213-73421 Satlength=209 Nr of Repeats=4 RepeatLength=52 seed=CCTCGGCATA Num.seqs=4 Similarity=0.901709
0 CCTCGGCATACTCCGGTTTGTTTTGTCTCCGAGAGCAGCTATCAAACACTTT

****
Consensus:

CCTCGGCATACTCCGGTTTGTTTTGTCTCCGAGAGCAGCTATCAAACACTTT

>Bacillus_Fam_1062_52_1 Nr. of seq. 1 Alignment length(with gaps) = 52
Alignment score = 0.000000
GCF_000285535.1_ASM28553v1_genomic.fna_3:346534-347418 Satlength=885 Nr of Repeats=17 RepeatLength=52 seed=TTCATTGCCT Num.seqs=17
Similarity=0.826934 0
TTCATTGCCTCGATGAAGAGCATAACGCAACAGAACTATGCTCGAATTCCTC

****
Consensus:

TTCATTGCCTCGATGAAGAGCATAACGCAACAGAACTATGCTCGAATTCCTC

>Bacillus_Fam_1063_52_1 Nr. of seq. 1 Alignment length(with gaps) = 52
Alignment score = 0.000000
GCF_000292245.2_ASM29224v2_genomic.fna_3:32494-32754 Satlength=261 Nr of Repeats=5 RepeatLength=52 seed=TTGGACATTC Num.seqs=5 Similarity=0.833333
0   TTGGACATTCTGACTACCTCCACTTTCTCCTTTGTCCTTTAAACTTGGGATA

****
Consensus:

TTGGACATTCTGACTACCTCCACTTTCTCCTTTGTCCTTTAAACTTGGGATA

>Bacillus_Fam_1064_52_1 Nr. of seq. 1 Alignment length(with gaps) = 52
Alignment score = 0.000000
GCF_000292245.2_ASM29224v2_genomic.fna_9:64736-64996 Satlength=261 Nr of Repeats=5 RepeatLength=52 seed=TACATGTTTT Num.seqs=5 Similarity=0.938462
0   TACATGTTTTGGCTGGTTGCTGACCATTATTTGGGTGCCATACTCATTTGGG

****
Consensus:

TACATGTTTTGGCTGGTTGCTGACCATTATTTGGGTGCCATACTCATTTGGG

>Bacillus_Fam_1065_52_1 Nr. of seq. 1 Alignment length(with gaps) = 52
Alignment score = 0.000000
GCF_000292245.2_ASM29224v2_genomic.fna_29:13895-14099 Satlength=205 Nr of Repeats=4 RepeatLength=51 seed=TAATTCGTAA Num.seqs=4 Similarity=0.692387
0   TAATTCGTAATGTAATTTCCCACTATGNACTAATTACGGTGCGTATAACCCC

****
Consensus:

TAATTCGTAATGTAATTTCCCACTATGNACTAATTACGGTGCGTATAACCCC

>Bacillus_Fam_1066_52_1 Nr. of seq. 1 Alignment length(with gaps) = 52
Alignment score = 0.000000
GCF_000294775.2_ASM29477v2_genomic.fna_1:1113586-1113995 Satlength=410 Nr of Repeats=6 RepeatLength=52 seed=GTTCGGACAG Num.seqs=4
Similarity=0.786325 0
GTTCGGACAGGAGAAGAGGAAAAACCGTGGATTGAGTCCGAAGTCGCCCTAT

****
Consensus:

GTTCGGACAGGAGAAGAGGAAAAACCGTGGATTGAGTCCGAAGTCGCCCTAT

>Bacillus_Fam_1067_52_1 Nr. of seq. 1 Alignment length(with gaps) = 52
Alignment score = 0.000000
GCF_000294775.2_ASM29477v2_genomic.fna_1:4788379-4788640 Satlength=262 Nr of Repeats=5 RepeatLength=52 seed=TTCGGACAGA Num.seqs=4
Similarity=0.863248 0
TTCGGACAGACTCTGGACTTTTTCTGCCTTGCCTGTCCGAACTTGCCCCAGG

****
Consensus:

TTCGGACAGACTCTGGACTTTTTCTGCCTTGCCTGTCCGAACTTGCCCCAGG

>Bacillus_Fam_1068_52_1 Nr. of seq. 1 Alignment length(with gaps) = 52
Alignment score = 0.000000
GCF_000307855.1_BAZO1.0_genomic.fna_40:158681-158889 Satlength=209 Nr of Repeats=4 RepeatLength=52 seed=TTTATCCTCA Num.seqs=4 Similarity=0.923077
0 TTTATCCTCACCAAACCGAGCCTAGATGAGGTTTGATAGTAAATAACTCGTG

****
Consensus:

TTTATCCTCACCAAACCGAGCCTAGATGAGGTTTGATAGTAAATAACTCGTG

>Bacillus_Fam_1069_52_1 Nr. of seq. 1 Alignment length(with gaps) = 52
Alignment score = 0.000000
GCF_000307855.1_BAZO1.0_genomic.fna_128:3586-3794 Satlength=209 Nr of Repeats=4 RepeatLength=52 seed=TTTGGTGAGG Num.seqs=4 Similarity=0.829060
0 TTTGGTGAGGATAAAAGCAGGTTATCTACTACCAAAAGTAAGTAGAGTGGAA

****
Consensus:

TTTGGTGAGGATAAAAGCAGGTTATCTACTACCAAAAGTAAGTAGAGTGGAA

>Bacillus_Fam_1070_52_1 Nr. of seq. 1 Alignment length(with gaps) = 52
Alignment score = 0.000000
GCF_000307875.1_BABA1.0_genomic.fna_114:10800-11164 Satlength=365 Nr of Repeats=7 RepeatLength=52 seed=AAAATCTAGC Num.seqs=7 Similarity=0.875458
0 AAAATCTAGCGGACTGTCCTTAATAGGCGCTGTTGAGGACAAAACGGGGTTG

****
Consensus:

AAAATCTAGCGGACTGTCCTTAATAGGCGCTGTTGAGGACAAAACGGGGTTG

>Bacillus_Fam_1071_52_1 Nr. of seq. 1 Alignment length(with gaps) = 52
Alignment score = 0.000000
GCF_000311725.1_ASM31172v1_genomic.fna_4:536064-536636 Satlength=573 Nr of Repeats=11 RepeatLength=52 seed=TCTGTCCGAA Num.seqs=11 Similarity=0.756253 0
TCTGTCCGAATGCACCTCTTATTCGGACTCCTTCACCCTCTCTACTACCTTT

****
Consensus:

TCTGTCCGAATGCACCTCTTATTCGGACTCCTTCACCCTCTCTACTACCTTT

>Bacillus_Fam_1072_52_1 Nr. of seq. 1 Alignment length(with gaps) = 52
Alignment score = 0.000000
GCF_000311725.1_ASM31172v1_genomic.fna_7:13528-13944 Satlength=417 Nr of Repeats=8 RepeatLength=52 seed=ATTCGGACAA Num.seqs=8 Similarity=0.889194
0 ATTCGGACAAGCCTATGGCTTCTTGCTTCCTACTTGTCCGAATATCCTTCCG

****
Consensus:

ATTCGGACAAGCCTATGGCTTCTTGCTTCCTACTTGTCCGAATATCCTTCCG

>Bacillus_Fam_1073_52_1 Nr. of seq. 1 Alignment length(with gaps) = 52
Alignment score = 0.000000
GCF_000331575.1_ASM33157v1_genomic.fna_1:30506-31078 Satlength=573 Nr of Repeats=11 RepeatLength=52 seed=TGTCCGAACT Num.seqs=11 Similarity=0.902098
0 TGTCCGAACTAGGCTTAAGTTCGGACAGAAATGACCGCTTTTTCTCAAATGG

****
Consensus:

TGTCCGAACTAGGCTTAAGTTCGGACAGAAATGACCGCTTTTTCTCAAATGG

>Bacillus_Fam_1074_52_1 Nr. of seq. 1 Alignment length(with gaps) = 52
Alignment score = 0.000000
GCF_000331575.1_ASM33157v1_genomic.fna_3:87769-87977 Satlength=209 Nr of Repeats=4 RepeatLength=52 seed=ATTGCGTTGC Num.seqs=4 Similarity=0.871795
0 ATTGCGTTGCTCTCGATCAAGAAATGGACTTCATAGCCGAGGTAACGATCGT

****
Consensus:

ATTGCGTTGCTCTCGATCAAGAAATGGACTTCATAGCCGAGGTAACGATCGT

>Bacillus_Fam_1075_52_1 Nr. of seq. 1 Alignment length(with gaps) = 52
Alignment score = 0.000000
GCF_000374345.1_ASM37434v1_genomic.fna_4:25818-26244 Satlength=427 Nr of Repeats=6 RepeatLength=52 seed=CGTTCATAAG Num.seqs=5 Similarity=0.779487
0 CGTTCATAAGGCGGATGAAAGCCATTGTAAAGAGAAAAAACAGGTGAGAAGG

****
Consensus:

CGTTCATAAGGCGGATGAAAGCCATTGTAAAGAGAAAAAACAGGTGAGAAGG

>Bacillus_Fam_1076_52_1 Nr. of seq. 1 Alignment length(with gaps) = 52
Alignment score = 0.000000
GCF_000374345.1_ASM37434v1_genomic.fna_7:32632-33048 Satlength=417 Nr of Repeats=8 RepeatLength=52 seed=AATGGCGTTC Num.seqs=8 Similarity=0.884615
0 AATGGCGTTCATCAGCCGTATGAACGCTATCTCTACCGGGGTTGATCCTTGA

****
Consensus:

AATGGCGTTCATCAGCCGTATGAACGCTATCTCTACCGGGGTTGATCCTTGA

>Bacillus_Fam_1077_52_1 Nr. of seq. 1 Alignment length(with gaps) = 52
Alignment score = 0.000000

GCF_000374345.1_ASM37434v1_genomic.fna_7:56200-56512 Satlength=313 Nr of Repeats=6 RepeatLength=52 seed=CGGGCTTTCA Num.seqs=6 Similarity=0.731070
0 CGGGCTTTCATATCCCCAATGAACGACCGTTTCCCCGGGCACTTGCCTGATT

****
Consensus:

CGGGCTTTCATATCCCCAATGAACGACCGTTTCCCCGGGCACTTGCCTGATT

>Bacillus_Fam_1078_52_1 Nr. of seq. 1 Alignment length(with gaps) = 52 Alignment score = 0.000000
GCF_000374345.1_ASM37434v1_genomic.fna_9:65991-66356 Satlength=366 Nr of Repeats=7 RepeatLength=52 seed=GCGAAATGAA Num.seqs=6 Similarity=0.789727
0 GCGAAATGAAGTTCATCAGGCCTATGAAAGCAATCGCGGCCGAAATTTTAGA

****
Consensus:

GCGAAATGAAGTTCATCAGGCCTATGAAAGCAATCGCGGCCGAAATTTTAGA

>Bacillus_Fam_1079_52_1 Nr. of seq. 1 Alignment length(with gaps) = 52 Alignment score = 0.000000
GCF_000374345.1_ASM37434v1_genomic.fna_24:14134-14498 Satlength=365 Nr of Repeats=7 RepeatLength=52 seed=GGGCTTTCAT Num.seqs=7 Similarity=0.714585
0 GGGCTTTCATTCGACCGATGATCGACCGCGCGGATGGTTTTTCTCTCGCGT

****
Consensus:

GGGCTTTCATTCGACCGATGATCGACCGCGCGGATGGTTTTTCTCTCGCGT

>Bacillus_Fam_1080_52_1 Nr. of seq. 1 Alignment length(with gaps) = 52 Alignment score = 0.000000
GCF_000374345.1_ASM37434v1_genomic.fna_26:26410-26774 Satlength=365 Nr of Repeats=6 RepeatLength=52 seed=ATGAACGCGA Num.seqs=5 Similarity=0.803086
0 ATGAACGCGATCACGAATGAAAAAGGGGGTTACGATGGTGTTCATAAGNCCG

****
Consensus:

ATGAACGCGATCACGAATGAAAAAGGGGGTTACGATGGTGTTCATAAGNCCG

>Bacillus_Fam_1081_52_1 Nr. of seq. 1 Alignment length(with gaps) = 52 Alignment score = 0.000000
GCF_000374345.1_ASM37434v1_genomic.fna_44:13486-13902 Satlength=417 Nr of Repeats=8 RepeatLength=52 seed=ATAGCGTTCA Num.seqs=8 Similarity=0.722527
0 ATAGCGTTCATCCCGGGTATGAACTTCATCTTGANTATCTTTTTTACTTGNA

****
Consensus:

ATAGCGTTCATCCCGGGTATGAACTTCATCTTGANTATCTTTTTTACTTGNA

>Bacillus_Fam_1082_52_1 Nr. of seq. 1 Alignment length(with gaps) = 52
Alignment score = 0.000000
GCF_000374345.1_ASM37434v1_genomic.fna_80:5979-6392 Satlength=414 Nr of Repeats=8 RepeatLength=52 seed=GCTTATGAAC Num.seqs=5 Similarity=0.861538
0   GCTTATGAACTGCATTTGGCATGGATTTTTTTACCGAGATAGCGTTCATTCC

****
Consensus:

GCTTATGAACTGCATTTGGCATGGATTTTTTTACCGAGATAGCGTTCATTCC

>Bacillus_Fam_1083_52_1 Nr. of seq. 1 Alignment length(with gaps) = 52
Alignment score = 0.000000
GCF_000374565.1_ASM37456v1_genomic.fna_1:689690-690002 Satlength=313 Nr of Repeats=6 RepeatLength=52 seed=CGGGCACTTT Num.seqs=6
Similarity=0.786373
0   CGGGCACTTTTAGCCTCTATTAGTGACCGGTTCTCTCGACAAACTCTATTTG

****
Consensus:

CGGGCACTTTTAGCCTCTATTAGTGACCGGTTCTCTCGACAAACTCTATTTG

>Bacillus_Fam_1084_52_1 Nr. of seq. 1 Alignment length(with gaps) = 52
Alignment score = 0.000000
GCF_000374565.1_ASM37456v1_genomic.fna_3:204349-204867 Satlength=519 Nr of Repeats=10 RepeatLength=52 seed=AATCGGTAAC Num.seqs=8
Similarity=0.749084
0   AATCGGTAACTAATAGGCAGGAAAGATGCCCGAATCCGNTGTGTCGTCCCGC

****
Consensus:

AATCGGTAACTAATAGGCAGGAAAGATGCCCGAATCCGNTGTGTCGTCCCGC

>Bacillus_Fam_1085_52_1 Nr. of seq. 1 Alignment length(with gaps) = 52
Alignment score = 0.000000
GCF_000374565.1_ASM37456v1_genomic.fna_5:148962-149638 Satlength=677 Nr of Repeats=13 RepeatLength=52 seed=CGTATATCCC Num.seqs=13
Similarity=0.844541 0
CGTATATCCCAAAAAGCGGTCACCAATCGTATGGATTAGTGCCCGTTCTGAG

****
Consensus:

CGTATATCCCAAAAAGCGGTCACCAATCGTATGGATTAGTGCCCGTTCTGAG

>Bacillus_Fam_1086_52_1 Nr. of seq. 1 Alignment length(with gaps) = 52
Alignment score = 0.000000
GCF_000374565.1_ASM37456v1_genomic.fna_6:158147-158511 Satlength=365 Nr of Repeats=7 RepeatLength=52 seed=AAAAGCGGTC Num.seqs=5
Similarity=0.769811
0   AAAAGCGGTCACGATTAGTGAGGAAAGATGCCCGTTTTAGTCGGTTTTCAGT

****
Consensus:

AAAAGCGGTCACGATTAGTGAGGAAAGATGCCCGTTTTAGTCGGTTTTCAGT

>Bacillus_Fam_1087_52_1 Nr. of seq. 1 Alignment length(with gaps) = 52
Alignment score = 0.000000
GCF_000374565.1_ASM37456v1_genomic.fna_7:2370-2942 Satlength=573 Nr of Repeats=11 RepeatLength=52 seed=TATTTGTGAC Num.seqs=11 Similarity=0.857343
0   TATTTGTGACCGGATTTCGTATTTCTCCCTTCAAACGGGCATCTTCCACTAC

****
Consensus:

TATTTGTGACCGGATTTCGTATTTCTCCCTTCAAACGGGCATCTTCCACTAC

>Bacillus_Fam_1088_52_1 Nr. of seq. 1 Alignment length(with gaps) = 52
Alignment score = 0.000000
GCF_000374565.1_ASM37456v1_genomic.fna_12:88054-88651 Satlength=598 Nr of Repeats=8 RepeatLength=52 seed=TCACTAAAAA Num.seqs=7 Similarity=0.860806
0   TCACTAAAAAGTGCTAAAAGTGCCCGTAATGTGGTGAATCACGAAAATCCGG

****
Consensus:

TCACTAAAAAGTGCTAAAAGTGCCCGTAATGTGGTGAATCACGAAAATCCGG

>Bacillus_Fam_1089_52_1 Nr. of seq. 1 Alignment length(with gaps) = 52
Alignment score = 0.000000
GCF_000374565.1_ASM37456v1_genomic.fna_16:33589-34317 Satlength=729 Nr of Repeats=12 RepeatLength=52 seed=ACCGCTTTTT Num.seqs=10 Similarity=0.750105
0   ACCGCTTTTTACCAAAATCTCTCGTAACGGGCACCTTTCTCCAACATTAGTG

****
Consensus:

ACCGCTTTTTACCAAAATCTCTCGTAACGGGCACCTTTCTCCAACATTAGTG

>Bacillus_Fam_1090_52_1 Nr. of seq. 1 Alignment length(with gaps) = 52
Alignment score = 0.000000
GCF_000374565.1_ASM37456v1_genomic.fna_22:20071-20488 Satlength=418 Nr of Repeats=8 RepeatLength=52 seed=ACCAATAAAG Num.seqs=7 Similarity=0.912088
0   ACCAATAAAGGTTAATAGTGCCCGAAAAGGAGAAAAAAGTGCTCTAATGGTC

****
Consensus:

ACCAATAAAGGTTAATAGTGCCCGAAAAGGAGAAAAAAGTGCTCTAATGGTC

>Bacillus_Fam_1091_52_1 Nr. of seq. 1 Alignment length(with gaps) = 52
Alignment score = 0.000000

GCF_000374565.1_ASM37456v1_genomic.fna_29:30054-30365 Satlength=312 Nr of Repeats=6 RepeatLength=52 seed=GATTAGTGAC Num.seqs=5 Similarity=0.826415
50   TTAGTGACCGNTTTTCGGATTAACGCAACTTTTCGGGCATCTTTTACTCCGA

****
Consensus:

GATTAGTGACCGNTTTTCGGATTAACGCAACTTTTCGGGCATCTTTTACTCC

>Bacillus_Fam_1092_52_1 Nr. of seq. 1 Alignment length(with gaps) = 52 Alignment score = 0.000000
GCF_000401235.1_BacNeaAAU1_genomic.fna_144:20-332 Satlength=313 Nr of Repeats=6 RepeatLength=52 seed=GTTCATCTTG Num.seqs=6 Similarity=0.859829
0   GTTCATCTTGTTTATGAACGATAGTTTTGCCCTCTTCTTTTGAAAAAGTGGC

****
Consensus:

GTTCATCTTGTTTATGAACGATAGTTTTGCCCTCTTCTTTTGAAAAAGTGGC

>Bacillus_Fam_1093_52_1 Nr. of seq. 1 Alignment length(with gaps) = 52 Alignment score = 0.000000
GCF_000401235.1_BacNeaAAU1_genomic.fna_153:115-374 Satlength=260 Nr of Repeats=5 RepeatLength=52 seed=TGGCTTTCAT Num.seqs=4 Similarity=0.871795
0   TGGCTTTCATTCCCTTGATGAACGCCACTTTCCTCCAAGATTACCTTAAAAC

****
Consensus:

TGGCTTTCATTCCCTTGATGAACGCCACTTTCCTCCAAGATTACCTTAAAAC

>Bacillus_Fam_1094_52_1 Nr. of seq. 1 Alignment length(with gaps) = 52 Alignment score = 0.000000
GCF_000430765.1_ASM43076v1_genomic.fna_3:286133-286444 Satlength=312 Nr of Repeats=6 RepeatLength=52 seed=TGTCCGAAGT Num.seqs=5 Similarity=0.861538
0   TGTCCGAAGTGGGTCTGTGTTCGGACAACTTCCGGATGAATCCAAGCCAACT

****
Consensus:

TGTCCGAAGTGGGTCTGTGTTCGGACAACTTCCGGATGAATCCAAGCCAACT

>Bacillus_Fam_1095_52_1 Nr. of seq. 1 Alignment length(with gaps) = 52 Alignment score = 0.000000
GCF_000430765.1_ASM43076v1_genomic.fna_5:148049-148621 Satlength=573 Nr of Repeats=11 RepeatLength=52 seed=TCTCTGAACC Num.seqs=9 Similarity=0.735755
0   TCTCTGAACCATTTNCCAGCGGAGCAGCCTCNGTTTTGGTCTTGAGACGCCT

****
Consensus:

TCTCTGAACCATTTNCCAGCGGAGCAGCCTCNGTTTTGGTCTTGAGACGCCT

>Bacillus_Fam_1096_52_1 Nr. of seq. 1 Alignment length(with gaps) = 52
Alignment score = 0.000000
GCF_000430765.1_ASM43076v1_genomic.fna_6:140008-140268 Satlength=261 Nr of Repeats=5 RepeatLength=52 seed=TCTCATGACC Num.seqs=5 Similarity=0.979487
0   TCTCATGACCAAAACTTCTGCCGCTTCCCCTCTTTATGGTTCTGAGACGCAC

****
Consensus:

TCTCATGACCAAAACTTCTGCCGCTTCCCCTCTTTATGGTTCTGAGACGCAC

>Bacillus_Fam_1097_52_1 Nr. of seq. 1 Alignment length(with gaps) = 52
Alignment score = 0.000000
GCF_000430765.1_ASM43076v1_genomic.fna_7:289548-290021 Satlength=474 Nr of Repeats=9 RepeatLength=52 seed=CCTCTCAAGA Num.seqs=6 Similarity=0.858700
0   CCTCTCAAGACCAAAATTCCTTTAAGCAAGCGGAGAAGTGGTTCAGAGAGGG

****
Consensus:

CCTCTCAAGACCAAAATTCCTTTAAGCAAGCGGAGAAGTGGTTCAGAGAGGG

>Bacillus_Fam_1098_52_1 Nr. of seq. 1 Alignment length(with gaps) = 52
Alignment score = 0.000000
GCF_000430765.1_ASM43076v1_genomic.fna_9:15-224 Satlength=210 Nr of Repeats=4 RepeatLength=52 seed=GGATGACGAA Num.seqs=3 Similarity=0.811321
0   GGATGACGAAGATAGGTCAATGAGAAGGGTTCTAATGGACACTACGCTGAAN

****
Consensus:

GGATGACGAAGATAGGTCAATGAGAAGGGTTCTAATGGACACTACGCTGAAN

>Bacillus_Fam_1099_52_1 Nr. of seq. 1 Alignment length(with gaps) = 52
Alignment score = 0.000000
GCF_000430765.1_ASM43076v1_genomic.fna_12:149140-149400 Satlength=261 Nr of Repeats=5 RepeatLength=52 seed=TCAGGACCAA Num.seqs=5 Similarity=0.948718
7   GGGCGTCTCAGGACCAAAAACAAAGACAGAGCTGGTGAAAGTGGTTCAGAGG

****
Consensus:

TCAGGACCAAAAACAAAGACAGAGCTGGTGAAAGTGGTTCAGAGGGGGCGTC

>Bacillus_Fam_1100_52_1 Nr. of seq. 1 Alignment length(with gaps) = 52
Alignment score = 0.000000
GCF_000473245.1_ASM47324v1_genomic.fna_1:4633527-4633839 Satlength=313 Nr of Repeats=6 RepeatLength=52 seed=GGGTCTTAGT Num.seqs=6 Similarity=0.859829 0
GGGTCTTAGTTTCTCTGACTAGCACCCTCTGGTTTGGTTTTTGTGATTCAGA

****
Consensus:

GGGTCTTAGTTTCTCTGACTAGCACCCTCTGGTTTGGTTTTTGTGATTCAGA

>Bacillus_Fam_1101_52_1 Nr. of seq. 1 Alignment length(with gaps) = 52
Alignment score = 0.000000
GCF_000508325.1_BAVI_1_genomic.fna_69:22489-22749 Satlength=261 Nr of
Repeats=5 RepeatLength=52 seed=CGATAAGCTA Num.seqs=5 Similarity=0.775472
0 CGATAAGCTACCCAACTGGTTCTTTCTTGATGGATTCGGTCGCTTATAANCC

****
Consensus:

CGATAAGCTACCCAACTGGTTCTTTCTTGATGGATTCGGTCGCTTATAANCC

>Bacillus_Fam_1102_52_1 Nr. of seq. 1 Alignment length(with gaps) = 52
Alignment score = 0.000000
GCF_000508325.1_BAVI_1_genomic.fna_161:5891-6101 Satlength=211 Nr of
Repeats=4 RepeatLength=52 seed=GGGCACTCTG Num.seqs=3 Similarity=0.846154
0 GGGCACTCTGGCTACTCCACAGTGCCCGGTTGATGCTTTTTTCTCCTCACAT

****
Consensus:

GGGCACTCTGGCTACTCCACAGTGCCCGGTTGATGCTTTTTTCTCCTCACAT

>Bacillus_Fam_1103_52_1 Nr. of seq. 1 Alignment length(with gaps) = 52
Alignment score = 0.000000
GCF_000612665.1_FF4_genomic.fna_19:82518-82985 Satlength=468 Nr of
Repeats=9 RepeatLength=52 seed=CGGTCATTAA Num.seqs=8 Similarity=0.904762
0 CGGTCATTAAGCGGGGCTCTAAATGCCCGCGGAGAGCGAAAAAGGAAGGGAG

****
Consensus:

CGGTCATTAAGCGGGGCTCTAAATGCCCGCGGAGAGCGAAAAAGGAAGGGAG

>Bacillus_Fam_1104_52_1 Nr. of seq. 1 Alignment length(with gaps) = 52
Alignment score = 0.000000
GCF_000612665.1_FF4_genomic.fna_65:35468-35676 Satlength=209 Nr of
Repeats=4 RepeatLength=52 seed=TCTTAGTTAC Num.seqs=4 Similarity=0.850427
0 TCTTAGTTACCGCGAGACCCGCATTTTCTCCTTCGCGGGCATTTAGAGCCCT

****
Consensus:

TCTTAGTTACCGCGAGACCCGCATTTTCTCCTTCGCGGGCATTTAGAGCCCT

>Bacillus_Fam_1105_52_1 Nr. of seq. 1 Alignment length(with gaps) = 52
Alignment score = 0.000000

GCF_000612665.1_FF4_genomic.fna_66:181657-182736 Satlength=1080 Nr of Repeats=9 RepeatLength=52 seed=TTATAGCCTT Num.seqs=8 Similarity=0.807692
0 TTATAGCCTTTATAAATGACCGACCGACCGGAAAAANCGCCAGCTCGGGCAT

****
Consensus:

TTATAGCCTTTATAAATGACCGACCGACCGGAAAAANCGCCAGCTCGGGCAT

>Bacillus_Fam_1106_52_1 Nr. of seq. 1 Alignment length(with gaps) = 52 Alignment score = 0.000000
GCF_000708755.2_ASM70875v2_genomic.fna_1:477291-477603 Satlength=313 Nr of Repeats=6 RepeatLength=52 seed=AACCAAGTAA Num.seqs=6 Similarity=0.882051
0 AACCAAGTAAGTGTTCTATACCGTCAATAGCAACACTTTCCCTCATCAATTG

****
Consensus:

AACCAAGTAAGTGTTCTATACCGTCAATAGCAACACTTTCCCTCATCAATTG

>Bacillus_Fam_1107_52_1 Nr. of seq. 1 Alignment length(with gaps) = 52 Alignment score = 0.000000
GCF_000708755.2_ASM70875v2_genomic.fna_1:508584-508844 Satlength=261 Nr of Repeats=4 RepeatLength=52 seed=TCGTTTAAGA Num.seqs=3 Similarity=0.836478
0 TCGTTTAAGAGCACTTTGGTCTTGCTGTGAATCGGAGTTCTTGTTGCAAACA

****
Consensus:

TCGTTTAAGAGCACTTTGGTCTTGCTGTGAATCGGAGTTCTTGTTGCAAACA

>Bacillus_Fam_1108_52_1 Nr. of seq. 1 Alignment length(with gaps) = 52 Alignment score = 0.000000
GCF_000708755.2_ASM70875v2_genomic.fna_2:662686-662998 Satlength=313 Nr of Repeats=5 RepeatLength=52 seed=TATTCAGCCG Num.seqs=4 Similarity=0.863248
0 TATTCAGCCGCAACCAATTTATAGGGTTGCATTTCGCCAGTATGGTACCCTC

****
Consensus:

TATTCAGCCGCAACCAATTTATAGGGTTGCATTTCGCCAGTATGGTACCCTC

>Bacillus_Fam_1109_52_1 Nr. of seq. 1 Alignment length(with gaps) = 52 Alignment score = 0.000000
GCF_000708755.2_ASM70875v2_genomic.fna_2:713425-713736 Satlength=312 Nr of Repeats=6 RepeatLength=52 seed=TAGAACACTT Num.seqs=5 Similarity=0.723077
0 TAGAACACTTTCTGCGGTCGGGATNCCCNACTTAATGTTGCTTACTCCGGTT

****
Consensus:

TAGAACACTTTCTGCGGTCGGGATNCCCNACTTAATGTTGCTTACTCCGGTT

>Bacillus_Fam_1110_52_1 Nr. of seq. 1 Alignment length(with gaps) = 52
Alignment score = 0.000000
GCF_000708755.2_ASM70875v2_genomic.fna_2:1183664-1183924 Satlength=261 Nr of Repeats=5 RepeatLength=52 seed=CAAAATGTTG Num.seqs=5
Similarity=0.784906 0
CAAAATGTTGCTAAAGCCAGGTTAGAACACTTTCNGGGCTTTCTGGCCTCTG

****
Consensus:

CAAAATGTTGCTAAAGCCAGGTTAGAACACTTTCNGGGCTTTCTGGCCTCTG

>Bacillus_Fam_1111_52_1 Nr. of seq. 1 Alignment length(with gaps) = 52
Alignment score = 0.000000
GCF_000708755.2_ASM70875v2_genomic.fna_2:1324156-1324364 Satlength=209 Nr of Repeats=4 RepeatLength=52 seed=CTGAAGGGCA Num.seqs=4
Similarity=0.854701 0
CTGAAGGGCACATTCCTGCCTGGTTTTCGACCTTCAGCTTACTTCCTTTTCC

****
Consensus:

CTGAAGGGCACATTCCTGCCTGGTTTTCGACCTTCAGCTTACTTCCTTTTCC

>Bacillus_Fam_1112_52_1 Nr. of seq. 1 Alignment length(with gaps) = 52
Alignment score = 0.000000
GCF_000708755.2_ASM70875v2_genomic.fna_2:1620621-1620829 Satlength=209 Nr of Repeats=4 RepeatLength=52 seed=ACCCTTCACA Num.seqs=4
Similarity=0.735043 0
ACCCTTCACACAATCCCACTCCTGATAAACGGTCATATCTTATCCACTTATA

****
Consensus:

ACCCTTCACACAATCCCACTCCTGATAAACGGTCATATCTTATCCACTTATA

>Bacillus_Fam_1113_52_1 Nr. of seq. 1 Alignment length(with gaps) = 52
Alignment score = 0.000000
GCF_000708755.2_ASM70875v2_genomic.fna_2:1684021-1684385 Satlength=365 Nr of Repeats=7 RepeatLength=52 seed=ACCTTCAGCT Num.seqs=5
Similarity=0.810256 0
ACCTTCAGCTTCTTCTCCACCAACTGAAGGGCACGATCCATCCGCTTTTGTG

****
Consensus:

ACCTTCAGCTTCTTCTCCACCAACTGAAGGGCACGATCCATCCGCTTTTGTG

>Bacillus_Fam_1114_52_1 Nr. of seq. 1 Alignment length(with gaps) = 52
Alignment score = 0.000000

GCF_000708755.2_ASM70875v2_genomic.fna_6:178665-178925 Satlength=261 Nr of Repeats=5 RepeatLength=52 seed=TAAGTGCGCT Num.seqs=5 Similarity=0.789091
0 TAAGTGCGCTTATCTCGGTTTTAGAACACTTTCACAGATANTGCNCTTTGGC

****
Consensus:

TAAGTGCGCTTATCTCGGTTTTAGAACACTTTCACAGATANTGCNCTTTGGC

>Bacillus_Fam_1115_52_1 Nr. of seq. 1 Alignment length(with gaps) = 52 Alignment score = 0.000000
GCF_000708755.2_ASM70875v2_genomic.fna_6:348631-348839 Satlength=209 Nr of Repeats=4 RepeatLength=52 seed=GCTGCGGCGG Num.seqs=4 Similarity=0.841880
0 GCTGCGGCGGAAAGGCATGAAGGGTCATAAGAGGCTTGGATTGTTACCTTCA

****
Consensus:

GCTGCGGCGGAAAGGCATGAAGGGTCATAAGAGGCTTGGATTGTTACCTTCA

>Bacillus_Fam_1116_52_1 Nr. of seq. 1 Alignment length(with gaps) = 52 Alignment score = 0.000000
GCF_000708755.2_ASM70875v2_genomic.fna_7:130520-130728 Satlength=209 Nr of Repeats=4 RepeatLength=52 seed=TTTCCTCCAG Num.seqs=4 Similarity=0.829060
0 TTTCCTCCAGGCTCACGCTCCACTTTGTGCTCTTAACCCCGCGGAAGCGCAC

****
Consensus:

TTTCCTCCAGGCTCACGCTCCACTTTGTGCTCTTAACCCCGCGGAAGCGCAC

>Bacillus_Fam_1117_52_1 Nr. of seq. 1 Alignment length(with gaps) = 52 Alignment score = 0.000000
GCF_000709935.2_ASM70993v2_genomic.fna_1:71045-71305 Satlength=261 Nr of Repeats=5 RepeatLength=52 seed=GAAATATAGG Num.seqs=5 Similarity=0.762963
0 GAAATATAGGGCAGCAAAACATGAAAATGCAACCCTATAAAATGCGCCATCT

****
Consensus:

GAAATATAGGGCAGCAAAACATGAAAATGCAACCCTATAAAATGCGCCATCT

>Bacillus_Fam_1118_52_1 Nr. of seq. 1 Alignment length(with gaps) = 52 Alignment score = 0.000000
GCF_000709935.2_ASM70993v2_genomic.fna_1:1027951-1028263 Satlength=313 Nr of Repeats=6 RepeatLength=52 seed=CTGTATTTTG Num.seqs=6 Similarity=0.776520 0
CTGTATTTTGGACAAGCNGATTTATAGGGCAGCATTTCACCTTTTTGGTGCC

****
Consensus:

CTGTATTTTGGACAAGCNGATTTATAGGGCAGCATTTCACCTTTTTGGTGCC

>Bacillus_Fam_1119_52_1 Nr. of seq. 1 Alignment length(with gaps) = 52 Alignment score = 0.000000 GCF_000709935.2_ASM70993v2_genomic.fna_1:1073715-1073923 Satlength=209 Nr of Repeats=4 RepeatLength=52 seed=CATAAGTGCT Num.seqs=4 Similarity=0.688034 0
CATAAGTGCTGCAAAGCTGGTTTAACAACATAAACTCAGCTGCAGGAAGCCG

****
Consensus:

CATAAGTGCTGCAAAGCTGGTTTAACAACATAAACTCAGCTGCAGGAAGCCG

>Bacillus_Fam_1120_52_1 Nr. of seq. 1 Alignment length(with gaps) = 52 Alignment score = 0.000000 GCF_000709935.2_ASM70993v2_genomic.fna_5:462972-463648 Satlength=677 Nr of Repeats=13 RepeatLength=52 seed=TTCTTATCGG Num.seqs=13 Similarity=0.793904 0
TTCTTATCGGACGAATNGGTGCCATCAAANCTGATTCTTCTCTGCTTGAGGG

****
Consensus:

TTCTTATCGGACGAATNGGTGCCATCAAANCTGATTCTTCTCTGCTTGAGGG

>Bacillus_Fam_1121_52_1 Nr. of seq. 1 Alignment length(with gaps) = 52 Alignment score = 0.000000 GCF_000709935.2_ASM70993v2_genomic.fna_19:19072-19436 Satlength=365 Nr of Repeats=7 RepeatLength=52 seed=GAACCCTCAA Num.seqs=7 Similarity=0.848596
0 GAACCCTCAAGCTTTGGATTCTGTGGATTGATGGCACTTATAGCAGAGTTTA

****
Consensus:

GAACCCTCAAGCTTTGGATTCTGTGGATTGATGGCACTTATAGCAGAGTTTA

>Bacillus_Fam_1122_52_1 Nr. of seq. 1 Alignment length(with gaps) = 52 Alignment score = 0.000000 GCF_000709935.2_ASM70993v2_genomic.fna_19:109951-110159 Satlength=209 Nr of Repeats=4 RepeatLength=52 seed=AAGTGTTCTT Num.seqs=4 Similarity=0.769231
0 AAGTGTTCTTATCCTGCTTTTACAGCACTTATCTTCGCTCCGGAGTCACTCA

****
Consensus:

AAGTGTTCTTATCCTGCTTTTACAGCACTTATCTTCGCTCCGGAGTCACTCA

>Bacillus_Fam_1123_52_1 Nr. of seq. 1 Alignment length(with gaps) = 52 Alignment score = 0.000000 GCF_000752035.1_Bacillus_rubiinfantis_genomic.fna_11:243707-244693 Satlength=987 Nr of Repeats=19 RepeatLength=52 seed=AGCGGGTAAA

Num.seqs=17 Similarity=0.943439 0
AGCGGGTAAAATAAAGCGCCGTTTTTACCGGTGATAGCCTTTTTCGCTCC

****
Consensus:

AGCGGGTAAAATAAAGCGCCGTTTTTACCGGTGATAGCCTTTTTCGCTCC

>Bacillus_Fam_1124_52_1 Nr. of seq. 1 Alignment length(with gaps) = 52
Alignment score = 0.000000
GCF_000752035.1_Bacillus_rubiinfantis_genomic.fna_11:495149-495669
Satlength=521 Nr of Repeats=10 RepeatLength=52 seed=CGCTTATACC
Num.seqs=10 Similarity=0.908262 0
CGCTTATACCCTTTATTGGTGACCGAACCAGTGTCAAACCCATGGTTCCGGG

****
Consensus:

CGCTTATACCCTTTATTGGTGACCGAACCAGTGTCAAACCCATGGTTCCGGG

>Bacillus_Fam_1125_52_1 Nr. of seq. 1 Alignment length(with gaps) = 52
Alignment score = 0.000000
GCF_000752035.1_Bacillus_rubiinfantis_genomic.fna_24:501342-502175
Satlength=834 Nr of Repeats=16 RepeatLength=52 seed=TTATTGGCGA
Num.seqs=15 Similarity=0.867155 0
TTATTGGCGACCGCTCACTACTGGAGNTCCTTCGCTCGGGCGCTTATCTCCC

****
Consensus:

TTATTGGCGACCGCTCACTACTGGAGNTCCTTCGCTCGGGCGCTTATCTCCC

>Bacillus_Fam_1126_52_1 Nr. of seq. 1 Alignment length(with gaps) = 52
Alignment score = 0.000000
GCF_000752395.1_Bacillus_andreraoultii_genomic.fna_24:95882-96453
Satlength=572 Nr of Repeats=11 RepeatLength=52 seed=GACCTTTTCA
Num.seqs=10 Similarity=0.829060
0 GACCTTTTCAACCTTTTTCACCACAATTCGGGCGTCTACAACTCTTCTAGAG

****
Consensus:

GACCTTTTCAACCTTTTTCACCACAATTCGGGCGTCTACAACTCTTCTAGAG

>Bacillus_Fam_1127_52_1 Nr. of seq. 1 Alignment length(with gaps) = 52
Alignment score = 0.000000
GCF_000752395.1_Bacillus_andreraoultii_genomic.fna_43:160186-160819
Satlength=634 Nr of Repeats=4 RepeatLength=52 seed=AGGGTTCTCA Num.seqs=3
Similarity=0.948718 0
AGGGTTCTCAAGGACAAAGTGCACGCAAAAACCGGTGAAACTGTCCTAGACA

****
Consensus:

AGGGTTCTCAAGGACAAAGTGCACGCAAAAACCGGTGAAACTGTCCTAGACA

>Bacillus_Fam_1128_52_1 Nr. of seq. 1 Alignment length(with gaps) = 52
Alignment score = 0.000000
GCF_000759675.1_ASM75967v1_genomic.fna_19:237050-237362 Satlength=313 Nr of Repeats=6 RepeatLength=52 seed=TCTTAAGGAC Num.seqs=6
Similarity=0.936752
0 TCTTAAGGACAAATCTTCTAGTTCTCTTTAGAGATTTGTCCTTCATACCCCT

****
Consensus:

TCTTAAGGACAAATCTTCTAGTTCTCTTTAGAGATTTGTCCTTCATACCCCT

>Bacillus_Fam_1129_52_1 Nr. of seq. 1 Alignment length(with gaps) = 52
Alignment score = 0.000000
GCF_000759675.1_ASM75967v1_genomic.fna_126:195884-196092 Satlength=209 Nr of Repeats=4 RepeatLength=52 seed=GTAACGATTA Num.seqs=4
Similarity=0.850427 0
GTAACGATTAGTCGCTAATCGTGCCCTTCTCCTCGATTTTTTGGAGTTTGCG

****
Consensus:

GTAACGATTAGTCGCTAATCGTGCCCTTCTCCTCGATTTTTTGGAGTTTGCG

>Bacillus_Fam_1130_52_1 Nr. of seq. 1 Alignment length(with gaps) = 52
Alignment score = 0.000000
GCF_000759675.1_ASM75967v1_genomic.fna_151:38292-38799 Satlength=508 Nr of Repeats=8 RepeatLength=52 seed=TATGAGTGAC Num.seqs=6
Similarity=0.814675
0 TATGAGTGACCGCTTGTCCTTTTCCAGGAGGTTCTTGGGCTCTCATGAACCT

****
Consensus:

TATGAGTGACCGCTTGTCCTTTTCCAGGAGGTTCTTGGGCTCTCATGAACCT

>Bacillus_Fam_1131_52_1 Nr. of seq. 1 Alignment length(with gaps) = 52
Alignment score = 0.000000
GCF_000759675.1_ASM75967v1_genomic.fna_158:33598-34014 Satlength=417 Nr of Repeats=8 RepeatLength=52 seed=TGTGACCAAA Num.seqs=8
Similarity=0.889194
0 TGTGACCAAAACTCATGGATGAAACTATCAAAATGGTCACAAGAAGGGTCGC

****
Consensus:

TGTGACCAAAACTCATGGATGAAACTATCAAAATGGTCACAAGAAGGGTCGC

>Bacillus_Fam_1132_52_1 Nr. of seq. 1 Alignment length(with gaps) = 52
Alignment score = 0.000000

GCF_000787375.1_ASM78737v1_genomic.fna_1:349174-349686 Satlength=513 Nr of Repeats=4 RepeatLength=52 seed=CTTGCTCTTT Num.seqs=3 Similarity=0.880342
0   CTTGCTCTTTTTCGGTAGGCAATACATCTTGTTGTCTACTCTTATTGCGGCT

****
Consensus:

CTTGCTCTTTTTCGGTAGGCAATACATCTTGTTGTCTACTCTTATTGCGGCT

>Bacillus_Fam_1133_52_1 Nr. of seq. 1 Alignment length(with gaps) = 52 Alignment score = 0.000000
GCF_000787375.1_ASM78737v1_genomic.fna_2:125037-125349 Satlength=313 Nr of Repeats=5 RepeatLength=52 seed=CTACCCAAAT Num.seqs=4 Similarity=0.790598
0   CTACCCAAATACGGGGGACCTCTCAAGTTCGGGCGCCATCACATCCCTTTGA

****
Consensus:

CTACCCAAATACGGGGGACCTCTCAAGTTCGGGCGCCATCACATCCCTTTGA

>Bacillus_Fam_1134_52_1 Nr. of seq. 1 Alignment length(with gaps) = 52 Alignment score = 0.000000
GCF_000787375.1_ASM78737v1_genomic.fna_2:172325-172585 Satlength=261 Nr of Repeats=5 RepeatLength=52 seed=CTCTTTTCGG Num.seqs=5 Similarity=0.853846
26   TTGACTCCCTTCCTCACNCCTCCTCACTCTTTTCGGTAGCCATTCCACCCCT

****
Consensus:

CTCTTTTCGGTAGCCATTCCACCCCTTTGACTCCCTTCCTCACNCCTCCTCA

>Bacillus_Fam_1135_52_1 Nr. of seq. 1 Alignment length(with gaps) = 52 Alignment score = 0.000000
GCF_000787375.1_ASM78737v1_genomic.fna_3:17378-17794 Satlength=417 Nr of Repeats=7 RepeatLength=52 seed=TTCGCTTCTC Num.seqs=6 Similarity=0.875472
0   TTCGCTTCTCTTCGGGAGTCAACACCCTCCTTTAGCTACCGTTCTCTTTGTC

****
Consensus:

TTCGCTTCTCTTCGGGAGTCAACACCCTCCTTTAGCTACCGTTCTCTTTGTC

>Bacillus_Fam_1136_52_1 Nr. of seq. 1 Alignment length(with gaps) = 52 Alignment score = 0.000000
GCF_000787375.1_ASM78737v1_genomic.fna_4:209708-209916 Satlength=209 Nr of Repeats=4 RepeatLength=52 seed=ACATTCGGGC Num.seqs=4 Similarity=0.846154
0   ACATTCGGGCTCCAGATCCCTCTTCTGCTTCCCTACTTTTCCCATTCCCACC

****
Consensus:

ACATTCGGGCTCCAGATCCCTCTTCTGCTTCCCTACTTTTCCCATTCCCACC

>Bacillus_Fam_1137_52_1 Nr. of seq. 1 Alignment length(with gaps) = 52
Alignment score = 0.000000
GCF_000787375.1_ASM78737v1_genomic.fna_16:30256-30516 Satlength=261 Nr of Repeats=5 RepeatLength=52 seed=ACGGGAGCCA Num.seqs=5 Similarity=0.912821
0   ACGGGAGCCATTCCCTTCCTTTGACTACCCAAATCCNGTTCGGTCCTTCTGC

****
Consensus:

ACGGGAGCCATTCCCTTCCTTTGACTACCCAAATCCNGTTCGGTCCTTCTGC

>Bacillus_Fam_1138_52_1 Nr. of seq. 1 Alignment length(with gaps) = 52
Alignment score = 0.000000
GCF_000787375.1_ASM78737v1_genomic.fna_22:64949-65313 Satlength=365 Nr of Repeats=7 RepeatLength=52 seed=TCGTTGACTA Num.seqs=7 Similarity=0.693710
0   TCGTTGACTACCCAAATTTGTGATNGACTCAGAGTTCGAGTGCCANTGNGCT

****
Consensus:

TCGTTGACTACCCAAATTTGTGATNGACTCAGAGTTCGAGTGCCANTGNGCT

>Bacillus_Fam_1139_52_1 Nr. of seq. 1 Alignment length(with gaps) = 52
Alignment score = 0.000000
GCF_000787375.1_ASM78737v1_genomic.fna_40:17325-17638 Satlength=314 Nr of Repeats=6 RepeatLength=52 seed=AGTCAATACA Num.seqs=5 Similarity=0.841026
0   AGTCAATACACCTTATTGGCTACCGTCCACTCCTCCTCCCTCGCTTTTCGGG

****
Consensus:

AGTCAATACACCTTATTGGCTACCGTCCACTCCTCCTCCCTCGCTTTTCGGG

>Bacillus_Fam_1140_52_1 Nr. of seq. 1 Alignment length(with gaps) = 52
Alignment score = 0.000000
GCF_000787375.1_ASM78737v1_genomic.fna_56:13660-13868 Satlength=209 Nr of Repeats=4 RepeatLength=52 seed=TTTCGGTAGT Num.seqs=4 Similarity=0.839099
0   TTTCGGTAGTCAACCCTTCTCTTTGTCTACCAAAATCCTCGCCCTCTCCCTT

****
Consensus:

TTTCGGTAGTCAACCCTTCTCTTTGTCTACCAAAATCCTCGCCCTCTCCCTT

>Bacillus_Fam_1141_52_1 Nr. of seq. 1 Alignment length(with gaps) = 52
Alignment score = 0.000000
GCF_000813125.1_ASM81312v1_genomic.fna_8:33426-33634 Satlength=209 Nr of Repeats=4 RepeatLength=52 seed=TAGACATTTT Num.seqs=4 Similarity=0.880342
0   TAGACATTTTGTAGTCAATTGGCATTCGAAATGTACATTAAACAAGGTTTTT

****

Consensus:

TAGACATTTTGTAGTCAATTGGCATTCGAAATGTACATTAAACAAGGTTTTT

>Bacillus_Fam_1142_52_1 Nr. of seq. 1 Alignment length(with gaps) = 52
Alignment score = 0.000000
GCF_000813125.1_ASM81312v1_genomic.fna_55:28017-28329 Satlength=313 Nr of Repeats=6 RepeatLength=52 seed=TCATTTGGGT Num.seqs=6 Similarity=0.830769
0 TCATTTGGGTAAGAGGAAAGCCCCTGCATTGCCCGAAAAAGCGCCAACTCCA

****
Consensus:

TCATTTGGGTAAGAGGAAAGCCCCTGCATTGCCCGAAAAAGCGCCAACTCCA

>Bacillus_Fam_1143_52_1 Nr. of seq. 1 Alignment length(with gaps) = 52
Alignment score = 0.000000
GCF_000813125.1_ASM81312v1_genomic.fna_62:7988-8195 Satlength=208 Nr of Repeats=4 RepeatLength=52 seed=GATGACGGAC Num.seqs=3 Similarity=0.758910
51 ATGACGGACACTTTCCCAGCGTTTTGGGCTATTTCTGTCCGTCATAGGCTGG

****
Consensus:

GATGACGGACACTTTCCCAGCGTTTTGGGCTATTTCTGTCCGTCATAGGCTG

>Bacillus_Fam_1144_52_1 Nr. of seq. 1 Alignment length(with gaps) = 52
Alignment score = 0.000000
GCF_000813125.1_ASM81312v1_genomic.fna_65:29-237 Satlength=209 Nr of Repeats=4 RepeatLength=52 seed=AAAAAGGGTA Num.seqs=4 Similarity=0.905983
0 AAAAAGGGTAAAAGGAAAGGCTCTGCATTACCCGAAAATGCTGGAGAGAAGT

****
Consensus:

AAAAAGGGTAAAAGGAAAGGCTCTGCATTACCCGAAAATGCTGGAGAGAAGT

>Bacillus_Fam_1145_52_1 Nr. of seq. 1 Alignment length(with gaps) = 52
Alignment score = 0.000000
GCF_000813125.1_ASM81312v1_genomic.fna_67:47637-47845 Satlength=209 Nr of Repeats=4 RepeatLength=52 seed=TGTCCATTAA Num.seqs=4 Similarity=0.910256
0 TGTCCATTAAATGCACTTTCTTAGACATTTCGGTTTGGAACTCATGCTGAAA

****
Consensus:

TGTCCATTAAATGCACTTTCTTAGACATTTCGGTTTGGAACTCATGCTGAAA

>Bacillus_Fam_1146_52_1 Nr. of seq. 1 Alignment length(with gaps) = 52
Alignment score = 0.000000
GCF_000813125.1_ASM81312v1_genomic.fna_88:82704-82913 Satlength=210 Nr of Repeats=4 RepeatLength=52 seed=TTTTGTCCGA Num.seqs=3 Similarity=0.897436
0 TTTTGTCCGAACCTGCCCCTACTTCAGACAATTTCCGCTTGTACGGCCGAGA

****
Consensus:

TTTTGTCCGAACCTGCCCCTACTTCAGACAATTTCCGCTTGTACGGCCGAGA

>Bacillus_Fam_1147_52_1 Nr. of seq. 1 Alignment length(with gaps) = 52
Alignment score = 0.000000
GCF_000813125.1_ASM81312v1_genomic.fna_92:44793-45053 Satlength=261 Nr of Repeats=5 RepeatLength=52 seed=TTAATAGACA Num.seqs=5 Similarity=0.846541
0   TTAATAGACATTTTGACGTCCGTTACTCTTCGAAATGTCTAAGAAGTGCTGT

****
Consensus:

TTAATAGACATTTTGACGTCCGTTACTCTTCGAAATGTCTAAGAAGTGCTGT

>Bacillus_Fam_1148_52_1 Nr. of seq. 1 Alignment length(with gaps) = 52
Alignment score = 0.000000
GCF_000813125.1_ASM81312v1_genomic.fna_102:37405-37665 Satlength=261 Nr of Repeats=4 RepeatLength=52 seed=AAACAGGTCA Num.seqs=3 Similarity=0.551440
0   AAACAGGTCACCAATACCCNTGATTGGTGACCTAAAAATCCATATTTCCGNA

****
Consensus:

AAACAGGTCACCAATACCCNTGATTGGTGACCTAAAAATCCATATTTCCGNA

>Bacillus_Fam_1149_52_1 Nr. of seq. 1 Alignment length(with gaps) = 52
Alignment score = 0.000000
GCF_000813125.1_ASM81312v1_genomic.fna_103:5511-5928 Satlength=418 Nr of Repeats=7 RepeatLength=52 seed=GTTCGGACAT Num.seqs=6 Similarity=0.838155
0   GTTCGGACATTTCAACGGCCTCTTCATTGAGTTTTGTCCGAAGTAGGATCAA

****
Consensus:

GTTCGGACATTTCAACGGCCTCTTCATTGAGTTTTGTCCGAAGTAGGATCAA

>Bacillus_Fam_1150_52_1 Nr. of seq. 1 Alignment length(with gaps) = 52
Alignment score = 0.000000
GCF_000813125.1_ASM81312v1_genomic.fna_105:68233-68494 Satlength=262 Nr of Repeats=5 RepeatLength=52 seed=GAAAAGCTGT Num.seqs=4 Similarity=0.580939
0   GAAAAGCTGTCAAGATTATCCCGGAATTATGACAGGCTCAAGCCCATAAAGC

****
Consensus:

GAAAAGCTGTCAAGATTATCCCGGAATTATGACAGGCTCAAGCCCATAAAGC

>Bacillus_Fam_1151_52_1 Nr. of seq. 1 Alignment length(with gaps) = 52
Alignment score = 0.000000

GCF_000813125.1_ASM81312v1_genomic.fna_121:45604-46123 Satlength=520 Nr of Repeats=10 RepeatLength=52 seed=GTTCGGACAA Num.seqs=9 Similarity=0.829060
0 GTTCGGACAAATCATCGGGAGTGACGGTACTTTTTGTCCGAAGGTGACCCAT

****
Consensus:

GTTCGGACAAATCATCGGGAGTGACGGTACTTTTTGTCCGAAGGTGACCCAT

>Bacillus_Fam_1152_52_1 Nr. of seq. 1 Alignment length(with gaps) = 52 Alignment score = 0.000000
GCF_000813125.1_ASM81312v1_genomic.fna_129:124410-124826 Satlength=417 Nr of Repeats=9 RepeatLength=52 seed=TCCCTCATCC Num.seqs=7 Similarity=0.846154 0
TCCCTCATCCGGGCACATATTTTCCCTTTATGTGCCCCGTCTCCCTTCATCT

****
Consensus:

TCCCTCATCCGGGCACATATTTTCCCTTTATGTGCCCCGTCTCCCTTCATCT

>Bacillus_Fam_1153_52_1 Nr. of seq. 1 Alignment length(with gaps) = 52 Alignment score = 0.000000
GCF_000832905.1_ASM83290v1_genomic.fna_1:2082727-2083091 Satlength=365 Nr of Repeats=7 RepeatLength=52 seed=TATGAAGGAC Num.seqs=7 Similarity=0.817011 0
TATGAAGGACATTTTGGGGGCGNGGGATTCTCCTTTTGTCTTTCATCCCTGT

****
Consensus:

TATGAAGGACATTTTGGGGGCGNGGGATTCTCCTTTTGTCTTTCATCCCTGT

>Bacillus_Fam_1154_52_1 Nr. of seq. 1 Alignment length(with gaps) = 52 Alignment score = 0.000000
GCF_000934845.1_ASM93484v1_genomic.fna_1:28106-28314 Satlength=209 Nr of Repeats=4 RepeatLength=52 seed=TTATTTTGAC Num.seqs=4 Similarity=0.881027
7 AGAGAGGTTATTTTGACAACTTTCAGGTGAAAAGGGCAAAAGTTGTCAAGAA

****
Consensus:

TTATTTTGACAACTTTCAGGTGAAAAGGGCAAAAGTTGTCAAGAAAGAGAGG

>Bacillus_Fam_1155_52_1 Nr. of seq. 1 Alignment length(with gaps) = 52 Alignment score = 0.000000
GCF_000986785.1_ASM98678v1_genomic.fna_6:11778-12141 Satlength=364 Nr of Repeats=7 RepeatLength=52 seed=TGATAGGCGC Num.seqs=6 Similarity=0.801467
0 TGATAGGCGCCCGTGGCGGTTGAAAATAGGAGTTGGCGGTCGCCAATAGGGT

****
Consensus:

TGATAGGCGCCCGTGGCGGTTGAAAATAGGAGTTGGCGGTCGCCAATAGGGT

>Bacillus_Fam_1156_52_1 Nr. of seq. 1 Alignment length(with gaps) = 52
Alignment score = 0.000000
GCF_000986785.1_ASM98678v1_genomic.fna_15:1-313 Satlength=313 Nr of Repeats=6 RepeatLength=52 seed=GGGCACTGAG Num.seqs=6 Similarity=0.801258
0   GGGCACTGAGAACACCTGTCAGCACCCAAAATCCCTGGCCCCTCCTAAAAAT

****
Consensus:

GGGCACTGAGAACACCTGTCAGCACCCAAAATCCCTGGCCCCTCCTAAAAAT

>Bacillus_Fam_1157_52_1 Nr. of seq. 1 Alignment length(with gaps) = 52
Alignment score = 0.000000
GCF_000986785.1_ASM98678v1_genomic.fna_19:60234-60494 Satlength=261 Nr of Repeats=5 RepeatLength=52 seed=TGTCAGTGCC Num.seqs=5 Similarity=0.675472
0   TGTCAGTGCCCCAAAAGTGGAGCAGNTCGTNAAAAAGGTTACTGAGGGTCGN

****
Consensus:

TGTCAGTGCCCCAAAAGTGGAGCAGNTCGTNAAAAAGGTTACTGAGGGTCGN

>Bacillus_Fam_1158_52_1 Nr. of seq. 1 Alignment length(with gaps) = 52
Alignment score = 0.000000
GCF_000986785.1_ASM98678v1_genomic.fna_31:25184-25444 Satlength=261 Nr of Repeats=5 RepeatLength=52 seed=CCTCTTTTTC Num.seqs=5 Similarity=0.887179
0   CCTCTTTTTCGGTAAACTCTCGGAAAAAGGTACCTGACAGGCTGCCTCAGTA

****
Consensus:

CCTCTTTTTCGGTAAACTCTCGGAAAAAGGTACCTGACAGGCTGCCTCAGTA

>Bacillus_Fam_1159_52_1 Nr. of seq. 1 Alignment length(with gaps) = 52
Alignment score = 0.000000
GCF_000986785.1_ASM98678v1_genomic.fna_38:27020-27332 Satlength=313 Nr of Repeats=6 RepeatLength=52 seed=TTGGTCGTCC Num.seqs=6 Similarity=0.841026
0   TTGGTCGTCCATACACCCGATGGAGGCCCGTTTTTCAGAATCAGCTTCAGTA

****
Consensus:

TTGGTCGTCCATACACCCGATGGAGGCCCGTTTTTCAGAATCAGCTTCAGTA

>Bacillus_Fam_1160_52_1 Nr. of seq. 1 Alignment length(with gaps) = 52
Alignment score = 0.000000
GCF_000986785.1_ASM98678v1_genomic.fna_100:49503-49815 Satlength=313 Nr of Repeats=6 RepeatLength=52 seed=GGGCTTCCAT Num.seqs=6 Similarity=0.876923
0   GGGCTTCCATCCACTTGATGGAGGACCTTTTTTGCGGCTTTCCTGCGAAAAC

****
Consensus:

GGGCTTCCATCCACTTGATGGAGGACCTTTTTTGCGGCTTTCCTGCGAAAAC

>Bacillus_Fam_1161_52_1 Nr. of seq. 1 Alignment length(with gaps) = 52
Alignment score = 0.000000
GCF_000986785.1_ASM98678v1_genomic.fna_101:18847-19056 Satlength=210 Nr of Repeats=4 RepeatLength=52 seed=TTTTTTGGTG Num.seqs=3
Similarity=0.700855
10   NTCAGAGCCCTTTTTTGGTGGTTTGCATGAATTTTGGGTACTGAGGAGGAGT

****
Consensus:

TTTTTTGGTGGTTTGCATGAATTTTGGGTACTGAGGAGGAGTNTCAGAGCCC

>Bacillus_Fam_1162_52_1 Nr. of seq. 1 Alignment length(with gaps) = 52
Alignment score = 0.000000
GCF_001278705.1_ASM127870v1_genomic.fna_1:2331510-2331718 Satlength=209 Nr of Repeats=4 RepeatLength=52 seed=GTCCTTCATC Num.seqs=4
Similarity=0.854701 0
GTCCTTCATCCAGGGTCTGAAGGACATAACAACACTAGTGATCACGTGAATT

****
Consensus:

GTCCTTCATCCAGGGTCTGAAGGACATAACAACACTAGTGATCACGTGAATT

>Bacillus_Fam_1163_52_1 Nr. of seq. 1 Alignment length(with gaps) = 52
Alignment score = 0.000000
GCF_001315045.1_ASM131504v1_genomic.fna_43:27341-27549 Satlength=209 Nr of Repeats=4 RepeatLength=52 seed=TAGTCCGAAG Num.seqs=4
Similarity=0.846154
0   TAGTCCGAAGGCAGGTCAAGTTCGGACAGAAAGCAGACATCAAAGCGCAAAC

****
Consensus:

TAGTCCGAAGGCAGGTCAAGTTCGGACAGAAAGCAGACATCAAAGCGCAAAC

>Bacillus_Fam_1164_52_1 Nr. of seq. 1 Alignment length(with gaps) = 52
Alignment score = 0.000000
GCF_001315165.1_ASM131516v1_genomic.fna_82:7303-7614 Satlength=312 Nr of Repeats=6 RepeatLength=52 seed=TTCGGCCAAA Num.seqs=5 Similarity=0.787179
0   TTCGGCCAAATCAGGAGCGNCGAGCCGTTGAAATGTCCGAAGTTGGTGTAGG

****
Consensus:

TTCGGCCAAATCAGGAGCGNCGAGCCGTTGAAATGTCCGAAGTTGGTGTAGG

>Bacillus_Fam_1165_52_1 Nr. of seq. 1 Alignment length(with gaps) = 52
Alignment score = 0.000000
GCF_001375535.1_Bacillus_niameyensis_genomic.fna_6:2141488-2141852
Satlength=365 Nr of Repeats=7 RepeatLength=52 seed=CATTTTTCCA Num.seqs=7
Similarity=0.792430 0
CATTTTTCCAGGGCTATGGTCTATGACAGACGTTATCATGACCCGTTGGCTG

****
Consensus:

CATTTTTCCAGGGCTATGGTCTATGACAGACGTTATCATGACCCGTTGGCTG

>Bacillus_Fam_1166_52_1 Nr. of seq. 1 Alignment length(with gaps) = 52
Alignment score = 0.000000
GCF_001375535.1_Bacillus_niameyensis_genomic.fna_6:2366660-2366868
Satlength=209 Nr of Repeats=4 RepeatLength=52 seed=CTCTCATGGA Num.seqs=4
Similarity=0.927350 0
CTCTCATGGACCTTTCGCATGTAAAAGTCGCTGCAATGGGACACGACAACGA

****
Consensus:

CTCTCATGGACCTTTCGCATGTAAAAGTCGCTGCAATGGGACACGACAACGA

>Bacillus_Fam_1167_52_1 Nr. of seq. 1 Alignment length(with gaps) = 52
Alignment score = 0.000000
GCF_001375535.1_Bacillus_niameyensis_genomic.fna_6:2468093-2468561
Satlength=469 Nr of Repeats=9 RepeatLength=52 seed=GAATCGTTAC Num.seqs=9
Similarity=0.769204 0
GAATCGTTACCGTCATGCGANGCTTTCAAGNCTAACGGGCACGATTCACTTC

****
Consensus:

GAATCGTTACCGTCATGCGANGCTTTCAAGNCTAACGGGCACGATTCACTTC

>Bacillus_Fam_1168_52_1 Nr. of seq. 1 Alignment length(with gaps) = 52
Alignment score = 0.000000
GCF_001375535.1_Bacillus_niameyensis_genomic.fna_6:2662491-2662803
Satlength=313 Nr of Repeats=6 RepeatLength=52 seed=CCTCTCATGT Num.seqs=6
Similarity=0.854701 0
CCTCTCATGTCCCGTATCATTGAAAAAAGGATGTGCTTGGGACATGAGACCA

****
Consensus:

CCTCTCATGTCCCGTATCATTGAAAAAAGGATGTGCTTGGGACATGAGACCA

>Bacillus_Fam_1169_52_1 Nr. of seq. 1 Alignment length(with gaps) = 52
Alignment score = 0.000000
GCF_001375535.1_Bacillus_niameyensis_genomic.fna_6:3404396-3408244
Satlength=3849 Nr of Repeats=74 RepeatLength=52 seed=CTATTCGCCC
Num.seqs=74 Similarity=0.942760 0
CTATTCGCCCGAGGAGGAAGAAAAGTGTATCTAGGGGTAGAATAGGACGTCT

****
Consensus:

CTATTCGCCCGAGGAGGAAGAAAAGTGTATCTAGGGGTAGAATAGGACGTCT

>Bacillus_Fam_1170_52_1 Nr. of seq. 1 Alignment length(with gaps) = 52
Alignment score = 0.000000
GCF_001375535.1_Bacillus_niameyensis_genomic.fna_6:3437377-3438313
Satlength=937 Nr of Repeats=18 RepeatLength=52 seed=GGTCGAATAG
Num.seqs=16 Similarity=0.812631 0
GGTCGAATAGGGCGTCTCTATTCGCCCGAAGCGCCAGGTCGAGTCGTTCAAA

****
Consensus:

GGTCGAATAGGGCGTCTCTATTCGCCCGAAGCGCCAGGTCGAGTCGTTCAAA

>Bacillus_Fam_1171_52_1 Nr. of seq. 1 Alignment length(with gaps) = 52
Alignment score = 0.000000
GCF_001375535.1_Bacillus_niameyensis_genomic.fna_6:3623417-3629501
Satlength=6085 Nr of Repeats=99 RepeatLength=52 seed=CCTTGAATGG
Num.seqs=81 Similarity=0.818276 0
CCTTGAATGGGACGTGATGAGTGTTATCAAAGCCCGATCGCCANCTTTTTTN

****
Consensus:

CCTTGAATGGGACGTGATGAGTGTTATCAAAGCCCGATCGCCANCTTTTTTN

>Bacillus_Fam_1172_52_1 Nr. of seq. 1 Alignment length(with gaps) = 52
Alignment score = 0.000000
GCF_001375535.1_Bacillus_niameyensis_genomic.fna_6:4106934-4107141
Satlength=208 Nr of Repeats=4 RepeatLength=52 seed=ACGGTAACGA Num.seqs=3
Similarity=0.863248 0
ACGGTAACGATTGAGGCCGAATCGTTCCCGCACCGTGCTATTTTTGGGAGTT

****
Consensus:

ACGGTAACGATTGAGGCCGAATCGTTCCCGCACCGTGCTATTTTTGGGAGTT

>Bacillus_Fam_1173_52_1 Nr. of seq. 1 Alignment length(with gaps) = 52
Alignment score = 0.000000
GCF_001420595.1_ASM142059v1_genomic.fna_11:959883-960403 Satlength=521 Nr
of Repeats=10 RepeatLength=52 seed=CGATTGAGTT Num.seqs=10
Similarity=0.834032 0
CGATTGAGTTCAGTTTATCCAAAAATTACCCGCTAACGGGAACCAATGAGCT

****
Consensus:

CGATTGAGTTCAGTTTATCCAAAAATTACCCGCTAACGGGAACCAATGAGCT

>Bacillus_Fam_1174_52_1 Nr. of seq. 1 Alignment length(with gaps) = 52
Alignment score = 0.000000
GCF_001420595.1_ASM142059v1_genomic.fna_11:1190597-1190910 Satlength=314
Nr of Repeats=6 RepeatLength=52 seed=TTCGGACAAG Num.seqs=5
Similarity=0.779487 0
TTCGGACAAGCCCAGGTGATTTTGAGCAATCNTAGTCCGAATAGCCGTCGAG

****
Consensus:

TTCGGACAAGCCCAGGTGATTTTGAGCAATCNTAGTCCGAATAGCCGTCGAG

>Bacillus_Fam_1175_52_1 Nr. of seq. 1 Alignment length(with gaps) = 52
Alignment score = 0.000000
GCF_001420595.1_ASM142059v1_genomic.fna_11:1214278-1214746 Satlength=469
Nr of Repeats=9 RepeatLength=52 seed=GTCGCCAATA Num.seqs=9
Similarity=0.757512 0
GTCGCCAATAAGGTGTATTGGTACCCGAAACTCTACTATATTTAATAAAACG

****
Consensus:

GTCGCCAATAAGGTGTATTGGTACCCGAAACTCTACTATATTTAATAAAACG

>Bacillus_Fam_1176_52_1 Nr. of seq. 1 Alignment length(with gaps) = 52
Alignment score = 0.000000
GCF_001420595.1_ASM142059v1_genomic.fna_11:1222858-1223222 Satlength=365
Nr of Repeats=7 RepeatLength=52 seed=GACAACCACA Num.seqs=7
Similarity=0.880342 0
GACAACCACACTCAAAATTCGGCTGAAGAAGTCCGAAGTGGTAGTGGGTTCA

****
Consensus:

GACAACCACACTCAAAATTCGGCTGAAGAAGTCCGAAGTGGTAGTGGGTTCA

>Bacillus_Fam_1177_52_1 Nr. of seq. 1 Alignment length(with gaps) = 52
Alignment score = 0.000000
GCF_001420595.1_ASM142059v1_genomic.fna_11:1804770-1804979 Satlength=210
Nr of Repeats=4 RepeatLength=52 seed=AGGGAACCAA Num.seqs=3
Similarity=0.842767 0
AGGGAACCAATGACTCCTATTGATACCTCAAACAACGCTAATTTGCCAAATA

****
Consensus:

AGGGAACCAATGACTCCTATTGATACCTCAAACAACGCTAATTTGCCAAATA

>Bacillus_Fam_1178_52_1 Nr. of seq. 1 Alignment length(with gaps) = 52
Alignment score = 0.000000
GCF_001420595.1_ASM142059v1_genomic.fna_11:2856360-2856620 Satlength=261
Nr of Repeats=5 RepeatLength=52 seed=GGAAACAAGC Num.seqs=5
Similarity=0.835897 0
GGAAACAAGCAGCCCTATTGAGTACACTATTCACTCGAATTCTTTAAAAATG

****
Consensus:

GGAAACAAGCAGCCCTATTGAGTACACTATTCACTCGAATTCTTTAAAAATG

>Bacillus_Fam_1179_52_1 Nr. of seq. 1 Alignment length(with gaps) = 52
Alignment score = 0.000000
GCF_001420595.1_ASM142059v1_genomic.fna_11:3118694-3118954 Satlength=261
Nr of Repeats=5 RepeatLength=52 seed=TGTCTGAACT Num.seqs=5
Similarity=0.787179 0
TGTCTGAACTTGACTGGTGTTCGGACTCGATTCACCTTAAAATCGCCATTGC

****
Consensus:

TGTCTGAACTTGACTGGTGTTCGGACTCGATTCACCTTAAAATCGCCATTGC

>Bacillus_Fam_1180_52_1 Nr. of seq. 1 Alignment length(with gaps) = 52
Alignment score = 0.000000
GCF_001420595.1_ASM142059v1_genomic.fna_11:3296695-3296903 Satlength=209
Nr of Repeats=4 RepeatLength=52 seed=CTTTTTTCTC Num.seqs=4
Similarity=0.816239 0
CTTTTTTCTCCAGTGTTCGGTCAACAATAACGGCGATTGGATACCCTTCTGT

****
Consensus:

CTTTTTTCTCCAGTGTTCGGTCAACAATAACGGCGATTGGATACCCTTCTGT

>Bacillus_Fam_1181_52_1 Nr. of seq. 1 Alignment length(with gaps) = 52
Alignment score = 0.000000
GCF_001420595.1_ASM142059v1_genomic.fna_11:3743605-3744124 Satlength=520
Nr of Repeats=10 RepeatLength=52 seed=TACTTCAGAC Num.seqs=9
Similarity=0.759971 0
TACTTCAGACTTCTTTTNCGCAATCAAGGGCTAACCTGTCCGAACCTGAGGC

****
Consensus:

TACTTCAGACTTCTTTTNCGCAATCAAGGGCTAACCTGTCCGAACCTGAGGC

>Bacillus_Fam_1182_52_1 Nr. of seq. 1 Alignment length(with gaps) = 52
Alignment score = 0.000000
GCF_001420595.1_ASM142059v1_genomic.fna_11:3931578-3931889 Satlength=312
Nr of Repeats=6 RepeatLength=52 seed=GGCTATTGAT Num.seqs=5
Similarity=0.892308 0
GGCTATTGATAACCTTTTTTGCTTTTTTTACAGGAAAACTGAACTCAATAGA

****
Consensus:

GGCTATTGATAACCTTTTTTGCTTTTTTTACAGGAAAACTGAACTCAATAGA

>Bacillus_Fam_1183_52_1 Nr. of seq. 1 Alignment length(with gaps) = 52
Alignment score = 0.000000
GCF_001420595.1_ASM142059v1_genomic.fna_11:4472676-4473040 Satlength=365
Nr of Repeats=7 RepeatLength=52 seed=TCTTGTTCAG Num.seqs=7
Similarity=0.801737 0
TCTTGTTCAGACTCGATCTCTCTTATTTTCACCAAACTTGTCCGAACTTGAC

****
Consensus:

TCTTGTTCAGACTCGATCTCTCTTATTTTCACCAAACTTGTCCGAACTTGAC

>Bacillus_Fam_1184_52_1 Nr. of seq. 1 Alignment length(with gaps) = 52
Alignment score = 0.000000
GCF_001420595.1_ASM142059v1_genomic.fna_11:4647606-4648228 Satlength=623
Nr of Repeats=12 RepeatLength=52 seed=CCTAACCTGT Num.seqs=10
Similarity=0.855556 0
CCTAACCTGTCCGAACTCATCCCAAGTTCGGACTTCTTTGCTCTTGAATCCT

****
Consensus:

CCTAACCTGTCCGAACTCATCCCAAGTTCGGACTTCTTTGCTCTTGAATCCT

>Bacillus_Fam_1185_52_1 Nr. of seq. 1 Alignment length(with gaps) = 52
Alignment score = 0.000000
GCF_001420595.1_ASM142059v1_genomic.fna_12:256320-256580 Satlength=261 Nr
of Repeats=5 RepeatLength=52 seed=AGAAAAGCGA Num.seqs=5
Similarity=0.861538 0
AGAAAAGCGAACTCAACAAGCGTTATTGGTTCCCGAAAACTAGCAGAAAGGC

****
Consensus:

AGAAAAGCGAACTCAACAAGCGTTATTGGTTCCCGAAAACTAGCAGAAAGGC

>Bacillus_Fam_1186_52_1 Nr. of seq. 1 Alignment length(with gaps) = 52
Alignment score = 0.000000
GCF_001420715.1_ASM142071v1_genomic.fna_22:194482-194899 Satlength=418 Nr
of Repeats=8 RepeatLength=52 seed=AGATGTCCTT Num.seqs=7
Similarity=0.699001 0
AGATGTCCTTCATCAATGAAATGAAGGCCAAGNTATAGGGTTTTCTACTGTC

****
Consensus:

AGATGTCCTTCATCAATGAAATGAAGGCCAAGNTATAGGGTTTTCTACTGTC

>Bacillus_Fam_1187_52_1 Nr. of seq. 1 Alignment length(with gaps) = 52
Alignment score = 0.000000
GCF_001420715.1_ASM142071v1_genomic.fna_22:204926-205656 Satlength=731 Nr
of Repeats=14 RepeatLength=52 seed=TGAATTGTTC Num.seqs=13
Similarity=0.747702 0
TGAATTGTTCCCGTTCATCAACACCATCCACGATTGCGGTCACGATACNTCG

****
Consensus:

TGAATTGTTCCCGTTCATCAACACCATCCACGATTGCGGTCACGATACNTCG

>Bacillus_Fam_1188_52_1 Nr. of seq. 1 Alignment length(with gaps) = 52
Alignment score = 0.000000
GCF_001420715.1_ASM142071v1_genomic.fna_22:348610-349234 Satlength=625 Nr of Repeats=12 RepeatLength=52 seed=AGAGGAAGTA Num.seqs=12
Similarity=0.812655 0
AGAGGAAGTATTGTGCCCGGAAAGGGAAAGCTCGAGAAGAAACGGGAACAAA

****
Consensus:

AGAGGAAGTATTGTGCCCGGAAAGGGAAAGCTCGAGAAGAAACGGGAACAAA

>Bacillus_Fam_1189_52_1 Nr. of seq. 1 Alignment length(with gaps) = 52
Alignment score = 0.000000
GCF_001420715.1_ASM142071v1_genomic.fna_22:408865-409073 Satlength=209 Nr of Repeats=4 RepeatLength=52 seed=GTATTGTTCC Num.seqs=4
Similarity=0.734277 0
GTATTGTTCCCATAATCCTCTGGGAAAGACAGAAACGGTAACGATTAGTTGA

****
Consensus:

GTATTGTTCCCATAATCCTCTGGGAAAGACAGAAACGGTAACGATTAGTTGA

>Bacillus_Fam_1190_52_1 Nr. of seq. 1 Alignment length(with gaps) = 52
Alignment score = 0.000000
GCF_001420715.1_ASM142071v1_genomic.fna_22:580070-580538 Satlength=469 Nr of Repeats=9 RepeatLength=52 seed=GGGAACAATA Num.seqs=9
Similarity=0.837607 0
GGGAACAATAGAAGTAGAATCGTGCCCGAAATCAGGAGAGAGCAGTGNCCCC

****
Consensus:

GGGAACAATAGAAGTAGAATCGTGCCCGAAATCAGGAGAGAGCAGTGNCCCC

>Bacillus_Fam_1191_52_1 Nr. of seq. 1 Alignment length(with gaps) = 52
Alignment score = 0.000000
GCF_001420715.1_ASM142071v1_genomic.fna_22:603974-604181 Satlength=208 Nr of Repeats=4 RepeatLength=52 seed=ACAACGGTAA Num.seqs=3
Similarity=0.760684 0
ACAACGGTAACGATCAGAGGTGGAATGTGCCCGTAGCGGAGGAAATCTCGGG

****
Consensus:

ACAACGGTAACGATCAGAGGTGGAATGTGCCCGTAGCGGAGGAAATCTCGGG

>Bacillus_Fam_1192_52_1 Nr. of seq. 1 Alignment length(with gaps) = 52
Alignment score = 0.000000
GCF_001420715.1_ASM142071v1_genomic.fna_22:614742-615418 Satlength=677 Nr of Repeats=13 RepeatLength=52 seed=TGTCCTTCAT Num.seqs=9
Similarity=0.781971 0
TGTCCTTCATGAAGGTAATGAAGTCCAAACTAGGTGTACCAAAATCGAAAAG

****
Consensus:

TGTCCTTCATGAAGGTAATGAAGTCCAAACTAGGTGTACCAAAATCGAAAAG

>Bacillus_Fam_1193_52_1 Nr. of seq. 1 Alignment length(with gaps) = 52
Alignment score = 0.000000
GCF_001420715.1_ASM142071v1_genomic.fna_22:972888-973200 Satlength=313 Nr of Repeats=6 RepeatLength=52 seed=AGCGATGAAG Num.seqs=6
Similarity=0.835897 0
AGCGATGAAGGCCAAAATGTGCTCGGTAACAAGAGCAGATGACCTTCATAAA

****
Consensus:

AGCGATGAAGGCCAAAATGTGCTCGGTAACAAGAGCAGATGACCTTCATAAA

>Bacillus_Fam_1194_52_1 Nr. of seq. 1 Alignment length(with gaps) = 52
Alignment score = 0.000000
GCF_001420715.1_ASM142071v1_genomic.fna_22:2501988-2502560 Satlength=573 Nr of Repeats=11 RepeatLength=52 seed=ATCGTGCCCA Num.seqs=11
Similarity=0.738721 0
ATCGTGCCCAATACCGAGGCNGACATGGAATAACGGGAACAATACCAGGAGA

****
Consensus:

ATCGTGCCCAATACCGAGGCNGACATGGAATAACGGGAACAATACCAGGAGA

>Bacillus_Fam_1195_52_1 Nr. of seq. 1 Alignment length(with gaps) = 52
Alignment score = 0.000000
GCF_001420715.1_ASM142071v1_genomic.fna_22:3462522-3462781 Satlength=260 Nr of Repeats=5 RepeatLength=52 seed=TGAAGGACAC Num.seqs=4
Similarity=0.777778 23
TATCTTGTCCCTCATAACCCCGATGAAGGACACTTCCCTTGTTTTCTATCAC

****
Consensus:

TGAAGGACACTTCCCTTGTTTTCTATCACTATCTTGTCCCTCATAACCCCGA

>Bacillus_Fam_1196_52_1 Nr. of seq. 1 Alignment length(with gaps) = 52
Alignment score = 0.000000
GCF_001420715.1_ASM142071v1_genomic.fna_24:148824-149188 Satlength=365 Nr of Repeats=7 RepeatLength=52 seed=CTTTGGTCTT Num.seqs=7
Similarity=0.863133 0
CTTTGGTCTTTATAACCCGCTTAAAGGACAATTCCTTTAAAATATTTGATTC

****
Consensus:

CTTTGGTCTTTATAACCCGCTTAAAGGACAATTCCTTTAAAATATTTGATTC

>Bacillus_Fam_1197_52_1 Nr. of seq. 1 Alignment length(with gaps) = 52
Alignment score = 0.000000
GCF_001420715.1_ASM142071v1_genomic.fna_24:150600-151016 Satlength=417 Nr of Repeats=8 RepeatLength=52 seed=TGTCCTTCAT Num.seqs=8
Similarity=0.746855 0
TGTCCTTCATATGCTCCATGAAGGACATGTCCATGGGTATTTTCGGTTGTCT

****
Consensus:

TGTCCTTCATATGCTCCATGAAGGACATGTCCATGGGTATTTTCGGTTGTCT

>Bacillus_Fam_1198_52_1 Nr. of seq. 1 Alignment length(with gaps) = 52
Alignment score = 0.000000
GCF_001439915.1_ASM143991v1_genomic.fna_83:3882-4089 Satlength=208 Nr of Repeats=4 RepeatLength=52 seed=ATTAGAGTAA Num.seqs=3 Similarity=0.811966
0   ATTAGAGTAAAAGGTCTTCATCGATGTTATGAAGACCAAACCGGGTTCAGAG

****
Consensus:

ATTAGAGTAAAAGGTCTTCATCGATGTTATGAAGACCAAACCGGGTTCAGAG

>Bacillus_Fam_1199_52_1 Nr. of seq. 1 Alignment length(with gaps) = 52
Alignment score = 0.000000
GCF_001439915.1_ASM143991v1_genomic.fna_83:129734-130312 Satlength=579 Nr of Repeats=11 RepeatLength=52 seed=TGATTGCGAC Num.seqs=7
Similarity=0.740153 0
TGATTGCGACACGGATTTCGGNAAAATTCAAAGATGAGTCCGAATAAGAGTG

****
Consensus:

TGATTGCGACACGGATTTCGGNAAAATTCAAAGATGAGTCCGAATAAGAGTG

>Bacillus_Fam_1200_52_1 Nr. of seq. 1 Alignment length(with gaps) = 52
Alignment score = 0.000000
GCF_001439915.1_ASM143991v1_genomic.fna_83:602105-602677 Satlength=573 Nr of Repeats=11 RepeatLength=52 seed=CTTGTGTTGT Num.seqs=11
Similarity=0.875058 0
CTTGTGTTGTCCGAATCAAGGCGTCATTCGGACAGCTACGCCAGATTTCCTG

****
Consensus:

CTTGTGTTGTCCGAATCAAGGCGTCATTCGGACAGCTACGCCAGATTTCCTG

>Bacillus_Fam_1201_52_1 Nr. of seq. 1 Alignment length(with gaps) = 52
Alignment score = 0.000000
GCF_001439915.1_ASM143991v1_genomic.fna_83:693863-694070 Satlength=208 Nr of Repeats=4 RepeatLength=52 seed=ATGATTCGGA Num.seqs=3
Similarity=0.708595 0
ATGATTCGGACACAGATCAGGAAAAACCGCACCCTCGAGTCCGAATGAAGCG

****
Consensus:

ATGATTCGGACACAGATCAGGAAAAACCGCACCCTCGAGTCCGAATGAAGCG

>Bacillus_Fam_1202_52_1 Nr. of seq. 1 Alignment length(with gaps) = 52
Alignment score = 0.000000
GCF_001439915.1_ASM143991v1_genomic.fna_84:126495-127119 Satlength=625 Nr of Repeats=12 RepeatLength=52 seed=AGTCCGAATG Num.seqs=12
Similarity=0.750143 0
AGTCCGAATGCAAGGCTGATTCGGACAGTTGGGTCAGGGGAATNCAGAAGCG

****
Consensus:

AGTCCGAATGCAAGGCTGATTCGGACAGTTGGGTCAGGGGAATNCAGAAGCG

>Bacillus_Fam_1203_52_1 Nr. of seq. 1 Alignment length(with gaps) = 52
Alignment score = 0.000000
GCF_001439915.1_ASM143991v1_genomic.fna_87:45812-46177 Satlength=366 Nr of Repeats=7 RepeatLength=52 seed=CTGTCAGAAT Num.seqs=6
Similarity=0.776955
0   CTGTCAGAATCAACCCTTCATTCGGACTCAACTACTCGTATTCCTTACTTCT

****
Consensus:

CTGTCAGAATCAACCCTTCATTCGGACTCAACTACTCGTATTCCTTACTTCT

>Bacillus_Fam_1204_52_1 Nr. of seq. 1 Alignment length(with gaps) = 52
Alignment score = 0.000000
GCF_001439915.1_ASM143991v1_genomic.fna_105:73746-74108 Satlength=363 Nr of Repeats=7 RepeatLength=52 seed=AGGTCTTCAT Num.seqs=6
Similarity=0.714747
0   AGGTCTTCATGAGGTTGATGAAGACCTATTTGCAACTAGTATTCTCTCGGAA

****
Consensus:

AGGTCTTCATGAGGTTGATGAAGACCTATTTGCAACTAGTATTCTCTCGGAA

>Bacillus_Fam_1205_52_1 Nr. of seq. 1 Alignment length(with gaps) = 52
Alignment score = 0.000000
GCF_001439915.1_ASM143991v1_genomic.fna_116:280129-280960 Satlength=832 Nr of Repeats=16 RepeatLength=52 seed=GAATCGTACC Num.seqs=15
Similarity=0.837118 0
GAATCGTACCCGATTCGGGGCAATTGCAAGGAAAAGGGTAACGCAAACGTGG

****
Consensus:

GAATCGTACCCGATTCGGGGCAATTGCAAGGAAAAGGGTAACGCAAACGTGG

>Bacillus_Fam_1206_52_1 Nr. of seq. 1 Alignment length(with gaps) = 52
Alignment score = 0.000000
GCF_001439915.1_ASM143991v1_genomic.fna_122:196858-197329 Satlength=472
Nr of Repeats=9 RepeatLength=52 seed=CTGTCCGAAT Num.seqs=7
Similarity=0.686791 0
CTGTCCGAATGAGGCATGAATTCGGACTCATAAACGTTTTTTGGGACCTACA

****
Consensus:

CTGTCCGAATGAGGCATGAATTCGGACTCATAAACGTTTTTTGGGACCTACA

>Bacillus_Fam_1207_52_1 Nr. of seq. 1 Alignment length(with gaps) = 52
Alignment score = 0.000000
GCF_001439915.1_ASM143991v1_genomic.fna_122:208982-209396 Satlength=415
Nr of Repeats=8 RepeatLength=52 seed=TGGTCTTCAT Num.seqs=6
Similarity=0.796581 0
TGGTCTTCATCACCTCAATGATAACCGTTTCCGCGATTCAGCGAACGAGTTT

****
Consensus:

TGGTCTTCATCACCTCAATGATAACCGTTTCCGCGATTCAGCGAACGAGTTT

>Bacillus_Fam_1208_52_1 Nr. of seq. 1 Alignment length(with gaps) = 52
Alignment score = 0.000000
GCF_001439915.1_ASM143991v1_genomic.fna_122:222503-222814 Satlength=312
Nr of Repeats=6 RepeatLength=52 seed=TGTCCGAATC Num.seqs=5
Similarity=0.902564 0
TGTCCGAATCATAGGCTCATTCGGACTTAAGGCAACAGAATCCTTTGCGACG

****
Consensus:

TGTCCGAATCATAGGCTCATTCGGACTTAAGGCAACAGAATCCTTTGCGACG

>Bacillus_Fam_1209_52_1 Nr. of seq. 1 Alignment length(with gaps) = 52
Alignment score = 0.000000
GCF_001439915.1_ASM143991v1_genomic.fna_123:110275-110849 Satlength=575
Nr of Repeats=11 RepeatLength=52 seed=GAATCGTTCC Num.seqs=9
Similarity=0.688823 0
GAATCGTTCCCGATTGCAAATAAAAATCATAAAAAGAGTAACGCAAGCCCTC

****
Consensus:

GAATCGTTCCCGATTGCAAATAAAAATCATAAAAAGAGTAACGCAAGCCCTC

>Bacillus_Fam_1210_52_1 Nr. of seq. 1 Alignment length(with gaps) = 52
Alignment score = 0.000000
GCF_001439965.1_ASM143996v1_genomic.fna_12:79325-79844 Satlength=520 Nr of Repeats=10 RepeatLength=52 seed=TTACCGGGGA Num.seqs=9 Similarity=0.804131
0 TTACCGGGGACTCTTGATTTCCTCCTTCACGGTAAGCATACTCCCTCTTTGN

****
Consensus:

TTACCGGGGACTCTTGATTTCCTCCTTCACGGTAAGCATACTCCCTCTTTGN

>Bacillus_Fam_1211_52_1 Nr. of seq. 1 Alignment length(with gaps) = 52
Alignment score = 0.000000
GCF_001439965.1_ASM143996v1_genomic.fna_121:100541-100822 Satlength=282 Nr of Repeats=5 RepeatLength=52 seed=ACCGAAAGAG Num.seqs=4 Similarity=0.805556 0
ACCGAAAGAGGCCCCAAGTCCCGCTCCAGGTAACCAAAACCCAGTAAAGGTA

****
Consensus:

ACCGAAAGAGGCCCCAAGTCCCGCTCCAGGTAACCAAAACCCAGTAAAGGTA

>Bacillus_Fam_1212_52_1 Nr. of seq. 1 Alignment length(with gaps) = 52
Alignment score = 0.000000
GCF_001587375.1_ASM158737v1_genomic.fna_2:11105-11469 Satlength=365 Nr of Repeats=7 RepeatLength=52 seed=ATGGTACCGG Num.seqs=7 Similarity=0.814408
0 ATGGTACCGGAAAAGAGAGAAGCTGTACGATGGCAACGCGAATCTCGTAGCC

****
Consensus:

ATGGTACCGGAAAAGAGAGAAGCTGTACGATGGCAACGCGAATCTCGTAGCC

>Bacillus_Fam_1213_52_1 Nr. of seq. 1 Alignment length(with gaps) = 52
Alignment score = 0.000000
GCF_001587375.1_ASM158737v1_genomic.fna_20:46870-47181 Satlength=312 Nr of Repeats=6 RepeatLength=52 seed=TGCTCTATCG Num.seqs=5 Similarity=0.766038
0 TGCTCTATCGGTTCGCTTTTGNCTCTTGCAGTACCATTGGCGTTNGTGCTCC

****
Consensus:

TGCTCTATCGGTTCGCTTTTGNCTCTTGCAGTACCATTGGCGTTNGTGCTCC

>Bacillus_Fam_1214_52_1 Nr. of seq. 1 Alignment length(with gaps) = 52
Alignment score = 0.000000
GCF_001587375.1_ASM158737v1_genomic.fna_20:50326-50534 Satlength=209 Nr of Repeats=4 RepeatLength=52 seed=GATCTTCCCG Num.seqs=4 Similarity=0.846154
0 GATCTTCCCGCATCACCGGGATTCGGGTACTTCATGCAATGCTAGCTGCCGC

****
Consensus:

GATCTTCCCGCATCACCGGGATTCGGGTACTTCATGCAATGCTAGCTGCCGC

>Bacillus_Fam_1215_52_1 Nr. of seq. 1 Alignment length(with gaps) = 52
Alignment score = 0.000000
GCF_001587375.1_ASM158737v1_genomic.fna_52:33919-34179 Satlength=261 Nr of Repeats=5 RepeatLength=52 seed=CAAAAATGTA Num.seqs=5 Similarity=0.782051
0 CAAAAATGTAAAAGGAANGCCGGCTTGTATATAGATTTTCGGTGTAGAAGGT

****
Consensus:

CAAAAATGTAAAAGGAANGCCGGCTTGTATATAGATTTTCGGTGTAGAAGGT

>Bacillus_Fam_1216_52_1 Nr. of seq. 1 Alignment length(with gaps) = 52
Alignment score = 0.000000
GCF_001587375.1_ASM158737v1_genomic.fna_68:1509-1873 Satlength=365 Nr of Repeats=7 RepeatLength=52 seed=GCACAGCAAT Num.seqs=7 Similarity=0.821734
0 GCACAGCAATGGTACCGCTTCTCCTTTAACCGGTTCCATAGCAGTACGCCCG

****
Consensus:

GCACAGCAATGGTACCGCTTCTCCTTTAACCGGTTCCATAGCAGTACGCCCG

>Bacillus_Fam_1217_52_1 Nr. of seq. 1 Alignment length(with gaps) = 52
Alignment score = 0.000000
GCF_001587375.1_ASM158737v1_genomic.fna_89:4001-4209 Satlength=209 Nr of Repeats=4 RepeatLength=52 seed=CAAGTGGACG Num.seqs=4 Similarity=0.901709
0 CAAGTGGACGAATCCGCCGGATGCGGGAAGATAAGGATGGATGAACCCTGCT

****
Consensus:

CAAGTGGACGAATCCGCCGGATGCGGGAAGATAAGGATGGATGAACCCTGCT

>Bacillus_Fam_1218_52_1 Nr. of seq. 1 Alignment length(with gaps) = 52
Alignment score = 0.000000
GCF_001587375.1_ASM158737v1_genomic.fna_89:39868-40128 Satlength=261 Nr of Repeats=5 RepeatLength=52 seed=CCAACTTGGT Num.seqs=3 Similarity=0.794872
0 CCAACTTGGTCGCCAAAGCCCCGGAATGAAGCCCAAGAACGAAGCAANNAGT

****
Consensus:

CCAACTTGGTCGCCAAAGCCCCGGAATGAAGCCCAAGAACGAAGCAANNAGT

>Bacillus_Fam_1219_52_1 Nr. of seq. 1 Alignment length(with gaps) = 52
Alignment score = 0.000000

GCF_001587375.1_ASM158737v1_genomic.fna_98:17349-17608 Satlength=260 Nr of Repeats=4 RepeatLength=52 seed=TGCCCGAAAG Num.seqs=3 Similarity=0.948718
0   TGCCCGAAAGAGCCGTATGCGGGAATTTGAACAAGACCCATCAATCCCGAAG

****
Consensus:

TGCCCGAAAGAGCCGTATGCGGGAATTTGAACAAGACCCATCAATCCCGAAG

>Bacillus_Fam_1220_52_1 Nr. of seq. 1 Alignment length(with gaps) = 52 Alignment score = 0.000000
GCF_001587375.1_ASM158737v1_genomic.fna_154:82039-82563 Satlength=525 Nr of Repeats=10 RepeatLength=52 seed=CAAAATTAAA Num.seqs=9 Similarity=0.890313 0
CAAAATTAAAACAAGAAGTGCTTCATACAGCGTTTATGAGGTACATGGTTTT

****
Consensus:

CAAAATTAAAACAAGAAGTGCTTCATACAGCGTTTATGAGGTACATGGTTTT

>Bacillus_Fam_1221_52_1 Nr. of seq. 1 Alignment length(with gaps) = 52 Alignment score = 0.000000
GCF_001587375.1_ASM158737v1_genomic.fna_167:65422-65630 Satlength=209 Nr of Repeats=4 RepeatLength=52 seed=TACATTTTAC Num.seqs=4 Similarity=0.803419
0   TACATTTTACAAGCATCTATTCAAAAATGTAAAAGCAAAGCAAGGATACCTA

****
Consensus:

TACATTTTACAAGCATCTATTCAAAAATGTAAAAGCAAAGCAAGGATACCTA

>Bacillus_Fam_1222_52_1 Nr. of seq. 1 Alignment length(with gaps) = 52 Alignment score = 0.000000
GCF_001587375.1_ASM158737v1_genomic.fna_171:145480-145896 Satlength=417 Nr of Repeats=8 RepeatLength=52 seed=GTCCGATGGA Num.seqs=8 Similarity=0.862637 0
GTCCGATGGAGAAGGAAAGCGTGAGGCTATGAGGCTGGAAAGAAGAGAAGCG

****
Consensus:

GTCCGATGGAGAAGGAAAGCGTGAGGCTATGAGGCTGGAAAGAAGAGAAGCG

>Bacillus_Fam_1223_52_1 Nr. of seq. 1 Alignment length(with gaps) = 52 Alignment score = 0.000000
GCF_001590785.1_ASM159078v1_genomic.fna_17:35789-35997 Satlength=209 Nr of Repeats=5 RepeatLength=52 seed=GCTCCCTTTT Num.seqs=3 Similarity=0.693416
0   GCTCCCTTTTGACCNCTTTTCGAGTTCAGCGCTNTTTTCNGGTGAATAGGTG

****

Consensus:

GCTCCCTTTTGACCNCTTTTCGAGTTCAGCGCTNTTTTCNGGTGAATAGGTG

>Bacillus_Fam_1224_52_1 Nr. of seq. 1 Alignment length(with gaps) = 52
Alignment score = 0.000000
GCF_001591445.1_ASM159144v1_genomic.fna_10:32260-32468 Satlength=209 Nr of Repeats=4 RepeatLength=52 seed=TCTTTCTTAC Num.seqs=4 Similarity=0.914530
0 TCTTTCTTACCGTAACCACCTCTCAAACTAGTCTTCGGTCGTTTAAGAGCCC

****
Consensus:

TCTTTCTTACCGTAACCACCTCTCAAACTAGTCTTCGGTCGTTTAAGAGCCC

>Bacillus_Fam_1225_52_1 Nr. of seq. 1 Alignment length(with gaps) = 52
Alignment score = 0.000000
GCF_001591485.1_ASM159148v1_genomic.fna_48:825-1138 Satlength=314 Nr of Repeats=6 RepeatLength=52 seed=TTCCAATCAA Num.seqs=5 Similarity=0.812820
0 TTCCAATCAAGGGNATTGGTGCATGAACCCGAAAAAAACCGNGTAGTTCAAG

****
Consensus:

TTCCAATCAAGGGNATTGGTGCATGAACCCGAAAAAAACCGNGTAGTTCAAG

>Bacillus_Fam_1226_52_1 Nr. of seq. 1 Alignment length(with gaps) = 52
Alignment score = 0.000000
GCF_001591585.1_ASM159158v1_genomic.fna_5:97301-97510 Satlength=210 Nr of Repeats=4 RepeatLength=52 seed=GCCACTTTTT Num.seqs=3 Similarity=0.982906
0 GCCACTTTTTTCGCTTCTCCCTTCCATTTTGGCTTTCATCTTGGTAATGAAC

****
Consensus:

GCCACTTTTTTCGCTTCTCCCTTCCATTTTGGCTTTCATCTTGGTAATGAAC

>Bacillus_Fam_1227_52_1 Nr. of seq. 1 Alignment length(with gaps) = 52
Alignment score = 0.000000
GCF_001591585.1_ASM159158v1_genomic.fna_8:46370-46578 Satlength=209 Nr of Repeats=4 RepeatLength=52 seed=ATAAGTGGTT Num.seqs=4 Similarity=0.893162
0 ATAAGTGGTTTTCATTCAACAAATGAACGCCACTTTTTATGGAAAAGATGGC

****
Consensus:

ATAAGTGGTTTTCATTCAACAAATGAACGCCACTTTTTATGGAAAAGATGGC

>Bacillus_Fam_1228_52_1 Nr. of seq. 1 Alignment length(with gaps) = 52
Alignment score = 0.000000
GCF_001591585.1_ASM159158v1_genomic.fna_13:90669-90981 Satlength=313 Nr of Repeats=6 RepeatLength=52 seed=GTTCATCAGT Num.seqs=6

Similarity=0.924786
0   GTTCATCAGTTGGATGAAAGCCGAAATCCCGTGGAGCAAGAGGAAAAGAGGC

****
Consensus:

GTTCATCAGTTGGATGAAAGCCGAAATCCCGTGGAGCAAGAGGAAAAGAGGC

>Bacillus_Fam_1229_52_1 Nr. of seq. 1 Alignment length(with gaps) = 52
Alignment score = 0.000000
GCF_001591645.1_ASM159164v1_genomic.fna_1:671330-671590 Satlength=261 Nr of Repeats=5 RepeatLength=52 seed=TTTAGTGCCC Num.seqs=5
Similarity=0.866667
0   TTTAGTGCCCGCGTATACCAGAAATTAGTAGAATGAGGTCACTAAAAGTTAA

****
Consensus:

TTTAGTGCCCGCGTATACCAGAAATTAGTAGAATGAGGTCACTAAAAGTTAA

>Bacillus_Fam_1230_52_1 Nr. of seq. 1 Alignment length(with gaps) = 52
Alignment score = 0.000000
GCF_001591645.1_ASM159164v1_genomic.fna_1:803082-803290 Satlength=209 Nr of Repeats=4 RepeatLength=52 seed=TCCAGATATT Num.seqs=4
Similarity=0.743590
0   TCCAGATATTAGGAAAACCAGGGCACTATATTCAGATTTAACACCCTTAAAA

****
Consensus:

TCCAGATATTAGGAAAACCAGGGCACTATATTCAGATTTAACACCCTTAAAA

>Bacillus_Fam_1231_52_1 Nr. of seq. 1 Alignment length(with gaps) = 52
Alignment score = 0.000000
GCF_001591645.1_ASM159164v1_genomic.fna_1:810484-810796 Satlength=313 Nr of Repeats=6 RepeatLength=52 seed=AGTCCGAATG Num.seqs=6
Similarity=0.801709
0   AGTCCGAATGACGGTCTGATTAGGACAGCAACAGCAGCTCAAACTAATTCAC

****
Consensus:

AGTCCGAATGACGGTCTGATTAGGACAGCAACAGCAGCTCAAACTAATTCAC

>Bacillus_Fam_1232_52_1 Nr. of seq. 1 Alignment length(with gaps) = 52
Alignment score = 0.000000
GCF_001591645.1_ASM159164v1_genomic.fna_5:34073-34385 Satlength=313 Nr of Repeats=6 RepeatLength=52 seed=GAAGTTCATC Num.seqs=6 Similarity=0.806838
0   GAAGTTCATCTGGCTCATGAACGTCATTACAAAGAGAAAAGACTGAGCAAAT

****
Consensus:

GAAGTTCATCTGGCTCATGAACGTCATTACAAAGAGAAAAGACTGAGCAAAT

>Bacillus_Fam_1233_52_1 Nr. of seq. 1 Alignment length(with gaps) = 52
Alignment score = 0.000000
GCF_001591645.1_ASM159164v1_genomic.fna_18:93814-94126 Satlength=313 Nr of Repeats=6 RepeatLength=52 seed=TCTCTTACCA Num.seqs=6 Similarity=0.747009
0   TCTCTTACCATCCATCATGATTTTCCGCCCGTTTGGGCACTGAGACGCCTTC

****
Consensus:

TCTCTTACCATCCATCATGATTTTCCGCCCGTTTGGGCACTGAGACGCCTTC

>Bacillus_Fam_1234_52_1 Nr. of seq. 1 Alignment length(with gaps) = 52
Alignment score = 0.000000
GCF_001591665.1_ASM159166v1_genomic.fna_28:471-678 Satlength=208 Nr of Repeats=4 RepeatLength=52 seed=ATCTTTCCCT Num.seqs=3 Similarity=0.897436
0   ATCTTTCCCTTCCTTTTTCAGCCAAACGTCGAAGTAGAACCACTCTACTTCA

****
Consensus:

ATCTTTCCCTTCCTTTTTCAGCCAAACGTCGAAGTAGAACCACTCTACTTCA

>Bacillus_Fam_1235_52_1 Nr. of seq. 1 Alignment length(with gaps) = 52
Alignment score = 0.000000
GCF_001591665.1_ASM159166v1_genomic.fna_41:6030-6288 Satlength=259 Nr of Repeats=5 RepeatLength=52 seed=GAAAGGGAGA Num.seqs=4 Similarity=0.893162
0   GAAAGGGAGAAACGGTCGATAAGAGCGGTTCATAACGACCATTTGGGTTGAG

****
Consensus:

GAAAGGGAGAAACGGTCGATAAGAGCGGTTCATAACGACCATTTGGGTTGAG

>Bacillus_Fam_1236_52_1 Nr. of seq. 1 Alignment length(with gaps) = 52
Alignment score = 0.000000
GCF_001591665.1_ASM159166v1_genomic.fna_57:81030-81238 Satlength=209 Nr of Repeats=4 RepeatLength=52 seed=CTCATGAGGA Num.seqs=4 Similarity=0.820513
0   CTCATGAGGACAGGAATCATGTTTAATTCACATTTTTTGTCCTCATGAAAGG

****
Consensus:

CTCATGAGGACAGGAATCATGTTTAATTCACATTTTTTGTCCTCATGAAAGG

>Bacillus_Fam_1237_52_1 Nr. of seq. 1 Alignment length(with gaps) = 52
Alignment score = 0.000000
GCF_001591665.1_ASM159166v1_genomic.fna_60:22815-23075 Satlength=261 Nr of Repeats=5 RepeatLength=52 seed=ATCGACCGTA Num.seqs=5 Similarity=0.912821
0   ATCGACCGTAATTGACTTTCAGCTTGGCTATCGGGCTTTATAAACCTTTCTT

****
Consensus:

ATCGACCGTAATTGACTTTCAGCTTGGCTATCGGGCTTTATAAACCTTTCTT

>Bacillus_Fam_1238_52_1 Nr. of seq. 1 Alignment length(with gaps) = 52
Alignment score = 0.000000
GCF_001591665.1_ASM159166v1_genomic.fna_94:124-764 Satlength=641 Nr of Repeats=7 RepeatLength=52 seed=ATGAAGGACT Num.seqs=5 Similarity=0.808805
0 ATGAAGGACTAATCCCTATTAGAAAGACAGCGTTTTGTCCTTCATCAAGCAT

****
Consensus:

ATGAAGGACTAATCCCTATTAGAAAGACAGCGTTTTGTCCTTCATCAAGCAT

>Bacillus_Fam_1239_52_1 Nr. of seq. 1 Alignment length(with gaps) = 52
Alignment score = 0.000000
GCF_001591665.1_ASM159166v1_genomic.fna_102:8439-8701 Satlength=263 Nr of Repeats=5 RepeatLength=52 seed=GGTTTCCAAT Num.seqs=3 Similarity=0.769231
0 GGTTTCCAATCAGCCCTATTGGAACAAGTTAAGGCNAAAAAGTCGGTCAAAA

****
Consensus:

GGTTTCCAATCAGCCCTATTGGAACAAGTTAAGGCNAAAAAGTCGGTCAAAA

>Bacillus_Fam_1240_52_1 Nr. of seq. 1 Alignment length(with gaps) = 52
Alignment score = 0.000000
GCF_001591665.1_ASM159166v1_genomic.fna_108:0-209 Satlength=210 Nr of Repeats=4 RepeatLength=52 seed=TTTTGTCCTC Num.seqs=3 Similarity=0.846154
0 TTTTGTCCTCATGAAGCACTCATGCGGACAGTTTCGCCTTGGGAACCCCGGG

****
Consensus:

TTTTGTCCTCATGAAGCACTCATGCGGACAGTTTCGCCTTGGGAACCCCGGG

>Bacillus_Fam_1241_52_1 Nr. of seq. 1 Alignment length(with gaps) = 52
Alignment score = 0.000000
GCF_001591805.1_ASM159180v1_genomic.fna_10:197189-197658 Satlength=470 Nr of Repeats=9 RepeatLength=52 seed=TCATAGTGCC Num.seqs=8 Similarity=0.825092 0
TCATAGTGCCCGAAACCTGAAAAAAACCAGCCACTCGGGCACTGAGAGCAGT

****
Consensus:

TCATAGTGCCCGAAACCTGAAAAAAACCAGCCACTCGGGCACTGAGAGCAGT

>Bacillus_Fam_1242_52_1 Nr. of seq. 1 Alignment length(with gaps) = 52
Alignment score = 0.000000

GCF_001591805.1_ASM159180v1_genomic.fna_28:10204-10514 Satlength=311 Nr of Repeats=6 RepeatLength=52 seed=CTATTGGACA Num.seqs=4 Similarity=0.858974
0   CTATTGGACAGTTGGGGTTGTATCTTCGTCGATATTGTCCTATAGACCGCTT

****
Consensus:

CTATTGGACAGTTGGGGTTGTATCTTCGTCGATATTGTCCTATAGACCGCTT

>Bacillus_Fam_1243_52_1 Nr. of seq. 1 Alignment length(with gaps) = 52 Alignment score = 0.000000
GCF_001591805.1_ASM159180v1_genomic.fna_38:47701-47961 Satlength=261 Nr of Repeats=5 RepeatLength=52 seed=GGATAACGGG Num.seqs=5 Similarity=0.861538
0   GGATAACGGGCTCCAATGAGGGTTATTGGCGACCGAAACGCTCTCGAATTCC

****
Consensus:

GGATAACGGGCTCCAATGAGGGTTATTGGCGACCGAAACGCTCTCGAATTCC

>Bacillus_Fam_1244_52_1 Nr. of seq. 1 Alignment length(with gaps) = 52 Alignment score = 0.000000
GCF_001591805.1_ASM159180v1_genomic.fna_49:18770-18980 Satlength=211 Nr of Repeats=4 RepeatLength=52 seed=GGTCACAGAG Num.seqs=3 Similarity=0.811966
0   GGTCACAGAGAGCGATCCTCAGTGCCCGAAGAGAGGCAAAAAGCTGTCTAGC

****
Consensus:

GGTCACAGAGAGCGATCCTCAGTGCCCGAAGAGAGGCAAAAAGCTGTCTAGC

>Bacillus_Fam_1245_52_1 Nr. of seq. 1 Alignment length(with gaps) = 52 Alignment score = 0.000000
GCF_001591945.1_ASM159194v1_genomic.fna_6:8074-8494 Satlength=421 Nr of Repeats=8 RepeatLength=52 seed=TGTCCCTTAG Num.seqs=7 Similarity=0.863248
0   TGTCCCTTAGATCCTCTCTCTCGGACACTCCTTCTCCTTTTTCCTCAATATT

****
Consensus:

TGTCCCTTAGATCCTCTCTCTCGGACACTCCTTCTCCTTTTTCCTCAATATT

>Bacillus_Fam_1246_52_1 Nr. of seq. 1 Alignment length(with gaps) = 52 Alignment score = 0.000000
GCF_001591945.1_ASM159194v1_genomic.fna_9:37775-37983 Satlength=209 Nr of Repeats=4 RepeatLength=52 seed=TGTCCTTTAG Num.seqs=4 Similarity=0.957265
0   TGTCCTTTAGACGACTTCTCTCGGACACTTCCAACCCCTTCTCCCGCGAGAC

****
Consensus:

TGTCCTTTAGACGACTTCTCTCGGACACTTCCAACCCCTTCTCCCGCGAGAC

>Bacillus_Fam_1247_52_1 Nr. of seq. 1 Alignment length(with gaps) = 52 Alignment score = 0.000000
GCF_001591945.1_ASM159194v1_genomic.fna_11:48233-48493 Satlength=261 Nr of Repeats=5 RepeatLength=52 seed=CTATTGGACA Num.seqs=5 Similarity=0.730409
0   CTATTGGACAGATCCTCTCCTCTTTTGGCTCGGACTGTCCTTTAGCGCCCTC

****
Consensus:

CTATTGGACAGATCCTCTCCTCTTTTGGCTCGGACTGTCCTTTAGCGCCCTC

>Bacillus_Fam_1248_52_1 Nr. of seq. 1 Alignment length(with gaps) = 52 Alignment score = 0.000000
GCF_001636315.1_ASM163631v1_genomic.fna_1:512977-513185 Satlength=209 Nr of Repeats=4 RepeatLength=52 seed=TATCGAAGAC Num.seqs=4 Similarity=0.679487
0   TATCGAAGACAAATTCCACCGCTTCCACCCTCGTTTTGTCCTCAAGACGCCT

****
Consensus:

TATCGAAGACAAATTCCACCGCTTCCACCCTCGTTTTGTCCTCAAGACGCCT

>Bacillus_Fam_1249_52_1 Nr. of seq. 1 Alignment length(with gaps) = 52 Alignment score = 0.000000
GCF_001636315.1_ASM163631v1_genomic.fna_1:972202-972514 Satlength=313 Nr of Repeats=6 RepeatLength=52 seed=GGGCACGTAG Num.seqs=6 Similarity=0.717400
0   GGGCACGTAGACGCNTTCTACGTTACCTTGAGGCGGGATATTTACGCTGGAT

****
Consensus:

GGGCACGTAGACGCNTTCTACGTTACCTTGAGGCGGGATATTTACGCTGGAT

>Bacillus_Fam_1250_52_1 Nr. of seq. 1 Alignment length(with gaps) = 52 Alignment score = 0.000000
GCF_001636315.1_ASM163631v1_genomic.fna_1:1021216-1021892 Satlength=677 Nr of Repeats=13 RepeatLength=52 seed=GGGCACGTAG Num.seqs=13 Similarity=0.788955 0
GGGCACGTAGANCACGTCTACGTGACCGAACCGTCAGTTTTTCAGCTGNTTG

****
Consensus:

GGGCACGTAGANCACGTCTACGTGACCGAACCGTCAGTTTTTCAGCTGNTTG

>Bacillus_Fam_1251_52_1 Nr. of seq. 1 Alignment length(with gaps) = 52 Alignment score = 0.000000

GCF_001636315.1_ASM163631v1_genomic.fna_1:1467817-1468025 Satlength=209
Nr of Repeats=4 RepeatLength=52 seed=CGGGCACGTA Num.seqs=4
Similarity=0.722222 86
CTATGTGACCTTACACTGCTTTTTCATGCCCGTTCGGGCACGTAGAACACTT

****
Consensus:

CGGGCACGTAGAACACTTCTATGTGACCTTACACTGCTTTTTCATGCCCGTT

>Bacillus_Fam_1252_52_1 Nr. of seq. 1 Alignment length(with gaps) = 52
Alignment score = 0.000000
GCF_001636315.1_ASM163631v1_genomic.fna_1:1863825-1864292 Satlength=468
Nr of Repeats=9 RepeatLength=52 seed=TATTGAAGAC Num.seqs=8
Similarity=0.775641 0
TATTGAAGACAAAATGAGATCACTCCCTTTTAAATTTGTCCCCAATACCCTC

****
Consensus:

TATTGAAGACAAAATGAGATCACTCCCTTTTAAATTTGTCCCCAATACCCTC

>Bacillus_Fam_1253_52_1 Nr. of seq. 1 Alignment length(with gaps) = 52
Alignment score = 0.000000
GCF_001636315.1_ASM163631v1_genomic.fna_1:1939675-1940039 Satlength=365
Nr of Repeats=6 RepeatLength=52 seed=AAGTGTCTCC Num.seqs=5
Similarity=0.843590 0
AAGTGTCTCCAATAAGCTTCATTGGAGACAAAACCCNCTCTTTTTTCACGAG

****
Consensus:

AAGTGTCTCCAATAAGCTTCATTGGAGACAAAACCCNCTCTTTTTTCACGAG

>Bacillus_Fam_1254_52_1 Nr. of seq. 1 Alignment length(with gaps) = 52
Alignment score = 0.000000
GCF_001636315.1_ASM163631v1_genomic.fna_1:2327343-2327760 Satlength=418
Nr of Repeats=8 RepeatLength=52 seed=AAAGTCACGT Num.seqs=7
Similarity=0.738389 0
AAAGTCACGTAGAAACCCTCTACGTGACCGTACCTCCCTTTTTNCCTGCTCT

****
Consensus:

AAAGTCACGTAGAAACCCTCTACGTGACCGTACCTCCCTTTTTNCCTGCTCT

>Bacillus_Fam_1255_52_1 Nr. of seq. 1 Alignment length(with gaps) = 52
Alignment score = 0.000000
GCF_001636315.1_ASM163631v1_genomic.fna_1:2431592-2432058 Satlength=467
Nr of Repeats=9 RepeatLength=52 seed=CGGATTTTCC Num.seqs=6
Similarity=0.835897 0
CGGATTTTCCCTTTCAGACATCACTTAGACGGNCTCTACTTAGTGTCTAAAC

****

Consensus:

CGGATTTTCCCTTTCAGACATCACTTAGACGGNCTCTACTTAGTGTCTAAAC

>Bacillus_Fam_1256_52_1 Nr. of seq. 1 Alignment length(with gaps) = 52
Alignment score = 0.000000
GCF_001636315.1_ASM163631v1_genomic.fna_1:2907285-2907649 Satlength=365
Nr of Repeats=7 RepeatLength=52 seed=ATGAGAACTG Num.seqs=7
Similarity=0.755316 0
ATGAGAACTGAAACCANGAAAAGCGCGGCNGTCTTGTCCTCATGAAGCGTTC

****
Consensus:

ATGAGAACTGAAACCANGAAAAGCGCGGCNGTCTTGTCCTCATGAAGCGTTC

>Bacillus_Fam_1257_52_1 Nr. of seq. 1 Alignment length(with gaps) = 52
Alignment score = 0.000000
GCF_001636315.1_ASM163631v1_genomic.fna_1:3080314-3080988 Satlength=675
Nr of Repeats=13 RepeatLength=52 seed=GACCGAGCAG Num.seqs=11
Similarity=0.820047 0
GACCGAGCAGCTGGAAAAATAGGAAGAAGAGTCGCGTAGAGAGGTTCTANGC

****
Consensus:

GACCGAGCAGCTGGAAAAATAGGAAGAAGAGTCGCGTAGAGAGGTTCTANGC

>Bacillus_Fam_1258_52_1 Nr. of seq. 1 Alignment length(with gaps) = 52
Alignment score = 0.000000
GCF_001636315.1_ASM163631v1_genomic.fna_1:3169529-3169945 Satlength=417
Nr of Repeats=8 RepeatLength=52 seed=TAAGAAAGGC Num.seqs=8
Similarity=0.836081 0
TAAGAAAGGCTCTCAAGGACAAAACGCTAGTAAAAATTAATAGAANTGTCTT

****
Consensus:

TAAGAAAGGCTCTCAAGGACAAAACGCTAGTAAAAATTAATAGAANTGTCTT

>Bacillus_Fam_1259_52_1 Nr. of seq. 1 Alignment length(with gaps) = 52
Alignment score = 0.000000
GCF_001636315.1_ASM163631v1_genomic.fna_1:3506345-3506708 Satlength=364
Nr of Repeats=7 RepeatLength=52 seed=TCCATTGCCC Num.seqs=6
Similarity=0.880342 0
TCCATTGCCCAAAACCGGCTAAATCATCAAATTATGGTCAATGCAGCCCCAT

****
Consensus:

TCCATTGCCCAAAACCGGCTAAATCATCAAATTATGGTCAATGCAGCCCCAT

>Bacillus_Fam_1260_52_1 Nr. of seq. 1 Alignment length(with gaps) = 52
Alignment score = 0.000000

GCF_001636315.1_ASM163631v1_genomic.fna_1:3527584-3527946 Satlength=363
Nr of Repeats=7 RepeatLength=52 seed=TGTCTGAATG Num.seqs=5
Similarity=0.902564 0
TGTCTGAATGATTATTTTGCAGCCAGAGATCAGTTAGAATCCGTCTAGTTGC

****
Consensus:

TGTCTGAATGATTATTTTGCAGCCAGAGATCAGTTAGAATCCGTCTAGTTGC

>Bacillus_Fam_1261_52_1 Nr. of seq. 1 Alignment length(with gaps) = 52
Alignment score = 0.000000
GCF_001636315.1_ASM163631v1_genomic.fna_1:3645171-3645639 Satlength=469
Nr of Repeats=9 RepeatLength=52 seed=ATGAACCTTT Num.seqs=9
Similarity=0.947293 0
ATGAACCTTTCATAGAGCGCGTCGGAGTAAAAAAATCGAGCTCCCGCGTACT

****
Consensus:

ATGAACCTTTCATAGAGCGCGTCGGAGTAAAAAAATCGAGCTCCCGCGTACT

>Bacillus_Fam_1262_52_1 Nr. of seq. 1 Alignment length(with gaps) = 52
Alignment score = 0.000000
GCF_001636315.1_ASM163631v1_genomic.fna_1:3709317-3709941 Satlength=625
Nr of Repeats=12 RepeatLength=52 seed=GTTCATAGAG Num.seqs=12
Similarity=0.770436 0
GTTCATAGAGCGCGAACNTGAGGGGAAATTCCAAAAACGCGCACTATAAAGA

****
Consensus:

GTTCATAGAGCGCGAACNTGAGGGGAAATTCCAAAAACGCGCACTATAAAGA

>Bacillus_Fam_1263_52_1 Nr. of seq. 1 Alignment length(with gaps) = 52
Alignment score = 0.000000
GCF_001636315.1_ASM163631v1_genomic.fna_1:3807647-3808011 Satlength=365
Nr of Repeats=7 RepeatLength=52 seed=TTTCGGAAAA Num.seqs=7
Similarity=0.824176 0
TTTCGGAAAAGGTGTTCATAGAAGAGGTCAATGAGCACATGAGGCTGAGTTT

****
Consensus:

TTTCGGAAAAGGTGTTCATAGAAGAGGTCAATGAGCACATGAGGCTGAGTTT

>Bacillus_Fam_1264_52_1 Nr. of seq. 1 Alignment length(with gaps) = 52
Alignment score = 0.000000
GCF_001636315.1_ASM163631v1_genomic.fna_1:3869085-3869761 Satlength=677
Nr of Repeats=13 RepeatLength=52 seed=ACTATGGGAG Num.seqs=13
Similarity=0.892176 0
ACTATGGGAGGTTCATAGAGCGNGAAATTAGGAAAAAGGAAAGACTCCGCGC

****

Consensus:

ACTATGGGAGGTTCATAGAGCGNGAAATTAGGAAAAAGGAAAGACTCCGCGC

>Bacillus_Fam_1265_52_1 Nr. of seq. 1 Alignment length(with gaps) = 52
Alignment score = 0.000000
GCF_001636315.1_ASM163631v1_genomic.fna_1:3880946-3881417 Satlength=472
Nr of Repeats=9 RepeatLength=52 seed=TATTGCCCGA Num.seqs=6
Similarity=0.801709 0
TATTGCCCGAAGAGAAGAAAAAATAAATCCCGCGGTAAAAAGACCGGGCTTC

****
Consensus:

TATTGCCCGAAGAGAAGAAAAAATAAATCCCGCGGTAAAAAGACCGGGCTTC

>Bacillus_Fam_1266_52_1 Nr. of seq. 1 Alignment length(with gaps) = 52
Alignment score = 0.000000
GCF_001636315.1_ASM163631v1_genomic.fna_1:4057906-4058531 Satlength=626
Nr of Repeats=11 RepeatLength=52 seed=AGCGTTTATG Num.seqs=9
Similarity=0.918091 0
AGCGTTTATGAGAACAGAGAGCTAATGATTTGGGTCGGAACTGTCCTCATGA

****
Consensus:

AGCGTTTATGAGAACAGAGAGCTAATGATTTGGGTCGGAACTGTCCTCATGA

>Bacillus_Fam_1267_52_1 Nr. of seq. 1 Alignment length(with gaps) = 52
Alignment score = 0.000000
GCF_001636315.1_ASM163631v1_genomic.fna_1:4191741-4192522 Satlength=782
Nr of Repeats=15 RepeatLength=52 seed=AAAAAAGAGA Num.seqs=13
Similarity=0.833333 0
AAAAAAGAGAGCGCCGGGTAGAATANAGAGCATCTATGCGACCGAGCCCGAG

****
Consensus:

AAAAAAGAGAGCGCCGGGTAGAATANAGAGCATCTATGCGACCGAGCCCGAG

>Bacillus_Fam_1268_52_1 Nr. of seq. 1 Alignment length(with gaps) = 52
Alignment score = 0.000000
GCF_001636315.1_ASM163631v1_genomic.fna_1:4719016-4719328 Satlength=313
Nr of Repeats=6 RepeatLength=52 seed=TCAATGAACA Num.seqs=6
Similarity=0.734382 0
TCAATGAACACGAATCGGCAAAATTTCAGGCAGGAAGTGTTCATAGAGAGGT

****
Consensus:

TCAATGAACACGAATCGGCAAAATTTCAGGCAGGAAGTGTTCATAGAGAGGT

>Bacillus_Fam_1269_52_1 Nr. of seq. 1 Alignment length(with gaps) = 52
Alignment score = 0.000000

GCF_001636315.1_ASM163631v1_genomic.fna_1:5285435-5285695 Satlength=261 Nr of Repeats=5 RepeatLength=52 seed=TCTACGTGCC Num.seqs=5 Similarity=0.846154 0
TCTACGTGCCCCAAGTTCAATAAATTAGTGGGTTTCGGTCGCGTAGAGCCCT

****
Consensus:

TCTACGTGCCCCAAGTTCAATAAATTAGTGGGTTTCGGTCGCGTAGAGCCCT

>Bacillus_Fam_1270_52_1 Nr. of seq. 1 Alignment length(with gaps) = 52 Alignment score = 0.000000
GCF_001636315.1_ASM163631v1_genomic.fna_1:5395730-5396199 Satlength=470 Nr of Repeats=9 RepeatLength=52 seed=TTGACCTTTT Num.seqs=8 Similarity=0.780997 0
TTGACCTTTTCTTTNAAAATCAATTAGTTTTAGGCAATGNACATGGCTTCCA

****
Consensus:

TTGACCTTTTCTTTNAAAATCAATTAGTTTTAGGCAATGNACATGGCTTCCA

>Bacillus_Fam_1271_52_1 Nr. of seq. 1 Alignment length(with gaps) = 52 Alignment score = 0.000000
GCF_001636325.1_ASM163632v1_genomic.fna_1:270063-270479 Satlength=417 Nr of Repeats=8 RepeatLength=52 seed=CATCCTCTTC Num.seqs=8 Similarity=0.888278
0 CATCCTCTTCAGGTGAAAAAAGCCCAATTATTCACCGGAGTGCTCATATTTT

****
Consensus:

CATCCTCTTCAGGTGAAAAAAGCCCAATTATTCACCGGAGTGCTCATATTTT

>Bacillus_Fam_1272_52_1 Nr. of seq. 1 Alignment length(with gaps) = 52 Alignment score = 0.000000
GCF_001636325.1_ASM163632v1_genomic.fna_1:669107-669471 Satlength=365 Nr of Repeats=7 RepeatLength=52 seed=TTATTCCTCT Num.seqs=7 Similarity=0.777778
0 TTATTCCTCTAAAGCAACGCCACACGCGCCTCTTCAGGTGAACAAATCGCAT

****
Consensus:

TTATTCCTCTAAAGCAACGCCACACGCGCCTCTTCAGGTGAACAAATCGCAT

>Bacillus_Fam_1273_52_1 Nr. of seq. 1 Alignment length(with gaps) = 52 Alignment score = 0.000000
GCF_001636325.1_ASM163632v1_genomic.fna_1:2472643-2473059 Satlength=417 Nr of Repeats=8 RepeatLength=52 seed=GTTATTTACA Num.seqs=8 Similarity=0.934066 0
GTTATTTACACGAAGGGATTTTGAACCGAATGCTTCAGGTGAATAAATTGCG

****

Consensus:

GTTATTTACACGAAGGGATTTTGAACCGAATGCTTCAGGTGAATAAATTGCG

>Bacillus_Fam_1274_52_1 Nr. of seq. 1 Alignment length(with gaps) = 52 Alignment score = 0.000000
GCF_001636325.1_ASM163632v1_genomic.fna_3:175232-175492 Satlength=261 Nr of Repeats=5 RepeatLength=52 seed=ATTTATTCCC Num.seqs=5 Similarity=0.893082
8 TAAACTGAATTTATTCCCATGAAGCGAGAGGAAAACCGGAGCTTCCGGTGGA

****
Consensus:

ATTTATTCCCATGAAGCGAGAGGAAAACCGGAGCTTCCGGTGGATAAACTGA

>Bacillus_Fam_1275_52_1 Nr. of seq. 1 Alignment length(with gaps) = 52 Alignment score = 0.000000
GCF_001636325.1_ASM163632v1_genomic.fna_3:896857-897117 Satlength=261 Nr of Repeats=5 RepeatLength=52 seed=AAAATGGTCA Num.seqs=5 Similarity=0.910256
0 AAAATGGTCACAGAGTGCCAAACTAAGACCAAAATGACAAGGGAGCAAGCGG

****
Consensus:

AAAATGGTCACAGAGTGCCAAACTAAGACCAAAATGACAAGGGAGCAAGCGG

>Bacillus_Fam_1276_52_1 Nr. of seq. 1 Alignment length(with gaps) = 52 Alignment score = 0.000000
GCF_001636325.1_ASM163632v1_genomic.fna_3:1103562-1104238 Satlength=677 Nr of Repeats=13 RepeatLength=52 seed=GGAAGGTAGG Num.seqs=13 Similarity=0.902696 0
GGAAGGTAGGAGAGAGGCAGTCTCATCGCCCGTTCCCAGCTATTTACCATTA

****
Consensus:

GGAAGGTAGGAGAGAGGCAGTCTCATCGCCCGTTCCCAGCTATTTACCATTA

>Bacillus_Fam_1277_52_1 Nr. of seq. 1 Alignment length(with gaps) = 52 Alignment score = 0.000000
GCF_001636335.1_ASM163633v1_genomic.fna_1:243388-244011 Satlength=624 Nr of Repeats=12 RepeatLength=52 seed=CATAGAGCGA Num.seqs=11 Similarity=0.809324 0
CATAGAGCGAGTCAATGGGACATTTNGCAATTTTCNAGAGCAGCAAATGTCC

****
Consensus:

CATAGAGCGAGTCAATGGGACATTTNGCAATTTTCNAGAGCAGCAAATGTCC

>Bacillus_Fam_1278_52_1 Nr. of seq. 1 Alignment length(with gaps) = 52 Alignment score = 0.000000

GCF_001636335.1_ASM163633v1_genomic.fna_1:698208-698468 Satlength=261 Nr of Repeats=5 RepeatLength=52 seed=TGATGGACAA Num.seqs=5 Similarity=0.833962
0   TGATGGACAAAACAGTAGATAGAGCGCAAAGCAAAGTCCATCAATCCTGAGA

****
Consensus:

TGATGGACAAAACAGTAGATAGAGCGCAAAGCAAAGTCCATCAATCCTGAGA

>Bacillus_Fam_1279_52_1 Nr. of seq. 1 Alignment length(with gaps) = 52 Alignment score = 0.000000
GCF_001636335.1_ASM163633v1_genomic.fna_1:1609919-1610545 Satlength=627 Nr of Repeats=12 RepeatLength=52 seed=TTCCTTCGTT Num.seqs=9 Similarity=0.924501 0
TTCCTTCGTTTCTTCATGGGTTTTGTACCATTGATTGCTTCTATGGGACATG

****
Consensus:

TTCCTTCGTTTCTTCATGGGTTTTGTACCATTGATTGCTTCTATGGGACATG

>Bacillus_Fam_1280_52_1 Nr. of seq. 1 Alignment length(with gaps) = 52 Alignment score = 0.000000
GCF_001636335.1_ASM163633v1_genomic.fna_1:2312900-2313420 Satlength=521 Nr of Repeats=10 RepeatLength=52 seed=GATTTTTCCT Num.seqs=10 Similarity=0.885470 0
GATTTTTCCTAAAGTACCTTGTTGCGGGCGACATGAGCGCTTCTTGGCTACC

****
Consensus:

GATTTTTCCTAAAGTACCTTGTTGCGGGCGACATGAGCGCTTCTTGGCTACC

>Bacillus_Fam_1281_52_1 Nr. of seq. 1 Alignment length(with gaps) = 52 Alignment score = 0.000000
GCF_001636335.1_ASM163633v1_genomic.fna_1:2486081-2486704 Satlength=624 Nr of Repeats=10 RepeatLength=52 seed=GACTTTTTCG Num.seqs=8 Similarity=0.874542 0
GACTTTTTCGGGAGACATGAGCTAGTCTTGGCGACCGGTTTCACAAGTTGAT

****
Consensus:

GACTTTTTCGGGAGACATGAGCTAGTCTTGGCGACCGGTTTCACAAGTTGAT

>Bacillus_Fam_1282_52_1 Nr. of seq. 1 Alignment length(with gaps) = 52 Alignment score = 0.000000
GCF_001636335.1_ASM163633v1_genomic.fna_1:2726431-2726899 Satlength=469 Nr of Repeats=9 RepeatLength=52 seed=TTCTATGAGA Num.seqs=9 Similarity=0.837607 0
TTCTATGAGACAAAAATCGGGGAGCAGACGAGGNGTTTGTCTCATTGGAGCG

****

Consensus:

TTCTATGAGACAAAAATCGGGGAGCAGACGAGGNGTTTGTCTCATTGGAGCG

>Bacillus_Fam_1283_52_1 Nr. of seq. 1 Alignment length(with gaps) = 52
Alignment score = 0.000000
GCF_001636335.1_ASM163633v1_genomic.fna_1:2864058-2864994 Satlength=937
Nr of Repeats=16 RepeatLength=52 seed=GACTCTTTGA Num.seqs=14
Similarity=0.827557 0
GACTCTTTGAGCNGATTTTTCGGGCGTTGGGTCCTCTATCNGGCTTATTGAN

****
Consensus:

GACTCTTTGAGCNGATTTTTCGGGCGTTGGGTCCTCTATCNGGCTTATTGAN

>Bacillus_Fam_1284_52_1 Nr. of seq. 1 Alignment length(with gaps) = 52
Alignment score = 0.000000
GCF_001636335.1_ASM163633v1_genomic.fna_1:2901338-2902222 Satlength=885
Nr of Repeats=17 RepeatLength=52 seed=TTTGTCCTTC Num.seqs=17
Similarity=0.832579 0
TTTGTCCTTCATTCGCTTGATGATGGACTTTTTCTAGAGCGATCGGCTTCGN

****
Consensus:

TTTGTCCTTCATTCGCTTGATGATGGACTTTTTCTAGAGCGATCGGCTTCGN

>Bacillus_Fam_1285_52_1 Nr. of seq. 1 Alignment length(with gaps) = 52
Alignment score = 0.000000
GCF_001636335.1_ASM163633v1_genomic.fna_1:2936390-2936910 Satlength=521
Nr of Repeats=10 RepeatLength=52 seed=ATGATGGACA Num.seqs=10
Similarity=0.884331 0
ATGATGGACATTTCGATAATTTCCTCAGCTGCTTTTGGCCTTCATCTCTTCG

****
Consensus:

ATGATGGACATTTCGATAATTTCCTCAGCTGCTTTTGGCCTTCATCTCTTCG

>Bacillus_Fam_1286_52_1 Nr. of seq. 1 Alignment length(with gaps) = 52
Alignment score = 0.000000
GCF_001636335.1_ASM163633v1_genomic.fna_1:3065276-3065640 Satlength=365
Nr of Repeats=7 RepeatLength=52 seed=ATGATGGACA Num.seqs=7
Similarity=0.823600 0
ATGATGGACACTTGGAGCGGATTCTTTTTCGCTTTTGTCCATCATCTCTCGC

****
Consensus:

ATGATGGACACTTGGAGCGGATTCTTTTTCGCTTTTGTCCATCATCTCTCGC

>Bacillus_Fam_1287_52_1 Nr. of seq. 1 Alignment length(with gaps) = 52
Alignment score = 0.000000

GCF_001636335.1_ASM163633v1_genomic.fna_1:3328615-3328927 Satlength=313
Nr of Repeats=6 RepeatLength=52 seed=TTTTGTCCCA Num.seqs=6
Similarity=0.887179 0
TTTTGTCCCATAGAACCCCTCTATGGGACATTTCCTCTTGCCTATTCTGATC

****
Consensus:

TTTTGTCCCATAGAACCCCTCTATGGGACATTTCCTCTTGCCTATTCTGATC

>Bacillus_Fam_1288_52_1 Nr. of seq. 1 Alignment length(with gaps) = 52
Alignment score = 0.000000
GCF_001636345.1_ASM163634v1_genomic.fna_1:2372700-2373012 Satlength=313
Nr of Repeats=6 RepeatLength=52 seed=AGGATTTCAG Num.seqs=6
Similarity=0.871795 0
AGGATTTCAGGAATCAGAACATCAATGTAAATCCCGAAACCAGTTCCATGCA

****
Consensus:

AGGATTTCAGGAATCAGAACATCAATGTAAATCCCGAAACCAGTTCCATGCA

>Bacillus_Fam_1289_52_1 Nr. of seq. 1 Alignment length(with gaps) = 52
Alignment score = 0.000000
GCF_001636345.1_ASM163634v1_genomic.fna_1:2479013-2479325 Satlength=313
Nr of Repeats=6 RepeatLength=52 seed=ATTTGGATAT Num.seqs=6
Similarity=0.830769 0
ATTTGGATATTAAGGTCGTGATAAGCGCCTAAACACCCCTCTGTTATACAGT

****
Consensus:

ATTTGGATATTAAGGTCGTGATAAGCGCCTAAACACCCCTCTGTTATACAGT

>Bacillus_Fam_1290_52_1 Nr. of seq. 1 Alignment length(with gaps) = 52
Alignment score = 0.000000
GCF_001636345.1_ASM163634v1_genomic.fna_1:2544842-2545362 Satlength=521
Nr of Repeats=10 RepeatLength=52 seed=TCTCCTTCCC Num.seqs=10
Similarity=0.807827 0
TCTCCTTCCCCAACTCTATTGATGACCGGCATTTCGGGAAGTACTCTGCCTN

****
Consensus:

TCTCCTTCCCCAACTCTATTGATGACCGGCATTTCGGGAAGTACTCTGCCTN

>Bacillus_Fam_1291_52_1 Nr. of seq. 1 Alignment length(with gaps) = 52
Alignment score = 0.000000
GCF_001636345.1_ASM163634v1_genomic.fna_1:2589875-2590238 Satlength=364
Nr of Repeats=7 RepeatLength=52 seed=ATTCCCGAAT Num.seqs=6
Similarity=0.808547 0
ATTCCCGAATTCCACTTAATGCACTTGTTTCGGGAATTACTACGGGCTTCTC

****

Consensus:

ATTCCCGAATTCCACTTAATGCACTTGTTTCGGGAATTACTACGGGCTTCTC

>Bacillus_Fam_1292_52_1 Nr. of seq. 1 Alignment length(with gaps) = 52
Alignment score = 0.000000
GCF_001636345.1_ASM163634v1_genomic.fna_1:2600820-2601080 Satlength=261
Nr of Repeats=5 RepeatLength=52 seed=ACGGGCACTT Num.seqs=5
Similarity=0.628205 0
ACGGGCACTTTGANCGCACNTTTGTTCCCNCCAACACGCTTTTTCCAAGCCG

****
Consensus:

ACGGGCACTTTGANCGCACNTTTGTTCCCNCCAACACGCTTTTTCCAAGCCG

>Bacillus_Fam_1293_52_1 Nr. of seq. 1 Alignment length(with gaps) = 52
Alignment score = 0.000000
GCF_001636345.1_ASM163634v1_genomic.fna_1:2885450-2885866 Satlength=417
Nr of Repeats=8 RepeatLength=52 seed=GAATTAGAAT Num.seqs=8
Similarity=0.846154 0
GAATTAGAATCCAAGAGTCATTCCTGAAACGGTCTCAACCATGGAAGTTCGG

****
Consensus:

GAATTAGAATCCAAGAGTCATTCCTGAAACGGTCTCAACCATGGAAGTTCGG

>Bacillus_Fam_1294_52_1 Nr. of seq. 1 Alignment length(with gaps) = 52
Alignment score = 0.000000
GCF_001636345.1_ASM163634v1_genomic.fna_1:3320349-3320660 Satlength=312
Nr of Repeats=6 RepeatLength=52 seed=AACGCTTAAT Num.seqs=5
Similarity=0.864103 0
AACGCTTAATCACGACAGTATTATCCAGATAACGGTAATTAGGGGCGTGATA

****
Consensus:

AACGCTTAATCACGACAGTATTATCCAGATAACGGTAATTAGGGGCGTGATA

>Bacillus_Fam_1295_52_1 Nr. of seq. 1 Alignment length(with gaps) = 52
Alignment score = 0.000000
GCF_001636345.1_ASM163634v1_genomic.fna_1:3986989-3987509 Satlength=521
Nr of Repeats=10 RepeatLength=52 seed=AGCGGATTTC Num.seqs=10
Similarity=0.888889 0
AGCGGATTTCGGGAATTACTTTCCCTTTGCATTTCCCGAAACTTTGTCTCCT

****
Consensus:

AGCGGATTTCGGGAATTACTTTCCCTTTGCATTTCCCGAAACTTTGTCTCCT

>Bacillus_Fam_1296_52_1 Nr. of seq. 1 Alignment length(with gaps) = 52
Alignment score = 0.000000

GCF_001636345.1_ASM163634v1_genomic.fna_1:4285214-4285526 Satlength=313
Nr of Repeats=6 RepeatLength=52 seed=TTACGCCTTA Num.seqs=6
Similarity=0.856410 0
TTACGCCTTAAACACGACTCCAATTCCCAGGTTTTGGTGTTTCGTGTCTCGA

****
Consensus:

TTACGCCTTAAACACGACTCCAATTCCCAGGTTTTGGTGTTTCGTGTCTCGA

>Bacillus_Fam_1297_52_1 Nr. of seq. 1 Alignment length(with gaps) = 52
Alignment score = 0.000000
GCF_001636345.1_ASM163634v1_genomic.fna_1:4405935-4406559 Satlength=625
Nr of Repeats=12 RepeatLength=52 seed=GTTCTATTTC Num.seqs=12
Similarity=0.870241 0
GTTCTATTTCCCCAAACCGGCACTCGCCTTCTCTTTCGGGAATTACTTTGCC

****
Consensus:

GTTCTATTTCCCCAAACCGGCACTCGCCTTCTCTTTCGGGAATTACTTTGCC

>Bacillus_Fam_1298_52_1 Nr. of seq. 1 Alignment length(with gaps) = 52
Alignment score = 0.000000
GCF_001636425.1_ASM163642v1_genomic.fna_1:1121864-1122176 Satlength=313
Nr of Repeats=6 RepeatLength=52 seed=TTTTGTCCTT Num.seqs=6
Similarity=0.811966 0
TTTTGTCCTTCATACCACCGATGAAGGACTTTTGACAACGAAAATTCACCAG

****
Consensus:

TTTTGTCCTTCATACCACCGATGAAGGACTTTTGACAACGAAAATTCACCAG

>Bacillus_Fam_1299_52_1 Nr. of seq. 1 Alignment length(with gaps) = 52
Alignment score = 0.000000
GCF_001636425.1_ASM163642v1_genomic.fna_1:2575815-2576335 Satlength=521
Nr of Repeats=10 RepeatLength=52 seed=TGAAGGACTT Num.seqs=10
Similarity=0.852991 0
TGAAGGACTTTTATCACCTTTTATTCTCGCGAAAAGTCCTTCATACCTGCGA

****
Consensus:

TGAAGGACTTTTATCACCTTTTATTCTCGCGAAAAGTCCTTCATACCTGCGA

>Bacillus_Fam_1300_52_1 Nr. of seq. 1 Alignment length(with gaps) = 52
Alignment score = 0.000000
GCF_001636425.1_ASM163642v1_genomic.fna_1:4150616-4151084 Satlength=469
Nr of Repeats=9 RepeatLength=52 seed=GATGAAGGAC Num.seqs=9
Similarity=0.829060 0
GATGAAGGACTATTCTTGCTTTGATTGTGCTCGAATGGTCCATCATCCCCTC

****

Consensus:

GATGAAGGACTATTCTTGCTTTGATTGTGCTCGAATGGTCCATCATCCCCTC

>Bacillus_Fam_1301_52_1 Nr. of seq. 1 Alignment length(with gaps) = 52
Alignment score = 0.000000
GCF_001636425.1_ASM163642v1_genomic.fna_1:4305654-4306121 Satlength=468
Nr of Repeats=9 RepeatLength=52 seed=AAGTCCTTCA Num.seqs=8
Similarity=0.836081 0
AAGTCCTTCATCGTGGTTATGAAGACCAATAAGAGGTTAATTAGGAAGCGAA

****
Consensus:

AAGTCCTTCATCGTGGTTATGAAGACCAATAAGAGGTTAATTAGGAAGCGAA

>Bacillus_Fam_1302_52_1 Nr. of seq. 1 Alignment length(with gaps) = 52
Alignment score = 0.000000
GCF_001645705.1_ASM164570v1_genomic.fna_54:62195-62753 Satlength=559 Nr
of Repeats=10 RepeatLength=52 seed=CGGTCGCATA Num.seqs=8
Similarity=0.845013
0 CGGTCGCATAGCAAGGTTCTATAAGCCCGGAAACACAGGAAATCCAATGGAA

****
Consensus:

CGGTCGCATAGCAAGGTTCTATAAGCCCGGAAACACAGGAAATCCAATGGAA

>Bacillus_Fam_1303_52_1 Nr. of seq. 1 Alignment length(with gaps) = 52
Alignment score = 0.000000
GCF_001645705.1_ASM164570v1_genomic.fna_60:318578-318994 Satlength=417 Nr
of Repeats=8 RepeatLength=52 seed=AGTGAACCGG Num.seqs=8
Similarity=0.891941 0
AGTGAACCGGCGGAAACAGAGTTCAAAGAAGGGGCCTATGTTCCCGAAATCG

****
Consensus:

AGTGAACCGGCGGAAACAGAGTTCAAAGAAGGGGCCTATGTTCCCGAAATCG

>Bacillus_Fam_1304_52_1 Nr. of seq. 1 Alignment length(with gaps) = 52
Alignment score = 0.000000
GCF_001648575.1_ASM164857v1_genomic.fna_4:219533-220209 Satlength=677 Nr
of Repeats=13 RepeatLength=52 seed=GCGTGTCATA Num.seqs=11
Similarity=0.763636 0
GCGTGTCATAGAGCCCGAATGAAACGCGAANTCGCTGGGGCTTAGTGAAAAC

****
Consensus:

GCGTGTCATAGAGCCCGAATGAAACGCGAANTCGCTGGGGCTTAGTGAAAAC

>Bacillus_Fam_1305_52_1 Nr. of seq. 1 Alignment length(with gaps) = 52
Alignment score = 0.000000

GCF_001648575.1_ASM164857v1_genomic.fna_14:26277-26899 Satlength=623 Nr of Repeats=12 RepeatLength=52 seed=TGGAATTGTC Num.seqs=10 Similarity=0.947578 0
TGGAATTGTCATCATAGCAATCATGAAGACAATCTCGAGCGGATTTTGTGAG

****
Consensus:

TGGAATTGTCATCATAGCAATCATGAAGACAATCTCGAGCGGATTTTGTGAG

>Bacillus_Fam_1306_52_1 Nr. of seq. 1 Alignment length(with gaps) = 52 Alignment score = 0.000000
GCF_001648575.1_ASM164857v1_genomic.fna_47:0-207 Satlength=208 Nr of Repeats=4 RepeatLength=52 seed=CCTTATGAAG Num.seqs=4 Similarity=0.918803
0   CCTTATGAAGACACTTTCGGTCCACTTTCTCTCTCGGATGTGTCTTCATCCA

****
Consensus:

CCTTATGAAGACACTTTCGGTCCACTTTCTCTCTCGGATGTGTCTTCATCCA

>Bacillus_Fam_1307_52_1 Nr. of seq. 1 Alignment length(with gaps) = 52 Alignment score = 0.000000
GCF_001648575.1_ASM164857v1_genomic.fna_52:38204-38672 Satlength=469 Nr of Repeats=9 RepeatLength=52 seed=CGTTTTCCTT Num.seqs=9 Similarity=0.849003
0   CGTTTTCCTTGCTCGCTTCGAAGTTTGCGTGCCATTGCGCCTCTATGACACG

****
Consensus:

CGTTTTCCTTGCTCGCTTCGAAGTTTGCGTGCCATTGCGCCTCTATGACACG

>Bacillus_Fam_1308_52_1 Nr. of seq. 1 Alignment length(with gaps) = 52 Alignment score = 0.000000
GCF_001648575.1_ASM164857v1_genomic.fna_59:39775-40244 Satlength=470 Nr of Repeats=9 RepeatLength=52 seed=CGCGAATTTC Num.seqs=8 Similarity=0.870879
0   CGCGAATTTCCCTCCCGCTCCTAATTTGGTGTGTCATACTGCTCCTATGACG

****
Consensus:

CGCGAATTTCCCTCCCGCTCCTAATTTGGTGTGTCATACTGCTCCTATGACG

>Bacillus_Fam_1309_52_1 Nr. of seq. 1 Alignment length(with gaps) = 52 Alignment score = 0.000000
GCF_001648575.1_ASM164857v1_genomic.fna_60:94014-94277 Satlength=264 Nr of Repeats=5 RepeatLength=52 seed=TTTCCCTCTC Num.seqs=4 Similarity=0.935897
0   TTTCCCTCTCAACATTGTCTTCATCCACCTTGTGAAGACAGTACCATGCTTG

****
Consensus:

TTTCCCTCTCAACATTGTCTTCATCCACCTTGTGAAGACAGTACCATGCTTG

>Bacillus_Fam_1310_52_1 Nr. of seq. 1 Alignment length(with gaps) = 52 Alignment score = 0.000000 GCF_001648575.1_ASM164857v1_genomic.fna_63:225987-226623 Satlength=637 Nr of Repeats=12 RepeatLength=52 seed=AGATTGTCTT Num.seqs=10 Similarity=0.777239 0
AGATTGTCTTCATCACNCTTATAATGACAATCTTGAACTGATTTTCACTGTG

****
Consensus:

AGATTGTCTTCATCACNCTTATAATGACAATCTTGAACTGATTTTCACTGTG

>Bacillus_Fam_1311_52_1 Nr. of seq. 1 Alignment length(with gaps) = 52 Alignment score = 0.000000 GCF_001648575.1_ASM164857v1_genomic.fna_66:89204-89675 Satlength=472 Nr of Repeats=9 RepeatLength=52 seed=AAGACACTTT Num.seqs=6 Similarity=0.924786
0 AAGACACTTTTGCCGTGTTTTTCCTGCTGACATTGTCTTCAGACCACTGATG

****
Consensus:

AAGACACTTTTGCCGTGTTTTTCCTGCTGACATTGTCTTCAGACCACTGATG

>Bacillus_Fam_1312_52_1 Nr. of seq. 1 Alignment length(with gaps) = 52 Alignment score = 0.000000 GCF_001712755.1_ASM171275v1_genomic.fna_37:69494-69702 Satlength=209 Nr of Repeats=4 RepeatLength=52 seed=CTCATGAACC Num.seqs=4 Similarity=0.777778
0 CTCATGAACCGCTTATGAGTAAGTAAAATACATGATTTAGATTATTTAGTTA

****
Consensus:

CTCATGAACCGCTTATGAGTAAGTAAAATACATGATTTAGATTATTTAGTTA

>Bacillus_Fam_1313_52_1 Nr. of seq. 1 Alignment length(with gaps) = 52 Alignment score = 0.000000 GCF_001887185.1_ASM188718v1_genomic.fna_148:402146-402406 Satlength=261 Nr of Repeats=5 RepeatLength=52 seed=AATGTGTCTC Num.seqs=5 Similarity=0.792121 0
AATGTGTCTCAAGAATAGGGTTGGAAACAGATAAACCGGGAATNNCGTTGAA

****
Consensus:

AATGTGTCTCAAGAATAGGGTTGGAAACAGATAAACCGGGAATNNCGTTGAA

>Bacillus_Fam_1314_52_1 Nr. of seq. 1 Alignment length(with gaps) = 52 Alignment score = 0.000000

GCF_001887185.1_ASM188718v1_genomic.fna_192:110193-111153 Satlength=961 Nr of Repeats=14 RepeatLength=52 seed=GGTAACCTTT Num.seqs=9 Similarity=0.749288 0
GGTAACCTTTCCGGGGCTTTGGTTACCCGGATCTCATCACAGAGCCNACTTC

****
Consensus:

GGTAACCTTTCCGGGGCTTTGGTTACCCGGATCTCATCACAGAGCCNACTTC

>Bacillus_Fam_1315_52_1 Nr. of seq. 1 Alignment length(with gaps) = 52 Alignment score = 0.000000
GCF_001887185.1_ASM188718v1_genomic.fna_276:156901-157109 Satlength=209 Nr of Repeats=4 RepeatLength=52 seed=TCTTCAAGGT Num.seqs=4 Similarity=0.846154 0
TCTTCAAGGTAATCAAAAGCTCACAAAGGTACCCGAGCCGCCGGAATTACCT

****
Consensus:

TCTTCAAGGTAATCAAAAGCTCACAAAGGTACCCGAGCCGCCGGAATTACCT

>Bacillus_Fam_1316_52_1 Nr. of seq. 1 Alignment length(with gaps) = 52 Alignment score = 0.000000
GCF_001887185.1_ASM188718v1_genomic.fna_428:41505-41868 Satlength=364 Nr of Repeats=7 RepeatLength=52 seed=TGGAGACACA Num.seqs=6 Similarity=0.683761
0 TGGAGACACATTTTATTGCTAAAAAGTAAATAATGTGTTTCAGAACCAGNGC

****
Consensus:

TGGAGACACATTTTATTGCTAAAAAGTAAATAATGTGTTTCAGAACCAGNGC

>Bacillus_Fam_1317_52_1 Nr. of seq. 1 Alignment length(with gaps) = 52 Alignment score = 0.000000
GCF_002009555.1_ASM200955v1_genomic.fna_79:0-258 Satlength=259 Nr of Repeats=5 RepeatLength=52 seed=TAAGACACAT Num.seqs=4 Similarity=0.709402
0 TAAGACACATAATGTGACCAAACCCCAAAAAATGTGTCTCACCCGCCAGTCT

****
Consensus:

TAAGACACATAATGTGACCAAACCCCAAAAAATGTGTCTCACCCGCCAGTCT

>Bacillus_Fam_1318_52_1 Nr. of seq. 1 Alignment length(with gaps) = 52 Alignment score = 0.000000
GCF_002019605.1_ASM201960v1_genomic.fna_1:2881499-2881761 Satlength=263 Nr of Repeats=5 RepeatLength=52 seed=CGTTGTTTCG Num.seqs=3 Similarity=0.825996 0
CGTTGTTTCGGGCACTATGAACCCTCTATAGTTCCCAGTCTCTTGCTTCCAT

****
Consensus:

CGTTGTTTCGGGCACTATGAACCCTCTATAGTTCCCAGTCTCTTGCTTCCAT

>Bacillus_Fam_1319_52_1 Nr. of seq. 1 Alignment length(with gaps) = 52
Alignment score = 0.000000
GCF_002019605.1_ASM201960v1_genomic.fna_1:3984598-3985376 Satlength=779
Nr of Repeats=15 RepeatLength=52 seed=CCGTTTGAGC Num.seqs=13
Similarity=0.911900 0
CCGTTTGAGCAGCTCGAAGGAGATAGCGGTCACTATGAAAGCGTCATGGTGA

****
Consensus:

CCGTTTGAGCAGCTCGAAGGAGATAGCGGTCACTATGAAAGCGTCATGGTGA

>Bacillus_Fam_1320_52_1 Nr. of seq. 1 Alignment length(with gaps) = 52
Alignment score = 0.000000
GCF_002019645.1_ASM201964v1_genomic.fna_1:123004-123368 Satlength=365 Nr
of Repeats=7 RepeatLength=52 seed=ACTTCAGACA Num.seqs=7
Similarity=0.857143
0   ACTTCAGACAACTTCTGCNGCCAATTACTTAAACCTGTCCGAACTCTTGCCT

****
Consensus:

ACTTCAGACAACTTCTGCNGCCAATTACTTAAACCTGTCCGAACTCTTGCCT

>Bacillus_Fam_1321_52_1 Nr. of seq. 1 Alignment length(with gaps) = 52
Alignment score = 0.000000
GCF_002019645.1_ASM201964v1_genomic.fna_1:208479-208999 Satlength=521 Nr
of Repeats=10 RepeatLength=52 seed=GAGCAACTTG Num.seqs=8
Similarity=0.878205 0
GAGCAACTTGATCATAGACCTCCTATGATTCACTTCCCTACAATTCATGTGC

****
Consensus:

GAGCAACTTGATCATAGACCTCCTATGATTCACTTCCCTACAATTCATGTGC

>Bacillus_Fam_1322_52_1 Nr. of seq. 1 Alignment length(with gaps) = 52
Alignment score = 0.000000
GCF_002019645.1_ASM201964v1_genomic.fna_1:1309331-1310008 Satlength=678
Nr of Repeats=13 RepeatLength=52 seed=TGTCTGAAGT Num.seqs=12
Similarity=0.897824 0
TGTCTGAAGTTGAGNAGCCTTCGGACAGGTTAGAAGCCTACAGAGTAAAAGC

****
Consensus:

TGTCTGAAGTTGAGNAGCCTTCGGACAGGTTAGAAGCCTACAGAGTAAAAGC

>Bacillus_Fam_1323_52_1 Nr. of seq. 1 Alignment length(with gaps) = 52
Alignment score = 0.000000

GCF_002019645.1_ASM201964v1_genomic.fna_1:1511589-1512577 Satlength=989
Nr of Repeats=19 RepeatLength=52 seed=AAGCTGTCCG Num.seqs=19
Similarity=0.906175 0
AAGCTGTCCGAAGAGAGCTCAGGTTCAGACAGGTTTGCTCAGGTTNAGTAGA

****
Consensus:

AAGCTGTCCGAAGAGAGCTCAGGTTCAGACAGGTTTGCTCAGGTTNAGTAGA

>Bacillus_Fam_1324_52_1 Nr. of seq. 1 Alignment length(with gaps) = 52
Alignment score = 0.000000
GCF_002019645.1_ASM201964v1_genomic.fna_1:2149247-2150387 Satlength=1141
Nr of Repeats=22 RepeatLength=52 seed=TCTGATTCCC Num.seqs=18
Similarity=0.902296 0
TCTGATTCCCTTTCAGTAGTGNATCGGGGTAGAAAGGGAACGAAAGAGCAAG

****
Consensus:

TCTGATTCCCTTTCAGTAGTGNATCGGGGTAGAAAGGGAACGAAAGAGCAAG

>Bacillus_Fam_1325_52_1 Nr. of seq. 1 Alignment length(with gaps) = 52
Alignment score = 0.000000
GCF_002019645.1_ASM201964v1_genomic.fna_1:2283102-2283310 Satlength=209
Nr of Repeats=4 RepeatLength=52 seed=AGATTCCCTT Num.seqs=4
Similarity=0.893162 0
AGATTCCCTTACTAGGCAAAAATGAAGTGCAAAGGGAACGAAAGGTAGTCAC

****
Consensus:

AGATTCCCTTACTAGGCAAAAATGAAGTGCAAAGGGAACGAAAGGTAGTCAC

>Bacillus_Fam_1326_52_1 Nr. of seq. 1 Alignment length(with gaps) = 52
Alignment score = 0.000000
GCF_002019645.1_ASM201964v1_genomic.fna_1:3845277-3845745 Satlength=469
Nr of Repeats=9 RepeatLength=52 seed=GAAAGCTGTC Num.seqs=9
Similarity=0.951567 0
GAAAGCTGTCCGAACTTCACTTGGGTTCGGACTACTTTTCCTTTACGACCAC

****
Consensus:

GAAAGCTGTCCGAACTTCACTTGGGTTCGGACTACTTTTCCTTTACGACCAC

>Bacillus_Fam_1327_52_1 Nr. of seq. 1 Alignment length(with gaps) = 52
Alignment score = 0.000000
GCF_002019645.1_ASM201964v1_genomic.fna_1:4572662-4573184 Satlength=523
Nr of Repeats=10 RepeatLength=52 seed=ATTCCCTTTG Num.seqs=8
Similarity=0.928571 0
ATTCCCTTTGTCGGAGAAAATACACAGGAAAGGGAACGAAAGGAGCGCTTTG

****

Consensus:

ATTCCCTTTGTCGGAGAAAATACACAGGAAAGGGAACGAAAGGAGCGCTTTG

>Bacillus_Fam_1328_52_1 Nr. of seq. 1 Alignment length(with gaps) = 52
Alignment score = 0.000000
GCF_002019645.1_ASM201964v1_genomic.fna_1:5404130-5406159 Satlength=2030
Nr of Repeats=39 RepeatLength=52 seed=AAGCTGTCTG Num.seqs=38
Similarity=0.813432 0
AAGCTGTCTGAACCTACCCCACCTTCGGACAGCTTGACTCCGAAAAACCCAA

****
Consensus:

AAGCTGTCTGAACCTACCCCACCTTCGGACAGCTTGACTCCGAAAAACCCAA

>Bacillus_Fam_1329_52_1 Nr. of seq. 1 Alignment length(with gaps) = 52
Alignment score = 0.000000
GCF_002019665.1_ASM201966v1_genomic.fna_1:2317880-2318296 Satlength=417
Nr of Repeats=8 RepeatLength=52 seed=ATTATGGGTA Num.seqs=8
Similarity=0.907509 0
ATTATGGGTAACGATAAAGGCGTGATCGTGCCCTGAACTCGAGAATATAGTG

****
Consensus:

ATTATGGGTAACGATAAAGGCGTGATCGTGCCCTGAACTCGAGAATATAGTG

>Bacillus_Fam_1330_52_1 Nr. of seq. 1 Alignment length(with gaps) = 52
Alignment score = 0.000000
GCF_900104555.1_IMG-
taxon_2651870172_annotated_assembly_genomic.fna_14:10803-11065
Satlength=263 Nr of Repeats=5 RepeatLength=52 seed=GAGTCTGAAC Num.seqs=3
Similarity=0.709402 0
GAGTCTGAACCTNGCGTGAGTTCGGACAGGAATCGTGANTGTGAATGTAAGC

****
Consensus:

GAGTCTGAACCTNGCGTGAGTTCGGACAGGAATCGTGANTGTGAATGTAAGC

>Bacillus_Fam_1331_52_1 Nr. of seq. 1 Alignment length(with gaps) = 52
Alignment score = 0.000000
GCF_900104555.1_IMG-
taxon_2651870172_annotated_assembly_genomic.fna_16:230959-231479
Satlength=521 Nr of Repeats=10 RepeatLength=52 seed=TGTCCAATAG
Num.seqs=10 Similarity=0.801709 0
TGTCCAATAGGATGCCTCTATCGGACATAACTCACTTCCCAAGACTAGATTC

****
Consensus:

TGTCCAATAGGATGCCTCTATCGGACATAACTCACTTCCCAAGACTAGATTC

>Bacillus_Fam_1332_52_1 Nr. of seq. 1 Alignment length(with gaps) = 52
Alignment score = 0.000000
GCF_900104555.1_IMG-
taxon_2651870172_annotated_assembly_genomic.fna_19:76324-76687
Satlength=364 Nr of Repeats=7 RepeatLength=52 seed=ACGGCTCTAT Num.seqs=6
Similarity=0.829060   0
ACGGCTCTATCGAACAAAACTATGGGCCTTCACGCTTGGTTTGTCCAATAGG

****
Consensus:

ACGGCTCTATCGAACAAAACTATGGGCCTTCACGCTTGGTTTGTCCAATAGG

>Bacillus_Fam_1333_52_1 Nr. of seq. 1 Alignment length(with gaps) = 52
Alignment score = 0.000000
GCF_900104555.1_IMG-
taxon_2651870172_annotated_assembly_genomic.fna_22:326079-326390
Satlength=312 Nr of Repeats=6 RepeatLength=52 seed=CTATTGGACA Num.seqs=5
Similarity=0.933333   0
CTATTGGACAAAATCGCTTCACGGCACTGGCGGTTTGTCTAATACAGGCTTC

****
Consensus:

CTATTGGACAAAATCGCTTCACGGCACTGGCGGTTTGTCTAATACAGGCTTC

>Bacillus_Fam_1334_52_1 Nr. of seq. 1 Alignment length(with gaps) = 52
Alignment score = 0.000000
GCF_900104555.1_IMG-
taxon_2651870172_annotated_assembly_genomic.fna_22:581244-581504
Satlength=261 Nr of Repeats=5 RepeatLength=52 seed=ATCAGAGACC Num.seqs=5
Similarity=0.804938   0
ATCAGAGACCTTTGGACAGCTTCTTCGNTTGATATGGTCATTGACACCTCTC

****
Consensus:

ATCAGAGACCTTTGGACAGCTTCTTCGNTTGATATGGTCATTGACACCTCTC

>Bacillus_Fam_1335_52_1 Nr. of seq. 1 Alignment length(with gaps) = 52
Alignment score = 0.000000
GCF_900104555.1_IMG-
taxon_2651870172_annotated_assembly_genomic.fna_24:146667-146929
Satlength=263 Nr of Repeats=5 RepeatLength=52 seed=TTTTGTCCGA Num.seqs=4
Similarity=0.935897   0
TTTTGTCCGATAGAGACCTCTTATTGGACAAAACCTTTGCTGGCACCGTTCG

****
Consensus:

TTTTGTCCGATAGAGACCTCTTATTGGACAAAACCTTTGCTGGCACCGTTCG

>Bacillus_Fam_1336_52_1 Nr. of seq. 1 Alignment length(with gaps) = 52
Alignment score = 0.000000

GCF_900104555.1_IMG-
taxon_2651870172_annotated_assembly_genomic.fna_27:4426-4682
Satlength=257 Nr of Repeats=5 RepeatLength=51 seed=ATAAGTGCCC Num.seqs=4
Similarity=0.786325 0
ATAAGTGCCCGTTCTAGGGTCCCTAGCAAGGAGAGCGGTCATCTAACTCGAG

****
Consensus:

ATAAGTGCCCGTTCTAGGGTCCCTAGCAAGGAGAGCGGTCATCTAACTCGAG

>Bacillus_Fam_1337_52_1 Nr. of seq. 1 Alignment length(with gaps) = 52
Alignment score = 0.000000
GCF_900104555.1_IMG-
taxon_2651870172_annotated_assembly_genomic.fna_27:9518-9884
Satlength=367 Nr of Repeats=7 RepeatLength=52 seed=ACTTCAGACA Num.seqs=5
Similarity=0.761538 0
ACTTCAGACAGAAGAAGGTAAAAGAAGTAAGGGAGAGTCTGAANTNGAAGTG

****
Consensus:

ACTTCAGACAGAAGAAGGTAAAAGAAGTAAGGGAGAGTCTGAANTNGAAGTG

>Bacillus_Fam_1338_52_1 Nr. of seq. 1 Alignment length(with gaps) = 52
Alignment score = 0.000000
GCF_900104555.1_IMG-
taxon_2651870172_annotated_assembly_genomic.fna_32:21776-21984
Satlength=209 Nr of Repeats=4 RepeatLength=52 seed=TTCTATTGGA Num.seqs=4
Similarity=0.876068 0
TTCTATTGGACATTCTCATCGATACGAACGCACGTTTTGTCCAATAGACGCC

****
Consensus:

TTCTATTGGACATTCTCATCGATACGAACGCACGTTTTGTCCAATAGACGCC

>Bacillus_Fam_1339_52_1 Nr. of seq. 1 Alignment length(with gaps) = 52
Alignment score = 0.000000
GCF_900107275.1_IMG-
taxon_2675903024_annotated_assembly_genomic.fna_18:52517-53042
Satlength=526 Nr of Repeats=5 RepeatLength=52 seed=AAGAGCAAAT Num.seqs=4
Similarity=0.927350 0
AAGAGCAAATCGTTGACGAAACGAGCATTTTGAAAGCTGAACGTCAACGAAT

****
Consensus:

AAGAGCAAATCGTTGACGAAACGAGCATTTTGAAAGCTGAACGTCAACGAAT

>Bacillus_Fam_1340_52_1 Nr. of seq. 1 Alignment length(with gaps) = 52
Alignment score = 0.000000
GCF_900109925.1_IMG-
taxon_2651870116_annotated_assembly_genomic.fna_16:495010-495218

Satlength=209 Nr of Repeats=4 RepeatLength=52 seed=TCGTTACCGT Num.seqs=4
Similarity=0.865226   0
TCGTTACCGTTTTTGGCGTACTCTTCTGTTTTCGGGCACATTAAACTCTTTN

****
Consensus:

TCGTTACCGTTTTTGGCGTACTCTTCTGTTTTCGGGCACATTAAACTCTTTN

>Bacillus_Fam_1341_52_1 Nr. of seq. 1 Alignment length(with gaps) = 52
Alignment score = 0.000000
GCF_900109925.1_IMG-
taxon_2651870116_annotated_assembly_genomic.fna_17:42538-42746
Satlength=209 Nr of Repeats=4 RepeatLength=52 seed=CACCAAGAGC Num.seqs=4
Similarity=0.871795   0
CACCAAGAGCTTTCCTTGGCGCAGTAATATAGGTTTCATCTACTAATAACTG

****
Consensus:

CACCAAGAGCTTTCCTTGGCGCAGTAATATAGGTTTCATCTACTAATAACTG

>Bacillus_Fam_1342_52_1 Nr. of seq. 1 Alignment length(with gaps) = 52
Alignment score = 0.000000
GCF_900156865.1_PRJEB18960_genomic.fna_3:1290871-1291391 Satlength=521 Nr of Repeats=10 RepeatLength=52 seed=GGTCTTCATA Num.seqs=10
Similarity=0.778917   0
GGTCTTCATAGCACCGATGAAGACCTGAAACAGTTGGAAACTGCTTCGTACA

****
Consensus:

GGTCTTCATAGCACCGATGAAGACCTGAAACAGTTGGAAACTGCTTCGTACA

>Bacillus_Fam_1343_52_1 Nr. of seq. 1 Alignment length(with gaps) = 52
Alignment score = 0.000000
GCF_900156865.1_PRJEB18960_genomic.fna_3:2588049-2588340 Satlength=292 Nr of Repeats=6 RepeatLength=52 seed=CTCTTCATCT Num.seqs=4
Similarity=0.809748   0
CTCTTCATCTCGCTTATGAAGGGGATTTGGATGAGGTAACTGCCTTGTTTTC

****
Consensus:

CTCTTCATCTCGCTTATGAAGGGGATTTGGATGAGGTAACTGCCTTGTTTTC

>Bacillus_Fam_1344_52_1 Nr. of seq. 1 Alignment length(with gaps) = 52
Alignment score = 0.000000
GCF_900156865.1_PRJEB18960_genomic.fna_3:4108755-4109589 Satlength=835 Nr of Repeats=16 RepeatLength=52 seed=ATGTCGTCAT Num.seqs=14
Similarity=0.767535   0
ATGTCGTCATGAGTGCGATGAAGACACAAACTCTCCTGGCGAGCGGAAGAAG

****

Consensus:

ATGTCGTCATGAGTGCGATGAAGACACAAACTCTCCTGGCGAGCGGAAGAAG

>Bacillus_Fam_1345_52_1 Nr. of seq. 1 Alignment length(with gaps) = 52
Alignment score = 0.000000
GCF_900156865.1_PRJEB18960_genomic.fna_3:4353231-4353439 Satlength=209 Nr of Repeats=4 RepeatLength=52 seed=GGAAAACACA Num.seqs=4
Similarity=0.897436 0
GGAAAACACAGCAAAAGCCGGATAGAAATCCCCTTCATCAAGGCCATGAAGG

****
Consensus:

GGAAAACACAGCAAAAGCCGGATAGAAATCCCCTTCATCAAGGCCATGAAGG

>Bacillus_Fam_1346_52_1 Nr. of seq. 1 Alignment length(with gaps) = 52
Alignment score = 0.000000
GCF_900156875.1_PRJEB18969_genomic.fna_10:153192-153608 Satlength=417 Nr of Repeats=8 RepeatLength=52 seed=GATGAAGACC Num.seqs=8
Similarity=0.677402
0   GATGAAGACCGTTTAAGGATCCCTCCGNGCCAGAAAAGGTTATCATGGGAGA

****
Consensus:

GATGAAGACCGTTTAAGGATCCCTCCGNGCCAGAAAAGGTTATCATGGGAGA

>Bacillus_Fam_1347_52_1 Nr. of seq. 1 Alignment length(with gaps) = 52
Alignment score = 0.000000
GCF_900156875.1_PRJEB18969_genomic.fna_10:1493463-1493980 Satlength=518 Nr of Repeats=10 RepeatLength=52 seed=ATCTATAAAA Num.seqs=9
Similarity=0.819801 0
ATCTATAAAAATGGTCATCATGTACGTGATGAAAACCGAAATCTGGTATGTG

****
Consensus:

ATCTATAAAAATGGTCATCATGTACGTGATGAAAACCGAAATCTGGTATGTG

>Bacillus_Fam_1348_52_1 Nr. of seq. 1 Alignment length(with gaps) = 52
Alignment score = 0.000000
GCF_900156875.1_PRJEB18969_genomic.fna_11:52363-52884 Satlength=522 Nr of Repeats=10 RepeatLength=52 seed=CGGTCTTCAT Num.seqs=7
Similarity=0.677249
0   CGGTCTTCATGGCCGGGATGAGAACCGATNTTNCTGATTTCTTTGCGAGAAC

****
Consensus:

CGGTCTTCATGGCCGGGATGAGAACCGATNTTNCTGATTTCTTTGCGAGAAC

>Bacillus_Fam_1349_52_1 Nr. of seq. 1 Alignment length(with gaps) = 52
Alignment score = 0.000000

GCF_900156875.1_PRJEB18969_genomic.fna_11:147252-147512 Satlength=261 Nr of Repeats=5 RepeatLength=52 seed=TGGTTTTCAT Num.seqs=5 Similarity=0.902564
0 TGGTTTTCATAAGCGGAATGAAGACCGTTATTAGGGGCTTTGTCCTGCGTAA

****
Consensus:

TGGTTTTCATAAGCGGAATGAAGACCGTTATTAGGGGCTTTGTCCTGCGTAA

>Bacillus_Fam_1350_52_1 Nr. of seq. 1 Alignment length(with gaps) = 52 Alignment score = 0.000000
GCF_900156875.1_PRJEB18969_genomic.fna_11:295252-297644 Satlength=2393 Nr of Repeats=46 RepeatLength=52 seed=GATGAAGACC Num.seqs=44 Similarity=0.826120 0
GATGAAGACCAATCCATNGGAAGGAGCGCGGGNAAACGGTCATCATCAAGGG

****
Consensus:

GATGAAGACCAATCCATNGGAAGGAGCGCGGGNAAACGGTCATCATCAAGGG

>Bacillus_Fam_1351_52_1 Nr. of seq. 1 Alignment length(with gaps) = 52 Alignment score = 0.000000
GCF_900156875.1_PRJEB18969_genomic.fna_11:372186-372602 Satlength=417 Nr of Repeats=8 RepeatLength=52 seed=GGTTTTCATA Num.seqs=8 Similarity=0.819632
0 GGTTTTCATAGCTGGAATGAAGACCGAAAGTAACGGTGATAGATCGAGTTTA

****
Consensus:

GGTTTTCATAGCTGGAATGAAGACCGAAAGTAACGGTGATAGATCGAGTTTA

>Bacillus_Fam_1352_52_1 Nr. of seq. 1 Alignment length(with gaps) = 52 Alignment score = 0.000000
GCF_900156875.1_PRJEB18969_genomic.fna_11:939998-946134 Satlength=6137 Nr of Repeats=118 RepeatLength=52 seed=TGAGTCTGAA Num.seqs=118 Similarity=0.789356 0
TGAGTCTGAATCCGAAGGTTATTCGGACTCCAGCAAGAGGAAACCCAGGCAT

****
Consensus:

TGAGTCTGAATCCGAAGGTTATTCGGACTCCAGCAAGAGGAAACCCAGGCAT

>Bacillus_Fam_1353_52_1 Nr. of seq. 1 Alignment length(with gaps) = 52 Alignment score = 0.000000
GCF_900156875.1_PRJEB18969_genomic.fna_11:1045732-1051088 Satlength=5357 Nr of Repeats=101 RepeatLength=52 seed=TCTTCATAAA Num.seqs=100 Similarity=0.741715 0
TCTTCATAAAGGCAATGAAGACCGTTTGGTTGGTTTTTTANAANGNCTTTGG

****

Consensus:

TCTTCATAAAGGCAATGAAGACCGTTTGGTTGGTTTTTTANAANGNCTTTGG

>Bacillus_Fam_1354_52_1 Nr. of seq. 1 Alignment length(with gaps) = 52
Alignment score = 0.000000
GCF_900156875.1_PRJEB18969_genomic.fna_11:1191426-1193454 Satlength=2029
Nr of Repeats=39 RepeatLength=52 seed=TTGGTTTTCA Num.seqs=39
Similarity=0.908371 0
TTGGTTTTCATAACGGCGATGATGACCGTTTTGCTGAGTGCTGGCTTGCTTT

****
Consensus:

TTGGTTTTCATAACGGCGATGATGACCGTTTTGCTGAGTGCTGGCTTGCTTT

>Bacillus_Fam_1355_52_1 Nr. of seq. 1 Alignment length(with gaps) = 52
Alignment score = 0.000000
GCF_900156875.1_PRJEB18969_genomic.fna_11:1294995-1295515 Satlength=521
Nr of Repeats=10 RepeatLength=52 seed=TAAAACTTCA Num.seqs=10
Similarity=0.839316 0
TAAAACTTCATGCTTATGGTCCTCATTCAACGGATGATGACCGTTTGCCTTA

****
Consensus:

TAAAACTTCATGCTTATGGTCCTCATTCAACGGATGATGACCGTTTGCCTTA

>Bacillus_Fam_1356_52_1 Nr. of seq. 1 Alignment length(with gaps) = 52
Alignment score = 0.000000
GCF_900156875.1_PRJEB18969_genomic.fna_11:1698345-1698605 Satlength=261
Nr of Repeats=5 RepeatLength=52 seed=ATGATGACCG Num.seqs=5
Similarity=0.640606 0
ATGATGACCGTTNTTGGTTCATAGAACANAGAAACTGGTCTTCATCTGGGCT

****
Consensus:

ATGATGACCGTTNTTGGTTCATAGAACANAGAAACTGGTCTTCATCTGGGCT

>Bacillus_Fam_1357_51_1 Nr. of seq. 1 Alignment length(with gaps) = 51
Alignment score = 0.000000
BACILLUS_PSEUDALCALIPHILUS_genomic.fna_1:64392-64647 Satlength=256 Nr of
Repeats=5 RepeatLength=51 seed=AATACGCAGC Num.seqs=5 Similarity=0.742138
0 AATACGCAGCAATGAAACCGNAAAAATAGTCCTCAATGCCGCACAAATAAA

****
Consensus:

AATACGCAGCAATGAAACCGNAAAAATAGTCCTCAATGCCGCACAAATAAA

>Bacillus_Fam_1358_51_1 Nr. of seq. 1 Alignment length(with gaps) = 51
Alignment score = 0.000000

BACILLUS_PSEUDALCALIPHILUS_genomic.fna_5:600241-600496 Satlength=256 Nr of Repeats=5 RepeatLength=51 seed=GGGGTTACTC Num.seqs=5 Similarity=0.823077
0 GGGGTTACTCATGAGAAAAGTGAGAGTAAGCCCGAGAAAAGAGAAATGCNC

****
Consensus:

GGGGTTACTCATGAGAAAAGTGAGAGTAAGCCCGAGAAAAGAGAAATGCNC

>Bacillus_Fam_1359_51_1 Nr. of seq. 1 Alignment length(with gaps) = 51 Alignment score = 0.000000
BACILLUS_PSEUDALCALIPHILUS_genomic.fna_6:120207-120411 Satlength=205 Nr of Repeats=4 RepeatLength=51 seed=CGTTTTCTCG Num.seqs=4 Similarity=0.801816
2 ATCGTTTTCTCGGGCTTTCTACTCTTTTTTCCGCCCGTAACCCCAAGCATC

****
Consensus:

CGTTTTCTCGGGCTTTCTACTCTTTTTTCCGCCCGTAACCCCAAGCATCAT

>Bacillus_Fam_1360_51_1 Nr. of seq. 1 Alignment length(with gaps) = 51 Alignment score = 0.000000
GCF_000161455.1_ASM16145v1_genomic.fna_1:4028285-4028540 Satlength=256 Nr of Repeats=5 RepeatLength=51 seed=CTAAACAAGA Num.seqs=5 Similarity=0.874510 0
CTAAACAAGACGAGCAAACACAAAACAAACAACATCAATCTGCTCAAGGTA

****
Consensus:

CTAAACAAGACGAGCAAACACAAAACAAACAACATCAATCTGCTCAAGGTA

>Bacillus_Fam_1361_51_1 Nr. of seq. 1 Alignment length(with gaps) = 51 Alignment score = 0.000000
GCF_000169195.2_ASM16919v2_genomic.fna_1:2424628-2425463 Satlength=836 Nr of Repeats=16 RepeatLength=52 seed=AAAGACAAAA Num.seqs=14 Similarity=0.612441 0
AAAGACAAAACGNGGNGCCGAGACGAAAGAAATGTCCTTCATAGGGTCATG

****
Consensus:

AAAGACAAAACGNGGNGCCGAGACGAAAGAAATGTCCTTCATAGGGTCATG

>Bacillus_Fam_1362_51_1 Nr. of seq. 1 Alignment length(with gaps) = 51 Alignment score = 0.000000
GCF_000175075.1_ASM17507v1_genomic.fna_33:209101-209305 Satlength=205 Nr of Repeats=4 RepeatLength=51 seed=TGAATGAGGA Num.seqs=4 Similarity=0.869281
0 TGAATGAGGACAAAGTAAGCGGGAAAACAGAAACATTGTCCTCGTAGGGCG

****
Consensus:

TGAATGAGGACAAAGTAAGCGGGAAAACAGAAACATTGTCCTCGTAGGGCG

>Bacillus_Fam_1363_51_1 Nr. of seq. 1 Alignment length(with gaps) = 51 Alignment score = 0.000000
GCF_000175075.1_ASM17507v1_genomic.fna_46:49896-50202 Satlength=307 Nr of Repeats=6 RepeatLength=51 seed=GATGACAAAA Num.seqs=6 Similarity=0.803050
0 GATGACAAAACTTCAATGAACNAAGGATTTCTGTCATCGGAAAGCTTCAAC

****
Consensus:

GATGACAAAACTTCAATGAACNAAGGATTTCTGTCATCGGAAAGCTTCAAC

>Bacillus_Fam_1364_51_1 Nr. of seq. 1 Alignment length(with gaps) = 51 Alignment score = 0.000000
GCF_000181495.1_ASM18149v1_genomic.fna_129:1421-1931 Satlength=511 Nr of Repeats=10 RepeatLength=51 seed=TAAAGGACGT Num.seqs=10 Similarity=0.755410
0 TAAAGGACGTTAACGAGTCAATTTAGAGACTAAAGTCCTATAAACAGCTTN

****
Consensus:

TAAAGGACGTTAACGAGTCAATTTAGAGACTAAAGTCCTATAAACAGCTTN

>Bacillus_Fam_1365_51_1 Nr. of seq. 1 Alignment length(with gaps) = 51 Alignment score = 0.000000
GCF_000181495.1_ASM18149v1_genomic.fna_164:12630-13239 Satlength=610 Nr of Repeats=12 RepeatLength=51 seed=TATCGTCCTA Num.seqs=11 Similarity=0.749457 0
TATCGTCCTATAGAGGGACTTTAAAGGACTTTAATATTGAATGTTGCNAAA

****
Consensus:

TATCGTCCTATAGAGGGACTTTAAAGGACTTTAATATTGAATGTTGCNAAA

>Bacillus_Fam_1366_51_1 Nr. of seq. 1 Alignment length(with gaps) = 51 Alignment score = 0.000000
GCF_000242895.2_ASM24289v3_genomic.fna_1:1809966-1810272 Satlength=307 Nr of Repeats=6 RepeatLength=51 seed=GTGACCAAAA Num.seqs=6 Similarity=0.907625 0
GTGACCAAAAAGAAAAAAAGAGAGCTACTTCGGTCACTCCAGAGAATTGGA

****
Consensus:

GTGACCAAAAAGAAAAAAAGAGAGCTACTTCGGTCACTCCAGAGAATTGGA

>Bacillus_Fam_1367_51_1 Nr. of seq. 1 Alignment length(with gaps) = 51 Alignment score = 0.000000

GCF_000245315.1_ASM24531v1_genomic.fna_55:29067-29424 Satlength=358 Nr of Repeats=7 RepeatLength=51 seed=GCACGCTGAG Num.seqs=7 Similarity=0.886710
0   GCACGCTGAGCCNGCGCTTCGTCAAGAAGAGAAAGCAGCGGCTCAACCGGA

****
Consensus:

GCACGCTGAGCCNGCGCTTCGTCAAGAAGAGAAAGCAGCGGCTCAACCGGA

>Bacillus_Fam_1368_51_1 Nr. of seq. 1 Alignment length(with gaps) = 51 Alignment score = 0.000000
GCF_000292245.2_ASM29224v2_genomic.fna_7:20889-21092 Satlength=204 Nr of Repeats=4 RepeatLength=51 seed=CTTATCCTCC Num.seqs=3 Similarity=0.773420
0   CTTATCCTCCGTTTTTCGTACCGTATTTTGGCCAGGGAGAATTTTTCATTC

****
Consensus:

CTTATCCTCCGTTTTTCGTACCGTATTTTGGCCAGGGAGAATTTTTCATTC

>Bacillus_Fam_1369_51_1 Nr. of seq. 1 Alignment length(with gaps) = 51 Alignment score = 0.000000
GCF_000292245.2_ASM29224v2_genomic.fna_27:36252-36558 Satlength=307 Nr of Repeats=6 RepeatLength=51 seed=GAAAAAGAAG Num.seqs=6 Similarity=0.839651
0   GAAAAAGAAGCCGAGCTAGCGGAAGTACGGCGTATAAGGAGTCATGAAGTT

****
Consensus:

GAAAAAGAAGCCGAGCTAGCGGAAGTACGGCGTATAAGGAGTCATGAAGTT

>Bacillus_Fam_1370_51_1 Nr. of seq. 1 Alignment length(with gaps) = 51 Alignment score = 0.000000
GCF_000292245.2_ASM29224v2_genomic.fna_38:108-363 Satlength=256 Nr of Repeats=5 RepeatLength=51 seed=TTTAAAGGAC Num.seqs=5 Similarity=0.900654
0   TTTAAAGGACAAAAAAAGGAAGCCGAAGCTGGAAAGTGTCCAATAACGAGAG

****
Consensus:

TTTAAAGGACAAAAAAAGGAAGCCGAAGCTGGAAAGTGTCCAATAACGAGAG

>Bacillus_Fam_1371_51_1 Nr. of seq. 1 Alignment length(with gaps) = 51 Alignment score = 0.000000
GCF_000292245.2_ASM29224v2_genomic.fna_38:28715-29235 Satlength=521 Nr of Repeats=10 RepeatLength=52 seed=AGTGAAAAGG Num.seqs=10 Similarity=0.711111
0   AGTGAAAAGGGAATCAAAGCCCTCAATGATTCCCAAAGAGCCCCAGAACCA

****
Consensus:

AGTGAAAAGGGAATCAAAGCCCTCAATGATTCCCAAAGAGCCCCAGAACCA

>Bacillus_Fam_1372_51_1 Nr. of seq. 1 Alignment length(with gaps) = 51
Alignment score = 0.000000
GCF_000292245.2_ASM29224v2_genomic.fna_100:13250-13504 Satlength=255 Nr of Repeats=5 RepeatLength=51 seed=AATACGCCGA Num.seqs=4 Similarity=0.869281
0 AATACGCCGAACTTCCCTTTACTCGGCTCTTTTTTCTGCTTCACAGTTCTA

****
Consensus:

AATACGCCGAACTTCCCTTTACTCGGCTCTTTTTTCTGCTTCACAGTTCTA

>Bacillus_Fam_1373_51_1 Nr. of seq. 1 Alignment length(with gaps) = 51
Alignment score = 0.000000
GCF_000292245.2_ASM29224v2_genomic.fna_107:6328-6635 Satlength=308 Nr of Repeats=6 RepeatLength=51 seed=GTGCGTATTC Num.seqs=5 Similarity=0.958170
0 GTGCGTATTCATCTGATTTGGTAATCAAATTTCAACCTGAGCCGAATTACG

****
Consensus:

GTGCGTATTCATCTGATTTGGTAATCAAATTTCAACCTGAGCCGAATTACG

>Bacillus_Fam_1374_51_1 Nr. of seq. 1 Alignment length(with gaps) = 51
Alignment score = 0.000000
GCF_000292245.2_ASM29224v2_genomic.fna_122:8353-8558 Satlength=206 Nr of Repeats=4 RepeatLength=51 seed=TTTTCATTAC Num.seqs=3 Similarity=0.808279
0 TTTTCATTACCTATTATNGTTTATTCAAACCCTAATTGAGNGAGTTTCCCT

****
Consensus:

TTTTCATTACCTATTATNGTTTATTCAAACCCTAATTGAGNGAGTTTCCCT

>Bacillus_Fam_1375_51_1 Nr. of seq. 1 Alignment length(with gaps) = 51
Alignment score = 0.000000
GCF_000307875.1_BABA1.0_genomic.fna_34:35332-35689 Satlength=358 Nr of Repeats=7 RepeatLength=51 seed=TTGGTCTTCA Num.seqs=7 Similarity=0.875506
0 TTGGTCTTCATCCGCTTAATGAAGACCTTTTTTCCGATTTCGTTTACACTT

****
Consensus:

TTGGTCTTCATCCGCTTAATGAAGACCTTTTTTCCGATTTCGTTTACACTT

>Bacillus_Fam_1376_51_1 Nr. of seq. 1 Alignment length(with gaps) = 51
Alignment score = 0.000000
GCF_000307875.1_BABA1.0_genomic.fna_52:16517-17289 Satlength=773 Nr of Repeats=5 RepeatLength=51 seed=CGTTACCTTT Num.seqs=4 Similarity=0.747277
0 CGTTACCTTTTCTTCGTATTTTCCCATTTTTGGTCACATAGACCCCTTCTA

****
Consensus:

CGTTACCTTTTCTTCGTATTTTCCCATTTTTGGTCACATAGACCCCTTCTA

>Bacillus_Fam_1377_51_1 Nr. of seq. 1 Alignment length(with gaps) = 51 Alignment score = 0.000000 GCF_000307875.1_BABA1.0_genomic.fna_121:73699-73903 Satlength=205 Nr of Repeats=4 RepeatLength=51 seed=GTCACGTAGA Num.seqs=4 Similarity=0.873638
0   GTCACGTAGAACCTCTCTACGTGACCTCTCCCATGCTTTTTCTCCAAAATA

****
Consensus:

GTCACGTAGAACCTCTCTACGTGACCTCTCCCATGCTTTTTCTCCAAAATA

>Bacillus_Fam_1378_51_1 Nr. of seq. 1 Alignment length(with gaps) = 51 Alignment score = 0.000000 GCF_000331575.1_ASM33157v1_genomic.fna_1:1364253-1364865 Satlength=613 Nr of Repeats=12 RepeatLength=51 seed=CGCTTTTATC Num.seqs=12 Similarity=0.887502 0
CGCTTTTATCGTCACGTAAAATGATTTCAACGCGTTTTGCTGATAATAAAT

****
Consensus:

CGCTTTTATCGTCACGTAAAATGATTTCAACGCGTTTTGCTGATAATAAAT

>Bacillus_Fam_1379_51_1 Nr. of seq. 1 Alignment length(with gaps) = 51 Alignment score = 0.000000 GCF_000331575.1_ASM33157v1_genomic.fna_1:1553326-1553721 Satlength=396 Nr of Repeats=4 RepeatLength=51 seed=TCCGTTATTT Num.seqs=3 Similarity=0.694989 0
TCCGTTATTTGTCCAAAACCCTATGCTACAGCTATTTTNCCGGACACCAGG

****
Consensus:

TCCGTTATTTGTCCAAAACCCTATGCTACAGCTATTTTNCCGGACACCAGG

>Bacillus_Fam_1380_51_1 Nr. of seq. 1 Alignment length(with gaps) = 51 Alignment score = 0.000000 GCF_000374565.1_ASM37456v1_genomic.fna_9:47516-47873 Satlength=358 Nr of Repeats=7 RepeatLength=51 seed=GCTTCAGTTT Num.seqs=7 Similarity=0.841892
0   GCTTCAGTTTCTTCATCCGAAATCATCTCAGCTGTACCTGCAGGCTCTTTT

****
Consensus:

GCTTCAGTTTCTTCATCCGAAATCATCTCAGCTGTACCTGCAGGCTCTTTT

>Bacillus_Fam_1381_51_1 Nr. of seq. 1 Alignment length(with gaps) = 51 Alignment score = 0.000000 GCF_000712595.1_ASM71259v1_genomic.fna_5:211104-211479 Satlength=376 Nr of Repeats=4 RepeatLength=51 seed=ATATTTTTTT Num.seqs=3

Similarity=0.912854
0   ATATTTTTTGAACGTTCTTCACGTTTGCTAGATCGTTTTTCGCGATCATA

****
Consensus:

ATATTTTTTGAACGTTCTTCACGTTTGCTAGATCGTTTTTCGCGATCATA

>Bacillus_Fam_1382_51_1 Nr. of seq. 1 Alignment length(with gaps) = 51
Alignment score = 0.000000
GCF_000752035.1_Bacillus_rubiinfantis_genomic.fna_11:404655-405420
Satlength=766 Nr of Repeats=15 RepeatLength=51 seed=AGTTGTCCAT
Num.seqs=15 Similarity=0.840842 0
AGTTGTCCATTTGCGGNGGGCTATTGGACAACTTTTTCNGAAAAACACCAG

****
Consensus:

AGTTGTCCATTTGCGGNGGGCTATTGGACAACTTTTTCNGAAAAACACCAG

>Bacillus_Fam_1383_51_1 Nr. of seq. 1 Alignment length(with gaps) = 51
Alignment score = 0.000000
GCF_000752035.1_Bacillus_rubiinfantis_genomic.fna_11:437106-437412
Satlength=307 Nr of Repeats=6 RepeatLength=51 seed=TTGGACAGCT Num.seqs=6
Similarity=0.864052 0
TTGGACAGCTTCGCGCTATTTTCCAAAAAGTTGTCCATTAGACACAAGCTA

****
Consensus:

TTGGACAGCTTCGCGCTATTTTCCAAAAAGTTGTCCATTAGACACAAGCTA

>Bacillus_Fam_1384_51_1 Nr. of seq. 1 Alignment length(with gaps) = 51
Alignment score = 0.000000
GCF_000752035.1_Bacillus_rubiinfantis_genomic.fna_30:51081-51439
Satlength=359 Nr of Repeats=7 RepeatLength=51 seed=TTTTCTCTAA Num.seqs=6
Similarity=0.914597 0
TTTTCTCTAATTACGATATTTCTGTCCAATAGGACCTGGCTATTGGACAGG

****
Consensus:

TTTTCTCTAATTACGATATTTCTGTCCAATAGGACCTGGCTATTGGACAGG

>Bacillus_Fam_1385_51_1 Nr. of seq. 1 Alignment length(with gaps) = 51
Alignment score = 0.000000
GCF_000986785.1_ASM98678v1_genomic.fna_52:2-207 Satlength=206 Nr of
Repeats=4 RepeatLength=51 seed=CTTGGCGCAA Num.seqs=3 Similarity=0.762821
0   CTTGGCGCAAAAAGTTTTCCCGAGGNTGGAGAAATTGCGTTAACGCAGGGT

****
Consensus:

CTTGGCGCAAAAAGTTTTCCCGAGGNTGGAGAAATTGCGTTAACGCAGGGT

>Bacillus_Fam_1386_51_1 Nr. of seq. 1 Alignment length(with gaps) = 51
Alignment score = 0.000000
GCF_001375535.1_Bacillus_niameyensis_genomic.fna_6:3343036-3343241
Satlength=206 Nr of Repeats=4 RepeatLength=51 seed=CTCAGAGACA Num.seqs=3
Similarity=0.773420 0
CTCAGAGACAAAATCCAGGNAAAAAGTAGNGATTTGTCTCTGATCGGGTGT

****
Consensus:

CTCAGAGACAAAATCCAGGNAAAAAGTAGNGATTTGTCTCTGATCGGGTGT

>Bacillus_Fam_1387_51_1 Nr. of seq. 1 Alignment length(with gaps) = 51
Alignment score = 0.000000
GCF_001375535.1_Bacillus_niameyensis_genomic.fna_6:4007424-4007779
Satlength=356 Nr of Repeats=7 RepeatLength=51 seed=CTTCGGTCGA Num.seqs=5
Similarity=0.848077 0
CTTCGGTCGAATAGAAACGCCCTATTCGCGCATTTCTGTGATTTTCTTTTN

****
Consensus:

CTTCGGTCGAATAGAAACGCCCTATTCGCGCATTTCTGTGATTTTCTTTTN

>Bacillus_Fam_1388_51_1 Nr. of seq. 1 Alignment length(with gaps) = 51
Alignment score = 0.000000
GCF_001375535.1_Bacillus_niameyensis_genomic.fna_6:4072217-4072880
Satlength=664 Nr of Repeats=13 RepeatLength=51 seed=CGCCCCAATT
Num.seqs=13 Similarity=0.887410 0
CGCCCCAATTCACCCGAAGAAAAGGAAATACACGTCAANGGTCGAATAGAT

****
Consensus:

CGCCCCAATTCACCCGAAGAAAAGGAAATACACGTCAANGGTCGAATAGAT

>Bacillus_Fam_1389_51_1 Nr. of seq. 1 Alignment length(with gaps) = 51
Alignment score = 0.000000
GCF_001375535.1_Bacillus_niameyensis_genomic.fna_6:4237328-4237736
Satlength=409 Nr of Repeats=8 RepeatLength=51 seed=GGTCGAATAG Num.seqs=6
Similarity=0.775641 0
GGTCGAATAGAAGCGATCAATTCGACCGTTGCTGGAAAATCCGCTTTGTTT

****
Consensus:

GGTCGAATAGAAGCGATCAATTCGACCGTTGCTGGAAAATCCGCTTTGTTT

>Bacillus_Fam_1390_51_1 Nr. of seq. 1 Alignment length(with gaps) = 51
Alignment score = 0.000000
GCF_001587375.1_ASM158737v1_genomic.fna_14:69179-69489 Satlength=311 Nr
of Repeats=6 RepeatLength=52 seed=CTTATACATT Num.seqs=4

Similarity=0.611616
0   CTTATACATTTTTTCTTTCTTCCCATGAAAATCTATACGCATCCTCCCTTC

****
Consensus:

CTTATACATTTTTTCTTTCTTCCCATGAAAATCTATACGCATCCTCCCTTC

>Bacillus_Fam_1391_51_1 Nr. of seq. 1 Alignment length(with gaps) = 51
Alignment score = 0.000000
GCF_001591465.1_ASM159146v1_genomic.fna_6:84138-84347 Satlength=210 Nr of Repeats=4 RepeatLength=52 seed=TTCGGACACA Num.seqs=3 Similarity=0.718107
0   TTCGGACACAGAAAGGGGGAACCCGTGGAATTGAGTCAGAACCCGGNCAAG

****
Consensus:

TTCGGACACAGAAAGGGGGAACCCGTGGAATTGAGTCAGAACCCGGNCAAG

>Bacillus_Fam_1392_51_1 Nr. of seq. 1 Alignment length(with gaps) = 51
Alignment score = 0.000000
GCF_001591645.1_ASM159164v1_genomic.fna_12:25703-25906 Satlength=204 Nr of Repeats=4 RepeatLength=51 seed=TAATCGTTCC Num.seqs=3
Similarity=0.557692
0   TAATCGTTCCCGAAAATGCNGTGTANGAGGCTGGNGGTAATGAATAGGGAG

****
Consensus:

TAATCGTTCCCGAAAATGCNGTGTANGAGGCTGGNGGTAATGAATAGGGAG

>Bacillus_Fam_1393_51_1 Nr. of seq. 1 Alignment length(with gaps) = 51
Alignment score = 0.000000
GCF_001636315.1_ASM163631v1_genomic.fna_1:3673042-3673458 Satlength=417 Nr of Repeats=5 RepeatLength=52 seed=ATAGAGCGCG Num.seqs=3
Similarity=0.524904 0
ATAGAGCGCGTGTCCAGTAATTTATTGGAAAAGGCGTCCTATGAACCATTC

****
Consensus:

ATAGAGCGCGTGTCCAGTAATTTATTGGAAAAGGCGTCCTATGAACCATTC

>Bacillus_Fam_1394_51_1 Nr. of seq. 1 Alignment length(with gaps) = 51
Alignment score = 0.000000
GCF_001636325.1_ASM163632v1_genomic.fna_1:1408479-1409142 Satlength=664 Nr of Repeats=13 RepeatLength=51 seed=AAAATGGTCC Num.seqs=13
Similarity=0.876655 0
AAAATGGTCCTAGTTCACCAAACTGGAACCAAAATCAAGAAAGACAGCGTA

****
Consensus:

AAAATGGTCCTAGTTCACCAAACTGGAACCAAAATCAAGAAAGACAGCGTA

>Bacillus_Fam_1395_51_1 Nr. of seq. 1 Alignment length(with gaps) = 51
Alignment score = 0.000000
GCF_001636325.1_ASM163632v1_genomic.fna_2:1137164-1137571 Satlength=408
Nr of Repeats=8 RepeatLength=51 seed=AACTGTGACC Num.seqs=7
Similarity=0.925303 0
AACTGTGACCAAAAATGGCGGAGAAATCAAAAAAACGGTCACAGAGTGTAA

****
Consensus:

AACTGTGACCAAAAATGGCGGAGAAATCAAAAAAACGGTCACAGAGTGTAA

>Bacillus_Fam_1396_51_1 Nr. of seq. 1 Alignment length(with gaps) = 51
Alignment score = 0.000000
GCF_001636335.1_ASM163633v1_genomic.fna_2:629683-630193 Satlength=511 Nr of Repeats=10 RepeatLength=51 seed=AAAAAAAGTA Num.seqs=10
Similarity=0.933188 0
AAAAAAAGTAAAAAAACATGTAAAGAGTCTCATAGGAGCATTCAATGGGAC

****
Consensus:

AAAAAAAGTAAAAAAACATGTAAAGAGTCTCATAGGAGCATTCAATGGGAC

>Bacillus_Fam_1397_51_1 Nr. of seq. 1 Alignment length(with gaps) = 51
Alignment score = 0.000000
GCF_002019605.1_ASM201960v1_genomic.fna_1:4244978-4245368 Satlength=391
Nr of Repeats=8 RepeatLength=51 seed=TAAAAGAGAA Num.seqs=6
Similarity=0.839651 0
TAAAAGAGAACTTAGGATGAATAGCTTCCACAAACTGTAAGTGGAAGCATC

****
Consensus:

TAAAAGAGAACTTAGGATGAATAGCTTCCACAAACTGTAAGTGGAAGCATC

>Bacillus_Fam_1398_51_1 Nr. of seq. 1 Alignment length(with gaps) = 51
Alignment score = 0.000000
GCF_002019605.1_ASM201960v1_genomic.fna_1:5051740-5052045 Satlength=306
Nr of Repeats=6 RepeatLength=51 seed=GACTTTAGAG Num.seqs=5
Similarity=0.725490 0
GACTTTAGAGNACATTNCTTCCTNATACTGGTAATTAAAGGAGCTAAGATT

****
Consensus:

GACTTTAGAGNACATTNCTTCCTNATACTGGTAATTAAAGGAGCTAAGATT

>Bacillus_Fam_1399_51_1 Nr. of seq. 1 Alignment length(with gaps) = 51
Alignment score = 0.000000
GCF_002019665.1_ASM201966v1_genomic.fna_1:947086-947749 Satlength=664 Nr of Repeats=13 RepeatLength=51 seed=GGATTTTGGA Num.seqs=13

Similarity=0.922574 0
GGATTTTGGAAGAAGGGAAAAGCATGCCCCCAAAGTGGCGTTAGGATACAT

****
Consensus:

GGATTTTGGAAGAAGGGAAAAGCATGCCCCCAAAGTGGCGTTAGGATACAT

>Bacillus_Fam_1400_51_1 Nr. of seq. 1 Alignment length(with gaps) = 51
Alignment score = 0.000000
GCF_002019665.1_ASM201966v1_genomic.fna_1:3812725-3813857 Satlength=1133
Nr of Repeats=21 RepeatLength=51 seed=TTCTATTACC Num.seqs=15
Similarity=0.838655 0
TTCTATTACCTCTATCCTGCTTTTGCGATCACCCTTTTCGGCTTCGGGCTT

****
Consensus:

TTCTATTACCTCTATCCTGCTTTTGCGATCACCCTTTTCGGCTTCGGGCTT

>Bacillus_Fam_1401_51_1 Nr. of seq. 1 Alignment length(with gaps) = 51
Alignment score = 0.000000
GCF_900094975.1_IMG-
taxon_2617270721_annotated_assembly_genomic.fna_13:191015-191219
Satlength=205 Nr of Repeats=4 RepeatLength=51 seed=CCATCCGGAA Num.seqs=4
Similarity=0.856209 0
CCATCCGGAAGTGGTGGAAAGATTTCATCCCCTGCAAGTTCTACCCCAGTC

****
Consensus:

CCATCCGGAAGTGGTGGAAAGATTTCATCCCCTGCAAGTTCTACCCCAGTC

>Bacillus_Fam_1402_51_1 Nr. of seq. 1 Alignment length(with gaps) = 51
Alignment score = 0.000000
GCF_900104555.1_IMG-
taxon_2651870172_annotated_assembly_genomic.fna_20:425714-425918
Satlength=205 Nr of Repeats=4 RepeatLength=51 seed=CATCCTTCAC Num.seqs=4
Similarity=0.825472 0
CATCCTTCACCATGATCACGGGCACTTATCTCCTCTTTGGTTACCTCTTTT

****
Consensus:

CATCCTTCACCATGATCACGGGCACTTATCTCCTCTTTGGTTACCTCTTTT

>Bacillus_Fam_1403_51_1 Nr. of seq. 1 Alignment length(with gaps) = 51
Alignment score = 0.000000
GCF_900104555.1_IMG-
taxon_2651870172_annotated_assembly_genomic.fna_22:4620-4862
Satlength=243 Nr of Repeats=4 RepeatLength=51 seed=TTTGGGGAAC Num.seqs=3
Similarity=0.728632 0
TTTGGGGAACCTATCTCGGCTTTGGTTACCTTTTCCTTGGCTTCGGNTCGA

****
Consensus:

TTTGGGGAACCTATCTCGGCTTTGGTTACCTTTTCCTTGGCTTCGGNTCGA

>Bacillus_Fam_1404_51_1 Nr. of seq. 1 Alignment length(with gaps) = 51
Alignment score = 0.000000
GCF_900156875.1_PRJEB18969_genomic.fna_11:2558203-2558548 Satlength=346
Nr of Repeats=4 RepeatLength=51 seed=TTCTTCAACA Num.seqs=3
Similarity=0.825708 0
TTCTTCAACAGCCTCTATTGCTTGTGGGACGGATTCCTCGGTCATTTCCAC

****
Consensus:

TTCTTCAACAGCCTCTATTGCTTGTGGGACGGATTCCTCGGTCATTTCCAC

>Bacillus_Fam_1405_50_1 Nr. of seq. 1 Alignment length(with gaps) = 50
Alignment score = 0.000000
GCF_000331575.1_ASM33157v1_genomic.fna_4:7266-7566 Satlength=301 Nr of
Repeats=6 RepeatLength=50 seed=CGACTGCCAC Num.seqs=6 Similarity=0.752889
0 CGACTGCCACTATTTGGAAAACAAAATGCACACACACTCATTTACCGCCT

****
Consensus:

CGACTGCCACTATTTGGAAAACAAAATGCACACACACTCATTTACCGCCT

>Bacillus_Fam_1406_50_1 Nr. of seq. 1 Alignment length(with gaps) = 50
Alignment score = 0.000000
GCF_000751775.1_A1A_genomic.fna_12:25055-25349 Satlength=295 Nr of
Repeats=6 RepeatLength=49 seed=TTTAAAATCA Num.seqs=6 Similarity=0.703111
0 TTTAAAATCAAAGGAATAGATTGTGGTCGGGTNAAATCAAGTAGCTCACT

****
Consensus:

TTTAAAATCAAAGGAATAGATTGTGGTCGGGTNAAATCAAGTAGCTCACT

>Bacillus_Fam_1407_48_1 Nr. of seq. 1 Alignment length(with gaps) = 48
Alignment score = 0.000000
GCF_000186145.1_Bacillus_sp_2_A_57_CT2_V1_genomic.fna_41:18-465
Satlength=448 Nr of Repeats=7 RepeatLength=48 seed=GCGATGATGC Num.seqs=5
Similarity=0.983333 0
GCGATGATGCCGCAGATGCCGCATCAGATGCCACATCAGGTGCAGGGA

****
Consensus:

GCGATGATGCCGCAGATGCCGCATCAGATGCCACATCAGGTGCAGGGA

>Bacillus_Fam_1408_48_1 Nr. of seq. 1 Alignment length(with gaps) = 48
Alignment score = 0.000000

GCF_000299035.1_ASM29903v1_genomic.fna_27:4592-4928 Satlength=337 Nr of Repeats=7 RepeatLength=48 seed=TCAGCCAGAA Num.seqs=7 Similarity=0.952381
0 TCAGCCAGAAAACAACGGAAAAGGATATAAACAAGAAAATAGAGAGTC

****
Consensus:

TCAGCCAGAAAACAACGGAAAAGGATATAAACAAGAAAATAGAGAGTC

>Bacillus_Fam_1409_48_1 Nr. of seq. 1 Alignment length(with gaps) = 48
Alignment score = 0.000000
GCF_000307855.1_BAZO1.0_genomic.fna_111:42295-42598 Satlength=304 Nr of Repeats=4 RepeatLength=48 seed=GGGATGCCGC Num.seqs=3 Similarity=0.888889
0 GGGATGCCGCTAAATCCGGCTCCTGGTACCCCTCCATAACCTGCTGGT

****
Consensus:

GGGATGCCGCTAAATCCGGCTCCTGGTACCCCTCCATAACCTGCTGGT

>Bacillus_Fam_1410_48_1 Nr. of seq. 1 Alignment length(with gaps) = 48
Alignment score = 0.000000
GCF_000430785.1_ASM43078v1_genomic.fna_5:99863-100391 Satlength=529 Nr of Repeats=11 RepeatLength=48 seed=AAACCAACAG Num.seqs=11 Similarity=0.825253
0 AAACCAACAGAGTCAGCATCATTGAGCAATGCTGCATAATCATCTTGT

****
Consensus:

AAACCAACAGAGTCAGCATCATTGAGCAATGCTGCATAATCATCTTGT

>Bacillus_Fam_1411_48_1 Nr. of seq. 1 Alignment length(with gaps) = 48
Alignment score = 0.000000
GCF_000504145.1_SOAPdenovo_for_version2.04_of_Bacillus_sp._FJAT-14515_genome_genomic.fna_27:69414-69702 Satlength=289 Nr of Repeats=6 RepeatLength=48 seed=TCATTTGCTG Num.seqs=6 Similarity=0.962963 0
TCATTTGCTGTCCTGAATACATTGGAGAAGGTATGCTAGAATATGGCC

****
Consensus:

TCATTTGCTGTCCTGAATACATTGGAGAAGGTATGCTAGAATATGGCC

>Bacillus_Fam_1412_48_1 Nr. of seq. 1 Alignment length(with gaps) = 48
Alignment score = 0.000000
GCF_000615945.1_ASM61594v1_genomic.fna_79:12436-12628 Satlength=193 Nr of Repeats=4 RepeatLength=48 seed=AGCCCGATGA Num.seqs=4 Similarity=0.939815
0 AGCCCGATGAGAAATTACTATAGAGCGATTGATCAGCACGTGACCGAT

****
Consensus:

AGCCCGATGAGAAATTACTATAGAGCGATTGATCAGCACGTGACCGAT

>Bacillus_Fam_1413_48_1 Nr. of seq. 1 Alignment length(with gaps) = 48
Alignment score = 0.000000
GCF_000787375.1_ASM78737v1_genomic.fna_23:9-231 Satlength=223 Nr of Repeats=5 RepeatLength=48 seed=GCTTCTTCTG Num.seqs=4 Similarity=0.925926
0 GCTTCTTCTGTTGTTTCCTCTTCTTGGCCTTCTTCATCTACAGCATCC

****
Consensus:

GCTTCTTCTGTTGTTTCCTCTTCTTGGCCTTCTTCATCTACAGCATCC

>Bacillus_Fam_1414_48_1 Nr. of seq. 1 Alignment length(with gaps) = 48
Alignment score = 0.000000
GCF_000812025.2_ASM81202v2_genomic.fna_60:32692-32932 Satlength=241 Nr of Repeats=5 RepeatLength=48 seed=GCAAAGAAAG Num.seqs=5 Similarity=0.904082
0 GCAAAGAAAGAAGCCGAAGAACAAGCGAAACGAGAGGCTGAAGAACAG

****
Consensus:

GCAAAGAAAGAAGCCGAAGAACAAGCGAAACGAGAGGCTGAAGAACAG

>Bacillus_Fam_1415_48_1 Nr. of seq. 1 Alignment length(with gaps) = 48
Alignment score = 0.000000
GCF_001274775.1_ASM127477v1_genomic.fna_3:296300-296492 Satlength=193 Nr of Repeats=4 RepeatLength=48 seed=GGTGTGATGG Num.seqs=4 Similarity=0.888889
0 GGTGTGATGGGAGCAGAGATGCATCATCAGATGCCGATGGGTCAGCCG

****
Consensus:

GGTGTGATGGGAGCAGAGATGCATCATCAGATGCCGATGGGTCAGCCG

>Bacillus_Fam_1416_48_1 Nr. of seq. 1 Alignment length(with gaps) = 48
Alignment score = 0.000000
GCF_001278705.1_ASM127870v1_genomic.fna_1:2286101-2286677 Satlength=577 Nr of Repeats=12 RepeatLength=48 seed=TTAGAAAGAA Num.seqs=12 Similarity=0.986111 0
TTAGAAAGAAGCAGAGACAGAAGTTCGGAAAGCAGTGTGGACAATGAC

****
Consensus:

TTAGAAAGAAGCAGAGACAGAAGTTCGGAAAGCAGTGTGGACAATGAC

>Bacillus_Fam_1417_48_1 Nr. of seq. 1 Alignment length(with gaps) = 48
Alignment score = 0.000000
GCF_001578185.1_ASM157818v1_genomic.fna_1:5252350-5252542 Satlength=193 Nr of Repeats=4 RepeatLength=48 seed=CGGCCTCCCC Num.seqs=4 Similarity=0.949074 0
CGGCCTCCCCGCGAATCCTCACGTTTAGGTCCGCGGCTGTTTTCCATC

****
Consensus:

CGGCCTCCCCGCGAATCCTCACGTTTAGGTCCGCGGCTGTTTTCCATC

>Bacillus_Fam_1418_48_1 Nr. of seq. 1 Alignment length(with gaps) = 48
Alignment score = 0.000000
GCF_001578205.1_ASM157820v1_genomic.fna_1:1115267-1115459 Satlength=193
Nr of Repeats=4 RepeatLength=48 seed=CATCATAAGA Num.seqs=4
Similarity=0.902778 0
CATCATAAGAAGAGCTGCCGCAAGTCACATCGCAGCAAGCACAGATCG

****
Consensus:

CATCATAAGAAGAGCTGCCGCAAGTCACATCGCAGCAAGCACAGATCG

>Bacillus_Fam_1419_48_1 Nr. of seq. 1 Alignment length(with gaps) = 48
Alignment score = 0.000000
GCF_001590785.1_ASM159078v1_genomic.fna_12:121693-122077 Satlength=385 Nr
of Repeats=8 RepeatLength=48 seed=ATGCCGTGGC Num.seqs=8
Similarity=0.957341 0
ATGCCGTGGCAAAATGGGATGATGCCAATGCCTGGACAGCAGGAAGGA

****
Consensus:

ATGCCGTGGCAAAATGGGATGATGCCAATGCCTGGACAGCAGGAAGGA

>Bacillus_Fam_1420_48_1 Nr. of seq. 1 Alignment length(with gaps) = 48
Alignment score = 0.000000
GCF_001592005.1_ASM159200v1_genomic.fna_4:110265-110505 Satlength=241 Nr
of Repeats=5 RepeatLength=48 seed=TATAAGAAGG Num.seqs=5
Similarity=0.894444
0   TATAAGAAGGACTACTACTACCATGAGCCTTACAAGAAGCATCACGAG

****
Consensus:

TATAAGAAGGACTACTACTACCATGAGCCTTACAAGAAGCATCACGAG

>Bacillus_Fam_1421_48_1 Nr. of seq. 1 Alignment length(with gaps) = 48
Alignment score = 0.000000
GCF_900109925.1_IMG-
taxon_2651870116_annotated_assembly_genomic.fna_12:1886-2102
Satlength=217 Nr of Repeats=5 RepeatLength=48 seed=CTGTTCCTTC Num.seqs=4
Similarity=0.740741 0
CTGTTCCTTCATCCGAACCAGGGGCGGTTCCTTCTTCCGGCTCATCTC

****
Consensus:

CTGTTCCTTCATCCGAACCAGGGGCGGTTCCTTCTTCCGGCTCATCTC

>Bacillus_Fam_1422_47_1 Nr. of seq. 1 Alignment length(with gaps) = 47
Alignment score = 0.000000
GCF_001895885.1_ASM189588v1_genomic.fna_2:13546-13781 Satlength=236 Nr of Repeats=5 RepeatLength=47 seed=AAACACGAGT Num.seqs=5 Similarity=0.971631
0 AAACACGAGTAAGAATATGAATTATTCAGCTACCTTGATTACGTTTA

****
Consensus:

AAACACGAGTAAGAATATGAATTATTCAGCTACCTTGATTACGTTTA

>Bacillus_Fam_1423_45_1 Nr. of seq. 1 Alignment length(with gaps) = 45
Alignment score = 0.000000
GCF_000161455.1_ASM16145v1_genomic.fna_1:4518727-4518952 Satlength=226 Nr of Repeats=5 RepeatLength=45 seed=TTGTTGATTT Num.seqs=5 Similarity=0.855556 0
TTGTTGATTTCTCTTCTTTTTTACCTTTTTCATCAGCTGAACCGC

****
Consensus:

TTGTTGATTTCTCTTCTTTTTTACCTTTTTCATCAGCTGAACCGC

>Bacillus_Fam_1424_45_1 Nr. of seq. 1 Alignment length(with gaps) = 45
Alignment score = 0.000000
GCF_000161455.1_ASM16145v1_genomic.fna_1:5389248-5389428 Satlength=181 Nr of Repeats=4 RepeatLength=45 seed=CACCATCAAG Num.seqs=4 Similarity=0.906173 0
CACCATCAAGTACAACAGGTGGATCTCAATCAAGTGGTGGAGGAG

****
Consensus:

CACCATCAAGTACAACAGGTGGATCTCAATCAAGTGGTGGAGGAG

>Bacillus_Fam_1425_45_1 Nr. of seq. 1 Alignment length(with gaps) = 45
Alignment score = 0.000000
GCF_000334155.1_L1_genomic.fna_18:2736-3096 Satlength=361 Nr of Repeats=8 RepeatLength=45 seed=GCCCGAAGCA Num.seqs=6 Similarity=0.871605
0 GCCCGAAGCATTAGGGGTCAGNGCAGAAGTGCCCAGAGCGTGAGA

****
Consensus:

GCCCGAAGCATTAGGGGTCAGNGCAGAAGTGCCCAGAGCGTGAGA

>Bacillus_Fam_1426_45_1 Nr. of seq. 1 Alignment length(with gaps) = 45
Alignment score = 0.000000
GCF_000473245.1_ASM47324v1_genomic.fna_1:550520-550790 Satlength=271 Nr of Repeats=5 RepeatLength=45 seed=CCGGAGTTTT Num.seqs=4 Similarity=0.624691
0 CCGGAGTATTAACAGAAATAGAATCACACACATCGACATCGACAA

****
Consensus:

CCGGAGTATTAACAGAAATAGAATCACACACATCGACATCGACAA

>Bacillus_Fam_1427_45_1 Nr. of seq. 1 Alignment length(with gaps) = 45
Alignment score = 0.000000
GCF_000712595.1_ASM71259v1_genomic.fna_33:9174-9894 Satlength=721 Nr of Repeats=16 RepeatLength=45 seed=TCCTTGTGGT Num.seqs=16 Similarity=0.978519
0   TCCTTGTGGTCCAGTGGGACCGATAGGCCCTGGTTCCCCTTGTGA

****
Consensus:

TCCTTGTGGTCCAGTGGGACCGATAGGCCCTGGTTCCCCTTGTGA

>Bacillus_Fam_1428_45_1 Nr. of seq. 1 Alignment length(with gaps) = 45
Alignment score = 0.000000
GCF_000712595.1_ASM71259v1_genomic.fna_43:3917-4656 Satlength=740 Nr of Repeats=5 RepeatLength=45 seed=GAGCAATCTA Num.seqs=3 Similarity=0.980247
0   GAGCAATCTAGTAATTATTGACGGTATCAGCCGTTATCTGAACTT

****
Consensus:

GAGCAATCTAGTAATTATTGACGGTATCAGCCGTTATCTGAACTT

>Bacillus_Fam_1429_45_1 Nr. of seq. 1 Alignment length(with gaps) = 45
Alignment score = 0.000000
GCF_000712615.1_ASM71261v1_genomic.fna_3:111271-111496 Satlength=226 Nr of Repeats=5 RepeatLength=45 seed=TCTTGAGTTT Num.seqs=5 Similarity=0.857778
0   TCTTGAGTTTGCTGAGATACGTGTTGATTTTGTTGACGTTGAGGT

****
Consensus:

TCTTGAGTTTGCTGAGATACGTGTTGATTTTGTTGACGTTGAGGT

>Bacillus_Fam_1430_45_1 Nr. of seq. 1 Alignment length(with gaps) = 45
Alignment score = 0.000000
GCF_000712615.1_ASM71261v1_genomic.fna_42:5062-5242 Satlength=181 Nr of Repeats=4 RepeatLength=45 seed=GGAACAGAAA Num.seqs=4 Similarity=0.901235
0   GGAACAGAAAGACAAGGAATGGGTGAAGGAAATGCAGTTCCAGTA

****
Consensus:

GGAACAGAAAGACAAGGAATGGGTGAAGGAAATGCAGTTCCAGTA

>Bacillus_Fam_1431_45_1 Nr. of seq. 1 Alignment length(with gaps) = 45
Alignment score = 0.000000

GCF_000724485.1_ASM72448v1_genomic.fna_1:2469191-2469371 Satlength=181 Nr of Repeats=4 RepeatLength=45 seed=TGTATCGGTG Num.seqs=4 Similarity=0.777778 0
TGTATCGGTGAAACGGGCATTTGCTTTTCTACTTTAGGAAATTCC

****
Consensus:

TGTATCGGTGAAACGGGCATTTGCTTTTCTACTTTAGGAAATTCC

>Bacillus_Fam_1432_45_1 Nr. of seq. 1 Alignment length(with gaps) = 45 Alignment score = 0.000000
GCF_000752395.1_Bacillus_andreraoultii_genomic.fna_22:32281-32461 Satlength=181 Nr of Repeats=5 RepeatLength=45 seed=GATAAACCGG Num.seqs=3 Similarity=0.822222 0
GATAAACCGGATGGCGAGAATCCAGATGTAGACAAGCCTGACACG

****
Consensus:

GATAAACCGGATGGCGAGAATCCAGATGTAGACAAGCCTGACACG

>Bacillus_Fam_1433_45_1 Nr. of seq. 1 Alignment length(with gaps) = 45 Alignment score = 0.000000
GCF_000812025.2_ASM81202v2_genomic.fna_70:2588-3101 Satlength=514 Nr of Repeats=9 RepeatLength=45 seed=TCTGTGCCTT Num.seqs=6 Similarity=0.747160
0   TCTGTGCCTTCCGTTCCTTCGGTTACCGCTTCTGCTCCTGTGTCG

****
Consensus:

TCTGTGCCTTCCGTTCCTTCGGTTACCGCTTCTGCTCCTGTGTCG

>Bacillus_Fam_1434_45_1 Nr. of seq. 1 Alignment length(with gaps) = 45 Alignment score = 0.000000
GCF_001591425.1_ASM159142v1_genomic.fna_9:41557-41833 Satlength=277 Nr of Repeats=4 RepeatLength=45 seed=TTTTCCTCTT Num.seqs=3 Similarity=0.812346
0   TTTTCCTCTTTAGGAGGAGTTGGATTCGAAGGCTTNGGAGCCGGT

****
Consensus:

TTTTCCTCTTTAGGAGGAGTTGGATTCGAAGGCTTNGGAGCCGGT

>Bacillus_Fam_1435_45_1 Nr. of seq. 1 Alignment length(with gaps) = 45 Alignment score = 0.000000
GCF_001591425.1_ASM159142v1_genomic.fna_27:20593-20812 Satlength=220 Nr of Repeats=5 RepeatLength=45 seed=CTCATGAAGA Num.seqs=4 Similarity=0.891358
0   CTCATGAAGAAGATCACGACCACGATCACGACCATGAACACGATG

****
Consensus:

CTCATGAAGAAGATCACGACCACGATCACGACCATGAACACGATG

>Bacillus_Fam_1436_45_1 Nr. of seq. 1 Alignment length(with gaps) = 45
Alignment score = 0.000000
GCF_001636315.1_ASM163631v1_genomic.fna_1:2589898-2590303 Satlength=406
Nr of Repeats=8 RepeatLength=45 seed=TCCTCTTCAT Num.seqs=7
Similarity=0.893030 0
TCCTCTTCATCTATAAAAGAGCTCTCTTCTTGGGGAGAACTGGAT

****
Consensus:

TCCTCTTCATCTATAAAAGAGCTCTCTTCTTGGGGAGAACTGGAT

>Bacillus_Fam_1437_45_1 Nr. of seq. 1 Alignment length(with gaps) = 45
Alignment score = 0.000000
GCF_001645555.1_ASM164555v1_genomic.fna_43:255404-255833 Satlength=430 Nr of Repeats=11 RepeatLength=45 seed=CCATTATTTC Num.seqs=8
Similarity=0.780952 0
CCATTATTTCCTTGCGAGCCTCTTCCGTTGTTCTCTTGCCCATTC

****
Consensus:

CCATTATTTCCTTGCGAGCCTCTTCCGTTGTTCTCTTGCCCATTC

>Bacillus_Fam_1438_45_1 Nr. of seq. 1 Alignment length(with gaps) = 45
Alignment score = 0.000000
GCF_001896335.1_ASM189633v1_genomic.fna_15:81928-82711 Satlength=784 Nr of Repeats=7 RepeatLength=45 seed=GCTCCGGTTA Num.seqs=6
Similarity=0.920988
0 GCTCCGGTTACTCCGGTCGCTCCCGTATCTCCCGTTGCACCTGTG

****
Consensus:

GCTCCGGTTACTCCGGTCGCTCCCGTATCTCCCGTTGCACCTGTG

>Bacillus_Fam_1439_43_1 Nr. of seq. 1 Alignment length(with gaps) = 43
Alignment score = 0.000000
GCF_000175075.1_ASM17507v1_genomic.fna_7:5367-5580 Satlength=214 Nr of Repeats=5 RepeatLength=43 seed=AAAACCTTCT Num.seqs=3 Similarity=0.958656
0 AAAACCTTCTAACAAAGATCCAACCCCAACCCCTAGTTACCCA

****
Consensus:

AAAACCTTCTAACAAAGATCCAACCCCAACCCCTAGTTACCCA

>Bacillus_Fam_1440_43_1 Nr. of seq. 1 Alignment length(with gaps) = 43
Alignment score = 0.000000
GCF_001591445.1_ASM159144v1_genomic.fna_9:156414-156582 Satlength=169 Nr of Repeats=4 RepeatLength=42 seed=GGCTGGTTAG Num.seqs=4

Similarity=0.801034
0   GGCTGGTTAGATTCGGTTCTTGCTTCTTCGTANATCCAATGAA

****
Consensus:

GGCTGGTTAGATTCGGTTCTTGCTTCTTCGTANATCCAATGAA

>Bacillus_Fam_1441_43_1 Nr. of seq. 1 Alignment length(with gaps) = 43
Alignment score = 0.000000
GCF_001742425.1_ASM174242v1_genomic.fna_38:12301-12469 Satlength=169 Nr of Repeats=4 RepeatLength=42 seed=TCAGTTTTAA Num.seqs=4
Similarity=0.816537
0   TCAGTTTTAAGCTCGGAGACATCTGTTTTTAAGNTTAGAAACC

****
Consensus:

TCAGTTTTAAGCTCGGAGACATCTGTTTTTAAGNTTAGAAACC

>Bacillus_Fam_1442_42_1 Nr. of seq. 1 Alignment length(with gaps) = 42
Alignment score = 0.000000
GCF_000011145.1_ASM1114v1_genomic.fna_1:2885595-2885763 Satlength=169 Nr of Repeats=4 RepeatLength=42 seed=TGATCATCTT Num.seqs=4
Similarity=0.915344
0   TGATCATCTTCCTCGTGACCATGCTCCTCATGGCTATGGTCG

****
Consensus:

TGATCATCTTCCTCGTGACCATGCTCCTCATGGCTATGGTCG

>Bacillus_Fam_1443_42_1 Nr. of seq. 1 Alignment length(with gaps) = 42
Alignment score = 0.000000
GCF_000017425.1_ASM1742v1_genomic.fna_1:1132359-1132527 Satlength=169 Nr of Repeats=4 RepeatLength=42 seed=AGGTTTTTGT Num.seqs=4
Similarity=0.947090
0   AGGTTTTTGTGCTTCAGGTTTAGCTGTTTCGTTGTTTACAGC

****
Consensus:

AGGTTTTTGTGCTTCAGGTTTAGCTGTTTCGTTGTTTACAGC

>Bacillus_Fam_1444_42_1 Nr. of seq. 1 Alignment length(with gaps) = 42
Alignment score = 0.000000
GCF_000017425.1_ASM1742v1_genomic.fna_1:1831558-1831726 Satlength=169 Nr of Repeats=4 RepeatLength=42 seed=AATCAAGGAA Num.seqs=4
Similarity=0.904762
0   AATCAAGGAAATCAAACGCCGCCAAATAGCGGAAACGGAAAT

****
Consensus:

AATCAAGGAAATCAAACGCCGCCAAATAGCGGAAACGGAAAT

>Bacillus_Fam_1445_42_1 Nr. of seq. 1 Alignment length(with gaps) = 42
Alignment score = 0.000000
GCF_000171615.1_ASM17161v1_genomic.fna_94:31227-32096 Satlength=870 Nr of Repeats=23 RepeatLength=42 seed=TTCTCGTGCG Num.seqs=14
Similarity=0.651723
0 TTCTCGTGCGGTTGCCGCTCCATCCTGTGCGGCTTTTCCCCT

****
Consensus:

TTCTCGTGCGGTTGCCGCTCCATCCTGTGCGGCTTTTCCCCT

>Bacillus_Fam_1446_42_1 Nr. of seq. 1 Alignment length(with gaps) = 42
Alignment score = 0.000000
GCF_000177235.2_ASM17723v2_genomic.fna_1:4299704-4300137 Satlength=434 Nr of Repeats=6 RepeatLength=42 seed=TAACTTTGAT Num.seqs=4
Similarity=0.820106 0
TAACTTTGATCAAATCGCTCGTAACTCGGGCCGAATCGCGCG

****
Consensus:

TAACTTTGATCAAATCGCTCGTAACTCGGGCCGAATCGCGCG

>Bacillus_Fam_1447_42_1 Nr. of seq. 1 Alignment length(with gaps) = 42
Alignment score = 0.000000
GCF_000186125.1_Bacillus_BT1B_CT2_V1_genomic.fna_2:24462-24672
Satlength=211 Nr of Repeats=5 RepeatLength=42 seed=TCTCTTTTCA Num.seqs=5
Similarity=0.987302 0
TCTCTTTTCAGCTCATATTCTTCCGGTTCCTCTTCCACTTTC

****
Consensus:

TCTCTTTTCAGCTCATATTCTTCCGGTTCCTCTTCCACTTTC

>Bacillus_Fam_1448_42_1 Nr. of seq. 1 Alignment length(with gaps) = 42
Alignment score = 0.000000
GCF_000285535.1_ASM28553v1_genomic.fna_1:1108238-1108853 Satlength=616 Nr of Repeats=5 RepeatLength=42 seed=CTGGACCTAT Num.seqs=4
Similarity=0.920635 0
CTGGACCTATGATGGGTGGGTTTAACCCTCAAGGACCTGGTG

****
Consensus:

CTGGACCTATGATGGGTGGGTTTAACCCTCAAGGACCTGGTG

>Bacillus_Fam_1449_42_1 Nr. of seq. 1 Alignment length(with gaps) = 42
Alignment score = 0.000000

GCF_000292245.2_ASM29224v2_genomic.fna_35:20426-20996 Satlength=571 Nr of Repeats=4 RepeatLength=42 seed=CTTCTTCAGG Num.seqs=3 Similarity=0.936508
0 CTTCTTCAGGAGTCTCTACTTCCTCTCCTGGTTCTTCTGTTC

****
Consensus:

CTTCTTCAGGAGTCTCTACTTCCTCTCCTGGTTCTTCTGTTC

>Bacillus_Fam_1450_42_1 Nr. of seq. 1 Alignment length(with gaps) = 42 Alignment score = 0.000000
GCF_000294775.2_ASM29477v2_genomic.fna_1:3171456-3171624 Satlength=169 Nr of Repeats=4 RepeatLength=42 seed=CAGCCTTTTA Num.seqs=4 Similarity=0.894180 0
CAGCCTTTTATCCATCGAGCTGACCTTTTGATCCAGAACGTC

****
Consensus:

CAGCCTTTTATCCATCGAGCTGACCTTTTGATCCAGAACGTC

>Bacillus_Fam_1451_42_1 Nr. of seq. 1 Alignment length(with gaps) = 42 Alignment score = 0.000000
GCF_000305495.1_BacPsy2.0_genomic.fna_3:23376-23586 Satlength=211 Nr of Repeats=5 RepeatLength=42 seed=ATTGTTAGAG Num.seqs=5 Similarity=0.819697
0 ATTGTTAGAGCTGCGTCGATCATTCGAACAACGACGGCGACG

****
Consensus:

ATTGTTAGAGCTGCGTCGATCATTCGAACAACGACGGCGACG

>Bacillus_Fam_1452_42_1 Nr. of seq. 1 Alignment length(with gaps) = 42 Alignment score = 0.000000
GCF_000331575.1_ASM33157v1_genomic.fna_1:1325868-1326120 Satlength=253 Nr of Repeats=6 RepeatLength=42 seed=TTCGCTTTCG Num.seqs=6 Similarity=0.794709 0
TTCGCTTTCGGGTTTGCTTTTGGGTCTACCTTCGAATTTCCG

****
Consensus:

TTCGCTTTCGGGTTTGCTTTTGGGTCTACCTTCGAATTTCCG

>Bacillus_Fam_1453_42_1 Nr. of seq. 1 Alignment length(with gaps) = 42 Alignment score = 0.000000
GCF_000331575.1_ASM33157v1_genomic.fna_3:40144-40312 Satlength=169 Nr of Repeats=4 RepeatLength=42 seed=TCAAGAATCG Num.seqs=4 Similarity=0.931217
0 TCAAGAATCGCGATGAAATGTCCGGTCGTACAATAGACGAGC

****
Consensus:

TCAAGAATCGCGATGAAATGTCCGGTCGTACAATAGACGAGC

>Bacillus_Fam_1454_42_1 Nr. of seq. 1 Alignment length(with gaps) = 42
Alignment score = 0.000000
GCF_000374345.1_ASM37434v1_genomic.fna_4:87852-88188 Satlength=337 Nr of Repeats=8 RepeatLength=42 seed=TGCGTGAAGT Num.seqs=8 Similarity=0.794785
0   TGCGTGAAGTATTGCGGTTTCTTGAAGTANAGCGGCTCTGCT

****
Consensus:

TGCGTGAAGTATTGCGGTTTCTTGAAGTANAGCGGCTCTGCT

>Bacillus_Fam_1455_42_1 Nr. of seq. 1 Alignment length(with gaps) = 42
Alignment score = 0.000000
GCF_000374565.1_ASM37456v1_genomic.fna_6:25006-25384 Satlength=379 Nr of Repeats=9 RepeatLength=42 seed=CAACCAAGAG Num.seqs=9 Similarity=0.970018
0   CAACCAAGAGAAGTGGCCCCTGGAGAGGTACCTTCTGAAGCT

****
Consensus:

CAACCAAGAGAAGTGGCCCCTGGAGAGGTACCTTCTGAAGCT

>Bacillus_Fam_1456_42_1 Nr. of seq. 1 Alignment length(with gaps) = 42
Alignment score = 0.000000
GCF_000374565.1_ASM37456v1_genomic.fna_11:44641-44941 Satlength=301 Nr of Repeats=8 RepeatLength=42 seed=GATGATGGTG Num.seqs=6 Similarity=0.862434
0   GATGATGGTGGCCATGCTCATGGTGATGATGCTCACGTTCAT

****
Consensus:

GATGATGGTGGCCATGCTCATGGTGATGATGCTCACGTTCAT

>Bacillus_Fam_1457_42_1 Nr. of seq. 1 Alignment length(with gaps) = 42
Alignment score = 0.000000
GCF_000408885.1_ASM40888v1_genomic.fna_1:3192978-3193251 Satlength=274 Nr of Repeats=7 RepeatLength=42 seed=AAGCGCTTGT Num.seqs=6 Similarity=0.811640 0
AAGCGCTTGTCGACCTCAACGAAACGTGCGTCCACTTCATCG

****
Consensus:

AAGCGCTTGTCGACCTCAACGAAACGTGCGTCCACTTCATCG

>Bacillus_Fam_1458_42_1 Nr. of seq. 1 Alignment length(with gaps) = 42
Alignment score = 0.000000
GCF_000429685.1_ASM42968v1_genomic.fna_11:127207-127375 Satlength=169 Nr of Repeats=4 RepeatLength=42 seed=TCTCCAGAGT Num.seqs=4 Similarity=0.888889
0   TCTCCAGAGTCATCATCGTTTCCGTCATCCGGATTCGAATCA

****

Consensus:

TCTCCAGAGTCATCATCGTTTCCGTCATCCGGATTCGAATCA

>Bacillus_Fam_1459_42_1 Nr. of seq. 1 Alignment length(with gaps) = 42
Alignment score = 0.000000
GCF_000429705.1_ASM42970v1_genomic.fna_1:1926382-1926520 Satlength=139 Nr of Repeats=4 RepeatLength=42 seed=TTGTTGTTCT Num.seqs=3 Similarity=0.830688 0
TTGTTGTTCTGGTTGTTATTTGGATCCTGATTGTTCCATTGG

****
Consensus:

TTGTTGTTCTGGTTGTTATTTGGATCCTGATTGTTCCATTGG

>Bacillus_Fam_1460_42_1 Nr. of seq. 1 Alignment length(with gaps) = 42
Alignment score = 0.000000
GCF_000429725.1_ASM42972v1_genomic.fna_9:71563-71857 Satlength=295 Nr of Repeats=7 RepeatLength=42 seed=CCGGCGGCGG Num.seqs=7 Similarity=0.869992
0 CCGGCGGCGGGAACAACGGGGCTGACAATGGCGCCAACAACG

****
Consensus:

CCGGCGGCGGGAACAACGGGGCTGACAATGGCGCCAACAACG

>Bacillus_Fam_1461_42_1 Nr. of seq. 1 Alignment length(with gaps) = 42
Alignment score = 0.000000
GCF_000430785.1_ASM43078v1_genomic.fna_16:70466-70970 Satlength=505 Nr of Repeats=4 RepeatLength=42 seed=GGAGGATCTT Num.seqs=3 Similarity=0.978836
0 GGAGGATCTTCTCACGCGGAAGGATCGAACACAACAGCCAAT

****
Consensus:

GGAGGATCTTCTCACGCGGAAGGATCGAACACAACAGCCAAT

>Bacillus_Fam_1462_42_1 Nr. of seq. 1 Alignment length(with gaps) = 42
Alignment score = 0.000000
GCF_000430785.1_ASM43078v1_genomic.fna_27:22202-22412 Satlength=211 Nr of Repeats=5 RepeatLength=42 seed=CTGTTCAACA Num.seqs=5 Similarity=0.917460
0 CTGTTCAACAGCAATCAGCTCAACAAGAAAAAACTCAGCAGC

****
Consensus:

CTGTTCAACAGCAATCAGCTCAACAAGAAAAAACTCAGCAGC

>Bacillus_Fam_1463_42_1 Nr. of seq. 1 Alignment length(with gaps) = 42
Alignment score = 0.000000
GCF_000474275.1_Bmar1.0_genomic.fna_50:55500-55668 Satlength=169 Nr of Repeats=4 RepeatLength=42 seed=TCTGCTCTTC Num.seqs=4 Similarity=0.962963
0 TCTGCTCTTCGACTTTTATAAATCTTTCATTCCTCTTATAAC

****
Consensus:

TCTGCTCTTCGACTTTTATAAATCTTTCATTCCTCTTATAAC

>Bacillus_Fam_1464_42_1 Nr. of seq. 1 Alignment length(with gaps) = 42
Alignment score = 0.000000
GCF_000709935.2_ASM70993v2_genomic.fna_1:258653-258947 Satlength=295 Nr of Repeats=7 RepeatLength=42 seed=CGCTCAGCTT Num.seqs=7 Similarity=0.853364
0 CGCTCAGCTTCTTGTTTAGCAGCAGCCTGTTCTTGAGCTTTT

****
Consensus:

CGCTCAGCTTCTTGTTTAGCAGCAGCCTGTTCTTGAGCTTTT

>Bacillus_Fam_1465_42_1 Nr. of seq. 1 Alignment length(with gaps) = 42
Alignment score = 0.000000
GCF_000709935.2_ASM70993v2_genomic.fna_13:94783-94999 Satlength=217 Nr of Repeats=5 RepeatLength=42 seed=CAGGTGAAGG Num.seqs=4 Similarity=0.899471
0 CAGGTGAAGGAGCCGGAGAAGGCGAAGGCCAGGGCGGCGGAA

****
Consensus:

CAGGTGAAGGAGCCGGAGAAGGCGAAGGCCAGGGCGGCGGAA

>Bacillus_Fam_1466_42_1 Nr. of seq. 1 Alignment length(with gaps) = 42
Alignment score = 0.000000
GCF_000712615.1_ASM71261v1_genomic.fna_10:48131-48341 Satlength=211 Nr of Repeats=5 RepeatLength=42 seed=GGATTCGTAC Num.seqs=5 Similarity=0.974603
0 GGATTCGTACCACCTTGGTTATTTTCGTTTCCACCATTTGGT

****
Consensus:

GGATTCGTACCACCTTGGTTATTTTCGTTTCCACCATTTGGT

>Bacillus_Fam_1467_42_1 Nr. of seq. 1 Alignment length(with gaps) = 42
Alignment score = 0.000000
GCF_000724485.1_ASM72448v1_genomic.fna_1:1410335-1410587 Satlength=253 Nr of Repeats=4 RepeatLength=42 seed=AAGAACTTTC Num.seqs=3 Similarity=0.682171 0
AAGAACTTTCTTCCTGCCAAGAAAAAGATTCCTCTTGGGAAA

****
Consensus:

AAGAACTTTCTTCCTGCCAAGAAAAAGATTCCTCTTGGGAAA

>Bacillus_Fam_1468_42_1 Nr. of seq. 1 Alignment length(with gaps) = 42
Alignment score = 0.000000

GCF_000724485.1_ASM72448v1_genomic.fna_1:2977273-2977777 Satlength=505 Nr of Repeats=12 RepeatLength=42 seed=TGTTCAGTCT Num.seqs=12 Similarity=0.831841 0
TGTTCAGTCTTGATCGCACCGATTTCTTTCCGCATTTCTGAC

****
Consensus:

TGTTCAGTCTTGATCGCACCGATTTCTTTCCGCATTTCTGAC

>Bacillus_Fam_1469_42_1 Nr. of seq. 1 Alignment length(with gaps) = 42 Alignment score = 0.000000
GCF_000751775.1_A1A_genomic.fna_90:16-268 Satlength=253 Nr of Repeats=6 RepeatLength=42 seed=TCAAACCGTT Num.seqs=6 Similarity=0.788360
0   TCAAACCGTTTATCCATGCTGTTCAGACGGCCATCCACATCA

****
Consensus:

TCAAACCGTTTATCCATGCTGTTCAGACGGCCATCCACATCA

>Bacillus_Fam_1470_42_1 Nr. of seq. 1 Alignment length(with gaps) = 42 Alignment score = 0.000000
GCF_000752395.1_Bacillus_andreraoultii_genomic.fna_31:29723-29891 Satlength=169 Nr of Repeats=4 RepeatLength=42 seed=AGCCACTTGT Num.seqs=4 Similarity=1.000000 0
AGCCACTTGTAAATTTATCGCTCCTTCTTTCACTGGAACGCG

****
Consensus:

AGCCACTTGTAAATTTATCGCTCCTTCTTTCACTGGAACGCG

>Bacillus_Fam_1471_42_1 Nr. of seq. 1 Alignment length(with gaps) = 42 Alignment score = 0.000000
GCF_000769555.1_ASM76955v1_genomic.fna_1:2684106-2684577 Satlength=472 Nr of Repeats=10 RepeatLength=42 seed=GCTCTTCTTC Num.seqs=7 Similarity=0.879063 0
GCTCTTCTTCTTCCCGCTCCCTCAGAATCGGGGCAGTCTCCG

****
Consensus:

GCTCTTCTTCTTCCCGCTCCCTCAGAATCGGGGCAGTCTCCG

>Bacillus_Fam_1472_42_1 Nr. of seq. 1 Alignment length(with gaps) = 42 Alignment score = 0.000000
GCF_000832605.1_ASM83260v1_genomic.fna_2:334361-334613 Satlength=253 Nr of Repeats=6 RepeatLength=42 seed=GTCAAACGCA Num.seqs=6 Similarity=0.894180
0   GTCAAACGCAAAATGGTGGTAGCCAGCCAGGTGGAAGTGACG

****
Consensus:

GTCAAACGCAAAATGGTGGTAGCCAGCCAGGTGGAAGTGACG

>Bacillus_Fam_1473_42_1 Nr. of seq. 1 Alignment length(with gaps) = 42
Alignment score = 0.000000
GCF_000934845.1_ASM93484v1_genomic.fna_23:28230-28440 Satlength=211 Nr of Repeats=5 RepeatLength=42 seed=GGAGGACCGG Num.seqs=5 Similarity=0.787302
0 GGAGGACCGGGTGGNTTTCCAGGCANTCCAGGTGGNTTCCCA

****
Consensus:

GGAGGACCGGGTGGNTTTCCAGGCANTCCAGGTGGNTTCCCA

>Bacillus_Fam_1474_42_1 Nr. of seq. 1 Alignment length(with gaps) = 42
Alignment score = 0.000000
GCF_000948175.1_BFA_2_A_genomic.fna_157:17151-17403 Satlength=253 Nr of Repeats=6 RepeatLength=42 seed=GATGTGGGAG Num.seqs=6 Similarity=0.908995
0 GATGTGGGAGGCCTAAAACAAAATGTCGAAATGCTAAAAGAC

****
Consensus:

GATGTGGGAGGCCTAAAACAAAATGTCGAAATGCTAAAAGAC

>Bacillus_Fam_1475_42_1 Nr. of seq. 1 Alignment length(with gaps) = 42
Alignment score = 0.000000
GCF_001278705.1_ASM127870v1_genomic.fna_1:1934684-1934978 Satlength=295 Nr of Repeats=7 RepeatLength=42 seed=CCTGCTACGT Num.seqs=7 Similarity=0.918367 0
CCTGCTACGTTTGGCTGTCCGTGATACGGATTTTCTGATGCG

****
Consensus:

CCTGCTACGTTTGGCTGTCCGTGATACGGATTTTCTGATGCG

>Bacillus_Fam_1476_42_1 Nr. of seq. 1 Alignment length(with gaps) = 42
Alignment score = 0.000000
GCF_001278705.1_ASM127870v1_genomic.fna_1:3048777-3049027 Satlength=251 Nr of Repeats=6 RepeatLength=42 seed=GGAATTGCAC Num.seqs=4 Similarity=0.809524 0
GGAATTGCACCGATAACAACGATAATTGCACCGTTGACCTTG

****
Consensus:

GGAATTGCACCGATAACAACGATAATTGCACCGTTGACCTTG

>Bacillus_Fam_1477_42_1 Nr. of seq. 1 Alignment length(with gaps) = 42
Alignment score = 0.000000
GCF_001420715.1_ASM142071v1_genomic.fna_24:439598-439808 Satlength=211 Nr of Repeats=5 RepeatLength=42 seed=GCTTTCGTTT Num.seqs=5

Similarity=0.844444 0
GCTTTCGTTTGTGATGCGTAGAGTATCTCGAATCTTCCTCGT

****
Consensus:

GCTTTCGTTTGTGATGCGTAGAGTATCTCGAATCTTCCTCGT

>Bacillus_Fam_1478_42_1 Nr. of seq. 1 Alignment length(with gaps) = 42
Alignment score = 0.000000
GCF_001439635.1_ASM143963v1_genomic.fna_173:188835-189045 Satlength=211
Nr of Repeats=4 RepeatLength=42 seed=CAGGAGTCGG Num.seqs=3
Similarity=0.873016 0
CAGGAGTCGGACAATTAGTTGACGGACAAAAAACAGCGGTTG

****
Consensus:

CAGGAGTCGGACAATTAGTTGACGGACAAAAAACAGCGGTTG

>Bacillus_Fam_1479_42_1 Nr. of seq. 1 Alignment length(with gaps) = 42
Alignment score = 0.000000
GCF_001484965.1_ASM148496v1_genomic.fna_5:164270-164606 Satlength=337 Nr of Repeats=7 RepeatLength=42 seed=GAGCTTCAAT Num.seqs=6
Similarity=0.928042
0 GAGCTTCAATCCGACAAGAGGAGCGCTCATTCGGACAGGAGG

****
Consensus:

GAGCTTCAATCCGACAAGAGGAGCGCTCATTCGGACAGGAGG

>Bacillus_Fam_1480_42_1 Nr. of seq. 1 Alignment length(with gaps) = 42
Alignment score = 0.000000
GCF_001484965.1_ASM148496v1_genomic.fna_6:187378-187747 Satlength=370 Nr of Repeats=9 RepeatLength=42 seed=TGATCGGTAA Num.seqs=6
Similarity=0.904762
0 TGATCGGTAAATCCTCTGACATGAGCGATACCATCTCTCACT

****
Consensus:

TGATCGGTAAATCCTCTGACATGAGCGATACCATCTCTCACT

>Bacillus_Fam_1481_42_1 Nr. of seq. 1 Alignment length(with gaps) = 42
Alignment score = 0.000000
GCF_001578185.1_ASM157818v1_genomic.fna_1:1320901-1321126 Satlength=226
Nr of Repeats=4 RepeatLength=42 seed=ATTTCTGCTA Num.seqs=3
Similarity=0.894180 0
ATTTCTGCTAGTGCGCCTTTGGTTTCTGCATGTAGATCTCCG

****
Consensus:

ATTTCTGCTAGTGCGCCTTTGGTTTCTGCATGTAGATCTCCG

>Bacillus_Fam_1482_42_1 Nr. of seq. 1 Alignment length(with gaps) = 42 Alignment score = 0.000000
GCF_001587375.1_ASM158737v1_genomic.fna_134:11775-12027 Satlength=253 Nr of Repeats=6 RepeatLength=42 seed=TTGAAGTCGA Num.seqs=6 Similarity=0.936508
0   TTGAAGTCGACAAACGGTTTGATGCTGTTGATCAAAAATTTA

****
Consensus:

TTGAAGTCGACAAACGGTTTGATGCTGTTGATCAAAAATTTA

>Bacillus_Fam_1483_42_1 Nr. of seq. 1 Alignment length(with gaps) = 42 Alignment score = 0.000000
GCF_001591425.1_ASM159142v1_genomic.fna_25:20219-20387 Satlength=169 Nr of Repeats=4 RepeatLength=42 seed=TTATTTAACA Num.seqs=4 Similarity=0.941799
0   TTATTTAACAGGTAGCAGAATTAACATAACAGAGTTTCGATT

****
Consensus:

TTATTTAACAGGTAGCAGAATTAACATAACAGAGTTTCGATT

>Bacillus_Fam_1484_42_1 Nr. of seq. 1 Alignment length(with gaps) = 42 Alignment score = 0.000000
GCF_001591425.1_ASM159142v1_genomic.fna_72:22227-22647 Satlength=421 Nr of Repeats=10 RepeatLength=42 seed=GTTGGCCGAT Num.seqs=10 Similarity=0.779189 0
GTTGGCCGATATATCGATTGTGGTGGCGCAATAACATGCTCA

****
Consensus:

GTTGGCCGATATATCGATTGTGGTGGCGCAATAACATGCTCA

>Bacillus_Fam_1485_42_1 Nr. of seq. 1 Alignment length(with gaps) = 42 Alignment score = 0.000000
GCF_001591425.1_ASM159142v1_genomic.fna_137:8995-9163 Satlength=169 Nr of Repeats=4 RepeatLength=42 seed=AAGTGCGTCA Num.seqs=4 Similarity=0.822997
0   AAGTGCGTCAATTACTTATCCAGCTGCGTCAAAAAGTTCGCC

****
Consensus:

AAGTGCGTCAATTACTTATCCAGCTGCGTCAAAAAGTTCGCC

>Bacillus_Fam_1486_42_1 Nr. of seq. 1 Alignment length(with gaps) = 42 Alignment score = 0.000000
GCF_001591445.1_ASM159144v1_genomic.fna_22:81979-82189 Satlength=211 Nr of Repeats=5 RepeatLength=42 seed=TTTGATAAAA Num.seqs=3

Similarity=0.957672
0   TTTGATAAAATTGAAACTCGACTTAATAATATGGATCAGCGG

****
Consensus:

TTTGATAAAATTGAAACTCGACTTAATAATATGGATCAGCGG

>Bacillus_Fam_1487_42_1 Nr. of seq. 1 Alignment length(with gaps) = 42
Alignment score = 0.000000
GCF_001591585.1_ASM159158v1_genomic.fna_13:66350-66497 Satlength=148 Nr of Repeats=4 RepeatLength=42 seed=AGAAACGCCA Num.seqs=3
Similarity=0.809524
0   AGAAACGCCAGGAGATAACGAAGAAACTCCTGGAGATAATGA

****
Consensus:

AGAAACGCCAGGAGATAACGAAGAAACTCCTGGAGATAATGA

>Bacillus_Fam_1488_42_1 Nr. of seq. 1 Alignment length(with gaps) = 42
Alignment score = 0.000000
GCF_001591665.1_ASM159166v1_genomic.fna_15:56816-56984 Satlength=169 Nr of Repeats=5 RepeatLength=42 seed=GCGAATTTCC Num.seqs=3
Similarity=0.542929
0   GCGAATTTCCGNGCCCAATGNGCGAATTATCGNACCTTACTN

****
Consensus:

GCGAATTTCCGNGCCCAATGNGCGAATTATCGNACCTTACTN

>Bacillus_Fam_1489_42_1 Nr. of seq. 1 Alignment length(with gaps) = 42
Alignment score = 0.000000
GCF_001592005.1_ASM159200v1_genomic.fna_21:10147-10315 Satlength=169 Nr of Repeats=4 RepeatLength=42 seed=GTGATCATTC Num.seqs=4
Similarity=0.915344
0   GTGATCATTCGGCCGGCGGTTTGAAAATCATTGGTTGATGAC

****
Consensus:

GTGATCATTCGGCCGGCGGTTTGAAAATCATTGGTTGATGAC

>Bacillus_Fam_1490_42_1 Nr. of seq. 1 Alignment length(with gaps) = 42
Alignment score = 0.000000
GCF_001636335.1_ASM163633v1_genomic.fna_1:2763111-2763578 Satlength=468 Nr of Repeats=7 RepeatLength=42 seed=TATTCGCCGT Num.seqs=5
Similarity=0.638636 0
TATTCGCCGTTCTNCTCNATTTATTGGACATTANCNGCACTT

****
Consensus:

TATTCGCCGTTCTNCTCNATTTATTGGACATTANCNGCACTT

>Bacillus_Fam_1491_42_1 Nr. of seq. 1 Alignment length(with gaps) = 42
Alignment score = 0.000000
GCF_001636425.1_ASM163642v1_genomic.fna_1:2163616-2163826 Satlength=211
Nr of Repeats=5 RepeatLength=42 seed=ACATATCTAT Num.seqs=5
Similarity=0.923810 0
ACATATCTATGCTGATAAAAGGCAATTACCCCCTTTAAGTAG

****
Consensus:

ACATATCTATGCTGATAAAAGGCAATTACCCCCTTTAAGTAG

>Bacillus_Fam_1492_42_1 Nr. of seq. 1 Alignment length(with gaps) = 42
Alignment score = 0.000000
GCF_001645685.2_ASM164568v2_genomic.fna_1:3182234-3182381 Satlength=148
Nr of Repeats=4 RepeatLength=42 seed=CTCGCCAATT Num.seqs=3
Similarity=0.772727 0
CTCGCCAATTCGGCACTTTTACTCGCGAGTTTNAGCTGTTTA

****
Consensus:

CTCGCCAATTCGGCACTTTTACTCGCGAGTTTNAGCTGTTTA

>Bacillus_Fam_1493_42_1 Nr. of seq. 1 Alignment length(with gaps) = 42
Alignment score = 0.000000
GCF_001645685.2_ASM164568v2_genomic.fna_1:3510154-3511004 Satlength=851
Nr of Repeats=19 RepeatLength=42 seed=TTTTTCGGCC Num.seqs=12
Similarity=0.854738 18
TTCGACCTGCATCCACTTTTTTTCGGCCTGCACTCAACTTAA

****
Consensus:

TTTTTCGGCCTGCACTCAACTTAATTCGACCTGCATCCACTT

>Bacillus_Fam_1494_42_1 Nr. of seq. 1 Alignment length(with gaps) = 42
Alignment score = 0.000000
GCF_001645705.1_ASM164570v1_genomic.fna_60:510572-511860 Satlength=1289
Nr of Repeats=31 RepeatLength=42 seed=GTTGCGCAAT Num.seqs=25
Similarity=0.780465 0
GTTGCGCAATANGGTANTGTTTTAGCGCAATTCCGTGTGNAA

****
Consensus:

GTTGCGCAATANGGTANTGTTTTAGCGCAATTCCGTGTGNAA

>Bacillus_Fam_1495_42_1 Nr. of seq. 1 Alignment length(with gaps) = 42
Alignment score = 0.000000

GCF_001654695.1_ASM165469v1_genomic.fna_17:33252-33504 Satlength=253 Nr of Repeats=6 RepeatLength=42 seed=TCGTAGCATT Num.seqs=6 Similarity=0.881481
0   TCGTAGCATTTTTTCTTCTTACAGCAAAAATCTACGTGATCG

****
Consensus:

TCGTAGCATTTTTTCTTCTTACAGCAAAAATCTACGTGATCG

>Bacillus_Fam_1496_42_1 Nr. of seq. 1 Alignment length(with gaps) = 42 Alignment score = 0.000000
GCF_001712755.1_ASM171275v1_genomic.fna_35:220736-221801 Satlength=1066 Nr of Repeats=19 RepeatLength=42 seed=TTTGGTCCCA Num.seqs=16 Similarity=0.741534 0
TTTGGTCCCATATATGCTGGCATTGTTTGTGGGAAGTTTGCA

****
Consensus:

TTTGGTCCCATATATGCTGGCATTGTTTGTGGGAAGTTTGCA

>Bacillus_Fam_1497_42_1 Nr. of seq. 1 Alignment length(with gaps) = 42 Alignment score = 0.000000
GCF_001742425.1_ASM174242v1_genomic.fna_13:64716-64926 Satlength=211 Nr of Repeats=5 RepeatLength=42 seed=GAAGTAGAAG Num.seqs=5 Similarity=0.761905
0   GAAGTAGAAGATCTAAAAGCAGGTCAAACGAAGTTAACAACG

****
Consensus:

GAAGTAGAAGATCTAAAAGCAGGTCAAACGAAGTTAACAACG

>Bacillus_Fam_1498_42_1 Nr. of seq. 1 Alignment length(with gaps) = 42 Alignment score = 0.000000
GCF_001742425.1_ASM174242v1_genomic.fna_37:154-322 Satlength=169 Nr of Repeats=4 RepeatLength=42 seed=TTCTTCTTCA Num.seqs=4 Similarity=0.874031
0   TTCTTCTTCAGCAACGGTGTTAACTTCATCCTCAGGAAGGTA

****
Consensus:

TTCTTCTTCAGCAACGGTGTTAACTTCATCCTCAGGAAGGTA

>Bacillus_Fam_1499_42_1 Nr. of seq. 1 Alignment length(with gaps) = 42 Alignment score = 0.000000
GCF_001877785.1_PlanoSAMM_genomic.fna_72:16191-16527 Satlength=337 Nr of Repeats=8 RepeatLength=42 seed=TACTTACTTG Num.seqs=8 Similarity=0.969388
0   TACTTACTTGTTGACCATTCTCCACTCTTTCCAAACGTTCGT

****
Consensus:

TACTTACTTGTTGACCATTCTCCACTCTTTCCAAACGTTCGT

>Bacillus_Fam_1500_42_1 Nr. of seq. 1 Alignment length(with gaps) = 42
Alignment score = 0.000000
GCF_002019635.1_ASM201963v1_genomic.fna_1:836663-836873 Satlength=211 Nr of Repeats=5 RepeatLength=42 seed=AGTTCTTTCG Num.seqs=5
Similarity=0.898413
0   AGTTCTTTCGTGTCTGCTTTTAATAGCCGGACGTCTCCTTTT

****
Consensus:

AGTTCTTTCGTGTCTGCTTTTAATAGCCGGACGTCTCCTTTT

>Bacillus_Fam_1501_42_1 Nr. of seq. 1 Alignment length(with gaps) = 42
Alignment score = 0.000000
GCF_002019665.1_ASM201966v1_genomic.fna_1:2650891-2651059 Satlength=169
Nr of Repeats=4 RepeatLength=42 seed=GTCACTTACA Num.seqs=4
Similarity=0.873016   0
GTCACTTACATTGGTTTTCAATTCACCAACATCGGTTGTCAG

****
Consensus:

GTCACTTACATTGGTTTTCAATTCACCAACATCGGTTGTCAG

>Bacillus_Fam_1502_42_1 Nr. of seq. 1 Alignment length(with gaps) = 42
Alignment score = 0.000000
GCF_900103955.1_IMG-taxon_2617270767_annotated_assembly_genomic.fna_4:117-426 Satlength=310
Nr of Repeats=4 RepeatLength=42 seed=TTTACTCCGA Num.seqs=3
Similarity=0.788360   0
TTTACTCCGATCCTCTTATACTTTACTAACAACCACCTATAC

****
Consensus:

TTTACTCCGATCCTCTTATACTTTACTAACAACCACCTATAC

>Bacillus_Fam_1503_42_1 Nr. of seq. 1 Alignment length(with gaps) = 42
Alignment score = 0.000000
GCF_900104555.1_IMG-taxon_2651870172_annotated_assembly_genomic.fna_22:838320-838572
Satlength=253 Nr of Repeats=6 RepeatLength=42 seed=GCTACTGAAG Num.seqs=6
Similarity=0.904762   0
GCTACTGAAGACACTCATTCTCATGACGATGAAGAGAGTCAT

****
Consensus:

GCTACTGAAGACACTCATTCTCATGACGATGAAGAGAGTCAT

>Bacillus_Fam_1504_42_1 Nr. of seq. 1 Alignment length(with gaps) = 42
Alignment score = 0.000000
GCF_900107275.1_IMG-
taxon_2675903024_annotated_assembly_genomic.fna_12:79980-80484
Satlength=505 Nr of Repeats=12 RepeatLength=42 seed=CGGAAGACGG
Num.seqs=12 Similarity=0.923040 0
CGGAAGACGGGGAGAACGCCGAAGGAGAAGCGCCAAGTCCGC

****
Consensus:

CGGAAGACGGGGAGAACGCCGAAGGAGAAGCGCCAAGTCCGC

>Bacillus_Fam_1505_42_1 Nr. of seq. 1 Alignment length(with gaps) = 42
Alignment score = 0.000000
GCF_900107275.1_IMG-
taxon_2675903024_annotated_assembly_genomic.fna_35:69303-69513
Satlength=211 Nr of Repeats=5 RepeatLength=42 seed=AGAGCAGTGC Num.seqs=5
Similarity=0.923810 0
AGAGCAGTGCGACAGCGCTGCGTCGTGCGAAGCACAAAAAAG

****
Consensus:

AGAGCAGTGCGACAGCGCTGCGTCGTGCGAAGCACAAAAAAG

>Bacillus_Fam_1506_42_1 Nr. of seq. 1 Alignment length(with gaps) = 42
Alignment score = 0.000000
GCF_900107275.1_IMG-
taxon_2675903024_annotated_assembly_genomic.fna_42:1093335-1093503
Satlength=169 Nr of Repeats=4 RepeatLength=42 seed=GCCGAATCCT Num.seqs=4
Similarity=0.925926 0
GCCGAATCCTGGAGTCGCATACGGAGAAACTGGACGAACACG

****
Consensus:

GCCGAATCCTGGAGTCGCATACGGAGAAACTGGACGAACACG

>Bacillus_Fam_1507_42_1 Nr. of seq. 1 Alignment length(with gaps) = 42
Alignment score = 0.000000
GCF_900111815.1_PRJEB17078_genomic.fna_1:571953-572162 Satlength=210 Nr
of Repeats=5 RepeatLength=42 seed=ATCAGCCAAC Num.seqs=4
Similarity=0.968254
0   ATCAGCCAACTTCGGGCGAGAATCAACCAAGCTTGGCGAAAA

****
Consensus:

ATCAGCCAACTTCGGGCGAGAATCAACCAAGCTTGGCGAAAA

>Bacillus_Fam_1508_42_1 Nr. of seq. 1 Alignment length(with gaps) = 42
Alignment score = 0.000000

GCF_900142675.1_IMG-
taxon_2654588196_annotated_assembly_genomic.fna_9:331825-331993
Satlength=169 Nr of Repeats=4 RepeatLength=42 seed=GAAAAGCGTT Num.seqs=4
Similarity=0.931217   0
GAAAAGCGTTTTGACGGTTTAGAAGGGCGTATTGATGGGCTA

****
Consensus:

GAAAAGCGTTTTGACGGTTTAGAAGGGCGTATTGATGGGCTA

>Bacillus_Fam_1509_41_1 Nr. of seq. 1 Alignment length(with gaps) = 41
Alignment score = 0.000000
GCF_000285535.1_ASM28553v1_genomic.fna_2:783130-783602 Satlength=473 Nr
of Repeats=11 RepeatLength=41 seed=TTTGCGTCTT Num.seqs=9
Similarity=0.820106
0   TTTGCGTCTTCTGATGAGNGATTTGCACCGGTTTGGTGGCA

****
Consensus:

TTTGCGTCTTCTGATGAGNGATTTGCACCGGTTTGGTGGCA

>Bacillus_Fam_1510_41_1 Nr. of seq. 1 Alignment length(with gaps) = 41
Alignment score = 0.000000
GCF_000331575.1_ASM33157v1_genomic.fna_1:1704740-1704945 Satlength=206 Nr
of Repeats=4 RepeatLength=41 seed=AATATTGGAA Num.seqs=3
Similarity=0.869919   0
AATATTGGAATTTGCTTCTGAAAATTTCAGGGTTAGTTCCC

****
Consensus:

AATATTGGAATTTGCTTCTGAAAATTTCAGGGTTAGTTCCC

>Bacillus_Fam_1511_41_1 Nr. of seq. 1 Alignment length(with gaps) = 41
Alignment score = 0.000000
GCF_000374565.1_ASM37456v1_genomic.fna_7:121779-122039 Satlength=261 Nr
of Repeats=6 RepeatLength=40 seed=TATACGAGAG Num.seqs=4
Similarity=0.794038
0   TATACGAGAGGTGGAGCTGCTATAAGTGAGTGGCACCTGTC

****
Consensus:

TATACGAGAGGTGGAGCTGCTATAAGTGAGTGGCACCTGTC

>Bacillus_Fam_1512_41_1 Nr. of seq. 1 Alignment length(with gaps) = 41
Alignment score = 0.000000
GCF_000401235.1_BacNeaAAU1_genomic.fna_215:29479-29643 Satlength=165 Nr
of Repeats=4 RepeatLength=41 seed=GATTTATCAG Num.seqs=4
Similarity=0.934959
0   GATTTATCAGCCAAAAACTCACTCTTTTCAGCCAAACTTCC

****
Consensus:

GATTTATCAGCCAAAAACTCACTCTTTTCAGCCAAACTTCC

>Bacillus_Fam_1513_41_1 Nr. of seq. 1 Alignment length(with gaps) = 41 Alignment score = 0.000000
GCF_000429705.1_ASM42970v1_genomic.fna_2:114683-114847 Satlength=165 Nr of Repeats=5 RepeatLength=41 seed=TCGATCGATA Num.seqs=3 Similarity=0.956640
0 TCGATCGATAAATCTCAAAGTCGATGAATAAACCAGGCAAC

****
Consensus:

TCGATCGATAAATCTCAAAGTCGATGAATAAACCAGGCAAC

>Bacillus_Fam_1514_41_1 Nr. of seq. 1 Alignment length(with gaps) = 41 Alignment score = 0.000000
GCF_000429705.1_ASM42970v1_genomic.fna_6:23293-23457 Satlength=165 Nr of Repeats=4 RepeatLength=41 seed=TTTATCAATC Num.seqs=4 Similarity=0.853659
0 TTTATCAATCACTTTCCCCTCGTTATCCATCACTTTCACAA

****
Consensus:

TTTATCAATCACTTTCCCCTCGTTATCCATCACTTTCACAA

>Bacillus_Fam_1515_41_1 Nr. of seq. 1 Alignment length(with gaps) = 41 Alignment score = 0.000000
GCF_000429725.1_ASM42972v1_genomic.fna_2:355518-356110 Satlength=593 Nr of Repeats=5 RepeatLength=41 seed=TGGACAATTC Num.seqs=3 Similarity=0.869919
0 TGGACAATTCGGCGGGATTGTGACCAAGCGGCGGCCAAGTC

****
Consensus:

TGGACAATTCGGCGGGATTGTGACCAAGCGGCGGCCAAGTC

>Bacillus_Fam_1516_41_1 Nr. of seq. 1 Alignment length(with gaps) = 41 Alignment score = 0.000000
GCF_000504145.1_SOAPdenovo_for_version2.04_of_Bacillus_sp._FJAT-14515_genome_genomic.fna_1:2086005-2086495 Satlength=491 Nr of Repeats=12 RepeatLength=41 seed=TTGCGGGCCG Num.seqs=10 Similarity=0.9407 0 TTGCGGGCCGGACCTAACCCTTTACATGCCGATTTCTTATA

****
Consensus:

TTGCGGGCCGGACCTAACCCTTTACATGCCGATTTCTTATA

>Bacillus_Fam_1517_41_1 Nr. of seq. 1 Alignment length(with gaps) = 41
Alignment score = 0.000000
GCF_000504145.1_SOAPdenovo_for_version2.04_of_Bacillus_sp._FJAT-14515_genome_genomic.fna_1:2113011-2113379 Satlength=369 Nr of Repeats=9 RepeatLength=41 seed=CACTTTTCAC Num.seqs=8 Similarity=0.907085   0
CACTTTTCACACCAAAATCGAAGTTTGCGGGCCGGTTCAAA

****
Consensus:

CACTTTTCACACCAAAATCGAAGTTTGCGGGCCGGTTCAAA

>Bacillus_Fam_1518_41_1 Nr. of seq. 1 Alignment length(with gaps) = 41
Alignment score = 0.000000
GCF_000712595.1_ASM71259v1_genomic.fna_1:175979-176594 Satlength=616 Nr of Repeats=15 RepeatLength=41 seed=AAAAAGTGAC Num.seqs=15 Similarity=0.995664   0
AAAAAGTGACCGCATTATCACCTGTTTTGACTGAATAAACG

****
Consensus:

AAAAAGTGACCGCATTATCACCTGTTTTGACTGAATAAACG

>Bacillus_Fam_1519_41_1 Nr. of seq. 1 Alignment length(with gaps) = 41
Alignment score = 0.000000
GCF_001273755.1_ASM127375v1_genomic.fna_17:206866-207071 Satlength=206 Nr of Repeats=5 RepeatLength=41 seed=AATTCGTCCT Num.seqs=5 Similarity=0.752381   0
AATTCGTCCTACATGGNAANAATTCGACCGTAACCGGCAAN

****
Consensus:

AATTCGTCCTACATGGNAANAATTCGACCGTAACCGGCAAN

>Bacillus_Fam_1520_41_1 Nr. of seq. 1 Alignment length(with gaps) = 41
Alignment score = 0.000000
GCF_001273755.1_ASM127375v1_genomic.fna_17:3549779-3549982 Satlength=204 Nr of Repeats=5 RepeatLength=41 seed=CTGAAGGTCG Num.seqs=3 Similarity=0.913279   0
CTGAAGGTCGATTTATTCCGACCCACGGACGAATTCATTCA

****
Consensus:

CTGAAGGTCGATTTATTCCGACCCACGGACGAATTCATTCA

>Bacillus_Fam_1521_41_1 Nr. of seq. 1 Alignment length(with gaps) = 41
Alignment score = 0.000000
GCF_001636325.1_ASM163632v1_genomic.fna_1:591647-591811 Satlength=165 Nr of Repeats=4 RepeatLength=41 seed=GAGACGCAAA Num.seqs=4 Similarity=0.967480
0   GAGACGCAAAAGTAGTTGCAGCTTACGCAAAAAACAGTGGA

****
Consensus:

GAGACGCAAAAGTAGTTGCAGCTTACGCAAAAAACAGTGGA

>Bacillus_Fam_1522_41_1 Nr. of seq. 1 Alignment length(with gaps) = 41
Alignment score = 0.000000
GCF_001636325.1_ASM163632v1_genomic.fna_3:885452-885657 Satlength=206 Nr of Repeats=5 RepeatLength=41 seed=TAAATATTTG Num.seqs=5
Similarity=0.930159
0   TAAATATTTGCGTCAGCCTCAGTGATTTAAGCGTCACCATT

****
Consensus:

TAAATATTTGCGTCAGCCTCAGTGATTTAAGCGTCACCATT

>Bacillus_Fam_1523_41_1 Nr. of seq. 1 Alignment length(with gaps) = 41
Alignment score = 0.000000
GCF_001636335.1_ASM163633v1_genomic.fna_2:321955-322365 Satlength=411 Nr of Repeats=10 RepeatLength=41 seed=TGCATATATT Num.seqs=10
Similarity=0.947245 0
TGCATATATTCGCCGTCCTCAAGCAGATATTGGACAAAACC

****
Consensus:

TGCATATATTCGCCGTCCTCAAGCAGATATTGGACAAAACC

>Bacillus_Fam_1524_41_1 Nr. of seq. 1 Alignment length(with gaps) = 41
Alignment score = 0.000000
GCF_001645685.2_ASM164568v2_genomic.fna_1:1284454-1284618 Satlength=165 Nr of Repeats=4 RepeatLength=41 seed=AGGGAAGCAG Num.seqs=4
Similarity=0.983740 0
AGGGAAGCAGGACGAATAAATTCGCGGACGGACGGAAAAAG

****
Consensus:

AGGGAAGCAGGACGAATAAATTCGCGGACGGACGGAAAAAG

>Bacillus_Fam_1525_41_1 Nr. of seq. 1 Alignment length(with gaps) = 41
Alignment score = 0.000000
GCF_001645685.2_ASM164568v2_genomic.fna_1:3245887-3246338 Satlength=452 Nr of Repeats=13 RepeatLength=41 seed=GGACGAATTC Num.seqs=9
Similarity=0.881662 0
GGACGAATTCTCTCGACGGTAGGTCGAATTAAACCTGTTTG

****
Consensus:

GGACGAATTCTCTCGACGGTAGGTCGAATTAAACCTGTTTG

>Bacillus_Fam_1526_41_1 Nr. of seq. 1 Alignment length(with gaps) = 41
Alignment score = 0.000000
GCF_001645685.2_ASM164568v2_genomic.fna_1:3591693-3592229 Satlength=537
Nr of Repeats=13 RepeatLength=41 seed=GGACGGATTA Num.seqs=10
Similarity=0.819332 0
GGACGGATTATTTCATCCCAGGTCGGATTCCACGCGCTGTC

****
Consensus:

GGACGGATTATTTCATCCCAGGTCGGATTCCACGCGCTGTC

>Bacillus_Fam_1527_41_1 Nr. of seq. 1 Alignment length(with gaps) = 41
Alignment score = 0.000000
GCF_001645685.2_ASM164568v2_genomic.fna_1:3945848-3946297 Satlength=450
Nr of Repeats=11 RepeatLength=41 seed=TTCGACCTAA Num.seqs=7
Similarity=0.724622 0
TTCGACCTAAACTCCATTTTTTCCGGCCCACTATCGATTAA

****
Consensus:

TTCGACCTAAACTCCATTTTTTCCGGCCCACTATCGATTAA

>Bacillus_Fam_1528_41_1 Nr. of seq. 1 Alignment length(with gaps) = 41
Alignment score = 0.000000
GCF_001645685.2_ASM164568v2_genomic.fna_1:4091702-4091905 Satlength=204
Nr of Repeats=5 RepeatLength=41 seed=GATATAATTC Num.seqs=3
Similarity=0.738095 0
GATATAATTCCAGTTTCGGCGATATATTGCCGGTTTTCGTA

****
Consensus:

GATATAATTCCAGTTTCGGCGATATATTGCCGGTTTTCGTA

>Bacillus_Fam_1529_41_1 Nr. of seq. 1 Alignment length(with gaps) = 41
Alignment score = 0.000000
GCF_001645685.2_ASM164568v2_genomic.fna_1:4831063-4831779 Satlength=717
Nr of Repeats=18 RepeatLength=41 seed=ATATTCCGGC Num.seqs=15
Similarity=0.847309 0
ATATTCCGGCCTTACCCGCTGGTTTCCGGCCTGCTTCATCG

****
Consensus:

ATATTCCGGCCTTACCCGCTGGTTTCCGGCCTGCTTCATCG

>Bacillus_Fam_1530_41_1 Nr. of seq. 1 Alignment length(with gaps) = 41
Alignment score = 0.000000
GCF_001645685.2_ASM164568v2_genomic.fna_1:4869499-4869784 Satlength=286
Nr of Repeats=7 RepeatLength=41 seed=ATTGGGTCGA Num.seqs=5
Similarity=0.834146 0
ATTGGGTCGATTTATCTCCTGTTAGGACGATTTATCCCCGC

****
Consensus:

ATTGGGTCGATTTATCTCCTGTTAGGACGATTTATCCCCGC

>Bacillus_Fam_1531_41_1 Nr. of seq. 1 Alignment length(with gaps) = 41
Alignment score = 0.000000
GCF_001645705.1_ASM164570v1_genomic.fna_57:5429-5921 Satlength=493 Nr of Repeats=12 RepeatLength=41 seed=GTAAGGAAGT Num.seqs=12 Similarity=0.907366
0   GTAAGGAAGTCGCGCAATCCCCAGGAAAGAAGCGCAATGCA

****
Consensus:

GTAAGGAAGTCGCGCAATCCCCAGGAAAGAAGCGCAATGCA

>Bacillus_Fam_1532_41_1 Nr. of seq. 1 Alignment length(with gaps) = 41
Alignment score = 0.000000
GCF_001648575.1_ASM164857v1_genomic.fna_3:67197-67361 Satlength=165 Nr of Repeats=4 RepeatLength=41 seed=AAAAGTAGCG Num.seqs=4 Similarity=0.902439
0   AAAAGTAGCGGATAAAACCCAAAAGTTGTTGGATAAATCCC

****
Consensus:

AAAAGTAGCGGATAAAACCCAAAAGTTGTTGGATAAATCCC

>Bacillus_Fam_1533_41_1 Nr. of seq. 1 Alignment length(with gaps) = 41
Alignment score = 0.000000
GCF_002019645.1_ASM201964v1_genomic.fna_1:1580930-1581300 Satlength=371 Nr of Repeats=5 RepeatLength=41 seed=TTCTCGTGCT Num.seqs=4 Similarity=1.000000 0
TTCTCGTGCTTCAAATGGAAGCATGAGAACCGTCCCACTGC

****
Consensus:

TTCTCGTGCTTCAAATGGAAGCATGAGAACCGTCCCACTGC

>Bacillus_Fam_1534_40_1 Nr. of seq. 1 Alignment length(with gaps) = 40
Alignment score = 0.000000
BACILLUS_PSEUDALCALIPHILUS_genomic.fna_3:56177-56333 Satlength=157 Nr of Repeats=4 RepeatLength=39 seed=CATGAAGAAG Num.seqs=4 Similarity=0.905556
0   CATGAAGAAGACGAACATGAGCATCACGAGCATGAGGGGC

****
Consensus:

CATGAAGAAGACGAACATGAGCATCACGAGCATGAGGGGC

>Bacillus_Fam_1535_40_1 Nr. of seq. 1 Alignment length(with gaps) = 40
Alignment score = 0.000000

GCF_000285535.1_ASM28553v1_genomic.fna_3:14224-14505 Satlength=282 Nr of Repeats=7 RepeatLength=40 seed=GATTTGCGTC Num.seqs=6 Similarity=0.797778
0 GATTTGCGTCTGCATTTCGTTTTTGCGTCGCACCCGGCA

****
Consensus:

GATTTGCGTCTGCATTTCGTTTTTGCGTCGCACCCGGCA

>Bacillus_Fam_1536_40_1 Nr. of seq. 1 Alignment length(with gaps) = 40
Alignment score = 0.000000
GCF_000429705.1_ASM42970v1_genomic.fna_1:1910664-1910961 Satlength=298 Nr of Repeats=6 RepeatLength=40 seed=ATCCACTTAT Num.seqs=5
Similarity=0.883333 0
ATCCACTTATTTTAACTTTGTCCGAGTTATTGGCAGATTG

****
Consensus:

ATCCACTTATTTTAACTTTGTCCGAGTTATTGGCAGATTG

>Bacillus_Fam_1537_40_1 Nr. of seq. 1 Alignment length(with gaps) = 40
Alignment score = 0.000000
GCF_001439915.1_ASM143991v1_genomic.fna_83:95603-96081 Satlength=479 Nr of Repeats=12 RepeatLength=40 seed=ACGCATAAAT Num.seqs=8
Similarity=0.858333
0 ACGCATAAATCCTGGAACCGATGCAAATAACTTGGATCCG

****
Consensus:

ACGCATAAATCCTGGAACCGATGCAAATAACTTGGATCCG

>Bacillus_Fam_1538_40_1 Nr. of seq. 1 Alignment length(with gaps) = 40
Alignment score = 0.000000
GCF_001712755.1_ASM171275v1_genomic.fna_36:70380-70659 Satlength=280 Nr of Repeats=7 RepeatLength=40 seed=ATTTTTAATT Num.seqs=6
Similarity=0.922222
0 ATTTTTAATTGCGATTACACGAATTTATTTGCCATTTCCC

****
Consensus:

ATTTTTAATTGCGATTACACGAATTTATTTGCCATTTCCC

>Bacillus_Fam_1539_39_1 Nr. of seq. 1 Alignment length(with gaps) = 39
Alignment score = 0.000000
GCF_000008505.1_ASM850v1_genomic.fna_1:2312216-2312489 Satlength=274 Nr of Repeats=7 RepeatLength=39 seed=TTATCTTTAG Num.seqs=7
Similarity=0.960928
0 TTATCTTTAGATTTCCACCAATCGTCATCACAATTGTGC

****
Consensus:

TTATCTTTAGATTTCCACCAATCGTCATCACAATTGTGC

>Bacillus_Fam_1540_39_1 Nr. of seq. 1 Alignment length(with gaps) = 39 Alignment score = 0.000000
GCF_000025825.1_ASM2582v1_genomic.fna_1:2728911-2729106 Satlength=196 Nr of Repeats=5 RepeatLength=39 seed=TCTTCTTTTG Num.seqs=5 Similarity=0.842735
0   TCTTCTTTTGCCGCTTGCTCCTCAGCTTTTCGTTTTGCT

****
Consensus:

TCTTCTTTTGCCGCTTGCTCCTCAGCTTTTCGTTTTGCT

>Bacillus_Fam_1541_39_1 Nr. of seq. 1 Alignment length(with gaps) = 39 Alignment score = 0.000000
GCF_000025825.1_ASM2582v1_genomic.fna_8:158379-158808 Satlength=430 Nr of Repeats=11 RepeatLength=39 seed=TTCATGCCTT Num.seqs=11 Similarity=0.987568
0   TTCATGCCTTCCATATCATGAGAAGTGTGCTCGTCCATA

****
Consensus:

TTCATGCCTTCCATATCATGAGAAGTGTGCTCGTCCATA

>Bacillus_Fam_1542_39_1 Nr. of seq. 1 Alignment length(with gaps) = 39 Alignment score = 0.000000
GCF_000186145.1_Bacillus_sp_2_A_57_CT2_V1_genomic.fna_2:34085-34280 Satlength=196 Nr of Repeats=5 RepeatLength=39 seed=CGCCTGGATT Num.seqs=5 Similarity=0.931624 0
CGCCTGGATTGCCCGGATCTTCAGGATCCGGCTCCTCTC

****
Consensus:

CGCCTGGATTGCCCGGATCTTCAGGATCCGGCTCCTCTC

>Bacillus_Fam_1543_39_1 Nr. of seq. 1 Alignment length(with gaps) = 39 Alignment score = 0.000000
GCF_000242895.2_ASM24289v3_genomic.fna_1:3783533-3783728 Satlength=196 Nr of Repeats=5 RepeatLength=39 seed=TCTGGTAATT Num.seqs=5 Similarity=1.000000 0
TCTGGTAATTTGTTAGTTGCTTCCTCTTCATGTGCAAGC

****
Consensus:

TCTGGTAATTTGTTAGTTGCTTCCTCTTCATGTGCAAGC

>Bacillus_Fam_1544_39_1 Nr. of seq. 1 Alignment length(with gaps) = 39 Alignment score = 0.000000

GCF_000285535.1_ASM28553v1_genomic.fna_2:942459-942748 Satlength=290 Nr of Repeats=5 RepeatLength=39 seed=GCGCTTTTCT Num.seqs=3 Similarity=0.977208
0 GCGCTTTTCTTATGCTTGTCGCCGATAAACAGGCGCCTT

****
Consensus:

GCGCTTTTCTTATGCTTGTCGCCGATAAACAGGCGCCTT

>Bacillus_Fam_1545_39_1 Nr. of seq. 1 Alignment length(with gaps) = 39 Alignment score = 0.000000
GCF_000299035.1_ASM29903v1_genomic.fna_14:15269-15464 Satlength=196 Nr of Repeats=5 RepeatLength=39 seed=TTACCATTAT Num.seqs=5 Similarity=0.952137
0 TTACCATTATCAGAGCCACTGCCATTGTTCGTGTTGTTA

****
Consensus:

TTACCATTATCAGAGCCACTGCCATTGTTCGTGTTGTTA

>Bacillus_Fam_1546_39_1 Nr. of seq. 1 Alignment length(with gaps) = 39 Alignment score = 0.000000
GCF_000305495.1_BacPsy2.0_genomic.fna_84:16859-17291 Satlength=433 Nr of Repeats=7 RepeatLength=39 seed=CTTTTCCTGA Num.seqs=5 Similarity=0.890598
0 CTTTTCCTGAAGTCTACGGATCCTGTGTAGTATGGTGCG

****
Consensus:

CTTTTCCTGAAGTCTACGGATCCTGTGTAGTATGGTGCG

>Bacillus_Fam_1547_39_1 Nr. of seq. 1 Alignment length(with gaps) = 39 Alignment score = 0.000000
GCF_000321185.1_ASM32118v1_genomic.fna_73:12532-12802 Satlength=271 Nr of Repeats=7 RepeatLength=39 seed=GCTGGTTGCT Num.seqs=6 Similarity=0.949858
0 GCTGGTTGCTGCTGCATGCCACCCATCGGTTGTTGGTAC

****
Consensus:

GCTGGTTGCTGCTGCATGCCACCCATCGGTTGTTGGTAC

>Bacillus_Fam_1548_39_1 Nr. of seq. 1 Alignment length(with gaps) = 39 Alignment score = 0.000000
GCF_000331575.1_ASM33157v1_genomic.fna_1:892265-892616 Satlength=352 Nr of Repeats=9 RepeatLength=39 seed=CTCCGTATCC Num.seqs=9 Similarity=0.798670
0 CTCCGTATCCAGGCATTGCTCCATAGCCCGCTGGGGCTG

****
Consensus:

CTCCGTATCCAGGCATTGCTCCATAGCCCGCTGGGGCTG

>Bacillus_Fam_1549_39_1 Nr. of seq. 1 Alignment length(with gaps) = 39
Alignment score = 0.000000
GCF_000331575.1_ASM33157v1_genomic.fna_1:893092-893287 Satlength=196 Nr of Repeats=5 RepeatLength=39 seed=AAACCTGGCT Num.seqs=5 Similarity=0.911111
0   AAACCTGGCTGAGCGCCATAAGGAACTCCTGCTGCGGCG

****
Consensus:

AAACCTGGCTGAGCGCCATAAGGAACTCCTGCTGCGGCG

>Bacillus_Fam_1550_39_1 Nr. of seq. 1 Alignment length(with gaps) = 39
Alignment score = 0.000000
GCF_000331575.1_ASM33157v1_genomic.fna_1:1527350-1527623 Satlength=274 Nr of Repeats=7 RepeatLength=39 seed=CAAAGTCTCA Num.seqs=5 Similarity=0.829060 0
CAAAGTCTCATAGCATGCTGATCATTGACTACATCAATG

****
Consensus:

CAAAGTCTCATAGCATGCTGATCATTGACTACATCAATG

>Bacillus_Fam_1551_39_1 Nr. of seq. 1 Alignment length(with gaps) = 39
Alignment score = 0.000000
GCF_000430785.1_ASM43078v1_genomic.fna_125:5214-5682 Satlength=469 Nr of Repeats=4 RepeatLength=39 seed=TTCACCTACT Num.seqs=3 Similarity=0.954416
0   TTCACCTACTCTTTGCTTCATTGCAGTTAAGTCCGTCAA

****
Consensus:

TTCACCTACTCTTTGCTTCATTGCAGTTAAGTCCGTCAA

>Bacillus_Fam_1552_39_1 Nr. of seq. 1 Alignment length(with gaps) = 39
Alignment score = 0.000000
GCF_000474275.1_Bmar1.0_genomic.fna_3:53101-53257 Satlength=157 Nr of Repeats=4 RepeatLength=39 seed=CTCAAGCAAA Num.seqs=4 Similarity=0.925926
0   CTCAAGCAAATCAGTCAATGATGCCAATGAATATAGGGC

****
Consensus:

CTCAAGCAAATCAGTCAATGATGCCAATGAATATAGGGC

>Bacillus_Fam_1553_39_1 Nr. of seq. 1 Alignment length(with gaps) = 39
Alignment score = 0.000000
GCF_000504145.1_SOAPdenovo_for_version2.04_of_Bacillus_sp._FJAT-14515_genome_genomic.fna_22:379727-380150 Satlength=424 Nr of Repeats=11 RepeatLength=39 seed=AGGATATTGA Num.seqs=9 Similarity=0.882241   0
AGGATATTGACCAGGGCCTCCTCCTTGGGGCGGCAGTCC

****
Consensus:

AGGATATTGACCAGGGCCTCCTCCTTGGGGCGGCAGTCC

>Bacillus_Fam_1554_39_1 Nr. of seq. 1 Alignment length(with gaps) = 39
Alignment score = 0.000000
GCF_000526655.1_ASM52665v1_genomic.fna_57:9993-10290 Satlength=298 Nr of Repeats=7 RepeatLength=39 seed=GGAATGGGAA Num.seqs=5 Similarity=0.952137
0   GGAATGGGAACAGGAGGCTATCCCGGCGGAGGATTTCCA

****
Consensus:

GGAATGGGAACAGGAGGCTATCCCGGCGGAGGATTTCCA

>Bacillus_Fam_1555_39_1 Nr. of seq. 1 Alignment length(with gaps) = 39
Alignment score = 0.000000
GCF_000612665.1_FF4_genomic.fna_72:73187-73343 Satlength=157 Nr of Repeats=4 RepeatLength=39 seed=GGGGGCCTGT Num.seqs=4 Similarity=0.891738
0   GGGGGCCTGTCCCCCGCTGTTTTAACGCATTACTCCGCC

****
Consensus:

GGGGGCCTGTCCCCCGCTGTTTTAACGCATTACTCCGCC

>Bacillus_Fam_1556_39_1 Nr. of seq. 1 Alignment length(with gaps) = 39
Alignment score = 0.000000
GCF_000615945.1_ASM61594v1_genomic.fna_21:1342-1537 Satlength=196 Nr of Repeats=5 RepeatLength=39 seed=AGAATCAACA Num.seqs=5 Similarity=0.805128
0   AGAATCAACAGAAGAAATTCAAGCAGTAGCAGAGGAAGT

****
Consensus:

AGAATCAACAGAAGAAATTCAAGCAGTAGCAGAGGAAGT

>Bacillus_Fam_1557_39_1 Nr. of seq. 1 Alignment length(with gaps) = 39
Alignment score = 0.000000
GCF_000712595.1_ASM71259v1_genomic.fna_1:262817-263012 Satlength=196 Nr of Repeats=5 RepeatLength=39 seed=TCTTTCTTCT Num.seqs=5 Similarity=0.917949
0   TCTTTCTTCTCTTCAGAAGAACCACTTGTTGATTTCTCT

****
Consensus:

TCTTTCTTCTCTTCAGAAGAACCACTTGTTGATTTCTCT

>Bacillus_Fam_1558_39_1 Nr. of seq. 1 Alignment length(with gaps) = 39
Alignment score = 0.000000

GCF_000712595.1_ASM71259v1_genomic.fna_4:298794-298989 Satlength=196 Nr of Repeats=5 RepeatLength=39 seed=TTTTCTTCAG Num.seqs=5 Similarity=0.840650
0 TTTTCTTCAGGAGCTACAGTTTCGTTACCTGTTTCAGGT

****
Consensus:

TTTTCTTCAGGAGCTACAGTTTCGTTACCTGTTTCAGGT

>Bacillus_Fam_1559_39_1 Nr. of seq. 1 Alignment length(with gaps) = 39 Alignment score = 0.000000
GCF_000712595.1_ASM71259v1_genomic.fna_8:89072-89945 Satlength=874 Nr of Repeats=24 RepeatLength=39 seed=GCAGGTAAAG Num.seqs=16 Similarity=0.991453
0 GCAGGTAAAGGTGGCGCAGGAGCAGGCGAAGGTCCAGGA

****
Consensus:

GCAGGTAAAGGTGGCGCAGGAGCAGGCGAAGGTCCAGGA

>Bacillus_Fam_1560_39_1 Nr. of seq. 1 Alignment length(with gaps) = 39 Alignment score = 0.000000
GCF_000712615.1_ASM71261v1_genomic.fna_52:17519-17675 Satlength=157 Nr of Repeats=4 RepeatLength=39 seed=CAAAACCACC Num.seqs=4 Similarity=0.811966
0 CAAAACCACCAGTAGACCCACCAACAGATCCAGGAGATA

****
Consensus:

CAAAACCACCAGTAGACCCACCAACAGATCCAGGAGATA

>Bacillus_Fam_1561_39_1 Nr. of seq. 1 Alignment length(with gaps) = 39 Alignment score = 0.000000
GCF_000769555.1_ASM76955v1_genomic.fna_1:2858012-2858741 Satlength=730 Nr of Repeats=19 RepeatLength=39 seed=TCTGCTGCGG Num.seqs=18 Similarity=0.988604 0
TCTGCTGCGGGAACGGTGTGTCCGGTGATGCGTCCGTCT

****
Consensus:

TCTGCTGCGGGAACGGTGTGTCCGGTGATGCGTCCGTCT

>Bacillus_Fam_1562_39_1 Nr. of seq. 1 Alignment length(with gaps) = 39 Alignment score = 0.000000
GCF_000829555.1_ASM82955v1_genomic.fna_11:1052056-1052221 Satlength=166 Nr of Repeats=4 RepeatLength=39 seed=GGCATATTTG Num.seqs=3 Similarity=0.954416 0
GGCATATTTGGCATCGGTTCATTCGGCATTGGCATGTTC

****
Consensus:

GGCATATTTGGCATCGGTTCATTCGGCATTGGCATGTTC

>Bacillus_Fam_1563_39_1 Nr. of seq. 1 Alignment length(with gaps) = 39
Alignment score = 0.000000
GCF_000831065.1_ASM83106v1_genomic.fna_1:809013-809169 Satlength=157 Nr of Repeats=4 RepeatLength=39 seed=CAAAGATAGA Num.seqs=4
Similarity=0.931624
0 CAAAGATAGAAGAGAAGAAAACTGAAGATAAAGAAACAG

****
Consensus:

CAAAGATAGAAGAGAAGAAAACTGAAGATAAAGAAACAG

>Bacillus_Fam_1564_39_1 Nr. of seq. 1 Alignment length(with gaps) = 39
Alignment score = 0.000000
GCF_001273755.1_ASM127375v1_genomic.fna_17:4517711-4518605 Satlength=895 Nr of Repeats=23 RepeatLength=39 seed=CCTTGACCTC Num.seqs=22
Similarity=0.957968 0
CCTTGACCTCCCGGCATCATCGGAAATCCTCCAGGCTGA

****
Consensus:

CCTTGACCTCCCGGCATCATCGGAAATCCTCCAGGCTGA

>Bacillus_Fam_1565_39_1 Nr. of seq. 1 Alignment length(with gaps) = 39
Alignment score = 0.000000
GCF_001274935.1_ASM127493v1_genomic.fna_27:120842-121037 Satlength=196 Nr of Repeats=4 RepeatLength=39 seed=AGATCTGGTC Num.seqs=3
Similarity=0.863248 0
AGATCTGGTCGTTCTCGTAGATCCCGCCGTTCTGGAGGC

****
Consensus:

AGATCTGGTCGTTCTCGTAGATCCCGCCGTTCTGGAGGC

>Bacillus_Fam_1566_39_1 Nr. of seq. 1 Alignment length(with gaps) = 39
Alignment score = 0.000000
GCF_001274935.1_ASM127493v1_genomic.fna_27:368943-369780 Satlength=838 Nr of Repeats=20 RepeatLength=39 seed=CCTGGCATCA Num.seqs=13
Similarity=0.903134 0
CCTGGCATCATTGGCTGGCCGCTCATTCCTTGACCCATT

****
Consensus:

CCTGGCATCATTGGCTGGCCGCTCATTCCTTGACCCATT

>Bacillus_Fam_1567_39_1 Nr. of seq. 1 Alignment length(with gaps) = 39
Alignment score = 0.000000

GCF_001274935.1_ASM127493v1_genomic.fna_36:331062-331251 Satlength=190 Nr of Repeats=5 RepeatLength=39 seed=TGATCATGAC Num.seqs=4 Similarity=0.931624 0
TGATCATGACTATGACTGTGACCTTCGTGACTGTGTTCG

****
Consensus:

TGATCATGACTATGACTGTGACCTTCGTGACTGTGTTCG

>Bacillus_Fam_1568_39_1 Nr. of seq. 1 Alignment length(with gaps) = 39 Alignment score = 0.000000
GCF_001315085.1_ASM131508v1_genomic.fna_47:6-162 Satlength=157 Nr of Repeats=4 RepeatLength=39 seed=GGATGGGGAA Num.seqs=4 Similarity=0.863248
0 GGATGGGGAAGCGGATTCCCACAAACACCAGGCCAAAGC

****
Consensus:

GGATGGGGAAGCGGATTCCCACAAACACCAGGCCAAAGC

>Bacillus_Fam_1569_39_1 Nr. of seq. 1 Alignment length(with gaps) = 39 Alignment score = 0.000000
GCF_001420595.1_ASM142059v1_genomic.fna_11:2191867-2192027 Satlength=161 Nr of Repeats=4 RepeatLength=40 seed=CAAATAAAAT Num.seqs=4 Similarity=0.566667 0
CAAATAAAATGAGAGTTTCGCAAAAATAAAGCGTTCTTG

****
Consensus:

CAAATAAAATGAGAGTTTCGCAAAAATAAAGCGTTCTTG

>Bacillus_Fam_1570_39_1 Nr. of seq. 1 Alignment length(with gaps) = 39 Alignment score = 0.000000
GCF_001578205.1_ASM157820v1_genomic.fna_2:10302-10458 Satlength=157 Nr of Repeats=4 RepeatLength=39 seed=AAGCTGTAGA Num.seqs=4 Similarity=0.931624
0 AAGCTGTAGAGAAGCAGGAACCGGCCAAGGAAGCAGCAC

****
Consensus:

AAGCTGTAGAGAAGCAGGAACCGGCCAAGGAAGCAGCAC

>Bacillus_Fam_1571_39_1 Nr. of seq. 1 Alignment length(with gaps) = 39 Alignment score = 0.000000
GCF_001591945.1_ASM159194v1_genomic.fna_7:147123-147357 Satlength=235 Nr of Repeats=6 RepeatLength=39 seed=TGCATTGGCA Num.seqs=6 Similarity=0.938462
0 TGCATTGGCATTTGACCTTGATCTGGCATATATGGTTGC

****
Consensus:

TGCATTGGCATTTGACCTTGATCTGGCATATATGGTTGC

>Bacillus_Fam_1572_39_1 Nr. of seq. 1 Alignment length(with gaps) = 39 Alignment score = 0.000000 GCF_001636325.1_ASM163632v1_genomic.fna_1:545978-546593 Satlength=616 Nr of Repeats=16 RepeatLength=39 seed=TTCTGCGAGA Num.seqs=13 Similarity=0.914092 0 TTCTGCGAGAACGGCATGATCTGCGCGTGCCTCTCGTGC

****
Consensus:

TTCTGCGAGAACGGCATGATCTGCGCGTGCCTCTCGTGC

>Bacillus_Fam_1573_39_1 Nr. of seq. 1 Alignment length(with gaps) = 39 Alignment score = 0.000000 GCF_001636335.1_ASM163633v1_genomic.fna_1:1653655-1653811 Satlength=157 Nr of Repeats=4 RepeatLength=39 seed=TTTGTGTTTG Num.seqs=4 Similarity=0.811966 0 TTTGTGTTTGCGTTTGAGCTGGTTGCTTTTCTACCGGCT

****
Consensus:

TTTGTGTTTGCGTTTGAGCTGGTTGCTTTTCTACCGGCT

>Bacillus_Fam_1574_39_1 Nr. of seq. 1 Alignment length(with gaps) = 39 Alignment score = 0.000000 GCF_001712755.1_ASM171275v1_genomic.fna_7:93822-93969 Satlength=148 Nr of Repeats=4 RepeatLength=39 seed=GCTTTAGAGT Num.seqs=3 Similarity=0.886040 0 GCTTTAGAGTTGAATCTGTATCAGAATTATTTGAATCGT

****
Consensus:

GCTTTAGAGTTGAATCTGTATCAGAATTATTTGAATCGT

>Bacillus_Fam_1575_39_1 Nr. of seq. 1 Alignment length(with gaps) = 39 Alignment score = 0.000000 GCF_001712755.1_ASM171275v1_genomic.fna_13:255755-256136 Satlength=382 Nr of Repeats=11 RepeatLength=39 seed=CAACAACTGG Num.seqs=7 Similarity=0.882784 0 CAACAACTGGTGAGACAACTGGAGGAACAACCGGCAGCA

****
Consensus:

CAACAACTGGTGAGACAACTGGAGGAACAACCGGCAGCA

>Bacillus_Fam_1576_39_1 Nr. of seq. 1 Alignment length(with gaps) = 39 Alignment score = 0.000000 GCF_002019605.1_ASM201960v1_genomic.fna_1:2847050-2847845 Satlength=796 Nr of Repeats=9 RepeatLength=39 seed=TGTGGTGCCA Num.seqs=6

Similarity=0.977208 0
TGTGGTGCCATTTGATGCGGTTGATACGGCATCGCCTGC

****
Consensus:

TGTGGTGCCATTTGATGCGGTTGATACGGCATCGCCTGC

>Bacillus_Fam_1577_39_1 Nr. of seq. 1 Alignment length(with gaps) = 39
Alignment score = 0.000000
GCF_002019635.1_ASM201963v1_genomic.fna_1:1500315-1500474 Satlength=160
Nr of Repeats=4 RepeatLength=39 seed=CATGTTCTTC Num.seqs=3
Similarity=0.954416 0
CATGTTCTTCTTCATGAGCAGCCTCAGTTTCATCATGTG

****
Consensus:

CATGTTCTTCTTCATGAGCAGCCTCAGTTTCATCATGTG

>Bacillus_Fam_1578_39_1 Nr. of seq. 1 Alignment length(with gaps) = 39
Alignment score = 0.000000
GCF_002019635.1_ASM201963v1_genomic.fna_1:2134773-2135010 Satlength=238
Nr of Repeats=6 RepeatLength=39 seed=AAAACAACAA Num.seqs=5
Similarity=0.833333 0
AAAACAACAACAAGAGCAGCAACAGCAGCAGGCCCAACA

****
Consensus:

AAAACAACAACAAGAGCAGCAACAGCAGCAGGCCCAACA

>Bacillus_Fam_1579_39_1 Nr. of seq. 1 Alignment length(with gaps) = 39
Alignment score = 0.000000
GCF_002019645.1_ASM201964v1_genomic.fna_1:1404723-1404996 Satlength=274
Nr of Repeats=7 RepeatLength=39 seed=TCTTCTTCCT Num.seqs=7
Similarity=0.916972 0
TCTTCTTCCTCATGGGCATGTTCATCGTCAGCGTGNTGC

****
Consensus:

TCTTCTTCCTCATGGGCATGTTCATCGTCAGCGTGNTGC

>Bacillus_Fam_1580_39_1 Nr. of seq. 1 Alignment length(with gaps) = 39
Alignment score = 0.000000
GCF_900093775.1_EVONIK_BGLY_genomic.fna_1:3164589-3164784 Satlength=196
Nr of Repeats=5 RepeatLength=39 seed=GCTTTCTTCC Num.seqs=5
Similarity=0.883761 0
GCTTTCTTCCATTCTCGGTTCCGCTTTTGCGTCCCTGCT

****
Consensus:

GCTTTCTTCCATTCTCGGTTCCGCTTTTGCGTCCCTGCT

>Bacillus_Fam_1581_39_1 Nr. of seq. 1 Alignment length(with gaps) = 39
Alignment score = 0.000000
GCF_900098925.1_PRJEB15625_genomic.fna_15:229366-229608 Satlength=243 Nr of Repeats=5 RepeatLength=39 seed=CACGGCTGGC Num.seqs=3
Similarity=0.886040
0   CACGGCTGGCCGGGTCAAGGCGGGTCTGGCTCCGGCGGG

****
Consensus:

CACGGCTGGCCGGGTCAAGGCGGGTCTGGCTCCGGCGGG

>Bacillus_Fam_1582_39_1 Nr. of seq. 1 Alignment length(with gaps) = 39
Alignment score = 0.000000
GCF_900098925.1_PRJEB15625_genomic.fna_16:204771-204963 Satlength=193 Nr of Repeats=5 RepeatLength=39 seed=CCCTGCATAT Num.seqs=4
Similarity=0.943020
0   CCCTGCATATGATGTCCGTGTCCCATATCCGGCATGTTT

****
Consensus:

CCCTGCATATGATGTCCGTGTCCCATATCCGGCATGTTT

>Bacillus_Fam_1583_39_1 Nr. of seq. 1 Alignment length(with gaps) = 39
Alignment score = 0.000000
GCF_900103955.1_IMG-taxon_2617270767_annotated_assembly_genomic.fna_20:6914-7262
Satlength=349 Nr of Repeats=8 RepeatLength=39 seed=CGTTCGGCTT Num.seqs=6
Similarity=0.835897   0
CGTTCGGCTTCTTCCGCAGCAATGCGCTCTTCCTCCTCA

****
Consensus:

CGTTCGGCTTCTTCCGCAGCAATGCGCTCTTCCTCCTCA

>Bacillus_Fam_1584_39_1 Nr. of seq. 1 Alignment length(with gaps) = 39
Alignment score = 0.000000
GCF_900104555.1_IMG-taxon_2651870172_annotated_assembly_genomic.fna_13:10452-10608
Satlength=157 Nr of Repeats=4 RepeatLength=39 seed=TATCAACCAG Num.seqs=4
Similarity=0.960114   0
TATCAACCAGCACCGTTTCCACAAGGTGGTCAAATGGGA

****
Consensus:

TATCAACCAGCACCGTTTCCACAAGGTGGTCAAATGGGA

>Bacillus_Fam_1585_38_1 Nr. of seq. 1 Alignment length(with gaps) = 38
Alignment score = 0.000000
GCF_900107275.1_IMG-taxon_2675903024_annotated_assembly_genomic.fna_37:151112-151470
Satlength=359 Nr of Repeats=5 RepeatLength=38 seed=TTTGCTTCGT Num.seqs=3
Similarity=1.000000 0
TTTGCTTCGTAGTCTTCCCTGTTTCTTCCTCTGCCAGC

****
Consensus:

TTTGCTTCGTAGTCTTCCCTGTTTCTTCCTCTGCCAGC

>Bacillus_Fam_1586_37_1 Nr. of seq. 1 Alignment length(with gaps) = 37
Alignment score = 0.000000
GCF_000331575.1_ASM33157v1_genomic.fna_4:174812-175053 Satlength=242 Nr of Repeats=6 RepeatLength=37 seed=GAAATCGGCC Num.seqs=4
Similarity=0.861862
0 GAAATCGGCCTTATTGCACGTTATCGGGCTTGTTGCA

****
Consensus:

GAAATCGGCCTTATTGCACGTTATCGGGCTTGTTGCA

>Bacillus_Fam_1587_36_1 Nr. of seq. 1 Alignment length(with gaps) = 36
Alignment score = 0.000000
GCF_000007825.1_ASM782v1_genomic.fna_1:2019353-2019731 Satlength=379 Nr of Repeats=13 RepeatLength=36 seed=AAAACCTGAT Num.seqs=8
Similarity=0.898148
0 AAAACCTGATACGGGAGGAAATCCAGACACAGGAGG

****
Consensus:

AAAACCTGATACGGGAGGAAATCCAGACACAGGAGG

>Bacillus_Fam_1588_36_1 Nr. of seq. 1 Alignment length(with gaps) = 36
Alignment score = 0.000000
GCF_000011145.1_ASM1114v1_genomic.fna_1:1314924-1315176 Satlength=253 Nr of Repeats=7 RepeatLength=36 seed=ATGGATCAAA Num.seqs=7
Similarity=0.961199
0 ATGGATCAAAACGTGCATCCGCAGCAATGGCCGCAA

****
Consensus:

ATGGATCAAAACGTGCATCCGCAGCAATGGCCGCAA

>Bacillus_Fam_1589_36_1 Nr. of seq. 1 Alignment length(with gaps) = 36
Alignment score = 0.000000
GCF_000262045.1_KCTC_13613_01_genomic.fna_39:441056-441350 Satlength=295 Nr of Repeats=8 RepeatLength=36 seed=TTTTGTGAGA Num.seqs=6

Similarity=0.886420 0
TTTTGTGAGAACGTGATTTTTTATGGCTGCGGGATT

****
Consensus:

TTTTGTGAGAACGTGATTTTTTATGGCTGCGGGATT

>Bacillus_Fam_1590_36_1 Nr. of seq. 1 Alignment length(with gaps) = 36
Alignment score = 0.000000
GCF_000285535.1_ASM28553v1_genomic.fna_4:98805-98913 Satlength=109 Nr of Repeats=5 RepeatLength=36 seed=ATGCAGGGTA Num.seqs=3 Similarity=0.925926
0 ATGCAGGGTATGCAGGGTAGAGCTGGCCTGGGGGGT

****
Consensus:

ATGCAGGGTATGCAGGGTAGAGCTGGCCTGGGGGGT

>Bacillus_Fam_1591_36_1 Nr. of seq. 1 Alignment length(with gaps) = 36
Alignment score = 0.000000
GCF_000305495.1_BacPsy2.0_genomic.fna_193:33-213 Satlength=181 Nr of Repeats=5 RepeatLength=36 seed=ACAGGTGATA Num.seqs=5 Similarity=0.796296
0 ACAGGTGATACCGGGGCNACCGGAGTAACNGGAGCA

****
Consensus:

ACAGGTGATACCGGGGCNACCGGAGTAACNGGAGCA

>Bacillus_Fam_1592_36_1 Nr. of seq. 1 Alignment length(with gaps) = 36
Alignment score = 0.000000
GCF_000307875.1_BABA1.0_genomic.fna_146:993-1137 Satlength=145 Nr of Repeats=4 RepeatLength=36 seed=AAAAGGGAAT Num.seqs=4 Similarity=0.919753
0 AAAAGGGAATTGGAACTGCAGTCAGAACAACAGAGA

****
Consensus:

AAAAGGGAATTGGAACTGCAGTCAGAACAACAGAGA

>Bacillus_Fam_1593_36_1 Nr. of seq. 1 Alignment length(with gaps) = 36
Alignment score = 0.000000
GCF_000321185.1_ASM32118v1_genomic.fna_87:51026-51170 Satlength=145 Nr of Repeats=4 RepeatLength=36 seed=CTTCTGGTTT Num.seqs=4 Similarity=0.944444
0 CTTCTGGTTTATTGTTATCAACAACTGGTTGATCTG

****
Consensus:

CTTCTGGTTTATTGTTATCAACAACTGGTTGATCTG

>Bacillus_Fam_1594_36_1 Nr. of seq. 1 Alignment length(with gaps) = 36
Alignment score = 0.000000

GCF_000331575.1_ASM33157v1_genomic.fna_1:1603161-1603305 Satlength=145 Nr of Repeats=4 RepeatLength=36 seed=TCGCTTTGAT Num.seqs=4 Similarity=0.827160 0
TCGCTTTGATTTTGGTTTTGGGACAAGCCCTCGTCC

****
Consensus:

TCGCTTTGATTTTGGTTTTGGGACAAGCCCTCGTCC

>Bacillus_Fam_1595_36_1 Nr. of seq. 1 Alignment length(with gaps) = 36 Alignment score = 0.000000
GCF_000374565.1_ASM37456v1_genomic.fna_18:1732-1914 Satlength=183 Nr of Repeats=5 RepeatLength=36 seed=TTTTCATCCA Num.seqs=3 Similarity=0.950617
0 TTTTCATCCAAAGGGCAGCCGGAAAAGCAAACCAGG

****
Consensus:

TTTTCATCCAAAGGGCAGCCGGAAAAGCAAACCAGG

>Bacillus_Fam_1596_36_1 Nr. of seq. 1 Alignment length(with gaps) = 36 Alignment score = 0.000000
GCF_000380245.2_ASM38024v2_genomic.fna_12:377405-377621 Satlength=217 Nr of Repeats=6 RepeatLength=36 seed=CAGACATATC Num.seqs=6 Similarity=0.967901
0 CAGACATATCAACCAAATCAAATGACGATGCCACAA

****
Consensus:

CAGACATATCAACCAAATCAAATGACGATGCCACAA

>Bacillus_Fam_1597_36_1 Nr. of seq. 1 Alignment length(with gaps) = 36 Alignment score = 0.000000
GCF_000401235.1_BacNeaAAU1_genomic.fna_112:56318-56642 Satlength=325 Nr of Repeats=9 RepeatLength=36 seed=CTATCTCTTC Num.seqs=9 Similarity=0.787287
0 CTATCTCTTCAGTTACTTCGACTTCTGGAGTTACAG

****
Consensus:

CTATCTCTTCAGTTACTTCGACTTCTGGAGTTACAG

>Bacillus_Fam_1598_36_1 Nr. of seq. 1 Alignment length(with gaps) = 36 Alignment score = 0.000000
GCF_000430785.1_ASM43078v1_genomic.fna_4:125493-125637 Satlength=145 Nr of Repeats=4 RepeatLength=36 seed=CCGCTTCCTG Num.seqs=4 Similarity=0.981481
0 CCGCTTCCTGGAGCATCTGGTTGATTTCCTTCTTGG

****
Consensus:

CCGCTTCCTGGAGCATCTGGTTGATTTCCTTCTTGG

>Bacillus_Fam_1599_36_1 Nr. of seq. 1 Alignment length(with gaps) = 36 Alignment score = 0.000000
GCF_000496285.1_ASM49628v1_genomic.fna_1:1885990-1886788 Satlength=799 Nr of Repeats=13 RepeatLength=36 seed=CTTCTGCAAC Num.seqs=11 Similarity=0.955556 0
CTTCTGCAACTGGTTGTTCTTCAGCGACTGGCCCTT

****
Consensus:

CTTCTGCAACTGGTTGTTCTTCAGCGACTGGCCCTT

>Bacillus_Fam_1600_36_1 Nr. of seq. 1 Alignment length(with gaps) = 36 Alignment score = 0.000000
GCF_000612625.1_JCE_genomic.fna_1:141216-141576 Satlength=361 Nr of Repeats=10 RepeatLength=36 seed=GGGCGGATTC Num.seqs=10 Similarity=0.758674
0 GGGCGGATTCCCTGGTCANGGTGGNCATGGNGGAAT

****
Consensus:

GGGCGGATTCCCTGGTCANGGTGGNCATGGNGGAAT

>Bacillus_Fam_1601_36_1 Nr. of seq. 1 Alignment length(with gaps) = 36 Alignment score = 0.000000
GCF_000612625.1_JCE_genomic.fna_1:153492-153708 Satlength=217 Nr of Repeats=6 RepeatLength=36 seed=TCCAAAGGCA Num.seqs=6 Similarity=0.906173
0 TCCAAAGGCAGTGGTGGATCGAAAGGAAGTCATGGC

****
Consensus:

TCCAAAGGCAGTGGTGGATCGAAAGGAAGTCATGGC

>Bacillus_Fam_1602_36_1 Nr. of seq. 1 Alignment length(with gaps) = 36 Alignment score = 0.000000
GCF_000612625.1_JCE_genomic.fna_6:65616-65796 Satlength=181 Nr of Repeats=5 RepeatLength=36 seed=CTGTTATTTC Num.seqs=5 Similarity=0.837037
0 CTGTTATTTCCGCCACTTCGACTACGTCATCTACAC

****
Consensus:

CTGTTATTTCCGCCACTTCGACTACGTCATCTACAC

>Bacillus_Fam_1603_36_1 Nr. of seq. 1 Alignment length(with gaps) = 36 Alignment score = 0.000000
GCF_000621445.1_ASM62144v1_genomic.fna_3:309868-310012 Satlength=145 Nr of Repeats=4 RepeatLength=36 seed=GTGTCATCCC Num.seqs=4

Similarity=0.888889
0   GTGTCATCCCCAGTACCTGGACTTCCTCCATTATCC

****
Consensus:

GTGTCATCCCCAGTACCTGGACTTCCTCCATTATCC

>Bacillus_Fam_1604_36_1 Nr. of seq. 1 Alignment length(with gaps) = 36
Alignment score = 0.000000
GCF_000706725.1_ASM70672v1_genomic.fna_1:3481500-3481644 Satlength=145 Nr of Repeats=4 RepeatLength=36 seed=GTTTCTTCCT Num.seqs=4
Similarity=0.932099 0
GTTTCTTCCTCTACTGCTTCTTCCGGCGCTTCTGAA

****
Consensus:

GTTTCTTCCTCTACTGCTTCTTCCGGCGCTTCTGAA

>Bacillus_Fam_1605_36_1 Nr. of seq. 1 Alignment length(with gaps) = 36
Alignment score = 0.000000
GCF_000712615.1_ASM71261v1_genomic.fna_6:44543-44699 Satlength=157 Nr of Repeats=4 RepeatLength=36 seed=ACTAGCTCTT Num.seqs=3 Similarity=0.950617
0   ACTAGCTCTTCTTCAAATGCTTCCTCTGTTTCCGCA

****
Consensus:

ACTAGCTCTTCTTCAAATGCTTCCTCTGTTTCCGCA

>Bacillus_Fam_1606_36_1 Nr. of seq. 1 Alignment length(with gaps) = 36
Alignment score = 0.000000
GCF_000724485.1_ASM72448v1_genomic.fna_1:1629403-1629607 Satlength=205 Nr of Repeats=4 RepeatLength=36 seed=GCTTCAAGAA Num.seqs=3
Similarity=0.827160 0
GCTTCAAGAAACACCATTGTGACTGCTGTGACAAGT

****
Consensus:

GCTTCAAGAAACACCATTGTGACTGCTGTGACAAGT

>Bacillus_Fam_1607_36_1 Nr. of seq. 1 Alignment length(with gaps) = 36
Alignment score = 0.000000
GCF_000742675.1_ASM74267v1_genomic.fna_1:639619-639763 Satlength=145 Nr of Repeats=4 RepeatLength=36 seed=TGCAGCAGCA Num.seqs=4
Similarity=0.888889
0   TGCAGCAGCAGGAAGCACGGAAGCATGAGGAGACGT

****
Consensus:

TGCAGCAGCAGGAAGCACGGAAGCATGAGGAGACGT

>Bacillus_Fam_1608_36_1 Nr. of seq. 1 Alignment length(with gaps) = 36
Alignment score = 0.000000
GCF_000759675.1_ASM75967v1_genomic.fna_79:5447-5843 Satlength=397 Nr of Repeats=11 RepeatLength=36 seed=ACAAGCATGA Num.seqs=7 Similarity=0.715507
0   ACAAGCATGATGACTGTAATAAACGAGACAGAAACG

****
Consensus:

ACAAGCATGATGACTGTAATAAACGAGACAGAAACG

>Bacillus_Fam_1609_36_1 Nr. of seq. 1 Alignment length(with gaps) = 36
Alignment score = 0.000000
GCF_000812025.2_ASM81202v2_genomic.fna_59:10582-10798 Satlength=217 Nr of Repeats=6 RepeatLength=36 seed=CGATGCATGC Num.seqs=6 Similarity=0.846914
0   CGATGCATGCTCAGCCAATGATGTACCCAGACAATG

****
Consensus:

CGATGCATGCTCAGCCAATGATGTACCCAGACAATG

>Bacillus_Fam_1610_36_1 Nr. of seq. 1 Alignment length(with gaps) = 36
Alignment score = 0.000000
GCF_000812025.2_ASM81202v2_genomic.fna_69:27049-27229 Satlength=181 Nr of Repeats=4 RepeatLength=36 seed=CGAAGAAACC Num.seqs=3 Similarity=0.827160
0   CGAAGAAACCAGACGGATACAAAAAGGATGACGATT

****
Consensus:

CGAAGAAACCAGACGGATACAAAAAGGATGACGATT

>Bacillus_Fam_1611_36_1 Nr. of seq. 1 Alignment length(with gaps) = 36
Alignment score = 0.000000
GCF_000813125.1_ASM81312v1_genomic.fna_55:48259-48403 Satlength=145 Nr of Repeats=4 RepeatLength=36 seed=TCTTCTTCGG Num.seqs=4 Similarity=0.796296
0   TCTTCTTCGGAGTGAGCGGCTTCTTCGTCATGGTGC

****
Consensus:

TCTTCTTCGGAGTGAGCGGCTTCTTCGTCATGGTGC

>Bacillus_Fam_1612_36_1 Nr. of seq. 1 Alignment length(with gaps) = 36
Alignment score = 0.000000
GCF_000832605.1_ASM83260v1_genomic.fna_1:2585613-2585757 Satlength=145 Nr of Repeats=4 RepeatLength=36 seed=GAACCGAAAA Num.seqs=4 Similarity=0.956790 0
GAACCGAAAACAGAAAAGCCAGATGGTAAACCAGAA

****

Consensus:

GAACCGAAAACAGAAAAGCCAGATGGTAAACCAGAA

>Bacillus_Fam_1613_36_1 Nr. of seq. 1 Alignment length(with gaps) = 36
Alignment score = 0.000000
GCF_000972245.2_ASM97224v2_genomic.fna_1:2081436-2082147 Satlength=712 Nr of Repeats=20 RepeatLength=36 seed=GTAGGTCCAG Num.seqs=19
Similarity=0.891705 0
GTAGGTCCAGTCGGTCCAAGGTCGCCGGTAGCACCT

****
Consensus:

GTAGGTCCAGTCGGTCCAAGGTCGCCGGTAGCACCT

>Bacillus_Fam_1614_36_1 Nr. of seq. 1 Alignment length(with gaps) = 36
Alignment score = 0.000000
GCF_000986785.1_ASM98678v1_genomic.fna_8:166666-166882 Satlength=217 Nr of Repeats=6 RepeatLength=36 seed=TTTTCCTCCG Num.seqs=6
Similarity=0.930864
0 TTTTCCTCCGGCTCCTGTTCCAGGACGAAATCCGGC

****
Consensus:

TTTTCCTCCGGCTCCTGTTCCAGGACGAAATCCGGC

>Bacillus_Fam_1615_36_1 Nr. of seq. 1 Alignment length(with gaps) = 36
Alignment score = 0.000000
GCF_000986785.1_ASM98678v1_genomic.fna_101:89652-90012 Satlength=361 Nr of Repeats=10 RepeatLength=36 seed=CCGCAAATGG Num.seqs=10
Similarity=0.847737 0
CCGCAAATGGGCTATGGCCCGCAAGCTGGATTCGCC

****
Consensus:

CCGCAAATGGGCTATGGCCCGCAAGCTGGATTCGCC

>Bacillus_Fam_1616_36_1 Nr. of seq. 1 Alignment length(with gaps) = 36
Alignment score = 0.000000
GCF_001273755.1_ASM127375v1_genomic.fna_17:1088058-1088238 Satlength=181 Nr of Repeats=5 RepeatLength=36 seed=GGAGGAGGTC Num.seqs=5
Similarity=0.933333 0
GGAGGAGGTCAACCGCCGCCGCGACCACCACAAGGG

****
Consensus:

GGAGGAGGTCAACCGCCGCCGCGACCACCACAAGGG

>Bacillus_Fam_1617_36_1 Nr. of seq. 1 Alignment length(with gaps) = 36
Alignment score = 0.000000

GCF_001439915.1_ASM143991v1_genomic.fna_105:131730-131874 Satlength=145 Nr of Repeats=4 RepeatLength=36 seed=GAAGATGATC Num.seqs=4 Similarity=0.808642 0
GAAGATGATCAATCGGACACCGATGACACAGAAGGG

****
Consensus:

GAAGATGATCAATCGGACACCGATGACACAGAAGGG

>Bacillus_Fam_1618_36_1 Nr. of seq. 1 Alignment length(with gaps) = 36 Alignment score = 0.000000
GCF_001578185.1_ASM157818v1_genomic.fna_1:1776217-1776385 Satlength=169 Nr of Repeats=5 RepeatLength=36 seed=CAATGGGACG Num.seqs=4 Similarity=0.944444 0
CAATGGGACGGACATGGCGGAGGTCATGGTGGCGGT

****
Consensus:

CAATGGGACGGACATGGCGGAGGTCATGGTGGCGGT

>Bacillus_Fam_1619_36_1 Nr. of seq. 1 Alignment length(with gaps) = 36 Alignment score = 0.000000
GCF_001590785.1_ASM159078v1_genomic.fna_23:164317-164461 Satlength=145 Nr of Repeats=4 RepeatLength=36 seed=TTTCCTCGCC Num.seqs=4 Similarity=0.870370 0
TTTCCTCGCCTCCGTCATTTTCTTCCGGAGACTCTG

****
Consensus:

TTTCCTCGCCTCCGTCATTTTCTTCCGGAGACTCTG

>Bacillus_Fam_1620_36_1 Nr. of seq. 1 Alignment length(with gaps) = 36 Alignment score = 0.000000
GCF_001590785.1_ASM159078v1_genomic.fna_23:392091-392217 Satlength=127 Nr of Repeats=4 RepeatLength=36 seed=TCTTCGCCTT Num.seqs=3 Similarity=0.925926 0
TCTTCGCCTTCGACTGGTTCTTCTCCCTCAACTGGC

****
Consensus:

TCTTCGCCTTCGACTGGTTCTTCTCCCTCAACTGGC

>Bacillus_Fam_1621_36_1 Nr. of seq. 1 Alignment length(with gaps) = 36 Alignment score = 0.000000
GCF_001591445.1_ASM159144v1_genomic.fna_59:421-646 Satlength=226 Nr of Repeats=4 RepeatLength=36 seed=TGTTGGTGCT Num.seqs=3 Similarity=0.864198 0 TGTTGGTGCTTTTGNGCTTGTCCCAGGTTTCGATAC

****
Consensus:

TGTTGGTGCTTTTGNGCTTGTCCCAGGTTTCGATAC

>Bacillus_Fam_1622_36_1 Nr. of seq. 1 Alignment length(with gaps) = 36
Alignment score = 0.000000
GCF_001591625.1_ASM159162v1_genomic.fna_26:7794-8010 Satlength=217 Nr of Repeats=6 RepeatLength=36 seed=AGAGAAAATA Num.seqs=6 Similarity=0.871605
0 AGAGAAAATAAACAAGAACAAGCAAACCAACAGGCA

****
Consensus:

AGAGAAAATAAACAAGAACAAGCAAACCAACAGGCA

>Bacillus_Fam_1623_36_1 Nr. of seq. 1 Alignment length(with gaps) = 36
Alignment score = 0.000000
GCF_001591665.1_ASM159166v1_genomic.fna_83:5099-5375 Satlength=277 Nr of Repeats=8 RepeatLength=36 seed=TTCTTCTGTT Num.seqs=7 Similarity=0.846561
0 TTCTTCTGTTACAACTTCAGTAGNTTCGTTAGTTGC

****
Consensus:

TTCTTCTGTTACAACTTCAGTAGNTTCGTTAGTTGC

>Bacillus_Fam_1624_36_1 Nr. of seq. 1 Alignment length(with gaps) = 36
Alignment score = 0.000000
GCF_001636325.1_ASM163632v1_genomic.fna_2:690522-690702 Satlength=181 Nr of Repeats=5 RepeatLength=36 seed=GGTTCTTTAA Num.seqs=5 Similarity=0.948148
0 GGTTCTTTAATTGGTTCTTCACTAGGTTGTTTAGCT

****
Consensus:

GGTTCTTTAATTGGTTCTTCACTAGGTTGTTTAGCT

>Bacillus_Fam_1625_36_1 Nr. of seq. 1 Alignment length(with gaps) = 36
Alignment score = 0.000000
GCF_001636345.1_ASM163634v1_genomic.fna_1:4287145-4287451 Satlength=307 Nr of Repeats=8 RepeatLength=36 seed=TTAGCGGCAT Num.seqs=6 Similarity=0.856790 0
TTAGCGGCATTTCCGCCATTGGCTGCGTCGCCTCCG

****
Consensus:

TTAGCGGCATTTCCGCCATTGGCTGCGTCGCCTCCG

>Bacillus_Fam_1626_36_1 Nr. of seq. 1 Alignment length(with gaps) = 36
Alignment score = 0.000000
GCF_001636425.1_ASM163642v1_genomic.fna_1:4311548-4311809 Satlength=262 Nr of Repeats=6 RepeatLength=36 seed=ATCCGGGACA Num.seqs=4

Similarity=0.938272 0
ATCCGGGACATCCAGGGTATGGCGGCATGACTGGTT

****
Consensus:

ATCCGGGACATCCAGGGTATGGCGGCATGACTGGTT

>Bacillus_Fam_1627_36_1 Nr. of seq. 1 Alignment length(with gaps) = 36
Alignment score = 0.000000
GCF_001645555.1_ASM164555v1_genomic.fna_12:311932-312166 Satlength=235 Nr of Repeats=7 RepeatLength=36 seed=GTTACTCCCG Num.seqs=6
Similarity=0.911111 0
GTTACTCCCGGTGATCCCGTTAAACCAGTTGCACCT

****
Consensus:

GTTACTCCCGGTGATCCCGTTAAACCAGTTGCACCT

>Bacillus_Fam_1628_36_1 Nr. of seq. 1 Alignment length(with gaps) = 36
Alignment score = 0.000000
GCF_001712755.1_ASM171275v1_genomic.fna_35:221909-222269 Satlength=361 Nr of Repeats=10 RepeatLength=36 seed=TTTCCTGTTG Num.seqs=10
Similarity=0.819753 0
TTTCCTGTTGATACTGGTGATACTTGCATATTTGGT

****
Consensus:

TTTCCTGTTGATACTGGTGATACTTGCATATTTGGT

>Bacillus_Fam_1629_36_1 Nr. of seq. 1 Alignment length(with gaps) = 36
Alignment score = 0.000000
GCF_002019645.1_ASM201964v1_genomic.fna_1:2358432-2358672 Satlength=241 Nr of Repeats=6 RepeatLength=36 seed=CAGGAGCACC Num.seqs=4
Similarity=0.962963 0
CAGGAGCACCGGGAGCAGCAGCTGGAACTCCATATC

****
Consensus:

CAGGAGCACCGGGAGCAGCAGCTGGAACTCCATATC

>Bacillus_Fam_1630_36_1 Nr. of seq. 1 Alignment length(with gaps) = 36
Alignment score = 0.000000
GCF_002019765.1_ASM201976v1_genomic.fna_1:651174-652542 Satlength=1369 Nr of Repeats=42 RepeatLength=36 seed=AGGAGAAACA Num.seqs=30
Similarity=0.903293 0
AGGAGAAACAGGGGCGACTGGTAGTCAAGGAGTTCA

****
Consensus:

AGGAGAAACAGGGGCGACTGGTAGTCAAGGAGTTCA

>Bacillus_Fam_1631_36_1 Nr. of seq. 1 Alignment length(with gaps) = 36
Alignment score = 0.000000
GCF_900098925.1_PRJEB15625_genomic.fna_16:204570-204750 Satlength=181 Nr of Repeats=5 RepeatLength=36 seed=GGCATCATTC Num.seqs=5
Similarity=0.925926
0 GGCATCATTCCCGGCATGCCTCCTTGCATTTGTCCC

****
Consensus:

GGCATCATTCCCGGCATGCCTCCTTGCATTTGTCCC

>Bacillus_Fam_1632_36_1 Nr. of seq. 1 Alignment length(with gaps) = 36
Alignment score = 0.000000
GCF_900104555.1_IMG-taxon_2651870172_annotated_assembly_genomic.fna_17:0-143 Satlength=144 Nr of Repeats=4 RepeatLength=36 seed=CAGGTCCAAA
Num.seqs=4 Similarity=0.876543 0
CAGGTCCAAAAGGAAAAGAAGGCCCACCCGGTCCAG

****
Consensus:

CAGGTCCAAAAGGAAAAGAAGGCCCACCCGGTCCAG

>Bacillus_Fam_1633_36_1 Nr. of seq. 1 Alignment length(with gaps) = 36
Alignment score = 0.000000
GCF_900104555.1_IMG-taxon_2651870172_annotated_assembly_genomic.fna_20:439434-439668
Satlength=235 Nr of Repeats=6 RepeatLength=36 seed=CTCCTGGTAC Num.seqs=5
Similarity=0.881481 0
CTCCTGGTACTGGATTTAATCCTGGCATGGGTTATG

****
Consensus:

CTCCTGGTACTGGATTTAATCCTGGCATGGGTTATG

>Bacillus_Fam_1634_34_1 Nr. of seq. 1 Alignment length(with gaps) = 34
Alignment score = 0.000000
GCF_000752035.1_Bacillus_rubiinfantis_genomic.fna_11:633923-634127
Satlength=205 Nr of Repeats=6 RepeatLength=34 seed=AACGTTTGAT Num.seqs=4
Similarity=0.676698 0
AACGTTTGATTGACTTCAGCGGTTCTACTAACTA

****
Consensus:

AACGTTTGATTGACTTCAGCGGTTCTACTAACTA

>Bacillus_Fam_1635_34_1 Nr. of seq. 1 Alignment length(with gaps) = 34
Alignment score = 0.000000

GCF_900111815.1_PRJEB17078_genomic.fna_1:193561-194089 Satlength=529 Nr of Repeats=4 RepeatLength=34 seed=GGGGGTCAGG Num.seqs=3 Similarity=0.895425
0   GGGGGTCAGGCACGCTTTAATGATTCACCGCACT

****
Consensus:

GGGGGTCAGGCACGCTTTAATGATTCACCGCACT

>Bacillus_Fam_1636_33_1 Nr. of seq. 1 Alignment length(with gaps) = 33 Alignment score = 0.000000
BACILLUS_PSEUDALCALIPHILUS_genomic.fna_5:362967-363159 Satlength=193 Nr of Repeats=6 RepeatLength=33 seed=GGAATGATGC Num.seqs=5 Similarity=0.862626
0   GGAATGATGCCAATGCCAGGTCAAAACGGAAAT

****
Consensus:

GGAATGATGCCAATGCCAGGTCAAAACGGAAAT

>Bacillus_Fam_1637_33_1 Nr. of seq. 1 Alignment length(with gaps) = 33 Alignment score = 0.000000
GCF_000009825.1_ASM982v1_genomic.fna_1:3221981-3222245 Satlength=265 Nr of Repeats=8 RepeatLength=33 seed=GAACAAGAGC Num.seqs=8 Similarity=0.911977
0   GAACAAGAGCCTGAAACTGACGCTAGCACAGAC

****
Consensus:

GAACAAGAGCCTGAAACTGACGCTAGCACAGAC

>Bacillus_Fam_1638_33_1 Nr. of seq. 1 Alignment length(with gaps) = 33 Alignment score = 0.000000
GCF_000380245.2_ASM38024v2_genomic.fna_12:1180413-1180611 Satlength=199 Nr of Repeats=6 RepeatLength=33 seed=AACAAATCCG Num.seqs=6 Similarity=0.973064   0
AACAAATCCGCCGTCAACAGGAGGAAATGAATC

****
Consensus:

AACAAATCCGCCGTCAACAGGAGGAAATGAATC

>Bacillus_Fam_1639_33_1 Nr. of seq. 1 Alignment length(with gaps) = 33 Alignment score = 0.000000
GCF_000504145.1_SOAPdenovo_for_version2.04_of_Bacillus_sp._FJAT-14515_genome_genomic.fna_1:1765976-1769141 Satlength=3166 Nr of Repeats=101 RepeatLength=33 seed=GGCATGCCTC Num.seqs=70 Similarity=0.84
0   GGCATGCCTCCACTTGGTTGACCTCCCAATCCC

****
Consensus:

GGCATGCCTCCACTTGGTTGACCTCCCAATCCC

>Bacillus_Fam_1640_33_1 Nr. of seq. 1 Alignment length(with gaps) = 33 Alignment score = 0.000000
GCF_000612805.1_FF3_genomic.fna_3:320276-320474 Satlength=199 Nr of Repeats=6 RepeatLength=33 seed=TAGAGCATAG Num.seqs=6 Similarity=0.843771
0 TAGAGCATAGATTAGAAGGCAAAATAGAAGAAC

****
Consensus:

TAGAGCATAGATTAGAAGGCAAAATAGAAGAAC

>Bacillus_Fam_1641_33_1 Nr. of seq. 1 Alignment length(with gaps) = 33 Alignment score = 0.000000
GCF_000712615.1_ASM71261v1_genomic.fna_2:456487-456652 Satlength=166 Nr of Repeats=5 RepeatLength=33 seed=ATTGTTGGAA Num.seqs=5 Similarity=0.919192
0 ATTGTTGGAAGTCTAAGGATAAACATCATTCTG

****
Consensus:

ATTGTTGGAAGTCTAAGGATAAACATCATTCTG

>Bacillus_Fam_1642_33_1 Nr. of seq. 1 Alignment length(with gaps) = 33 Alignment score = 0.000000
GCF_000752395.1_Bacillus_andreraoultii_genomic.fna_56:326201-326366 Satlength=166 Nr of Repeats=5 RepeatLength=33 seed=CAACTAACAG Num.seqs=5 Similarity=0.854545 0
CAACTAACAGAAACGAAAGATAACTTACAGTCA

****
Consensus:

CAACTAACAGAAACGAAAGATAACTTACAGTCA

>Bacillus_Fam_1643_33_1 Nr. of seq. 1 Alignment length(with gaps) = 33 Alignment score = 0.000000
GCF_000775975.1_ASM77597v1_genomic.fna_1:1324054-1324219 Satlength=166 Nr of Repeats=5 RepeatLength=33 seed=ATCAGCATGA Num.seqs=5 Similarity=0.919192 0
ATCAGCATGACCTTCTTGCTTAGGAGCAGGGAA

****
Consensus:

ATCAGCATGACCTTCTTGCTTAGGAGCAGGGAA

>Bacillus_Fam_1644_33_1 Nr. of seq. 1 Alignment length(with gaps) = 33 Alignment score = 0.000000

GCF_000787375.1_ASM78737v1_genomic.fna_14:44934-45126 Satlength=193 Nr of Repeats=5 RepeatLength=33 seed=AAAAGAAGAA Num.seqs=4 Similarity=0.872054
0 AAAAGAAGAAAGCTTGTCGCAGCGAGAAAAGCA

****
Consensus:

AAAAGAAGAAAGCTTGTCGCAGCGAGAAAAGCA

>Bacillus_Fam_1645_33_1 Nr. of seq. 1 Alignment length(with gaps) = 33
Alignment score = 0.000000
GCF_001243895.1_Bacillus_testis_genomic.fna_9:2298830-2299094
Satlength=265 Nr of Repeats=8 RepeatLength=33 seed=GGTTGGTTTT Num.seqs=8
Similarity=0.888889 0
GGTTGGTTTTGTTCTGGTGTGTTGTCCACTTCC

****
Consensus:

GGTTGGTTTTGTTCTGGTGTGTTGTCCACTTCC

>Bacillus_Fam_1646_33_1 Nr. of seq. 1 Alignment length(with gaps) = 33
Alignment score = 0.000000
GCF_001587375.1_ASM158737v1_genomic.fna_152:5556-5678 Satlength=123 Nr of Repeats=4 RepeatLength=33 seed=AGTGTTTTAC Num.seqs=3 Similarity=0.688889
0 AGTGTTTTACTAACTTNTTCATCTAATTCAGTT

****
Consensus:

AGTGTTTTACTAACTTNTTCATCTAATTCAGTT

>Bacillus_Fam_1647_33_1 Nr. of seq. 1 Alignment length(with gaps) = 33
Alignment score = 0.000000
GCF_001636325.1_ASM163632v1_genomic.fna_2:478165-478297 Satlength=133 Nr of Repeats=4 RepeatLength=33 seed=TAAGTTTGAT Num.seqs=4
Similarity=0.939394
0 TAAGTTTGATGGAAAGCTTGAGGATTTAAGCAA

****
Consensus:

TAAGTTTGATGGAAAGCTTGAGGATTTAAGCAA

>Bacillus_Fam_1648_33_1 Nr. of seq. 1 Alignment length(with gaps) = 33
Alignment score = 0.000000
GCF_001636335.1_ASM163633v1_genomic.fna_1:2556735-2556966 Satlength=232
Nr of Repeats=7 RepeatLength=33 seed=GTTAAACCTT Num.seqs=7
Similarity=0.896104 0
GTTAAACCTTGGTTCATTTCATTTCTTACTTCC

****
Consensus:

GTTAAACCTTGGTTCATTTCATTTCTTACTTCC

>Bacillus_Fam_1649_33_1 Nr. of seq. 1 Alignment length(with gaps) = 33
Alignment score = 0.000000
GCF_900094975.1_IMG-
taxon_2617270721_annotated_assembly_genomic.fna_4:325661-325793
Satlength=133 Nr of Repeats=4 RepeatLength=33 seed=CTGACGTACA Num.seqs=4
Similarity=0.684921 0
CTGACGTACAAGCGGAAATGCAAGAGTTAAGAA

****
Consensus:

CTGACGTACAAGCGGAAATGCAAGAGTTAAGAA

>Bacillus_Fam_1650_33_1 Nr. of seq. 1 Alignment length(with gaps) = 33
Alignment score = 0.000000
GCF_900156865.1_PRJEB18960_genomic.fna_3:2936055-2936187 Satlength=133 Nr
of Repeats=4 RepeatLength=33 seed=ATTTCATCTT Num.seqs=4
Similarity=0.912458 0
ATTTCATCTTTTAAGTCCACTCTCACTACCGAG

****
Consensus:

ATTTCATCTTTTAAGTCCACTCTCACTACCGAG

>Bacillus_Fam_1651_32_1 Nr. of seq. 1 Alignment length(with gaps) = 32
Alignment score = 0.000000
GCF_900156865.1_PRJEB18960_genomic.fna_3:759687-760190 Satlength=504 Nr
of Repeats=16 RepeatLength=32 seed=CAGTTGTACA Num.seqs=10
Similarity=0.856481 0
CAGTTGTACAAGAACCCCTGTTTGCCAACTGT

****
Consensus:

CAGTTGTACAAGAACCCCTGTTTGCCAACTGT

>Bacillus_Fam_1652_31_1 Nr. of seq. 1 Alignment length(with gaps) = 31
Alignment score = 0.000000
GCF_000331575.1_ASM33157v1_genomic.fna_4:6822-6946 Satlength=125 Nr of
Repeats=4 RepeatLength=31 seed=TAGAATATAT Num.seqs=4 Similarity=0.913979
0 TAGAATATATGAGAAAACGTTAATTCCGTTG

****
Consensus:

TAGAATATATGAGAAAACGTTAATTCCGTTG

>Bacillus_Fam_1653_30_1 Nr. of seq. 1 Alignment length(with gaps) = 30
Alignment score = 0.000000

GCF_000008505.1_ASM850v1_genomic.fna_1:4693242-4693602 Satlength=361 Nr of Repeats=12 RepeatLength=30 seed=GGATCTTCCG Num.seqs=12 Similarity=0.934680 0
GGATCTTCCGGTTTCACTTCTGGTTCTTTC

****
Consensus:

GGATCTTCCGGTTTCACTTCTGGTTCTTTC

>Bacillus_Fam_1654_30_1 Nr. of seq. 1 Alignment length(with gaps) = 30 Alignment score = 0.000000
GCF_000025825.1_ASM2582v1_genomic.fna_7:66068-66788 Satlength=721 Nr of Repeats=24 RepeatLength=30 seed=CTTCATCCTA Num.seqs=24 Similarity=0.882770
0   CTTCATCCTATCGTGAGGCCGATCTCGAGA

****
Consensus:

CTTCATCCTATCGTGAGGCCGATCTCGAGA

>Bacillus_Fam_1655_30_1 Nr. of seq. 1 Alignment length(with gaps) = 30 Alignment score = 0.000000
GCF_000245315.1_ASM24531v1_genomic.fna_45:128844-129054 Satlength=211 Nr of Repeats=6 RepeatLength=30 seed=ATGCAAGGCG Num.seqs=5 Similarity=0.866667
0   ATGCAAGGCGGAGGCTACCCGGGCTACGGT

****
Consensus:

ATGCAAGGCGGAGGCTACCCGGGCTACGGT

>Bacillus_Fam_1656_30_1 Nr. of seq. 1 Alignment length(with gaps) = 30 Alignment score = 0.000000
GCF_000331575.1_ASM33157v1_genomic.fna_1:32436-32556 Satlength=121 Nr of Repeats=4 RepeatLength=30 seed=ATATTTCGGT Num.seqs=4 Similarity=0.933333
0   ATATTTCGGTTAGCTTTTCAACATAATGGG

****
Consensus:

ATATTTCGGTTAGCTTTTCAACATAATGGG

>Bacillus_Fam_1657_30_1 Nr. of seq. 1 Alignment length(with gaps) = 30 Alignment score = 0.000000
GCF_000429685.1_ASM42968v1_genomic.fna_3:302688-302835 Satlength=148 Nr of Repeats=5 RepeatLength=30 seed=TGCATGCCCT Num.seqs=4 Similarity=0.933333
0   TGCATGCCCTGCACATTGTTGGGTGGATAC

****
Consensus:

TGCATGCCCTGCACATTGTTGGGTGGATAC

>Bacillus_Fam_1658_30_1 Nr. of seq. 1 Alignment length(with gaps) = 30 Alignment score = 0.000000
GCF_000712615.1_ASM71261v1_genomic.fna_15:76408-77458 Satlength=1051 Nr of Repeats=27 RepeatLength=30 seed=TAATCAAAGA Num.seqs=20 Similarity=0.587214 0
TAANCAAAGAGCCTATGAGTCCAACAGGAG

****
Consensus:

TAANCAAAGAGCCTATGAGTCCAACAGGAG

>Bacillus_Fam_1659_30_1 Nr. of seq. 1 Alignment length(with gaps) = 30 Alignment score = 0.000000
GCF_000712615.1_ASM71261v1_genomic.fna_18:57595-57835 Satlength=241 Nr of Repeats=8 RepeatLength=30 seed=TTTTGTCCAT Num.seqs=8 Similarity=0.898413
0 TTTTGTCCATCCGGCTTCTGCTCTTCTGGA

****
Consensus:

TTTTGTCCATCCGGCTTCTGCTCTTCTGGA

>Bacillus_Fam_1660_30_1 Nr. of seq. 1 Alignment length(with gaps) = 30 Alignment score = 0.000000
GCF_000712615.1_ASM71261v1_genomic.fna_26:20583-20787 Satlength=205 Nr of Repeats=7 RepeatLength=30 seed=AAGAAAAAAG Num.seqs=6 Similarity=0.896296
0 AAGAAAAAAGCTGAAGACGACGCAGCAGCC

****
Consensus:

AAGAAAAAAGCTGAAGACGACGCAGCAGCC

>Bacillus_Fam_1661_30_1 Nr. of seq. 1 Alignment length(with gaps) = 30 Alignment score = 0.000000
GCF_000832605.1_ASM83260v1_genomic.fna_1:3770138-3770468 Satlength=331 Nr of Repeats=11 RepeatLength=30 seed=TTACCTGTTC Num.seqs=11 Similarity=0.936970 0
TTACCTGTTCCAGGGTCTGGGTTTCCTCCA

****
Consensus:

TTACCTGTTCCAGGGTCTGGGTTTCCTCCA

>Bacillus_Fam_1662_30_1 Nr. of seq. 1 Alignment length(with gaps) = 30 Alignment score = 0.000000
GCF_000986785.1_ASM98678v1_genomic.fna_16:91585-91696 Satlength=112 Nr of Repeats=4 RepeatLength=30 seed=CCAGCCAAGC Num.seqs=3 Similarity=0.911111
0 CCAGCCAAGCAGCAGCCTGCAGCTCCTGCA

****
Consensus:

CCAGCCAAGCAGCAGCCTGCAGCTCCTGCA

>Bacillus_Fam_1663_30_1 Nr. of seq. 1 Alignment length(with gaps) = 30
Alignment score = 0.000000
GCF_001273755.1_ASM127375v1_genomic.fna_17:3266495-3266615 Satlength=121
Nr of Repeats=4 RepeatLength=30 seed=TTTTCTTCAG Num.seqs=4
Similarity=0.711111 0
TTTTCTTCAGCTATTTGCTTTGCCTTTGCC

****
Consensus:

TTTTCTTCAGCTATTTGCTTTGCCTTTGCC

>Bacillus_Fam_1664_30_1 Nr. of seq. 1 Alignment length(with gaps) = 30
Alignment score = 0.000000
GCF_001273755.1_ASM127375v1_genomic.fna_17:3552762-3553117 Satlength=356
Nr of Repeats=12 RepeatLength=30 seed=GATGAGCTTT Num.seqs=11
Similarity=0.772121 0
GATGAGCTTTCTGTTGTACTACTTTCNTCT

****
Consensus:

GATGAGCTTTCTGTTGTACTACTTTCNTCT

>Bacillus_Fam_1665_30_1 Nr. of seq. 1 Alignment length(with gaps) = 30
Alignment score = 0.000000
GCF_001315065.1_ASM131506v1_genomic.fna_25:60788-60956 Satlength=169 Nr of Repeats=5 RepeatLength=30 seed=GAGGAAGATC Num.seqs=3
Similarity=0.970370
0 GAGGAAGATCACGATCACGAACATGGACAT

****
Consensus:

GAGGAAGATCACGATCACGAACATGGACAT

>Bacillus_Fam_1666_30_1 Nr. of seq. 1 Alignment length(with gaps) = 30
Alignment score = 0.000000
GCF_001578185.1_ASM157818v1_genomic.fna_1:1359899-1360049 Satlength=151
Nr of Repeats=4 RepeatLength=30 seed=TATGGCGGTC Num.seqs=3
Similarity=0.881481 0
TATGGCGGTCATTCTGGATATGGTATGGGC

****
Consensus:

TATGGCGGTCATTCTGGATATGGTATGGGC

>Bacillus_Fam_1667_30_1 Nr. of seq. 1 Alignment length(with gaps) = 30
Alignment score = 0.000000
GCF_001591585.1_ASM159158v1_genomic.fna_4:226-586 Satlength=361 Nr of Repeats=11 RepeatLength=30 seed=CTTGTTTCGC Num.seqs=10
Similarity=0.853827
0   CTTGTTTCGCTTTTTCCTTCACTAATGCTG

****
Consensus:

CTTGTTTCGCTTTTTCCTTCACTAATGCTG

>Bacillus_Fam_1668_30_1 Nr. of seq. 1 Alignment length(with gaps) = 30
Alignment score = 0.000000
GCF_001591625.1_ASM159162v1_genomic.fna_70:12964-13156 Satlength=193 Nr of Repeats=5 RepeatLength=30 seed=CACCATTGTC Num.seqs=4
Similarity=0.874074
0   CACCATTGTCATGATGGACATAAACCTTGC

****
Consensus:

CACCATTGTCATGATGGACATAAACCTTGC

>Bacillus_Fam_1669_30_1 Nr. of seq. 1 Alignment length(with gaps) = 30
Alignment score = 0.000000
GCF_001645685.2_ASM164568v2_genomic.fna_1:3346551-3346701 Satlength=151 Nr of Repeats=5 RepeatLength=30 seed=TCTTTGATCG Num.seqs=5
Similarity=0.776344 0
TCTTTGATCGGTGCNTGATACTTCGGCTGT

****
Consensus:

TCTTTGATCGGTGCNTGATACTTCGGCTGT

>Bacillus_Fam_1670_30_1 Nr. of seq. 1 Alignment length(with gaps) = 30
Alignment score = 0.000000
GCF_001742425.1_ASM174242v1_genomic.fna_11:173420-173555 Satlength=136 Nr of Repeats=5 RepeatLength=30 seed=TACTACTTCT Num.seqs=4
Similarity=0.948148 0
TACTACTTCTTCAGCTGCCGCTTCTTCAGT

****
Consensus:

TACTACTTCTTCAGCTGCCGCTTCTTCAGT

>Bacillus_Fam_1671_30_1 Nr. of seq. 1 Alignment length(with gaps) = 30
Alignment score = 0.000000
GCF_002019595.1_ASM201959v1_genomic.fna_1:1449566-1449722 Satlength=157 Nr of Repeats=5 RepeatLength=30 seed=CCAGGACCAG Num.seqs=4
Similarity=0.955556 0
CCAGGACCAGGCATGATGCCAGGAGGAATG

****
Consensus:

CCAGGACCAGGCATGATGCCAGGAGGAATG

>Bacillus_Fam_1672_30_1 Nr. of seq. 1 Alignment length(with gaps) = 30 Alignment score = 0.000000 GCF_900156865.1_PRJEB18960_genomic.fna_3:1860072-1860222 Satlength=151 Nr of Repeats=5 RepeatLength=30 seed=CCACCTTGTG Num.seqs=5 Similarity=0.763441 0 CCACCTTGTGGTTGCTTTGGCTGCTGTGCT

****
Consensus:

CCACCTTGTGGTTGCTTTGGCTGCTGTGCT

>Bacillus_Fam_1673_29_1 Nr. of seq. 1 Alignment length(with gaps) = 29 Alignment score = 0.000000 GCF_000331575.1_ASM33157v1_genomic.fna_4:32194-32368 Satlength=175 Nr of Repeats=6 RepeatLength=29 seed=TTGCGGTAAT Num.seqs=6 Similarity=0.898851 0 TTGCGGTAATAAAACTTCTAATACCCCTT

****
Consensus:

TTGCGGTAATAAAACTTCTAATACCCCTT

>Bacillus_Fam_1674_28_1 Nr. of seq. 1 Alignment length(with gaps) = 28 Alignment score = 0.000000 GCF_001636335.1_ASM163633v1_genomic.fna_1:2184233-2184341 Satlength=109 Nr of Repeats=4 RepeatLength=27 seed=TTGGCATTAA Num.seqs=4 Similarity=0.769841 0 TTGGCATTAACGGCATTTGATTTTGCGN

****
Consensus:

TTGGCATTAACGGCATTTGATTTTGCGN

>Bacillus_Fam_1675_28_1 Nr. of seq. 1 Alignment length(with gaps) = 28 Alignment score = 0.000000 GCF_002019705.1_ASM201970v1_genomic.fna_1:3847148-3847274 Satlength=127 Nr of Repeats=6 RepeatLength=27 seed=CCAGTAACGC Num.seqs=4 Similarity=0.714286 0 CCAGTAACGCCAGTAACACCTGTNTAAG

****
Consensus:

CCAGTAACGCCAGTAACACCTGTNTAAG

>Bacillus_Fam_1676_27_1 Nr. of seq. 1 Alignment length(with gaps) = 27 Alignment score = 0.000000
BACILLUS_PSEUDALCALIPHILUS_genomic.fna_2:129576-130332 Satlength=757 Nr of Repeats=20 RepeatLength=27 seed=GGACCAACAG Num.seqs=15 Similarity=0.854203 0
GGACCAACAGGNGAGCAAGGGATCCAA

****
Consensus:

GGACCAACAGGNGAGCAAGGGATCCAA

>Bacillus_Fam_1677_27_1 Nr. of seq. 1 Alignment length(with gaps) = 27 Alignment score = 0.000000
GCF_000177235.2_ASM17723v2_genomic.fna_1:1354085-1354274 Satlength=190 Nr of Repeats=7 RepeatLength=27 seed=GGAGATGACG Num.seqs=5 Similarity=0.695556 0
GGAGATGACGGCGAAACNCCAGGCACA

****
Consensus:

GGAGATGACGGCGAAACNCCAGGCACA

>Bacillus_Fam_1678_27_1 Nr. of seq. 1 Alignment length(with gaps) = 27 Alignment score = 0.000000
GCF_000504145.1_SOAPdenovo_for_version2.04_of_Bacillus_sp._FJAT-14515_genome_genomic.fna_27:57375-57516 Satlength=142 Nr of Repeats=5 RepeatLength=27 seed=CCACCATATC Num.seqs=3 Similarity=0.769547 0
CCACCATATCCATGATGGTACGGGTAA

****
Consensus:

CCACCATATCCATGATGGTACGGGTAA

>Bacillus_Fam_1679_27_1 Nr. of seq. 1 Alignment length(with gaps) = 27 Alignment score = 0.000000
GCF_000507145.1_KCTC_13622_01_genomic.fna_39:156813-156975 Satlength=163 Nr of Repeats=6 RepeatLength=27 seed=CCATACATGC Num.seqs=6 Similarity=0.947325 0
CCATACATGCCGGATCCTGGGAAACCC

****
Consensus:

CCATACATGCCGGATCCTGGGAAACCC

>Bacillus_Fam_1680_27_1 Nr. of seq. 1 Alignment length(with gaps) = 27 Alignment score = 0.000000
GCF_000621445.1_ASM62144v1_genomic.fna_1:408565-408724 Satlength=160 Nr of Repeats=6 RepeatLength=27 seed=TCTTCTTCTC Num.seqs=5 Similarity=0.841667
0   TCTTCTTCTCCTCCCGGTGGCAACTCT

****
Consensus:

TCTTCTTCTCCTCCCGGTGGCAACTCT

>Bacillus_Fam_1681_27_1 Nr. of seq. 1 Alignment length(with gaps) = 27
Alignment score = 0.000000
GCF_000712615.1_ASM71261v1_genomic.fna_26:13505-13964 Satlength=460 Nr of Repeats=17 RepeatLength=27 seed=CCTGGTCCGA Num.seqs=17
Similarity=1.000000
0   CCTGGTCCGAATGGAGATACGTTTGGC

****
Consensus:

CCTGGTCCGAATGGAGATACGTTTGGC

>Bacillus_Fam_1682_27_1 Nr. of seq. 1 Alignment length(with gaps) = 27
Alignment score = 0.000000
GCF_000829555.1_ASM82955v1_genomic.fna_1:540159-540303 Satlength=145 Nr of Repeats=6 RepeatLength=27 seed=CCCGGTTCTC Num.seqs=4
Similarity=0.917695
0   CCCGGTTCTCCTGGCTCTCCCGGTTTA

****
Consensus:

CCCGGTTCTCCTGGCTCTCCCGGTTTA

>Bacillus_Fam_1683_27_1 Nr. of seq. 1 Alignment length(with gaps) = 27
Alignment score = 0.000000
GCF_001274775.1_ASM127477v1_genomic.fna_3:360890-361097 Satlength=208 Nr of Repeats=9 RepeatLength=27 seed=CACAGGGGCC Num.seqs=7
Similarity=0.727219
0   CACAGGGGCCACAAGGGTTGACGGGGN

****
Consensus:

CACAGGGGCCACAAGGGTTGACGGGGN

>Bacillus_Fam_1684_27_1 Nr. of seq. 1 Alignment length(with gaps) = 27
Alignment score = 0.000000
GCF_001315065.1_ASM131506v1_genomic.fna_30:1799-1955 Satlength=157 Nr of Repeats=6 RepeatLength=27 seed=CCAGAGCCTC Num.seqs=5 Similarity=0.930864
0   CCAGAGCCTCCTTCTCCGTCTCCAGTG

****
Consensus:

CCAGAGCCTCCTTCTCCGTCTCCAGTG

>Bacillus_Fam_1685_27_1 Nr. of seq. 1 Alignment length(with gaps) = 27
Alignment score = 0.000000
GCF_001439635.1_ASM143963v1_genomic.fna_165:21253-21388 Satlength=136 Nr of Repeats=4 RepeatLength=27 seed=TTTCTGAAAT Num.seqs=3 Similarity=0.595238
0   TTTCTGAAATTGCTNCCACTTCTTGAN

****
Consensus:

TTTCTGAAATTGCTNCCACTTCTTGAN

>Bacillus_Fam_1686_27_1 Nr. of seq. 1 Alignment length(with gaps) = 27
Alignment score = 0.000000
GCF_001578185.1_ASM157818v1_genomic.fna_1:2745149-2745257 Satlength=109 Nr of Repeats=6 RepeatLength=27 seed=TGAAGCTGTT Num.seqs=4 Similarity=0.909465 0
TGAAGCTGTTGAAGCTGTTGATGCTCT

****
Consensus:

TGAAGCTGTTGAAGCTGTTGATGCTCT

>Bacillus_Fam_1687_27_1 Nr. of seq. 1 Alignment length(with gaps) = 27
Alignment score = 0.000000
GCF_001591625.1_ASM159162v1_genomic.fna_178:4262-4433 Satlength=172 Nr of Repeats=6 RepeatLength=27 seed=GAGGTCCTTG Num.seqs=4 Similarity=0.901235
0   GAGGTCCTTGAGGCCCAGTTGCTCCCT

****
Consensus:

GAGGTCCTTGAGGCCCAGTTGCTCCCT

>Bacillus_Fam_1688_27_1 Nr. of seq. 1 Alignment length(with gaps) = 27
Alignment score = 0.000000
GCF_001636325.1_ASM163632v1_genomic.fna_2:1328784-1328973 Satlength=190 Nr of Repeats=7 RepeatLength=27 seed=AGCAAAAACC Num.seqs=5 Similarity=0.743210 0
AGCAAAAACCGTTGCAGCCTATTCAAC

****
Consensus:

AGCAAAAACCGTTGCAGCCTATTCAAC

>Bacillus_Fam_1689_27_1 Nr. of seq. 1 Alignment length(with gaps) = 27
Alignment score = 0.000000
GCF_001636425.1_ASM163642v1_genomic.fna_2:151590-151686 Satlength=97 Nr of Repeats=4 RepeatLength=27 seed=GGTTCTACTG Num.seqs=3 Similarity=0.934156
0   GGTTCTACTGGTTCAACAGGAACATCA

****
Consensus:

GGTTCTACTGGTTCAACAGGAACATCA

>Bacillus_Fam_1690_27_1 Nr. of seq. 1 Alignment length(with gaps) = 27
Alignment score = 0.000000
GCF_001645685.2_ASM164568v2_genomic.fna_1:3248002-3248306 Satlength=305
Nr of Repeats=10 RepeatLength=27 seed=AACTGCTTGA Num.seqs=9
Similarity=0.950617 0
AACTGCTTGATGAGCTCTCGCATGAGG

****
Consensus:

AACTGCTTGATGAGCTCTCGCATGAGG

>Bacillus_Fam_1691_27_1 Nr. of seq. 1 Alignment length(with gaps) = 27
Alignment score = 0.000000
GCF_001648575.1_ASM164857v1_genomic.fna_8:6481-6859 Satlength=379 Nr of
Repeats=13 RepeatLength=27 seed=CCTTCTGGTC Num.seqs=12
Similarity=0.976057
0   CCTTCTGGTCCCGCTGGTCCTATTGCT

****
Consensus:

CCTTCTGGTCCCGCTGGTCCTATTGCT

>Bacillus_Fam_1692_27_1 Nr. of seq. 1 Alignment length(with gaps) = 27
Alignment score = 0.000000
GCF_001712755.1_ASM171275v1_genomic.fna_36:39981-40521 Satlength=541 Nr
of Repeats=20 RepeatLength=27 seed=CCTGCATTTC Num.seqs=20
Similarity=0.893437 0
CCTGCATTTCCTGTTCTTTCTGCTCTG

****
Consensus:

CCTGCATTTCCTGTTCTTTCTGCTCTG

>Bacillus_Fam_1693_27_1 Nr. of seq. 1 Alignment length(with gaps) = 27
Alignment score = 0.000000
GCF_001887185.1_ASM188718v1_genomic.fna_197:67384-67492 Satlength=109 Nr
of Repeats=4 RepeatLength=27 seed=GCCAATTTTA Num.seqs=4
Similarity=0.975309
0   GCCAATTTTACTAGGTAAAATTCTACT

****
Consensus:

GCCAATTTTACTAGGTAAAATTCTACT

>Bacillus_Fam_1694_27_1 Nr. of seq. 1 Alignment length(with gaps) = 27
Alignment score = 0.000000
GCF_002019605.1_ASM201960v1_genomic.fna_1:2443028-2443136 Satlength=109
Nr of Repeats=4 RepeatLength=27 seed=TCATGTCCAT Num.seqs=4
Similarity=0.942387   0
TCATGTCCATGCTCTTCATGCTCGTGA

****
Consensus:

TCATGTCCATGCTCTTCATGCTCGTGA

>Bacillus_Fam_1695_26_1 Nr. of seq. 1 Alignment length(with gaps) = 26
Alignment score = 0.000000
GCF_001877785.1_PlanoSAMM_genomic.fna_128:141796-141971 Satlength=176 Nr
of Repeats=7 RepeatLength=25 seed=TGTACCAGGT Num.seqs=7
Similarity=0.567460
0   TGTACCAGGTACAANACCNCAACTNN

****
Consensus:

TGTACCAGGTACAANACCNCAACTNN

>Bacillus_Fam_1696_25_1 Nr. of seq. 1 Alignment length(with gaps) = 25
Alignment score = 0.000000
GCF_000331575.1_ASM33157v1_genomic.fna_4:37610-37783 Satlength=174 Nr of
Repeats=7 RepeatLength=25 seed=TACTACCCTA Num.seqs=5 Similarity=0.957333
0   TACTACCCTATGGTAAAATATGTAA

****
Consensus:

TACTACCCTATGGTAAAATATGTAA

>Bacillus_Fam_1697_25_1 Nr. of seq. 1 Alignment length(with gaps) = 25
Alignment score = 0.000000
GCF_001877785.1_PlanoSAMM_genomic.fna_117:71061-71186 Satlength=126 Nr of
Repeats=5 RepeatLength=25 seed=TGTACCTGGT Num.seqs=5 Similarity=0.553086
0   TGTACCTGGTACATGANCCNGTNNT

****
Consensus:

TGTACCTGGTACATGANCCNGTNNT

>Bacillus_Fam_1698_24_1 Nr. of seq. 1 Alignment length(with gaps) = 24
Alignment score = 0.000000
GCF_000161455.1_ASM16145v1_genomic.fna_1:1443980-1444148 Satlength=169 Nr
of Repeats=7 RepeatLength=24 seed=TGGAGTTGGA Num.seqs=7
Similarity=1.000000   0
TGGAGTTGGAGGATTCGGCGGACC

****

Consensus:

TGGAGTTGGAGGATTCGGCGGACC

>Bacillus_Fam_1699_24_1 Nr. of seq. 1 Alignment length(with gaps) = 24
Alignment score = 0.000000
GCF_000186125.1_Bacillus_BT1B_CT2_V1_genomic.fna_1:1895116-1895212
Satlength=97 Nr of Repeats=4 RepeatLength=24 seed=GGGACACAGC Num.seqs=4
Similarity=0.826923 0
GGGACACAGCTGTTGGAGCTGTGC

****
Consensus:

GGGACACAGCTGTTGGAGCTGTGC

>Bacillus_Fam_1700_24_1 Nr. of seq. 1 Alignment length(with gaps) = 24
Alignment score = 0.000000
GCF_000245335.1_ASM24533v1_genomic.fna_8:553929-554097 Satlength=169 Nr
of Repeats=7 RepeatLength=24 seed=CCTCCGCCAA Num.seqs=7
Similarity=0.756614
0 CCTCCGCCAATACCAGGCACGGCG

****
Consensus:

CCTCCGCCAATACCAGGCACGGCG

>Bacillus_Fam_1701_24_1 Nr. of seq. 1 Alignment length(with gaps) = 24
Alignment score = 0.000000
GCF_000307855.1_BAZO1.0_genomic.fna_16:13575-13719 Satlength=145 Nr of
Repeats=6 RepeatLength=24 seed=GGAGGATTTT Num.seqs=6 Similarity=0.900000
0 GGAGGATTTTGCGCAGGCAGGTGC

****
Consensus:

GGAGGATTTTGCGCAGGCAGGTGC

>Bacillus_Fam_1702_24_1 Nr. of seq. 1 Alignment length(with gaps) = 24
Alignment score = 0.000000
GCF_000331575.1_ASM33157v1_genomic.fna_1:75628-75724 Satlength=97 Nr of
Repeats=4 RepeatLength=24 seed=ACTGTTTTTC Num.seqs=4 Similarity=0.907407
0 ACTGTTTTTCGTTCTGCTATTTAC

****
Consensus:

ACTGTTTTTCGTTCTGCTATTTAC

>Bacillus_Fam_1703_24_1 Nr. of seq. 1 Alignment length(with gaps) = 24
Alignment score = 0.000000
GCF_000331575.1_ASM33157v1_genomic.fna_3:126837-127053 Satlength=217 Nr
of Repeats=9 RepeatLength=24 seed=GACCAAAACA Num.seqs=9

Similarity=0.833333
0   GACCAAAACACAATACAGAAGTTG

****
Consensus:

GACCAAAACACAATACAGAAGTTG

>Bacillus_Fam_1704_24_1 Nr. of seq. 1 Alignment length(with gaps) = 24
Alignment score = 0.000000
GCF_000374565.1_ASM37456v1_genomic.fna_6:153087-153207 Satlength=121 Nr of Repeats=5 RepeatLength=24 seed=CCGGGTGAAG Num.seqs=5
Similarity=0.922222
0   CCGGGTGAAGGGCCAGGAGAAAAC

****
Consensus:

CCGGGTGAAGGGCCAGGAGAAAAC

>Bacillus_Fam_1705_24_1 Nr. of seq. 1 Alignment length(with gaps) = 24
Alignment score = 0.000000
GCF_000496285.1_ASM49628v1_genomic.fna_1:3406349-3406469 Satlength=121 Nr of Repeats=5 RepeatLength=24 seed=AGTGTCTTTT Num.seqs=5
Similarity=0.922222 0
AGTGTCTTTTGTTCTCTCAGAGTT

****
Consensus:

AGTGTCTTTTGTTCTCTCAGAGTT

>Bacillus_Fam_1706_24_1 Nr. of seq. 1 Alignment length(with gaps) = 24
Alignment score = 0.000000
GCF_000504145.1_SOAPdenovo_for_version2.04_of_Bacillus_sp._FJAT-14515_genome_genomic.fna_1:918376-919127 Satlength=752 Nr of Repeats=28 RepeatLength=24 seed=TCGGCACTTT Num.seqs=18 Similarity=0.714597   0
TCGGCACTTTCATCTNCACTTTCG

****
Consensus:

TCGGCACTTTCATCTNCACTTTCG

>Bacillus_Fam_1707_24_1 Nr. of seq. 1 Alignment length(with gaps) = 24
Alignment score = 0.000000
GCF_000612625.1_JCE_genomic.fna_19:40197-40437 Satlength=241 Nr of Repeats=10 RepeatLength=24 seed=TCCTCCAGTG Num.seqs=10
Similarity=0.944444
0   TCCTCCAGTGCTGCCTCCGTTGTC

****
Consensus:

TCCTCCAGTGCTGCCTCCGTTGTC

>Bacillus_Fam_1708_24_1 Nr. of seq. 1 Alignment length(with gaps) = 24 Alignment score = 0.000000
GCF_000712595.1_ASM71259v1_genomic.fna_3:200804-201365 Satlength=562 Nr of Repeats=23 RepeatLength=24 seed=TTACCTTGGC Num.seqs=20 Similarity=0.994444 0
TTACCTTGGCCACCACCTTGGTCG

****
Consensus:

TTACCTTGGCCACCACCTTGGTCG

>Bacillus_Fam_1709_24_1 Nr. of seq. 1 Alignment length(with gaps) = 24 Alignment score = 0.000000
GCF_000712595.1_ASM71259v1_genomic.fna_14:27323-27635 Satlength=313 Nr of Repeats=13 RepeatLength=24 seed=TTGTACTTGC Num.seqs=13 Similarity=0.921652
0 TTGTACTTGCTCCTGCACCGGCTC

****
Consensus:

TTGTACTTGCTCCTGCACCGGCTC

>Bacillus_Fam_1710_24_1 Nr. of seq. 1 Alignment length(with gaps) = 24 Alignment score = 0.000000
GCF_000712615.1_ASM71261v1_genomic.fna_12:99179-99620 Satlength=442 Nr of Repeats=18 RepeatLength=24 seed=CCATGATGAT Num.seqs=17 Similarity=1.000000
0 CCATGATGATTGTGATGGTGTCCG

****
Consensus:

CCATGATGATTGTGATGGTGTCCG

>Bacillus_Fam_1711_24_1 Nr. of seq. 1 Alignment length(with gaps) = 24 Alignment score = 0.000000
GCF_000724485.1_ASM72448v1_genomic.fna_1:2088487-2088577 Satlength=91 Nr of Repeats=4 RepeatLength=24 seed=CCTGGTCCAT Num.seqs=3 Similarity=0.962963
0 CCTGGTCCATAACCAGGGCCCATA

****
Consensus:

CCTGGTCCATAACCAGGGCCCATA

>Bacillus_Fam_1712_24_1 Nr. of seq. 1 Alignment length(with gaps) = 24 Alignment score = 0.000000

GCF_000724485.1_ASM72448v1_genomic.fna_1:2102157-2102379 Satlength=223 Nr of Repeats=8 RepeatLength=24 seed=CGGTACTGGC Num.seqs=7 Similarity=0.915344 0
CGGTACTGGCGCCGGTGGCAACGG

****
Consensus:

CGGTACTGGCGCCGGTGGCAACGG

>Bacillus_Fam_1713_24_1 Nr. of seq. 1 Alignment length(with gaps) = 24
Alignment score = 0.000000
GCF_000775975.1_ASM77597v1_genomic.fna_1:3273312-3273480 Satlength=169 Nr of Repeats=6 RepeatLength=24 seed=TGATTCATAT Num.seqs=5 Similarity=0.777778 0
TGATTCATATGCGCTTGTTGCGGC

****
Consensus:

TGATTCATATGCGCTTGTTGCGGC

>Bacillus_Fam_1714_24_1 Nr. of seq. 1 Alignment length(with gaps) = 24
Alignment score = 0.000000
GCF_001274935.1_ASM127493v1_genomic.fna_1:6173-6293 Satlength=121 Nr of Repeats=5 RepeatLength=24 seed=GGTGGCGTTG Num.seqs=5 Similarity=0.866667
0 GGTGGCGTTGTACCTCCATCTCCG

****
Consensus:

GGTGGCGTTGTACCTCCATCTCCG

>Bacillus_Fam_1715_24_1 Nr. of seq. 1 Alignment length(with gaps) = 24
Alignment score = 0.000000
GCF_001420595.1_ASM142059v1_genomic.fna_11:3031878-3031998 Satlength=121 Nr of Repeats=5 RepeatLength=24 seed=GTTTGTGCTG Num.seqs=5 Similarity=0.933333 0
GTTTGTGCTGGCTTTTGAGTCTGA

****
Consensus:

GTTTGTGCTGGCTTTTGAGTCTGA

>Bacillus_Fam_1716_24_1 Nr. of seq. 1 Alignment length(with gaps) = 24
Alignment score = 0.000000
GCF_001420715.1_ASM142071v1_genomic.fna_22:560318-560419 Satlength=102 Nr of Repeats=5 RepeatLength=20 seed=GGCTAATAGA Num.seqs=4 Similarity=0.528935 0
GGCTAATAGANCGAAAGTGATAAC

****
Consensus:

GGCTAATAGANCGAAAGTGATAAC

>Bacillus_Fam_1717_24_1 Nr. of seq. 1 Alignment length(with gaps) = 24
Alignment score = 0.000000
GCF_001578185.1_ASM157818v1_genomic.fna_1:4072028-4072112 Satlength=85 Nr of Repeats=4 RepeatLength=21 seed=TTCACGAGTA Num.seqs=4
Similarity=0.583333 0
TTCACGAGTAAATGACCCAATNAN

****
Consensus:

TTCACGAGTAAATGACCCAATNAN

>Bacillus_Fam_1718_24_1 Nr. of seq. 1 Alignment length(with gaps) = 24
Alignment score = 0.000000
GCF_001591445.1_ASM159144v1_genomic.fna_22:102672-102756 Satlength=85 Nr of Repeats=4 RepeatLength=21 seed=ATTCCGCCAA Num.seqs=4
Similarity=0.546296
0 ATTCCGCCAACTATTGGAGTTNGG

****
Consensus:

ATTCCGCCAACTATTGGAGTTNGG

>Bacillus_Fam_1719_24_1 Nr. of seq. 1 Alignment length(with gaps) = 24
Alignment score = 0.000000
GCF_001591585.1_ASM159158v1_genomic.fna_10:33080-33226 Satlength=147 Nr of Repeats=7 RepeatLength=21 seed=ATATCAGCCA Num.seqs=6
Similarity=0.616204
0 ATATCAGCCAANGTAACGGGGAGC

****
Consensus:

ATATCAGCCAANGTAACGGGGAGC

>Bacillus_Fam_1720_24_1 Nr. of seq. 1 Alignment length(with gaps) = 24
Alignment score = 0.000000
GCF_001636315.1_ASM163631v1_genomic.fna_1:5656908-5657010 Satlength=103 Nr of Repeats=5 RepeatLength=24 seed=TTTCAATTTC Num.seqs=3
Similarity=0.925926 0
TTTCAATTTCAATTCGTCTTCTTC

****
Consensus:

TTTCAATTTCAATTCGTCTTCTTC

>Bacillus_Fam_1721_24_1 Nr. of seq. 1 Alignment length(with gaps) = 24
Alignment score = 0.000000

GCF_001645685.2_ASM164568v2_genomic.fna_1:664949-665049 Satlength=101 Nr of Repeats=4 RepeatLength=24 seed=TTGCAGTACG Num.seqs=3 Similarity=0.962963
0   TTGCAGTACGCTAGAGTACTCCAG

****
Consensus:

TTGCAGTACGCTAGAGTACTCCAG

>Bacillus_Fam_1722_24_1 Nr. of seq. 1 Alignment length(with gaps) = 24 Alignment score = 0.000000
GCF_001654695.1_ASM165469v1_genomic.fna_23:346230-346323 Satlength=94 Nr of Repeats=4 RepeatLength=24 seed=GAAAAACAGC Num.seqs=3 Similarity=0.888889
0   GAAAAACAGCCTGCAACAGAAGAA

****
Consensus:

GAAAAACAGCCTGCAACAGAAGAA

>Bacillus_Fam_1723_24_1 Nr. of seq. 1 Alignment length(with gaps) = 24 Alignment score = 0.000000
GCF_001654695.1_ASM165469v1_genomic.fna_34:84196-84424 Satlength=229 Nr of Repeats=6 RepeatLength=21 seed=TGGTTCAATC Num.seqs=4 Similarity=0.527778
0   TGGTTCAATCNCTCGNTCTTCAAA

****
Consensus:

TGGTTCAATCNCTCGNTCTTCAAA

>Bacillus_Fam_1724_24_1 Nr. of seq. 1 Alignment length(with gaps) = 24 Alignment score = 0.000000
GCF_001712755.1_ASM171275v1_genomic.fna_1:111413-111521 Satlength=109 Nr of Repeats=5 RepeatLength=24 seed=GTGCCGTCAC Num.seqs=3 Similarity=0.925926
0   GTGCCGTCACCAGTACCAGTACCC

****
Consensus:

GTGCCGTCACCAGTACCAGTACCC

>Bacillus_Fam_1725_24_1 Nr. of seq. 1 Alignment length(with gaps) = 24 Alignment score = 0.000000
GCF_001742425.1_ASM174242v1_genomic.fna_18:1219-1339 Satlength=121 Nr of Repeats=5 RepeatLength=24 seed=GAAGAACCAA Num.seqs=5 Similarity=0.772222
0   GAAGAACCAACAACAGAACAACCT

****
Consensus:

GAAGAACCAACAACAGAACAACCT

>Bacillus_Fam_1726_24_1 Nr. of seq. 1 Alignment length(with gaps) = 24
Alignment score = 0.000000
GCF_001742425.1_ASM174242v1_genomic.fna_33:4678-4870 Satlength=193 Nr of Repeats=8 RepeatLength=24 seed=AAACCAAGTG Num.seqs=8 Similarity=0.847222
0   AAACCAAGTGAAATAGACGAGACA

****
Consensus:

AAACCAAGTGAAATAGACGAGACA

>Bacillus_Fam_1727_24_1 Nr. of seq. 1 Alignment length(with gaps) = 24
Alignment score = 0.000000
GCF_002019665.1_ASM201966v1_genomic.fna_1:4130416-4130536 Satlength=121 Nr of Repeats=5 RepeatLength=24 seed=TTATTACCTG Num.seqs=5 Similarity=0.855556 0
TTATTACCTGGTTTCTCGCCCGGT

****
Consensus:

TTATTACCTGGTTTCTCGCCCGGT

>Bacillus_Fam_1728_24_1 Nr. of seq. 1 Alignment length(with gaps) = 24
Alignment score = 0.000000
GCF_900109925.1_IMG-taxon_2651870116_annotated_assembly_genomic.fna_23:96772-97084 Satlength=313 Nr of Repeats=13 RepeatLength=24 seed=CGACCATCAG Num.seqs=13 Similarity=0.881054 0
CGACCATCAGGTCAAGGCCAACAA

****
Consensus:

CGACCATCAGGTCAAGGCCAACAA

>Bacillus_Fam_1729_23_1 Nr. of seq. 1 Alignment length(with gaps) = 23
Alignment score = 0.000000
GCF_000171615.1_ASM17161v1_genomic.fna_93:12659-12804 Satlength=146 Nr of Repeats=5 RepeatLength=21 seed=GTGCACGCAA Num.seqs=4 Similarity=0.719807
0   GTGCACGCAAGAAGNCCGAAAAA

****
Consensus:

GTGCACGCAAGAAGNCCGAAAAA

>Bacillus_Fam_1730_23_1 Nr. of seq. 1 Alignment length(with gaps) = 23
Alignment score = 0.000000
GCF_000186145.1_Bacillus_sp_2_A_57_CT2_V1_genomic.fna_6:213928-214012 Satlength=85 Nr of Repeats=4 RepeatLength=21 seed=TTGGCCGGTA Num.seqs=4

Similarity=0.396368 0
TTGGCCGGTAATTTNCATTNAAA

****
Consensus:

TTGGCCGGTAATTTNCATTNAAA

>Bacillus_Fam_1731_23_1 Nr. of seq. 1 Alignment length(with gaps) = 23
Alignment score = 0.000000
GCF_000305495.1_BacPsy2.0_genomic.fna_90:7041-7359 Satlength=319 Nr of Repeats=7 RepeatLength=21 seed=ATTCGGCCCT Num.seqs=6 Similarity=0.508213
0 ATTCGGCCCTTANTCTCCATCNN

****
Consensus:

ATTCGGCCCTTANTCTCCATCNN

>Bacillus_Fam_1732_23_1 Nr. of seq. 1 Alignment length(with gaps) = 23
Alignment score = 0.000000
GCF_000331575.1_ASM33157v1_genomic.fna_4:173707-173821 Satlength=115 Nr of Repeats=5 RepeatLength=23 seed=TACATGTAAA Num.seqs=4
Similarity=0.876157
0 TACATGTAAAATCATATTTTCGA

****
Consensus:

TACATGTAAAATCATATTTTCGA

>Bacillus_Fam_1733_23_1 Nr. of seq. 1 Alignment length(with gaps) = 23
Alignment score = 0.000000
GCF_000709935.2_ASM70993v2_genomic.fna_5:604719-605171 Satlength=453 Nr of Repeats=6 RepeatLength=21 seed=TACATCCAAC Num.seqs=4
Similarity=0.555556
0 TACATCCAACAATTAGAGCAATT

****
Consensus:

TACATCCAACAATTAGAGCAATT

>Bacillus_Fam_1734_23_1 Nr. of seq. 1 Alignment length(with gaps) = 23
Alignment score = 0.000000
GCF_001591425.1_ASM159142v1_genomic.fna_90:13367-13451 Satlength=85 Nr of Repeats=4 RepeatLength=21 seed=GGTGATAAAT Num.seqs=4 Similarity=0.572464
0 GGTGATAAATAAATACCACTCTT

****
Consensus:

GGTGATAAATAAATACCACTCTT

>Bacillus_Fam_1735_23_1 Nr. of seq. 1 Alignment length(with gaps) = 23
Alignment score = 0.000000
GCF_001591425.1_ASM159142v1_genomic.fna_135:8975-9359 Satlength=385 Nr of Repeats=11 RepeatLength=21 seed=GTTGAACAGA Num.seqs=8 Similarity=0.610766
0   GTTGAACAGAANNATCAAGTTGA

****
Consensus:

GTTGAACAGAANNATCAAGTTGA

>Bacillus_Fam_1736_23_1 Nr. of seq. 1 Alignment length(with gaps) = 23
Alignment score = 0.000000
GCF_001591825.1_ASM159182v1_genomic.fna_26:22973-23057 Satlength=85 Nr of Repeats=4 RepeatLength=21 seed=AATCCAGCCG Num.seqs=4 Similarity=0.507246
0   AATCCAGCCGANAATACGAAANG

****
Consensus:

AATCCAGCCGANAATACGAAANG

>Bacillus_Fam_1737_23_1 Nr. of seq. 1 Alignment length(with gaps) = 23
Alignment score = 0.000000
GCF_001636315.1_ASM163631v1_genomic.fna_1:1929121-1929205 Satlength=85 Nr of Repeats=4 RepeatLength=21 seed=TTCGCCGATA Num.seqs=4 Similarity=0.518519 0
TTCGCCGATAACTNGCTNCATGN

****
Consensus:

TTCGCCGATAACTNGCTNCATGN

>Bacillus_Fam_1738_23_1 Nr. of seq. 1 Alignment length(with gaps) = 23
Alignment score = 0.000000
GCF_001645685.2_ASM164568v2_genomic.fna_1:4339164-4339477 Satlength=314 Nr of Repeats=15 RepeatLength=21 seed=TTTTCCGGCC Num.seqs=10 Similarity=0.686634 0
TTTTCCGGCCGGTCGACTCGACT

****
Consensus:

TTTTCCGGCCGGTCGACTCGACT

>Bacillus_Fam_1739_23_1 Nr. of seq. 1 Alignment length(with gaps) = 23
Alignment score = 0.000000
GCF_001712755.1_ASM171275v1_genomic.fna_37:34288-34763 Satlength=476 Nr of Repeats=16 RepeatLength=20 seed=AATCGCAATT Num.seqs=12 Similarity=0.595749 0
AATCGCAAATAAAATCNAAGNNN

****
Consensus:

AATCGCAAATAAAATCNAAGNNN

>Bacillus_Fam_1740_23_1 Nr. of seq. 1 Alignment length(with gaps) = 23
Alignment score = 0.000000
GCF_900111815.1_PRJEB17078_genomic.fna_1:496274-496585 Satlength=312 Nr of Repeats=11 RepeatLength=21 seed=AAATCAACCA Num.seqs=8 Similarity=0.576605
0 AAATCAACCAACTNATGAGTGAG

****
Consensus:

AAATCAACCAACTNATGAGTGAG

>Bacillus_Fam_1741_22_1 Nr. of seq. 1 Alignment length(with gaps) = 22
Alignment score = 0.000000
GCF_000175075.1_ASM17507v1_genomic.fna_31:195679-195783 Satlength=105 Nr of Repeats=4 RepeatLength=21 seed=TATTTTCCGT Num.seqs=3 Similarity=0.521739
0 TATTTTCCGTTACNGNTATATT

****
Consensus:

TATTTTCCGTTACNGNTATATT

>Bacillus_Fam_1742_22_1 Nr. of seq. 1 Alignment length(with gaps) = 22
Alignment score = 0.000000
GCF_000175075.1_ASM17507v1_genomic.fna_32:57067-57168 Satlength=102 Nr of Repeats=5 RepeatLength=20 seed=TTTCTTGATA Num.seqs=4 Similarity=0.589646
0 TTTCTTGATATATCTTGCAANA

****
Consensus:

TTTCTTGATATATCTTGCAANA

>Bacillus_Fam_1743_22_1 Nr. of seq. 1 Alignment length(with gaps) = 22
Alignment score = 0.000000
GCF_000242895.2_ASM24289v3_genomic.fna_1:4424659-4424743 Satlength=85 Nr of Repeats=4 RepeatLength=21 seed=TTTATCGGCC Num.seqs=4 Similarity=0.641304
0 TTTATCGGCCGCTTTTGATAAT

****
Consensus:

TTTATCGGCCGCTTTTGATAAT

>Bacillus_Fam_1744_22_1 Nr. of seq. 1 Alignment length(with gaps) = 22
Alignment score = 0.000000
GCF_000307875.1_BABA1.0_genomic.fna_135:3379-3463 Satlength=85 Nr of Repeats=4 RepeatLength=21 seed=TTTATTTGCG Num.seqs=4 Similarity=0.525463
19 ATTTGCGAAACTAGCCACATTT

****
Consensus:

TTTATTTGCGAAACTAGCCACA

>Bacillus_Fam_1745_22_1 Nr. of seq. 1 Alignment length(with gaps) = 22
Alignment score = 0.000000
GCF_000311725.1_ASM31172v1_genomic.fna_2:350998-351082 Satlength=85 Nr of Repeats=4 RepeatLength=21 seed=ATTGCGGGCG Num.seqs=4 Similarity=0.601010
0 ATTGCGGGCGTTCTANGCCATA

****
Consensus:

ATTGCGGGCGTTCTANGCCATA

>Bacillus_Fam_1746_22_1 Nr. of seq. 1 Alignment length(with gaps) = 22
Alignment score = 0.000000
GCF_000331575.1_ASM33157v1_genomic.fna_4:23784-24242 Satlength=459 Nr of Repeats=13 RepeatLength=22 seed=GATACCTTAT Num.seqs=8
Similarity=0.902597
0 GATACCTTATGAGTACGGAAAT

****
Consensus:

GATACCTTATGAGTACGGAAAT

>Bacillus_Fam_1747_22_1 Nr. of seq. 1 Alignment length(with gaps) = 22
Alignment score = 0.000000
GCF_000429685.1_ASM42968v1_genomic.fna_13:95209-95355 Satlength=147 Nr of Repeats=6 RepeatLength=21 seed=GAGGAGAAAA Num.seqs=4 Similarity=0.606061
0 GAGGAGAAGAAANAGCGGAGCN

****
Consensus:

GAGGAGAAGAAANAGCGGAGCN

>Bacillus_Fam_1748_22_1 Nr. of seq. 1 Alignment length(with gaps) = 22
Alignment score = 0.000000
GCF_000429705.1_ASM42970v1_genomic.fna_3:352016-352120 Satlength=105 Nr of Repeats=5 RepeatLength=21 seed=TATAATGAAT Num.seqs=4
Similarity=0.637626
0 TATAATGAATTCCTCAACNATA

****
Consensus:

TATAATGAATTCCTCAACNATA

>Bacillus_Fam_1749_22_1 Nr. of seq. 1 Alignment length(with gaps) = 22
Alignment score = 0.000000
GCF_000986785.1_ASM98678v1_genomic.fna_5:35642-35726 Satlength=85 Nr of Repeats=4 RepeatLength=21 seed=TAATCCTGAT Num.seqs=4 Similarity=0.543981
0   TAATCCTGATTTCCGCTGCATT

****
Consensus:

TAATCCTGATTTCCGCTGCATT

>Bacillus_Fam_1750_22_1 Nr. of seq. 1 Alignment length(with gaps) = 22
Alignment score = 0.000000
GCF_000986785.1_ASM98678v1_genomic.fna_5:81208-81561 Satlength=354 Nr of Repeats=5 RepeatLength=21 seed=CAATAATTGA Num.seqs=4 Similarity=0.757576
0   CAATAATTGATGCAATCAACTT

****
Consensus:

CAATAATTGATGCAATCAACTT

>Bacillus_Fam_1751_22_1 Nr. of seq. 1 Alignment length(with gaps) = 22
Alignment score = 0.000000
GCF_001591425.1_ASM159142v1_genomic.fna_1:26204-26330 Satlength=127 Nr of Repeats=6 RepeatLength=21 seed=TTTTCTTAAC Num.seqs=6 Similarity=0.546377
0   TTTACTTAACAGAGCTTCGGAA

****
Consensus:

TTTACTTAACAGAGCTTCGGAA

>Bacillus_Fam_1752_22_1 Nr. of seq. 1 Alignment length(with gaps) = 22
Alignment score = 0.000000
GCF_001591425.1_ASM159142v1_genomic.fna_10:28205-28351 Satlength=147 Nr of Repeats=6 RepeatLength=21 seed=TGGTGCAACC Num.seqs=4
Similarity=0.513285
0   TGGTGCAACACCACCGCAAAAN

****
Consensus:

TGGTGCAACACCACCGCAAAAN

>Bacillus_Fam_1753_22_1 Nr. of seq. 1 Alignment length(with gaps) = 22
Alignment score = 0.000000
GCF_001591445.1_ASM159144v1_genomic.fna_16:43521-43605 Satlength=85 Nr of Repeats=4 RepeatLength=21 seed=TCTATTGGCG Num.seqs=4 Similarity=0.717172
0   TCTATTGGCGAATTTCACCTAN

****
Consensus:

TCTATTGGCGAATTTCACCTAN

>Bacillus_Fam_1754_22_1 Nr. of seq. 1 Alignment length(with gaps) = 22
Alignment score = 0.000000
GCF_001636345.1_ASM163634v1_genomic.fna_1:454483-454588 Satlength=106 Nr
of Repeats=5 RepeatLength=21 seed=TTTGATAGGA Num.seqs=5
Similarity=0.542029
0   TTTGATAGGAAAGCTGNTTANN

****
Consensus:

TTTGATAGGAAAGCTGNTTANN

>Bacillus_Fam_1755_22_1 Nr. of seq. 1 Alignment length(with gaps) = 22
Alignment score = 0.000000
GCF_001636425.1_ASM163642v1_genomic.fna_1:4479552-4479636 Satlength=85 Nr
of Repeats=4 RepeatLength=21 seed=TTTACCCGAT Num.seqs=4
Similarity=0.631313 0
TTTACCCGATTANACGCATANT

****
Consensus:

TTTACCCGATTANACGCATANT

>Bacillus_Fam_1756_22_1 Nr. of seq. 1 Alignment length(with gaps) = 22
Alignment score = 0.000000
GCF_001712755.1_ASM171275v1_genomic.fna_37:29313-29417 Satlength=105 Nr
of Repeats=5 RepeatLength=21 seed=TTTAATTGCA Num.seqs=4
Similarity=0.585859
0   TTTAATTGCAGATATAGCATAN

****
Consensus:

TTTAATTGCAGATATAGCATAN

>Bacillus_Fam_1757_22_1 Nr. of seq. 1 Alignment length(with gaps) = 22
Alignment score = 0.000000
GCF_002019665.1_ASM201966v1_genomic.fna_1:3363210-3363336 Satlength=127
Nr of Repeats=6 RepeatLength=21 seed=ATTCGTCCAG Num.seqs=6
Similarity=0.657071 0
ATTCGTCCGAAATCCGCCTTCN

****
Consensus:

ATTCGTCCGAAATCCGCCTTCN

>Bacillus_Fam_1758_22_1 Nr. of seq. 1 Alignment length(with gaps) = 22
Alignment score = 0.000000
GCF_900104555.1_IMG-taxon_2651870172_annotated_assembly_genomic.fna_19:64922-65582
Satlength=661 Nr of Repeats=20 RepeatLength=21 seed=TCGCGGGATT
Num.seqs=14 Similarity=0.665668 0
TCGCGGGATTNCCATGCTCTTT

****
Consensus:

TCGCGGGATTNCCATGCTCTTT

>Bacillus_Fam_1759_21_1 Nr. of seq. 1 Alignment length(with gaps) = 21
Alignment score = 0.000000
GCF_000153365.1_ASM15336v1_genomic.fna_17:503213-503318 Satlength=106 Nr of Repeats=4 RepeatLength=21 seed=TCAAACCTTT Num.seqs=3
Similarity=0.788360
0   TCAAACCTTTTATCAACAGCA

****
Consensus:

TCAAACCTTTTATCAACAGCA

>Bacillus_Fam_1760_21_1 Nr. of seq. 1 Alignment length(with gaps) = 21
Alignment score = 0.000000
GCF_000161455.1_ASM16145v1_genomic.fna_1:1878049-1878322 Satlength=274 Nr of Repeats=12 RepeatLength=21 seed=GGTGGCACAA Num.seqs=11
Similarity=0.866089 0
GGTGGCACAACAAACCCAGGC

****
Consensus:

GGTGGCACAACAAACCCAGGC

>Bacillus_Fam_1761_21_1 Nr. of seq. 1 Alignment length(with gaps) = 21
Alignment score = 0.000000
GCF_000171615.1_ASM17161v1_genomic.fna_106:18545-18854 Satlength=310 Nr of Repeats=10 RepeatLength=21 seed=CCGCACGAGA Num.seqs=7
Similarity=0.617605
0   CCGCACGAGAAGAAGGTCCNC

****
Consensus:

CCGCACGAGAAGAAGGTCCNC

>Bacillus_Fam_1762_21_1 Nr. of seq. 1 Alignment length(with gaps) = 21
Alignment score = 0.000000
GCF_000245315.1_ASM24531v1_genomic.fna_45:163118-163205 Satlength=88 Nr of Repeats=4 RepeatLength=21 seed=GCCGGGCACA Num.seqs=3

Similarity=1.000000
0   GCCGGGCACACCGCCGCCGAA

****
Consensus:

GCCGGGCACACCGCCGCCGAA

>Bacillus_Fam_1763_21_1 Nr. of seq. 1 Alignment length(with gaps) = 21
Alignment score = 0.000000
GCF_000290995.1_Baci_cere_AND1407_G13175_V1_genomic.fna_1:1730757-1730841
Satlength=85 Nr of Repeats=4 RepeatLength=21 seed=TCTTTTTTTG Num.seqs=4
Similarity=0.957672   0
TCTTTTTTTGGCTCACAATCC

****
Consensus:

TCTTTTTTTGGCTCACAATCC

>Bacillus_Fam_1764_21_1 Nr. of seq. 1 Alignment length(with gaps) = 21
Alignment score = 0.000000
GCF_000331575.1_ASM33157v1_genomic.fna_1:475624-475876 Satlength=253 Nr
of Repeats=12 RepeatLength=21 seed=TTACCCATAT Num.seqs=12
Similarity=0.735308   0
TTACCCATATATCTGNGCACT

****
Consensus:

TTACCCATATATCTGNGCACT

>Bacillus_Fam_1765_21_1 Nr. of seq. 1 Alignment length(with gaps) = 21
Alignment score = 0.000000
GCF_000331575.1_ASM33157v1_genomic.fna_2:594953-595079 Satlength=127 Nr
of Repeats=5 RepeatLength=21 seed=TTCCTTCCGT Num.seqs=4
Similarity=0.862434
0   TTCCTTCCGTGGTAACGTGGA

****
Consensus:

TTCCTTCCGTGGTAACGTGGA

>Bacillus_Fam_1766_21_1 Nr. of seq. 1 Alignment length(with gaps) = 21
Alignment score = 0.000000
GCF_000331575.1_ASM33157v1_genomic.fna_4:107598-107787 Satlength=190 Nr
of Repeats=7 RepeatLength=21 seed=AATCGAGTGC Num.seqs=5
Similarity=0.911111
0   AATCGAGTGCTTTTCTCGCGG

****
Consensus:

AATCGAGTGCTTTTCTCGCGG

>Bacillus_Fam_1767_21_1 Nr. of seq. 1 Alignment length(with gaps) = 21
Alignment score = 0.000000
GCF_000331575.1_ASM33157v1_genomic.fna_4:141893-142040 Satlength=148 Nr of Repeats=7 RepeatLength=21 seed=TTTACCCATA Num.seqs=7
Similarity=0.687590
0   TTTACCCATATGAATTAGCTA

****
Consensus:

TTTACCCATATGAATTAGCTA

>Bacillus_Fam_1768_21_1 Nr. of seq. 1 Alignment length(with gaps) = 21
Alignment score = 0.000000
GCF_000331575.1_ASM33157v1_genomic.fna_4:228625-228873 Satlength=249 Nr of Repeats=10 RepeatLength=21 seed=TACCCACATA Num.seqs=9
Similarity=0.669082
0   TACCCACATAAAATGCGGATT

****
Consensus:

TACCCACATAAAATGCGGATT

>Bacillus_Fam_1769_21_1 Nr. of seq. 1 Alignment length(with gaps) = 21
Alignment score = 0.000000
GCF_000334155.1_L1_genomic.fna_69:26716-27262 Satlength=547 Nr of Repeats=26 RepeatLength=21 seed=CATGAAGAAG Num.seqs=26
Similarity=0.867936
0   CATGAAGAAGAAGAACATGGA

****
Consensus:

CATGAAGAAGAAGAACATGGA

>Bacillus_Fam_1770_21_1 Nr. of seq. 1 Alignment length(with gaps) = 21
Alignment score = 0.000000
GCF_000401235.1_BacNeaAAU1_genomic.fna_109:50519-50812 Satlength=294 Nr of Repeats=14 RepeatLength=21 seed=TTGGCTGATA Num.seqs=13
Similarity=0.808081 0
TTGGCTGATATCGGTGTTGGA

****
Consensus:

TTGGCTGATATCGGTGTTGGA

>Bacillus_Fam_1771_21_1 Nr. of seq. 1 Alignment length(with gaps) = 21
Alignment score = 0.000000

GCF_000429705.1_ASM42970v1_genomic.fna_1:164813-164917 Satlength=105 Nr of Repeats=5 RepeatLength=21 seed=TATAATGAAT Num.seqs=4 Similarity=0.498843
0   TATAATGAATCTCGAANCATT

****
Consensus:

TATAATGAATCTCGAANCATT

>Bacillus_Fam_1772_21_1 Nr. of seq. 1 Alignment length(with gaps) = 21 Alignment score = 0.000000
GCF_000429705.1_ASM42970v1_genomic.fna_1:212906-213009 Satlength=104 Nr of Repeats=5 RepeatLength=21 seed=AAAGTGATGG Num.seqs=3 Similarity=0.682540
0   AAAGTGATTGATAANNGGATN

****
Consensus:

AAAGTGATTGATAANNGGATN

>Bacillus_Fam_1773_21_1 Nr. of seq. 1 Alignment length(with gaps) = 21 Alignment score = 0.000000
GCF_000429705.1_ASM42970v1_genomic.fna_1:1695051-1695135 Satlength=85 Nr of Repeats=4 RepeatLength=21 seed=TTAAACAGGT Num.seqs=4 Similarity=0.693122
0   TTAAACAGGTTAGGCTCTCAT

****
Consensus:

TTAAACAGGTTAGGCTCTCAT

>Bacillus_Fam_1774_21_1 Nr. of seq. 1 Alignment length(with gaps) = 21 Alignment score = 0.000000
GCF_000429705.1_ASM42970v1_genomic.fna_2:604162-604246 Satlength=85 Nr of Repeats=4 RepeatLength=21 seed=TAATTCTACC Num.seqs=4 Similarity=0.520833
0   TAATTCTACCTAAAAAGNCAT

****
Consensus:

TAATTCTACCTAAAAAGNCAT

>Bacillus_Fam_1775_21_1 Nr. of seq. 1 Alignment length(with gaps) = 21 Alignment score = 0.000000
GCF_000430765.1_ASM43076v1_genomic.fna_7:272233-272397 Satlength=165 Nr of Repeats=5 RepeatLength=21 seed=GAGCAAAAAA Num.seqs=3 Similarity=0.416667
0   GAGCAAAAAAGNACCNTGCNG

****
Consensus:

GAGCAAAAAAGNACCNTGCNG

>Bacillus_Fam_1776_21_1 Nr. of seq. 1 Alignment length(with gaps) = 21 Alignment score = 0.000000 GCF_000430765.1_ASM43076v1_genomic.fna_14:8047-8192 Satlength=146 Nr of Repeats=7 RepeatLength=21 seed=TTTTTGCTCA Num.seqs=5 Similarity=0.525333
0   TTTTTGCTCANCACACCNATN

****
Consensus:

TTTTTGCTCANCACACCNATN

>Bacillus_Fam_1777_21_1 Nr. of seq. 1 Alignment length(with gaps) = 21 Alignment score = 0.000000 GCF_000430785.1_ASM43078v1_genomic.fna_3:131543-131690 Satlength=148 Nr of Repeats=7 RepeatLength=21 seed=TGCCACAAGG Num.seqs=7 Similarity=0.945578
0   TGCCACAAGGAATGCATCAAG

****
Consensus:

TGCCACAAGGAATGCATCAAG

>Bacillus_Fam_1778_21_1 Nr. of seq. 1 Alignment length(with gaps) = 21 Alignment score = 0.000000 GCF_000474275.1_Bmar1.0_genomic.fna_20:6145-6290 Satlength=146 Nr of Repeats=4 RepeatLength=21 seed=ACTGACGCAA Num.seqs=3 Similarity=1.000000
0   ACTGACGCAATAGCGTCTGGA

****
Consensus:

ACTGACGCAATAGCGTCTGGA

>Bacillus_Fam_1779_21_1 Nr. of seq. 1 Alignment length(with gaps) = 21 Alignment score = 0.000000 GCF_000496285.1_ASM49628v1_genomic.fna_1:2486611-2486716 Satlength=106 Nr of Repeats=5 RepeatLength=21 seed=TTGGATCTTT Num.seqs=5 Similarity=0.898413 0
TTGGATCTTTCGGATCTGTGC

****
Consensus:

TTGGATCTTTCGGATCTGTGC

>Bacillus_Fam_1780_21_1 Nr. of seq. 1 Alignment length(with gaps) = 21 Alignment score = 0.000000 GCF_000504145.1_SOAPdenovo_for_version2.04_of_Bacillus_sp._FJAT-14515_genome_genomic.fna_1:1809470-1809638 Satlength=169 Nr of Repeats=8

RepeatLength=21 seed=CTTCTTGGGA Num.seqs=8 Similarity=0.895692 0
CTTCTTGGGACCGTCATCCGG

****
Consensus:

CTTCTTGGGACCGTCATCCGG

>Bacillus_Fam_1781_21_1 Nr. of seq. 1 Alignment length(with gaps) = 21
Alignment score = 0.000000
GCF_000508325.1_BAVI_1_genomic.fna_92:129782-130070 Satlength=289 Nr of Repeats=4 RepeatLength=21 seed=AAGTGACCGG Num.seqs=3 Similarity=0.628019 0 AAGTGACCGGTATTTNNACNA

****
Consensus:

AAGTGACCGGTATTTNNACNA

>Bacillus_Fam_1782_21_1 Nr. of seq. 1 Alignment length(with gaps) = 21
Alignment score = 0.000000
GCF_000526655.1_ASM52665v1_genomic.fna_1:7-112 Satlength=106 Nr of Repeats=5 RepeatLength=21 seed=GAACTGCGAC Num.seqs=5 Similarity=0.847619 0 GAACTGCGACGGTTGCGAGAA

****
Consensus:

GAACTGCGACGGTTGCGAGAA

>Bacillus_Fam_1783_21_1 Nr. of seq. 1 Alignment length(with gaps) = 21
Alignment score = 0.000000
GCF_000612625.1_JCE_genomic.fna_8:31125-31331 Satlength=207 Nr of Repeats=5 RepeatLength=21 seed=GGATCATTTT Num.seqs=3 Similarity=0.509259 0 GGATCATTTTCCAATATCNTT

****
Consensus:

GGATCATTTTCCAATATCNTT

>Bacillus_Fam_1784_21_1 Nr. of seq. 1 Alignment length(with gaps) = 21
Alignment score = 0.000000
GCF_000612665.1_FF4_genomic.fna_12:2775-2932 Satlength=158 Nr of Repeats=4 RepeatLength=21 seed=AATTTGTAGA Num.seqs=3 Similarity=0.788360 0 AATTTGTAGATATCTAGACCA

****
Consensus:

AATTTGTAGATATCTAGACCA

>Bacillus_Fam_1785_21_1 Nr. of seq. 1 Alignment length(with gaps) = 21
Alignment score = 0.000000

GCF_000612665.1_FF4_genomic.fna_25:19828-19954 Satlength=127 Nr of Repeats=6 RepeatLength=21 seed=CAGCCAGCAC Num.seqs=6 Similarity=0.957672
0   CAGCCAGCACCGCAGCAGGCA

****
Consensus:

CAGCCAGCACCGCAGCAGGCA

>Bacillus_Fam_1786_21_1 Nr. of seq. 1 Alignment length(with gaps) = 21 Alignment score = 0.000000
GCF_000612805.1_FF3_genomic.fna_6:195760-195886 Satlength=127 Nr of Repeats=5 RepeatLength=21 seed=CCTGGGAAAA Num.seqs=4 Similarity=0.798942
0   CCTGGGAAAAACGGACCCACA

****
Consensus:

CCTGGGAAAAACGGACCCACA

>Bacillus_Fam_1787_21_1 Nr. of seq. 1 Alignment length(with gaps) = 21 Alignment score = 0.000000
GCF_000709935.2_ASM70993v2_genomic.fna_5:763265-763757 Satlength=493 Nr of Repeats=5 RepeatLength=21 seed=TTTGGATTGT Num.seqs=3 Similarity=1.000000
0   TTTGGATTGTTACACGCCGCA

****
Consensus:

TTTGGATTGTTACACGCCGCA

>Bacillus_Fam_1788_21_1 Nr. of seq. 1 Alignment length(with gaps) = 21 Alignment score = 0.000000
GCF_000724485.1_ASM72448v1_genomic.fna_1:2060503-2060703 Satlength=201 Nr of Repeats=4 RepeatLength=21 seed=AACGGGAGTA Num.seqs=3 Similarity=0.681818 0
AACGGGAGTAAANTCCTCCTG

****
Consensus:

AACGGGAGTAAANTCCTCCTG

>Bacillus_Fam_1789_21_1 Nr. of seq. 1 Alignment length(with gaps) = 21 Alignment score = 0.000000
GCF_000751775.1_A1A_genomic.fna_84:23149-23297 Satlength=149 Nr of Repeats=6 RepeatLength=21 seed=ATTCAACAGT Num.seqs=4 Similarity=0.502645
0   ATTCAACAGTAGATTACGAGT

****
Consensus:

ATTCAACAGTAGATTACGAGT

>Bacillus_Fam_1790_21_1 Nr. of seq. 1 Alignment length(with gaps) = 21
Alignment score = 0.000000
GCF_000751775.1_A1A_genomic.fna_102:29051-29177 Satlength=127 Nr of Repeats=5 RepeatLength=21 seed=ATGGACAAGC Num.seqs=4 Similarity=0.936508
0   ATGGACAAGCTTGAATCCCGA

****
Consensus:

ATGGACAAGCTTGAATCCCGA

>Bacillus_Fam_1791_21_1 Nr. of seq. 1 Alignment length(with gaps) = 21
Alignment score = 0.000000
GCF_000752035.1_Bacillus_rubiinfantis_genomic.fna_11:391259-391490 Satlength=232 Nr of Repeats=11 RepeatLength=21 seed=TACGAGAGTA Num.seqs=11 Similarity=0.611331 0
TACGAGAGTAAACCTGNGTTA

****
Consensus:

TACGAGAGTAAACCTGNGTTA

>Bacillus_Fam_1792_21_1 Nr. of seq. 1 Alignment length(with gaps) = 21
Alignment score = 0.000000
GCF_000752035.1_Bacillus_rubiinfantis_genomic.fna_11:573150-573648 Satlength=499 Nr of Repeats=18 RepeatLength=21 seed=TCTACAAATT Num.seqs=12 Similarity=0.615200 0
TCTACAAATTCAGGNCCGTAT

****
Consensus:

TCTACAAATTCAGGNCCGTAT

>Bacillus_Fam_1793_21_1 Nr. of seq. 1 Alignment length(with gaps) = 21
Alignment score = 0.000000
GCF_000752035.1_Bacillus_rubiinfantis_genomic.fna_23:26534-26639 Satlength=106 Nr of Repeats=4 RepeatLength=21 seed=TGTTTCTACA Num.seqs=3 Similarity=0.957672 0
TGTTTCTACAAATTAAAGATG

****
Consensus:

TGTTTCTACAAATTAAAGATG

>Bacillus_Fam_1794_21_1 Nr. of seq. 1 Alignment length(with gaps) = 21
Alignment score = 0.000000
GCF_000752035.1_Bacillus_rubiinfantis_genomic.fna_30:64121-64390 Satlength=270 Nr of Repeats=11 RepeatLength=21 seed=TTTTGTAGAT Num.seqs=9 Similarity=0.602623
0   TTTTGTAGANTNAGCACTTAA

****
Consensus:

TTTTGTAGANTNAGCACTTAA

>Bacillus_Fam_1795_21_1 Nr. of seq. 1 Alignment length(with gaps) = 21
Alignment score = 0.000000
GCF_000752395.1_Bacillus_andreraoultii_genomic.fna_12:197233-197359
Satlength=127 Nr of Repeats=6 RepeatLength=21 seed=TACTGTTGAA Num.seqs=6
Similarity=0.624074 0
TACTGTTGAAGTTTCATCTTT

****
Consensus:

TACTGTTGAAGTTTCATCTTT

>Bacillus_Fam_1796_21_1 Nr. of seq. 1 Alignment length(with gaps) = 21
Alignment score = 0.000000
GCF_000752395.1_Bacillus_andreraoultii_genomic.fna_45:59415-59652
Satlength=238 Nr of Repeats=9 RepeatLength=20 seed=AACTTCGGAA Num.seqs=6
Similarity=0.503535 0
AACTTCGGAAATNATAGCNTC

****
Consensus:

AACTTCGGAAATNATAGCNTC

>Bacillus_Fam_1797_21_1 Nr. of seq. 1 Alignment length(with gaps) = 21
Alignment score = 0.000000
GCF_000759675.1_ASM75967v1_genomic.fna_150:11338-11443 Satlength=106 Nr of Repeats=5 RepeatLength=21 seed=AGTCCAAAAC Num.seqs=3
Similarity=0.682540
0   AGTCCAAAACCNCATGGAAAT

****
Consensus:

AGTCCAAAACCNCATGGAAAT

>Bacillus_Fam_1798_21_1 Nr. of seq. 1 Alignment length(with gaps) = 21
Alignment score = 0.000000
GCF_000759675.1_ASM75967v1_genomic.fna_158:119179-119305 Satlength=127 Nr of Repeats=5 RepeatLength=21 seed=CTGTTCTACA Num.seqs=4
Similarity=0.777778 0
CTGTTCTACAGCTTCGACAAG

****
Consensus:

CTGTTCTACAGCTTCGACAAG

>Bacillus_Fam_1799_21_1 Nr. of seq. 1 Alignment length(with gaps) = 21
Alignment score = 0.000000
GCF_000812025.2_ASM81202v2_genomic.fna_59:9522-9690 Satlength=169 Nr of Repeats=8 RepeatLength=21 seed=AAGGAACAGC Num.seqs=6 Similarity=0.671212
0   AAGGAACAGCCGAAACAAGTA

****
Consensus:

AAGGAACAGCCGAAACAAGTA

>Bacillus_Fam_1800_21_1 Nr. of seq. 1 Alignment length(with gaps) = 21
Alignment score = 0.000000
GCF_000813125.1_ASM81312v1_genomic.fna_110:11398-11482 Satlength=85 Nr of Repeats=4 RepeatLength=21 seed=AGAGATTTAT Num.seqs=4 Similarity=0.875000
0   AGAGATTTATCTCCAGTTAAG

****
Consensus:

AGAGATTTATCTCCAGTTAAG

>Bacillus_Fam_1801_21_1 Nr. of seq. 1 Alignment length(with gaps) = 21
Alignment score = 0.000000
GCF_000813125.1_ASM81312v1_genomic.fna_129:71964-72048 Satlength=85 Nr of Repeats=4 RepeatLength=21 seed=ATTCCGCGAA Num.seqs=4 Similarity=0.587302
0   ATTCCGCGAAAAAAATTCTTTA

****
Consensus:

ATTCCGCGAAAAAAATTCTTTA

>Bacillus_Fam_1802_21_1 Nr. of seq. 1 Alignment length(with gaps) = 21
Alignment score = 0.000000
GCF_000813125.1_ASM81312v1_genomic.fna_142:7599-7683 Satlength=85 Nr of Repeats=4 RepeatLength=21 seed=TTTTTCTACG Num.seqs=4 Similarity=0.586957
0   TTTTTCTACGGTTGGCAGCAN

****
Consensus:

TTTTTCTACGGTTGGCAGCAN

>Bacillus_Fam_1803_21_1 Nr. of seq. 1 Alignment length(with gaps) = 21
Alignment score = 0.000000
GCF_000831065.1_ASM83106v1_genomic.fna_1:1513435-1513582 Satlength=148 Nr of Repeats=7 RepeatLength=21 seed=AAAACCAGAA Num.seqs=7 Similarity=0.897203 0
AAAACCAGAAGGCGAAGGTGG

****
Consensus:

AAAACCAGAAGGCGAAGGTGG

>Bacillus_Fam_1804_21_1 Nr. of seq. 1 Alignment length(with gaps) = 21 Alignment score = 0.000000
GCF_000832605.1_ASM83260v1_genomic.fna_1:783460-783565 Satlength=106 Nr of Repeats=5 RepeatLength=21 seed=AGACTAAGCT Num.seqs=5 Similarity=1.000000
0 AGACTAAGCTGATACTTAATG

****
Consensus:

AGACTAAGCTGATACTTAATG

>Bacillus_Fam_1805_21_1 Nr. of seq. 1 Alignment length(with gaps) = 21 Alignment score = 0.000000
GCF_000948175.1_BFA_2_A_genomic.fna_39:29576-29984 Satlength=409 Nr of Repeats=4 RepeatLength=21 seed=GGTTGGATCG Num.seqs=3 Similarity=0.915344
0 GGTTGGATCGGGTTGAAGAGC

****
Consensus:

GGTTGGATCGGGTTGAAGAGC

>Bacillus_Fam_1806_21_1 Nr. of seq. 1 Alignment length(with gaps) = 21 Alignment score = 0.000000
GCF_000986785.1_ASM98678v1_genomic.fna_38:8628-8733 Satlength=106 Nr of Repeats=5 RepeatLength=21 seed=TTGGAAAATA Num.seqs=5 Similarity=0.476812
0 TTGGAAAATAAACTTNCTNTN

****
Consensus:

TTGGAAAATAAACTTNCTNTN

>Bacillus_Fam_1807_21_1 Nr. of seq. 1 Alignment length(with gaps) = 21 Alignment score = 0.000000
GCF_001273755.1_ASM127375v1_genomic.fna_17:3548301-3548609 Satlength=309 Nr of Repeats=13 RepeatLength=21 seed=GACGAATTAT Num.seqs=8 Similarity=0.615823 0
GACGAATTATCTTCTCTCAAG

****
Consensus:

GACGAATTATCTTCTCTCAAG

>Bacillus_Fam_1808_21_1 Nr. of seq. 1 Alignment length(with gaps) = 21 Alignment score = 0.000000
GCF_001278705.1_ASM127870v1_genomic.fna_1:21006-21130 Satlength=125 Nr of Repeats=6 RepeatLength=21 seed=GGCACATAAA Num.seqs=4 Similarity=0.586957
0 GGCACATAAACTACGTCGAGT

****
Consensus:

GGCACATAAACTACGTCGAGT

>Bacillus_Fam_1809_21_1 Nr. of seq. 1 Alignment length(with gaps) = 21
Alignment score = 0.000000
GCF_001278705.1_ASM127870v1_genomic.fna_1:3026647-3026771 Satlength=125
Nr of Repeats=6 RepeatLength=21 seed=TTATGCGCGA Num.seqs=4
Similarity=0.693122 0
TTATGCGCGATCTTCCCTCGG

****
Consensus:

TTATGCGCGATCTTCCCTCGG

>Bacillus_Fam_1810_21_1 Nr. of seq. 1 Alignment length(with gaps) = 21
Alignment score = 0.000000
GCF_001315065.1_ASM131506v1_genomic.fna_5:75663-75783 Satlength=121 Nr of
Repeats=4 RepeatLength=21 seed=AACAAGCACA Num.seqs=3 Similarity=0.601010
0 AACAAGCACAGCANGCAATTC

****
Consensus:

AACAAGCACAGCANGCAATTC

>Bacillus_Fam_1811_21_1 Nr. of seq. 1 Alignment length(with gaps) = 21
Alignment score = 0.000000
GCF_001315165.1_ASM131516v1_genomic.fna_94:3047-3130 Satlength=84 Nr of
Repeats=4 RepeatLength=21 seed=TTTGCGAATA Num.seqs=3 Similarity=0.646465
0 TTTGCGAATAAACCATNNATT

****
Consensus:

TTTGCGAATAAACCATNNATT

>Bacillus_Fam_1812_21_1 Nr. of seq. 1 Alignment length(with gaps) = 21
Alignment score = 0.000000
GCF_001420595.1_ASM142059v1_genomic.fna_11:777561-777645 Satlength=85 Nr
of Repeats=4 RepeatLength=21 seed=TGCTAGATAA Num.seqs=4
Similarity=0.642677
0 TGCTAGATAAATAGCTACAAT

****
Consensus:

TGCTAGATAAATAGCTACAAT

>Bacillus_Fam_1813_21_1 Nr. of seq. 1 Alignment length(with gaps) = 21
Alignment score = 0.000000

GCF_001420715.1_ASM142071v1_genomic.fna_22:401748-401832 Satlength=85 Nr of Repeats=4 RepeatLength=21 seed=GCGGGATTAT Num.seqs=4 Similarity=0.703704
0   GCGGGATTATAGTAAAAATTC

****
Consensus:

GCGGGATTATAGTAAAAATTC

>Bacillus_Fam_1814_21_1 Nr. of seq. 1 Alignment length(with gaps) = 21 Alignment score = 0.000000
GCF_001439635.1_ASM143963v1_genomic.fna_162:47575-47699 Satlength=125 Nr of Repeats=5 RepeatLength=21 seed=TAATAGACAA Num.seqs=3 Similarity=0.537778
0   TAATAGACAAAATACTTNGGN

****
Consensus:

TAATAGACAAAATACTTNGGN

>Bacillus_Fam_1815_21_1 Nr. of seq. 1 Alignment length(with gaps) = 21 Alignment score = 0.000000
GCF_001439915.1_ASM143991v1_genomic.fna_90:1-81 Satlength=81 Nr of Repeats=4 RepeatLength=20 seed=CGACGCAAAT Num.seqs=4 Similarity=0.671958
0   CGACGCAAATAATCCAGTAAT

****
Consensus:

CGACGCAAATAATCCAGTAAT

>Bacillus_Fam_1816_21_1 Nr. of seq. 1 Alignment length(with gaps) = 21 Alignment score = 0.000000
GCF_001439915.1_ASM143991v1_genomic.fna_105:333431-333536 Satlength=106 Nr of Repeats=5 RepeatLength=21 seed=TAGGGCACAA Num.seqs=5 Similarity=0.574603   0
TAGGGCACAATTANTTCCANN

****
Consensus:

TAGGGCACAATTANTTCCANN

>Bacillus_Fam_1817_21_1 Nr. of seq. 1 Alignment length(with gaps) = 21 Alignment score = 0.000000
GCF_001439915.1_ASM143991v1_genomic.fna_122:184310-184415 Satlength=106 Nr of Repeats=5 RepeatLength=21 seed=ACCTCCTAGT Num.seqs=5 Similarity=0.961905   0
ACCTCCTAGTCCAACAGGAAA

****
Consensus:

ACCTCCTAGTCCAACAGGAAA

>Bacillus_Fam_1818_21_1 Nr. of seq. 1 Alignment length(with gaps) = 21
Alignment score = 0.000000
GCF_001484965.1_ASM148496v1_genomic.fna_38:7986-8153 Satlength=168 Nr of Repeats=7 RepeatLength=21 seed=GCGAGAATCC Num.seqs=6 Similarity=0.788889
0   GCGAGAATCCAGACAAGAGGA

****
Consensus:

GCGAGAATCCAGACAAGAGGA

>Bacillus_Fam_1819_21_1 Nr. of seq. 1 Alignment length(with gaps) = 21
Alignment score = 0.000000
GCF_001584325.1_ASM158432v1_genomic.fna_23:115575-115659 Satlength=85 Nr of Repeats=4 RepeatLength=21 seed=GTCCTCCAAT Num.seqs=4
Similarity=0.814394
0   GTCCTCCAATGCTTCAACTCT

****
Consensus:

GTCCTCCAATGCTTCAACTCT

>Bacillus_Fam_1820_21_1 Nr. of seq. 1 Alignment length(with gaps) = 21
Alignment score = 0.000000
GCF_001590785.1_ASM159078v1_genomic.fna_16:23682-23806 Satlength=125 Nr of Repeats=6 RepeatLength=21 seed=GGAAGCGGAG Num.seqs=5
Similarity=0.898413
0   GGAAGCGGAGACGGAGATAGC

****
Consensus:

GGAAGCGGAGACGGAGATAGC

>Bacillus_Fam_1821_21_1 Nr. of seq. 1 Alignment length(with gaps) = 21
Alignment score = 0.000000
GCF_001591425.1_ASM159142v1_genomic.fna_6:69823-70055 Satlength=233 Nr of Repeats=10 RepeatLength=21 seed=GTTGCACCAG Num.seqs=6
Similarity=0.729101
0   GTTGCACCAGTTTGTCACGGT

****
Consensus:

GTTGCACCAGTTTGTCACGGT

>Bacillus_Fam_1822_21_1 Nr. of seq. 1 Alignment length(with gaps) = 21
Alignment score = 0.000000
GCF_001591425.1_ASM159142v1_genomic.fna_57:20937-21147 Satlength=211 Nr of Repeats=9 RepeatLength=21 seed=TGGCACAAAA Num.seqs=8

Similarity=0.649327
0   TGGCACAAAAGGATGTTGGGT

****
Consensus:

TGGCACAAAAGGATGTTGGGT

>Bacillus_Fam_1823_21_1 Nr. of seq. 1 Alignment length(with gaps) = 21
Alignment score = 0.000000
GCF_001591425.1_ASM159142v1_genomic.fna_130:10418-10565 Satlength=148 Nr of Repeats=7 RepeatLength=21 seed=TTGACGCACT Num.seqs=7
Similarity=0.576950
0   TTGACGCACTTGNAAATTTAA

****
Consensus:

TTGACGCACTTGNAAATTTAA

>Bacillus_Fam_1824_21_1 Nr. of seq. 1 Alignment length(with gaps) = 21
Alignment score = 0.000000
GCF_001591425.1_ASM159142v1_genomic.fna_153:1987-2338 Satlength=352 Nr of Repeats=11 RepeatLength=21 seed=TTTATGTGGG Num.seqs=7
Similarity=0.476190
0   TTTATGTGTGCCNGTGAANCN

****
Consensus:

TTTATGTGTGCCNGTGAANCN

>Bacillus_Fam_1825_21_1 Nr. of seq. 1 Alignment length(with gaps) = 21
Alignment score = 0.000000
GCF_001591445.1_ASM159144v1_genomic.fna_5:214153-214237 Satlength=85 Nr of Repeats=4 RepeatLength=21 seed=CCGAAACCTG Num.seqs=4
Similarity=0.904762
0   CCGAAACCTGGGCCTCCTGGG

****
Consensus:

CCGAAACCTGGGCCTCCTGGG

>Bacillus_Fam_1826_21_1 Nr. of seq. 1 Alignment length(with gaps) = 21
Alignment score = 0.000000
GCF_001591465.1_ASM159146v1_genomic.fna_19:29474-29577 Satlength=104 Nr of Repeats=5 RepeatLength=21 seed=TGCAAATAGA Num.seqs=3
Similarity=0.497585
0   TGCAAATAGAANGCANGAATN

****
Consensus:

TGCAAATAGAANGCANGAATN

>Bacillus_Fam_1827_21_1 Nr. of seq. 1 Alignment length(with gaps) = 21
Alignment score = 0.000000
GCF_001591465.1_ASM159146v1_genomic.fna_20:25050-25133 Satlength=84 Nr of Repeats=4 RepeatLength=21 seed=TTTGGCCGGT Num.seqs=3 Similarity=0.585859
0   TTTGGCCGGTAAATTATGCAN

****
Consensus:

TTTGGCCGGTAAATTATGCAN

>Bacillus_Fam_1828_21_1 Nr. of seq. 1 Alignment length(with gaps) = 21
Alignment score = 0.000000
GCF_001591565.1_ASM159156v1_genomic.fna_44:15843-15969 Satlength=127 Nr of Repeats=4 RepeatLength=21 seed=GAAGCTCTTC Num.seqs=3
Similarity=0.746032
0   GAAGCTCTTCGGACTGCGTTT

****
Consensus:

GAAGCTCTTCGGACTGCGTTT

>Bacillus_Fam_1829_21_1 Nr. of seq. 1 Alignment length(with gaps) = 21
Alignment score = 0.000000
GCF_001591585.1_ASM159158v1_genomic.fna_5:250479-251099 Satlength=621 Nr of Repeats=9 RepeatLength=21 seed=TATCAGCCAA Num.seqs=8
Similarity=0.826028
0   TATCAGCCAAACTCGAGGGCT

****
Consensus:

TATCAGCCAAACTCGAGGGCT

>Bacillus_Fam_1830_21_1 Nr. of seq. 1 Alignment length(with gaps) = 21
Alignment score = 0.000000
GCF_001591665.1_ASM159166v1_genomic.fna_56:43606-43761 Satlength=156 Nr of Repeats=5 RepeatLength=21 seed=AAATAGGTAG Num.seqs=4
Similarity=0.756614
0   AAATAGGTAGTAAGTGACCAT

****
Consensus:

AAATAGGTAGTAAGTGACCAT

>Bacillus_Fam_1831_21_1 Nr. of seq. 1 Alignment length(with gaps) = 21
Alignment score = 0.000000
GCF_001591805.1_ASM159180v1_genomic.fna_38:45950-46055 Satlength=106 Nr of Repeats=5 RepeatLength=21 seed=TTTTGTTGAA Num.seqs=5

Similarity=0.703030
0   TTTTGTTGAATGGCACCTGGT

****
Consensus:

TTTTGTTGAATGGCACCTGGT

>Bacillus_Fam_1832_21_1 Nr. of seq. 1 Alignment length(with gaps) = 21
Alignment score = 0.000000
GCF_001592005.1_ASM159200v1_genomic.fna_23:5049-5133 Satlength=85 Nr of Repeats=4 RepeatLength=21 seed=CGCCTTGACG Num.seqs=4 Similarity=0.915344
0   CGCCTTGACGGACTAGACCAA

****
Consensus:

CGCCTTGACGGACTAGACCAA

>Bacillus_Fam_1833_21_1 Nr. of seq. 1 Alignment length(with gaps) = 21
Alignment score = 0.000000
GCF_001636315.1_ASM163631v1_genomic.fna_1:2331927-2332076 Satlength=150 Nr of Repeats=6 RepeatLength=21 seed=TTAGCGATTT Num.seqs=5 Similarity=0.606349 0
TTAGCGATTTNANACNGCCTA

****
Consensus:

TTAGCGATTTNANACNGCCTA

>Bacillus_Fam_1834_21_1 Nr. of seq. 1 Alignment length(with gaps) = 21
Alignment score = 0.000000
GCF_001636315.1_ASM163631v1_genomic.fna_1:5159452-5159597 Satlength=146 Nr of Repeats=6 RepeatLength=21 seed=GACGGGTAAA Num.seqs=5 Similarity=0.568182 0
GACGGGTAAAATCNAAAANTT

****
Consensus:

GACGGGTAAAATCNAAAANTT

>Bacillus_Fam_1835_21_1 Nr. of seq. 1 Alignment length(with gaps) = 21
Alignment score = 0.000000
GCF_001636335.1_ASM163633v1_genomic.fna_1:60506-60879 Satlength=374 Nr of Repeats=18 RepeatLength=21 seed=GGCGAATAAA Num.seqs=15 Similarity=0.855480
0   GGCGAATAAAACAGTGAGCAT

****
Consensus:

GGCGAATAAAACAGTGAGCAT

>Bacillus_Fam_1836_21_1 Nr. of seq. 1 Alignment length(with gaps) = 21
Alignment score = 0.000000
GCF_001636345.1_ASM163634v1_genomic.fna_1:402480-402564 Satlength=85 Nr of Repeats=4 RepeatLength=21 seed=TTCCTATCAA Num.seqs=4
Similarity=0.474638
0 TTCCTATCAAAANAAGCGGAC

****
Consensus:

TTCCTATCAAAANAAGCGGAC

>Bacillus_Fam_1837_21_1 Nr. of seq. 1 Alignment length(with gaps) = 21
Alignment score = 0.000000
GCF_001636345.1_ASM163634v1_genomic.fna_1:537147-537252 Satlength=106 Nr of Repeats=4 RepeatLength=21 seed=AATGATCGAA Num.seqs=3
Similarity=0.536232
0 AATGATCGAAAANATCTCNAN

****
Consensus:

AATGATCGAAAANATCTCNAN

>Bacillus_Fam_1838_21_1 Nr. of seq. 1 Alignment length(with gaps) = 21
Alignment score = 0.000000
GCF_001636425.1_ASM163642v1_genomic.fna_1:4338946-4339051 Satlength=106 Nr of Repeats=5 RepeatLength=21 seed=AAATGTTGCA Num.seqs=3
Similarity=0.703704 0
AAATGTTGCACAAAAACGAAA

****
Consensus:

AAATGTTGCACAAAAACGAAA

>Bacillus_Fam_1839_21_1 Nr. of seq. 1 Alignment length(with gaps) = 21
Alignment score = 0.000000
GCF_001645685.2_ASM164568v2_genomic.fna_1:4195211-4195333 Satlength=123 Nr of Repeats=5 RepeatLength=21 seed=GGACGAATTA Num.seqs=4
Similarity=0.765152 0
GGACGAATTATCTCCTGATTG

****
Consensus:

GGACGAATTATCTCCTGATTG

>Bacillus_Fam_1840_21_1 Nr. of seq. 1 Alignment length(with gaps) = 21
Alignment score = 0.000000
GCF_001645705.1_ASM164570v1_genomic.fna_58:251245-252259 Satlength=1015 Nr of Repeats=41 RepeatLength=21 seed=ATTGCGCAAC Num.seqs=25

Similarity=0.654747 0
ATTGCGCGACTTTCATNTGGN

****
Consensus:

ATTGCGCGACTTTCATNTGGN

>Bacillus_Fam_1841_21_1 Nr. of seq. 1 Alignment length(with gaps) = 21
Alignment score = 0.000000
GCF_001645705.1_ASM164570v1_genomic.fna_61:116393-116850 Satlength=458 Nr of Repeats=22 RepeatLength=21 seed=AGTCGCGCAA Num.seqs=18
Similarity=0.733908 0
AGTCGCGCAATGCAGGTATAA

****
Consensus:

AGTCGCGCAATGCAGGTATAA

>Bacillus_Fam_1842_21_1 Nr. of seq. 1 Alignment length(with gaps) = 21
Alignment score = 0.000000
GCF_001648575.1_ASM164857v1_genomic.fna_55:22587-22754 Satlength=168 Nr of Repeats=6 RepeatLength=21 seed=TGACGGGATT Num.seqs=5
Similarity=0.506061
0 TGACGGGATTNCCNNTTCTTA

****
Consensus:

TGACGGGATTNCCNNTTCTTA

>Bacillus_Fam_1843_21_1 Nr. of seq. 1 Alignment length(with gaps) = 21
Alignment score = 0.000000
GCF_001648575.1_ASM164857v1_genomic.fna_61:58107-58295 Satlength=189 Nr of Repeats=6 RepeatLength=21 seed=TGAAGGGATT Num.seqs=4
Similarity=0.502645
0 TGAAGGGATTTTNCACACTTG

****
Consensus:

TGAAGGGATTTTNCACACTTG

>Bacillus_Fam_1844_21_1 Nr. of seq. 1 Alignment length(with gaps) = 21
Alignment score = 0.000000
GCF_001648575.1_ASM164857v1_genomic.fna_64:41402-41486 Satlength=85 Nr of Repeats=4 RepeatLength=21 seed=GCACGAACTC Num.seqs=4 Similarity=0.968254
0 GCACGAACTCCGAGCGTTTCC

****
Consensus:

GCACGAACTCCGAGCGTTTCC

>Bacillus_Fam_1845_21_1 Nr. of seq. 1 Alignment length(with gaps) = 21
Alignment score = 0.000000
GCF_001712755.1_ASM171275v1_genomic.fna_2:319626-319913 Satlength=288 Nr of Repeats=11 RepeatLength=21 seed=TGCAAATAAA Num.seqs=7
Similarity=0.477460 0
TGCAAATAAAANTNNTCAAGN

****
Consensus:

TGCAAATAAAANTNNTCAAGN

>Bacillus_Fam_1846_21_1 Nr. of seq. 1 Alignment length(with gaps) = 21
Alignment score = 0.000000
GCF_001712755.1_ASM171275v1_genomic.fna_36:229235-229442 Satlength=208 Nr of Repeats=10 RepeatLength=21 seed=TTATTTGCAG Num.seqs=9
Similarity=0.379630 0
TTATTTGCAGNNTTNAATNTN

****
Consensus:

TTATTTGCAGNNTTNAATNTN

>Bacillus_Fam_1847_21_1 Nr. of seq. 1 Alignment length(with gaps) = 21
Alignment score = 0.000000
GCF_001889165.1_ASM188916v1_genomic.fna_1:3543299-3543404 Satlength=106 Nr of Repeats=5 RepeatLength=21 seed=TTTATTTTCG Num.seqs=5
Similarity=0.520833 0
TTTATTTTCGNTTTTCCTNGT

****
Consensus:

TTTATTTTCGNTTTTCCTNGT

>Bacillus_Fam_1848_21_1 Nr. of seq. 1 Alignment length(with gaps) = 21
Alignment score = 0.000000
GCF_001889165.1_ASM188916v1_genomic.fna_1:3636446-3636551 Satlength=106 Nr of Repeats=4 RepeatLength=21 seed=TTTATTTTCG Num.seqs=3
Similarity=0.512077 0
TTTATTTTCGNTTTCTACGCA

****
Consensus:

TTTATTTTCGNTTTCTACGCA

>Bacillus_Fam_1849_21_1 Nr. of seq. 1 Alignment length(with gaps) = 21
Alignment score = 0.000000
GCF_002009555.1_ASM200955v1_genomic.fna_561:6003-6229 Satlength=227 Nr of Repeats=8 RepeatLength=21 seed=TCGTGCGAAT Num.seqs=5 Similarity=0.654545
0   TCGTGCGAATCATNTCGGAAT

****
Consensus:

TCGTGCGAATCATNTCGGAAT

>Bacillus_Fam_1850_21_1 Nr. of seq. 1 Alignment length(with gaps) = 21
Alignment score = 0.000000
GCF_002019645.1_ASM201964v1_genomic.fna_1:2245902-2246234 Satlength=333
Nr of Repeats=9 RepeatLength=21 seed=AATCCGGCGG Num.seqs=6
Similarity=0.548000 0
AATCCGGCGGATNTGAAGCAG

****
Consensus:

AATCCGGCGGATNTGAAGCAG

>Bacillus_Fam_1851_21_1 Nr. of seq. 1 Alignment length(with gaps) = 21
Alignment score = 0.000000
GCF_002019665.1_ASM201966v1_genomic.fna_1:767787-767892 Satlength=106 Nr
of Repeats=4 RepeatLength=21 seed=ATTCGTCCAA Num.seqs=3
Similarity=0.564815
0   ATTCGTCCAAAATCCNGTAAC

****
Consensus:

ATTCGTCCAAAATCCNGTAAC

>Bacillus_Fam_1852_21_1 Nr. of seq. 1 Alignment length(with gaps) = 21
Alignment score = 0.000000
GCF_002019665.1_ASM201966v1_genomic.fna_1:1184140-1184299 Satlength=160
Nr of Repeats=6 RepeatLength=21 seed=TTGGACGAAT Num.seqs=5
Similarity=0.528986 0
TTGGACGAATNAGNGCGNGAA

****
Consensus:

TTGGACGAATNAGNGCGNGAA

>Bacillus_Fam_1853_21_1 Nr. of seq. 1 Alignment length(with gaps) = 21
Alignment score = 0.000000
GCF_900094975.1_IMG-
taxon_2617270721_annotated_assembly_genomic.fna_9:8023-8157 Satlength=135
Nr of Repeats=4 RepeatLength=21 seed=TATTATCGAA Num.seqs=3
Similarity=0.550725 0
TATTATCGAAATCNANAAATT

****
Consensus:

TATTATCGAAATCNANAAATT

>Bacillus_Fam_1854_21_1 Nr. of seq. 1 Alignment length(with gaps) = 21
Alignment score = 0.000000
GCF_900094975.1_IMG-taxon_2617270721_annotated_assembly_genomic.fna_13:445117-445197
Satlength=81 Nr of Repeats=4 RepeatLength=20 seed=GGCGCAATTA Num.seqs=4
Similarity=0.576720   0   GGCGCAATTAATTAGANAGTN

****
Consensus:

GGCGCAATTAATTAGANAGTN

>Bacillus_Fam_1855_21_1 Nr. of seq. 1 Alignment length(with gaps) = 21
Alignment score = 0.000000
GCF_900103955.1_IMG-taxon_2617270767_annotated_assembly_genomic.fna_27:91618-91930
Satlength=313 Nr of Repeats=5 RepeatLength=21 seed=TTAGATGTTA Num.seqs=4
Similarity=0.798942   0   TTAGATGTTAGTATAGCGTGT

****
Consensus:

TTAGATGTTAGTATAGCGTGT

>Bacillus_Fam_1856_21_1 Nr. of seq. 1 Alignment length(with gaps) = 21
Alignment score = 0.000000
GCF_900107275.1_IMG-taxon_2675903024_annotated_assembly_genomic.fna_11:61167-61272
Satlength=106 Nr of Repeats=5 RepeatLength=21 seed=TCGTCTTCTT Num.seqs=5
Similarity=0.898413   0   TCGTCTTCTTCATGCGCGTGA

****
Consensus:

TCGTCTTCTTCATGCGCGTGA

>Bacillus_Fam_1857_21_1 Nr. of seq. 1 Alignment length(with gaps) = 21
Alignment score = 0.000000
GCF_900107275.1_IMG-taxon_2675903024_annotated_assembly_genomic.fna_35:83306-83474
Satlength=169 Nr of Repeats=8 RepeatLength=21 seed=CGGCTTGGAA Num.seqs=8
Similarity=0.768707   0   CGGCTTGGAAAACAAAGTCGA

****
Consensus:

CGGCTTGGAAAACAAAGTCGA

>Bacillus_Fam_1858_21_1 Nr. of seq. 1 Alignment length(with gaps) = 21
Alignment score = 0.000000
GCF_900156865.1_PRJEB18960_genomic.fna_3:2979413-2979928 Satlength=516 Nr of Repeats=15 RepeatLength=21 seed=TTATGGGACA Num.seqs=11

Similarity=0.639669 0
TTATGGGACACTGGTTTCATN

****
Consensus:

TTATGGGACACTGGTTTCATN

>Bacillus_Fam_1859_21_1 Nr. of seq. 1 Alignment length(with gaps) = 21
Alignment score = 0.000000
GCF_900156875.1_PRJEB18969_genomic.fna_11:3145660-3145883 Satlength=224
Nr of Repeats=5 RepeatLength=21 seed=TTCATTTCTT Num.seqs=4
Similarity=0.756614 0
TTCATTTCTTGTAGATCCCGC

****
Consensus:

TTCATTTCTTGTAGATCCCGC

>Bacillus_Fam_1860_20_1 Nr. of seq. 1 Alignment length(with gaps) = 20
Alignment score = 0.000000
GCF_000175075.1_ASM17507v1_genomic.fna_29:81534-81653 Satlength=120 Nr of
Repeats=6 RepeatLength=20 seed=TTTCTTGATA Num.seqs=5 Similarity=0.561905
0 TTTCTTGATATANTGTNAAT

****
Consensus:

TTTCTTGATATANTGTNAAT

>Bacillus_Fam_1861_20_1 Nr. of seq. 1 Alignment length(with gaps) = 20
Alignment score = 0.000000
GCF_000285535.1_ASM28553v1_genomic.fna_2:955930-956292 Satlength=363 Nr
of Repeats=14 RepeatLength=20 seed=TTTGCGTCAA Num.seqs=9
Similarity=0.588183
0 TTTGCGTCAACANCCACAGA

****
Consensus:

TTTGCGTCAACANCCACAGA

>Bacillus_Fam_1862_20_1 Nr. of seq. 1 Alignment length(with gaps) = 20
Alignment score = 0.000000
GCF_000285535.1_ASM28553v1_genomic.fna_60:15-376 Satlength=362 Nr of
Repeats=18 RepeatLength=20 seed=ATTTGCGTCT Num.seqs=17
Similarity=0.590463
0 ATTTGCGTCNNTTCCNNCNG

****
Consensus:

ATTTGCGTCNNTTCCNNCNG

>Bacillus_Fam_1863_20_1 Nr. of seq. 1 Alignment length(with gaps) = 20
Alignment score = 0.000000
GCF_000331575.1_ASM33157v1_genomic.fna_1:1625853-1626033 Satlength=181 Nr of Repeats=9 RepeatLength=20 seed=TAATGAGGGT Num.seqs=9 Similarity=0.974074 0
TAATGAGGGTATTAAACTTT

****
Consensus:

TAATGAGGGTATTAAACTTT

>Bacillus_Fam_1864_20_1 Nr. of seq. 1 Alignment length(with gaps) = 20
Alignment score = 0.000000
GCF_000429705.1_ASM42970v1_genomic.fna_2:323368-323473 Satlength=106 Nr of Repeats=5 RepeatLength=21 seed=TTATCCATCA Num.seqs=3 Similarity=0.666667
0   TTATCCATCACTTCGGCCGG

****
Consensus:

TTATCCATCACTTCGGCCGG

>Bacillus_Fam_1865_20_1 Nr. of seq. 1 Alignment length(with gaps) = 20
Alignment score = 0.000000
GCF_000612665.1_FF4_genomic.fna_66:43632-44026 Satlength=395 Nr of Repeats=8 RepeatLength=21 seed=TTTACCGGCA Num.seqs=5 Similarity=0.461728
0   TTTACCGGCACTTTCACNAT

****
Consensus:

TTTACCGGCACTTTCACNAT

>Bacillus_Fam_1866_20_1 Nr. of seq. 1 Alignment length(with gaps) = 20
Alignment score = 0.000000
GCF_000752035.1_Bacillus_rubiinfantis_genomic.fna_13:80293-80396 Satlength=104 Nr of Repeats=5 RepeatLength=20 seed=GTTACTTGCG Num.seqs=3 Similarity=0.592593 0
TTACTTGCGGTTGGGGNGCG

****
Consensus:

TTACTTGCGGTTGGGGNGCG

>Bacillus_Fam_1867_20_1 Nr. of seq. 1 Alignment length(with gaps) = 20
Alignment score = 0.000000
GCF_000752395.1_Bacillus_andreraoultii_genomic.fna_12:210479-210580 Satlength=102 Nr of Repeats=5 RepeatLength=20 seed=TTTCCGAAGT Num.seqs=4 Similarity=0.562169 0
TTTCCGAAGTTGGCGTGTTT

****
Consensus:

TTTCCGAAGTTGGCGTGTTT

>Bacillus_Fam_1868_20_1 Nr. of seq. 1 Alignment length(with gaps) = 20
Alignment score = 0.000000
GCF_000759675.1_ASM75967v1_genomic.fna_13:139600-139845 Satlength=246 Nr of Repeats=5 RepeatLength=20 seed=TTTACGTGCC Num.seqs=4
Similarity=0.593915
0   TTTACGTGCCAAATTATAGA

****
Consensus:

TTTACGTGCCAAATTATAGA

>Bacillus_Fam_1869_20_1 Nr. of seq. 1 Alignment length(with gaps) = 20
Alignment score = 0.000000
GCF_001420595.1_ASM142059v1_genomic.fna_11:1231069-1231171 Satlength=103 Nr of Repeats=5 RepeatLength=20 seed=TATTTGCAAA Num.seqs=3
Similarity=0.644444 0
TAATTGCAAAAATCCTTGAA

****
Consensus:

TAATTGCAAAAATCCTTGAA

>Bacillus_Fam_1870_20_1 Nr. of seq. 1 Alignment length(with gaps) = 20
Alignment score = 0.000000
GCF_001420715.1_ASM142071v1_genomic.fna_22:116628-116728 Satlength=101 Nr of Repeats=5 RepeatLength=20 seed=TACATTAAAG Num.seqs=5
Similarity=0.933333 0
TACATTAAAGTTCACTACTT

****
Consensus:

TACATTAAAGTTCACTACTT

>Bacillus_Fam_1871_20_1 Nr. of seq. 1 Alignment length(with gaps) = 20
Alignment score = 0.000000
GCF_001636325.1_ASM163632v1_genomic.fna_2:476021-476159 Satlength=139 Nr of Repeats=6 RepeatLength=20 seed=GGCGCAAAAA Num.seqs=5
Similarity=0.590909
0   GGCGCAAAAATCGAATGAGA

****
Consensus:

GGCGCAAAAATCGAATGAGA

>Bacillus_Fam_1872_20_1 Nr. of seq. 1 Alignment length(with gaps) = 20
Alignment score = 0.000000
GCF_001636345.1_ASM163634v1_genomic.fna_1:867035-867350 Satlength=316 Nr of Repeats=4 RepeatLength=20 seed=GCGAATATAT Num.seqs=3
Similarity=0.600000
0   GCGAATATATTCNTATNACG

****
Consensus:

GCGAATATATTCNTATNACG

>Bacillus_Fam_1873_20_1 Nr. of seq. 1 Alignment length(with gaps) = 20
Alignment score = 0.000000
GCF_001645685.2_ASM164568v2_genomic.fna_1:349315-349619 Satlength=305 Nr of Repeats=14 RepeatLength=20 seed=CGAATAAATC Num.seqs=9
Similarity=0.758377 0
CGAATAAATCAGAGTAAGGT

****
Consensus:

CGAATAAATCAGAGTAAGGT

>Bacillus_Fam_1874_20_1 Nr. of seq. 1 Alignment length(with gaps) = 20
Alignment score = 0.000000
GCF_001648575.1_ASM164857v1_genomic.fna_64:202939-203079 Satlength=141 Nr of Repeats=6 RepeatLength=20 seed=TTATCCTGAA Num.seqs=5
Similarity=0.760000 0
TTATCCTGAAATCTATGGGT

****
Consensus:

TTATCCTGAAATCTATGGGT

>Bacillus_Fam_1875_20_1 Nr. of seq. 1 Alignment length(with gaps) = 20
Alignment score = 0.000000
GCF_001648575.1_ASM164857v1_genomic.fna_66:119260-119427 Satlength=168 Nr of Repeats=6 RepeatLength=21 seed=TGACGGGATT Num.seqs=5
Similarity=0.461538 0
TGACGGGATTTTAGTNGTTA

****
Consensus:

TGACGGGATTTTAGTNGTTA

>Bacillus_Fam_1876_20_1 Nr. of seq. 1 Alignment length(with gaps) = 20
Alignment score = 0.000000
GCF_001648575.1_ASM164857v1_genomic.fna_67:289399-289588 Satlength=190 Nr of Repeats=8 RepeatLength=21 seed=ATTGTGACTT Num.seqs=7
Similarity=0.590608 0
ATTGTGACTTTTCCATNGTT

****
Consensus:

ATTGTGACTTTTCCATNGTT

>Bacillus_Fam_1877_20_1 Nr. of seq. 1 Alignment length(with gaps) = 20
Alignment score = 0.000000
GCF_001887185.1_ASM188718v1_genomic.fna_259:169310-169414 Satlength=105
Nr of Repeats=4 RepeatLength=20 seed=CGAGTTTTTT Num.seqs=3
Similarity=0.671958 0
CGAGTTTTTTAGGTCAGGAG

****
Consensus:

CGAGTTTTTTAGGTCAGGAG

>Bacillus_Fam_1878_20_1 Nr. of seq. 1 Alignment length(with gaps) = 20
Alignment score = 0.000000
GCF_002019665.1_ASM201966v1_genomic.fna_1:4193194-4193625 Satlength=432
Nr of Repeats=21 RepeatLength=21 seed=TTTATGAGCC Num.seqs=16
Similarity=0.603684 0
TTTATGAGCCANTACCTCTN

****
Consensus:

TTTATGAGCCANTACCTCTN

>Bacillus_Fam_1879_20_1 Nr. of seq. 1 Alignment length(with gaps) = 20
Alignment score = 0.000000
GCF_002019705.1_ASM201970v1_genomic.fna_1:2674002-2674287 Satlength=286
Nr of Repeats=14 RepeatLength=20 seed=CCTGAGCTCC Num.seqs=9
Similarity=0.874074 0
CCTGAGCTCCTCTCTCTAAT

****
Consensus:

CCTGAGCTCCTCTCTCTAAT

>Bacillus_Fam_1880_20_1 Nr. of seq. 1 Alignment length(with gaps) = 20
Alignment score = 0.000000
GCF_900103955.1_IMG-
taxon_2617270767_annotated_assembly_genomic.fna_7:15309-15864
Satlength=556 Nr of Repeats=5 RepeatLength=20 seed=AGTAAAATAG Num.seqs=3
Similarity=0.955556 0 AGTAAAATAGCGGGCGCGAC

****
Consensus:

AGTAAAATAGCGGGCGCGAC

>Bacillus_Fam_1881_20_1 Nr. of seq. 1 Alignment length(with gaps) = 20
Alignment score = 0.000000
GCF_900156875.1_PRJEB18969_genomic.fna_11:3436401-3436481 Satlength=81 Nr of Repeats=4 RepeatLength=20 seed=TTTATCCGTA Num.seqs=4
Similarity=0.547980 0
TTTATCCGTAAGAATTTCAN

****
Consensus:

TTTATCCGTAAGAATTTCAN

>Bacillus_Fam_1882_19_1 Nr. of seq. 1 Alignment length(with gaps) = 19
Alignment score = 0.000000
GCF_000331575.1_ASM33157v1_genomic.fna_1:1363446-1363522 Satlength=77 Nr of Repeats=4 RepeatLength=19 seed=TATATTCTCT Num.seqs=4
Similarity=0.918129
0   TATATTCTCTACGTTATTA

****
Consensus:

TATATTCTCTACGTTATTA

>Bacillus_Fam_1883_19_1 Nr. of seq. 1 Alignment length(with gaps) = 19
Alignment score = 0.000000
GCF_000331575.1_ASM33157v1_genomic.fna_1:1618267-1618343 Satlength=77 Nr of Repeats=4 RepeatLength=19 seed=CTTGATTGAG Num.seqs=4
Similarity=0.929825
0   CTTGATTGAGAATCCTCAG

****
Consensus:

CTTGATTGAGAATCCTCAG

>Bacillus_Fam_1884_19_1 Nr. of seq. 1 Alignment length(with gaps) = 19
Alignment score = 0.000000
GCF_001889165.1_ASM188916v1_genomic.fna_1:874722-874938 Satlength=217 Nr of Repeats=9 RepeatLength=18 seed=TCATCCTCAG Num.seqs=6
Similarity=0.696491
0   TCATCCTCAGAATGAGTGC

****
Consensus:

TCATCCTCAGAATGAGTGC

>Bacillus_Fam_1885_18_1 Nr. of seq. 1 Alignment length(with gaps) = 18
Alignment score = 0.000000
GCF_000005825.2_ASM582v2_genomic.fna_1:3039924-3040044 Satlength=121 Nr of Repeats=6 RepeatLength=18 seed=GGCAATGGAA Num.seqs=5
Similarity=1.000000
0   GGCAATGGAAACGGGAAT

****
Consensus:

GGCAATGGAAACGGGAAT

>Bacillus_Fam_1886_18_1 Nr. of seq. 1 Alignment length(with gaps) = 18 Alignment score = 0.000000
GCF_000008505.1_ASM850v1_genomic.fna_1:4344849-4345011 Satlength=163 Nr of Repeats=9 RepeatLength=18 seed=CGTCTGGTTG Num.seqs=9
Similarity=0.909465
0   CGTCTGGTTGTTTGTCTC

****
Consensus:

CGTCTGGTTGTTTGTCTC

>Bacillus_Fam_1887_18_1 Nr. of seq. 1 Alignment length(with gaps) = 18 Alignment score = 0.000000
GCF_000025805.1_ASM2580v1_genomic.fna_1:1137271-1137343 Satlength=73 Nr of Repeats=4 RepeatLength=18 seed=TCAAAACCGT Num.seqs=4
Similarity=1.000000
0   TCAAAACCGTAGTTAAGG

****
Consensus:

TCAAAACCGTAGTTAAGG

>Bacillus_Fam_1888_18_1 Nr. of seq. 1 Alignment length(with gaps) = 18 Alignment score = 0.000000
GCF_000025805.1_ASM2580v1_genomic.fna_1:2256750-2257164 Satlength=415 Nr of Repeats=23 RepeatLength=18 seed=CCTGGTATGG Num.seqs=23
Similarity=0.839054 0
CCTGGTATGGGCGGATCA

****
Consensus:

CCTGGTATGGGCGGATCA

>Bacillus_Fam_1889_18_1 Nr. of seq. 1 Alignment length(with gaps) = 18 Alignment score = 0.000000
GCF_000161455.1_ASM16145v1_genomic.fna_1:1820901-1821153 Satlength=253 Nr of Repeats=13 RepeatLength=18 seed=TGTGTTTACG Num.seqs=12
Similarity=0.852974 0
TGTGTTTACGGCATGTTG

****
Consensus:

TGTGTTTACGGCATGTTG

>Bacillus_Fam_1890_18_1 Nr. of seq. 1 Alignment length(with gaps) = 18
Alignment score = 0.000000
GCF_000161455.1_ASM16145v1_genomic.fna_1:4034040-4034130 Satlength=91 Nr of Repeats=5 RepeatLength=18 seed=TGACCGTGAT Num.seqs=5 Similarity=0.822222
0   TGACCGTGATGGACGTGA

****
Consensus:

TGACCGTGATGGACGTGA

>Bacillus_Fam_1891_18_1 Nr. of seq. 1 Alignment length(with gaps) = 18
Alignment score = 0.000000
GCF_000242895.2_ASM24289v3_genomic.fna_1:1111316-1112777 Satlength=1462 Nr of Repeats=65 RepeatLength=18 seed=GTTGTCGTCG Num.seqs=47 Similarity=0.825813 0
GTTGTCGTCGGTGCGGCN

****
Consensus:

GTTGTCGTCGGTGCGGCN

>Bacillus_Fam_1892_18_1 Nr. of seq. 1 Alignment length(with gaps) = 18
Alignment score = 0.000000
GCF_000292245.2_ASM29224v2_genomic.fna_57:3327-3507 Satlength=181 Nr of Repeats=10 RepeatLength=18 seed=GTTAACTGAG Num.seqs=10 Similarity=0.965432
0   GTTAACTGAGGAAGAAGG

****
Consensus:

GTTAACTGAGGAAGAAGG

>Bacillus_Fam_1893_18_1 Nr. of seq. 1 Alignment length(with gaps) = 18
Alignment score = 0.000000
GCF_000307875.1_BABA1.0_genomic.fna_120:4680-4752 Satlength=73 Nr of Repeats=4 RepeatLength=18 seed=AAAGAACAAC Num.seqs=4 Similarity=0.851852
0   AAAGAACAACCAATCATC

****
Consensus:

AAAGAACAACCAATCATC

>Bacillus_Fam_1894_18_1 Nr. of seq. 1 Alignment length(with gaps) = 18
Alignment score = 0.000000
GCF_000321185.1_ASM32118v1_genomic.fna_87:138564-139038 Satlength=475 Nr of Repeats=26 RepeatLength=18 seed=GATGCCTGGT Num.seqs=25 Similarity=0.892839 0
GATGCCTGGTGGTGGAAT

****
Consensus:

GATGCCTGGTGGTGGAAT

>Bacillus_Fam_1895_18_1 Nr. of seq. 1 Alignment length(with gaps) = 18
Alignment score = 0.000000
GCF_000331575.1_ASM33157v1_genomic.fna_4:14090-14162 Satlength=73 Nr of Repeats=4 RepeatLength=18 seed=TAATTAGTTA Num.seqs=4 Similarity=0.802469
0 TAATTAGTTACCAAGCAA

****
Consensus:

TAATTAGTTACCAAGCAA

>Bacillus_Fam_1896_18_1 Nr. of seq. 1 Alignment length(with gaps) = 18
Alignment score = 0.000000
GCF_000429725.1_ASM42972v1_genomic.fna_19:52932-53076 Satlength=145 Nr of Repeats=8 RepeatLength=18 seed=TGCTGCTGAA Num.seqs=8 Similarity=0.928571
0 TGCTGCTGAAGAGCAGGC

****
Consensus:

TGCTGCTGAAGAGCAGGC

>Bacillus_Fam_1897_18_1 Nr. of seq. 1 Alignment length(with gaps) = 18
Alignment score = 0.000000
GCF_000430765.1_ASM43076v1_genomic.fna_12:155300-155504 Satlength=205 Nr of Repeats=10 RepeatLength=18 seed=GGAAAGAGAA Num.seqs=9 Similarity=0.843621 0
GGAAAGAGAACGCGAGCG

****
Consensus:

GGAAAGAGAACGCGAGCG

>Bacillus_Fam_1898_18_1 Nr. of seq. 1 Alignment length(with gaps) = 18
Alignment score = 0.000000
GCF_000430785.1_ASM43078v1_genomic.fna_4:98178-98286 Satlength=109 Nr of Repeats=6 RepeatLength=18 seed=CATCTAAACC Num.seqs=6 Similarity=0.876543
0 CATCTAAACCAACTACAG

****
Consensus:

CATCTAAACCAACTACAG

>Bacillus_Fam_1899_18_1 Nr. of seq. 1 Alignment length(with gaps) = 18
Alignment score = 0.000000

GCF_000430785.1_ASM43078v1_genomic.fna_13:56715-57603 Satlength=889 Nr of Repeats=13 RepeatLength=18 seed=AGAGCGTTTA Num.seqs=9 Similarity=0.905350
0   AGAGCGTTTAGACTGCTT

****
Consensus:

AGAGCGTTTAGACTGCTT

>Bacillus_Fam_1900_18_1 Nr. of seq. 1 Alignment length(with gaps) = 18 Alignment score = 0.000000
GCF_000496285.1_ASM49628v1_genomic.fna_1:3550735-3550825 Satlength=91 Nr of Repeats=5 RepeatLength=18 seed=TATTATGAAT Num.seqs=5 Similarity=0.911111
0   TATTATGAATAAAGAATC

****
Consensus:

TATTATGAATAAAGAATC

>Bacillus_Fam_1901_18_1 Nr. of seq. 1 Alignment length(with gaps) = 18 Alignment score = 0.000000
GCF_000706725.1_ASM70672v1_genomic.fna_1:3385744-3385960 Satlength=217 Nr of Repeats=10 RepeatLength=18 seed=AGCTCTTGAA Num.seqs=9 Similarity=0.827160 0
AGCTCTTGAAGACGACAC

****
Consensus:

AGCTCTTGAAGACGACAC

>Bacillus_Fam_1902_18_1 Nr. of seq. 1 Alignment length(with gaps) = 18 Alignment score = 0.000000
GCF_000712595.1_ASM71259v1_genomic.fna_17:58333-58441 Satlength=109 Nr of Repeats=6 RepeatLength=18 seed=CACGGACATC Num.seqs=6 Similarity=0.846914
0   CACGGACATCACCATGGC

****
Consensus:

CACGGACATCACCATGGC

>Bacillus_Fam_1903_18_1 Nr. of seq. 1 Alignment length(with gaps) = 18 Alignment score = 0.000000
GCF_000712615.1_ASM71261v1_genomic.fna_14:133-205 Satlength=73 Nr of Repeats=4 RepeatLength=18 seed=CCTAAGGACC Num.seqs=4 Similarity=0.888889
0   CCTAAGGACCCTATAGAC

****
Consensus:

CCTAAGGACCCTATAGAC

>Bacillus_Fam_1904_18_1 Nr. of seq. 1 Alignment length(with gaps) = 18
Alignment score = 0.000000
GCF_000724485.1_ASM72448v1_genomic.fna_1:375647-375902 Satlength=256 Nr of Repeats=14 RepeatLength=18 seed=CCAGGACAGC Num.seqs=13
Similarity=0.856600 0
CCAGGACAGCCAGGCANC

****
Consensus:

CCAGGACAGCCAGGCANC

>Bacillus_Fam_1905_18_1 Nr. of seq. 1 Alignment length(with gaps) = 18
Alignment score = 0.000000
GCF_000751775.1_A1A_genomic.fna_98:19034-19286 Satlength=253 Nr of Repeats=14 RepeatLength=18 seed=TCACCTGGTT Num.seqs=14
Similarity=0.844526
0   TCACCTGGTTCTTCGTTA

****
Consensus:

TCACCTGGTTCTTCGTTA

>Bacillus_Fam_1906_18_1 Nr. of seq. 1 Alignment length(with gaps) = 18
Alignment score = 0.000000
GCF_000759675.1_ASM75967v1_genomic.fna_158:156695-156902 Satlength=208 Nr of Repeats=8 RepeatLength=18 seed=AGCAGAGGGT Num.seqs=7
Similarity=0.795414 0
AGCAGAGGGTCAAAAGGC

****
Consensus:

AGCAGAGGGTCAAAAGGC

>Bacillus_Fam_1907_18_1 Nr. of seq. 1 Alignment length(with gaps) = 18
Alignment score = 0.000000
GCF_000759675.1_ASM75967v1_genomic.fna_162:138323-138431 Satlength=109 Nr of Repeats=6 RepeatLength=18 seed=CACTAGCCGT Num.seqs=6
Similarity=0.801170 0
CACTAGCCGTCGTCGCTT

****
Consensus:

CACTAGCCGTCGTCGCTT

>Bacillus_Fam_1908_18_1 Nr. of seq. 1 Alignment length(with gaps) = 18
Alignment score = 0.000000

GCF_000775975.1_ASM77597v1_genomic.fna_1:4349695-4349794 Satlength=100 Nr of Repeats=6 RepeatLength=18 seed=TCTTGTTTGT Num.seqs=4 Similarity=0.913580 0
TCTTGTTTGTCCTGTTTA

****
Consensus:

TCTTGTTTGTCCTGTTTA

>Bacillus_Fam_1909_18_1 Nr. of seq. 1 Alignment length(with gaps) = 18 Alignment score = 0.000000
GCF_001278705.1_ASM127870v1_genomic.fna_1:501198-501288 Satlength=91 Nr of Repeats=5 RepeatLength=18 seed=AGCCAGGCCC Num.seqs=5 Similarity=0.925926
0   AGCCAGGCCCGGATCCGC

****
Consensus:

AGCCAGGCCCGGATCCGC

>Bacillus_Fam_1910_18_1 Nr. of seq. 1 Alignment length(with gaps) = 18 Alignment score = 0.000000
GCF_001315085.1_ASM131508v1_genomic.fna_3:122565-122745 Satlength=181 Nr of Repeats=10 RepeatLength=18 seed=AAGCTCTTGG Num.seqs=10 Similarity=0.965432 0
AAGCTCTTGGTCTACATC

****
Consensus:

AAGCTCTTGGTCTACATC

>Bacillus_Fam_1911_18_1 Nr. of seq. 1 Alignment length(with gaps) = 18 Alignment score = 0.000000
GCF_001439915.1_ASM143991v1_genomic.fna_83:56856-56928 Satlength=73 Nr of Repeats=4 RepeatLength=18 seed=TGGCGTTTGA Num.seqs=4 Similarity=0.888889
0   TGGCGTTTGATTATTACC

****
Consensus:

TGGCGTTTGATTATTACC

>Bacillus_Fam_1912_18_1 Nr. of seq. 1 Alignment length(with gaps) = 18 Alignment score = 0.000000
GCF_001439915.1_ASM143991v1_genomic.fna_105:175228-175444 Satlength=217 Nr of Repeats=10 RepeatLength=18 seed=GGATCAGGAA Num.seqs=9 Similarity=0.893004 0
GGATCAGGAAACGGCGAA

****
Consensus:

GGATCAGGAAACGGCGAA

>Bacillus_Fam_1913_18_1 Nr. of seq. 1 Alignment length(with gaps) = 18 Alignment score = 0.000000
GCF_001584325.1_ASM158432v1_genomic.fna_11:35994-36075 Satlength=82 Nr of Repeats=5 RepeatLength=18 seed=CGTTCGCACC Num.seqs=3 Similarity=0.802469
0   CGTTCGCACCGTTCGCGT

****
Consensus:

CGTTCGCACCGTTCGCGT

>Bacillus_Fam_1914_18_1 Nr. of seq. 1 Alignment length(with gaps) = 18 Alignment score = 0.000000
GCF_001590785.1_ASM159078v1_genomic.fna_7:4154-4364 Satlength=211 Nr of Repeats=11 RepeatLength=18 seed=TCATCTCCTC Num.seqs=10 Similarity=0.957202
0   TCATCTCCTCCTGTTCCG

****
Consensus:

TCATCTCCTCCTGTTCCG

>Bacillus_Fam_1915_18_1 Nr. of seq. 1 Alignment length(with gaps) = 18 Alignment score = 0.000000
GCF_001591665.1_ASM159166v1_genomic.fna_33:162141-162213 Satlength=73 Nr of Repeats=4 RepeatLength=18 seed=ATCACTGACA Num.seqs=4 Similarity=0.888889
0   ATCACTGACACCATTGAT

****
Consensus:

ATCACTGACACCATTGAT

>Bacillus_Fam_1916_18_1 Nr. of seq. 1 Alignment length(with gaps) = 18 Alignment score = 0.000000
GCF_001636425.1_ASM163642v1_genomic.fna_1:588164-588470 Satlength=307 Nr of Repeats=12 RepeatLength=18 seed=TGCCTTGACC Num.seqs=11 Similarity=0.846465 0
TGCCTTGACCGCTGCCAT

****
Consensus:

TGCCTTGACCGCTGCCAT

>Bacillus_Fam_1917_18_1 Nr. of seq. 1 Alignment length(with gaps) = 18 Alignment score = 0.000000
GCF_001645555.1_ASM164555v1_genomic.fna_1:264316-264442 Satlength=127 Nr of Repeats=5 RepeatLength=18 seed=GTTGTTCTCA Num.seqs=3

Similarity=0.802469
0   GTTGTTCTCAGTTTTTGC

****
Consensus:

GTTGTTCTCAGTTTTTGC

>Bacillus_Fam_1918_18_1 Nr. of seq. 1 Alignment length(with gaps) = 18
Alignment score = 0.000000
GCF_001645685.2_ASM164568v2_genomic.fna_1:2895676-2895748 Satlength=73 Nr of Repeats=4 RepeatLength=18 seed=GCTGCATTTG Num.seqs=4
Similarity=0.851852   0
GCTGCATTTGCGGTGTCG

****
Consensus:

GCTGCATTTGCGGTGTCG

>Bacillus_Fam_1919_18_1 Nr. of seq. 1 Alignment length(with gaps) = 18
Alignment score = 0.000000
GCF_001648575.1_ASM164857v1_genomic.fna_66:20878-20950 Satlength=73 Nr of Repeats=4 RepeatLength=18 seed=TCTTCCATGT Num.seqs=4 Similarity=0.802469
0   TCTTCCATGTTTGTATCA

****
Consensus:

TCTTCCATGTTTGTATCA

>Bacillus_Fam_1920_18_1 Nr. of seq. 1 Alignment length(with gaps) = 18
Alignment score = 0.000000
GCF_001712755.1_ASM171275v1_genomic.fna_1:278428-278518 Satlength=91 Nr of Repeats=4 RepeatLength=18 seed=GTCCATGGGG Num.seqs=3
Similarity=0.802469
0   GTCCATGGGGCTGGGGCC

****
Consensus:

GTCCATGGGGCTGGGGCC

>Bacillus_Fam_1921_18_1 Nr. of seq. 1 Alignment length(with gaps) = 18
Alignment score = 0.000000
GCF_002019595.1_ASM201959v1_genomic.fna_1:4176749-4176929 Satlength=181 Nr of Repeats=8 RepeatLength=18 seed=CCTTGCATCA Num.seqs=7
Similarity=0.978836   0
CCTTGCATCATCGGCTGG

****
Consensus:

CCTTGCATCATCGGCTGG

>Bacillus_Fam_1922_18_1 Nr. of seq. 1 Alignment length(with gaps) = 18
Alignment score = 0.000000
GCF_900093775.1_EVONIK_BGLY_genomic.fna_1:1113432-1113558 Satlength=127
Nr of Repeats=5 RepeatLength=18 seed=CAGCTCCAGC Num.seqs=3
Similarity=0.950617 0
CAGCTCCAGCAGCTTGAA

****
Consensus:

CAGCTCCAGCAGCTTGAA

>Bacillus_Fam_1923_18_1 Nr. of seq. 1 Alignment length(with gaps) = 18
Alignment score = 0.000000
GCF_900093775.1_EVONIK_BGLY_genomic.fna_1:1232676-1232982 Satlength=307
Nr of Repeats=16 RepeatLength=18 seed=GAGAAAAGCA Num.seqs=15
Similarity=0.950617 0
GAGAAAAGCAAGGAGCAC

****
Consensus:

GAGAAAAGCAAGGAGCAC

>Bacillus_Fam_1924_18_1 Nr. of seq. 1 Alignment length(with gaps) = 18
Alignment score = 0.000000
GCF_900109925.1_IMG-
taxon_2651870116_annotated_assembly_genomic.fna_16:169169-169439
Satlength=271 Nr of Repeats=9 RepeatLength=18 seed=CCTGGTTGCG Num.seqs=7
Similarity=0.802840 0 CCTGGTTGCGGNGCAAAG

****
Consensus:

CCTGGTTGCGGNGCAAAG

>Bacillus_Fam_1925_17_1 Nr. of seq. 1 Alignment length(with gaps) = 17
Alignment score = 0.000000
GCF_000331575.1_ASM33157v1_genomic.fna_3:53390-53458 Satlength=69 Nr of
Repeats=4 RepeatLength=17 seed=ATGATGCAAA Num.seqs=4 Similarity=0.960784
0 ATGATGCAAAAAAGACG

****
Consensus:

ATGATGCAAAAAAGACG

>Bacillus_Fam_1926_15_1 Nr. of seq. 1 Alignment length(with gaps) = 15
Alignment score = 0.000000
GCF_000161455.1_ASM16145v1_genomic.fna_1:5573108-5573288 Satlength=181 Nr
of Repeats=12 RepeatLength=15 seed=GTAACAATGA Num.seqs=12
Similarity=0.894949 0
GTAACAATGAACCAA

****
Consensus:

GTAACAATGAACCAA

>Bacillus_Fam_1927_15_1 Nr. of seq. 1 Alignment length(with gaps) = 15 Alignment score = 0.000000
GCF_000242895.2_ASM24289v3_genomic.fna_1:2772134-2772299 Satlength=166 Nr of Repeats=10 RepeatLength=15 seed=GCAGCAGTTC Num.seqs=9 Similarity=0.829630 0
GCAGCAGTTCNCGGC

****
Consensus:

GCAGCAGTTCNCGGC

>Bacillus_Fam_1928_15_1 Nr. of seq. 1 Alignment length(with gaps) = 15 Alignment score = 0.000000
GCF_000292245.2_ASM29224v2_genomic.fna_7:15608-15698 Satlength=91 Nr of Repeats=6 RepeatLength=15 seed=GGATCTTCTT Num.seqs=6 Similarity=0.638562
0   GGATCTTCTTTNACC

****
Consensus:

GGATCTTCTTTNACC

>Bacillus_Fam_1929_15_1 Nr. of seq. 1 Alignment length(with gaps) = 15 Alignment score = 0.000000
GCF_000307875.1_BABA1.0_genomic.fna_55:97122-97182 Satlength=61 Nr of Repeats=4 RepeatLength=15 seed=AATAATGGCA Num.seqs=4 Similarity=0.896296
0   AATAATGGCAACGGC

****
Consensus:

AATAATGGCAACGGC

>Bacillus_Fam_1930_15_1 Nr. of seq. 1 Alignment length(with gaps) = 15 Alignment score = 0.000000
GCF_000331575.1_ASM33157v1_genomic.fna_1:1522649-1522889 Satlength=241 Nr of Repeats=16 RepeatLength=15 seed=TATTCGCTCC Num.seqs=16 Similarity=0.870370 0
TATTCGCTCCAGTGC

****
Consensus:

TATTCGCTCCAGTGC

>Bacillus_Fam_1931_15_1 Nr. of seq. 1 Alignment length(with gaps) = 15 Alignment score = 0.000000

GCF_000331575.1_ASM33157v1_genomic.fna_2:602668-602743 Satlength=76 Nr of Repeats=4 RepeatLength=15 seed=ATCACGAATT Num.seqs=3 Similarity=1.000000
13   CACGAATTCTATAAT

****
Consensus:

ATCACGAATTCTATA

>Bacillus_Fam_1932_15_1 Nr. of seq. 1 Alignment length(with gaps) = 15
Alignment score = 0.000000
GCF_000331575.1_ASM33157v1_genomic.fna_4:168656-168731 Satlength=76 Nr of Repeats=5 RepeatLength=15 seed=ATTTCTCCGC Num.seqs=5 Similarity=0.813333
0   ATTTCTCCGCTTGGC

****
Consensus:

ATTTCTCCGCTTGGC

>Bacillus_Fam_1933_15_1 Nr. of seq. 1 Alignment length(with gaps) = 15
Alignment score = 0.000000
GCF_000331575.1_ASM33157v1_genomic.fna_6:23405-23463 Satlength=59 Nr of Repeats=4 RepeatLength=15 seed=ATTGTAAAAA Num.seqs=3 Similarity=1.000000
0   ATTGTAAAAATAAAT

****
Consensus:

ATTGTAAAAATAAAT

>Bacillus_Fam_1934_15_1 Nr. of seq. 1 Alignment length(with gaps) = 15
Alignment score = 0.000000
GCF_000374345.1_ASM37434v1_genomic.fna_6:64384-64486 Satlength=103 Nr of Repeats=4 RepeatLength=15 seed=TTCAAGTTCA Num.seqs=3 Similarity=1.000000
0   TTCAAGTTCAAGCAA

****
Consensus:

TTCAAGTTCAAGCAA

>Bacillus_Fam_1935_15_1 Nr. of seq. 1 Alignment length(with gaps) = 15
Alignment score = 0.000000
GCF_000474275.1_Bmar1.0_genomic.fna_8:95892-95952 Satlength=61 Nr of Repeats=4 RepeatLength=15 seed=AGATTGAGTA Num.seqs=4 Similarity=1.000000
0   AGATTGAGTAAACAG

****
Consensus:

AGATTGAGTAAACAG

>Bacillus_Fam_1936_15_1 Nr. of seq. 1 Alignment length(with gaps) = 15
Alignment score = 0.000000
GCF_000612625.1_JCE_genomic.fna_2:32740-32845 Satlength=106 Nr of Repeats=7 RepeatLength=15 seed=GCTGGTGCCT Num.seqs=7 Similarity=0.898413
0   GCTGGTGCCTTCGCT

****
Consensus:

GCTGGTGCCTTCGCT

>Bacillus_Fam_1937_15_1 Nr. of seq. 1 Alignment length(with gaps) = 15
Alignment score = 0.000000
GCF_000759675.1_ASM75967v1_genomic.fna_19:633-873 Satlength=241 Nr of Repeats=16 RepeatLength=15 seed=TTAATTGGTG Num.seqs=16 Similarity=0.873333
0   TTAATTGGTGCTNCT

****
Consensus:

TTAATTGGTGCTNCT

>Bacillus_Fam_1938_15_1 Nr. of seq. 1 Alignment length(with gaps) = 15
Alignment score = 0.000000
GCF_000759675.1_ASM75967v1_genomic.fna_19:225767-226489 Satlength=723 Nr of Repeats=13 RepeatLength=15 seed=TGAACTTCTT Num.seqs=10 Similarity=0.952593 0
TGAACTTCTTATTTC

****
Consensus:

TGAACTTCTTATTTC

>Bacillus_Fam_1939_15_1 Nr. of seq. 1 Alignment length(with gaps) = 15
Alignment score = 0.000000
GCF_000787375.1_ASM78737v1_genomic.fna_1:295361-295436 Satlength=76 Nr of Repeats=4 RepeatLength=15 seed=TTCATCGGTT Num.seqs=3 Similarity=0.881481
5   GCTGGTTCATCGGTT

****
Consensus:

TTCATCGGTTGCTGG

>Bacillus_Fam_1940_15_1 Nr. of seq. 1 Alignment length(with gaps) = 15
Alignment score = 0.000000
GCF_000812025.2_ASM81202v2_genomic.fna_59:10143-10233 Satlength=91 Nr of Repeats=5 RepeatLength=15 seed=ATGCATGAGA Num.seqs=4 Similarity=0.911111
0   ATGCATGAGAACACC

****
Consensus:

ATGCATGAGAACACC

>Bacillus_Fam_1941_15_1 Nr. of seq. 1 Alignment length(with gaps) = 15 Alignment score = 0.000000 GCF_001420715.1_ASM142071v1_genomic.fna_24:95921-96146 Satlength=226 Nr of Repeats=14 RepeatLength=15 seed=AGACGTTCTA Num.seqs=13 Similarity=0.831339 0 AGACGTTCTAGTAGA

****
Consensus:

AGACGTTCTAGTAGA

>Bacillus_Fam_1942_15_1 Nr. of seq. 1 Alignment length(with gaps) = 15 Alignment score = 0.000000 GCF_001590785.1_ASM159078v1_genomic.fna_7:40479-40902 Satlength=424 Nr of Repeats=8 RepeatLength=15 seed=TCTCCGTGCT Num.seqs=6 Similarity=0.875556 0 TCTCCGTGCTCATGG

****
Consensus:

TCTCCGTGCTCATGG

>Bacillus_Fam_1943_15_1 Nr. of seq. 1 Alignment length(with gaps) = 15 Alignment score = 0.000000 GCF_001591665.1_ASM159166v1_genomic.fna_29:1963-2053 Satlength=91 Nr of Repeats=4 RepeatLength=15 seed=GAAGTGTTCC Num.seqs=3 Similarity=1.000000 0 GAAGTGTTCCGTCTC

****
Consensus:

GAAGTGTTCCGTCTC

>Bacillus_Fam_1944_15_1 Nr. of seq. 1 Alignment length(with gaps) = 15 Alignment score = 0.000000 GCF_001591805.1_ASM159180v1_genomic.fna_17:30976-31126 Satlength=151 Nr of Repeats=10 RepeatLength=15 seed=CAACCGATCC Num.seqs=10 Similarity=0.899259 0 CAACCGATCCAGGAC

****
Consensus:

CAACCGATCCAGGAC

>Bacillus_Fam_1945_15_1 Nr. of seq. 1 Alignment length(with gaps) = 15 Alignment score = 0.000000 GCF_001636325.1_ASM163632v1_genomic.fna_1:2446901-2447021 Satlength=121 Nr of Repeats=7 RepeatLength=15 seed=AATCCTGGAC Num.seqs=6

Similarity=0.946667 0
AATCCTGGACCTCCA

****
Consensus:

AATCCTGGACCTCCA

>Bacillus_Fam_1946_15_1 Nr. of seq. 1 Alignment length(with gaps) = 15
Alignment score = 0.000000
GCF_002019595.1_ASM201959v1_genomic.fna_1:1565861-1565951 Satlength=91 Nr of Repeats=6 RepeatLength=15 seed=CCGGGGAAAC Num.seqs=6
Similarity=0.946667 0
CCGGGGAAACCGCCA

****
Consensus:

CCGGGGAAACCGCCA

>Bacillus_Fam_1947_15_1 Nr. of seq. 1 Alignment length(with gaps) = 15
Alignment score = 0.000000
GCF_002019635.1_ASM201963v1_genomic.fna_1:1407148-1407343 Satlength=196 Nr of Repeats=12 RepeatLength=15 seed=CACTGATGAT Num.seqs=11
Similarity=0.938586 0
CACTGATGATCCGGG

****
Consensus:

CACTGATGATCCGGG

>Bacillus_Fam_1948_15_1 Nr. of seq. 1 Alignment length(with gaps) = 15
Alignment score = 0.000000
GCF_900156875.1_PRJEB18969_genomic.fna_11:2364715-2365012 Satlength=298 Nr of Repeats=8 RepeatLength=15 seed=TGCATCATTG Num.seqs=7
Similarity=0.718254 0
TGCATCATTGGTGAT

****
Consensus:

TGCATCATTGGTGAT

>Bacillus_Fam_1949_14_1 Nr. of seq. 1 Alignment length(with gaps) = 14
Alignment score = 0.000000
GCF_000331575.1_ASM33157v1_genomic.fna_1:1707310-1707394 Satlength=85 Nr of Repeats=6 RepeatLength=14 seed=ATTTTGTAGT Num.seqs=6
Similarity=0.917460
0 ATTTTGTAGTCGTG

****
Consensus:

ATTTTGTAGTCGTG

>Bacillus_Fam_1950_13_1 Nr. of seq. 1 Alignment length(with gaps) = 13 Alignment score = 0.000000
GCF_000331575.1_ASM33157v1_genomic.fna_3:37579-37631 Satlength=53 Nr of Repeats=4 RepeatLength=13 seed=TACTAGGAGA Num.seqs=4 Similarity=0.948718
0 TACTAGGAGACAG

****
Consensus:

TACTAGGAGACAG

>Bacillus_Fam_1951_13_1 Nr. of seq. 1 Alignment length(with gaps) = 13 Alignment score = 0.000000
GCF_000430765.1_ASM43076v1_genomic.fna_6:130918-130983 Satlength=66 Nr of Repeats=5 RepeatLength=13 seed=AGTGTTCAGG Num.seqs=5 Similarity=1.000000
0 AGTGTTCAGGATA

****
Consensus:

AGTGTTCAGGATA

>Bacillus_Fam_1952_12_1 Nr. of seq. 1 Alignment length(with gaps) = 12 Alignment score = 0.000000
GCF_000161455.1_ASM16145v1_genomic.fna_1:175339-175456 Satlength=118 Nr of Repeats=5 RepeatLength=12 seed=CGTGATGGTA Num.seqs=3 Similarity=0.851852
0 CGTGATGGTAAT

****
Consensus:

CGTGATGGTAAT

>Bacillus_Fam_1953_12_1 Nr. of seq. 1 Alignment length(with gaps) = 12 Alignment score = 0.000000
GCF_000242895.2_ASM24289v3_genomic.fna_1:2189079-2189571 Satlength=493 Nr of Repeats=39 RepeatLength=12 seed=CGTTCAAGCC Num.seqs=37 Similarity=0.830831 0
CGTTCAAGCCGT

****
Consensus:

CGTTCAAGCCGT

>Bacillus_Fam_1954_12_1 Nr. of seq. 1 Alignment length(with gaps) = 12 Alignment score = 0.000000
GCF_000307875.1_BABA1.0_genomic.fna_97:105197-105245 Satlength=49 Nr of Repeats=4 RepeatLength=12 seed=CAAATATGAT Num.seqs=4 Similarity=1.000000
0 CAAATATGATGC

****
Consensus:

CAAATATGATGC

>Bacillus_Fam_1955_12_1 Nr. of seq. 1 Alignment length(with gaps) = 12
Alignment score = 0.000000
GCF_000331575.1_ASM33157v1_genomic.fna_1:1702114-1702162 Satlength=49 Nr of Repeats=4 RepeatLength=12 seed=CGTTGTATTA Num.seqs=4
Similarity=1.000000
0   CGTTGTATTATT

****
Consensus:

CGTTGTATTATT

>Bacillus_Fam_1956_12_1 Nr. of seq. 1 Alignment length(with gaps) = 12
Alignment score = 0.000000
GCF_000331575.1_ASM33157v1_genomic.fna_4:145562-145634 Satlength=73 Nr of Repeats=6 RepeatLength=12 seed=ATGCAAACTC Num.seqs=6 Similarity=0.731624
0   ATGCAAACTCTA

****
Consensus:

ATGCAAACTCTA

>Bacillus_Fam_1957_12_1 Nr. of seq. 1 Alignment length(with gaps) = 12
Alignment score = 0.000000
GCF_000380245.2_ASM38024v2_genomic.fna_12:2893696-2893756 Satlength=61 Nr of Repeats=5 RepeatLength=12 seed=CTTTTTTACA Num.seqs=5
Similarity=1.000000 0
CTTTTTTACAAC

****
Consensus:

CTTTTTTACAAC

>Bacillus_Fam_1958_12_1 Nr. of seq. 1 Alignment length(with gaps) = 12
Alignment score = 0.000000
GCF_000429725.1_ASM42972v1_genomic.fna_2:378953-379145 Satlength=193 Nr of Repeats=12 RepeatLength=12 seed=ACGACATGGA Num.seqs=8
Similarity=0.773810
0   ACGACATGGACA

****
Consensus:

ACGACATGGACA

>Bacillus_Fam_1959_12_1 Nr. of seq. 1 Alignment length(with gaps) = 12
Alignment score = 0.000000
GCF_000430765.1_ASM43076v1_genomic.fna_4:178616-178796 Satlength=181 Nr of Repeats=9 RepeatLength=12 seed=TGGTTCCATT Num.seqs=7 Similarity=0.883598
0   TGGTTCCATTGC

****
Consensus:

TGGTTCCATTGC

>Bacillus_Fam_1960_12_1 Nr. of seq. 1 Alignment length(with gaps) = 12
Alignment score = 0.000000
GCF_000751775.1_A1A_genomic.fna_68:68366-68414 Satlength=49 Nr of Repeats=4 RepeatLength=12 seed=TCTTTGATTC Num.seqs=4 Similarity=0.944444
0   TCTTTGATTCCT

****
Consensus:

TCTTTGATTCCT

>Bacillus_Fam_1961_12_1 Nr. of seq. 1 Alignment length(with gaps) = 12
Alignment score = 0.000000
GCF_000752035.1_Bacillus_rubiinfantis_genomic.fna_13:49986-50062 Satlength=77 Nr of Repeats=5 RepeatLength=12 seed=ATTGGTGCCT Num.seqs=3 Similarity=1.000000 0
ATTGGTGCCTTT

****
Consensus:

ATTGGTGCCTTT

>Bacillus_Fam_1962_12_1 Nr. of seq. 1 Alignment length(with gaps) = 12
Alignment score = 0.000000
GCF_001636345.1_ASM163634v1_genomic.fna_1:3481278-3481350 Satlength=73 Nr of Repeats=6 RepeatLength=12 seed=GGGCGTTCTT Num.seqs=6 Similarity=1.000000 0
GGGCGTTCTTCG

****
Consensus:

GGGCGTTCTTCG

>Bacillus_Fam_1963_11_1 Nr. of seq. 1 Alignment length(with gaps) = 11
Alignment score = 0.000000
GCF_000331575.1_ASM33157v1_genomic.fna_1:1378343-1378409 Satlength=67 Nr of Repeats=6 RepeatLength=11 seed=GCACTATTTT Num.seqs=6 Similarity=0.935354
0   GCACTATTTTC

****
Consensus:

GCACTATTTTC

>Bacillus_Fam_1964_11_1 Nr. of seq. 1 Alignment length(with gaps) = 11
Alignment score = 0.000000
GCF_000831065.1_ASM83106v1_genomic.fna_1:2961625-2961669 Satlength=45 Nr of Repeats=4 RepeatLength=11 seed=AGGGGCTTAG Num.seqs=4 Similarity=1.000000
0   AGGGGCTTAGA

****
Consensus:

AGGGGCTTAGA

>Bacillus_Fam_1965_11_1 Nr. of seq. 1 Alignment length(with gaps) = 11
Alignment score = 0.000000
GCF_001636345.1_ASM163634v1_genomic.fna_1:595400-595488 Satlength=89 Nr of Repeats=8 RepeatLength=11 seed=CCAAAAACCG Num.seqs=8 Similarity=1.000000
0   CCAAAAACCGC

****
Consensus:

CCAAAAACCGC

>Bacillus_Fam_1966_10_1 Nr. of seq. 1 Alignment length(with gaps) = 10
Alignment score = 0.000000
GCF_000331575.1_ASM33157v1_genomic.fna_2:525802-525852 Satlength=51 Nr of Repeats=4 RepeatLength=10 seed=TTGTCGCGGA Num.seqs=3 Similarity=1.000000
0   TTGTCGCGGA

****
Consensus:

TTGTCGCGGA
