# Supplementary material for: Tandem Repeats in Bacillus: Unique Features and Taxonomic Distribution
Source: Int J Mol Sci. 2021 May 20;22(10):5373. doi: 10.3390/ijms22105373 (PMC8161180; doi:10.3390/ijms22105373)
Supplement: Supplementary file 1 [file ijms-22-05373-s001.zip › Table S4.List of Bacillus tandem repeat families.pdf]

```
The program is running under the following parameters:
   Output:
      Prefix of the family name Bacillus
      Circular permutations are allowed
   Reverse sequences are considered.
   Sequences are aligned while similarity: 0.600000
   Alignment score: Normalized respect to the length and number of
sequences.
   Alignment parameters:
      opening gap: -3.000000
      extensio gap: -1.000000
      extreme gaph: -0.500000

 ************************************************************
There are 1966 alignments:

>Bacillus_Fam_1_60_41  Nr. of seq. 41 Alignment length(with gaps) = 64
Alignment score = 0.721735
 TCCGCCTTTTTngCCgATtTCtTGaTAGAATTCTTTgTCATGgTTtTnnGcaGTnGCTTC
>Bacillus_Fam_2_52_36  Nr. of seq. 36 Alignment length(with gaps) = 57
Alignment score = 0.678177
 CTTAACgTAGACGngCTCTATGTgAAGgAAGgcCnTCntTTTTnCnnGCTTc
>Bacillus_Fam_3_50_31  Nr. of seq. 31 Alignment length(with gaps) = 64
Alignment score = 0.605623
 gAATCnCGGGGTTGCGaGCnGAAAAAGaGGAGAAAgCCCGAGCAAAcGcn
>Bacillus_Fam_4_52_27  Nr. of seq. 27 Alignment length(with gaps) = 53
Alignment score = 0.745847
 AAgGGAATCAaACAAcgCtTTTCaTTCCCTTTgttggGCTTTtGGCATgAGA
>Bacillus_Fam_5_52_25  Nr. of seq. 25 Alignment length(with gaps) = 56
Alignment score = 0.706369
 GTTAATGTGaAGATACgGAgGCcAAACcttGgAGTAtCTGCACAAAGAGggG
>Bacillus_Fam_6_20_21  Nr. of seq. 21 Alignment length(with gaps) = 22
Alignment score = 0.672366
 TGCTAATAAAanngAAAAGt
>Bacillus_Fam_7_52_18  Nr. of seq. 18 Alignment length(with gaps) = 60
Alignment score = 0.620516
 ATCGTACCCTcgnAAACcGaaAAaCGATnTgggAGGGTAAGCAAanGcnnGA
>Bacillus_Fam_8_52_17  Nr. of seq. 17 Alignment length(with gaps) = 56
Alignment score = 0.680278
 gacGACGAGAAnnGGTcTCGTCGCCAAAAAAngGaGTTTtnCGnCtCAAAAn
>Bacillus_Fam_9_52_16  Nr. of seq. 16 Alignment length(with gaps) = 60
Alignment score = 0.601759
 CCTtTGATTCCCTTTncGGCTtTtATTcaatgGCTTTtGgcaTCATTGcnGn
>Bacillus_Fam_10_20_16  Nr. of seq. 16 Alignment length(with gaps) = 22
Alignment score = 0.662626
 tCGCCAATAAAAttanATTT
>Bacillus_Fam_11_51_15  Nr. of seq. 15 Alignment length(with gaps) = 56
Alignment score = 0.625454
 TTTTCATTACcTATtcCnntTTaTTCGCACCcTAATtcnncaCAgCnCgGc
>Bacillus_Fam_12_52_15  Nr. of seq. 15 Alignment length(with gaps) = 54
Alignment score = 0.703939
 TTTTGgCGACGAGaCccntTCTCGTCACCATTTTgaggtGAgAAAtGCtCaa
```

>Bacillus_Fam_13_21_15 Nr. of seq. 15 Alignment length(with gaps) = 24
Alignment score = 0.642196
TTACCGTaGATAAATnGcngt
>Bacillus_Fam_14_21_15 Nr. of seq. 15 Alignment length(with gaps) = 23
Alignment score = 0.651484
AATCGCGGATAGAgcnccnAn
>Bacillus_Fam_15_52_14 Nr. of seq. 14 Alignment length(with gaps) = 54
Alignment score = 0.680301
ATGAACGACCgcGggCgcGGnTTTTCttCCGgcgCGGGCTTTCATTCccCtT
>Bacillus_Fam_16_52_14 Nr. of seq. 14 Alignment length(with gaps) = 54
Alignment score = 0.664021
ATGAGAACCGAAAgAGgtccAGcGnaAgcaGgaAaCGGTCtTCATaAagGgg
>Bacillus_Fam_17_12_14 Nr. of seq. 14 Alignment length(with gaps) = 14
Alignment score = 0.607535
CTTTtTCtTTtT
>Bacillus_Fam_18_52_13 Nr. of seq. 13 Alignment length(with gaps) = 57
Alignment score = 0.612086
GTTCATGAGTAAGTAAAAnAGcAAAAAnanGnAAAntagTtACTCATAaagg
>Bacillus_Fam_19_53_13 Nr. of seq. 13 Alignment length(with gaps) = 57
Alignment score = 0.601589
TGctGCCACTTTtacCnGaGatGgATcCtcaTTTGTGGCGTCATTCgcngtAG
>Bacillus_Fam_20_52_13 Nr. of seq. 13 Alignment length(with gaps) = 53
Alignment score = 0.740364
ATGAAGACnTcAGTGACgAggAAaaAGGAGgAGAGAaGTCCTCATcGccGTt
>Bacillus_Fam_21_36_13 Nr. of seq. 13 Alignment length(with gaps) = 36
Alignment score = 0.755935
TgTTATCCATnATtGGCATnATgTTtGGcatTTGaT
>Bacillus_Fam_22_53_12 Nr. of seq. 12 Alignment length(with gaps) = 56
Alignment score = 0.655032
GTTCCCTCTaTTTcTCnTTTTTCgnntTTTgAGGGCACGATtACGCncTGATC
>Bacillus_Fam_23_52_12 Nr. of seq. 12 Alignment length(with gaps) = 55
Alignment score = 0.736639
TTTTGTCCTTTTGACaGctTCAAAAgGACATTTCGngCCCgGATGCagCncG
>Bacillus_Fam_24_21_12 Nr. of seq. 12 Alignment length(with gaps) = 23
Alignment score = 0.627141
CGCcCATTGAAcncnnAAATT
>Bacillus_Fam_25_20_12 Nr. of seq. 12 Alignment length(with gaps) = 21
Alignment score = 0.723545
tTTTTATAGCGAAAATtTan
>Bacillus_Fam_26_53_11 Nr. of seq. 11 Alignment length(with gaps) = 58
Alignment score = 0.666458
nGtTGGTCGACATGATCAtGgGnAAAAAAGGncaAGAACcTGTCGATGAaGGn
>Bacillus_Fam_27_39_11 Nr. of seq. 11 Alignment length(with gaps) = 40
Alignment score = 0.799394
CTACATTTTCTTTcTTcACTTGcGGcGAtACTATaTTtC
>Bacillus_Fam_28_21_11 Nr. of seq. 11 Alignment length(with gaps) = 25
Alignment score = 0.604364
TTTACTCGCCAATTcGnGngn
>Bacillus_Fam_29_20_11 Nr. of seq. 11 Alignment length(with gaps) = 22
Alignment score = 0.720386
TTTATTGGCGATtTTcaaAT
>Bacillus_Fam_30_18_11 Nr. of seq. 11 Alignment length(with gaps) = 19
Alignment score = 0.651675
CCtGTTACTCCnGTTGga

>Bacillus_Fam_31_51_10 Nr. of seq. 10 Alignment length(with gaps) = 56
Alignment score = 0.632407
gtTGTCCAATAGaAGcggTCTAaTGGACAAgAAtctgGtnaaAagcGAAAA
>Bacillus_Fam_32_52_10 Nr. of seq. 10 Alignment length(with gaps) = 56
Alignment score = 0.659061
TTTcGTTACCcggCaCaCncatTTTcCTCCTTCtcGGTAAcCAAtCgCTctC
>Bacillus_Fam_33_52_10 Nr. of seq. 10 Alignment length(with gaps) = 55
Alignment score = 0.634747
gTGTaTCATACgaaggCAATGACACgtGAgAAAGtaGaaGaaacgnAATAAa
>Bacillus_Fam_34_52_10 Nr. of seq. 10 Alignment length(with gaps) = 54
Alignment score = 0.660905
TGTCCGAAgtngCgCcAAcTTCGGACTCatTCtCtCngaTTTctgCTtCTcC
>Bacillus_Fam_35_52_10 Nr. of seq. 10 Alignment length(with gaps) = 53
Alignment score = 0.701887
TTGATGGTtCCgAaCtCagaGATAAGTGCCAtCAAAacgGAaaatCCnCcGn
>Bacillus_Fam_36_20_10 Nr. of seq. 10 Alignment length(with gaps) = 26
Alignment score = 0.611966
GTTTTGGCTAtTTTtCaAgg
>Bacillus_Fam_37_21_10 Nr. of seq. 10 Alignment length(with gaps) = 24
Alignment score = 0.601389
TTTGATAGGAAAnncaaaAAn
>Bacillus_Fam_38_15_10 Nr. of seq. 10 Alignment length(with gaps) = 15
Alignment score = 0.786667
CCaGAaCCgTTgCCa
>Bacillus_Fam_39_81_9 Nr. of seq. 9 Alignment length(with gaps) = 83
Alignment score = 0.715640

AgCAAATTCAGtCCgGAAACgATtTTaacagAgCTGGATAAgTTcACaACnTCaTTcATGaGnAAAGTgGAcG
aaATcGCn
>Bacillus_Fam_40_52_9 Nr. of seq. 9 Alignment length(with gaps) = 58
Alignment score = 0.648467
tCTTTTGTCTTTTTGAnncCTCCAAAAGGACAaTTtGcnCCtggtCCgGcTc
>Bacillus_Fam_41_51_9 Nr. of seq. 9 Alignment length(with gaps) = 57
Alignment score = 0.622157
TTTTTCATTACcTATcCncnnTTTTTCGCACcnTAATTTggcCtgCtcggC
>Bacillus_Fam_42_53_9 Nr. of seq. 9 Alignment length(with gaps) = 56
Alignment score = 0.615245
CanaCCAACAtCcCTcncAtAATcCatTCTCaTTGGnctGaTTActCCcTTTT
>Bacillus_Fam_43_53_9 Nr. of seq. 9 Alignment length(with gaps) = 56
Alignment score = 0.632275
nGCttGCATAAGGGTATCtTTCcgCntGAaaGaTgCCCTTATngaGGaGgAAn
>Bacillus_Fam_44_51_9 Nr. of seq. 9 Alignment length(with gaps) = 54
Alignment score = 0.728567
GTTTTGCTGATAGTAAAtCCtTTTTATCGTCAGCAAATcgAGnaTcaCgnC
>Bacillus_Fam_45_52_9 Nr. of seq. 9 Alignment length(with gaps) = 53
Alignment score = 0.615129
gATGAAgGACAtTTcGccgttngcangCCnntgtTTTGTCCTTCATCacCCc
>Bacillus_Fam_46_36_9 Nr. of seq. 9 Alignment length(with gaps) = 36
Alignment score = 0.777263
taCACCAGTggCACCgGTAGgtCCaGTAtctCCAGT
>Bacillus_Fam_47_21_9 Nr. of seq. 9 Alignment length(with gaps) = 24
Alignment score = 0.626157
tCACGAGTAAAnnGCtcAAAc

>Bacillus_Fam_48_21_9 Nr. of seq. 9 Alignment length(with gaps) = 23
Alignment score = 0.644525
ATTCGCTAATAAACnnncaAg
>Bacillus_Fam_49_21_9 Nr. of seq. 9 Alignment length(with gaps) = 22
Alignment score = 0.671296
tTACTCGTGAaTTtngtGCat
>Bacillus_Fam_50_18_9 Nr. of seq. 9 Alignment length(with gaps) = 21
Alignment score = 0.615520
cAnAAGAAAgnAGAAGAa
>Bacillus_Fam_51_21_9 Nr. of seq. 9 Alignment length(with gaps) = 21
Alignment score = 0.772046
ACTCGTGAgTTTcgcnCnTTT
>Bacillus_Fam_52_18_9 Nr. of seq. 9 Alignment length(with gaps) = 18
Alignment score = 0.911008
GAGCTTTAGCTGCTTCtT
>Bacillus_Fam_53_18_9 Nr. of seq. 9 Alignment length(with gaps) = 18
Alignment score = 0.913580
CCTTGGTTGTcGTTTCCg
>Bacillus_Fam_54_114_8 Nr. of seq. 8 Alignment length(with gaps) = 115
Alignment score = 0.790890

TCGCTTACATtGCATTGGCCATaCTtATTnnaACCCACAGCcacCACcGTaCCGTCcGATTTAAGnCCgATgG
TATGannacAaCCCGCCGCnAcCGCCACAATAtCGcGCCAG
>Bacillus_Fam_55_52_8 Nr. of seq. 8 Alignment length(with gaps) = 59
Alignment score = 0.632062
TCATGAGGACAAAgTCGgacGAAAacCaaGaAAgttTGTCCTCATAgAGGGt
>Bacillus_Fam_56_52_8 Nr. of seq. 8 Alignment length(with gaps) = 58
Alignment score = 0.632389
nagTTTTGTCCTTCATCacccTTATGAAGGACACTTCtCtCtcTTTtCaCtC
>Bacillus_Fam_57_52_8 Nr. of seq. 8 Alignment length(with gaps) = 57
Alignment score = 0.671261
GGACAAAATgaaGngtCAAtCAGgAAAAAcTGTCCAATAGAACGGcTCTCaT
>Bacillus_Fam_58_52_8 Nr. of seq. 8 Alignment length(with gaps) = 57
Alignment score = 0.619779
TTTCTTGACAgCTTTgcgCTcttcaccctgAAAcCTGTCACAATAACggCTT
>Bacillus_Fam_59_55_8 Nr. of seq. 8 Alignment length(with gaps) = 57
Alignment score = 0.625731
CGCCACAAAcnAggAtcCnaTCacngGnAAAAGTGGCagCACTACnGcgGAATGA
>Bacillus_Fam_60_52_8 Nr. of seq. 8 Alignment length(with gaps) = 56
Alignment score = 0.673044
ATGGCACTTATCcGGgAGAAtAGAACCaTCAAaaaGAagaATnctCaGGTTG
>Bacillus_Fam_61_52_8 Nr. of seq. 8 Alignment length(with gaps) = 56
Alignment score = 0.638924
gnAtCTTGGTCGtCAAAGcGAGnGAATGaAGCCCAAGATnngaAtcnaAAAg
>Bacillus_Fam_62_52_8 Nr. of seq. 8 Alignment length(with gaps) = 55
Alignment score = 0.629437
GGTctTAGTcAagccgACTAanACCcTCTgAnccggAaAAAAggAAaCAGAg
>Bacillus_Fam_63_52_8 Nr. of seq. 8 Alignment length(with gaps) = 54
Alignment score = 0.661155
gATGAAGGACtTTTCtcTGGaTcTctTnCncctTTTTGTCCTTCAgnACCCg
>Bacillus_Fam_64_45_8 Nr. of seq. 8 Alignment length(with gaps) = 45
Alignment score = 0.881481
TTTTTGcTCTTCTTGTTGTTTACGAGCTTGTTCaTCAGCtTGaCG

```
>Bacillus_Fam_65_26_8  Nr. of seq. 8 Alignment length(with gaps) = 26
Alignment score = 0.630495
 ATcTGAGCCAGGCtCAAAaggngtgn
>Bacillus_Fam_66_21_8  Nr. of seq. 8 Alignment length(with gaps) = 24
Alignment score = 0.630208
 TTCGCAAATAAAACnGgnaAa
>Bacillus_Fam_67_21_8  Nr. of seq. 8 Alignment length(with gaps) = 21
Alignment score = 0.806689
 TGaAGaAACAGAAAaaCCAGG
>Bacillus_Fam_68_52_7  Nr. of seq. 7 Alignment length(with gaps) = 54
Alignment score = 0.639624
 CGGTCACttATAGCCtTcATtaGTgACCACTTTtTCnATTtnaaCnagATTt
>Bacillus_Fam_69_52_7  Nr. of seq. 7 Alignment length(with gaps) = 54
Alignment score = 0.627572
 tGACagCTTTTCgnCTTttCCnnCcAAACCTGTCcGAAgTTGgnnCTncTTC
>Bacillus_Fam_70_52_7  Nr. of seq. 7 Alignment length(with gaps) = 54
Alignment score = 0.638448
 aAaatGGaAAATgGTGTCgcGaAAAggngAGAAACACCCCtcTaaAaTCCAG
>Bacillus_Fam_71_52_7  Nr. of seq. 7 Alignment length(with gaps) = 54
Alignment score = 0.641387
 TGTCTGAAGTaGCCTnGnGTTCGGACAGcTTTgaTtgtTTnnaaGcnAAAGC
>Bacillus_Fam_72_52_7  Nr. of seq. 7 Alignment length(with gaps) = 52
Alignment score = 0.731380
 CATAGgCctTCTATGATtcAGTTGCcgaaGCgAAAACAAGGAGnAAGTgAAT
>Bacillus_Fam_73_20_7  Nr. of seq. 7 Alignment length(with gaps) = 22
Alignment score = 0.655123
 TTTTATAGCGAntTTTnCnT
>Bacillus_Fam_74_21_7  Nr. of seq. 7 Alignment length(with gaps) = 22
Alignment score = 0.698413
 TTTATTTATCAcCGAnCnCnn
>Bacillus_Fam_75_22_7  Nr. of seq. 7 Alignment length(with gaps) = 22
Alignment score = 0.622655
 TTTACTCGCcAAtTTngnnAcn
>Bacillus_Fam_76_21_7  Nr. of seq. 7 Alignment length(with gaps) = 21
Alignment score = 0.702948
 TTACTTAACAnnTtttcAAAa
>Bacillus_Fam_77_15_7  Nr. of seq. 7 Alignment length(with gaps) = 16
Alignment score = 0.610119
 cCtGGtTCTTCnGTn
>Bacillus_Fam_78_12_7  Nr. of seq. 7 Alignment length(with gaps) = 12
Alignment score = 0.830688
 CGGCGAGtAGAa
>Bacillus_Fam_79_12_7  Nr. of seq. 7 Alignment length(with gaps) = 12
Alignment score = 0.640212
 nTgTTGAtnTTG
>Bacillus_Fam_80_52_6  Nr. of seq. 6 Alignment length(with gaps) = 58
Alignment score = 0.624904
 CGGTCACTCATAaGGTagATGAGAGCCCGngAAtaGcaAAAAGAAGnaAGaG
>Bacillus_Fam_81_52_6  Nr. of seq. 6 Alignment length(with gaps) = 57
Alignment score = 0.684016
 GGAGAAAccGaAGcaAAAGTGTCCTTCATAAAGGnGATGAAGGACAAAAcaA
>Bacillus_Fam_82_52_6  Nr. of seq. 6 Alignment length(with gaps) = 57
Alignment score = 0.644444
 aaAAGGGcAGTCATGTATnCcGGGAgcgtTncGGgaTACATGAGTGGgTcGa
```

>Bacillus_Fam_83_52_6 Nr. of seq. 6 Alignment length(with gaps) = 56
Alignment score = 0.775397
AACCaCTTTTCGCGaAATCCnCATCATTTTcGGCTCATAGaACTCTTCTATG
>Bacillus_Fam_84_53_6 Nr. of seq. 6 Alignment length(with gaps) = 56
Alignment score = 0.659127
cTTTGCTTACCCTcCccAncCgtTTTTTCGcTcTTGCTGGGTAcGATTCCTCC
>Bacillus_Fam_85_52_6 Nr. of seq. 6 Alignment length(with gaps) = 55
Alignment score = 0.734949
CAcTCAACGAAGGTcATCATAAGcAAGCAATGCTaCCCCAAAACCAAAcCcn
>Bacillus_Fam_86_53_6 Nr. of seq. 6 Alignment length(with gaps) = 55
Alignment score = 0.654545
CTGatTTTTCtGTTCGTTCGGGGtTCTATaCacCTTATTGAaGACTCTTTCgn
>Bacillus_Fam_87_52_6 Nr. of seq. 6 Alignment length(with gaps) = 53
Alignment score = 0.628512
TCTACGTGACCcaacgtCctctTTTTcnTCcgnTtCGGTCaCGTAGAACCtC
>Bacillus_Fam_88_52_6 Nr. of seq. 6 Alignment length(with gaps) = 53
Alignment score = 0.782809
GTCAATCGTTACCGTTTCACAGaAGaTCAaCtCATTAcGGGCACGATTCAGt
>Bacillus_Fam_89_53_6 Nr. of seq. 6 Alignment length(with gaps) = 53
Alignment score = 0.780503
aGnCAAaAATGTGTGTCCAAGGcGaaAGTGGAGACAcATaAcGAaGgAAAAAc
>Bacillus_Fam_90_42_6 Nr. of seq. 6 Alignment length(with gaps) = 42
Alignment score = 0.812169
TCTTCCACTTGTGGTTCtACTACcACTTGCTGCAcTGGtTtT
>Bacillus_Fam_91_20_6 Nr. of seq. 6 Alignment length(with gaps) = 22
Alignment score = 0.625758
aATTTAATTGCGATTCtCgg
>Bacillus_Fam_92_21_6 Nr. of seq. 6 Alignment length(with gaps) = 22
Alignment score = 0.633333
AActngaAAnTTGGCGGAATA
>Bacillus_Fam_93_21_6 Nr. of seq. 6 Alignment length(with gaps) = 22
Alignment score = 0.603030
ATCGCGGAaAGaagtGcCGGA
>Bacillus_Fam_94_21_6 Nr. of seq. 6 Alignment length(with gaps) = 21
Alignment score = 0.647619
ATTcCGCCAAACtnaggATTa
>Bacillus_Fam_95_20_6 Nr. of seq. 6 Alignment length(with gaps) = 20
Alignment score = 0.731111
ATACTCTTCACtTTcgaGAA
>Bacillus_Fam_96_15_6 Nr. of seq. 6 Alignment length(with gaps) = 15
Alignment score = 0.751111
TAAAaAAGAAAAaaC
>Bacillus_Fam_97_12_6 Nr. of seq. 6 Alignment length(with gaps) = 12
Alignment score = 0.807407
GGTTCTGGcTTT
>Bacillus_Fam_98_52_5 Nr. of seq. 5 Alignment length(with gaps) = 61
Alignment score = 0.610929
tttctnctctGnTTcCtatTtTcggcttCatgaagngctcatgGcgaccatT
>Bacillus_Fam_99_61_5 Nr. of seq. 5 Alignment length(with gaps) = 61
Alignment score = 0.663934
CCtgTTtgCATtGcaCCgtttcCgTTnAAGTAgTACCATgttCCanttAntTGctTgCCAa
>Bacillus_Fam_100_52_5 Nr. of seq. 5 Alignment length(with gaps) = 59
Alignment score = 0.628531
AGAGtGtgcCTtGTGACCAAAaGtAAgnTngGAatcCATagAAGTGGTCACA

>Bacillus_Fam_101_51_5 Nr. of seq. 5 Alignment length(with gaps) = 58
Alignment score = 0.637356
GGAATtgTGACAGgTttGnaGTGaaAGccgAAAAGCTGTCAAGAAAAccac
>Bacillus_Fam_102_52_5 Nr. of seq. 5 Alignment length(with gaps) = 58
Alignment score = 0.601149
nGcTATTGgCGACAtTtCTnttagcAAAAntcAacaaAAGTGTTTCCAATgg
>Bacillus_Fam_103_53_5 Nr. of seq. 5 Alignment length(with gaps) = 57
Alignment score = 0.626901
CACTCgGnGTAgTGGcGCCAcAAAngAGGatcCaTcaCgaGaAAAAGTGGcAg
>Bacillus_Fam_104_52_5 Nr. of seq. 5 Alignment length(with gaps) = 56
Alignment score = 0.618750
ATGACGGACAcTTTTntCntntTTccntngaTTTcTGTCCGTCATggCCTtg
>Bacillus_Fam_105_53_5 Nr. of seq. 5 Alignment length(with gaps) = 56
Alignment score = 0.664286
AgTCATGTAtccCAacagGCaTCcGgGATACATgACccGGCnGagaAAAGnGA
>Bacillus_Fam_106_53_5 Nr. of seq. 5 Alignment length(with gaps) = 56
Alignment score = 0.671429
tTGGaTaAAatTAGGaGAAaGCGaACCaGtAAAgtcGTnaaTCaTCCtcGCAa
>Bacillus_Fam_107_52_5 Nr. of seq. 5 Alignment length(with gaps) = 55
Alignment score = 0.752727
gAGTaAAGTAACGAAAAGaGccGAACgaGnCAGTTACTcGaGGGaAGtgAGn
>Bacillus_Fam_108_52_5 Nr. of seq. 5 Alignment length(with gaps) = 55
Alignment score = 0.681818
TaATGTGCAGATAcgGAaGAGanaAGgGctaGTAtCTGcACAAAgAGgcGgt
>Bacillus_Fam_109_52_5 Nr. of seq. 5 Alignment length(with gaps) = 55
Alignment score = 0.613333
ancCtGTCAGTtnCcTTTTTTCnnGccTcnCcngatTTTtGGGcACTGAGAg
>Bacillus_Fam_110_52_5 Nr. of seq. 5 Alignment length(with gaps) = 54
Alignment score = 0.714815
TGGTAGTTAATCgaGAnGaaTAACGACGAAAAagacgaaggTCnAaGGAAAa
>Bacillus_Fam_111_52_5 Nr. of seq. 5 Alignment length(with gaps) = 54
Alignment score = 0.714815
TTTGcTACCCcATATTtCnCnCaCtCcgtTTTATAaGGatGCAtTTTCttcT
>Bacillus_Fam_112_52_5 Nr. of seq. 5 Alignment length(with gaps) = 54
Alignment score = 0.676543
ACTTATCtCGnntTtCAGtACCACCAAnaCgcagatntCCTTtcTTGATGGC
>Bacillus_Fam_113_52_5 Nr. of seq. 5 Alignment length(with gaps) = 54
Alignment score = 0.609877
GACAgTTTtnntaatTTttgaagcggtTTTGTCTtCaAGAagcCTTATTgAA
>Bacillus_Fam_114_52_5 Nr. of seq. 5 Alignment length(with gaps) = 54
Alignment score = 0.701235
gATGAgaACCGaAACaAncgcagcAaGAGAAGGaAaCGGTCTTCATcAaGgg
>Bacillus_Fam_115_53_5 Nr. of seq. 5 Alignment length(with gaps) = 53
Alignment score = 0.694969
GGTCAAAAGGagaAGCnCTATTCACCcGAAAAgngaGccGnacCaaGaAnaAc
>Bacillus_Fam_116_51_5 Nr. of seq. 5 Alignment length(with gaps) = 53
Alignment score = 0.735220
aAtcncggggttgCnggnGnaAAaaGcaGGagtaaccccgAGcAAAcGgnG
>Bacillus_Fam_117_52_5 Nr. of seq. 5 Alignment length(with gaps) = 53
Alignment score = 0.725786
AGTAACTGaCAtgtTcaGcCttTTTCGTGtaGTTACTCACcctnttCCTtcg
>Bacillus_Fam_118_52_5 Nr. of seq. 5 Alignment length(with gaps) = 53
Alignment score = 0.699371
aGTTcCCGagccCgCGnaAaaTccTCCATCaaGGTAACCAAaCgCaCTCatt

```
>Bacillus_Fam_119_52_5  Nr. of seq. 5 Alignment length(with gaps) = 53
Alignment score = 0.620755
 GtTTTGTCtAaTAgAGnncttCTATtGgACAaAatCaTtgCtCagAnCatGt
>Bacillus_Fam_120_52_5  Nr. of seq. 5 Alignment length(with gaps) = 52
Alignment score = 0.853846
 ACACATTTTTcgCtTnTcCCcaTGATAATCTGTCTCAgGcATGGCtTCTGAC
>Bacillus_Fam_121_52_5  Nr. of seq. 5 Alignment length(with gaps) = 52
Alignment score = 0.823077
 tnaTTGAACCCCGAaCagGCGAAAAAAAgCatCAAAGaGTCtTCAATAGggG
>Bacillus_Fam_122_45_5  Nr. of seq. 5 Alignment length(with gaps) = 48
Alignment score = 0.736806
 ACTGGACCTCAAGGtgtTCAAGGAcCagcaGGtgCTACtGGtGCt
>Bacillus_Fam_123_39_5  Nr. of seq. 5 Alignment length(with gaps) = 42
Alignment score = 0.642857
 tTCTnCTcTttGcTgCTCtTCtTGCTTaCGtTTtTCttc
>Bacillus_Fam_124_40_5  Nr. of seq. 5 Alignment length(with gaps) = 42
Alignment score = 0.684127
 ATTTGCGTCTcCtCngnGATTTtTGCGTCGgTtTtCtCan
>Bacillus_Fam_125_42_5  Nr. of seq. 5 Alignment length(with gaps) = 42
Alignment score = 0.888889
 AAAGAAGAAGTaAAAGAgCCggtAAAAGAAGTTGAAGaAaCa
>Bacillus_Fam_126_39_5  Nr. of seq. 5 Alignment length(with gaps) = 39
Alignment score = 0.788034
 nAAgCAaCAAAgTtCTAAaCAAGAnGATTCnTCTCAagA
>Bacillus_Fam_127_36_5  Nr. of seq. 5 Alignment length(with gaps) = 36
Alignment score = 0.792593
 gTTGtTCTTCtaCGACcGGtgcTTCtTCTgCAACTg
>Bacillus_Fam_128_36_5  Nr. of seq. 5 Alignment length(with gaps) = 36
Alignment score = 0.874074
 CTTCcACTTCTTCTgaCTCTTctGtTTCcGCAATTA
>Bacillus_Fam_129_29_5  Nr. of seq. 5 Alignment length(with gaps) = 30
Alignment score = 0.824444
 GAGCAGCGgAGCGTACAagggACGTACGt
>Bacillus_Fam_130_21_5  Nr. of seq. 5 Alignment length(with gaps) = 24
Alignment score = 0.626389
 TCGACCAcATttngaAnTAAT
>Bacillus_Fam_131_24_5  Nr. of seq. 5 Alignment length(with gaps) = 24
Alignment score = 0.822222
 AGCTTctTCtTCaGCTTtTTTctT
>Bacillus_Fam_132_21_5  Nr. of seq. 5 Alignment length(with gaps) = 23
Alignment score = 0.630435
 AGTcGAGCATAaAncCtgAaa
>Bacillus_Fam_133_21_5  Nr. of seq. 5 Alignment length(with gaps) = 23
Alignment score = 0.650725
 TGCAAATAAAaanngTgaAAC
>Bacillus_Fam_134_21_5  Nr. of seq. 5 Alignment length(with gaps) = 23
Alignment score = 0.628986
 TTTATCAGCGaAttTnanncT
>Bacillus_Fam_135_21_5  Nr. of seq. 5 Alignment length(with gaps) = 23
Alignment score = 0.630435
 TTTATTTTCgaaTTTgaTcan
>Bacillus_Fam_136_20_5  Nr. of seq. 5 Alignment length(with gaps) = 22
Alignment score = 0.613636
 GATATatTTnganAATCGCt
```

>Bacillus_Fam_137_20_5 Nr. of seq. 5 Alignment length(with gaps) = 22
Alignment score = 0.633333
 CcGATATAaTcagAtTtTcg
>Bacillus_Fam_138_20_5 Nr. of seq. 5 Alignment length(with gaps) = 22
Alignment score = 0.601515
 taTCaGCCAAACTTnnnatT
>Bacillus_Fam_139_21_5 Nr. of seq. 5 Alignment length(with gaps) = 22
Alignment score = 0.645455
 nTcGnGATaTATGAGCgAGan
>Bacillus_Fam_140_22_5 Nr. of seq. 5 Alignment length(with gaps) = 22
Alignment score = 0.630303
 TTTATTGTCCnGaTncgcnnCA
>Bacillus_Fam_141_20_5 Nr. of seq. 5 Alignment length(with gaps) = 21
Alignment score = 0.666667
 ATCGGTCtaAcTtnaaAtAT
>Bacillus_Fam_142_20_5 Nr. of seq. 5 Alignment length(with gaps) = 20
Alignment score = 0.746667
 tctCgCTATacgAGAGTATt
>Bacillus_Fam_143_20_5 Nr. of seq. 5 Alignment length(with gaps) = 20
Alignment score = 0.686667
 cGCAAATAAAanctngGaAT
>Bacillus_Fam_144_18_5 Nr. of seq. 5 Alignment length(with gaps) = 18
Alignment score = 0.718518
 GGagcAACnGGcCCaACa
>Bacillus_Fam_145_12_5 Nr. of seq. 5 Alignment length(with gaps) = 12
Alignment score = 0.811111
 GAAGAnGAaGAt
>Bacillus_Fam_146_52_4 Nr. of seq. 4 Alignment length(with gaps) = 58
Alignment score = 0.652778
 gTTCGGACTgCtCtgaaCGGgTTTCtTCTTCTCCTGTCCGAAgttGCAtCaG
>Bacillus_Fam_147_54_4 Nr. of seq. 4 Alignment length(with gaps) = 57
Alignment score = 0.632554
 TTCATccgcTtGATGAAcACCAcTTTTtCCTcnGnCTTCntCTggaTTgTGGTg
>Bacillus_Fam_148_52_4 Nr. of seq. 4 Alignment length(with gaps) = 57
Alignment score = 0.626218
 gCTcTTGgTGACCGAaAtccaaGGaAAAtaGaAAaaGTGGTaACCAAgaAGg
>Bacillus_Fam_149_53_4 Nr. of seq. 4 Alignment length(with gaps) = 57
Alignment score = 0.627680
 GACAGCTTtaGCtanAtcAAcAgAGaAAGCTGTCcGAACatAGccAgACTTCG
>Bacillus_Fam_150_53_4 Nr. of seq. 4 Alignment length(with gaps) = 56
Alignment score = 0.619048
 GATTAACTaCCATTTTcgcCTTactcCAcCTcAaTTTcGgCGTtAaTCCatct
>Bacillus_Fam_151_52_4 Nr. of seq. 4 Alignment length(with gaps) = 56
Alignment score = 0.669643
 TTATGAAGGACAtTTCagccTGCgGtTcgcaacCtTTTGTCCTTCATCCcGc
>Bacillus_Fam_152_53_4 Nr. of seq. 4 Alignment length(with gaps) = 56
Alignment score = 0.633929
 tGAaGGTCATCATAcGaAGGnCAATGCTacCCGAgAAAgcAaaAcagAaGcgc
>Bacillus_Fam_153_52_4 Nr. of seq. 4 Alignment length(with gaps) = 56
Alignment score = 0.611607
 cTcTTCaTTTcagttTTCGGTCACTCCAGacCcCcTGgAgTgACCcAAtCnT
>Bacillus_Fam_154_53_4 Nr. of seq. 4 Alignment length(with gaps) = 56
Alignment score = 0.640377
 TCTACGTTACCCttcagCcTtatTTTTcgACCttTCGGgCACGTAGAcaCCCt

>Bacillus_Fam_155_54_4 Nr. of seq. 4 Alignment length(with gaps) = 56
Alignment score = 0.630952
CCTGAAaCaCtcTccgATcaCTCTnCTTCAGagGAATAAAtgCatCtTTATtCA
>Bacillus_Fam_156_51_4 Nr. of seq. 4 Alignment length(with gaps) = 55
Alignment score = 0.623232
ATTgGTAATGnAaaTtTcactTTCtCTctTTTaCGGTGCGTATTAtcctga
>Bacillus_Fam_157_53_4 Nr. of seq. 4 Alignment length(with gaps) = 55
Alignment score = 0.639899
ctttaTTCGCaCCCTaATTTgnCTgCnAtcCtCgATTtTCATTACCtATCACg
>Bacillus_Fam_158_51_4 Nr. of seq. 4 Alignment length(with gaps) = 55
Alignment score = 0.638384
AGTCCGAACCtGccaCaACTTCgGACAgaAaAacGAAAAAtCCcgtGccAG
>Bacillus_Fam_159_52_4 Nr. of seq. 4 Alignment length(with gaps) = 55
Alignment score = 0.621212
GTTCGGACaCgATTTcGCTtcTnCgacctTtttCTGTCCGAAcTacCaCCaA
>Bacillus_Fam_160_52_4 Nr. of seq. 4 Alignment length(with gaps) = 55
Alignment score = 0.711616
aTAAGTGCCAtCAAcaACagAGATTcCtggTTTGgTGGTTCcTtTCtGcCTG
>Bacillus_Fam_161_52_4 Nr. of seq. 4 Alignment length(with gaps) = 55
Alignment score = 0.627778
ctcAaGTTTaCGcgAAAacgGGcgcATAgAACCTTcCTATgAaCCcAaaATc
>Bacillus_Fam_162_53_4 Nr. of seq. 4 Alignment length(with gaps) = 55
Alignment score = 0.636364
ggAgAGaATcGTgCCCGAAATcnaaGgaAGGcgAAGgaAAACGGtAACAATag
>Bacillus_Fam_163_52_4 Nr. of seq. 4 Alignment length(with gaps) = 55
Alignment score = 0.742929
cAAAATGGTCTTCATCacccTTATGAAGaaCAtTTCTCTTcGgCTttTCatC
>Bacillus_Fam_164_52_4 Nr. of seq. 4 Alignment length(with gaps) = 55
Alignment score = 0.659596
GCGTTcCATtCACCttCTATGACACgCGttttCaCTccCGCTCtCtattTTc
>Bacillus_Fam_165_52_4 Nr. of seq. 4 Alignment length(with gaps) = 55
Alignment score = 0.602525
gTTCAGACAGCTTTTgccgcctaGCaCcgaAAgCTGTCCGAAgTtGgccaaG
>Bacillus_Fam_166_52_4 Nr. of seq. 4 Alignment length(with gaps) = 54
Alignment score = 0.626543
tTTTCATTACcTATCaCaaCTTtTTCGcACccTAATcCcagCAtCcCacaan
>Bacillus_Fam_167_52_4 Nr. of seq. 4 Alignment length(with gaps) = 54
Alignment score = 0.618827
aAAAATGGTCATAGAgagagaAACTGGAACCAAAAatgctcGaGcaAAgcga
>Bacillus_Fam_168_50_4 Nr. of seq. 4 Alignment length(with gaps) = 54
Alignment score = 0.652778
TTTGTCTTTCATAagCTcGATGAAaGACTTTTTCttgaagcTCcAcCgAT
>Bacillus_Fam_169_54_4 Nr. of seq. 4 Alignment length(with gaps) = 54
Alignment score = 0.745885
GaCGAAAAcGTGgnaGaGAgGtAGaGAAAatGGTAGcTAATCaACcGGATTAAC
>Bacillus_Fam_170_52_4 Nr. of seq. 4 Alignment length(with gaps) = 54
Alignment score = 0.600823
GGACaaCTTTctCcgCctgcCgCcTcAACCTGTCcGAACttacaccgaCTTC
>Bacillus_Fam_171_53_4 Nr. of seq. 4 Alignment length(with gaps) = 54
Alignment score = 0.641975
GTCGCaTAgAAgGcGTATAcGCGaCcGAgCGGcaGaaAAAataGaGnngCtCG
>Bacillus_Fam_172_53_4 Nr. of seq. 4 Alignment length(with gaps) = 54
Alignment score = 0.720165
aTGAAGGACTTTgCtCGcTcTTCTtgACCtgGTTTTGgcCTTCATtagCttga

>Bacillus_Fam_173_52_4 Nr. of seq. 4 Alignment length(with gaps) = 54
Alignment score = 0.661523
ATCGTGCCCTcaAAAcgagaAAAAcGAaAAtagaGGtAACGATgaAGcGctG
>Bacillus_Fam_174_54_4 Nr. of seq. 4 Alignment length(with gaps) = 54
Alignment score = 0.763375
GTCATGTATcCCAaCAGGTTcCCGGaATACATGaGTAtGacaGaAAaAAGGaTa
>Bacillus_Fam_175_54_4 Nr. of seq. 4 Alignment length(with gaps) = 54
Alignment score = 0.627572
CATGagACATACtTTcCtCTaaaAAccccCTcaacTtaTGTttCATGAaCCCtT
>Bacillus_Fam_176_53_4 Nr. of seq. 4 Alignment length(with gaps) = 54
Alignment score = 0.659465
gAaaAAATTAGGaAAaagCgAACCaGcAAAcgcgatAtaCAGGTtCGCAtaTG
>Bacillus_Fam_177_51_4 Nr. of seq. 4 Alignment length(with gaps) = 53
Alignment score = 0.768344
AAAAAtGGGAAccAAAGAGCCgcAaTgATGCCCAAAATcCaaGAGtCGCgG
>Bacillus_Fam_178_52_4 Nr. of seq. 4 Alignment length(with gaps) = 53
Alignment score = 0.633124
ATAAGAAAGcgCcGgATTccCaTCAAGAAaaccGcaAnCncGaaTaAAGtTG
>Bacillus_Fam_179_53_4 Nr. of seq. 4 Alignment length(with gaps) = 53
Alignment score = 0.742138
CTTAGTCaGCTGaACTAGaaCCCTCTGaTaaGacAATTaagAGtCCAGAGGGc
>Bacillus_Fam_180_53_4 Nr. of seq. 4 Alignment length(with gaps) = 53
Alignment score = 0.778826
ACCGCTATTTCtaTGAAAAaCgAAAATAaTGGTTcCAGCaggAGaGAGcTAGa
>Bacillus_Fam_181_53_4 Nr. of seq. 4 Alignment length(with gaps) = 53
Alignment score = 0.796646
TTcCTcgTcCtCTCTTcCaTTTTCGTCgTCATGAAcCCTCcATGaCGACCATt
>Bacillus_Fam_182_53_4 Nr. of seq. 4 Alignment length(with gaps) = 53
Alignment score = 0.667715
GTCAGtGCCCaAAATccGCgGagGcCnGccaAAAAAGGTAaCTGAcAggCcgc
>Bacillus_Fam_183_52_4 Nr. of seq. 4 Alignment length(with gaps) = 53
Alignment score = 0.642034
GGgTGATGAAgGcCaaAACgtGgaaAaaGagaagcaGAAAAGTCcTTCATaa
>Bacillus_Fam_184_52_4 Nr. of seq. 4 Alignment length(with gaps) = 53
Alignment score = 0.692872
TCCAATaagcCCgATTGGAtACCTTTTcTCCnAttTTTccagcgGaACGGGt
>Bacillus_Fam_185_52_4 Nr. of seq. 4 Alignment length(with gaps) = 53
Alignment score = 0.707547
TTTTGgcCTTCATCngcCTTATGAAGacCATTTtGaaCCtGGctCaCgcCTg
>Bacillus_Fam_186_51_4 Nr. of seq. 4 Alignment length(with gaps) = 52
Alignment score = 0.607906
GCaCCgTAATttcggcacccCacctgTTTCATTACCtATTCatcgtTATtC
>Bacillus_Fam_187_51_4 Nr. of seq. 4 Alignment length(with gaps) = 52
Alignment score = 0.695513
TTTCATTACcTATCccagGTTTTTCGcACCcTAATccctGCaAAacTcaAt
>Bacillus_Fam_188_52_4 Nr. of seq. 4 Alignment length(with gaps) = 52
Alignment score = 0.668803
TGAAGGACAAAACcCtgtAgaAAaacagaAgAAaTGTCCTTCATaaaGccaA
>Bacillus_Fam_189_52_4 Nr. of seq. 4 Alignment length(with gaps) = 52
Alignment score = 0.784188
GGcccTAGTCGagCGaACTAAcACCCTCTATtCtcCAAAATGacaTTCAGAG
>Bacillus_Fam_190_52_4 Nr. of seq. 4 Alignment length(with gaps) = 52
Alignment score = 0.841880
AAAATGTGTCTCAacCcgGTCaATGGAAaCACATAAcAAGaGATtaTGCTcA

>Bacillus_Fam_191_52_4 Nr. of seq. 4 Alignment length(with gaps) = 52
Alignment score = 0.735043
ttTTGGTTCCAGTTAcaCaatCTAtGACCATTTTctGcttgaCtaCACCcaA
>Bacillus_Fam_192_45_4 Nr. of seq. 4 Alignment length(with gaps) = 45
Alignment score = 0.887654
CAAAATCCATATGcAGCAcCaCaAAacCAAGAACAACAATATCAa
>Bacillus_Fam_193_42_4 Nr. of seq. 4 Alignment length(with gaps) = 43
Alignment score = 0.714470
TGGCCGaAAAAGcgacgCAGTTGGCGcgAAAGtgaGtTcaGT
>Bacillus_Fam_194_40_4 Nr. of seq. 4 Alignment length(with gaps) = 40
Alignment score = 0.730556
AAAaGAGCCAacGGaaCcGTCCCCCcGGCCCaGcTcCAaa
>Bacillus_Fam_195_40_4 Nr. of seq. 4 Alignment length(with gaps) = 40
Alignment score = 0.661111
nTAAAGCggTaatGcgcTGgGGGaCAGgCCCCCgCtnaaG
>Bacillus_Fam_196_40_4 Nr. of seq. 4 Alignment length(with gaps) = 40
Alignment score = 0.686111
cAGACGCAAAAATcagaaTTtcGACGCAAATCctcaGaag
>Bacillus_Fam_197_36_4 Nr. of seq. 4 Alignment length(with gaps) = 36
Alignment score = 0.608025
CCAGTcGgtCCAgTaaTaCCaGTntctCCaGTagca
>Bacillus_Fam_198_36_4 Nr. of seq. 4 Alignment length(with gaps) = 36
Alignment score = 0.804012
TCTTCCTTAGccTTtgCTAcTTCTTGgaCTTTcgCT
>Bacillus_Fam_199_36_4 Nr. of seq. 4 Alignment length(with gaps) = 36
Alignment score = 0.658951
CCcGTGTtACCtTgAgctCCttgaGgtCCaGTaGCg
>Bacillus_Fam_200_36_4 Nr. of seq. 4 Alignment length(with gaps) = 36
Alignment score = 0.780864
CCAGTAGGtCCaGTGaTnCCaGTAaATCCgGTagcC
>Bacillus_Fam_201_33_4 Nr. of seq. 4 Alignment length(with gaps) = 35
Alignment score = 0.651587
CctCCCATCATgCCtGgnTGaCcTCCttgaTGa
>Bacillus_Fam_202_27_4 Nr. of seq. 4 Alignment length(with gaps) = 28
Alignment score = 0.646825
CCAgcTaaTCCTGTcGcTCCcGtTGct
>Bacillus_Fam_203_27_4 Nr. of seq. 4 Alignment length(with gaps) = 27
Alignment score = 0.711934
GGAGcaACaGGtcCaACaGGAATaaCa
>Bacillus_Fam_204_25_4 Nr. of seq. 4 Alignment length(with gaps) = 25
Alignment score = 0.617778
gCctccGCtTCTGCcTccGCtTTTG
>Bacillus_Fam_205_24_4 Nr. of seq. 4 Alignment length(with gaps) = 24
Alignment score = 0.805556
CCtGaAAcAcCaGATCCAGAaAAA
>Bacillus_Fam_206_20_4 Nr. of seq. 4 Alignment length(with gaps) = 23
Alignment score = 0.718599
TATGATCCATTtCaGAAATa
>Bacillus_Fam_207_21_4 Nr. of seq. 4 Alignment length(with gaps) = 23
Alignment score = 0.642512
aacaTgGAATAAACTCcGaaA
>Bacillus_Fam_208_21_4 Nr. of seq. 4 Alignment length(with gaps) = 22
Alignment score = 0.604798
tCTATTTGCaGnTTTccgatt

>Bacillus_Fam_209_22_4 Nr. of seq. 4 Alignment length(with gaps) = 22
Alignment score = 0.651515
AGTCGCAAGtAAaaGtGgnGan
>Bacillus_Fam_210_21_4 Nr. of seq. 4 Alignment length(with gaps) = 22
Alignment score = 0.650253
GTAGAgTAAAnTncagAAAtT
>Bacillus_Fam_211_21_4 Nr. of seq. 4 Alignment length(with gaps) = 22
Alignment score = 0.656566
TTaGCGAatTTCaaaCatTTA
>Bacillus_Fam_212_21_4 Nr. of seq. 4 Alignment length(with gaps) = 22
Alignment score = 0.631313
TAtCTACGAAAttTanAgATA
>Bacillus_Fam_213_21_4 Nr. of seq. 4 Alignment length(with gaps) = 22
Alignment score = 0.611111
AcaAaaaGAAATGaAAgaaGa
>Bacillus_Fam_214_22_4 Nr. of seq. 4 Alignment length(with gaps) = 22
Alignment score = 0.623737
aTTTATTGTCCgGAaaCaaatg
>Bacillus_Fam_215_22_4 Nr. of seq. 4 Alignment length(with gaps) = 22
Alignment score = 0.694444
aTCAGCCAAAgtgaGnagGGAA
>Bacillus_Fam_216_22_4 Nr. of seq. 4 Alignment length(with gaps) = 22
Alignment score = 0.702020
ATCTCTCGGTTTtGCGnctttA
>Bacillus_Fam_217_22_4 Nr. of seq. 4 Alignment length(with gaps) = 22
Alignment score = 0.601010
tATTTGCGAATcTagcacctTT
>Bacillus_Fam_218_20_4 Nr. of seq. 4 Alignment length(with gaps) = 21
Alignment score = 0.669312
tTGCGagTTTctCcAtATAT
>Bacillus_Fam_219_20_4 Nr. of seq. 4 Alignment length(with gaps) = 21
Alignment score = 0.671958
ATtATCaAAAAancaAtTAT
>Bacillus_Fam_220_21_4 Nr. of seq. 4 Alignment length(with gaps) = 21
Alignment score = 0.625661
AgTTgGCGGaaTTAtTtacGa
>Bacillus_Fam_221_21_4 Nr. of seq. 4 Alignment length(with gaps) = 21
Alignment score = 0.634921
AcTcaccgATAGAAacgCAAa
>Bacillus_Fam_222_21_4 Nr. of seq. 4 Alignment length(with gaps) = 21
Alignment score = 0.701058
TGTcgTAAGtggCtgAAAATa
>Bacillus_Fam_223_21_4 Nr. of seq. 4 Alignment length(with gaps) = 21
Alignment score = 0.666667
TATCAGCGaATtTaanCaaTa
>Bacillus_Fam_224_21_4 Nr. of seq. 4 Alignment length(with gaps) = 21
Alignment score = 0.724868
TTTACTGTCCacTTtTanccA
>Bacillus_Fam_225_20_4 Nr. of seq. 4 Alignment length(with gaps) = 20
Alignment score = 0.666667
TTATctgCGAtTtCtgattT
>Bacillus_Fam_226_20_4 Nr. of seq. 4 Alignment length(with gaps) = 20
Alignment score = 0.788889
aTTCAGgATAAATCccAAAa

>Bacillus_Fam_227_18_4 Nr. of seq. 4 Alignment length(with gaps) = 19
Alignment score = 0.688596
TCgCCtTGaGGtCCTtGT
>Bacillus_Fam_228_18_4 Nr. of seq. 4 Alignment length(with gaps) = 18
Alignment score = 0.641975
GTgAcgCCtGTaGgaCCA
>Bacillus_Fam_229_18_4 Nr. of seq. 4 Alignment length(with gaps) = 18
Alignment score = 0.716049
CCAGGaGAtCCaaGcaAa
>Bacillus_Fam_230_18_4 Nr. of seq. 4 Alignment length(with gaps) = 18
Alignment score = 0.864198
AGACCATTtGGtTTTGGc
>Bacillus_Fam_231_15_4 Nr. of seq. 4 Alignment length(with gaps) = 15
Alignment score = 0.700000
aCCgAAaaAAGaAGa
>Bacillus_Fam_232_12_4 Nr. of seq. 4 Alignment length(with gaps) = 12
Alignment score = 0.888889
GGCAACGGcAAt
>Bacillus_Fam_233_234_3 Nr. of seq. 3 Alignment length(with gaps) = 234
Alignment score = 0.864198

CCAAAACAACGACGAAGTATGTCAATGTGGAtAAaGGnTCcCATtTAATaTTaCGtTCnAAAGCCTCCaaaaC
GTCCAGCATTTTaGcaAGCTTGgCAAGAGGngAAAAaGTGACGGTtTACTCtATTTCCGGGGAnTGGGCaAAA
GTAAAAGCCGGgAGCAAAACGGGCTATGTTCATGCATCTTTTTTGgCGAAcTCAAATCCcGAtAGCAaTGCAG
acACTTCtACtCCaG
>Bacillus_Fam_234_213_3 Nr. of seq. 3 Alignment length(with gaps) = 222
Alignment score = 0.719720

TTAAACcTtCGgtCgGGtGCTGGcACgtctCAtCGngTTtTaACgACnCTtCCtGTTGGaCAgAAGCTtGAgC
TTCTTcAAAAAcAAGGCAATTGGTATCAAGTTAAAGCtGGtaacCAaACtGGcTGGGTtTCtGCTGAtTTcAT
TAAAaCagAtGGtAATaatGTcGAAGaCAACAAgCCTTCTcTtGGCTCTGCgACgACtACaGCTCGC
>Bacillus_Fam_235_166_3 Nr. of seq. 3 Alignment length(with gaps) = 168
Alignment score = 0.659392

attaAcTcCnnaTCCacTATtcCCGTCtCCcAgCTGACCaaaATcGTTntCttCCCCACGtATACAnCGTtCC
ATCACTTGCTAAcGCTAcTGtgTGancnantCCTgCnGnTACTTGTTCGATctTGGCTCCTcCgGGgATnGng
ATCgCTgTCGGACTAGnttg
>Bacillus_Fam_236_151_3 Nr. of seq. 3 Alignment length(with gaps) = 159
Alignment score = 0.705101

GCCGATgAccAAGgtgCTtccgctTTTCtTcTGTCTAGCTCCAgCGCCTATCGGCTaGCgGatTTCTcCGTCT
cCTCCCTgCGATAAGtCAACATCAGCTCGTaCCTCGCTGTGTnTCCTTtATCTCAGtCgnAgacTctGaaAtC
CGtAC
>Bacillus_Fam_237_144_3 Nr. of seq. 3 Alignment length(with gaps) = 148
Alignment score = 0.644895

TGGTGGGAcGGncgaaGnTGGAGcnagTGGGAaAATCTtGGttCnCCgCaaGGnGGatTTGAaGGctCGCCag
gtGCtGTcTCaTGGGgacCAAATCGnATCGACTGtTTTGTcAGGGganGnaAtAAtCanATGTGGCAtAAa
>Bacillus_Fam_238_122_3 Nr. of seq. 3 Alignment length(with gaps) = 125
Alignment score = 0.682667

GGCTCGcTCAGAATgggAGGaggaTGGAGCTTCtGaCcTtGAgGCGCTtTTTGCcTCgnggGAAGAnGCGAAg
ccaCCGaacaTTCTaGCCaCTGgAGCTGGATatCaTTAAAAGCGgAAgt

>Bacillus_Fam_239_104_3 Nr. of seq. 3 Alignment length(with gaps) = 104
Alignment score = 0.978632

ATGCTAGAAACTGATTCAAAAGCTTGAACAAGAATATCTGTAAACATAAGATCCACCTCCAGTTTGAAAATAA
GGAAAAAgAGAATTAgCCAAGAGTGCCGATA
>Bacillus_Fam_240_84_3 Nr. of seq. 3 Alignment length(with gaps) = 84
Alignment score = 0.825397

AAGAGTTTTCaGaGATGCTTGAAGAAAGATCTCCcGAATGtGAtGAgTcTGAATcAATcAAaGAaGAaGatAC
tTCAtTAGAAg
>Bacillus_Fam_241_59_3 Nr. of seq. 3 Alignment length(with gaps) = 66
Alignment score = 0.611111
GGCTGGgTgCAAActGgnGGcAaATGGTATTACCTtAATcaAtcGGnGCgATGCAAACc
>Bacillus_Fam_242_66_3 Nr. of seq. 3 Alignment length(with gaps) = 66
Alignment score = 0.752525
ACAGGcTGGGnAcAAGTAGanGGnAAATGGTACTaCCTaGATGcaAAtGatGGTggaGCAATGAAA
>Bacillus_Fam_243_66_3 Nr. of seq. 3 Alignment length(with gaps) = 66
Alignment score = 1.000000
GGGGTCTGACCCCGTTTGTTATAATCAAGAGCTACGAACCCTTGTCTTTCCTGGAATTAACGCTTG
>Bacillus_Fam_244_55_3 Nr. of seq. 3 Alignment length(with gaps) = 58
Alignment score = 0.607280
TaTTGGTGACCTTTCtTccTCtaGnaCaCttcccACTTtGGGCACCAAtGaGCCT
>Bacillus_Fam_245_53_3 Nr. of seq. 3 Alignment length(with gaps) = 57
Alignment score = 0.606238
TGGcCCATTTAnCaCcTtAaTGGcCCAACATCcTCTcAtAacTtgAtTCTCAt
>Bacillus_Fam_246_53_3 Nr. of seq. 3 Alignment length(with gaps) = 56
Alignment score = 0.684524
AAGTGGCGCCATTCtGcanAGTGgTGCCACtTTTcGAGTGAngAGActCtCaa
>Bacillus_Fam_247_50_3 Nr. of seq. 3 Alignment length(with gaps) = 55
Alignment score = 0.630303
TTTTCATTACCtATTaACCTttATTCGcACcgTAATTCgccCagCTcGga
>Bacillus_Fam_248_51_3 Nr. of seq. 3 Alignment length(with gaps) = 55
Alignment score = 0.612121
GGTAACAGAGAagGCgTCTCAAgGCCCGAAgCaAGnagcGAAncnCnAAAC
>Bacillus_Fam_249_53_3 Nr. of seq. 3 Alignment length(with gaps) = 55
Alignment score = 0.616162
aAACGGTAACAGAGAgGCcGTCTCAaaGCCcGAAgctgCTCngaactnCgCCa
>Bacillus_Fam_250_51_3 Nr. of seq. 3 Alignment length(with gaps) = 55
Alignment score = 0.620202
GTTTcTCttngAATCtTACCATTATAAatCggtTTCCCTcTGtAgtGGTAC
>Bacillus_Fam_251_52_3 Nr. of seq. 3 Alignment length(with gaps) = 55
Alignment score = 0.634343
TACCtCTTTTCcattCnCTcCTTCCCtTTTnTTGTcTCCATCtccnTTTGGt
>Bacillus_Fam_252_53_3 Nr. of seq. 3 Alignment length(with gaps) = 55
Alignment score = 0.638384
ATGAAgGaCaatTaGGnnTGAAAtTTttGGTcGAAATGntgTTCATAAGcCCG
>Bacillus_Fam_253_52_3 Nr. of seq. 3 Alignment length(with gaps) = 55
Alignment score = 0.731313
CCACCAAACTTCttccTCaCGcgCTTGATGGTTCTTATaaCCGTTTTcAGTA
>Bacillus_Fam_254_53_3 Nr. of seq. 3 Alignment length(with gaps) = 55
Alignment score = 0.628283
TTGGCTaCCGTTCTTtcGCnnTcTCnCtCggtTTCGGGatCCAtCacACCTCT
>Bacillus_Fam_255_52_3 Nr. of seq. 3 Alignment length(with gaps) = 55
Alignment score = 0.660606

TAGACATTTcAAgCtncAnCAGctTcTGAAATGTCtATTAAAcaCCTtTTaT
>Bacillus_Fam_256_52_3 Nr. of seq. 3 Alignment length(with gaps) = 55
Alignment score = 0.668687
CTCTATGtgCACTTtnCncGCTTTTTtcAtcGaTTTCGTGTTCATTGaaGAC
>Bacillus_Fam_257_52_3 Nr. of seq. 3 Alignment length(with gaps) = 55
Alignment score = 0.634343
TGCATTGCCCAAAAancGGtGAtAgAtcnAAAtTTGGTCAAtGGAaGaGGgn
>Bacillus_Fam_258_51_3 Nr. of seq. 3 Alignment length(with gaps) = 55
Alignment score = 0.612121
aTTTaTTCCTCTaAAGCtgcAaAAttcGcAAGCTTcAGGTGAAtAAanGaG
>Bacillus_Fam_259_52_3 Nr. of seq. 3 Alignment length(with gaps) = 55
Alignment score = 0.723232
AATGTGTCTCAAGACtgGatCTTGAGACAgATAAAgAGGGaGaAAggAgAAA
>Bacillus_Fam_260_52_3 Nr. of seq. 3 Alignment length(with gaps) = 55
Alignment score = 0.717172
ATGAAaACCAttTTctCTTCCGGAACaAgGAgAAACGGTCTTCATgaCGGCg
>Bacillus_Fam_261_53_3 Nr. of seq. 3 Alignment length(with gaps) = 55
Alignment score = 0.640404
ACCGCAAGAangGTTTcacGGTACAtGCTCAntcAAAAAnAGancaAGCaTGT
>Bacillus_Fam_262_53_3 Nr. of seq. 3 Alignment length(with gaps) = 55
Alignment score = 0.696970
CGGGCACTATGAACtCTcTATAGTgACCGCncaCTtCTTnTgAcTcCCTCCtg
>Bacillus_Fam_263_52_3 Nr. of seq. 3 Alignment length(with gaps) = 54
Alignment score = 0.633745
GTCAAATAGAAcGgnTCTATAcGCCCGAAnGCcCGtgaAAaAgaGgnaAAcG
>Bacillus_Fam_264_52_3 Nr. of seq. 3 Alignment length(with gaps) = 54
Alignment score = 0.641975
cTCAnGACACTTTTCtcTCTcnTTTTngnnTTTcATGtCCTTGAGAnaCTtC
>Bacillus_Fam_265_52_3 Nr. of seq. 3 Alignment length(with gaps) = 54
Alignment score = 0.637860
ACTTCGGACTCattCCTCcagATttCCnCTttTtCTGTCcGAAtGnTGACCn
>Bacillus_Fam_266_52_3 Nr. of seq. 3 Alignment length(with gaps) = 54
Alignment score = 0.699588
CGGATTCGGACAACattaCcgnTTCatCCTgCTTccCTGTCCGAATATcAAG
>Bacillus_Fam_267_53_3 Nr. of seq. 3 Alignment length(with gaps) = 54
Alignment score = 0.627572
cTAAAGGACAAAACtcgGatgnagCAgCAAAGATnnGTCCATGAgAaGcggTc
>Bacillus_Fam_268_52_3 Nr. of seq. 3 Alignment length(with gaps) = 54
Alignment score = 0.633745
AGTGCCCatgcgCatGggAAAAagCcAAgnAcGGGCACTCTGAGaGGTtcAC
>Bacillus_Fam_269_52_3 Nr. of seq. 3 Alignment length(with gaps) = 54
Alignment score = 0.625514
atTTTTGAGGGTaCtatTCTGnctTtTtAGTgCCCTCAAttTTTgnTTTTcg
>Bacillus_Fam_270_52_3 Nr. of seq. 3 Alignment length(with gaps) = 54
Alignment score = 0.703704
gtGGAAATGaCCCTTCAGCAnGaTcaAAtCtGtGcGAAGGTCACAAAggCGc
>Bacillus_Fam_271_53_3 Nr. of seq. 3 Alignment length(with gaps) = 54
Alignment score = 0.600823
CGGGCACATGTtnGCCtcTCTTGTtACCgtgCtCTtgcttTTTCttgCTTtcn
>Bacillus_Fam_272_52_3 Nr. of seq. 3 Alignment length(with gaps) = 54
Alignment score = 0.633745
CTCTCTTACCATCcnACcCtcnTTtccTCnCgAAtGGGCACTGACaACnnCT
>Bacillus_Fam_273_53_3 Nr. of seq. 3 Alignment length(with gaps) = 54
Alignment score = 0.651235

aCGGTAAAtGaAaGAgcGnTtCATTtACCGAAAAGngGaAAAAnAgAGCnCTt
>Bacillus_Fam_274_52_3 Nr. of seq. 3 Alignment length(with gaps) = 54
Alignment score = 0.720165
CACAGTGCCCnnGCncGGCTTTTTTTggcctCGAcGGGCACTCTGaaCTCTT
>Bacillus_Fam_275_53_3 Nr. of seq. 3 Alignment length(with gaps) = 54
Alignment score = 0.672840
GgAACGAAAGAGgtGcATTcGTgCccgTaaGAGGnAAAAATtAGGAGAAgnCa
>Bacillus_Fam_276_53_3 Nr. of seq. 3 Alignment length(with gaps) = 54
Alignment score = 0.738683
gTGTACCgCTAGAcnCnTCtTGCGGnACATGCTCacCcTATTTCACaCCaAGC
>Bacillus_Fam_277_51_3 Nr. of seq. 3 Alignment length(with gaps) = 53
Alignment score = 0.639413
GAATAGAAgCnccCTATTCGACCGTTncACGnggTTtCacTTccTTCGGgc
>Bacillus_Fam_278_51_3 Nr. of seq. 3 Alignment length(with gaps) = 53
Alignment score = 0.635220
TGTCtGAACTTgagCTAACTTCGGACTnCttctGccTtTTTctcCtGAtCt
>Bacillus_Fam_279_51_3 Nr. of seq. 3 Alignment length(with gaps) = 53
Alignment score = 0.681342
ACTTTATtTTnGATTaTTaaTtgaAAATGTCtTTCATaAGgtcGATGAAgG
>Bacillus_Fam_280_52_3 Nr. of seq. 3 Alignment length(with gaps) = 53
Alignment score = 0.666667
TTTtGtCCTTCATcCcCCttATGAAGGACcGTTCtCTGnAttTtnCnCcTcG
>Bacillus_Fam_281_52_3 Nr. of seq. 3 Alignment length(with gaps) = 53
Alignment score = 0.752621
aGTTTCTGCTGCTAAaTCcaGTTaAgAACACTTTCAcCCGCaaACCaaCcAg
>Bacillus_Fam_282_52_3 Nr. of seq. 3 Alignment length(with gaps) = 53
Alignment score = 0.746331
cACAtGAAAAnnGGCTcATAGAAcCTnTCTATGAACCCaAATTTgAtCGAAn
>Bacillus_Fam_283_52_3 Nr. of seq. 3 Alignment length(with gaps) = 53
Alignment score = 0.658281
TTGaCTnCCGTTCTTcCnCnCnTTCtCcTTTTTCGGgAGcCAtCaCnnCtCT
>Bacillus_Fam_284_52_3 Nr. of seq. 3 Alignment length(with gaps) = 53
Alignment score = 0.618449
CCTCCATCACcCtCATGGAgGACCttTTTTCCnntTctCaCgcnAnntcGGt
>Bacillus_Fam_285_52_3 Nr. of seq. 3 Alignment length(with gaps) = 53
Alignment score = 0.907757
AATGTGTGTCCAAGAGAnGAGcTGAAACACATTATAAGAGAAACCGGAGGAg
>Bacillus_Fam_286_53_3 Nr. of seq. 3 Alignment length(with gaps) = 53
Alignment score = 0.851153
ATTGGTTACCCgGTTGcGCgGATTTCACccGgACTCGGTCACCTTTcGGCNTG
>Bacillus_Fam_287_52_3 Nr. of seq. 3 Alignment length(with gaps) = 53
Alignment score = 0.633124
TTAAAGACATTTTgagCantttgAnaGGnaGTTtTGTCtTTAAGaAgGGcTA
>Bacillus_Fam_288_52_3 Nr. of seq. 3 Alignment length(with gaps) = 53
Alignment score = 0.731656
cTTATAAGCTACCCAACTTgggcganTttCtCGGtTTCGGTcGCTTATACCc
>Bacillus_Fam_289_52_3 Nr. of seq. 3 Alignment length(with gaps) = 53
Alignment score = 0.645702
nAAgTGTtTCCAATgAaGGCTATTGGcgACAaAACTCagtangAAAanggGa
>Bacillus_Fam_290_52_3 Nr. of seq. 3 Alignment length(with gaps) = 53
Alignment score = 0.607966
TGGAatCACTTTaCTCTnTTTTnCtCcgagtTTTGTttCCAATagccntgAT
>Bacillus_Fam_291_52_3 Nr. of seq. 3 Alignment length(with gaps) = 53
Alignment score = 0.714885

```
 GCTGTCCGAAgTnGagTGAGGTTCAGACAGcTTgaGggtgagAAGaGGAAAa
>Bacillus_Fam_292_52_3  Nr. of seq. 3 Alignment length(with gaps) = 53
Alignment score = 0.704403
 aaGGTTCGGACAGCTTTtGcTcncaGGcanTCGAAGTTGTCCGAAGTnGctC
>Bacillus_Fam_293_52_3  Nr. of seq. 3 Alignment length(with gaps) = 53
Alignment score = 0.637317
 GTCTGAAGTCAccnTAgGTTCgGACaCngtTGcAttTGaAATCaCtnaTCcT
>Bacillus_Fam_294_51_3  Nr. of seq. 3 Alignment length(with gaps) = 52
Alignment score = 0.651709
 TATaAGgAAGaaATGAGtGCTAAAgcgnAgGATAGanCattATaTTaCCAG
>Bacillus_Fam_295_52_3  Nr. of seq. 3 Alignment length(with gaps) = 52
Alignment score = 0.707265
 TtAAGGAAGncCGnnAAAAAgngtcnCTaTCTTAACgTAGAggCGTTCTAnG
>Bacillus_Fam_296_52_3  Nr. of seq. 3 Alignment length(with gaps) = 52
Alignment score = 0.841880
 GGcGCGCTtaCCCtAgCGCGnGgCTCTTCTCtCTTTTTTCTTCTTCTTGGGa
>Bacillus_Fam_297_52_3  Nr. of seq. 3 Alignment length(with gaps) = 52
Alignment score = 0.675214
 AagCGTGTCATACgAgggCAATGcCaCATGAAAAAtaggaAncaAgcaAATA
>Bacillus_Fam_298_52_3  Nr. of seq. 3 Alignment length(with gaps) = 52
Alignment score = 0.688034
 AAaCGGtAACTAATcGtGagGAAAgGTtcCCGnTTtGtGcCcGagTTcGAAa
>Bacillus_Fam_299_52_3  Nr. of seq. 3 Alignment length(with gaps) = 52
Alignment score = 0.846154
 AAAGTGTTCTAAnCCAGAGTtAGCAGCACAAAgTGAGTAGAgCaCaGgCcCg
>Bacillus_Fam_300_52_3  Nr. of seq. 3 Alignment length(with gaps) = 52
Alignment score = 0.811966
 GAGTTTgTGCTGCTAAcTCaTGTtTgGAACACTTTcGcCgnGGaCCCGCACc
>Bacillus_Fam_301_52_3  Nr. of seq. 3 Alignment length(with gaps) = 52
Alignment score = 0.809829
 AAgGGTCATAAngaCaCaTGATtTAACCCTTCAGCATGAAGGAaAcTccGTG
>Bacillus_Fam_302_52_3  Nr. of seq. 3 Alignment length(with gaps) = 52
Alignment score = 0.760684
 CTGGAGTGACCGaAaAgCATntAgaAAAnGCaTtTGGGTCAATGCAGatAGt
>Bacillus_Fam_303_52_3  Nr. of seq. 3 Alignment length(with gaps) = 52
Alignment score = 0.713675
 GACtgCTTTtacGGAAAaagGCGgAAAgctGTCcGAACTTGGggcTACTTCg
>Bacillus_Fam_304_52_3  Nr. of seq. 3 Alignment length(with gaps) = 52
Alignment score = 0.675214
 AATCGTtaCCgTTTcanATGnGAnCACggCAAnAcGGtAACGAtTCaGAGcn
>Bacillus_Fam_305_52_3  Nr. of seq. 3 Alignment length(with gaps) = 52
Alignment score = 0.705128
 GGcTnATTcGGACAGaaCAAgcnagAAatGcCgnTAGAGAGTCCgAATGCAA
>Bacillus_Fam_306_52_3  Nr. of seq. 3 Alignment length(with gaps) = 52
Alignment score = 0.726496
 tGaTTGaTTACCCTGaaGatGaAAATTGCatgTGTACGGgAACCAtanaGAG
>Bacillus_Fam_307_52_3  Nr. of seq. 3 Alignment length(with gaps) = 52
Alignment score = 0.899573
 GGTAACCATTGagGGcCTTTGGTGACCCTTTnGGNGGAAAACTCCTTTCtTC
>Bacillus_Fam_308_52_3  Nr. of seq. 3 Alignment length(with gaps) = 52
Alignment score = 0.929487
 AGAAAGAGGAAGAAAGGTAACCAATCGCtCGGAAAGGTTaCCGAAACtnGAA
>Bacillus_Fam_309_52_3  Nr. of seq. 3 Alignment length(with gaps) = 52
Alignment score = 0.739316
```

TgGAtACACATTtTgngAGAATATTgcaGAtAATgTGTCTCAAGgATgGcGg
>Bacillus_Fam_310_52_3 Nr. of seq. 3 Alignment length(with gaps) = 52
Alignment score = 0.982906
CTTAAATAGAGGAAACCAgGTGTGTAGGGTAACTAAAGCGAGCGAAAGGTTA
>Bacillus_Fam_311_52_3 Nr. of seq. 3 Alignment length(with gaps) = 52
Alignment score = 0.730769
aGGTTACCGAaTCcatGAGAaATCCACCTanAGGGTAACgAAaCnaGcgcaA
>Bacillus_Fam_312_52_3 Nr. of seq. 3 Alignment length(with gaps) = 52
Alignment score = 0.649573
AAAGGnAGAAGAGaGngcGTcTCAnCGCCCAtcTGAgngAGAAnAagaGaaG
>Bacillus_Fam_313_52_3 Nr. of seq. 3 Alignment length(with gaps) = 52
Alignment score = 0.675214
TATGGGACAnttgcTTncnntTtTCnGCTtnTTTTGTCtCATTGAGCtCTCC
>Bacillus_Fam_314_52_3 Nr. of seq. 3 Alignment length(with gaps) = 52
Alignment score = 0.982906
AAAAGGGAACATAGAGTCCTTGTATGTACGCGAAACTGGTGgGAAGACTCCG
>Bacillus_Fam_315_52_3 Nr. of seq. 3 Alignment length(with gaps) = 52
Alignment score = 0.946581
GAGCAAAGAACATGTCATCATCGAnaGGATGAAGACACGAATAGAaGTGTAG
>Bacillus_Fam_316_48_3 Nr. of seq. 3 Alignment length(with gaps) = 48
Alignment score = 0.981481
TTCTTCTGATGACGTTGAGAACGACTCAGAtGCTGACAGTGACGATGA
>Bacillus_Fam_317_45_3 Nr. of seq. 3 Alignment length(with gaps) = 46
Alignment score = 0.666667
GnACAAAgCCAGAcGATAnCgAAAcAAAaCCggATGAccAAaACg
>Bacillus_Fam_318_40_3 Nr. of seq. 3 Alignment length(with gaps) = 43
Alignment score = 0.666667
AAttCGCAGATAAgTnaTGAAAacCGCAGATAACtCGtGa
>Bacillus_Fam_319_39_3 Nr. of seq. 3 Alignment length(with gaps) = 42
Alignment score = 0.722222
AtAAAAAaTCtTaCTGtTCTCACAAaAGATCnCGCAGCc
>Bacillus_Fam_320_42_3 Nr. of seq. 3 Alignment length(with gaps) = 42
Alignment score = 0.978836
GCAGAAGAGAGAAAGTAgCAGAATTAAAACAGACGAAGCAAAAT
>Bacillus_Fam_321_40_3 Nr. of seq. 3 Alignment length(with gaps) = 40
Alignment score = 0.766667
tAAGAGTATTTCcgatTTCcGAAGAGTaTcCncGCAAAGT
>Bacillus_Fam_322_39_3 Nr. of seq. 3 Alignment length(with gaps) = 39
Alignment score = 0.863248
ACaaCaCCTCCAGCaAATAAcGGAGGAGGtCAAGGAAAT
>Bacillus_Fam_323_39_3 Nr. of seq. 3 Alignment length(with gaps) = 39
Alignment score = 0.749288
TCAggggCAACAgaAAcACTCTAAgCAAGACgACTCcaA
>Bacillus_Fam_324_36_3 Nr. of seq. 3 Alignment length(with gaps) = 36
Alignment score = 0.811728
tAAGAAaCAAGAaGAgGCgAAAAAgCTAGAAGAGag
>Bacillus_Fam_325_36_3 Nr. of seq. 3 Alignment length(with gaps) = 36
Alignment score = 0.969136
AATGCAACAAGGGATGGGAGGAAACCACCAnGCCCC
>Bacillus_Fam_326_34_3 Nr. of seq. 3 Alignment length(with gaps) = 35
Alignment score = 0.615873
CGACGAGtAAtCGCAGGAGCAnacgGttttnaTG
>Bacillus_Fam_327_25_3 Nr. of seq. 3 Alignment length(with gaps) = 26
Alignment score = 0.846154

tTGTgCCTGGCACCTTTtCCCCTTT
>Bacillus_Fam_328_24_3 Nr. of seq. 3 Alignment length(with gaps) = 24
Alignment score = 0.759259
GaAaCCgCCCATGCCCATtcCnGG
>Bacillus_Fam_329_21_3 Nr. of seq. 3 Alignment length(with gaps) = 23
Alignment score = 0.661836
TTTACTGGCGGAtAnCnCCaC
>Bacillus_Fam_330_21_3 Nr. of seq. 3 Alignment length(with gaps) = 23
Alignment score = 0.642512
gttnTgtcTaTtTttntGagn
>Bacillus_Fam_331_21_3 Nr. of seq. 3 Alignment length(with gaps) = 23
Alignment score = 0.671498
GTTGGCgCAAAAgGtATCtCG
>Bacillus_Fam_332_21_3 Nr. of seq. 3 Alignment length(with gaps) = 23
Alignment score = 0.690821
TTCGCAAATAAAaCCnTcAtA
>Bacillus_Fam_333_21_3 Nr. of seq. 3 Alignment length(with gaps) = 23
Alignment score = 0.637681
TGACGGGATTnTCtcGngTTa
>Bacillus_Fam_334_23_3 Nr. of seq. 3 Alignment length(with gaps) = 23
Alignment score = 0.647343
TCnATaCgGACAaGAGcgAGcGc
>Bacillus_Fam_335_23_3 Nr. of seq. 3 Alignment length(with gaps) = 23
Alignment score = 0.743961
TATTTGCGAATTaCctnGcntTT
>Bacillus_Fam_336_20_3 Nr. of seq. 3 Alignment length(with gaps) = 22
Alignment score = 0.676768
TaTATCGGCGAtTCTCgAtT
>Bacillus_Fam_337_20_3 Nr. of seq. 3 Alignment length(with gaps) = 22
Alignment score = 0.651515
GACGCAAAAAncttgGTGGa
>Bacillus_Fam_338_21_3 Nr. of seq. 3 Alignment length(with gaps) = 22
Alignment score = 0.732323
ATCAGCCAAAGcAGnTGnGAT
>Bacillus_Fam_339_21_3 Nr. of seq. 3 Alignment length(with gaps) = 22
Alignment score = 0.772727
tTTGGCTGATTTCtCCTCCgc
>Bacillus_Fam_340_21_3 Nr. of seq. 3 Alignment length(with gaps) = 22
Alignment score = 0.616162
nnTGGATCATAAATctccAAt
>Bacillus_Fam_341_21_3 Nr. of seq. 3 Alignment length(with gaps) = 22
Alignment score = 0.727273
aCTCCTATAATAAAaAngaTA
>Bacillus_Fam_342_22_3 Nr. of seq. 3 Alignment length(with gaps) = 22
Alignment score = 0.636364
TTTGGCGGAATtnCcantnaAg
>Bacillus_Fam_343_21_3 Nr. of seq. 3 Alignment length(with gaps) = 22
Alignment score = 0.737374
TACGaGAGTAAnCgCACTCnT
>Bacillus_Fam_344_21_3 Nr. of seq. 3 Alignment length(with gaps) = 22
Alignment score = 0.747475
CTTGAGCGGTATTCncCTCcn
>Bacillus_Fam_345_21_3 Nr. of seq. 3 Alignment length(with gaps) = 22
Alignment score = 0.727273

TtAACcaCTGCTaTTCGTCgG
>Bacillus_Fam_346_21_3 Nr. of seq. 3 Alignment length(with gaps) = 22
Alignment score = 0.631313
TGTCCCATAnAaTcgAAgTnn
>Bacillus_Fam_347_18_3 Nr. of seq. 3 Alignment length(with gaps) = 21
Alignment score = 0.767196
CCAGGAgCAGGcTTTGGC
>Bacillus_Fam_348_20_3 Nr. of seq. 3 Alignment length(with gaps) = 21
Alignment score = 0.708995
GtAGGaCGAAtTATTtGcGT
>Bacillus_Fam_349_20_3 Nr. of seq. 3 Alignment length(with gaps) = 21
Alignment score = 0.687831
ACTGtCACTATnccnAAAAT
>Bacillus_Fam_350_21_3 Nr. of seq. 3 Alignment length(with gaps) = 21
Alignment score = 0.687831
tTTATTTGCGATAacTaCgtc
>Bacillus_Fam_351_21_3 Nr. of seq. 3 Alignment length(with gaps) = 21
Alignment score = 0.957672
GCCAACCACCGTGTCCgCCTT
>Bacillus_Fam_352_21_3 Nr. of seq. 3 Alignment length(with gaps) = 21
Alignment score = 0.788360
AGAACGgCGAGagCgGCGaGT
>Bacillus_Fam_353_21_3 Nr. of seq. 3 Alignment length(with gaps) = 21
Alignment score = 0.661376
ATtCCGCGAAAncnGAGnngA
>Bacillus_Fam_354_21_3 Nr. of seq. 3 Alignment length(with gaps) = 21
Alignment score = 0.756614
AACCGAGAGAtTAAgggCtGa
>Bacillus_Fam_355_21_3 Nr. of seq. 3 Alignment length(with gaps) = 21
Alignment score = 0.788360
GATGtGAGagAGAAGaGCgCG
>Bacillus_Fam_356_21_3 Nr. of seq. 3 Alignment length(with gaps) = 21
Alignment score = 0.671958
ATTCCGCCAAcTtnGGanngN
>Bacillus_Fam_357_20_3 Nr. of seq. 3 Alignment length(with gaps) = 20
Alignment score = 0.711111
GaCGCAAaTATtgTGAAact
>Bacillus_Fam_358_20_3 Nr. of seq. 3 Alignment length(with gaps) = 20
Alignment score = 0.877778
CaCaTATAAAAGCTcCAACT
>Bacillus_Fam_359_20_3 Nr. of seq. 3 Alignment length(with gaps) = 20
Alignment score = 0.788889
TATCaGGAAAAaaTtnAATA
>Bacillus_Fam_360_18_3 Nr. of seq. 3 Alignment length(with gaps) = 18
Alignment score = 0.703704
GGtGgaCCnGGnTTCGGT
>Bacillus_Fam_361_18_3 Nr. of seq. 3 Alignment length(with gaps) = 18
Alignment score = 0.746914
TCAGCAGTAGagTCnGcg
>Bacillus_Fam_362_18_3 Nr. of seq. 3 Alignment length(with gaps) = 18
Alignment score = 0.679012
TGctcATGATggTGnTCA
>Bacillus_Fam_363_16_3 Nr. of seq. 3 Alignment length(with gaps) = 16
Alignment score = 0.722222

AACCCGGGTTaaaGgg
>Bacillus_Fam_364_15_3 Nr. of seq. 3 Alignment length(with gaps) = 15
Alignment score = 0.762963
GTtTTTCTTCtTtaG
>Bacillus_Fam_365_15_3 Nr. of seq. 3 Alignment length(with gaps) = 15
Alignment score = 0.837037
TCTGgTTTtTCTACt
>Bacillus_Fam_366_15_3 Nr. of seq. 3 Alignment length(with gaps) = 15
Alignment score = 0.755556
TCTTCtTCAGTnacG
>Bacillus_Fam_367_12_3 Nr. of seq. 3 Alignment length(with gaps) = 12
Alignment score = 0.675926
TnCTTtGTtATt
>Bacillus_Fam_368_12_3 Nr. of seq. 3 Alignment length(with gaps) = 12
Alignment score = 0.666667
ATagAAnAAGGt
>Bacillus_Fam_369_12_3 Nr. of seq. 3 Alignment length(with gaps) = 12
Alignment score = 0.777778
GCTGCTgCaACg
>Bacillus_Fam_370_12_3 Nr. of seq. 3 Alignment length(with gaps) = 12
Alignment score = 0.888889
CCAGGnGAAGAA
>Bacillus_Fam_371_12_3 Nr. of seq. 3 Alignment length(with gaps) = 12
Alignment score = 0.777778
CAcCAgCACCaA
>Bacillus_Fam_372_12_3 Nr. of seq. 3 Alignment length(with gaps) = 12
Alignment score = 1.000000
CCGTAACCGCCG
>Bacillus_Fam_373_12_3 Nr. of seq. 3 Alignment length(with gaps) = 12
Alignment score = 0.777778
TGaCCtTGaCCT
>Bacillus_Fam_374_12_3 Nr. of seq. 3 Alignment length(with gaps) = 12
Alignment score = 0.777778
GAAgCaGAAgCA
>Bacillus_Fam_375_240_2 Nr. of seq. 2 Alignment length(with gaps) = 240
Alignment score = 0.920833

GGTTGGGCGAAAGTGAaTGTAAATGGGAAAACAGGaTATGTAAGTAGTCAaTATTTAACAGGCAAGACGAACA
CAGCCCCAtCAAAACCaTCaACATCCACACCAGCAAAGACGGAAACAAAATATGTATCTGTTAACTCAGGTTC
TAtTTTGAATCTACGCAGTAGTGCcAGTACAAGTGCaTCaATAATCGGGAaCcTAaCAAATGGCACAGCaGTA
ACGGTTCAATCAGATTCCAAa
>Bacillus_Fam_376_210_2 Nr. of seq. 2 Alignment length(with gaps) = 210
Alignment score = 0.853968

GGCTGGTACAGTGATGAAGCACgAACGAATTTGTACGATTTTaAtGaGCCAGTNAAAGCAGCgAcaAagTTGT
ATGCGAAATGGACcCATAATCAATATGAAGTaAaTTTTGATACNAATGGCGGcAGTNANaTTGCTTCgGAAAA
TGTGAANTACAATGAAAAAGCaANGAAGCCAgNaGtACCAACGAAAGAAGGcTACACCTTCGCN
>Bacillus_Fam_377_200_2 Nr. of seq. 2 Alignment length(with gaps) = 200
Alignment score = 0.746667

ATTGATAAAACaGCTCCAGTcGTAAcTGGaGTTAgAAaACaACGGcCTtTACAATaAaGATGTaACAgTTTCa
TTTAATGAAGGAACaGCgACATTAAATGGTAAaGaAgTgaaAaCTGGaACAGcAGTTAAaaCAAgAtGGAacc
TAcACAgTAGTGGTAACaGAcGCAGCAGGaAAcAAAACaACtGTgAAGTTCACg

>Bacillus_Fam_378_154_2 Nr. of seq. 2 Alignment length(with gaps) = 154
Alignment score = 0.696970

ACATGTGGCATCAcTTCTTcACCGCTTCTAAGAAAAaCgAAAAACgaGcTTcacTaagcTCGTTTTTaaTcaA
aacATAaGAgAGTgaTAAAAtAtGGCGGTGGAAaTGAaGCCACGCGTAGTAGaGAGAAGAGCACTCACTACTA
CTTTAAAa
>Bacillus_Fam_379_153_2 Nr. of seq. 2 Alignment length(with gaps) = 153
Alignment score = 0.644880

aATAAGTCAACATCGAaTCGCTaaCGCTCaTCGTGTTTCCTTTATcTcGATgCcAAaCCCTCCAagCCaTACa
cCcCTAAACaGgCGccTCCGCTTTTCTaTTGTCCAGCTcCacCgCCcAgGcGCTaATcGaccTtCGGccTTcT
CaCCTaC
>Bacillus_Fam_380_148_2 Nr. of seq. 2 Alignment length(with gaps) = 148
Alignment score = 0.718468

TAGAcGAaGCaACAAAAGAAAAAGCGAAaTCaATcTTaGAaCAAGAAAAaTCCGGaAaAcTAACaCGTGAaCA
AGCAAAAGAaaAATTaAaAaAaCTAGGcGTGAAATTCCCaGAGAAAGGgtAAGCAcGaaGAcATGTTcGCgAA
cT
>Bacillus_Fam_381_145_2 Nr. of seq. 2 Alignment length(with gaps) = 145
Alignment score = 0.671264

CCACCATTTATGCCACAgaTgaTCGTTGTcTCCaCgGACAAAgGTATCaATcCGaTTcGccCCCCATGATAAa
GCacCCGGtaGAaCCCTtcAAaCCaCccCGCGGaGCcCCaAGaTCcTCCCAaTCaCTCCAGCgcGAACCaTC
>Bacillus_Fam_382_140_2 Nr. of seq. 2 Alignment length(with gaps) = 140
Alignment score = 0.814286

ATCGTCCGaAAACTaTAaAACGcCGCGGGGTGGAGCAACGAGCaAaGCATCAgTGAAAAAaCAaCGAGTTGTC
TCGACGCATaaGCAgCGaAGcaAAGCAaGAAGAGGAAGAGACAGTCAAAGGACaTCGAAGCaAaAGC
>Bacillus_Fam_383_138_2 Nr. of seq. 2 Alignment length(with gaps) = 138
Alignment score = 0.990338

GTTGTCTCGGAAGCGAGGCTTCGTGAAGAGCAGGAAAATGAAGAGGAAGAAGCTGAGCGGTCAGCGCCATTGG
CTTTAAAAGCAGAGGAAAGCGGCGGTATAAAAGCAGAAGAAGCGCCAGCTGAGCCGCCaGAGCTT
>Bacillus_Fam_384_128_2 Nr. of seq. 2 Alignment length(with gaps) = 128
Alignment score = 0.695312

acCTCGAGGTCATAAGCcAAAaacaCCAAAAAGGCaAAGAaCGCCTTTCCGGtaAGTctTCaTCTTATGCTTG
TCGCccCaGAGCaAcCCgCCTCCGCTTTTCTacaTGTCCAGCTaCGGCGccTaGa
>Bacillus_Fam_385_127_2 Nr. of seq. 2 Alignment length(with gaps) = 127
Alignment score = 0.616798

TcCGCTTTTCTcTTGTCcAGCTGCAGcGCtCTaGCcCCTCGaGGTcAAATAACCTaCCTcaAaaAAGcCAAAa
AGCGGaCTTcTTGtgCaGgaAGAACATTTaCcgGTCGGaGCaGAaCAaGaGcCT
>Bacillus_Fam_386_127_2 Nr. of seq. 2 Alignment length(with gaps) = 127
Alignment score = 0.619423

cAGCTaGACAtAgCaaAGAAAAGCGGaGcCGACTGcTCaGcccCGACAAGCATAaGAccGGCTgGcGaaGCGG
CGCaGTTTGCCGCAcAGcCAGACcaaCaTATGACCTCGAGcccCaAGGAGcCaa
>Bacillus_Fam_387_127_2 Nr. of seq. 2 Alignment length(with gaps) = 127
Alignment score = 0.674541

GGGcGAAgAAATCTCGACGCTCcTaccCGCCGcAGCTaGAcaAAGAAAAGCGaAGGCGGGTTGcaCAGGAGCG
AGAATgTcTGGAGcaCCTGACAaaaGAAaTCCCGcccTTGGCTaaCGcTTGcCa

>Bacillus_Fam_388_96_2 Nr. of seq. 2 Alignment length(with gaps) = 96
Alignment score = 1.000000

GTTCGTTCGCTTCTTCTTGGTCCATTTCGCCCGGCAACGCCTCGGTTCCACTCATTACCTCTGCCTCTTCAAT
CTCTGGTTCCCCGCGTCCATACC
>Bacillus_Fam_389_84_2 Nr. of seq. 2 Alignment length(with gaps) = 84
Alignment score = 0.603175

TGGCGAGcTTgccGCtgCGaGgCGaCGaGGTTcGgacCaTACCTCGACGTACCTCTccCcTCCAGTGGCGAGG
caGccaCCaTg
>Bacillus_Fam_390_84_2 Nr. of seq. 2 Alignment length(with gaps) = 84
Alignment score = 0.904762

GGAAAAGTAACAGCaGGTTTAAATACGTTAAATaGCAAAACaGGTGAGATGCAGACTGGTATAGGGAAATTAG
TAGAcGGgTCa
>Bacillus_Fam_391_78_2 Nr. of seq. 2 Alignment length(with gaps) = 78
Alignment score = 0.914530

GAAGATCTTGaTGACCGATATGATTTGGAaGAACTTTTGGaaTCTGATGAACGATCATATgTTGACGATTGAT
AATCT
>Bacillus_Fam_392_75_2 Nr. of seq. 2 Alignment length(with gaps) = 75
Alignment score = 0.951111

CTTTCCGCCAACGATGCCGACTCATTTTTACCAGaAAACTCCGGNTCGGCCTGCCATTCATCCTGCaCCACTC
CA
>Bacillus_Fam_393_74_2 Nr. of seq. 2 Alignment length(with gaps) = 74
Alignment score = 0.689189

TTcacCTgcTTGTCaTCaGTTTgaCTGTTTGTTGCcTTTTCaTCAGcAGAACCaTTcGTTGATTTCTCTTCct
t
>Bacillus_Fam_394_67_2 Nr. of seq. 2 Alignment length(with gaps) = 67
Alignment score = 0.611940
GTCATATCTCCATCTAaTCCGTTGCTGTGATATGATatNAATgnCngTaTatttCtgtATNcgttAt
>Bacillus_Fam_395_66_2 Nr. of seq. 2 Alignment length(with gaps) = 66
Alignment score = 0.626263
TGGTAcTACTTggatGAAgcAgcTAGCGGaGCAATGaAAACAGGTTGGTTGaAagACGaaGaAAAc
>Bacillus_Fam_396_62_2 Nr. of seq. 2 Alignment length(with gaps) = 62
Alignment score = 0.618280
GGTGTAATGCAAACaGGcTGGcAcAAAGATcGGcAGcAAGtaaGTAcTACTTcGaCaaAAAc
>Bacillus_Fam_397_61_2 Nr. of seq. 2 Alignment length(with gaps) = 61
Alignment score = 0.661202
TGGTGCGATGAAaACNGGcTGGgTnTaAAcCTGGcgccAaaTGGTATTACTTAGAaAAAAa
>Bacillus_Fam_398_60_2 Nr. of seq. 2 Alignment length(with gaps) = 60
Alignment score = 0.911111
AAATGGTACTAcCTTAACGCAAACGGCGAaATGGCAACAGGCTGGAAaCTGGTTAGCGGa
>Bacillus_Fam_399_60_2 Nr. of seq. 2 Alignment length(with gaps) = 60
Alignment score = 0.811111
AAATGGTACTaCCTcCAaGCTGAcGGcgCNATGAAAACCGGCTGGCTgAGCACAAAcAAC
>Bacillus_Fam_400_60_2 Nr. of seq. 2 Alignment length(with gaps) = 60
Alignment score = 0.716667
TATTcAaGTAATACCAcTTaCCcCCaAaagaTAaCCAGCCTGTTTGCATTaCTCCgGAAC
>Bacillus_Fam_401_60_2 Nr. of seq. 2 Alignment length(with gaps) = 60
Alignment score = 0.688889
CCTGGTAaTGGaCCcTCaAAATTCGGTGGaTATGGgaaaTTCCAacCTCCaCCaTTTGGa

>Bacillus_Fam_402_57_2 Nr. of seq. 2 Alignment length(with gaps) = 57
Alignment score = 0.625731
cGGCTTCATGAAGCacTtCATGGCGACCgTTTTCagCCggCTaCcCtTCgaTTTTTg
>Bacillus_Fam_403_57_2 Nr. of seq. 2 Alignment length(with gaps) = 57
Alignment score = 0.605263
aGATGAAGACCGcTTTAtGcAccGGAtccAgGCtaGAGGAAAGGTTcTCATAGCgAG
>Bacillus_Fam_404_57_2 Nr. of seq. 2 Alignment length(with gaps) = 57
Alignment score = 0.684211
AacAAGAAAGGTCACCAAcAAGGCcCATTGGTGCCCAAAGTgggAAGaGTcTGctag
>Bacillus_Fam_405_56_2 Nr. of seq. 2 Alignment length(with gaps) = 56
Alignment score = 0.636905
ATGAAGACCTGtcaAtGGaGGAAtttgaAGTaGGAAGAGatGTCaTCATAAGGTGG
>Bacillus_Fam_406_56_2 Nr. of seq. 2 Alignment length(with gaps) = 56
Alignment score = 0.622024
TCTCTcTTACCGtTTTTCCTgTTTcccGAAtcGcagTTCGGGCACaGAGAcCGCCt
>Bacillus_Fam_407_56_2 Nr. of seq. 2 Alignment length(with gaps) = 56
Alignment score = 0.601190
GTTCCCgTCAgGCTCtgacTTTTCtCTgCTGACGGGCACTTcCtGCtcaCAacTTA
>Bacillus_Fam_408_56_2 Nr. of seq. 2 Alignment length(with gaps) = 56
Alignment score = 0.669643
aCTTCGGACAGCTTTaGCtTcTTTcAGaGCttcAAAGCTGTCTGAACtCTGGaCTc
>Bacillus_Fam_409_56_2 Nr. of seq. 2 Alignment length(with gaps) = 56
Alignment score = 0.625000
TTTGTTCAATAGAcAGaCcTCTATcGGACAAActCTATcGATtCaaGcCTATccGT
>Bacillus_Fam_410_56_2 Nr. of seq. 2 Alignment length(with gaps) = 56
Alignment score = 0.601190
TaTaGaGGTTGcCGATTGaagGTcgTGaGAGGAATCgGaGGTaGTCAAAGAGaGCC
>Bacillus_Fam_411_55_2 Nr. of seq. 2 Alignment length(with gaps) = 55
Alignment score = 0.654545
cTCAAGACACTTTccTcaGCtaATTTTAcCCgGTTcATGTCCTTGAGAcaCcTCC
>Bacillus_Fam_412_55_2 Nr. of seq. 2 Alignment length(with gaps) = 55
Alignment score = 0.624242
CTTCATCAaCCtCGATGAAGGACTTTCCacCgAgcAaTcTaCgCTcCATTCGaGA
>Bacillus_Fam_413_55_2 Nr. of seq. 2 Alignment length(with gaps) = 55
Alignment score = 0.633333
CacAAaAAATGGgAGcTAATCGaAATGAATAACGACGAAAAAGaaacCTcTacnc
>Bacillus_Fam_414_55_2 Nr. of seq. 2 Alignment length(with gaps) = 55
Alignment score = 0.639394
TTTTGTCTTTAATAAGGGcTCTTAAAGACaaAaaTcCAGcGAAAgGaaCATGcag
>Bacillus_Fam_415_55_2 Nr. of seq. 2 Alignment length(with gaps) = 55
Alignment score = 0.642424
TTCATAAAGCCCGtcGaCaGagGAAAAAAGccGtCaaAgCGGTCAATAAGAGaGG
>Bacillus_Fam_416_55_2 Nr. of seq. 2 Alignment length(with gaps) = 55
Alignment score = 0.624242
TTTGTCcaATAGAGCCTTTCTATcGGACActTTTcTCCGaTTcTTgCaCgTcCcT
>Bacillus_Fam_417_55_2 Nr. of seq. 2 Alignment length(with gaps) = 55
Alignment score = 0.636364
AaTTGCTCCagCTGATTTATaaGGaACCATaaTTGacgCTTTTTGaTaCCCcATA
>Bacillus_Fam_418_55_2 Nr. of seq. 2 Alignment length(with gaps) = 55
Alignment score = 0.624242
CCTcCATTCGGACTCaGAgAcAcCaCTTgCtCTCgCTcgGcCTGTCCGAATCAGa
>Bacillus_Fam_419_55_2 Nr. of seq. 2 Alignment length(with gaps) = 55
Alignment score = 0.669697
tTCAAAAGACCAAGAGGAAGGaAAAAcCaGaaGtCGAGATGgcCTTTATaaGGGa

>Bacillus_Fam_420_55_2 Nr. of seq. 2 Alignment length(with gaps) = 55
Alignment score = 0.627273
GGTAACAATAGaacaAGTATcGTGCCCGAAATCAAGcccGtAAGaAGGcAAaAac
>Bacillus_Fam_421_55_2 Nr. of seq. 2 Alignment length(with gaps) = 55
Alignment score = 0.630303
GGggATGAAGGACgaaAggaCGAaGAGAGAAcngCGAcGAAaAGTCCTTCATCGA
>Bacillus_Fam_422_55_2 Nr. of seq. 2 Alignment length(with gaps) = 55
Alignment score = 0.624242
TTTCGGACTCAaCAggcTaaaAAAgTATGCaCgCggcTGTCCGAATCAAGcGCTC
>Bacillus_Fam_423_55_2 Nr. of seq. 2 Alignment length(with gaps) = 55
Alignment score = 0.651515
TcTGTCCTTTAGcACGCTcTCTCTcaGACAtCTTcCTCCCTgaTTCTcaCCCACt
>Bacillus_Fam_424_55_2 Nr. of seq. 2 Alignment length(with gaps) = 55
Alignment score = 0.612121
ACCgaaaaTaTCAtAgagTTcCCTCTTTTTCGGGCAATGCAACCCaCTccCATTa
>Bacillus_Fam_425_55_2 Nr. of seq. 2 Alignment length(with gaps) = 55
Alignment score = 0.603030
gATGAAGGACaTTTcGcCATgGGaAACCaaCcTCGaTaaGGgCTTCATCgACGCC
>Bacillus_Fam_426_55_2 Nr. of seq. 2 Alignment length(with gaps) = 55
Alignment score = 0.657576
gAAACAGGACANgAAACCCCAGTAAcTGGaAAATaGaGAGaCGtGtAAAtGAGaG
>Bacillus_Fam_427_55_2 Nr. of seq. 2 Alignment length(with gaps) = 55
Alignment score = 0.690909
aaCGAGcAGaAGAAaAAAtgaGAaAACGGTAACTATaaAGCGTTCATaATGCCCG
>Bacillus_Fam_428_55_2 Nr. of seq. 2 Alignment length(with gaps) = 55
Alignment score = 0.636364
AAAAAGagCAAGAAccTGcCGACcAAAGctCGTGacaTCGACATGAaCAGaGcGA
>Bacillus_Fam_429_55_2 Nr. of seq. 2 Alignment length(with gaps) = 55
Alignment score = 0.660606
CCCTTATGCaGaGgAAaagGTCacCTATAAGGGTACAATTAgaGAGcGAATCaTA
>Bacillus_Fam_430_55_2 Nr. of seq. 2 Alignment length(with gaps) = 55
Alignment score = 0.660606
ATTGGTGACCTTTCTTCaaCtTTCAcCcACCtAcaTAGGGCACCAATcAaCCtcC
>Bacillus_Fam_431_54_2 Nr. of seq. 2 Alignment length(with gaps) = 54
Alignment score = 0.669753
gCGCGGTGCAgaAAAGAGGAGaAAaGAAGaGAGAAcAGCaCCGCGCGaGccGaG
>Bacillus_Fam_432_54_2 Nr. of seq. 2 Alignment length(with gaps) = 54
Alignment score = 0.743827
aATGAAGACAcCTCTCaCTCCtAaTTTCACCTCACgcACGTCTTCATACCgTCg
>Bacillus_Fam_433_54_2 Nr. of seq. 2 Alignment length(with gaps) = 54
Alignment score = 0.629630
GGtaAACATTGTCCTCGTAGGaGTGtcAATGAaGCCcTcaaCCGaTGaAAAAGa
>Bacillus_Fam_434_54_2 Nr. of seq. 2 Alignment length(with gaps) = 54
Alignment score = 0.771605
TCTTACCTTCACACTaTTTTTTaTTcGCTCAAGGGCAGATGGcaGACtgCgCCA
>Bacillus_Fam_435_54_2 Nr. of seq. 2 Alignment length(with gaps) = 54
Alignment score = 0.654321
TTTCCTTTCTaAcGCaCgTTTCaTCcTcAGAAAGGAATaCTAgACTCcTcTcGC
>Bacillus_Fam_436_54_2 Nr. of seq. 2 Alignment length(with gaps) = 54
Alignment score = 0.617284
TTCATgaAGaCCAAAAagAaaGTGGAaaTcaAGaTTAGGGTGTCTCATGAAaGc
>Bacillus_Fam_437_54_2 Nr. of seq. 2 Alignment length(with gaps) = 54
Alignment score = 0.635802
gTTaGACCCgGTGGAATccaAgAAAaAAGAgAATCGaCCGgGTTAAAgAGGtTG

>Bacillus_Fam_438_54_2 Nr. of seq. 2 Alignment length(with gaps) = 54
Alignment score = 0.635802
AAAAAGtacccAGnCGGTaACTgAAACaAGtGAAGTAGTGACCAAAGAGaGtGa
>Bacillus_Fam_439_54_2 Nr. of seq. 2 Alignment length(with gaps) = 54
Alignment score = 0.611111
TGTCCGAATCaCAcCTAcTaTTCaGACTCcacCTTcGcTTTTcCGCtCaACaTC
>Bacillus_Fam_440_54_2 Nr. of seq. 2 Alignment length(with gaps) = 54
Alignment score = 0.660494
TTTcCTGTCCGAACTgGGcCTcAGTTCGGACaCaAtTTTAGCagTTgCTGcGga
>Bacillus_Fam_441_54_2 Nr. of seq. 2 Alignment length(with gaps) = 54
Alignment score = 0.626543
AGaAAATAgCaGGccGGAATGGCGTTCATagGGgaaATGAAAGCcATcTTGaGa
>Bacillus_Fam_442_54_2 Nr. of seq. 2 Alignment length(with gaps) = 54
Alignment score = 0.617284
GCGTTCATCCCcCTtATGAACGcCATcaCaatTCAAnTTTTcCAtCCtGgGATa
>Bacillus_Fam_443_54_2 Nr. of seq. 2 Alignment length(with gaps) = 54
Alignment score = 0.641975
AGGTAACAGAGAGaaCTcCTCAGTGaCCGTaaGCAcGaAAAacAAAggAGcAAA
>Bacillus_Fam_444_54_2 Nr. of seq. 2 Alignment length(with gaps) = 54
Alignment score = 0.654321
aAcTTCGGACAGaTTgcGGACCCcaaTagaTCAAAAGCTGTCCGAAcTCAGAgC
>Bacillus_Fam_445_54_2 Nr. of seq. 2 Alignment length(with gaps) = 54
Alignment score = 0.694444
AAAAAAGaaGGAGaCGGgCACTgTaGAGaGGTTCAgAGTGCCCGTgGaAGCTGa
>Bacillus_Fam_446_54_2 Nr. of seq. 2 Alignment length(with gaps) = 54
Alignment score = 0.604938
TaGACCCTTCAGaTcTaTgcTGGTcTgCccTGAAGaGTCATATGcgaTCCcGTT
>Bacillus_Fam_447_54_2 Nr. of seq. 2 Alignment length(with gaps) = 54
Alignment score = 0.629630
GTCTATGAaCCCaAAAAtAaCaGAAAccCCGcAAAAAgCGGTcCtATAGAAaTc
>Bacillus_Fam_448_54_2 Nr. of seq. 2 Alignment length(with gaps) = 54
Alignment score = 0.620370
TTgGGTTCCCTTcaAacGgaaAAAAGcTGAGTTcgaGGGAATCAAAAGaGAGTc
>Bacillus_Fam_449_54_2 Nr. of seq. 2 Alignment length(with gaps) = 54
Alignment score = 0.654321
TAaTCcTCTcTCAagCTCTcTTTTCGGGcgaCCAaCCTTCCtCTTTGaCTACCG
>Bacillus_Fam_450_54_2 Nr. of seq. 2 Alignment length(with gaps) = 54
Alignment score = 0.654321
GGGgGACTTCGGACAAATTgaaGcCTCtTCCagCaGaTgcTTTGTCCGAACCTG
>Bacillus_Fam_451_54_2 Nr. of seq. 2 Alignment length(with gaps) = 54
Alignment score = 0.669753
tGGAAATGTCTGAAGTAGCCcCAGGTTCaGCCAAAAcAaCgtCAcTaGgaaCGa
>Bacillus_Fam_452_54_2 Nr. of seq. 2 Alignment length(with gaps) = 54
Alignment score = 0.611111
AAAaACCAGaAAcTaGGgAAAacAGgaCACCAATaaAGCCGtATTGGaGCaCgA
>Bacillus_Fam_453_54_2 Nr. of seq. 2 Alignment length(with gaps) = 54
Alignment score = 0.648148
aTCAGtGACCacTTTTcGGAGcgGGcCgGCTCATTTTGGGcACTGACAGCccaT
>Bacillus_Fam_454_54_2 Nr. of seq. 2 Alignment length(with gaps) = 54
Alignment score = 0.663580
TGTCAGTGCCcGTTTTTTcgaGtGaGGAgcGagTTTTGGGTACTGAGGgGaGTc
>Bacillus_Fam_455_54_2 Nr. of seq. 2 Alignment length(with gaps) = 54
Alignment score = 0.617284
aaCCAAAAccAAccTGaGcGAGCAcAggAAAAAGGTCATCATAAaGcCGATGAa

>Bacillus_Fam_456_54_2 Nr. of seq. 2 Alignment length(with gaps) = 54
Alignment score = 0.604938
GGTAACCAaAGGnAAGCaTaTGaTGCCCGTaAAcGagGAAAAtAGtcGaTCaAC
>Bacillus_Fam_457_54_2 Nr. of seq. 2 Alignment length(with gaps) = 54
Alignment score = 0.632716
TCTTGTTACCGcGAACaCaCTcTTTTCTaCTTTCagGGGcACATGacaCAtCac
>Bacillus_Fam_458_54_2 Nr. of seq. 2 Alignment length(with gaps) = 54
Alignment score = 0.635802
TGTCCTTCATAGacGgTaATGAAGGACAAaTTcGaAaaAAAgAATAaGcAaaGa
>Bacillus_Fam_459_54_2 Nr. of seq. 2 Alignment length(with gaps) = 54
Alignment score = 0.722222
CTcTATGTTACCGTaaACcgCtTTTTTCTTcgCTcCACGGTCACTTAgAAGCCT
>Bacillus_Fam_460_54_2 Nr. of seq. 2 Alignment length(with gaps) = 54
Alignment score = 0.608025
tGgCCTTCATTCCaCTgATGAAgGACaaTTTTCaCaCGAgcCACAACCaTggTa
>Bacillus_Fam_461_54_2 Nr. of seq. 2 Alignment length(with gaps) = 54
Alignment score = 0.617284
TgCTCTCaCTTTTcGGcTTTCATCAaccTcaTGaACACCAtCTTTcTcCTTcCC
>Bacillus_Fam_462_54_2 Nr. of seq. 2 Alignment length(with gaps) = 54
Alignment score = 0.608025
aACGTTCTTCATCaGGaggATGAAAGACGTTAcGGaGcGAtaTAaaTGAttGaA
>Bacillus_Fam_463_54_2 Nr. of seq. 2 Alignment length(with gaps) = 54
Alignment score = 0.709877
aTCGAAGTAGAcAGtacTCTACTTCAACaTTCaaGcTCtAAAATCCAGGGAAAC
>Bacillus_Fam_464_54_2 Nr. of seq. 2 Alignment length(with gaps) = 54
Alignment score = 0.682099
TTTTTTCCgGgCcaCGGGCACTATGAACCTcTTATAGTGCCCatGaaCGtcTCt
>Bacillus_Fam_465_54_2 Nr. of seq. 2 Alignment length(with gaps) = 54
Alignment score = 0.604938
TGCTGTCTgaAAAGtcaAAAATccAaTaTAGAGAgCAAGTAGAAaccCgCTAGc
>Bacillus_Fam_466_54_2 Nr. of seq. 2 Alignment length(with gaps) = 54
Alignment score = 0.703704
cTATAGGCGACCGAGAGaGCGaGaAAAAAAGaGAggAcGGGTAGCGTAgAAGGa
>Bacillus_Fam_467_54_2 Nr. of seq. 2 Alignment length(with gaps) = 54
Alignment score = 0.697531
gtTccAATTTTaaCTaaAGTATTGTCGcCAATAcCcGCcATTGGAAACACTTTG
>Bacillus_Fam_468_54_2 Nr. of seq. 2 Alignment length(with gaps) = 54
Alignment score = 0.611111
ATCGCCCGTTCGccGcccAAAGnTacAgCAGAAGGTaGAAgAGAAGcGTgcCTC
>Bacillus_Fam_469_54_2 Nr. of seq. 2 Alignment length(with gaps) = 54
Alignment score = 0.623457
AaAGTCCaTCATaaaGgTGATGATGGACAAAACGcGGTcaGAAAaCggAAAagc
>Bacillus_Fam_470_54_2 Nr. of seq. 2 Alignment length(with gaps) = 54
Alignment score = 0.679012
GGAGGaAccAaTCCGTGTccAGGGTAACCAAAGAaCGTGaTAGATTaCCCTaaa
>Bacillus_Fam_471_54_2 Nr. of seq. 2 Alignment length(with gaps) = 54
Alignment score = 0.604938
GTTTTGTCTaATAgGAgCGCtTcCTATcaGACAAACTaacCTCcCaGaCCaCCT
>Bacillus_Fam_472_54_2 Nr. of seq. 2 Alignment length(with gaps) = 54
Alignment score = 0.666667
GCcGCTTCtCAAggCTacTTTCGAaCACGATTAGCTcCTaTTCgTTaACGTaCA
>Bacillus_Fam_473_54_2 Nr. of seq. 2 Alignment length(with gaps) = 54
Alignment score = 0.654321
TcGAAAAaaAGcTAAaCGacCCAAcGAGAAaGGGAAACCaAGcGGATGTaGGtG

```
>Bacillus_Fam_474_54_2  Nr. of seq. 2 Alignment length(with gaps) = 54
Alignment score = 0.635802
 TGaAATGATGTaCCGCGcCTGaCtGaGCGCATGaCATCATTaTGaaaTTCcTga
>Bacillus_Fam_475_54_2  Nr. of seq. 2 Alignment length(with gaps) = 54
Alignment score = 0.709877
 ATGTTGGTCTGaTTAGGAgcTaaTCAGgCCAaTGAGAcTCAATaTTCgAAGGaG
>Bacillus_Fam_476_54_2  Nr. of seq. 2 Alignment length(with gaps) = 54
Alignment score = 0.827160
 TTATCGGCgTGATTAGAACCAcCAAgGGAaAGGaAGAAGCATGCTTGAGGGTAc
>Bacillus_Fam_477_54_2  Nr. of seq. 2 Alignment length(with gaps) = 54
Alignment score = 0.913580
 GACTTGTGGCACCACTAGGCGGAGTGAcACCACTTTTCACTCTTCCAnCCAcCG
>Bacillus_Fam_478_54_2  Nr. of seq. 2 Alignment length(with gaps) = 54
Alignment score = 0.669753
 aTGAAGGACAAAAcgAAGcAAGAGAAaGAtAaaAAgTGTCCTTCAcaAAGGgTa
>Bacillus_Fam_479_54_2  Nr. of seq. 2 Alignment length(with gaps) = 54
Alignment score = 0.657407
 caaTTTGaAGGTcCACcATATaCCAaTAATTAGGAGAATaGTGCCaCTCcAatt
>Bacillus_Fam_480_54_2  Nr. of seq. 2 Alignment length(with gaps) = 54
Alignment score = 0.790123
 TCATAGTGaCCaTCCGAGaAGGAAAcCAGGTGAGaaCGGTCACTATgaAAGAGC
>Bacillus_Fam_481_54_2  Nr. of seq. 2 Alignment length(with gaps) = 54
Alignment score = 0.882716
 TATAACAAATaTAGTAGCaGAaCGAGGAATCGTaTCcCCAGCACCAGTAAATAG
>Bacillus_Fam_482_53_2  Nr. of seq. 2 Alignment length(with gaps) = 53
Alignment score = 0.672956
 AGGACgATaAAAaAAGcAAAAgcaAAATTAAAGTaCTATAgAaAGGTTTcTAA
>Bacillus_Fam_483_53_2  Nr. of seq. 2 Alignment length(with gaps) = 53
Alignment score = 0.676101
 aaACcCCAAaTTATCGTCCTTTaAAAcGGCtTCTATAaGACaTTAATcTaCTc
>Bacillus_Fam_484_53_2  Nr. of seq. 2 Alignment length(with gaps) = 53
Alignment score = 0.625786
 tTCATTACcTATcCcTgacTATTCGCACCTTAATTTgGCAcgTTCgCCgTggT
>Bacillus_Fam_485_53_2  Nr. of seq. 2 Alignment length(with gaps) = 53
Alignment score = 0.685535
 TgTTTTGTCCAATAGACCGCccCTATCGaACAAaTTcTTTCCcaAaACacGCT
>Bacillus_Fam_486_53_2  Nr. of seq. 2 Alignment length(with gaps) = 53
Alignment score = 0.635220
 AAAaaGGAGaaaCaGAAAAATaGGTAGTTAAGccAgGCgTTTAaAACgCCCGA
>Bacillus_Fam_487_53_2  Nr. of seq. 2 Alignment length(with gaps) = 53
Alignment score = 0.679245
 TGAAGGACaTTTTaAcGtGccCcTgGCTCgCGcTTTGTCCTTCATTaaCCCGA
>Bacillus_Fam_488_53_2  Nr. of seq. 2 Alignment length(with gaps) = 53
Alignment score = 0.613208
 cTTCCTTAACGTAGAaCCaCTCTATGTgAAcaTAtcACcGcAgaTTTCagCTg
>Bacillus_Fam_489_53_2  Nr. of seq. 2 Alignment length(with gaps) = 53
Alignment score = 0.628931
 AaTGTCTTTTACAAcGGcTCAAAAaGACAAAAGcGgTcCAccTTgCCgaccGA
>Bacillus_Fam_490_53_2  Nr. of seq. 2 Alignment length(with gaps) = 53
Alignment score = 0.622642
 GtcAGAGAGGTCTTcATaGCacTTATGAaGACaTCaGtTTGGaGAGAAAgGac
>Bacillus_Fam_491_53_2  Nr. of seq. 2 Alignment length(with gaps) = 53
Alignment score = 0.672956
 TCTCTGTTACCGtaaCcCcacTGTTgcTCGGcGcTTCGGGCTTTGAGAcGCCC
```

>Bacillus_Fam_492_53_2 Nr. of seq. 2 Alignment length(with gaps) = 53
Alignment score = 0.638365
 TTCGGTCaaATAGAAGGGCTCTATAaGCCcaAAGaGcAAcTgAgAcccGGAGc
>Bacillus_Fam_493_53_2 Nr. of seq. 2 Alignment length(with gaps) = 53
Alignment score = 0.660377
 ATGAAGACATCaGTaGcgcTaAaaACaGcTaGAGAGAGGTCTTGAGAAGaGGa
>Bacillus_Fam_494_53_2 Nr. of seq. 2 Alignment length(with gaps) = 53
Alignment score = 0.660377
 AGcgaATGAAGACATcAGTGaAcCaGGAAaCtGAGtgAGAGAaGTCTTCATGa
>Bacillus_Fam_495_53_2 Nr. of seq. 2 Alignment length(with gaps) = 53
Alignment score = 0.647799
 aGGTcCTCATAAGgcTgATGAAGACaTGAGtTGGTTgGaATaTTaTgCGAaAG
>Bacillus_Fam_496_53_2 Nr. of seq. 2 Alignment length(with gaps) = 53
Alignment score = 0.713836
 GCTAaTTTAaTTTGCTGCCCTTTCTTATGCTgTTcCcaaCTTacAATgGGCTc
>Bacillus_Fam_497_53_2 Nr. of seq. 2 Alignment length(with gaps) = 53
Alignment score = 0.698113
 TTCATCgGGCTcTATGATagaCATTTcGAGCCaaAaTTTTGcCTGTGATaGCG
>Bacillus_Fam_498_53_2 Nr. of seq. 2 Alignment length(with gaps) = 53
Alignment score = 0.672956
 CCACTTTTcCCTccTaCTgACgGaTTTTcTGGCTTTCATCccCTccATGAACA
>Bacillus_Fam_499_53_2 Nr. of seq. 2 Alignment length(with gaps) = 53
Alignment score = 0.622642
 CGaGaAcGcAaGAAAAAGagAAAaaaCGGTAAcTAGTaGAGGCGaCTAaTGCC
>Bacillus_Fam_500_53_2 Nr. of seq. 2 Alignment length(with gaps) = 53
Alignment score = 0.823899
 GTCACGTAGAGcCTGTCTACGTTACCCAaATCcAGcTCtTTTCAATGTTTaCG
>Bacillus_Fam_501_53_2 Nr. of seq. 2 Alignment length(with gaps) = 53
Alignment score = 0.757862
 AATAGAACCATCAAAAcCGaaAATcAaaAGCTTGATGGCNCTAAaCAGGCnGt
>Bacillus_Fam_502_53_2 Nr. of seq. 2 Alignment length(with gaps) = 53
Alignment score = 0.710692
 TCTATGAgCCCaAAAaTCaaGTGnAAaGAgCAAAAATgGGTTcATAGAaCGAA
>Bacillus_Fam_503_53_2 Nr. of seq. 2 Alignment length(with gaps) = 53
Alignment score = 0.672956
 TGACAGcTTTgaTGaTGaaTGAgGaCAAAGCTGTCATGATTaCaCaGTTATTc
>Bacillus_Fam_504_53_2 Nr. of seq. 2 Alignment length(with gaps) = 53
Alignment score = 0.679245
 gCggaaTCTTGACAGCTTTGGCCCCGgATCCagaAAAcCTGTCACAAaAACtA
>Bacillus_Fam_505_53_2 Nr. of seq. 2 Alignment length(with gaps) = 53
Alignment score = 0.723270
 ATGACGGACACTTTTCcTCTCAAtTaTCGcaaTTTCTGTCCGTCATCacGgcT
>Bacillus_Fam_506_53_2 Nr. of seq. 2 Alignment length(with gaps) = 53
Alignment score = 0.622642
 GACAgaTccTTaGGATacTGtCCccCTGTTTGgCCGATAGAcCCCTTCTAacG
>Bacillus_Fam_507_53_2 Nr. of seq. 2 Alignment length(with gaps) = 53
Alignment score = 0.610063
 aAAaaCaGGgaACCAATACAGCcTATTGacGACCTaAaTcCTCcAAAATaCGa
>Bacillus_Fam_508_53_2 Nr. of seq. 2 Alignment length(with gaps) = 53
Alignment score = 0.915094
 TCTCAGTGCCCGAAATGTCTCCTGCCACTACAAAcAGGgCACTGACAcCTCCt
>Bacillus_Fam_509_53_2 Nr. of seq. 2 Alignment length(with gaps) = 53
Alignment score = 0.628931
 TGTCCGAAGAGAGaGcAacTTCGGACAgGAAAacCGGaacAAgAGCgAaAAGc

>Bacillus_Fam_510_53_2 Nr. of seq. 2 Alignment length(with gaps) = 53
Alignment score = 0.622642
AGTTCGGACAAGAtaTcAcggGAAgAAGCacAAAGcaGTCCGAAGaaGGCaCA
>Bacillus_Fam_511_53_2 Nr. of seq. 2 Alignment length(with gaps) = 53
Alignment score = 0.650943
aAaAGGTCaTCATCAAGcTaATGAAGAcCCGAAAGCAtcTccAaaGCaaAGAA
>Bacillus_Fam_512_53_2 Nr. of seq. 2 Alignment length(with gaps) = 53
Alignment score = 0.628931
GACTTCaGACaaTcgTTCAgCcTTTTCCTaTcacACCTGTCTGAACTcGGaTc
>Bacillus_Fam_513_53_2 Nr. of seq. 2 Alignment length(with gaps) = 53
Alignment score = 0.716981
GTCCTTCATCCCtCCGATGAAcGACATCTcaaGCtTTTCCTcTcGcCcgTTTT
>Bacillus_Fam_514_53_2 Nr. of seq. 2 Alignment length(with gaps) = 53
Alignment score = 0.663522
gTTcTTcTCATCTTCCCGCATCcCTCgGaTTCGAGCAcTTCGCcacgGaTCcC
>Bacillus_Fam_515_53_2 Nr. of seq. 2 Alignment length(with gaps) = 53
Alignment score = 0.613208
CTCCTcATCTTCCCGcATCCgATccTTGCGGGaAcTTcAgacGGnGaTcTCTt
>Bacillus_Fam_516_53_2 Nr. of seq. 2 Alignment length(with gaps) = 53
Alignment score = 0.622642
CGAATcGGaTGGATaCGGGAACTTGagAGcAaGAaAcccGaAGtcGAAGTGCC
>Bacillus_Fam_517_53_2 Nr. of seq. 2 Alignment length(with gaps) = 53
Alignment score = 0.647799
AcTAGTGGAATcaGTGAACTAAacgagCaTTTAGaACATatGtAaTTCCAGAA
>Bacillus_Fam_518_53_2 Nr. of seq. 2 Alignment length(with gaps) = 53
Alignment score = 0.710692
TGTCCTTCATAAgGcAGATGAAGGACATTTaTcTTGGtAAaaCAagaGaGATT
>Bacillus_Fam_519_53_2 Nr. of seq. 2 Alignment length(with gaps) = 53
Alignment score = 0.672956
GTAACGTAGAGCcCGTCTACGTGCCCGAacaGAtGgAtaaTTTaGCaGTaAGG
>Bacillus_Fam_520_53_2 Nr. of seq. 2 Alignment length(with gaps) = 53
Alignment score = 0.874214
TTCCTGAAATCTGTGGCTTCCgCTnCTTTTCGGGAATGACTCGCCTTGGGgcA
>Bacillus_Fam_521_53_2 Nr. of seq. 2 Alignment length(with gaps) = 53
Alignment score = 0.773585
CTGTATGACACGCAaAATCaTGTcTCCaTTaGaATgTTCGCGTcTCATTCGTG
>Bacillus_Fam_522_53_2 Nr. of seq. 2 Alignment length(with gaps) = 53
Alignment score = 0.672956
GGTAACTATGaAaGCGtTCATAGTGACCaAAcccAAagAGAAAAAGCaAaAaC
>Bacillus_Fam_523_53_2 Nr. of seq. 2 Alignment length(with gaps) = 53
Alignment score = 0.751572
TGTCCTCAAGaAGgGcTTATGAGGACAAACTcGaGTccGAAGCGTaGgCGAAn
>Bacillus_Fam_524_53_2 Nr. of seq. 2 Alignment length(with gaps) = 53
Alignment score = 0.855346
AGGTCATCATGGAGGTGATGAAGACTCtGAAGCgGTGTTGcTTGGaaGgGAAG
>Bacillus_Fam_525_53_2 Nr. of seq. 2 Alignment length(with gaps) = 53
Alignment score = 1.000000
CTCTCTTGAGTGACTCATATCGGTTCTCTGAGGCACTCTGGGGCGGGGGGTCT
>Bacillus_Fam_526_53_2 Nr. of seq. 2 Alignment length(with gaps) = 53
Alignment score = 0.974843
GTACAGAGTGCCTCAAACGAAGTCTATGAAGCACTCTAaAGATGGAAAGGAGC
>Bacillus_Fam_527_53_2 Nr. of seq. 2 Alignment length(with gaps) = 53
Alignment score = 0.943396
CATGTATCCAGTAGCTTTTTCGGGATACATGACACCNCNTTTTTTTNCTCGGT

>Bacillus_Fam_528_53_2 Nr. of seq. 2 Alignment length(with gaps) = 53
Alignment score = 0.654088
AATGAcaCCCCGCGCGgcCGaAGCGCATGtCaTCATTgTGAacaTTaaGAgGA
>Bacillus_Fam_529_53_2 Nr. of seq. 2 Alignment length(with gaps) = 53
Alignment score = 0.622642
CTTATGACTTTCccaCcTAAaGTCgGAagCTaGTTgcCcgcTCCCGCTCTTTc
>Bacillus_Fam_530_53_2 Nr. of seq. 2 Alignment length(with gaps) = 53
Alignment score = 0.930818
AAATAGCATAAGATAAGCAaGGAATCGTACGCTATAATCTcaAGCCAGTGTAC
>Bacillus_Fam_531_53_2 Nr. of seq. 2 Alignment length(with gaps) = 53
Alignment score = 0.811321
TGGTTCTAAAcCGaGTTAATAGTGCCATCAAAGGaggGGGaTTGGgAGcTTGA
>Bacillus_Fam_532_53_2 Nr. of seq. 2 Alignment length(with gaps) = 53
Alignment score = 0.880503
TGtTGCCACTTTaCTTCACcCATGaAGCCTGTTTGTGGCGCAACTCACCGGAg
>Bacillus_Fam_533_53_2 Nr. of seq. 2 Alignment length(with gaps) = 53
Alignment score = 0.628931
GCGGTACAcGCTcGcaAaAAATcCGCcaaAGCGTGTACCGcaAGACcacTCTa
>Bacillus_Fam_534_52_2 Nr. of seq. 2 Alignment length(with gaps) = 52
Alignment score = 0.826923
CAAAAGGACAATTCaGGNAGAaACAAAGaGCAAAcTGTCCTTTAgAGnGGTT
>Bacillus_Fam_535_52_2 Nr. of seq. 2 Alignment length(with gaps) = 52
Alignment score = 0.743590
TTTTCATTACCTATTATgaaGcTaTaCGCACCCTAAcCTTGGCTTTGCTaGa
>Bacillus_Fam_536_52_2 Nr. of seq. 2 Alignment length(with gaps) = 52
Alignment score = 0.602564
GTAATGAAATTactCGacTcaCcTGAtTTTCAgTaCcTATTACTCctgATTG
>Bacillus_Fam_537_52_2 Nr. of seq. 2 Alignment length(with gaps) = 52
Alignment score = 0.641026
TCAcCAAAtTCGCTgaaaaaTCCgTTTTGCTGATaATAAAaCcGATTTACcA
>Bacillus_Fam_538_52_2 Nr. of seq. 2 Alignment length(with gaps) = 52
Alignment score = 0.692308
ccAAGAAGcGTTCAaaACGCCCGaaATTTcaAAAAGAAgGAAAAATcGGTAG
>Bacillus_Fam_539_52_2 Nr. of seq. 2 Alignment length(with gaps) = 52
Alignment score = 0.698718
ACTTTTTcCTagTGAaCTAcTCgATTTTGTCcTTCATcAGCnCgcATGAAAG
>Bacillus_Fam_540_52_2 Nr. of seq. 2 Alignment length(with gaps) = 52
Alignment score = 1.000000
AGAGGGTGTTAGTGGTAGCGACTAAAGCCGTCTAAGAGAGATTTTGCTAAAC
>Bacillus_Fam_541_52_2 Nr. of seq. 2 Alignment length(with gaps) = 52
Alignment score = 1.000000
AGCCACTATTTTCATTTTTCTTCAGGATAGTGGTCCCAGCTCCTGTGGCTGG
>Bacillus_Fam_542_52_2 Nr. of seq. 2 Alignment length(with gaps) = 52
Alignment score = 1.000000
GAACCGCTATTTTAGCTTTACACAGAAATAGTGGTCCCAGCGAGCGTGGCTA
>Bacillus_Fam_543_52_2 Nr. of seq. 2 Alignment length(with gaps) = 52
Alignment score = 1.000000
GTCACTCTAACCAAAGAACATAGAATCAGAGTGCCTCAAAAAGAGGATATGA
>Bacillus_Fam_544_52_2 Nr. of seq. 2 Alignment length(with gaps) = 52
Alignment score = 1.000000
AAATCAGAGGGCCTCAAACGAGCGAAATGGAACCCTCTAAACTGAAATAATC
>Bacillus_Fam_545_52_2 Nr. of seq. 2 Alignment length(with gaps) = 52
Alignment score = 0.820513
aCTCTaTTTCGGACAGAGTTcCTTGTTcAaTATTCAACTCTGTCcGATTGCa

>Bacillus_Fam_546_52_2 Nr. of seq. 2 Alignment length(with gaps) = 52
Alignment score = 0.801282
AAGGgCAgCATACGAAGcCTATGCcaCCCGAAAAAatGAAAcCGAGCAGATG
>Bacillus_Fam_547_52_2 Nr. of seq. 2 Alignment length(with gaps) = 52
Alignment score = 0.878205
CGCTATGATTACCgTCAACTCATTTTGGGCTCgcTCTCGGGAAGCATACAag
>Bacillus_Fam_548_52_2 Nr. of seq. 2 Alignment length(with gaps) = 52
Alignment score = 0.955128
GTTCAGACAGGAAGAACGAGAAATAGcCTGTTTGTGTCCGAAGAaGGGGCAG
>Bacillus_Fam_549_52_2 Nr. of seq. 2 Alignment length(with gaps) = 52
Alignment score = 0.910256
TGGGAGTTAAcCAAAgGGAATAAAGcCGAAATCCCAGAGCaAGCCAACAAAA
>Bacillus_Fam_550_52_2 Nr. of seq. 2 Alignment length(with gaps) = 52
Alignment score = 0.974359
GTCGCCATAAGAGGTTCATGACGACGAAAAATTGGAGGTCCAAGTAAAAACa
>Bacillus_Fam_551_52_2 Nr. of seq. 2 Alignment length(with gaps) = 52
Alignment score = 0.923077
TTCGGACTCAaCGAcCATCCGTTCGCGGTTTTGaGTCCGAACACCAGTCAAC
>Bacillus_Fam_552_52_2 Nr. of seq. 2 Alignment length(with gaps) = 52
Alignment score = 0.974359
CATGAAATATGGGAGTTAATcCTAATCATTATCGACCAAACAAGAGATGCCA
>Bacillus_Fam_553_52_2 Nr. of seq. 2 Alignment length(with gaps) = 52
Alignment score = 0.948718
CACCTTCGGACTCGCTCACGTTGATTTCTCCCcTTTCTGTCCGAACCTcCTC
>Bacillus_Fam_554_52_2 Nr. of seq. 2 Alignment length(with gaps) = 52
Alignment score = 0.897436
TCCTCTTCATAAGCccCGTGAAGaGGAAACGTAACCAAAATCTCATTCcTTT
>Bacillus_Fam_555_52_2 Nr. of seq. 2 Alignment length(with gaps) = 52
Alignment score = 0.820513
TGAAGAGGATTACAaGCTTaaaTTCcTCCCaCTTTCCTCTTCATCTAGGCTa
>Bacillus_Fam_556_52_2 Nr. of seq. 2 Alignment length(with gaps) = 52
Alignment score = 0.692308
TGTCcTcAATAAGGGCTaTTaAaGACAAAACTCccCgGGAaAGCAagaAAAA
>Bacillus_Fam_557_52_2 Nr. of seq. 2 Alignment length(with gaps) = 52
Alignment score = 0.628205
ATTCGGACTcaCTTactccTTTTaCaGCTcGacCTGTCCGAATacCCCcTCT
>Bacillus_Fam_558_52_2 Nr. of seq. 2 Alignment length(with gaps) = 52
Alignment score = 0.641026
GGCggTCATcCCCcTTATGAaCGACCaTaCCgATGTGTTTTaCaaTcgCaCG
>Bacillus_Fam_559_52_2 Nr. of seq. 2 Alignment length(with gaps) = 52
Alignment score = 0.666667
AATCAaACGGTCGaTCATccCCcTTATGAACGaCCaCGCCgGCTGAaaaTcc
>Bacillus_Fam_560_52_2 Nr. of seq. 2 Alignment length(with gaps) = 52
Alignment score = 0.724359
ATTAGTGACCGagaTaCGGGATTTacCcTAAATACGGGaACCTTTTaCcGCg
>Bacillus_Fam_561_52_2 Nr. of seq. 2 Alignment length(with gaps) = 52
Alignment score = 0.615385
TTAGTgACCGgTTTTCcCaCTcaaACcCgTaTTCGGGCACcaTTaCcCgCGA
>Bacillus_Fam_562_52_2 Nr. of seq. 2 Alignment length(with gaps) = 52
Alignment score = 0.910256
CAGAaAATCAGaTTTGATGGCACTGAACATcGAGAATAGgACCACCAACGAA
>Bacillus_Fam_563_52_2 Nr. of seq. 2 Alignment length(with gaps) = 52
Alignment score = 1.000000
TGAAAGTGTTGCTAACCCAGTTTAAGAACACTTTTCCGCAGAGCAGAAGGGT

>Bacillus_Fam_564_52_2 Nr. of seq. 2 Alignment length(with gaps) = 52
Alignment score = 0.980769
TGATGTGAAGGGTTATAAGTAAGTGAGAATTGACCCTTNAGTGCAGCCGTAG
>Bacillus_Fam_565_52_2 Nr. of seq. 2 Alignment length(with gaps) = 52
Alignment score = 0.974359
GTGAATGAACCCTTCaTACGAAGAATCAGGAGCTGAAGGGTCAAAAGGCGTC
>Bacillus_Fam_566_52_2 Nr. of seq. 2 Alignment length(with gaps) = 52
Alignment score = 1.000000
CATGAAGGGTCATATCATGCTCGCTTATGACCACTCAGCTCACCCTGCCCTG
>Bacillus_Fam_567_52_2 Nr. of seq. 2 Alignment length(with gaps) = 52
Alignment score = 0.826923
aAGGTTTAAGAGCACaAACGcGTCTaCCCAGatAAGAGTTTGTGCTGCaAAC
>Bacillus_Fam_568_52_2 Nr. of seq. 2 Alignment length(with gaps) = 52
Alignment score = 0.948718
TCTTACCCTCCCAAACCGTTATTCGATCTGACAGGGcAAGCAAACCTACgTT
>Bacillus_Fam_569_52_2 Nr. of seq. 2 Alignment length(with gaps) = 52
Alignment score = 0.897436
AAGTGTTCTTAAcCTGaGTTAGCAACACAAACTcTGTTGATcGTTCACTGGA
>Bacillus_Fam_570_52_2 Nr. of seq. 2 Alignment length(with gaps) = 52
Alignment score = 0.794872
TTGATGGTTCTTATAAGCCCGATAAGTaCCAcCAAaCTCTGAgTcccTTGaA
>Bacillus_Fam_571_52_2 Nr. of seq. 2 Alignment length(with gaps) = 52
Alignment score = 0.948718
GAAGGTCACAAAACaGGAAGGAATGTGCCCTTCGTGAAGTGTTCCAAGaTGT
>Bacillus_Fam_572_52_2 Nr. of seq. 2 Alignment length(with gaps) = 52
Alignment score = 1.000000
GGAAAGTGTTCTAAAAAGGGTTTAGCAACATTAACCCTGGCTGGAGAAGAGA
>Bacillus_Fam_573_52_2 Nr. of seq. 2 Alignment length(with gaps) = 52
Alignment score = 1.000000
TGAAAGGTCAATAAGCGGAAGGATAAGACCCTTTATCAAAGCTGGTCCGCCG
>Bacillus_Fam_574_52_2 Nr. of seq. 2 Alignment length(with gaps) = 52
Alignment score = 0.641026
CTTTGACTaCCGgTCTcgcgGCTTCCTCcaacTTTCGGgaGcCAaCCCCTTC
>Bacillus_Fam_575_52_2 Nr. of seq. 2 Alignment length(with gaps) = 52
Alignment score = 0.910256
AATCTCCACATACTGGaAAATCgTAGTCCCAATaAGCCCTATTGGTGCTCCg
>Bacillus_Fam_576_52_2 Nr. of seq. 2 Alignment length(with gaps) = 52
Alignment score = 0.974359
GGGCCTCCATCaGGGCGATGGACGACCGTTTATTGCTCAATCTCAGGTTTCA
>Bacillus_Fam_577_52_2 Nr. of seq. 2 Alignment length(with gaps) = 52
Alignment score = 0.730769
TTTTTCTCcCacTCtGCcGATTTCGGGTACTGAcaCcaCCTGTCAGTaaCTC
>Bacillus_Fam_578_52_2 Nr. of seq. 2 Alignment length(with gaps) = 52
Alignment score = 0.666667
gATGAAGACCgGAAcGAAGCTTGAGccaGaccaAAAAGGTaTTCATcaGGTT
>Bacillus_Fam_579_52_2 Nr. of seq. 2 Alignment length(with gaps) = 52
Alignment score = 0.692308
GCGaaTTCGGGACaTGAGaGaCgCCATCATGAaCCATcaaCaCGACTTTTcC
>Bacillus_Fam_580_52_2 Nr. of seq. 2 Alignment length(with gaps) = 52
Alignment score = 0.705128
GgCCCGTaGGtTCccTTTTTTgaCaCGAACGGgaCaTGATAAAGCgTCTCAT
>Bacillus_Fam_581_52_2 Nr. of seq. 2 Alignment length(with gaps) = 52
Alignment score = 0.653846
ATGAAGGaCAAGTgACAcCAAGAaAaaAaGaAaAaTGTCCTTCATcAaGTaa

>Bacillus_Fam_582_52_2 Nr. of seq. 2 Alignment length(with gaps) = 52 Alignment score = 0.801282
TGGgCTTTTGATaCCTTATAAAGACCATTcGGAacCGGATTCTTCCaCaaAT
>Bacillus_Fam_583_52_2 Nr. of seq. 2 Alignment length(with gaps) = 52 Alignment score = 0.775641
AGTTCCCGaaCCcAGGCAAAaTcCCCcTCCAGGGTAATtAATCGgTCACTTa
>Bacillus_Fam_584_52_2 Nr. of seq. 2 Alignment length(with gaps) = 52 Alignment score = 0.666667
TCAGTGCCCaTGcAGGGgaAAAAaaaAgGCccAcGGGCACTCAGAaAGGTTg
>Bacillus_Fam_585_52_2 Nr. of seq. 2 Alignment length(with gaps) = 52 Alignment score = 0.647436
aTGAGGACAagTTaaGCcCTTGCTcaacGAgTTcTGTCCTCATGAACaCCcC
>Bacillus_Fam_586_52_2 Nr. of seq. 2 Alignment length(with gaps) = 52 Alignment score = 0.717949
CTATTGGACAAAACcCccTTATAAAcCaCccAaAaTGTCCAaTAGAcCCCTT
>Bacillus_Fam_587_52_2 Nr. of seq. 2 Alignment length(with gaps) = 52 Alignment score = 0.717949
aGTGCACATTGAAGccTTCAATGAACACGaGAGaAAGGaAaATCaaCaaAGA
>Bacillus_Fam_588_52_2 Nr. of seq. 2 Alignment length(with gaps) = 52 Alignment score = 0.621795
TTATTCCcCTGAAGaAGaaaaAAaGaaCcgTcTTCaGGTGAATAACCAcaGT
>Bacillus_Fam_589_52_2 Nr. of seq. 2 Alignment length(with gaps) = 52 Alignment score = 0.641026
AaGaAAAAGTCCaTCATAGGGccAATGAAaGACAAAAccCAAccagCAaAGa
>Bacillus_Fam_590_52_2 Nr. of seq. 2 Alignment length(with gaps) = 52 Alignment score = 0.698718
ATGATGGACTTTTcccGCgTTcCCTcAaaCGcTTTTGTCCTTCAgAaCCTTg
>Bacillus_Fam_591_52_2 Nr. of seq. 2 Alignment length(with gaps) = 52 Alignment score = 0.769231
CTGGATTATAGAGGTGTGTTTCgCGCcgaATcGGGACaCcGaTTACCAAAaT
>Bacillus_Fam_592_52_2 Nr. of seq. 2 Alignment length(with gaps) = 52 Alignment score = 0.647436
GGagAAACGAGaCacAAaAGTCCAGTAaaTGGAAAAaAGaGTCGTGaaaACa
>Bacillus_Fam_593_52_2 Nr. of seq. 2 Alignment length(with gaps) = 52 Alignment score = 0.608974
TCATGAAGGACgTtTCcCaaGAcATaGcAcatCcAAaaGTCCTTCATCaCcC
>Bacillus_Fam_594_52_2 Nr. of seq. 2 Alignment length(with gaps) = 52 Alignment score = 0.974359
CACATTTTTCTGCATTTCGCTCCATTATCTGTTTCAAAACCTCgAGTTGACG
>Bacillus_Fam_595_52_2 Nr. of seq. 2 Alignment length(with gaps) = 52 Alignment score = 0.929487
GGGCACCTTTCTCCCTCTTTCGTTACCGCGAAACCGcTTTTTCCTCTCgaGC
>Bacillus_Fam_596_52_2 Nr. of seq. 2 Alignment length(with gaps) = 52 Alignment score = 0.794872
CTTCAATGTgACCGTTTCTaTGATTTcAaTaaCgTTTTGGTCACATTGAGCc
>Bacillus_Fam_597_52_2 Nr. of seq. 2 Alignment length(with gaps) = 52 Alignment score = 0.955128
ATGAAGACCGTTAGTTTTGGTGAATGagCAAGAACCGGTTATCATAGTTGAG
>Bacillus_Fam_598_52_2 Nr. of seq. 2 Alignment length(with gaps) = 52 Alignment score = 1.000000
GGCTTTCATTCTTCTCATGATGACCTTTTCTCTAGCTCTCTAGAATGATTTC
>Bacillus_Fam_599_51_2 Nr. of seq. 2 Alignment length(with gaps) = 51 Alignment score = 0.712418
TTTCATTACCaATcaCCagTTaTTCGTACCCTAATTaAGcCAACaCTCcAg

```
>Bacillus_Fam_600_51_2  Nr. of seq. 2 Alignment length(with gaps) = 51
Alignment score = 0.895425
 TTCGGCTTATAATTCTTacTCTCCATTCTTTTTACGCCGAACTcgCTCTTA
>Bacillus_Fam_601_51_2  Nr. of seq. 2 Alignment length(with gaps) = 51
Alignment score = 1.000000
 TTCTTGCTCTTCTCTAGCATCGTCCTCGCTGAGCTCTTCTGTACTGTCTTC
>Bacillus_Fam_602_51_2  Nr. of seq. 2 Alignment length(with gaps) = 51
Alignment score = 1.000000
 AAAAGGAAAGCGCCAAAAGCCCGTTTGTGAACACGAAAATCGAAAAACAGG
>Bacillus_Fam_603_48_2  Nr. of seq. 2 Alignment length(with gaps) = 48
Alignment score = 1.000000
 CAGATAGAGGAGGTTGAAGAACCGGAGCAAACTGAGGAGTCAGAAGAG
>Bacillus_Fam_604_48_2  Nr. of seq. 2 Alignment length(with gaps) = 48
Alignment score = 0.951389
 TTGGATTATCCACTGCATTACTtTCTTCTTGaGAAACAGCACTATTAC
>Bacillus_Fam_605_48_2  Nr. of seq. 2 Alignment length(with gaps) = 48
Alignment score = 0.694444
 TCTTTcGGcTCTTCcaTTGacCCATCacCAGGcTTTGTcCCaTCCTGA
>Bacillus_Fam_606_47_2  Nr. of seq. 2 Alignment length(with gaps) = 47
Alignment score = 1.000000
 TCCGTGGACAGATGGGGTGAAGTGTCTTTTTGTGGTGCCAGGTACGC
>Bacillus_Fam_607_46_2  Nr. of seq. 2 Alignment length(with gaps) = 46
Alignment score = 0.753623
 CCAGGTGAAGaGaGAATCTGGcCAaCCaGAaGAaGAAaTAACGGAG
>Bacillus_Fam_608_45_2  Nr. of seq. 2 Alignment length(with gaps) = 45
Alignment score = 0.629630
 AATCCAACaACACCACCAAcgaCAaGTGaAGaaAAcAAcGGaGAa
>Bacillus_Fam_609_45_2  Nr. of seq. 2 Alignment length(with gaps) = 45
Alignment score = 0.614815
 GGCATTTGaCCaTATCCcGCTGGcAcTTgCTgaCCAgaACCCagT
>Bacillus_Fam_610_45_2  Nr. of seq. 2 Alignment length(with gaps) = 45
Alignment score = 0.874074
 GAAAAAAGAAGCAGCAATgCAagaACAACAAAAACAAGATaAAAT
>Bacillus_Fam_611_45_2  Nr. of seq. 2 Alignment length(with gaps) = 45
Alignment score = 0.822222
 GATTCCTCaTCaGATTCaTAacCATCGCTaGACTCGTTATCTTCA
>Bacillus_Fam_612_43_2  Nr. of seq. 2 Alignment length(with gaps) = 43
Alignment score = 0.837209
 GAAGCtaCCgGCTGAAGAaCCTGTAACAGAGGaACCAGCAGAG
>Bacillus_Fam_613_43_2  Nr. of seq. 2 Alignment length(with gaps) = 43
Alignment score = 0.689922
 CATGCgGAAGGTNaaaNcACAACaGCAAGTgGGANATgCTTCN
>Bacillus_Fam_614_43_2  Nr. of seq. 2 Alignment length(with gaps) = 43
Alignment score = 0.732558
 CCTTCCGCATGCGAgNcAacTCCACTGGCTATTGTaTtaANtn
>Bacillus_Fam_615_43_2  Nr. of seq. 2 Alignment length(with gaps) = 43
Alignment score = 0.658915
 GcAGGAGCaATGGAcGCcCAAtATgCCaCAcATGCCGCAaGTa
>Bacillus_Fam_616_42_2  Nr. of seq. 2 Alignment length(with gaps) = 42
Alignment score = 0.793651
 aGATTGTGACCAAGCGCGGCGnAAAcTcCGGaCAATTGGCGa
>Bacillus_Fam_617_42_2  Nr. of seq. 2 Alignment length(with gaps) = 42
Alignment score = 0.690476
 AtTTCGtGATTTGCGgGCCaGAAcaCAcaCTTTTCaTGCCGG
```

>Bacillus_Fam_618_42_2 Nr. of seq. 2 Alignment length(with gaps) = 42
Alignment score = 0.638889
tcCGaTCcCTATcCGTcGGTAAAaCcCgTcATTCGTTGGTAa
>Bacillus_Fam_619_42_2 Nr. of seq. 2 Alignment length(with gaps) = 42
Alignment score = 0.809524
GGaATaACTGGTCCTACTGGaGTTACAGGacCTACCGGAATc
>Bacillus_Fam_620_42_2 Nr. of seq. 2 Alignment length(with gaps) = 42
Alignment score = 1.000000
CTTGCTTTCAATGATGAAATGCGCCATTTCCAAAAGGAGCAG
>Bacillus_Fam_621_42_2 Nr. of seq. 2 Alignment length(with gaps) = 42
Alignment score = 0.809524
GGATCaGGTTCcGCaTCCGGaACGACcGCAACGGGCAACACc
>Bacillus_Fam_622_42_2 Nr. of seq. 2 Alignment length(with gaps) = 42
Alignment score = 1.000000
TTTGATTCGGTTGATCAGAAGTTTGATTCCATTGATCAGAGA
>Bacillus_Fam_623_42_2 Nr. of seq. 2 Alignment length(with gaps) = 42
Alignment score = 0.650794
AGaaGTAGTGGAaaCTCaAGaaGAAGAGAGACCaGTAcAgCa
>Bacillus_Fam_624_42_2 Nr. of seq. 2 Alignment length(with gaps) = 42
Alignment score = 0.690476
GGAGAAATGCCacaAGccTAcGGCCAaATGCCaaCAGGaTAC
>Bacillus_Fam_625_42_2 Nr. of seq. 2 Alignment length(with gaps) = 42
Alignment score = 0.730159
AaAcTCGTGGGTAAAgCaCCGaGTTATaTGGGTATTCcACca
>Bacillus_Fam_626_42_2 Nr. of seq. 2 Alignment length(with gaps) = 42
Alignment score = 0.968254
CCAGGAGGCGGAACGGGAACAGAcAACGGAACCGGAGGAAAC
>Bacillus_Fam_627_42_2 Nr. of seq. 2 Alignment length(with gaps) = 42
Alignment score = 1.000000
GAAGTCGCGAAATTCCGGTGGGAAGTTGAAAGATTAACGGGA
>Bacillus_Fam_628_41_2 Nr. of seq. 2 Alignment length(with gaps) = 41
Alignment score = 0.617886
TTCTTTTACTggAgcgGTTTTGttACAaTTGGCTgtGGcGC
>Bacillus_Fam_629_41_2 Nr. of seq. 2 Alignment length(with gaps) = 41
Alignment score = 0.626016
GCgGATAAAaCaaAgAAAGTCGCgGATATATcggAAAgTTa
>Bacillus_Fam_630_40_2 Nr. of seq. 2 Alignment length(with gaps) = 40
Alignment score = 0.754167
tGTTCaTCTGTTGGCTCTGTaacTTCTACTTCTTCaGtCT
>Bacillus_Fam_631_39_2 Nr. of seq. 2 Alignment length(with gaps) = 39
Alignment score = 0.897436
GGcTTCACTTCTGGcTCCTTCGGcTCTTTCGGATCTTCC
>Bacillus_Fam_632_39_2 Nr. of seq. 2 Alignment length(with gaps) = 39
Alignment score = 0.897436
AATCCGGAACAAAAcCCAGCAACTAAACCTGcTACTGAc
>Bacillus_Fam_633_39_2 Nr. of seq. 2 Alignment length(with gaps) = 39
Alignment score = 0.760684
TGaTCaTGcTCgTCccCATGAGCTTCTTCCTCATGCTCa
>Bacillus_Fam_634_39_2 Nr. of seq. 2 Alignment length(with gaps) = 39
Alignment score = 0.641026
ATGaCCATGATGGaAGCCaTaACCaGGGTaaTAaccgCC
>Bacillus_Fam_635_39_2 Nr. of seq. 2 Alignment length(with gaps) = 39
Alignment score = 0.794872
CATGcTCGTGATCATGATCTTCCTCATGccCGTcCTCac

>Bacillus_Fam_636_39_2 Nr. of seq. 2 Alignment length(with gaps) = 39
Alignment score = 0.974359
AGAAGAAGTTGTGGAATTAGAGAAAtCTGAGGAAGCGAC
>Bacillus_Fam_637_39_2 Nr. of seq. 2 Alignment length(with gaps) = 39
Alignment score = 0.666667
AAGaAaGAAGAAcaAGaaCGtTTAGCaGAaGAACAGCGa
>Bacillus_Fam_638_39_2 Nr. of seq. 2 Alignment length(with gaps) = 39
Alignment score = 0.940171
GCTCCCATCACtCCGGACGGCTGACCCATCGGGGATTGc
>Bacillus_Fam_639_37_2 Nr. of seq. 2 Alignment length(with gaps) = 37
Alignment score = 0.662162
TGtCCTGGaTAaCCTCCCATGCCaCCaTAaCCaCCca
>Bacillus_Fam_640_37_2 Nr. of seq. 2 Alignment length(with gaps) = 37
Alignment score = 0.603604
cACGGGAcCaACGGGAGcAACCgGGacCaAcCGGAGc
>Bacillus_Fam_641_37_2 Nr. of seq. 2 Alignment length(with gaps) = 37
Alignment score = 0.747748
TGCCACTGaTGCCAAgGTAccGTaaGGATGcTGCTGA
>Bacillus_Fam_642_36_2 Nr. of seq. 2 Alignment length(with gaps) = 36
Alignment score = 0.666667
ACcGGAGCaACaGGccCaACgGGAaTAACgGGCCCA
>Bacillus_Fam_643_36_2 Nr. of seq. 2 Alignment length(with gaps) = 36
Alignment score = 0.898148
AAAAGCAAGAAACCcGATGACTGCAAAAAaCCtGAC
>Bacillus_Fam_644_36_2 Nr. of seq. 2 Alignment length(with gaps) = 36
Alignment score = 0.925926
GAAATTAAaCAGGAAAAAGATATAGGAATTAAaAAG
>Bacillus_Fam_645_36_2 Nr. of seq. 2 Alignment length(with gaps) = 36
Alignment score = 0.925926
TCTTTTTGAGCcTGTTCAGCTTGAGCcTGCTGCTGC
>Bacillus_Fam_646_36_2 Nr. of seq. 2 Alignment length(with gaps) = 36
Alignment score = 1.000000
GGTCCTTGAACCCCTTGTGTGCCTTGATCTCCTTGC
>Bacillus_Fam_647_36_2 Nr. of seq. 2 Alignment length(with gaps) = 36
Alignment score = 0.944444
CAAGGATCAACTGGNGCNCAGGGATCCCAAGGACCG
>Bacillus_Fam_648_36_2 Nr. of seq. 2 Alignment length(with gaps) = 36
Alignment score = 0.666667
GGaAATACAACgCCGCCAaaCAaTGGaAaaGGCcAA
>Bacillus_Fam_649_36_2 Nr. of seq. 2 Alignment length(with gaps) = 36
Alignment score = 0.962963
GCAACAACCGGAATAACGGaAACGGCCAGGCCGCAA
>Bacillus_Fam_650_36_2 Nr. of seq. 2 Alignment length(with gaps) = 36
Alignment score = 0.703704
CaTgGacTGCaGTgCCACCATTCGGaCCTTGaCCTC
>Bacillus_Fam_651_36_2 Nr. of seq. 2 Alignment length(with gaps) = 36
Alignment score = 0.629630
CGCCAGTAGCcCCgGaAaaaCCaGTaGGaCCGGTAa
>Bacillus_Fam_652_36_2 Nr. of seq. 2 Alignment length(with gaps) = 36
Alignment score = 0.666667
GGacCgACAGGAGCAACAGGAaTAGaaGGaaTCACc
>Bacillus_Fam_653_36_2 Nr. of seq. 2 Alignment length(with gaps) = 36
Alignment score = 0.824074
AGTACAGCAAacGGAAGAAaaGCAGCCGAGAGTAcC

>Bacillus_Fam_654_34_2 Nr. of seq. 2 Alignment length(with gaps) = 34
Alignment score = 1.000000
TCTGTCCCCTTGGCCCAGTTTTCGGGCCAGGGGG
>Bacillus_Fam_655_33_2 Nr. of seq. 2 Alignment length(with gaps) = 33
Alignment score = 1.000000
CGGCGGGAATGCCGGAATGCCAGGTCAAGGAAG
>Bacillus_Fam_656_30_2 Nr. of seq. 2 Alignment length(with gaps) = 30
Alignment score = 1.000000
AAAATGAAAGAAACTCCAAATGTAGTACCG
>Bacillus_Fam_657_28_2 Nr. of seq. 2 Alignment length(with gaps) = 28
Alignment score = 0.601190
aGCcAAGCaaAAGCaACAAGAacAACAa
>Bacillus_Fam_658_27_2 Nr. of seq. 2 Alignment length(with gaps) = 27
Alignment score = 0.697531
tTGTACCTGGTACATgGaGcGtTtGAT
>Bacillus_Fam_659_27_2 Nr. of seq. 2 Alignment length(with gaps) = 27
Alignment score = 0.654321
cCTccaGCTcccccaGgacCTccTGaa
>Bacillus_Fam_660_24_2 Nr. of seq. 2 Alignment length(with gaps) = 24
Alignment score = 0.631944
GgACAgGTAAAAcCAcAAAAAGaa
>Bacillus_Fam_661_24_2 Nr. of seq. 2 Alignment length(with gaps) = 24
Alignment score = 0.833333
ATGGCaGGAGCGAACATGGcGCCa
>Bacillus_Fam_662_24_2 Nr. of seq. 2 Alignment length(with gaps) = 24
Alignment score = 0.944444
CAGAAAAGAATCAAAAAaCAGAGA
>Bacillus_Fam_663_23_2 Nr. of seq. 2 Alignment length(with gaps) = 23
Alignment score = 0.666667
TATTTGCGTCGgTcCCAnaCnAT
>Bacillus_Fam_664_23_2 Nr. of seq. 2 Alignment length(with gaps) = 23
Alignment score = 0.717391
aTTAGGATCGTaaATTTCTGaGA
>Bacillus_Fam_665_23_2 Nr. of seq. 2 Alignment length(with gaps) = 23
Alignment score = 0.615942
cCAACTaGCGGATaAcCaCGaTC
>Bacillus_Fam_666_23_2 Nr. of seq. 2 Alignment length(with gaps) = 23
Alignment score = 0.760870
TTTATTTTCCATTcaAGCCGnAt
>Bacillus_Fam_667_23_2 Nr. of seq. 2 Alignment length(with gaps) = 23
Alignment score = 0.666667
ATTCCGCGAAAnAACcaAcaATA
>Bacillus_Fam_668_22_2 Nr. of seq. 2 Alignment length(with gaps) = 22
Alignment score = 0.772727
tTATATCAGCGaAACTCCaAAt
>Bacillus_Fam_669_22_2 Nr. of seq. 2 Alignment length(with gaps) = 22
Alignment score = 0.621212
aATTCCGgAACTTTTcGgaTTa
>Bacillus_Fam_670_22_2 Nr. of seq. 2 Alignment length(with gaps) = 22
Alignment score = 0.643939
cTaACtATaAcATTTTTGGGTC
>Bacillus_Fam_671_22_2 Nr. of seq. 2 Alignment length(with gaps) = 22
Alignment score = 0.628788
aAAAAAGCATTAtGaTcAaGAc

>Bacillus_Fam_672_22_2 Nr. of seq. 2 Alignment length(with gaps) = 22
Alignment score = 0.628788
 TATTCCCGTCTGaGaagGaaGt
>Bacillus_Fam_673_22_2 Nr. of seq. 2 Alignment length(with gaps) = 22
Alignment score = 0.681818
 TTATTCCCGTCTCagaATaaGC
>Bacillus_Fam_674_22_2 Nr. of seq. 2 Alignment length(with gaps) = 22
Alignment score = 0.696970
 TcCGACAGTAAaGcGTGGCtTA
>Bacillus_Fam_675_22_2 Nr. of seq. 2 Alignment length(with gaps) = 22
Alignment score = 0.674242
 tAATTCCGGAACTTTcaacCAt
>Bacillus_Fam_676_22_2 Nr. of seq. 2 Alignment length(with gaps) = 22
Alignment score = 0.643939
 cTTTATTGTCCgGAAacGCtCt
>Bacillus_Fam_677_22_2 Nr. of seq. 2 Alignment length(with gaps) = 22
Alignment score = 0.810606
 TTCATTCCAAAAcTGGGNTTTt
>Bacillus_Fam_678_22_2 Nr. of seq. 2 Alignment length(with gaps) = 22
Alignment score = 0.810606
 CTAACTCGACTAAAGGcgGAGg
>Bacillus_Fam_679_22_2 Nr. of seq. 2 Alignment length(with gaps) = 22
Alignment score = 0.666667
 TTTAAACAGGTTgGcAcGcgCG
>Bacillus_Fam_680_22_2 Nr. of seq. 2 Alignment length(with gaps) = 22
Alignment score = 0.674242
 TTTAcgATCCAAaTTgAACCAa
>Bacillus_Fam_681_22_2 Nr. of seq. 2 Alignment length(with gaps) = 22
Alignment score = 0.848485
 GGAATGATTATTcGcCGAATCC
>Bacillus_Fam_682_22_2 Nr. of seq. 2 Alignment length(with gaps) = 22
Alignment score = 0.628788
 aCTTCTcccTTTGGaTCTgTCa
>Bacillus_Fam_683_22_2 Nr. of seq. 2 Alignment length(with gaps) = 22
Alignment score = 0.727273
 ATTCTACAAAAaTcAAAGagGA
>Bacillus_Fam_684_22_2 Nr. of seq. 2 Alignment length(with gaps) = 22
Alignment score = 0.613636
 GTTACTCCCGcATaGaCTcCac
>Bacillus_Fam_685_22_2 Nr. of seq. 2 Alignment length(with gaps) = 22
Alignment score = 0.727273
 GCAAATAGAAaTggGaAAAtCG
>Bacillus_Fam_686_22_2 Nr. of seq. 2 Alignment length(with gaps) = 22
Alignment score = 0.674242
 GATAATTCGACCgTcacGaGCc
>Bacillus_Fam_687_22_2 Nr. of seq. 2 Alignment length(with gaps) = 22
Alignment score = 0.613636
 cTCTCGGCGTTCCgAcccCTcC
>Bacillus_Fam_688_22_2 Nr. of seq. 2 Alignment length(with gaps) = 22
Alignment score = 0.803030
 TCGCAAATAAACAgGaTAaGAT
>Bacillus_Fam_689_22_2 Nr. of seq. 2 Alignment length(with gaps) = 22
Alignment score = 0.643939
 gTTTTGTCTAtTTTTaAgagAG

>Bacillus_Fam_690_22_2 Nr. of seq. 2 Alignment length(with gaps) = 22
Alignment score = 0.659091
AGTTCGTGCCAAAAtcaGaCag
>Bacillus_Fam_691_22_2 Nr. of seq. 2 Alignment length(with gaps) = 22
Alignment score = 0.750000
aGGTTCTCACGCTTCTCaGcAa
>Bacillus_Fam_692_22_2 Nr. of seq. 2 Alignment length(with gaps) = 22
Alignment score = 0.621212
AGGTTCTCACagaaCTCaCcTG
>Bacillus_Fam_693_22_2 Nr. of seq. 2 Alignment length(with gaps) = 22
Alignment score = 0.651515
TTcGATAATaAAAcTTgGaATT
>Bacillus_Fam_694_22_2 Nr. of seq. 2 Alignment length(with gaps) = 22
Alignment score = 0.795455
cTATaCTTTACTCCGATCtCTT
>Bacillus_Fam_695_21_2 Nr. of seq. 2 Alignment length(with gaps) = 21
Alignment score = 0.603175
AAGAACCccATAACaCAnGct
>Bacillus_Fam_696_21_2 Nr. of seq. 2 Alignment length(with gaps) = 21
Alignment score = 0.603175
CtAaGcTCAaATGCCAATGgg
>Bacillus_Fam_697_21_2 Nr. of seq. 2 Alignment length(with gaps) = 21
Alignment score = 0.634921
gGCAAATAGAACGaggTTTcc
>Bacillus_Fam_698_21_2 Nr. of seq. 2 Alignment length(with gaps) = 21
Alignment score = 0.658730
CTCCAaCTTGcGGATaGaGCn
>Bacillus_Fam_699_21_2 Nr. of seq. 2 Alignment length(with gaps) = 21
Alignment score = 0.785714
aTATATCAAGAAATTTaGtGA
>Bacillus_Fam_700_21_2 Nr. of seq. 2 Alignment length(with gaps) = 21
Alignment score = 0.626984
TCGCCCgTAAATCtCaAaAtt
>Bacillus_Fam_701_21_2 Nr. of seq. 2 Alignment length(with gaps) = 21
Alignment score = 0.706349
CGCAGATAAcTCAaGAAAaag
>Bacillus_Fam_702_21_2 Nr. of seq. 2 Alignment length(with gaps) = 21
Alignment score = 0.603175
TTTaCGGTaGcACaAcTTTnA
>Bacillus_Fam_703_21_2 Nr. of seq. 2 Alignment length(with gaps) = 21
Alignment score = 0.682540
TTTGTCGTAAGtGCaGctAgT
>Bacillus_Fam_704_21_2 Nr. of seq. 2 Alignment length(with gaps) = 21
Alignment score = 0.650794
TTGGCGaaATtACagTCAAGN
>Bacillus_Fam_705_21_2 Nr. of seq. 2 Alignment length(with gaps) = 21
Alignment score = 0.666667
ACtGGCGAaAAAAAccTAATa
>Bacillus_Fam_706_21_2 Nr. of seq. 2 Alignment length(with gaps) = 21
Alignment score = 0.706349
GCAAATAAAAAaTGggaATAc
>Bacillus_Fam_707_21_2 Nr. of seq. 2 Alignment length(with gaps) = 21
Alignment score = 0.666667
TTATCaACAcAcTTTTAGaaT

>Bacillus_Fam_708_21_2 Nr. of seq. 2 Alignment length(with gaps) = 21
Alignment score = 0.666667
aaAATACGGCGGgTTTGAgCa
>Bacillus_Fam_709_21_2 Nr. of seq. 2 Alignment length(with gaps) = 21
Alignment score = 0.619048
TTGGTTGATTTcCTacaGaAg
>Bacillus_Fam_710_21_2 Nr. of seq. 2 Alignment length(with gaps) = 21
Alignment score = 0.690476
GAaAGTaTaTCcAAAATAGCc
>Bacillus_Fam_711_21_2 Nr. of seq. 2 Alignment length(with gaps) = 21
Alignment score = 0.809524
GCAACATCcTGTTTcAGCaCT
>Bacillus_Fam_712_21_2 Nr. of seq. 2 Alignment length(with gaps) = 21
Alignment score = 0.873016
TGTTAGTacATTAAAGAGTGA
>Bacillus_Fam_713_21_2 Nr. of seq. 2 Alignment length(with gaps) = 21
Alignment score = 0.746032
GGaGgcTCTTCgCCATCACCC
>Bacillus_Fam_714_21_2 Nr. of seq. 2 Alignment length(with gaps) = 21
Alignment score = 0.746032
ATCCGACAGTaAGCcGCgGcT
>Bacillus_Fam_715_21_2 Nr. of seq. 2 Alignment length(with gaps) = 21
Alignment score = 0.809524
TTCAAAaTTTCACTGTGaTCg
>Bacillus_Fam_716_21_2 Nr. of seq. 2 Alignment length(with gaps) = 21
Alignment score = 0.650794
TtTaTCAACCACTTcTCcgCt
>Bacillus_Fam_717_21_2 Nr. of seq. 2 Alignment length(with gaps) = 21
Alignment score = 0.873016
TTACTACCAACaACCaCAGAT
>Bacillus_Fam_718_21_2 Nr. of seq. 2 Alignment length(with gaps) = 21
Alignment score = 0.682540
AgaGCcGATACaCCCGTaAAA
>Bacillus_Fam_719_21_2 Nr. of seq. 2 Alignment length(with gaps) = 21
Alignment score = 0.698413
AATCACTACGaAAaTcAaAaT
>Bacillus_Fam_720_21_2 Nr. of seq. 2 Alignment length(with gaps) = 21
Alignment score = 0.746032
GTATTCGACAcAagTTGGATc
>Bacillus_Fam_721_21_2 Nr. of seq. 2 Alignment length(with gaps) = 21
Alignment score = 0.888889
cTCACGAGTAAATCgTGAAAA
>Bacillus_Fam_722_21_2 Nr. of seq. 2 Alignment length(with gaps) = 21
Alignment score = 0.682540
acCTTcATCTCCGTCaCCaGG
>Bacillus_Fam_723_21_2 Nr. of seq. 2 Alignment length(with gaps) = 21
Alignment score = 0.698413
gTGGTCaATAaAAcCAgAAAA
>Bacillus_Fam_724_21_2 Nr. of seq. 2 Alignment length(with gaps) = 21
Alignment score = 0.619048
TGTTAAaaAAAAcCaCAAAcc
>Bacillus_Fam_725_21_2 Nr. of seq. 2 Alignment length(with gaps) = 21
Alignment score = 0.825397
CGCCAATAGAACCTctaAATC

>Bacillus_Fam_726_21_2 Nr. of seq. 2 Alignment length(with gaps) = 21
Alignment score = 0.619048
aTcCGACAGTTGGaaCacGCT
>Bacillus_Fam_727_21_2 Nr. of seq. 2 Alignment length(with gaps) = 21
Alignment score = 0.682540
TTGGCGAAATcCcNgCacAGT
>Bacillus_Fam_728_20_2 Nr. of seq. 2 Alignment length(with gaps) = 20
Alignment score = 0.800000
TATCCATTGTaAATTgaATA
>Bacillus_Fam_729_20_2 Nr. of seq. 2 Alignment length(with gaps) = 20
Alignment score = 0.616667
GACGCAATTAaaccatATTA
>Bacillus_Fam_730_20_2 Nr. of seq. 2 Alignment length(with gaps) = 20
Alignment score = 0.700000
TTTGCGTCTCCcacctGATA
>Bacillus_Fam_731_20_2 Nr. of seq. 2 Alignment length(with gaps) = 20
Alignment score = 0.750000
TATATCGGCGtTTTTaGaaA
>Bacillus_Fam_732_20_2 Nr. of seq. 2 Alignment length(with gaps) = 20
Alignment score = 0.883333
CTCATATATTTcGTTCTCcG
>Bacillus_Fam_733_20_2 Nr. of seq. 2 Alignment length(with gaps) = 20
Alignment score = 0.750000
TTTGGCTGATATTTTcCcac
>Bacillus_Fam_734_19_2 Nr. of seq. 2 Alignment length(with gaps) = 19
Alignment score = 0.649123
cCcGGaTGaAGGTCCTGGT
>Bacillus_Fam_735_19_2 Nr. of seq. 2 Alignment length(with gaps) = 19
Alignment score = 0.622807
cTaGAcAAGCAAGaACAaT
>Bacillus_Fam_736_19_2 Nr. of seq. 2 Alignment length(with gaps) = 19
Alignment score = 0.649123
AAgCCAAaGCcGCCaCACA
>Bacillus_Fam_737_18_2 Nr. of seq. 2 Alignment length(with gaps) = 18
Alignment score = 0.666667
ACGGGGaacACCGGAcCa
>Bacillus_Fam_738_18_2 Nr. of seq. 2 Alignment length(with gaps) = 18
Alignment score = 0.629630
CCaGATACAccAaAaAAA
>Bacillus_Fam_739_18_2 Nr. of seq. 2 Alignment length(with gaps) = 18
Alignment score = 0.629630
TGaTGaTGcTGaCCaTGG
>Bacillus_Fam_740_18_2 Nr. of seq. 2 Alignment length(with gaps) = 18
Alignment score = 0.648148
aACAACAaCAaCAGCaGt
>Bacillus_Fam_741_18_2 Nr. of seq. 2 Alignment length(with gaps) = 18
Alignment score = 0.925926
CCAGGaGAAGGTGAAGGC
>Bacillus_Fam_742_18_2 Nr. of seq. 2 Alignment length(with gaps) = 18
Alignment score = 0.629630
aTCTTcaTCTagTCCTGG
>Bacillus_Fam_743_18_2 Nr. of seq. 2 Alignment length(with gaps) = 18
Alignment score = 0.629630
CACCcAATCCAaCTaagC

>Bacillus_Fam_744_18_2 Nr. of seq. 2 Alignment length(with gaps) = 18
Alignment score = 0.703704
GCCTCTTGcTcAGCTTca
>Bacillus_Fam_745_18_2 Nr. of seq. 2 Alignment length(with gaps) = 18
Alignment score = 0.777778
ATcAGTACCAGTaCCaGG
>Bacillus_Fam_746_18_2 Nr. of seq. 2 Alignment length(with gaps) = 18
Alignment score = 0.629630
AaaACTTcTAGAAaCCGg
>Bacillus_Fam_747_18_2 Nr. of seq. 2 Alignment length(with gaps) = 18
Alignment score = 0.777778
GGcCCGAATCCCATgCCa
>Bacillus_Fam_748_18_2 Nr. of seq. 2 Alignment length(with gaps) = 18
Alignment score = 0.851852
GGAGCACCTGTTaCGCCc
>Bacillus_Fam_749_18_2 Nr. of seq. 2 Alignment length(with gaps) = 18
Alignment score = 0.629630
CCggcAGaTCCAGTAAcT
>Bacillus_Fam_750_18_2 Nr. of seq. 2 Alignment length(with gaps) = 18
Alignment score = 0.703704
GGTGGaATgGGCGGaCaT
>Bacillus_Fam_751_18_2 Nr. of seq. 2 Alignment length(with gaps) = 18
Alignment score = 0.777778
ATACCGGaGGcGAAGaAG
>Bacillus_Fam_752_18_2 Nr. of seq. 2 Alignment length(with gaps) = 18
Alignment score = 0.629630
CcTaCTgGTGcTGCTcGT
>Bacillus_Fam_753_18_2 Nr. of seq. 2 Alignment length(with gaps) = 18
Alignment score = 0.666667
CCAGCaGGaGCaACaGGa
>Bacillus_Fam_754_18_2 Nr. of seq. 2 Alignment length(with gaps) = 18
Alignment score = 0.703704
cTGGCTTTGaTcGTTcTG
>Bacillus_Fam_755_18_2 Nr. of seq. 2 Alignment length(with gaps) = 18
Alignment score = 0.648148
CcTGCTGtTTTgcTTGcT
>Bacillus_Fam_756_18_2 Nr. of seq. 2 Alignment length(with gaps) = 18
Alignment score = 0.629630
GGAaCcGGATTTGcTccT
>Bacillus_Fam_757_16_2 Nr. of seq. 2 Alignment length(with gaps) = 16
Alignment score = 0.625000
CTgAAcTCAACCAAcA
>Bacillus_Fam_758_15_2 Nr. of seq. 2 Alignment length(with gaps) = 15
Alignment score = 0.688889
TTATTatGTTTTTCc
>Bacillus_Fam_759_15_2 Nr. of seq. 2 Alignment length(with gaps) = 15
Alignment score = 0.733333
GGcGAACCaGGaGAT
>Bacillus_Fam_760_15_2 Nr. of seq. 2 Alignment length(with gaps) = 15
Alignment score = 0.666667
CaAAAACCAGaGGat
>Bacillus_Fam_761_15_2 Nr. of seq. 2 Alignment length(with gaps) = 15
Alignment score = 0.733333
TTCTTgTTCTTTgTa

>Bacillus_Fam_762_15_2 Nr. of seq. 2 Alignment length(with gaps) = 15
Alignment score = 1.000000
 GGCATGATGTGCGGC
>Bacillus_Fam_763_15_2 Nr. of seq. 2 Alignment length(with gaps) = 15
Alignment score = 0.644444
 CCaGGCaaTGGCGaT
>Bacillus_Fam_764_15_2 Nr. of seq. 2 Alignment length(with gaps) = 15
Alignment score = 0.844444
 GGcATTTCTTTgATC
>Bacillus_Fam_765_14_2 Nr. of seq. 2 Alignment length(with gaps) = 14
Alignment score = 0.738095
 TTcTCTTTTTTCtt
>Bacillus_Fam_766_14_2 Nr. of seq. 2 Alignment length(with gaps) = 14
Alignment score = 0.714286
 TcATTACaTATCaC
>Bacillus_Fam_767_12_2 Nr. of seq. 2 Alignment length(with gaps) = 12
Alignment score = 0.666667
 GGAgGAGTaaTT
>Bacillus_Fam_768_12_2 Nr. of seq. 2 Alignment length(with gaps) = 12
Alignment score = 0.694444
 CCTgAaCCTaAG
>Bacillus_Fam_769_12_2 Nr. of seq. 2 Alignment length(with gaps) = 12
Alignment score = 0.666667
 TGGcaTTTCTgT
>Bacillus_Fam_770_12_2 Nr. of seq. 2 Alignment length(with gaps) = 12
Alignment score = 0.805556
 GATGCcGATGCg
>Bacillus_Fam_771_12_2 Nr. of seq. 2 Alignment length(with gaps) = 12
Alignment score = 0.666667
 aCaGGaGGAACA
>Bacillus_Fam_772_12_2 Nr. of seq. 2 Alignment length(with gaps) = 12
Alignment score = 0.777778
 TCATGGccGTGA
>Bacillus_Fam_773_12_2 Nr. of seq. 2 Alignment length(with gaps) = 12
Alignment score = 0.777778
 TTGaAcTGGTTG
>Bacillus_Fam_774_12_2 Nr. of seq. 2 Alignment length(with gaps) = 12
Alignment score = 0.777778
 CaGGAACAGAcC
>Bacillus_Fam_775_11_2 Nr. of seq. 2 Alignment length(with gaps) = 11
Alignment score = 0.636364
 GTTTCgaTATg
>Bacillus_Fam_776_321_1 Nr. of seq. 1 Alignment length(with gaps) = 321
Alignment score = 0.000000

TGAACGTTACGGTATGTCTTCCATCTCTAAATTTGGTTGTGTCGAGAGTATAATGAAAGCCACCATTCCCATT
GTGGTACTCTGGAAAGACTTTCTGGACATCTGGTCTCGCATCTCCATAGATCGCCTGACCTGCCACTACACCG
TCTACCAGTACTTCAATTTTCGACACTCCGCTTTCATCTAAAAACCAGCCCCGTACGTTTTGGGACCCGATAA
GTGTTTTGCCAGAAACCGGATCATCGATATACCCGAGGACATTTGCAATCGTTATAGTCTTTTCAGGCAATGT
TGTGACATAGCCGTTCATTCCTGTTTCTC
>Bacillus_Fam_777_255_1 Nr. of seq. 1 Alignment length(with gaps) = 255
Alignment score = 0.000000

AGTAGGACGTTGCCAGGCAGATTAAACACANTCCTTTAGGGATGTGTTTTTTAATATGCATAAAGAGTATACT

CGGCGCAAAAGAGCGCTGGTTCGAAGAAGCAGCCCCTACGAAGCGAAGCCCCGGAAAGCGATCGAAGGAAGAA
AACCAAGTACGTTCGCGAGACGAATCGGTTCTCTTCGTGCGACCTCGTGAGAGGCATCGGGGAAATCGCTACA
TGCCATACTTCCCCGAAGAAGAGAATCCGAAGAGAC
>Bacillus_Fam_778_252_1 Nr. of seq. 1 Alignment length(with gaps) = 252
Alignment score = 0.000000

TTTTGCTTCGATTTAATCCTGACTGAGAAGCTACCTTTACTACTTGCTGTCCCTGTCCCCAGCACCGTTTTAC
CACGTTTAATCGTCACTTTGGCGCCGGCTTCTGCTTTACCTGTAATCGTTGTTTGATTATCGCCAAACCGATT
GACGGACGGGGCTGATGGTGCTGTTTTGTCTTCCACTTTGAACGATTTGCCTGCACCCGTATTTCCAGCTTTA
TCTGTCGCATAGGCTGTCAGAACCGTTCCTGCC
>Bacillus_Fam_779_249_1 Nr. of seq. 1 Alignment length(with gaps) = 249
Alignment score = 0.000000

AAACAAAAAGCAGGAACGAAGCTTGTCGTTTATGCGGAAGATGCGGCAGGGAACAAGAGCGNTGAAACNGTNG
TAACGGTTATTGATAAAACGGCTCCGGCAGCGCCGAAAGTCGGAGAAGTAAGCGATACAAGTACTGTGGTTAC
AGGAACCACAGAAGCGGGCGCGAAAGTAACAGTAAAATCCGGCTCGAACATTTTAGGNACGGCAACGGCTGAT
CATACCGGTGCATTTAAGGTAACCATTGCA
>Bacillus_Fam_780_246_1 Nr. of seq. 1 Alignment length(with gaps) = 246
Alignment score = 0.000000

GTTTTATCTACTTTTACTTCTACTGTGTTTTTATTTTCNGTATTACCNGCATTATCTACACTGTAGTAAGTTA
GAGTGTGGATGCCTTCTTTTTCTANGGTNAAGCTTGTGCCTTCGCTGAATTCAGCNCCATCGATAGAATAGTA
GGTTGCTTTCACACCGCTTAGGTCATCAGTTGCTGTCAGCTCAACATTCACTGCTTCTGTGTACCATTGATCC
TCGACATTACTTACAGTAGTAGGAGCA
>Bacillus_Fam_781_237_1 Nr. of seq. 1 Alignment length(with gaps) = 237
Alignment score = 0.000000

CCATTGGCTTTCACCCTTGCCCAGCCGTTCGATTCGGATATCACTGATACTGCCGTTCCGCTGGACAGCTTGC
TTATCACTTTTCCTTGTGTAGACGCCTTGTCTCGGACATTCAGCGAGGATCCAGAGTCGACTTTCACATATTT
GGTAACCGGNTTGGCCGACGGTTTTGGTGTACCTGTGCTGGCTGACGGCTTGCTTGATGACAAATATTGTGAA
CTTACGTAGCCTGTTTTG
>Bacillus_Fam_782_237_1 Nr. of seq. 1 Alignment length(with gaps) = 237
Alignment score = 0.000000

TATACGTTTGTTGGCTGGAACACCGAAGCGGATGGAAGTGGTGAAACCTATAAAGAGGGTGACACGTTTACCC
AAGGAATAGGAAATGTGACGTTCTATGCGCAGTGGTCTGTAAATCACTACGAGCTGCGATATGACGGGAACGG
GCAAGATGGAGGAAGTGCGCCAGAAGCGGAATCTGTTGCGTATGGAAGTGAAGTGGAGGTATCGGATTCTCAA
ACGTTAAGCCGAACCGGT
>Bacillus_Fam_783_234_1 Nr. of seq. 1 Alignment length(with gaps) = 234
Alignment score = 0.000000

TTACTTTTTCTCCGTTTTTTAAATTTCCGAGAATTGCACTATTTGTATTTGCTTGACTTCTTACTCGCAGGCT
CGTTGCGTTGATTGTTCCGCTTTCTTGTACTCCATTTGTGATGTCTTCAACTGGCTTTTCTTCTTGTTTCCCA
GCTTCTAGCTTCACAAACTCTAACGATACGTAACCTTCTTTTCCTTTATAATTGATCTTCGCCCAGCCGTTTG
CTTTTCCTAAGATTG
>Bacillus_Fam_784_232_1 Nr. of seq. 1 Alignment length(with gaps) = 232
Alignment score = 0.000000

TTCCATTTGACTCCTCTGTGTTAGACGGAGAAATTCCGTTTATCTCGGGAAATACTACTATTTCCCTTGAATT
AAGGGAAGCTTTTCCGCTTATATGATCCAAATCCTTGGATTTTGCCTTTTTTGAAGGTAGTAAGCGGAATTTC
TACCCTTATATCTCCCCTTTAAGCCGCAAAATTTTCATTAGACGGAAATTCTCCGCTTATGCTTCTCCACCCT
ATCCTTTGGCAAC
>Bacillus_Fam_785_231_1 Nr. of seq. 1 Alignment length(with gaps) = 231
Alignment score = 0.000000

GAAAAACTTGGTTGCCGCTAGATTTTCTGTAAATTGCCCTCTTTACTTGTCATTTGCTGTCTTTCCATTCGAA
GATAGCAGTTCAGCGGCAGCCTTAGCTTTTTCTTACCTCCCTTCAGCCCACGCTCTTTCACCCTTAATAATGG
TTGTTCAAGAAAAACAAGCCTGTTTTTCCACCTCTCTTTATGACAGGGAGGAATCACTTCTCTTTTTGCCTTC
AACCTTTATTTC
>Bacillus_Fam_786_231_1 Nr. of seq. 1 Alignment length(with gaps) = 231
Alignment score = 0.000000

GTTGTCGATAAATATTTAGTACTGACATATCCTTCTTTTCCATTAGCTTTGATCTTCGCCCAACCTTTAGATT
CCGAATACACCGTTACCTGCATGCCTTTCGAAAGCTTCGCAACTATGCTGGCAGTTTCCGTCCCACTTTTTCG
CATATTGAGCGTCCCAGAACTTACATTTACATATTTCGTCGTCGTTTTAACAGGGGTGGATGGTGTGGAACCC
GTTACAGGCTTC
>Bacillus_Fam_787_230_1 Nr. of seq. 1 Alignment length(with gaps) = 230
Alignment score = 0.000000

GAACAACCATTATGAAGGGTGAGAGAGGGAAAAGTTTGGGAAGTAAGAAAAGCTAAGGGCTCTGCCGAACTTC
CGTCTTTGAAGGGAGGAAAGAGGACTTCGAAAAGAATGCACTCTTTAAAAAGAATCTGGCAGAGCCCAAGCTT
TGAAACAGAGGTTTATAAGGTCTGAAAAAGAGCGCCCCTCCCCTATCATAAAGAGGGGAGGAAAAACAGCGGT
CTGTTTTTCTC
>Bacillus_Fam_788_226_1 Nr. of seq. 1 Alignment length(with gaps) = 226
Alignment score = 0.000000

ATTTACGTGCCGATTTGGCTTCTTTTCGGTCCGGAACGGAGCTCTTTCGTGCCGAAATCATCACTTTTCGAGC
GAGATTGGCGATTTTCGCGCCGGAGCTTAAATCTCTACGGTTCAACCACTTAATTACTATTTTTTTATGAACC
CCGTTGCTGTCCCAGTTATGTTAGCTATGGCACATCAGCTAATGCACATGCACAAACCATCATTACGGGCCGG
ATTTCAA
>Bacillus_Fam_789_226_1 Nr. of seq. 1 Alignment length(with gaps) = 226
Alignment score = 0.000000

GCTTCGCTTTCCTCGGGGCGAGCGCTGAGCTTCCTCGTCGTTTCTCTCCTGCGGGATCTCACCTGTCTCGCTC
ATCCCGTAGGAGTCTTCTCATCTTTCACTGCCTTCCTTTGGTCTTTCGACTAGGAACTATTTAGTAGCCTGCT
TTTTGGAATGTGTATTTCAGATAAGCTATGTTCATTCCACATGAAACACCCTTTACTTAGTCGGATTCCAGCT
CCAGATT
>Bacillus_Fam_790_225_1 Nr. of seq. 1 Alignment length(with gaps) = 225
Alignment score = 0.000000

AAGCTTGATGAGGTTTTAAACGGGAACTCTAGACCTGCCACTCTTCGCTTTGGTGACCGTCATCCGTACGTCA
TTCAGCTTAAAAAAGATTTAGCTGAAGCAGGATTCCCGGTCTCCGGTAGCCCGACGGAGTATTTTGGATCAGT
CACCGAATCCCAAGTCAAGGCTTTCCAACGAGCTCACGGACTTACTGCTAATGGCGTTGTCGGATCTGCTACT
TACGCA
>Bacillus_Fam_791_222_1 Nr. of seq. 1 Alignment length(with gaps) = 222
Alignment score = 0.000000

TTATCCAAACTGATTAACCACGCAAAAACATATGAAAAGGATAAATATACCGAAACTTCATACGGNGCNCTGC
AACAAGCAATTAGCCATGCNGAAGCAGTGGTACAAAATGCGAANACACAAGATGAAGTAACGGAAGCNATTAG
TCNTCTCCAAAAAGCGATTAATGGATTAGAAAAAATAGCAGAACCAGAACCAGAANCTGAAGTAGATACGGGC
GAG
>Bacillus_Fam_792_219_1 Nr. of seq. 1 Alignment length(with gaps) = 219
Alignment score = 0.000000

GATGGGTTTCTGCGGAGTACTTAAATGTCAGTGGGTCGGGCAGCGATGCGAGTGAAGGGTCTTCCTCCATTGG
ATCGGCTACCACNACCGCAAGGCTTAACCTTCGCTCCGGTGCAGGGACTTCGAATCGTATTTTAACGACATTG
AACAACGGGCAGAACGTNGCCTTGCTTCAGAAACAAGGCAACTGGTATCAGGTTAAAGCCGGTTCTCAGACGG

>Bacillus_Fam_793_214_1 Nr. of seq. 1 Alignment length(with gaps) = 214
Alignment score = 0.000000

TCAGGGTAGACCTTTATGCGAATAAGTATTGGCACCATGGAAGATGAAAAAGTTTTATACTCAGTGTGGAATG
AGCAGAGAGTCACCTGACTCCTGCGGGATCTAGCGGTCTCGTGAGACCCCGCAGGAGCCAAAAGCGACGAGGA
GGCTCACGGGGCGCCCCGCGGAAAGCAGGTGGATCGCAGCGAATGGAACTCCACTATCTAAGTTATTT
>Bacillus_Fam_794_213_1 Nr. of seq. 1 Alignment length(with gaps) = 213
Alignment score = 0.000000

CGCCTTAACTTGCGTTCTGGAGCAGGCACTAACCATAGCATTATCACTACCCTTGCTAAAGGACAAAAAGTCG
AGTTGCTTAAAAAGCAAGGTGGCTGGTACCAAGTTAAAGCGGGCAACCGTACTGGATGGGTTTCTGTTGACTA
CTTAAATGTCAGCGGCTCTGGCAATGTGGACAACGCTCCTTCTAACGGCTCTGCCACAACCACTGCA
>Bacillus_Fam_795_213_1 Nr. of seq. 1 Alignment length(with gaps) = 213
Alignment score = 0.000000

CCACCAGAAAAAGAAGGCCATTCATTCAAAGGATGGTATCAAGATGAGTTGCTCACAGAAGCGTGGGATTTCG
AAACGAACACGGTTAGCGAAGATATGACCCTGTATGCAAAGTGGGAAATCAACGTTTATACTGTTAGTTTTGA
ATCGAATGGNGGCAGCCAAGTATCGGAAGTAGAAGCCGAGTATGGTTCGTCNATCACAGAGCCAGCT
>Bacillus_Fam_796_210_1 Nr. of seq. 1 Alignment length(with gaps) = 210
Alignment score = 0.000000

GACAATCCGAATGCTGNTTACGGTCCAACGACGACAACTCGGGTGAAGGAATTCCAGAAATATTATGGATTAG
TGGTCAACGGCATTGGTGATGAAGTAACACTTGCTAAAATTGAAGAGATTTTAGCATCNCCGCTCCAAAACGG
AGGCAGGCATGAAGACGTGATAGCGTTAAAGGAAAACTTNTCTCGCCTCGGTTTCCATGTATCG
>Bacillus_Fam_797_208_1 Nr. of seq. 1 Alignment length(with gaps) = 208
Alignment score = 0.000000

AGGGTGAGAGAGGGAGGAGTTTGGGAGGTAAGAAAAACTAAGGCCTCCGCCGATCTGCCGTCTTTGGTAAAGG
GAATCGGACAGTTTTTAGGAGTGCTCTTCTTAACGGGATACAGGCAAGGTAGTGACGCTGAAAAACATCCCGA
TCCGCCCTGTCATAAAGAGCGGTGGAAAAGCAAGCTTGCTTTTCATGATTAAACTTTATGAC
>Bacillus_Fam_798_206_1 Nr. of seq. 1 Alignment length(with gaps) = 206
Alignment score = 0.000000

TCGTGCCTGCGGGGTCTCGGTCATCCCTCTTTTCCGAAGGAGTCCGGGCCCCTTCGCTTCTTTTCACTCTTGG
TGATACTTTGATTCCCTTTTTTCATTCATTGTTTCCGCCTAGTTCGTTTTTGGTTCTGCTTCATTGGTGTGAT
GGATTGGGCGTTCCATCCCGCTGTGGGACCGGCTTTTCTTGGGAGGGCGCCCGAGCCTCT
>Bacillus_Fam_799_206_1 Nr. of seq. 1 Alignment length(with gaps) = 206
Alignment score = 0.000000

AAAGAAAAGCGTAAACGCCCGTTTAGCGACGTACAAACTTTAATAATAAGGATGAACGGCTAAAGTCGCGCCG
TCCTGGCGCAACGCCGTTCTGACCAACGTCGTGTTGGCCCGAGATAAAGGAAACACAGCGAGGGACGAGCTGA
TGTTGACTTATCGTAGGAAGAAGCTGAAAGTTTGCTAGTCGTTGGGCGTTGAAGCTGGAC
>Bacillus_Fam_800_205_1 Nr. of seq. 1 Alignment length(with gaps) = 205
Alignment score = 0.000000

AAAGTGGAAGATCTGTTCACGAATGATAAGTTCGACACATTGAAAGGAAGTACCGATCAAAGNGCGATTGATG
AAGCTCAAAAAGCNGTCAATAAACTTCCGGAAGGTCCAGAAAAAAAATCGNCTTGAANGATTTAGTGAACAAAG
CACAAGATCTATTAAATAAAAAAAAACAAGCCGAAAAAGACTTGAACGATGCAAAGAAC
>Bacillus_Fam_801_196_1 Nr. of seq. 1 Alignment length(with gaps) = 196
Alignment score = 0.000000

CGAATTAATACGTGGATTGGACCGAAGTGGATCAAACCAAACAATCCACTAATAGGGGAACCAACTCCGATCT
CAACGAGGATNCACGATAACAGAAAATACCTATTTGCATAATCAACCGTGGAATAGTGCTAGAAGAAATGAAT
TTTTACGTCCGCAAACAGTGACAGCATTTGAAGAATGGAATGGATGGTAC

>Bacillus_Fam_802_195_1 Nr. of seq. 1 Alignment length(with gaps) = 195
Alignment score = 0.000000

TCCATCACCACAAAGTGCAATGCAGCGGAGACCGGCCGACTCCTTCGGGAATAGAGTGAAGTTCGATACCCCG
GACGCGCAGCTGAGGAGGCTCGACTCACTCCCCGAGGAAAGCGGACGGCCGCAGCGAAATGGAACGGACCTTG
ACTCCACCAAGATCCTCATCTAAAGCAGAAAGACATCATGCCTACCTCC
>Bacillus_Fam_803_195_1 Nr. of seq. 1 Alignment length(with gaps) = 195
Alignment score = 0.000000

ATAATCAACCATTTGCNAATACAAGAAGATCAGAAGTNTTACGTCCNCAAACGGTACAGGCAACAGGAGAATG
GAATGGCTGGTACCGAATTAATACATGGGTAGGACCAAAGTGGATTAAACCGTCGAATGCGATTGTAGGAGAA
ATTAAANAAATCTCGCAAANTATAACATTAACGCAAAATACGCATTTGC
>Bacillus_Fam_804_194_1 Nr. of seq. 1 Alignment length(with gaps) = 194
Alignment score = 0.000000

ACGTCGAATACACTAAGNCCGTTCCACTCGGATTGAGTTTGCTTATAAACACATCAAANTTCCGTTANAACTT
TGATCATATGCTCCNGCGGTCGTTGGGAAATTCGNTGATGTTGTATANCCNGTCACATACGCATTTCCATCCT
CATCGANNGCAATTCCATNTCCTNNATCANNATCACTTCCNCCTAAAT
>Bacillus_Fam_805_190_1 Nr. of seq. 1 Alignment length(with gaps) = 190
Alignment score = 0.000000

TTAAATCAAAATTGGGATTGTACATAGANATANGCAGTGTTCTAATCATCCTGCTGGGTTGATTTCCGTTGCA
GGCGCTCGCTTTCCGAGGCCTGCCGGGAGTCTCATCATAGTAAGCTCCTTCGGGGTTTCCATAAAGCCAGCTG
ATCCCGCAGGAGTCTCGCACCTTCCGCTCCAATCAACCTTTGAA
>Bacillus_Fam_806_177_1 Nr. of seq. 1 Alignment length(with gaps) = 177
Alignment score = 0.000000

CAGAAACAACAAATGCTGAAGTAGTAGCAAAAACAGAAGCCATCCAAAAAGCACTAGCAGGCTTAGAATTTGC
AGGCCAAACAGCCTTAAATGCAGCAAAAGCAAAAGCTGAGGAAAAGCAAGAAGCCGACTACACATCTGAAAGC
TATAGCCCATTAAAAGCAGCATTAGAACTGC
>Bacillus_Fam_807_172_1 Nr. of seq. 1 Alignment length(with gaps) = 172
Alignment score = 0.000000

TTACATATGGCTAGAGTAAAAGCTTTGTCGCTCTTTTACTTCGTCCATCATCGTTTGAATTTTTTGATCGTGT
AAAGCTTCGGTTTCAGAAAGCTTATCTTTTTGAATCGTAATTTTAAAAGAAAAAATTTGAAATATCATAATGT
AATCGCTCCTTTAGGTTGTTTTCGTA
>Bacillus_Fam_808_171_1 Nr. of seq. 1 Alignment length(with gaps) = 171
Alignment score = 0.000000

ATGTTGTTCCACGATTGAAGTTGCGCTACGGTTACGCCGAATTTCACTGCAATTCCACTAAGTGTATCACCTG
ATTTAACGGTGTACGTGGATACGGTTGAATCGCCACCACCACTCGTAGGAGCTTTAACTTTCAAAACTTGTCC
AATCTTAATCACATCAGCGTTTGAG
>Bacillus_Fam_809_168_1 Nr. of seq. 1 Alignment length(with gaps) = 168
Alignment score = 0.000000

TCTACCAATTTTAATNCCGAAAGTTCTTCCATCANTTCTTGATGTGTTTGTTTTAATTGTTCAAGTTCTTGGT
ACTGACTTTGTTCTTTCAANGTAGCNNCCGATAACTGTTCGAGTACTTCTTCATATGATTGNTTTAATTTTTC
TAGTTCTTCGCTTTGGACTTGT
>Bacillus_Fam_810_164_1 Nr. of seq. 1 Alignment length(with gaps) = 164
Alignment score = 0.000000

AACAGGGACGGTTCTCACTTGCCAGTTTGATAGTTTTTTTATGATTTGGTCATAGTTATGTGCGATTTCTTTC
AAGTTACACGCGATTTTGATCGAGTTACGCGCGATTTCACACGAGTTTGAAGCGATTTGGAAACCAACTGGAA
AAACACTTCAATTAGGCT

>Bacillus_Fam_811_164_1 Nr. of seq. 1 Alignment length(with gaps) = 164
Alignment score = 0.000000

GGTCACATTCCATGAGGTAAGATTCAGCTTGTTCAGTGATTTGATCACCGCACTCGGGACATTGTTTAGGTGG
TAGGGTTCTAAAGAACTCCAACGGATTTTTAGTTTGCATGATGGTTTCCTCCTTTAAATGGTTTAGTAGTCTT
CGCGTTTAGAAAGGCAGT
>Bacillus_Fam_812_156_1 Nr. of seq. 1 Alignment length(with gaps) = 156
Alignment score = 0.000000

ATAAAGTGAATCTTCCATCAGTGGAGGGTTTGCTTCATCCTCCACTGATGGTTAGTTGAACCAATCGGGGTTT
TACAGGCTGTTTTATCACTACACACTTCTCTCTATTTCTCCAACGAAAATGAAAGAGGGATTATTACAGCCTG
TTAAACCGGG
>Bacillus_Fam_813_156_1 Nr. of seq. 1 Alignment length(with gaps) = 156
Alignment score = 0.000000

TTCTTTCTTAATAGTATTTTTAAGAATTAACAGAAGAACCAGCTTGCGTGACTACTTCCTTTACATTGAATGC
TTTTAGAGGATTTTGCTTTTAGTTGTACATCTTTTGATACAGCTTCTTTTTGTAAAGATAATACTTTGTTCAT
TTCTCATTCC
>Bacillus_Fam_814_156_1 Nr. of seq. 1 Alignment length(with gaps) = 156
Alignment score = 0.000000

ATTTTGACTGGGGGCGGGAACCTAAGGGTGCGGTTGATTACCGAGAAGGAAGAAATTCCGGGGGTTCGGGAAC
CTAAGGGAGGGTTTGGTTACCGGGATGGAGAAAAATGNCTGTGNTCGGTAACTAAAGAGTGTGGTTGGTTACC
CTGGTGGGGA
>Bacillus_Fam_815_156_1 Nr. of seq. 1 Alignment length(with gaps) = 156
Alignment score = 0.000000

AAAAAGGACTGCCTGGCACTGGAGCAAACCCGCCTGAACCATTACCTAAAAAAACCGAGACATCATCGGAACC
AAAGTTCACGACCGCAAGATCCATAATCCCGTCTCCATTAAAATCTGCAGACGTGATGGAANTTGGACCCAAA
CCTCCACTAG
>Bacillus_Fam_816_155_1 Nr. of seq. 1 Alignment length(with gaps) = 155
Alignment score = 0.000000

CGCTTGCCATTCCGGACCACCACGTTTCTCCCAAATAGCAGCATAACGATCCTGACCATTACTACCGTAACCA
CTTACNTGCATATCAACCGATAGCCTTGCGCAACCAGTTCATTGAACGTCCGCTGATATTGCTCTGCTGTCAA
ACCATGTCG
>Bacillus_Fam_817_155_1 Nr. of seq. 1 Alignment length(with gaps) = 155
Alignment score = 0.000000

TGCTCTACCGACTGAGCTATCGAGCCATAATAATATTTAACTTACAACTTTATACTCCACTTGCAAAACGTCC
TCTGTCCCTTCCTCTTCTAGCTTATTCTTTGATGCTTATGCGTCGGGACAACTCGCAGCTGATTCGCTGATGT
GGTGCTCGT
>Bacillus_Fam_818_155_1 Nr. of seq. 1 Alignment length(with gaps) = 155
Alignment score = 0.000000

TTTGTTGTAACAAAAGCAGAAAAAACCATGCACAAATCCAGAATGCGCATGCATTTCAATTAATAGCTGAAGC
AAAGCCATGTGTAATATCCAGGACATGGAAGAAAAACCCATAAAGTCAGTTATAACAGTAAAAAAGTTCCACA
CCCGCATGA
>Bacillus_Fam_819_154_1 Nr. of seq. 1 Alignment length(with gaps) = 154
Alignment score = 0.000000

GGTTCTCCTATATCTCGCTCGGCATCAGTCCAGGTCATACGGCGATGAACAGGACCCTTTCGCTTTTCGTTTT
TGTCCAGCAGCAGGGCCCTATCGCCTAGTGTCCTTCCCTTACCTCGGGTCGATAAGTCGAAAACGAAAGGAGA
GCCCTTTC

>Bacillus_Fam_820_153_1 Nr. of seq. 1 Alignment length(with gaps) = 153
Alignment score = 0.000000

CTAGAAAACTCGTAAAACCCATGCGAATCTCACGGTTTCACATGGTTTTATCAAAAGATCTCAAGAAAAGCAT
GTGGAAGTGGAAAAGTTCTCCAAATATTGACCGTTTTAGAGCTAGAATTTCCAAACTATGGATGTTGCGCATG
CCATTTC
>Bacillus_Fam_821_150_1 Nr. of seq. 1 Alignment length(with gaps) = 150
Alignment score = 0.000000

TTCCGGCAATGAATTTTTAATGTGCTTCCAAACTCGATCGTCCACAAACACATCCGGAGTGGACAACGAAGTC
GAAAACAATGAGTTATAAGAATCTCCTCTCATCGGTTCAAAAATTTCTTTTGTCACCGGGTTAGGAAGCACAT
AGTT
>Bacillus_Fam_822_150_1 Nr. of seq. 1 Alignment length(with gaps) = 150
Alignment score = 0.000000

TATGGTGGATTGGAGCAAACGGCTCAGTGCAAGGGGCTTATTGGTATGAAGGAATGGCACGACGAGAGAGTTA
TGAATTAGCACCAGCGGGAAGCGCTTCTACCAATGGAGGTATTGCAGCGGTGTCGAGAAGGCCAAACACTTTC
GAAC
>Bacillus_Fam_823_150_1 Nr. of seq. 1 Alignment length(with gaps) = 150
Alignment score = 0.000000

ACTACCCACATAACGTTTACACGTCCNTGNTTATCTACGAAAAACAAATCAAGTTGCTCATCGGTTTGACGTG
CAGTTGCTNNNACCGNATCCAATGGAACGTAGTTNGGTGCAGTNAGAGGAATTGGTCCTTGCCAATTTCCGGT
TCCG
>Bacillus_Fam_824_148_1 Nr. of seq. 1 Alignment length(with gaps) = 148
Alignment score = 0.000000

GAACTTTTAAAAGCAGTAATGTAAGTTCATTATTCCGCAATTATTTATAAAAATTATCATACCTTAATACGAA
AAAAGAGGAGGAAATCATATGGCAATCGCAATGGCAGTTTTAAAATTTGTAGGTGGAGCACTTCCATTAGTAC
AA
>Bacillus_Fam_825_147_1 Nr. of seq. 1 Alignment length(with gaps) = 147
Alignment score = 0.000000

TTTTTCTTTGGTGGCTGCATCAAGATTTGCGAACATGCCGTGGCGTTCACCCTTTTCCGGAAGTGTTACACCA
AGCTTTTTCAGCTGGTCCTGTGCCTGGGTCTGTGTCAGGGTGCCGGCCTTCAGCTTTTCCATAATCGCCCGCG
C
>Bacillus_Fam_826_144_1 Nr. of seq. 1 Alignment length(with gaps) = 144
Alignment score = 0.000000

ATGAAATTACCGAGCTGACTAATCTCGATCCCTCAATGGAAGATACCAATTTGGATGAAGTGGCGTCTGTAAA
GGAAATCACTAATTCCATCGAGATTATTTCTACAAATGAAGATATCGAGACGATCGAAGGTGTCACTTCAC
>Bacillus_Fam_827_143_1 Nr. of seq. 1 Alignment length(with gaps) = 143
Alignment score = 0.000000

ACTTTTTCAGTGCCCTTTTATTTGTATTGCAATGGCTACGTTCGTTGCGCGCCTGATAGGATAAACCCACTCC
TATCAGGCTTTTAAACGATTGCAACGACTACGCACATTGCTTAGGGGCACTTCAAAAGTGAAAGAACACC
>Bacillus_Fam_828_139_1 Nr. of seq. 1 Alignment length(with gaps) = 139
Alignment score = 0.000000

TTTTGTGGAAAAGGGGACGGAGCTTACAGGATGATAAAGCAGGAAGCCTATCGCTCACACCCCTTCATTGAAA
GGTTACTCAGCGTTACTGGACGTACTCATTTTCATCCCAGTGGGAAGAGCGAGCCACTTATGCTAA
>Bacillus_Fam_829_135_1 Nr. of seq. 1 Alignment length(with gaps) = 135
Alignment score = 0.000000

GACGGTTCCCGTGCCTACACAGCAAATGTTAACAATGACACGGTTTCCGTGATCGGCACGGCCACGAATACTG
TCTTGACCACCATTTCGCTCGCTCCCGCAGGAAACGGTCCTNNTGCTATTGCCATCACGCCG
>Bacillus_Fam_830_132_1 Nr. of seq. 1 Alignment length(with gaps) = 132
Alignment score = 0.000000

CATCAATGACAGAAACNGTATCACTATTTTCNTCATTAGCTACATAAATACGATTATTAGAGGGATTAACACC
TACATCAGAAGGCTGANTCTCCGACAGGTACTATTCCAATAACAGCATTTGTNNACCTG
>Bacillus_Fam_831_132_1 Nr. of seq. 1 Alignment length(with gaps) = 132
Alignment score = 0.000000

TTTTCTTCCCAGAACAATTGAATTTGCTCCAATGCTCCAGAAATAATCTCACTTATAAATGCCATTGCCTCTG
AAATAATGGTCCCTACAACTTCCATCGTACTAGTCAATATTTCCATTATCCATGTGCCG
>Bacillus_Fam_832_132_1 Nr. of seq. 1 Alignment length(with gaps) = 132
Alignment score = 0.000000

TCGCGGGGTGGAGCATGGAGCAAAGCTTCTTGATTTTACATCGACATGTCGTGACGCATCAAGCTACAAAGCG
TTACGAGAAGAGGAAACGATGACCTCTTAGAAAAAACATCATCTGTAAGCTATATAACG
>Bacillus_Fam_833_131_1 Nr. of seq. 1 Alignment length(with gaps) = 131
Alignment score = 0.000000

CATTAAACCATNNACTTAAGANTAGCCCATTGTTCCATNACCTAATCCTTTACAGCATCCCACCCATCTGAAA
GCCAGTCCNANNGTTGTCAAAATNTCAACATCTTCCTATAATGGCTCCCATACAGTTT
>Bacillus_Fam_834_129_1 Nr. of seq. 1 Alignment length(with gaps) = 129
Alignment score = 0.000000

TTTTTGAACAATCACGAAACGATGAGCGAACAGCCGCAACGCTTTTAAGCCAGTCATGAACGCATTTGTCATG
ATTGGCGAGCGGGGGAGGAAGCCATCGTCCTACAAATAAAAAAGGGCGCTTTTCCC
>Bacillus_Fam_835_128_1 Nr. of seq. 1 Alignment length(with gaps) = 128
Alignment score = 0.000000

TGGTTTACAGAANATACAGGTAACAAAATAGGAAGAATNNCAACGGANGGTGAAATTACAGAATATNATATNC
CTACNNCAGATGCAGGTGCNTATNGATTATAGCNNNAGGTNCTGATGGTGCACTN
>Bacillus_Fam_836_127_1 Nr. of seq. 1 Alignment length(with gaps) = 127
Alignment score = 0.000000

TTTCTGTAATTGATGGGTTAACAAATACTGTAATTGCCACGATTCCAGTAGGAAACTCAACCGACCGGTATAG
AGATCAATCCATACATCAATCGAATTTATATAACAAATAGAGGTAGTAATACAG
>Bacillus_Fam_837_127_1 Nr. of seq. 1 Alignment length(with gaps) = 127
Alignment score = 0.000000

CGTCGTTTTTCCAAAAGGGAAATGCGCCCTTTTATTTTAGTTTACGATGGCTCGCTTTTGTCGCACGTCGGTT
ATGACAAATGCGCTCATAACCTACTTAAAAAACGCGACAGCTCCCTCATCGTAC
>Bacillus_Fam_838_126_1 Nr. of seq. 1 Alignment length(with gaps) = 126
Alignment score = 0.000000

GATGTTGAAGCATTAACAACAGAAAAAGAAGCTGTATCTTCTTCTCATNCTGAAGGAAAAGAAACACACGATT
CAGTAAGCAAGGAAGAGAAAGCATCATCTCTAGTTGATGTAAAAGCGGACTTA
>Bacillus_Fam_839_125_1 Nr. of seq. 1 Alignment length(with gaps) = 125
Alignment score = 0.000000

AGCGCCCTCCGCATTTCCTTGTCCAGCTTCGGGCGCTATCGGCTCGAGGGCAAATGCCCAGATGCCCAAGAGG
TGGAAGAGCGATCTTCCGTGTCATCTGGGCTTTGGCTGGTCGCCGATGGGCC

>Bacillus_Fam_840_124_1 Nr. of seq. 1 Alignment length(with gaps) = 124
Alignment score = 0.000000

GAACGTCCGAGCCGGCCGGCGGAGTGTCCGCCCAGCGCCGGAGTGACCGCTTTGCTCTTCGATGTGACCGCTC
ACCTCCTCCGAAGCGCCGTCCGCGGCCGCCTTCCGACCGCTTTTCCCGGCC
>Bacillus_Fam_841_124_1 Nr. of seq. 1 Alignment length(with gaps) = 124
Alignment score = 0.000000

GGAAGCGCCTTGGTCAGACCCGACCAGCATAAGTCGCTCCAGGATAGAAGGCGTCCTTTGCCTTCAATCCTGG
AGTGGCTTATGACTCGAGGGTCTAGGCGCTGGAGCTGGACAATCGAAAAGC
>Bacillus_Fam_842_123_1 Nr. of seq. 1 Alignment length(with gaps) = 123
Alignment score = 0.000000

GCTTCCGCTTTTCTTATTGTCCAGCTGCAGCGGCTAGCCCCTCGAGACACTTCGGTCCATCACCTGAAGGCAA
AGAACGCCTTCAACTGCTGGCCCTCCAGTGCTTTTCGGGGCTGGACAGCC
>Bacillus_Fam_843_122_1 Nr. of seq. 1 Alignment length(with gaps) = 122
Alignment score = 0.000000

CGAAAAGCGGAAGCGCTCGTTTAGCTCCGGGAGTCAGATAAGGAAGTGGCGGAAAAGGCGCTTTTTGCCTTTN
CNGACAATTCCGTTCTGACAGAGGAGCTGAGCGCTGGAGCTAGACAATG
>Bacillus_Fam_844_122_1 Nr. of seq. 1 Alignment length(with gaps) = 122
Alignment score = 0.000000

CGAAAAGCGGAGCCGACTGTTCAGTCCTNTCAGTCAGATAAGAAATCACCGGAAAAGTCCGGTTTTGACTTTT
TCGGGGATTTTGTTCTGACAGAAGGACTAGGAGGCGGAGCTGGACAATG
>Bacillus_Fam_845_122_1 Nr. of seq. 1 Alignment length(with gaps) = 122
Alignment score = 0.000000

GCATCATTCTCTTGTTTCCATTGGTTGTTGTTCATAAAAGCAACATCTCCTTTGGGTCATGAGAATGGTTTAT
ACTTATTAGNTTACAGGTATTCCATNTTTAGTCGCCGGGACTAAAAACN
>Bacillus_Fam_846_121_1 Nr. of seq. 1 Alignment length(with gaps) = 121
Alignment score = 0.000000

GAAATTCGCCGCAATCTCTCCTCCCCTGACGAACGAGAAGCAAGAAGTTCAAGGAGATGAGAGAGAGAGGACC
GGAGCGTATCTATAATACGTGAGGATCCGAGCGAGCGAAATCGACGCA
>Bacillus_Fam_847_120_1 Nr. of seq. 1 Alignment length(with gaps) = 120
Alignment score = 0.000000

TGGTATTACTTTGAGAGTAATGGCGTAATGAAAACAGATTGGTATTTTGATGGAACGAATTGGTTTTATATGA
ATAGCAATGGTGGAATGGAGACAGGATGGAAAAAGATTCAAGGAACG
>Bacillus_Fam_848_120_1 Nr. of seq. 1 Alignment length(with gaps) = 120
Alignment score = 0.000000

GAAGAAATCGATGTGATTCCTTTCCANGAAACCGACAATGCCGGGCAACGCGAAAAGCTGCCATCAGATGCGG
AAAATGAAACAGTTGGCGGTGCGANAGGTGAAGACACAGACGAACCG
>Bacillus_Fam_849_118_1 Nr. of seq. 1 Alignment length(with gaps) = 118
Alignment score = 0.000000

GCTTATAAGAAACCTCGCCTTTTTGAACTGCGCCGTGCTGTAGCAGCTGGCGGCCATTAATCTCATATAGCAT
TTGACATCTAGTACTCTATTTTAGGAGGGATGCTCAATGAAAAAA
>Bacillus_Fam_850_116_1 Nr. of seq. 1 Alignment length(with gaps) = 116
Alignment score = 0.000000

CAAAAGGTCGAACTTTACCTTTTGGGGATTTTACGTAACGAGAGCGAAGTCGTCACAAAGAAGAGCACAAATG
GAGTGAACTTCTCCATTAGTGCGAGTGACGAACGGAGCCTCGC
>Bacillus_Fam_851_115_1 Nr. of seq. 1 Alignment length(with gaps) = 115
Alignment score = 0.000000

CTCATAGAACGGCTATATGAGACAAAATTAGTTGAAAATCAAGCCAAAATGTCCCATAGAATTGCCCGGATCC
CCTGCTATGGGACAAAACCAGCGGAAGCCGAAGCAAAAATGG
>Bacillus_Fam_852_114_1 Nr. of seq. 1 Alignment length(with gaps) = 114
Alignment score = 0.000000

AAAAGGTATAGGGCGACATTTTGGGTCGGGAGACGCTCGTTGCTCGCCATTCAGAAGTACACTGAATGGCTTT
AAGAGCGGGCAACGGCTTTCCCCCTCTTACGAGAAAGAACC
>Bacillus_Fam_853_114_1 Nr. of seq. 1 Alignment length(with gaps) = 114
Alignment score = 0.000000

TTCCTCTGTTCCTTCACTTTCCGACTCCGCTGTTTTTTCCTTCTGCTTCGCGAGAGCCTGTGCTTTTGCCTTT
GCGGCTGCTGCGGCCTTTTTCTTCGCCAGGGCTAGTGCGTC
>Bacillus_Fam_854_114_1 Nr. of seq. 1 Alignment length(with gaps) = 114
Alignment score = 0.000000

CACTTGATTCAATGGAAGAAGATGACTCCTCGTCTGAGACCTCCGAATCAATGCTACTGGATTCATCCGGTGA
GGGGGCAGAGTCATCGCAACAGGTAACCGTTTCATCCTCCA
>Bacillus_Fam_855_111_1 Nr. of seq. 1 Alignment length(with gaps) = 111
Alignment score = 0.000000

TGTGGCGGAATGGCAGACGCGCTAGACTTAGGATCTAGTGTCTTATGACGTGGGGGTTCAAGTCCCTTCACCC
GCACTTTGAATTTTTAGAGTGATGAAAACTGAAGCGGT
>Bacillus_Fam_856_111_1 Nr. of seq. 1 Alignment length(with gaps) = 111
Alignment score = 0.000000

TTCGCTGCTGCGGCGGCTTTCTTTTTGGCTAGCTCCTTGGCGTCATCTGTTTCGGTTGCTGGAGCTTCGCCTG
CTTCACTCTTTTGTTTTGCCAGTGCGGCAGCCTTTGCT
>Bacillus_Fam_857_111_1 Nr. of seq. 1 Alignment length(with gaps) = 111
Alignment score = 0.000000

CAGCGGCTTTTTTCTTCGCTAAGTCGTCTGCTTCCCCAGCTGGCGGCGTTTCTGAGTCTGACTCTTTCGCTTT
CATTTTCGCGAGTGCGGCAGCCTTTGCTTTCGCTGCTG
>Bacillus_Fam_858_110_1 Nr. of seq. 1 Alignment length(with gaps) = 110
Alignment score = 0.000000

GATATGGCAAAAGTGTTTCATGTTTTATATGTGTCAAATATTANNGAAACANCTATAAAACGTTGATATATAA
GGGTNTATAAGATGTTTCNNATATTTATTAGGTCAAG
>Bacillus_Fam_859_110_1 Nr. of seq. 1 Alignment length(with gaps) = 110
Alignment score = 0.000000

AGGATACCGCAAACGTGTTGCAAGTTAAGTTATANGTATATGAATAAGAAAAAAACGNCTATCCTTTGATATA
TAAGGNATTCGAGTNAAATGACAAAAAAGTTAGGGTA
>Bacillus_Fam_860_108_1 Nr. of seq. 1 Alignment length(with gaps) = 108
Alignment score = 0.000000

ACAGGTCCAGTTGGTCCNCNAGGAGCGACGGGCCCCCCCGGNCCTACAGGAGCAGTAGGCCCAGCAGGGCCAA
CAGGAGCCCCAGGCCCGATCGGCCCAATGGGAGCG

>Bacillus_Fam_861_106_1 Nr. of seq. 1 Alignment length(with gaps) = 106
Alignment score = 0.000000

TTTTTAGGAGATAGCGAACCTGCAACATGCCTTTGCGGGTTCGCAATTGGATAATATTAGGAAACACCATTCC
TGTATAACTCATTTGCATCCTCGCACTTGGATA
>Bacillus_Fam_862_105_1 Nr. of seq. 1 Alignment length(with gaps) = 105
Alignment score = 0.000000

CTGAAACAGCAACTGGAAACGAAAGACGAGCAGATTCAGAAGCTAGAACAAGAAAAGCAAAAGTTAGAAACCC
AAATGAATCAGTTGCAAGGAGGGCCAGAAAAT
>Bacillus_Fam_863_105_1 Nr. of seq. 1 Alignment length(with gaps) = 105
Alignment score = 0.000000

AGCGCCCCATTCTAAAGGGAAAAATATATGTAGAAAGGCGCACAAATCGAAATAGCGGGCCATTGAGAGAAAG
AAAAAGCAATAGAATGGCCAGCAAAATAAAAT
>Bacillus_Fam_864_105_1 Nr. of seq. 1 Alignment length(with gaps) = 105
Alignment score = 0.000000

TATCTATGTCAACTGGTATAGCAATGAAGCAATTCGATCAAAGCTTAAAAAAACTAACGGCTCTTCCAACTGA
GAATCTAAACAGTGTAATAAATTCCTCATTGC
>Bacillus_Fam_865_105_1 Nr. of seq. 1 Alignment length(with gaps) = 105
Alignment score = 0.000000

CTTCAGACAGAGGCNGGCATGCCTTTCTCTTTGTTGTCCGAACCTACGGTAACTTCGGACTGTACTCGAGTTT
TTCTTGAGGTTAAGTGTCCAAACCTTCTNCGA
>Bacillus_Fam_866_104_1 Nr. of seq. 1 Alignment length(with gaps) = 104
Alignment score = 0.000000

GGACTCTGGTTGAGGATATCACGTATTTTGGGTCCAAACCTGAGCCTACTTCAGACTCTGGTTGAGCTCATCC
TGAAATGTGAGTCCGAACCTAGTACAACTTC
>Bacillus_Fam_867_104_1 Nr. of seq. 1 Alignment length(with gaps) = 104
Alignment score = 0.000000

TGGTTTCTTACCGTACCAATGATTTCCCTCTTTCCTAGGGCAAAATCAACGCTTGATTTTTACCGCCTGAACC
GTTTTTTCTCTTCCTTGGGAAAATTCATTAA
>Bacillus_Fam_868_104_1 Nr. of seq. 1 Alignment length(with gaps) = 104
Alignment score = 0.000000

GAGGTGTGTTTCGGAGCTAAACGCGACTATGAAATCCTATATATGGAAAATAGTGTCGTGGAAATGCTCTAAT
CACGACAGAATAATCCAATAAATGGATAACA
>Bacillus_Fam_869_104_1 Nr. of seq. 1 Alignment length(with gaps) = 104
Alignment score = 0.000000

TTTTCTGAATTGCAACGCTTTATGGGTTCTATGAAACCTGCTATATTACCACTTTTGAAGATTACAACCCATT
ACGGGAACTATGAACCGCACTATGCGACCGC
>Bacillus_Fam_870_104_1 Nr. of seq. 1 Alignment length(with gaps) = 104
Alignment score = 0.000000

CGTCAAGTCCCGGTGTANGATTAAAAATCCGGCGGGTTCCGAATGAAATCGGGCCAGATGGAAGACAAATCGG
GCGGGAATCAAGGTAAATCCGGCAAGCCGGG
>Bacillus_Fam_871_104_1 Nr. of seq. 1 Alignment length(with gaps) = 104
Alignment score = 0.000000

GAAAGCGGCGACTTTCAGAAAGAATCCGGCGGGAAAGCGAGGAAATCCGGCGAGANACATTCGAGTGACTGGT
TCGGGTGAAAAAAACGGGCGGGATTTAGAAG
>Bacillus_Fam_872_104_1 Nr. of seq. 1 Alignment length(with gaps) = 104
Alignment score = 0.000000

ACCCGCCCGATTATTCACTTTTCTCGCCGGATAATTGACCCGGATTTGACCCAGATTCCAGAACTGGCCCGAT
TTTCTTTGGATCCCGCCGCATTCGAGCTCAC
>Bacillus_Fam_873_104_1 Nr. of seq. 1 Alignment length(with gaps) = 104
Alignment score = 0.000000

ACAAGAAATTGGGTCTGAATAAAATCGTAATTCAGACAAGAGATCTGCTAAGGTGAGAAATCAAGTCCGAATG
GGCGGGGGATTCAGACAGAAAAGAGACTTCA
>Bacillus_Fam_874_103_1 Nr. of seq. 1 Alignment length(with gaps) = 103
Alignment score = 0.000000

TTTATTCTACCTTCACACGATTCTCCNCGTCATGACGGGACTTTAAAACATGGTTCTTACCCCTTCAGACCGG
GGCGTTCACTCTAAAAGGATATTAAATCGT
>Bacillus_Fam_875_103_1 Nr. of seq. 1 Alignment length(with gaps) = 103
Alignment score = 0.000000

AACTTTTTGGAAAAAGTGCTGAACTAAAGAGTGGAAACGCGGAACCGCCAGAGGAATGCTGAAAAAATGGAGA
TTAACGCTGAAATAGGGGAGGAAAACGCTG
>Bacillus_Fam_876_102_1 Nr. of seq. 1 Alignment length(with gaps) = 102
Alignment score = 0.000000

GGTTGGAAGCACATGGAACTCTGCAATCAAAAATATAGATACACTAAAATCATTCTCTGACCCTTTGAAAGAT
ACACGAAAAGCCCTTGCTTCAATTGCAGA
>Bacillus_Fam_877_99_1 Nr. of seq. 1 Alignment length(with gaps) = 99
Alignment score = 0.000000

AACAAAAATAACAGTAACGATGAAGATACAGAAACAACATCGAATGAAAAAATAAACAATACAAAACAAGATA
ACGATAATCAACAAAATTCTAACAAC
>Bacillus_Fam_878_99_1 Nr. of seq. 1 Alignment length(with gaps) = 99
Alignment score = 0.000000

CGAAGAAGAACTACATGTAGANAAACTACTAAGCATNAATTTGGAAAAAACGCTAGAAACGTATGCGCAAAGT
GGTGAGATAGCAGAACAAATNGAACG
>Bacillus_Fam_879_93_1 Nr. of seq. 1 Alignment length(with gaps) = 93
Alignment score = 0.000000

ATTACCCAACTAGAAATGAACCTGCACCTCCAGATACACAACACATAATGCAAATGATGACAAGTCTCGAGGA
TCGATTAAATAAACTCACTA
>Bacillus_Fam_880_90_1 Nr. of seq. 1 Alignment length(with gaps) = 90
Alignment score = 0.000000

CGTTCTACTTGCTGACGATCACCTTTTGCCTGATCGTTTGGCACTTCATTTCTTCCTGGGTTATTTATTCGAT
TAGCTACTTCCAATTCT
>Bacillus_Fam_881_84_1 Nr. of seq. 1 Alignment length(with gaps) = 84
Alignment score = 0.000000

ACTTCTCGTTGAACTGTCGTTCCATTTACGCTCACGTTTCCGCTATTTACTGATCCTTGATTCTTCACATTAT
CCTGTACGTTC

>Bacillus_Fam_882_84_1 Nr. of seq. 1 Alignment length(with gaps) = 84
Alignment score = 0.000000

AATTAGCAGATGGCTCAAGTCAAGTAACAGGCGGTTTAGGAACATTATCTGTAGGAGCAAATCAAATGGCAGG
TGGAGTAAATC
>Bacillus_Fam_883_84_1 Nr. of seq. 1 Alignment length(with gaps) = 84
Alignment score = 0.000000

AACTCTTTCTTTGCGCATTCATCAATTACTTCTTCGCTAATAAATGGTGCCACNCNTGTTAATCCTTTAATAC
CATTCGTTTCA
>Bacillus_Fam_884_84_1 Nr. of seq. 1 Alignment length(with gaps) = 84
Alignment score = 0.000000

AAGCTGTGGAACAAGAAGACACAGAATCGAAACAAGCTGATGCAGAAATCGCTGTTCAAGAAAAGGAAGAGCT
AAAACAAGTTG
>Bacillus_Fam_885_81_1 Nr. of seq. 1 Alignment length(with gaps) = 81
Alignment score = 0.000000

ACTCAGGTTGGTTGACTTGGTCATTAAGATCTTCAATAACTTCTGTGATTGGAGCAGAGTCAATAGGTATTTC
TGAACCTG
>Bacillus_Fam_886_78_1 Nr. of seq. 1 Alignment length(with gaps) = 78
Alignment score = 0.000000

AGGAGCAAGAATCGGAAGAAGAAGTAGCATTAAATTCTGATGCAAATCAACCAGAAGTAGAAGAACAATCACT
GCAAG
>Bacillus_Fam_887_78_1 Nr. of seq. 1 Alignment length(with gaps) = 78
Alignment score = 0.000000

CAGAGAACAATCAGGAAGGAACAGCGAAAACAGTGTTGGCAGAAAGTAATCAGGCAGGAGCATCAGAACCAGC
GTCGT
>Bacillus_Fam_888_76_1 Nr. of seq. 1 Alignment length(with gaps) = 76
Alignment score = 0.000000

TGAAGCTTGACGCGAAAAGATGAAGTAAAACGTTGATATAAGGGATAGAACTGCGCATAGCTTGACGTAAGCA
ACG
>Bacillus_Fam_889_75_1 Nr. of seq. 1 Alignment length(with gaps) = 75
Alignment score = 0.000000

TATTATCTGGGTCAGTTGCATCTTTAGGGTCTTCAATCGGATCTTCTTTATTAGTGCCCCCATCATTTTCATC
GA
>Bacillus_Fam_890_75_1 Nr. of seq. 1 Alignment length(with gaps) = 75
Alignment score = 0.000000

GAGCAAGATTCTGATTCCTCTCTAGATGAAGATGTTTCTCTTGATAACGACGCTTCTGAAGACCAGGATGCAG
AT
>Bacillus_Fam_891_74_1 Nr. of seq. 1 Alignment length(with gaps) = 74
Alignment score = 0.000000

CTTTCAATCCCCTAAATCGGGTCACATCTTTTGCTACGANNTATGAAGATTCGTTAANAATCTAACAGTTATT
N
>Bacillus_Fam_892_73_1 Nr. of seq. 1 Alignment length(with gaps) = 73
Alignment score = 0.000000

CACGCAAGTATCACTATGGTAATTTGACGGACATAACTCCACAACGAGATCCCAGAAAGAATCTCTAAAATGA

>Bacillus_Fam_893_72_1 Nr. of seq. 1 Alignment length(with gaps) = 72
Alignment score = 0.000000
 CAGTAACACCAGTTGCGCCAATCGAAAATGAAGTACCAAATTCAGAAGTGGCCGCTGGGCCAATGTCAACAC
>Bacillus_Fam_894_70_1 Nr. of seq. 1 Alignment length(with gaps) = 70
Alignment score = 0.000000
 TGAGTGCGTGGATTGAAATAACTACGNCNTTGANCAATGCAATATCNCAATTAGCTAGTCGCACTTTTTG
>Bacillus_Fam_895_69_1 Nr. of seq. 1 Alignment length(with gaps) = 69
Alignment score = 0.000000
 TAATTTCTGACTTTGTACCTTCAATAGATGTTCCGTTTGTTAATGTTCAGGTAAAGAGAATTATATGGT
>Bacillus_Fam_896_69_1 Nr. of seq. 1 Alignment length(with gaps) = 69
Alignment score = 0.000000
 GTCGCACCCTACATGGGTGCGTGGATTGAAATNAANGNGNCAATNAANNCGNATNNTTNATNAGNNNCN
>Bacillus_Fam_897_68_1 Nr. of seq. 1 Alignment length(with gaps) = 68
Alignment score = 0.000000
 GTTTTATCTGAACGTAGTGGGATATAAAGTTTAANTTCGCATAANAANCTNTNACCTTAGCCGANNNT
>Bacillus_Fam_898_68_1 Nr. of seq. 1 Alignment length(with gaps) = 68
Alignment score = 0.000000
 GTCGCACTCTATGTGAGTGCGTGGATTGAAATANCTNTTTGNNCCNGNTCAGCNTANNTTNCAGTACN
>Bacillus_Fam_899_68_1 Nr. of seq. 1 Alignment length(with gaps) = 68
Alignment score = 0.000000
 TCGCACTCTATATGAGTGCGTGGATTGAAATTCTTTCTNCTNTTTCTTTAGCCGTTCNNCGNTTGCAG
>Bacillus_Fam_900_68_1 Nr. of seq. 1 Alignment length(with gaps) = 68
Alignment score = 0.000000
 GTCGCACTCTTAGTGAGTGCGTGGATTGAAATNTAATGGCAACTACACCCAAAAGAAAAAGGCAAATC
>Bacillus_Fam_901_68_1 Nr. of seq. 1 Alignment length(with gaps) = 68
Alignment score = 0.000000
 TGGATTGAAATNNGATTANNCAGNNTTNAATGATTNNAATAATCNNTGTCGCACTCCTTGTGAGTGCG
>Bacillus_Fam_902_67_1 Nr. of seq. 1 Alignment length(with gaps) = 67
Alignment score = 0.000000
 GTCATATCTCCATCTAATCCGTTGCTGTGATATGATANNNGCNNTCANCANNTNGATGNTGNNAANN
>Bacillus_Fam_903_67_1 Nr. of seq. 1 Alignment length(with gaps) = 67
Alignment score = 0.000000
 AAAAACTGAAAGAGTGCCAAAACTANGTTNANATNGTNCATNTTNNTCNANANNGTTTTCCTATACC
>Bacillus_Fam_904_67_1 Nr. of seq. 1 Alignment length(with gaps) = 67
Alignment score = 0.000000
 GGTACTCTCTCAGTTTTCGGTATAGAGAAACAAGTCCACGANTGTGNAAAGANTACGATTNNGTTTT
>Bacillus_Fam_905_66_1 Nr. of seq. 1 Alignment length(with gaps) = 66
Alignment score = 0.000000
 ATCTTTTGATAATTCGTAGCCCGCTGGTGCTTCTATTTCAACGATTTTGTATTCACCTATCGGTAA
>Bacillus_Fam_906_66_1 Nr. of seq. 1 Alignment length(with gaps) = 66
Alignment score = 0.000000
 ATCATATCACAGCAATAATCTTAGGGGAACTATGACNGNTNNTNNNGANNNATNTNNCGNTNNNNN
>Bacillus_Fam_907_66_1 Nr. of seq. 1 Alignment length(with gaps) = 66
Alignment score = 0.000000
 GTCGCATCCCACGCGGATGCGTGGATTGAAATGNCNANCATGNCGAGATANANCCGNNANTCTNGN
>Bacillus_Fam_908_66_1 Nr. of seq. 1 Alignment length(with gaps) = 66
Alignment score = 0.000000
 GACAGCACCACCAACGCTACTGATACTTCCACCGCTACAAGCGGCTCCACCAGCGAGTCCGACACG
>Bacillus_Fam_909_66_1 Nr. of seq. 1 Alignment length(with gaps) = 66
Alignment score = 0.000000
 ATCATAATACAGCAATGGCTTTAATGGAACTATGACNTACAAATGTANGANAAATATTNGATTTNA
>Bacillus_Fam_910_66_1 Nr. of seq. 1 Alignment length(with gaps) = 66
Alignment score = 0.000000
 GGATTGAAATNNCGNTNGTATGCTNANNAGGTTCGTTGGCNGNCGTCGCATCCCATGTGGGTGCGC

>Bacillus_Fam_911_66_1 Nr. of seq. 1 Alignment length(with gaps) = 66
Alignment score = 0.000000
GTCGCACTCTTCATGAGTGCGTGGATTGAAATNTTATANTCNTGATGGTCGAGNGCATNNGATCGN
>Bacillus_Fam_912_65_1 Nr. of seq. 1 Alignment length(with gaps) = 65
Alignment score = 0.000000
ATTTCAATCCTCGCACTCACATGGAGTGCGACCANTAAGTCTATTNCTGTTATGCAAGANNGNCT
>Bacillus_Fam_913_65_1 Nr. of seq. 1 Alignment length(with gaps) = 65
Alignment score = 0.000000
TCCACGCACTCACATGGAGTGCGATTACGNCNNANNNATNNCGNNGANAACGTCNGANATTTCAA
>Bacillus_Fam_914_65_1 Nr. of seq. 1 Alignment length(with gaps) = 65
Alignment score = 0.000000
GTCGCACTCTTTGTGAGTGCGTGGATTGAAATAANCATNTTTCTGTAANCGGCAAAATAATNCNG
>Bacillus_Fam_915_63_1 Nr. of seq. 1 Alignment length(with gaps) = 63
Alignment score = 0.000000
TTTTTTCTACCGCAAAGAGAGATATTCCTACCACAAANAGCAATAATCCTACCGAAAATCCGA
>Bacillus_Fam_916_63_1 Nr. of seq. 1 Alignment length(with gaps) = 63
Alignment score = 0.000000
CAAGAAATAGAAAATTTAAATGAAACAATCATCAGTTACAAAACAAAAGAAACTGAACATAAA
>Bacillus_Fam_917_63_1 Nr. of seq. 1 Alignment length(with gaps) = 63
Alignment score = 0.000000
AATACCATTTTNCNCCNGATTGAACCCATGCTTTTGTTACCATAGCTCCATCAGCACCTAAGT
>Bacillus_Fam_918_63_1 Nr. of seq. 1 Alignment length(with gaps) = 63
Alignment score = 0.000000
TTTGTCCCATAGACCGTGAATCCGGGAATTCTATGGGACATTTTATTTTACTTTTCAACTAAA
>Bacillus_Fam_919_63_1 Nr. of seq. 1 Alignment length(with gaps) = 63
Alignment score = 0.000000
TAGGAAAACTATCTGACGGATCTACTCAGCTAGCAACAGGAAAAGGCGATTTATCAAATGGTT
>Bacillus_Fam_920_63_1 Nr. of seq. 1 Alignment length(with gaps) = 63
Alignment score = 0.000000
AGAGTACCAAAACNTNNNNANANNNNTNNGNTANTANGTNGTTTCTCTATACCGAAAACTGAG
>Bacillus_Fam_921_63_1 Nr. of seq. 1 Alignment length(with gaps) = 63
Alignment score = 0.000000
GAAAAAGAGCAGCAAGCCAGNCTTACCATCCAAGAGCTGGAGAACAAAATCCAACACTCTTTG
>Bacillus_Fam_922_62_1 Nr. of seq. 1 Alignment length(with gaps) = 62
Alignment score = 0.000000
ATGAAAGCCATNATGAAGGAAAAACGCGGGTNAANTNGCTTTCATCTGTAAAAAAGATCCAA
>Bacillus_Fam_923_61_1 Nr. of seq. 1 Alignment length(with gaps) = 61
Alignment score = 0.000000
AATCGAAACAAGGACGAAATACTCCAATGTAGGCCGAATTAATAGCAGTCAAGGCCGGATT
>Bacillus_Fam_924_60_1 Nr. of seq. 1 Alignment length(with gaps) = 60
Alignment score = 0.000000
AATACCAAGTAGATCCTTCTTTTAACCAGCCAGTAGCCATAGAACCATTTGCATTTAGAT
>Bacillus_Fam_925_60_1 Nr. of seq. 1 Alignment length(with gaps) = 60
Alignment score = 0.000000
TGGTATTACTTGAAAGAAAATGGATCNATGATGACAGGTTGGACGTTAGTAAATGGTNAG
>Bacillus_Fam_926_60_1 Nr. of seq. 1 Alignment length(with gaps) = 60
Alignment score = 0.000000
TGGTATTATTTAAACGCTAATGGAGCAATGAAAACGGGCTGGCTATTANATCAAGGAGTT
>Bacillus_Fam_927_60_1 Nr. of seq. 1 Alignment length(with gaps) = 60
Alignment score = 0.000000
TAGTACCAAGTATTANCAAGNTGAAGCCATCCNGTATGCATNNCACCGTTATTNTTTAAN
>Bacillus_Fam_928_60_1 Nr. of seq. 1 Alignment length(with gaps) = 60
Alignment score = 0.000000
ATTTACATTTTTCTTACATTCACTCTCTTGTGATACCCTTGCTTTTACCTCATCCTTTGA

>Bacillus_Fam_929_60_1 Nr. of seq. 1 Alignment length(with gaps) = 60
Alignment score = 0.000000
ATCACAACACCACAGTCAGGAACAACACCATCACCAGCGAATCCGATGGAAAGTAAGCCA
>Bacillus_Fam_930_60_1 Nr. of seq. 1 Alignment length(with gaps) = 60
Alignment score = 0.000000
CCCAACCAGTAGCCATCGCACCACTAGGATGCATGAAGTACCAGGTGCTCCCATCNNNTA
>Bacillus_Fam_931_60_1 Nr. of seq. 1 Alignment length(with gaps) = 60
Alignment score = 0.000000
AGGTAGTACCACTTCCCACCTGTATAAAGCCAACCGGTCTTCATCGAGCCATTACTAGTC
>Bacillus_Fam_932_60_1 Nr. of seq. 1 Alignment length(with gaps) = 60
Alignment score = 0.000000
TCAGCCAGCCNTTNTGAATNAGACCGTTTGATNCAAAGTAATANTGCTNNNATCCCTNGG
>Bacillus_Fam_933_60_1 Nr. of seq. 1 Alignment length(with gaps) = 60
Alignment score = 0.000000
AAGAGCGGAGCCATGCAAACGGGCTGGCTGAAAGACGGATCGAAATGGTACTACCTCGGT
>Bacillus_Fam_934_60_1 Nr. of seq. 1 Alignment length(with gaps) = 60
Alignment score = 0.000000
GGAGTAATGAAAACTAGTTGGTTAGTATTAGGTAATAAACATTATTACTTTGATAAATAT
>Bacillus_Fam_935_60_1 Nr. of seq. 1 Alignment length(with gaps) = 60
Alignment score = 0.000000
TGACGGTAGCCATGAGGAGGCAGGACATGAAGACGAAAATCATGAAGCTGACGCTCATGG
>Bacillus_Fam_936_58_1 Nr. of seq. 1 Alignment length(with gaps) = 58
Alignment score = 0.000000
TGGTATTATTTTGATNNNACCGGTGCAATGAAAACAGGNTGGCTGCTATNGTGGCAAN
>Bacillus_Fam_937_58_1 Nr. of seq. 1 Alignment length(with gaps) = 58
Alignment score = 0.000000
AGGATGGCGACATTCCGCTGTTACTCACAGGACGTGAGTGGTACTTAAGCGGAATTGG
>Bacillus_Fam_938_57_1 Nr. of seq. 1 Alignment length(with gaps) = 57
Alignment score = 0.000000
TTTCCAATGCCAAGCCACCATGGAGGAATGATGCCAATGCCAGAGCAAAGTGGAGGA
>Bacillus_Fam_939_57_1 Nr. of seq. 1 Alignment length(with gaps) = 57
Alignment score = 0.000000
AATGACTCATAAATACTCGATTCCTTTTTTATCATGGATTCTTCAGCTGGTCTTATT
>Bacillus_Fam_940_57_1 Nr. of seq. 1 Alignment length(with gaps) = 57
Alignment score = 0.000000
CAAATGTTGGAAATGTAGTTGGTGCTCCTACTGGAACTCCGCCTTGCGCCCCTCCTA
>Bacillus_Fam_941_57_1 Nr. of seq. 1 Alignment length(with gaps) = 57
Alignment score = 0.000000
GGGCCTACTGGACCTACCGGAATAACTGGAGCAACTGGACCTTCTGGTGGACCTCCA
>Bacillus_Fam_942_56_1 Nr. of seq. 1 Alignment length(with gaps) = 56
Alignment score = 0.000000
TTTGGAGGTACTTCCTTTTAGCTATTCGCTAGCATGATGTGACNTCATGACACTCT
>Bacillus_Fam_943_55_1 Nr. of seq. 1 Alignment length(with gaps) = 55
Alignment score = 0.000000
GTCTTCCTTAACNTAGANGCGNTTCTACGTTAAGATACCCGGAAAAATTCCGANG
>Bacillus_Fam_944_55_1 Nr. of seq. 1 Alignment length(with gaps) = 55
Alignment score = 0.000000
GGACCATGATTAAGCTGCTATTTGTTGGTAGTTTTATACATAGCACTTGATTCGC
>Bacillus_Fam_945_55_1 Nr. of seq. 1 Alignment length(with gaps) = 55
Alignment score = 0.000000
ATGAAGGACAANTTGAAGGAAAGAATCGAAGGAAAATCAGTCCTTCATCGGAAGT
>Bacillus_Fam_946_55_1 Nr. of seq. 1 Alignment length(with gaps) = 55
Alignment score = 0.000000
CTTTATTTTGACCGGTGACCTCCTTCTTGGTACCTTTGCGGGAAGAATCNGACGN

>Bacillus_Fam_947_55_1 Nr. of seq. 1 Alignment length(with gaps) = 55
Alignment score = 0.000000
CTCATGAACCCTCTATGAGAACAATTGCNAGCACCCTCCTTCANCCCAGTCTGTC
>Bacillus_Fam_948_54_1 Nr. of seq. 1 Alignment length(with gaps) = 54
Alignment score = 0.000000
CCCGTCTTTCAGCATGATGTTCAGGCAAATCCGGGGGAACACGGCGCTCTATTT
>Bacillus_Fam_949_54_1 Nr. of seq. 1 Alignment length(with gaps) = 54
Alignment score = 0.000000
TGTCTTTCATCANACCGATGAAGGACAAATAGCTACCAGCCACNCCTTGTAAAA
>Bacillus_Fam_950_54_1 Nr. of seq. 1 Alignment length(with gaps) = 54
Alignment score = 0.000000
TTCAGTGCCATCAAAACCGATTCCGGACGCTTCTTGAGGGTTCTTATCCTGNTG
>Bacillus_Fam_951_54_1 Nr. of seq. 1 Alignment length(with gaps) = 54
Alignment score = 0.000000
ACAAAGACTTCCGGCTCTTCTTGGNTTTCCTCTACAACTTCTGCTTCAGGTTCC
>Bacillus_Fam_952_54_1 Nr. of seq. 1 Alignment length(with gaps) = 54
Alignment score = 0.000000
ATTGTTGGAAACACAAACATCACGATGATTGTTCAAAACATAAACATCATTGTG
>Bacillus_Fam_953_54_1 Nr. of seq. 1 Alignment length(with gaps) = 54
Alignment score = 0.000000
AGAGAAAGAAAGTGAAGAAACTGAATCAAAAAAAGAAGATAAAGCAATTGAATT
>Bacillus_Fam_954_54_1 Nr. of seq. 1 Alignment length(with gaps) = 54
Alignment score = 0.000000
CTGACAGCCCTCCTCAGTGCCCGTTTCTTGGGAATAGGAAACCATTTTAGGGCT
>Bacillus_Fam_955_54_1 Nr. of seq. 1 Alignment length(with gaps) = 54
Alignment score = 0.000000
ATGGGGCCAACAGGAGCCACCGGATTAGCAGGAGCAACCGGGCCAGCAGGAGTA
>Bacillus_Fam_956_54_1 Nr. of seq. 1 Alignment length(with gaps) = 54
Alignment score = 0.000000
TTTGTCTCTGAGCACGTCTATCAGAGACATTAATTATCCAATTAAAAGCGAAGA
>Bacillus_Fam_957_54_1 Nr. of seq. 1 Alignment length(with gaps) = 54
Alignment score = 0.000000
TAAGCCAGTAGCGCCAGTAGGCCCAGGATCGCCGGTGGCCCCAGTAGCCCCAGC
>Bacillus_Fam_958_54_1 Nr. of seq. 1 Alignment length(with gaps) = 54
Alignment score = 0.000000
CTCAGCCAGCAGCTCGGTTTCTTCTGCAGCCTCTTCGAGTAATTCTGTCTCATT
>Bacillus_Fam_959_54_1 Nr. of seq. 1 Alignment length(with gaps) = 54
Alignment score = 0.000000
AAAGCAGAAGAGCAACAGCGTGAAGCATTGAAGAAACAAGAAGAGGCCCGTAAA
>Bacillus_Fam_960_53_1 Nr. of seq. 1 Alignment length(with gaps) = 53
Alignment score = 0.000000
GGTCGACATGATCGAGGAGAAAATAGAGAAAGAACAGGTCGATGAGTAGCCGA
>Bacillus_Fam_961_53_1 Nr. of seq. 1 Alignment length(with gaps) = 53
Alignment score = 0.000000
ATATGAGTCACTNTGGTGGTGGNGAAAGCCGNACAGAGTGACTCAAGAGGAAG
>Bacillus_Fam_962_53_1 Nr. of seq. 1 Alignment length(with gaps) = 53
Alignment score = 0.000000
GCTTCCTTAACATAGAAGCGCTCTACATTAAGGAAAGGCGAAAATTCGTGACN
>Bacillus_Fam_963_53_1 Nr. of seq. 1 Alignment length(with gaps) = 53
Alignment score = 0.000000
GAATCGGTAGGGTCAAAAGAGCACTTAGACATGGTCGAATCCAGGTAAGAGAA
>Bacillus_Fam_964_53_1 Nr. of seq. 1 Alignment length(with gaps) = 53
Alignment score = 0.000000
GTAGGTCATAAGGGTCTCAACATGAAAATGAAGCCATGCGCTCGTCCCGCGCG

>Bacillus_Fam_965_53_1 Nr. of seq. 1 Alignment length(with gaps) = 53
Alignment score = 0.000000
TCTCAATGGGCTAATTACGTCGTTTACGGGCCAACACCTCATCAAAAAGTCAA
>Bacillus_Fam_966_53_1 Nr. of seq. 1 Alignment length(with gaps) = 53
Alignment score = 0.000000
TACATGAGTTACTTCAGATGGTCCGACTCATGTATGAAAAACTNGATCNTTGA
>Bacillus_Fam_967_53_1 Nr. of seq. 1 Alignment length(with gaps) = 53
Alignment score = 0.000000
GTCCCTTTGACGACTCCGATTCTCCCTGACTTCCGATTCCACACCCTCATTGA
>Bacillus_Fam_968_53_1 Nr. of seq. 1 Alignment length(with gaps) = 53
Alignment score = 0.000000
CTAAAGACCAAAACGAATGAAATTCCAGGTGTAAAAGTGTCTTTAGAGACGTT
>Bacillus_Fam_969_53_1 Nr. of seq. 1 Alignment length(with gaps) = 53
Alignment score = 0.000000
AAAGGGTGTAAACGGGCCAACGAGAAAGCAGAACCAGAAGGATGCTGGCCCGC
>Bacillus_Fam_970_53_1 Nr. of seq. 1 Alignment length(with gaps) = 53
Alignment score = 0.000000
CATCAAAAGGTGTGGACGGTGAAAATGATGTATGAAAAGAGGGATGATTCCAG
>Bacillus_Fam_971_53_1 Nr. of seq. 1 Alignment length(with gaps) = 53
Alignment score = 0.000000
ACGTTTCTTCTCTAATCGTGCACCTTCACNTCAGATTCCCGCCTTCTAGGGGC
>Bacillus_Fam_972_53_1 Nr. of seq. 1 Alignment length(with gaps) = 53
Alignment score = 0.000000
GTTGTTGAAGGGTAAAGTTTAAGAGCTAAACGTACCCCTTGAGTGCAGATTGA
>Bacillus_Fam_973_53_1 Nr. of seq. 1 Alignment length(with gaps) = 53
Alignment score = 0.000000
GTACATCATTCGAGAGTCAACCAGGAAATGACACCCCGCGCGGGAGGAGCGCG
>Bacillus_Fam_974_53_1 Nr. of seq. 1 Alignment length(with gaps) = 53
Alignment score = 0.000000
CCAACATCTCGAGGAATTCTGAATCACAATGGTCCGATAACCCTCTAAACGGA
>Bacillus_Fam_975_53_1 Nr. of seq. 1 Alignment length(with gaps) = 53
Alignment score = 0.000000
GTAAACGTACCACTACAGAAGGNGAATCGGGCTTCTAATGGTACGATTAGCTT
>Bacillus_Fam_976_53_1 Nr. of seq. 1 Alignment length(with gaps) = 53
Alignment score = 0.000000
AAAAGAGAATCGACCCTGTCGAAGAGGCGTAAAGACCAGGTCGAATGGTTGCA
>Bacillus_Fam_977_53_1 Nr. of seq. 1 Alignment length(with gaps) = 53
Alignment score = 0.000000
GGCTTCATTTGTGGATTCAGGTGCTTATGACAACACGCGCTCGAGGTGCGCGG
>Bacillus_Fam_978_53_1 Nr. of seq. 1 Alignment length(with gaps) = 53
Alignment score = 0.000000
CATCATTCTGGGCCGCCGTGAAAAATGATGTAGGAAAACAATCGGAATTAGGG
>Bacillus_Fam_979_53_1 Nr. of seq. 1 Alignment length(with gaps) = 53
Alignment score = 0.000000
TTGGTACATCAAATCAAGGTGGTCCACGCAATGATGTCCTAATAAAGTCAGAA
>Bacillus_Fam_980_53_1 Nr. of seq. 1 Alignment length(with gaps) = 53
Alignment score = 0.000000
TGGCCCATTTAACCATTTAATGGACCAACACCCTTTAGAGTCGCAACTCTCAT
>Bacillus_Fam_981_53_1 Nr. of seq. 1 Alignment length(with gaps) = 53
Alignment score = 0.000000
GTCATTTTGTGAGAATTGGTCGAATGACACCCNGCGCCCCGTCCGCGCGGTAG
>Bacillus_Fam_982_53_1 Nr. of seq. 1 Alignment length(with gaps) = 53
Alignment score = 0.000000
CACTGGCCCAATAATCCAATAAATGGGCCAACATGATCTGGTTTTAGCAATCT

>Bacillus_Fam_983_53_1 Nr. of seq. 1 Alignment length(with gaps) = 53
Alignment score = 0.000000
AAATGATGACATGCGCGGCGNGNGCGCGGGGTGTCATTGTGTTGGATTTGCGG
>Bacillus_Fam_984_53_1 Nr. of seq. 1 Alignment length(with gaps) = 53
Alignment score = 0.000000
GTAATCGACCCAACATCTTCTCCAAAACCCAATCTCCTTGGGCTGTTTACCTC
>Bacillus_Fam_985_53_1 Nr. of seq. 1 Alignment length(with gaps) = 53
Alignment score = 0.000000
ATACCATTTATCGTTGCTCGGCTTCGTGATATTCCGTTTACGCCGTTTGCGGT
>Bacillus_Fam_986_53_1 Nr. of seq. 1 Alignment length(with gaps) = 53
Alignment score = 0.000000
ATGAAGAGGATACACCACGAATGAAAGCTACGAAATCCCCTTCATGAGAGCTA
>Bacillus_Fam_987_53_1 Nr. of seq. 1 Alignment length(with gaps) = 53
Alignment score = 0.000000
GAGGAAAGTCAGAAGGAGAAGCTNNGNACGCGTCGGCAGGTTCAGACTTTAGG
>Bacillus_Fam_988_53_1 Nr. of seq. 1 Alignment length(with gaps) = 53
Alignment score = 0.000000
TTGTCCGAATAANCCTTCTATTCGGACTANAACCACTTGGTGCTTCCNTCNTC
>Bacillus_Fam_989_53_1 Nr. of seq. 1 Alignment length(with gaps) = 53
Alignment score = 0.000000
TTTGTCTCATGGAGCNTTCATGTTACATGAGTTACTCCCTTTCTTGAGGAAAG
>Bacillus_Fam_990_53_1 Nr. of seq. 1 Alignment length(with gaps) = 53
Alignment score = 0.000000
ATTAGTGACCGTTTTCTNTTGCAACTACAAAAAACGGGCATCTANTACACTCG
>Bacillus_Fam_991_53_1 Nr. of seq. 1 Alignment length(with gaps) = 53
Alignment score = 0.000000
ATTATAACGTAAGGAAACACTCGAATCGTACGCTATACGTACACCGGATGAAA
>Bacillus_Fam_992_53_1 Nr. of seq. 1 Alignment length(with gaps) = 53
Alignment score = 0.000000
CCAGAAATGGTGACCGGAAACAGAGAAAAGTGGCGTCACTAAGGCGAGTGGCG
>Bacillus_Fam_993_53_1 Nr. of seq. 1 Alignment length(with gaps) = 53
Alignment score = 0.000000
AGAGTGCTGCCACTTTTGCCGTTGAGGGATCATCGGAAGTGGCGTCAATCCAC
>Bacillus_Fam_994_53_1 Nr. of seq. 1 Alignment length(with gaps) = 53
Alignment score = 0.000000
TGACGCCACAAAGCTGTTTGCAATATAAGTGAAAGTGGCGCAACTCNCCGGAG
>Bacillus_Fam_995_53_1 Nr. of seq. 1 Alignment length(with gaps) = 53
Alignment score = 0.000000
TGCCACTTTTTGCGTGATGGGTTTGCCAATTGTGGTGTCATTGGCCGTAGTGG
>Bacillus_Fam_996_53_1 Nr. of seq. 1 Alignment length(with gaps) = 53
Alignment score = 0.000000
CGGGCTCCTATTTCAACGATTGGCTACCGTTTCCACTCCTTGAGAGCTACGCT
>Bacillus_Fam_997_53_1 Nr. of seq. 1 Alignment length(with gaps) = 53
Alignment score = 0.000000
GTTTTGTCCTCAAGACCCCCGATTGAGGACGAATCCTCTGGGGACTCTTGNTA
>Bacillus_Fam_998_53_1 Nr. of seq. 1 Alignment length(with gaps) = 53
Alignment score = 0.000000
AAGAAAGATACCCTTATGCAGCCGTTTGTCATCACATAAGGGTACGAAAACCG
>Bacillus_Fam_999_53_1 Nr. of seq. 1 Alignment length(with gaps) = 53
Alignment score = 0.000000
TATTGGTTACCTATAATTCTAGTTTCAGCCCTTTTTAGGGCACCAATAACGCC
>Bacillus_Fam_1000_53_1 Nr. of seq. 1 Alignment length(with gaps) = 53
Alignment score = 0.000000
GTACCCTTATGGAGAGCTTTCGCTTGGTATAAGGATACGATTAGGAGAAAATC

>Bacillus_Fam_1001_53_1 Nr. of seq. 1 Alignment length(with gaps) = 53
Alignment score = 0.000000
CTTCAGACAAAATCTGCACGCCTAAGACGTGGTTTTGGCCGAACATGGCATCA
>Bacillus_Fam_1002_53_1 Nr. of seq. 1 Alignment length(with gaps) = 53
Alignment score = 0.000000
TATCCTTATGAGGTAAAATCCAGCAACATAAGGGTACCGTTCAAGCCNAATCG
>Bacillus_Fam_1003_53_1 Nr. of seq. 1 Alignment length(with gaps) = 53
Alignment score = 0.000000
CCTAAAAAGAGGAAAATGGTGACCGTCAAAACGAAAGCAATGGTCACCAATCT
>Bacillus_Fam_1004_53_1 Nr. of seq. 1 Alignment length(with gaps) = 53
Alignment score = 0.000000
GTATTGCGACACAAAGCAAGAATTTTCAGCAAGCCGAGTCCGAATANAGCNTC
>Bacillus_Fam_1005_53_1 Nr. of seq. 1 Alignment length(with gaps) = 53
Alignment score = 0.000000
CTTATGAAGACCTTTTCGACGAGCCACGAGTTCTGATTCGAGTCTTCATCACG
>Bacillus_Fam_1006_53_1 Nr. of seq. 1 Alignment length(with gaps) = 53
Alignment score = 0.000000
ATGAAAGACAAAACTCAGGAGAATGGGTGAGGGAATTGTCCTTCATCCGGTGT
>Bacillus_Fam_1007_53_1 Nr. of seq. 1 Alignment length(with gaps) = 53
Alignment score = 0.000000
GATGAAGCCCAAANCCCACTAAGCCCACNCAAGGATATGTNCTTCATAGGCNC
>Bacillus_Fam_1008_53_1 Nr. of seq. 1 Alignment length(with gaps) = 53
Alignment score = 0.000000
GTCAATGAAAGCCTCTTCCATTTACCATGGAGGCCCNATTATTTCTCTTTTCG
>Bacillus_Fam_1009_53_1 Nr. of seq. 1 Alignment length(with gaps) = 53
Alignment score = 0.000000
TATTGGAGCCCATTCTGTTTGAAAACTTGGGCAAAAGGTATCCAATCGATCCG
>Bacillus_Fam_1010_53_1 Nr. of seq. 1 Alignment length(with gaps) = 53
Alignment score = 0.000000
TTTTTACCGCCTGACCCCCTTCTCAGCACTCCGCGGGCAATAGAACCATCTTC
>Bacillus_Fam_1011_53_1 Nr. of seq. 1 Alignment length(with gaps) = 53
Alignment score = 0.000000
TATTGCCCGATTGNGGTGTCCAAGGGGTGCAAACGGTAAAAAGAACAGGCTTC
>Bacillus_Fam_1012_53_1 Nr. of seq. 1 Alignment length(with gaps) = 53
Alignment score = 0.000000
TGTCCATCATCCGTTAGATGAAGGCCATTTTTAGCAGGCAAATCATGGAGTAA
>Bacillus_Fam_1013_53_1 Nr. of seq. 1 Alignment length(with gaps) = 53
Alignment score = 0.000000
TTCTGTCCCGAATCCGTTTCATACGGGACACTAGGGCATAAAAGTGCAGACGC
>Bacillus_Fam_1014_53_1 Nr. of seq. 1 Alignment length(with gaps) = 53
Alignment score = 0.000000
GTCCTCATCCCTACCATGATAACAATTCCGAACCCTACTTTCTCTCCAAACAT
>Bacillus_Fam_1015_53_1 Nr. of seq. 1 Alignment length(with gaps) = 53
Alignment score = 0.000000
TTGCGGGTTCGCACTTGGAACTTTCTAGGAGATACCGAACCCGATTCCACCAC
>Bacillus_Fam_1016_53_1 Nr. of seq. 1 Alignment length(with gaps) = 53
Alignment score = 0.000000
AGAAAATAGGTAACAGCGAACCAGTAAAACCATTATAGTGGTATGCAAATGGA
>Bacillus_Fam_1017_52_1 Nr. of seq. 1 Alignment length(with gaps) = 52
Alignment score = 0.000000
TAATCGTGTAGATACTGTGACCATTGTGGGGAGTAACTGACATGAAAAGGGC
>Bacillus_Fam_1018_52_1 Nr. of seq. 1 Alignment length(with gaps) = 52
Alignment score = 0.000000
TAGCAACATATCGAGTGAAAAGGGCAGAGTTGTTGTAGGAAAAGTCGGGAAG

>Bacillus_Fam_1019_52_1 Nr. of seq. 1 Alignment length(with gaps) = 52
Alignment score = 0.000000
GCTTTTGTCCATTTAGAACAGCTAACGGGACAATTCCACCGGAGCTGCCAGC
>Bacillus_Fam_1020_52_1 Nr. of seq. 1 Alignment length(with gaps) = 52
Alignment score = 0.000000
TGAAGGACATTTTAGGGTTGTGGCGNCTTTGTTTTGTCCTTCATNGGGCGGA
>Bacillus_Fam_1021_52_1 Nr. of seq. 1 Alignment length(with gaps) = 52
Alignment score = 0.000000
AAAAGCCAAAAGGCACCTGGNNANCGTCCAAACTGTCCTTTAGAACGTCTCA
>Bacillus_Fam_1022_52_1 Nr. of seq. 1 Alignment length(with gaps) = 52
Alignment score = 0.000000
GCTCTTCTCTTCTTCCTGCTGCCTTTGAGACACGCGCTACCCCTAGCGCGGC
>Bacillus_Fam_1023_52_1 Nr. of seq. 1 Alignment length(with gaps) = 52
Alignment score = 0.000000
GGCGCGGTGCAGAAAGTAGGTTTGGGCAGGGGTGAGAGCACCGCGCGGGGGT
>Bacillus_Fam_1024_52_1 Nr. of seq. 1 Alignment length(with gaps) = 52
Alignment score = 0.000000
TTTTGGGCACCGCGCGGACGCGGCGCGGCGCTCTTCTCACGCTGCAACTCAC
>Bacillus_Fam_1025_52_1 Nr. of seq. 1 Alignment length(with gaps) = 52
Alignment score = 0.000000
GAGATGTCTTCATCCATGCGATGAGGACCTGTCTACGCACAAATAGGAACGA
>Bacillus_Fam_1026_52_1 Nr. of seq. 1 Alignment length(with gaps) = 52
Alignment score = 0.000000
AGGTCTTCATACACCTTATGAAGACACTTCCAAGCCTGAAATCCTTCCACTA
>Bacillus_Fam_1027_52_1 Nr. of seq. 1 Alignment length(with gaps) = 52
Alignment score = 0.000000
GACATCTCTCCACCGGAAATCTTCTCTCACATGTCTTCATTCCGCTTATGAG
>Bacillus_Fam_1028_52_1 Nr. of seq. 1 Alignment length(with gaps) = 52
Alignment score = 0.000000
GATGAAGACATTCCCATGATGATTTCCTTTTCCAAAGTGTCTTCATCATGCG
>Bacillus_Fam_1029_52_1 Nr. of seq. 1 Alignment length(with gaps) = 52
Alignment score = 0.000000
CAACATTGTCCTCGTAGGTAGCGTACGACTCCCTGAGAAGGTGAAAACCCGC
>Bacillus_Fam_1030_52_1 Nr. of seq. 1 Alignment length(with gaps) = 52
Alignment score = 0.000000
AACGAGGACAATGTTGACGTGATTTCGGGGTTACCGGGTTTCATAGGAGTTC
>Bacillus_Fam_1031_52_1 Nr. of seq. 1 Alignment length(with gaps) = 52
Alignment score = 0.000000
TGTTTGGTAAGATAGAGGGGTTCTATACGCCCGAAGCAGCATGGTAAAAGGC
>Bacillus_Fam_1032_52_1 Nr. of seq. 1 Alignment length(with gaps) = 52
Alignment score = 0.000000
GCTTCTATACTACCGAAGACCTCGGGATTCCTACCGTTCGGGCGTATAGAAC
>Bacillus_Fam_1033_52_1 Nr. of seq. 1 Alignment length(with gaps) = 52
Alignment score = 0.000000
AGGTAGTCATACGCTTTGAACGAGGACAATGTTTTACACTTTTCAGTGGGGA
>Bacillus_Fam_1034_52_1 Nr. of seq. 1 Alignment length(with gaps) = 52
Alignment score = 0.000000
CTATACGACCGAAGCTACTTATCAAGCTCCCCTTCCGTCTTATAGAACCCCT
>Bacillus_Fam_1035_52_1 Nr. of seq. 1 Alignment length(with gaps) = 52
Alignment score = 0.000000
GACCGAAGCAACCCCTATTCACCTCGAAACGTCGTATAGAACGCTTCTATAC
>Bacillus_Fam_1036_52_1 Nr. of seq. 1 Alignment length(with gaps) = 52
Alignment score = 0.000000
CCCGTTAGAGCAGGATTCAATAACTAAGTGTCGATTAAACAGGGTTTAAAAT

>Bacillus_Fam_1037_52_1 Nr. of seq. 1 Alignment length(with gaps) = 52
Alignment score = 0.000000
GTGTCATAGCACTCGTATGACGCATCTTATATTGAATCTCTAGCTATGTNGT
>Bacillus_Fam_1038_52_1 Nr. of seq. 1 Alignment length(with gaps) = 52
Alignment score = 0.000000
TAGTCTGAGTGGATATAAAACACGAAATAAAATCTCGTTGTGATGGTGAGTC
>Bacillus_Fam_1039_52_1 Nr. of seq. 1 Alignment length(with gaps) = 52
Alignment score = 0.000000
CCCGTTAGAATGATTATCCCGTGCTGAAGGTAGATAAAACAGCGTTTAAAAG
>Bacillus_Fam_1040_52_1 Nr. of seq. 1 Alignment length(with gaps) = 52
Alignment score = 0.000000
TGCGAGTGGACTTTAAAGTGCCTATAAAATCCCATGAGGTAGAGGGAGCATG
>Bacillus_Fam_1041_52_1 Nr. of seq. 1 Alignment length(with gaps) = 52
Alignment score = 0.000000
TTTTCCGTTCAGTGGATCCTCTCTTCCTTGAACGGAATTCTTTGTCATTTTC
>Bacillus_Fam_1042_52_1 Nr. of seq. 1 Alignment length(with gaps) = 52
Alignment score = 0.000000
TTGCTTTCCTTTCTAAGGCACTCAGCTTCCTGGAAAGGAATACTAAGCCACT
>Bacillus_Fam_1043_52_1 Nr. of seq. 1 Alignment length(with gaps) = 52
Alignment score = 0.000000
AATGGGTTGCTATTTTGTTTTGCGTGCCTTTTGCGCGTTGTTTTTCTGCTCG
>Bacillus_Fam_1044_52_1 Nr. of seq. 1 Alignment length(with gaps) = 52
Alignment score = 0.000000
AAGGAATGGGGCGCTATTCTTATTTGCTGGCCTTTACCCTCTGATTTTTGTA
>Bacillus_Fam_1045_52_1 Nr. of seq. 1 Alignment length(with gaps) = 52
Alignment score = 0.000000
CTTTCTCGGATAGTTTTCCTTTCAGAGACTTTTTCCTTCTTTGAACGGAACG
>Bacillus_Fam_1046_52_1 Nr. of seq. 1 Alignment length(with gaps) = 52
Alignment score = 0.000000
TTCCTTTCTACTAGTAAAATCAGCCATGAAAGGAATACTAAACGCCTTCACT
>Bacillus_Fam_1047_52_1 Nr. of seq. 1 Alignment length(with gaps) = 52
Alignment score = 0.000000
TAAAGGAATACTAAGCACTCTGGCTTTCCTTTCTACGTCGATTCCCTCGTCC
>Bacillus_Fam_1048_52_1 Nr. of seq. 1 Alignment length(with gaps) = 52
Alignment score = 0.000000
GTTGTCGATTAGGATTATGAAAAAATCGATGGTGCCAAAAGGTGCGAATGGG
>Bacillus_Fam_1049_52_1 Nr. of seq. 1 Alignment length(with gaps) = 52
Alignment score = 0.000000
CCCCGTCAAAGAGAGGGTATGAGGGTGTCGAATTGAATTTTGGTTGAATCGG
>Bacillus_Fam_1050_52_1 Nr. of seq. 1 Alignment length(with gaps) = 52
Alignment score = 0.000000
GAAACGTACCAATATAGAGTCAGAATCCTGCTGTAGTGGTAAGATTCGAGAA
>Bacillus_Fam_1051_52_1 Nr. of seq. 1 Alignment length(with gaps) = 52
Alignment score = 0.000000
AGGAATGATGTAGGAATGCAGGGGTAATTAGAGCATCAAATCGGAGAAGTGA
>Bacillus_Fam_1052_52_1 Nr. of seq. 1 Alignment length(with gaps) = 52
Alignment score = 0.000000
GGGTGTCTCATGAACGCTTCATGGGGCCATTTTGCCTGCATATTTGAAGTTA
>Bacillus_Fam_1053_52_1 Nr. of seq. 1 Alignment length(with gaps) = 52
Alignment score = 0.000000
GGTGGTCGAATCCCAAAAAAAGAGAATCGGCCCCGTCAAAGTGCTGCTTCGA
>Bacillus_Fam_1054_52_1 Nr. of seq. 1 Alignment length(with gaps) = 52
Alignment score = 0.000000
GGTGGAATTTAGTTTTGGAGAAATCGGCATGGTCAAAGCGGGCGAAAGACAG

>Bacillus_Fam_1055_52_1 Nr. of seq. 1 Alignment length(with gaps) = 52
Alignment score = 0.000000
CTCAACCAGAGTCCGAAGTTGTACTAGGTTCGGACTCAAATTNCAGGATGAG
>Bacillus_Fam_1056_52_1 Nr. of seq. 1 Alignment length(with gaps) = 52
Alignment score = 0.000000
ATGAAGCCGAAAAGTCGTGNAGCGATAAGAAAACGGTCGCCATGAGGCAGTC
>Bacillus_Fam_1057_52_1 Nr. of seq. 1 Alignment length(with gaps) = 52
Alignment score = 0.000000
GACGAAAATTCAGTGGCTCACCAANAAATGGTCGACATAGAGCCTTCATGAA
>Bacillus_Fam_1058_52_1 Nr. of seq. 1 Alignment length(with gaps) = 52
Alignment score = 0.000000
TCACAGACAATTCATTCAACTTTTTACTNCGNTTTGTCTTCGAGACTGGCTA
>Bacillus_Fam_1059_52_1 Nr. of seq. 1 Alignment length(with gaps) = 52
Alignment score = 0.000000
TATGAAGGACTTTCTCACCGGCCAGGCTGCTCGTTCGAGGCTTCATCGGCCT
>Bacillus_Fam_1060_52_1 Nr. of seq. 1 Alignment length(with gaps) = 52
Alignment score = 0.000000
TCAGTTTCTCAGACCNAATCCCCTTCATAGACCACTTGAAGAGGATACACCT
>Bacillus_Fam_1061_52_1 Nr. of seq. 1 Alignment length(with gaps) = 52
Alignment score = 0.000000
CCTCGGCATACTCCGGTTTGTTTTGTCTCCGAGAGCAGCTATCAAACACTTT
>Bacillus_Fam_1062_52_1 Nr. of seq. 1 Alignment length(with gaps) = 52
Alignment score = 0.000000
TTCATTGCCTCGATGAAGAGCATAACGCAACAGAACTATGCTCGAATTCCTC
>Bacillus_Fam_1063_52_1 Nr. of seq. 1 Alignment length(with gaps) = 52
Alignment score = 0.000000
TTGGACATTCTGACTACCTCCACTTTCTCCTTTGTCCTTTAAACTTGGGATA
>Bacillus_Fam_1064_52_1 Nr. of seq. 1 Alignment length(with gaps) = 52
Alignment score = 0.000000
TACATGTTTTGGCTGGTTGCTGACCATTATTTGGGTGCCATACTCATTTGGG
>Bacillus_Fam_1065_52_1 Nr. of seq. 1 Alignment length(with gaps) = 52
Alignment score = 0.000000
TAATTCGTAATGTAATTTCCCACTATGNACTAATTACGGTGCGTATAACCCC
>Bacillus_Fam_1066_52_1 Nr. of seq. 1 Alignment length(with gaps) = 52
Alignment score = 0.000000
GTTCGGACAGGAGAAGAGGAAAAACCGTGGATTGAGTCCGAAGTCGCCCTAT
>Bacillus_Fam_1067_52_1 Nr. of seq. 1 Alignment length(with gaps) = 52
Alignment score = 0.000000
TTCGGACAGACTCTGGACTTTTTCTGCCTTGCCTGTCCGAACTTGCCCCAGG
>Bacillus_Fam_1068_52_1 Nr. of seq. 1 Alignment length(with gaps) = 52
Alignment score = 0.000000
TTTATCCTCACCAAACCGAGCCTAGATGAGGTTTGATAGTAAATAACTCGTG
>Bacillus_Fam_1069_52_1 Nr. of seq. 1 Alignment length(with gaps) = 52
Alignment score = 0.000000
TTTGGTGAGGATAAAAGCAGGTTATCTACTACCAAAAGTAAGTAGAGTGGAA
>Bacillus_Fam_1070_52_1 Nr. of seq. 1 Alignment length(with gaps) = 52
Alignment score = 0.000000
AAAATCTAGCGGACTGTCCTTAATAGGCGCTGTTGAGGACAAAACGGGGTTG
>Bacillus_Fam_1071_52_1 Nr. of seq. 1 Alignment length(with gaps) = 52
Alignment score = 0.000000
TCTGTCCGAATGCACCTCTTATTCGGACTCCTTCACCCTCTCTACTACCTTT
>Bacillus_Fam_1072_52_1 Nr. of seq. 1 Alignment length(with gaps) = 52
Alignment score = 0.000000
ATTCGGACAAGCCTATGGCTTCTTGCTTCCTACTTGTCCGAATATCCTTCCG

>Bacillus_Fam_1073_52_1 Nr. of seq. 1 Alignment length(with gaps) = 52
Alignment score = 0.000000
TGTCCGAACTAGGCTTAAGTTCGGACAGAAATGACCGCTTTTTCTCAAATGG
>Bacillus_Fam_1074_52_1 Nr. of seq. 1 Alignment length(with gaps) = 52
Alignment score = 0.000000
ATTGCGTTGCTCTCGATCAAGAAATGGACTTCATAGCCGAGGTAACGATCGT
>Bacillus_Fam_1075_52_1 Nr. of seq. 1 Alignment length(with gaps) = 52
Alignment score = 0.000000
CGTTCATAAGGCGGATGAAAGCCATTGTAAAGAGAAAAAACAGGTGAGAAGG
>Bacillus_Fam_1076_52_1 Nr. of seq. 1 Alignment length(with gaps) = 52
Alignment score = 0.000000
AATGGCGTTCATCAGCCGTATGAACGCTATCTCTACCGGGGTTGATCCTTGA
>Bacillus_Fam_1077_52_1 Nr. of seq. 1 Alignment length(with gaps) = 52
Alignment score = 0.000000
CGGGCTTTCATATCCCCAATGAACGACCGTTTCCCCGGGCACTTGCCTGATT
>Bacillus_Fam_1078_52_1 Nr. of seq. 1 Alignment length(with gaps) = 52
Alignment score = 0.000000
GCGAAATGAAGTTCATCAGGCCTATGAAAGCAATCGCGGCCGAAATTTTAGA
>Bacillus_Fam_1079_52_1 Nr. of seq. 1 Alignment length(with gaps) = 52
Alignment score = 0.000000
GGGCTTTCATTCGACCGATGATCGACCGCGCGGATGGTTTTTTCTCTCGCGT
>Bacillus_Fam_1080_52_1 Nr. of seq. 1 Alignment length(with gaps) = 52
Alignment score = 0.000000
ATGAACGCGATCACGAATGAAAAAGGGGGTTACGATGGTGTTCATAAGNCCG
>Bacillus_Fam_1081_52_1 Nr. of seq. 1 Alignment length(with gaps) = 52
Alignment score = 0.000000
ATAGCGTTCATCCCGGGTATGAACTTCATCTTGANTATCTTTTTTACTTGNA
>Bacillus_Fam_1082_52_1 Nr. of seq. 1 Alignment length(with gaps) = 52
Alignment score = 0.000000
GCTTATGAACTGCATTTGGCATGGATTTTTTTACCGAGATAGCGTTCATTCC
>Bacillus_Fam_1083_52_1 Nr. of seq. 1 Alignment length(with gaps) = 52
Alignment score = 0.000000
CGGGCACTTTTAGCCTCTATTAGTGACCGGTTCTCTCGACAAACTCTATTTG
>Bacillus_Fam_1084_52_1 Nr. of seq. 1 Alignment length(with gaps) = 52
Alignment score = 0.000000
AATCGGTAACTAATAGGCAGGAAAGATGCCCGAATCCGNTGTGTCGTCCCGC
>Bacillus_Fam_1085_52_1 Nr. of seq. 1 Alignment length(with gaps) = 52
Alignment score = 0.000000
CGTATATCCCAAAAAGCGGTCACCAATCGTATGGATTAGTGCCCGTTCTGAG
>Bacillus_Fam_1086_52_1 Nr. of seq. 1 Alignment length(with gaps) = 52
Alignment score = 0.000000
AAAAGCGGTCACGATTAGTGAGGAAAGATGCCCGTTTTAGTCGGTTTTCAGT
>Bacillus_Fam_1087_52_1 Nr. of seq. 1 Alignment length(with gaps) = 52
Alignment score = 0.000000
TATTTGTGACCGGATTTCGTATTTCTCCCTTCAAACGGGCATCTTCCACTAC
>Bacillus_Fam_1088_52_1 Nr. of seq. 1 Alignment length(with gaps) = 52
Alignment score = 0.000000
TCACTAAAAAGTGCTAAAAGTGCCCGTAATGTGGTGAATCACGAAAATCCGG
>Bacillus_Fam_1089_52_1 Nr. of seq. 1 Alignment length(with gaps) = 52
Alignment score = 0.000000
ACCGCTTTTTACCAAAATCTCTCGTAACGGGCACCTTTCTCCAACATTAGTG
>Bacillus_Fam_1090_52_1 Nr. of seq. 1 Alignment length(with gaps) = 52
Alignment score = 0.000000
ACCAATAAAGGTTAATAGTGCCCGAAAAGGAGAAAAAAGTGCTCTAATGGTC

>Bacillus_Fam_1091_52_1 Nr. of seq. 1 Alignment length(with gaps) = 52
Alignment score = 0.000000
GATTAGTGACCGNTTTTCGGATTAACGCAACTTTTCGGGCATCTTTTACTCC
>Bacillus_Fam_1092_52_1 Nr. of seq. 1 Alignment length(with gaps) = 52
Alignment score = 0.000000
GTTCATCTTGTTTATGAACGATAGTTTTGCCCTCTTCTTTTGAAAAAGTGGC
>Bacillus_Fam_1093_52_1 Nr. of seq. 1 Alignment length(with gaps) = 52
Alignment score = 0.000000
TGGCTTTCATTCCCTTGATGAACGCCACTTTCCTCCAAGATTACCTTAAAAC
>Bacillus_Fam_1094_52_1 Nr. of seq. 1 Alignment length(with gaps) = 52
Alignment score = 0.000000
TGTCCGAAGTGGGTCTGTGTTCGGACAACTTCCGGATGAATCCAAGCCAACT
>Bacillus_Fam_1095_52_1 Nr. of seq. 1 Alignment length(with gaps) = 52
Alignment score = 0.000000
TCTCTGAACCATTTNCCAGCGGAGCAGCCTCNGTTTTGGTCTTGAGACGCCT
>Bacillus_Fam_1096_52_1 Nr. of seq. 1 Alignment length(with gaps) = 52
Alignment score = 0.000000
TCTCATGACCAAAACTTCTGCCGCTTCCCCTCTTTATGGTTCTGAGACGCAC
>Bacillus_Fam_1097_52_1 Nr. of seq. 1 Alignment length(with gaps) = 52
Alignment score = 0.000000
CCTCTCAAGACCAAAATTCCTTTAAGCAAGCGGAGAAGTGGTTCAGAGAGGG
>Bacillus_Fam_1098_52_1 Nr. of seq. 1 Alignment length(with gaps) = 52
Alignment score = 0.000000
GGATGACGAAGATAGGTCAATGAGAAGGGTTCTAATGGACACTACGCTGAAN
>Bacillus_Fam_1099_52_1 Nr. of seq. 1 Alignment length(with gaps) = 52
Alignment score = 0.000000
TCAGGACCAAAAACAAAGACAGAGCTGGTGAAAGTGGTTCAGAGGGGGCGTC
>Bacillus_Fam_1100_52_1 Nr. of seq. 1 Alignment length(with gaps) = 52
Alignment score = 0.000000
GGGTCTTAGTTTCTCTGACTAGCACCCTCTGGTTTGGTTTTTGTGATTCAGA
>Bacillus_Fam_1101_52_1 Nr. of seq. 1 Alignment length(with gaps) = 52
Alignment score = 0.000000
CGATAAGCTACCCAACTGGTTCTTTCTTGATGGATTCGGTCGCTTATAANCC
>Bacillus_Fam_1102_52_1 Nr. of seq. 1 Alignment length(with gaps) = 52
Alignment score = 0.000000
GGGCACTCTGGCTACTCCACAGTGCCCGGTTGATGCTTTTTTCTCCTCACAT
>Bacillus_Fam_1103_52_1 Nr. of seq. 1 Alignment length(with gaps) = 52
Alignment score = 0.000000
CGGTCATTAAGCGGGGCTCTAAATGCCCGCGGAGAGCGAAAAAGGAAGGGAG
>Bacillus_Fam_1104_52_1 Nr. of seq. 1 Alignment length(with gaps) = 52
Alignment score = 0.000000
TCTTAGTTACCGCGAGACCCGCATTTTCTCCTTCGCGGGCATTTAGAGCCCT
>Bacillus_Fam_1105_52_1 Nr. of seq. 1 Alignment length(with gaps) = 52
Alignment score = 0.000000
TTATAGCCTTTATAAATGACCGACCGACCGGAAAAANCGCCAGCTCGGGCAT
>Bacillus_Fam_1106_52_1 Nr. of seq. 1 Alignment length(with gaps) = 52
Alignment score = 0.000000
AACCAAGTAAGTGTTCTATACCGTCAATAGCAACACTTTCCCTCATCAATTG
>Bacillus_Fam_1107_52_1 Nr. of seq. 1 Alignment length(with gaps) = 52
Alignment score = 0.000000
TCGTTTAAGAGCACTTTGGTCTTGCTGTGAATCGGAGTTCTTGTTGCAAACA
>Bacillus_Fam_1108_52_1 Nr. of seq. 1 Alignment length(with gaps) = 52
Alignment score = 0.000000
TATTCAGCCGCAACCAATTTATAGGGTTGCATTTCGCCAGTATGGTACCCTC

>Bacillus_Fam_1109_52_1 Nr. of seq. 1 Alignment length(with gaps) = 52
Alignment score = 0.000000
TAGAACACTTTCTGCGGTCGGGATNCCCNACTTAATGTTGCTTACTCCGGTT
>Bacillus_Fam_1110_52_1 Nr. of seq. 1 Alignment length(with gaps) = 52
Alignment score = 0.000000
CAAAATGTTGCTAAAGCCAGGTTAGAACACTTTCNGGGCTTTCTGGCCTCTG
>Bacillus_Fam_1111_52_1 Nr. of seq. 1 Alignment length(with gaps) = 52
Alignment score = 0.000000
CTGAAGGGCACATTCCTGCCTGGTTTTCGACCTTCAGCTTACTTCCTTTTCC
>Bacillus_Fam_1112_52_1 Nr. of seq. 1 Alignment length(with gaps) = 52
Alignment score = 0.000000
ACCCTTCACACAATCCCACTCCTGATAAACGGTCATATCTTATCCACTTATA
>Bacillus_Fam_1113_52_1 Nr. of seq. 1 Alignment length(with gaps) = 52
Alignment score = 0.000000
ACCTTCAGCTTCTTCTCCACCAACTGAAGGGCACGATCCATCCGCTTTTGTG
>Bacillus_Fam_1114_52_1 Nr. of seq. 1 Alignment length(with gaps) = 52
Alignment score = 0.000000
TAAGTGCGCTTATCTCGGTTTTAGAACACTTTCACAGATANTGCNCTTTGGC
>Bacillus_Fam_1115_52_1 Nr. of seq. 1 Alignment length(with gaps) = 52
Alignment score = 0.000000
GCTGCGGCGGAAAGGCATGAAGGGTCATAAGAGGCTTGGATTGTTACCTTCA
>Bacillus_Fam_1116_52_1 Nr. of seq. 1 Alignment length(with gaps) = 52
Alignment score = 0.000000
TTTCCTCCAGGCTCACGCTCCACTTTGTGCTCTTAACCCCGCGGAAGCGCAC
>Bacillus_Fam_1117_52_1 Nr. of seq. 1 Alignment length(with gaps) = 52
Alignment score = 0.000000
GAAATATAGGGCAGCAAAACATGAAAATGCAACCCTATAAAATGCGCCATCT
>Bacillus_Fam_1118_52_1 Nr. of seq. 1 Alignment length(with gaps) = 52
Alignment score = 0.000000
CTGTATTTTGGACAAGCNGATTTATAGGGCAGCATTTCACCTTTTTGGTGCC
>Bacillus_Fam_1119_52_1 Nr. of seq. 1 Alignment length(with gaps) = 52
Alignment score = 0.000000
CATAAGTGCTGCAAAGCTGGTTTAACAACATAAACTCAGCTGCAGGAAGCCG
>Bacillus_Fam_1120_52_1 Nr. of seq. 1 Alignment length(with gaps) = 52
Alignment score = 0.000000
TTCTTATCGGACGAATNGGTGCCATCAAANCTGATTCTTCTCTGCTTGAGGG
>Bacillus_Fam_1121_52_1 Nr. of seq. 1 Alignment length(with gaps) = 52
Alignment score = 0.000000
GAACCCTCAAGCTTTGGATTCTGTGGATTGATGGCACTTATAGCAGAGTTTA
>Bacillus_Fam_1122_52_1 Nr. of seq. 1 Alignment length(with gaps) = 52
Alignment score = 0.000000
AAGTGTTCTTATCCTGCTTTTACAGCACTTATCTTCGCTCCGGAGTCACTCA
>Bacillus_Fam_1123_52_1 Nr. of seq. 1 Alignment length(with gaps) = 52
Alignment score = 0.000000
AGCGGGTAAAATAAAGCGCCGTTTTTTACCGGTGATAGCCTTTTTTCGCTCC
>Bacillus_Fam_1124_52_1 Nr. of seq. 1 Alignment length(with gaps) = 52
Alignment score = 0.000000
CGCTTATACCCTTTATTGGTGACCGAACCAGTGTCAAACCCATGGTTCCGGG
>Bacillus_Fam_1125_52_1 Nr. of seq. 1 Alignment length(with gaps) = 52
Alignment score = 0.000000
TTATTGGCGACCGCTCACTACTGGAGNTCCTTCGCTCGGGCGCTTATCTCCC
>Bacillus_Fam_1126_52_1 Nr. of seq. 1 Alignment length(with gaps) = 52
Alignment score = 0.000000
GACCTTTTCAACCTTTTTCACCACAATTCGGGCGTCTACAACTCTTCTAGAG

>Bacillus_Fam_1127_52_1 Nr. of seq. 1 Alignment length(with gaps) = 52
Alignment score = 0.000000
AGGGTTCTCAAGGACAAAGTGCACGCAAAAACCGGTGAAACTGTCCTAGACA
>Bacillus_Fam_1128_52_1 Nr. of seq. 1 Alignment length(with gaps) = 52
Alignment score = 0.000000
TCTTAAGGACAAATCTTCTAGTTCTCTTTAGAGATTTGTCCTTCATACCCCT
>Bacillus_Fam_1129_52_1 Nr. of seq. 1 Alignment length(with gaps) = 52
Alignment score = 0.000000
GTAACGATTAGTCGCTAATCGTGCCCTTCTCCTCGATTTTTGGAGTTTGCG
>Bacillus_Fam_1130_52_1 Nr. of seq. 1 Alignment length(with gaps) = 52
Alignment score = 0.000000
TATGAGTGACCGCTTGTCCTTTTCCAGGAGGTTCTTGGGCTCTCATGAACCT
>Bacillus_Fam_1131_52_1 Nr. of seq. 1 Alignment length(with gaps) = 52
Alignment score = 0.000000
TGTGACCAAAACTCATGGATGAAACTATCAAAATGGTCACAAGAAGGGTCGC
>Bacillus_Fam_1132_52_1 Nr. of seq. 1 Alignment length(with gaps) = 52
Alignment score = 0.000000
CTTGCTCTTTTTCGGTAGGCAATACATCTTGTTGTCTACTCTTATTGCGGCT
>Bacillus_Fam_1133_52_1 Nr. of seq. 1 Alignment length(with gaps) = 52
Alignment score = 0.000000
CTACCCAAATACGGGGGACCTCTCAAGTTCGGGCGCCATCACATCCCTTTGA
>Bacillus_Fam_1134_52_1 Nr. of seq. 1 Alignment length(with gaps) = 52
Alignment score = 0.000000
CTCTTTTCGGTAGCCATTCCACCCCTTTGACTCCCTTCCTCACNCCTCCTCA
>Bacillus_Fam_1135_52_1 Nr. of seq. 1 Alignment length(with gaps) = 52
Alignment score = 0.000000
TTCGCTTCTCTTCGGGAGTCAACACCCTCCTTTAGCTACCGTTCTCTTTGTC
>Bacillus_Fam_1136_52_1 Nr. of seq. 1 Alignment length(with gaps) = 52
Alignment score = 0.000000
ACATTCGGGCTCCAGATCCCTCTTCTGCTTCCCTACTTTTCCCATTCCCACC
>Bacillus_Fam_1137_52_1 Nr. of seq. 1 Alignment length(with gaps) = 52
Alignment score = 0.000000
ACGGGAGCCATTCCCTTCCTTTGACTACCCAAATCCNGTTCGGTCCTTCTGC
>Bacillus_Fam_1138_52_1 Nr. of seq. 1 Alignment length(with gaps) = 52
Alignment score = 0.000000
TCGTTGACTACCCAAATTTGTGATNGACTCAGAGTTCGAGTGCCANTGNGCT
>Bacillus_Fam_1139_52_1 Nr. of seq. 1 Alignment length(with gaps) = 52
Alignment score = 0.000000
AGTCAATACACCTTATTGGCTACCGTCCACTCCTCCTCCCTCGCTTTTCGGG
>Bacillus_Fam_1140_52_1 Nr. of seq. 1 Alignment length(with gaps) = 52
Alignment score = 0.000000
TTTCGGTAGTCAACCCTTCTCTTTGTCTACCAAAATCCTCGCCCTCTCCCTT
>Bacillus_Fam_1141_52_1 Nr. of seq. 1 Alignment length(with gaps) = 52
Alignment score = 0.000000
TAGACATTTTGTAGTCAATTGGCATTCGAAATGTACATTAAACAAGGTTTTT
>Bacillus_Fam_1142_52_1 Nr. of seq. 1 Alignment length(with gaps) = 52
Alignment score = 0.000000
TCATTTGGGTAAGAGGAAAGCCCCTGCATTGCCCGAAAAAGCGCCAACTCCA
>Bacillus_Fam_1143_52_1 Nr. of seq. 1 Alignment length(with gaps) = 52
Alignment score = 0.000000
GATGACGGACACTTTCCCAGCGTTTTGGGCTATTTCTGTCCGTCATAGGCTG
>Bacillus_Fam_1144_52_1 Nr. of seq. 1 Alignment length(with gaps) = 52
Alignment score = 0.000000
AAAAAGGGTAAAAGGAAAGGCTCTGCATTACCCGAAAATGCTGGAGAGAAGT

>Bacillus_Fam_1145_52_1 Nr. of seq. 1 Alignment length(with gaps) = 52
Alignment score = 0.000000
TGTCCATTAAATGCACTTTCTTAGACATTTCGGTTTGGAACTCATGCTGAAA
>Bacillus_Fam_1146_52_1 Nr. of seq. 1 Alignment length(with gaps) = 52
Alignment score = 0.000000
TTTTGTCCGAACCTGCCCCTACTTCAGACAATTTCCGCTTGTACGGCCGAGA
>Bacillus_Fam_1147_52_1 Nr. of seq. 1 Alignment length(with gaps) = 52
Alignment score = 0.000000
TTAATAGACATTTTGACGTCCGTTACTCTTCGAAATGTCTAAGAAGTGCTGT
>Bacillus_Fam_1148_52_1 Nr. of seq. 1 Alignment length(with gaps) = 52
Alignment score = 0.000000
AAACAGGTCACCAATACCCNTGATTGGTGACCTAAAAATCCATATTTCCGNA
>Bacillus_Fam_1149_52_1 Nr. of seq. 1 Alignment length(with gaps) = 52
Alignment score = 0.000000
GTTCGGACATTTCAACGGCCTCTTCATTGAGTTTTGTCCGAAGTAGGATCAA
>Bacillus_Fam_1150_52_1 Nr. of seq. 1 Alignment length(with gaps) = 52
Alignment score = 0.000000
GAAAAGCTGTCAAGATTATCCCGGAATTATGACAGGCTCAAGCCCATAAAGC
>Bacillus_Fam_1151_52_1 Nr. of seq. 1 Alignment length(with gaps) = 52
Alignment score = 0.000000
GTTCGGACAAATCATCGGGAGTGACGGTACTTTTTGTCCGAAGGTGACCCAT
>Bacillus_Fam_1152_52_1 Nr. of seq. 1 Alignment length(with gaps) = 52
Alignment score = 0.000000
TCCCTCATCCGGGCACATATTTTCCCTTTATGTGCCCCGTCTCCCTTCATCT
>Bacillus_Fam_1153_52_1 Nr. of seq. 1 Alignment length(with gaps) = 52
Alignment score = 0.000000
TATGAAGGACATTTTGGGGGCGNGGGATTCTCCTTTTGTCTTTCATCCCTGT
>Bacillus_Fam_1154_52_1 Nr. of seq. 1 Alignment length(with gaps) = 52
Alignment score = 0.000000
TTATTTTGACAACTTTCAGGTGAAAAGGGCAAAAGTTGTCAAGAAAGAGAGG
>Bacillus_Fam_1155_52_1 Nr. of seq. 1 Alignment length(with gaps) = 52
Alignment score = 0.000000
TGATAGGCGCCCGTGGCGGTTGAAAATAGGAGTTGGCGGTCGCCAATAGGGT
>Bacillus_Fam_1156_52_1 Nr. of seq. 1 Alignment length(with gaps) = 52
Alignment score = 0.000000
GGGCACTGAGAACACCTGTCAGCACCCAAAATCCCTGGCCCCTCCTAAAAAT
>Bacillus_Fam_1157_52_1 Nr. of seq. 1 Alignment length(with gaps) = 52
Alignment score = 0.000000
TGTCAGTGCCCCAAAAGTGGAGCAGNTCGTNAAAAAGGTTACTGAGGGTCGN
>Bacillus_Fam_1158_52_1 Nr. of seq. 1 Alignment length(with gaps) = 52
Alignment score = 0.000000
CCTCTTTTTCGGTAAACTCTCGGAAAAAGGTACCTGACAGGCTGCCTCAGTA
>Bacillus_Fam_1159_52_1 Nr. of seq. 1 Alignment length(with gaps) = 52
Alignment score = 0.000000
TTGGTCGTCCATACACCCGATGGAGGCCCGTTTTTCAGAATCAGCTTCAGTA
>Bacillus_Fam_1160_52_1 Nr. of seq. 1 Alignment length(with gaps) = 52
Alignment score = 0.000000
GGGCTTCCATCCACTTGATGGAGGACCTTTTTTGCGGCTTTCCTGCGAAAAC
>Bacillus_Fam_1161_52_1 Nr. of seq. 1 Alignment length(with gaps) = 52
Alignment score = 0.000000
TTTTTTGGTGGTTTGCATGAATTTTGGGTACTGAGGAGGAGTNTCAGAGCCC
>Bacillus_Fam_1162_52_1 Nr. of seq. 1 Alignment length(with gaps) = 52
Alignment score = 0.000000
GTCCTTCATCCAGGGTCTGAAGGACATAACAACACTAGTGATCACGTGAATT

>Bacillus_Fam_1163_52_1 Nr. of seq. 1 Alignment length(with gaps) = 52
Alignment score = 0.000000
TAGTCCGAAGGCAGGTCAAGTTCGGACAGAAAGCAGACATCAAAGCGCAAAC
>Bacillus_Fam_1164_52_1 Nr. of seq. 1 Alignment length(with gaps) = 52
Alignment score = 0.000000
TTCGGCCAAATCAGGAGCGNCGAGCCGTTGAAATGTCCGAAGTTGGTGTAGG
>Bacillus_Fam_1165_52_1 Nr. of seq. 1 Alignment length(with gaps) = 52
Alignment score = 0.000000
CATTTTTCCAGGGCTATGGTCTATGACAGACGTTATCATGACCCGTTGGCTG
>Bacillus_Fam_1166_52_1 Nr. of seq. 1 Alignment length(with gaps) = 52
Alignment score = 0.000000
CTCTCATGGACCTTTCGCATGTAAAAGTCGCTGCAATGGGACACGACAACGA
>Bacillus_Fam_1167_52_1 Nr. of seq. 1 Alignment length(with gaps) = 52
Alignment score = 0.000000
GAATCGTTACCGTCATGCGANGCTTTCAAGNCTAACGGGCACGATTCACTTC
>Bacillus_Fam_1168_52_1 Nr. of seq. 1 Alignment length(with gaps) = 52
Alignment score = 0.000000
CCTCTCATGTCCCGTATCATTGAAAAAAGGATGTGCTTGGGACATGAGACCA
>Bacillus_Fam_1169_52_1 Nr. of seq. 1 Alignment length(with gaps) = 52
Alignment score = 0.000000
CTATTCGCCCGAGGAGGAAGAAAAGTGTATCTAGGGGTAGAATAGGACGTCT
>Bacillus_Fam_1170_52_1 Nr. of seq. 1 Alignment length(with gaps) = 52
Alignment score = 0.000000
GGTCGAATAGGGCGTCTCTATTCGCCCGAAGCGCCAGGTCGAGTCGTTCAAA
>Bacillus_Fam_1171_52_1 Nr. of seq. 1 Alignment length(with gaps) = 52
Alignment score = 0.000000
CCTTGAATGGGACGTGATGAGTGTTATCAAAGCCCGATCGCCANCTTTTTTN
>Bacillus_Fam_1172_52_1 Nr. of seq. 1 Alignment length(with gaps) = 52
Alignment score = 0.000000
ACGGTAACGATTGAGGCCGAATCGTTCCCGCACCGTGCTATTTTTGGGAGTT
>Bacillus_Fam_1173_52_1 Nr. of seq. 1 Alignment length(with gaps) = 52
Alignment score = 0.000000
CGATTGAGTTCAGTTTATCCAAAAATTACCCGCTAACGGGAACCAATGAGCT
>Bacillus_Fam_1174_52_1 Nr. of seq. 1 Alignment length(with gaps) = 52
Alignment score = 0.000000
TTCGGACAAGCCCAGGTGATTTTGAGCAATCNTAGTCCGAATAGCCGTCGAG
>Bacillus_Fam_1175_52_1 Nr. of seq. 1 Alignment length(with gaps) = 52
Alignment score = 0.000000
GTCGCCAATAAGGTGTATTGGTACCCGAAACTCTACTATATTTAATAAAACG
>Bacillus_Fam_1176_52_1 Nr. of seq. 1 Alignment length(with gaps) = 52
Alignment score = 0.000000
GACAACCACACTCAAAATTCGGCTGAAGAAGTCCGAAGTGGTAGTGGGTTCA
>Bacillus_Fam_1177_52_1 Nr. of seq. 1 Alignment length(with gaps) = 52
Alignment score = 0.000000
AGGGAACCAATGACTCCTATTGATACCTCAAACAACGCTAATTTGCCAAATA
>Bacillus_Fam_1178_52_1 Nr. of seq. 1 Alignment length(with gaps) = 52
Alignment score = 0.000000
GGAAACAAGCAGCCCTATTGAGTACACTATTCACTCGAATTCTTTAAAAATG
>Bacillus_Fam_1179_52_1 Nr. of seq. 1 Alignment length(with gaps) = 52
Alignment score = 0.000000
TGTCTGAACTTGACTGGTGTTCGGACTCGATTCACCTTAAAATCGCCATTGC
>Bacillus_Fam_1180_52_1 Nr. of seq. 1 Alignment length(with gaps) = 52
Alignment score = 0.000000
CTTTTTTCTCCAGTGTTCGGTCAACAATAACGGCGATTGGATACCCTTCTGT

>Bacillus_Fam_1181_52_1 Nr. of seq. 1 Alignment length(with gaps) = 52
Alignment score = 0.000000
TACTTCAGACTTCTTTTNCGCAATCAAGGGCTAACCTGTCCGAACCTGAGGC
>Bacillus_Fam_1182_52_1 Nr. of seq. 1 Alignment length(with gaps) = 52
Alignment score = 0.000000
GGCTATTGATAACCTTTTTGCTTTTTTTACAGGAAAACTGAACTCAATAGA
>Bacillus_Fam_1183_52_1 Nr. of seq. 1 Alignment length(with gaps) = 52
Alignment score = 0.000000
TCTTGTTCAGACTCGATCTCTCTTATTTTCACCAAACTTGTCCGAACTTGAC
>Bacillus_Fam_1184_52_1 Nr. of seq. 1 Alignment length(with gaps) = 52
Alignment score = 0.000000
CCTAACCTGTCCGAACTCATCCCAAGTTCGGACTTCTTTGCTCTTGAATCCT
>Bacillus_Fam_1185_52_1 Nr. of seq. 1 Alignment length(with gaps) = 52
Alignment score = 0.000000
AGAAAAGCGAACTCAACAAGCGTTATTGGTTCCCGAAAACTAGCAGAAAGGC
>Bacillus_Fam_1186_52_1 Nr. of seq. 1 Alignment length(with gaps) = 52
Alignment score = 0.000000
AGATGTCCTTCATCAATGAAATGAAGGCCAAGNTATAGGGTTTTCTACTGTC
>Bacillus_Fam_1187_52_1 Nr. of seq. 1 Alignment length(with gaps) = 52
Alignment score = 0.000000
TGAATTGTTCCCGTTCATCAACACCATCCACGATTGCGGTCACGATACNTCG
>Bacillus_Fam_1188_52_1 Nr. of seq. 1 Alignment length(with gaps) = 52
Alignment score = 0.000000
AGAGGAAGTATTGTGCCCGGAAAGGGAAAGCTCGAGAAGAAACGGGAACAAA
>Bacillus_Fam_1189_52_1 Nr. of seq. 1 Alignment length(with gaps) = 52
Alignment score = 0.000000
GTATTGTTCCCATAATCCTCTGGGAAAGACAGAAACGGTAACGATTAGTTGA
>Bacillus_Fam_1190_52_1 Nr. of seq. 1 Alignment length(with gaps) = 52
Alignment score = 0.000000
GGGAACAATAGAAGTAGAATCGTGCCCGAAATCAGGAGAGAGCAGTGNCCCC
>Bacillus_Fam_1191_52_1 Nr. of seq. 1 Alignment length(with gaps) = 52
Alignment score = 0.000000
ACAACGGTAACGATCAGAGGTGGAATGTGCCCGTAGCGGAGGAAATCTCGGG
>Bacillus_Fam_1192_52_1 Nr. of seq. 1 Alignment length(with gaps) = 52
Alignment score = 0.000000
TGTCCTTCATGAAGGTAATGAAGTCCAAACTAGGTGTACCAAAATCGAAAAG
>Bacillus_Fam_1193_52_1 Nr. of seq. 1 Alignment length(with gaps) = 52
Alignment score = 0.000000
AGCGATGAAGGCCAAAATGTGCTCGGTAACAAGAGCAGATGACCTTCATAAA
>Bacillus_Fam_1194_52_1 Nr. of seq. 1 Alignment length(with gaps) = 52
Alignment score = 0.000000
ATCGTGCCCAATACCGAGGCNGACATGGAATAACGGGAACAATACCAGGAGA
>Bacillus_Fam_1195_52_1 Nr. of seq. 1 Alignment length(with gaps) = 52
Alignment score = 0.000000
TGAAGGACACTTCCCTTGTTTTCTATCACTATCTTGTCCCTCATAACCCCGA
>Bacillus_Fam_1196_52_1 Nr. of seq. 1 Alignment length(with gaps) = 52
Alignment score = 0.000000
CTTTGGTCTTTATAACCCGCTTAAAGGACAATTCCTTTAAAATATTTGATTC
>Bacillus_Fam_1197_52_1 Nr. of seq. 1 Alignment length(with gaps) = 52
Alignment score = 0.000000
TGTCCTTCATATGCTCCATGAAGGACATGTCCATGGGTATTTTCGGTTGTCT
>Bacillus_Fam_1198_52_1 Nr. of seq. 1 Alignment length(with gaps) = 52
Alignment score = 0.000000
ATTAGAGTAAAAGGTCTTCATCGATGTTATGAAGACCAAACCGGGTTCAGAG

>Bacillus_Fam_1199_52_1 Nr. of seq. 1 Alignment length(with gaps) = 52
Alignment score = 0.000000
TGATTGCGACACGGATTTCGGNAAAATTCAAAGATGAGTCCGAATAAGAGTG
>Bacillus_Fam_1200_52_1 Nr. of seq. 1 Alignment length(with gaps) = 52
Alignment score = 0.000000
CTTGTGTTGTCCGAATCAAGGCGTCATTCGGACAGCTACGCCAGATTTCCTG
>Bacillus_Fam_1201_52_1 Nr. of seq. 1 Alignment length(with gaps) = 52
Alignment score = 0.000000
ATGATTCGGACACAGATCAGGAAAAACCGCACCCTCGAGTCCGAATGAAGCG
>Bacillus_Fam_1202_52_1 Nr. of seq. 1 Alignment length(with gaps) = 52
Alignment score = 0.000000
AGTCCGAATGCAAGGCTGATTCGGACAGTTGGGTCAGGGGAATNCAGAAGCG
>Bacillus_Fam_1203_52_1 Nr. of seq. 1 Alignment length(with gaps) = 52
Alignment score = 0.000000
CTGTCAGAATCAACCCTTCATTCGGACTCAACTACTCGTATTCCTTACTTCT
>Bacillus_Fam_1204_52_1 Nr. of seq. 1 Alignment length(with gaps) = 52
Alignment score = 0.000000
AGGTCTTCATGAGGTTGATGAAGACCTATTTGCAACTAGTATTCTCTCGGAA
>Bacillus_Fam_1205_52_1 Nr. of seq. 1 Alignment length(with gaps) = 52
Alignment score = 0.000000
GAATCGTACCCGATTCGGGGCAATTGCAAGGAAAAGGGTAACGCAAACGTGG
>Bacillus_Fam_1206_52_1 Nr. of seq. 1 Alignment length(with gaps) = 52
Alignment score = 0.000000
CTGTCCGAATGAGGCATGAATTCGGACTCATAAACGTTTTTTGGGACCTACA
>Bacillus_Fam_1207_52_1 Nr. of seq. 1 Alignment length(with gaps) = 52
Alignment score = 0.000000
TGGTCTTCATCACCTCAATGATAACCGTTTCCGCGATTCAGCGAACGAGTTT
>Bacillus_Fam_1208_52_1 Nr. of seq. 1 Alignment length(with gaps) = 52
Alignment score = 0.000000
TGTCCGAATCATAGGCTCATTCGGACTTAAGGCAACAGAATCCTTTGCGACG
>Bacillus_Fam_1209_52_1 Nr. of seq. 1 Alignment length(with gaps) = 52
Alignment score = 0.000000
GAATCGTTCCCGATTGCAAATAAAAATCATAAAAAGAGTAACGCAAGCCCTC
>Bacillus_Fam_1210_52_1 Nr. of seq. 1 Alignment length(with gaps) = 52
Alignment score = 0.000000
TTACCGGGGACTCTTGATTTCCTCCTTCACGGTAAGCATACTCCCTCTTTGN
>Bacillus_Fam_1211_52_1 Nr. of seq. 1 Alignment length(with gaps) = 52
Alignment score = 0.000000
ACCGAAAGAGGCCCCAAGTCCCGCTCCAGGTAACCAAAACCCAGTAAAGGTA
>Bacillus_Fam_1212_52_1 Nr. of seq. 1 Alignment length(with gaps) = 52
Alignment score = 0.000000
ATGGTACCGGAAAAGAGAGAAGCTGTACGATGGCAACGCGAATCTCGTAGCC
>Bacillus_Fam_1213_52_1 Nr. of seq. 1 Alignment length(with gaps) = 52
Alignment score = 0.000000
TGCTCTATCGGTTCGCTTTTGNCTCTTGCAGTACCATTGGCGTTNGTGCTCC
>Bacillus_Fam_1214_52_1 Nr. of seq. 1 Alignment length(with gaps) = 52
Alignment score = 0.000000
GATCTTCCCGCATCACCGGGATTCGGGTACTTCATGCAATGCTAGCTGCCGC
>Bacillus_Fam_1215_52_1 Nr. of seq. 1 Alignment length(with gaps) = 52
Alignment score = 0.000000
CAAAAATGTAAAAGGAANGCCGGCTTGTATATAGATTTTCGGTGTAGAAGGT
>Bacillus_Fam_1216_52_1 Nr. of seq. 1 Alignment length(with gaps) = 52
Alignment score = 0.000000
GCACAGCAATGGTACCGCTTCTCCTTTAACCGGTTCCATAGCAGTACGCCCG

>Bacillus_Fam_1217_52_1 Nr. of seq. 1 Alignment length(with gaps) = 52
Alignment score = 0.000000
CAAGTGGACGAATCCGCCGGATGCGGGAAGATAAGGATGGATGAACCCTGCT
>Bacillus_Fam_1218_52_1 Nr. of seq. 1 Alignment length(with gaps) = 52
Alignment score = 0.000000
CCAACTTGGTCGCCAAAGCCCCGGAATGAAGCCCAAGAACGAAGCAANNAGT
>Bacillus_Fam_1219_52_1 Nr. of seq. 1 Alignment length(with gaps) = 52
Alignment score = 0.000000
TGCCCGAAAGAGCCGTATGCGGGAATTTGAACAAGACCCATCAATCCCGAAG
>Bacillus_Fam_1220_52_1 Nr. of seq. 1 Alignment length(with gaps) = 52
Alignment score = 0.000000
CAAAATTAAAACAAGAAGTGCTTCATACAGCGTTTATGAGGTACATGGTTTT
>Bacillus_Fam_1221_52_1 Nr. of seq. 1 Alignment length(with gaps) = 52
Alignment score = 0.000000
TACATTTTACAAGCATCTATTCAAAAATGTAAAAGCAAAGCAAGGATACCTA
>Bacillus_Fam_1222_52_1 Nr. of seq. 1 Alignment length(with gaps) = 52
Alignment score = 0.000000
GTCCGATGGAGAAGGAAAGCGTGAGGCTATGAGGCTGGAAAGAAGAGAAGCG
>Bacillus_Fam_1223_52_1 Nr. of seq. 1 Alignment length(with gaps) = 52
Alignment score = 0.000000
GCTCCCTTTTGACCNCTTTTCGAGTTCAGCGCTNTTTTCNGGTGAATAGGTG
>Bacillus_Fam_1224_52_1 Nr. of seq. 1 Alignment length(with gaps) = 52
Alignment score = 0.000000
TCTTTCTTACCGTAACCACCTCTCAAACTAGTCTTCGGTCGTTTAAGAGCCC
>Bacillus_Fam_1225_52_1 Nr. of seq. 1 Alignment length(with gaps) = 52
Alignment score = 0.000000
TTCCAATCAAGGGNATTGGTGCATGAACCCGAAAAAAACCGNGTAGTTCAAG
>Bacillus_Fam_1226_52_1 Nr. of seq. 1 Alignment length(with gaps) = 52
Alignment score = 0.000000
GCCACTTTTTTCGCTTCTCCCTTCCATTTTGGCTTTCATCTTGGTAATGAAC
>Bacillus_Fam_1227_52_1 Nr. of seq. 1 Alignment length(with gaps) = 52
Alignment score = 0.000000
ATAAGTGGTTTTCATTCAACAAATGAACGCCACTTTTTATGGAAAAGATGGC
>Bacillus_Fam_1228_52_1 Nr. of seq. 1 Alignment length(with gaps) = 52
Alignment score = 0.000000
GTTCATCAGTTGGATGAAAGCCGAAATCCCGTGGAGCAAGAGGAAAAGAGGC
>Bacillus_Fam_1229_52_1 Nr. of seq. 1 Alignment length(with gaps) = 52
Alignment score = 0.000000
TTTAGTGCCCGCGTATACCAGAAATTAGTAGAATGAGGTCACTAAAAGTTAA
>Bacillus_Fam_1230_52_1 Nr. of seq. 1 Alignment length(with gaps) = 52
Alignment score = 0.000000
TCCAGATATTAGGAAAACCAGGGCACTATATTCAGATTTAACACCCTTAAAA
>Bacillus_Fam_1231_52_1 Nr. of seq. 1 Alignment length(with gaps) = 52
Alignment score = 0.000000
AGTCCGAATGACGGTCTGATTAGGACAGCAACAGCAGCTCAAACTAATTCAC
>Bacillus_Fam_1232_52_1 Nr. of seq. 1 Alignment length(with gaps) = 52
Alignment score = 0.000000
GAAGTTCATCTGGCTCATGAACGTCATTACAAAGAGAAAAGACTGAGCAAAT
>Bacillus_Fam_1233_52_1 Nr. of seq. 1 Alignment length(with gaps) = 52
Alignment score = 0.000000
TCTCTTACCATCCATCATGATTTTCCGCCCGTTTGGGCACTGAGACGCCTTC
>Bacillus_Fam_1234_52_1 Nr. of seq. 1 Alignment length(with gaps) = 52
Alignment score = 0.000000
ATCTTTCCCTTCCTTTTTCAGCCAAACGTCGAAGTAGAACCACTCTACTTCA

>Bacillus_Fam_1235_52_1 Nr. of seq. 1 Alignment length(with gaps) = 52
Alignment score = 0.000000
GAAAGGGAGAAACGGTCGATAAGAGCGGTTCATAACGACCATTTGGGTTGAG
>Bacillus_Fam_1236_52_1 Nr. of seq. 1 Alignment length(with gaps) = 52
Alignment score = 0.000000
CTCATGAGGACAGGAATCATGTTTAATTCACATTTTTTGTCCTCATGAAAGG
>Bacillus_Fam_1237_52_1 Nr. of seq. 1 Alignment length(with gaps) = 52
Alignment score = 0.000000
ATCGACCGTAATTGACTTTCAGCTTGGCTATCGGGCTTTATAAACCTTTCTT
>Bacillus_Fam_1238_52_1 Nr. of seq. 1 Alignment length(with gaps) = 52
Alignment score = 0.000000
ATGAAGGACTAATCCCTATTAGAAAGACAGCGTTTTGTCCTTCATCAAGCAT
>Bacillus_Fam_1239_52_1 Nr. of seq. 1 Alignment length(with gaps) = 52
Alignment score = 0.000000
GGTTTCCAATCAGCCCTATTGGAACAAGTTAAGGCNAAAAAGTCGGTCAAAA
>Bacillus_Fam_1240_52_1 Nr. of seq. 1 Alignment length(with gaps) = 52
Alignment score = 0.000000
TTTTGTCCTCATGAAGCACTCATGCGGACAGTTTCGCCTTGGGAACCCCGGG
>Bacillus_Fam_1241_52_1 Nr. of seq. 1 Alignment length(with gaps) = 52
Alignment score = 0.000000
TCATAGTGCCCGAAACCTGAAAAAAACCAGCCACTCGGGCACTGAGAGCAGT
>Bacillus_Fam_1242_52_1 Nr. of seq. 1 Alignment length(with gaps) = 52
Alignment score = 0.000000
CTATTGGACAGTTGGGGTTGTATCTTCGTCGATATTGTCCTATAGACCGCTT
>Bacillus_Fam_1243_52_1 Nr. of seq. 1 Alignment length(with gaps) = 52
Alignment score = 0.000000
GGATAACGGGCTCCAATGAGGGTTATTGGCGACCGAAACGCTCTCGAATTCC
>Bacillus_Fam_1244_52_1 Nr. of seq. 1 Alignment length(with gaps) = 52
Alignment score = 0.000000
GGTCACAGAGAGCGATCCTCAGTGCCCGAAGAGAGGCAAAAAGCTGTCTAGC
>Bacillus_Fam_1245_52_1 Nr. of seq. 1 Alignment length(with gaps) = 52
Alignment score = 0.000000
TGTCCCTTAGATCCTCTCTCTCGGACACTCCTTCTCCTTTTTCCTCAATATT
>Bacillus_Fam_1246_52_1 Nr. of seq. 1 Alignment length(with gaps) = 52
Alignment score = 0.000000
TGTCCTTTAGACGACTTCTCTCGGACACTTCCAACCCCTTCTCCCGCGAGAC
>Bacillus_Fam_1247_52_1 Nr. of seq. 1 Alignment length(with gaps) = 52
Alignment score = 0.000000
CTATTGGACAGATCCTCTCCTCTTTTGGCTCGGACTGTCCTTTAGCGCCCTC
>Bacillus_Fam_1248_52_1 Nr. of seq. 1 Alignment length(with gaps) = 52
Alignment score = 0.000000
TATCGAAGACAAATTCCACCGCTTCCACCCTCGTTTTGTCCTCAAGACGCCT
>Bacillus_Fam_1249_52_1 Nr. of seq. 1 Alignment length(with gaps) = 52
Alignment score = 0.000000
GGGCACGTAGACGCNTTCTACGTTACCTTGAGGCGGGATATTTACGCTGGAT
>Bacillus_Fam_1250_52_1 Nr. of seq. 1 Alignment length(with gaps) = 52
Alignment score = 0.000000
GGGCACGTAGANCACGTCTACGTGACCGAACCGTCAGTTTTTCAGCTGNTTG
>Bacillus_Fam_1251_52_1 Nr. of seq. 1 Alignment length(with gaps) = 52
Alignment score = 0.000000
CGGGCACGTAGAACACTTCTATGTGACCTTACACTGCTTTTTCATGCCCGTT
>Bacillus_Fam_1252_52_1 Nr. of seq. 1 Alignment length(with gaps) = 52
Alignment score = 0.000000
TATTGAAGACAAAATGAGATCACTCCCTTTTAAATTTGTCCCCAATACCCTC

>Bacillus_Fam_1253_52_1 Nr. of seq. 1 Alignment length(with gaps) = 52
Alignment score = 0.000000
AAGTGTCTCCAATAAGCTTCATTGGAGACAAAACCCNCTCTTTTTTCACGAG
>Bacillus_Fam_1254_52_1 Nr. of seq. 1 Alignment length(with gaps) = 52
Alignment score = 0.000000
AAAGTCACGTAGAAACCCTCTACGTGACCGTACCTCCCTTTTTNCCTGCTCT
>Bacillus_Fam_1255_52_1 Nr. of seq. 1 Alignment length(with gaps) = 52
Alignment score = 0.000000
CGGATTTTCCCTTTCAGACATCACTTAGACGGNCTCTACTTAGTGTCTAAAC
>Bacillus_Fam_1256_52_1 Nr. of seq. 1 Alignment length(with gaps) = 52
Alignment score = 0.000000
ATGAGAACTGAAACCANGAAAAGCGCGGCNGTCTTGTCCTCATGAAGCGTTC
>Bacillus_Fam_1257_52_1 Nr. of seq. 1 Alignment length(with gaps) = 52
Alignment score = 0.000000
GACCGAGCAGCTGGAAAAATAGGAAGAAGAGTCGCGTAGAGAGGTTCTANGC
>Bacillus_Fam_1258_52_1 Nr. of seq. 1 Alignment length(with gaps) = 52
Alignment score = 0.000000
TAAGAAAGGCTCTCAAGGACAAAACGCTAGTAAAAATTAATAGAANTGTCTT
>Bacillus_Fam_1259_52_1 Nr. of seq. 1 Alignment length(with gaps) = 52
Alignment score = 0.000000
TCCATTGCCCAAAACCGGCTAAATCATCAAATTATGGTCAATGCAGCCCCAT
>Bacillus_Fam_1260_52_1 Nr. of seq. 1 Alignment length(with gaps) = 52
Alignment score = 0.000000
TGTCTGAATGATTATTTTGCAGCCAGAGATCAGTTAGAATCCGTCTAGTTGC
>Bacillus_Fam_1261_52_1 Nr. of seq. 1 Alignment length(with gaps) = 52
Alignment score = 0.000000
ATGAACCTTTCATAGAGCGCGTCGGAGTAAAAAAATCGAGCTCCCGCGTACT
>Bacillus_Fam_1262_52_1 Nr. of seq. 1 Alignment length(with gaps) = 52
Alignment score = 0.000000
GTTCATAGAGCGCGAACNTGAGGGGAAATTCCAAAAACGCGCACTATAAAGA
>Bacillus_Fam_1263_52_1 Nr. of seq. 1 Alignment length(with gaps) = 52
Alignment score = 0.000000
TTTCGGAAAAGGTGTTCATAGAAGAGGTCAATGAGCACATGAGGCTGAGTTT
>Bacillus_Fam_1264_52_1 Nr. of seq. 1 Alignment length(with gaps) = 52
Alignment score = 0.000000
ACTATGGGAGGTTCATAGAGCGNGAAATTAGGAAAAAGGAAAGACTCCGCGC
>Bacillus_Fam_1265_52_1 Nr. of seq. 1 Alignment length(with gaps) = 52
Alignment score = 0.000000
TATTGCCCGAAGAGAAGAAAAAATAAATCCCGCGGTAAAAAGACCGGGCTTC
>Bacillus_Fam_1266_52_1 Nr. of seq. 1 Alignment length(with gaps) = 52
Alignment score = 0.000000
AGCGTTTATGAGAACAGAGAGCTAATGATTTGGGTCGGAACTGTCCTCATGA
>Bacillus_Fam_1267_52_1 Nr. of seq. 1 Alignment length(with gaps) = 52
Alignment score = 0.000000
AAAAAAGAGAGCGCCGGGTAGAATANAGAGCATCTATGCGACCGAGCCCGAG
>Bacillus_Fam_1268_52_1 Nr. of seq. 1 Alignment length(with gaps) = 52
Alignment score = 0.000000
TCAATGAACACGAATCGGCAAAATTTCAGGCAGGAAGTGTTCATAGAGAGGT
>Bacillus_Fam_1269_52_1 Nr. of seq. 1 Alignment length(with gaps) = 52
Alignment score = 0.000000
TCTACGTGCCCCAAGTTCAATAAATTAGTGGGTTTCGGTCGCGTAGAGCCCT
>Bacillus_Fam_1270_52_1 Nr. of seq. 1 Alignment length(with gaps) = 52
Alignment score = 0.000000
TTGACCTTTTCTTTNAAAATCAATTAGTTTTAGGCAATGNACATGGCTTCCA

>Bacillus_Fam_1271_52_1 Nr. of seq. 1 Alignment length(with gaps) = 52
Alignment score = 0.000000
CATCCTCTTCAGGTGAAAAAAGCCCAATTATTCACCGGAGTGCTCATATTTT
>Bacillus_Fam_1272_52_1 Nr. of seq. 1 Alignment length(with gaps) = 52
Alignment score = 0.000000
TTATTCCTCTAAAGCAACGCCACACGCGCCTCTTCAGGTGAACAAATCGCAT
>Bacillus_Fam_1273_52_1 Nr. of seq. 1 Alignment length(with gaps) = 52
Alignment score = 0.000000
GTTATTTACACGAAGGGATTTTGAACCGAATGCTTCAGGTGAATAAATTGCG
>Bacillus_Fam_1274_52_1 Nr. of seq. 1 Alignment length(with gaps) = 52
Alignment score = 0.000000
ATTTATTCCCATGAAGCGAGAGGAAAACCGGAGCTTCCGGTGGATAAACTGA
>Bacillus_Fam_1275_52_1 Nr. of seq. 1 Alignment length(with gaps) = 52
Alignment score = 0.000000
AAAATGGTCACAGAGTGCCAAACTAAGACCAAAATGACAAGGGAGCAAGCGG
>Bacillus_Fam_1276_52_1 Nr. of seq. 1 Alignment length(with gaps) = 52
Alignment score = 0.000000
GGAAGGTAGGAGAGAGGCAGTCTCATCGCCCGTTCCCAGCTATTTACCATTA
>Bacillus_Fam_1277_52_1 Nr. of seq. 1 Alignment length(with gaps) = 52
Alignment score = 0.000000
CATAGAGCGAGTCAATGGGACATTTNGCAATTTTCNAGAGCAGCAAATGTCC
>Bacillus_Fam_1278_52_1 Nr. of seq. 1 Alignment length(with gaps) = 52
Alignment score = 0.000000
TGATGGACAAAACAGTAGATAGAGCGCAAAGCAAAGTCCATCAATCCTGAGA
>Bacillus_Fam_1279_52_1 Nr. of seq. 1 Alignment length(with gaps) = 52
Alignment score = 0.000000
TTCCTTCGTTTCTTCATGGGTTTTGTACCATTGATTGCTTCTATGGGACATG
>Bacillus_Fam_1280_52_1 Nr. of seq. 1 Alignment length(with gaps) = 52
Alignment score = 0.000000
GATTTTTCCTAAAGTACCTTGTTGCGGGCGACATGAGCGCTTCTTGGCTACC
>Bacillus_Fam_1281_52_1 Nr. of seq. 1 Alignment length(with gaps) = 52
Alignment score = 0.000000
GACTTTTTCGGGAGACATGAGCTAGTCTTGGCGACCGGTTTCACAAGTTGAT
>Bacillus_Fam_1282_52_1 Nr. of seq. 1 Alignment length(with gaps) = 52
Alignment score = 0.000000
TTCTATGAGACAAAAATCGGGGAGCAGACGAGGNGTTTGTCTCATTGGAGCG
>Bacillus_Fam_1283_52_1 Nr. of seq. 1 Alignment length(with gaps) = 52
Alignment score = 0.000000
GACTCTTTGAGCNGATTTTTCGGGCGTTGGGTCCTCTATCNGGCTTATTGAN
>Bacillus_Fam_1284_52_1 Nr. of seq. 1 Alignment length(with gaps) = 52
Alignment score = 0.000000
TTTGTCCTTCATTCGCTTGATGATGGACTTTTTCTAGAGCGATCGGCTTCGN
>Bacillus_Fam_1285_52_1 Nr. of seq. 1 Alignment length(with gaps) = 52
Alignment score = 0.000000
ATGATGGACATTTCGATAATTTCCTCAGCTGCTTTTGGCCTTCATCTCTTCG
>Bacillus_Fam_1286_52_1 Nr. of seq. 1 Alignment length(with gaps) = 52
Alignment score = 0.000000
ATGATGGACACTTGGAGCGGATTCTTTTTCGCTTTTGTCCATCATCTCTCGC
>Bacillus_Fam_1287_52_1 Nr. of seq. 1 Alignment length(with gaps) = 52
Alignment score = 0.000000
TTTTGTCCCATAGAACCCCTCTATGGGACATTTCCTCTTGCCTATTCTGATC
>Bacillus_Fam_1288_52_1 Nr. of seq. 1 Alignment length(with gaps) = 52
Alignment score = 0.000000
AGGATTTCAGGAATCAGAACATCAATGTAAATCCCGAAACCAGTTCCATGCA

>Bacillus_Fam_1289_52_1 Nr. of seq. 1 Alignment length(with gaps) = 52
Alignment score = 0.000000
ATTTGGATATTAAGGTCGTGATAAGCGCCTAAACACCCCTCTGTTATACAGT
>Bacillus_Fam_1290_52_1 Nr. of seq. 1 Alignment length(with gaps) = 52
Alignment score = 0.000000
TCTCCTTCCCCAACTCTATTGATGACCGGCATTTCGGGAAGTACTCTGCCTN
>Bacillus_Fam_1291_52_1 Nr. of seq. 1 Alignment length(with gaps) = 52
Alignment score = 0.000000
ATTCCCGAATTCCACTTAATGCACTTGTTTCGGGAATTACTACGGGCTTCTC
>Bacillus_Fam_1292_52_1 Nr. of seq. 1 Alignment length(with gaps) = 52
Alignment score = 0.000000
ACGGGCACTTTGANCGCACNTTTGTTCCCNCCAACACGCTTTTTCCAAGCCG
>Bacillus_Fam_1293_52_1 Nr. of seq. 1 Alignment length(with gaps) = 52
Alignment score = 0.000000
GAATTAGAATCCAAGAGTCATTCCTGAAACGGTCTCAACCATGGAAGTTCGG
>Bacillus_Fam_1294_52_1 Nr. of seq. 1 Alignment length(with gaps) = 52
Alignment score = 0.000000
AACGCTTAATCACGACAGTATTATCCAGATAACGGTAATTAGGGGCGTGATA
>Bacillus_Fam_1295_52_1 Nr. of seq. 1 Alignment length(with gaps) = 52
Alignment score = 0.000000
AGCGGATTTCGGGAATTACTTTCCCTTTGCATTTCCCGAAACTTTGTCTCCT
>Bacillus_Fam_1296_52_1 Nr. of seq. 1 Alignment length(with gaps) = 52
Alignment score = 0.000000
TTACGCCTTAAACACGACTCCAATTCCCAGGTTTTGGTGTTTCGTGTCTCGA
>Bacillus_Fam_1297_52_1 Nr. of seq. 1 Alignment length(with gaps) = 52
Alignment score = 0.000000
GTTCTATTTCCCCAAACCGGCACTCGCCTTCTCTTTCGGGAATTACTTTGCC
>Bacillus_Fam_1298_52_1 Nr. of seq. 1 Alignment length(with gaps) = 52
Alignment score = 0.000000
TTTTGTCCTTCATACCACCGATGAAGGACTTTTGACAACGAAAATTCACCAG
>Bacillus_Fam_1299_52_1 Nr. of seq. 1 Alignment length(with gaps) = 52
Alignment score = 0.000000
TGAAGGACTTTTATCACCTTTTATTCTCGCGAAAAGTCCTTCATACCTGCGA
>Bacillus_Fam_1300_52_1 Nr. of seq. 1 Alignment length(with gaps) = 52
Alignment score = 0.000000
GATGAAGGACTATTCTTGCTTTGATTGTGCTCGAATGGTCCATCATCCCCTC
>Bacillus_Fam_1301_52_1 Nr. of seq. 1 Alignment length(with gaps) = 52
Alignment score = 0.000000
AAGTCCTTCATCGTGGTTATGAAGACCAATAAGAGGTTAATTAGGAAGCGAA
>Bacillus_Fam_1302_52_1 Nr. of seq. 1 Alignment length(with gaps) = 52
Alignment score = 0.000000
CGGTCGCATAGCAAGGTTCTATAAGCCCGGAAACACAGGAAATCCAATGGAA
>Bacillus_Fam_1303_52_1 Nr. of seq. 1 Alignment length(with gaps) = 52
Alignment score = 0.000000
AGTGAACCGGCGGAAACAGAGTTCAAAGAAGGGGCCTATGTTCCCGAAATCG
>Bacillus_Fam_1304_52_1 Nr. of seq. 1 Alignment length(with gaps) = 52
Alignment score = 0.000000
GCGTGTCATAGAGCCCGAATGAAACGCGAANTCGCTGGGGCTTAGTGAAAAC
>Bacillus_Fam_1305_52_1 Nr. of seq. 1 Alignment length(with gaps) = 52
Alignment score = 0.000000
TGGAATTGTCATCATAGCAATCATGAAGACAATCTCGAGCGGATTTTGTGAG
>Bacillus_Fam_1306_52_1 Nr. of seq. 1 Alignment length(with gaps) = 52
Alignment score = 0.000000
CCTTATGAAGACACTTTCGGTCCACTTTCTCTCTCGGATGTGTCTTCATCCA

>Bacillus_Fam_1307_52_1 Nr. of seq. 1 Alignment length(with gaps) = 52
Alignment score = 0.000000
CGTTTTCCTTGCTCGCTTCGAAGTTTGCGTGCCATTGCGCCTCTATGACACG
>Bacillus_Fam_1308_52_1 Nr. of seq. 1 Alignment length(with gaps) = 52
Alignment score = 0.000000
CGCGAATTTCCCTCCCGCTCCTAATTTGGTGTGTCATACTGCTCCTATGACG
>Bacillus_Fam_1309_52_1 Nr. of seq. 1 Alignment length(with gaps) = 52
Alignment score = 0.000000
TTTCCCTCTCAACATTGTCTTCATCCACCTTGTGAAGACAGTACCATGCTTG
>Bacillus_Fam_1310_52_1 Nr. of seq. 1 Alignment length(with gaps) = 52
Alignment score = 0.000000
AGATTGTCTTCATCACNCTTATAATGACAATCTTGAACTGATTTTCACTGTG
>Bacillus_Fam_1311_52_1 Nr. of seq. 1 Alignment length(with gaps) = 52
Alignment score = 0.000000
AAGACACTTTTGCCGTGTTTTTCCTGCTGACATTGTCTTCAGACCACTGATG
>Bacillus_Fam_1312_52_1 Nr. of seq. 1 Alignment length(with gaps) = 52
Alignment score = 0.000000
CTCATGAACCGCTTATGAGTAAGTAAAATACATGATTTAGATTATTTAGTTA
>Bacillus_Fam_1313_52_1 Nr. of seq. 1 Alignment length(with gaps) = 52
Alignment score = 0.000000
AATGTGTCTCAAGAATAGGGTTGGAAACAGATAAACCGGGAATNNCGTTGAA
>Bacillus_Fam_1314_52_1 Nr. of seq. 1 Alignment length(with gaps) = 52
Alignment score = 0.000000
GGTAACCTTTCCGGGGCTTTGGTTACCCGGATCTCATCACAGAGCCNACTTC
>Bacillus_Fam_1315_52_1 Nr. of seq. 1 Alignment length(with gaps) = 52
Alignment score = 0.000000
TCTTCAAGGTAATCAAAAGCTCACAAAGGTACCCGAGCCGCCGGAATTACCT
>Bacillus_Fam_1316_52_1 Nr. of seq. 1 Alignment length(with gaps) = 52
Alignment score = 0.000000
TGGAGACACATTTTATTGCTAAAAAGTAAATAATGTGTTTCAGAACCAGNGC
>Bacillus_Fam_1317_52_1 Nr. of seq. 1 Alignment length(with gaps) = 52
Alignment score = 0.000000
TAAGACACATAATGTGACCAAACCCCAAAAAATGTGTCTCACCCGCCAGTCT
>Bacillus_Fam_1318_52_1 Nr. of seq. 1 Alignment length(with gaps) = 52
Alignment score = 0.000000
CGTTGTTTCGGGCACTATGAACCCTCTATAGTTCCCAGTCTCTTGCTTCCAT
>Bacillus_Fam_1319_52_1 Nr. of seq. 1 Alignment length(with gaps) = 52
Alignment score = 0.000000
CCGTTTGAGCAGCTCGAAGGAGATAGCGGTCACTATGAAAGCGTCATGGTGA
>Bacillus_Fam_1320_52_1 Nr. of seq. 1 Alignment length(with gaps) = 52
Alignment score = 0.000000
ACTTCAGACAACTTCTGCNGCCAATTACTTAAACCTGTCCGAACTCTTGCCT
>Bacillus_Fam_1321_52_1 Nr. of seq. 1 Alignment length(with gaps) = 52
Alignment score = 0.000000
GAGCAACTTGATCATAGACCTCCTATGATTCACTTCCCTACAATTCATGTGC
>Bacillus_Fam_1322_52_1 Nr. of seq. 1 Alignment length(with gaps) = 52
Alignment score = 0.000000
TGTCTGAAGTTGAGNAGCCTTCGGACAGGTTAGAAGCCTACAGAGTAAAAGC
>Bacillus_Fam_1323_52_1 Nr. of seq. 1 Alignment length(with gaps) = 52
Alignment score = 0.000000
AAGCTGTCCGAAGAGAGCTCAGGTTCAGACAGGTTTGCTCAGGTTNAGTAGA
>Bacillus_Fam_1324_52_1 Nr. of seq. 1 Alignment length(with gaps) = 52
Alignment score = 0.000000
TCTGATTCCCTTTCAGTAGTGNATCGGGGTAGAAAGGGAACGAAAGAGCAAG

>Bacillus_Fam_1325_52_1 Nr. of seq. 1 Alignment length(with gaps) = 52
Alignment score = 0.000000
 AGATTCCCTTACTAGGCAAAAATGAAGTGCAAAGGGAACGAAAGGTAGTCAC
>Bacillus_Fam_1326_52_1 Nr. of seq. 1 Alignment length(with gaps) = 52
Alignment score = 0.000000
 GAAAGCTGTCCGAACTTCACTTGGGTTCGGACTACTTTTCCTTTACGACCAC
>Bacillus_Fam_1327_52_1 Nr. of seq. 1 Alignment length(with gaps) = 52
Alignment score = 0.000000
 ATTCCCTTTGTCGGAGAAAATACACAGGAAAGGGAACGAAAGGAGCGCTTTG
>Bacillus_Fam_1328_52_1 Nr. of seq. 1 Alignment length(with gaps) = 52
Alignment score = 0.000000
 AAGCTGTCTGAACCTACCCCACCTTCGGACAGCTTGACTCCGAAAAACCCAA
>Bacillus_Fam_1329_52_1 Nr. of seq. 1 Alignment length(with gaps) = 52
Alignment score = 0.000000
 ATTATGGGTAACGATAAAGGCGTGATCGTGCCCTGAACTCGAGAATATAGTG
>Bacillus_Fam_1330_52_1 Nr. of seq. 1 Alignment length(with gaps) = 52
Alignment score = 0.000000
 GAGTCTGAACCTNGCGTGAGTTCGGACAGGAATCGTGANTGTGAATGTAAGC
>Bacillus_Fam_1331_52_1 Nr. of seq. 1 Alignment length(with gaps) = 52
Alignment score = 0.000000
 TGTCCAATAGGATGCCTCTATCGGACATAACTCACTTCCCAAGACTAGATTC
>Bacillus_Fam_1332_52_1 Nr. of seq. 1 Alignment length(with gaps) = 52
Alignment score = 0.000000
 ACGGCTCTATCGAACAAAACTATGGGCCTTCACGCTTGGTTTGTCCAATAGG
>Bacillus_Fam_1333_52_1 Nr. of seq. 1 Alignment length(with gaps) = 52
Alignment score = 0.000000
 CTATTGGACAAAATCGCTTCACGGCACTGGCGGTTTGTCTAATACAGGCTTC
>Bacillus_Fam_1334_52_1 Nr. of seq. 1 Alignment length(with gaps) = 52
Alignment score = 0.000000
 ATCAGAGACCTTTGGACAGCTTCTTCGNTTGATATGGTCATTGACACCTCTC
>Bacillus_Fam_1335_52_1 Nr. of seq. 1 Alignment length(with gaps) = 52
Alignment score = 0.000000
 TTTTGTCCGATAGAGACCTCTTATTGGACAAAACCTTTGCTGGCACCGTTCG
>Bacillus_Fam_1336_52_1 Nr. of seq. 1 Alignment length(with gaps) = 52
Alignment score = 0.000000
 ATAAGTGCCCGTTCTAGGGTCCCTAGCAAGGAGAGCGGTCATCTAACTCGAG
>Bacillus_Fam_1337_52_1 Nr. of seq. 1 Alignment length(with gaps) = 52
Alignment score = 0.000000
 ACTTCAGACAGAAGAAGGTAAAAGAAGTAAGGGAGAGTCTGAANTNGAAGTG
>Bacillus_Fam_1338_52_1 Nr. of seq. 1 Alignment length(with gaps) = 52
Alignment score = 0.000000
 TTCTATTGGACATTCTCATCGATACGAACGCACGTTTTGTCCAATAGACGCC
>Bacillus_Fam_1339_52_1 Nr. of seq. 1 Alignment length(with gaps) = 52
Alignment score = 0.000000
 AAGAGCAAATCGTTGACGAAACGAGCATTTTGAAAGCTGAACGTCAACGAAT
>Bacillus_Fam_1340_52_1 Nr. of seq. 1 Alignment length(with gaps) = 52
Alignment score = 0.000000
 TCGTTACCGTTTTTGGCGTACTCTTCTGTTTTCGGGCACATTAAACTCTTTN
>Bacillus_Fam_1341_52_1 Nr. of seq. 1 Alignment length(with gaps) = 52
Alignment score = 0.000000
 CACCAAGAGCTTTCCTTGGCGCAGTAATATAGGTTTCATCTACTAATAACTG
>Bacillus_Fam_1342_52_1 Nr. of seq. 1 Alignment length(with gaps) = 52
Alignment score = 0.000000
 GGTCTTCATAGCACCGATGAAGACCTGAAACAGTTGGAAACTGCTTCGTACA

>Bacillus_Fam_1343_52_1 Nr. of seq. 1 Alignment length(with gaps) = 52
Alignment score = 0.000000
CTCTTCATCTCGCTTATGAAGGGGATTTGGATGAGGTAACTGCCTTGTTTTC
>Bacillus_Fam_1344_52_1 Nr. of seq. 1 Alignment length(with gaps) = 52
Alignment score = 0.000000
ATGTCGTCATGAGTGCGATGAAGACACAAACTCTCCTGGCGAGCGGAAGAAG
>Bacillus_Fam_1345_52_1 Nr. of seq. 1 Alignment length(with gaps) = 52
Alignment score = 0.000000
GGAAAACACAGCAAAAGCCGGATAGAAATCCCCTTCATCAAGGCCATGAAGG
>Bacillus_Fam_1346_52_1 Nr. of seq. 1 Alignment length(with gaps) = 52
Alignment score = 0.000000
GATGAAGACCGTTTAAGGATCCCTCCGNGCCAGAAAAGGTTATCATGGGAGA
>Bacillus_Fam_1347_52_1 Nr. of seq. 1 Alignment length(with gaps) = 52
Alignment score = 0.000000
ATCTATAAAAATGGTCATCATGTACGTGATGAAAACCGAAATCTGGTATGTG
>Bacillus_Fam_1348_52_1 Nr. of seq. 1 Alignment length(with gaps) = 52
Alignment score = 0.000000
CGGTCTTCATGGCCGGGATGAGAACCGATNTTNCTGATTTCTTTGCGAGAAC
>Bacillus_Fam_1349_52_1 Nr. of seq. 1 Alignment length(with gaps) = 52
Alignment score = 0.000000
TGGTTTTCATAAGCGGAATGAAGACCGTTATTAGGGGCTTTGTCCTGCGTAA
>Bacillus_Fam_1350_52_1 Nr. of seq. 1 Alignment length(with gaps) = 52
Alignment score = 0.000000
GATGAAGACCAATCCATNGGAAGGAGCGCGGGNAAACGGTCATCATCAAGGG
>Bacillus_Fam_1351_52_1 Nr. of seq. 1 Alignment length(with gaps) = 52
Alignment score = 0.000000
GGTTTTCATAGCTGGAATGAAGACCGAAAGTAACGGTGATAGATCGAGTTTA
>Bacillus_Fam_1352_52_1 Nr. of seq. 1 Alignment length(with gaps) = 52
Alignment score = 0.000000
TGAGTCTGAATCCGAAGGTTATTCGGACTCCAGCAAGAGGAAACCCAGGCAT
>Bacillus_Fam_1353_52_1 Nr. of seq. 1 Alignment length(with gaps) = 52
Alignment score = 0.000000
TCTTCATAAAGGCAATGAAGACCGTTTGGTTGGTTTTTTANAANGNCTTTGG
>Bacillus_Fam_1354_52_1 Nr. of seq. 1 Alignment length(with gaps) = 52
Alignment score = 0.000000
TTGGTTTTCATAACGGCGATGATGACCGTTTTGCTGAGTGCTGGCTTGCTTT
>Bacillus_Fam_1355_52_1 Nr. of seq. 1 Alignment length(with gaps) = 52
Alignment score = 0.000000
TAAAACTTCATGCTTATGGTCCTCATTCAACGGATGATGACCGTTTGCCTTA
>Bacillus_Fam_1356_52_1 Nr. of seq. 1 Alignment length(with gaps) = 52
Alignment score = 0.000000
ATGATGACCGTTNTTGGTTCATAGAACANAGAAACTGGTCTTCATCTGGGCT
>Bacillus_Fam_1357_51_1 Nr. of seq. 1 Alignment length(with gaps) = 51
Alignment score = 0.000000
AATACGCAGCAATGAAACCGNAAAAATAGTCCTCAATGCCGCACAAATAAA
>Bacillus_Fam_1358_51_1 Nr. of seq. 1 Alignment length(with gaps) = 51
Alignment score = 0.000000
GGGGTTACTCATGAGAAAAGTGAGAGTAAGCCCGAGAAAAGAGAAATGCNC
>Bacillus_Fam_1359_51_1 Nr. of seq. 1 Alignment length(with gaps) = 51
Alignment score = 0.000000
CGTTTTCTCGGGCTTTCTACTCTTTTTTCCGCCCGTAACCCCAAGCATCAT
>Bacillus_Fam_1360_51_1 Nr. of seq. 1 Alignment length(with gaps) = 51
Alignment score = 0.000000
CTAAACAAGACGAGCAAACACAAAACAAACAACATCAATCTGCTCAAGGTA

>Bacillus_Fam_1361_51_1 Nr. of seq. 1 Alignment length(with gaps) = 51
Alignment score = 0.000000
AAAGACAAAACGNGGNGCCGAGACGAAAGAAATGTCCTTCATAGGGTCATG
>Bacillus_Fam_1362_51_1 Nr. of seq. 1 Alignment length(with gaps) = 51
Alignment score = 0.000000
TGAATGAGGACAAAGTAAGCGGGAAAACAGAAACATTGTCCTCGTAGGGCG
>Bacillus_Fam_1363_51_1 Nr. of seq. 1 Alignment length(with gaps) = 51
Alignment score = 0.000000
GATGACAAAACTTCAATGAACNAAGGATTTCTGTCATCGGAAAGCTTCAAC
>Bacillus_Fam_1364_51_1 Nr. of seq. 1 Alignment length(with gaps) = 51
Alignment score = 0.000000
TAAAGGACGTTAACGAGTCAATTTAGAGACTAAAGTCCTATAAACAGCTTN
>Bacillus_Fam_1365_51_1 Nr. of seq. 1 Alignment length(with gaps) = 51
Alignment score = 0.000000
TATCGTCCTATAGAGGGACTTTAAAGGACTTTAATATTGAATGTTGCNAAA
>Bacillus_Fam_1366_51_1 Nr. of seq. 1 Alignment length(with gaps) = 51
Alignment score = 0.000000
GTGACCAAAAAGAAAAAAAGAGAGCTACTTCGGTCACTCCAGAGAATTGGA
>Bacillus_Fam_1367_51_1 Nr. of seq. 1 Alignment length(with gaps) = 51
Alignment score = 0.000000
GCACGCTGAGCCNGCGCTTCGTCAAGAAGAGAAAGCAGCGGCTCAACCGGA
>Bacillus_Fam_1368_51_1 Nr. of seq. 1 Alignment length(with gaps) = 51
Alignment score = 0.000000
CTTATCCTCCGTTTTTCGTACCGTATTTTGGCCAGGGAGAATTTTTCATTC
>Bacillus_Fam_1369_51_1 Nr. of seq. 1 Alignment length(with gaps) = 51
Alignment score = 0.000000
GAAAAAGAAGCCGAGCTAGCGGAAGTACGGCGTATAAGGAGTCATGAAGTT
>Bacillus_Fam_1370_51_1 Nr. of seq. 1 Alignment length(with gaps) = 51
Alignment score = 0.000000
TTTAAAGGACAAAAAAGGAAGCCGAAGCTGGAAAGTGTCCAATAACGAGAG
>Bacillus_Fam_1371_51_1 Nr. of seq. 1 Alignment length(with gaps) = 51
Alignment score = 0.000000
AGTGAAAAGGGAATCAAAGCCCTCAATGATTCCCAAAGAGCCCCAGAACCA
>Bacillus_Fam_1372_51_1 Nr. of seq. 1 Alignment length(with gaps) = 51
Alignment score = 0.000000
AATACGCCGAACTTCCCTTTACTCGGCTCTTTTTTCTGCTTCACAGTTCTA
>Bacillus_Fam_1373_51_1 Nr. of seq. 1 Alignment length(with gaps) = 51
Alignment score = 0.000000
GTGCGTATTCATCTGATTTGGTAATCAAATTTCAACCTGAGCCGAATTACG
>Bacillus_Fam_1374_51_1 Nr. of seq. 1 Alignment length(with gaps) = 51
Alignment score = 0.000000
TTTTCATTACCTATTATNGTTTATTCAAACCCTAATTGAGNGAGTTTCCCT
>Bacillus_Fam_1375_51_1 Nr. of seq. 1 Alignment length(with gaps) = 51
Alignment score = 0.000000
TTGGTCTTCATCCGCTTAATGAAGACCTTTTTTCCGATTTCGTTTACACTT
>Bacillus_Fam_1376_51_1 Nr. of seq. 1 Alignment length(with gaps) = 51
Alignment score = 0.000000
CGTTACCTTTTCTTCGTATTTTCCCATTTTTGGTCACATAGACCCCTTCTA
>Bacillus_Fam_1377_51_1 Nr. of seq. 1 Alignment length(with gaps) = 51
Alignment score = 0.000000
GTCACGTAGAACCTCTCTACGTGACCTCTCCCATGCTTTTTCTCCAAAATA
>Bacillus_Fam_1378_51_1 Nr. of seq. 1 Alignment length(with gaps) = 51
Alignment score = 0.000000
CGCTTTTATCGTCACGTAAAATGATTTCAACGCGTTTTGCTGATAATAAAT

>Bacillus_Fam_1379_51_1 Nr. of seq. 1 Alignment length(with gaps) = 51
Alignment score = 0.000000
 TCCGTTATTTGTCCAAAACCCTATGCTACAGCTATTTTNCCGGACACCAGG
>Bacillus_Fam_1380_51_1 Nr. of seq. 1 Alignment length(with gaps) = 51
Alignment score = 0.000000
 GCTTCAGTTTCTTCATCCGAAATCATCTCAGCTGTACCTGCAGGCTCTTTT
>Bacillus_Fam_1381_51_1 Nr. of seq. 1 Alignment length(with gaps) = 51
Alignment score = 0.000000
 ATATTTTTTTGAACGTTCTTCACGTTTGCTAGATCGTTTTTCGCGATCATA
>Bacillus_Fam_1382_51_1 Nr. of seq. 1 Alignment length(with gaps) = 51
Alignment score = 0.000000
 AGTTGTCCATTTGCGGNGGGCTATTGGACAACTTTTTCNGAAAAACACCAG
>Bacillus_Fam_1383_51_1 Nr. of seq. 1 Alignment length(with gaps) = 51
Alignment score = 0.000000
 TTGGACAGCTTCGCGCTATTTTCCAAAAAGTTGTCCATTAGACACAAGCTA
>Bacillus_Fam_1384_51_1 Nr. of seq. 1 Alignment length(with gaps) = 51
Alignment score = 0.000000
 TTTTCTCTAATTACGATATTTCTGTCCAATAGGACCTGGCTATTGGACAGG
>Bacillus_Fam_1385_51_1 Nr. of seq. 1 Alignment length(with gaps) = 51
Alignment score = 0.000000
 CTTGGCGCAAAAAGTTTTCCCGAGGNTGGAGAAATTGCGTTAACGCAGGGT
>Bacillus_Fam_1386_51_1 Nr. of seq. 1 Alignment length(with gaps) = 51
Alignment score = 0.000000
 CTCAGAGACAAAATCCAGGNAAAAAGTAGNGATTTGTCTCTGATCGGGTGT
>Bacillus_Fam_1387_51_1 Nr. of seq. 1 Alignment length(with gaps) = 51
Alignment score = 0.000000
 CTTCGGTCGAATAGAAACGCCCTATTCGCGCATTTCTGTGATTTTCTTTTN
>Bacillus_Fam_1388_51_1 Nr. of seq. 1 Alignment length(with gaps) = 51
Alignment score = 0.000000
 CGCCCCAATTCACCCGAAGAAAAGGAAATACACGTCAANGGTCGAATAGAT
>Bacillus_Fam_1389_51_1 Nr. of seq. 1 Alignment length(with gaps) = 51
Alignment score = 0.000000
 GGTCGAATAGAAGCGATCAATTCGACCGTTGCTGGAAAATCCGCTTTGTTT
>Bacillus_Fam_1390_51_1 Nr. of seq. 1 Alignment length(with gaps) = 51
Alignment score = 0.000000
 CTTATACATTTTTTCTTTCTTCCCATGAAAATCTATACGCATCCTCCCTTC
>Bacillus_Fam_1391_51_1 Nr. of seq. 1 Alignment length(with gaps) = 51
Alignment score = 0.000000
 TTCGGACACAGAAAGGGGGAACCCGTGGAATTGAGTCAGAACCCGGNCAAG
>Bacillus_Fam_1392_51_1 Nr. of seq. 1 Alignment length(with gaps) = 51
Alignment score = 0.000000
 TAATCGTTCCCGAAAATGCNGTGTANGAGGCTGGNGGTAATGAATAGGGAG
>Bacillus_Fam_1393_51_1 Nr. of seq. 1 Alignment length(with gaps) = 51
Alignment score = 0.000000
 ATAGAGCGCGTGTCCAGTAATTTATTGGAAAAGGCGTCCTATGAACCATTC
>Bacillus_Fam_1394_51_1 Nr. of seq. 1 Alignment length(with gaps) = 51
Alignment score = 0.000000
 AAAATGGTCCTAGTTCACCAAACTGGAACCAAAATCAAGAAAGACAGCGTA
>Bacillus_Fam_1395_51_1 Nr. of seq. 1 Alignment length(with gaps) = 51
Alignment score = 0.000000
 AACTGTGACCAAAAATGGCGGAGAAATCAAAAAAACGGTCACAGAGTGTAA
>Bacillus_Fam_1396_51_1 Nr. of seq. 1 Alignment length(with gaps) = 51
Alignment score = 0.000000
 AAAAAAAGTAAAAAAACATGTAAAGAGTCTCATAGGAGCATTCAATGGGAC

>Bacillus_Fam_1397_51_1 Nr. of seq. 1 Alignment length(with gaps) = 51
Alignment score = 0.000000
TAAAAGAGAACTTAGGATGAATAGCTTCCACAAACTGTAAGTGGAAGCATC
>Bacillus_Fam_1398_51_1 Nr. of seq. 1 Alignment length(with gaps) = 51
Alignment score = 0.000000
GACTTTAGAGNACATTNCTTCCTNATACTGGTAATTAAAGGAGCTAAGATT
>Bacillus_Fam_1399_51_1 Nr. of seq. 1 Alignment length(with gaps) = 51
Alignment score = 0.000000
GGATTTTGGAAGAAGGGAAAAGCATGCCCCCAAAGTGGCGTTAGGATACAT
>Bacillus_Fam_1400_51_1 Nr. of seq. 1 Alignment length(with gaps) = 51
Alignment score = 0.000000
TTCTATTACCTCTATCCTGCTTTTGCGATCACCCTTTTCGGCTTCGGGCTT
>Bacillus_Fam_1401_51_1 Nr. of seq. 1 Alignment length(with gaps) = 51
Alignment score = 0.000000
CCATCCGGAAGTGGTGGAAAGATTTCATCCCCTGCAAGTTCTACCCCAGTC
>Bacillus_Fam_1402_51_1 Nr. of seq. 1 Alignment length(with gaps) = 51
Alignment score = 0.000000
CATCCTTCACCATGATCACGGGCACTTATCTCCTCTTTGGTTACCTCTTTT
>Bacillus_Fam_1403_51_1 Nr. of seq. 1 Alignment length(with gaps) = 51
Alignment score = 0.000000
TTTGGGGAACCTATCTCGGCTTTGGTTACCTTTTCCTTGGCTTCGGNTCGA
>Bacillus_Fam_1404_51_1 Nr. of seq. 1 Alignment length(with gaps) = 51
Alignment score = 0.000000
TTCTTCAACAGCCTCTATTGCTTGTGGGACGGATTCCTCGGTCATTTCCAC
>Bacillus_Fam_1405_50_1 Nr. of seq. 1 Alignment length(with gaps) = 50
Alignment score = 0.000000
CGACTGCCACTATTTGGAAAACAAAATGCACACACACTCATTTACCGCCT
>Bacillus_Fam_1406_50_1 Nr. of seq. 1 Alignment length(with gaps) = 50
Alignment score = 0.000000
TTTAAAATCAAAGGAATAGATTGTGGTCGGGTNAAATCAAGTAGCTCACT
>Bacillus_Fam_1407_48_1 Nr. of seq. 1 Alignment length(with gaps) = 48
Alignment score = 0.000000
GCGATGATGCCGCAGATGCCGCATCAGATGCCACATCAGGTGCAGGGA
>Bacillus_Fam_1408_48_1 Nr. of seq. 1 Alignment length(with gaps) = 48
Alignment score = 0.000000
TCAGCCAGAAAACAACGGAAAAGGATATAAACAAGAAAATAGAGAGTC
>Bacillus_Fam_1409_48_1 Nr. of seq. 1 Alignment length(with gaps) = 48
Alignment score = 0.000000
GGGATGCCGCTAAATCCGGCTCCTGGTACCCCTCCATAACCTGCTGGT
>Bacillus_Fam_1410_48_1 Nr. of seq. 1 Alignment length(with gaps) = 48
Alignment score = 0.000000
AAACCAACAGAGTCAGCATCATTGAGCAATGCTGCATAATCATCTTGT
>Bacillus_Fam_1411_48_1 Nr. of seq. 1 Alignment length(with gaps) = 48
Alignment score = 0.000000
TCATTTGCTGTCCTGAATACATTGGAGAAGGTATGCTAGAATATGGCC
>Bacillus_Fam_1412_48_1 Nr. of seq. 1 Alignment length(with gaps) = 48
Alignment score = 0.000000
AGCCCGATGAGAAATTACTATAGAGCGATTGATCAGCACGTGACCGAT
>Bacillus_Fam_1413_48_1 Nr. of seq. 1 Alignment length(with gaps) = 48
Alignment score = 0.000000
GCTTCTTCTGTTGTTTCCTCTTCTTGGCCTTCTTCATCTACAGCATCC
>Bacillus_Fam_1414_48_1 Nr. of seq. 1 Alignment length(with gaps) = 48
Alignment score = 0.000000
GCAAAGAAAGAAGCCGAAGAACAAGCGAAACGAGAGGCTGAAGAACAG

>Bacillus_Fam_1415_48_1 Nr. of seq. 1 Alignment length(with gaps) = 48
Alignment score = 0.000000
GGTGTGATGGGAGCAGAGATGCATCATCAGATGCCGATGGGTCAGCCG
>Bacillus_Fam_1416_48_1 Nr. of seq. 1 Alignment length(with gaps) = 48
Alignment score = 0.000000
TTAGAAAGAAGCAGAGACAGAAGTTCGGAAAGCAGTGTGGACAATGAC
>Bacillus_Fam_1417_48_1 Nr. of seq. 1 Alignment length(with gaps) = 48
Alignment score = 0.000000
CGGCCTCCCCGCGAATCCTCACGTTTAGGTCCGCGGCTGTTTTCCATC
>Bacillus_Fam_1418_48_1 Nr. of seq. 1 Alignment length(with gaps) = 48
Alignment score = 0.000000
CATCATAAGAAGAGCTGCCGCAAGTCACATCGCAGCAAGCACAGATCG
>Bacillus_Fam_1419_48_1 Nr. of seq. 1 Alignment length(with gaps) = 48
Alignment score = 0.000000
ATGCCGTGGCAAAATGGGATGATGCCAATGCCTGGACAGCAGGAAGGA
>Bacillus_Fam_1420_48_1 Nr. of seq. 1 Alignment length(with gaps) = 48
Alignment score = 0.000000
TATAAGAAGGACTACTACTACCATGAGCCTTACAAGAAGCATCACGAG
>Bacillus_Fam_1421_48_1 Nr. of seq. 1 Alignment length(with gaps) = 48
Alignment score = 0.000000
CTGTTCCTTCATCCGAACCAGGGGCGGTTCCTTCTTCCGGCTCATCTC
>Bacillus_Fam_1422_47_1 Nr. of seq. 1 Alignment length(with gaps) = 47
Alignment score = 0.000000
AAACACGAGTAAGAATATGAATTATTCAGCTACCTTGATTACGTTTA
>Bacillus_Fam_1423_45_1 Nr. of seq. 1 Alignment length(with gaps) = 45
Alignment score = 0.000000
TTGTTGATTTCTCTTCTTTTTTACCTTTTTCATCAGCTGAACCGC
>Bacillus_Fam_1424_45_1 Nr. of seq. 1 Alignment length(with gaps) = 45
Alignment score = 0.000000
CACCATCAAGTACAACAGGTGGATCTCAATCAAGTGGTGGAGGAG
>Bacillus_Fam_1425_45_1 Nr. of seq. 1 Alignment length(with gaps) = 45
Alignment score = 0.000000
GCCCGAAGCATTAGGGGTCAGNGCAGAAGTGCCCAGAGCGTGAGA
>Bacillus_Fam_1426_45_1 Nr. of seq. 1 Alignment length(with gaps) = 45
Alignment score = 0.000000
CCGGAGTATTAACAGAAATAGAATCACACACATCGACATCGACAA
>Bacillus_Fam_1427_45_1 Nr. of seq. 1 Alignment length(with gaps) = 45
Alignment score = 0.000000
TCCTTGTGGTCCAGTGGGACCGATAGGCCCTGGTTCCCCTTGTGA
>Bacillus_Fam_1428_45_1 Nr. of seq. 1 Alignment length(with gaps) = 45
Alignment score = 0.000000
GAGCAATCTAGTAATTATTGACGGTATCAGCCGTTATCTGAACTT
>Bacillus_Fam_1429_45_1 Nr. of seq. 1 Alignment length(with gaps) = 45
Alignment score = 0.000000
TCTTGAGTTTGCTGAGATACGTGTTGATTTTGTTGACGTTGAGGT
>Bacillus_Fam_1430_45_1 Nr. of seq. 1 Alignment length(with gaps) = 45
Alignment score = 0.000000
GGAACAGAAAGACAAGGAATGGGTGAAGGAAATGCAGTTCCAGTA
>Bacillus_Fam_1431_45_1 Nr. of seq. 1 Alignment length(with gaps) = 45
Alignment score = 0.000000
TGTATCGGTGAAACGGGCATTTGCTTTTCTACTTTAGGAAATTCC
>Bacillus_Fam_1432_45_1 Nr. of seq. 1 Alignment length(with gaps) = 45
Alignment score = 0.000000
GATAAACCGGATGGCGAGAATCCAGATGTAGACAAGCCTGACACG

>Bacillus_Fam_1433_45_1 Nr. of seq. 1 Alignment length(with gaps) = 45
Alignment score = 0.000000
TCTGTGCCTTCCGTTCCTTCGGTTACCGCTTCTGCTCCTGTGTCG
>Bacillus_Fam_1434_45_1 Nr. of seq. 1 Alignment length(with gaps) = 45
Alignment score = 0.000000
TTTTCCTCTTTAGGAGGAGTTGGATTCGAAGGCTTNGGAGCCGGT
>Bacillus_Fam_1435_45_1 Nr. of seq. 1 Alignment length(with gaps) = 45
Alignment score = 0.000000
CTCATGAAGAAGATCACGACCACGATCACGACCATGAACACGATG
>Bacillus_Fam_1436_45_1 Nr. of seq. 1 Alignment length(with gaps) = 45
Alignment score = 0.000000
TCCTCTTCATCTATAAAAGAGCTCTCTTCTTGGGGAGAACTGGAT
>Bacillus_Fam_1437_45_1 Nr. of seq. 1 Alignment length(with gaps) = 45
Alignment score = 0.000000
CCATTATTTCCTTGCGAGCCTCTTCCGTTGTTCTCTTGCCCATTC
>Bacillus_Fam_1438_45_1 Nr. of seq. 1 Alignment length(with gaps) = 45
Alignment score = 0.000000
GCTCCGGTTACTCCGGTCGCTCCCGTATCTCCCGTTGCACCTGTG
>Bacillus_Fam_1439_43_1 Nr. of seq. 1 Alignment length(with gaps) = 43
Alignment score = 0.000000
AAAACCTTCTAACAAAGATCCAACCCCAACCCCTAGTTACCCA
>Bacillus_Fam_1440_43_1 Nr. of seq. 1 Alignment length(with gaps) = 43
Alignment score = 0.000000
GGCTGGTTAGATTCGGTTCTTGCTTCTTCGTANATCCAATGAA
>Bacillus_Fam_1441_43_1 Nr. of seq. 1 Alignment length(with gaps) = 43
Alignment score = 0.000000
TCAGTTTTAAGCTCGGAGACATCTGTTTTTAAGNTTAGAAACC
>Bacillus_Fam_1442_42_1 Nr. of seq. 1 Alignment length(with gaps) = 42
Alignment score = 0.000000
TGATCATCTTCCTCGTGACCATGCTCCTCATGGCTATGGTCG
>Bacillus_Fam_1443_42_1 Nr. of seq. 1 Alignment length(with gaps) = 42
Alignment score = 0.000000
AGGTTTTTGTGCTTCAGGTTTAGCTGTTTCGTTGTTTACAGC
>Bacillus_Fam_1444_42_1 Nr. of seq. 1 Alignment length(with gaps) = 42
Alignment score = 0.000000
AATCAAGGAAATCAAACGCCGCCAAATAGCGGAAACGGAAAT
>Bacillus_Fam_1445_42_1 Nr. of seq. 1 Alignment length(with gaps) = 42
Alignment score = 0.000000
TTCTCGTGCGGTTGCCGCTCCATCCTGTGCGGCTTTTCCCCT
>Bacillus_Fam_1446_42_1 Nr. of seq. 1 Alignment length(with gaps) = 42
Alignment score = 0.000000
TAACTTTGATCAAATCGCTCGTAACTCGGGCCGAATCGCGCG
>Bacillus_Fam_1447_42_1 Nr. of seq. 1 Alignment length(with gaps) = 42
Alignment score = 0.000000
TCTCTTTTCAGCTCATATTCTTCCGGTTCCTCTTCCACTTTC
>Bacillus_Fam_1448_42_1 Nr. of seq. 1 Alignment length(with gaps) = 42
Alignment score = 0.000000
CTGGACCTATGATGGGTGGGTTTAACCCTCAAGGACCTGGTG
>Bacillus_Fam_1449_42_1 Nr. of seq. 1 Alignment length(with gaps) = 42
Alignment score = 0.000000
CTTCTTCAGGAGTCTCTACTTCCTCTCCTGGTTCTTCTGTTC
>Bacillus_Fam_1450_42_1 Nr. of seq. 1 Alignment length(with gaps) = 42
Alignment score = 0.000000
CAGCCTTTTATCCATCGAGCTGACCTTTTGATCCAGAACGTC

>Bacillus_Fam_1451_42_1 Nr. of seq. 1 Alignment length(with gaps) = 42
Alignment score = 0.000000
ATTGTTAGAGCTGCGTCGATCATTCGAACAACGACGGCGACG
>Bacillus_Fam_1452_42_1 Nr. of seq. 1 Alignment length(with gaps) = 42
Alignment score = 0.000000
TTCGCTTTCGGGTTTGCTTTTGGGTCTACCTTCGAATTTCCG
>Bacillus_Fam_1453_42_1 Nr. of seq. 1 Alignment length(with gaps) = 42
Alignment score = 0.000000
TCAAGAATCGCGATGAAATGTCCGGTCGTACAATAGACGAGC
>Bacillus_Fam_1454_42_1 Nr. of seq. 1 Alignment length(with gaps) = 42
Alignment score = 0.000000
TGCGTGAAGTATTGCGGTTTCTTGAAGTANAGCGGCTCTGCT
>Bacillus_Fam_1455_42_1 Nr. of seq. 1 Alignment length(with gaps) = 42
Alignment score = 0.000000
CAACCAAGAGAAGTGGCCCCTGGAGAGGTACCTTCTGAAGCT
>Bacillus_Fam_1456_42_1 Nr. of seq. 1 Alignment length(with gaps) = 42
Alignment score = 0.000000
GATGATGGTGGCCATGCTCATGGTGATGATGCTCACGTTCAT
>Bacillus_Fam_1457_42_1 Nr. of seq. 1 Alignment length(with gaps) = 42
Alignment score = 0.000000
AAGCGCTTGTCGACCTCAACGAAACGTGCGTCCACTTCATCG
>Bacillus_Fam_1458_42_1 Nr. of seq. 1 Alignment length(with gaps) = 42
Alignment score = 0.000000
TCTCCAGAGTCATCATCGTTTCCGTCATCCGGATTCGAATCA
>Bacillus_Fam_1459_42_1 Nr. of seq. 1 Alignment length(with gaps) = 42
Alignment score = 0.000000
TTGTTGTTCTGGTTGTTATTTGGATCCTGATTGTTCCATTGG
>Bacillus_Fam_1460_42_1 Nr. of seq. 1 Alignment length(with gaps) = 42
Alignment score = 0.000000
CCGGCGGCGGGAACAACGGGGCTGACAATGGCGCCAACAACG
>Bacillus_Fam_1461_42_1 Nr. of seq. 1 Alignment length(with gaps) = 42
Alignment score = 0.000000
GGAGGATCTTCTCACGCGGAAGGATCGAACACAACAGCCAAT
>Bacillus_Fam_1462_42_1 Nr. of seq. 1 Alignment length(with gaps) = 42
Alignment score = 0.000000
CTGTTCAACAGCAATCAGCTCAACAAGAAAAAACTCAGCAGC
>Bacillus_Fam_1463_42_1 Nr. of seq. 1 Alignment length(with gaps) = 42
Alignment score = 0.000000
TCTGCTCTTCGACTTTTATAAATCTTTCATTCCTCTTATAAC
>Bacillus_Fam_1464_42_1 Nr. of seq. 1 Alignment length(with gaps) = 42
Alignment score = 0.000000
CGCTCAGCTTCTTGTTTAGCAGCAGCCTGTTCTTGAGCTTTT
>Bacillus_Fam_1465_42_1 Nr. of seq. 1 Alignment length(with gaps) = 42
Alignment score = 0.000000
CAGGTGAAGGAGCCGGAGAAGGCGAAGGCCAGGGCGGCGGAA
>Bacillus_Fam_1466_42_1 Nr. of seq. 1 Alignment length(with gaps) = 42
Alignment score = 0.000000
GGATTCGTACCACCTTGGTTATTTTCGTTTCCACCATTTGGT
>Bacillus_Fam_1467_42_1 Nr. of seq. 1 Alignment length(with gaps) = 42
Alignment score = 0.000000
AAGAACTTTCTTCCTGCCAAGAAAAAGATTCCTCTTGGGAAA
>Bacillus_Fam_1468_42_1 Nr. of seq. 1 Alignment length(with gaps) = 42
Alignment score = 0.000000
TGTTCAGTCTTGATCGCACCGATTTCTTTCCGCATTTCTGAC

>Bacillus_Fam_1469_42_1 Nr. of seq. 1 Alignment length(with gaps) = 42
Alignment score = 0.000000
TCAAACCGTTTATCCATGCTGTTCAGACGGCCATCCACATCA
>Bacillus_Fam_1470_42_1 Nr. of seq. 1 Alignment length(with gaps) = 42
Alignment score = 0.000000
AGCCACTTGTAAATTTATCGCTCCTTCTTTCACTGGAACGCG
>Bacillus_Fam_1471_42_1 Nr. of seq. 1 Alignment length(with gaps) = 42
Alignment score = 0.000000
GCTCTTCTTCTTCCCGCTCCCTCAGAATCGGGGCAGTCTCCG
>Bacillus_Fam_1472_42_1 Nr. of seq. 1 Alignment length(with gaps) = 42
Alignment score = 0.000000
GTCAAACGCAAAATGGTGGTAGCCAGCCAGGTGGAAGTGACG
>Bacillus_Fam_1473_42_1 Nr. of seq. 1 Alignment length(with gaps) = 42
Alignment score = 0.000000
GGAGGACCGGGTGGNTTTCCAGGCANTCCAGGTGGNTTCCCA
>Bacillus_Fam_1474_42_1 Nr. of seq. 1 Alignment length(with gaps) = 42
Alignment score = 0.000000
GATGTGGGAGGCCTAAAACAAAATGTCGAAATGCTAAAAGAC
>Bacillus_Fam_1475_42_1 Nr. of seq. 1 Alignment length(with gaps) = 42
Alignment score = 0.000000
CCTGCTACGTTTGGCTGTCCGTGATACGGATTTTCTGATGCG
>Bacillus_Fam_1476_42_1 Nr. of seq. 1 Alignment length(with gaps) = 42
Alignment score = 0.000000
GGAATTGCACCGATAACAACGATAATTGCACCGTTGACCTTG
>Bacillus_Fam_1477_42_1 Nr. of seq. 1 Alignment length(with gaps) = 42
Alignment score = 0.000000
GCTTTCGTTTGTGATGCGTAGAGTATCTCGAATCTTCCTCGT
>Bacillus_Fam_1478_42_1 Nr. of seq. 1 Alignment length(with gaps) = 42
Alignment score = 0.000000
CAGGAGTCGGACAATTAGTTGACGGACAAAAAACAGCGGTTG
>Bacillus_Fam_1479_42_1 Nr. of seq. 1 Alignment length(with gaps) = 42
Alignment score = 0.000000
GAGCTTCAATCCGACAAGAGGAGCGCTCATTCGGACAGGAGG
>Bacillus_Fam_1480_42_1 Nr. of seq. 1 Alignment length(with gaps) = 42
Alignment score = 0.000000
TGATCGGTAAATCCTCTGACATGAGCGATACCATCTCTCACT
>Bacillus_Fam_1481_42_1 Nr. of seq. 1 Alignment length(with gaps) = 42
Alignment score = 0.000000
ATTTCTGCTAGTGCGCCTTTGGTTTCTGCATGTAGATCTCCG
>Bacillus_Fam_1482_42_1 Nr. of seq. 1 Alignment length(with gaps) = 42
Alignment score = 0.000000
TTGAAGTCGACAAACGGTTTGATGCTGTTGATCAAAAATTTA
>Bacillus_Fam_1483_42_1 Nr. of seq. 1 Alignment length(with gaps) = 42
Alignment score = 0.000000
TTATTTAACAGGTAGCAGAATTAACATAACAGAGTTTCGATT
>Bacillus_Fam_1484_42_1 Nr. of seq. 1 Alignment length(with gaps) = 42
Alignment score = 0.000000
GTTGGCCGATATATCGATTGTGGTGGCGCAATAACATGCTCA
>Bacillus_Fam_1485_42_1 Nr. of seq. 1 Alignment length(with gaps) = 42
Alignment score = 0.000000
AAGTGCGTCAATTACTTATCCAGCTGCGTCAAAAAGTTCGCC
>Bacillus_Fam_1486_42_1 Nr. of seq. 1 Alignment length(with gaps) = 42
Alignment score = 0.000000
TTTGATAAAATTGAAACTCGACTTAATAATATGGATCAGCGG

>Bacillus_Fam_1487_42_1 Nr. of seq. 1 Alignment length(with gaps) = 42
Alignment score = 0.000000
AGAAACGCCAGGAGATAACGAAGAAACTCCTGGAGATAATGA
>Bacillus_Fam_1488_42_1 Nr. of seq. 1 Alignment length(with gaps) = 42
Alignment score = 0.000000
GCGAATTTCCGNGCCCAATGNGCGAATTATCGNACCTTACTN
>Bacillus_Fam_1489_42_1 Nr. of seq. 1 Alignment length(with gaps) = 42
Alignment score = 0.000000
GTGATCATTCGGCCGGCGGTTTGAAAATCATTGGTTGATGAC
>Bacillus_Fam_1490_42_1 Nr. of seq. 1 Alignment length(with gaps) = 42
Alignment score = 0.000000
TATTCGCCGTTCTNCTCNATTTATTGGACATTANCNGCACTT
>Bacillus_Fam_1491_42_1 Nr. of seq. 1 Alignment length(with gaps) = 42
Alignment score = 0.000000
ACATATCTATGCTGATAAAAGGCAATTACCCCCTTTAAGTAG
>Bacillus_Fam_1492_42_1 Nr. of seq. 1 Alignment length(with gaps) = 42
Alignment score = 0.000000
CTCGCCAATTCGGCACTTTTACTCGCGAGTTTNAGCTGTTTA
>Bacillus_Fam_1493_42_1 Nr. of seq. 1 Alignment length(with gaps) = 42
Alignment score = 0.000000
TTTTTCGGCCTGCACTCAACTTAATTCGACCTGCATCCACTT
>Bacillus_Fam_1494_42_1 Nr. of seq. 1 Alignment length(with gaps) = 42
Alignment score = 0.000000
GTTGCGCAATANGGTANTGTTTTAGCGCAATTCCGTGTGNAA
>Bacillus_Fam_1495_42_1 Nr. of seq. 1 Alignment length(with gaps) = 42
Alignment score = 0.000000
TCGTAGCATTTTTTCTTCTTACAGCAAAAATCTACGTGATCG
>Bacillus_Fam_1496_42_1 Nr. of seq. 1 Alignment length(with gaps) = 42
Alignment score = 0.000000
TTTGGTCCCATATATGCTGGCATTGTTTGTGGGAAGTTTGCA
>Bacillus_Fam_1497_42_1 Nr. of seq. 1 Alignment length(with gaps) = 42
Alignment score = 0.000000
GAAGTAGAAGATCTAAAAGCAGGTCAAACGAAGTTAACAACG
>Bacillus_Fam_1498_42_1 Nr. of seq. 1 Alignment length(with gaps) = 42
Alignment score = 0.000000
TTCTTCTTCAGCAACGGTGTTAACTTCATCCTCAGGAAGGTA
>Bacillus_Fam_1499_42_1 Nr. of seq. 1 Alignment length(with gaps) = 42
Alignment score = 0.000000
TACTTACTTGTTGACCATTCTCCACTCTTTCCAAACGTTCGT
>Bacillus_Fam_1500_42_1 Nr. of seq. 1 Alignment length(with gaps) = 42
Alignment score = 0.000000
AGTTCTTTCGTGTCTGCTTTTAATAGCCGGACGTCTCCTTTT
>Bacillus_Fam_1501_42_1 Nr. of seq. 1 Alignment length(with gaps) = 42
Alignment score = 0.000000
GTCACTTACATTGGTTTTCAATTCACCAACATCGGTTGTCAG
>Bacillus_Fam_1502_42_1 Nr. of seq. 1 Alignment length(with gaps) = 42
Alignment score = 0.000000
TTTACTCCGATCCTCTTATACTTTACTAACAACCACCTATAC
>Bacillus_Fam_1503_42_1 Nr. of seq. 1 Alignment length(with gaps) = 42
Alignment score = 0.000000
GCTACTGAAGACACTCATTCTCATGACGATGAAGAGAGTCAT
>Bacillus_Fam_1504_42_1 Nr. of seq. 1 Alignment length(with gaps) = 42
Alignment score = 0.000000
CGGAAGACGGGGAGAACGCCGAAGGAGAAGCGCCAAGTCCGC

>Bacillus_Fam_1505_42_1 Nr. of seq. 1 Alignment length(with gaps) = 42
Alignment score = 0.000000
AGAGCAGTGCGACAGCGCTGCGTCGTGCGAAGCACAAAAAAG
>Bacillus_Fam_1506_42_1 Nr. of seq. 1 Alignment length(with gaps) = 42
Alignment score = 0.000000
GCCGAATCCTGGAGTCGCATACGGAGAAACTGGACGAACACG
>Bacillus_Fam_1507_42_1 Nr. of seq. 1 Alignment length(with gaps) = 42
Alignment score = 0.000000
ATCAGCCAACTTCGGGCGAGAATCAACCAAGCTTGGCGAAAA
>Bacillus_Fam_1508_42_1 Nr. of seq. 1 Alignment length(with gaps) = 42
Alignment score = 0.000000
GAAAAGCGTTTTGACGGTTTAGAAGGGCGTATTGATGGGCTA
>Bacillus_Fam_1509_41_1 Nr. of seq. 1 Alignment length(with gaps) = 41
Alignment score = 0.000000
TTTGCGTCTTCTGATGAGNGATTTGCACCGGTTTGGTGGCA
>Bacillus_Fam_1510_41_1 Nr. of seq. 1 Alignment length(with gaps) = 41
Alignment score = 0.000000
AATATTGGAATTTGCTTCTGAAAATTTCAGGGTTAGTTCCC
>Bacillus_Fam_1511_41_1 Nr. of seq. 1 Alignment length(with gaps) = 41
Alignment score = 0.000000
TATACGAGAGGTGGAGCTGCTATAAGTGAGTGGCACCTGTC
>Bacillus_Fam_1512_41_1 Nr. of seq. 1 Alignment length(with gaps) = 41
Alignment score = 0.000000
GATTTATCAGCCAAAAACTCACTCTTTTCAGCCAAACTTCC
>Bacillus_Fam_1513_41_1 Nr. of seq. 1 Alignment length(with gaps) = 41
Alignment score = 0.000000
TCGATCGATAAATCTCAAAGTCGATGAATAAACCAGGCAAC
>Bacillus_Fam_1514_41_1 Nr. of seq. 1 Alignment length(with gaps) = 41
Alignment score = 0.000000
TTTATCAATCACTTTCCCCTCGTTATCCATCACTTTCACAA
>Bacillus_Fam_1515_41_1 Nr. of seq. 1 Alignment length(with gaps) = 41
Alignment score = 0.000000
TGGACAATTCGGCGGGATTGTGACCAAGCGGCGGCCAAGTC
>Bacillus_Fam_1516_41_1 Nr. of seq. 1 Alignment length(with gaps) = 41
Alignment score = 0.000000
TTGCGGGCCGGACCTAACCCTTTACATGCCGATTTCTTATA
>Bacillus_Fam_1517_41_1 Nr. of seq. 1 Alignment length(with gaps) = 41
Alignment score = 0.000000
CACTTTTCACACCAAAATCGAAGTTTGCGGGCCGGTTCAAA
>Bacillus_Fam_1518_41_1 Nr. of seq. 1 Alignment length(with gaps) = 41
Alignment score = 0.000000
AAAAAGTGACCGCATTATCACCTGTTTTGACTGAATAAACG
>Bacillus_Fam_1519_41_1 Nr. of seq. 1 Alignment length(with gaps) = 41
Alignment score = 0.000000
AATTCGTCCTACATGGNAANAATTCGACCGTAACCGGCAAN
>Bacillus_Fam_1520_41_1 Nr. of seq. 1 Alignment length(with gaps) = 41
Alignment score = 0.000000
CTGAAGGTCGATTTATTCCGACCCACGGACGAATTCATTCA
>Bacillus_Fam_1521_41_1 Nr. of seq. 1 Alignment length(with gaps) = 41
Alignment score = 0.000000
GAGACGCAAAAGTAGTTGCAGCTTACGCAAAAAACAGTGGA
>Bacillus_Fam_1522_41_1 Nr. of seq. 1 Alignment length(with gaps) = 41
Alignment score = 0.000000
TAAATATTTGCGTCAGCCTCAGTGATTTAAGCGTCACCATT

>Bacillus_Fam_1523_41_1 Nr. of seq. 1 Alignment length(with gaps) = 41
Alignment score = 0.000000
 TGCATATATTCGCCGTCCTCAAGCAGATATTGGACAAAACC
>Bacillus_Fam_1524_41_1 Nr. of seq. 1 Alignment length(with gaps) = 41
Alignment score = 0.000000
 AGGGAAGCAGGACGAATAAATTCGCGGACGGACGGAAAAAG
>Bacillus_Fam_1525_41_1 Nr. of seq. 1 Alignment length(with gaps) = 41
Alignment score = 0.000000
 GGACGAATTCTCTCGACGGTAGGTCGAATTAAACCTGTTTG
>Bacillus_Fam_1526_41_1 Nr. of seq. 1 Alignment length(with gaps) = 41
Alignment score = 0.000000
 GGACGGATTATTTCATCCCAGGTCGGATTCCACGCGCTGTC
>Bacillus_Fam_1527_41_1 Nr. of seq. 1 Alignment length(with gaps) = 41
Alignment score = 0.000000
 TTCGACCTAAACTCCATTTTTTCCGGCCCACTATCGATTAA
>Bacillus_Fam_1528_41_1 Nr. of seq. 1 Alignment length(with gaps) = 41
Alignment score = 0.000000
 GATATAATTCCAGTTTCGGCGATATATTGCCGGTTTTCGTA
>Bacillus_Fam_1529_41_1 Nr. of seq. 1 Alignment length(with gaps) = 41
Alignment score = 0.000000
 ATATTCCGGCCTTACCCGCTGGTTTCCGGCCTGCTTCATCG
>Bacillus_Fam_1530_41_1 Nr. of seq. 1 Alignment length(with gaps) = 41
Alignment score = 0.000000
 ATTGGGTCGATTTATCTCCTGTTAGGACGATTTATCCCCGC
>Bacillus_Fam_1531_41_1 Nr. of seq. 1 Alignment length(with gaps) = 41
Alignment score = 0.000000
 GTAAGGAAGTCGCGCAATCCCCAGGAAAGAAGCGCAATGCA
>Bacillus_Fam_1532_41_1 Nr. of seq. 1 Alignment length(with gaps) = 41
Alignment score = 0.000000
 AAAAGTAGCGGATAAAACCCAAAAGTTGTTGGATAAATCCC
>Bacillus_Fam_1533_41_1 Nr. of seq. 1 Alignment length(with gaps) = 41
Alignment score = 0.000000
 TTCTCGTGCTTCAAATGGAAGCATGAGAACCGTCCCACTGC
>Bacillus_Fam_1534_40_1 Nr. of seq. 1 Alignment length(with gaps) = 40
Alignment score = 0.000000
 CATGAAGAAGACGAACATGAGCATCACGAGCATGAGGGGC
>Bacillus_Fam_1535_40_1 Nr. of seq. 1 Alignment length(with gaps) = 40
Alignment score = 0.000000
 GATTTGCGTCTGCATTTCGTTTTTTGCGTCGCACCCGGCA
>Bacillus_Fam_1536_40_1 Nr. of seq. 1 Alignment length(with gaps) = 40
Alignment score = 0.000000
 ATCCACTTATTTTAACTTTGTCCGAGTTATTGGCAGATTG
>Bacillus_Fam_1537_40_1 Nr. of seq. 1 Alignment length(with gaps) = 40
Alignment score = 0.000000
 ACGCATAAATCCTGGAACCGATGCAAATAACTTGGATCCG
>Bacillus_Fam_1538_40_1 Nr. of seq. 1 Alignment length(with gaps) = 40
Alignment score = 0.000000
 ATTTTTAATTGCGATTACACGAATTTATTTGCCATTTCCC
>Bacillus_Fam_1539_39_1 Nr. of seq. 1 Alignment length(with gaps) = 39
Alignment score = 0.000000
 TTATCTTTAGATTTCCACCAATCGTCATCACAATTGTGC
>Bacillus_Fam_1540_39_1 Nr. of seq. 1 Alignment length(with gaps) = 39
Alignment score = 0.000000
 TCTTCTTTTGCCGCTTGCTCCTCAGCTTTTCGTTTTGCT

>Bacillus_Fam_1541_39_1 Nr. of seq. 1 Alignment length(with gaps) = 39
Alignment score = 0.000000
TTCATGCCTTCCATATCATGAGAAGTGTGCTCGTCCATA
>Bacillus_Fam_1542_39_1 Nr. of seq. 1 Alignment length(with gaps) = 39
Alignment score = 0.000000
CGCCTGGATTGCCCGGATCTTCAGGATCCGGCTCCTCTC
>Bacillus_Fam_1543_39_1 Nr. of seq. 1 Alignment length(with gaps) = 39
Alignment score = 0.000000
TCTGGTAATTTGTTAGTTGCTTCCTCTTCATGTGCAAGC
>Bacillus_Fam_1544_39_1 Nr. of seq. 1 Alignment length(with gaps) = 39
Alignment score = 0.000000
GCGCTTTTCTTATGCTTGTCGCCGATAAACAGGCGCCTT
>Bacillus_Fam_1545_39_1 Nr. of seq. 1 Alignment length(with gaps) = 39
Alignment score = 0.000000
TTACCATTATCAGAGCCACTGCCATTGTTCGTGTTGTTA
>Bacillus_Fam_1546_39_1 Nr. of seq. 1 Alignment length(with gaps) = 39
Alignment score = 0.000000
CTTTTCCTGAAGTCTACGGATCCTGTGTAGTATGGTGCG
>Bacillus_Fam_1547_39_1 Nr. of seq. 1 Alignment length(with gaps) = 39
Alignment score = 0.000000
GCTGGTTGCTGCTGCATGCCACCCATCGGTTGTTGGTAC
>Bacillus_Fam_1548_39_1 Nr. of seq. 1 Alignment length(with gaps) = 39
Alignment score = 0.000000
CTCCGTATCCAGGCATTGCTCCATAGCCCGCTGGGGCTG
>Bacillus_Fam_1549_39_1 Nr. of seq. 1 Alignment length(with gaps) = 39
Alignment score = 0.000000
AAACCTGGCTGAGCGCCATAAGGAACTCCTGCTGCGGCG
>Bacillus_Fam_1550_39_1 Nr. of seq. 1 Alignment length(with gaps) = 39
Alignment score = 0.000000
CAAAGTCTCATAGCATGCTGATCATTGACTACATCAATG
>Bacillus_Fam_1551_39_1 Nr. of seq. 1 Alignment length(with gaps) = 39
Alignment score = 0.000000
TTCACCTACTCTTTGCTTCATTGCAGTTAAGTCCGTCAA
>Bacillus_Fam_1552_39_1 Nr. of seq. 1 Alignment length(with gaps) = 39
Alignment score = 0.000000
CTCAAGCAAATCAGTCAATGATGCCAATGAATATAGGGC
>Bacillus_Fam_1553_39_1 Nr. of seq. 1 Alignment length(with gaps) = 39
Alignment score = 0.000000
AGGATATTGACCAGGGCCTCCTCCTTGGGGCGGCAGTCC
>Bacillus_Fam_1554_39_1 Nr. of seq. 1 Alignment length(with gaps) = 39
Alignment score = 0.000000
GGAATGGGAACAGGAGGCTATCCCGGCGGAGGATTTCCA
>Bacillus_Fam_1555_39_1 Nr. of seq. 1 Alignment length(with gaps) = 39
Alignment score = 0.000000
GGGGGCCTGTCCCCCGCTGTTTTAACGCATTACTCCGCC
>Bacillus_Fam_1556_39_1 Nr. of seq. 1 Alignment length(with gaps) = 39
Alignment score = 0.000000
AGAATCAACAGAAGAAATTCAAGCAGTAGCAGAGGAAGT
>Bacillus_Fam_1557_39_1 Nr. of seq. 1 Alignment length(with gaps) = 39
Alignment score = 0.000000
TCTTTCTTCTCTTCAGAAGAACCACTTGTTGATTTCTCT
>Bacillus_Fam_1558_39_1 Nr. of seq. 1 Alignment length(with gaps) = 39
Alignment score = 0.000000
TTTTCTTCAGGAGCTACAGTTTCGTTACCTGTTTCAGGT

>Bacillus_Fam_1559_39_1 Nr. of seq. 1 Alignment length(with gaps) = 39
Alignment score = 0.000000
GCAGGTAAAGGTGGCGCAGGAGCAGGCGAAGGTCCAGGA
>Bacillus_Fam_1560_39_1 Nr. of seq. 1 Alignment length(with gaps) = 39
Alignment score = 0.000000
CAAAACCACCAGTAGACCCACCAACAGATCCAGGAGATA
>Bacillus_Fam_1561_39_1 Nr. of seq. 1 Alignment length(with gaps) = 39
Alignment score = 0.000000
TCTGCTGCGGGAACGGTGTGTCCGGTGATGCGTCCGTCT
>Bacillus_Fam_1562_39_1 Nr. of seq. 1 Alignment length(with gaps) = 39
Alignment score = 0.000000
GGCATATTTGGCATCGGTTCATTCGGCATTGGCATGTTC
>Bacillus_Fam_1563_39_1 Nr. of seq. 1 Alignment length(with gaps) = 39
Alignment score = 0.000000
CAAAGATAGAAGAGAAGAAAACTGAAGATAAAGAAACAG
>Bacillus_Fam_1564_39_1 Nr. of seq. 1 Alignment length(with gaps) = 39
Alignment score = 0.000000
CCTTGACCTCCCGGCATCATCGGAAATCCTCCAGGCTGA
>Bacillus_Fam_1565_39_1 Nr. of seq. 1 Alignment length(with gaps) = 39
Alignment score = 0.000000
AGATCTGGTCGTTCTCGTAGATCCCGCCGTTCTGGAGGC
>Bacillus_Fam_1566_39_1 Nr. of seq. 1 Alignment length(with gaps) = 39
Alignment score = 0.000000
CCTGGCATCATTGGCTGGCCGCTCATTCCTTGACCCATT
>Bacillus_Fam_1567_39_1 Nr. of seq. 1 Alignment length(with gaps) = 39
Alignment score = 0.000000
TGATCATGACTATGACTGTGACCTTCGTGACTGTGTTCG
>Bacillus_Fam_1568_39_1 Nr. of seq. 1 Alignment length(with gaps) = 39
Alignment score = 0.000000
GGATGGGGAAGCGGATTCCCACAAACACCAGGCCAAAGC
>Bacillus_Fam_1569_39_1 Nr. of seq. 1 Alignment length(with gaps) = 39
Alignment score = 0.000000
CAAATAAAATGAGAGTTTCGCAAAAATAAAGCGTTCTTG
>Bacillus_Fam_1570_39_1 Nr. of seq. 1 Alignment length(with gaps) = 39
Alignment score = 0.000000
AAGCTGTAGAGAAGCAGGAACCGGCCAAGGAAGCAGCAC
>Bacillus_Fam_1571_39_1 Nr. of seq. 1 Alignment length(with gaps) = 39
Alignment score = 0.000000
TGCATTGGCATTTGACCTTGATCTGGCATATATGGTTGC
>Bacillus_Fam_1572_39_1 Nr. of seq. 1 Alignment length(with gaps) = 39
Alignment score = 0.000000
TTCTGCGAGAACGGCATGATCTGCGCGTGCCTCTCGTGC
>Bacillus_Fam_1573_39_1 Nr. of seq. 1 Alignment length(with gaps) = 39
Alignment score = 0.000000
TTTGTGTTTGCGTTTGAGCTGGTTGCTTTTCTACCGGCT
>Bacillus_Fam_1574_39_1 Nr. of seq. 1 Alignment length(with gaps) = 39
Alignment score = 0.000000
GCTTTAGAGTTGAATCTGTATCAGAATTATTTGAATCGT
>Bacillus_Fam_1575_39_1 Nr. of seq. 1 Alignment length(with gaps) = 39
Alignment score = 0.000000
CAACAACTGGTGAGACAACTGGAGGAACAACCGGCAGCA
>Bacillus_Fam_1576_39_1 Nr. of seq. 1 Alignment length(with gaps) = 39
Alignment score = 0.000000
TGTGGTGCCATTTGATGCGGTTGATACGGCATCGCCTGC

>Bacillus_Fam_1577_39_1 Nr. of seq. 1 Alignment length(with gaps) = 39
Alignment score = 0.000000
CATGTTCTTCTTCATGAGCAGCCTCAGTTTCATCATGTG
>Bacillus_Fam_1578_39_1 Nr. of seq. 1 Alignment length(with gaps) = 39
Alignment score = 0.000000
AAAACAACAACAAGAGCAGCAACAGCAGCAGGCCCAACA
>Bacillus_Fam_1579_39_1 Nr. of seq. 1 Alignment length(with gaps) = 39
Alignment score = 0.000000
TCTTCTTCCTCATGGGCATGTTCATCGTCAGCGTGNTGC
>Bacillus_Fam_1580_39_1 Nr. of seq. 1 Alignment length(with gaps) = 39
Alignment score = 0.000000
GCTTTCTTCCATTCTCGGTTCCGCTTTTGCGTCCCTGCT
>Bacillus_Fam_1581_39_1 Nr. of seq. 1 Alignment length(with gaps) = 39
Alignment score = 0.000000
CACGGCTGGCCGGGTCAAGGCGGGTCTGGCTCCGGCGGG
>Bacillus_Fam_1582_39_1 Nr. of seq. 1 Alignment length(with gaps) = 39
Alignment score = 0.000000
CCCTGCATATGATGTCCGTGTCCCATATCCGGCATGTTT
>Bacillus_Fam_1583_39_1 Nr. of seq. 1 Alignment length(with gaps) = 39
Alignment score = 0.000000
CGTTCGGCTTCTTCCGCAGCAATGCGCTCTTCCTCCTCA
>Bacillus_Fam_1584_39_1 Nr. of seq. 1 Alignment length(with gaps) = 39
Alignment score = 0.000000
TATCAACCAGCACCGTTTCCACAAGGTGGTCAAATGGGA
>Bacillus_Fam_1585_38_1 Nr. of seq. 1 Alignment length(with gaps) = 38
Alignment score = 0.000000
TTTGCTTCGTAGTCTTCCCTGTTTCTTCCTCTGCCAGC
>Bacillus_Fam_1586_37_1 Nr. of seq. 1 Alignment length(with gaps) = 37
Alignment score = 0.000000
GAAATCGGCCTTATTGCACGTTATCGGGCTTGTTGCA
>Bacillus_Fam_1587_36_1 Nr. of seq. 1 Alignment length(with gaps) = 36
Alignment score = 0.000000
AAAACCTGATACGGGAGGAAATCCAGACACAGGAGG
>Bacillus_Fam_1588_36_1 Nr. of seq. 1 Alignment length(with gaps) = 36
Alignment score = 0.000000
ATGGATCAAAACGTGCATCCGCAGCAATGGCCGCAA
>Bacillus_Fam_1589_36_1 Nr. of seq. 1 Alignment length(with gaps) = 36
Alignment score = 0.000000
TTTTGTGAGAACGTGATTTTTTATGGCTGCGGGATT
>Bacillus_Fam_1590_36_1 Nr. of seq. 1 Alignment length(with gaps) = 36
Alignment score = 0.000000
ATGCAGGGTATGCAGGGTAGAGCTGGCCTGGGGGGT
>Bacillus_Fam_1591_36_1 Nr. of seq. 1 Alignment length(with gaps) = 36
Alignment score = 0.000000
ACAGGTGATACCGGGGCNACCGGAGTAACNGGAGCA
>Bacillus_Fam_1592_36_1 Nr. of seq. 1 Alignment length(with gaps) = 36
Alignment score = 0.000000
AAAAGGGAATTGGAACTGCAGTCAGAACAACAGAGA
>Bacillus_Fam_1593_36_1 Nr. of seq. 1 Alignment length(with gaps) = 36
Alignment score = 0.000000
CTTCTGGTTTATTGTTATCAACAACTGGTTGATCTG
>Bacillus_Fam_1594_36_1 Nr. of seq. 1 Alignment length(with gaps) = 36
Alignment score = 0.000000
TCGCTTTGATTTTGGTTTTGGGACAAGCCCTCGTCC

>Bacillus_Fam_1595_36_1 Nr. of seq. 1 Alignment length(with gaps) = 36
Alignment score = 0.000000
TTTTCATCCAAAGGGCAGCCGGAAAAGCAAACCAGG
>Bacillus_Fam_1596_36_1 Nr. of seq. 1 Alignment length(with gaps) = 36
Alignment score = 0.000000
CAGACATATCAACCAAATCAAATGACGATGCCACAA
>Bacillus_Fam_1597_36_1 Nr. of seq. 1 Alignment length(with gaps) = 36
Alignment score = 0.000000
CTATCTCTTCAGTTACTTCGACTTCTGGAGTTACAG
>Bacillus_Fam_1598_36_1 Nr. of seq. 1 Alignment length(with gaps) = 36
Alignment score = 0.000000
CCGCTTCCTGGAGCATCTGGTTGATTTCCTTCTTGG
>Bacillus_Fam_1599_36_1 Nr. of seq. 1 Alignment length(with gaps) = 36
Alignment score = 0.000000
CTTCTGCAACTGGTTGTTCTTCAGCGACTGGCCCTT
>Bacillus_Fam_1600_36_1 Nr. of seq. 1 Alignment length(with gaps) = 36
Alignment score = 0.000000
GGGCGGATTCCCTGGTCANGGTGGNCATGGNGGAAT
>Bacillus_Fam_1601_36_1 Nr. of seq. 1 Alignment length(with gaps) = 36
Alignment score = 0.000000
TCCAAAGGCAGTGGTGGATCGAAAGGAAGTCATGGC
>Bacillus_Fam_1602_36_1 Nr. of seq. 1 Alignment length(with gaps) = 36
Alignment score = 0.000000
CTGTTATTTCCGCCACTTCGACTACGTCATCTACAC
>Bacillus_Fam_1603_36_1 Nr. of seq. 1 Alignment length(with gaps) = 36
Alignment score = 0.000000
GTGTCATCCCCAGTACCTGGACTTCCTCCATTATCC
>Bacillus_Fam_1604_36_1 Nr. of seq. 1 Alignment length(with gaps) = 36
Alignment score = 0.000000
GTTTCTTCCTCTACTGCTTCTTCCGGCGCTTCTGAA
>Bacillus_Fam_1605_36_1 Nr. of seq. 1 Alignment length(with gaps) = 36
Alignment score = 0.000000
ACTAGCTCTTCTTCAAATGCTTCCTCTGTTTCCGCA
>Bacillus_Fam_1606_36_1 Nr. of seq. 1 Alignment length(with gaps) = 36
Alignment score = 0.000000
GCTTCAAGAAACACCATTGTGACTGCTGTGACAAGT
>Bacillus_Fam_1607_36_1 Nr. of seq. 1 Alignment length(with gaps) = 36
Alignment score = 0.000000
TGCAGCAGCAGGAAGCACGGAAGCATGAGGAGACGT
>Bacillus_Fam_1608_36_1 Nr. of seq. 1 Alignment length(with gaps) = 36
Alignment score = 0.000000
ACAAGCATGATGACTGTAATAAACGAGACAGAAACG
>Bacillus_Fam_1609_36_1 Nr. of seq. 1 Alignment length(with gaps) = 36
Alignment score = 0.000000
CGATGCATGCTCAGCCAATGATGTACCCAGACAATG
>Bacillus_Fam_1610_36_1 Nr. of seq. 1 Alignment length(with gaps) = 36
Alignment score = 0.000000
CGAAGAAACCAGACGGATACAAAAAGGATGACGATT
>Bacillus_Fam_1611_36_1 Nr. of seq. 1 Alignment length(with gaps) = 36
Alignment score = 0.000000
TCTTCTTCGGAGTGAGCGGCTTCTTCGTCATGGTGC
>Bacillus_Fam_1612_36_1 Nr. of seq. 1 Alignment length(with gaps) = 36
Alignment score = 0.000000
GAACCGAAAACAGAAAAGCCAGATGGTAAACCAGAA

>Bacillus_Fam_1613_36_1 Nr. of seq. 1 Alignment length(with gaps) = 36
Alignment score = 0.000000
GTAGGTCCAGTCGGTCCAAGGTCGCCGGTAGCACCT
>Bacillus_Fam_1614_36_1 Nr. of seq. 1 Alignment length(with gaps) = 36
Alignment score = 0.000000
TTTTCCTCCGGCTCCTGTTCCAGGACGAAATCCGGC
>Bacillus_Fam_1615_36_1 Nr. of seq. 1 Alignment length(with gaps) = 36
Alignment score = 0.000000
CCGCAAATGGGCTATGGCCCGCAAGCTGGATTCGCC
>Bacillus_Fam_1616_36_1 Nr. of seq. 1 Alignment length(with gaps) = 36
Alignment score = 0.000000
GGAGGAGGTCAACCGCCGCCGCGACCACCACAAGGG
>Bacillus_Fam_1617_36_1 Nr. of seq. 1 Alignment length(with gaps) = 36
Alignment score = 0.000000
GAAGATGATCAATCGGACACCGATGACACAGAAGGG
>Bacillus_Fam_1618_36_1 Nr. of seq. 1 Alignment length(with gaps) = 36
Alignment score = 0.000000
CAATGGGACGGACATGGCGGAGGTCATGGTGGCGGT
>Bacillus_Fam_1619_36_1 Nr. of seq. 1 Alignment length(with gaps) = 36
Alignment score = 0.000000
TTTCCTCGCCTCCGTCATTTTCTTCCGGAGACTCTG
>Bacillus_Fam_1620_36_1 Nr. of seq. 1 Alignment length(with gaps) = 36
Alignment score = 0.000000
TCTTCGCCTTCGACTGGTTCTTCTCCCTCAACTGGC
>Bacillus_Fam_1621_36_1 Nr. of seq. 1 Alignment length(with gaps) = 36
Alignment score = 0.000000
TGTTGGTGCTTTTGNGCTTGTCCCAGGTTTCGATAC
>Bacillus_Fam_1622_36_1 Nr. of seq. 1 Alignment length(with gaps) = 36
Alignment score = 0.000000
AGAGAAAATAAACAAGAACAAGCAAACCAACAGGCA
>Bacillus_Fam_1623_36_1 Nr. of seq. 1 Alignment length(with gaps) = 36
Alignment score = 0.000000
TTCTTCTGTTACAACTTCAGTAGNTTCGTTAGTTGC
>Bacillus_Fam_1624_36_1 Nr. of seq. 1 Alignment length(with gaps) = 36
Alignment score = 0.000000
GGTTCTTTAATTGGTTCTTCACTAGGTTGTTTAGCT
>Bacillus_Fam_1625_36_1 Nr. of seq. 1 Alignment length(with gaps) = 36
Alignment score = 0.000000
TTAGCGGCATTTCCGCCATTGGCTGCGTCGCCTCCG
>Bacillus_Fam_1626_36_1 Nr. of seq. 1 Alignment length(with gaps) = 36
Alignment score = 0.000000
ATCCGGGACATCCAGGGTATGGCGGCATGACTGGTT
>Bacillus_Fam_1627_36_1 Nr. of seq. 1 Alignment length(with gaps) = 36
Alignment score = 0.000000
GTTACTCCCGGTGATCCCGTTAAACCAGTTGCACCT
>Bacillus_Fam_1628_36_1 Nr. of seq. 1 Alignment length(with gaps) = 36
Alignment score = 0.000000
TTTCCTGTTGATACTGGTGATACTTGCATATTTGGT
>Bacillus_Fam_1629_36_1 Nr. of seq. 1 Alignment length(with gaps) = 36
Alignment score = 0.000000
CAGGAGCACCGGGAGCAGCAGCTGGAACTCCATATC
>Bacillus_Fam_1630_36_1 Nr. of seq. 1 Alignment length(with gaps) = 36
Alignment score = 0.000000
AGGAGAAACAGGGGCGACTGGTAGTCAAGGAGTTCA

>Bacillus_Fam_1631_36_1 Nr. of seq. 1 Alignment length(with gaps) = 36
Alignment score = 0.000000
 GGCATCATTCCCGGCATGCCTCCTTGCATTTGTCCC
>Bacillus_Fam_1632_36_1 Nr. of seq. 1 Alignment length(with gaps) = 36
Alignment score = 0.000000
 CAGGTCCAAAAGGAAAAGAAGGCCCACCCGGTCCAG
>Bacillus_Fam_1633_36_1 Nr. of seq. 1 Alignment length(with gaps) = 36
Alignment score = 0.000000
 CTCCTGGTACTGGATTTAATCCTGGCATGGGTTATG
>Bacillus_Fam_1634_34_1 Nr. of seq. 1 Alignment length(with gaps) = 34
Alignment score = 0.000000
 AACGTTTGATTGACTTCAGCGGTTCTACTAACTA
>Bacillus_Fam_1635_34_1 Nr. of seq. 1 Alignment length(with gaps) = 34
Alignment score = 0.000000
 GGGGGTCAGGCACGCTTTAATGATTCACCGCACT
>Bacillus_Fam_1636_33_1 Nr. of seq. 1 Alignment length(with gaps) = 33
Alignment score = 0.000000
 GGAATGATGCCAATGCCAGGTCAAAACGGAAAT
>Bacillus_Fam_1637_33_1 Nr. of seq. 1 Alignment length(with gaps) = 33
Alignment score = 0.000000
 GAACAAGAGCCTGAAACTGACGCTAGCACAGAC
>Bacillus_Fam_1638_33_1 Nr. of seq. 1 Alignment length(with gaps) = 33
Alignment score = 0.000000
 AACAAATCCGCCGTCAACAGGAGGAAATGAATC
>Bacillus_Fam_1639_33_1 Nr. of seq. 1 Alignment length(with gaps) = 33
Alignment score = 0.000000
 GGCATGCCTCCACTTGGTTGACCTCCCAATCCC
>Bacillus_Fam_1640_33_1 Nr. of seq. 1 Alignment length(with gaps) = 33
Alignment score = 0.000000
 TAGAGCATAGATTAGAAGGCAAAATAGAAGAAC
>Bacillus_Fam_1641_33_1 Nr. of seq. 1 Alignment length(with gaps) = 33
Alignment score = 0.000000
 ATTGTTGGAAGTCTAAGGATAAACATCATTCTG
>Bacillus_Fam_1642_33_1 Nr. of seq. 1 Alignment length(with gaps) = 33
Alignment score = 0.000000
 CAACTAACAGAAACGAAAGATAACTTACAGTCA
>Bacillus_Fam_1643_33_1 Nr. of seq. 1 Alignment length(with gaps) = 33
Alignment score = 0.000000
 ATCAGCATGACCTTCTTGCTTAGGAGCAGGGAA
>Bacillus_Fam_1644_33_1 Nr. of seq. 1 Alignment length(with gaps) = 33
Alignment score = 0.000000
 AAAAGAAGAAAGCTTGTCGCAGCGAGAAAAGCA
>Bacillus_Fam_1645_33_1 Nr. of seq. 1 Alignment length(with gaps) = 33
Alignment score = 0.000000
 GGTTGGTTTTGTTCTGGTGTGTTGTCCACTTCC
>Bacillus_Fam_1646_33_1 Nr. of seq. 1 Alignment length(with gaps) = 33
Alignment score = 0.000000
 AGTGTTTTACTAACTTNTTCATCTAATTCAGTT
>Bacillus_Fam_1647_33_1 Nr. of seq. 1 Alignment length(with gaps) = 33
Alignment score = 0.000000
 TAAGTTTGATGGAAAGCTTGAGGATTTAAGCAA
>Bacillus_Fam_1648_33_1 Nr. of seq. 1 Alignment length(with gaps) = 33
Alignment score = 0.000000
 GTTAAACCTTGGTTCATTTCATTTCTTACTTCC

>Bacillus_Fam_1649_33_1 Nr. of seq. 1 Alignment length(with gaps) = 33
Alignment score = 0.000000
CTGACGTACAAGCGGAAATGCAAGAGTTAAGAA
>Bacillus_Fam_1650_33_1 Nr. of seq. 1 Alignment length(with gaps) = 33
Alignment score = 0.000000
ATTTCATCTTTTAAGTCCACTCTCACTACCGAG
>Bacillus_Fam_1651_32_1 Nr. of seq. 1 Alignment length(with gaps) = 32
Alignment score = 0.000000
CAGTTGTACAAGAACCCCTGTTTGCCAACTGT
>Bacillus_Fam_1652_31_1 Nr. of seq. 1 Alignment length(with gaps) = 31
Alignment score = 0.000000
TAGAATATATGAGAAAACGTTAATTCCGTTG
>Bacillus_Fam_1653_30_1 Nr. of seq. 1 Alignment length(with gaps) = 30
Alignment score = 0.000000
GGATCTTCCGGTTTCACTTCTGGTTCTTTC
>Bacillus_Fam_1654_30_1 Nr. of seq. 1 Alignment length(with gaps) = 30
Alignment score = 0.000000
CTTCATCCTATCGTGAGGCCGATCTCGAGA
>Bacillus_Fam_1655_30_1 Nr. of seq. 1 Alignment length(with gaps) = 30
Alignment score = 0.000000
ATGCAAGGCGGAGGCTACCCGGGCTACGGT
>Bacillus_Fam_1656_30_1 Nr. of seq. 1 Alignment length(with gaps) = 30
Alignment score = 0.000000
ATATTTCGGTTAGCTTTTCAACATAATGGG
>Bacillus_Fam_1657_30_1 Nr. of seq. 1 Alignment length(with gaps) = 30
Alignment score = 0.000000
TGCATGCCCTGCACATTGTTGGGTGGATAC
>Bacillus_Fam_1658_30_1 Nr. of seq. 1 Alignment length(with gaps) = 30
Alignment score = 0.000000
TAANCAAAGAGCCTATGAGTCCAACAGGAG
>Bacillus_Fam_1659_30_1 Nr. of seq. 1 Alignment length(with gaps) = 30
Alignment score = 0.000000
TTTTGTCCATCCGGCTTCTGCTCTTCTGGA
>Bacillus_Fam_1660_30_1 Nr. of seq. 1 Alignment length(with gaps) = 30
Alignment score = 0.000000
AAGAAAAAAGCTGAAGACGACGCAGCAGCC
>Bacillus_Fam_1661_30_1 Nr. of seq. 1 Alignment length(with gaps) = 30
Alignment score = 0.000000
TTACCTGTTCCAGGGTCTGGGTTTCCTCCA
>Bacillus_Fam_1662_30_1 Nr. of seq. 1 Alignment length(with gaps) = 30
Alignment score = 0.000000
CCAGCCAAGCAGCAGCCTGCAGCTCCTGCA
>Bacillus_Fam_1663_30_1 Nr. of seq. 1 Alignment length(with gaps) = 30
Alignment score = 0.000000
TTTTCTTCAGCTATTTGCTTTGCCTTTGCC
>Bacillus_Fam_1664_30_1 Nr. of seq. 1 Alignment length(with gaps) = 30
Alignment score = 0.000000
GATGAGCTTTCTGTTGTACTACTTTCNTCT
>Bacillus_Fam_1665_30_1 Nr. of seq. 1 Alignment length(with gaps) = 30
Alignment score = 0.000000
GAGGAAGATCACGATCACGAACATGGACAT
>Bacillus_Fam_1666_30_1 Nr. of seq. 1 Alignment length(with gaps) = 30
Alignment score = 0.000000
TATGGCGGTCATTCTGGATATGGTATGGGC

>Bacillus_Fam_1667_30_1 Nr. of seq. 1 Alignment length(with gaps) = 30
Alignment score = 0.000000
CTTGTTTCGCTTTTTCCTTCACTAATGCTG
>Bacillus_Fam_1668_30_1 Nr. of seq. 1 Alignment length(with gaps) = 30
Alignment score = 0.000000
CACCATTGTCATGATGGACATAAACCTTGC
>Bacillus_Fam_1669_30_1 Nr. of seq. 1 Alignment length(with gaps) = 30
Alignment score = 0.000000
TCTTTGATCGGTGCNTGATACTTCGGCTGT
>Bacillus_Fam_1670_30_1 Nr. of seq. 1 Alignment length(with gaps) = 30
Alignment score = 0.000000
TACTACTTCTTCAGCTGCCGCTTCTTCAGT
>Bacillus_Fam_1671_30_1 Nr. of seq. 1 Alignment length(with gaps) = 30
Alignment score = 0.000000
CCAGGACCAGGCATGATGCCAGGAGGAATG
>Bacillus_Fam_1672_30_1 Nr. of seq. 1 Alignment length(with gaps) = 30
Alignment score = 0.000000
CCACCTTGTGGTTGCTTTGGCTGCTGTGCT
>Bacillus_Fam_1673_29_1 Nr. of seq. 1 Alignment length(with gaps) = 29
Alignment score = 0.000000
TTGCGGTAATAAAACTTCTAATACCCCTT
>Bacillus_Fam_1674_28_1 Nr. of seq. 1 Alignment length(with gaps) = 28
Alignment score = 0.000000
TTGGCATTAACGGCATTTGATTTTGCGN
>Bacillus_Fam_1675_28_1 Nr. of seq. 1 Alignment length(with gaps) = 28
Alignment score = 0.000000
CCAGTAACGCCAGTAACACCTGTNTAAG
>Bacillus_Fam_1676_27_1 Nr. of seq. 1 Alignment length(with gaps) = 27
Alignment score = 0.000000
GGACCAACAGGNGAGCAAGGGATCCAA
>Bacillus_Fam_1677_27_1 Nr. of seq. 1 Alignment length(with gaps) = 27
Alignment score = 0.000000
GGAGATGACGGCGAAACNCCAGGCACA
>Bacillus_Fam_1678_27_1 Nr. of seq. 1 Alignment length(with gaps) = 27
Alignment score = 0.000000
CCACCATATCCATGATGGTACGGGTAA
>Bacillus_Fam_1679_27_1 Nr. of seq. 1 Alignment length(with gaps) = 27
Alignment score = 0.000000
CCATACATGCCGGATCCTGGGAAACCC
>Bacillus_Fam_1680_27_1 Nr. of seq. 1 Alignment length(with gaps) = 27
Alignment score = 0.000000
TCTTCTTCTCCTCCCGGTGGCAACTCT
>Bacillus_Fam_1681_27_1 Nr. of seq. 1 Alignment length(with gaps) = 27
Alignment score = 0.000000
CCTGGTCCGAATGGAGATACGTTTGGC
>Bacillus_Fam_1682_27_1 Nr. of seq. 1 Alignment length(with gaps) = 27
Alignment score = 0.000000
CCCGGTTCTCCTGGCTCTCCCGGTTTA
>Bacillus_Fam_1683_27_1 Nr. of seq. 1 Alignment length(with gaps) = 27
Alignment score = 0.000000
CACAGGGGCCACAAGGGTTGACGGGGN
>Bacillus_Fam_1684_27_1 Nr. of seq. 1 Alignment length(with gaps) = 27
Alignment score = 0.000000
CCAGAGCCTCCTTCTCCGTCTCCAGTG

>Bacillus_Fam_1685_27_1 Nr. of seq. 1 Alignment length(with gaps) = 27
Alignment score = 0.000000
 TTTCTGAAATTGCTNCCACTTCTTGAN
>Bacillus_Fam_1686_27_1 Nr. of seq. 1 Alignment length(with gaps) = 27
Alignment score = 0.000000
 TGAAGCTGTTGAAGCTGTTGATGCTCT
>Bacillus_Fam_1687_27_1 Nr. of seq. 1 Alignment length(with gaps) = 27
Alignment score = 0.000000
 GAGGTCCTTGAGGCCCAGTTGCTCCCT
>Bacillus_Fam_1688_27_1 Nr. of seq. 1 Alignment length(with gaps) = 27
Alignment score = 0.000000
 AGCAAAAACCGTTGCAGCCTATTCAAC
>Bacillus_Fam_1689_27_1 Nr. of seq. 1 Alignment length(with gaps) = 27
Alignment score = 0.000000
 GGTTCTACTGGTTCAACAGGAACATCA
>Bacillus_Fam_1690_27_1 Nr. of seq. 1 Alignment length(with gaps) = 27
Alignment score = 0.000000
 AACTGCTTGATGAGCTCTCGCATGAGG
>Bacillus_Fam_1691_27_1 Nr. of seq. 1 Alignment length(with gaps) = 27
Alignment score = 0.000000
 CCTTCTGGTCCCGCTGGTCCTATTGCT
>Bacillus_Fam_1692_27_1 Nr. of seq. 1 Alignment length(with gaps) = 27
Alignment score = 0.000000
 CCTGCATTTCCTGTTCTTTCTGCTCTG
>Bacillus_Fam_1693_27_1 Nr. of seq. 1 Alignment length(with gaps) = 27
Alignment score = 0.000000
 GCCAATTTTACTAGGTAAAATTCTACT
>Bacillus_Fam_1694_27_1 Nr. of seq. 1 Alignment length(with gaps) = 27
Alignment score = 0.000000
 TCATGTCCATGCTCTTCATGCTCGTGA
>Bacillus_Fam_1695_26_1 Nr. of seq. 1 Alignment length(with gaps) = 26
Alignment score = 0.000000
 TGTACCAGGTACAANACCNCAACTNN
>Bacillus_Fam_1696_25_1 Nr. of seq. 1 Alignment length(with gaps) = 25
Alignment score = 0.000000
 TACTACCCTATGGTAAAATATGTAA
>Bacillus_Fam_1697_25_1 Nr. of seq. 1 Alignment length(with gaps) = 25
Alignment score = 0.000000
 TGTACCTGGTACATGANCCNGTNNT
>Bacillus_Fam_1698_24_1 Nr. of seq. 1 Alignment length(with gaps) = 24
Alignment score = 0.000000
 TGGAGTTGGAGGATTCGGCGGACC
>Bacillus_Fam_1699_24_1 Nr. of seq. 1 Alignment length(with gaps) = 24
Alignment score = 0.000000
 GGGACACAGCTGTTGGAGCTGTGC
>Bacillus_Fam_1700_24_1 Nr. of seq. 1 Alignment length(with gaps) = 24
Alignment score = 0.000000
 CCTCCGCCAATACCAGGCACGGCG
>Bacillus_Fam_1701_24_1 Nr. of seq. 1 Alignment length(with gaps) = 24
Alignment score = 0.000000
 GGAGGATTTTGCGCAGGCAGGTGC
>Bacillus_Fam_1702_24_1 Nr. of seq. 1 Alignment length(with gaps) = 24
Alignment score = 0.000000
 ACTGTTTTTCGTTCTGCTATTTAC

>Bacillus_Fam_1703_24_1 Nr. of seq. 1 Alignment length(with gaps) = 24
Alignment score = 0.000000
GACCAAAACACAATACAGAAGTTG
>Bacillus_Fam_1704_24_1 Nr. of seq. 1 Alignment length(with gaps) = 24
Alignment score = 0.000000
CCGGGTGAAGGGCCAGGAGAAAAC
>Bacillus_Fam_1705_24_1 Nr. of seq. 1 Alignment length(with gaps) = 24
Alignment score = 0.000000
AGTGTCTTTTGTTCTCTCAGAGTT
>Bacillus_Fam_1706_24_1 Nr. of seq. 1 Alignment length(with gaps) = 24
Alignment score = 0.000000
TCGGCACTTTCATCTNCACTTTCG
>Bacillus_Fam_1707_24_1 Nr. of seq. 1 Alignment length(with gaps) = 24
Alignment score = 0.000000
TCCTCCAGTGCTGCCTCCGTTGTC
>Bacillus_Fam_1708_24_1 Nr. of seq. 1 Alignment length(with gaps) = 24
Alignment score = 0.000000
TTACCTTGGCCACCACCTTGGTCG
>Bacillus_Fam_1709_24_1 Nr. of seq. 1 Alignment length(with gaps) = 24
Alignment score = 0.000000
TTGTACTTGCTCCTGCACCGGCTC
>Bacillus_Fam_1710_24_1 Nr. of seq. 1 Alignment length(with gaps) = 24
Alignment score = 0.000000
CCATGATGATTGTGATGGTGTCCG
>Bacillus_Fam_1711_24_1 Nr. of seq. 1 Alignment length(with gaps) = 24
Alignment score = 0.000000
CCTGGTCCATAACCAGGGCCCATA
>Bacillus_Fam_1712_24_1 Nr. of seq. 1 Alignment length(with gaps) = 24
Alignment score = 0.000000
CGGTACTGGCGCCGGTGGCAACGG
>Bacillus_Fam_1713_24_1 Nr. of seq. 1 Alignment length(with gaps) = 24
Alignment score = 0.000000
TGATTCATATGCGCTTGTTGCGGC
>Bacillus_Fam_1714_24_1 Nr. of seq. 1 Alignment length(with gaps) = 24
Alignment score = 0.000000
GGTGGCGTTGTACCTCCATCTCCG
>Bacillus_Fam_1715_24_1 Nr. of seq. 1 Alignment length(with gaps) = 24
Alignment score = 0.000000
GTTTGTGCTGGCTTTTGAGTCTGA
>Bacillus_Fam_1716_24_1 Nr. of seq. 1 Alignment length(with gaps) = 24
Alignment score = 0.000000
GGCTAATAGANCGAAAGTGATAAC
>Bacillus_Fam_1717_24_1 Nr. of seq. 1 Alignment length(with gaps) = 24
Alignment score = 0.000000
TTCACGAGTAAATGACCCAATNAN
>Bacillus_Fam_1718_24_1 Nr. of seq. 1 Alignment length(with gaps) = 24
Alignment score = 0.000000
ATTCCGCCAACTATTGGAGTTNGG
>Bacillus_Fam_1719_24_1 Nr. of seq. 1 Alignment length(with gaps) = 24
Alignment score = 0.000000
ATATCAGCCAANGTAACGGGGAGC
>Bacillus_Fam_1720_24_1 Nr. of seq. 1 Alignment length(with gaps) = 24
Alignment score = 0.000000
TTTCAATTTCAATTCGTCTTCTTC

>Bacillus_Fam_1721_24_1 Nr. of seq. 1 Alignment length(with gaps) = 24
Alignment score = 0.000000
 TTGCAGTACGCTAGAGTACTCCAG
>Bacillus_Fam_1722_24_1 Nr. of seq. 1 Alignment length(with gaps) = 24
Alignment score = 0.000000
 GAAAAACAGCCTGCAACAGAAGAA
>Bacillus_Fam_1723_24_1 Nr. of seq. 1 Alignment length(with gaps) = 24
Alignment score = 0.000000
 TGGTTCAATCNCTCGNTCTTCAAA
>Bacillus_Fam_1724_24_1 Nr. of seq. 1 Alignment length(with gaps) = 24
Alignment score = 0.000000
 GTGCCGTCACCAGTACCAGTACCC
>Bacillus_Fam_1725_24_1 Nr. of seq. 1 Alignment length(with gaps) = 24
Alignment score = 0.000000
 GAAGAACCAACAACAGAACAACCT
>Bacillus_Fam_1726_24_1 Nr. of seq. 1 Alignment length(with gaps) = 24
Alignment score = 0.000000
 AAACCAAGTGAAATAGACGAGACA
>Bacillus_Fam_1727_24_1 Nr. of seq. 1 Alignment length(with gaps) = 24
Alignment score = 0.000000
 TTATTACCTGGTTTCTCGCCCGGT
>Bacillus_Fam_1728_24_1 Nr. of seq. 1 Alignment length(with gaps) = 24
Alignment score = 0.000000
 CGACCATCAGGTCAAGGCCAACAA
>Bacillus_Fam_1729_23_1 Nr. of seq. 1 Alignment length(with gaps) = 23
Alignment score = 0.000000
 GTGCACGCAAGAAGNCCGAAAAA
>Bacillus_Fam_1730_23_1 Nr. of seq. 1 Alignment length(with gaps) = 23
Alignment score = 0.000000
 TTGGCCGGTAATTTNCATTNAAA
>Bacillus_Fam_1731_23_1 Nr. of seq. 1 Alignment length(with gaps) = 23
Alignment score = 0.000000
 ATTCGGCCCTTANTCTCCATCNN
>Bacillus_Fam_1732_23_1 Nr. of seq. 1 Alignment length(with gaps) = 23
Alignment score = 0.000000
 TACATGTAAAATCATATTTTCGA
>Bacillus_Fam_1733_23_1 Nr. of seq. 1 Alignment length(with gaps) = 23
Alignment score = 0.000000
 TACATCCAACAATTAGAGCAATT
>Bacillus_Fam_1734_23_1 Nr. of seq. 1 Alignment length(with gaps) = 23
Alignment score = 0.000000
 GGTGATAAATAAATACCACTCTT
>Bacillus_Fam_1735_23_1 Nr. of seq. 1 Alignment length(with gaps) = 23
Alignment score = 0.000000
 GTTGAACAGAANNATCAAGTTGA
>Bacillus_Fam_1736_23_1 Nr. of seq. 1 Alignment length(with gaps) = 23
Alignment score = 0.000000
 AATCCAGCCGANAATACGAAANG
>Bacillus_Fam_1737_23_1 Nr. of seq. 1 Alignment length(with gaps) = 23
Alignment score = 0.000000
 TTCGCCGATAACTNGCTNCATGN
>Bacillus_Fam_1738_23_1 Nr. of seq. 1 Alignment length(with gaps) = 23
Alignment score = 0.000000
 TTTTCCGGCCGGTCGACTCGACT

>Bacillus_Fam_1739_23_1 Nr. of seq. 1 Alignment length(with gaps) = 23
Alignment score = 0.000000
AATCGCAAATAAAATCNAAGNNN
>Bacillus_Fam_1740_23_1 Nr. of seq. 1 Alignment length(with gaps) = 23
Alignment score = 0.000000
AAATCAACCAACTNATGAGTGAG
>Bacillus_Fam_1741_22_1 Nr. of seq. 1 Alignment length(with gaps) = 22
Alignment score = 0.000000
TATTTTCCGTTACNGNTATATT
>Bacillus_Fam_1742_22_1 Nr. of seq. 1 Alignment length(with gaps) = 22
Alignment score = 0.000000
TTTCTTGATATATCTTGCAANA
>Bacillus_Fam_1743_22_1 Nr. of seq. 1 Alignment length(with gaps) = 22
Alignment score = 0.000000
TTTATCGGCCGCTTTTGATAAT
>Bacillus_Fam_1744_22_1 Nr. of seq. 1 Alignment length(with gaps) = 22
Alignment score = 0.000000
TTTATTTGCGAAACTAGCCACA
>Bacillus_Fam_1745_22_1 Nr. of seq. 1 Alignment length(with gaps) = 22
Alignment score = 0.000000
ATTGCGGGCGTTCTANGCCATA
>Bacillus_Fam_1746_22_1 Nr. of seq. 1 Alignment length(with gaps) = 22
Alignment score = 0.000000
GATACCTTATGAGTACGGAAAT
>Bacillus_Fam_1747_22_1 Nr. of seq. 1 Alignment length(with gaps) = 22
Alignment score = 0.000000
GAGGAGAAGAAANAGCGGAGCN
>Bacillus_Fam_1748_22_1 Nr. of seq. 1 Alignment length(with gaps) = 22
Alignment score = 0.000000
TATAATGAATTCCTCAACNATA
>Bacillus_Fam_1749_22_1 Nr. of seq. 1 Alignment length(with gaps) = 22
Alignment score = 0.000000
TAATCCTGATTTCCGCTGCATT
>Bacillus_Fam_1750_22_1 Nr. of seq. 1 Alignment length(with gaps) = 22
Alignment score = 0.000000
CAATAATTGATGCAATCAACTT
>Bacillus_Fam_1751_22_1 Nr. of seq. 1 Alignment length(with gaps) = 22
Alignment score = 0.000000
TTTACTTAACAGAGCTTCGGAA
>Bacillus_Fam_1752_22_1 Nr. of seq. 1 Alignment length(with gaps) = 22
Alignment score = 0.000000
TGGTGCAACACCACCGCAAAAN
>Bacillus_Fam_1753_22_1 Nr. of seq. 1 Alignment length(with gaps) = 22
Alignment score = 0.000000
TCTATTGGCGAATTTCACCTAN
>Bacillus_Fam_1754_22_1 Nr. of seq. 1 Alignment length(with gaps) = 22
Alignment score = 0.000000
TTTGATAGGAAAGCTGNTTANN
>Bacillus_Fam_1755_22_1 Nr. of seq. 1 Alignment length(with gaps) = 22
Alignment score = 0.000000
TTTACCCGATTANACGCATANT
>Bacillus_Fam_1756_22_1 Nr. of seq. 1 Alignment length(with gaps) = 22
Alignment score = 0.000000
TTTAATTGCAGATATAGCATAN

>Bacillus_Fam_1757_22_1 Nr. of seq. 1 Alignment length(with gaps) = 22
Alignment score = 0.000000
ATTCGTCCGAAATCCGCCTTCN
>Bacillus_Fam_1758_22_1 Nr. of seq. 1 Alignment length(with gaps) = 22
Alignment score = 0.000000
TCGCGGGATTNCCATGCTCTTT
>Bacillus_Fam_1759_21_1 Nr. of seq. 1 Alignment length(with gaps) = 21
Alignment score = 0.000000
TCAAACCTTTTATCAACAGCA
>Bacillus_Fam_1760_21_1 Nr. of seq. 1 Alignment length(with gaps) = 21
Alignment score = 0.000000
GGTGGCACAACAAACCCAGGC
>Bacillus_Fam_1761_21_1 Nr. of seq. 1 Alignment length(with gaps) = 21
Alignment score = 0.000000
CCGCACGAGAAGAAGGTCCNC
>Bacillus_Fam_1762_21_1 Nr. of seq. 1 Alignment length(with gaps) = 21
Alignment score = 0.000000
GCCGGGCACACCGCCGCCGAA
>Bacillus_Fam_1763_21_1 Nr. of seq. 1 Alignment length(with gaps) = 21
Alignment score = 0.000000
TCTTTTTTTGGCTCACAATCC
>Bacillus_Fam_1764_21_1 Nr. of seq. 1 Alignment length(with gaps) = 21
Alignment score = 0.000000
TTACCCATATATCTGNGCACT
>Bacillus_Fam_1765_21_1 Nr. of seq. 1 Alignment length(with gaps) = 21
Alignment score = 0.000000
TTCCTTCCGTGGTAACGTGGA
>Bacillus_Fam_1766_21_1 Nr. of seq. 1 Alignment length(with gaps) = 21
Alignment score = 0.000000
AATCGAGTGCTTTTCTCGCGG
>Bacillus_Fam_1767_21_1 Nr. of seq. 1 Alignment length(with gaps) = 21
Alignment score = 0.000000
TTTACCCATATGAATTAGCTA
>Bacillus_Fam_1768_21_1 Nr. of seq. 1 Alignment length(with gaps) = 21
Alignment score = 0.000000
TACCCACATAAAATGCGGATT
>Bacillus_Fam_1769_21_1 Nr. of seq. 1 Alignment length(with gaps) = 21
Alignment score = 0.000000
CATGAAGAAGAAGAACATGGA
>Bacillus_Fam_1770_21_1 Nr. of seq. 1 Alignment length(with gaps) = 21
Alignment score = 0.000000
TTGGCTGATATCGGTGTTGGA
>Bacillus_Fam_1771_21_1 Nr. of seq. 1 Alignment length(with gaps) = 21
Alignment score = 0.000000
TATAATGAATCTCGAANCATT
>Bacillus_Fam_1772_21_1 Nr. of seq. 1 Alignment length(with gaps) = 21
Alignment score = 0.000000
AAAGTGATTGATAANNGGATN
>Bacillus_Fam_1773_21_1 Nr. of seq. 1 Alignment length(with gaps) = 21
Alignment score = 0.000000
TTAAACAGGTTAGGCTCTCAT
>Bacillus_Fam_1774_21_1 Nr. of seq. 1 Alignment length(with gaps) = 21
Alignment score = 0.000000
TAATTCTACCTAAAAAGNCAT

>Bacillus_Fam_1775_21_1 Nr. of seq. 1 Alignment length(with gaps) = 21
Alignment score = 0.000000
 GAGCAAAAAAGNACCNTGCNG
>Bacillus_Fam_1776_21_1 Nr. of seq. 1 Alignment length(with gaps) = 21
Alignment score = 0.000000
 TTTTTGCTCANCACACCNATN
>Bacillus_Fam_1777_21_1 Nr. of seq. 1 Alignment length(with gaps) = 21
Alignment score = 0.000000
 TGCCACAAGGAATGCATCAAG
>Bacillus_Fam_1778_21_1 Nr. of seq. 1 Alignment length(with gaps) = 21
Alignment score = 0.000000
 ACTGACGCAATAGCGTCTGGA
>Bacillus_Fam_1779_21_1 Nr. of seq. 1 Alignment length(with gaps) = 21
Alignment score = 0.000000
 TTGGATCTTTCGGATCTGTGC
>Bacillus_Fam_1780_21_1 Nr. of seq. 1 Alignment length(with gaps) = 21
Alignment score = 0.000000
 CTTCTTGGGACCGTCATCCGG
>Bacillus_Fam_1781_21_1 Nr. of seq. 1 Alignment length(with gaps) = 21
Alignment score = 0.000000
 AAGTGACCGGTATTTNNACNA
>Bacillus_Fam_1782_21_1 Nr. of seq. 1 Alignment length(with gaps) = 21
Alignment score = 0.000000
 GAACTGCGACGGTTGCGAGAA
>Bacillus_Fam_1783_21_1 Nr. of seq. 1 Alignment length(with gaps) = 21
Alignment score = 0.000000
 GGATCATTTTCCAATATCNTT
>Bacillus_Fam_1784_21_1 Nr. of seq. 1 Alignment length(with gaps) = 21
Alignment score = 0.000000
 AATTTGTAGATATCTAGACCA
>Bacillus_Fam_1785_21_1 Nr. of seq. 1 Alignment length(with gaps) = 21
Alignment score = 0.000000
 CAGCCAGCACCGCAGCAGGCA
>Bacillus_Fam_1786_21_1 Nr. of seq. 1 Alignment length(with gaps) = 21
Alignment score = 0.000000
 CCTGGGAAAAACGGACCCACA
>Bacillus_Fam_1787_21_1 Nr. of seq. 1 Alignment length(with gaps) = 21
Alignment score = 0.000000
 TTTGGATTGTTACACGCCGCA
>Bacillus_Fam_1788_21_1 Nr. of seq. 1 Alignment length(with gaps) = 21
Alignment score = 0.000000
 AACGGGAGTAAANTCCTCCTG
>Bacillus_Fam_1789_21_1 Nr. of seq. 1 Alignment length(with gaps) = 21
Alignment score = 0.000000
 ATTCAACAGTAGATTACGAGT
>Bacillus_Fam_1790_21_1 Nr. of seq. 1 Alignment length(with gaps) = 21
Alignment score = 0.000000
 ATGGACAAGCTTGAATCCCGA
>Bacillus_Fam_1791_21_1 Nr. of seq. 1 Alignment length(with gaps) = 21
Alignment score = 0.000000
 TACGAGAGTAAACCTGNGTTA
>Bacillus_Fam_1792_21_1 Nr. of seq. 1 Alignment length(with gaps) = 21
Alignment score = 0.000000
 TCTACAAATTCAGGNCCGTAT

>Bacillus_Fam_1793_21_1 Nr. of seq. 1 Alignment length(with gaps) = 21
Alignment score = 0.000000
TGTTTCTACAAATTAAAGATG
>Bacillus_Fam_1794_21_1 Nr. of seq. 1 Alignment length(with gaps) = 21
Alignment score = 0.000000
TTTTGTAGANTNAGCACTTAA
>Bacillus_Fam_1795_21_1 Nr. of seq. 1 Alignment length(with gaps) = 21
Alignment score = 0.000000
TACTGTTGAAGTTTCATCTTT
>Bacillus_Fam_1796_21_1 Nr. of seq. 1 Alignment length(with gaps) = 21
Alignment score = 0.000000
AACTTCGGAAATNATAGCNTC
>Bacillus_Fam_1797_21_1 Nr. of seq. 1 Alignment length(with gaps) = 21
Alignment score = 0.000000
AGTCCAAAACCNCATGGAAAT
>Bacillus_Fam_1798_21_1 Nr. of seq. 1 Alignment length(with gaps) = 21
Alignment score = 0.000000
CTGTTCTACAGCTTCGACAAG
>Bacillus_Fam_1799_21_1 Nr. of seq. 1 Alignment length(with gaps) = 21
Alignment score = 0.000000
AAGGAACAGCCGAAACAAGTA
>Bacillus_Fam_1800_21_1 Nr. of seq. 1 Alignment length(with gaps) = 21
Alignment score = 0.000000
AGAGATTTATCTCCAGTTAAG
>Bacillus_Fam_1801_21_1 Nr. of seq. 1 Alignment length(with gaps) = 21
Alignment score = 0.000000
ATTCCGCGAAAAAATTCTTTA
>Bacillus_Fam_1802_21_1 Nr. of seq. 1 Alignment length(with gaps) = 21
Alignment score = 0.000000
TTTTTCTACGGTTGGCAGCAN
>Bacillus_Fam_1803_21_1 Nr. of seq. 1 Alignment length(with gaps) = 21
Alignment score = 0.000000
AAAACCAGAAGGCGAAGGTGG
>Bacillus_Fam_1804_21_1 Nr. of seq. 1 Alignment length(with gaps) = 21
Alignment score = 0.000000
AGACTAAGCTGATACTTAATG
>Bacillus_Fam_1805_21_1 Nr. of seq. 1 Alignment length(with gaps) = 21
Alignment score = 0.000000
GGTTGGATCGGGTTGAAGAGC
>Bacillus_Fam_1806_21_1 Nr. of seq. 1 Alignment length(with gaps) = 21
Alignment score = 0.000000
TTGGAAAATAAACTTNCTNTN
>Bacillus_Fam_1807_21_1 Nr. of seq. 1 Alignment length(with gaps) = 21
Alignment score = 0.000000
GACGAATTATCTTCTCTCAAG
>Bacillus_Fam_1808_21_1 Nr. of seq. 1 Alignment length(with gaps) = 21
Alignment score = 0.000000
GGCACATAAACTACGTCGAGT
>Bacillus_Fam_1809_21_1 Nr. of seq. 1 Alignment length(with gaps) = 21
Alignment score = 0.000000
TTATGCGCGATCTTCCCTCGG
>Bacillus_Fam_1810_21_1 Nr. of seq. 1 Alignment length(with gaps) = 21
Alignment score = 0.000000
AACAAGCACAGCANGCAATTC

>Bacillus_Fam_1811_21_1 Nr. of seq. 1 Alignment length(with gaps) = 21
Alignment score = 0.000000
TTTGCGAATAAACCATNNATT
>Bacillus_Fam_1812_21_1 Nr. of seq. 1 Alignment length(with gaps) = 21
Alignment score = 0.000000
TGCTAGATAAATAGCTACAAT
>Bacillus_Fam_1813_21_1 Nr. of seq. 1 Alignment length(with gaps) = 21
Alignment score = 0.000000
GCGGGATTATAGTAAAAATTC
>Bacillus_Fam_1814_21_1 Nr. of seq. 1 Alignment length(with gaps) = 21
Alignment score = 0.000000
TAATAGACAAAATACTTNGGN
>Bacillus_Fam_1815_21_1 Nr. of seq. 1 Alignment length(with gaps) = 21
Alignment score = 0.000000
CGACGCAAATAATCCAGTAAT
>Bacillus_Fam_1816_21_1 Nr. of seq. 1 Alignment length(with gaps) = 21
Alignment score = 0.000000
TAGGGCACAATTANTTCCANN
>Bacillus_Fam_1817_21_1 Nr. of seq. 1 Alignment length(with gaps) = 21
Alignment score = 0.000000
ACCTCCTAGTCCAACAGGAAA
>Bacillus_Fam_1818_21_1 Nr. of seq. 1 Alignment length(with gaps) = 21
Alignment score = 0.000000
GCGAGAATCCAGACAAGAGGA
>Bacillus_Fam_1819_21_1 Nr. of seq. 1 Alignment length(with gaps) = 21
Alignment score = 0.000000
GTCCTCCAATGCTTCAACTCT
>Bacillus_Fam_1820_21_1 Nr. of seq. 1 Alignment length(with gaps) = 21
Alignment score = 0.000000
GGAAGCGGAGACGGAGATAGC
>Bacillus_Fam_1821_21_1 Nr. of seq. 1 Alignment length(with gaps) = 21
Alignment score = 0.000000
GTTGCACCAGTTTGTCACGGT
>Bacillus_Fam_1822_21_1 Nr. of seq. 1 Alignment length(with gaps) = 21
Alignment score = 0.000000
TGGCACAAAAGGATGTTGGGT
>Bacillus_Fam_1823_21_1 Nr. of seq. 1 Alignment length(with gaps) = 21
Alignment score = 0.000000
TTGACGCACTTGNAAATTTAA
>Bacillus_Fam_1824_21_1 Nr. of seq. 1 Alignment length(with gaps) = 21
Alignment score = 0.000000
TTTATGTGTGCCNGTGAANCN
>Bacillus_Fam_1825_21_1 Nr. of seq. 1 Alignment length(with gaps) = 21
Alignment score = 0.000000
CCGAAACCTGGGCCTCCTGGG
>Bacillus_Fam_1826_21_1 Nr. of seq. 1 Alignment length(with gaps) = 21
Alignment score = 0.000000
TGCAAATAGAANGCANGAATN
>Bacillus_Fam_1827_21_1 Nr. of seq. 1 Alignment length(with gaps) = 21
Alignment score = 0.000000
TTTGGCCGGTAAATTATGCAN
>Bacillus_Fam_1828_21_1 Nr. of seq. 1 Alignment length(with gaps) = 21
Alignment score = 0.000000
GAAGCTCTTCGGACTGCGTTT

>Bacillus_Fam_1829_21_1 Nr. of seq. 1 Alignment length(with gaps) = 21
Alignment score = 0.000000
TATCAGCCAAACTCGAGGGCT
>Bacillus_Fam_1830_21_1 Nr. of seq. 1 Alignment length(with gaps) = 21
Alignment score = 0.000000
AAATAGGTAGTAAGTGACCAT
>Bacillus_Fam_1831_21_1 Nr. of seq. 1 Alignment length(with gaps) = 21
Alignment score = 0.000000
TTTTGTTGAATGGCACCTGGT
>Bacillus_Fam_1832_21_1 Nr. of seq. 1 Alignment length(with gaps) = 21
Alignment score = 0.000000
CGCCTTGACGGACTAGACCAA
>Bacillus_Fam_1833_21_1 Nr. of seq. 1 Alignment length(with gaps) = 21
Alignment score = 0.000000
TTAGCGATTTNANACNGCCTA
>Bacillus_Fam_1834_21_1 Nr. of seq. 1 Alignment length(with gaps) = 21
Alignment score = 0.000000
GACGGGTAAAATCNAAAANTT
>Bacillus_Fam_1835_21_1 Nr. of seq. 1 Alignment length(with gaps) = 21
Alignment score = 0.000000
GGCGAATAAAACAGTGAGCAT
>Bacillus_Fam_1836_21_1 Nr. of seq. 1 Alignment length(with gaps) = 21
Alignment score = 0.000000
TTCCTATCAAAANAAGCGGAC
>Bacillus_Fam_1837_21_1 Nr. of seq. 1 Alignment length(with gaps) = 21
Alignment score = 0.000000
AATGATCGAAAANATCTCNAN
>Bacillus_Fam_1838_21_1 Nr. of seq. 1 Alignment length(with gaps) = 21
Alignment score = 0.000000
AAATGTTGCACAAAAACGAAA
>Bacillus_Fam_1839_21_1 Nr. of seq. 1 Alignment length(with gaps) = 21
Alignment score = 0.000000
GGACGAATTATCTCCTGATTG
>Bacillus_Fam_1840_21_1 Nr. of seq. 1 Alignment length(with gaps) = 21
Alignment score = 0.000000
ATTGCGCGACTTTCATNTGGN
>Bacillus_Fam_1841_21_1 Nr. of seq. 1 Alignment length(with gaps) = 21
Alignment score = 0.000000
AGTCGCGCAATGCAGGTATAA
>Bacillus_Fam_1842_21_1 Nr. of seq. 1 Alignment length(with gaps) = 21
Alignment score = 0.000000
TGACGGGATTNCCNNTTCTTA
>Bacillus_Fam_1843_21_1 Nr. of seq. 1 Alignment length(with gaps) = 21
Alignment score = 0.000000
TGAAGGGATTTTNCACACTTG
>Bacillus_Fam_1844_21_1 Nr. of seq. 1 Alignment length(with gaps) = 21
Alignment score = 0.000000
GCACGAACTCCGAGCGTTTCC
>Bacillus_Fam_1845_21_1 Nr. of seq. 1 Alignment length(with gaps) = 21
Alignment score = 0.000000
TGCAAATAAAANTNNTCAAGN
>Bacillus_Fam_1846_21_1 Nr. of seq. 1 Alignment length(with gaps) = 21
Alignment score = 0.000000
TTATTTGCAGNNTTNAATNTN

>Bacillus_Fam_1847_21_1 Nr. of seq. 1 Alignment length(with gaps) = 21
Alignment score = 0.000000
TTTATTTTCGNTTTTCCTNGT
>Bacillus_Fam_1848_21_1 Nr. of seq. 1 Alignment length(with gaps) = 21
Alignment score = 0.000000
TTTATTTTCGNTTTCTACGCA
>Bacillus_Fam_1849_21_1 Nr. of seq. 1 Alignment length(with gaps) = 21
Alignment score = 0.000000
TCGTGCGAATCATNTCGGAAT
>Bacillus_Fam_1850_21_1 Nr. of seq. 1 Alignment length(with gaps) = 21
Alignment score = 0.000000
AATCCGGCGGATNTGAAGCAG
>Bacillus_Fam_1851_21_1 Nr. of seq. 1 Alignment length(with gaps) = 21
Alignment score = 0.000000
ATTCGTCCAAAATCCNGTAAC
>Bacillus_Fam_1852_21_1 Nr. of seq. 1 Alignment length(with gaps) = 21
Alignment score = 0.000000
TTGGACGAATNAGNGCGNGAA
>Bacillus_Fam_1853_21_1 Nr. of seq. 1 Alignment length(with gaps) = 21
Alignment score = 0.000000
TATTATCGAAATCNANAAATT
>Bacillus_Fam_1854_21_1 Nr. of seq. 1 Alignment length(with gaps) = 21
Alignment score = 0.000000
GGCGCAATTAATTAGANAGTN
>Bacillus_Fam_1855_21_1 Nr. of seq. 1 Alignment length(with gaps) = 21
Alignment score = 0.000000
TTAGATGTTAGTATAGCGTGT
>Bacillus_Fam_1856_21_1 Nr. of seq. 1 Alignment length(with gaps) = 21
Alignment score = 0.000000
TCGTCTTCTTCATGCGCGTGA
>Bacillus_Fam_1857_21_1 Nr. of seq. 1 Alignment length(with gaps) = 21
Alignment score = 0.000000
CGGCTTGGAAAACAAAGTCGA
>Bacillus_Fam_1858_21_1 Nr. of seq. 1 Alignment length(with gaps) = 21
Alignment score = 0.000000
TTATGGGACACTGGTTTCATN
>Bacillus_Fam_1859_21_1 Nr. of seq. 1 Alignment length(with gaps) = 21
Alignment score = 0.000000
TTCATTTCTTGTAGATCCCGC
>Bacillus_Fam_1860_20_1 Nr. of seq. 1 Alignment length(with gaps) = 20
Alignment score = 0.000000
TTTCTTGATATANTGTNAAT
>Bacillus_Fam_1861_20_1 Nr. of seq. 1 Alignment length(with gaps) = 20
Alignment score = 0.000000
TTTGCGTCAACANCCACAGA
>Bacillus_Fam_1862_20_1 Nr. of seq. 1 Alignment length(with gaps) = 20
Alignment score = 0.000000
ATTTGCGTCNNTTCCNNCNG
>Bacillus_Fam_1863_20_1 Nr. of seq. 1 Alignment length(with gaps) = 20
Alignment score = 0.000000
TAATGAGGGTATTAAACTTT
>Bacillus_Fam_1864_20_1 Nr. of seq. 1 Alignment length(with gaps) = 20
Alignment score = 0.000000
TTATCCATCACTTCGGCCGG

>Bacillus_Fam_1865_20_1 Nr. of seq. 1 Alignment length(with gaps) = 20
Alignment score = 0.000000
TTTACCGGCACTTTCACNAT
>Bacillus_Fam_1866_20_1 Nr. of seq. 1 Alignment length(with gaps) = 20
Alignment score = 0.000000
TTACTTGCGGTTGGGGNGCG
>Bacillus_Fam_1867_20_1 Nr. of seq. 1 Alignment length(with gaps) = 20
Alignment score = 0.000000
TTTCCGAAGTTGGCGTGTTT
>Bacillus_Fam_1868_20_1 Nr. of seq. 1 Alignment length(with gaps) = 20
Alignment score = 0.000000
TTTACGTGCCAAATTATAGA
>Bacillus_Fam_1869_20_1 Nr. of seq. 1 Alignment length(with gaps) = 20
Alignment score = 0.000000
TAATTGCAAAAATCCTTGAA
>Bacillus_Fam_1870_20_1 Nr. of seq. 1 Alignment length(with gaps) = 20
Alignment score = 0.000000
TACATTAAAGTTCACTACTT
>Bacillus_Fam_1871_20_1 Nr. of seq. 1 Alignment length(with gaps) = 20
Alignment score = 0.000000
GGCGCAAAAAATCGAATGAGA
>Bacillus_Fam_1872_20_1 Nr. of seq. 1 Alignment length(with gaps) = 20
Alignment score = 0.000000
GCGAATATATTCNTATNACG
>Bacillus_Fam_1873_20_1 Nr. of seq. 1 Alignment length(with gaps) = 20
Alignment score = 0.000000
CGAATAAATCAGAGTAAGGT
>Bacillus_Fam_1874_20_1 Nr. of seq. 1 Alignment length(with gaps) = 20
Alignment score = 0.000000
TTATCCTGAAATCTATGGGT
>Bacillus_Fam_1875_20_1 Nr. of seq. 1 Alignment length(with gaps) = 20
Alignment score = 0.000000
TGACGGGATTTTAGTNGTTA
>Bacillus_Fam_1876_20_1 Nr. of seq. 1 Alignment length(with gaps) = 20
Alignment score = 0.000000
ATTGTGACTTTTCCATNGTT
>Bacillus_Fam_1877_20_1 Nr. of seq. 1 Alignment length(with gaps) = 20
Alignment score = 0.000000
CGAGTTTTTTAGGTCAGGAG
>Bacillus_Fam_1878_20_1 Nr. of seq. 1 Alignment length(with gaps) = 20
Alignment score = 0.000000
TTTATGAGCCANTACCTCTN
>Bacillus_Fam_1879_20_1 Nr. of seq. 1 Alignment length(with gaps) = 20
Alignment score = 0.000000
CCTGAGCTCCTCTCTCTAAT
>Bacillus_Fam_1880_20_1 Nr. of seq. 1 Alignment length(with gaps) = 20
Alignment score = 0.000000
AGTAAAATAGCGGGCGCGAC
>Bacillus_Fam_1881_20_1 Nr. of seq. 1 Alignment length(with gaps) = 20
Alignment score = 0.000000
TTTATCCGTAAGAATTTCAN
>Bacillus_Fam_1882_19_1 Nr. of seq. 1 Alignment length(with gaps) = 19
Alignment score = 0.000000
TATATTCTCTACGTTATTA

>Bacillus_Fam_1883_19_1 Nr. of seq. 1 Alignment length(with gaps) = 19
Alignment score = 0.000000
CTTGATTGAGAATCCTCAG
>Bacillus_Fam_1884_19_1 Nr. of seq. 1 Alignment length(with gaps) = 19
Alignment score = 0.000000
TCATCCTCAGAATGAGTGC
>Bacillus_Fam_1885_18_1 Nr. of seq. 1 Alignment length(with gaps) = 18
Alignment score = 0.000000
GGCAATGGAAACGGGAAT
>Bacillus_Fam_1886_18_1 Nr. of seq. 1 Alignment length(with gaps) = 18
Alignment score = 0.000000
CGTCTGGTTGTTTGTCTC
>Bacillus_Fam_1887_18_1 Nr. of seq. 1 Alignment length(with gaps) = 18
Alignment score = 0.000000
TCAAAACCGTAGTTAAGG
>Bacillus_Fam_1888_18_1 Nr. of seq. 1 Alignment length(with gaps) = 18
Alignment score = 0.000000
CCTGGTATGGGCGGATCA
>Bacillus_Fam_1889_18_1 Nr. of seq. 1 Alignment length(with gaps) = 18
Alignment score = 0.000000
TGTGTTTACGGCATGTTG
>Bacillus_Fam_1890_18_1 Nr. of seq. 1 Alignment length(with gaps) = 18
Alignment score = 0.000000
TGACCGTGATGGACGTGA
>Bacillus_Fam_1891_18_1 Nr. of seq. 1 Alignment length(with gaps) = 18
Alignment score = 0.000000
GTTGTCGTCGGTGCGGCN
>Bacillus_Fam_1892_18_1 Nr. of seq. 1 Alignment length(with gaps) = 18
Alignment score = 0.000000
GTTAACTGAGGAAGAAGG
>Bacillus_Fam_1893_18_1 Nr. of seq. 1 Alignment length(with gaps) = 18
Alignment score = 0.000000
AAAGAACAACCAATCATC
>Bacillus_Fam_1894_18_1 Nr. of seq. 1 Alignment length(with gaps) = 18
Alignment score = 0.000000
GATGCCTGGTGGTGGAAT
>Bacillus_Fam_1895_18_1 Nr. of seq. 1 Alignment length(with gaps) = 18
Alignment score = 0.000000
TAATTAGTTACCAAGCAA
>Bacillus_Fam_1896_18_1 Nr. of seq. 1 Alignment length(with gaps) = 18
Alignment score = 0.000000
TGCTGCTGAAGAGCAGGC
>Bacillus_Fam_1897_18_1 Nr. of seq. 1 Alignment length(with gaps) = 18
Alignment score = 0.000000
GGAAAGAGAACGCGAGCG
>Bacillus_Fam_1898_18_1 Nr. of seq. 1 Alignment length(with gaps) = 18
Alignment score = 0.000000
CATCTAAACCAACTACAG
>Bacillus_Fam_1899_18_1 Nr. of seq. 1 Alignment length(with gaps) = 18
Alignment score = 0.000000
AGAGCGTTTAGACTGCTT
>Bacillus_Fam_1900_18_1 Nr. of seq. 1 Alignment length(with gaps) = 18
Alignment score = 0.000000
TATTATGAATAAAGAATC

>Bacillus_Fam_1901_18_1 Nr. of seq. 1 Alignment length(with gaps) = 18
Alignment score = 0.000000
 AGCTCTTGAAGACGACAC
>Bacillus_Fam_1902_18_1 Nr. of seq. 1 Alignment length(with gaps) = 18
Alignment score = 0.000000
 CACGGACATCACCATGGC
>Bacillus_Fam_1903_18_1 Nr. of seq. 1 Alignment length(with gaps) = 18
Alignment score = 0.000000
 CCTAAGGACCCTATAGAC
>Bacillus_Fam_1904_18_1 Nr. of seq. 1 Alignment length(with gaps) = 18
Alignment score = 0.000000
 CCAGGACAGCCAGGCANC
>Bacillus_Fam_1905_18_1 Nr. of seq. 1 Alignment length(with gaps) = 18
Alignment score = 0.000000
 TCACCTGGTTCTTCGTTA
>Bacillus_Fam_1906_18_1 Nr. of seq. 1 Alignment length(with gaps) = 18
Alignment score = 0.000000
 AGCAGAGGGTCAAAAGGC
>Bacillus_Fam_1907_18_1 Nr. of seq. 1 Alignment length(with gaps) = 18
Alignment score = 0.000000
 CACTAGCCGTCGTCGCTT
>Bacillus_Fam_1908_18_1 Nr. of seq. 1 Alignment length(with gaps) = 18
Alignment score = 0.000000
 TCTTGTTTGTCCTGTTTA
>Bacillus_Fam_1909_18_1 Nr. of seq. 1 Alignment length(with gaps) = 18
Alignment score = 0.000000
 AGCCAGGCCCGGATCCGC
>Bacillus_Fam_1910_18_1 Nr. of seq. 1 Alignment length(with gaps) = 18
Alignment score = 0.000000
 AAGCTCTTGGTCTACATC
>Bacillus_Fam_1911_18_1 Nr. of seq. 1 Alignment length(with gaps) = 18
Alignment score = 0.000000
 TGGCGTTTGATTATTACC
>Bacillus_Fam_1912_18_1 Nr. of seq. 1 Alignment length(with gaps) = 18
Alignment score = 0.000000
 GGATCAGGAAACGGCGAA
>Bacillus_Fam_1913_18_1 Nr. of seq. 1 Alignment length(with gaps) = 18
Alignment score = 0.000000
 CGTTCGCACCGTTCGCGT
>Bacillus_Fam_1914_18_1 Nr. of seq. 1 Alignment length(with gaps) = 18
Alignment score = 0.000000
 TCATCTCCTCCTGTTCCG
>Bacillus_Fam_1915_18_1 Nr. of seq. 1 Alignment length(with gaps) = 18
Alignment score = 0.000000
 ATCACTGACACCATTGAT
>Bacillus_Fam_1916_18_1 Nr. of seq. 1 Alignment length(with gaps) = 18
Alignment score = 0.000000
 TGCCTTGACCGCTGCCAT
>Bacillus_Fam_1917_18_1 Nr. of seq. 1 Alignment length(with gaps) = 18
Alignment score = 0.000000
 GTTGTTCTCAGTTTTTGC
>Bacillus_Fam_1918_18_1 Nr. of seq. 1 Alignment length(with gaps) = 18
Alignment score = 0.000000
 GCTGCATTTGCGGTGTCG

>Bacillus_Fam_1919_18_1 Nr. of seq. 1 Alignment length(with gaps) = 18
Alignment score = 0.000000
TCTTCCATGTTTGTATCA
>Bacillus_Fam_1920_18_1 Nr. of seq. 1 Alignment length(with gaps) = 18
Alignment score = 0.000000
GTCCATGGGGCTGGGGCC
>Bacillus_Fam_1921_18_1 Nr. of seq. 1 Alignment length(with gaps) = 18
Alignment score = 0.000000
CCTTGCATCATCGGCTGG
>Bacillus_Fam_1922_18_1 Nr. of seq. 1 Alignment length(with gaps) = 18
Alignment score = 0.000000
CAGCTCCAGCAGCTTGAA
>Bacillus_Fam_1923_18_1 Nr. of seq. 1 Alignment length(with gaps) = 18
Alignment score = 0.000000
GAGAAAAGCAAGGAGCAC
>Bacillus_Fam_1924_18_1 Nr. of seq. 1 Alignment length(with gaps) = 18
Alignment score = 0.000000
CCTGGTTGCGGNGCAAAG
>Bacillus_Fam_1925_17_1 Nr. of seq. 1 Alignment length(with gaps) = 17
Alignment score = 0.000000
ATGATGCAAAAAAGACG
>Bacillus_Fam_1926_15_1 Nr. of seq. 1 Alignment length(with gaps) = 15
Alignment score = 0.000000
GTAACAATGAACCAA
>Bacillus_Fam_1927_15_1 Nr. of seq. 1 Alignment length(with gaps) = 15
Alignment score = 0.000000
GCAGCAGTTCNCGGC
>Bacillus_Fam_1928_15_1 Nr. of seq. 1 Alignment length(with gaps) = 15
Alignment score = 0.000000
GGATCTTCTTTNACC
>Bacillus_Fam_1929_15_1 Nr. of seq. 1 Alignment length(with gaps) = 15
Alignment score = 0.000000
AATAATGGCAACGGC
>Bacillus_Fam_1930_15_1 Nr. of seq. 1 Alignment length(with gaps) = 15
Alignment score = 0.000000
TATTCGCTCCAGTGC
>Bacillus_Fam_1931_15_1 Nr. of seq. 1 Alignment length(with gaps) = 15
Alignment score = 0.000000
ATCACGAATTCTATA
>Bacillus_Fam_1932_15_1 Nr. of seq. 1 Alignment length(with gaps) = 15
Alignment score = 0.000000
ATTTCTCCGCTTGGC
>Bacillus_Fam_1933_15_1 Nr. of seq. 1 Alignment length(with gaps) = 15
Alignment score = 0.000000
ATTGTAAAAATAAAT
>Bacillus_Fam_1934_15_1 Nr. of seq. 1 Alignment length(with gaps) = 15
Alignment score = 0.000000
TTCAAGTTCAAGCAA
>Bacillus_Fam_1935_15_1 Nr. of seq. 1 Alignment length(with gaps) = 15
Alignment score = 0.000000
AGATTGAGTAAACAG
>Bacillus_Fam_1936_15_1 Nr. of seq. 1 Alignment length(with gaps) = 15
Alignment score = 0.000000
GCTGGTGCCTTCGCT

>Bacillus_Fam_1937_15_1 Nr. of seq. 1 Alignment length(with gaps) = 15
Alignment score = 0.000000
 TTAATTGGTGCTNCT
>Bacillus_Fam_1938_15_1 Nr. of seq. 1 Alignment length(with gaps) = 15
Alignment score = 0.000000
 TGAACTTCTTATTTC
>Bacillus_Fam_1939_15_1 Nr. of seq. 1 Alignment length(with gaps) = 15
Alignment score = 0.000000
 TTCATCGGTTGCTGG
>Bacillus_Fam_1940_15_1 Nr. of seq. 1 Alignment length(with gaps) = 15
Alignment score = 0.000000
 ATGCATGAGAACACC
>Bacillus_Fam_1941_15_1 Nr. of seq. 1 Alignment length(with gaps) = 15
Alignment score = 0.000000
 AGACGTTCTAGTAGA
>Bacillus_Fam_1942_15_1 Nr. of seq. 1 Alignment length(with gaps) = 15
Alignment score = 0.000000
 TCTCCGTGCTCATGG
>Bacillus_Fam_1943_15_1 Nr. of seq. 1 Alignment length(with gaps) = 15
Alignment score = 0.000000
 GAAGTGTTCCGTCTC
>Bacillus_Fam_1944_15_1 Nr. of seq. 1 Alignment length(with gaps) = 15
Alignment score = 0.000000
 CAACCGATCCAGGAC
>Bacillus_Fam_1945_15_1 Nr. of seq. 1 Alignment length(with gaps) = 15
Alignment score = 0.000000
 AATCCTGGACCTCCA
>Bacillus_Fam_1946_15_1 Nr. of seq. 1 Alignment length(with gaps) = 15
Alignment score = 0.000000
 CCGGGGAAACCGCCA
>Bacillus_Fam_1947_15_1 Nr. of seq. 1 Alignment length(with gaps) = 15
Alignment score = 0.000000
 CACTGATGATCCGGG
>Bacillus_Fam_1948_15_1 Nr. of seq. 1 Alignment length(with gaps) = 15
Alignment score = 0.000000
 TGCATCATTGGTGAT
>Bacillus_Fam_1949_14_1 Nr. of seq. 1 Alignment length(with gaps) = 14
Alignment score = 0.000000
 ATTTTGTAGTCGTG
>Bacillus_Fam_1950_13_1 Nr. of seq. 1 Alignment length(with gaps) = 13
Alignment score = 0.000000
 TACTAGGAGACAG
>Bacillus_Fam_1951_13_1 Nr. of seq. 1 Alignment length(with gaps) = 13
Alignment score = 0.000000
 AGTGTTCAGGATA
>Bacillus_Fam_1952_12_1 Nr. of seq. 1 Alignment length(with gaps) = 12
Alignment score = 0.000000
 CGTGATGGTAAT
>Bacillus_Fam_1953_12_1 Nr. of seq. 1 Alignment length(with gaps) = 12
Alignment score = 0.000000
 CGTTCAAGCCGT
>Bacillus_Fam_1954_12_1 Nr. of seq. 1 Alignment length(with gaps) = 12
Alignment score = 0.000000
 CAAATATGATGC

>Bacillus_Fam_1955_12_1 Nr. of seq. 1 Alignment length(with gaps) = 12
Alignment score = 0.000000
 CGTTGTATTATT
>Bacillus_Fam_1956_12_1 Nr. of seq. 1 Alignment length(with gaps) = 12
Alignment score = 0.000000
 ATGCAAACTCTA
>Bacillus_Fam_1957_12_1 Nr. of seq. 1 Alignment length(with gaps) = 12
Alignment score = 0.000000
 CTTTTTTACAAC
>Bacillus_Fam_1958_12_1 Nr. of seq. 1 Alignment length(with gaps) = 12
Alignment score = 0.000000
 ACGACATGGACA
>Bacillus_Fam_1959_12_1 Nr. of seq. 1 Alignment length(with gaps) = 12
Alignment score = 0.000000
 TGGTTCCATTGC
>Bacillus_Fam_1960_12_1 Nr. of seq. 1 Alignment length(with gaps) = 12
Alignment score = 0.000000
 TCTTTGATTCCT
>Bacillus_Fam_1961_12_1 Nr. of seq. 1 Alignment length(with gaps) = 12
Alignment score = 0.000000
 ATTGGTGCCTTT
>Bacillus_Fam_1962_12_1 Nr. of seq. 1 Alignment length(with gaps) = 12
Alignment score = 0.000000
 GGGCGTTCTTCG
>Bacillus_Fam_1963_11_1 Nr. of seq. 1 Alignment length(with gaps) = 11
Alignment score = 0.000000
 GCACTATTTTC
>Bacillus_Fam_1964_11_1 Nr. of seq. 1 Alignment length(with gaps) = 11
Alignment score = 0.000000
 AGGGGCTTAGA
>Bacillus_Fam_1965_11_1 Nr. of seq. 1 Alignment length(with gaps) = 11
Alignment score = 0.000000
 CCAAAAACCGC
>Bacillus_Fam_1966_10_1 Nr. of seq. 1 Alignment length(with gaps) = 10
Alignment score = 0.000000
 TTGTCGCGGA
