# Supplementary material for: Tandem Repeats in Bacillus: Unique Features and Taxonomic Distribution
Source: Int J Mol Sci. 2021 May 20;22(10):5373. doi: 10.3390/ijms22105373 (PMC8161180; doi:10.3390/ijms22105373)
Supplement: Supplementary file 1 [file ijms-22-05373-s001.zip › Table S5. Examples of complete tandem repeat sequences.pdf]

Table S5

EXAMPLES OF COMPLETE TANDEM REPEAT SEQUENCES IN FIVE GENOMES

B.coagulans
B.alkalitelluris
B.mesonae
B.cellulosilyticus
B.weihaiensis

***B.coagulans*. NCBI code: NZ_CP026649.1.**
All tandem repeats with repeat 52

The CG sequence is highlighted to demonstrate the irregular sequence of each repeat, while its size is constant with a very few exceptions. There are two types of tandem repeats, with characteristic signals either **TCTAYG** or **AARGACA** (or reverse). In practically all cases the direct and reverse sequence are present in the same repeat, which may indicate that they form an RNA hairpin. There is only one exception, a short tandem repeat at position 2867866.

```
> TTAAGGAAGC Con:1 reps=11 [16146,16780] l-int=635 l-seq = 52
TTAAGGAAGCGTCGAAAAACGAAGGCCTTTCTTTACATAGAGCCCCTCTATG
TTAAGGAAGCAAACAAAAACGAAGGTCTTCCTTCACATAGAGTGTGTCTACG
TTAAGAAAGCGCGGAAAAACGAAGGTCTTTCTTTACCTAGAGCGCGTCTACG
TTAAGGAAGCAAACAAAAACGAAGATCTTCCTTCACTTAGAGCGCATCTACG
TTAAGGAAGCGCGGAAAAAAGGCGGTCTTTCTTTACCTAGAGCGCGTCTACG
TTAAGGAAGCAAGCAAAAACAAAGGTCTTCCTTAACATAGGGCGCATCTACA
TTAAGGAAGCGCGGAAAAAAGGCGGTCTTTCTTTACCTAGAGCGCGTCTACG
TTAAGGAAGCAAGCAAAAACAAAGGTCTTCCTTAACATAGGGCGCATCTACA
TTAAGGAAGCGCGGAAAAAAGGCGGTCTTTCTTTACCTAGAGCGCGTCTATG
TTAAGGAATCAAACAAAAACAAAGGTCTTCCTTAACATAGGGCGCGTCTACG
TTAAGGAAGCAAACAAAAACGAAGGTCTTCCTTCACTTAGAGCCCGTCTATG
TTAAGGAAGCAAGCAAAAACAAAGGTCTTCCTTAACATAGGGCGCGTCTACGTTAAGGAAGC
> TTTGTCCTTT Con:1 reps=5 [91213,91536] l-int=324 l-seq = 52
TTTGTCCTTTTGGCAGGTTCAAAAAGACATTTCGGACCCGGATGCAGCTTGT
TTTGTCTTTTTGACAGGTTCAAAAAGACATTTTGGGGCCAGGTGCGGTATGT
TTTGTCTTTTTGACGGCTTCAAAAGGACATTTCGAACCCGGATGCAATCGTGT
TTTGTCCTTTTGACGGCTTCAAAGGGACATTTCGGGCCAGGATGCAACGTGT
TTTGTCCTTTTGGCAGGTTCAAGAAGACATTTTGGGGCCAGGTGCAGTTTGA
TTTGTCCTTTTGGCGGTTTCAAAAGACCATTTCGAGCCCAGGTGCGTCTTGTTTTGTCCTTT
> CTACGTTAAG Con:1 reps=13 [246661,247295] l-int=635 l-seq = 52
CTACGTTAAGGAAGCGAGCCAAAACCGCCGTGTATCTTAACATAGAAGCGCT
CTACGTTAAGGAAGCAGCTGAAATTTTCCGTGTATCTTAACATAGAGAGGCT
CTACGTTAAGGAAGCAAGTCAAATCTGCCGTGTATCTTAACATAGAGAGGCT
CTACGTTAAGGAAGCAAGTCAAATCTGCCGTGTATCTTAACATAGAGAGGCT
CTACGTTAAGGAAGCAGCCGAAATCTGTCTTGTATCTTAACATAGAGAGGCC
CTACGTTAAGGAAGCGAACCAAATCCGCCGCGTATCTTAACATAGAGAGGCT
CTACGTTAAGGAAGCAGTCGAAAACTGCTGTGTATCTTAACATAGAAGAGGT
CTACGTTAAGGAAGCAGCTGAAATTTTCCGTGTATCTTAACATAGAGTGGCT
CTACGTTAAGGAAGCAGTCGAAAACTGCTGTATATCTTAACATAGAAGGGCT
CTACGTTAAGGAAGCAAGCCAAACCTTCGCCGTATCTAAACATAGAGTGGCT
CTACGTTAAGGAAGCAGCTGAAATCCGTCGCGTATCTTAACATAGAAGTGCT
CTACGTTAAGGAAGCAGTCGAAAACAGCCGTGTATCTTAACATAGAAGGGCTCTACGTTAAG
> CTATGTGAAG Con:1 reps=4 [272402,272568] l-int=167 l-seq = 52
CTATGTGAAGGAAGATCTTCATTTTTCCGTTCTTCCTTAACGTAGATGGGCT
CTATGTGAAGAAAGCCCTTCATTTTTCCACGCTTTCTTAACATAGCCGGGCT
CTATGTGAAGAAAGACCTTCGTTTTTCCATGCTCCCTTAACGTAGCCGGGTTCTATGTGAAG
> AAAAGACAAA Con:1 reps=9 [304761,305189] l-int=429 l-seq = 52
AAAAGACAAAAGGAAATGATTCCCACCCTGAAATGTCCCCTAGAACACTTCA
AAAAGACAAAAAGGAGGTGGGACCGAGTCCAAATGTCTTTTTAAAACGTTCA
AAAAGACAAAAAAGGATCCGGGCGGAGGTCCAAATGTCCTTTAGAGACCTTCA
AAAAGACAAAAGGCAATGATTCTCGCCCCAAGATGTCCCCTAGACGCCTTCA
AAAAGACAAAAAGGAGTGGGGATCGAGCCAAAATGTCTTTTTAAGATGCTCA
AAAAGACAAAAAGGATCTGGGTAGTGGTCCAAGTGTCCTTTAGAAGCCTTCA
AAAAGACAAAACGGGGGTGGGACTGAGTCCAAATGTCTTTTTAAAACGTTCA
```

AAAAGACAAAAAAGGATCCGTACGATGGTCCCAATGTTTTTAGAGGTCTTTAAAAAGACAAA
> CTTAACGTAG Con:1 reps=17 [312592,313642] l-int=1051 l-seq = 52
CTTAACGTAGACGGGCTCTATGTTAAGAAAGGCCGTCAATTTTTCGTACTTC
CTTAACGTAGACGCCCTCTATGTGAAGAAAGACCTTCAATTTTCCGTGCTTC
CTTAACATAGACGGGCTCTATGTGAAGAAAGGTCATCTTTTTTTCGTGCTTC
CTTAACGTAGACGGGCTCTATGTTAAGAAAGGCCGTCAATTTTTCATGCTTT
CTTAACGTAGACGCGCCCTAAGTGAAGGAAGGTCTTCGATTTTTCGTGCTTT
CTTAACGTAGACGGGCTCTATGTCAAGGAAGGTCTTCGGTTTTCCGTGCTTT
CTTAACGTAGACACCCTCTATGTGAAGAAAGACCTTCAATTTTCCTTGCTTT
CTTAACATAGACGGGCTCTATGTTAAGAAAGGCCGTCAATTTTTCATGCTTT
CTTAACGTAGACGCGCCCTATGTGAAGGAAGGCCGTCAATTTTTCATGCTTT
CTTAACGTAGACGCCCTCTATGTGAAGAAAGACCTTCATTTTTCCGCGCTTC
CTTAACATAGACGGGCCCTATGTGAAGGAAGGTCATCTTTTTTTCATGCTAT
CTTAACGTAGACGCGCCCTATGTAAAGGAAGGTCTTCGTTTTTGCTTGCTTT
CTTAACGTAGACGGGCTCTATGTTAAGAAAGGCCGTCAATTTTTCGTACTTC
CTTAACGTAGACGCCCTCTATGTGAAGAAAGACCTTCAATTTTCCGTGCTTC
CTTCACATAGACGGACTCTATGTGAAGAAAGGTCATCTTTTTTTCGTGCTAT
CTTAACGTAGACGCGCCCTATGTAAAGGAAGGTCTTCGTTTTTTCGTGCTTT
CTTAACGTAGACGGGCTCTATGTTAAGGAAGGCCATCTTTTTTTCGTGCTTT
CTTAACGTAGACGGGCTCTATGTGAAGAAAGGCCGTCAATTTTTCATGCTTC
CTTAACGTAGACGGGCCCTATGTTAAGGAAGGCCATCTTTTTTTTGTGCTTT
CTTAACGTAGACGCCCTCTATGTGAAGGAAGGCCGCCAATTTTCCGCGCTTCCTTAACGTAG
> TCGAAATGTC Con:1 reps=9 [374204,374914] l-int=711 l-seq = 52
TCGAAATGTCCTTTTGGGGGTTTGAAAAGGCCAAAAGCGGCTTGGTTGGTGT
TCGAAATGTCTTTTTGGAGGTTACAAAAGGACAAAAGTGGCTTGGGTAGTGT
TCGAAATGCCTTTTTGGAGAGCTCAAAAAGACAAAAGGAACCCGGGCAGTGA
TCGAAATGTCCTTTTGGAGGGCTCAAAAGGACAAAAGGAATCCGGGTAGCGA
TCGAAATGTCCTTTTGGAGGGTTCAAAAGGCCAAAAGTGGCTTGGGTAGTGT
TCGAAATGTCTTTTTGGAGGTTTGAAAAGGCCAAAAGTGGCCTGGTTGGTGT
TCGGAATGTCCTTTTGGAGGCTTCGAGGCCACTGAAGAAGTGCGAAATAATC
TCGCTAAACACTGTATTTAGATGTATTTACGGGTTTTATCATACTATATATTGTGTTAGTGAATCTATTCAAAAAG
TTTTTCAGTGGCCTCGGAGGCTTCAAAAGGACAAAAGCGGCTTGGGTAGTGT
TCGAAATGTCTTTTTTGAGAGTTCAAAAGGACAAAAGGAATCCGGGCAGTGA
TCGAAATGTCCTTTTGGAGGCTTCAAAAAGACAAAAGGACCCCGGGTAGCGA
TCGAAATGTCCTTTTGGAGGTTTCAAAAAGACAAAAGTGGCCTGGTTGGTGT
TCAAAATGTCTTTTTGGAGGTTTCAAAAGGCCAAAAGCGGCTTGGGTAGTGTTCGAAATGTC
> TCTAAGTTAA Con:1 reps=4 [378507,378725] l-int=219 l-seq = 52
TCTAAGTTAAGGAAGCGCAGAAAAAAGGAGGTCTTCCTTCACGTAGAGCCCG
TCTAAGTTAAGGAAGCAAGCAAAAATAAAGGCCCTTCTTAACATAGAGCCCG
TCTAAGTTAAGGAAGCGAGGAAAAAAGAAGGTCTTCCTTCACGTAGAGCCCG
TCTAAGTGAAGGAAGCGTGGAAAAAAGACGGCCTTTCTTCACATAGAACCCATCTAAGTTAA
> AAAGACAAAA Con:1 reps=5 [419590,419808] l-int=219 l-seq = 52
AAAGACAAAAGTGAAGTGCCCGCGCAGCGAATTGTCTTTCATCAAGGTGATG
AAAGACAAAAGGGGGTTGCCCACGCAGCAAAATGTCTTTCATCGGAGTTATG
AAAGACAAAAGAGAGCTGGAATCCGAGCAAATTGTCCTTTTGGGGGATTCAA
AAAGACAAAAGAAAGCCGGACCCGGGGTGAATTGTCCTTTTGGAGGTTTCAAAAAGACAAAA
> TCAAAAAGAC Con:1 reps=7 [421586,421908] l-int=323 l-seq = 52
TCAAAAAGACAAAAGAGAGCTGGAATCCGAGCAAATTGTCCATTTGGAGGTC
TCAAAAAGACAAAAGAAAGTCGGACCAGAGGCAAACTGTCTTTTTGGAGGTC
TCAAAAAGACAAAAGAAAGCCGGACCCGGGGCAAATTGTCCTTTTGAAGGTC
TCAAAAAGACAAAAGAGAGCTGGGATCCGTACAAATTGTCCTTTTGAAGGTC
TCAAAAAGACAAAAGAGAGCTGGAATCCGGGCAAACTGTCCTTTTGGAGGTT
TCAAAAAGACAAAAGAAAGCCGGACCCGGGGCAAATTGTCCTTTTGGGGGGTTCAAAAAGAC
> TTCAAAAGGA Con:1 reps=6 [430682,430952] l-int=271 l-seq = 52
TTCAAAAGGACATTTCGGGCCCGGATGCAGCTCGTTTTGTCTTTTTGACGGC
TTCAAAAGGACATTTCGGGCCCGGATGCAACTCCTTTTGTCCTTTTGACAGG
TTCAAAAGGACATTTCGTTACCTGATGCAGCTCGTTTTGTCTTTTTGACGGC
TTCAAAAGGACATTTTGGGCCCGGATGCAATTCCTTTTGTCCTTTTGACAGG
TTCAAAAGGACATTTCATTCCCGGATGCGGCACGTTTTGTCCTTTTGACAGGTTCAAAAGGA
> GCTCAAAAAG Con:1 reps=5 [447227,447445] l-int=219 l-seq = 52
GCTCAAAAAGACAAAAGAGAGCTGGACCAGGAGCAAATTGTCCTTTTGGAGA
GCTCAAAAAGACAAAAGAGAGTCGGATCAGGGGCGAATTGTCCTTTTGGAGG
GCTCAAAAAGACAAAAGAAAGCCGGACCAAGAGCAAATTGTCTTTTTGAATG
GCTCAAAAAGACAAAAGAGAGTCGGATCAGGGGCGAATTGTCCTTTTGGAGAGCTCAAAAAG
> GTTCTACGTT Con:1 reps=9 [795794,796220] l-int=427 l-seq = 52
GTTCTACGTTAAGGAACAGACGAAAAAATTGGTCAAAAGTTCACATAGAATG
GTTCTACGTTAAGGAACAGACAAAAAATCCGGTCAAAAGTTCACGTAAAGTG
GTTCTACGTTAAGGAACAGACGAAAAAATCGGTCAAAAGTTCACATAGAACG
GTTCTACGTTAAGGAACAGACGAAAAAATCGGTCAAAAGTTCACATAGAATG
GTTCTACGTTAAGGAACAGACAAAAAATCCGGTCAAAAGTTCACATAGAGCG
GTTCTACGTTAAGGAACAGACAAAAAATCCGGTCAAAAGTTCACATAGAGCG
GTTCTACGTTAAGGAACGGACGAAAAATCCGGTCATAAGTTCACATAGAGTG
GTTCTACGTTAAGGAACAATGCAAAAATCCGGCCATAAGTTCACATAGAGCAGTTCTACGTT
> TGTTCCTTAA Con:1 reps=4 [814905,815123] l-int=219 l-seq = 52
TGTTCCTTAACATAGAGCGCGTCTATGTGAAGGAAGCGAGGAAAAAATGCCT

TGTTCCTTAACGTAGAGTGCGTCTACGTGAAGGAAGCACGCAAAAAAATCGC
TGTTCCTTAACGTAGAGTGCGTCTATGTGAAGGAAACGCGCACAAAAATCGA
TATTCCTTAACATAGAGTGCGTCTACGTGAAGGAAACACGCAAAGAAATCGCTGTTCCTTAA
> CTTAACGTAG Con:1 reps=8 [859087,859513] l-int=427 l-seq = 52
CTTAACGTAGAACGGGTCTAAGTGAAGGAAGCGCGGGAAAAAATTATTGTTT
CTTAACGTAGGACGCGCCTATGTGAAGGAAGCGGGCGAAAATTCCGTTGTTC
CTTAACGTAGAGCGCGCCTACGTTAAGGAAGCGAGCAAAAAACGCAGTGTTC
CTTAACATAGAGTGCGTCTATGTTAAGGAAGCGGGCGCAAAAGACGTTGTTC
CTTAACGTAGAACGGGTCTAAGTGAAGGAAGCGCGGAAAAAACGCCGTGTTT
CTTAACGTAGAACGGGTCTACGTTAAGGAAGCGGGCGAAAATTCCGTTGTTC
CTTAACGTAGAACGGGTCTGAGTGAAGGAAGCGCGGAAAAAACGCGTTGTTT
CTTAACGTAGGATGCGTCTAAGTGAAGGAAGCGAGCGAAAAATCTGGTGTACCTTAACGTAG
> CTTCCTTAAC Con:1 reps=6 [880079,880505] l-int=427 l-seq = 52
CTTCCTTAACATAGAGCGGTTCTACGTTAAGATATCCATAAAAATTCCGCGT
CTTCCTTAACGTAGAAGCGTTCTATGTTAAGATACCCGGCAAAACCGAGGGT
CTCCCTTAACCTAGAGCGCGACTACGTTAAGATATCCGCAAAAATTTTCTCG
CTTCCTTAACATAGAGTGCGTCTACGTTAAGATACCCGGCAAAATTGAGAGT
CTTTCTTAACATAGAGCACGTCTACGTTAAGATATCCATAAAAATTCCGCGT
CTTCCTTAACGTAGAGTGCTTCTATGTTAAGATATCCGGCAAAATCGAAGGT
CTTTCTTAACCTAGAGCGCGACTACGTTCAGATACCCGTAAAAATTCCGCGT
CTTCCTTAACATAGAAGCGTTCTATGTTAAGATACCCGGCAAAATCGAGGGTCTTCCTTAAC
> TTCATAAGCG Con:1 reps=4 [942373,942540] l-int=168 l-seq = 52
TTCATAAGCGGGATGAAAGACAAAAAAGCAAGAGCTGCGGACCGAAATGTCC
TTCATAAGCGGGATGAAGGACAAAAGGAGGCGAACCGCAGGCTGAAATGTCC
TTCATAAGCGGGATGAAAGGACAATAGGGGGCGAACTGCAGTCAGAAATGTCCTTCATAAGCG
> ATGAAAGACA Con:1 reps=5 [975899,976117] l-int=219 l-seq = 52
ATGAAAGACATTTTAAAGGGTGGCGGGCGCCTATTTGTCCTTCACCGGTTTG
ATGAAAGACAAAACAAAGCGCGACTGCTTGCCATTTGTCCCTCATCGATGCG
ATGAAAGACAAAACAAGGTATGACGGGTATCGATTTGTCCTTCATCACCCCG
ATGAAAGACATTTTATAAGATGGCTGCCAGTATTCTGTCCTTCACCGGCATAATGAAAGACA
> AAGGACAAAA Con:1 reps=10 [1089498,1090080] l-int=583 l-seq = 52
AAGGACAAAATGTATGCGTGTGACCGGGGAAATGTCCTTCATTGCGGCGGGA
TGAACAAGAGGTATGCGTGTGAGGCGGCGAACTGTCCTTTTGAACCTGTCAA
AAGGACAAAACAGGATGCACCCGGGCCCGAAATGTCTTTTTAAAGCCGCCAA
AAGGACAAAAGGAGTGGCGATTGGGCTCGAAATGTCTTTTTGGGGCTGTCAA
AAGGACAAAACAGGCTGCATTCGGGCTCGAAATGTCTTTTTGAGCTTGTCAA
AAGGACAAAACAGGCTGGATCAGGGGTCAAACTGTCTTTTTGAAGCCGTCAA
AAGGACAAAAGGAGTCGCGATTGGGCTCGAACTGTCTTTTTGGGGCTGTCAA
AAGGACAAAACATGTTGCGTCCGGGGCCAAACTGTCTTTTTGAACCTGTCAA
AAAGACAAAACAGGCTGCGTCCGGGTTCAAACTGTCTTTTTGATGCCGCCAA
AAGGACAAAAGGAGTGACGTTTGAGCCCGAAATGTCTTTTTGAGCTTGTCAA
AAGGACAAAACAGGCTGGATCAGGGGTCAAACTGTCTTTTTGAAGCCGTCAAAAGGACAAAA
> AAGGACAAAA Con:1 reps=14 [1091587,1092481] l-int=895 l-seq = 52
AAGGACAAAACATGTTGCGTCCGGGGCCAAACTGTCTTTTTGAACCTGTCAA
AAAGACAAAACAGGCTGCGTCCGGGTTCAAACTGTCTTTTTGATGCCGCCAA
AAGGACAAAAGGAGTGACGTTTGAGCCCGAAATGTCTTTTTGAACTTGTCAAA
AAGGACAGAACACGCTGCATACAGGCCCGAAACGTCCTTTTGAATCTGTAAA
AGGGAAAAAACGCATGCCCGAAACCGTTGAAATGTCCTTCATCGGGGTGATG
AAGGACAAAAGTGAGCCGCCAAACATTGAAATGTCTTTCATCGGGCAAATG
AAGGACAAAATGCAGGCCCGAGACCGTGAAAATGTCCTTCACCGGGTTGATG
AAGGACAAAACGCGAGCCCGAGACCGTGAAAATGTCTTTCACCGGGTTGATG
AAGGACAAAACGCGGGCCGCCAACCATTGAAACGTCCTTCATCGGGCAAATG
AAGGACAAAACGCATGCCCGAAACCGTTGAAATGTCCTTCATACGGCAAATG
AAGGACAAAACGCGAGCCCGAGACCGTGAAAATGTCTTTCACCGGGTTGATG
AAGGACAAAACGCATGCCCGAGACCATGAAAATGTCCTTCATCGAGGTAATG
AAGGACAAAACGCGTGCCACCACACATTAAAATGTCCTTCATTGGGTTAATG
AAGGACAAAACGCGTGCCACCACACATTAAAATGTCCTTCATTGGGTTAATG
AAGGACAAAACGCATGCCACCAACCATTGAAATGTCTTTAATAGGGCAGATG
AAAGACAAAACGCAGGCCCGAGACCGGGAAAATGTCCTTCACCGGGTTGATG
AAGGACAAAACACGAGCTCGAAACCGTAGAAATGTCCTTCATCGAGGTAATGAAGGACAAAA
> TGTTTTGTCC Con:1 reps=10 [1107902,1108435] l-int=534 l-seq = 52
TGTTTTGTCCTTTTAACGGCTTCATAAAGACATTTCAGACCCAGTCGTGACT
CCTTTTGTGCTTTTGGCGGCAGGTTCAAAAGGACATTTCAGGCCCGGAAGCAGCG
TGTTTTGTCCGTGTGGCAACTTCAAAAAGACATTTTGGACCCGGAAGCAGCG
TGTTTTGTCCTTTTGACGGTTACAAAAGGACATTTCGGGCCCGGAAGCAGCG
TGTTTTGTCCTTTTGGCAGGTTCAAAAAGACATTTCGAGCCCGGATGCAACA
TGTTTTGTCCTTTTGACGGCTTCAAAAGGACATTTCGAGCCCGGATGCAACA
TGTTTTGTCCTTTTGACGGCTTCAAAAGGACATTTCGGGCCCGGATACATCG
TGTTTTGTCCTTTTGACGGTTACAAAAGGACATTTCGGGCCCGGATACATCG
TGTTTTGTCCTTTTGACGGCTTCAAAAGGACATTTCGGATCCAGACGTAACG
TGTTTTGTCCTTTTGACGGCTTCAAAAAGACATTTCGAGCTCGGATGAAACGTGTTTTGTCC
> GCGTCTATGT Con:1 reps=5 [1175303,1175521] l-int=219 l-seq = 52
GCGTCTATGTTAAGGAAGCACAGAAAAATGAAGGTCTTTCTTCACGTATGGG
GCGTCTATGTTAAGGAAGCATGGAAAAAAGACGGTCTTTCTTCATGTATGGG
GCGTCTATGTTAAGGAAGCATGGAAAAATGAGTTTCTTTCTTCACGTAGAGG

GCGTCTATGTTAAGGAAGCGCGAAAAAACGACAGTCTTTCTTAACGTAGGGAGCGTCTATGT
> GTTAAGGAAG Con:1 reps=5 [1179203,1179473] l-int=271 l-seq = 52
GTTAAGGAAGCACGGAAAAAAGATGGTCTTTCTTCATCTAGAGGTCGTCTAC
GTTAAGGAAGCAAGCAAAAAAGGCGGCCTTTCTTTACGTAGAGGTCGTCTAT
GTTAAGGAAGCATGGAAAAAAGAAGGTCTTTCTTCACGTATGGGGCGTCTAT
GTTAAGGAAGCGCGAAAAAACGACAGTCTTTCTTAACGTAGGGAGCGTCTAT
GTTAAGAAAGCAAGCAAAAACGAGGTTCTTCCTTCACATAGAGCCCGCCTACGTTAAGGAAG
> CAAAAGGACA Con:1 reps=11 [1310475,1311005] l-int=531 l-seq = 52
CAAAAGGACAAAACACGCTGCATCCGGGCCCGAAATGTCTTTTTGAAGCTGC
CAAAAGGACAAAACACGTTGCATCTGGTCTCGGAATGTCCTTTTGAAGCTGC
CAAAAGGACAAAACGCGTTGCATCTGGGATCGGAATGTCCTTTTGAAGCAGT
CAAAAGGACAAAAGGAGCCGTGAACTGAAACAAAATGTCCTTTTGAAGCTGT
CAAAAGGACAAAAGGAGCCGTGGACCGGAGCAAAAAGTCCTTTTGGAGTATT
CAAAAGGACAAAAGGAGCCGTGGACCGGAGCAAAAAGTCCTTTTGGAGTATT
CAAAAGGACAAAAGGAGCCGTGAACCAGAGCAAAAAGTCCTTTTGGAGTATT
CAAAAGGACAAAAGGAGCCGTGAACTGAAACAAAATGTCCTTTTGAAGCTGT
CAAAAGGACAAAAGGAGCCGTGGACCGGAGCAAATTGTTCTTTTGGAGCATC
CAAAAGGACAAAAGGAGCCATTGACCGGAACAAAATGTCTTTTTGGAGTGTTCAAAAGGACA
> GTCTACGTTA Con:1 reps=5 [1334998,1335268] l-int=271 l-seq = 52
GTCTACGTTAAGAAAGCAAGCAAAAACGAAGGCCTTCCTTAACATAGAATGC
ATCTATATTAAGTAAGCGTGAAAAAACGAAGTCCTTTCTTCACATAGAGGAC
GTCTACGTTAAGGAAGTGCGGAAAAAAGACGGCCTTTTTTCACCTAGAGGGC
GTCTACGTTAAGGAAGCAAGCAAAAACAAAGTCCTTCCTTCACCTAGAGCCC
GTCTACGTTAAGGAAGCGTGAAAAAACAAAGTCCTTCCTTCACCTAGAGCCTGTCTACGTTA
> GTTAAGGAAG Con:1 reps=5 [1568001,1568271] l-int=271 l-seq = 52
GTTAAGGAAGCAAGCAAAAACGAGGCTCTTCCTTGACATAGAGCCCGTCTAT
GTTAAGGAAGCGTGGAAAAATTGGTGGCCTTCCTTAACATAGGGCGCATCTAC
GTTAAGGAAGCACGAAAAAACGAGGCTCTTCCTTCACATAGAGCGCGTATAT
GTTAAGGAAGCGCGAAAAATTGACGGCCTTTCTTCACATAGAGCGCGTCTAC
GTTAAGAAAGCGTGAAAAAACGAGGGTTTTCCTTCATATAGAGCCTGTCTACGTTAAGGAAG
> GATGTTTCTT Con:1 reps=5 [1642950,1643272] l-int=323 l-seq = 52
GATGTTTCTTAATGTAGAGTGCGTCTCCGTTAAGGAAGCGCGTGGAAAAAAC
GATGTTTCTTAACGTAGAGTGCGTCTAGGTGAAGGAAGCTGGGGCAAAAGTC
GGTATTTCTTAACGTAGAAGGCGTCTACGTGAAAGAAGCGCGGAAAAAAGAC
GATGTTCCTTAAAATAGAGTGCGTCTACGTTAAGAAAGCAGGCGCAAAAGAC
GATGTTTCTTAACGTAGAAGGTGTCTACGTTAAGGAAACGCGTGGAAAAAAC
GATGTTTCTTAACGTAGAGTGCGTCTATGTTAAGGAAGGGAGCGAAAAAATTGATGTTTCTT
> CTTAACGTAG Con:1 reps=6 [2233857,2234179] l-int=323 l-seq = 52
CTTAACGTAGACGGGGGCTATGGGAAGGAGGGACTTCGTTTTTGCTTGCTTC
CTTAACGTAGACGAGCTCTATGTTAAGAAAGACCTTCATTTTTCCGTGCTTC
CTTAACGTAGACGAGCTCTATGTTAAGGAAGACCTTCATTTTTGCGCGCTTT
CTTAACTTAGACGCGCTCTATGTGAAGGAAGGCCTTCGTTTTTCCAAGCTTC
CTTAACGTAGACACCCTCTATGTTAAGGAAGGCCTTCGTTTTTCCAAGCTTC
CTTAACGTAGATGGGCTCTATGTTAAGAAAGGCCTTCGTTTTTCCGTGCTTCCTTAACGTAG
> GCTTCCTTAA Con:1 reps=9 [2251337,2251815] l-int=479 l-seq = 52
GCTTCCTTAACGTAGACGCGCTCTACGTGAAGATAGACCGTCTTTTTTCCGC
GCTTCCTTAACATAGACACGCTCTATGTTAAGGAACAACCTCAATTTCCTTC
GCTTCCTTAACGTAGACGCACTCTATGTGAAGATAGACCGACTTTTTTCCGC
GCTTCCTTAACATAGACACGCTCTATGTTAAGGAACAACTTCAATTTCCTTC
GCTTCCTTAACGTAGACGCACTCTATGTGAAGATAGACCGACTTTTTTCCGC
GCTTCCTTAACATAGACACGCTCTATGTTAAGGAACAACTTCAATTTCCTTC
GCTTCCTTAACATAGACGCGCTCTATGTAAAGAAAGGACTCATTTTTTCCAC
GCTTTCTTAACGTAGACGGGCTCTAGGTTAAAGAAGAGGACCATTTTCCTTC
GCTTCCTTAACATAGACGCGCCCTATGTGAAGAAAGGCTGCCTTTTTTCCACGCTTCCTTAA
> GCTTCCTTAA Con:1 reps=6 [2366309,2366944] l-int=636 l-seq = 52
GCTTCCTTAACGTAGACGGGCTCTAGGTGAAAAAAGGCTTTCGTTTTTGCGC
GCTTCCTTCACGTAGACGCCCTCTAGGTGAAGGAAGAACCTCATTTTTGCGT
GCTTCCTTCACGTAGACGCGCCCTATGTGAAGGAAGGCCGTCTTTTTTCCGC
GCTTTCTTAACGTAGACGCCCTCTAGGTTAAGGAAGACCCTCAATTTTCCGC
GCTTCCTTAACGTAGACGGGCTCTATGTGAAGAAAGACCTTCGTTTTTCCGT
GCTTCCTTAACGTAGACGGGCTCTATGTGAAGAAAGACCTTCGTTTTTCCGC
GCTTCCTTAACATAGACGGGTTCTATGTTAAGAAAGGCCTTCGTTTTTCCAT
CCTTCCTTAATGTAGACGCCCTCTATGTAAAGCAAGAGCCTCGTTTTTGCTT
GCTTCCTTAACGTAAACGCCCTCTATGTAAAGAAAGGCCGTCTTTTTTCCGC
GCTTTCTTAACGTAGACGCACTCTAGGTTAAGGAAGATCCTCAATTTTTCGC
GTTTCCTTAGCGTAGACAGGCTCTAGATTAAGAAAGCGCCTTGTTTTTGCTT
GCTTTCTTAATGTAGACAGGCTCTAGGTGAAGAAAGGCCCATCTTTTTTCCGTGCTTCCTTAA
> GATGAAGGAC Con:1 reps=4 [2375388,2375659] l-int=272 l-seq = 52
GATGAAGGACAAAACATAGGTCGGGGAAGCGGCATATTGTCTTTCATCGGCGG
GATGAAAGACAAATCAAAGGTCGACAAGCGGAATATTGTCCTTCATAGGCGT
GATGAAAGACAAAACACGGGCCGGCAAACGGCGAATTGTCCTTCATAACTAT
GATGAAGGACAAAACATGGGCCCGCAAACGGCGAATTGTCCTTCATAACTAT
GATGAAGGACAATCACCTCCCAGCAAATAGCTAAATGTCTTTCATCGGCATGATGAAGGAC
> CTTAACGTAG Con:1 reps=6 [2399253,2399523] l-int=271 l-seq = 52
CTTAACGTAGACGCGCTCTATGTGAAGAAAGAGACTCGTTTTTGCTTGCTTC

CTTAA**CGTAGACGCG**CTCTATGTGAAGAAAGGCCTTCATTTTTC**CG**TGCTTC
CTTAA**CGTAGACG**CTCTCTATGTGAAGGAAG**CG**CCTC**CG**TTTTTCCA**CG**CTTC
CTTAA**CGTAGA**TGCACTCTATGTAAAGGAAG**CG**CCTC**CG**TTTTTC**CGCG**CTTC
CTTAA**CGTAGA**TACTCTCTATGTGA**CG**GAAGACCTTCATTTTTC**CGCG**CTTCCTTAA**CG**TAG
> GCTTCCTTAA Con:1 reps=6 [2405830,2406100] l-int=271 l-seq = 52
GCTTCCTTAA**CGTAGACG**CACCCTATGTTAAGATA**CG**CCTTCTTTTTTCCAT
GCTTCCTTAATGTAGA**CG**TGCTCTATGTTAAGGAACTA**CG**TCTTTTTTTCAC
GCTTCCTTAA**CGTAGACG**CATCCTATGTTAAGAAACATTTTCTTTTTTTCAC
GCTTCCTTAA**CGTAGA**CA**CG**CCCTATGTAAAAAAACAATGTCTTTTTTGCTC
GCTTCCTTAACATAGA**CGCG**CCCTATGTGAAGGAACATCTTCTTTTTTTCA**CG**CTTCCTTAA
> TGTCCTTTTG Con:1 reps=6 [2412501,2412771] l-int=271 l-seq = 52
TGTCCTTTTGAACCTCTTTAAAGGACAATTTGTGCTTTGTTTCTCCCTGAAA
TGTCCTTTTGAATCCCTGTAAAGGACAGTTTA**CG**CTT**CG**TTTCTACCTGAAT
TGTCCTTTTGAACCCTTCTAAAGGACATTTTGCTTTTTGTCTCTCCCTGAAT
TGTCCTTTTGGAACA**CG**CTAAAAGACAATTTGACCTTTGTCAGTTGC**CG**AAA
TGTCCTTTTGAATCCCTGTAAAAGACAATTTGCTCTT**CG**GCTTTCCCTAAATTGTCCTTTTG
> TTTCAT**CG**GC Con:1 reps=4 [2493743,2493909] l-int=167 l-seq = 52
TTTCAT**CG**GCTCTATGAAGGGCAGTTCACTGTTTCC**CG**GCCAGTCTTTTGTT
TTTCAT**CG**GCTCTATGAAGGACAATT**CG**T**CG**TTTCC**CG**GCCAGTCTTTTGTT
TTTCAT**CG**GCTCTATGAAGGACAGTTCAC**CG**TTCATGGCC**CG**GTGTTTTGTCTTTCAT**CG**GC
> TGTCCTTCAT Con:1 reps=7 [2511339,2511869] l-int=531 l-seq = 52
TGTCCTTCAT**CG**GCTGTATGAAAGACAAAATCAATGGCTC**CG**TGCTC**CG**AAT
TGTCCTTCATCAATC**CG**ATGAAAGACAAATTTCAAATCCCAGAA**CG**G**CG**AAA
TGTCCTTCATAGACTGAATGAAGGACAAAATCAATGGATC**CG**TACCCAGAAT
TGTCCTTCATAAGCTGTATGAAAGACAAAATCAATGGCTCCATACCA**CG**ATT
TGTCCTGCATCAACC**CG**ATGAAAGACAAATTTCAGATCCCAGGA**CG**G**CG**AAA
TGTCTTTCATAAACTGAATGAAGGACAAAATCAAGGACTCCTTACCCCAAAT
TGTCCTTCATAAGCTGTATGAAAGACAAAAACAAAGGCTC**CG**TGCTC**CG**AAT
TGTCCTT**CG**TCAACC**CG**ATGAAGGACAAATTTCAAATCCCAGAA**CG**G**CG**AAA
TGTCTTTCATAGACTGAATGAAGGATAAAATCAAGGACTCCTTACCCCAAAT
TGTCCTTCAT**CG**TTTGTATGAAAGACAAAAA**CG**TGGG**CG**C**CG**CTCCCCAAATTGTCCTTCAT
> TGAAGGACAT Con:1 reps=4 [2533354,2533520] l-int=167 l-seq = 52
TGAAGGACATTTTATGCTTTCAGGCACAGTGGTTTGTCTTTCATGATCTGGA
TGAAGGACATTT**CG**CTGGTTCT**CG**GCTCATGTTTTGTCTTTCAT**CG**CCCTGA
TGAAGGACATTT**CG**C**CG**GTTCT**CG**GCA**CGCG**TTTTGTCTTTCATTACCT**CG**ATGAAGGACAT
> TTAA**CGTAGA** Con:1 reps=5 [2557529,2557747] l-int=219 l-seq = 52
TTAA**CGTAGACGCG**CTCTATGTGAAGGAAGGCCTT**CG**TTTTTCCA**CG**CTTCC
TTAA**CGTAGACGCG**CTCTATGTGAAGGAAGGCCTT**CG**TTTTTCCA**CG**CTTCC
TTAA**CGTAGACG**TGCCCTATGTGAAGGAAGACCCT**CG**TTTTTGCTTGCTTCC
TTAA**CGTAGACG**GGGTCTATGTTAAGGAAGGGCTT**CG**TTTTTCCATGCTTCCTTAA**CGTAGA**
> TTTGTCCTTT Con:1 reps=8 [2559923,2560297] l-int=375 l-seq = 52
TTTGTCCTTTTGA**CG**GCTTCAAAAGGACATTT**CG**GGCT**CG**GATGCAA**CG**TGT
TTTGTCCTTTTGA**CG**GCTTCAAAGGGACATTT**CG**AGCC**CG**GATGCTGCTCCT
TTTGTCCTTTTGATAGCTTCAAAAAGACATTTCAGACC**CG**GATGCTGCTCCT
TTTGTCCTTTTGATAGCTTCAAAAGGACATTT**CG**AGCC**CG**GCTGAAGCTCCT
TTTGTCCTTTTGGCAGTTTCAAAAGGACATTT**CG**GGTCTGGATGCAA**CG**TGT
TTTGTCCTTTTGACAGCTTCAAAAAGACATTTTGGGCC**CG**GGTGGAGGTCAA
TTTGTCCTTTTGA**CG**GCTTCAAAAGGACATTTCAGACC**CG**GATGCAA**CG**TGTTTTGTCCTTT
> TTTTGTCTTT Con:1 reps=11 [2601680,2602405] l-int=726 l-seq = 52
TTTTGTCTTTCATAGATCCAATGAAGGACATTTCTTT**CG**CCCTGGTGCCTCA
TTTTGTCTTTCATAAGCTTGATGAAGGACACTTCTCTGGTTAT**CG**GTCCCTG
TTTTGTCTTTCATAACCTTATTGAAGGACATTTCCTTGTTTCTCAGCC**CG**TG
TTTTGTCTTTCATGAACCCTATGAAGGACATTTCCTT**CG**TCC**CG**G**CG**CCTCT
TTTTGTCTTTCATGAACCTTATGAAGGACATTTCCTT**CG**TCC**CG**G**CG**CCT**CG**
TTTTGTCTTTCATGGAAC**CG**ATGAAGGACATTTCTTT**CG**TCT**CG**ACTCCC**CG**
TTTTGTCTTTCATGAACCCTATGAAGGACATTTCCTT**CG**TTCCAGAGCCT**CG**
TTTTGTCCTTCATAAGCT**CG**ATGAAGGACATTTTCTTCATCCAGG**CG**CCT**CG**
TTTTGTCTTTCATAAGCCCAGTGAAGGACATTTTCTT**CG**TCC**CG**ACTCCC**CG**
TTTTGTCTTTCATGAACCCTATGAAGGACATTTCTTTCACCT**CG**GCCCCT**CG**TTTTGTCTTT
> GATGAAGGAC Con:1 reps=9 [2756365,2756895] l-int=531 l-seq = 52
GATGAAGGACAAAAGGAGA**CG**CACCCCAAGCTGAAATGTCCTTCATAAA**CG**G
GATGAAAGACAAAAGGGAA**CG**AAC**CGCG**GGATGAAATGTCCTTCATAAA**CG**G
GATGAAGGACAAAAAGGGG**CG**AACTGCAAGCTGAAATGTCCTTCATAAA**CG**G
GATGAAAGACAAAAGGAGG**CG**ATC**CGCG**GGATGAAATGTCCTTCATAACTGG
GATGAAGGACAAAAGGGAA**CG**AAC**CGCG**GGATGAAATGTCCTTCATAAA**CG**G
GATGAAGGACAAAAAGGGG**CG**AACTGCAAGCTGAAATGTCCTTCATAAACAG
GATGAAGGACAAAAGGGGA**CG**ATC**CGCG**GGATGAAATGTCTTTCATAACAGG
GATGAAGGACAAAAGGAGA**CG**CACCCCAAGCTGAAATGTCCTTCATAAACAC
GATGAAGGACAAAAGGGGA**CG**ATC**CGCG**GGATGAAATGTCTTTCATAACAAG
GATGAAGGACAAAAGGAGA**CG**CACCCCAAGCTGAAATGTCCTTCATAAACA**CG**ATGAAGGAC
> AAAAGACATT Con:1 reps=18 [2756984,2757881] l-int=898 l-seq = 52
AAAAGACATTTCAACATGGGA**CG**GGCATGCTTTTGTCTTTTTG**CG**CCTTTGT
AAAAGACATTTCAACCAGCAT**CG**TGCACCCTTTTGTCCTTTTAAACCTCTAT
AAAAGACATTT**CG**AC**CG**GCAT**CG**TGCACCCTTTTGTCCTTTTGAACCCTTGT
AAAAGACATTTCAAC**CG**GCCACATGCACCCTTTTGTCCTTTTGAACCTCTGT

AAAAGACATTTCAACCGGCCACATGCACCCTTTTGTCCTTTTGCGCCCCTCT
AAAAGACATTTCAACCGGCAACGTGCCCTCTTTTGTCCTTTTGAGCCTTTGT
AAAAGACATTTCAACCGGCCACGTGCACCCTTTTGTCCTTTTGAACCTCTGT
AAAAGACATTTCAACCGGCCACATGCACCCTTTTGTCCTTTTGAACCTCTGT
AAAAGACATTTCAACCGGCCACATGCACCCTTTTGTCCTTTTAAACCTCTAT
AAAAGACATTTCGACCGGCATCGTGCACCCTTTTGTCCTTTTGTGCCCCTATAA
AAAAGACATTTCGACCGGCATCGTGCACCCTTTTGTCCTTTTGAACCCTTGT
AAAAGACATTTCAACCGGCAACGTGTCCCCTTTTGTCCTTTTGCGCCATTGT
AAAAGACATTTCGACCGGCCACGTGTCCCCTTTTGTCCTTTTGCGCCCCTCT
AAAAGACATTTCAACCAGCTTCGTGCACCCTTTTGTCCTTTTGAACCTCTGT
AAAAGACATTTCAACCGGCCACATGCACCCTTTTGTCCTTTTGTGCCCCTATAA
AAAAGACATTTCGACCGGCATCGTGCACCCTTTTGTCCTTTTGCGCCATTGT
AAAAGACATTTCGACCGGCCACGTGTCCCTTTTGTCCTTTTGCGCCCCTCTAAAAGACATT
> TAAAAGACAT Con:1 reps=7 [2759662,2759984] l-int=323 l-seq = 52
TAAAAGACATTTCAACCAGCTTCGTGCACCCTTTTGTCCTTTTGAGCCTCTA
TAAAAGACATTTCAACCAGCTTCGTGCACCCTTTTGTCCTTTTGAGCCTCTA
TAAAAGACATTTCAACCGGCCACGTGTCCCCTTTTGTCCTTTTGCGCCTCTA
TAAAAGACATTTCAACCGGCCACATGCCCCCTTTTGTCCTTTTGAACCCTTG
TAAAAGACATTTCAACCGGCAACGTGTCCCCTTTTGTCCTTTTGCACCTTTG
TAAAAGACATTTCAACCAGCATCGTGCACCACTTTTGTCCTTTTGAGCCCTATAAAAGACAT
> GTCTAATTAG Con:1 reps=5 [2867866,2868084] l-int=219 l-seq = 52
GTCTAATTAGCTTTTTCAATTAGATAAAAGCAGATGAGTGCCACGATCATTT
GTCTAATTAGCGTTTCCAATTAGACAATTGCAGATGAGCGGCGTGGTCATTT
GTCTAATTAGAACCGCCAATTGGACATTTGCAGGAGGACTGCACGCTCATTC
GTCTAATTAGCGTTTCTAATTAGACAATTGCAGGTGGGCGGCGCGGCCTTTTGTCTAATTAG
> CTATGTTAAG Con:1 reps=9 [2952075,2952501] l-int=427 l-seq = 52
CTATGTTAAGGAACAAGGCAATTTTTGCTCGCTTCCTTTACGTAGACGGGCT
CTATGTTAAGGAACAACGCCTTTTGACCTCGCTTCCTCAACATAGACGGCCT
CTATGTTAAGGAACAACGTCTTTTTTGCTCGCTTTCTTAACGTAGACGCGCT
CTATGTTAAGAAACAACACTTTTTTTGCTCGCTTCCTTAATGTAGACGTGCA
CTATGTTAAGGAACAATGTCTTTTTACCTCGCTTCCTTTACGTAGACAGCCT
CTATGTTAAGAAACAACACCTTTTTTGCTCGCTTTCTTCACGTAGACGTACT
CTATGTTAAGGAACAACGCAATTTTCGCTCGCTTCCTTAACGTAGCCGACCT
CTATGTTAAGAAACAAAGCTATTTTCGCTCGCTTTCTTAACGTAGACGCGCTCTATGTTAAG
> CTTAACGTAG Con:1 reps=7 [2987594,2988175] l-int=582 l-seq = 52
CTTAACGTAGACGCTTTCTATGTGAAGGAAGGCCTTCGTTTTTCCGCGCTTT
CTTAACGTAGACGCCTCCTATGTGAAGGAAGACCGTCATTTTTCCACGCTTC
CTTAACATAGACGGACTCTATGTGAAGGAACAACGCACTTTTTGCTCGGTTC
CTTAACGTAGGCGCACTCTATATTAAGGAAGACCTTCATTTTTCCACGCTTC
CTTAACATAGACGGACTCTATGTAAAGGAAGAGCCTCGTTTTATCGTGCTTT
CTTAACATAGACGCGCCCTATATGAAGAAAGGACGTCATTTTTCTATGATTC
CTTAACATGACAGGCTCTATGTTAAGGAACAACGCATTTTTTTCTCGATTC
CTTAACGTAGACGCCTTCTATGTTAAGGAAGACCTTCATTTTTCCACGCTTC
CTTAACGTAGACGCGCTCTATGTGAAGGAAGAGCCTCGTTTTATCGTGCTTC
CTTAACGTAGACGCACTCTATGTTAAGGAAGGCCTTCATTTTTCCACGCTTT
CTTAACATAGACAGGCTCTATGTGAAGAAAGTCCTTCGTTTTTCCGTGCTTTCTTAACGTAG
> TCTACGTTAA Con:1 reps=15 [3137925,3138715] l-int=791 l-seq = 52
TCTACGTTAAGGAAGCGTGCGAAAATGGCGTTGTTCCTTTACATAGAGGCTA
TCTACGTTAAGGAAGCATGGAAAAATTGTCTTGTTCCTGAACATAGAGGGCG
TCTACGTTAAGAAAGCATGAAAAATTGGCCTCATTCCTTCGCATGAAGCATG
TCTACGTTAAGAAAGCGTGAAAAAATTGCCTCGTTCCTTTACATAGAGCATG
TCTACGTTAAGGAAGCATGAAAAATTGGCCTCGTTCCTTCACATAGAGCATG
TCTACGTTAAGGAAGCGTGCGAAATTGGCCTCGTTCCTTCACATAGAGCATG
TCTATGTTAAGGAAGCGTGCGAAATTGGCATTGTTCCTTCACATAGAGGGCG
TCTACGTTAAGAAAGCATGAAAAATTGGCCTTGTTCCTTCACATAGAGGCCG
TCTACGTTAAGAAAGCATGAAAAATTGGCCTCGTTCCTTCACATAGAGCATG
TCTACGTTAAGGAAGCGTGCGAAAATGGCATTGTTCCTTAACATAGAGGGCG
TCTACGTTAAGAAAGCATGAAAAATTGGCCTTGTTCCTTCACATAGAGGCCG
TCTACGTTAAGAAAGCATGAAAAATTGGCCTCGTTCCTTCACATAGAGCATG
TCTACGTTAAGAAAGCATGAAAAATTGGCCTCGTTCCTTTACATAGAGGCTG
TCTACGTTAAGAAAGCATGGAAAAATTGTCTTGTTCCTGAACATAGAGGGCG
TCTACGTTAAGGAAGCGCGCAAAAATGGCCTTGTTCCTTCACATAGAGGGCGTCTACGTTAA
> TTTTGTCCTT Con:1 reps=4 [3150825,3150991] l-int=167 l-seq = 52
TTTTGTCCTTCATTACCTCGATGAAAGACATTCCGAGCCGCGAGCAAATCTG
TTTTGTCCTTCATTGACTCTCTGAAGGACATTTCCGGACTCAACCAAACGCC
TTTTGTCCTTCATAACCTCGATGAAAGACATCCCCAGCCACGGCCAACTCGGTTTTGTCCTT
> TCGCTTCCTT Con:1 reps=5 [3159024,3159294] l-int=271 l-seq = 52
TCGCTTCCTTAACGTAGACGCCTTCTAGGTTAAGATAGAGCCGCAATTTCCT
TCGCTTCCTTTACATAGACGCACTCTATGTTAAGAAAGAACCCCGATTTGCT
TTGCTTCCTTTACATAGACGGCTTCTATGTTAAGATATAGCTGCGATTTTTC
TCGCTTCCTTAACGTAGACGCCTTCTATGTTAAGATAGAACCACAGTTTTTC
TCGCTTCCTTAACGTAGACGCACTCTACGTTAAGAAAGACCTGCAATTTTTCTCGCTTCCTT
> GTGAAGGAAC Con:1 reps=4 [3165204,3165422] l-int=219 l-seq = 52
GTGAAGGAACAACGGCTTTTTCACTCTCTTCCTTAACGTAGACGTTCTCTAT
GTTAAGGAACAACGGCTTTTTTGCATGCTTCCTTAACGTAGACGGCCTCTAC

```
GTGAAGGAACAAGGCAATTTTTGCTCGCTTCCTTAACGTAGATGGCCTCTAT
GTGAAGGAACAGCGACTTTTTACCTCGCTTCCCTAACGTAGACAACCTCTATGTGAAGGAAC
> TTTTGTCTTT Con:1 reps=9 [3215264,3215690] l-int=427 l-seq = 52
TTTTGTCTTTTTAAGCCCTCCAAAAGGACAATTGGCCCCGGGTCTGGCTCTC
TTTTGTCTTTTTGAAGCCCCCAAAAGGACAATTCGAACGGATTCCAGTTCCC
TTTTGTCTTTTTAAACCCTCCAAAAGGACAATTCACTCGGATTACAGCTCTC
TTTTGTCTTTTTGAACCCTTCAAAAGGACAATTGGCCCCAGGTCTGGCTCTC
TTTTGTCTTTTTGAGGCACTCAAAAGGACAATTCGACCGGATTCCAGTTCCC
TTTTGTCTTTTTGAACCCTCCAAAAGGACAGTTCGACCGGATTTCAATCCCC
TTTTGTCTTTTTGAACCCTCCAAAAGGACAATTCGACCGGATTCCAGCTTTC
TTTTGTCTTTTTAAAGCCTCCAAAAGGACAATTGGCCCCGGGTCTGGCTCTCTTTTGTCTTT
> CTTAACGTAG Con:1 reps=8 [3382355,3382833] l-int=479 l-seq = 52
CTTAACGTAGATGCGGCCTATGTGAAGAAAGGCCGGCTTTTTCCCCCGCTTG
CTTCACTTAGAGGTAGTCTATGTGAAGGAAGAGATCCTTTTTGGCTCGGTTT
CTTAATGTGGACGCGCTCTATGTTAAGAAACAAGCTCATTTTCCTCCGCTTC
CTTAACGTAGACGCGCTCTATGTTAAGAAACTATCTCATTTTCCCTCGCTTC
CTTAACGTAGACGCGCTCTATGTTAAGAAACAAGCCCATTTTCCTCCGCTTC
CTTAACGTAGACGGGCTCTAAGTTAAGAAACAAGCCCATTTTCCTCCGCTTC
CTTAACGTAGACGCGCTCTATGTGAAGGAAGCACCTCATTTTCCCTCGCTTC
CTTAACGTAGACGTGCTCCATGTTAAGAAACTATCTCATTTTCCCTCGCTTC
CTTAACGTAGACGCGCTCTATGTTAAGAAACAAGCTCATTTTCCTCCGCTTCCTTAACGTAG
> TTTTGGCTTT Con:1 reps=11 [3474529,3475266] l-int=738 l-seq = 52
TTTTGGCTTTTAAGATGTTCTAAAGGACAGTTTGTTTGATTCACAGGCGCT
TTTTGGCTTTTTGAGATGTTCTAAAAGACAGTTTGGACGCTGTCAAGGTGTC
TTTTGGCTTTTTGAGACGCTCTAAAGGACAATTGGCACGTTTCCCCAGCACC
TTTTGGCTTTTTGAAACGTTCTAAAGGACAGTTTGGATGATTTCCAGGTGCA
TTTTGGCTTTTTGAGATGCTCTAAAAGACAGTTTGGACGCTACCAAGGTGTC
TTTTGGCTTTTTGAGATGTTCTAAAAGACAATTTGAACGCTGCCTAGGTTTC
TTTTGGCTTTTTGGAACGTTCTAAAGGACAGTTTGGATGATTTCCAGGTGCA
TTTCGTCTTTTTGAGATGCTCTAAAAGACAATTTGGACGCTGCCTAGGTGTC
TTTTGGCTTTTTGAAACGCTCTAAAGGACAATTGGCACGTTTCCCCAGCATC
TTTTGTCTTTTTGAAACGTTCTAAAGGACAATTGGGCTGATTTCCAGGTGCG
TTTTGGCTTTTTGAGATGCTCTAAAGGACAGTTTGGACGCTACCAAGGTGTC
TTTTGGCTTTTTGAGACGCTCTAAAGGACAATTAGCACGTTCCCCGAGCACC
TTTTGTCTTTTTGAAACGTTCTAAAGGACAGTTTGGATGATTTCCAGGTGCA
TTTCGTCTTTTTGAGATGCTCTAAAAGACAATTTGGACGCTGCCTAGGTGTCTTTTGGCTTT
> ATGAAGGACA Con:1 reps=7 [3475558,3475933] l-int=376 l-seq = 52
ATGAAGGACAAAACAGAGACCATCTCCGACCCCAAATGTCTTTCATCCGCTCC
ATGAAGGACAAAACAAACTCGCCATAACCCTAAAATGTCCTTCATTCACCCG
ATGAAGGACAAATCAAAGACATTACAGACCCCAAATGTCTTTCATCTGCCCA
ATGAAGGACAAAACAAACTCACCATAACCCTAAAATGTCCTTCATTCACCCA
ATGAAGGACAAATCAAAGACATCACAGAGCCCAAATGTCTTTCATCTGCCCC
ATGAAGGACAAAATAAAGTCGCCACAACCCCAAATTGTCCTTCATGCGCCAG
ATGAAAGACAAAAACAGGCTACCATCACTTTAAAATGTCCTTCATCCACTTCATGAAGGACA
> CTATGTGAAG Con:1 reps=6 [3502875,3503248] l-int=374 l-seq = 52
CTATGTGAAGGAAGACCTTCATTTTTCCGTGCTTCCTAACGTAGACAGCCT
CTATGTGAAGAAAGGCCGTCAATTTTTTGTTCTTCCTTAACATAGACACGCC
CTAGGTTAAGGAAGGCCTTCATTTTTCTGCGCTTCCTTAACGTAGACGCCCT
CTATGTGAAGAAAGGCCTTCGTTTTTTCGTGTTTCCTTAACGTAGACAGCCT
CTATGTAAAGGAACAACGTCATTTTTTCACGCTTCCTTAACTTAGACGCGAC
CTATGTGAAGAAAAGCCTTCAATTTTCCGCGCTTCCTTAACGTAGACGGGCT
CTATGTGAAGGAAGACCTTCATTTTTTCGTGCTTCCTTAGCGTAGACGCGCCCTATGTGAAG
> GTTAAGGAAG Con:1 reps=6 [3514468,3514791] l-int=324 l-seq = 52
GTTAAGGAAGAGACTCATTTTTCACCGCGTTCTTAACGTAGACGCGTTCTAA
GTTAAGGAAGGAAACCCATTTCCTCCGTTTCCTTAACGTAGACGCGTTCTAG
GTTAAGGAAGGGAACCCATTTCCTTCGCTTTCTTAACGTAGAAGCACTCTAG
GTTAAGGAAGAGGACCCCATTTCCTTCGCTTCCTTAACGTAGGCGCACTCTAG
GTTAAGGAAGACCCTCATTTTCCTTCGCTTCCTTAATGTAGACGCGCTCTAG
> GCTTCCTTAA Con:1 reps=8 [3567366,3567793] l-int=428 l-seq = 52
GCTTCCTTAACGTAGACAGGCTCTAGGTTGAAGAAAGCGCCTCGTTTTTGCTT
GCTTCCTTAACGTAGACGCACTCTATGTGAAGGAAGACCTTCGTTTTTCTAC
GCTTCCTTAACGTAGACGCACTCTATGTGAAGGAAGACCTTCGTTTTTCCGC
GCTTCCTTAACGTAGACGCACTCTATGTGAAGGAAGACCTTCGTTTTTCTAC
GCTTCCTTAACATAGACGAGCTCTACGTGAAGAAAGGTCGCCTTTTTTCCAC
GCTTCTTTAACATAGACAGGCTCTACGTGAAGGAAGACCTTCATTTTTCCAC
GCTTCCTTAACATAGACGGGCTCTAGGTGAAGAAAGCGCCTCGTTTTTGCTT
GCTTCCTTAACGTAGACGGGCTCTACGTGAAGAAAGGCCTTCGTTTTTCTGCGCTTCCTTAA
```

***B.alkalitelluris* (NZ KV917374.1). All tandem repeats with repeat 51-52**

Practically all tandem repeats have two short palindromic signals in each 52 nt repeat. There are three main signals, shown in different colors:
AAAgGGAAT, **AAAGCTGTC**, **AATCATAG**
Only part of the repeat is highlighted.

```
> GCTGTCCGAA Con:1 reps=14 [115528,116213] l-int=686 l-seq = 52
GCTGTCCGAAGTTGGTTGAGGTTCAGACAGCTTGAGGATCAAAAGAGAGAAA
GCTGTCCGAACTTGGCTGAGGTTCAGACAGCTTGGGAGACACAAGAGAGAAA
GCTGTCCGAACTTGGCTGAGGTTCAGACAGCTTGAGGATCAAAAGAAAGAAA
GCTGTCCGAATTAGCTAAGGTTCAGACAGCTTAAGGATCAAAAGAGGGAAA
GCTGTCCGAACTTGGATGAGGTTCAAACAGCTTGAGGGAGAACACAGATAAA
GCTGTCCGAACTTAGCTTAGGTTCAGACAGCTTGAGGGAGAAAACAGATAAA
GCTGTCCGAACTTGGCTTAGGTTCAGACAGCTTGGGAGACAAAAGAGAGAAA
GCTGTCCGAACTTGGCTGAGGTTCAGACAGCTTGGGAGACACAAGAGAGAAA
GCTGTCCGAACTTGGCTGAGGTTCAGACAGCTTAAGGATCAAAAGAGGGAAA
GCTGTCCGAACTTAGCTTAGGTTCAGACAGCTTGAGGGAGAACACAGAGAAA
GCTGTCCGAACTTGGCTGAGGTTCAGACAGCTTAAGGCTCAAAAGAGGGAAA
GCTGTCCGAACTTAGCTTAGGTTCAGACAGCTTGAGAAAGAAAACAGATAAA
GCTGTCCGAACTTGGCTTGAATTCAGACAGCTTGAACGAGAAAACATGTAATGCTGTCCGAA
> ACTTCAGACA Con:1 reps=8 [123004,123378] l-int=375 l-seq = 52
ACTTCAGACAACTTCTGCAACCAAGAACCTAAAGCTGTCCGAACTCTTGCCT
ACTTCAGACAACTTCTGCTGCCAATTGCTTAAACCTGTCCGAACTCTTGCCT
ACTTCAGACAACTTCTGCCGCCAATTGCTTAAACCTGTCCGAACTCTTGCCT
ACTTCAGACAACTTCTGCCGCCAATTACTTAAACCTGTCCGAACTCTTGCCT
ACTTCAGACAACTTCTGCCGCCAATTACTTAAACCTGTCCGAACTCTTAACT
ACTTCAGACAGCTTCACCGACCAATTACCTAAAGCTGTCCGAACTCTTGCCT
ACTTCAGACAACTTCAGCAACCAAGAACCTAAAGCTGTCCGAACTCTTGCCTACTTCAGACA
> GAGCAACTTG Con:1 reps=9 [208479,209009] l-int=531 l-seq = 52
GAGCAACTTGATCGTAGAACTCCTATGATTCACTTCCCTACAATTAATGTGC
GAGCAACTTGATGATAGACCTACTATGATTGACTTCCTTACAAATCATGTGC
GAGCAACTTGATGATAGACCTACTATGATTGACTTCCTTACAAATCATGTGC
GAGCAACTTGATCGTAGACCTCCTATGATTCACTTCCCTACAATTCATGTGC
GAGCAACTTGATCATAGAACTCCTTTGATTCACTTCCTTACAATTCATGTGT
GAGCAACTTGATCATAGAACTCCTATGATTCATTTCCCTACAATTAATGTGC
GAGCAACTTGATCATAGACCTCCTATGATTCACTTCCCTACAATTAATGTGC
GAGCAACTTGGTCATAGACCTCCTATGATTCACTTACCTATAATTAATGTGC
AAGCAACTTGATCATAGACCTCCTATGATTCACTTCCCTACAATTAATGTGC
GAACAACTTGATCATAGACCTCCTATGATTCACTTCCCTACAATTCATGTGCGAGCAACTTG
> CTTCGGACAG Con:1 reps=14 [287166,287852] l-int=687 l-seq = 52
CTTCGGACAGCTTTAAAGGCTTGGCTCCTCAACCTGTCTGAACTTACTCTGA
CTTCGGACAGCCTTAACCGCTGGACTCCTCAACCGGTCTGAACTTACTCTAT
CTTCGGACAGCTTTTCCCGTGTGGCTCCTCAACCTGTCTGAACTTACTCTGA
CTTCGGACAGCTTTAACCGCTGGACTCCTCAACCTGTCTGAACTTACTCTAT
CTTCGGACAGCTTTTCCCGTGTGGCTCCTCAACCTGTCTGAACTTACTCTAT
CTTCGGACAGCTTTTCCCGTTTGGCTCCTCAACCTGTCTGTACTTACTCTGA
CTTCGGACAGCTTTAACCGCTGGACTCCTCAACCTGTCTGAACTTACTCTGA
CTTCGGACAGCTTTAACCGCTTGGCTCCTCAACCTGTCTGAACTTACTCTGA
CTTCGGACAGCCTTAACCGCTGGACTCCTCAACCGGTCTGAACTTACTCTAT
CTTCGGACAGCTTTTCCCGTGTGGCTCCTCAACCTGTCTGAACTTACTCTGA
CTTCGGACAGCCTTAACCGCTGGACTCCTCAACCGGTCTGAACTTACTCTAT
CTTCGGACAGCTTTTCCCGTGTGGCTCCTCAACCTGTCTGTACTTACTCTGA
CTTCGGACAGCCTTAACCGCTGGACTCCTCAACCGGTCTGAACTTACTCTGACTTCGGACAG
> TGTTTGATTC Con:1 reps=11 [341445,341974] l-int=530 l-seq = 52
TGTTTGATTCCCTTTCTCATGCCCAAAGCCCAACAAAGGGAATGAAAGGCAC
TGTTTGATTCCCTTTCTCATGCAAAAATCCAACAAAGGGAATGAAAGGCAT
TGTTTGATTCCCTTTCTCATGCCAAAAGCCCAACAAAGGGAATGAAAGGCAT
TGTTTGATTCCCTTTCTCATGTCAAAAGCCCAACAAAGGGAATGAAAGGCAT
TGTTTGATTCCCTTTCTCATGCCAAAAGCCCAACAAAGGGAATGAAAGGCAT
TGTTTGATTCCCTTTCTCATGCCAAAAGCCCAACAAAGGGAATGAAAGGCAT
TGTTTGATTCCCTTTCTCATGCCAAAAGCCCAACAAAGGGAATGAAAGGCAT
TGTTTGATTCCCTTTCTCATGCCAAAAGCCCAACAAAGGGAATGAAAGGCAT
```

TGTTTGATT**CCCTTT**CTCATGCCAAAAGCCCAAC**AAAGGG**AATGAAAGGCAT
TGTTTGATT**CCCTTT**CTCATGCCAAAAGCCCAAC**AAAGGG**AATGAAAGGCATTGTTTGATTC
> TTCTGATTCC Con:1 reps=4 [502640,502805] l-int=166 l-seq = 52
TTCTGATTCCCTTCCTCATGCCAAAGAAGCAAG**AAAGGG**AACGAAAGGAGCG
TTCTGATTCCCTTCCTCATGCCAAAGAAGCAAG**AAAGGG**AACGAAAGGAGTA
TTCTGATTCCCTTCCTCATGCCAAAAATCAAG**AAAGGG**AACGAAAGAAGTATTCTGATTCC
> AGGGAACGAA Con:1 reps=8 [542786,543160] l-int=375 l-seq = 52
AGGGAACGAAAGACGTTGTCAGATTCCCTTGCTGGTGGCAAAAGTCGAAAAA
AGGGAACGAAAGGT**GCTGTC**AGATTCCTTTACTGGTGCTAAAAGTCGAAGAA
AGGGAACGAAAGGT**GCTGTC**AGATTCCTTTACTGGTGCTAAAAGTCGAAGAA
AGGGAACGAAAGGT**GCTGTC**AGATTCCTTTACTGGTGCTAAAAGTCGAAGAA
AGGGAACGAAAGGT**GCTGTC**AGATTCCTTTACTGGTGCTAAAAGTCGAAGAA
AGGGAACGAAAGACGTTGTCAGATTCCCTTGCTGATGTCAAAAGTCGAAGAA
AGGGAACGAAAGACGTTGTCAGATTCCCTTGCTGATGCTAAAAGTCGAAG**AAAGGG**AACGAA
> T**CTATGA**TCC Con:1 reps=4 [546114,546280] l-int=167 l-seq = 52
T**CTATGA**TCCAGTTGCCAAGGCGAAAGCAAGGAGAAAGTAAA**TCATAG**GCCT
T**CTATGA**TCCAGTTGCCTGAGCGAAAACAAGGAGAAAATGAA**TCATAG**ACCT
T**CTATGA**TCCAGTTGCCTGAGCGAAAACAAGGAGAAAGTGAA**TCATAG**ACCTT**CTATGA**TCC
> TGTCTGAAGT Con:1 reps=36 [579578,581412] l-int=1835 l-seq = 52
TGTCTGAAGTAGCTTTGGGTTCG**GACAGC**TTTGATTGTTTTGAAGTAAAAGT
TGTCTGAAGTAGCTTCGGGTTCG**GACAGC**TTTGATTGTTTTGAAGCAAAAGC
TGTCTGAAGTAGCTTCGGGTTCG**GACAGC**TTTGTTGGTTTGTTTGTGAAAGC
TGTCTGAAGTAGCCCCGTGTTCG**GACAGC**TTTGATTGTTTAGAAGTAAAAGC
TGTCTGAAGTAGCCTCGAGTTCG**GACAGC**TTTGATTGGCTAGAAACAAAAGC
TGTCTGAAGTAGCCCCGTGTTCG**GACAGC**TTTGATTGTTTAGAAGTAAAAGC
TGTCTGAAGTAGCCCCGTGTTCG**GACAGC**TTTGATTGTTTAGAAGTAAAAGC
TGTCTGAAGTAGCCTCGAGTTCG**GACAGC**TTTGATTGGCTAGAAACAAAAGC
TGTCTGAAGTAGCCTCGAGTTCG**GACAGC**TTTGATTGTTTAGAAGTAAAAGC
TGTCTGAAGTAGCCTCGGGTTCG**GACAGC**TTTGATTGTTTAGAAGTAAAAGC
TGTCTGAAGTAGCCTCGGGTTCG**GACAGC**TTTGATTGTTTAGAAGTAAAAGC
TGTCTGAAGTAGCCCCGGGTTCG**GACAGC**TTTGATTGTTTAGAAGTAAAAGC
TGTCTGAAGTAGCCCCGTGTTCG**GACAGC**TTTGATTGTTTAGAAGTAAAAGC
TGTCTGAAGTAGCCTCGAGTTCG**GACAGC**TTTGATTGTTTAGAAGTAAAAGC
TGTCTGAAGTAGCCTCGAGTTCG**GACAGC**TTTGATTGTTTAGAGGTAAAAGC
TGTCTGAAGTAGCTTAGGGTTCG**GACAGC**ACTGTTAGTTTGTTTGTGAAAGC
TGTCTGAAGTAGCTTAGGGTTCGGACAGGTTTGATTGTTTAGAAGTAAAAGC
TGTCTGAAGTAGCCTCGAGTTCG**GACAGC**TTTGATTGTTTAGAAGTAAAAGC
TGTCTGAAGTAGCTTAGGGTTCG**GACAGC**TTTGATTGTTTAGAAGTAAAAGC
TGTCTGAAGTAGCCCCGTGTTCG**GACAGC**TTTGATTGTTTAGAAGTAAAAGC
TGTCTGAAGTAGCCTCGAGTTCG**GACAGC**TTTGATTGTTTAGAAGTAAAAGC
TGTCTGAAGTAGCCTCGAGTTCG**GACAGC**TTTGATTGTTTAGAGGTAAAAGC
TGTCTGAAGTAGCCTCGAGTTCGGCCAGCTTTGATTGTTTAGAAGTAAAAGC
TGTCTGAAGTAGCCTCGAGTTCG**GACAGC**TTTGATTGTTTAGAAGTAAAAGC
TGTCTGAAGTAGCCTCGAGTTCG**GACAGC**TTTGATTGTTTAGAAGTAAAAGC
TGTCTGAAGTAGCCTCGAGTTCG**GACAGC**TTTGATTGTTTAGAAGTAAAAGC
TGTCTGAAGTAGCCTCGAGTTCG**GACAGC**TTTGATTGTTTAGAGGTAAAAGC
TGTCTGAAGTAGCCTCGAGTTCG**GACAGC**TTTGATTGTTTAGAAGTAAAAGC
TGTCTGAAGTAGCTTCGGGTTCG**GACAGC**TTTGATTGTTTAGAAGCAAAAGC
TGTCTGAAGTAGCTTCAGGTTCG**GACAGC**TTTGATTGTTTAGAAGTAAAAGC
TGTCTGAAGTAGCTTCGGGTTCG**GACAGC**TTTGATTGTTTAGAAGTAAAAGT
TGTCTGAAGTAGCCTCGAGTTCG**GACAGC**TTTGATTGGCTAGAAACAAAAGC
TGTCTGAAGTAGCCCCGAGTTCG**GACAGC**TTTGATTGTTTAGAAGTAAAAGC
TGTCTGAAGTAGCTTCGGGTTCG**GACAGC**TTTGATTGTTTAGAAGCAAAAGT
TGTCTGAAGTAGCCCCGTGTTCG**GACAGC**TTTGATTGTTTAGAAGCAAAA**GCTGTC**TGTCTGAAGT
> G**AAAGGG**AAT Con:1 reps=7 [908246,908566] l-int=321 l-seq = 52
G**AAAGGG**AATCA**GACAGC**ATCTTTGAAT**CCCTTT**CTTGTGCTTTTTGCGAGA
G**AAAGGG**AATCA**GACAGC**ACCTTTGATTCCTTTTTTGGATTTTTGGTATGA
G**AAAGGG**AATCAGACAACACCTTTCATT**CCCTTT**CTTGGACTTTTTGCGTGA
G**AAAGGG**AATCA**GACAGC**ACCTTTCATT**CCCTTT**CTTGGGTTTTGGTATGG
G**AAAGGG**AATCA**GACAGC**ACCTTTGAAT**CCCTTT**CTTGGACTTTTTGCGTGA
G**AAAGGG**AATCAGACAACACCTTTCATT**CCCTTT**CTTGGGTTTTGGGTATGAG**AAAGGG**AAT
> AAGGGAATCA Con:1 reps=24 [953299,954484] l-int=1186 l-seq = 51
AAGGGAATCA**GACAGC**GTCTTTCGTT**CCCTTT**CTTCGACTTTTGGCATCAA
AAGGGAATCA**GACAGC**GCCTTTCGTT**CCCTTT**CTTTGACTTTTGGCATCAA
AAGGGAATCA**GACAGC**GTCTTTCGTT**CCCTTT**CTTCGACTTTTGGCACCAGT

AAGGGAATCA**GACAGC**GTCTTTCGTT**CCCTTT**CTTCGACTTTTGGCATCAA
AAGGGAATCA**GACAGC**GCCTTTCGTT**CCCTTT**CTTCGACTTTTGGCACCAA
AAGGGAATCA**GACAGC**GTCTTTCGTT**CCCTTT**CTTTGACTTTTGGCATCAA
AAGGGAATCA**GACAGC**GTCTTTCGTT**CCCTTT**CTTTGACTTTTGGCACCAA
AAGGGAATCA**GACAGC**GCCTTTCGTT**CCCTTT**CTTCGACTTTTGGCACCAA
AAGGGAATCA**GACAGC**GTCTTTCGTT**CCCTTT**CTTCGACTTTTGGCATCAA
AAGGGAATCA**GACAGC**GTCTTTCGTT**CCCTTT**CTTCGACTTTTGGCATCAA
AAGGGAATCA**GACAGC**GCCTTTCGTT**CCCTTT**CTTCGACTTTTGGCATCAA
AAGGGAATCA**GACAGC**GCCTTTCGTT**CCCTTT**CTTTGACTTTTCGCACCAGT
AAGGGAATCA**GACAGC**GCCTTTCGTT**CCCTTT**CTTCGACTTTTGGCACCAA
AAGGGAATCA**GACAGC**GTCTTTCGTTCGCTTTCTTCGACTTTTGGCATCAA
AAGGGAATCA**GACAGC**GTCTTTCGTT**CCCTTT**CTTTGACTATTGGCACCAA
AAGGGAATCAAACAGCGCCTTTCGTT**CCCTTT**CTTTGACTTTTGGCACCAA
AAGGGAATCA**GACAGC**GCCTTTCGTT**CCCTTT**CTTTGAATTTTGGCATCAA
AAGGGAATCA**GACAGC**GTCTTTCGTT**CCCTTT**CTTCGACTTTTGGCATCAA
AAGGGAATCA**GACAGC**GCCTTTCGTT**CCCTTT**CTTCGACTTTTGGCATCAA
AAGGGAATCA**GACAGC**GCCTTTCGTT**CCCTTT**CTTCGACTTTTGGCATCAA
AAGGGAATCA**GACAGC**GTCTTTCGTT**CCCTTT**CTTCGACTTTTGGCATCAA
AAGGGAATCA**GACAGC**GTCTTTCGTT**CCCTTT**CTTCGACTTTTGGCACCAA
AAGGGAATCAAACAGCGTCTTTCGTT**CCCTTT**CTTTGACTTTTGGCATCA**AAAGGG**AATCA
> AGGGAATCAA Con:1 reps=5 [991762,991980] l-int=219 l-seq = 52
AGGGAATCAAAGAGTGTGTCTGATT**CCCTTT**TTGAAGCCAAAAGCCTTGGAA
AGGGAATCAAAGAGTGTTTCTGATTCCTTTTCTGATGCCAGAAGCCTGAGAA
AGGGAATCAAAGAGTGTGTCTGATTCCTTTTCTGATGCCAGAAGCCTGAGAA
AGGGAATCAAAGAGTGTGTCTGATTCCTTTTCTGATGCCAAAAGCCTTGG**AAAGGG**AATCAA
> AAGGTTCGGA Con:1 reps=17 [995262,996156] l-int=895 l-seq = 52
AAGGTTCG**GACAGC**TTTTGTTCGAAGGCTCATGAAGTTGTCCGAAGTCGCTT
AAGGTTCGGACAAATTTTGCTTGATGGCCCTCGAAGTTGTCCGAAGAGGGCA
AAGGTTCG**GACAGC**TTTAGCTCGAAGGCCCTCGAAGTTGTCCGAAGTCGCTC
AAGGTTCA**GACAGC**TTAAGCTTGAAGGCCCTCGAAGTTGTCCGAAGATGCCC
AAGGTTCG**GACAGC**TTTTGCTCAATGGCCCTTGAAGTTATCCGAAGAGGGCA
AAGGTTCG**GACAGC**TTTAGCTTGATGGCCCTCGAAGTTGTCCGAAGTCGCTC
AAGGTTCG**GACAGC**TTTTGCTTGAAGGCCCTCGAAGTTGTCCGAAGAGGCCC
AAGGTTCG**GACAGC**TTTTGCTCGAAGGCTCATGAAGTTGTCCGAAGTCGTCC
AAGGTTCG**GACAGC**TTTTGTTCGAAGGCTCATGAAGTTGTCCGAAGTTGCTC
AAGGTTCG**GACAGC**TTTAGCTTGATGGCCCTCGAAGTTGTCCGAAGAGGGCA
AAGGTTCG**GACAGC**TTTAGCTCGAAGGCCCTCGAAGTTGTCCGAAGTCGCTC
AAGGTTCG**GACAGC**TTAAGCTTGAAGGCCCTCGAAGTTGTCCGAAGATGCCC
AAGGTTCG**GACAGC**TTTTGCTCAATGGCCCTTGAAGTTATCCGAAGTCGTCC
AAGGTTCG**GACAGC**TTTAGCTTGATGGCCCTCGAAGTTGTCCGAAGAGGCCC
AAGGTTCG**GACAGC**TTTTGCTCGAAGGCTCATGAAGTTGTCCGAAGTCGCCC
AAGGTTCG**GACAGC**TTTTGCTCGGAGGCTCATGAAGTTGTCCGAAGTCGTCC
AAGGTTCG**GACAGC**TTTTGCTCGAAGGCTCATGAAGTTGTCCGAAGTGGTTCAAGGTTCGGA
> TGTCCGAAGT Con:1 reps=13 [1098526,1099160] l-int=635 l-seq = 52
TGTCCGAAGTTGGCTGGAGTTCA**GACAGC**TTTAAGGGTTTGGCAGCAGAAGC
TGTCCGAAGTTGATTAGAGTTCA**GACAGC**TTTAAGTGTTTGGAAGCAGAAGC
TGTCCGAAGTTGGTTAGAGTTCAGACGGCTTTAGGGGTTTGGAAGCAAAAGT
TGTCCGAAGTTGCTCTGAGTTCAGACAGGTTTAGGGGTTTGGAAGCAAAAGC
TGTCCGAAGTTGCTCTGAGTTCA**GACAGC**TTTAGGGGTTTGGAAGCAAAAGC
TGTCCGAAGTTGCTCTGAGTTCAGACAGGTTTAGGGGTTTGGGAGCAAAAGC
TGTCCGAAGTTGCTCTGAGTTCAGACAGGTTTAGGGGTTTGGAAGCAAAAGC
TGTCCGAAGTTGCTCTGAGTTCAGACAGGTTTAGGGGTTTGGAAGCAAAAGC
TGTCCGAAGTTGCTCTGAGTTCAGACAGGTTTAGGGGTTTGGAAGCAAAAGC
TGTCCGAAGTTGCTCTGAGTTCA**GACAGC**TTTAAGTGTTTGGAAGTAAAAGC
TGTCCGAAGTTGCTCTGAGTTCAGACAGGTTTAGGGATTTGGAAGTAAAACC
TGTCCGAAGTTGGCTAGAGTTCAGACAGTTTTAAGTGTTTGGTCCCAAAA**GCTGTC**CGAAGT
> TGTCTGAAGT Con:1 reps=13 [1309331,1310018] l-int=688 l-seq = 52
TGTCTGAAGTTGAGAGGCCTTCGGACAGGTTAGAAGCCCACAAATGAAAAGC
TGTCTGAAGTTGGGTAGCCTTTGGACAGGTTAGAAGCCTACAGAGTAAAAGC
TGTCTGAAGTTGGGTAGCCTTCGGACAGGTTAGAAGCCTACAGAGTAAAAGT
TGTCTGAAGTTGAGCAGCCTTTGGACAGGTTAGAAGCCTACAGAGTAAAAGT
TGTCTGAAGTTGAGCAGCCTTTGGACAGGTTAGAAGCCTACAGAGTAAAAGT
TGTCTGAAGTTGAGCAGCCTTCGGACAGGTTAGAAGCCTACAGAGTAAAAGC
TGTCTGAAGTTGGGTAGCCTTCGGACAGGTTAGAAGCCTACAGAGTAAAAGC
TGTCTGAAGTTGAGAGGCCTTCGGACAGGT**TCATAG**CCTACAGAGTAAAAGT

TGTCTGAAGTTGGGTAGCCTTCGGACAGGTTAGAAGCCTACAGAGTAAAAGC
TGTCTGAAGTTGAGAGGCCTTCGGACAGGTTAGAAGCCTACAGAGTAAAAGC
TGTCTGAAGTTGAGAGGCCTTCGGACAGGTTAGAAGCCTACAGAGTAAAAGT
TGTCTGAAGTTGAGAGGCCTTCGGACAGGT**TCATAG**CCCTACAGAGTAAGAGT
TGTCCGAAGTTGAGCAGCCTTCGGACAGGTTAGAAGCCTACAGAGTAAAAGTTGTCTGAAGT
> TGTTTGATTC Con:1 reps=5 [1336769,1336987] l-int=219 l-seq = 52
TGTTTGATTCCCTTACTAATGCCCAAAGCCCAAC**AAAGGG**AATGAAAGGCAC
TGTTTGATT**CCCTTT**CTCATGCCCAAAGCCCAAC**AAAGGG**AATGAAAGGCAC
TGTTTGATT**CCCTTT**CTCATGCCCAAAGCCCAAC**AAAGGG**AATGAAAGGCAT
TGTTTGATT**CCCTTT**CTCATGCCCAAAGCCCAAC**AAAGGG**AATGAAAGGCATTGTTTGATTC
> C**AAAGGG**AAT Con:1 reps=5 [1400861,1401079] l-int=219 l-seq = 52
C**AAAGGG**AATAAAAAGCGTTGTTTCATT**CCCTTT**CTCATGCACAAAGCTCAA
C**AAAGGG**AATGAAAAGCATTGTTTCATTCCTTTTCTCATGCCAAAAGCTATC
C**AAAGGG**AATGAAAACCATTGTTTCATTCCTTTTCTCATGCCAAAAGCTATC
C**AAAGGG**AATGAAAAGCATTGTTTCATTCCTTTTCTCATGCCAAAAGCTATCC**AAAGGG**AAT
> TTTGGCATGA Con:1 reps=10 [1412262,1412738] l-int=477 l-seq = 52
TTTGGCATGAG**AAAGGG**AATGAAACAATGCTTTTCATT**CCCTTT**GGTAGGCT
TTTGGCATGAG**AAAGGG**AATCAGACAACTCCTTTCATT**CCCTTT**GTTGGGCT
TTTGGCATGAG**AAAGGG**AATCAGACAACTCCTTTCATT**CCCTTT**GTTGGGCT
TTTGGCATGAG**AAAGGG**AATCAGACAACTCCTTTCATT**CCCTT****TCATAG**GCT
TTTGGCATGAG**AAAGGG**AATCAAACAAGCTTTTCATT**CCCTTT**GGTAGGCT
TTTGGCATGAG**AAAGGG**AATCAAACAACTCCTTTCATTCCCTG**TCATAG**GCT
TTTGGCATGAG**AAAGGG**AATCAAACAATGCTTTTCATT**CCCTTT**GTTGGGCT
TTTGGCATGAG**AAAGGG**AATCAGACAACTCCTTTCATT**CCCTTT**GTTGGGCT
TTTGGCATGAG**AAAGGG**AATCAAACAATGCTTTTCATT**CCCTTT**GTTGGGCTTTGGCATGA
> AA**GCTGTC**CG Con:1 reps=20 [1511589,1512587] l-int=999 l-seq = 52
AA**GCTGTC**CGAAGAGAGCTCAGGTTCAGACAGGTTTGCTCTGGTTAAGTAGA
AA**GCTGTC**CGAAGAGAGTTCAGGTTCAGACAGGTTTGCTCTGGTTAAGTAGA
AA**GCTGTC**CGAAGAGAGTTCAGGTTCAGACAGGTTTGCTTGGTTTTAGTGGA
AA**GCTGTC**CGAAGAGAGCTCAGGTTCAGACAGGTTTGCTCAGGTTGAGTGGA
AA**GCTGTC**CGAAGAGAGTTCAGGTTCAGACAGGTTTGCTCTGGTTAAGTAGA
AA**GCTGTC**CGAAGAGAGCTCAGGTTCAGACAGGTTTGCTCAGGTTGAGTAGA
AA**GCTGTC**CGAAGAGAGTTCAGGTTCAGACAGGTTTGCTCAGGTTGAGTGGA
AA**GCTGTC**CGAAGAGAGCTCAGGTTCAGACAGGTTTGCTCAGGTTGAGTAGA
AA**GCTGTC**CGAAGAGAGCGCAGGTTCAGACAGGTTTGCTCTGGTTAAGTAGA
AA**GCTGTC**CGAAGAGAGCGCAGGTTCAGACAGGTTTGCTCTGGTTAAGTAGA
AA**GCTGTC**CGAAGAGAGTTCAGGTTCAGACAGGTTTGCTCAGGTTAAGTAGA
AA**GCTGTC**CGAAGAGAGTTCAGGTTCAGACAGGTTTGCTCAGGTTGAGTAGA
AA**GCTGTC**CGAAGAGAGCGCAGGTTCAGACAGGTTTGCTCTGGTTAAGTAGA
AA**GCTGTC**CGAAGAGAGCTCAGGTTCAGACAGGTTTGCTCAGGTTGAGTAGA
AA**GCTGTC**CGAAGAGAGCGCAGGTTCAGACAGGTTTGCTCTGGTTAAGTAGA
AA**GCTGTC**CGAAGAGAGCTCAGGTTCAGACAGGTTTGCTCTGGTTAAGTAGA
AA**GCTGTC**CGAAGAGAGTTCAGGTTCAGACAGGTTTGCTCAGGTTGAGCGGG
AA**GCTGTC**CGAAGAGAGTTCAGGTTCAGACAGGTTTGCTCAGGTTGAGTGGA
AA**GCTGTC**CGAAGAGAGCTCAGGTTCAGACAGGTTAGCTCAGGTTGAGTGGAAA**GCTGTC**CG
> TTTCGTTCCC Con:1 reps=26 [1602707,1604017] l-int=1311 l-seq = 52
TTTCGTT**CCCTTT**CATGGGCTATTGGTGTGGG**AAAGGG**AATCAGAGAACAGC
TTTCGTT**CCCTTT**CATGGGCTATTGGTGTGGG**AAAGGG**AATCAGAGAACAGC
TTTCGTT**CCCTTT**CATGGGCTTTGGGTATGAG**AAAGGG**AATCAGAGAACAGC
TTTCGTT**CCCTTT**CATGGGCTATTGGTGTGGG**AAAGGG**AATCAGAGAACAGC
TTTCGTT**CCCTTT**CATGGGCTATTTGTGTGGG**AAAGGG**AATCAGAGAACAGC
TTTCGTT**CCCTTT**CATGGGCAATTGGTATGGG**AAAGGG**AATCAGCCAGCATC
TTTCGTT**CCCTTT**CATGGGCTTTGGGTGTGGG**AAAGGG**AATCAGAGAACAGC
TTTCGTT**CCCTTT**CATGGGCTATTGGTGTGGG**AAAGGG**AATCAGAGAACATC
TTTCGTT**CCCTTT**CATGGGCTTTGTGTGTGGG**AAAGGG**AATCAGAGAACATC
TTTCGTT**CCCTTT**CATGGGCTATTGGTGTGGG**AAAGGG**AATCAAACACGACC
TTTCGTT**CCCTTT**CATGGGCTTTGTGTGTGGG**AAAGGG**AATCAGAGAACATC
TTTCGTT**CCCTTT**CATGGGCAATTGGTATGGG**AAAGGG**AATCAGCCAGCATC
TTTCGTT**CCCTTT**CATGGGCTTTGGGTGTGGG**AAAGGG**AATCAGAGAACATT
TTTCGTT**CCCTTT**CATGGGCTATTGGTGTGGG**AAAGGG**AATCAGAGAACATC
TTTCGTT**CCCTTT**CATGGGCTTTGTGTGTGGG**AAAGGG**AATCAGAGAACATC
TTTCGTT**CCCTTT**CATGGGCGATTGGTGTGGG**AAAGGG**AATCAAACACGAGC
TTTCGTT**CCCTTT**CATGGGCTTTGGGTATGGG**AAAGGG**AATCAGAGAACATC
TTTCGTT**CCCTTT**CCGGGGCGATTGGTGTGGG**AAAGGG**AATGAAACACGACC
TTTCGTT**CCCTTT**CATGGGCGTTGGGTATGGG**AAAGGG**AATCAGAGAACATC

TTTCGTT**CCCTTT**CCGGGGCGATTGGTGTGGG**AAAGGG**AATGAAACACGACC
TTTCGTT**CCCTTT**CATGGGCGTTGGGTATGGG**AAAGGG**AATCAGAGAACATC
TTTCGTT**CCCTTT**CATGGGCTTTGGGGGTGGG**AAAGGG**AATCAGAGAACATC
TTTCGTT**CCCTTT**CATGGGCTTTGTGTGTGGG**AAAGGG**AATCAGAGAACATC
TTTCGTT**CCCTTT**CATGGGCTATTGGTGTGGG**AAAGGG**AATCAAACACGACC
TTTCGTT**CCCTTT**CATGGGCTTTGGGTATGGG**AAAGGG**AATCAGAGAACATCTTTCGTTCCC
> GAG**AAAGGG**A Con:1 reps=9 [1747684,1748108] l-int=425 l-seq = 52
GAG**AAAGGG**AATCAAACAACGTCTTTCGTTCCCTTGCACCGGTGTTTGGCGG
GAG**AAAGGG**AATCAAACAACGTCTTTCGTTCCCTTGCACCGGCTTTTGGCGA
GAG**AAAGGG**AATGAAACAGCGCCTTTCGTTCCCTTGCACCGGCTTTTGGCAA
GAG**AAAGGG**AATGAAACAGCGCCTTTCGTTCCCTTGCACCGGCTTTGGCAA
GAG**AAAGGG**AATGAAACAGCGCCTTTCGTTCCCTTGCACCGGCTTTGGCAA
GAG**AAAGGG**AATGAAACAGCGCCTTTCGTTCCCTTGCACCGGCGTTTGGCGA
GAG**AAAGGG**AATCAGACACCGCCTTTCGTTCCCTTGCACCGGCTTTTGGCGA
GAG**AAAGGG**AATCAAACAGCGTCTTTCGTTCCCTTCCGACGGCTTTTGGCAAGAGAG**AAAGGG**A
> CTT**CTATGA**T Con:1 reps=4 [1932894,1933060] l-int=167 l-seq = 52
CTT**CTATGA**TCCAGTTGTCGAGGCGAAAACAAGAAGGAAGTGAA**TCATAG**TC
CTT**CTATGA**TCCAGTTGCCGAAGTGAAAACAAGAAGGAAGTGAA**TCATAG**GC
CTT**CTATGA**TCCAGTTGCCGAAGTGAAAATAAGGAGGAAATGTA**TCATAG**GGCTT**CTATGA**T
> **GACAGC**TTTG Con:1 reps=20 [1958292,1959394] l-int=1103 l-seq = 52
GACAGCTTTGGTTAGATCTAATAGAAAGTTGTCCGAACCTGGGCAGACTTCG
GACAGGTTTGGTTAGATTTACGAGATA**GCTGTC**CGAACCTGGGTAGACTTCG
GACAGCTTTGGTTAGATCTAATAGAAAGTTGTCCGAACCTAAGTAGACTTCG
GACAGCTTTGGTTAGATCTAATAGAAAGTTGTCCGAACCTAAGTAGACTTCGG
ACAGCTTTAGCTCATACAACAAAAAA**GCTGTC**CGAACACAGGTGTACTTCA
GACAGCTTTGTGTCTAACAACAAGAAA**GCTGTC**CGAACCTAGGTAGACTTCG
GACAGCTTTGGCAATATCAACAAGAAA**GCTGTC**CGAACCCAGGCGCACTTCA
GACAGCTTTGGCTAGATCAACAAGAAA**GCTGTC**CGAACCAAGGTAGACTTCA
GACAGCTTTGGCTCATACAACAAAAAA**GCTGTC**CGAACCTAGGTAGACTTCG
GACAGCTTTGGCTCATACAACAAAAAA**GCTGTC**CGAACACAGGTGTACTTCA
GACAGCTTTGTGTCTAACAACAAGAAA**GCTGTC**CGAACCTAGGTAGACTTCG
GACAGCTTTGGCAATAACAACAAGAAA**GCTGTC**CGAACCTAGGTAGACTTCG
GACAGCTTTGGCAATAACAACAAGAAA**GCTGTC**CGAACCTAGGTAGACTTCG
GACAGCTTTGGTAATAACAACAAGAAA**GCTGTC**CGAACCTAGGTAGACTTCG
GACAGCTTTGGCAATATCAACAAGAAA**GCTGTC**CGAACCTAGGCGCACTTCA
GACAGCTTTGGCTAAATCAACAAGAAA**GCTGTC**CGAACCTAGGTAGACTTCG
GACAGCTTTGGCTAGATCAACAAGAAA**GCTGTC**CGAACCCAGGCGCACTTCA
GACAGCTTTGGCTAAATCAACAAGAAA**GCTGTC**CGAACCCAGGCGCACTTCA
GACAGCTTTGGCTAAATCAACAAGAAA**GCTGTC**CGAACCAAGGTAGGCTTCG
GACAGCTTTGGCTCATACAACAAAAAA**GCTGTC**CGAACACAGGTGTACTTCA
GACAGCTTTGTGTCTAACAACAAGAAA**GCTGTC**CGAACCCAGGCGCACTTCA**GACAGC**TTTG
> AA**GCTGTC**CG Con:1 reps=6 [1960889,1961159] l-int=271 l-seq = 52
AA**GCTGTC**CGAACCTAGGTAGACTTCG**GACAGC**TTTGGCAATATCAACAAGA
AA**GCTGTC**CGAACCTAGGCGCACTTCA**GACAGC**TTTGGCTAAATCAACAAGA
AA**GCTGTC**CGAACCTAGGTAGACTTCG**GACAGC**TTTGGCTAGATCAACAAGA
AA**GCTGTC**CGAACCAAGGTAGACTTCG**GACAGC**TTTGGCTATATCAACAAGA
AA**GCTGTC**CGAACCCAGGCGCACTTTA**GACAGC**TTTGGCTAAATCAACTAAGAA**GCTGTC**CG
> AA**GCTGTC**CG Con:1 reps=19 [1988468,1989414] l-int=947 l-seq = 52
AA**GCTGTC**CGAACCCAAGGTGACTTCA**GACAGC**TTTAGCGAGAAATGAAGTC
AA**GCTGTC**CGAACCCAAGGTGACTTCA**GACAGC**TTTAGCGAGAAAGCGATAA
AA**GCTGTC**CGAACCCAAGGCAACTTCAGACAGGTTTAGCAAGAAATGAAGTC
AA**GCTGTC**CGAACCCAGGGCAACTTCGGACAACTTTAGCAAGAAATGAAGTC
AA**GCTGTC**CGAACCCAGGGCAACTTCGGACAACTTTAGCAAGAAATGAAGTC
AA**GCTGTC**CGAACCCAAGGCAACTTCA**GACAGC**TTTAGCGAGAAAGCGATAA
AA**GCTGTC**CGAACCCAAGGCAACTTCA**GACAGC**TTTAGCGAGAAAGCGAGAA
AA**GCTGTC**CGAACCCAAGGCAACTTCAGACAGGGTTAGCGAGAAATGAAGTC
AA**GCTGTC**CGAACCCAGGGCAACTTCGGACAACTTTAGCAAGAAATGAAGAC
AA**GCTGTC**CGAACCCAGGGCAACTTCGGACAACTTTAGCAAGAAATGAAGAC
AA**GCTGTC**CGAACCCAGGGCAACTTCGGACAACTTTAGCAAGAAATGAAGAC
AA**GCTGTC**CGAACCCAGGGCAACTTCGGACAACTTTAGCAAGAAATGAAGAC
AA**GCTGTC**CGAACCCAGGGCAACTTCAGACAGATTTAGCGAGAAAGCGATAA
AA**GCTGTC**CGAACCCAAGGCAACTTCAGACAGGTTTAGCAAGAAATGAAGTC
AA**GCTGTC**CGAACCCAAGGTGACTTCA**GACAGC**TTTAGCGAGAAATGAAGTC
AA**GCTGTC**CGAACCCAAGGCAACTTCAGACAGGTTTAGCAAGAAATGAAGTC
AA**GCTGTC**CGAACCCAAGGTGACTTCA**GACAGC**CTTAGCGAGAAATGAAGTC

AA**GCTGTC**CGAACCCAGGGCAACTTCAGACAGATTTAGCGAGAAAGCGATAAAA**GCTGTC**CG
> GTTCAGACAG Con:1 reps=21 [2030290,2031340] l-int=1051 l-seq = 52
GTTCA**GACAGC**TTTTAGCGTTTGAGTTGAGAA**GCTGTC**CGAAGTAGAGCAAG
GTTCAGACAGTTTTTAGTGTTTGAGTTGAGAA**GCTGTC**CGAAGTAGAGCAAG
GTTCA**GACAGC**TTTTAGCGTCTGAGTTGAAAA**GCTGTC**CGAAGTAGAGCAAG
GTTCAGACAGTTTTTAGTGTTTGGCTCCGAAA**GCTGTC**CGAAGTAGAGCAAG
GTTCA**GACAGC**TTTTAGCGTTTGAGTTGAAAA**GCTGTC**CGAAGTAGAGCAAG
GTTCAGACAGTTTTTAGTGTTTGGCTCCGAAA**GCTGTC**CGAAGTAGAGCAAG
GTTCA**GACAGC**TTTTAGCGTTTGAGTTGAAAA**GCTGTC**CGAAGTAGTGTCAG
GTTCA**GACAGC**TTTTAGCGTCTGGCTCGAAAA**GCTGTC**CGAAGTCGTGCGAG
GTTCA**GACAGC**TTTTAGCGTCTGAGTTGAGAA**GCTGTC**CGAAGTCGTGCGAG
GTTCA**GACAGC**TTTTAGCGTCTGGCTCCGAAA**GCTGTC**CGAAGTGGATCAAG
GTTCA**GACAGC**TTTTAGCGTCTGGCTCCGAAA**GCTGTC**CGAAGTGGATCAAG
GTTCA**GACAGC**TTTTAGTGTTTGGCTCCGAAA**GCTGTC**CGAAGTCGTGTGAG
GTTCA**GACAGC**TTTTAGCGTCTGGCTCGAAAA**GCTGTC**CGAAGTCGTGTGAG
GTTCA**GACAGC**TTTTAGCGTCTGAGTTGAGAA**GCTGTC**CGAAGTGGATCAAG
GTTCA**GACAGC**TTTTAGCGTTTGAGTTGAGAA**GCTGTC**CGAAGTAGAGCAAG
GTTCA**GACAGC**TTTTAGTGTTTGGCTCCGAAA**GCTGTC**CGAAGTCGTGTGAG
GTTCA**GACAGC**TTTTAGCGTCTGAGTTGAGAA**GCTGTC**CGAAGTCGTGCGAG
GTTCA**GACAGC**TTTTAGCGTCTGGCTCCGAAA**GCTGTC**CGAAGTGGATCAAG
GTTCA**GACAGC**TTTTAGCGTCTGGCTCCGAAA**GCTGTC**CGAAGTGGATCAAG
GTTCA**GACAGC**TTTTAGTGTTTAGGTCGAAAA**GCTGTC**CGAAGTAGAGCAAAGTTCAGACAG
> TTCA**GACAGC** Con:1 reps=10 [2086406,2086936] l-int=531 l-seq = 52
TTCA**GACAGC**TTTATCTCAAAACCTGTCAAAGTTGTCCGAAGTGGAGGTAAC
TTCA**GACAGC**TTTGGCCGAGCCCGAAGCAAAGTTGTCCAAAGACGCTGGAAG
TTCA**GACAGC**TTTGGCTCAAAACCTGTCAAA**GCTGTC**CGAAGTGGTTGGAAG
TTCA**GACAGC**TTTGGCTCAGAAGGAAGCAAA**GCTGTC**CGAAGAGGTTGGAAG
TTCA**GACAGC**TTTGGCCCAGAACGAATCAAA**GCTGTC**CGAAGTGGAGGTAAC
TTCAGGCAGCTTTGGCCCAGAACAAAACAAAGTTGTCCGAAGTGAAGGTAAC
TTCA**GACAGC**TTTGGCCCAGAACGAATCAAA**GCTGTC**CGAAGGGGTTGGAAG
TTCA**GACAGC**TTTGGCTCAGAAGAAAGCAAA**GCTGTC**CGAAGAGGTTGGAAG
TTCA**GACAGC**TTTGGCCCAGAACGAATCAAA**GCTGTC**CGAAGTGGAGGTAAC
TTCA**GACAGC**TTTGGCCCAGAACAAAACAAAGTTGTCCGAAGTGAAGGTAACTTCA**GACAGC**
> GAAA**GCTGTC** Con:1 reps=10 [2116675,2117153] l-int=479 l-seq = 52
GAAA**GCTGTC**CGAAGTAGGTCTGGGTTCA**GACAGC**TTTAACGTCCAAGCACC
GAAA**GCTGTC**CGAATGTGGACCGGGTTCA**GACAGC**TTTTCCGTCTGAGCACC
GAAA**GCTGTC**CGAAGTTGGTCTAGGTTCA**GACAGC**TTTTCCGTCCGAGCACC
GAAA**GCTGTC**CGAAGTTGGTCTAGGTTCA**GACAGC**TTTTCCGTCCGAGCGCG
GAAA**GCTGTC**CGAAGTAGGTCTGGGTTCAGACAGTTTTTCCGCCCGAGCACG
GAAA**GCTGTC**CGAAGTTGCCCCGGGTTCA**GACAGC**TTCAACGCCCGAGCGCG
GAAA**GCTGTC**CGAAGTTGCCCCGGGTTCA**GACAGC**TTCAACGCCCGAGCACG
GAAA**GCTGTC**CGAAGTTGCCCCGGGTTCA**GACAGC**TTCAACGCCCGAGCACG
GAAA**GCTGTC**CGAAGTTGCCCCGGGTTCAGACAACTTTTCAGTCCGAGCGCGGAAA**GCTGTC**
> TCTGATTCCC Con:1 reps=23 [2149247,2150397] l-int=1151 l-seq = 52
TCTGATT**CCCTTT**CAGTAGTGTATTGGGGTAGAAGGGAATGAAAGAGCGAG
TCTGATT**CCCTTT**CAGTAGTGTATTGGGGTAGAAGGGAATGAAAGAGCGAT
TCTGATT**CCCTTT**CAATAGTGAATCGAGATAA**AAAGGG**AACGAAAGAGCAAG
TCTGATT**CCCTTT**CAATAGTGAATCGGGGTAG**AAAGGG**AACGAAAGAGCAAG
TCTGATT**CCCTTT**CAATAGTGTATCGGGTAG**AAAGGG**AACGAAAGAGCGAG
TCTGATT**CCCTTT**CAATAGTGTATCGGGTAG**AAAGGG**AACGAAAGAGCGAG
TCTGATT**CCCTTT**CAATAGTGAATCGGGGTAG**AAAGGG**AACGAAAGAGCAAG
TCTGATT**CCCTTT**CAATAGTGTATCGGGGTAG**AAAGGG**AACGAAAGAGCAAG
TCTGATT**CCCTTT**CAGTAGTGCATCGGAGGAG**AAAGGG**AACGAAAGAGCAAG
TCTGATT**CCCTTT**CAGTAGTGAATCGGAGGAG**AAAGGG**AACGAAAGAGCAAG
TCTGATT**CCCTTT**CAATAGTGAATCGGGGTAG**AAAGGG**AACGAAAGAGCAAG
TCTGATT**CCCTTT**CAGTAGTGCATCGGAGGAG**AAAGGG**AACGAAAGAGCAAG
TCTGATT**CCCTTT**CAGTAGTGCATCGGGGTAG**AAAGGG**AATGAAAGAGCGAG
TCTGATT**CCCTTT**CAGTAGTGAATCGGGGTAG**AAAGGG**AACGAAAGAGCAAG
TCTGATT**CCCTTT**CAGTAGTGCATCGGGGTAG**AAAGGG**AATGAAAGAGCGAG
TCTGATT**CCCTTT**CAGTAGTGTATCGGGGTAG**AAAGGG**AATGAAAGAGCAAG
TCTGATT**CCCTTT**CAGTAGTGCATCGGAGGAG**AAAGGG**AACGAAAGAGCAAG
TCTGATT**CCCTTT**CAGTAGTGAATCGAGGTAG**AAAGGG**AATGAAAGAGCAAG
TCTGATT**CCCTTT**CAGTAGTGCATCGGGGTAG**AAAGGG**AATGAAAGAGCGAG
TCTGATT**CCCTTT**CAGTAGTGTATCGGGGTAG**AAAGGG**AATGAAAGAGCAAG
TCTGATT**CCCTTT**CGGTAGTGCATCGGGGGAG**AAAGGG**AATGAAAGAGCGAG

TCTGATT**CCCTTT**CAGTAGTTAATTGGGGTAGAAAAGGAATGAAAGAACGAGTCTGATTCCC
> GCTACTTCAG Con:1 reps=7 [2223040,2223414] l-int=375 l-seq = 52
GCTACTTCA**GACAGC**TTTTGCTTTAAAACAATTAAAGTTGTCTGAACCCGAG
GCTATTTCA**GACAGC**TTTTGCTTTAAAACAATTAAA**GCTGTC**CGAACCCGAA
GCTACTTCA**GACAGC**TTTTGCTTTAAAACAATCAAA**GCTGTC**CGAACCCGAA
GCTACTTCA**GACAGC**TTTTGATTAAAAACAATCAAA**GCTGTC**CGAACCTGAG
GCTACTTCAGACATCTTTTGCTTTAAAACAATCAAA**GCTGTC**CGAACCCGAA
GCTACTTCA**GACAGC**TTTTGATTAAAAACAATCAAA**GCTGTC**CGAACCCGAG
GCTACTTCA**GACAGC**TTTTGCTTTAAAACAATCAAA**GCTGTC**CGAACCCGAGGCTACTTCAG
> GCATGAGAAA Con:1 reps=5 [2268253,2268471] l-int=219 l-seq = 52
GCATGAG**AAAGGG**AATCAAACAACGCTTTTCATT**CCCTTT**GGATAGCTTTTG
GCATGAGAAAAGGAATGAAACAATGCTTTTCATT**CCCTTT**GGATAGCTTTTG
GCATGAGAAAAGGAATGAAACAACGCTTTTTATT**CCCTTT**GTAGGGCTTTGG
GCATGAG**AAAGGG**AATGAAACAACACTTTTCATT**CCCTTT**GTTGAGCTTTGTGCATGAGAAA
> AGATTCCCTT Con:1 reps=5 [2283102,2283320] l-int=219 l-seq = 52
AGATTCCCTTACTAGGCAAAAATGAGGTGC**AAAGGG**AACGAAAGGTAGTCAC
AGATTCCCTTACTAGGCAAAAATGAGGTGC**AAAGGG**AACGAAAGGTAGTCAC
AGATTCCCTTACTAGGTAAAAATGAAGCGAGAAGGGAACGAAAGGTAGTCAC
AGATTCCCTTACTAGGCAAAAATGAAGTGC**AAAGGG**AACGAAAGGTAGTGTTAGATTCCCTT
> TCAGACAACC Con:1 reps=7 [2379685,2380007] l-int=323 l-seq = 52
TCAGACAACCACTTTCGTT**CCCTTT**CTCGCGCTCTTGGCATTAG**AAAGGG**AA
TCAGACAACCACTTCCGTT**CCCTTT**CTCGCGCTCTTGGCACTAG**AAAGGG**AT
TCAGACAACCACTTCCGTT**CCCTTT**CTCGCGCTCTTGGTACCAG**AAAGGG**AA
TCAGACAACCACTTCCGTT**CCCTTT**CTCGCGCTCTTGGTACCAG**AAAGGG**AA
TCAGACAACCACTTCCGTT**CCCTTT**CTCGCGCTCTTGGTACCAG**AAAGGG**AA
TCAGACAACCACTTCCGTT**CCCTTT**CTCGCGCTCTTGGCACTAG**AAAGGG**ATTCAGACAACC
> CATAGGCCTT Con:1 reps=5 [2495463,2495733] l-int=271 l-seq = 52
CATAGGCCTT**CTATGA**TTTAGTTGCTTGAGCGAAAACAAGGAGAAAGTGAAT
CATAATCCTC**CTATGA**TCCAGTTGTCTGAGCGAAAACAGGGAGAAAGTAAAT
CATAGGCCTT**CTATGA**TTTAGTTGCTTGAGCGAAAACAAGGAGAAAGTGAAT
CATAGGCCTT**CTATGA**TCCAGTTGTCTGAGCGAAAAGAAGGAGAAAGTAAAT
CATAGGCCTT**CTATGA**TTTAGTTGTCTGAGCGAGAACAGGTAGGAAGTAAACCATAGGCCTT
> GGGTTGTCAG Con:1 reps=8 [2516063,2516488] l-int=426 l-seq = 52
GGGTTGTCAGATTTTCCTATCTGTATGCCAAAAGCCCAAG**AAAGGG**AACGAAAG
GGGTTGTCAGATT**CCCTTT**CTGGCACCAAAAGCCCGAG**AAAGGG**AACGAAAG
GGGTTGTCAGATT**CCCTTT**CTGGCACCAAAAGCCCGAG**AAAGGG**AACAAAAG
GGGTTGTCAGATT**CCCTTT**CTGGCACCAAAAGCCCGAG**AAAGGG**AACGAAAG
GG**GCTGTC**AGATT**CCCTTT**CTGGCACCAAAAGCCCGAG**AAAGGG**AACAAAA
GGGTTGTCAGATTCCTTTTCTGGCACCAAAAGCCCGAG**AAAGGG**AACGAAAG
GGGTTGTCAGATT**CCCTTT**CTGGCACCAAAGCCCGAG**AAAGGG**AACGAAAG
GGGTTGTCAGATTCCTTTTCTGGCACCAAAAGCCCGAA**AAAGGG**AACG**AAAGGG**TTGTCAG
> AAGAGGAAAA Con:1 reps=6 [2536105,2536375] l-int=271 l-seq = 52
AAGAGGAAAA**GCTGTC**GGAAGTGGAGTGAGGTTCAGACAGGTTGGGGGTGAG
AAGAGGAAAA**GCTGTC**CGAAGTGGAGTAAGGTTCAGACAGGTTGAGGGTGAG
AAGAGGAAAA**GCTGTC**CGAAGTGGAGTGAGGTTCAGACAGGTTGGGGGTGAG
AAGAGGAAAA**GCTGTC**CGAAGTGGAGTGAGGTTCAGACAGGTTGAGGGTGAG
AAGAGGAAAA**GCTGTC**CGAAGTGGAGTGAGGTTCAGACAGGTTGAGGGTGTAAAGAGGAAAA
> CCAAGAAAGG Con:1 reps=7 [2696032,2696348] l-int=317 l-seq = 52
CCAAG**AAAGGG**AATCAAAGGTGTTGTCTGATTCCCTTACTGTTGCCAAAAG
CCAAG**AAAGGG**AATCAAAGCTGTTGTCTGATTCCCTTACTGTTGCCAAAAGC
CCAAG**AAAGGG**AATCAAAGGCGTTGTCTGATTCCCTTATTGTTGTCAAAAGC
CCAAG**AAAGGG**AATCAAAGGCGTTGTCTGATTCCCTTATTGTTGTCAAAAGC
CCAAG**AAAGGG**AATCAAAGGCGTGATTCCCTTACTGTTGTCAAAAGC
CCAAG**AAAGGG**AATCAAAGGCGTTGTCTGATTCCCTTACTGTTGTCAAAAGCCCAAGAAAGG
> AAGGAGGAAG Con:1 reps=7 [2741337,2741711] l-int=375 l-seq = 52
AAGGAGGAAGTGAATCATACGCCTT**CTATGA**TTCAGTTGCCGAGGCGAAACC
AAGGAGGAAGTGAATCATACACACT**CTATGA**TCCAGTTGCCAAGGCGAAGCC
AAGGAGGAAGTGAATCATACACACT**CTATGA**TCCAGTTGCCAAGGCGAAAAT
AAGGAGGAAGTGAATCATACGCCCT**CTATGA**TCCAGTTGCCGAGGCGAAACC
AAGGAGGAAGTGAATCATACGCCCTCTACGATCCAGTTGGCAAGGCGAAAAT
AAGGAGGAAGTGAA**TCATAG**CCCCT**CTATGA**TACAGTTGCCGAGGCGAAGCC
AAGGAGGAAATAAA**TCATAG**CCCCT**CTATGA**TCCAGTTGCCGAGGCGAAACCAAGGAGGAAG
> C**AAAGGG**AAT Con:1 reps=12 [2767735,2768316] l-int=582 l-seq = 52
C**AAAGGG**AATGAAAAGCGTTGTTTGATT**CCCTTT**CTCATGCCAAAAGCTCAA
C**AAAGGG**AATGAAAAGCGTTGTTTGATT**CCCTTT**CTCATCCCAAAAGCTCAA

CAAAGGGAATGAAAAGCGTTGTTTGATTCCCTTTCTCAGGCCAAAAGCTCAA
CAAAGGGAATGAAAAGCGTTGTTTCATTCCCTTTCTCAGGCCAAAAGCCATC
CAAAGGGAATGAAAAGCATTGTTTGATTCCCTTTCTCAGGCCAAAAGCTCAA
CAAAGGGAATGAAAAGCGTTGTTTCATTCCCTTTCTCAGGCCAAAAGCCATC
CAAAGGGAATGAAAGCATTGTTTGATTCCCTTTCTCAGGCCAAAAGCTCAA
CAAAGGGAATGAAAAGCGTTGTTTGATTCCCTTTCTCATGCCAAAAGCCATC
CAAAGGGAATGAAAAGCATTGTTTGATTCCCTTTCTCAGGCCAAAAGCCATC
CAAAGGGAATGAAAAGCGTTGTTTCATTCCCTTTCTCATGCCAAAAGCCATC
CAAAGGGAATGAAAAGCATTGTTTGATTCCCTTTCTCATGCCAAAAGCCATCCAAAGGGAAT
> AAAGGGAATG Con:1 reps=7 [2792762,2793084] l-int=323 l-seq = 52
AAAGGGAATGAAAAGAGTTGTTTGATTCCCTTTCTCATGCCCAGAGCCCAAC
AAAGGGAATGAAAAGCGTTGTTTCATCCCCTTTCTCATGACCAAAGCCCAAC
AAAGGGAATGAAAAGAGTTGTTTGATTCCCTTTCTCATGCCCGAAGCCCAAC
AAAGGGAATGAAAAGAGTTGTTTGATTCCCTTTCTCATGCCCAAAGCCCAAC
AAAGGGAATGAAAAGCATTGTTTGATTCCCTTTCTCATGCCCAAAGCCCAAC
AAAGGGAATGAAAAGCATTGTTTGATTCCCTTTCTCATGCCCAGAGCCTATGAAAGGGAATG
> AATCATAGGC Con:1 reps=4 [2999947,3000113] l-int=167 l-seq = 52
AATCATAGGCTCTCTATGATTTAGTTGACAAGGCGAAAGCAAGGAGGAAGTG
AATCATAGGCTCTCTATGATTTAGTTGCCAAGGCAAAAACAAGGAAGAAGTG
AATCATAGGCCTTCTATGATTTAGTTGCCAAAGCGAAAGCAAGGAGGAAGTAAATCATAGGC
> TTTCATTCCC Con:1 reps=7 [3095308,3095632] l-int=325 l-seq = 52
TTTCATTCCCTTTCATAGGCTTTGTGTATGAGAAAGGGAATCAAACAACGCT
TTTCATTCCCTTTGGATGGCTTTTGGCATAAGAAAGGGAATCAAACAACGCT
TTTCATTCCCTTTGGATGGCTTTTGGCATGAGAAAGGGAATCAAACAATGCT
TTTCATTCCCTTTGGATGGCTTTTGGCATGAGAAAGGGAATCAAACAATGCT
TTTCATTCCCTTTGGATGGCTTTTGGCATGAGAAAGGGAATCAAACAATGCT
TTTCATTCCCTTTGTAGAGCTTTGTGCATGAGAAAGGGAATCAAACAACGCGCTTTTCATTCCC
> TGTCTGATTC Con:1 reps=6 [3491116,3491382] l-int=267 l-seq = 52
TGTCTGATTCCTTTACTAATACCAAAAGCCCAACAAAGGGAATTAAAGAGGT
TGTCTGATTCCCTTTTTGATGCCAGAAGCCCAACAAAGGGAATCAAAGCGGT
TGTCTGATTCCCTTTCTGATGCCAAGTGCCCTAGAAAGGGAACCAAAGGGGC
TGTCTGATTCCCTTTCTGATGCCAAGTGCCCTAGGAAGGGAACAAAAGGC
TGTCTGATTCCCTTTCTGATGCCAGAGCCCACAAAGAGAATCAAAGCGGTTGTCTGATTC
> ATCAAACAAC Con:1 reps=7 [3630624,3630945] l-int=322 l-seq = 52
ATCAAACAACTCTTTTCATTCCCTTTGCTGGTCTTATGGCATGAGAAAGGGA
ATCAAACAACTCCTTTCATTCCCTTTGTTGGGCTTATGGCATGAGAAAGGG
ATCAAACAACTCCTTTCATTCCCTTTGTTGGGCTTATGGCATGAGAAAGGGA
ATCAAACAACTCCTTTCATTCCCTTTGTTGGGCTTATGGCATGAGAAAGGGA
ATCAAACAACTCTTTTCATTCCCTTTGTTGGGCTTATGGCATGGGAAAGGGA
ATCAAACAACTCTTTTCATTCCCTTTGTTGGGCTTATGGCATGGGAAAGGGAATCAAACAAC
> TTTCTCCTTG Con:1 reps=6 [3790499,3790820] l-int=322 l-seq = 52
TTTCTCCTTGTTTTCGCGACGGGAACTAAATTATAGAAGCTTATGATTTAC
TTTCTCCTTGTTTTTGCCTCAGCAACTGAATCATAGAGAGCCTATGATTCAC
TTTCTCCTTGTTTTTGCGACGGGAACTAAATCATAGAGAGCCTATGATTCAC
TTTCTCCTTGTTTTCCCCCCGCCAACTAAATCATAGAGAGCCTTTGATTTAC
TTTCTCCCTGTTTTCGCCTCATCAACTGAATCATAGAGAGCCTTTGATTTAC
TTTCTCCTTGTTTTTTCCTCAGCAACTGAATCATAGAGAGCCTATGATTCACTTTCTCCTTG
> GAAAGCTGTC Con:1 reps=10 [3845277,3845755] l-int=479 l-seq = 52
GAAAGCTGTCCGAACTTCACTTGGGTTCTGACTACTTTTCCTTTACGACCAC
GAAAGCTGTCCGAACTTCACTTGGGTTCGGACTACTTTCCCTTTACGACCAC
GAAAGCTGTCCGAACTTCACTTGGGTTCGGACTACTTTCCCTTTACGACCAC
GAAAGCTGTCCGAACTTCACTTGGGTTCGGACTACTTTTCCTTTTTGACCGC
GAAAGCTGTCCGAACTTCACTTGGGTTCGGACTACTTTTCCTTTACGACCAC
GAAAGCTGTCCGAACTTCACTTGGGTTCGGACTACTTTCCCTTTACGACCAC
GAAAGCTGTCCGAACTTCACTTGGGTTCGGACTACTTTTCCTTTACGACCAT
GAAAGCTGTCCAAACTTCACTTGGGTTCGGACTACTTTTCCTTTACGACCAC
GAAAGCTGTCCGAACTTCACTTGGGTTCGGACTACTTTCCCTTTACGACCACGAAAGCTGTC
> AAAGGGAATG Con:1 reps=4 [3876626,3876792] l-int=167 l-seq = 52
AAAGGGAATGAAAAGAGTTGTTTGATTCCCTTTCTCATGCCCAAAGCCCAAC
AAAGGGAATGAAAAGCGTTGTTTCATCCCCTTTCTCATGACCAAAGCCCAAC
AAAGGGAATGAAAAGAGTTGTTTGATTCCCTTTCTCATGCCCAGAGCCTATGAAAGGGAATG
> TTGGCATGAG Con:1 reps=5 [3924084,3924302] l-int=219 l-seq = 52
TTGGCATGAGAAAGGGTATGAAACAACGCTTTTCATTTCCTTTCTTGAGCTC
TTGGCATGAGAAAGGGAATGAAACAATGCTTTTTATTCCCTTTGTTGAGCTT
TTGGCATGAGAAAGGGAATGAAACAACGCTTTTCATTCCGTTTGTTGAGCTT

TTGGCATGAGAAAGGGAATGAAACAATGCTTTTTATTCCCTTTGTTGAGCTTTTGGCATGAG
> GCCTATGATT Con:1 reps=8 [3938951,3939325] l-int=375 l-seq = 52
GCCTATGATTTAGTTCTTCCTTGTTCTCGCTTCGGCAACTGACTCATAGAAG
GCCTATGATTCACTTCCTCCTTGTTTTCGCTTCAGCAACTGACTCATAGAAG
GCCTATGATTCACTTCCTCCTTGTTTTCGCTTCAGCAACTGAATCATAGAAG
GCCTATGATTCACTTCCTCCTTGTCTTCGCTTCGGCAACTGACTCATAGAAG
GCCTATGATTTAGTTCCTCCTTGTTTTCTCTTCGGCAACTGAATCATAGAAG
GCCTATGATTCACTTCCTCCTTGTCTTCGCTTCGGCAACTGACTCATAGAAG
GCCTATGATTTAGTTCCTCCTTGTTTTCTCTTCAGCAACTGAATCATTAAGAGCCTATGATT
> ACTTCGGACA Con:1 reps=20 [3948839,3949836] l-int=998 l-seq = 52
ACTTCGGACAGCTTTACCCAGTTTTAGTGCAAAGCTGTCTGAACTCTGGCCT
ACTTCGGACAGCTTTACCCAGTTTTAAAGCAAAGCTGTTTGAACTCTGGCAA
ACTTCGGACAGCTTTAGCTTGTTTAGAGCAAAACTGTCTGAACTCATACCT
ACTTCGGACAGCTTTACCTATTTTTAGAGCAAAGCTGTCTGAACTCTGGCAA
ACTTCGGACAGCTTTAGCTTGTTTTAGAGCAAAGCTGTCTGAACTCTGGCCT
ACTTCGGACAGCTTTAGCTTGTTTTAGAGCAAAGCTGTCTGCGCTCTTGCCA
ACTTCGGACAGCTTTAGCGTGTTTTAGAGCAAGGCTGTCGGAACTCTGGCCT
ACTTCGGACAACTTTAGCTTGTTTTAGAGCCAAGCTGTCTGAACTCTAGCCT
ACTTCGGACAGCTTTAGCGTGTTTTAGAGCAAGGCTGTCGGAACTCTGGCCT
ACTTCGGACAACTTTAGCTTGTTTTAGAGCCAAGCTGTCTGAACTCTAGCCT
ACTTCGGACAGCTTTAGCGTGTTTTAGAGCAAGGCTGTCGGAACTCTGGCCT
ACTTCGGACAACTTTAGCTTGTTTTAGAGCCAAGCTGTCTGAACTCTAGCCT
ACTTCGGACAGCTTTAGCGTGTTTTAGAGCAAGGCTGTCGGAACTCTGGCCT
ACTTCGGACAACTTTAGCTTGTTTTAGAGCCAAGCTGTCTGAACTCTAGCCT
ACTTCGGACAGCTTTAGCTTGTTTTAGAGCAAAGCTGTCTGAACTCTGGCCT
ACTTCGGACAGCTTTAGCTTGTTTTAGAGCAAAGCTGTCTGAACTCTGGCCT
ACTTCGGACAGCTTTACCTATTTTTAGAGCAAAGCTGTCTGAACTCTGGCAA
ACTTCGGACAACTTTACCTAGTTTTAAAGCAAAGCTGTCTGAACTCTGGCCT
ACTTCGGACAGCTTTACCTAGTTTTAGAGCAAAGCTGTCTGAACTCACACCTACTTCGGACA
> GGAATGAAAA Con:1 reps=8 [3983763,3984137] l-int=375 l-seq = 52
GGAATGAAAAGAGTTGTTTCATTCCCTTTCTCATGCCCAAAGCCCAACAAAG
GGAATGAAAAGAGTTGTTTCATTCCCTTTCTCATGCCCAAAGCCCAACAAAG
GGAATGAAAAGCGTTGGTTCGTTCCCTTTCTCATGCCAAAAGCCCAACAAAG
GGAATGAAAAGCATTGTTTCGTTCCCTTTCTCATGCCAAAAGCTCAACAAAG
GGAATGAAAAGAGTTGTTTCATTCCCTTTCTCATGCCCAAAGCCCAACAAAA
GGAATGAAAAGCAGTGTTTCATTCCCTTTCTCATGCCGAAAGCCCAACAAAG
GGAATGAAAAGCATTGTTTGATTCCCTTTCTCATGCCCAAAGCCCAACAAAAGGAATGAAAA
> TTTCTCATGC Con:1 reps=6 [4004918,4005188] l-int=271 l-seq = 52
TTTCTCATGCACAAAGCTCAACAAAGGGAATGAAAAGCATTGTTTCATTCCT
TTTCTCATGCCAAAAGCTATCCAAAGGTAATGAAAAGCATTGTTTCATTCCT
TTTCTCATGCCAAAAGCTATCCAAAGGGAATGAAAAGCGTTGTTTGATTCCC
TTTCTCATGCCAAAAGCTATCCAAAGGGAATGAAAAGCATTGTTTCATTCCT
TTTCTCATGCCAAAAGCTATCCAAAGGGAATGAAAAGCGTTGTTTGATTCCCTTTCTCATGC
> TTTCATTCCC Con:1 reps=8 [4016265,4016639] l-int=375 l-seq = 52
TTTCATTCCCTTTCATAGGCTTTGTGCATGAGAAAGGGAATCAAACAACGCT
TTTCATTCCCTTTGGATGGCTTTTGGCATGAGAAAGGGAATCAAACAATGCT
TTTCATTCCCTTTGTTGAGCTTTTGGCATAAGAAAGGGAATCAAACAATGCT
TTTCATTCCCTTTGGATGGCTTTTGCATGAGAAAGGGAATGAAACAATGCT
TTTCATTCCCTTTGGATGGCTTTTGGCATGAGAAAGGGAATCAAACAATGCT
TTTCATTCCCTTTGGATGGCTTTTGCATGAGAAAGGGAATGAAACAATGCT
TTTCATTCCCTTTGGATGGCTTTTGGCATGAGAAAGGGAATCAAACAACGCGCTTTTCATTCCC
> ACTTCGGACA Con:1 reps=25 [4106739,4108001] l-int=1263 l-seq = 52
ACTTCGGACAACTTCGAGAGGCACGAAGCAAAAGCTGTCCGAACCTTGAGTG
ACTTCGGACAACTTCAAGTGTCCGGAAAGCAAAAGCTGTCCGAACCTTGAGTG
ACTTCGGACAACTTCGAGTGCCCGGAAGCAAAAGCTGTCCGAACCTTGAGTG
ACTTCGGACAACTTCGAGTGCCCGGAAGCAAAAGCTGTCCGAACCTTGAGCG
ACTTCGGACAACTTCGAATGCCCGGAAGCTAAAGCTGTCCAAACCTTGAGCA
ACTTCGGACAACTTCGAATACCCGGAAGCTAAAGCTGTCCGAACCTTGAGCA
ACTTCGGACAACTTCGAGTGCCCGGAAGCTAAAGCTGTCCGAACCTTGAGCG
ACTTCGGACAACTTCGAATGCCCGGAAGCAAAAGCTGTCCGAACCTTGAGCA
ACTTCGGACAACTTCAAGTGTCCGGAAAGCAAAAGCTGTCCGAACCTTGAGCG
ACTTCGGACAACTTCGAATGCCCGGAAGCAAAAGCTGTCCGAACCTTGAGCG
ACTTCGGACAACTTCGAATGCCCGGAAGCTAAAGCTGTCCGAACCTTGAGCG
ACTTCGGACAACTTCGAATGCCCGGAAGCAAAAGCTGTCCGAACCTTGAGCG
ACTTCGGACAACTTCGAGTGCCCGGAAGCTAAAGCTGTCCAAACCTTGAGCA

ACTTCGGACAACTTCAAGTGTCCGGAAAGCAAAA**GCTGTC**CGAACCTTGAGCG
ACTTCGGACAACTTCGAGTGCCCGGAAGCAAAA**GCTGTC**CGAACCTTGAGCG
ACTTCGGACAACTTCGAGTGCCCGGAAGCTAAA**GCTGTC**CGAACCTTGAGTG
ACTTCGGACAACTTCGAATACCCGGAAGCTAAA**GCTGTC**CGAACCTTGAGCA
ACTTCGGACAACTTCGAGTGCCCGGAAGCTAAA**GCTGTC**CGAACCTTGAGCG
ACTTCGGACAACTTCGAATGCCCGGAAGCAAAA**GCTGTC**CGAACCTTGAGCA
ACTTCGGACAACTTCAAGTGTCCGGAAAGCAAAA**GCTGTC**CGAACCTTGAGCG
ACTTCGGACAACTTCGAATGCCCGGAAGCAAAA**GCTGTC**CGAACCTTGAGCG
ACTTCGGACAACTTCGAATGCCCGGAAGCTAAA**GCTGTC**CGAACCTTGAGCG
ACTTCGGACAACTTCGAATGCCCGGAAGCTAAA**GCTGTC**CGAACCTTGAGCA
ACTTCGGACAACTTCGAGTGCCCAGTTGCTAAA**GCTGTC**CGAACCTTGAGTGACTTCGGACA
> TTCATTCCCT Con:1 reps=6 [4144195,4144465] l-int=271 l-seq = 52
TTCATT**CCCTTTCATAG**GCTCTGGGCATGAG**AAAGGG**AATCAAACAATGCTT
TTCATT**CCCTTT**GTTGGGCTTTGGGCATGAG**AAAGGG**AATCAAACAACTCTT
TTCATT**CCCTTT**GTTGGGCTTCGGGCATGAG**AAAGGG**AATCAAACAACTCTT
TTCATT**CCCTTT**GTTGGGCTTTGGTCATGAG**AAAGGG**GATGAAACAACGCTT
TTCATT**CCCTTT**GTTGGGCTTTGGGCATGAGAAATGGAATCAAACAACTCTTTTCATTCCCT
> **AAAGGG**AATG Con:1 reps=7 [4262761,4263081] l-int=321 l-seq = 52
AAAGGGAATGAAAAGAGGTGTTTGATT**CCCTTT**CTCTTGCCAAAAGCCCAAC
AAAGGGAATGAAAAGCATTGTTTCATT**CCCTTT**CTTATGCCAAAAGCCCAAC
AAAGGGAATGAAAAGAGTTGTTTCACT**CCCTTT**CACTTCAAATGCCCAAC
AAAGGGAATGAAAAGAGGTGTTTGATT**CCCTTT**CTCATGCCAAAAGCCCAAC
AAAGGGAATGAAAAGCATTGTTTGATT**CCCTTT**CTCATGCCAAAAGCCCAAC
AAAGGGAATGAAAAGCATTGTTTCATT**CCCTTT**CTCTTGCCAAAAGCCCAAC**AAAGGG**AATG
> TTTCATTCCC Con:1 reps=6 [4272434,4272704] l-int=271 l-seq = 52
TTTCATT**CCCTTT**GGATGGCTTTTGGCATGAA**AAAGGG**AATCAAACAACGCT
TTTCATT**CCCTTT**GTTGAGCTTTTGGCATGAG**AAAGGG**AATCAAACAATGCT
TTTCATT**CCCTTT**GTAGGGCTTTTGGCATGAG**AAAGGG**AATCAAACAATGCT
TTTCATT**CCCTTT**GTTGAGCTTTTGGCATGAG**AAAGGG**AATGAAACAACGCT
TTTCATT**CCCTTT**GGATGGCTTTTGGCATGAG**AAAGGG**AATGAAACAACGCTTTTCATTCCC
> AGGAACTCAA Con:1 reps=5 [4292665,4292883] l-int=219 l-seq = 52
AGGAACTCAA**TCATAG**ACCTGCTTTGATCCAGTTACCGAAGTGAAAACAAAG
AGGAACTCAATCACAGACCTGCTTTGATCCAGTTGCCGAAGTGGAAACAAAG
AGGAACTCAA**TCATAG**ACCTGCTTTGATCCAGTTGCAGAAGCAAAAACAAAG
AGGAACTCAA**TCATAG**ACCTGCTTTGATCCAGTTGCAGAAGCAAAAACAAAGAGGAACTCAA
> AAACAACTCT Con:1 reps=10 [4292959,4293487] l-int=529 l-seq = 52
AAACAACTCTTTTCGTTCCTTTTTTTGGGCTTTTGGCATGAATAAGGGAATC
AAACAACTCTTTTCATT**CCCTTT**GTTGGGTTTTGGGCATTAGTAAGGGAATC
AAACAACTCTTTTCATTCCCTATTTTGGGCTTTTGGGATGGTAAAGGAATCA
AACAATGCCTTTCATT**CCCTTT**GTTGGGCTTTTGGCATGAGTAAGGGAATC
AAACAACTCTTCTCATT**CCCTTT**GTTGAGCTTTTGGCATGAG**AAAGGG**AATG
AAACAACTCTTTTCTT**CCCTTT**GTTGGGCTTTTGGCATGAG**AAAGGG**AATG
AAACAACTCTTTTCATT**CCCTTT**GTTGAGCTTTTGGCATGAG**AAAGGG**AATC
AAACAACTCTTTTCATT**CCCTTT**GTTGGGCTTTTGGCATGAG**AAAGGG**AATG
AAACAACTCTTTTCATT**CCCTTT**GTTGAGCTTTTGGCATGAG**AAAGGG**AATC
AAACAACTCTTTTCATT**CCCTTT**GTTGGGCTTTTGGCATGAG**AAAGGG**AATGAAACAACTCT
> CTCTCTTCGG Con:1 reps=21 [4298062,4299591] l-int=1530 l-seq = 52
CTCTCTTCG**GACAGC**TTTGGCTCTTTTGGCTTCAAA**GCTGTC**TGAACCTGGA
CTCTCTTCG**GACAGC**TTTGGCTCTTTTGGCTTCAAA**GCTGTC**TGAACCTGGA
CTCTCTTCG**GACAGC**TTTNNNNNNNNNNNNNNNNNNNNNNNNNNNNNNNNNNNNNNNNNNNNNNNNNNNNNNNNNNNNNNNNNNNNNNNNNNNNNN
NNNNNNNNNNNNNNNNNNNNNNNNNNNNNNNNNNNNNNNNNNNNNNNNNNNNNNNNNNNNNNNNNNNNNNNNNNNNNNNNNNNNNNNNNNNNNNNN
NNNNNNNNNNNNNNNNNNNNNNNNNNNNNNNNNNNNNNNNNNNNNNNNNNNNNNNNNNNNNNNNNNNNNNNNNNNNNNNNNNNNNNNNNNNNNNNN
NNNNNNNNNNNNNNNNNNNNNNNNNNNNNNNNNNNNNNNNNNNNNNNNNNNNNNNNNNNNNNNNNNNNNNNNNNNNNNNNNNNNNNNNNNNNNNNN
NNNNNNNNNNNNNNNNNNNNNNNNNNNNNNNNNNNNNNNNNNNNNNNNNNNNNNNNNNNNNNNNNNNNNNNNNNNNNNNNNNNNNNNNNNNNNNNN
NNNNNNNNNNNNNTCTTTAGCTCTTTTTGCGTCAAA**GCTGTC**TCAACCTGGG
CTCTCTTCG**GACAGC**TTTAGCTCTTTTGGCTTCAAA**GCTGTC**TGAACCCGGA
CTCTCTTCG**GACAGC**TTTGGCTCTTTTGGCTTCAAA**GCTGTC**TGAACCTGGA
CTCTCTTCG**GACAGC**TTTGGCTCTTTTGGCTTCAAA**GCTGTC**TGAACCTGGA
CTCTCTTCG**GACAGC**TTTGGCTCTTTCAGACATAAA**GCTGTC**TGAACCTGGA
CTCTCTTCGGACAACTTTGGCTCTTTCAGCCTTAAA**GCTGTC**TAAACCTGGA
CTCTCTTCGGACAACTTTAGCTCTTTTGGCTTCAAA**GCTGTC**TGAACCCAGA
CTCTCTTCG**GACAGC**TTTGGCTCTTTTGGCTTCAAA**GCTGTC**TGAACCTGGA
CTCTCTTCG**GACAGC**TTTAGCTCTTTCAGACATAAA**GCTGTC**TGAACCTAGG
CTCTCTTCGGACAACTTTGGCTCTTTCAGCCTCAAA**GCTGTC**TGAACCTGGA
CTCTCTTCG**GACAGC**TTTAGCTCTTTTGGATTCAAA**GCTGTC**TGAACCCAG

CTCTCTTCG**GACAGC**TTTGGCTCTTTCAGACATAAA**GCTGTC**TGAACCTGGA
CTCTCTTCG**GACAGC**TTTAGCTCTTTTGGCTTCAAA**GCTGTC**TGAACCCAGA
CTCTCTTCGGACAACTTTGGCTCTTTCAGCCTTAAA**GCTGTC**TGAACCTGGA
CTCTCTTCG**GACAGC**TTTAGCTCTTTTGGCTTCAAA**GCTGTC**TGAACCTGGA
CTCTCTTCGGACAACTTTGGCTCTTTCAGACATAAA**GCTGTC**TGAACCTGGA
CTCTCTTCGGACAACTTTGGCTCTTTCAGACATAAA**GCTGTC**TGAACCTAGG
CTCTCTTCGGACAACTTTGGCTCTTTCAGCCTTAAA**GCTGTC**TGAACCTGGACTCTCTTCGG
> AGTTCGGACA Con:1 reps=13 [4385482,4386323] l-int=842 l-seq = 52
AGTTCG**GACAGC**TTTTGCCACTTGGTAGCTTAA**GCTGTC**TAAAGTTACTCCG
AGTTCGGACAACTTTCACTCCCTGACTCTCAAA**GCTGTC**CGAGGTTGCTCCG
AGTTCG**GACAGC**TTTCACTCTCTGACTCTCGAAGTTGTCCGAAGTTACTCCA
AGTTCGGACAACTTTCACTCCTTTGGCCCTCAAA**GCTGTC**CGAAGTTGCTCCG
AGTTCGGACAACTTTTGGCACTTGGCTACTAAA**GCTGTC**TGAACTTGGCTAA
GGTTCGGATAGCTTTTCCTCCTTGGACCTCGAAGTTGTCCGAAGTTACTCCA
AGTTCG**GACAGC**TTTACTCTCTGACTCTCGAAGTTGTCCGAAGTTACTCCA
AGTTCG**GACAGC**TTTCACTCTCTGACTCTCGAAGTTGTCCGAAGTTACTCCA
AGTTCGGACAACTTTCACTCCTTGGCCCTCAAA**GCTGTC**CGAAGTTGCTCCG
AGTTCGGACAACTTTTGGCACTTGGCTACTAAA**GCTGTC**TGAACTTGGCTAA
GGTTCG**GACAGC**TTTTGCTCCTTGGCCCTCAAAGTTGTCCGAAGTTACTCCG
AGTTCA**GACAGC**TTTACTCTCTGACTCTCGAAGTTGTCCGAAGTTACTCCA
AGTTCGGACAACTTTCACTCCTTGGCCCTCAAA**GCTGTC**CGAAGTTGCTCCG
AGTTCGGACAACTTTTGGCACTTGGCTACTAAA**GCTGTC**TGAACTTGGCTAA
GGTTCG**GACAGC**TTTTCCTCCTTGGACCTCGAAGTTGTCCAATGTTACTCCA
AGTTCGGACAACTTTTGCTCCCTGGCCCTCAAA**GCTGTC**CGAAGTTGCTCCAAGTTCGGACA
> ACTTCGGACA Con:1 reps=13 [4426732,4427366] l-int=635 l-seq = 52
ACTTCGGACAAGTTCGATCCTCTTAGACTAAAA**GCTGTC**CGAACCCCGAGCA
ACTTCGGACAACTTCAATCCTCTGAGACCAAAA**GCTGTC**CGAACCCCGAGCA
ACTTCGGACAACTTCAATCCTCTGAGACCAAAA**GCTGTC**CGAACCCAGCACG
ACTTCGGACAACTTCGATTCTCTGTGACCAAAA**GCTGTC**CGAACCCAGCACG
ACTTCGGACAACTTCGATCCTCTGAGACCAAAA**GCTGTC**CGAACCCAGCACG
ACTTCGGACAACTTCGATCCTCTTAGACTAAAA**GCTGTC**CGAACCCTGAACA
ACTTCGGACAGGTTCGACCCTCTGTGACCTAAAGTTGTCCGAACCCCGAGCA
ACTTCG**GACAGC**TTCAATCTTCTGAGACCAAAA**GCTGTC**CGAACCCTGAACA
ACTTCGGACAGGTTCGACCCTCTGTGACCTAAAGTTGTCCGAACCCCGAGCA
ACTTCG**GACAGC**TTCAATCTTCTGAGACCAAAA**GCTGTC**CGAACCCAGCACG
ACTTCGGACAGGTTCGATCCTCTTAGACCAAAA**GCTGTC**CGAACCCCGCACG
ACTTCGGACAGGTTCGATCCGCTGAGACCAAAA**GCTGTC**CGAACCCCACGCAACTTCGGACA
> TACTTCAGAC Con:1 reps=27 [4437435,4438792] l-int=1358 l-seq = 52
TACTTCAGACAACTTGGCCTTGTAAAGCACGAAA**GCTGTC**CGAACACAGAGC
TACTTCAGACAACTTTGCTTTGCTATCCACAAAAACTGTCCGAACACAGAGC
TACTTCAGACAACTTTTCCTTGTAAAGCACGAAA**GCTGTC**CGAACACAGAGA
TACTTCAGACAACTTGGCCTTGTAAAGCACGAAA**GCTGTC**CGAACACAGAGA
TACTTCAGACAACTTTGCTTTGCTATCCACAAAA**GCTGTC**CGAACACAGAGA
TACTTCAGACAACTTGGCCTTGTAATTCCCAAAAGTTGTCCGAACACCAGGC
TACTTCAGACAACTTTTCCTTGTAAAGCACGAAA**GCTGTC**CGAACACTAGGC
TACTTCA**GACAGC**TTGGCCTTGTAATTCCCAAAAGTTGTCCGAACACCCGGC
TACTTCAGACAACTTTTCCTTGTAAAGCACAAAA**GCTGTC**CGAACACCAGGC
TACTTCAGACAACTTTTCCTGGTAAAGCACGAAA**GCTGTC**CGAACACTAGGC
TACTTCAGACAACTTGGCCTTGTAAAGCACGAAA**GCTGTC**CGAACACAGAGC
TACTTCAGACAACTTTGCTTTGCTATCCACAAAAACTGTCAGAACACAGAGC
TACTTCAGACAACTTTTCCTTGTAAAGCACGAAA**GCTGTC**CGAACACTAGGC
TACTTCA**GACAGC**TTGGCCCTGTAATTCCCAAAAGTTGTCCGAACACTGGGC
TACTTCA**GACAGC**TTGGCCTTGTAATTCCCGAAAGTTGTCCGAACACTGGGC
TACTTCAGACAACTTGGCCTTGTAAAGCACGAAA**GCTGTC**CGAACACAGAGC
TACTTCA**GACAGC**TTTGCTTTGCTATCCACAAAA**GCTGTC**CGAACACAGAGA
TACTTCAGACAACTTTTCCTGGTAAAACACAAAA**GCTGTC**CGTACACCAGGC
TACTTCAGACAACTTGGCCTTGTAATTCCCAAAAGTTGTCGAACACCAGGC
TACTTCAGACAACTTTTCCTGGTAAAGCACGAAA**GCTGTC**CGAACACTAGGC
TACTTCA**GACAGC**TTGGCCTTGTAATTCCCGAAAGTTGTCCACTGGGC
TACTTCAGACAACTTGGCCTTGTAAAGCACGAAAACTGTCCGAACACAGAGC
TACTTCA**GACAGC**TTTGCTTTGCTATCCACAAAA**GCTGTC**CGAACACAGAGC
TACTTCAGACAACTTTTCCTTGTAGAGCACGAAAACTGTCCGAACACAGAGC
TACTTCA**GACAGC**TTTGCTTTGCTATCCACAAAA**GCTGTC**CGAACACAGAGC
TACTTCAGACAACTTTTCCTTGTAAAGCACGAAAATTGTCCGAACACAGAGATACTTCAGAC
> GTTCAGACAG Con:1 reps=13 [4452843,4453477] l-int=635 l-seq = 52

GTTCA**GACAGC**TTTTGCCTCCTAGGACCTGAACCTGTCCAATCTTGACTCGT
GTTCA**GACAGC**TTTTGCTTCTTCGCTCATGAACCTGTCCGAACTTGACTCGG
GTTCA**GACAGC**TTTTGCCTCCTAGGACCTGAACCTGTCCAAACTTGACTCGG
GTTCA**GACAGC**TTTTGCCTCCTGGCCCCCGAA**GCTGTC**CGAACTTAGCTCAG
GTTCA**GACAGC**TTTTGCTTTCTGGCCACCGAA**GCTGTC**CGAACTTAGCTCGG
GTTCA**GACAGC**TTTTGCCTCCTGGCCCCCGAA**GCTGTC**CGAACTTAGCTCGG
GTTCA**GACAGC**TTTTGCCTCCTAGCCACCGATCCTGTCCGAACTTGACTCGG
GTTCA**GACAGC**TTTTGCCTCCTGGCCACCGAA**GCTGTC**CGAACTTAGCTCGA
GTTCA**GACAGC**TTTTGCCTCCTAGGACCTGAACCTGTCCAAACTTGACTCGG
GTTCA**GACAGC**TTTTGCTTTCTGGCCACCGATCCTGTCCGAACTTGACTCGG
GTTCA**GACAGC**TTTTGCCCGCTAGCCACTGAACCTGTCCGAACTTGACTCGG
GTTCA**GACAGC**TTTTGCCTCCTAGCCACCGTACCTGTCCGAACTTGACTCGGGTTCAGACAG
> AATGCTTTTC Con:1 reps=8 [4471530,4471903] l-int=374 l-seq = 52
AATGCTTTTCATTCCTTTTGTTGGATTTGGGCATGTG**AAAGGG**AATGAAAC
AATGCTTTTCATT**CCCTTT**GTTGGGCTTTGGGCATGTG**AAAGGG**AATCAAAC
AATGCTTTTCATTC**CCCTTT**GTTGGGCTTTGGGCATGTG**AAAGGG**AATGAAAC
AATGCTTTTCATTCCTTTTGTTGGGCTTTGGGCATGTG**AAAGGG**AATCAAAC
AATGCTTTTCATT**CCCTTT**GTTAGGCTTTGGGCATGTG**AAAGGG**AATGAAAC
AATGCTTTTCATTCCTTTTGTTGGGCTTTGGGCATGAGTAAGGGAATCAAAC
AATGCTTTTCATTCCTTTTGTTGGATTTGGGCATGTG**AAAGGG**AATCAAACAATGCTTTTC
> GTTCGGACAG Con:1 reps=23 [4520185,4521337] l-int=1153 l-seq = 52
GTTCG**GACAGC**TATGGGACCAAAAGATCAAAACCTGTCTGAAGTCAGATCAG
GTTCG**GACAGC**TATGGGACCAAAAGATCAAAACCTGTCTGAAGTCAGAGCAG
GTTCG**GACAGC**TATGGGACCAAAAGATCAAAA**GCTGTC**TGAAGTCGGAGCAG
GTTCG**GACAGC**TTGGACCCCAAAAGATCAAAA**GCTGTC**CGAAGTCAAGGTAA
GTTCG**GACAGC**TTGGACCCCAAAAGTTCAAAA**GCTGTC**CGAAGTCAAGGTAA
GTTCG**GACAGC**TTGGACCCCAATAAATCAAAA**GCTGTC**CGAAGTCAAGGTAA
GTTCG**GACAGC**TTGGACCCCAATAAATCAAAA**GCTGTC**CGAAGTCGGAGCAG
GTTCG**GACAGC**TTGGACCCCAATAAATCAAAA**GCTGTC**CGAAGTCAAGGTAA
GTTCG**GACAGC**TTAGGGACCAATAGATCAAAA**GCTGTC**TGAAGTCGGAGCAG
GTTCG**GACAGC**TTGGACCCCAATAAATCAAAA**GCTGTC**CGAAGTCAAGGTAA
GTTCG**GACAGC**TTAGGGACCAATAGATCAAAACCTGTCTGAAGTCGGAGCAA
GTTCG**GACAGC**TATGGGACCAAAAGATCAAAA**GCTGTC**GAAGTCGGAGCAG
GTTCG**GACAGC**TTGGACCCCAATAAATCAAAA**GCTGTC**CGAAGTCGGAGCAA
GTTCG**GACAGC**TATGGGACCAAAAGATCAAAACCTGTCTGAAGTCAGATCAG
GTTCG**GACAGC**TATGGGACCAAAAGATCAAAA**GCTGTC**GAAGTTGGAGCAG
GTTCG**GACAGC**TTGGACCCCAAAAGATCAAAA**GCTGTC**CGAAGTCAAGGTAA
GTTCG**GACAGC**TTGGACCCCTATAAATCAAAA**GCTGTC**CGAAGTCAAGGTAA
GTTCG**GACAGC**TCGGACCCCAATAAATCAAAA**GCTGTC**CGAAGTCAAGGTAA
GTTCG**GACAGC**TTAGGGACCAATAGATCAAAA**GCTGTC**TGAAGTCGGAGCAG
GTTCG**GACAGC**TTGGACCCCAATAGATCAAAA**GCTGTC**TGAAGTCGGAGCAG
GTTCG**GACAGC**TTGGACCCCAATAAATCAAAA**GCTGTC**CGAAGTCAAGGTAA
GTTCG**GACAGC**TTAGGGACCAATAGATCAAAA**GCTGTC**CGAAGTCGGAGCAAGTTCGGACAG
> CAGGCAACTT Con:1 reps=4 [4542766,4542983] l-int=218 l-seq = 52
CAGGCAACTTGATTATAGAAAGC**CTATGA**TTCACTTACACCATATTTTCGCT
CAGGCAACTTGA**TCATAG**AAGGT**CTATGA**TTTACTTCCTCCTCGTTTTCGT
CAGGCAACTGAA**TCATAG**AAGGCCTATGGTTTACTTTCTCCTCGTTTTCGTC
CAGGCAACTTGA**TCATAG**AAGGCCTATGGTTTACTTGCTCCTCGTTTTCGTCCAGGCAACTT
> ATT**CCCTTT**G Con:1 reps=10 [4572662,4573194] l-int=533 l-seq = 52
ATT**CCCTTT**GTCGGAGAAATTACACAGG**AAAGGG**AACGAAAGGAGCGCTTTG
ATT**CCCTTT**GTCGGAGAAAATACACAGG**AAAGGG**AACGAAAGGAGCGCTTTG
ATT**CCCTTT**GTCGGAGGAAATACACAGG**AAAGGG**AACGAAAGGAGTGCTTTG
ATT**CCCTTT**GTCGGAGGAAATACACAGG**AAAGGG**AACGAAAGGAGCGCTTTG
ATT**CCCTTT**GTTGGAGAAAATACACAGG**AAAGGG**AACGAAAGGAGTGCTTTCA
ATTCCCTTAGTCGGAGAAAATACACAGG**AAAGGG**AACGAAAGGAACGCTTTG
ATT**CCCTTT**GTCGGAGAAAATACACTGGAAAGGTAACGAAAGATGTGCTTTG
ATT**CCCTTT**GTCGGAGAAAATACACTGG**AAAGGG**CACGAAAGGAGCGCTTTCA
ATT**CCCTTT**GTCGGAGAAAATACACAGG**AAAGGG**AACGAAAGGAGCGCTTTG
ATT**CCCTTT**GTCGGAGAAAATACACTGG**AAAGGG**AACGAAAGGAGCGCTTTGATT**CCCTTT**G
> TGAACCTGTC Con:1 reps=18 [4704557,4705503] l-int=947 l-seq = 52
TGAACCTGTCTGAACTTACCTTCACTTCA**GACAGC**TTTTGGCTTCTGGCACA
TGAACCTGTCCGAACTTGCCTTCACTTTG**GACAGC**TTTCGGCTCCTGGCGCT
TGAACCTGTCCGAACTTGTCTCCACTTCG**GACAGC**TTTCGGCTTCTGGCGCT
TGAACCTGTCCGAAGTTGAGCACTCTTCGGACAACTTTCGGCTTCTAACGCA
TGAACCTGTCTCAAGTTGCGCACTCTTCG**GACAGC**TTTCGGCTCCTGGCGCA

TGAACCTGTCCGAACTTGCCTTCACTTCA**GACAGC**TTTCGGCTTCTAGCACA
TGAACCTGTCCGAACTTGCCTTCACTTCA**GACAGC**TTTCGGCTTCTAGCACA
TGAACCTGTCCGAACATGCCTTCACTTCA**GACAGC**TTTCGGCTTCTAACGCA
TGAACCTGTCTCAAGTTGCGCACTCTTCG**GACAGC**TTTCGGCTTCTGGCGCA
TGAACCTGTCCGAACTTGCCTTCACTTCA**GACAGC**TTTCGGCTTCTGGCGGA
TGAACCTGTCTCAAGTTGCGCACTCTTCG**GACAGC**TTTCGGCTCCTGGCGCA
TGAACCTGTCCGAACTTGCCTTCACTTCA**GACAGC**TTTCGGCTTCTAGCACA
TGAACCTGTCCGAACTTGCCTTCACTTCA**GACAGC**TTTCGGCTCCTGGCGCG
TGAACCTGTCCGAACTTGCCTTCACTTCG**GACAGC**TTTCGGCTTCTGGCGCA
TGAACCTGTCTCAAGTTGCGCACTCTTCG**GACAGC**TTTCGGCTCCTGGCGCA
TGAACCTGTCCGAACTTGCCTTCACTTCG**GACAGC**TTTCGGCTTCTGGCGCT
TGAACCTGTCCGAACTTGTCTCCACTTCG**GACAGC**TTTCGGCATCTGGCGCG
TGAACCTATCCGAACTTGCCTTCACTTCG**GACAGC**TTTCGGCTCCTGGCGCGTGAACCTGTC
> GCTACTTCAG Con:1 reps=11 [4753408,4753938] l-int=531 l-seq = 52
GCTACTTCA**GACAGC**TTGACCCTAAAACCATCCAAACCTGTCCGAACCCCAT
GCTACTTCA**GACAGC**TTTAGCCTAAAACCAATCAAACCTGTCCGAACCCAAC
GCTACTTCA**GACAGC**TTGACCCTAAAACCATCCAAACCTGTCCGAACCCCAT
GCTACTTCA**GACAGC**TTTAGCCTAAAACCAATCAAACTTGTCCGAATCCAAC
GCTACTTCA**GACAGC**TTTAGCCTAAAACCAACCAAAGTTGTCCAAACCCAAC
GCTACTTCA**GACAGC**TTTAACCTAAAACCAACTAAAGTTGTCCGAACTCAAC
GCTACTTCA**GACAGC**TTTAGCCTAAAACCAACCAATGTTGTCCGAACCCAAC
GCTACTTCA**GACAGC**TTTAGCCTAAAACCCCCAAAAGGTGTCCGAACCCAAA
GCTACTTCA**GACAGC**TTTAACCTAAAACCGACCAAACCTGTCCGAACCCAAC
GCTACTTCA**GACAGC**TTTAACCTAAAACCGACCAAACCTGTCCGAACCCCATGCTACTTCAG
> CTTTCGTTCC Con:1 reps=6 [4783299,4783568] l-int=270 l-seq = 52
CTTTCGTT**CCCTTT**CTCGATCTTTTGCTGTAAG**AAAGGG**AACCTGAGAGCAG
CTTTCGTT**CCCTTT**CTTGGGCTCTTGGTGTAAG**AAAGGG**AACCAAAGAACAG
CTTTCGTT**CCCTTT**TTCGAGCTTTTGGTGTAAG**AAAGGG**AACCTGAGAACAC
CTTTCGTT**CCCTTT**CATGGGCTCTTGGTGTAAGAAGGGAACCTGAGAACAC
CTTTCGTT**CCCTTT**CTTGGGCTTTTGGCGTGAG**AAAGGG**AACCTGAGAACACCTTTCGTTCC
> TACTTCGGAC Con:1 reps=26 [4937598,4938907] l-int=1310 l-seq = 52
TACTTCG**GACAGC**TTTTCCTCTTCGCACACTCAA**GCTGTC**TGAACCTTACCC
TACTTCG**GACAGC**TTTTCCTCTCCACACACCCTAGCTTCTGAACCTTACCC
TACTTCG**GACAGC**TTTTCCTCTTCTCACACTCAAGCTGGCTGAACCTTACCC
TACTTCG**GACAGC**TTTTCCACTTTGCACACCAAA**GCTGTC**TGAACCTCACTC
TACTTCG**GACAGC**TTTTCCCCTCCTAACACCAAA**GCTGTC**TGAACTTCACTC
TACTTCG**GACAGC**TTTTCCCATCCTAACACCAAA**GCTGTC**TGAACTTCACCC
TACTTCG**GACAGC**TTTTCCACTTTGCACACCAAA**GCTGTC**TGAACTTCACTC
TACTTCG**GACAGC**TTTTCCTCTTTGCACACCAAA**GCTGTC**TGAACCTTACCC
TACTTCG**GACAGC**TTTTCCCCTCCTAACACCAAA**GCTGTC**TGAACTTCACTC
TACTTCG**GACAGC**TTTTCCTCTTTGCACACCAAA**GCTGTC**TGAACCTCACTC
TACTTCG**GACAGC**TTTTCCTCTTTGCACACCAAAGCTGGCTGAACCTTACTC
TACTTCG**GACAGC**TTTTCCCATCCTAACACAAAA**GCTGTC**TGAACCTCACTC
TACTTCG**GACAGC**TTTTCCCCTCCTAACACCAAA**GCTGTC**TGAACTTCACTC
TACTTCG**GACAGC**TTTTCCCATCCTAACACCAAA**GCTGTC**TGAACTTCACCC
TACTTCG**GACAGC**TTTTCCCCTCCTAACACCAAA**GCTGTC**TGAACCTTACTC
TACTTCG**GACAGC**TTTTCCTCTTTGCACACCAAA**GCTGTC**TGAACCTTACTC
TACTTCG**GACAGC**TTTTCCATTTGGCACACCAAA**GCTGTC**TGAACCTCACTC
TACTTCG**GACAGC**TTTTCCTCTTTGCACACCAAA**GCTGTC**TGAACCTCACTC
TACTTCG**GACAGC**TTTTCCCCTCCTAACACCAAA**GCTGTC**TGAACTTCACTC
TACTTCG**GACAGC**TTTTCCCATCCTAACACCAAA**GCTGTC**TGAACCTCACTC
TACTTCG**GACAGC**TTTTCCATTTCGAACACCAAG**GCTGTC**TGAACCTCACTC
TACTTCG**GACAGC**TTTTCCTCTCCACACACCCTA**GCTGTC**TGAACCTTACTC
TACTTCG**GACAGC**TTTTCCTCTTTGCACACCAAA**GCTGTC**TGAACCTCACTC
TACTTCG**GACAGC**TTTTCCTCTTTGCACACCAAA**GCTGTC**TGAACTTCACCC
TACTTCG**GACAGC**TTTTCCTCTCCTAACACCAAA**GCTGTC**TGAACCTCACTCTACTTCGGAC
> GGAATCAAAC Con:1 reps=8 [5084517,5084891] l-int=375 l-seq = 52
GGAATCAAACAACTCTTTTCATT**CCCTTT**GTTGGGCTTTTAACATGAGAAAG
GGAATCAAACAACTCTTTTCATT**CCCTTT**GTTGGGCTTTAGGCATGAGAAAA
GGAATCAAACAACTCTTTTCATT**CCCTTT**GTTGGGCTTTAGGCATGAGAAAG
GGAATCAAACAACTCTTTTCATT**CCCTTT**GTTGGGCTTTAGGCATGAGAAAA
GGAATCAAACAACTCTTTTTATT**CCCTTT**GTTGGGCTTTAGGCATGAGAAAA
GGAATCAAACAACTCTTTTCATT**CCCTTT**GTTGGGCTTTAGGCATGAGAAAG
GGAATCAAACAACTCTTTTCATT**CCCTTT**GTTGGGCTTTTAACATGAGAAAAGGAATCAAAC
> AAGTGAATCA Con:1 reps=4 [5108997,5109219] l-int=223 l-seq = 52

AAGTGAATCATCATAAGCTTCT**CTATGA**CCCAGTTGCCTGAGTGAAAACAAAGAGA
AGGTGAATCATTGGCTCTTTATGATCCAGTTGCCTGAGCGGAAACAAGGAGA
AAGTGAATCATTGGCTCT**CTATGA**TTCAGTTGCTAAAGCGGAAACAAGGAGA
AAGTGAATCATTGGCTCT**CTATGA**TTCAGTTGCTAAAGCGGAAACAAGGAGAAAGTGAATCA
> AAAA**GCTGTC** Con:1 reps=15 [5152559,5153297] l-int=739 l-seq = 52
AAAA**GCTGTC**TGAACTTGAGCCTACTTCGGACAACTTAGGGAGCCATGCGGC
AAAA**GCTGTC**TGAACTTGAGCTTACTTCG**GACAGC**TTTAGGAGCCATGCGGC
AAAA**GCTGTC**TGAACTTGAGCCTACTTCG**GACAGC**TTAGGGAGCCATGCGGC
AAAA**GCTGTC**TGAACTTGAGCTTACTTCG**GACAGC**TTTAGGAGCCATGAGGT
AAAA**GCTGTC**TGAACTTGAGCCTACTTCG**GACAGC**TTTAGGAGCCATGAGGT
AAAA**GCTGTC**TGAACTTGAGCCTACTTCGGACAACTTTAGGAGCCATGCGGC
AAAA**GCTGTC**TGAACTTGAGCCTACTTCG**GACAGC**TTAGGGAGCCATGCGGC
AAAA**GCTGTC**TGAACTTGAGCTTACTTCG**GACAGC**TTTAGGAGCCATGCGGT
AAAA**GCTGTC**TGAACTTGAGCCTACTTCG**GACAGC**TTTAGGAGCCATGAGGT
AAAA**GCTGTC**TGAACTTGAGCCTACTTCGGACAACTTTAGGAGCCATGCGGC
AAAA**GCTGTC**TGAACTTGAGCCTACTTCG**GACAGC**TTTAGGAGCCATGAGGT
AAAA**GCTGTC**TGAACTTGAGCCTACTTCGGACAACTTTAGGAGCCATGCGGC
AAAA**GCTGTC**TGAACTTAAGCCTACTTCGTACAGCTTTAGGAGCCATGCGGC
AAAA**GCTGTC**TGAACTTGAGCTTACTTCG**GACAGC**TTGGAGAGACAAACGACAAAA**GCTGTC**
> CTGTTCAGAC Con:1 reps=10 [5249431,5249908] l-int=478 l-seq = 52
CTGTTCAGACATCTTGCCTCTTTTTCCGACTGAAGTTGTCCGAAGTTGCGCT
CTGTTCA**GACAGC**TTCTCTCTTTTTCTAGCTAAATCTGTCCGAAGTTGCGCT
CTGTTCAGACATCTTGCCTCTTTTTCCGACTGAAGTTGTCCGAAGTTGCGCT
CTGTTCA**GACAGC**TTTCCTCTTTTCTAGCTAAG**GCTGTC**CGAAGATAGGCT
CTGTTCA**GACAGC**TTTCCTCTTTTTCTAACTAAA**GCTGTC**TGAAGATCGGCC
CTGTTCA**GACAGC**TTTCCTCTTTTTCTAGCTAAA**GCTGTC**CGAAGATAGGCT
CTGTTCA**GACAGC**TTCTCTCTTTTTCTAGCTAAATCTGTCCGAAGTTGCGTT
CTGTTCA**GACAGC**TTCTCTCTTTTTCTAGCTAAATCTGTCCGAAGTTGCGTT
CTGTTCA**GACAGC**TTCTCTCTTTTTCTAGCTAAA**GCTGTC**CGAAGTTGCGCTCTGTTCAGAC
> ACTTCGGACA Con:1 reps=10 [5267087,5267720] l-int=634 l-seq = 52
ACTTCG**GACAGC**TTTAGGGCTCAGACAGAAAGAGCTATCTGAACTTAAACTA
ACTTCGGATTCTTCAAGCTCCAGACAGGGAAA**GCTGTC**CGAACACAAGCCG
ACTTCG**GACAGC**TTTAGGACTCAGACAGAGAAAACCGTCCGAACACAAGCTA
ACTTCG**GACAGC**TTCAGCACTCAAACGGAGAAA**GCTGTC**CGAACACAAGCCA
ACTTCGGACACCTTCAGCCTCCTTACAGAGAAAACTGTCCGAACACAAGCCA
ACTTTGGACACCTTCTGCGCTCAAACGGAGAAA**GCTGTC**TGAACACAAGGCC
ACTTCGGACACCTTCAGCGCTCAAACGAGTCAA**GCTGTC**CGAACCTATGCTA
ACTTCG**GACAGC**TTTAGGACTCAGACAGAGAAAACTGTCCGAACATAAGTTA
ACTTCG**GACAGC**TTCAGCACTCAAACGGAGAAA**GCTGTC**CGAACACAAGCCA
ACTTCGGACACCTTCAGCCTCCTTACAGAAAAA**GCTGTC**CGAACACAAACCA
ACTTTG**GACAGC**TTCAGCGCTCAAACGAGGAAA**GCTGTC**CGAACCTATGCCA
ACTTCG**GACAGC**TTCAAGCTCCAGACAGGGAAA**GCTGTC**CGAACACAAGCCAACTTCGGACA
> AA**GCTGTC**TG Con:1 reps=33 [5404130,5406169] l-int=2040 l-seq = 52
AA**GCTGTC**TGAAGTTACCCTAACTTCGAACAGCCTGACTCCGAAAAACCCTA
AA**GCTGTC**TGAACCTACCCCACCTTCG**GACAGC**TTGACTCCCTAAAACCCAA
AA**GCTGTC**TGAACCTACTCCACCTTCG**GACAGC**TTGACTCCCTAAAAACCAA
AA**GCTGTC**TGAACCTACCCCACCTTCGGACAACTTGACTCCGAAAAACCCAA
AA**GCTGTC**TGAACCTACTCCACCTTTGGACAACTTGACTCCGAAAAACCCAA
AA**GCTGTC**TGAACCTACCCTGACTTCGGACAACTTGACTCCGAAAAACCCAA
AA**GCTGTC**TGAACCTACTCCACCTTTGGACAACTTGACTCCGAAAAACCCAA
AA**GCTGTC**TGAACCTACCCTGACTTCGGACAACTTGACTCCGAAAAATCCAA
AA**GCTGTC**TGAACCTACCCTGACTTCGGACAACTTGACTCCGAAAAATCCAA
AA**GCTGTC**TGAACCTACTCCACCTTCGGACAACTTGACTCCGAAAAACCCAA
AA**GCTGTC**TGAACCTACCCCACCTTCG**GACAGC**TTGACTCCTAAAAACCCAA
AA**GCTGTC**TGAACCTACTCCACCTTCG**GACAGC**TTGACTCCCTAAAACCCAA
AA**GCTGTC**CGAACCTACTCCACCTTCG**GACAGC**TTGACTCCCTAAAACCCAA
AA**GCTGTC**TGAACCTACCCCACCTTCG**GACAGC**TTGACTCCCTAAAACCCAA
AA**GCTGTC**CGAACCTACTCCACCTTTG**GACAGC**TTGACTCCCTAAAACCCAA
AA**GCTGTC**CGAACCTTCCCCACCTTCG**GACAGC**TTGACTCCCTAAAACCCAA
AA**GCTGTC**CGAACCTACCCCACCTTCGGACAACTTGACTCCTAAAACCCATA
AA**GCTGTC**TGAACATTACTCGACTTCG**GACAGC**TTCACTCCTAAAAACCATA
AA**GCTGTC**TGAACATTACTCGACTTCG**GACAGC**TTGACTCCGAAAAACCCAA
AA**GCTGTC**TGAACCTAATCCACCTTTGGACAACTTGACTCCGAAAAACCCAA
AA**GCTGTC**TGAACCTACTCCACCTTTGGACAACTTGACTCCGAAAAACCCAA
AA**GCTGTC**TGAACCTACTCCACCTTTGGACAACTTGACTCCGAAAAACCCAA

AA**GCTGTC**TGAACCTACCCTGACTTCGGACAACTTGACTCCGAAAAATCCAA

AAGCTATCTGAACCTACTCCACCTTCGGACAACTTGACTCCGAAAAACCCAA

AA**GCTGTC**TGAACCTACTCCACCTTTGGACAACTTGACTCCGAAAAACCCAA

AA**GCTGTC**TGAACCTACCCTGACTTCGGACAACTTGACTCCGAAAAATCCAA

AAGCTATCTGAACCTACTCCACCTTCGGACAACTTGACTCCGAAAAACCCAA

AA**GCTGTC**TGAACCTACCCCACCTTCG**GACAGC**TTGACTCCTAAAAACCCAA

AA**GCTGTC**TGAACCTAATCCACCTTCG**GACAGC**TTGACTCCCTAAAACCCAA

AA**GCTGTC**TGAACCTACTCCACCTTCGGACAACTTGACTCCGAAAAACCCAA

AA**GCTGTC**TGAACCTTCCCCACCTTCG**GACAGC**TTGACTCCCTAAAACCCAA

AA**GCTGTC**CGAACCTACCCTGACTTCGGACAACTTGACTCCGAAAAACCCAA

AA**GCTGTC**TGAACCTACCCTGACTTCG**GACAGC**TTCACTCCTAAAAACCATA

AA**GCTGTC**TGAACATTACTCGACTTCG**GACAGC**TTCACTCCTAAAAACCATA

AA**GCTGTC**TGAACATTACTCGACTTCG**GACAGC**TTGACTCCTAAAAACCATA

AA**GCTGTC**TGAACCTTCCCCACCTTCG**GACAGC**TTCACTCCTAAAAACCATA

AA**GCTGTC**TGAACCTACCCTGACTTCG**GACAGC**TTCACTCCTAAAACCCATT

AA**GCTGTC**TGAACCTTCCCCGACTTCG**GACAGC**TTGACCGCTCAAAATCAAA

AA**GCTGTC**TGAACCTACCTCACCTTCG**GACAGC**TTGACTCCCAAGAAAGCTAAAA**GCTGTC**TG

B. mesonae (NZ KV440949.1). All tandem repeats with repeat 52-53

Signal sequences: TTTTC and TTCAT

```
> TTTTTACCGC Con:1 reps=7 [69909,70770] l-int=862 l-seq = 53
TTTTTACCGCTTAACCCTGGTTGAGGCGCTGCCCGGGCAAAATCAAGACTTCT
TTTTTACCGCTGACCTGGAATTGTGGTGCTGATCGGCAGAATCATGCCACTA
………….
TTTTTACCGGGTGACCCCCTTCTCAGCACACCGCGGGTAATAGAACTCTTTTC
TTTTTACCGCTTGACCTCCTTCTAAGCACTCCGCGGGCAATAGAACCCTCTTC
TTTTTACCGCCTGACCCCCTACTCAGCACTCCTCGGGCAATAGAACCATTTTC
TTTTTACCGCCTAACCCCATACTCAGCACTCCTCGGGCAATAGAACCCTCTTC
TTTTTACCGCCTCACACCCTTCAAAGGACCCAGCGGGCAATAGAACCATCTAC
TTTTTACCACCTTACCCCTTCCGAGCACCCAGCGGGCAATAGACCCCTCTTCTTTTTACCGC
> TCTACGTTAC Con:1 reps=9 [122623,123151] l-int=529 l-seq = 52
TCTACGTTACATTACAGTCTTTCTTTTCGCTCTTTCGGTCACGTAGACCCCT
TCTACGTTACCCTACTGCCTTTCTTTTCACTCTTTCAGTCACCTAGACCCCT
TGTACGTTACCCTACTGCCTTTCTTTTCACCTCTTCGGTCACGTTGACCCTT
TCTACGTTACCTTACATGCTTCCTTTTCATTCTTTTTGTCACGTAGACCCTT
TCTACGTTACCCTACAGCCTGGCTTTTCACTCTTTCGGTCACGTAGACCCTT
TCTACGTTACCCTACAGCCTGGCTTTTCACTCTTTCGGTCACGTAGACCCCT
TCTACGTTACCCTAATGCCTTTCTTTTCACCTCTTCGGTCACGTAGACCCCT
CCTATGTTACCCTAATGCCTTTCTTTTCACCTCTTCGGTCACGTAGACCCCT
TCTACGTTACCTACAACCTTTCTTTTCACCTTTTCGGTCACGTAGACATCC
TCTACGTTACCTACAACCTTTCTTTTCACCTTTTCGGTCACATAGACATCCTCTACGTTAC
> AACCTCTCTA Con:1 reps=6 [247094,247364] l-int=271 l-seq = 52
AACCTCTCTACGTGACCCTACGACCACATTTTCATCCGGTTCGGTCACGTAT
AACCTCTCTACGTGACCACACGATCACTTTTTCATCCGGTTCGGTCACGTAT
AACCTCTCTACGTGACCACACGATCACTTTTTCATCCGGTTCGGTCACGTAG
AACCTCTCTACGTGACCTTACGACCACATTTTCATCCGGTTCGGTCACGTAG
AACCTCTCTACGCGACCACACGACCACATTTTCATCCGGTTCGGTCACGTAGAACCTCTCTA
> ACCTTTTTCT Con:1 reps=9 [263113,263539] l-int=427 l-seq = 52
ACCTTTTTCTTCAGCTACGGTCGCGTAGAACCTCTCTATGTGACCCAACATC
ACCTTTTTCTTCTGCTACGGTCGCGTAGAACCTCTCTACGTGACCCAACATC
ACCTTTTTCTTCGTCTACGGTCGCGTAGAACCTCTCTATGTGACCCAACATC
ACCTTTTTCTTCTGCTGCGGTCACTTAGAACCTCTCTATGTGACCCAACATC
ACCTTTTTCTTCGTCTACGGTCACTTAGAACCTCCCTACGTGACCGAACATC
ACCTTTTTCTTCGGCTACGGTCACGTAGAACCTCCCTACGTGACCCAACATT
ACCTTTTTCTTCAGCTACGGTCGCGTAGAACCTCTCTATGTGACCCAACATC
ACCTTTTTCTTCAGCTACGGTCACGTAGAACCTCTTTACGTGACCGAACATCACCTTTTTCT
> AAGACAGTTT Con:1 reps=4 [309597,309763] l-int=167 l-seq = 52
AAGACAGTTTTGGAGATATTATTACTGATTTTGTCCTAAACAAGACTTATTA
AAGACAGTTTATTAGATTTTTCAACCTGTTTTGTTCTCAAGAAGCCTTATTG
AAGACAGTTTATATGATTTTTCAAACTGTATTGTCCTCAAGAAGCCTTATTGAAGACAGTTT
> CCTCTATGTG Con:1 reps=5 [379346,379564] l-int=219 l-seq = 52
CCTCTATGTGCACTTACCGAGCTTTTTCCATCGATTTAGTGTTCATTGAGAC
CCTCTATGTGCACTTCACAAGCTTTTTCAATCGGTTTCATGTTCATTGAGAT
CCTCTATGTGCACTTACCGAGCGTTTTCCATCGATTTTGTGTTCATTGAGAC
CCTCTATGTGCACTTCACAAGCTTTTTCAAACCGTTCCGTGTTCATTGAGACCCTCTATGTG
> TATCGAAGAC Con:1 reps=4 [512977,513195] l-int=219 l-seq = 52
TATCGAAGACATTTTCCGATGCTTCTACCTTCGTTTTTTCCTCAAGATGCCT
TATCGAAGACAAATTCTACCACTTCCACCCGCGTTTTGTCCTCAAGGCACCC
TATCGAAGACATTTTCGCCGCTTCTACCTTCGTTTTGTCCTCAAGATGCCT
TATCAAAGACAAATTCTACCGCTTTCCCACAGGTTTTGTCCTAAAGACGCCTTATCGAAGAC
> AAAGTGTTTC Con:1 reps=8 [834005,834379] l-int=375 l-seq = 52
AAAGTGTTTCTAATCGAGGCTGTTGGCGACATAACTCAGTACAAAAATGAGT
AAAGTGTTTCCAATCAAGGCTATTGGCGACATAACTCAGTGTAAAAATTTGA
AAAGTGTTTCCAATCAAGGCTATTGACGACATAGCTTGGTGTAAAAATTTGA
AAAGTGTTTCCAATCTGGGCTATTGGTGACATAACTCGGTACAAAAATGAGT
AAAGTGTTTCCAATCGAGGCTATTGGCGACATAGCTCAGTATAAAAATCTGA
AAAGTGTTTCCAATCAAGGCTGTTGGCGACATAACTCAGTGTAAAAAGCTGA
AAAGTGTTTCCAATCAAGGCTATTGGCGACATAACTCGGTACAAAAATGAGGAAAGTGTTTC
> GGGCACGTAG Con:1 reps=7 [972202,972524] l-int=323 l-seq = 52
```

GGGCACGTAGATGGAGGATACGTTACGTTGAGGCGAGGGATTTGCGCTTGAG
GGGCACGTAGAGGGGGCCTACGTTACCATGAGTCAGGATATTTACGCTGGAC
GGGCACGTAGACGCCTTCTACGTTTCCTTGAGGCGGTGAATTCGCGCTGGAT
GGGCACGTAGACGCGTTCTACGTTTCCTTGAGGCGGGATATTTACTCTGGAC
GGGCACGTAGACGCCTTCTACGTTTCCTTGAGACTGGATATTTACTCTGGAT
GGGCACGTAGACGCTTTCTATGTTACCTTGAGGCGGGATATTTACTCTGGATGGGCACGTAG
> CAATGCAACC Con:1 reps=8 [972743,973118] l-int=376 l-seq = 52
CAATGCAACCCCCCTTCCATTGACCAAAATATCAATC**TTTTC**CCCATTTCGGG
CAATGCAACCCGCCTCCATTAACC**GAAAA**TACAAA**TTTTC**CTCTTTTTTGGG
CAATGCAACCTCTTTCCATTGACCAAAACATCAATC**TTTTC**TTC**TTTTC**AGT
CAATGCAACCTCTTTCCATTGACCAAAATATCAACCTATTCTTC**TTTTC**GGG
CAATGCAACCCCCTTCCATTAACC**GAAAA**TACAAA**TTTTC**CTCT**TTTTC**GGG
CAATGCAACCCCTTTCCATTGACCAAAATATCAATC**TTTTC**CCCATTTCGGG
CAATGCAACCCGCCTCCATTAACC**GAAAA**TACAAT**TTTTC**CTCTTTTTTGGGCAATGCAACC
> GGGCACGTAG Con:1 reps=12 [1021216,1021902] l-int=687 l-seq = 52
GGGCACGTAGATCACGTCTACGGGACCGAACCTGCAGT**TTTTC**TGCTGATTG
GGGCACGTAGAGAGCGTCTACGCGACCGAACTGTCAGT**TTTTC**AGCTGGTTG
GGGCACGTAGAACCCTTCTACGTGACCGAACCGTCTCTATTTCAGCTGATTG
GGGCACGTAGAGAGCGTCTTCGTGACCGAACCTTCAGT**TTTTC**AGATGCTTG
GGGCACGTAGAACACGTCTTCGTCACCGAACCGTCTCTATTTCAGCTGCTTG
GGGCATGTAGAACACCTCTACGTGACCGAACTGTCAGT**TTTTC**AGCTGGTTG
GGGCACGTAGAACACCTCTACGTGACCGAACCGTCTCTATTTCAGCTGATTG
GGGCACGTAGAGAGCGTCTACGTGACCGAACCTTCAGT**TTTTC**AGATGCTTT
GGGCACGTAGAACACGTCTTCGTGACCGAACCGTCTCTATTTCAGCTGATTG
GGGCACGTAGAGAGCGTCTACGTGACCGAACCTTCAGT**TTTTC**AGATGCTTG
GGGAACGTAGAGCACGCCTACGTGACCGAACCTTCAGT**TTTTC**AGCTGATTG
GGGCACGTAGAGCACGACTACGTGACCGAACTGTCAGT**TTTTC**TGCTACTCG
GGGCACGTAGAACACGCCTACGTAACCAAACCGTCACTATTTCAGCTGATTGGGGCACGTAG
> TTCTTTTTAC Con:1 reps=4 [1410805,1411023] l-int=219 l-seq = 52
TTCTTTTTACAATAGACTGACTTTTTATCCTCTGCTGGTAATAGGGAACGG
TTCTTTTTACTGTTTCACCCTT**TTTTCAT**CTTCAACGGGCAATTGAAACCTT
CTCTTTTTACCGTCTCGCCCTT**TTTTC**TTCTCCCGCGGGCATTAGAAACCTT
TTCTTTTTACCGTTTCTCGCT**TTTTC**TATCCTCAACGGGCAATTGAAACC**TTTTC**TTTTTAC
> CGGGCACGTA Con:1 reps=5 [1467817,1468035] l-int=219 l-seq = 52
CGGGCACGTAGAACACTTCTAAATGACCTTATACTGCTAT**TTCAT**GCTCGTT
CGGGCACGTAGAACACTTCTATGTGACCTGACGCTGCT**TTTTCAT**GCCTGTT
CGGGCACGTAGACCGCTTCTATGTGACCTTGCGCTGATTG**TTCAT**GCCTGTT
CGGGCACGTAGACCCATTATACGTGACCTTACACCACTTTTTTATGCTCTTTCGGGCACGTA
> GACAGTTTTG Con:1 reps=5 [1832094,1832416] l-int=323 l-seq = 52
GACAGTTTTGATAGGTTTTGTGCTGATTTTGTCTTCAAGAACGCTTATTGAA
GACAGTTTTGCTAGTTTTTGAACTGGTTTTGTCTCCAAGAACACTTATTGAA
GACAGTTTTGCTAGT**TTTTC**AACTGGTTTTGTCTTCAAGAACACTTATTGAA
GACAATTTTGCTAGTTTTTGTGCTGGTTTTGTCTTCAACAAAGCTTATTGAA
GACAGTTTTGCTAG**TTTTC**GAGCGAGTTTTGTCTTCAAGAACGCTTTTTAAA
GACAGTTCCACTTGGTTTCGAGCGAGTTTTGTCTTTAAGAACGCTTTTTAAAGACAGTTTTG
> TATTGAAGAC Con:1 reps=9 [1863825,1864302] l-int=478 l-seq = 52
TATTGAAGACAAAACAAACTGCTTTCCTTATAAATTTGTCCCCAATACCTTC
TATTGAAGACAAAACGAGATCACTCCCTTTTATATTTGTCTCCAATACCCTC
TATTGAAGACAAATTGAGATCACTCCCTTTTAATTTTGTCCTCAATACTCTC
TATTGAAGACAAAATGAGATCCCTCCTTTTAAATTTGTCTCCAATACCCTT
TATTGAAGACAAATTGAAATCACTCCCTTTTAATTTTGTCTCCAATACCCTT
TATTGAAGACAAATTGAGATCACTCCCTTTTAATTTTGTCTCCAATACCCTT
TATTGAAGACAAATTGAGATCACTCCCTTTTATATTTGTCTCCAATACCCTC
TATTGAAGACTAAATGAGCTCACTCCCTTTTAAATTTGTCCCCAATACGCTC
TATCGAAGACATGACGAACTGCTTTCCTTATAAATTTGTCCCCAGTACCCTCTATTGAAGAC
> TGTGCACATT Con:1 reps=6 [1902145,1902519] l-int=375 l-seq = 52
TGTGCACATTGGAGTGTTTA**ATGAA**CACGAGGGAACGGACTATCCACATAGA
TGGGCACATTGGAGCGTTCA**ATGAA**CACGAGAGAATGGAATATCCACATAGA
TATGCACATTGAAGCGTTCC**ATGAA**CACGAGAGAATGGAATATCCACATAGA
TGTGCACATTGGAGCGTTCC**ATGAA**CACCAAAGAAAGGAATATCCACATAGA
TGTGCACATTGAAGCGTTCA**ATGAA**CATGAGAACGAG**GAAAA**TTCCCATAGT
TGTGCACATTGAAGTATTCA**ATGAA**CATCAGAGAAAGGAATATCCACAAAGT
TGTGCACATTGAAGCGTCCA**ATGAA**CAC**GAAAA**CAAG**GAAAA**TTCCCATAGTTGTGCACATT
> CTTTATTTTG Con:1 reps=7 [1907579,1907907] l-int=329 l-seq = 53
CTTTATTTTGACTGGTGACCTCCTTCTTGGTACCTTTGCGGGAAGAATCGGAC

CTTTATTTTGACCGGTGACCTCCTTCTTGCTACCTTTGCGGGAAGAATTACGC
CTTTATTTTGACCGACGACCTTCTTCTTGGTACCTTCACGGGAAGAATCAGAC
CTTTATTTTGACCGGTGACCTCCTTCTTGGTACCTTTGCGGGAAGAATCGGAC
CTTTATTTTGACCGGTGACCTCCTTCTTGATACCTTTGCGGGAAGAATTACGC
CTTTATTTTGACCGGCGACCTCCTTCTTGTTACCTTTGCGGAAAGAATCACGACTTTATTTTG
> ACCTTTTTCA Con:1 reps=5 [1916684,1916902] l-int=219 l-seq = 52
ACCTTTTTCATAGAGCTTCCTCTTTTTCGGGCAATGCACCCCACTCTCATCG
ACCTTTTTCATAGAGTTCCCTCTTTTTTGGGCAATGCAACCCACTCTCATTT
ACCTTTTTCATAGAGTTCCCTCTTTTTCGGGCAATGCAACCCGCTTGCATTT
ACCTTTTTCATTGAGTTTCCTCTTTTTTAGGCAATGCAACCCACTTTCATTTACCTTTTTCA
> TTTTGTCCTT Con:1 reps=11 [1920272,1920905] l-int=634 l-seq = 52
TTTTGTCCTTCATCGGGCTTATGAAGGACATTCCGGACCTGGATCCCGCCTG
TTTTGTCCTTCATCGACTCTATGAAGACCATTTTGGACTTGACCCACGCCTG
TTTTGTCCTTCATCAGCCTTATGAAGACCATTTTGAAACTAGCTCCCGGCTA
TTTTGTCCTTCATCTGCCTTATGAAGACCATTTTGGACTTAACCCACGCCTG
TTTTGTCCTTCATCGACCCTATGAAGACCATTTTGGCCTTGACCCACGACTG
TTTTGTCCTTCATCAGCCTTATGAAGACCATTTTGAACCTGGCTCCCGCCTA
TTTTGTCCTTCATCAGCCTTATGAAGACCATTTTGAACCTGGCTCACGCCTG
TTTCGTCCTTCATCAAACTTATGAAGACCATTTTGGTCCTGGTTCCCGGCTA
TTTTGGCCTTCATCGACCTTATGAAGACCATTTCCGACTTAACCCACGCCTG
TTTTGTCCTTCATCGACCCTATGAAGACCA
TTTTGGCCTTGACCCACGACTG
TTTTGTCCTTCATCGACCCTATGAAGACCATTTTAGCCTTGACCCACGCCTG
TTTTGTCCTTCTCGACCCTATGAGGACCATTTTGGACCTGGCTCCCGCCTGTTTTGTCCTT
> AAGTGTCTCC Con:1 reps=7 [1939675,1940049] l-int=375 l-seq = 52
AAGTGTCTCCAATAAGCTTCATTGGAGACAAAACTCTCTCTTTTTTAACGAG
AAGTGTCTCCAATAAGCTCCATTGAAGACAAAATCCGCTTCCTTTTCACGAA
AACTGTCTTTAATAAGCTTCATTGAAGACAAAATCCACTCTTTTTTCACGAA
AAGTGTCTCCAATAAGCTTCATTCAAGACAAAACCCGCTCCATGTTCACGAG
AAGTGTCTCCAATAAGCTCCATTGAAGACAAAATCCGCTCTTTTTTCACGAG
AAGTGTCTCCAATAAGCTCCATTGGAGACAAAACCCACTCTTTTTTCACGAA
AAGTGTCTCCAATAAGCTTCATTGGAGACAAAACTCTCTCTATTTTTACGAAAAGTGTCTCC
> TATGAAGGAC Con:1 reps=6 [1954829,1955099] l-int=271 l-seq = 52
TATGAAGGACATTTTGAACCTGGTTCCCAGCTATTTTGGCCTTCATCGGATT
TATGAAGGACATTTTGAACCCGGGTCCCAGCTATTTTGGTCTTCATCGGGTT
TATGAAGGACATTTTGAACCCGGGTCCCAGCTATTTTGGTCTTCATCGGGCT
TATGAAGGACATTTTGAACCCGGGTCCCAGCTATTTTGGTCTTCATCGGGCT
TATGAAGGACATTTTGAACCCGGGTCCCAGCTATTTTGATCTTCATCGGGCTTATGAAGGAC
> TTGACCAAAA Con:1 reps=4 [1966100,1966266] l-int=167 l-seq = 52
TTGACCAAAATAGTGATAAATCCTCTAAAAAGGGTAATGCAATATGGTTCCT
TTGACCAAAATAGTGATAAATCCTCTAAAAAGGGTAATGCATGCTGGTTCCA
TTGACCAAAATAGTGAAAAATCCTCTAAAAAGGGGAATGCAACGGGCTTCCTTTGACCAAAA
> CAAAATGGTC Con:1 reps=10 [2290722,2291200] l-int=479 l-seq = 52
CAAAATGGTCTTCATCAACCTTATGAAGACCAGTTCTCTTCGGCTTTTCATC
CAAAATGGTCTTCATCAACCTTATGAAGACCAGTTCTCTTCGGCTTTTCTTT
CAAAATGGTCTTCATCAACCTTATGAAGACCAGTTCCCTTCGGCTTTTCATC
CAAAATGGTCTTCATCACACTTATGAAGACCATTTCTCTTCGGCTTTTCGTT
CAAAATGGTCTTCATCACACTTATGAAGACCATTTCTCTTCGGCTTTTCATC
CAAAATGGTCTTCATCACACTTATGAAGACCATTTCTCTTCGGCTTTTCATC
CAAAATGGTCTTCATCACCTTTATGAAGACCATTTCCCTTCGGCTTTTCGTT
CAAAATGGTCTTCATCACACTTATGAAGACCATTTCTCTTTGGCTTTTCATC
CAAAATGGTCTTCAACAACCTTATGAAGACCATTTCTCTTTGGCTTTTCATCCAAAATGGTC
> GGTTTCGTGT Con:1 reps=9 [2299724,2300151] l-int=428 l-seq = 52
GGTTTCGTGTTCATTGAAGCCCTCTATGTGCACCTTCATCGTTTTTTTCATT
GGTTTCGTGTTCATTGAAATCCTCTATGTGCACTTTCCTCGCCTTTTTCATC
GGTTTCGTGTTCATTGAAGACCTCTATGTGCACTTTCCTCGCTTTTTTCATT
GGTTTCGTGTTCATTGAAGACCTCTATGTGCACTTGCCTCCCTTTTTTCATT
GGTTTCGTGTTCATTGAAGACCTCTATGTGCACTTTCCTCGTTTTTTTTCATT
GGTTTCGTGTTCATTGAAGACCTCTATGTGCACTTTCCTCGCTTTTTTCATC
GGTTTCGTGTTCATTGAAGCTCTCTATGTGCACTTGCCTCGCTTTTTTTATC
GGTTTCGTGTTCATTAAAGACCTCTATGTGCACTTTCAAACGTTTTCTCATTGGTTTCGTGT
> CACGTAGAAC Con:1 reps=5 [2317829,2318047] l-int=219 l-seq = 52
CACGTAGAACCTTTTTACGTTACCCATCTGCTTGATTTTTCGACCTTTCGGG
CACGTAGAACCCATCTACGTTACCCATCTGCTTGATTTTTCGATCTTTTGGG
CACGTAGAACCCTTCTACGTTACCTATCTGCTTGGTTTTTCGTCCTTTCGGG

CACGTAGAACCTTTCTACGTTACCCATTGACTTGGTTTTTCAGCCTTTCGGACACGTAGAAC
> AAAGTCACGT Con:1 reps=8 [2327343,2327770] l-int=428 l-seq = 52
AAAGTCACGTAGTAACCTTCTACGTGACCGTACTTCCTTTTTTCCCTGCTCT
AAAGTCACGTAGAAACCCTATACGCGACCGTACCTCCCTTTTTTTCTGCTCT
AAAGTCACGTAGAAACCCTATACACGACCTTACCTCCCTTTTTTTCTGCTCT
AAAGTCACGTAGAAACCCTCTACGTGACCTTACCTCCCTTTTTTTCTGCTCT
AAAGTCACGTAGAAACCCTCTACGTGACCGTACTTTCCTTTTTTCCTGCTCT
AAAGTCACGTAGAACTTCTCTACGTGACCGAATTTCCCTTTTTCCCTGCTCC
AAAGTCACGTAGAACTCCTCTACGTGACCTTACCTCCCTTTTTTCCTTGCCCA
AAAGGCACGTTGAAACCCTCTACAAGACCAAACCACTCTTTTTCCTTGCTCCAAAGTCACGT
> CTCATGAACC Con:1 reps=4 [2333462,2333680] l-int=219 l-seq = 52
CTCATGAACCCTTTATGAGAACAATTGCAGCACTTTCCTTCACCAGTCTGTT
CTCATGAACCTTCTATGAGAACTGGCCAGACCCCGCTTGATCCCTTTCTGTC
CTCACGAACCCTTTATGAGAACAATTGCAGCACTTTCCTTCACCAGTCTGTC
CTCATGAACCTTCCATGAGAACTCGCTTGGCCCTGCTTGACCCTTTTCTGTTCTCATGAACC
> GATGAAGGAC Con:1 reps=7 [2344320,2344642] l-int=323 l-seq = 52
GATGAAGGACATTTTCCATGGGAAACCTGCTTCGTTTGGTCTCCATCACGCC
GATGAAGGACATTTTGCATGGGAAGCCTGCTTCGTTTGGTCTTCATCACGCT
GATGAAGGACATTTTGCATGCGAAACCTGCTTCGTTTGGTCTTCATCACGCC
GATGAAGGACATTTTGCATGGGAAGCCTGCTTCGTTTGGTCTTCATCACGCC
GATGAAGGACATTTTGCATGCGAAACCTGCTTCGTTTGGTCTTCATCACGCC
GATGAAGGACATTTTGCATGGGAAACCTACTTCGTTTGGTCTCCATCACGCCGATGAAGGAC
> TGGAATCACT Con:1 reps=13 [2347811,2348444] l-int=634 l-seq = 52
TGGAATCACTTCTCGCGGGTTTTCTGCAAGTTTTGTTTCCAATGACCCTGAT
TGGAATCACTTCTCATGTTTTTTCTCCGAGTTTGTTTCCAATGACCCTGAT
TGGAATCACTTCTCTCTTTTTTTCTCCAAGTTTTGTTTCCAATGACCCTGAT
TGGAATCACTTTTCGTGGGTTTTCTCCGAGTTTTGTTTCAAATGACCCTGAT
TGGAATCACTTTTCTCTTTTTTCCTCTCAGTTTTGTTTCCAATGACCCTGAT
TGGAATCACTTCTCTCTTTTTTTCTCCAAGTTTTGTTTCCAATGACCCTGAT
TGGAATCACTTTTCGTGGGTTTTCTCCGAGTTTTGTTTCCAATAACCCTGAT
TGGAATCACTTTTCTCTTTTTTCCTCTCAGTTTTGTTTCCAATGACCCTGAT
TGGAATCACTTCTCTCTGTTTTTCTCCGAGTTTTGTTTCCAATAACCCTGAT
TGGAATCACTTTTCTCTTTTTTCCTCTCAGTTTTGTTTCCAATGACCCCGAT
TGGAATCACTTTTCTCTCTTTTTCTCCGAGTTTTGTTTCCAATAACCCTGAT
TGGAATCACTTTTCTCTCTTTTCCTCTCAGTTTTGTTTCCAATGACCTCGAATGGAATCACT
> AAAATGGTCT Con:1 reps=11 [2363645,2364175] l-int=531 l-seq = 52
AAAATGGTCTTCATCATCCTTATGAAGGACATTTCTCTTTGGCTTTTCTTTC
AAAATGGTCTTCATCTCCCTTATGAAGGACTTTTCTCTTCGGCTTTTCATTC
AAAATGGTCTTCATCAACCTTATGAAGGACTTTTCTCCTCGGCTTTTCATCC
AAAATGGTCTTCATCAACCCTATGAAGGACATTTCTCTTTGGCTTTTCATCC
AAAATGGTCTTCATCTCCCTTATGAAGGACTTTTCTCTTCAGATTTTCATCC
AAAATGGTCTTCATCTCCCTTATGAAGGACATTTCTCTTCAGATTTTCATTC
AAAATGGTCTTCATCATCCTTATGAAGGACATTTCTCTTCAGATTTTCATCC
AAAATGGTCTTCATCATCCTTATGAAGGACATTTCTCTTTGGTTTTTCATCC
AAAATGGTCTTCATCAACCTTATGAAGGACATTTCTCTTTGGCTCTTCATCC
AAAATGGTCTTCATCAACCTTATGAAGGACATTTCTCTTTGGTTTTTCATCCAAAATGGTCT
> TCTACGTTAC Con:1 reps=5 [2367649,2367868] l-int=220 l-seq = 52
TCTACGTTACCCTAAGCTCTTTTTTTCTCCCACTCGGGCACGTAGAAACCCC
TCTACGTTACCCAAGCTTCTATTTTTCCTCCGATTCGGGCACCTAGACCCCC
TCTACGTTACCTCAAGCCCTATTTTCCCTATCGTTCGGGCACGTAGAACCTC
TCTACGTTACCCAAGCCCTGATTTTTCCTCCGGTTCGGGCATCTAGAACCCTTCTACGTTAC
> CTTATTGAAG Con:1 reps=5 [2472381,2472599] l-int=219 l-seq = 52
CTTATTGAAGACAGTTTTAGTAGTTTTCGTAGCGATTTTGTCCCCAAGAGGC
CTTATTGAAGACAGTTTTAGTAGTTTTCGCAGCGGTTTTGTCTCCAAGAGGC
CTTATTGAAGACAGTTTTAGTAGTTTTCGCAGCGGTTTTGTCTCCTAGAGGC
CTTATTGAAGACAGTTTTAGTAGTTTTAACAGCAGGTTTGTCTCCAAGAGGCCTTATTGAAG
> TCTACGTTAC Con:1 reps=15 [2481435,2482171] l-int=737 l-seq = 52
TCTACGTTACCCTTGACCCTTATTTTTTGCTGGGTGGGCACCGTAGACACCT
TCTACGTTACCCTTAAACCTTATTTTTAGCTGGGTGGGCACGTAGACACCC
TCTACGTTACCCTTAAACCTTATTTTTAGCTGGGTGGGCACGTAGACACCC
TCTACGTTACCCTTGACCCTTATTTTTTACCTGATTGGGCACGTAGACACCC
TCTACGTTACCCTTGACTCTTATTTTTTAGCTGGGCGGGCACGTAGACACCT
TCTACGTTACCTTAGAACCTTATTTTTTAGCTGGGTGGGCACGTAGACACCC
TCTACGTTACCCTTGACCCTTATTTTTTGCCTGATCGGGCACGTAGACGCCT
TCTACGTTACCTTAGAACCTTATTTTTTACCTGATCGGGCACGTAGACACCC

TCTACGTTACCCTTAACCCTTATTTTTTAGCTGGGTGGGCACGTAGACACCT
TCTACGTTACCCTTAACCCGTATTTTTTAGCTGATCGGGCACGTAGACACCC
TCTACGTTACCCTTGACCCTTATTTTTTGCCTGATCGGGCACGTAGACGCCT
TCTACGTTACCTTAGAACCTTATTTTTTACCTGATCGGGCACGTAGACACCC
TCTACGTTACCCTTAAACCTTATTTTTTACATGATCGGGCACGTAGACGCCT
TCTACGTTACCCTTGACCCGTATTTTTTAGCTGGGCGGGCACGTAGACACCCTCTACGTTAC
> TT**ATGAA**GAC Con:1 reps=7 [2583930,2584252] l-int=323 l-seq = 52
TT**ATGAA**GACCAAAATTCTCTTCTGATTCCTCCAAAATGGCC**TTCAT**CCTTC
TT**ATGAA**GACCA**TTTTC**CTCTTCTGTCTCCTCCAAAATGGTC**TTCAT**CCTTT
TT**ATGAA**GACCAAAATTCTCTTCTGACTCCTCCAAAATGGTC**TTCAT**CCTTC
TT**ATGAA**GACCAATATTCTCTTCTGATTCCTCTAAAATGGTC**TTCAT**CCTTC
TT**ATGAA**GACCA**TTTTC**CTCTTCTGACTCCTCCAAAATGGTC**TTCAT**CCTTC
TT**ATGAA**GACCAAAATTCTCTTCCGAATCCTCCAAAATGGTC**TTCAT**CCTTCTT**ATGAA**GAC
> TGCTGTCTGA Con:1 reps=15 [2650478,2651320] l-int=843 l-seq = 52
TGCTGTCT**GAAAA**GGAAATTTGAATGTAGAGAACAAGTAGAGAGGCTCTAGG
TGCTGTCTGAGAAG**GAAAA**TTGAGTATAGAGAGCAAGTAGAAAGGTTCTAGC
CGCTGTCT**GAAAA**G**GAAAA**TAGGGTTTAGAGGGCAAGTAGAAAGGTTCTAGCT
GCTGTCTTGAAGG**GAAAA**TTGAGTATAGAGAGCAAGTAGAAAGACGCTAGG
TGCTGTCT**GAAAAGAAAA**ATTGGGTTCAGAGAGCAAGTAGAAAGGCCCTAGG
TGCTGTCT**GAAAA**G**GAAAA**TTGAGTATAGAGAGCAAGTAGAAAGACGCTAGG
TGCTGTCT**GAAAA**G**GAAAA**TTGAGTATAGAGAGCAAGTAGAAAGACGCTAGG
TGCTGTCT**GAAAAGAAAA**ATTGGGTTCAGAGAGGAAATAGAAAGGCCCTAGC
TGCTGTCT**GAAAA**G**GAAAA**TTGAGTATAGAGAGCAAGTAGAAAGACGCTAGG
TGCTGTCT**GAAAA**G**GAAAA**TTGAGTATAGAGAGCAAGTAGAAAGACGCTAGG
TGCTGTCT**GAAAA**G**GAAAA**TTGGGTTCAGAGAGCAAGTAGAAAGGCCTTAGC
TGCTGTCT**GAAAA**GGAAATTTGAATGTAGAGAACAAGTAGAGAGGCTCTAGG
TGCTGTCTGAGAAG**GAAAA**TTGAGTATAGAGAGCAAGTAGAAAGGCGCTAGG
TGCTGTCT**GAAAA**G**GAAAA**TTGAGTATAGAGAGCAAGTAGAAAGACGCTAGG
TGCTGTCT**GAAAA**G**GAAAA**TTGGGTTCAGAGAGCAAGTAGAAAGGCCTTAGC
TGCTGTCT**GAAAA**GGAAATTTGAATGTAGAGAACAAGTAGAGAGGCTCTAGCTGCTGTCTGA
> GCATAAAAAA Con:1 reps=7 [2851663,2851985] l-int=323 l-seq = 52
GCATAAAAAATCATCGAAACTGTCTTCAATAAGCCGTCTTGGATACAAAACG
GCATAAAAAATCATCGAAACTGTCTTCAATAAGCCGTCTTGAAGACAAACCA
GCATAAAAAATCATCGAAACTGTCTTCAATAAGCCGTCTTGGATACAAAACG
GCATAAAAAATCATCGAAACTGTCTTCAATAAGCCGTCTTGGATACAAAACG
GCATAAAAAATCATCGAAACTGTCTTCAATAAGCCGTCTTGAAGACAAACCA
GCATAAAAAATCATCGAAACTGTCTTCAATAAGCCGTCTTGAAGACAAACCAGCATAAAAAA
> ATGAGAACTG Con:1 reps=7 [2907285,2907659] l-int=375 l-seq = 52
ATGAGAACTGGAATCAC**GAAAA**GCAAGGCCATTTTGTCCTC**ATGAA**GCGTTC
ATGAGAACTGGAACCAA**GAAAA**GCGCGGTAGTTTTGTC**TTCATGAA**GCGTTC
ATGAGAACTGAAACCAA**GAAAA**GCACGGCCGTCTTGTCCTC**ATGAA**GCGTTC
ATGAGAACTGAAACCAG**GAAAA**GCGCGGCGGTCTTGTTCTCATAAAGCGTTC
ATGAGAACTGAAACCAG**GAAAA**GCGTGGCGGTCTTGTCCCC**ATGAA**GCGTCC
ATGAGAACAGATACCAA**GAAAA**GAGCGACGTCCTTGTCCTC**ATGAA**GCGTTT
ATGAGAACTGGAACCACG**GAAAA**CGAGGCCGTTTTGTCCCC**ATGAA**GTG**TTCAT**GAGAACTG
> TGCATTGCCC Con:1 reps=6 [2912216,2912485] l-int=270 l-seq = 52
TGCATTGCCCAAAAACCAGGGAGAGCAGCAATTTTGGTCAAAGGAAGAGGG
TGCATTGCCCAAAAATCGTGGAGAGCGGCTATTTTGGTCAAAGGAAATGGGA
TGCATTGCCC**GAAAA**ACGGGAAGAGCCACTAATTTGGTCAAAGGAAGAGGGA
TGCATTGCCCAAAAAACGGGAAGAGCCACTAATTTGCTCAAAGGAAGAGGGA
TGCATTGCCC**GAAAA**ACGGGAAGAGCCACTAATTTGGTCAAAGGAAGAGAGTTGCATTGCCC
> **ATGAA**GACCA Con:1 reps=14 [2922406,2923195] l-int=790 l-seq = 52
ATGAAGACCAAATAGCCCAGGCGCAGATAAGAAATGGTC**TTCAT**AAGCGGG
ATGAAGACCAAAACAGTCGTGGTGCAGGTAA**GAAAA**GGTC**TTCAT**AAGCGGG
ATGAAGACCAAAACAGTCGTGGTGCAGGTAAGAAATGGTC**TTCAT**AAGCGGG
ATGAAGACCAAAATAGCTGTGGTCGAGGTGCGAAATGGTC**TTCAT**AAGCGGG
ATGAAGACCAAAACAGCCGTGGCCGAGGTGCGAAATGGTC**TTCAT**AAGCCGG
ATGAAGACCAAAACAGCCGGGTTGTAGGTAAGAAATGGTC**TTCAT**AAGCCGA
ATGAAGACCAAAACAGCTGTGGCCGAGGTAAGAAATGGTCTTCACAAGCAGG
ATGAAGACCAAAACAGCTGTGGCCGAGGAAC**GAAAA**GGTC**TTCAT**AAGCGGG
ATGAAGACCAAAACAGCCGTGCCCGAGGAAC**GAAAA**GGTC**TTCAT**AAGCGGG
ATGAAGACCAAAACAGCTGTGGCCGAGGAAC**GAAAA**GGTC**TTCAT**AAGCGGG
ATGAAGGCCAAAACAGCTGTGGCCGAGGTAAGAAATGGTCTTTATAAGCCGA
ATGAAGACCAAAACAGCCGTGGCCGAGGAAC**GAAAA**GGTC**TTCAT**AAGCCGA
ATGAAGACCAAAACAGCCGTGCCCGAGGAAC**GAAAA**GGTCTTAATAAACCGA

ATGAAGACCAAAACAGCCGTGGCCGAGGAACGAAATGATCTTCACAAGCAGG
ATGAAGATCAAAACAGCTGTGGCCGAGGA**ATGAA**ATGGTC**TTCAT**AAGCCGA**ATGAA**GACCA
> G**ATGAA**GGAC Con:1 reps=8 [2936759,2937132] l-int=374 l-seq = 52
G**ATGAA**GGACAAAACAGGCGGGATCCAGGTCCAAATGGTC**TTCAT**AAGGCT
G**ATGAA**GGACAAAACAGGCGGGAGCCAAGTTCAAAATGGTC**TTCAT**AAGGCT
G**ATGAA**GGACAAAACAAGCGGGATCCAGGTTCAAAATGGTC**TTCAT**AAGGCT
G**ATGAA**GGACAAAACAGGCGGGATCCAGGTTCAAAATGGTC**TTCAT**AAGGCT
G**ATGAA**GGACAAAACAGGCGGGATCCAGGTTCAAAATGGTC**TTCAT**AAGCCC
G**ATGAA**GGACAAAACAGGCGGAATCCAGGTCTAAAATGGTC**TTCAT**AAGGCT
G**ATGAA**GGACAAAACAGGCGGGAGCCAAGTTCAAAATGGTC**TTCAT**AAGGCTG**ATGAA**GGAC
> AAACTGTCTT Con:1 reps=4 [2942377,2942598] l-int=222 l-seq = 52
AAACTGTCTTTAATAAGGAATCTTGGAGACAAACCGCCCTACAGAAATTGAG
AAACTGTCTTCAAAAAAGGTTCTTCTTGTAGACAAACGGCCCTACAAAAATGGAG
AAACTGTCCTCAAAAAGGGTTCTTGGAGACAAACGGCCCTACAAAAATGGAG
AAACTGTCTTCAATAAGGCTTCTTAAGGACAAAATGCCTTACAAAATAGT**GAAAA**CTGTCTT
> GACCGAGCAG Con:1 reps=12 [3080314,3080998] l-int=685 l-seq = 52
GACCGAGCAGCAG**GAAAA**ATAGTCGAAAGGGTCGCATAGAGAGGTTATATCC
GACCGAGCAGCTG**GAAAA**ATAGTCGGTAGGGTCGCATAGAGAGGTTCTAGGC
GACCGAGCAGCTG**GAAAA**ATAGTCGGTAGGGTCGCATAGAGAGGTTCTAGGC
GACCGAGCAGCTG**GAAAA**TAGGAAGAAGGGTCGCGTAGAGAGGCCCTACGC
GACCGAGCGGCT**GAAAA**ATTAGGAAGAAGAGTCGCGTAGAGAGATTCTACGT
GACCGAGCAGGTG**GAAAA**ATAGGAAGAAGGGTCGCGTAGAGAGGTTCTAGGC
GACCGAGCAGCT**GAAAA**AATAGGAAGAAGAGTCGCGTAAAGAGGTTCTATGC
GACCGAGCAGCTG**GAAAA**AAAGAAAGAAGAGTCGCGTAGAGAGATTCTACGT
GACCGAGCAGCTG**GAAAA**ATAGAAAGAAGAGTCGCGTAGAGAGGTTCTATGC
GACCGAGCAGGTG**GAAAA**ATAGGAAGAAGAGTCGCGTAGAGAGGTTCTATGC
GACTGAGCAGCTG**GAAAA**ATAGGAAGAAGAGTCGCGTAGAGAGGTTCTAAGC
GACCGAGCAGCAG**GAAAA**AAAGAAGAAGAGTAGCGTAAAGAGGTTCTATGC
GACCGAGCAGCAG**GAAAA**AAAGGAAGAAGAGTAGCGTAGAGAGGTTCTAAGCGACCGAGCAG
> CAAGTAGAAG Con:1 reps=4 [3094934,3095152] l-int=219 l-seq = 52
CAAGTAGAAGCTCTCTAGCTGCTGTCCAAAGTCAAAAATCCAATTTAGAGAT
CAAGTAGAAGCCCTCTAGTTGCTGTCTAAAGCCAAAAATCCAATTTAGAGAT
CAATTAGAAGTGCCCTAGCTGCTGTCTAAAGTCAAAAATCCAATTTAGAGGT
CAAGTAGAAGCCCTCTAGCTGCTGTCTAAAGTCAAAAATTCGATTTAGAGATCAAGTAGAAG
> GTCGCCTATA Con:1 reps=8 [3109160,3109638] l-int=479 l-seq = 52
GTCGCCTATAAGGTGTATACGCGACCGAACGGAA**GAAAA**AGAATCCCCATCG
GTCGCCTATAAGGTGTATACGCGACCGAACGGGAGCCAAAAAGAGTCCCTCG
GTCGCCTATAAGGCGTATACGCGACCGAACGGGAGCCAAAAAGAGTCCCTCG
GTCGCCTATAAGGCGTATACGCCACCGAACGGAAGCCAAAAGGAGCCTTCCG
GTCGCCTATAAGGCGTATACGTGACCGAACGGGAGCCAAAAAGAGCCCTCCG
GTCGCATATAACTCGTATACGCGACCGAGCGGCA**GAAAA**AAAGCGCTCCCCG
GTCGCATATAAGGTGTATACGCGACCGAACGGGAGCCAAAAAGAGCCCTCCG
GTCGCCTATAAGGCGTATACGCGACCGAGCGGCA**GAAAA**AAAGCGCTCCCCG
GTCGCCTATAAGGCGTATACGTGACCGAACGGCA**GAAAA**AAAGAGAGCCAAAGTCGCCTATA
> CTATAGGCGA Con:1 reps=9 [3142771,3143197] l-int=427 l-seq = 52
CTATAGGCGACCGAGGAGCGA**GAAAA**AAAGTGAGCAGGGTAGCGTATAAGGG
CTATAGGCGACCGAGGGGCGA**GAAAA**AAAGTGAGGAGGGTAGCGTATAAGGA
CTATAGGCGACCGAGAGGGGA**GAAAA**GAAGTGAGGAGGGTAGCGTATGAGGG
CTATAGGCGACCGAGGGGCGA**GAAAA**AAAGTGAGGAGGGTAGCGTATAAGGA
CTATAGGCGACCGAGAGGGGA**GAAAA**GAAGTGAGGAGGGTAGCGTATGAGGG
CTATAGGCGACCGAGAGGGGA**GAAAA**AAAGTGAGGAGGGTAGCGTATAAGGG
CTATAGGCGACCGAGAGGCGA**GAAAA**AGAGCCAGCAGGGTAGCGTATAAGGA
CTATAGGCGACCGAGAGGCGA**GAAAA**AGAGCCAGCAGAGTCGCGTATAAGGACTATAGGCGA
> AAATGGTCTT Con:1 reps=9 [3151872,3152297] l-int=426 l-seq = 52
AAATGGTC**TTCAT**AAGCGGA**ATGAA**GGCCAAAAAAGGTTGTGGCTCGAGTGA
AAATGGTC**TTCAT**AAGCGGA**ATGAA**GGCCAAAATCGGTTGTGGCTGGAGAGA
AAATGGTC**TTCAT**AGGCTGA**ATGAA**GGCCAAAATTAGTTGTGGCTGGAGAGA
AAATGGTC**TTCAT**AAGTGGA**ATGAA**GGCCAAAAAAGGCTGTGACTCGAGTGA
AAATGGTC**TTCAT**AAGCGGA**ATGAA**GACCAAAAAAGGCTGTGGCACGAGTGG
AAATGGTC**TTCAT**AAGCGGA**ATGAA**GACCAAAAAAGGCTGTGGCACGAGTGG
AAATGGTC**TTCAT**AAGCGGA**ATGAA**GACCAAAAACGGTTGTGGCTGGGGAGA
AAATGGTCTTCACAAGCGGA**ATGAA**GACCAAAAAAGGCTGTGACACGAGAGAAATGGTCTT
> TAAGAAAGGC Con:1 reps=9 [3169529,3169955] l-int=427 l-seq = 52
TAAGAAAGGCTCTTAAGGACAAAACGCTAGTAAAAATCAGTAGAAGTGTCTT
TAAGAAAGGCCCTCAAGGACAAAACGCTACTAAAAATTAATAGAAGTGTCCT

TAAGAAAGGCTCTTAAGGACAAAACGCTGATGGAAATCAGTAAAAGTGTCTA
TAAGAAAGGCTCTCAAGGACAAAACGCTAGTAAAAATTAATAGAACTGTCTT
TAAGAAAGGCTCTTAAGGACAAAACGCTAGTTCGAATTAATAAAACTGTCTT
TAAGAAAGGCTCTCAAGGACAAAACGCTAGT**GAAAA**TTAGTAGAACTGTCTT
TAAGAAAGGCTCTCAAGGACAAAACGCTAGTAAAAATCAATAGGAATGTCCT
TAAGAAAGGCTCTTAAGGACAAAACGCTGGT**GAAAA**TTAATAGAAATGTCCTTAAGAAAGGC
> GCGACAAAAG Con:1 reps=5 [3352830,3353048] l-int=219 l-seq = 52
GCGACAAAAGCCGGAGCAAAAGAGAGTAAAGTGAGTCCA**ATGAA**GAATATTG
GCGACAAAAGCCGAAGCAAAAAAGAGTAAAGTGAGTCCA**ATGAA**GGCTATTG
GCGACAAAAGTCGGAGCAAAGGAGAGCAAAGTGAATCCA**ATGAA**GGCTATTG
GCGACAAAAGCCGGAGCAAAGGAGAGGAAAGTGAATCCA**ATGAA**GGCTATTGGCGACAAAAG
> GTAACGTAGA Con:1 reps=8 [3498555,3498933] l-int=379 l-seq = 52
GTAACGTAGAGCTCGTCTACGTGCCCGCCCGAAGGATT**TTTTC**GCTGTTAGG
GTAACGTAGAGCTCGTCTACGTGCCCGACCGGAGTACT**TTTTC**GCTGTTAGG
GTAACGTAGAGGCTGTCTACGTGCCCGAATGGAGTATTTTTACGCTGTAAAG
GTAACGTAGAGCTCGTCCACTCCACGTGCCCGCCCGGAGGTT**TTTTC**CGCAATAAGT
GTAACGTAGAGCTTGTCTACGTGCCCGAATGGAGTATT**TTTTC**GCTGTAAAG
GTAACGTAGAGCTCGTCTACATGCCCGCCCGGAGGATT**TTTTC**GCAATAAGT
GTAACGTAGAGCCTGTCTACTTGCCCGA**ATGAA**GGTTTTTACGCTATAAGGTAACGTAGA
> GGTAACGTAG Con:1 reps=9 [3505767,3506245] l-int=479 l-seq = 52
GGTAACGTAGAGCCCGTCTACATGCACGAAGAGATAGTATTTTAGCAGTTTG
GGTAACGTAGAGCCTGTCTACGTGCCCGAAGAGATGGAAATTTAGCAGTTTG
GGTCATGTAGAGCCCGTCTACGTGCCTGAAGAGATG**GAAAA**TTAGCAGTTTG
GGTAACGTAGAACCCCTCTACATGCCCGAAGAGATGGTATTTTAGTAGTTTG
GGTAACGTAGAGCCCGTCTACGTGCCCGAAGAGATGGAAATTTAGCAGTTTA
GGTAACGTAGAGCCCGTCTACGTGCCGGAAGAGATGGAAATATAGCAGTTAG
GGTAACGTAGAGCCCGTCTACGTGCCCGAAGAGATGGAAATATAGCAGTTTA
GGTAACGTAGAGCCCGTCTACGTGCCCGAAGAGATGGAAATAGAGCAGTTAG
GGTAACGTAGAACCCGTCTACGTGCCCGAAGAGATAGAAGTTTAGCAGTTAGGGTAACGTAG
> TCCATTGCCC Con:1 reps=8 [3506345,3506718] l-int=374 l-seq = 52
TCCATTGCCCAAAACGGGCTAAAACATCAAATTATGGTCAATGGAGCCCCAC
TCCATTGCCCAAAACCGGCTAAATTATCAAATTATGGTCAATGCATCCCCGT
TCCATTGCCCAAAACCGGGTAAATCAACAAATTATGGTCAATGCATCCCCGT
TCCATTGCCCAAAACCGGCTAAATTATCAAATTATGGTCAATGCATCCCGT
TCCATTGCCCAAAACCGGGTAAATCAACAAATTATGGTCAATGCATCCCCGT
TCCATTGCCCAAAACCGGTTAAATTATCAAATTATGGTCAATGGAGCCCCAT
TCCATTGCCCAAAACCGGCTAAATTATCAAATTATGGTCAATGGAGCCCCATTCCATTGCCC
> AGCCTATTGG Con:1 reps=9 [3520892,3521318] l-int=427 l-seq = 52
AGCCTATTGGCGACATTTCTCTTAGTAAAAACAACAAAAGTGTTTCCAATAG
AGCCTATTGGCGACATTTCTCTTAGCAAAAATAACAAAAGTGTTTCCAATAG
AGCCTATTGGCGACATTTCCCTTAGCAAAAATAACAAAAGTGTTTCCAATAG
AGCCTATTGGCGACATTTCCCTTAGCAAAAATAACAAAAGTGTTTCCAATAG
AGCCTATTGGCGACATTTCTCTTTGCAAGAACTACTAAAGTGTTTCCAATAA
AGCCTATTGGCGACATTTCTCTTTGCAAGAACTACTAAAGTGTTTCCAATAA
AGCCTATTGGCGACATTTCTCTTTGCAAGAACTACTAAAGTGTTTCCAAAAG
AGCCTATTGGCGACATTTCTCTTTGCAAGAACTACTAAAGTGTTTCCAATAAAGCCTATTGG
> TGTCTGAATG Con:1 reps=8 [3527584,3527956] l-int=373 l-seq = 52
TGTCTGAATGATTTTTTTGCAGCCAGAGATCAGTTAGAATCCGTCTAGTTGC
TGTCTGAATGATTTTTTTGCAGCCAGAGATTAGTTAGAATCCGTCTAGTTGC
TGTCTGAATGATTATTTTGCAGCCAGAGATCAGTTAGAATCCGTCTAGTTAT
TGTCTGAATGATTATTTTGCAATCAGAGATCAGTTAGAAGCAGTCTAGTTGC
TGTCTGAATGATTATTTTGCAGCCAGAGATCAGTTAGAAGCAGTCTAGTTGC
TGTCTGAATGATTATTTTGCAGTCAGAGATCAGGTAGAAGCCGTCTGTTAC
TGTCTGAATGTTTTTTTTGCGGATTGAGATCAGTTAGAAACCGTTTAGTTGCTGTCTGAATG
> GTTGTAATTT Con:1 reps=8 [3535170,3535544] l-int=375 l-seq = 52
GTTGTAA**TTTTC**TAGAGTATTGTCACCAATAGCCGCCATTGGAAACACTTTG
GTTGTAA**TTTTC**TAGAGTTGTGTCGCCAATAGCCGTCATTGGAAACACTTTG
GTTGTAA**TTTTC**TAGAGTATTGTCGGCAATAGCCGCCGTTGGAAACACATTA
GTTGTAA**TTTTC**TAGAGTATTGTCGGCAATAGCCGCCATTAGAAACACTTTG
GTTGTAA**TTTTC**TAGAGTTGTGTCGCCAATAGCCGTCATTGGAAACACATTG
GTTGTAA**TTTTC**TAGAGTATTGTCGGCAATAGCCGCCGTTGGAAACACATTG
GTTGTAA**TTTTC**TAGAGTATTGTCGGCAATAGCCGCCATTAGAAACACTTTGGTTGTAATTT
> TGTCGCCAAT Con:1 reps=4 [3537636,3537802] l-int=167 l-seq = 52
TGTCGCCAATAGCGCCCATTGGAAACACTTAAGTTGTTTATTCTAAGAATTT
TGTCGCCAATAGCGGTTATTGGAAACACTTTGATTGTATTTTTTAAGAGTTA

TGTCGCCAATAGCCGCCATTGGAAACACTTAAGTTGTTTATTCCAAGAGTTATGTCGCCAAT
> ATAGAAATGT Con:1 reps=7 [3568862,3569184] l-int=323 l-seq = 52
ATAGAAATGTCGCCAATAGCTGTTATTGGAAACACTTATGTTGGTTTTTTGA
ATAGAAATGTCACCAATAGCTGCCATTGGAAACACTTATGTTGACTTTTTGG
ATAGAAATGTCGCCAATAGCTGCGATTGGAAACACTTATGTTGAC**TTTTC**GA
ATAGAAATGTCGCCAATAGCTGCGATTGGAAACACTTATGTTGAC**TTTTC**GA
ATAGAAATGTCGCCAAAAGCCGCCATTGGAAACACTCATGTTGGTTTTTTGA
ATAGAAATGTCGCCAAAAGCCACCATTGGGAACACTTATGTTAGTTTTTTGAATAGAAATGT
> **ATGAA**CCTTT Con:1 reps=10 [3645171,3645649] l-int=479 l-seq = 52
ATGAACCT**TTCAT**AGAGCGCATCGGAGTAAAAAAATCGAGCCCCCACGTACT
ATGAACCT**TTCAT**AGAGCGCGTCGGAGTAAAAAAATCGAGCTCCCGCGTACT
ATGAACCT**TTCAT**AGGGCGCGTCGGAGTAAAAAAATCGAGCCCCCACGTACT
ATGAACCT**TTCAT**AGAGCGCGTCGGAGTAAAAAAATCGAGCTCCCGCGTACC
ATGAACCT**TTCAT**AGAGCGCGTCGGAGTAAAAAAATCGAGCTCCCGCGTACT
ATGAACCT**TTCAT**AGAGCGTATCGGAGTAAAAAAATCGAGCTCCCGCGTACT
ATGAACCT**TTCAT**AGAGCGCGTCGGAGTAAAAAAATCGAGCTCCCGCGTACT
ATGAACCT**TTCAT**AGAGCGCGTCGGAGTAAAAAAATCGAGCTCCCGCGTACT
ATGAACCT**TTCAT**AGAGCGCGTAGGAGTAAAAAAATCGAGCTCCCGCGTACT**ATGAA**CCTTT
> ATGAGGACAA Con:1 reps=10 [3657440,3657918] l-int=479 l-seq = 52
ATGAGGACAAGTTCGGAGGAAACCCCCAGGAATCTGTCCTC**ATGAA**AGGTTC
ATGAGGACAAAATCGGAGGAAACCCCCAGGAATCTGTCCTC**AT****GAAAA**GCTT
ATGAGGACAAAGTCGGAG**GAAAA**CCCCAGGAATCTGTTCTC**ATGAA**AGGCTC
ATGAGGACAAAATCGGAGGAAACCCCCAGGAATCTGTCCTC**ATGAA**AGGGTC
ATGAGGACAAGTTCGGGG**GAAAA**GCCCAGGAATCTGTTCTC**ATGAA**AGGGTC
ATGAGGACAAAATCGGAG**GAAAA**CCCCAGGAATCTGTCCTC**ATGAA**AGGCTC
ATGAGGACAAGTTCGGAGGAAACCCCCAGGAATCTGTCCTC**ATGAA**AGGGTC
ATGAGGACAAAATCGGAGGAAACCCCGAGGAATCTGTCCTC**AT****GAAAA**GCTT
ATGAGGACAAAATCGGAGGAAACCCCGAGGAATCTGTCCTC**AT****GAAAA**GCTTATGAGGACAA
> ATAGAGCGCG Con:1 reps=5 [3673042,3673468] l-int=427 l-seq = 52
ATAGAGCGCGTGTCCTGTAATTTTATTG**GAAAA**GGCGAACTAAGAACCATTC
ATAGAGCGCGAAAGGAGGCCTAATTTAAGAATCTGCGTCCTATGAGGGTCTT
……………. . .
ATAGAGCGCGAAAGCAAGCTAATTTAAAGCAATCGCGTCTT**ATGAA**GGTCTT
ATAGAGCGCGTGTCCAGTGATTTATTCG**GAAAA**GGCGTCCT**ATGAA**CCATTC
ATAGAGCGGGAAAGGCTGCCTAATTTAAGAATCCGCGTCCTATGAGGGGCTT
ATAGTACGCGTAGCCTGGGATTTATTCGGAGAAGGCGAACTAAGAACCATTTATAGAGCGCG
> AAACGAGGTG Con:1 reps=5 [3674698,3674916] l-int=219 l-seq = 52
AAACGAGGTGATATATCAAAATTTGGTCAATGGAAGAGGGGGGCACTGCCCA
AAACGAGGTGA**TTCAT**CAAAATTTGGTTAATGGAAGAGGGGTGCATTGCCCA
AAACGAGGTGA**TTCAT**CAAAATTTGGTTAATGGAAGAGGGTTGCATTGCCCA
AAACGAGGTGATATATCAAAATTCGGTCAATGGAAGTAGGTTGCATTGCCCAAAACGAGGTG
> G**TTCAT**AGAG Con:1 reps=11 [3709317,3709951] l-int=635 l-seq = 52
G**TTCAT**AGAGCGCGAACATAAGGGAAATTTCCAAGAACGCGCACTATAGAAG
G**TTCAT**AGAGCGCGGACTGAAGGGGAAATGTCAAAAACGCGTACTATAAAGA
G**TTCAT**AGAGCGCGAACCTGAGGGAGATTTCCAAAAACGCGCACTATAGAAG
GTTCAAAGAGCGCGAACCTGAGGGGAAACTCCAAAAACGCGCACTATAAAGA
G**TTCAT**AGAGCGCGAACTTGAGA**GAAAA**CTCTAAAAACGCGCACTAAAGAAG
G**TTCAT**AGAGCGCGAACCTGAGGGGAATTTCCAAGAACGCGCACTAAAGAAG
G**TTCAT**AGAGCGCGGACTGAAGGGGAAATGTCAAAAACGCGCACTATAGAGG
G**TTCAT**AGAGCGCGGACTGAAGGGGAAATGCCAAAAACGCGTACTATAAAGA
G**TTCAT**AGAGCGCGGACTGAAGGGGAAATGCCAAAAACGCGTACTATAAAGA
G**TTCAT**AGAGCGTGGACTGAAGGGGAAATGCCAAAAACGCGTACTATAAAGA
G**TTCAT**AGAGCGCGAACCTGAGGGAGATTTCCAAAAACGCGCACTATAGAAG
GTTCAAAGAGCGCGAACATGAGGGGAAACTCCAAAAACGAGCACTATAAAGAG**TTCAT**AGAG
> GGTAACGTAG Con:1 reps=9 [3718417,3718842] l-int=426 l-seq = 52
GGTAACGTAGAGGGTGTCTAGGTGCCCGAACGGCC**GAAAA**AAGAAGAGGAG
GGTAACGTAGAGGGTGTCTAGGTGCCCGAACGGCCGAA**GAAAA**GAAGAGGAG
GGTAACGTAGAAGCAGTCTACGTGCCCGAACGGTC**GAAAA**AAAGAAGAGGAG
GGTAACGTAGAGGGTGTCTACGTGCCCGAACGGCC**GAAAA**AAAGAAGAGGAG
GGTAACGTAGAGGGTGTCTAGGTGCCCGAACGGCCGAA**GAAAA**GATGAGCAG
GGTAACGTAGAAGCAGTCTACGTGTCCGAACGGTC**GAAAA**AAAGAAGAGGAG
GGTAACGTAGAAGCAGTCTACGTGCCCGAACGGTCGAA**GAAAA**GAAGAGGAG
GGTAACGTAGAGGGTGTCTAGGTGTCCGAACGGTCGAA**GAAAA**GATGAGTTAGGTAACGTAG
> ATTGGAAACA Con:1 reps=4 [3723004,3723222] l-int=219 l-seq = 52
ATTGGAAACACTTTTGAGAATTTTGTTAGGGGAATTGTCGGCAATAAGCTCT

TTTGGAAACACTTTAGTTGTTTTAGCTAAGGGAAATGTCGCCAATAGGCTGT
ATTGGAAACACTTTAGTTGTTTTAGCTAAGGGAAATGTCAGCAATAAGCGGT
ATTGGAAACAGTTTAGTAGTTTTTGTTAAGGGAAATATCGCCAATAGGCATTATTGGAAACA
> GCGGTAAAAA Con:1 reps=12 [3747415,3748272] l-int=858 l-seq = 53
GCGGTAAAA**ATGAA**GGGTTGATTTTGCCCAAGGAGCTGCGAGGAAGGGGGCTA
GCGGTAAAAATAAAGGGTTGATTCTGCCTGAGAGGCTGAGAGGAAGGGGGGTG
GCGGTAAAA**ATGAA**GGGTTGATTTTGCCCGAGAAGCTGAGAGGAAGGGGGCTA
TCGGTAAAAATAAAGGGTTGATTCTGCCCGTGAAGCTGAGAGGAAGGAGGCTG
GCGGTAAAAATAAAGGGTTGATTCTGCCCGAGGAGCTGCGAGGAAGGGGGGG
AGCGTAAAAATAAAGGGTTGATTCTGCCCGAGGAGCTGCTTGAAGGAGGCTG
GCGGTAAA**ATGAA**GGGCTGATTTTGCCCGAGGAGCTGCGAGGAAGGGTGTTG
GCGGTAAAA**ATGAA**GGGCTAATTTTGCCCGAGGAGCTGCTTGGTAGGGTGCTG
GCGGTAAAAA**ATGAAAA**GGTGATTTTGCCCGTGAAGCTGCTTGGCCGGGTGCTG
GCGGTAAAA**ATGAA**GGGTTGATTCTGCCCGTGAAGCTGAGAGGAAGGGGGCTA
TCGGTAAAAATAAAGGGTTGATTTTGCCCGAGAAGGTGTCGGTAAGGGGGCTC
GCGGTAAAAAGAACAGAGCTCTATTGCCC**GAAAA**GGTACCCAAGCAGTCCTCA
ACGGTAAAAAGAACCGGTATCTATTGCCCGAAGAGGCACCCAAGCAGTCCCCT
GCGGTAAAAAAGAACCGGTATCTATTGCCCGAAGAGGCACCCAAGCAGTCCCCC
GCGGTAAAAAGAACAGAGCTCTATTGCCCGAAGAGGCACCCAAGCAGTCCCCC
GCGGTAAAAAGAACTGAGTTCTATTGCCCGATGAGGTACCCAAGCAGTCTCCAGCGGTAAAAA
> TGTCTTTAAT Con:1 reps=8 [3763475,3763900] l-int=426 l-seq = 52
TGTCTTTAATAAGCCCTATTGAGGACAAACCCGGCCTAGAAACCT**ATGAAAA**
TGTCTTTAATAAGGCTTCTAGAAGACAAACCCGGCCTAGAAACCT**ATGAAAA**
TGTCTTTAATAAGGCTTCTAGAAGACAAAACCCGCCTAGGAACTT**ATGAAAA**
TGTCTTTAATAAGGCTTCTAGAAGACAAAACCCGCCTAGGAACTT**ATGAA**AC
TGTCTTTAATAAGGCTTCTAGAAGACAAAACCGGCCGAGAAACTT**ATGAA**C
TGTCTTTAATAAGACCTATTGAGGACAAACCCGGCCTAGGAACTT**ATGAAAA**
TGTCTTTAATAAGCTCTCTTAAGACCAAAACCAGACCAGAAACCACATAAATT
TGTCTTTAACAAAACAA**ATGAA**GGACCAAATCTAACCTCCCCCCTAAAAAATGTCTTTAAT
> GTCGCATAGA Con:1 reps=7 [3775783,3776105] l-int=323 l-seq = 52
GTCGCATAGAAGGGGTATACGAGCCCGGGCGGCG**GAAAA**AATGGAGGTGCCG
GTCGCATAGAAGGGGTATACGCGCCCGGGCGGCGGAAGAAATGGAGGTGCCG
GTCGCATAGAAGGGGTATAGGCGACCGGGCGGCGG**GAAAA**GTGGAGGAGCCG
GTCGCATAGAAGGGTTATAGACGACCGAACGGCA**GAAAA**AATGGAGGTGCCA
GTCGCATAGAAGGGGTATACGCGCCCGAGCGGCGGAAAGAGAAGAGTTGTCG
GTCGCATAGAAAGGGTATACGCGCCCGAGCGGCG**GAAAA**AGAAGAAGCGTCGGTCGCATAGA
> ATGAGAACAG Con:1 reps=4 [3779055,3779221] l-int=167 l-seq = 52
ATGAGAACAGAGAGCTGTGGGAGCGTGCCGTCCTTGTTCTCATAAAGTGCCC
ATGAGAACA**GAAAA**CCAAGCAACTGCCCTAATCTTGTCCTCATAAAGCGTTC
ATGAGAACAGAAAGCTGA**GAAAA**AGTGCCGCCCTTGTCCTCATAAAGCGCCCATGAGAACAG
> TTGGAAACAC Con:1 reps=6 [3785428,3785698] l-int=271 l-seq = 52
TTGGAAACACTTTTGTTGCTTTTGCTAAGAGAAATGTCGCCAACAGGCCCCA
TTGGAAACACTTTTGTTGCTTTTGCTAAGAGAAATGTCGCCAACAGGCCCCA
TTGGAAACACTTTTGTTGCTTTTGCTAAGAGAAATGTCGCCAATAGACCCCG
TTGGAAACACTTTTGTTGCTTGTACTAAGAGAAATGTCGTCAATAGGCCCTA
TTGGAAACACTTAAGTTGTATTTTAAAAGAGAAATGTCGGCAATGGGTCCTATTGGAAACAC
> TTTCG**GAAAA** Con:1 reps=7 [3807647,3808021] l-int=375 l-seq = 52
TTTCG**GAAAA**GGCGTTTATTGAGGGGGTCATTAAGCACATGAGGCTGAGTTT
TTTCG**GAAAA**GGTG**TTCAT**AGAAGAGGTCA**ATGAA**CACATGAGGCTAAGTTT
TTTCG**GAAAA**AGTG**TTCAT**AGAAGAGGTCAATGAGCACATGGCTTTGAGTTT
TTTCG**GAAAA**AGTG**TTCAT**AGAAGAGGTCAATGAGCACATGAGGCTGAATTT
TTTCA**GAAAA**GGTG**TTCAT**AGAAGAGGCCAATGAGCAAATGTGGCTAAGTTT
TTTCG**GAAAA**GGTG**TTCAT**AGAAGAGGTCA**ATGAA**CACATGAGGCTGAGTTT
TTTCG**GAAAA**AGTG**TTCAT**AGAAGAGGTCAATGAGCACACGGCTCTGAGTT**TTTTC**G**GAAAA**
> TTGGAAACAC Con:1 reps=15 [3814968,3815757] l-int=790 l-seq = 52
TTGGAAACACTT**TTTTC**GTTTTTTTGAAGAGATTTGTTTCCAATAGGTGCGA
TTGGAAACACTTTACTCGTA**TTTTC**GCAGAGTTTTGTTTCCAATAGGCGTGA
TTGGAAACACTTTACTCGTA**TTTTCAT**TGAGATTTGTTTCCAATAGATGCGA
TTGGAAACACTTTACTAGTA**TTTTC**GCAGAGATTTGTTTCCAATAGGCGTGA
TTGGAAACACTTTACTCTAT**TTTTCAT**ATAGATTTGTTTCCAATAGATGCGA
TTGGAAACACTTTACTCGTA**TTTTC**ACAGAGTTTTGTTTCCAATAGGTGCGA
TTGGAAACACTTTACTCGTA**TTTTC**GTAGAGATTTGTTTCCAATAGTTGTGA
TTGGGAACACTTTACTCGTA**TTTTC**GCAGAGTTTTGTTTCCAATAGGCGTGA
TTGGAAACACTTTACTCTAT**TTTTCAT**ATAGATTTGTTTCCAATAGATGCGA
TTGGAAACACTTTACTCGTA**TTTTC**ACAGAGTTTTGTTTCCAATAGGTGCGA

TTGGAAACACTTTACTCGTA**TTTTC**GCAGAGTTTTG**TTTTC**AATAGGCGTGA
TTGGAAACACTTTACTCGTA**TTTTCAT**TGAGATTTGTTTCCAATAGATGCGA
TTGGAAACACTTTACTAGTA**TTTTC**GCAGAGATTTGTTTCCAATAGGTGTGA
TTGGAAACACTTTACTCTAT**TTTTCAT**AGAGATTTGTTTCTAATAGGTCCGA
TTGGAAACACTTTACTCGT**TTTTC**GCGGAGATTTGTTTCCAATAGGTAGGATTGGAAACAC
> TATTGCCCGA Con:1 reps=13 [3822748,3823390] l-int=643 l-seq = 53
TATTGCCCGATTGGGGTGTCCAAGGGGT**GAAAA**TGGTAAAAAGAAGTGGTTTC
TATTGCCCGAAATGGGTTCGGAAGAGGTCTAAGCGGTAAAAAGAACTCGCCTC
TATTGCCCGATTGGGTGTCCAAGGGGTGCAAATGGTAAAAA**GAAAA**GCTTTC
TATTGCCC**GAAAA**AGGTCCGGAAGAGGTCCTAACGGTAAAAAGAACAAGCTTC
TATTGCCCGATTGGGTGTCCAAGAGGTGCAAATGGTAAAAA**GAAAA**GGTTTC
TATTGCCCGAAATGGGTCCGGAAGAGGGCTAATGGTAAAAAGAACACGCTTC
TATTGCCCGATTGGTGTGTCCAAGGGGTGCAAATGGTAAAAA**GAAAA**GGTTTC
TATTGCCCGAAATGGGTCCGGAAGAGGGCTAATGGTAATAAGAACACGCTTC
TATTGCCCGATTGTGGTGTCCAAGGGGTGTAAATGGTAAAAAGAAGTGGTTTT
TATTGCCCGAGCAAGGTCTTGGAGGGGGGCAAACGGTAAAAA**GAAAA**GGTTTC
TATTGCCC**GAAAA**AGGTCCGGAAGAGGTCCAAACGGTAAAAAGAACACGCTTC
TATTGCCCGATTGGGTGTCCAAGGGGGTGCAAATGGTAAAAAGAACACGCTTCTATTGCCCGA
> TAGGCGACCG Con:1 reps=7 [3843104,3843426] l-int=323 l-seq = 52
TAGGCGACCGAGAGAGTGA**GAAAA**AGAGATTACGGGTAGCGTAGAAGGGTTA
TAGGCGACCGAGGGAGCG**GAAAA**AAGAGTTTACGGGTAGAGTAGAAGGGATA
TAGGCGACCGAGAGAGTG**GAAAA**AAGAGATTGCGGGTAGCGTAGAAGGGTTA
TAGGCGACCGAGAGAGCG**GAAAA**AAGAGATTGCGGGTAGCGTAGAAGGGTTA
TAGGCGACCGAGGGAGCG**GAAAA**AAGAGATTGCGGGTAGCGTAGAAGGGCTA
TAGGCGACCGAGAGAGCGA**GAAAA**AGGAGTCACGGGTAGCGTAGAAGGGCTTTAGGCGACCG
> CTATTGGAAA Con:1 reps=5 [3851718,3851935] l-int=218 l-seq = 52
CTATTGGAAACATTTTGTCCAATTTTAACTCAAGTCTTGTTGCCAATACCGG
CTATTGGAAACACTTTGCCAATCTTAACTCGAGTATTGTCGCCAATAGCGC
CTATTGGAAACACTTTGTCCAATTTTAACTCAAGTATTGTCGCCAATACCGG
CTATTGGAAACACTTTGTCCAATTTTAACTCGAGTATTGTCGCCAATAGCGCCTATTGGAAA
> ACTATGGGAG Con:1 reps=14 [3869085,3869771] l-int=687 l-seq = 52
ACTATGGGAGG**TTCAT**AGAGCGCGAAATTAG**GAAAA**AGGAAAGACTCCACGC
ACTATGGGAGG**TTCAT**AGAGCGCGAAATTGT**GAAAA**AGGAAAGACTCCGCGC
ACTATGGGAGGGTCATAGAGCGTGAAATTAG**GAAAA**A**GAAAA**GATTCCGCGT
ACTATGGGAGG**TTCAT**AGAGCGTGAAATTAG**GAAAA**A**GAAAA**GATTCCGCGT
ACTATGGGAGG**TTCAT**AGAGCGTGAAATTAG**GAAAA**A**GAAAA**GATTCCGCGT
ACTATGGGAGG**TTCAT**AGAGCGCGAAATTAG**GAAAA**AGGAAAGACTCCGCGC
ACTATGGGAGG**TTCAT**AGAGCGCGAAATTAG**GAAAA**AGGAAAGACTCCACGC
ACTATGGGAGG**TTCAT**AGAGCGCGAAATTGT**GAAAA**AGGAAAGACTCCCCGC
ACTATGGGAGG**TTCAT**AGAGCGCGAAATTAG**GAAAA**AGGAAAGACTCCGCGT
ACTATGGGAGG**TTCAT**CGAGCGAGAAATAAG**GAAAA**AGGAAAGACTCCGCGC
ACTATGGGAGG**TTCAT**CGAGCGAGAAATAAG**GAAAA**AGGAAAGACTCCGCGC
ACTATGGGAGG**TTCAT**AAAGCGTGAAATTAG**GAAAA**A**GAAAA**GATTCCGCGT
ACTATGGGAGG**TTCAT**AAAGCGTGAAATTAG**GAAAA**A**GAAAA**GATTCCGCGTACTATGGGAG
> CA**ATGAA**CAC Con:1 reps=5 [3869826,3870044] l-int=219 l-seq = 52
CA**ATGAA**CACTAAAACCTA**GAAAA**AGAGTAAAAAAGAGCGCATAGGGGGAGT
CA**ATGAA**CACGAAACTCTA**GAAAA**AGAGTAAAAAAGTGCACATAGAGGGTCT
CA**ATGAA**CACGTAGCCCCA**GAAAA**ATAACAAATACATGCGCATTGAAGGGTT
CA**ATGAA**CACGAAACTCCA**GAAAA**AAAGCAAGTTAGTGCCTATAGAAAGGTTCA**ATGAA**CAC
> TATTGCCCGA Con:1 reps=8 [3880946,3881427] l-int=482 l-seq = 52
TATTGCCCGAGGAGGAGCGAGAAAGGAGGTAAGCGGTAAGAATGAGGGGGTGA
TATTGCCCGAGGAGGTGCGGGAAGAGAAGTCAGCGGTAAGAATAAGATGTTGA
TATTGCCCGAGGAGACGCAGGAAGGGAGGTAAGCGGTAAGAATGAGAGGTTGA
TATTGCCCGAAGAGAA**GAAAA**AATTAATCCCGCGGTAAAAAGACCAGGCTTC
TATTGCCC**GAAAA**GAG**GAAAA**AGTAAGTCCCGCGGTAAAAAGAAAGTGTCTC
TATTGCCCAAAGAGAA**GAAAA**AATTAATCCCGCGGTAAAAAGACCGGGCTTC
TATTGCCCGAATAGAG**GAAAA**AGTAAGTCCCGCGGTAAAAAGAAAGTGTCTC
TATTGCCCGAAGAGAA**GAAAA**AATTAATCCCGCGGTAAAAAGACCGGGTTTC
TATTGCCCAAAGGCGC**GAAAA**AGTAAGTCCCGCGGTAAAAAGACCGGGCTTCTATTGCCCGA
> TGGAAACAAA Con:1 reps=11 [3938063,3939318] l-int=1256 l-seq = 52
…………….
TGGAAACAAAACTCACGAAGAAAGCGGCAGAAATGTTTCCAATGAGGGCTAT
TGGAAACAAAACTCACGAAGAAAGCGGGAGAAATGTTTCCA**ATGAA**GGCTAT
TGGAAACAAATCTCACGAAGAAAGCGGGAGAAATGTTTCCA**ATGAA**GGCTAT
TGGAAACAAAACTCACGAAGAAAGCGGCAGAAATG**TTTTC**AATGAGGGCTAT

TGGAAACAAAACTCACGAAGAAAGCGGCAGAAATG**TTTTC**A**ATGAA**GGCTAT
TGGAAACAAATCTCACGAAGAAAGCGGGAGAAATGTTTCCAATAAGGGCTAT
TGGAAACAAATCTCACGAAGAAAGCGGGAGAAATGTTTCCAATGAGGGCTAT
TGGAAACAAAACTCAC**GAAAA**AAGCGGGAGAAATGATTCCAATAAGGGCTAT
TGGAAACAAAACTCACGAAGAAAGCGGGAGAAATGATTCCAATGAGGGCTATTGGAAACAAA
> AGCGTTTATG Con:1 reps=12 [4057906,4058541] l-int=636 l-seq = 52
AGCGTTTATGAGAACGGCAAGTGAATGATTTTGGTTTGTCCAGTCCTCATGA
AGGGGTTATTGGAACAAAGAGCCGAAGATTTGAGTCCGTACTGTCCTCATGA
AGCGTTTATGAGAACAGAGAGCTAATGATTTGGGCCGAAACTGTCCTCATGA
AGCGTTTATGAGAACAGAGAGCTAATGATTTGAGTCAGAACTGTCCTCATGA
AGCGTTTATGAGAACAGAGAGCTAATGATTTGAGTCAGAACTGTCCTCATGA
AGCGTTTATGAGAACAGAGAGCGGAGGATTTGGGTCGGAACTGTCCTCATGA
AGCGTTTATGGGAACGGCAAGCGAAAGATTTGGGTCAAAAACTGTCCTCATGA
AGCGTTTATGAGAACAGAGAGCTAATGATTTGGGCCGAAACTGTCCTCATGA
AGCGTTTATGAGAACAGAGAGCTAATGATTTGGGCCGAAACTGTCCCCATGA
AGCGTTTATGAGAACAGAGAGCTAATGATTTGAGTCAGAACTGTCCTCATGA
AGCGTTTATGAGAACAGAGAGCTAATGATTTGAGTCAGAACTGTCCTCATGA
AGCGTTTATGAGAACAGAGAGCTAAAGATTTGGGTCGGAACTGTCCTC**ATGAA**GCGTTTATG
> AAGAAGTGTC Con:1 reps=6 [4170771,4171041] l-int=271 l-seq = 52
AAGAAGTGTCCATAGAGGTCCTCA**ATGAA**CACAAAATTGT**GAAAA**AAAGAGA
AAGAAGTGTCCATAGAGGTCCTCAGTGAACACGAAGTCGT**GAAAA**AAAGCGG
AAGAAGTGTCCATAGAGATCCTCA**ATGAA**CACGAAGCCGTGAAATAAAGCGG
AAGAAGTGTCCATAGAGGTCCTCA**ATGAA**CACGAAATCGT**GAAAA**AAAGAGA
AAGAAGTGTCCATAGAGGTATTCA**ATGAA**CACGAAATCGT**GAAAA**AAAGCGGAAGAAGTGTC
> AAAAAAGAGA Con:1 reps=16 [4191741,4192532] l-int=792 l-seq = 52
AAAAAAGAGAGTCCAGGGTCACATAAAAGCTTCTACGCGACCGGGCACATG
AAAAAAGAGAGCGCCGAGTAGCATAAAGAGCGTCTATGCGACCGAGCCAGAG
AAAAAAGAGAGCGCCGGGTAGAGTAGAGAGCGTCTATGCGACTTAGCCAGAG
AAAAAAGAGAGCGCCGAGTAGCATAAAGAGCATCTATGCGACCGAGCACGAG
AAAAAAGAGAGAGCCGGGTAGAATATAGAGAGCGTTTATGCGACCAAGCCCAAG
AAAAAAGAGAGCGCGTGGTAGAATAGAGAACGTCTATGCCACCGAGTCCAAG
AAAAAAGAGAGAGCCGGGTAGAATAGAGAACGTCTATGCGACCGAGTCCAAG
AAAAAAGAGAGCGCCGAGTAGCATAAAGAGCATCTATGCGACCGAGCCAGAG
AAAAAAGAGAGCGCGTGGTAGAATAGAGAGCGTCTATGCGACCAAGCCCAAG
AAAAAAGAGAGAGCCGAGTAGCATAAAGAGCATCTATGCGACCGAGCACGAG
AAAAAAGAGAGAGCCGAGTAGCATAAAGAGCATCTATGCGACCGAGCACGAG
AAAAAAGAGAGAGCCGGGTAGCATAAAGAGTATCTATGCGACCGAGTACGAG
AAAAAAGAGAGCGCCGGGTAGAGTAGAGAGCATCTATGCGACCGAGCACGAG
AAAAAAGAGAGCGCCGGGTAGAGTATAGAGCGTCTATGCGACCGAGCCAGAG
AAAAAAGAGAGAGCCGGGTAGAATAGAGAGCATCTATGCGACCGAGCCAGA**GAAAA**AAGAGA
> GCGACAAAAC Con:1 reps=7 [4300776,4301098] l-int=323 l-seq = 52
GCGACAAAACACTGTTG**GAAAA**GGGGCTAAGTGTTTCTA**ATGAA**GGCTATTA
GCGACAAAACTCGAG**GAAAA**ATAAGGCGAAGTGTATCCAATGAGGACTATTG
GCGACAAAACTCTGTTG**GAAAA**GGAGCGAAGTGTATCCA**ATGAA**GGCTGTTG
GCGACAAAACTCTGTTG**GAAAA**GGTGCGAAGTGTATCCA**ATGAA**GGCTATTG
GCGACAAAACTCTGTTG**GAAAA**GAGGCAAAGTGTTACCAATCGTGGCTATAG
GCGACAAAACTCGAGGAGCAAGAGAGTAAAGTGTATCCAATGAGGGCTATAGGCGACAAAAC
> TCA**ATGAA**CA Con:1 reps=6 [4719016,4719338] l-int=323 l-seq = 52
TCA**ATGAA**CATGATTTGGTAAAATTTCCGGTTGGAAGTG**TTCAT**AGAAAGGT
TCA**ATGAA**C**ATGAA**TCGGCAAAACTTTAGGTGGGAAGTG**TTCAT**AGAGAGGT
TCAATTAACACGAATCGTCAAAATTTTAGGTGGGAAGTG**TTCAT**AGAGAGGT
TCA**ATGAA**CACGAATCAGCAAAATTTCAGTCAAGAAGTGTTCGTAGAGAGGT
TCA**ATGAA**CACGAATCAGCAAAATTTCGGTCAAGAAGTGTTCGTAGAGAGGT
TCA**ATGAA**CACAAATTGGCAAAATTTAATTCAGGAAGTGTCCATAGAGGAAGTCA**ATGAA**CA
> TCTACGTGAC Con:1 reps=6 [4729327,4729597] l-int=271 l-seq = 52
TCTACGTGACCAAACAACCTCT**TTTTC**TTCCGTTACGGTCACGTAGAACCTC
TCTACGTGACCAAACAACGCCT**TTTTC**TTCGGCTACGGTCACGTAGAACCTC
TCTACGTGACCGAACAACCTCT**TTTTC**TTCCGTTACGGTCACGTAGAACCTC
TCTACGTGACCGAACAGCCCCT**TTTTC**TTCGGCTAGGGTCACGTAGAACCTC
TCTACGTGACCGGACAACCCCT**TTTTC**TTCGGCTAGGGTCGCGTAGAACCTGTCTACGTGAC
> ATTGCCCAAA Con:1 reps=15 [4951452,4952190] l-int=739 l-seq = 52
ATTGCCCAAAAATCGGTGATTAGATGGAAATTGGTCAATGGACGGGGCCTGC
ATTGCCCAAAAATCGGTGATTAGATGGAAATTGGTCAATGGCCGGGGCTTGC
ATTGCCCAAAAATCGGTGATTAGATGGAAATTGGTCAATGGCCGGGGGCTGC
ATTGCCCAAAAATCGCTGATTAGATGGAAATTAGTCAATGGATGGGGCTTGC

ATTGCCCAAAATCGGTGATTAGATGGAAATTGGTCAATGGCCGGGGCCTGC
ATTGCCCAAAAATCGGTGATTAGATGGAAATTGGTCAATGGCCGGGGCCTGC
ATTGCCCAAAAATCGGGTGATTAGATGGAAATTGGTCAATGGACGAGGCCTGC
ATTGCCCAAAAATCGGTGATTAGATGGAAATTAGTCAATGGCCGGGGCTTGC
ATTGCCCAAAAATCGGTGATTAGATGGAAATTGGTCAATGGACGGGGCTTGC
ATTGCCCAAAAATCGATGATTAGATGGAAATTAGTCAATGGCCGGGGCTTGC
ATTGCCCAAAAATCGGTGATTAGATGGAAATTAGTCAATGGATGGGGCTTGC
ATTGCCCAAAAATCGGTGATTAGATGGAAATTGGTCAATGGACCGGGCTTGC
ATTGCCCAAAAATCGGTGATTAGATGGAAATTAGTCAATGGATGGGGCCTGC
ATTGCCCAAAAGTCGGTGATTAGATGGAAATTGGTCAATGGACGAGGCTTGCATTGCCCAAA
> GTCGCATAGA Con:1 reps=8 [4975737,4976319] l-int=583 l-seq = 52
GTCGCATAGAAGGTGAATACGCGATTGAAGCGGC**GAAAA**AATCGTGAGCGCG
GTCGCGTAGAAGGTGTATACGCGACTGAAGAGCCG**GAAAA**ATTAGGAGCTGG
GTCGCATAGAAGGTGTATATACGACCGAAGCGGC**GAAAA**AATCGTTAGCTGG
GTCGTATAGAAGGTGTATACTCGACCGAAGAGCCG**GAAAA**ATTAGGAGCTCG
GTCGCATAGAAGGCGCATACGCAACCGAAGCGGC**GAAAA**ATCGGGAACTCG
GTCGCATAGAAGGCGCATACGCGACCGAAGCGGC**GAAAA**AATCGTGCCCCCG
GTCGCGTAGAAGGCGTATATACGACCGAAGCGGC**GAAAA**AATCGTGAGCTCG
GTCGCATAGAAGGCACATATGCTACCGAAGCGGCAAAAAAACCGTTCACTCA
GTCGAATAGAAGGCGTATACGCGACTGAAGCGGCAAAAAAACCGTGCAGCGG
GTCGCATAGAAGGCGCATACGCGACTGAAGCGGCAAAAAAACCGTGCCCGGG
GTCGCATAGAAGGTGAATACGCGACTGAAGCGGC**GAAAA**AAATCGTGCCCCATGTCGCATAGA
> A**GAAAA**AGAA Con:1 reps=5 [5059185,5059455] l-int=271 l-seq = 52
A**GAAAA**AGAAGAGAGGCCGGTCGAATAGAAAGAGTATATGCGACCGAGCGTC
A**GAAAA**AGAAGGTCCACCGGTCGCATAGAAAGAGTATATGCGACCGAGCGCC
A**GAAAA**AGAGGAGCCGTCGGTCGCATAGAAAGGGTATATGCGACTGAGCGGC
A**GAAAA**AGAAGAGAGGCCGGTCGAATAGAAAGGGCATATGCGACTGAGCGGC
A**GAAAA**AGAAGAGTAATTGGTCGCATAGAAAGAGTATACGCGACTGAGCGGTA**GAAAA**AGAA
> TCTACGTGCC Con:1 reps=6 [5285435,5285705] l-int=271 l-seq = 52
TCTACGTGCCCCAAGTTCAATAAATTAGTGAGTTTCGGTCACGTAGAGCCGT
TCTACGTGCCCCAAGTTCAATTAATTAGTGAGTTTCGGTCACGTAGACCTCT
TCTACGTGCCCCAAGTCCAATAAATTAGTGGGTTTCGGTCGCGTAGAGCCCA
TCTACGTGCCCCAAATCCAATAAATTAGTAGGTTTCGGTCGCGTAGAGCCCA
TCTACGTGCCCCAAGTTCAATAAATTAGTAGGTTTCGGTCGTGTAGAGATCTTCTACGTGCC
> TTGACCTTTT Con:1 reps=8 [5395730,5396209] l-int=480 l-seq = 52
TTGACC**TTTTC**TTT**GAAAA**ACAAATAGTTTAGGGCA**ATGAA**CATGGCTCCCA
TTGACC**TTTTC**TTTTAAAATCAATTAGTTTTAGGCAATGCACATGGCTTCCA
TTGACC**TTTTC**TTT**GAAAA**ACAAATAGTTTAGGGCAATGCGCGGGGCTCCCA
TTGACC**TTTTC**TTTAAATATCAATTAGTTTTAGGTAATGCACATGGCTTCCA
TTGACC**TTTTC**TTTCAAAAATCAACTAATTTTGGGCA**ATGAA**CAAAGCTTCCA
TTGACCGTTTCT**TTCAT**AATCAAATAGTTTTGGGCA**ATGAA**CAGGGCTTCCA
TTGACCGTTTCTTTAAATATCAATTAGTTTTAGGCAATGGACATGGTTCCCA
TTGACC**TTTTC**TTTAAATATCAATTAGTTTTAGGCAATGGACATGGCTTCCA
TTGACC**TTTTC**TATTAAAACTATCTAGTTTTGGGCAATGGACATGGCTTCCTTTGACCTTTT
> CTTCAATGTG Con:1 reps=5 [5398707,5398978] l-int=272 l-seq = 52
CTTCAATGTGCACTTCCTCGATGA**TTTTC**CTTGCTTCCGTG**TTCAT**TGGGGG
CTTCAATGTGCACTTCTTCGTGGATTTCTTTTGGCTCTCGTG**TTCAT**TGAGCC
CTTCAATGTGTACTTCTTCGATGA**TTTTC**CTTGCTTCCGTG**TTCAT**TGGGGG
CTTCAATGTGCACCTCTTTGTGGATTTCTCTTGCTCTCGAG**TTCAT**TGAATC
CTTCAATGCGCACTTATTCGGTAATTTCTCTTGGCCTCGTG**TTCAT**TGAACCCTTCAATGTG
> TTGACCAAAA Con:1 reps=11 [5450221,5450858] l-int=638 l-seq = 52
TTGACCAAAAAAGAGGATAATCCTCAAAAAGGTCAATGCGAGCAGGGTCCA
TTGACCAAAAGAGAGGAAACCCTTCAAAAAAGGTCAATGCAAGCAGGTTCCT
TTGACCAAAAGAGAGGAAACCCTTCAAAAAAGGTCAATGCAAGCAGGCTCCT
TTGACCAAAAGTGA**GAAAA**CCCTTCAAAAAAGGTCAATGCAAGCAGGCTCCA
TTTACCAAAAGAGAGGAAACCCTTCAAAAATGGTCAATGCAAGCAGGGTCCA
TTGACCAAAAGAGAGGAAACCCTTCAAAAAAGGTCAATGCAAGCAGGTTCCT
TTGACCAAAAGAGAGGAAACCCTTCAAAAAAGGTCAATGCAAGCAGGCTCCT
TTGACCAAAAGTGA**GAAAA**CCCTTCAA**GAAAA**GGTCAATGCAAGCAGGCTCCA
TTTACCAAAAGAGAGGAAACCCTTCAAAAAAGGTCAATGCAAGCAGGCTCCA
TTGACCAAAAGAGAGGAAACCCTTCAAAAAAGGTCAATGCAAGCAGGGTCCT
TTGACCAAAAGTGA**GAAAA**CCCTTCAAAAAAGGTTAATGCAAGCAGGGTCCA
TTGACCAAAAGTGAGGAAACCCAGTCAAAAATGGGTAATGCGAACAGCTTCTCCTTTGACCAAAA
> TGTCC**TTCAT** Con:1 reps=10 [5707911,5708389] l-int=479 l-seq = 52
TGTCC**TTCAT**AAGGTTG**ATGAA**GACCATTTTGG**ATGAAAA**GCCGAAGTGAAA

TGTCC**TTCAT**AAGGGTG**ATGAA**GACCATTTTGG**ATGAAAA**GCCAAAGAGAAA

TGTCC**TTCAT**AAGGTGT**ATGAA**GACCATTTTGG**ATGAAAA**GCCTAAGAGAAT

TGTCC**TTCAT**AAGGGTG**ATGAA**GACCATTTTGG**ATGAA**ATGCCGAAGAGAAA

TGTCC**TTCAT**AAGGGTG**ATGAA**GACCATTTTGGATAAAAAGCCAAAGAGAAA

TGTCC**TTCAT**AAGGTGT**ATGAA**GACCATTTTGG**ATGAAAA**GCCAAAGAGAAA

TGTCC**TTCAT**AAGGTGT**ATGAA**GACCATTTTGG**ATGAA**ATGCCGAAGAGAAA

TGTCC**TTCAT**AAAGGTG**ATGAA**GACCATTTTGG**ATGAAAA**GCCAAAGAGATA

TGTCC**TTCAT**AAGGTGT**ATGAA**GACCATTTTGAAC**GAAAA**GCCGAAGTGAAATGTCC**TTCAT**

***B.cellulosilyticus* (NC 014829). All tandem repeats l-seq=52**

Total 54 tandem repeats. Only 31 have one or more of the following signals or its reverse:
GGTCATCAT, CAATGCT, ACGAAGG, ATGACAC

> GTGTATCATA Con:1 reps=6 [18432,18858] l-int=427 l-seq = 52
GTGTATCATACGAGGCCAATGATACGCGAGAAGGTAGTGGGAACGAGATAAA
GTGTATCATACGAGACCAATGACACGCGAGAAAGAAGTGGGAATGAAATAAA
GTGTGTCATACGAAGGCAATGACGCATATAAAAGTGGGAGGAACGGAATAAG
GTGTATCATACGAGGCCAATGACACGCGAGAAAGTAGTAGGAACGAGATAAA
GTGTGTCATAGAAGTGCAATGACACGTGAGAATTTAGGTGGAAACGGATAAA
GTGTATCATACGAAGGCAATGACACATATAAAAGTGGGAGGAACGGAACAAA
GTGTATCATACGAGGCCAATGACACGCGAGAAAGTAGTGAGAATGAAATAAA
GTGTGTCATACGAGAGTAATGAAACATGAAATCTTAAGTAAAATAAAATAAGGTGTATCATA
> CACTCAACGA Con:1 reps=10 [18889,19522] l-int=634 l-seq = 52
CACTCAACGAAGGTCATCATAATCAAGCAATGCTACCCCAAAACCAACCCCG
CACTCAACGAAGGTAATCATAATCAAGCAATGCTACCCCAAAACCAACCCCG
CACTCAACGAAGGTAATCATAGTAAGCCAATGCTACCCCAAAACCAACCCCG
CACTCAACGAAGGTAATCATAGTAAGCCAATGCTACCCCAAAAACCAAACCG
CACTCAACGAAGGTAATCATAGTAAGCCAATGCTACCCCAAAAACCAAACCG
CACTCAACGAAGGTAATCATAGTAAGCCAATGCTACCCCAAAAACAAAAACT
CACCTAACGAAGGTAATCATAGGCGGCCAATGCTGCCCCAAAAGCCAACTCA
CACTCAACGAAGGTAATCATAGTAAGCCAATGCTACCCCAAAAACCAAACCG
CACCCAACAAAGGTAATCATAAGCAAGCAATACTACCCCAAAACTAAACCAC
CACTCAACGAAGGTAATCATAGTAAGCCAATGCCACCCCAAAACCAAACCCG
CACTCAACAAAGGTCACCAAAAGCAAGCAATGCTGCCCCAAAACCAAACCAG
CACTCAACGAAGGTCATCATACCCAAGCATGCCGCCCCCAAAACCAATCCTCACTCAACGA
> AATAAAGTGT Con:1 reps=7 [43977,44455] l-int=479 l-seq = 52
AATAAAGTGTTTTATACAAGTGTAATGACGCAAGAAAAAGTGGGAGGAACGG
AATAAGGTGTATCATACGAGACCAATGACACGCGAGAAGGTAGTGGGAACGA
GATAAAGTGTGTCATAGAAGTGCAATGACACGTGAAAAAGTATGTGGAAACG
AATAAAGTGTATCATACGAAGGCAATGACGCATAAGAAAGTGGGAGGAACGG
AATAAGGTGTATCATACGAGGCCAATGACACGCGAGAAGGTAGTGGGAACGA
AATAAAGTGTGTCATAGAAGTGCAATGACACGTGAAAAAGTATGTGGAAACG
AATAAAGTGTATCATACGAAGGCAATGATGCATAAGAAAGTGGGAGGAACGA
AATAAAGTGTGTCATATGAGGCAAATGACACGCGAGAAAGAAGTGGGAACGG
AATAAAGTGTATCATACGAAGCCAATGACACGCGAGAAAGTAGTGGGAATGAAATAAAGTGT
> CAATGCTACC Con:1 reps=5 [44519,44789] l-int=271 l-seq = 52
CAATGCTACCCTAAAAGCTAATCCTCACTCATCGAAGGTAATCATACGCAAG
CAATGCTACCCCAAAAGCTAATCCTCACTCAACGATGGTCGCCATACGCAAG
CAATGCTACCCTAAAAGCTAATCCTCACTCATCGAAGGTAATCATAGGCAAG
CAATGCTGCCCCAAAAACGAATCCCCACTCAACGAAGGTCATCATATGAAGC
CAATGCTACCCCAAAACTAAAACCGCTCCCAACGAAGGTCACCATATGAAGCCAATGCTACC
> AATAAAGTGT Con:1 reps=7 [112827,113253] l-int=427 l-seq = 52
AATAAAGTGTTTTATACAAGTGCAATGGCGCAATGAAAAGTGGAAGGAACGG
AATAAAGTGTATCATACGAGGCCAATGACACGCTAGAAAGAAGTGGGAATGA
AATAAAGTGTGTCATAGAAGTGCAATGACACGTGAGAAAGTATGAGGAAAGG
AATAAGGTGTATCATACGAAGGCAATGATGCATAAGAAAGTGGGAGGAACGG
AATAAAGTGTGTCATACGAAGGCAATGATACACGAGAAAGAAGTTGGACCGA
GATAAAGTGTGTCATAGAAGTGCAATGAAACGTGAGAAAGTATGTGGAAACG
AATAAAGTGTATCATACGAAGGCAATGACGCATATAAAAGTGGGAGGAACGG
AATAAAGTGTATCATACGAGGCCAATGACACGCGAGAAAGTAGTGGGAATGAAATAAAGTGT
> AAGGTCATCA Con:1 reps=7 [113351,113778] l-int=428 l-seq = 52
AAGGTCATCATAGGACGCCAATGCTACCCCAAAACCAAATCCCCACTGAACG
AAGGTCATCATAGGAAGTCAATGCTGCCCCAAAAGCAAAATCGCATCCAACA
AAGGTCATCATAAGCAAGCAATGCTCCCCCAAAACCAAACTCACACTCAACA
AAGGTCACCATAAGCAAGCAATGCTACCCCAAAAGCAAAACCGCACCCAACA
AAGGTCATCATAAAGCAAGCAATGCTCCCCCAAACCCAACCCCGCACTCAACG
AAGGTCATCATAGGAAGTCAATGCTCCCCCAAAACCAACCCCACACTCAACG

AAGGTCATCATACGCAAGCAATACTACCCCAAAACTAAACCACCACTCAACG
AAGGTAATCATAAGCAAG**CAATGCT**GCCCCAAAAACAAAAGTTCACCTA**ACGAAGG**TCATCA
> ATAAGATGTG Con:1 reps=10 [275907,276436] l-int=530 l-seq = 52
ATAAGAT**GTGTCAT**AGAGGAGCAATGCCACGTGAAAAAGTCGAGTTGTCAAA
ATAAGAT**GTGTCAT**AGAGGAGCAATGCCACGCGAGAAAACCGAGTCGTCAAA
ATAAGAT**GTGTCAT**ACGAGTGCAATGCCACGCGAAAAAGTCGAGTTGTCAAA
ATAAGAT**GTGTCAT**ACGAGTGCA**ATGACAC**GCGAGAAAACCGAGTCACGAAG
ATAAGAT**GTGTCAT**ACGAGTGCAATGCCACGCGAAAAAGTCGAGTCGTCAAA
ATAAGAT**GTGTCAT**AGAGGAGCAATGCCACGCGAAAAAGTCGAATCGTCAAG
ATAAGAT**GTGTCAT**ACGAGTGCAATGCCACGCGAAAAAGTCGAGTCGTCAAA
ATAAGAT**GTGTCAT**AGAGGAGCAATGCCACGCGAAAAAGTCGAATCGTCAAA
ATAAGAT**GTGTCAT**ACGAGTGCAATGCCACCCGAAAAAGCTAATTAACCAAA
ATAAAGGTCATCATACGAAGCCAACACCACAAAAAAATTTCCAAGCTCAGCATAAGATGTG
> CTGAAGGTCA Con:1 reps=5 [299755,299973] l-int=219 l-seq = 52
CTGAAGGTCATCATACGAGCC**CAATGCT**TACCGAGAAAACAAGAAGAAGTTC
CTGAAGGTCATCATAGAAGCG**CAATGCT**TACCGAGAAAACGGGAAGAAGCAT
CTGAAGGTCATCATAGAAGCG**CAATGCT**TACCGAGAAAACAAGAAGAAGCAC
CTGAAGGTCATCATAGAAGTGCAATGCATACCGAGAAAACAAGAAGAAGCATCTGAAGGTCA
> CTGAGTGTCC Con:1 reps=4 [314448,314665] l-int=218 l-seq = 52
CTGAGTGTCCATTAAACAGGATTAAAAATCCGGTTAGAGCGCGAGTCATTGT
CTGAGTGTCCATTAAACAGGATTTAAAATCCGGTTAGAGCGAGGGTCATTGT
CTGAGTGTCCATTAAACAGGATTAAAATCCCGTTAGAACAAGAATCATAGT
CTGAAAGTCCAATAAACAAGGTATAAAATCCCATCAGAGCGAGAAACATGACCTGAGTGTCC
> CCCGTTAGAG Con:1 reps=5 [339003,339220] l-int=218 l-seq = 52
CCCGTTAGAGCAAGATTCAATAACTAAGTGTCGAATAAACAGGGTTTAAAAT
CCCGTTAGAGCGGGATTCAATAACTGAGTGTCGATTAAACAGGGTTTAAAAT
CCCGTTAGAGCAGGATCAATAACTAAGTGTCGATTAAACAGGGTTTAAAAG
CCCGTTAGAGCAGGATTCAATAACTAAGTGTCGATTAAACAGGGTTTAAAATCCCGTTAGAG
> TGTTGAACGG Con:1 reps=4 [465532,465698] l-int=167 l-seq = 52
TGTTGAACGGAAAGGAACTGCAGAAAGTATTCCGTTTAAGTGGAGAATGCGT
TGTTGAACGGAAAGGAACTGCAGAAAGTATTCCGATCAAGTGGAGAAGATGC
TGTTGAACGGAAAACAACAGAAGAAAGTATTCCGTTCAAGTGGAGAAGATGCTGTTGAACGG
> ACTCTATTTC Con:1 reps=14 [494305,494992] l-int=688 l-seq = 52
ACTCTATTTCGG**ACAGAGTT**TCTTGTTTAATATTC**AACTCTGT**CCGATTGCT
ACTCTATTTCGG**ACAGAGTT**TCTTGTTTAATATTC**AACTCTGT**CTGATTGCA
ACTCTATTTCGG**ACAGAGTT**TCTTGTTTAATATTGACCTCTGTCCGATTGCT
ACTCTATTTCGG**ACAGAGTT**TCTAGTTCATTACTC**AACTCTGT**CCGATTGCA
ACTCTATTTCGG**ACAGAGTT**CCTTGTTTAATATTG**AACTCTGT**CTGATTGCT
ACTCTATTTCGGACAGAGATCCTTGTTTAATATTG**AACTCTGT**CTGATTGCA
ACTCTATTTCGG**ACAGAGTT**TTCTTGTTTAATATTC**AACTCTGT**CCGATTGCA
ACTCTATTTCGGACAGAGTCTCTCGTTTAATATTG**AACTCTGT**CTGATTGCA
ACTCTATTTCGGACAGAGTCTCTAGTTCATTACTC**AACTCTGT**CCGATTGCA
ACTCTATTTCGG**ACAGAGTT**CCTTGTTTAATATTC**AACTCTGT**CTGATTGCA
ACTCTATTTCGGACAGAGTCTCTTGTTTAATATTC**AACTCTGT**CTGATTGCA
ACTCTATTTCGGACAGAGTCTCTTGTTTAATATTC**AACTCTGT**CCGATTGCA
ACTCTATTTCGGACAGAGTCTCTTGTTTAATATTG**AACTCTGT**CCGATTGCAACTCTATTTC
> **GTGTCAT**AGC Con:1 reps=8 [650939,651417] l-int=479 l-seq = 52
GTGTCATAGCACTCGTATGACGCATTTTATATTGAATCTTTAGCTAAGTCGT
GTGTCATAGCGGATGTATGATACACCTTATTTTAAGATTCTATTTATTTGGC
GTGTCATAGCACTCGTATGACGCATTTTATATTGTATCTCTAGCGATGTTGT
GTGTCATAGCGGATGTATGATGCACCTTATTTTGAAATTCTATCTATTTCAC
GTTGCATAGTGCTCGTATGATACATCTTGAATGGAATATTTAGCAATGTTGT
GTGTCATAGCGGATGTATGATACACCTTATTTTAAGATTCTATTTATTTGGC
GTGTCATAGTACTCGTATGACGCATCTTATATTGAATCTTTAGCTAAGTCGT
GTGTCATAGCGGATGTATGATACACCATATTTTAAGATTCTATTTATTTGGC
GTGTCATAGCACTCGTATGACGCATCTTATATTGTATCTCTAGCGATGTTGT**GTGTCAT**AGC
> AAGCGTGTCA Con:1 reps=19 [726998,727944] l-int=947 l-seq = 52
AAGC**GTGTCATACGAAGG**CAATGCCACATGAAAAATCGGGAGGCAGGAAATG
AAGC**GTGTCATACGAAGG**CGATGCCGCACGAAAAATCGGGACGCAGGAAATA
AAGC**GTGTCATACGAAGG**CAATGCCGCATGAAAAATTGGGACGCAGGAAATA
AAGC**GTGTCATACGAAGG**CAATGCCGCATGAAAAATCGGGAGACAGGAAATG
AAGC**GTGTCATACGAAGG**CAATGCCGCATGAAAAATCGGGACGCAGGAAATA
AAGC**GTGTCATACGAAGG**CAATGCCGCACGAAAAATCGGGACGCAGGAAATA
AAGC**GTGTCAT**ACGAAGACAATGCCGCATGAAAAATCGGGAGACAGGAAATG
AAGC**GTGTCATACGAAGG**CAATGCCGCATGAAAAATCGGGAGACAGGAAATG

AAGC**GTGTCATACGAAGG**CAATGCCGCATGAAAAATCGGGAGACAGGAAATG
AAGC**GTGTCATACGAAGG**CAATGCCGCATGAAAAATCGGGACGCAGGAAATA
AAGC**GTGTCAT**ACAAAGGCAATGCCGCATGAAAAATCGGGACGCAGGAAATA
AAGC**GTGTCATACGAAGG**CAATGCCACATGAAAAATCAGAATCGAGTAAATG
AAGC**GTGTCATACGAAGG**CAATGCCGCATGAAAAATCGGGAGACAGGAAATG
AAGC**GTGTCATACGAAGG**CAATGCCGCATGAAAAATCGGGACGCAGGAAATA
AAGC**GTGTCATACGAAGG**CAATGATGCATGAAAAATCAGAATCGAGGAAATA
AAGC**GTGTCAT**ACAAAGGCAATGCCGCATGAAAAATCGGGACGCAGGAAATA
AAGC**GTGTCATACGAAGG**CAATGCCGCATGAAAAATCGGGACGCAGGAAATA
AAGC**GTGTCATACGAAGG**CAATGACGCATGAAAAATCGGGACACAGGAAATAAAGCGTGTCA
> TAGTCTGAGT Con:1 reps=8 [857866,858240] l-int=375 l-seq = 52
TAGTCTGAGTGGATATAAAACACGAAATAAAATCTCGTTGTGATGGTGAGTC
TAGTCTGAGTGGATATAAAACACGAAATAAAATCTCGTTGTGATGGTGAGTC
TAGTCTGAGTGGATATAAAACACGAAATAAAATCTCGTTGTGATGGTGAGTC
TAGTCTGAGTGGATATAAAACACGAAATAAAATCTCGTTGTGATGGTGAGTC
TAGTCTGAGTGGATATAAAACACGAAATAAAATCCCGTCGTGATGATGAGTC
TAGTCTGAGTGGATATAAAACACGAAATAAAATCTCGTCGTGAGAGTGAGCA
TAGTCTGAGTGGATATAAAACACGAAATAAAATCCCGTCGTGAGAGTGAGCATAGTCTGAGT
> **GCCATC**TGCC Con:1 reps=4 [949082,949248] l-int=167 l-seq = 52
GCCATCTGCCCGTGAGCAAGAAAAAAGAAGTGTGAAGGTAAGATGGGAAGCC
GCCATCTGCCCATGAGCAAACAAAAAGAAGTGTGAAGGTAAGATGGGAAGCT
GCCATCTGCCCATGAGCAAACAAAAAGAAGTGTGAAGGTAAGATGGGAAGCT**GCCATC**TGCC
> AAAGCAGAAA Con:1 reps=12 [1015273,1015855] l-int=583 l-seq = 52
AAAGCAGAAATAATTGTCTGAAGGTCATCACACGAAGT**CAATGCT**ACCCGAA
AAAGCAGAAAAAAATGTTCTGAAGGTCATCATACGAAGT**CAATGCT**ACCCGAA
AAAGCAGAAAAAAATGTTCTGAAGGTCATCATACGAAGT**CAATGCT**GCTCGAA
AAAGCAGAAAAAAATGTTCTGAAGGTCATCATACGAGGT**CAATGCT**GCTCGAA
AAAGCAGAAAAAAATGTTCTGAAGGTCATCATACGAGGT**CAATGCT**ACCCGAA
AAAGCAGAAAAAAATGTTCTGAAGGTCATCATACGAGGT**CAATGCT**ACCCGAA
AAAGCAGAAAAAAATGTTCTGAAGGTCATCATACGAGGT**CAATGCT**ACCTGAA
AAAGCAGAAAAAAATGTTCTGAAGGTCATCATACGAGGT**CAATGCT**ACCCGAA
AAAGCAGAAAAAAATGTTCTGAAGGTCATCATACGAGGT**CAATGCT**ACCCGAA
AAAGCAGAAAAAAATGTTCTGAAGGTCATCATACGAAGT**CAATGCT**ACCTGAA
AAAGCAGAAAAAAATGTTCTGAAGGTCATCATACGAGGTCAATACTACCCGAGAAAGCAGAAA
> GTAGCATAGC Con:1 reps=7 [1178020,1178446] l-int=427 l-seq = 52
GTAGCATAGCTTCTCTATGCTGACCGTGATTGTTTAATTGTTTCACTGACGA
GTAGCATAGCGCTTGTATGATAACCTTTAGAAATAGTGTGATCGATTTTCGG
GTAGCATAGCTTCTCTATGCTGACCGTGATTGTTTAATTGTTTCACTGACGG
TC**AGCATTG**CGCTTGTATGATTACCTTTAGAAATAGTGTGATCGTTTTTGGG
GTAGCATAGCTTCAGT**ATGATGACC**GTGATTGTTTAATTGTTTCACTGACGA
GTAGCATAGCGCTTGTATGATAACCTTTAGAAATAGTGTGATCGTTTTTCGG
GTAGCATAGCTTCTCTATGCTGACCGTGATTGTTTAATTGTTTCACTGACGG
TCAGCATAGCGCTTGTATGATTACCGTGATTGTTTAATTGTTTCACTGACGAGTAGCATAGC
> AT**GTGTCAT**T Con:1 reps=10 [1193617,1194303] l-int=687 l-seq = 52
AT**GTGTCAT**TGGCCTTGTATGAAACGGAATATTGGATTGCTTTTTATTTTAC
AT**GTGTCAT**TGATCTCGT**ATGACAC**GAGTTATTGGATTGCTTCTTATTTTTC
AT**GTGTCAT**TGGCCTTGT**ATGACAC**GGAATATTGGCTGGCTTTTTATTTTTC
AT**GTGTCAT**TCCCCATGT**ATGACAC**AAGTTATTGGATTGCTTTCTAATTTTT
AA**GTGTCAT**TGCTCTTGT**ATGACAC**ACGTTATTGGAATACTTTCTAATTTTC
AT**GTGTCAT**TGCCCTCGT**ATGACAC**ACGTTATTGGAATACTTTCTAATTTTC
AT**GTGTCAT**TCCCCATGT**ATGACAC**ACGTTATTGGATTGTTTTCTAATTTTC
AA**GTGTCAT**TGGTCTTGT**ATGACAC**ACGTTATTGGATTGTTTTCTAATTTTC
AA**GTGTCAT**TGGTCTTGT**ATGACAC**ACGTTATTGGAATACTTTCTAATTTTC
AT**GTGTCAT**TCCCCATGT**ATGACAC**ACGTTATTGGATTGCTTTCTAATTTTT
AT**GTGTCAT**TGCCCTCGT**ATGACAC**AAGTTATTGGATTGCTTTCTAATTTTC
ATGTGTCTTTGGTCTTGT**ATGACAC**GTGTTATTGGATTGCTTTTTATTTTTC
AT**GTGTCAT**TCCCCATGT**ATGACAC**GCGTTATTGGATTGCTTCTTATTTTTCAT**GTGTCAT**T
> CCCGTTAGAA Con:1 reps=6 [1710465,1710734] l-int=270 l-seq = 52
CCCGTTAGAAAGATTACCCCGTGCTGAAGGTAGATAAAACAGCGTTTAAAAT
CCCGTTAGAATGATTATCCCGTGCTGAAGGTAGATAAAACAGCGTTTAAAAT
CCCGTTAGAATGATTATCCCGTGCTGAAGGTAGATAAAACAGTGTTTAAAAG
CCCGTTAGAAGGAGTCTCTCGTGCTGAAGGTAGATAAAACAGTGTTTAAAAG
CCCGTTAGAAGTATTATCTCGTGCTGAAGGTAGATAAAACAAAGTTTAAAGCCCGTTAGAA
> AAGGTAATCA Con:1 reps=8 [2263203,2263629] l-int=427 l-seq = 52
AAGGTAATCATAAGCAAGCAACGCTACCCCAAAACCACCCCCGCACTCAACG

AAGGTAATCATAGGCAAGCAATGCTACCCCAAAACTAAACCGGCACCCCAACA
AAGGTAATCATAAGCAAGCAATGCTGCCCGAAAAGCCAAACCTCACTCAACG
AAGGTCATCATAGAGAAGCAATGCTACCCCAAAACTAAACCGGCACTTAACA
AAGGTAATCATAAGCAAGCAACGCTATCCCAAAACCACCCCCGCACTCAACG
AAGGTAATCATAAGCAAGCAATGCTACCCCAAAACTAAACCGGCACTTAACA
AAGGTAATCATAAGCAAGCAATGCTACCCCAAAACCACCCCCGCACTCAACG
AAGGTAATCATAAGCAAGCAATGCTACCCCCAAACTAAACCGGCACCCAACAAAGGTAATCA
> AAGGTCATCA Con:1 reps=11 [2323006,2323563] l-int=558 l-seq = 52
AAGGTCATCATACAAAGGCTATGCTACCCGACAAAACGAAGTTGAGCAGATG
AAGGTCATCATACGAAGCCTATGCTGCCTGAAAAATTGAAACCGAGCATGCTACCCGACAAAACGAAACCGAGCAGATG
AAGGTCATCATACGAAGTCTATGCTGCCTGAAAAATTGAAACCGAGCAGATA
AAGGTCATCATACGAAGTCTATGCTACCCGACAAAACGAAACCGAGTAGATG
AAGGTCATCATACGAAGTCTATGCTGCCTGAAAAATAGAAACCGAGCAGATA
AAGGTCATCATACGAAGTCTATGCTACCCGACAAAACGAAACCGAGTAGATG
AAGGTCATCATACGAAGTCTATGCTGCCTGAAAAATAGAAACCGAGCAGATA
AAGGTCATCATACGAAGTCTATGCTACCCGAAAAATAGAAATCGAGCAGATG
AAGGTCATCATACGAAGTCTATGCTACCCAAAAAAATGAAATCGAGCAGATG
AAGGTCATCATACGAAGCCTATGCTACCCGAAAAATAGAAACCGAGCAGATGAAGGTCATCA
> GTATGATGAC Con:1 reps=4 [2455362,2455527] l-int=166 l-seq = 52
GTATGATGACCTTTAGCAGCTCGGGTTTGTTTTGTCGGGTAGCATAGACTTT
GTATGATGACCTTCAGCAGCTCGGGTTTGTTTTGTCGGGTAGCATAGGCTCC
GTATGATGACCTTCAGCGGTTCGGGTTCTTTTTTCGGGTAGCATAGACTTTGTATGATGAC
> ACACTTTATT Con:1 reps=7 [2556156,2556529] l-int=374 l-seq = 52
ACACTTTATTCGTATCGACATACTTTCTCACGTGTCATTGCACTTCTATGAT
ACACTTTATTTGTTCCTACTACTTTCTCATGCGTCATTGCCTTCGTATGAC
ACACTTTATTCGTTTCCACATAAATCTTCACGTGTCATTGCACTTCTATGAC
ACACTTTATTTTGATCCCACTACTTTCTCACGTGTCATTGAGCTCGTATGAT
ACACCTTATTCGTTTCCTCCAACATTCTCATGCGTCATTGCCTTCGTATGAC
ACACTTTATTTCATTCCCACTACTTTCTCGCGTGTCATTGGCCTCGTATGAT
ACACTTTATTCCGTTCCTCCCACTTTCTCATGCATCATTCCACTTCTATGACACACTTTATT
> GCGTGTCATT Con:1 reps=6 [2719945,2720267] l-int=323 l-seq = 52
GCGTGTCATTGGCCTCGTATGACACGTATTATTTCAGTTATTGTGCTCTCTC
GTGTGTCATTGACCTCGTATGACACGTCTTATTTCAGTTCTTCAGCTTTCTT
GCGTGTCATTGCTCCCGTATGACACATCTTATTTCAATTTTTCAGATTTCTC
GCGTGTCATTGATCCCGTATGACGCATCTTATTTCAATTCTTCAGCTTTCTC
GCGTGTCATTGATCCCGTATGACGCATCTTATTTCAATTCTTCAGCTTTCTC
GCGTGTCATTGACCCCGTATGACACGTCTTATTTCGGTTTTTCCGCTTTCTCGCGTGTCATT
> CTATGATGAC Con:1 reps=8 [2730527,2730954] l-int=428 l-seq = 52
CTATGATGACCTTCTTGGATTCGTTTATGAATTTTTGGGCAGTAATGCTTTT
CTATGATGACCTTCGTGCATTGGTTTTTTGATTTTCGGGTAGCATTGCTTTT
CTATGATGACCTTCGTGGATTGACTTTTGGATTATCGGGCAGCATTGCTTGC
CTATGGTGACCTTCGTTGAGTGTGGTTTGGCTTTTGGGGCAGCATTGGCCGC
CTATGATGACCTTCGTGCATTGGTTTTTTGATTTTCGGGTAGCATTGCTTTT
CTATGATGACTTTCGTGGATTGACTTTTTGATTATCGGGCAGCATTGAGTCT
CTATGATGACTTTCGGTGGATTGACTTTTTGATTATCGGGCAGCATTGCTTAT
CTATGATGACCTTCGTTGAGTGTGGTTTGGCTTTTGGGGCAGCATTGGCCGCCTATGATGAC
> ACACTTTATT Con:1 reps=7 [2731003,2731378] l-int=376 l-seq = 52
ACACTTTATTCGTATCGACATACTTTCTCACGTGTCATTGCACTTCTATGAT
ACACTTTATTTGTTCCTACTACTTTCTCATGCGTCATTGCCTTCGTATGAC
ACACTTTATTCGTTTCCACATAAATCTTCACGTGTCATTGCACTTCTATGAC
ACACTTTATTTTGATCCCACTACTTTCTCACGTGTCATTGAGCTCGTATGAT
ACACCTTATTCGTTTCCTCCAACATTCTCATGCGTCATTGCCTTCGTATGAC
ACACTTTATTTCATTCCCACTACTTTCTCGCGTGTCATTGGCCTCGTATGAT
ACACTTTATTCCGTTCCTCCCACTTTCTCATGCATCATTCCACTTCTATGACACACACTTTATT
> TCGTGTGTCA Con:1 reps=8 [2784025,2784399] l-int=375 l-seq = 52
TCGTGTGTCATTGCCTTTGTACGACACATCTTATTTTCATTCTTCTTCTTTC
TCGTGTGTCATTGCCTTTGTATGATACATCTTATTTTCATTCTTCTTCTTTC
TCGTGTGTCATTGCCTTCGTATGATACATCTTATTTTTATTCTTCTTCTTTC
TCGTGTGTCATTGCCTTTCTATGATGCATATTATTTTCATTCTTCTTCTCTC
TCGTGTGTCATTGCCTTTGTATGACACATCTTATTTTCATTCTTCTTCTTTC
TCGTGTGTCATTGCCTTTGTACGACACATCTTATTTTCATTCTTCTTCTCTT
TCGTGTGTCATTGCCTTCGTATGATACACCTTATTTTCATTCTTCTTCTCTTTCGTGTGTCA
> AAGGGCAGCA Con:1 reps=6 [2835835,2836208] l-int=374 l-seq = 52
AAGGGCAGCATACGAAGCATATGCTGCCCGAAAAAACGAAGTTGAGCAGATC
ATGGTCATCATACGAAGCATATGCCACCCGAAAAAACGAAAGCGAGCAGATA

AAGGTCACCATACGAAGCCTATGCCACCCGAAAATATGAAAGCGAGCAGATG
AAGGGCAGCATACGAAGCCTATGCCACCCGAAAAAACGAAATCGAGCAGATG
AAGGGCAGCATACGAAGCATATGCTTCCCGAAAATATGAAATCGAGCAGATG
AAGGGCAGCATACGATGCCTATGCCTCCCGAAAAAATCAAATCGAGCAGATG
AAGGGCAGCATACGATGCCTATGCTTCCCGAAAATATGAAATCGAGCAGATAAGGGCAGCA
> CCATCTTACC Con:1 reps=4 [2888411,2888577] l-int=167 l-seq = 52
CCATCTTACCTTCACACTCTTTTTCCTTGCCTCAAGGGCAGTTGGCGGCTTC
CCATCTTACCTTCACGCTCTTTTTCTTTTGCTCAAGGGCA**GATGGC**AGCTTC
CCATCTTACCTCCACACTCTTTTTTCTCTGCTCAAGGGCAGTTGTCGACTC**GCCATC**TTACC
> TGCGAGTGGA Con:1 reps=6 [3148329,3148598] l-int=270 l-seq = 52
TGCGAGTGGACATTAAAGTTCGTTAATAGCCCATGTGGTAAAGGGAGCATG
TGCGAGTGGACTTTAATGTGCCTATAAAGTCCCATGAGGTCGAGGGAGCATG
TGCGAGTGGACTTTAAAGTACCTTTAAAATCCCGTGAGGTAGAGAGAGCATG
TGCGAGTGGACATTAAAATGCCTTTAAAATCCCGTGAGGTAGAGGGATTATG
TGCGAGTGGACTTTAATGTGCCTATAAAGTCCCATGAAGTCGAGGGAGCATGTGCGAGTGGA
> GCTTTAGCAA Con:1 reps=4 [3334663,3334829] l-int=167 l-seq = 52
GCTTTAGCAACTCTATTGGAGTTTTAAACATTGTTTCTTAGTCCTTTAGTCT
GCTTTAGCAACTATAATGGGATTTTAAACATTGTTTCTTAGCCCTTCAGTCT
GCTTTAGCAACTCTATTGGGATTTTAAACATTGTTTCTTAGCCCTTCAGTCTGCTTTAGCAA
> ATGATGGGAT Con:1 reps=4 [3428157,3428323] l-int=167 l-seq = 52
ATGATGGGATATTAAATATTTGTTCATGTACACTCAGACATGCGGTGCTCTC
ATGATGGGATATTAAACTTCTGTTCATAGACACTCACACTTGGATTCCTCTC
ATGATGGGATATTAAATATTAGTTCTTGTACACTCAGACATGCGGTGCTCTGATGATGGGAT
> TTTTCCGTTC Con:1 reps=9 [3704894,3705320] l-int=427 l-seq = 52
TTTTCCGTTCAGTGGTTGCGCTCTTGCTTGAACGGAATACTTTGCCTTGTTG
TTTTCCGTTCAATGGATCCTCTCTTCCTTGAACGGAATTCTTTTTCATTTTC
TTTTCCGTTCAGTGGATCCTCTCTTCCTTGAACGGAATTCTTTGTCGTTTTC
TTTTCCGTTCAGTGGATCCTCTCTTCTTTGATCGGAATTCTTTTTCATTTTC
TTTTCCGTTCGGTGGATCCTCTCTTCCTTGAACGGAATTCTTTGTCATTTTC
TTTTCCGTTCAGTGGATCCTCTCTTCCTTGAACGGAATTCTTTTTCATTTTC
TTTTCCGTTCAGTGGATCCTCTCTTCCTTGAACGGAATTCTTTTTCATTTTC
TTTTCCGTTCAGTGGATCCTCTCTTCTTTGAACGGAATTCTTTGTCATTTTCTTTTCCGTTC
> TCTTACCTTC Con:1 reps=5 [3741040,3741258] l-int=219 l-seq = 52
TCTTACCTTCGCACACCTTTTTCTTTGCTCACGGGCA**GATGGC**AGACTCCCT
TCTTACCTTCACTCTTCTTTTTGCTTGCTCACGGGCA**GATGGC**GACCTCCCA
TCTTACCTTCACACTCTTTTTTCTTTGCTCAAGGGCA**GATGGC**AGACTCCCA
TCTTACCTTCACACTCTTTTTTCTTTGCTCAAGGGCA**GATGGC**AGACTCCCATCTTACCTTC
> TTGCTTTCCT Con:1 reps=7 [3773627,3773949] l-int=323 l-seq = 52
TTGCTTTCCTTTCTACTGCATACAGCTTCCTGGAAAGGAATACTAAGCCACT
TTGCTTTCCTTTCTAAGGCACTCTGCATCCTGGAAAGGAATACTAAGCCACT
TTGCTTTCCTTTCTAAGCCATTCAGCTTCCTGGAAAGGAATACTAAGCCACT
TTGCTTTCCTTTCTAAGGCATTCAGCTTCCTGGAAAGGAATACTAAGCCACT
TTGCTTTCCTTTCTAAGGCACTCAGATTCCTGGAAAGGAATACTAAGCCACT
TTGCTTTCCTTTCTGAGGCACGCAGATTCCTGGAAAGGAATACTAAGCCACTTTGCTTTCCT
> AATGGGTTGC Con:1 reps=10 [3821441,3821919] l-int=479 l-seq = 52
AATGGGTTGCTATTTTGTTTTGTACGCTTTTGTCACGTTGTTTTCCTGATGG
AATGGGTTGCTATTTCGTTTTGTACGCTTTTGTCACGTTGTTTTCCTGATGG
AATGGGTTGCTATTTTATTTTGTGTGCCTTTTGCGCGTTGTTTTTCTGCTCG
AATGGGTTGCTATTTTATTTTGTGTGCCTTTTGCGCGTTGTTTTTCTGCTCG
AATGGGTTGCTATTTTGTTTTGCGTGCCTTTTGCGCGTTGTTTTTCTGCTCG
AATGGGTTGCTATTTTGTTTTGCGTGCCTTTTGCACGTTGTTTTTCTGCTCG
AATGGGTTGCTATTTTGTTTTGCGTGCCTTTTGCGCGTTGTTTTTCTGCTCG
AATGGGTTGCTATTTTGTTTTGCGTGCCTTTTGCGCGTTGTTTTTCTGCTCG
AATGGGTTGCTATTTTGTTTTGCGTGCCTTTTGCGCGTTGTTTTTCTGCTCGAATGGGTTGC
> GCTATTTTAG Con:1 reps=5 [3849630,3849848] l-int=219 l-seq = 52
GCTATTTTAGTTTGCTGCCCTTTCTTATGCTGTTCCTTACTTTGAATGGGAC
GCTATTTTAGTTTGCTGCCCTTTCTTATGCTGTTCCTTACTTTGAATGGGCT
GCTATTTTAGTTTGCTGCCCTTTTTTATGCTGTTCCTTACTTTGAATGGGCT
GCTATTTTAGTTTGCTGCCCTTTTTTATGCTGTTCCTTACTTTGAATGGGCTGCTATTTTAG
> **TTTATTTGCT** Con:1 reps=38? SON 44!![3973488,3975783] l-int=2296 l-seq = 52
TTTATTTGCTTCCTTCTTTATTCTCGT**GTGTCAT**TCTCTGCGT**ATGACAC**AT
CTTATTTGGTTTTTTCTTCAATTTCAC**GTGTCAT**TGATTGCGTATGAAACAC
TTTATTTGCTTCTTTCTTCATTCTCAT**GTGTCAT**TGATTGCGTATGAAACAC
TTTATTTGCTTCTTTCTTCATTCTCAT**GTGTCAT**TGACTTCGTATGAAACAC
TTTATTTGCTTCTTTCTTCATTCTCAT**GTGTCAT**TGACTTCGTATGAAACAC

TTTATTTGCTTCTTTCTTCATTCTCAT**GTGTCAT**TGACTTCGTATGAAACAC
TTTATTTGCTTCTTTTTCATTCTCAT**GTGTCAT**TGACTTCGTATGAAACAC
TTTATTTGCTTCTTTCTTTATTCTCGC**GTGTCAT**TGACTCCGTATGAAACAC
TTTATTTGCTTCTTTCTTTATTCTCGT**GTGTCAT**TGTTGACGT**ATGACAC**AT
TTTATTTGCTTCTTTCTTCATTCTCAT**GTGTCAT**TGACTTTGTATGAAACAC
TTTATTTGCTTCTTTCTTTATTCTCAT**GTGTCAT**TGGCGGCGT**ATGACAC**AT
TTTATTTGCTTCTTTCTTCATTCTCAT**GTGTCAT**TGACTTCGTATGAAACAC
TTTATTTGCTTCTTTCTTTATTCTCGC**GTGTCAT**TCGCTGCGT**ATGACAC**AT
CTTATTTGGTTTTTTCTTCAATTTCAC**GTGTCAT**TGATTGCGT**ATGACAC**AT
TTTATTTGCTTCTTTCTTTATTCTCGC**GTGTCAT**TGACTCCGTATGAAACAC
TTTATTTGCTTCTTTCTTTATTCTCAA**GTGTCAT**TGTTGACGT**ATGACAC**AT
CTTATTTGGTTTTTTCTTCAATTTCAC**GTGTCAT**TGAATGCATATGAAACAC
TTTATTTGCTTCTTTCTTCATTCTCGT**GTGTCAT**TGTTGACGT**ATGACAC**AT
CTTATTTGGTTTTTTCTTCAATTTCAC**GTGTCAT**TGGATGCATATGAAACAC
TTTATTTGCTTCTTTCTTCATTCTCGTGCGTCATTGATTGCCT**ATGACAC**AC
TTTATTTGCTTCTTTCTTTATTCTCGC**GTGTCAT**TGATTGCGT**ATGACAC**AT
TTTATTTGCTTCCTTCTTCATTCTCATGCGTCATTGACTTCGTATGAAACAC
TTTATTTGCTTCTTTTTCATTCTCAT**GTGTCAT**TGATTGCGT**ATGACAC**AT
TTTATTTGCTTCTTTCTTCATTCTCAT**GTGTCAT**TGACTTCGTATGAAACAC
TTTATTTGCTTCTTTCTTTATTCTCAT**GTGTCAT**TGGCGGCGT**ATGACAC**AT
TTTATTTGCTTCTTTCTTTATTCTCAT**GTGTCAT**TGACTTCGTATGAAACAC
TTTATTTGCTTCTTTCTTTATTCTCGTATGTCATTGTTGACGT**ATGACAC**AT
TTTATTTGCTTCTTTCTTCATTCTCAT**GTGTCAT**TGACTTTGTATGAAACAC
TTTATTTGCTTCTTTCTTTATTCTCAT**GTGTCAT**TGGCGGCGT**ATGACAC**AT
TTTATTTGCTTCTTTCTTCATTCTCAT**GTGTCAT**TGACTTCGTATGAAACAC
TTTATTTGCTTCTTTCTTTATTCTCGC**GTGTCAT**TGACTCCGTATGAAACAC
TTTATTTGCTTCTTTCTTTATTCTCGT**GTGTCAT**TGTTGACGT**ATGACAC**AT
CTTATTTGGTTTTTTCTTCAATTTCAC**GTGTCAT**TGAATGCATATGAAACAC
TTTATTTGCTTCTTTCTTCATTCTCGT**GTGTCAT**TGTTGACGT**ATGACAC**AT
CTTATTTGGTTTTTTCTTCAATTTCAC**GTGTCAT**TGAATGCATATGAAACAC
TTTATTTGCTTCTTTCTTCATTCTCGT**GTGTCAT**TGTTGACGT**ATGACAC**AT
CTTATTTGGTTTTTTCTTCAATTTCAC**GTGTCAT**TAAATGCATATGAAACAC
TTTATTTGCTTCTTTCTTCATTCTCGTGCGTCATTGATTGCGT**ATGACAC**AT
TTTATTTGCTTCCTTCTTCATTCTCAT**GTGTCAT**TGATTGCGT**ATGACAC**AT
TTTATTTGCTTCTTTCTTCATTCTCAT**GTGTCAT**TGACTTCGTATGAAACAC
TTTATTTGCTTCTTTCTTCATTCTCAT**GTGTCAT**TGACTTCGTATGAAACAC
TTTATTTGCTTCTTTCTTCATTCTCAT**GTGTCAT**TGACTTCGTATGAAACAC
TTTATTTGCTTCTTTCTTTATTCTCAT**GTGTCAT**TGGCGGCGT**ATGACAC**AT
TTTATTTGCTTCTTTCTTTATTCTCAT**GTGTCAT**TGACTTCGTATAAAACA**TTTATTTGCT**
> CACCTTACAA Con:1 reps=10 [3977361,3977891] l-int=531 l-seq = 52
CACCTTACAATGTCTCTCTAATTTATTTGCTGCCCTTTCTTATGCTTTTTT
CACCTTACAATGACTCACTAATTTAATTTGCTTCCCTTTCTTATGCTTTTTC
CACCTTACAATGGCTCGCTAATTTATTTTGCTGACCTTTATAATGCTTTTTT
CACCTTGCAATGGCTCACTAATTTAATTTGCTGCCCTTTCTTATGCTTTTTC
CACCTTACAATGGCTCGCTAATTTAATTTGCTGCCCTTTCTTATGCTTTTTC
CACCTTACAATGGCTCGCAAAATTAACTTGCTGCCCTTTCTTATGCTTTTTC
CACCTTACAATGGCTCGCTAATTTAATTTGCTGCCCTTTCTTATGCTTTTTT
CACCTTACAATGGCTCGCTAATTTAATTTGCTTCCCTTTCTTATGCTTTTTT
CACCTTACAATGGCTCGCTAATTTAATTTGTTGCCCTTTCTTATGCTTTTTC
CACCTTACAATGGCTCGCTAATTTAATTTCCTGCCCTTTCTTATGCTTTTTCCACCTTACAA
> AAGGAATGGG Con:1 reps=11 [3996570,3997100] l-int=531 l-seq = 52
AAGGAATGGGGCGCTATTCTTATTTGCTGGCCTTTACCCTCTGATTTTTGTA
AAGGAATGGGGCGCTATTCTTATTTGCTGGCCTTTACCCTCTGATTCTTGTA
AAGGAATGGGGCGCTATTCTTATTTGCTGGCCTTTACCCTCTGATTTTTGTA
AAGGAATGGGGTGCTATTCTTGTTTGCTGCCCTTTACATTCTGATTCTTGTA
AAGGAATGGGGCGCTATTCTTGTTTGCTGCCCTTTACCTTCTGATTTTTGTC
AAGGAATGGGGCGCTATTCTTATTTGCTGACCTTTACCTTCTGATTTTTGTA
AAGGAATGGGGCGCTATTCTTGTTTGCTGGCCTTTACCTTCTGATTTTTGTA
AAGGAATGGGGCGCTATTCTTATTTGCTGGCCTTTACCTTCTGATTTTTGTA
AAGGAATGGGGCGCTATTCTTATTTGCTGGCCTTTACCCTCTGATTCTTGTA
AAGGAATGGGGCGCTATTCTTGTTTGCTGCCCTTTACCCTCTGATTTTTTTAAGGAATGGG
> TTTCCTTTCT Con:1 reps=17 [4033931,4034772] l-int=842 l-seq = 52
TTTCCTTTCTGTGCAC
ATTTCCTTCTTAGAAAGGAATACTAACTTCTCTGGC
TTTCCTTTCTATGCACACTTCCTTCTTAGAAAGGAATACTAACTCCTCTGGC

TTTCCTTTCTATGCACTTTTCATCCTTAGAAAGGAATACTAACTCCTCTGGC
TTTCCTTTCTGTGCACTTTTCATCCTTAGAAAGGAATACTAACACTTTTGGC
TTTCCTTTCTGTGCATACTTCCTCCTTAAAAAGGAATACTAACTCCTCTGGC
TTTCCTTTCTATGCACTTTTCATCCTTAGAAAGGAATACTAACTCCTCTGGC
TTTCCTTTCTGTGCACTTTTCATCCTTAGAAAGGAATACTAGCTCCTCTGGC
TTTCCTTTCTGTGCACTTTTCATCCTTAGAAAGGAATACTAACTCCTCTGGC
TTTCCTTTCTATGCACTTTTCATCCTTAGAAAGGAATACTAACTCCTCTGGC
TTTCCTTTCTATGCACACTTCCTCAATAGAAAGGAATACTAACTCCTCTGGC
TTTCCTTTCTATGCACACTTCCTCAATAGAAAGGAATACTAACTCCTCTGGC
TTTCCTTTCTATGTCCACTTCCTTCTTAGAAAGGAATACTAGCTTCTCTGGC
TTTCCTTTCTATGCACTTTTCATCCTTAGAAAGGAATACTAACTCCTCTGGC
TTTCCTTTCTGTGCACACTTCCTCCTTAGAAAGGAATACTAGCTCCTCTGGC
TTTCCTTTCTATGCACTTTTCATCCTTAAAAAGGAATACTAGCTCCTCTGGC
TTTCCTTTCTATGCACACATCCCAATAGAAAGGAATACTAGCTACTCTGGCTTTCCTTTCT
> CATTGCTCTC Con:1 reps=19 [4063448,4064391] l-int=944 l-seq = 52
CATTGCTCTCGT**ATGACAC**GC**TTTATTTGCT**TGTTTGCTTTTTTTCATGTGG
CATTGCTCTCGT**ATGACAC**GC**TTTATTTGCT**TGTTTCTTTTTTTCACGTGG
CATTGCTCTCGTATGGCACGC**TTTATTTGCT**TGTTTCTTTTTTTCACGTGG
CATTGCTCTCGT**ATGACAC**GCTTTATTCGATTGTTTCCTATTTTTCATGTGT
CATTGCTCTCGT**ATGACAC**GC**TTTATTTGCT**TGTTTCTTTTTTTCACGTGG
CATTGCTCTCGT**ATGACAC**GCTTTATTCGATTGTTTCCTATTTT**TCAT**GTGG
CATTGCTCTCGTATGGCACGC**TTTATTTGCT**TGTTTGCTTTTTTTCATGTGG
CATTGCTCTCGTATGGCACGCGTTATTTGCTTGTTTGCTTTTTTTCATGTGT
CATTGCTCTCGT**ATGACACA**CTTTATTCGCTTGTTTGCTTTTTTTCA**TGTGT**
CATTGCTCTCGT**ATGACAC**ACTTTATTCGCTTGTTTGCTTTTTTTCATGTGG
CATTGCTCTCGT**ATGACAC**GC**TTTATTTGCT**TGTTTGCTTTTTTTCATGTGT
CATTGCTCTCGT**ATGACAC**GC**TTTATTTGCT**TGTGTGCTTTTTTTCATGTGT
CATTGCTCTCGT**ATGACAC**GC**TTTATTTGCT**TGTGTGCTTTTTTTCACGTGT
CATTGCTCTCGT**ATGACAC**ACTTTATTCGCTTGTTTGCTTTTTTTCACGTGG
CATTGCTCTCGT**ATGACAC**GC**TTTATTTGCT**TGTGTGCTTTTTTTCACGTGT
CATTGCTCTCGT**ATGACAC**GCGTTATTCGATTGTTTGCTTTTTTTCATGTGG
CATTGCTCTCGT**ATGACAC**GC**TTTATTTGCT**TGTTTGCTTTTTTTCATGTGG
CATTGCTCTCGT**ATGACAC**GCGTTATTCACTTGTTTGCTTTTTTTCATGTGGCATTGCTCTC
> CTTCGTATGA Con:1 reps=6 [4069392,4069818] l-int=427 l-seq = 52
CTTCGTATGATACACATTATGCTGGCTCTTCGGCTTTTTCAT**GTGTCAT**TGA
CTTCGTATGATACATCTTATTTCGGCTCTTCGGCTTTTTCAT**GTGTCAT**TCG
ATTCGTATGATACACATTATTCTGGCTCTTCGGCTTTTTCAT**GTGTCAT**TCG
ATTCGT**ATGACAC**ACATTATACTGGCTCCACTGCTTTTTCAC**GTGTCAT**TGA
CCTCGT**ATGACAC**ACATTATGCTGACTCTTCTGCTTTTTCAT**GTGTCAT**TGC
CTTCGTATGATACATCTTATTTCGGCTCTTCGGCTTTTTCGC**GTGTCAT**TGA
CTTCGTATGATACATCTTATTTCGGCTCTTCGGCTTTTTCAT**GTGTCAT**TCG
CTTCGT**ATGACAC**ACATTATGCTGGCTCCACTGCTTTTTCAC**GTGTCAT**TGGCTTCGTATGA
> GTATGATGAC Con:1 reps=11 [4072932,4073566] l-int=635 l-seq = 52
GT**ATGATGACC**TTCGCGACTTCCGTTTCGATTTCTCGGGC**AGCATTG**CCTTC
GT**ATGATGACC**TTCGCGGCTTCAGTTTTGGTTTCTCGGGC**AGCATTG**CCTTC
GTATGTTGACCGTCGCGACTTCCGTTTCGATTTCTCGGGC**AGCATTG**CCTTC
GTATGTTGACCTTCGCGGCTTCAGTTTTGGTTTCTCGGGC**AGCATTG**CCTTC
GT**ATGATGACC**TTCAGCTCTTCAGTTTTACTCTCTCGGGTTGCATAGCCTTC
GT**ATGATGACC**GTCGCGACTTCAGTTTCGATTTCTCGGGCTGCATTGAACTC
GT**ATGATGACC**GTCGCGACTTCAGTATCGATTTCTCGGGC**AGCATTG**AACTC
GTATGATGACTTTCAGCTCTTCAGTTTTACTCTCTCGGGTTGCATTGGACTT
GT**ATGATGACC**GTCTCGACTTCCGTTTTGTTTTCTCGGGCTGCATTGAACTT
GT**ATGATGACC**GTCGAGGCTTCCGTTTAAATTTCTCGGGC**AGCATTG**CCTTC
GT**ATGATGACC**TTCGCGGCTTCAGTTTTGATTTCTCGGGC**AGCATTG**AACTC
GT**ATGATGACC**GTCTCGACTTCAGTTTCGATTTCTCGGGCTGCATTAGCTTCGTATGATGAC
> AAAGGAATGC Con:1 reps=13 [4080458,4081091] l-int=634 l-seq = 52
AAAGGAATGCTAGACTCGTTCGCTTTCCTTTCTAACGCTCGTTCCCCTTCAG
AAAGGAATGCTAGACTCGTTCGCTTTCCTTTCTAATGCTCGTTTCTCTTCAG
AAAGGAATGCTAGACTCGTTCGCTTTCCTTTCTAATGCTGGTTTCTATTCAG
AAAGGAATGCTAGACTCGTTCGCTTTCCTTTCTAATGCTGGTTTCTATTCAG
AAAGGAATGCTAGACTCTTTGGCTTTCCTTTGTAACGCTCGTTTCTATTCAG
AAAGGAATGCCAGCCTCGTTCGCTTTCCTTTCTAACGCTCGTTTCTATTCAG
AAAGGAATGCTAGACTCGTTGGCTTTCCTTTCTAACACTGGTTCTTCTTCAG
AAAGGAATGCCAGACTCGTTGGCTTTCCTTTCTAACACTGGTTCTTCTTCAG
AAAGGAATGCCAGACTCGTTCGCTTTCCTTTCTAATGCTCGTTTCTCTTCAG

AAAGGAATGCTAGACTCTTTCGCTTTCCTTTCTAATGCTGGTTTCTATTCAG
AAAGGAATGCTAGACTCGTTCGCTTTCCTTTCTATGCTCGTTTCTCTTCAC
AAAGGAATGCTAGACTCGTTTCCTTTTCTTTCTAACGCTCGTTACCCTTCAGAAAGGAATGC
> CTTTCTCGGA Con:1 reps=8 [4116764,4117138] l-int=375 l-seq = 52
CTTTCTCGGATAGTTTTCCTTTCAGAGACTTTTTCCTTCTTTGAACGGAACA
CTTTCTCGGATAGTTTTCCTTTCAGAGACTTGTTCCTTCTTTGAACGGAACA
CTTTCTCGGATAGTTTTCCTTTCAGAGACTTTTTCCTTCTTTGAACGGAATG
CTTTCTCGGATAGTTTTCCTTTCAGAGACTTGTTCCTTCTATGAACGGAATG
CTTTCTCGGATAGTTTTCCGTTCAGTGACTTTTTCCTTCTTTGAACGGAACG
CTTTCTCGGATAGTTTTCCGTTCAGTGACTGACTCCTCCTCTGAACGGAATG
CTTTCTCGGATAGTTTTCCTTTCATAGACTTTTTCCTTCTTTGAACGGAACGCTTTCTCGGA
> CTTTCTCGGG Con:1 reps=8 [4124120,4124702] l-int=583 l-seq = 52
CTTTCTCGGGTAGCATTGGAATCGTATGTTGACCTTCAGCTTCTAAGTTTTT
CTTTCTCAGGTAGCATTGGCTTCGTATGACGACCTTCACCGCTTCAGTTTTA
CTTTCTCGGGCATCATTGCCTTCGTATGACGACCTTCAGCTTCTAAGTTTTA
CTTTTTCGGGTAGCATTGCCTTCGTATGATGACCTTCACCGCTTTAGTTCCA
CTATCTTGGGCAGCATTGAACTCGTATGACGACCTTCAGCTTCTAAGTTTCA
CTTTCTCGCGTAGCATTGCCTTAGTATGATGACCTTCAGCTCTTCAGTTTTA
CTTTCTCGGGTAGCATTGCCTTCGTATGACGACCTTCACCGCTTCAGTTTTA
CTTTCTCGGGCATCATTGCCTTCGTATGACGACCTTCACCGCTTCAGTTTTA
CTTTCTCGGGTAGCATTGCCTTCGTATGATGACCTTCAGCTTCTAAGTTTGG
CTTTCTCGGGCAGCATTGCCTTCGTATGATGACCTTCAGCTTCTAAGTTTTA
CTTTCTCGGGTAGCATTGGCTTCGTATAATGACCTTCACCGCTTCAGCTTGGCTTTCTCGGG
> TTCCTTTCTA Con:1 reps=12 [4188801,4189429] l-int=629 l-seq = 52
TTCCTTTCTACTAGAAAAATTGATGAGGAAAGGAATACTAATCGTGTTTGTA
TTCCTTTCGCACATGTAAATCAATCTCGAACGGCATGCTAAACGCCTTCACT
TTCCTTTCTACTAGTAAAATCAGCCATGAAAGGAACACTAAACGCT
TTCCTTTCTACTAGTAAAATCAATCGTGAAAGGAATGCCAAACGCTTTCACT
TTCCTTTCTACTAGTAAAATCAGCCATGAAAGGAATACTATACGCCTTCACT
TTCCTTTCTACTAGTAAAATCAATCGTGAAAGGAATACTATACGCTTTCACT
TTCCTTTCTACTAGTAAGATCAGCCATGAAAGGAACACTAAACGCCTTCACT
TTCCTTTCTACTAGTAAAATCAGCCATGAAAGGAATACTAAACGTCTTCACT
TTCCTTTCTACTAGTAAAATCAGCCATGAAAGGAATACTAAACACCTTTGCA
TTCCTTTCTACTAGTAAAATCAGCCATGAAAGGAACACTAAACGCTTTCGCT
TTCCTTTCTACTAGTAAAATCAGCCAAGAAAGGAACACTAAACGCTTTCGCT
TTCCTTTCTACTAGTAAAATCAGCCAAGAAAGGAATACTAAACACCTTTGCATTCCTTTCTA
> TAAAGGAATA Con:1 reps=19 [4365838,4366784] l-int=947 l-seq = 52
TAAAGGAATACTAAGCACTCTGGCTTTCCTTTCTTCGTCGATTTCCTCTTTC
TAAAGGAATACTAAACGCTCTGGCTTTCCTTTCTACGCCGATTCCCTCGTCC
TAAAGGAATACTAAGCACTCTGGCTTTCCTTTCTTCGTCGATTTCCTCTTTC
TAAAGGAATACTAAACGCTCTAGCTTTCCTTTCTTCGTCGATTTCCTCTTTC
TAAAGGAATACTAAACGCTCTGGCTTTCCTTTCTTCGTCGATTCCCTCGTCC
TAAAGGAATACTAAACGCTCTAGCTTTCCTTTCTTCGTCGATTCCCTCGCCA
TAAAGGAATACTAAGCACTCTGGCTTTCCTTTCTACATCGATTTCCTCTTAC
TAAAGGAATACTAAACGCTCTGGCTTTCCTTTCTACGCCGATTTCCTCGTCC
TAAAGGAATACTAAGCACTCTGGCTTTCCTTTCTACATCGATTCCCTCGACA
TAAAGGAATACTAAACACTCTGGCTTTCCTTTCTTCGTCGATTCCCTCGACA
TAAAGGAATACTAAGCACTCTGGCTTTCCTTTCTACGCCGATTCCCTCGACA
TAAAGGAATACTAAGCACTCTGGCTTTCCTTTCTACATCGATTTCCTCTTAC
TAAAGGAATACTAAACGCTCTGGCTTTCCTTTCTACGCCGATTTCCTCGTCC
TAAAGGAATACTAAGCACTCTGGCTTTCCTTTCTTCGTCGATTCCCTCGCCA
TAAAGGAATACTAAGCACTCTGGCTTTCCTTTCTACGCCGATTCCCTCGACA
TAAAGGAATACTAAACACTCTGGCTTTCCTTTCTACATCGATTCCCTCGACA
TAAAGGAATACTAAGCACTCTTGCTTTCCTTTCTACGCCGATTTCCTCGTCC
TAAAGGAATACTAAGCACTCTGGCTTTCCTATCTTCGTCGATTCCCTCGCCATAAAGGAATA
> CGCTATGATT Con:1 reps=9 [4408835,4409261] l-int=427 l-seq = 52
CGCTATGATTACCGTCAACTCATTTTGGGCTCGTTCTCGGTAAGCATACAAT
CGCTATGATTACCTTCATCTCATTTTGGACTGGATCTCGGGAAGCATACACG
CGCTATGATTACCTTCAACTCTTTTTGGGCTCTCTCTCGGGAAGCATACACG
CGCTATGATTACCGTCAACTCATTTTGGGCTCGTTCTCGGTAAGCATACAAT
CGCTATGATTACCTTCATCTCATTTTGGACTGGATCTCGGGAAGCATACACG
CGCTATGATTACCGTCAACTCATTTTGGACTCGTTCTCGGGCAGCATACAAT
CGCTATGATTACCTTCAACTCATTTTGGGCTCGTTCTCGGGAAGCATACAAA
CGCTATGATTACCTTCAACTCATTTTGGGCTCGTTCTCGGGCAGCATACAATCGCTATGATT
> CTATGATTAC Con:1 reps=5 [4410749,4411019] l-int=271 l-seq = 52

CTATGATTACCTTCAACTCATTTTGGGCTCTCTCTCGGGAAGCATACACGAG
CTATGATTACCTTCATCTCATTTTGCACTCGTTCTCGGGAAGCATACACGAG
CTATGGTTACCGTCAACTCATTTTGGGCTCTCTCTCGGGAAGCATACACGCG
CTATGATTACCGTCAACTCATTTTGGACTCGTTCTCGGGCAGCATACAAACG
CTATGATTACCGTCAACTCATTTTGGGCTCACTCTCAGGCAGCATACAACCTCTATGATTAC
> GCTCAAGGGC Con:1 reps=26 [4577046,4578356] l-int=1311 l-seq = 52
GCTCAAGGGCTGATGGGGCGCC**GCCATC**TTTCCTTCACACTTTTTTTATTC
GCTCAAGGGCAGTTGGGGCAAC**GCCATC**TTACCTTCACGACACTTTTTATTT
GCTCAAGGGCT**GATGGC**AACGC**GCCATC**TTAACTTCACGCTATTTTTTATTT
GCTCAAGGGCT**GATGGC**AACGC**GCCATC**TTACCTTCACAACACTTTTTATTT
GCTCAAGGGCT**GATGGC**AACGC**GCCATC**TTACCTTCACACTATTTTTTATTC
GCTCAAGGGCAGATGGGGCGCC**GCCATC**TTACTTTCACACTATTTTTTATTT
GCTCAAGGGCAGATGGGACAGC**GCCATC**TTACCTTCACGCTATTTTTTATTC
GCTCAAGGGCT**GATGGC**AACGC**GCCATC**TTACCTTCACGCTATTTTTTATTC
GCTCAAGGGCT**GATGGC**AACGCGCCTTCTTTCCTTCACAACACTTTTTATTT
GCTCAAGGGCT**GATGGC**AACGC**GCCATC**TTACCTTCACACTATTTTTTATTC
GCTCAAGGGCAGTTGGGGCGGCTCCATCTTACCTTCACAACACTTTTTATTT
GCTCAAGGGCT**GATGGC**AACGC**GCCATC**TTACCTTCACGCTATTTTTTATTC
GCTCAAGGGCAGATGGGGCGGCTCCATCTTACCTTCACGCTATTTTTTATTC
GCTCAAGGGCAGTTGGGGCAAC**GCCATC**TTACCTTCACGCTATTTTTTATTT
GCTCAAGGGCA**GATGGC**AACGCACCATCTTACCTTCACGCTATTTTTTATTT
GCTCAAGGGCT**GATGGC**AACGC**GCCATC**TTACTTTCACACTATTTTTTATTT
GCTCAAGGGCAGATGGGACAGC**GCCATC**TTACCTTCACGCTATTTTTTATTC
GCTCAAGGGCA**GATGGC**AACGC**GCCATC**TTACCTTCACGCTATTTTTTATTC
GCTCAAGGGCAGTTGAGGCGGCTCCATCTTACCTTCACACTTTTTTTTATTC
GCTCAAGGGCAGATGGGGCGGCTCCATCTTACCTTCACGCTATTTTTTATTC
GCTCAAGGGCT**GATGGC**AACGC**GCCATC**TTACCTTCACAACACTTTTTATTT
GCTCAAGGGCAGATGGGACGGCTCCATCTTACCTTCACGCTATTTTTTATTT
GCTCAAGGGCA**GATGGC**AACGC**GCCATC**TTACCTTCACACTTTTTTTTATTC
GCTCAAGGGCAGTTGGGGCAAC**GCCATC**TTACCTTCACAACACTTTTTATTT
GCTCAAGGGCAGTTGGGGCGGCGCCTTCTTTCCTTCACACTTCTTTTTCTACGCTCAAGGGC
> ATTGCTTCTC Con:1 reps=15 [4603616,4604354] l-int=739 l-seq = 52
ATTGCTTCTCTTTTTCGG**ACAGAGTT**CCTTGTTCATTACTC**AACTCTGT**CCA
ATTGCTTCTCTTTTTCGG**ACAGAGTT**CTTTGTTTCTTATTC**AACTCTGT**CTG
ATTGCTTCTCTCTTTCGG**ACAGAGTT**CCTTGTTTATTATTC**AACTCTGT**CTG
ATTGCTTCTCTTTTTCGG**ACAGAGTT**CCTTGTTTATTATTC**AACTCTGT**CTG
ATTGCTTCTCTTTTTCGG**ACAGAGTT**CCTTGTTCATTATTCTACTCTGTCCG
ATTGCTTCTCTTTTTCGG**ACAGAGTT**CCTTGTTTATTATTC**AACTCTGT**CTG
ATTGCTTCTCTTTTTCGG**ACAGAGTT**CCTTGTTCATTACTC**AACTCTGT**CTG
ATTGCTTCTCTTTTTCGG**ACAGAGTT**CCTTGTTCATTACTC**AACTCTGT**CTG
ATTGCTTCTCTTTTTCGGACAGATTTCCTTGTTCATTATTCTACTCTGTCTG
ATTGCTTCTCTTTTTCGG**ACAGAGTT**CCTTGTTTATTATTC**AACTCTGT**CTG
ATTGCTTCTCTTTTTCGG**ACAGAGTT**CCTTGTTTCTTATTC**AACTCTGT**CTG
ATTGCTTCTCTTTTTCGG**ACAGAGTT**CCTTGTTTATTACTC**AACTCTGT**CCA
ATTGCTTCTCTTTTTCGG**ACAGAGTT**CCTTGTTCATTACTC**AACTCTGT**CCA
ATTGCTTCTCTTTTTCGGACAGAGTCTCTTGCTCATTACTC**AACTCTGT**CCGATTGCTTCTC

***B. weihaiensis* (NZ CP016020.1). All tandem repeats with repeat 21**

We find the TCGCGG signal or its reverse (CCGCGA) in most of the repeats.
The tandem repeat with l-seq = 60 is also included. It is not related to the other tandem repeats

--

> AA**TCGCGG**AT Con:1 reps=7 [21005,21182] l-int=178 l-seq = 21
AA**TCGCGG**ATAGAGCTCGGAA
AA**TCGCGG**ATAGGGCCCGGGA
AACCGCGGATAGAACTCGGAA
AA**TCGCGG**ATAGAGCCCGGAA
AATAGCGGATAGGGCCCGGGA
AA**TCGCGG**ATAGAGCCCGGAA
AA**TCGCGG**ATAGGCCCGAGAA
AA**TCGCGG**ATAGGGCCGGAAAA**TCGCGG**AT
> CGGATAGAGC Con:1 reps=10 [25511,25710] l-int=200 l-seq = 21
CGGATAGAGCACTAAGAA**TCG**
CGGATAGAGCACTAAGAA**TCG**
CGGATAGAGCCCTGAAAA**TCG**
CGGATAGAGCACTGAAAA**TCG**
CGGATAGAGCACTGAAAA**TCG**
CGGATAGAGCCCTGAAAA**TCG**
CGGATAGAGCACCGAAAA**TCG**
CGGATAGAGCCCTGAAAA**TCG**
CGGATAGAGCACCGAGAATCA**CGG**ATAGAGC
> AA**TCGCGG**AT Con:1 reps=6 [29979,30094] l-int=116 l-seq = 21
AA**TCGCGG**ATAGACGTACCGG
AA**TCGCGG**ATAGGAGGCCAAT
AA**TCGCGG**ATAGACGTACCGG
AA**TCGCGG**ATAGGAGGCCAAT
AA**TCGCGG**ATAGACGCACCAGAA**TCGCGG**AT
> GGAACAGATA Con:1 reps=5 [134977,135092] l-int=116 l-seq = 21
GGAACAGATAACCCAGGGGAA
GGAACAGATAATCCAGGTGAG
GGAACAGATAACCCAGGAGAA
GGAACAGATAATCCAGGTGAGGGAACAGACCAGCCAGGAGAAGGAACAGATA
> GGAGAAGAAA Con:1 reps=8 [146949,147106] l-int=158 l-seq = 21
GGAGAAGAAACGCCAGGTGAC
GGAGAAGAAACACCGGGTGAT
GGAGAAGAAACACCGGGTGAT
GGAGAAGAAACACCAGGTGAT
GGAGAAGAAACACCGGGTGAT
GGAGAAGAAACACCAGGTGAT
GGAGAAGAAACACCGGGTGATGGAGAAGAAA
> CGAAAATAAA Con:1 reps=4 [177918,177991] l-int=74 l-seq = 21
CGAAAATAAAATAGTGAATGT
CGAAAATAAAATGCAGGATTT
CGAAAATAAATAGAGTGAAAGCGAAAATAAA
> CGAAAATAAA Con:1 reps=4 [414219,414292] l-int=74 l-seq = 21
CGAAAATAAAAGGTGAAATAT
CGAAAATAAAGTAGCTACATC
CGAAAATAAAAGTGCCAAATCCGAAAATAAA
> A**TCGCGG**AAA Con:1 reps=15 [473933,474237] l-int=305 l-seq = 21
A**TCGCGG**AAAGGAGTGCCGGA
A**TCGCGG**AAAGGAGTGCAGGA
A**TCGCGG**AAAGGAGTGCCGGA
A**TCGCGG**AAAGAGGAGCCTGA
A**TCGCGG**AAAGAGGAGCCGGA
A**TCGCGG**AAAGGAGTGCCAGA
A**TCGCGG**AAAGAGGAGCCGGA
A**TCGCGG**AAAGGAGTGCCGGA
A**TCGCGG**AAAGGAGTGCCGGG
A**TCGCGG**AAAGAGGAGCCGGA

A**TCGCGG**AAAGGAGTGCCGGA
A**TCGCGG**AAAGGAGTGCCGGG
A**TCGCGG**AAAGAGGAGCCGGA
A**TCGCGG**AAAGGAGTGCCAGGA**TCGCGG**AAA
> AA**TCGCGG**AT Con:1 reps=6 [497918,498033] l-int=116 l-seq = 21
AA**TCGCGG**ATAGGAGAGCTAA
AA**TCGCGG**ATAGAGGGCTGAA
AA**TCGCGG**ATAGAGGGCTGAA
AA**TCGCGG**ATAGAGGGCTGAA
AA**TCGCGG**ATAGAGGGCTGAAAA**TCGCGG**AT
> AGAA**TCGCGG** Con:1 reps=4 [499255,499328] l-int=74 l-seq = 21
AGAA**TCGCGG**AAAGAGAGGTC
AGAA**TCGCGG**AAAGAGAGGTC
AGAA**TCGCGG**AAAGAGGGGTCAGAA**TCGCGG**
> GAA**TCGCGG**A Con:1 reps=8 [684231,684388] l-int=158 l-seq = 21
GAA**TCGCGG**AAAGAGAGGTCA
GAA**TCGCGG**AAAGAGAGGTCA
GAA**TCGCGG**AAAGAGAGGTCA
GAA**TCGCGG**AAAGAGAGGACG
GAA**TCGCGG**AAAGAGAGGACG
GAA**TCGCGG**AAAGAGAGGACG
GAA**TCGCGG**AAAGAGAGGTCGGAA**TCGCGG**A
> GAA**TCGCGG**A Con:1 reps=7 [757324,757664] l-int=341 l-seq = 21
GAA**TCGCGG**ATAGAGGAGCCG
GAA**TCGCGG**AAAGAGGTGTGG
GAA**TCGCGG**AAAGAGGTGTGG
GAA**TCGCGG**AAAGAGGTGCCG
GAA**TCGCGG**AAAGAGGTGCCG
GAA**TCGCGG**ATAGAAGCCTGA
> AA**TCGCGG**AT Con:1 reps=11 [757325,757833] l-int=509 l-seq = 21
AA**TCGCGG**ATAGAGGAGCCGG
AA**TCGCGG**AAAGAGGTGTGGG
AA**TCGCGG**AAAGAGGTGTGGG
AA**TCGCGG**AAAGAGGTGCCGG
AA**TCGCGG**AAAGAGGTGCCGG
AA**TCGCGG**ATAGAAGCCTGAA
AA**TCGCGG**ATAGAAGCCCCAA
AA**TCGCGG**ATAGAAGCCCCAA
AA**TCGCGG**ATAGAAGCCCCAA
AA**TCGCGG**ATAGAAGCCCCAA
AA**TCGCGG**ATAGAGCACCCAA
AA**TCGCGG**ATAGAAGTCCAAG
AA**TCGCGG**ATAGAGCACCCGA
AA**TCGCGG**ATAGAAGCCCCAAAA**TCGCGG**AT
> TAT**CCGCGA**T Con:1 reps=7 [790108,790244] l-int=137 l-seq = 21
TAT**CCGCGA**TTATGGTTGCTG
TAT**CCGCGA**TTTTAGGTGTTG
TAT**CCGCGA**TTATGGTTGCTG
TAT**CCGCGA**TTTTAGGTGTTG
TAT**CCGCGA**TTATGGTTGCTC
TAT**CCGCGA**TTTTAGGTGCTCTAT**CCGCGA**T
> AA**TCGCGG**AT Con:1 reps=5 [802803,802897] l-int=95 l-seq = 21
AA**TCGCGG**ATAGAGCGTGTAA
AA**TCGCGG**ATAGAGCACCCAT
AA**TCGCGG**ATAGAGCGTGTAA
AA**TCGCGG**ATAGATCATCCATAA**TCGCGG**AT
> ACAGCAAGAG Con:1 reps=5 [1762840,1763090] l-int=251 l-seq = 60
ACAGCAAGAGAGCATGATAAGGAATTCTATCAAGAGATTGGCCAAAAGGGCGGAGAAAAA
ACAGCAAGAGAGCATGACAAGGAATTTTACCAAGAGATTGGTCAAAAGGGTGGAGAAAAA
ACAGCAAGAGAGCATGATAAGGAGTACTATCAGGAAATTGGCCAAAAAGGTGGAGAAAAA
ACAGCAAGAGAGCACGATAAAGAATTCTATCAAGAGATTGGCCAAAAGGGCGGGGAAAAGACAGCAAGAG
> TAT**CCGCGA**T Con:1 reps=15 [2579124,2579428] l-int=305 l-seq = 21
TAT**CCGCGA**TTCTGGGCACTT
TAT**CCGCGA**TTCTGGGCACTC
TAT**CCGCGA**TTGTGAGTGCTC

TAT**CCGCGA**TTATGAGTGTTC
TAT**CCGCGA**TTCTGGGCACTC
TAT**CCGCGA**TTGTGAGTGCTC
TAT**CCGCGA**TTATGAGTGTTC
TAT**CCGCGA**TTCTGAGCACTC
TAT**CCGCGA**TTCTGGGCACTC
TAT**CCGCGA**TTCTGGGCACTC
TAT**CCGCGA**TTCTGAGTGTTC
TAT**CCGCGA**TAATGGGCGCTC
TAT**CCGCGA**TTCTGAGTGTTC
TAT**CCGCGA**TAATGGGCGCTCTAT**CCGCGA**T
> CTAT**CCGCGA** Con:1 reps=7 [2862732,2862868] l-int=137 l-seq = 21
CTAT**CCGCGA**TTTTACATGTG
CTAT**CCGCGA**TTATTGATGCC
CTAT**CCGCGA**TTTTACATGTG
CTAT**CCGCGA**TTATTGGTGCT
CTAT**CCGCGA**TTTTACACGTG
CTAT**CCGCGA**TTATTGGTGCTCTAT**CCGCGA**
> A**TCGCGG**ATA Con:1 reps=12 [2986923,2987185] l-int=263 l-seq = 21
A**TCGCGG**ATAGGGCTCCCGGA
A**TCGCGG**ATAGAACCCTCATA
A**TCGCGG**ATAGAACCCTCATA
A**TCGCGG**ATAGAGCACTCATA
A**TCGCGG**ATAGAGCACCCTTA
A**TCGCGG**ATAGAGCACCCTTA
A**TCGCGG**ATAGGGCACCGGGA
A**TCGCGG**ATAGAACTCCCTTA
A**TCGCGG**ATAGAGCCCCCTTA
A**TCGCGG**ATAGAACACCCTTA
ATCGTGGATAGAGCTCCCTTA
A**TCGCGG**ATAGAGCCCCCATTA**TCGCGG**ATA
> T**CCGCGA**TTT Con:1 reps=11 [3155990,3156787] l-int=798 l-seq = 21
T**CCGCGA**TTTTGGGTGTTCTA
T**CCGCGA**TTTTCGGCGCTCTT
T**CCGCGA**TTTTGGGTGTTCTA
T**CCGCGA**TTTCCGCCTCTCTT
T**CCGCGA**TTTTCGGTGTTCTA
T**CCGCGA**TTCGGGATGTTCCGACAAATTTCGCACATCCCTCATCCTCACACTTTCACTTGTTCATTCCCCCAGCTACTTAATCCTTTCTAAAGAAA
CCAATTTTGAGTCTTTTTAAGAATTCCTAGCTAAATTAGGGTGAATGATCCTTTTCTGAGGCCCCTATGCTTGTCAAAACATCCATTTAGAAAGAA
TTCATCGTTAGATTCAAGTCTATTCGGCTTATTTTATGAGGTCTAACCGCTGTTTTGGGTGCTCTT
T**CCGCGA**TTTTCGGCGCTCTT
T**CCGCGA**TTTTGGGTGTTCTA
T**CCGCGA**TTTCCGCCTCTCTA
T**CCGCGA**TTCGGGATGTTCCGACAAATTTCACACATACCTCATCCTCACACTTTCACTTGTTCATTCCCCCAGCTACTTAATCCTTTCTAAAGAAA
CCAATTTTGAGTCTTTTTAAGAATTCCTAGCTAAATTAGGGTGAATGATCCTTTTCTGAGGCCCCTATGCTTGTCAAAACATCCATTTAGAAAGAA
TTCATCGTTAGATTCAAGTCTATTCGGCTATTTTATGAGGTCTAACCCTGTTTTGGTTGCTCTT
C**CCGCGA**TTTTGGGTGTTCTT
T**CCGCGA**TTTTGGGTGTTCTA
T**CCGCGA**TTTTCGCCTCTCTT
C**CCGCGA**TTTTCGCCTCTCTT
C**CCGCGA**TTTCCGCCTCTCTTT**CCGCGA**TTT
> TTTATTTTCG Con:1 reps=4 [3221394,3221467] l-int=74 l-seq = 21
TTTATTTTCGATTTATGTCCA
TTTATTTTCGAATTTGACCAA
TTTATTTTCGAATTTCGCCTGTTTATTTTCG
> TAT**CCGCGA**T Con:1 reps=5 [3277057,3277151] l-int=95 l-seq = 21
TAT**CCGCGA**TTTTCACCGGTC
TAT**CCGCGA**TTATGGTGCTCT
TAT**CCGCGA**TTTTCACCGGTC
TAT**CCGCGA**TTATCGTACTCTTAT**CCGCGA**T
> CTTT**CCGCGA** Con:1 reps=16 [3348404,3348992] l-int=589 l-seq = 21
CTTT**CCGCGA**TTATTGCCTCT
CTTT**CCGCGA**TTATTGCCCCT
CTAT**CCGCGA**TTATCGCCTCT
CTAT**CCGCGA**TTCGGGAAAATGCGACAATCTTTTTAAAATTTCACGTAATTTTCATGACACTACGTACTGCACTCACCTACTTGACTTCGCTTTGC

TACGATACCGCAGGATCCGGAGAATTTACTTAAGATTTCGAGGCTCTCTTCACAATTTGAGGTTTCTCTCTCTGAATTTTGTACTTTTACCACACT
TTCAAGTACACTATTCACGTTTTAGCTGC
TCTTTCGCGATTCCGCCCTCT
CTTT**CCGCGA**TTCCATCACCC
CTTT**CCGCGA**TTCCATCACCC
CTTT**CCGCGA**TTCCATCACCC
CTTT**CCGCGA**TTCCACCACCT
CTTT**CCGCGA**TTCCGCCCTCT
CTTT**CCGCGA**TTCCATCACCC
CTTT**CCGCGA**TTCCACCACCC
CTTT**CCGCGA**TTCCACCACCT
CTTT**CCGCGA**TTCCATCACCC
CTTT**CCGCGA**TTCCACCACCT
CTTT**CCGCGA**TTCCACCACCT
CTTT**CCGCGA**TTCCATCACCC
CTTT**CCGCGA**TTCCACCACCTCTTT**CCGCGA**
> A**TCGCGG**ATA Con:1 reps=6 [3362340,3362455] l-int=116 l-seq = 21
A**TCGCGG**ATAGAACACTCATA
A**TCGCGG**ATAGAGCCCCCATA
A**TCGCGG**ATAGAACTCCCATA
A**TCGCGG**ATAGAACCCCCATT
A**TCGCGG**ATAGACCCCTCATAA**TCGCGG**ATA
> TTTATTTTCG Con:1 reps=4 [3505433,3505526] l-int=94 l-seq = 21
TTTATTTTCGGCTTTGAGTCA
TTTATTTTCGCTTTTCACCA
TTTATCTTCGGATTTGAGTCA
TTTATTTTCGCTTTTTCCTATTTTATTTTCG
> T**CCGCGA**TTA Con:1 reps=12 [3509098,3509360] l-int=263 l-seq = 21
T**CCGCGA**TTATGGCACCTCTT
T**CCGCGA**TTTTACGGGTCCTA
T**CCGCGA**TTACGGCACCTCTT
T**CCGCGA**TTACGGCACCTCTT
T**CCGCGA**TTACAGCACCTCTT
T**CCGCGA**TTACGGCACCTCTT
T**CCGCGA**TTATGGCACCTCTT
T**CCGCGA**TTACGGCACCTCTT
T**CCGCGA**TTACGGCACCTCTT
T**CCGCGA**TTATGGCACTCCTA
T**CCGCGA**TTATGACACCTCTT
T**CCGCGA**TTATGGCACGCCTAT**CCGCGA**TTA
> TTTATTTTCG Con:1 reps=6 [3543299,3543414] l-int=116 l-seq = 21
TTTATTTTCGCTTTTCCGTGT
TTTATTTTCGGAATTGCCTTG
TTTATTTTCGCTTTCACGGGT
TTTATTTTCGTTTTCATACTA
TTTATTTTCGTTTTCTAGCACTTTATTTTCG
> T**CCGCGA**TTC Con:1 reps=13 [3632787,3633049] l-int=263 l-seq = 21
T**CCGCGA**TTCCAGGCCGCTTA
T**CCGCGA**TTCTGGACCTCCTT
T**CCGCGA**TTCCGAACCTCTTA
T**CCGCGA**TTCTGGACCTCCTA
T**CCGCGA**TTCCGACTCTCCTA
T**CCGCGA**TTCCGACTCTCCTA
T**CCGCGA**TTCCGACTCTCCTA
T**CCGCGA**TTCCGAGACCCCTA
T**CCGCGA**TTCCGGACCTCCTA
T**CCGCGA**TTCCGGACCTCCTA
T**CCGCGA**TTCCGACTCTCCTA
T**CCGCGA**TTCCGACTCTCCTAT**CCGCGA**TTC
> TTTATTTTCG Con:1 reps=5 [3636446,3636561] l-int=116 l-seq = 21
TTTATTTTCGGTTTTTAATTA
TTTATTCACGAATTTAACCAC
TTTATTTTCGGTTTTTAATCA
TTTATTTTCGAAATTCCACGA
TTTATTTTCGCTTTCTCGCAGTTTATTTTCG

> A**TCGCGG**ATA Con:1 reps=16 [3702467,3702792] l-int=326 l-seq = 21
A**TCGCGG**ATAGAGCGCCCATA
A**TCGCGG**ATAAAGCACCCACT
A**TCGCGG**ATAGAACACCCACT
A**TCGCGG**ATAGAGCACCCACA
A**TCGCGG**ATAGAACACCTACA
A**TCGCGG**ATAGAGCACCCACT
A**TCGCGG**ATAGAGCGCCCACA
A**TCGCGG**ATAGAGCACCCACA
A**TCGCGG**ATAAAGCACCCACT
A**TCGCGG**ATAGAGCACCCACA
A**TCGCGG**ATAGAGCACTCATA
A**TCGCGG**ATAGAGCACTCATA
A**TCGCGG**ATAGAGCACTCATA
A**TCGCGG**ATAGAGCACTCATA
A**TCGCGG**ATAGAGCACCCACAA**TCGCGG**ATA
> TTTATTTTCG Con:1 reps=4 [3812432,3812505] l-int=74 l-seq = 21
TTTATTTTCGGTTTTGGCTCA
TTTATTTTCGCTTTTAGCCAA
TTTATTTTCGCTTTTCCACAATTTATTTTCG
> TTTATTTTCG Con:1 reps=5 [3842895,3843010] l-int=116 l-seq = 21
TTTATTTTCGATTTATTGTGG
TTTATTTTCGAATTTCTCCTT
TTTATTTTCGAATTTGATTAG
TTTATTTTCGAATTTTCACTA
> TTTATTTTCG Con:1 reps=4 [3860025,3860119] l-int=95 l-seq = 21
TTTATTTTCGAAATTTCACCT
TTTATTTTCGAAGTTGATGGA
TATATTTTCGATATTTCAGGG
TTTATTTTCGAATTTGATCGTTTTATTTTCG
> TTTATTTTCG Con:1 reps=5 [4154491,4154583] l-int=93 l-seq = 21
TTTATTTTCGGGTTTGACT
TTTATTTTCGAAATTTTAGTG
TTTATTTTCGAATTTGATCAT
TTTATTTTCGAATCTGATCATTTTATTTTCG
> TTTATTTTCG Con:1 reps=4 [4176659,4176732] l-int=74 l-seq = 21
TTTATTTTCGAGTTTGATGGA
TTTATTTTCGAATTTGATCAT
TTTATTTTCGAATCTGATCTTTTTATTTTCG
> AA**TCGCGG**AT Con:1 reps=10 [4295617,4296106] l-int=490 l-seq = 21
AA**TCGCGG**ATAGAGACCGGAT
AA**TCGCGG**AAAGGACGTGCAG
AA**TCGCGG**ATAGAGGTCCGAT
AA**TCGCGG**ATAAGGCGTGTCG
AA**TCGCGG**ATAGGAGCCCAAT
AA**TCGCGG**ATAGAGCCTGATGAGATAGTCGATCACAACCCAAATTACCAAAAAAGTCTCAAGAAATCAGAGGTCCCCCCCAAAGTCTGAAGAAAAA
ACACCAATTCCCACCTGAAATGCTGAGAAAATACATCAAAAACAAGAATTGAACTCCAAAAATGAGGTTAGTGCTTAAGAACAGAGATCAACACTA
AAACTCAGTAAGAGCTCTGAGAAAAATGGTCAAACTAAATGAGATCGTGTAGATGCGAAATTTGTCGGACTAACCCG
AA**TCGCGG**ATAGAGACCGGATAATCACGGAAAGGACGTGCAG
AA**TCGCGG**ATAGAGGTCCGAT
AA**TCGCGG**ATAAGGCGTGTCG
AA**TCGCGG**ATAGGAGCCCAATAA**TCGCGG**AT
