# Supplementary material for: Tandem Repeats in Bacillus: Unique Features and Taxonomic Distribution
Source: Int J Mol Sci. 2021 May 20;22(10):5373. doi: 10.3390/ijms22105373 (PMC8161180; doi:10.3390/ijms22105373)
Supplement: Supplementary file 1 [file ijms-22-05373-s001.zip › Table S7.Signals in genes.pdf]

Table S7. Blastn of TGTCCTTTT in *B. coagulans* (NC-015634)

We have determined the exact matches of the TGTCCTTTT signal on the genome of *B. coagulans* (NC-015634). Blastn has been used. Hits on tandem repeats have been eliminated from the list. We find the sequence to be present in about 50 genes of different types. The first three hits, for example, correspond to tRNA synthetase, FadR transcription regulator and cytochrome oxidase

Start	End
56984	56976
519694	519686
559411	559403
649485	649477
656518	656510
698568	698560
709956	709948
744478	744470
847693	847685
852480	852472
971570	971562
980361	980353
1017354	1017346
1041636	1041628
1480845	1480837
1643601	1643593
1828597	1828589
1872121	1872113
1969558	1969550
2115600	2115592
2253684	2253676
2521570	2521562
2640895	2640887
2718035	2718027
2718087	2718079
2793789	2793781
2871401	2871393
3061604	3061596

Reverse

Start	End
48298	48306
51918	51926
109813	109821
167466	167474
331471	331479
380074	380082
424918	424926
439756	439764
769753	769761
1049399	1049407
1068682	1068690
1108089	1108097
1108505	1108513
1147117	1147125
1280019	1280027
1408525	1408533
1643858	1643866
1711096	1711104
1911361	1911369
2038675	2038683
2154002	2154010
2288686	2288694
2407275	2407283
2417011	2417019
2437651	2437659
2565198	2565206
2583949	2583957
2875492	2875500
2886659	2886667
